# Clinical Features of Children with Pulmonary Microscopic Polyangiitis: Report of 9 Cases

**DOI:** 10.1371/journal.pone.0124352

**Published:** 2015-04-29

**Authors:** Haiyan Wang, Liangzhong Sun, Weiping Tan

**Affiliations:** 1 Department of Pediatrics, Sun Yat-sen Memorial Hospital, Sun Yat-sen University, Guangzhou, Guangdong, Peoples’ Republic of China; 2 Children’s Kidney Disease Center, Department of Pediatrics, The First Affiliated Hospital, Sun Yat-sen University, Guangzhou, Guangdong, Peoples’ Republic of China; University of Leicester, UNITED KINGDOM

## Abstract

Kidneys and lungs are the most common organs involved in microscopic polyangiitis (MPA). A retrospective analysis of pediatric MPA patients with pulmonary lesions over the past 10 years was performed to investigate clinical features of MPA in children with pulmonary lesions. There were 9 patients enrolled in our study, including 2 boys and 7 girls, with a median age of 6.6 years at the time of disease onset and a median disease course of 2 months. All of the patients exhibited tachypnea, and 7 exhibited cough and hemoptysis. The most common presentation on pulmonary imaging was ground glass or patchy shadows, which were observed in 6 cases. Seven patients manifested with hematuria and proteinuria, with renal histopathology of fibrinoid necrosis/exudation of the glomerular capillaries. All of the patients presented with normocytic normochromic anemia. Of the 9 patients, 7 were positive for perinuclear antineutrophil cytoplasmic antibody (p-ANCA) and/or myeloperoxidase (MPO), and 2 were positive for p-ANCA/MPO and cytoplasmic ANCA/proteinase 3. Eight patients had normal complement 3 (C3) levels, and one had an elevated C3 level. Five of the 9 patients were positive for antinuclear antibody ANA, and 4 were positive for double strand DNA (ds-DNA) antibody (3 were positive for both). The 7 patients who exhibited renal involvement received steroid plus cyclophosphamide (CTX) treatment. Of these patients, 4 achieved various degrees of remission, 2 were at the beginning of induction therapy, and one was lost to follow-up. Two patients with isolated pulmonary involvement received steroid plus leflunomide treatment and achieved complete remission. Diffuse alveolar hemorrhage was the most frequent presentation of lung involvement in children with MPA, and tachypnea, cough, hemoptysis and anemia were the common clinical symptoms. The majority of these patients exhibited hematuria, proteinuria and renal insufficiency. The efficacy of steroid plus CTX or leflunomide was evident in these patients.

## Introduction

Microscopic polyangiitis (MPA) is a type of systematic vasculitis that is characterized by pauci-immune glomerulonephritis with glomerular capillary necrosis and crescent formation. MPA mainly involves small arteries, veins and capillaries [1]. Patients with MPA are usually positive for antineutrophil cytoplasmic antibody (ANCA), and more specifically perinuclear ANCA (p-ANCA) and/or myeloperoxidase (MPO). MPA typically exhibits multiple organ involvement, and kidneys and lungs are the most common organs involved [2]. The morbidity of MPA in adults is ten per million (2.5–7.5/million in Europe and 14.8/million in Japan) and is more rare in children [3]. The aim of the present study was to investigate the clinical features, treatment and prognosis of 9 pediatric MPA patients with pulmonary involvement.

## Subjects and Methods

A retrospective analysis of pediatric MPA patients with pulmonary lesions who were diagnosed at Sun Yat-sen Memorial Hospital and The First Affiliated Hospital, Sun Yat-sen University, between 2004 and 2014 was performed. The study was conducted in accordance with the principles outlined in the 1964 Declaration of Helsinki and with approval from the ethics committee of Sun Yat-sen Memorial Hospital, Sun Yat-sen University. Written informed consent was obtained from all of the patients' parents or guardians. The diagnosis of primary MPA was based on the 2012 revised International Chapel Hill Consensus Conference Nomenclature of Vasculitides [4]. Routine blood and urine tests, blood biochemistry tests, and quantitation of proteinuria and autoimmune antibodies [antinuclear antibody (ANA), double stand DNA antibody (dsDNA), anti-glomerular basement membrane antibody (anti-GBM) and ANCA were performed in all of the 9 patients. Moreover, gender and the age of onset, initial symptoms, disease course, level of serum creatinine (Scr), therapeutic regimen and prognosis were recorded. Chronic kidney disease (CKD) was defined using K/DOQI staging (stage 1 to stage 5) [5]. And GFR was calculated according to the Schwartz formula [6]. Underweight and stunting were was defined when the body weight and height were ≤10th smoothed percentiles curves [7].

Induction therapy consisted of a corticosteroid (prednisone: 1–2 mg/kg/d, tapered gradually at 4–8 weeks) plus cyclophosphamide (CTX: 0.75 g/m^2^/month for 6 months). For patients with severe organ involvement (pulmonary hemorrhage, rapidly progressive glomerulonephritis, digestive tract hemorrhage, central neural system involvement), methylprednisolone (MP) pulse therapy (MP: 7.5–15 mg/kg, qod, 3 to 6 administrations per course) was initially used, followed by prednisone plus CTX. Maintenance therapy consisted of low-dose corticosteroid (prednisone: 5–10 mg/d) plus CTX 0.5–0.75 g/m^2^ every 3 months) pulse therapy. If CTX was not tolerated or not accepted, mycophenolate mofetil [MMF (CellCept): 20–30 mg/kg/d bid] was used as a substitute. The patients received maintenance therapy for 1 to 2 years [8]. Furthermore, for patients with lung lesions only, leflunomide (0.3–0.5 mg/kg/day) plus prednisone (1–2 mg/kg/day) were chosen instead of CTX pulse [9].

## Results

### General data

Nine MPA patients (patients 7, 8 and 9 have been described previously [10]) with lung involvement were evaluated in this study. There were 2 boys and 7 girls, with a median age of 6.6 years (1.9–16.2 years) at the time of disease onset and a median disease course of 2 months (0.5–42 months). Underweight/stunting were observed in 4 patients (patients 2, 3, 7 and 8). Glomerular filtration rate (GFR) decrease was observed in 3 patients at the time of diagnosis (patient 5, 6 and patient 8). All the clinical features stated above were list in Table 1.

**Table 1:** Clinical featuresof the 9 MPA patients.

Patient	Sex	Wt(kg)/*P*; Ht(cm)/*P*	Onset age (years)	Disease course (months)	Syndrome of lung lesions	GFR at diagnosis	Extra-pulmonary and extra-renal symptoms	Period of follow up (months)	Treatment	Prognosis
1	M	24/*P*_97_; 112/*P*_75_	5	0.5	Tachypnea, cough	202.5	Fever	10	MP+P+LEF	CR
2	F	14/*P*_3_; 101/<*P*_3_	2	36	Tachypnea, cough and hemoptysis	88.7	Debility	10	MP+P+LEF	CR
3	M	18/<*P*_3_; 110/<*P*_3_	6.6	2	Tachypnea, hemoptysis and chest pain	125.0	Fever, headache, abdominal pain	16	MP+CTX +P+ACEI	CR
4	F	22/*P*_25_; 122/*P*_50_	6.7	4	Tachypnea, cough and hemoptysis	85.1	Arthralgia, myalgia	12	MP+CTX +P+ACEI	CR
5	F	33/*P*_25th_; 146/*P*_50_	10.9	1	Tachypnea, cough and hemoptysis	10.7	Debility, myalgia	10	MP+CTX +P+ACEI	CKD 5 and underwent KT
6	F	29/*P*_75_; 128/ *P*_25_	7.8	8.0	Tachypnea, cough and hemoptysis	28.1	Fever	9	MP+CTX +P	CKD 5
7	F	8.5/<*P*_3_; 86.8/<*P*_3_	1.9	12	Tachypnea, cough and hemoptysis	119.1	Fever, digestive tract hemorrhage	24	MP+CTX +P+MMF	CR
8	F	17.5/*P*_10_; 113/*P*_10_	3.0	42	Tachypnea, cough and hemoptysis	60.7	Fever	86	MP+CTX+P	CKD 3
9	F	48/*P*_50_; 161/*P*_50_	16.2	1	Tachypnea	86.5	Debility	0	MP+CTX+P	Lost

ACEI, angiotensin-converting enzyme inhibitor; CKD, chronic kidney disease; CR, complete remission; CT, computerized tomography; CTX, cyclophosphamide; GFR, glomerular filtration rate (ml/min/1.73m^2^); Hb, hemoglobin; Ht, height; KT, kidney transplantation; LEF, Leflunomide; MP, methylprednisolone; p-ANCA/MPO, perinuclear ANCA/myeloperoxidase; N, none or not detected; P, oral prednisone; *P*, percentile; PR, partial remission; Wt, weight.

For 7 patients, the diagnosis was evident from the patients’ renal biopsies. The remaining 2 patients were positive for both p-ANCA/MPO and cytoplasmic ANCA (c-ANCA)/proteinase 3 (PR3), but without pathology, and their diagnosis was based on the clinical manifestations of and the serological changes in their disease. Granuloma and other autoimmune diseases, such as Goodpasture syndrome and systemic lupus erythematosus (SLE), were excluded.

### Clinical and pathologic features

**Fig 1:**
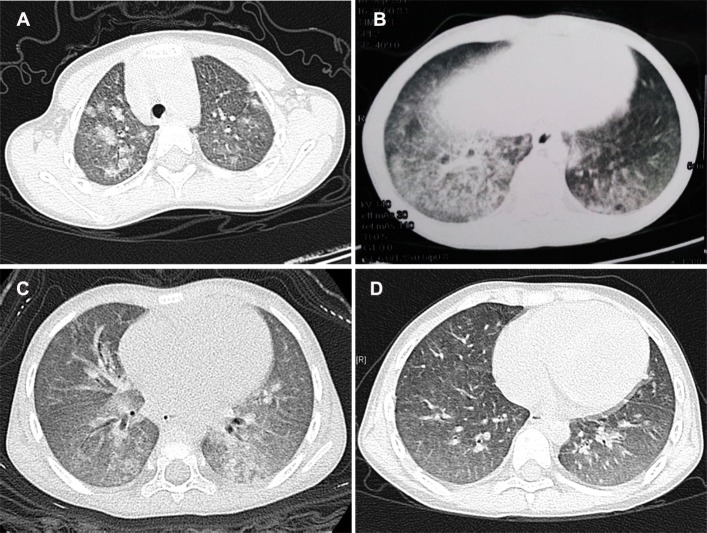
Lung CT of MPA patients with diffuse alveolar hemorrhage. (A): Shadows of gobbets (arrow) were observed in both lungs, and especially in the right one (patient 2). (B) and (C): Chest CT of patients 3 and 7, with ground glass opacity observed in the imaging. (D): Lung CT of patient 3 after 2 months of therapy, with the ground glass opacity having disappeared in contrast to 1B.

Of the 9 MPA patients, who all had pulmonary lesions, all presented with tachypnea, and 7 exhibited hemoptysis or blood-streaked sputum (Table 1). Additionally, 6 of the 9 patients had a computerized tomography (CT) finding of slightly and diffused increased density of pulmonary field (ground glass opacity), diffuse exfiltration or patchy shadows (Table 2 and Fig 1), and interstitial pneumonia, pneumonia accompanied with pleural effusion and a normal chest radiograph were each observed in one of the other 3 patients. Seven patients manifested with cough or hemoptysis as the initial symptom, 4 (patients 3, 4, 7 and 8) of whom had been diagnosed with idiopathic pulmonary hemosiderosis (IPH). One patient (patient 9) presented with hematuria and tachypnea simultaneously as the initial syndromes, and another (patient 6) first manifested with edema and renal insufficiency, with cough and hemoptysis emerging during the disease course.

**Table 2:** Summary of lab examination and pathology of 9 patients.

Patient	Hematuria/ proteinuria	p-ANCA/MPO, c-ANCA/PR3	ANA/ ds-DNA	C3 (0.77–1.95 g/l)	Scr (μmol/l)	BUN (mmol/l)	Hb (g/l)	Imaging of lungs (CT or X ray)	Renal pathology (Fibrinoid necrosis or exudation/ Crescents/Sclerosis)
1	-/-	+/+	-/-	1.46	22	4.8	106	Interstitial pneumonia	N
2	-/-	+/+	-/-	0.97	48	4.1	57	Multiple large patchy shadows	N
3	+/+	+/-	-/+	1.08	35	4.5	53	Ground glass opacity and patchy shadows	+/+/-
4	+/+	+/-	-/-	1.54	57	5.6	62	Diffuse exudation, ground glass	+/+/+
5	+/+	+/-	+/-	0.97	538	24.3	84	Multiple masses, with shadows in left lung and right lung	+/+/+
6	+/+	+/-	+/-	1.79	181	38.6	97	Pneumonia and pleural effusions	+/+/+
7	+/+	+/-	+/+	0.91	29	3.6	55	Ground glass opacity	+/+/+
8	+/+	+/-	+/+	0.94	74	13.2	50	Ground glass opacity	+/+/+
9	+/+	+/-	+/+	0.90	74	6.2	67	Normal	+/+/+

BUN, blood urine nitrogen; C3, complement 3; CT, computerized tomography; CTX, cyclophosphamide; Hb, hemoglobin; p-ANCA/MPO, perinuclear ANCA/myeloperoxidase; N, none or not detected; c-ANCA/PR3, cytoplasmic ANCA/proteinase 3; Scr, serum creatinine (The ranges for children has been previously reported[11]); +, positive;-, negative.

Renal involvement was observed in 7 patients, with a presentation of hematuria and proteinuria, and 3 of the 7 patients (patients 5–7) showed massive proteinuria that reached a nephrotic range (≥ 50 mg/kg/24 h; Table 2). Three patients (patients 5, 6 and 8) exhibited renal insufficiency at the time of diagnosis. All of the 7 patients with hematuria and proteinuria underwent a renal biopsy that showed histopathology typical of vasculitis: fibrinoid necrosis/exudation of the glomerular capillaries, as well as crescents and/or segmental or global sclerosis in the glomerular capillaries.

All of the 9 patients presented with normocytic normochromic anemia (Table 2): 2 patients had mild anemia, with hemoglobin (Hb) ≥ 90 g/L; 3 patients had moderate anemia, with Hb 60–90 g/L; and 4 patients had severe anemia, with Hb < 60 g/L, who exhibited underweight and/or stunting (patients 2, 3, 7 and 8). Five patients presented with irregular fever during the early disease course, without apparent infection. In addition, 3 presented with debility; 2, with myalgia; and one, with alimentary tract hemorrhage (accompanied with severe anemia and underweight/stunting).

### ANCA and other laboratory results

Positive p-ANCA/MPO results were observed in all of the patients (Table 2). Positive results for both p-ANCA/MPO and c-ANCA/PR3 were observed in 2 patients (patients 1 and 2), of whom lung is the only organ involved. Two of the patients were positive for ANA only, and one was positive for ds-DNA only, whereas 3 of the patients were positive for both ANA and ds-DNA. Eight cases had normal complement 3 (C3) levels, and one (patient 1) had an elevated C3 level.

### Treatment and prognosis

Of the 7 of the 9 patients who had renal involvement received steroid plus cyclophosphamide (CTX) induction therapy, 3 patients achieved complete remission, 3 achieved partial remission, and one was lost to follow-up. The other 2 patients (patients 1 and 2) with isolated pulmonary lesions received steroid plus leflunomide and also achieved complete remission. The median period time of follow-up was 10 month (0–86months) (Table 2).

For the 3 patients (patients 3, 4, and 7) who achieved complete remission, their routine urine tests returned to normal after 3 months, 4 months, or 8 months of treatment. Hb levels returned to normal and blood samples were ANCA negative after 11 months for patient 3 and after 8 months for patient 7, whereas patient 4 remained p-ANCA/MPO positive. Mycophenolate mofetil (MMF) was added for maintenance therapy after CTX induction treatment, during which a routine urine test was negative in 2 patients (patients 3 and 4). Patient 7 achieved complete remission after 8 months of therapy, with normal renal function, but hemoptysis, hematuria and proteinuria were recurrent during the maintenance treatment; complete remission was achieved again after a methylprednisolone pulse. Of the 3 patients achieved PR, 2 (patient 5 and patient 6) progressed to CKD at the beginning of diagnosis, respiratory symptoms were disappeared after 2 weeks in both 2 patients, ANCA turned negative after 4 months and 3 months, respectively; while anemia, hematuria and proteinuria were consistent and the patients progressed to CKD stage 5 finally (patient 5 underwent kidney transplantation at 10 months of treatment). The extra-renal symptoms (anemia and hemoptysis) of the other patient (patient 8) who achieved partial remission disappeared, and he became ANCA negative after 10 months of the regimen; however, his renal function did not recover (chronic kidney disease stage 3), with proteinuria but no hematuria. Respiratory symptoms of the 2 patients with isolated pulmonary lesions were disappeared after 2 weeks of treatment, and these 2 patients became ANCA negative after approximately one month (patient 1) or 4 months (patient 2) of treatment accompanied with restoration of anemia.

## Discussion

MPA is the most frequent form of ANCA-associated vasculitis in China and Asia. Compared with adult MPA, the morbidity of MPA in children is lower (1/10 that of adult MPA) [12]. Pediatric MPA exhibits a female predilection, and the mean age of onset is 10–12 years old [2]. In the present study, the median age of onset was approximately 6 years, and the earliest onset age was just 1.9 years old. Seven of the 9 patients were girls. All of these findings indicated that MPA with pulmonary involvement might occur in patients with an early onset age but deserves more study.

In children with MPA, the kidneys and lungs are the most frequent organs involved. It has been reported that renal involvement was observed in 100% of a sample of pediatric MPA patients, and pulmonary lesions were exhibited in 55–57% of these patients [2, 12]. And in China Mainland, reports of MPA are not common; the prevalence of pulmonary lesion of MPA is 50–60% according to the literatures [13, 14]. Hematuria and proteinuria are the primary manifestations of MPA [2, 15]. In the present study, most of the patients had renal lesions, with manifestations of hematuria and proteinuria. Mild-to-moderate proteinuria was frequently noted. However, only a few patients fulfilled the criteria for nephrotic syndrome, which are similar to those for adults [16]. The typical renal histopathology of MPA is pauci-immune glomerulonephritis with glomerular capillary necrosis and crescent formation [17]. In the present study, typical vasculitis lesions were evident in all of the patients biopsied. Crescents and sclerosis were also evident in the majority of the patients.

Diffuse alveolar hemorrhage is the most common presentation of lung involvement in children with MPA, which causes tachypnea, cough, hemoptysis, chest pain and anemia [18, 19]. slightly and diffused increased density of pulmonary field and patchy shadows are common imaging findings of lung involvement [20, 21]. In the present study, the clinical symptoms and chest imaging of the patients with pulmonary involvement were similar to those reported in the literature. Wegener’s granuloma (WG), IPH, MPA, Goodpasture syndrome and isolated pauci-immune pulmonary vasculitis are common causes of diffuse alveolar hemorrhage [22]. In contrast, MPA is the most frequent cause of pulmonary renal syndrome [23]. Therefore, differential diagnosis between these diseases can be challenging [24]. Moreover, pulmonary interstitial fibrosis, pulmonary infiltrates, pulmonary edema, and pleural effusion could also be observed in these patients [25]. The MPA patients with pulmonary interstitial fibrosis had a high mortality and a poor prognosis [26]. Furthermore, many of the patients in the present study had been diagnosed with IPH. These data suggest that differential diagnosis from IPH when patients exhibit isolated lung lesions can be challenging but important, as early diagnosis and standard treatment are key factors for improving MPA prognosis [15].

In addition, fever; skin involvement; joint, eye and ear damage; and digestive system involvement can also be observed in patients with MPA. In the present study, fever was present in 5 patients, which was consistent with fever being common in pediatric MPA [10] and which indicated that a differential diagnosis should be made between lung infection and the pulmonary lesions of MPA.

ANCAs are predominantly IgG autoantibodies directed against constituents of the primary granules of neutrophils and monocytes’ lysosomes [27]. c-ANCA/PR3 exists in most WG patients, and p-ANCA/MPO is specific for MPA [28]. c-ANCA/PR3 can also be observed in certain MPA patients. ANCAs could be used as an index for assessing MPA relapse [29]. These changes were noted in our study. The majority of the patients were only positive for p-ANCA/MPO, and 5 of the 6 who achieved various degrees of remission became ANCA negative after 1–11 months. Of the two patients who were positive for p-ANCA/MPO and c-ANCA/PR3, only pulmonary lesion and anemia were observed. Whether patients with positive of both types of ANCAs trend to isolated pulmonary lesion deserves further observation. Approximately half of the patients in our study were positive for ANA and/or ds-DNA but had normal levels of C3, which indicated that ANA and dsDNA positivity was prevalent in the MPA patients.

Steroid plus cytotoxic agents constitutes an effective therapy for MPA and can improve the survival rate of MPA [30]. The treatments for MPA in children are mainly the same as those in adults [31]. CTX is the most frequently used cytotoxic drug for MPA induction treatment. The effective rate and CR rate of CTX in children are 71.4% and 28.5%, respectively [12]. Many other types of drugs are used for maintenance therapy, such as MMF, azathioprine or a continuous CTX pulse. For severe organ involvement (e.g., necrotizing glomerulonephritis, rapidly progressive glomerulonephritis, diffuse alveolar hemorrhage), plasmapheresis is an essential therapy [32]. In the present study, 6 of the 7 patients who received CTX induction therapy achieved various degrees of remission; our previous study also showed efficiency of CTX in pediatric MPA [10]. In recent years, leflunomide has been used in treating WG and MPA and is superior even to azathioprine [33–35]. Furthermore, leflunomide appears to be safe and well tolerated among patients, with fewer side effects, such as drug accumulation and leukopenia, than other cytotoxic agents [36]. In the present study, steroid plus leflunomide treatment was effective in 2 patients with isolated pulmonary lesions. However, the relationships between onset age and response to therapy and prognosis were not observed.

## Conclusion

In conclusion, diffuse alveolar hemorrhage is the most frequent manifestation in pediatric MPA patients with pulmonary lesions, with the common phenotype being tachypnea, cough, hemoptysis and anemia. The majority of these patients exhibit renal involvement, such as hematuria, proteinuria and renal insufficiency. The efficacy of corticosteroid plus CTX treatment was evident in the induction therapy for these patients and steroid plus leflunomide was also effective in patients with isolated pulmonary lesions.

## Supporting Information

S1 Dataset Normal levels of serum creatinine between different ages were described in the “Supporting Information”, which was the pediatric textbook of high education. (PDF)
