# Supplementary material for: Clinical Features of Children with Pulmonary Microscopic Polyangiitis: Report of 9 Cases
Source: PLoS One. 2015 Apr 29;10(4):e0124352. doi: 10.1371/journal.pone.0124352 (PMC4414499; doi:10.1371/journal.pone.0124352)
Supplement: S1 Dataset — (PDF) [file pone.0124352.s001.pdf]

卫生部“十一五”规划教材

全国高等医药教材建设研究会规划教材

全国高等学校教材

供基础、临床、预防、口腔医学类专业用

儿科学

第7版

主　编　沈晓明　王卫平

副主编　常立文　李廷玉　申昆玲

人民卫生出版社

第一章　绪　论

第一节 儿科学的范围和任务

儿科学属临床医学的二级学科，其研究对象是自胎儿至青春期的儿童，研究内容可以分为以下四个方面：

1．研究儿童生长发育的规律及其影响因素，不断提高儿童体格、智力发育水平和社会适应性能力。

2．研究儿童各种疾病的发生、发展规律以及临床诊断和治疗的理论和技术，不断降低疾病的发生率和死亡率，提高疾病的治愈率。

3．研究各种疾病的预防措施，包括免疫接种、先天性遗传性疾病的筛查、科学知识普及教育等，这是现代儿科学最具有发展潜力的内容，将会占据越来越重要的地位。

4．研究儿童中各种疾病的康复可能性以及具体方法，尽可能地帮助这些儿童提高他们的生活质量乃至完全恢复健康。

以上研究内容归结而言就是儿科学的宗旨：保障儿童健康，提高生命质量。

随着医学研究的进展，儿科学也不断向更深入专业的三级学科细化发展，同时也不断派生出新的专业。儿科学的三级学科分支类似内科学，主要以系统划分，如呼吸、消化、循环、神经、血液、肾脏、内分泌等。此外，还有传染病和急救医学等特殊专业。小儿外科学则为外科学下的三级学科。上述学科虽然在分类上与内科学相似，但是其研究内容及内在规律与成人差别颇大，应予以注意，不能混淆或替代。

新生儿医学和儿童保健医学是儿科学中最具特色的学科，其研究内容是其他临床学科极少涉及的方面：新生儿期的死亡率仍然非常高，占婴儿死亡率的60％～70％，此期疾病的种类和处理方法与其他时期有诸多不同，是一个非常时期；儿童保健医学是研究儿童各时期正常体格生长、智力和心理发育规律及其影响因素的学科，通过各种措施，促进有利因素，防止不利因素，及时处理各种偏离、异常，保证儿童健康成长。由于某些年龄阶段的儿童具有特殊的临床特点，近年来发展出了围生期医学。围生期医学实际上是介于儿科学和妇产科学间的边缘学科，一般指胎龄28周至出生后不满1周的小儿，由于此期受环境因素影响颇大，发病率和死亡率最高，而且与妇产科的工作有密切联系，需要两个学科的积极合作来共同研究处理这一时期的问题。随着医学科学和技术的不断发展，儿科学必将向各个分支纵深分化，新的学科、边缘性的学科必将继续应运而生。然而，儿科学的分化发展趋势绝不是儿科学自身的肢解终结，在学习和研究儿科学某一分支学科时，切不可忽略对儿科学基础和学科总体的潜心研究和关注。

第二节 儿科学的特点

与其他临床学科相比，儿科学有其不同的特点，这些特点产生的根本原因在于儿科学研究的对象是儿童。儿童时期是机体处于不断生长发育的阶段，因此表现出的基本特点有三方面：①个体差异、性别差异和年龄差异都非常大，无论是对健康状态的评价，还是对疾病的临床诊断都不宜用单一标准衡量。②对疾病造成损伤的恢复能力较强，常常在生长发育的过程中对比较严重损伤的转归可以为自然改善或完全修复，因此，只要度过危重期，常可满意恢复，适宜的康复治疗常有事半功倍的效果。③自身防护能力较弱，易受各种不良因素影响导致疾病发生和性格行为的偏离，而且一旦造成损伤，往往影响一生，因此应该特别注重预防保健工作。下

面从基础和临床两个方面具体说明儿科学的主要特点。

一、基础医学方面

1．解剖 随着体格生长发育的进展，身体各部位逐渐长大，头、躯干和四肢的比例发生改变，内脏的位置也随年龄增长而不同，如肝脏右下缘位置在3岁前可在右肋缘下2cm内，3 岁后逐渐抬高，6～7岁后在正常情况下不应触及。在体格检查时必须熟悉各年龄儿童的体格生长发育规律，才能正确判断和处理临床问题。

2．机能 各系统器官的机能也随年龄增长逐渐发育成熟，因此不同年龄儿童的生理、生化正常值各自不同，如心率、呼吸频率、血压、血清和其他体液的生化检验值等。此外，某年龄阶段的功能不成熟常是疾病发生的内在因素，如婴幼儿的代谢旺盛，营养的需求量相对较高，但是此时期胃肠的消化吸收功能尚不完善，易发生消化不良。因此，熟悉掌握各年龄儿童的机能变化特点是儿科临床工作的基本要求。

3．病理 对同一致病因素，儿童与成人的病理反应和疾病过程会有相当大的差异，即或是不同年龄的儿童之间也会出现这种差异，如由肺炎球菌所致的肺炎，婴儿常表现为支气管肺炎，而成人和年长儿则可引起大叶性肺炎病变。

4．免疫 小年龄儿童的非特异性免疫、体液免疫和细胞免疫功能都不成熟，因此抗感染的能力比成人和年长儿低下，如婴幼儿时期slgA和IgG水平均较低，容易发生呼吸道和消化道感染。因此适当的预防措施对小年龄儿童特别重要。

5．心理 儿童时期是心理、行为形成的基础阶段，可塑性非常强。及时发现小儿的天赋气质特点，并通过训练予以调适；根据不同年龄儿童的心理特点，提供合适的环境和条件，给予耐心的引导和正确的教养，可以培养儿童良好的个性和行为习惯。

二、临床方面

1．疾病种类 儿童疾病发生的种类与成人有非常大的差别，如心血管疾病，在儿童中主要以先天性心脏病为主，而成人则以冠状动脉心脏病为多；儿童白血病中以急性淋巴细胞性白血病占多数，而成人则以粒细胞性白血病居多。此外，不同年龄儿童的疾病种类也有相当差异，如新生儿疾病常与先天遗传和围生期因素有关，婴幼儿疾病中感染性疾病占多数等。

2．临床表现 儿科患者在临床表现方面的特殊性主要集中在小年龄儿童，年幼体弱儿对疾病的反应差，往往表现为体温不升、不哭、纳呆、表情淡漠，且无明显定位症状和体征。婴幼儿易患急性感染性疾病，由于免疫功能不完善，感染容易扩散甚至发展成败血症，病情发展快，来势凶险。因此儿科医护人员必须密切观察病情，随时注意病情的细微变化，不轻易放过任何可疑表现。

3．诊断 儿童对病情的表述常有困难且不准确，但仍应认真听取和分析，同时必须详细倾听家长陈述病史。全面准确的体格检查对于儿科的临床诊断非常重要，有时甚至是关键性的。发病的年龄和季节，以及流行病学史往往非常有助于某些疾病的诊断。不同年龄儿童的检验正常值常不相同，应该特别注意。

4．治疗 儿科的治疗应该强调综合治疗，不仅要重视对主要疾病的治疗，也不可忽视对各类并发症的治疗，有时并发症可能是致死的原因；不仅要进行临床的药物治疗，还要重视护理和支持疗法。小儿的药物剂量必须按体重或体表面积仔细计算，并且要重视适当的液体出入量和液体疗法。

5．预后 儿童疾病往往来势凶猛，但是如能及时处理，度过危重期后，恢复也较快，且较

少转成慢性或留下后遗症。因此，临床的早期诊断和治疗显得特别重要，适时正确的处理不仅有助于患儿的转危为安，也有益于病情的转归预后。

6．预防 已有不少严重威胁人类健康的急性传染病可以通过预防接种得以避免，此项工作基本上是在儿童时期进行，是儿科工作的重要方面。目前许多成人疾病或老年性疾病的儿童期预防已经受到重视，如动脉粥样硬化引起的冠状动脉心脏病、高血压和糖尿病等都与儿童时期的饮食有关；成人的心理问题也与儿童时期的环境条件和心理卫生有关。

第三节 小儿年龄分期

儿童的生长发育是一个连续渐进的动态过程，不应被人为地割裂认识。但是在这个过程中，随着年龄的增长，儿童的解剖、生理和心理等功能确实在不同的阶段表现出与年龄相关的规律性。因此，在实际工作中将其分为七期，以便熟悉掌握。

（一）胎儿期

从受精卵形成到胎儿出生为止，共40周。胎儿的周龄即为胎龄，或称为妊娠龄。母亲妊娠期间如受外界不利因素影响，包括感染、创伤、滥用药物、接触放射性物质、毒品等，以及营养缺乏、严重疾病和心理创伤等都可能影响胎儿的正常生长发育，导致流产、畸形或宫内发育不良等。

（二）新生儿期

自胎儿娩出脐带结扎至28天之前，按年龄划分，此期实际包含在婴儿期内。由于此期在生长发育和疾病方面具有非常明显的特殊性，且发病率高，死亡率也高，因此单独列为婴儿期中的一个特殊时期。在此期间，小儿脱离母体转而独立生存，所处的内外环境发生根本的变化，但其适应能力尚不完善。此外，分娩过程中的损伤、感染延续存在，先天性畸形也常在此期表现。

（三）婴儿期

自出生到1周岁之前为婴儿期。此期是生长发育极其迅速的阶段，因此对营养的需求量相对较高。此时，各系统器官的生长发育虽然也在继续进行，但是不够成熟完善，尤其是消化系统常常难以适应对大量食物的消化吸收，容易发生营养和消化紊乱。同时，婴儿体内来自母体的抗体逐渐减少，自身的免疫功能尚未成熟，抗感染能力较弱，易发生各种感染和传染性疾病。

（四）幼儿期

自1岁至满3周岁之前为幼儿期。体格生长发育速度较前稍减慢，而智能发育迅速，同时活动范围渐广，接触社会事物渐多。此阶段消化系统功能仍不完善，营养的需求量仍然相对较高，而断乳和其他食物添加须在此时完成，因此适宜的喂养仍然是保持正常生长发育的重要环节。此期小儿对危险的识别和自我保护能力都有限，因此意外伤害发生率非常高，应格外注意防护。

（五）学龄前期

自3周岁至6～7岁入小学前为学龄前期。此时体格生长发育速度已经减慢，处于稳步增长状态；而智能发育更加迅速，与同龄儿童和社会事物有了广泛的接触，知识面能够得以扩大，自理能力和初步社交能力能够得到锻炼。

（六）学龄期

自入小学始（6～7岁）至青春期前为学龄期。此期儿童的体格生长速度相对缓慢，除生殖系统外，各系统器官外形均已接近成人。智能发育更加成熟，可以接受系统的科学文化教育。

（七）青春期

青春期年龄范围一般从10岁～20岁，女孩的青春期开始年龄和结束年龄都比男孩早2 年左右。青春期的进入和结束年龄存在较大个体差异，约可相差2～4岁。此期儿童的体格生长发育再次加速，出现第二次高峰，同时生殖系统的发育也加速并渐趋成熟。

第四节 儿科学的发展与展望

与西方医学比较而言，我国的中医儿科起源要早得多，自扁鹊“为小儿医”以来已有2400余年，自宋代钱乙建立中医儿科学体系以来也有近900年。此前在唐代已在太医署正规培养5年制少小科专科医生，隋、唐时代已有多部儿科专著问世，如“诸病源候论”和“小儿药证直诀”等，收集论述小儿杂病诸候6卷255候，建立了中医儿科以五脏为中心的临床辨证方法。16世纪中叶发明的接种人痘预防天花的方法比欧洲发明牛痘接种早百余年。进入19世纪后，西方儿科学发展迅速，并随着商品和教会进入我国。

20世纪30年代西医儿科学在我国开始受到重视，至20世纪40年代儿科临床医疗规模初具，当时的工作重点在于诊治各种传染病和防治营养不良。由于儿科人才日趋紧缺，儿科学教育应运而生。1943年，我国现代儿科学的奠基人诸福棠教授主编的《实用儿科学》首版问世，成为我国第一部大型儿科医学参考书，标志着我国现代儿科学的建立。

自19世纪至20世纪末，西方儿科学的重大贡献主要在于有效地防治传染病和营养不良方面，两者为当时儿童中死亡的首要原因。对预防多种传染病疫苗的研制成功使得儿童常见传染病的发生率明显下降，婴儿死亡率逐年降低。同时，由于抗生素的不断发展和广泛应用，儿童中感染性疾病的发病率和死亡率也大幅度地下降。代乳食品和配方乳的研究和提供曾经拯救了大量儿童的生命，近年来大力提倡母乳喂养使得儿童的生长发育水平更加提高。

中华人民共和国成立以后，党和政府对于儿童的医疗卫生事业非常关心。在城乡各地建立和完善了儿科的医疗机构，并且按照预防为主的方针在全国大多数地区建立起儿童保健机构，同时普遍办起了各种形式的托幼机构。这些机构对于保障我国儿童的健康和提高儿童的生命质量起了至关重要的作用。通过这些机构，儿童的生长发育监测、先天性遗传性疾病的筛查、疫苗的接种、“四病”的防治得以落实，儿童中常见病、多发病能够得到及时的诊治（图1－1、图1－2）。

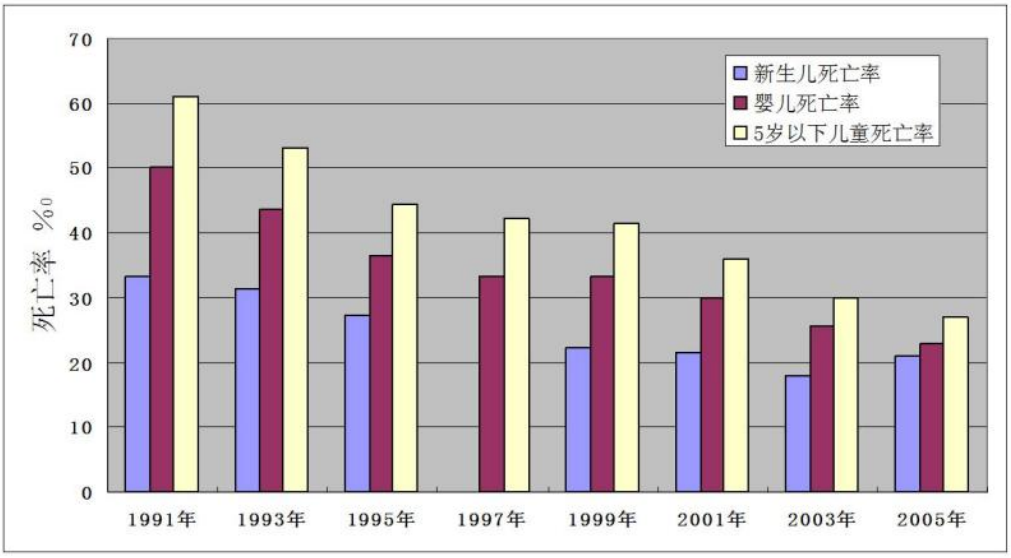

图1-1 1991～2005年我国监测地区5岁以下儿童死亡率（‰）

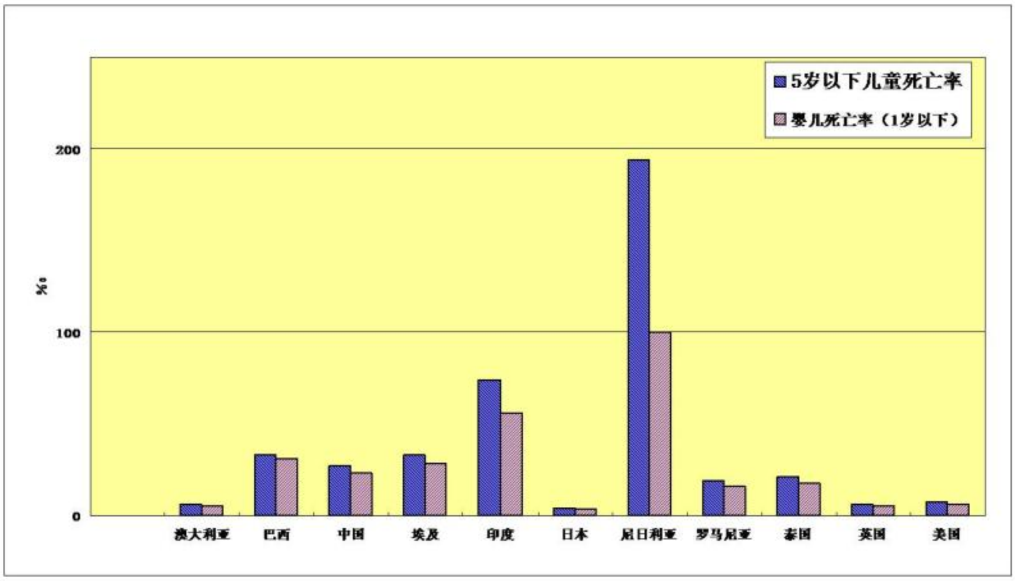

图1-2　我国婴儿死亡率和5岁以下儿童死亡率与其他国家的比较

摘自联合国儿童基金会《2006年统计报告》

尽管我国儿童目前的主要健康问题从总体上看还集中在感染性和营养性疾病等常见病、多发病方面，但是与20世纪比较而言，这些疾病的发生率和严重性大大降低；并且在某些发达地区，严重的营养不良和急性传染病已经少见。这些疾病谱的变化昭示我国儿科工作者的注意力应该开始向新的领域发展延伸，儿科学的任务不仅要着重降低发病率和死亡率，更应该着眼于保障儿童健康，提高生命质量的远大目标。因此，研究儿童正常生长发育规律及其影响因素的儿童保健学应该受到重视，儿童保健的临床服务应该由大城市逐渐普及到中小城市和乡村，以保证儿童的体格生长、心理健康、智能发育和社会应对能力得到全面均衡的发展。同时，研究儿童罹患各种疾病后得以尽量完善恢复的儿童康复医学应该受到重视，儿童时期疾患的后遗症将可能影响今后一生的健康和幸福，而处于生长发育阶段的儿童具有非常强的修复和再塑能力，在适宜的康复治疗下往往可能获得令人难以想象的效果。此外，某些成人疾病的儿童期预防应该受到重视，疾病预防的范围不应仅局限于对感染性疾病，许多疾病在成人后（或在老年期）出现临床表现，实际上发病的过程在儿童期已经开始，如能在儿童期进行早期预防干预，就可能防止或延缓疾病的发生、发展。

对儿科学的研究和探索是依托现代医学进展的大背景展开的。当前，现代医学的几个革命性突破及其引领的发展趋势应该受到儿科工作者的高度重视。迄今为止，虽然对于外部因素致病为主导的创伤、感染性等疾病研究取得令人瞩目的进展，但是对致病基因等内部致病因素研究相对滞后，这是目前疾病谱中肿瘤、心脑血管疾病和代谢性疾病居高不下的基本原因。著名的诺贝尔生理学与医学奖获得者杜伯克曾说："人类的DNA序列是人类的真谛，这个世界上发生的一切事情都与这一序列息息相关，包括癌症在内的人类疾病的发生都与基因直接或间接相关……"。2005年人类基因组DNA全序列测定最终完成，对于人类攻克目前威胁生命健康的疑难顽症具有里程碑的意义。值得注意的是，基因组学虽然在基因活性和疾病的相关性为破解疾病发生、发展的本源提供了有力根据和方向，但并没有解决攻克疾病的所有问题。随之后基因组学的研究应运而生并不断取得进展，从目前的结果看，后基因组学的研究内容是相当广泛的。已经证实，大部分疾病并不仅因为基因改变所造成，还与基因的表达相关，而基因的表达方式又非常错综复杂，成为研究人类健康问题的新方向。此外，人体内真正发挥生理功能的是蛋白质，所谓蛋白质组学，就是要研究清楚人类体内存在的全部蛋白质的种类、结构、结构与功能

的关系、各种蛋白质之间的相互关系以及蛋白质异常的致病性。因此，蛋白质组学的研究是突破重大疾病问题的重要方向。最近发现，除了DNA上的遗传密码，在染色质中与DNA结合的组蛋白也具有所谓的组蛋白密码。这类组蛋白密码也可以遗传，但不像DNA上的遗传密码那么稳定，它受到各种环境因素影响，可能反映了生命体对环境适应的一种机制，这就是表现遗传学。表观遗传学通过研究环境因素对基因遗传的作用，可以更详尽地了解疾病发生、发展的过程。在后基因组学发展的基础上，系统生物医学已经诞生。系统生物医学将基因组和蛋白质组水平的各种因素的相互作用、代谢途径及调控途径综合起来，运用现代生物学的科学和技术，解析人类的行为和疾病发生的根本原因。系统生物学必将极大地提升医学研究的能力。

上述现代医学的重大研究进展对儿科学的进展将是影响最大的，因为这些研究必将涉及人类生命和健康的本质性问题，儿科学正是在这些问题的源头上。

（王卫平）

人的生长发育是指从受精卵到成人的成熟过程。生长和发育是儿童不同于成人的重要特点。生长是指儿童身体各器官、系统的长大，可有相应的测量值来表示其的量的变化；发育是指细胞、组织、器官的分化与功能成熟。生长和发育两者紧密相关，生长是发育的物质基础，生长的量的变化可在一定程度上反映身体器官、系统的成熟状况。

第一节 生长发育规律

生长发育，不论在总的速度上或各器官、系统的发育顺序，都遵循的一定规律。认识总的规律性有助于儿科医生对儿童生长发育状况的正确评价与指导。

1．生长发育是连续的、有阶段性的过程 生长发育在整个儿童时期不断进行，但各年龄阶段生长发育有一定的特点，不同年龄阶段生长速度不同。例如，体重和身长在生后第1年，尤其前3个月增加很快，第1年为生后的第一个生长高峰；第2年以后生长速度逐渐减慢，至青春期生长速度又加快，出现第二个生长高峰。

2．各系统器官生长发育不平衡 人体各器官系统的发育顺序遵循一定规律。如神经系统发育较早，脑在生后2年发育较快；淋巴系统在儿童期迅速生长，于青春期前达高峰，以后逐渐下降；生殖系统发育较晚。其它系统如心、肝、肾、肌肉的发育基本与体格生长相平行（图2-1）。这种各系统发育速度的不同与其在不同年龄的生理功能有关。

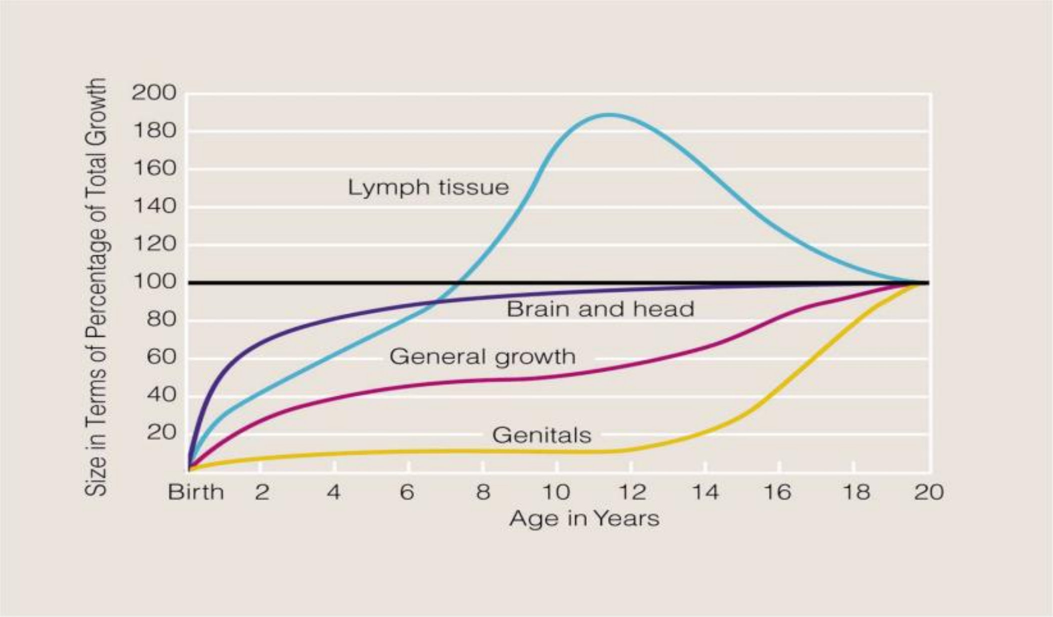

图2-1 各系统器官发育不平衡

3．生长发育的个体差异 儿童生长发育虽按一定总规律发展，但在一定范围内受遗传、环境的影响，存在着相当大的个体差异，每个人生长的“轨道”不会完全相同。因此，儿童的生长发育水平有一定的正常范围，所谓的正常值不是绝对的，评价时必须考虑个体的不同的影响因素，才能作出正确的判断。

4．生长发育的一般规律 生长发育遵循由上到下、由近到远、由粗到细、由低级到高级、由简单到复杂的规律。如出生后运动发育的规律是：先抬头、后抬胸，再会坐、立、行（从上到下）；从臂到手，从腿到脚的活动（近到远）；从全掌抓握到手指拾取（从粗到细）；先画直

线后画圈、图形（简单到复杂）；先会看、听、感觉事物，认识事物，发展到有记忆、思维、分析、判断（低级到高级）。

第二节 影响生长发育的因素

一、遗传因素

细胞染色体所载基因是决定遗传的物质基础。父母双方的遗传因素决定小儿生长发育的“轨道”，或特征、潜力、趋向。种族、家族的遗传信息影响深远，如皮肤、头发的颜色、面型特征、身材高矮、性成熟的迟早、对营养素的需要量、对传染病的易感性等。在异常情况下，严重影响生长的遗传代谢缺陷病、内分泌障碍、染色体畸形等，更与遗传直接有关。

二、环境因素

1．营养 儿童的生长发育，包括宫内胎儿生长发育，需充足的营养素供给。当营养素供给比例恰当，加之适宜的生活环境，可使生长潜力得到最好的发挥。宫内营养不良的胎儿不仅体格生长落后，严重时还影响脑的发育；生后营养不良，特别是第1～2年的严重营养不良，可影响体重、身高及智能的发育。

2．疾病 疾病对生长发育的阻扰作用十分明显。急性感染常使体重减轻；长期慢性疾病则影响体重和身高的发育；内分泌疾病常引起骨骼生长和神经系统发育迟缓；先天性疾病，如先天性心脏病可造成生长迟缓。

3．母亲情况 胎儿在宫内的发育受孕母生活环境、营养、情绪、疾病等各种因素的影响。母亲妊娠早期的病毒性感染可导致胎儿先天畸形；妊娠期严重营养不良可引起流产、早产和胎儿体格生长以及脑的发育迟缓；妊娠早期受到某些药物、X线照射、环境中毒物和精神创伤的影响，均可影响胎儿的发育。

4．家庭和社会环境 家庭环境对儿童健康的重要作用易被家长和儿科医生忽视。良好的居住环境，如阳光充足、空气新鲜、水源清洁、无噪声、无噪光、居住条件舒适，配合良好的生活习惯、科学护理、良好教养、体育锻炼、完善的医疗保健服务等都是促进儿童生长发育达到最付佳状态的重要因素。近年来，社会环境对儿童健康的影响引起高度关注。自两伊战争以来，伊拉克儿童健康状况急剧下降是社会环境影响儿童健康的最好例证。

综上所述，遗传决定了生长发育的潜力，这种潜力从受精卵开始就受到环境因素的作用与调节，表现出个人的生长发育模式。因此，生长发育水平是遗传与环境的共同作用的结果。

第三节 体格生长

一、体格生长常用指标

体格生长应选择易于测量、有较大人群代表性的指标来指示。一般常用的形态指标有体重、身高（长）、坐高（顶臀长）、头围、胸围、上臂围、皮下脂肪等。

二、出生至青春前期的体格生长规律

（一）体重的增长

体重为各器官、系统、体液的总重量。其中骨骼、肌肉、内脏、体脂、体液为主要成分。因体脂与体液变化较大，体重在体格生长指标中最易波动。体重易于准确测量，是最易获得的

反映儿童生长与营养状况的指标。儿科临床中用体重计算药量、静脉输液量。

新生儿出生体重与胎次、胎龄、性别以及宫内营养状况有关。我国2005年九市城区调查结果显示平均男婴出生体重为3.33±0.39kg，女婴为3.24±0.39kg，与世界卫生组织（WHO）的参考值相近（男3.3kg，女3.2kg）。出生后体重增长应为胎儿宫内体重生长的延续。生后一周内因奶量摄入不足，加之水分丢失、胎粪排出，可出现暂时性体重下降或称生理性体重下降，约在生后3～4日达最低点，下降范围为3％～9％，以后逐渐回升，至出生后第7～10日应恢复到出生时的体重。如果体重下降超过10％或至第10天还未恢复到出生时的体重，则为病理状态，应分析其原因。如生后及时合理喂哺，可减轻或避免生理性体重下降的发生。出生时体重受宫内因素的影响大，生后的体重与营养、疾病等因素密切相关。

随年龄的增加儿童体重的增长逐渐减慢。我国1975年、1985年、1995年及2005年调查资料显示，正常足月婴儿生后第1个月体重增加可达1～1.7kg，生后3～4个月体重约等于出生时的体重的2倍（附录一）；第1年内婴儿前3个月体重的增加值约等于后9个月内体重的增加值，即122+8月龄时婴儿体重约为出生时的3倍（10kg），是生后体重增长最快的时期，系第一个生长高峰；生后第2年体重增加2.5～3.5kg；2岁至青春前期体重增长减慢，年增长值约2kg。

儿童体重的增长为非等速的增加，进行评价时应以个体儿童自己体重增长的变化为依据，不可用“公式”计算来评价，也不宜以人群均数（所谓“正常值”）当作“标准”看待。当无条件测量体重时，为便于医务人员计算小儿用药量和液体量，可用以下公式估计体重（表2-1）：

表2-1　正常儿童体重、身高估计公式

年龄	体重（kg）
12个月	10
1～12岁	年龄（岁）×2+8
**年龄**	**身高（cm）**
12个月	75
2～12岁	年龄（岁）×7+75

（二）身材的增长

1．身高（长）　身高指头部、脊柱与下肢长度的总和。多数3岁以下儿童立位测量不易准确，应仰卧位测量，称为身长。立位时测量称为身高。立位的测量值比仰卧位少1～2cm。

身高（长）的增长规律与体重相似。年龄越小增长越快，也出现婴儿期和青春期两个生长高峰。出生时身长平均为50cm，生后第1年身长增长最快，约为25cm；前3个月身长增长约11～12cm，约等于后9个月的增长值，1岁时身长约75cm；第2年身长增长速度减慢，约10～12cm，即2岁时身长约87cm；2岁以后身高每年增长6～7cm。2岁以后每年身高增长低于5cm，为生长速度下降。

身高（长）的生长受遗传、内分泌、宫内生长水平的影响较明显，短期的疾病与营养波动不易影响身高（长）的生长。

2．坐高（顶臀长）　是头顶到坐骨结节的长度。3岁以下儿童仰卧位测量为顶臀长。坐高增长代表头颅与脊柱的生长。

3．指距　是两上肢水平伸展时两中指尖距离，代表上肢长骨生长。

（三）头围的增长

头围的增长与脑和颅骨和生长有关。胎儿期脑生长居全身各系统的领先地位，故出生时头围相对大，平均32～34cm；与体重、身长增长相似，第1年前3个月头围的增长（6cm）约等于后9个月头围的增长值（6cm），即1岁时头围约为46cm；生后第2年头围增长减慢，约为2cm；2岁时头围约48cm；2～15岁头围仅增加6～7cm。头围的测量在2岁以内最有价值。

婴幼儿期连续追踪测量头围比一次测量更重要。头围大小与双亲的头围有关；头围＜ $\overline{X}-2SD$ 常提示有脑发育不良的可能，＜ $\overline{X}-3SD$ 以上常提示脑发育不良；头围增长过速往往提示脑积水。

（四）胸围的增长

胸围代表肺与胸廓的生长。出生时胸围32cm，略小于头围1～2cm。1岁左右胸围约等于头围。1岁至青春前期胸围应大于头围（约为头围+年龄-1cm）。1岁左右头围与胸围的增长在生长曲线上形成头、胸围的交叉，此交叉时间与儿童营养、胸廓的生长发育有关，生长较差者头、胸围交叉时间延后。我国2005年9市城区体格生长的衡量数字显示男童头、胸围交叉时间为15个月龄，提示我国儿童胸廓生长较落后，除营养因素外，可能与不重视爬的训练和胸廓锻炼有关。

（五）上臂围的增长

上臂围代表肌肉、骨骼、皮下脂肪和皮肤的生长。1岁以内上臂围增长迅速，1～5岁增长缓慢，约1～2cm。因此，有人认为在无条件测体重和身高的地方，可用左上臂围测量筛查5岁以下儿童营养状况：>13.5cm为营养良好；12.5～13.5cm为营养中等；<12.5cm为营养不良。

（六）皮下脂肪

通过测量皮脂厚度反映皮下脂肪。常用的测量部位有：①腹壁皮下脂肪；②背部皮下脂肪。要用皮下脂肪测量工具（测皮褶卡钳）测量才能得出正确的数据。

（七）身体比例与匀称性

在生长过程中，身体的比例与匀称性生长有一定规律。

1．头与身长比例　在宫内与婴幼儿期头领先生长，而躯干、下肢生长则较晚，生长时间也较长。这样，头、躯干、下肢长度的比例在生长进程中发生变化。头长占身长（高）的比例在婴幼儿为1/4，到成人后为1/8（图2-2）。

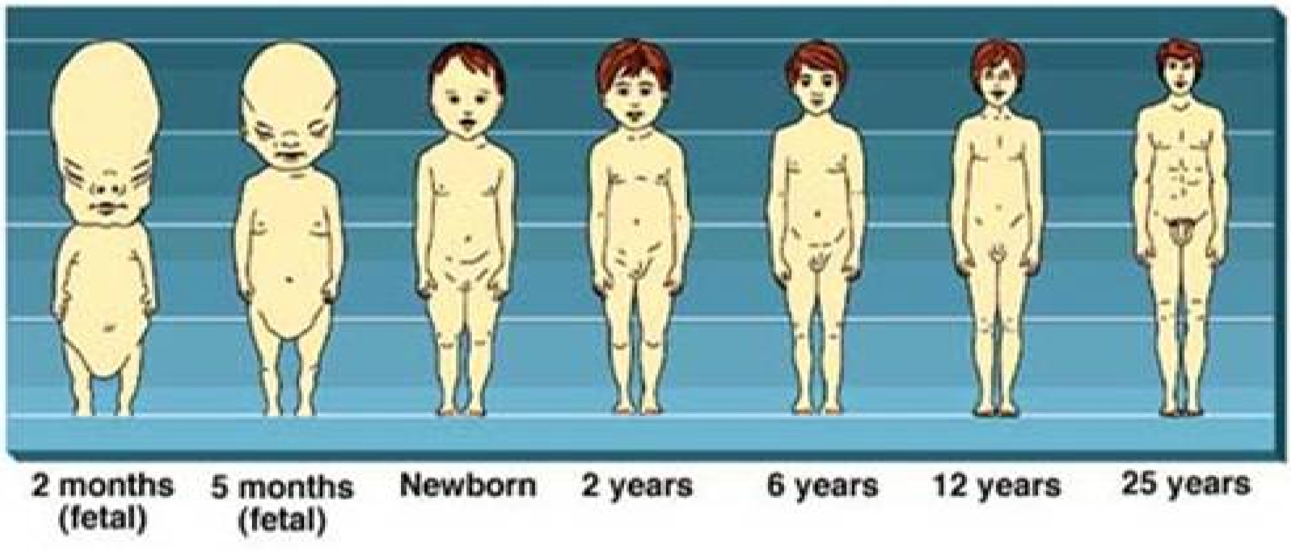

图2-2　头与身长的比例

2．体型匀称 表示体型（形态）生长的比例关系，如身高的体重（Weight-for height，W/H）；胸围/身高（身高胸围指数）；体重（kg）/身高（cm）×1000（Quetelet指数），体重（kg）/身高（cm）^{2}×10^4（Kaup指数，幼儿用），年龄的体块指数（BMI/age）等。

3．身材匀称 以坐高（顶臀长）与身高（长）的比例表示，反映下肢的生长情况。坐高（顶臀长）占身高（长）的比例由出生时的0.67下降到14岁时的0.53。

任何影响下肢生长的疾病，可使坐高（顶臀长）与身高（长）的比例停留在幼年状态，如甲状腺功能低下与软骨营养不良。

4．指距与身高 正常时，指距略小于身高（长）。如指距大于身高1～2cm，对诊断长骨的异常生长有参考价值，如蜘蛛样指（趾）（马凡综合征）。

三、青春期的体格生长规律

青春期是儿童到成人的过渡期，受性激素等因素的影响，体格生长出现生后的第二个高峰（peak height velocity，PHV），有明显的性别差异。男孩的身高增长高峰约晚于女孩2年，且每年身高的增长值大于女孩，因此最终的身高一般来说男孩比女孩高。一般的说男孩骨龄15岁，女孩骨龄13岁时，身高长度达最终身高的95％。

不论男女孩，在青春期前的1～2年中生长速度略有减慢。女孩在乳房发育后（约9～11岁），男孩在睾丸增大后（11～13岁）身高开始加速生长，1～2年生长达PHV，此时女孩年身高平均年增加8～9cm，男孩9～10cm。在第二生长高峰期，身高增加值约为最终身高的15％。PHV提前者，身高的停止增长较早。

青春期体重的增长与身高平行，同时内脏器官增长。女性耻骨与髂骨下部的生长与脂肪堆积，臀围加大。男性则有肩部增宽，下肢较长，肌肉增强的不同体形特点。

四、体格生长评价

儿童处于快速生长发育阶段，身体形态及各部分比例变化较大。充分了解儿童各阶段生长发育的规律、特点，正确评价儿童生长发育状况，及早发现问题，给予适当的指导与干预，对促进儿童的健康生长十分重要。

（一）资料分析及表示方法

1．衡量体格生长的统计学表示方法 常用以下方法：

（1）均值离差法：正常儿童生长发育状况多呈正态分布，常用均值离差法，以平均值（$\overline{X}$）加减标准差（*SD*）来表示，如68.3％的儿童生长水平在$\overline{X}\pm1SD$范围内；95.4％的儿童在$\overline{X}\pm2SD$范围内；99.7％的儿童在$\overline{X}\pm3SD$ 范围内。

（2）百分位数法：当测量值呈偏正态分布时，百分位数法能更准确的反映所测数值的分布情况。当变量呈正态分布时，百分位数法与离差法两者相应数相当接近。由于样本常呈偏正态分布，则两者的相应数值略有差别。

在体格生长评价时两者都广泛应用，目前一般都用百分位法。离差法计算较简单；百分位数法计算相对较复杂，但精确。

（3）标准差的离差法（Z积分或Z score，SDS）：可进行不同质人群间比较，用偏离该年龄组标准差的程度来反映生长情况，结果表示也较精确。

$$\text{Z score}=\frac{X-\overline{X}}{SD}$$

其中，*X*为测得值，$\overline{X}$ 为平均值，*SD*为标准差。Z积分可为正值，也可为负值。

（4）中位数法：当样本变量为正态分布时中位数等于均数与第50百分位数。当样本变量分布不是完全正态时，选用中位数而不是算术平均数作为中间值。因此时样本中少数变量分布

在一端，用算术平均数表示则对个别变量值影响大。故用中位数表示变量的平均水平较妥。

2．界值点的选择 通常以均值离差法$\overline{X}\pm2SD$（包括总体的95％）为正常范围；百分位数法以P_3～P_{97}，（包括总体的94％）为正常范围；标准差的离差值以±2以内为正常范围。

3．测量值的表示

（1）表格：将测量数值以表格形式列出，便于查询，但不够直观。

（2）生长曲线：按各等级的数值绘制成曲线图。优点是较等级数值直观，不仅能较准确了解儿童的发育水平，还能对儿童某项指标进行定期纵向观察，易看出该小儿生长的趋势有无偏离现象，以便及早发现原因及采取干预措施 （图2-3）。

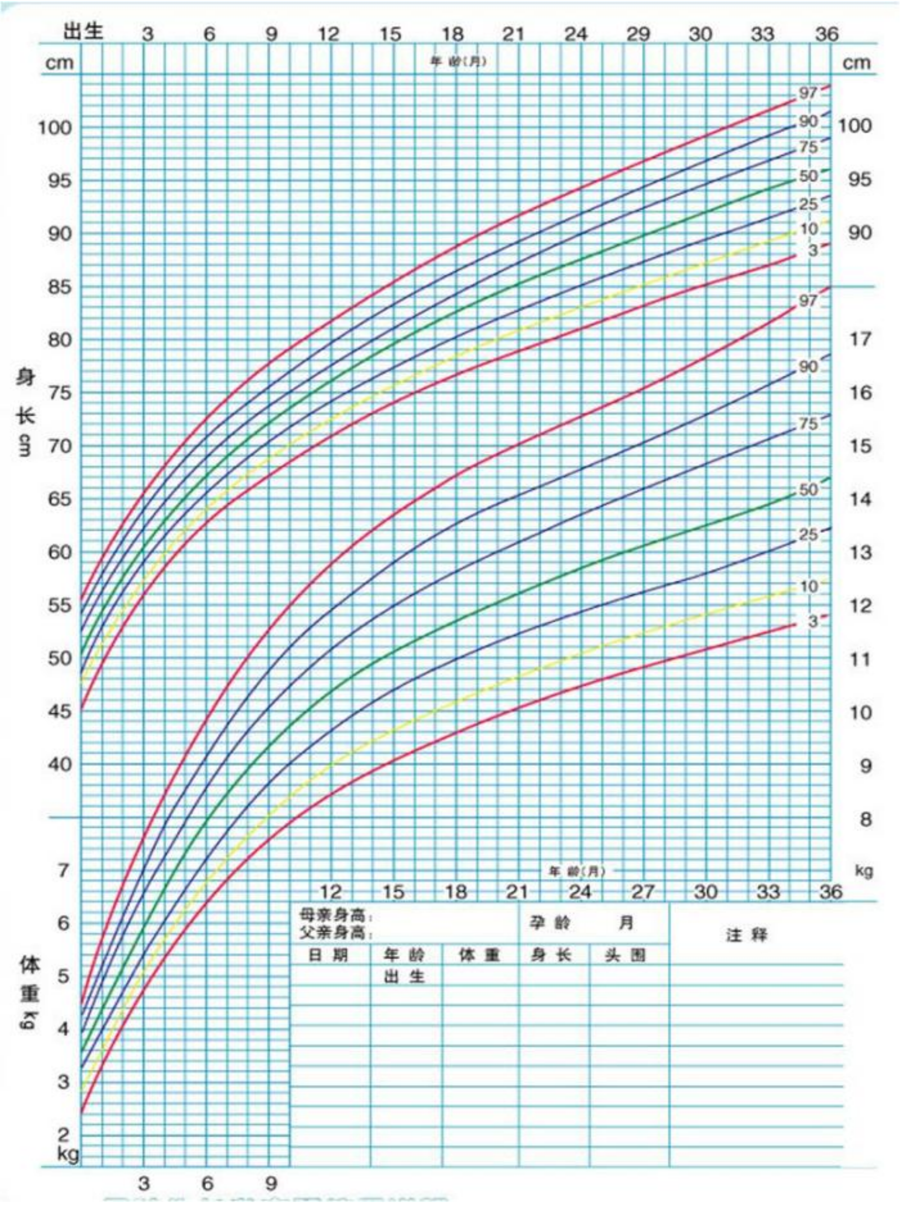

图2-3 生长曲线

4．评价结果表示

（1）等级划分：方法简单，利用均值加减标准差或直接用百分位数进行分级，据细分要求的不同可分为三等、五等、六等级等。五等级划分方法见表2-2。三等级划分法以＞$\overline{X}$＞$+2SD$为上、$\overline{X}\pm2SD$ 为中、＜$\overline{X}-2SD$ 为下。而六等级划分法把五等级划分法的“中”（$\overline{X}\pm1SD$）再分为$\overline{X}-1SD$的“中⁻”和$\overline{X}+1SD$的“中⁺”。等级划分法用于横断面的测量值分析，如发育水平、体型匀称的评价。

表2-2　五等级划分方法

等级	离差法	百分位数法
上	＞$\overline{X}$＋SD	＞P_{97}
中上	$\overline{X}$＋（1SD～2SD）	$P_{75\sim97}$
中	$\overline{X}$±1SD	$P_{25\sim75}$
中下	$\overline{X}$－（1SD～2SD）	$P_{3\sim25}$
下	＜$\overline{X}$-2SD	＜P_3

（2）测量值的计算：如用于定期纵向的测量值分析（生长速度的评价），即将两次连续测量值的差与参数中相同年龄的数值差比较；或身材匀称度的计算等。

（二）体格生长评价

正确评价儿童体格生长状况，必须注意采用准确的测量用具及统一的测量方法，定期纵向观察。同时有可用的参考人群值，参照人群值的选择决定评价的结果。WHO推荐美国国家卫生统计中心（NCHS）汇集的测量资料作为国际参照人群值。我国采用2005年中国九大城市儿童的体格生长数据为中国儿童参照人群值（附录一）。

儿童体格生长评价包括发育水平、生长速度以及匀称程度三个方面。

1．发育水平 将某一年龄时点所获得的某一项体格生长指标测量值（横断面测量）与参考人群值比较，得到该儿童在同质人群中所处的位置，即为此儿童该项体格生长指标在此年龄的生长水平，通常以等级表示其结果。生长水平包括所有单项体格生长指标，如体重、身高（长）、头围、胸围、上臂围等，可用于个体或群体儿童的评价。

早产儿体格生长有一允许的“落后”年龄范围，即此年龄后应“追上”正常足月儿的生长。进行生长水平评价时应矫正胎龄至40周胎龄（足月）后再评价，身长至40月龄、头围至18月龄、体重至24月龄后不再矫正。

有些单项测量，如骨龄代表发育成熟度，也反映发育水平。同样，体格测量值也可以生长的年龄来代表发育水平或成熟度。如一个2岁男孩身高76cm，身高生长水平为下等，其身高的生长年龄相当1岁。

发育水平评价的优点是简单、易于掌握与应用。对群体儿童体格发育水平评价可了解该群体儿童的体格状况；对个体儿童评价仅表示该儿童已达到的水平，不能说明过去存在的问题，也不能预示该儿童的生长趋势。

2．生长速度 是对某一单项体格生长指标定期连续测量（纵向观察），将获得的该项指标在某一年龄阶段的增长值与参照人群值比较，得到该儿童该项体格生长指标的生长速度。

以生长曲线表示生长速度最简单、直观，定期体检是生长速度评价的关键。儿童年龄小，生长较快，定期检查间隔时间不宜太长。这种动态纵向观察个体儿童的生长规律方法，可发现每个儿童有自己稳定的生长轨道，体现个体差异。因此，生长速度的评价较发育水平更能真实了解儿童生长状况。生长速度正常的儿童生长基本正常。

3．匀称程度 是对体格生长指标之间关系的评价。

（1）体型匀称度：表示体型（形态）生长的比例关系。实际工作中常选用身高的体重表示一定身高的相应体重增长范围，间接反映身体的密度与充实度。将实际测量与参照人群值比较，结果常以等级表示。

（2）身材匀称：以坐高（顶臀高）/身高（长）的比值反映下肢生长状况。按实际测量计算结果与参照人群值计算结果比较。结果以匀称、不匀称表示。

第四节 与体格生长有关的其他系统的发育

一、骨 骼

1．头颅骨 除头围外，还可据骨缝闭合、前囟大小及前后囟闭合时间来评价颅骨的生长及发育情况。婴儿娩出生时经过产道，故出生时颅骨缝稍有重叠，不久重叠现象消失。出生时后囟很小或已闭合，至迟约6～8周龄闭合。前囟出生时约1～2cm，以后随颅骨生长而增大，6月龄左右逐渐骨化而变小，最迟于1.5岁闭合。前囟大小以两个对边中点连线的长短表示。前囟检查在儿科临床很重要，如脑发育不良时头围小、前囟小或关闭早；甲状腺功能低下时前囟闭合延迟；颅内压增高时前囟饱满；脱水时前囟凹陷。（图2-4）

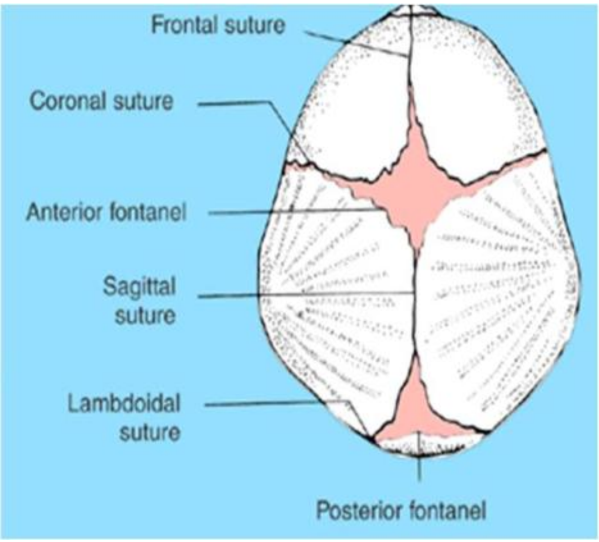

图2-4 前囟大小

2．脊柱 脊柱的增长反映脊椎骨的生长。生后第一年脊柱生长快于四肢，以后四肢生长快于脊柱。出生时脊柱无弯曲，仅呈轻微后凸。3个月左右抬头动作的出现使颈椎前凸；6个月后能坐，出现胸椎后凸；1岁左右开始行走，出现腰椎前凸。这样的脊椎自然弯曲至6～7岁才为韧带所固定。注意小儿坐、立、走姿势，选择适宜的桌椅，对保证儿童脊柱正常形态很重要。

3．长骨 是从胎儿到成人期逐渐完成的。长骨的生长主要由长骨干骺端的软骨骨化，骨膜下成骨，使长骨增长、增粗，当骨骺与骨干融合时，标志长骨停止生长。

随年龄的增加，长骨干骺端的软骨次级骨化中心按一定顺序及骨解剖部位有规律的出现。骨化中心出现可反映长骨的生长成熟程度。用X线检查测定不同年龄儿童长骨干骺端骨化中心的出现的时间、数目、形态的变化，并将其标准化，即为骨龄（bone age）。出生时腕部尚无骨化中心，股骨远端及胫骨近端已出现骨化中心。因此判断长骨的生长，婴儿早期应摄膝部X线骨片，年长儿摄左手及腕部X线骨片，以了解其腕骨、掌骨、指骨的发育。腕部于出生时无骨化中心，其出生后的出现次序为：头状骨、钩骨（3个月左右）、下桡骨骺（约1岁）、三角骨（2～2.5岁）、月骨（3岁左右）、大、小多角骨（3.5～5岁）、舟骨（5～6岁）、下尺骨骺（6～7岁）、豆状骨（9～10岁）。10岁时出全，共10个，故1～9岁腕部骨化中心的数目大约为其岁数加1。具体评价骨龄时应对照图谱。骨生长与生长激素、甲状腺素、性激素有关。骨龄在临床上有重要诊断价值，如甲状腺功能低下症、生长激素缺乏症骨龄明显延后；真性性早熟、先天性肾上腺皮质增生症骨龄超前。但正常骨化中心出现的年龄差异较大，诊断骨龄延迟时一定要慎重。

二、牙 齿

牙齿生长与骨骼有一定关系，但因胚胎来源不完全相同，牙齿与骨骼的生长不完全平行。出生时乳牙已骨化，乳牙牙孢隐藏在颌骨中，被牙龈覆盖；恒牙的骨化从新生儿期开始，18～24个月时第三恒臼齿已骨化。人一生有乳牙（共20个）和恒牙（28～32个）两副牙齿。生后4～10个月乳牙开始萌出，12个月后未萌出者为乳牙萌出延迟。乳牙萌出顺序一般为下颌先于上颌、自前向后（图2-8），约于2.5岁时乳牙出齐。乳牙萌出时间个体差异较大，与遗传、内分泌、食物性状有关。

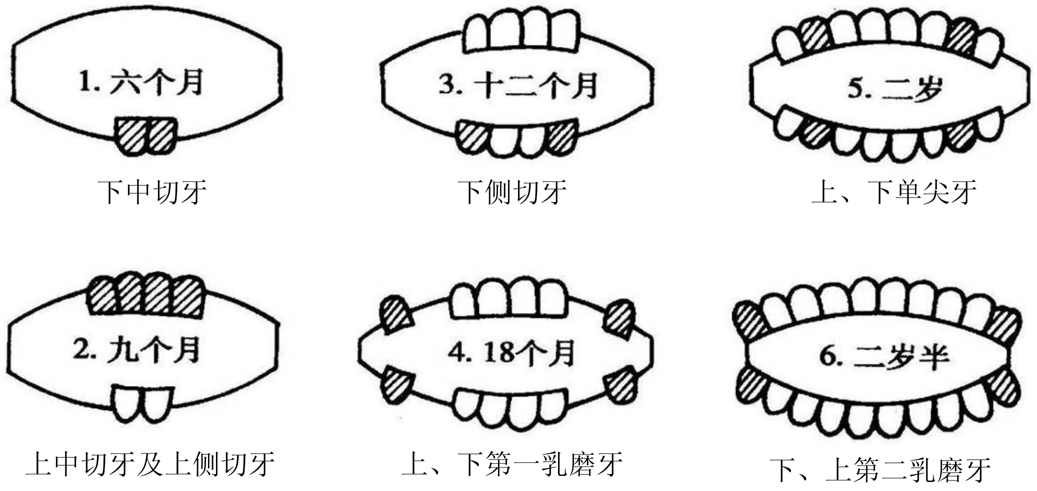

图2-5 乳牙萌出顺序

6岁左右萌出第一颗恒牙（第一恒磨牙，在第二乳磨牙之后，又称6龄齿）；6～12岁阶段乳牙逐个被同位恒牙替换，其中第1、2前磨牙代替第1、2乳磨牙，此期为混合牙列期；12岁萌出第二恒磨牙；约在18岁以后萌出第三恒磨牙（智齿），也有终生第三恒磨牙不萌出者。

出牙为生理现象，出牙时个别婴儿可有低热，唾液增多、发生流涎及睡眠不安、烦躁等症状。牙齿的健康生长与蛋白质、钙、磷、氟、维生素A、C、D等营养素和甲状腺激素有关。食物的咀嚼有利于牙齿生长。牙齿生长异常时可见外胚层生长不良、钙或氟缺乏、甲状腺功能低下等疾病。

三、生殖系统发育

参见第十七章内分泌疾病。

第五节 神经心理发育

在儿童成长过程中，神经心理的正常发育与体格生长具有同等重要的意义。神经心理发育包括感知、运动、语言、情感、思维、判断和意志性格等方面，以神经系统的发育和成熟为物质基础。和体格生长一样，神经心理发育的异常可能是某些系统疾病的早期表现，因此，了解儿童心理发育规律对疾病的早期诊断很有帮助。

（一）神经系统的发育

在胎儿期，神经系统的发育领先于其他各系统，新生儿脑重已达成人脑重25%左右，此时神经细胞数目已与成人相同，但其树突与轴突少而短。出生后脑重的增加主要由于神经细胞体积增大和树突的增多、加长，以及神经髓鞘的形成和发育。神经髓鞘的形成和发育约在4岁左右完成，在此之前，尤其在婴儿期，各种刺激引起的神经冲动传导缓慢，且易于泛化；不易形成兴奋灶，易疲劳而进入睡眠状态。

脊髓随年龄而增长。在胎儿期，脊髓下端在第2腰椎下缘，4岁时上移至第1腰椎，在进行腰椎穿刺时应注意。婴儿肌腱反射较弱，腹壁反射和提睾反射也不易引出，到1岁时才稳定。3～4个月前的婴儿肌张力较高，凯尔尼格征可为阳性，2岁以下儿童巴宾斯基征阳性亦可为生理现象。

（二）感知的发育

1．视感知发育 新生儿已有视觉感应功能，瞳孔有对光反应，在安静清醒状态下可短暂注视物体，但只能看清15～20cm内的事物。第2个月起可协调地注视物体，开始有头眼协调；3～

4个月时喜看自己的手，头眼协调较好；6～7个月时目光可随上下移动的物体垂直方向转动；8～9个月时开始出现视深度感觉，能看到小物体；18个月时已能区别各种形状；2岁时可区别垂直线与横线；5岁时已可区别各种颜色；6岁时视深度已充分发育。

2．听感知发育 出生时鼓室无空气，听力差；生后3～7日听觉已相当良好；3～4个月时头可转向声源，听到悦耳声时会微笑；7～9个月时能确定声源，区别语言的意义；13～16个月时可寻找不同响度的声源，听懂自己的名字；4岁时听觉发育已经完善。听感知发育和儿童的语言发育直接相关，听力障碍如果不能在语言发育的关键期内（6个月内）或之前得到确诊和干预，则可因聋致哑。

3．味觉和嗅觉发育

（1）味觉：出生时味觉发育已很完善；4～5月甚至对食物轻微的味道改变已很敏感，为味觉发育关键期，此期应适时添加各类转乳期食物。

（2）嗅觉：出生时嗅觉中枢与神经末梢已发育成熟；3～4月时能区别愉快与不愉快的气味；7～8月开始对芳香气味有反应。

4．皮肤感觉的发育 皮肤感觉包括触觉、痛觉、温度觉及深感觉等。触觉是引起某些反射的基础。新生儿眼、口周、手掌、足底等部位的触觉已很灵敏，而前臂、大腿、躯干的触觉则较迟钝。新生儿已有痛觉，但较迟钝；第2个月起才逐渐改善。出生时温度觉就很灵敏。

（三）运动的发育

运动发育可分为大运动（包括平衡）和细运动两大类。

1．平衡与大运动

（1）抬头：新生儿俯卧时能抬头1～2秒；3个月时抬头较稳；4个月时抬头很稳。

（2）坐：6个月时能双手向前撑住独坐；8个月时能坐稳。

（3）翻身：7个月是能有意识地从仰卧位翻身至俯卧位、然后从俯卧位翻至仰卧位。

（4）爬：应从3～4个月时开始训练，8～9月可用双上肢向前爬。

（5）站、走、跳：11个月时可独自站立片刻；15个月可独自走稳；24个月时可双足并跳；30个月时会独足跳。

2．细动作 3～4个月握持反射消失之后手指可以活动；6～7个月时出现换手与捏、敲等探索性动作；9～10个月时可用拇、食指拾物，喜撕纸；12～15个月时学会用匙，乱涂画；18个月时能叠2～3块方积木；2岁时可叠6～7块方积木，会翻书。

（四）语言的发育

语言的发育与大脑、咽喉部肌肉的正常发育及听觉的完善有关。要经过发音、理解和表达3 个阶段。新生儿已会哭叫，3～4个月咿呀发音；6月龄时能听懂自己的名字；12月龄时能说简单的单词，如“再见”、“没了”。18月龄时能用15～20个字，指认并说出家庭主要成员的称谓；24月龄时能指出简单的人、物名和图片，而到3岁时几乎能指认许多物品名，并说有2～3个字组成的短句；4岁时能讲述简单的故事情节。

（五）心理活动的发展

1．早期的社会行为 2～3个月时小儿以笑、停止啼哭等行为，以眼神和发音表示认识父母；3～4个月的婴儿开始出现社会反应性的大笑；7～8月的小儿可表现出认生、对发声玩具感兴趣等；9～12月时是认生的高峰；12～13个月小儿喜欢玩变戏法和躲猫猫游戏；18个月时逐渐有自我控制能力，成人在附近时可独自玩很久；2岁时不再认生，易与父母分开；3岁后可与小朋友做游戏。

2．注意的发展 婴儿期以无意注意为主，随着年龄的增长逐渐出现有意注意。5～6岁后儿

童能较好控制自己的注意力。

3．记忆的发展 记忆是将所学得的信息贮存和“读出”的神经活动过程，可分为感觉、短暂记忆和长久记忆3个不同的系统。长久记忆又分为再认和重现两种，再认是以前感知的事物在眼前重现时能被认识，重现是以前感知的事物虽不在眼前重现，但可在脑中重现。1岁内婴儿只有再认而无重现，随年龄的增长，重现能力亦增强。幼年儿童只按事物的表面特性记忆信息，以机械记忆为主。随着年龄的增加和理解、语言思维能力的加强，逻辑记忆逐渐发展。

4．思维的发展 1岁以后的儿童开始产生思维，在3岁以前只有最初级的形象思维；3岁以后开始有初步抽象思维；6～11岁以后儿童逐渐学会综合分析、分类比较等抽象思维方法，具有进一步独立思考的能力。

5．想象的发展 新生儿无想象能力；1～2岁儿童仅有想象的萌芽。学龄前期儿童仍以无意想象为主，有意想象和创造性想象到学龄期才迅速发展。

6．情绪、情感的发展 新生儿因生后不易适应宫外环境，较多处于消极情绪中，表现不安、啼哭，而哺乳、抱、摇、抚摸等则可使其情绪愉快。婴幼儿情绪表现特点是时间短暂、反应强烈、容易变化、外显而真实。随着年龄的增长，儿童对不愉快因素的耐受性逐渐增加，能够有意识地控制自己，使情绪逐趋向稳定。

7．个性和性格的发展 婴儿期由于一切生理需要均依赖成人，逐渐建立对亲人的依赖性和信任感。幼儿时期已能独立行走，说出自己的需要，故有一定自主感，但又未脱离对亲人的依赖，常出现违拗言行与依赖行为相交替现象。学龄前期小儿生活基本能自理，主动性增强，但主动行为失败时易出现失望和内疚。学龄期开始正规学习生活，重视自己勤奋学习的成就，如不能发现自己学习潜力将产生自卑。青春期体格生长和性发育开始成熟，社交增多，心理适应能力增强但容易波动，在感情问题、伙伴问题、职业选择、道德评价和人生观等问题上处理不当时易发生性格变化。性格一旦形成即相对稳定。

小儿神经精神发育进程见表2-3。

表 2-3 小儿神经精神发育进程

年龄	粗、细动作	语言	适应周围人物的能力与行为
新生儿	无规律、不协调动作；紧握拳	能哭叫	铃声使全身活动减少
2 月	直立及俯卧位时能抬头	发出和谐的喉音	能微笑，有面部表情；眼随物转动
3 月	仰卧位变为侧卧位；用手摸东西	咿呀发音	头可随看到的物品或听到的声音转动180°；注意自己的手
4 月	扶着髋部时能坐；可在俯卧位时用两手支持抬起胸部；手能握持玩具	笑出声	抓面前物体；自己玩弄手，见食物表示喜悦；较有意识的哭和笑
5 月	扶腋下能站得直；两手各握一玩具	能喃喃地发出单词音节	伸手取物；能辨别人声；望镜中人笑
6 月	能独坐一会；用手摇玩具		能认识熟人和陌生人；自拉衣服；自握足玩
7 月	会翻身；自己独坐很久；将玩具从一手换入另一手	能发“爸爸”、“妈妈”等复音，但无意识	能听懂自己的名字；自握饼干吃
8 月	会爬；会自己坐起来、躺下去；会扶着栏杆站起来；会拍手	重复大人所发简单音节	注意观察大人的行动；开始认识物体；两手会传递玩具
9 月	试独站；会从抽屉中取出玩具	能懂几个较复杂的词句，如“再见”等	看见熟人会手伸出来要人抱；或与人合作游戏
10～11 月	能独站片刻；扶椅或推车能走几步；拇、	开始用单词，一个单词表	能模仿成人的动作；招手、“再见”；抱

年龄	粗、细动作	语言	适应周围人物的能力与行为
	食指对指拿东西	示很多意义	奶瓶自食
12 月	独走；弯腰拾东西；会将圆套在木棍上	能叫出物品的名字，如灯、碗；指出自己的手、眼	对人和事物有喜憎之分；穿衣能合作，用杯喝水
15 月	走得好；能蹲着玩；能叠一块方木	能说出几个词和自己的名字	能表示同意、不同意
18 月	能爬台阶；有目标地扔皮球	能认识和指出身体名部分	会表示大小便；懂命令；会自己进食
2 岁	能双脚跳；手的动作更准确；会用勺子吃饭	会说 2～3 个字构成的句子	能完成简单的动作，如拾起地上的物品；能表达喜、怒、怕、懂
3 岁	能跑；会骑三轮车；会洗手、洗脸；脱、穿简单衣服	能说短歌谣，数几个数	能认识画上的东西；认识男、女；自称“我”；表现自尊心、同情心、害羞
4 岁	能爬梯子；会穿鞋	能唱歌	能画人像；初步思考问题；记忆力强、好发问
5 岁	能单足跳；会系鞋带	开始识字	能分辨颜色；数 10 个数；知物品用途及性能
6～7 岁	参加简单劳动，如扫地、擦桌子、剪纸、泥塑、结绳等	能讲故事；开始写字	能数几十个数；可简单加减；喜独立自主

第六节　儿童神经心理发育的评价

儿童神经心理发育的水平表现在儿童在感知、运动、语言和心理等过程中的各种能力，对这些能力的评价称为心理测试。心理测试仅能判断儿童神经心理发育的水平，没有诊断疾病的意义。心理测试需由经专门训练的专业人员根据实际需要选用，不可滥用。

（一）能力测验

1．筛查性测验

（1）丹佛发育筛查法（DDST）：DDST主要用于6岁以下儿童的发育筛查，实际应用时对4.5岁以下的儿童较为适用。测试内容分为大运动、细运动、语言、个人适应性行为四个能区。1990年Denover Ⅱ出版，在1966年DDST的基础上修订。国内有地区性的修订常模。

（2）绘人测试：适用于5～9.5岁儿童。要求被测儿童依据自己的想象绘一全身正面人像，以身体部位、各部比例和表达方式的合理性计分。绘人法测试结果与其他智能测试的相关系数在0.5以上，与推理、空间概念、感知能力的相关性更显著。该法可个别测试，也可进行集体测试。

（3）图片词汇测试（PPVT）：适用于4～9岁儿童的一般智能筛查。PPVT的工具是120张图片，每张有黑白线条画四幅，测试者说一个词汇，要求儿童指出其中相应的一幅画。测试方法简单，尤适用于语言或运动障碍者。1981年PPVT-R出版，有L及M版本，测试年龄为2.5～16岁，测试图片增至175张。

2．诊断测验

（1）Gesell发育量表：适用于4周至3岁的婴幼儿，从大运动、细动作、个人-社会、语言和适应性行为五个方面测试，结果以发育商（DQ）表示。

（2）Bayley婴儿发育量表：适用于2～30个月婴幼儿，包括精神发育量表、运动量表和婴儿行为记录。

（3）Standford-Binet智能量表：适用2～18岁儿童。测试内容包括幼儿的具体智能（感知、

认知、记忆）和年长儿的抽象智能（思维、逻辑、数量、词汇），用以评价儿童学习能力以及对智能发育迟缓者进行诊断及程度分类，结果以智商（IQ）表示。

（4）Wechsler学前及初小儿童智能量表（WPPSI）：适用于4～6.5岁儿童。通过编制一整套不同测试题，分别衡量不同性质的能力，将得分综合后可获得儿童多方面能力的信息，较客观地反映学前儿童的智能水平。

（5）Wechsler儿童智能量表修订版（WISC-R）：适用于6～16岁儿童，内容与评分方法同WPPSI。国外已有新版。

（二）适应性行为测试

智力低下的诊断与分级必须结合适应性行为的评定结果。国内现多采用日本S-M社会生活能力检查，即婴儿-初中学生社会生活能力量表。此量表适用于6个月～15岁儿童社会生活能力的评定。

第七节 心理行为异常

一、儿童行为问题

儿童在发育过程中出现的行为问题较为常见，对儿童身心健康的影响很大。近年调查资料表明我国少年儿童的行为问题检出率为8.3～12.9%。儿童行为问题表现在儿童日常生活中，容易被家长忽略，或被过分严重估计。因此，区别正常的和异常的儿童行为非常必要，目前有多种衡量儿童行为的量表可用于帮助区分儿童异常的行为问题。

儿童的行为问题一般可分为：①生物功能行为问题，如遗尿、遗便、多梦、睡眠不安、夜惊、食欲不佳、过分挑剔饮食等；②运动行为问题，如儿童擦腿综合征、咬指甲、磨牙、吸吮手指、咬或吸衣物、挖鼻孔、咬或吸唇、活动过多等；③社会行为问题，如破坏、偷窃、说谎、攻击等；④性格行为问题，如惊恐、害羞、忧郁、社交退缩、交往不良、违拗、易激动、烦闹、胆怯、过分依赖、要求注意、过分敏感、嫉妒、发脾气等；⑤语言问题，如口吃等。男孩的行为问题常多于女孩，男孩多表现运动与社会行为问题；女孩多性格行为问题。儿童行为问题的发生与父母对子女的期望、教养方式、父母的文化、学习环境等显著相关。多数儿童的行为问题可在发育过程中自行消失。

1．屏气发作 为表现为呼吸运动暂停的一种异常性格行为问题，多发于6～18月婴幼儿，5岁前会逐渐自然消失。呼吸暂停发作常在情绪急剧变化时，如发怒、恐惧、剧痛、剧烈叫喊时出现，常有换气过度，使呼吸中枢受抑制，哭喊时屏气，脑血管扩张，脑缺氧时可有昏厥、丧失意志、口唇发绀，躯干、四肢挺直，甚至四肢抽动，持续0.5～1分钟后呼吸恢复，症状缓解，口唇返红，全身肌肉松弛而清醒，一日可发作数次。这种婴儿性格多暴躁、任性、好发脾气。对此类儿童应加强家庭教养，遇矛盾冲突时应耐心说理解释，避免粗暴打骂，尽量不让孩子有发脾气、哭闹的机会。有时需与癫痫鉴别。

2．吮拇指癖、咬指甲癖 3～4个月后的婴儿生理上有吮吸要求，常自吮手指尤其是拇指以安定自己。这种行为常发生在饥饿时和睡前，多随年龄增长而消失。但有时婴儿因心理上得不到满足而精神紧张、恐惧焦急，未获父母充分的爱，又缺少玩具、音乐、图片等视听觉刺激，孤独时便吮拇指自娱，渐成习惯，直至年长时尚不能戒除。长期吮手指可影响牙齿、牙龈及下颌发育，致下颌前突、齿列不齐，妨碍咀嚼。咬指甲癖的形成过程与吮拇指癖相似，也系情绪紧张、感情需求得不到满足而产生的不良行为，多见于学龄前期和学龄期儿童。对这类孩子要多加爱护和关心，消除其抑郁孤独心理；当其吮拇指或咬指甲时应将其注意力分散到其他事物

上，鼓励儿童建立改正坏习惯的信心，切勿打骂讽刺，使之产生自卑心理。在手指上涂抹苦药等方法也往往起不到好的效果。

3．遗尿症 正常幼儿在2～3岁时已能控制排尿，如在5岁后仍发生不随意排尿即为遗尿症，大多数发生在夜间熟睡时，称夜间遗尿症。遗尿症可分为原发性和继发性两类：原发性遗尿症较多见，多半有家族史，男多于女（2：1～3：1），无器质性病变，多因控制排尿的能力迟滞所致；继发性遗尿症大多由于全身性或泌尿系疾病如糖尿病、尿崩症等引起，其他如智力低下、神经精神创伤、泌尿道畸形、感染，尤其是膀胱炎、尿道炎、会阴部炎症等也可引起继发性遗尿现象。继发性遗尿症在处理原发疾病后症状即可消失。

原发性遗尿发生在夜间为多，偶见白天午睡时。自每周1～2次至每夜1次、甚至一夜数次不等。健康状况欠佳、疲倦、过度兴奋紧张、情绪波动等都可使症状加重，有时会自动减轻或消失，亦可复发。约50％患儿可于3～4年内发作次数逐渐减少而自愈，也有一部分患儿持续遗尿直至青春期，往往造成严重的心理负担，影响正常生活与学习。对遗尿症患儿必须首先除外能引起继发性遗尿的全身或局部疾病

原发性遗尿症的治疗首先要取得家长和患儿的合作。医生应指导家长安排适宜的生活制度和坚持排尿训练，绝对不能在小儿发生遗尿时加以责骂、讽刺、处罚等，否则会加重患儿心理负担。应训练患儿将排尿间隔逐渐延长，每次排尿务必排尽；晚餐后应控制入水量，睡前排尿，不宜过度兴奋；睡熟后父母可在其经常遗尿时间之前唤醒，使其习惯于觉醒时主动排尿，必要时亦可采用警报器协助训练。药物治疗效果约80％左右，常用者为去氨加压素（desmopessin），为抗利尿药，以减少泌尿量，每次0.1～0.2/μg，睡前口服，疗程3～6个月。亦可应用盐酸丙咪嗪类药物治疗。

4．儿童擦腿综合征 是儿童通过擦腿引起兴奋的一种运动行为障碍。在儿童中并不少见，女孩与幼儿更多见。发生擦腿综合征的儿童智力正常，发作时神志清醒，多在入睡前、醒后或玩耍时发作，可被分散注意力而终止。发作时，女孩喜坐硬物，手按腿或下腹部，双下肢伸直交叉夹紧，手握拳或抓住东西使劲；男孩多表现伏卧在床上、来回蹭，或与女孩类似表现。女孩发作后外阴充血，分泌物增多或阴唇色素加深；男孩阴茎勃起，尿道口稍充血，有轻度水肿。使小儿平时生活轻松愉快，解除心理压力，鼓励其参与各种游戏活动等心理行为治疗是公认的必要措施。发作时以有趣事物分散儿童的注意力、睡前让儿童疲倦后很快入睡、醒后立即起床等均可减少发作机会。从小应注意儿童的会阴清洁。儿童擦腿综合征多随年龄增长而逐渐自行缓解。

5．注意力缺乏多动症 为学龄儿童中常见的行为问题，主要表现为注意力不集中、多动、冲动行为，常伴有学习困难，但智能正常或接近正常。男孩发生率明显高于女孩。病因尚不肯定。

二、学习障碍

学习不仅是阅读、书写、计算等能力，还包括获得这些技能的整个学习过程。学习的必要条件是要有正常发展的认知能力、正常的感觉（尤其是听、视觉）器官功能、正常的运动发育、正常情绪和良好的环境。学习障碍属特殊发育障碍，是指在获得和运用听、说、读、写、计算、推理等特殊技能上有明显困难，并表现出相应的多种障碍综合征。临床上常把由于各种原因如智力低下、多动、情绪和行为问题、特殊发育障碍所引起的学业失败统称学习困难。中枢神经系统的某些功能障碍也会导致学习技能上的困难。学龄期儿童发生学习障碍者较多，小学2～3年级为发病的高峰；男孩多于女孩。学习障碍可有学习能力的偏异（如操作或语言能力）；协

调运动障碍，如眼手协调差、影响绘图等精细运动技能的获得；分不清近似音，影响听、说与理解；理解与语言表达缺乏平衡，听与阅读时易遗漏或替换，不能正确诵读，构音障碍，交流困难；知觉转换障碍，如听到“狗”时不能就想到“狗”，立即写出“狗”字；视觉-空间知觉障碍，辨别能力差，常分不清6与9，b与d等，影响阅读能力等。学习障碍的儿童不一定智力低下，但由于其认知特性导致患儿不能适应学校学习和日常生活。在拒绝上学的儿童中有相当部分是学习障碍儿童，对他们应仔细了解、分析原因，采取特殊教育对策。

（沈晓明）

第三章　儿童保健原则

儿童保健同属儿科学与预防医学的分支，为两者的交叉学科，其主要任务是研究儿童各年龄期生长发育的规律及其影响因素，以通过有效措施，促进有利因素，防止不利因素，保障儿童健康成长。儿童保健研究涉及的内容包括：儿童的体格生长和社会心理发育、儿童营养、儿童健康促进和儿科疾病的管理等。

自19世纪80年代初，儿童保健问题、特别是儿童的生存问题显得更为迫切。根据当时全球形势及发展中国家的经济，由联合国儿童基金会发起的一揽子的组合干预措施，简称为GOBI（即生长监测、口服补液治疗腹泻病、母乳喂养及免疫接种）；以后又推出造成婴幼儿死亡的主要疾病，即肺炎及腹泻的简化治疗技术，使儿童的死亡率明显下降。

随着时代的进展，造成儿童死亡的原因也在改变，显出以前专门“单一问题”工作的局限性。据最近全球的统计，5岁以下小儿有6种致使性疾病，占死亡率的70%～90%以上，这6种疾病为急性呼吸道感染，绝大部分为肺炎（19%），腹泻病（18%），疟疾（8%），麻疹（4%），HIV/AIDS（3%）及与新生儿有关的疾病，主要为早产、产中窒息及感染（37%）；而这些疾病通过现行的卫生保健措施是可以预防的。

应对这种新情况需要一揽子的简单易行、效果显著的方法，并利用这些方法在儿童疾病综合管理（integrated management of childhood illness, IMCI）的指导下综合管理严重的儿童疾病及营养不良。IMCI综合了一些有效措施来防止患儿死亡、促进儿童健康成长及发育。

IMCI 在不同层次上有着不同的含义。从患者的角度来看，综合就是病案的管理；从保健的角度来看，综合意味着通过一种服务渠道进行多种形式的服务，例如定期体格检查的同时进行免疫接种，可以为家长提供咨询的机会，密切了医务保健人员与家长之间的关系，使医务保健人员更加关心儿童的营养、体格及社会心理的发育；在机制层次上，综合便是把管理结合起来，支持不同的辅助性保健工作，保障不同层次保健工作的综合性。IMCI 就是成功地将初级保健设施的病案管理和工作任务结合起来，医务保健人员要为它的服务对象提供一整套的技术服务。所以 IMCI 是当今儿童保健的唯一策略，在以上三个层次上同时加强保健的综合，将保健从家庭和社区延伸到初级卫生单位以及转诊机构，并且强调提供咨询和解决问题。IMCI 已被 100 多个国家所，我国正在开始相关前期工作。

第一节 各年龄期儿童的保健重点

（一）胎儿期及围生期

胎儿的发育与孕母的躯体健康、心理卫生、营养状况和生活环境等密切相关，胎儿期保健主要通过对孕母的保健来实现。

1．预防遗传性疾病与先天畸形　应大力提倡和普及婚前遗传咨询，禁止近亲结婚；应避免接触放射线和铅、苯、汞、有机磷农药等化学毒物；应避免吸烟、酗酒；患有心肾疾病、糖尿病、甲状腺功能亢进、结核病等慢性疾病的育龄妇女应在医生指导下确定怀孕与否及孕期用药；对高危产妇除定期产前检查外，应加强观察，一旦出现异常情况，应及时就诊，必要时可终止妊娠。

2．保证充足营养　妊娠后期应加强铁、锌、钙、维生素D 等重要营养素的补充。但也应防止营养摄入过多而导致胎儿体重过重，影响分娩和成年期的健康。

3．预防感染　包括孕期及分娩时。孕妇早期应预防弓形虫、风疹病毒、巨细胞病毒及单

纯疱疹病毒的感染，以免造成胎儿畸形及宫内发育不良。分娩时应预防来自产道的感染而影响即将出生的新生儿。

4．给予良好的生活环境，注意劳逸结合，减少精神负担和心理压力。

5．尽可能避免妊娠期合并症，预防流产、早产、异常分娩的发生。对高危孕妇应加强随访。

6．加强对高危新生儿的监护　对高危妊娠孕妇所分娩的新生儿及早产儿、低体重儿、新生儿窒息、低体温、低血糖、低血钙和颅内出血等疾病的高危新生儿应予以特殊监护和积极处理。

（二）新生儿期

新生儿期，特别是生后1周内的新生儿发病率和死亡率极高，婴儿死亡中约2/3 是新生儿，<1周的新生儿占新生儿死亡数的70％左右。故新生儿保健是儿童保健的重点，而生后1周内新生儿的保健是重中之重。因此在2005年的世界卫生组织（WHO）年度报告中，把过去的儿童保健，建议改主新生儿及儿童保健，突出新生儿保健的重要性。

1．出生时的护理　新生儿娩出后应迅速清理口腔内粘液，保证呼吸道通畅；严格消毒、结扎脐带；记录出生时Apgar评分、体温、呼吸、心率、体重与身长；设立新生儿观察室，出生后观察6小时，正常者进入婴儿室，高危儿送入新生儿重症监护室；提倡母婴同室，尽早喂母奶。新生儿出院回家前应根据要求进行先天性遗传代谢病筛查（目前开展的有先天性甲状腺功能低下和苯丙酮尿症）和听力筛查。

2.新生儿居家保健　有条件的家庭在冬季应使室内温度保持在20～22℃左右，湿度以55％为宜；保持新生儿体温正常恒定。提倡母乳喂养，指导母亲正确的哺乳方法。新生儿皮肤娇嫩，应保持皮肤清洁，避免损伤。父母应多与婴儿交流，抚摸有利于早期的情感交流。应尽量避免过多的外来人员接触。注意脐部护理，预防感染。应接种卡介苗和乙型肝炎疫苗。

（三）婴儿期保健

婴儿期的体格生长十分迅速，需大量各种营养素满足其生长的需要，但婴儿的消化功能尚未成熟，故易发生消化紊乱和营养缺乏性疾病。部分母乳喂养或人工喂养婴儿则应选择配方奶粉。自4～6 个月开始应添加辅食，为断离母乳做准备。定期进行体格检查，便于早期发现缺铁性贫血、佝偻病、营养不良、发育异常等疾病并予以及时的干预和治疗。坚持户外活动，进行空气浴、日光浴和主、被动体操有利于体格生长。给予各种感知觉的刺激，促进大脑发育。该时期应按计划免疫程序完成基础免疫。预防异物吸入及窒息。

（四）幼儿期

由于感知能力和自我意识的发展，对周围环境产生好奇、乐于模仿，幼儿期是社会心理发育最为迅速的时期。该时期应重视与幼儿的语言交流，通过游戏、讲故事、唱歌等促进幼儿语言发育与大运动能力的发展。同时，应培养幼儿的独立生活能力，安排规律生活，养成良好的生活习惯，如睡眠、进食、排便、沐浴、游戏、户外活动等。定期进行体格检查，预防龋齿。由于该时期的儿童已经具备一定的活动能力，且凡事都喜欢探个究竟，故还应注意异物吸入、烫伤、跌伤等损伤的预防。

（五）学龄前期

学龄前期儿童智力发展快、独立活动范围大，是性格形成的关键时期。因此，加强学龄前期儿童的教育较重要，应注意培养其学习习惯、想象与思维能力，使之具有良好的心理素质。应通过游戏、体育活动增强体质，在游戏中学习遵守规则和与人交往。每年应进行1～2次体格检查，进行视力、龋齿、缺铁性贫血等常见病的筛查与矫治。保证充足营养，预防溺水、外伤、

误服药物以及食物中毒等损伤。

（六）学龄期与青春期

此期儿童求知欲强，是获取知识的最重要时期，也是体格发育的第二个高峰期。该时期应提供适宜的学习条件，培养良好的学习习惯，并加强素质教育；应引导积极的体育锻炼，不仅可增强体质同时也培养了儿童的毅力和意志力；合理安排生活，供给充足营养，预防屈光不正、龋齿、缺铁性贫血等常见病的发生；进行法制教育，学习交通规则和意外伤害的防范知识。在青春期应进行正确的性教育以使其在生理和心理上有正确的认识。

第二节 儿童保健的具体措施

（一）护理

对小儿的护理是儿童保健、医疗工作的基础内容，年龄愈小的儿童，愈需要合适的护理。①居室：应阳光充足、通气良好，冬季室内温度尽可能达到18～20℃，湿度为55%～60%。对哺乳期婴儿，主张母婴同室，便于母亲哺乳和料理婴儿。患病者不应进入婴儿居室，尤其是新生儿、早产儿的居室。②衣着（尿布）：应选择浅色、柔软的纯棉织物，宽松而少接缝，以避免摩擦皮肤和便于穿、脱。存放新生儿衣物的衣柜内不宜放置樟脑丸，以免发生新生儿溶血。新生儿应衣着宽松，保持双下肢屈曲姿势，有利于髋关节的发育。婴儿最好穿连衣裤或背带裤，不用松紧腰裤，以利胸廓发育。

（二）营养

营养是保证儿童生长发育及健康的先决条件，必须及时对家长和有关人员进行有关母乳喂养、断乳期婴儿的其它食物添加、幼儿期正确的进食行为培养、学前及学龄期儿童的膳食安排等内容的宣教和指导（见第四章）。

（三）计划免疫

计划免疫是根据儿童的免疫特点和传染病发生的情况制定的免疫程序，通过有计划地使用生物制品进行预防接种，以提高人群的免疫水平、达到控制和消灭传染病的目的。按照我国卫生部的规定，婴儿必须在1岁内完成卡介苗、脊髓灰质炎三价混合疫苗、百日咳、白喉、破伤风类毒素混合制剂、麻疹减毒疫苗及乙型肝炎病毒疫苗接种的基础免疫（表3-1）。根据流行地区和季节，或根据家长的自己的意愿，有时也进行乙型脑炎疫苗、流行性脑脊髓膜炎疫苗、风疹疫苗、流感疫苗、腮腺炎疫苗、甲型肝炎病毒疫苗、水痘疫苗、流感杆菌疫苗、肺炎疫苗、轮状病毒疫苗等的接种。

表3-1　我国卫生部规定的儿童计划免疫程序

年龄	接种疫苗		
出生	卡介苗		乙肝疫苗
1个月			乙肝疫苗
2个月	脊髓灰质炎三价混合疫苗		
3个月	脊髓灰质炎三价混合疫苗、	百白破混合制剂	
4个月	脊髓灰质炎三价混合疫苗、	百白破混合制剂	
5个月		百白破混合制剂	
6个月			乙肝疫苗
8个月	麻疹疫苗		
1.5～2岁		百白破混合制剂	
4岁	脊髓灰质炎三价混合疫苗复种		
6岁	麻疹疫苗复种	百白破混合制剂	

预防接种可能引起一些反应：①卡介苗接种后2周左右局部可出现红肿浸润，8～12周后结痂。若化脓形成小溃疡，腋下淋巴结肿大，可局部处理以防感染扩散，但不可切开引流。②脊髓灰质炎三型混合疫苗接种后有极少数婴儿发生腹泻，但往往能不治自愈。③百日咳、白喉、破伤风类毒素混合制剂接种后局部可出现红肿、疼痛或伴低热、疲倦等，偶见过敏性皮疹、血管性水肿。若全身反应严重，应及时到医院诊治。④麻疹疫苗接种后，局部一般无反应，少数人可在6～10日内产生轻微的麻疹，予对症治疗即可。⑤乙型肝炎病毒疫苗接种后很少有不良反应。个别人可有发热，或局部轻痛，不必处理。

（四）儿童心理卫生

世界卫生组织（WHO）给健康所下的定义是：不仅是没有疾病和病痛，而且是个体在身体上、精神上、社会上的完满状态。由此可知，心理健康和身体健康同等重要。

1．习惯的培养

（1）睡眠习惯：①应从小培养儿童有规律的睡眠习惯；②儿童居室应安静、光线应柔和，睡前避免过度兴奋；③儿童应该有相对固定的作息时间，包括睡眠；④婴儿可利用固定乐曲催眠入睡，不拍、不摇、不抱、不可用喂哺催眠。⑤保证充足睡眠时间；⑥培养用独自睡觉。

（2）进食习惯：①按时添加辅食；②进食量根据小儿的自愿，不要强行喂食；③培养定时、定位（位置）、自己用餐；④不偏食、不挑食、不吃零食；⑤饭前洗手；⑥培养用餐礼貌。

（3）排便习惯：东西方文化及传统的差异，对待大小便的训练意见绝对不同。我国多数的家长习惯于及早训练大小便；而西方的家长一切均顺其自然。但用尿布不会影响控制大小便能力的培养。

（4）卫生习惯：从婴儿期起就应培养良好的卫生习惯，定时洗澡、勤剪指甲、勤换衣裤，不随地大、小便。3岁以后培养小儿自己早晚刷牙、饭后漱口、食前便后洗手的习惯。儿童应养成不吃生水和未洗净的瓜果、不食掉在地上的食物、不随地吐痰、不乱扔瓜果纸屑的良好卫生习惯。

2．社会适应性的培养 从小培养儿童良好的适应社会的能力是促进儿童健康成长的重要内容之

一。儿童的社会适应性行为是各年龄阶段相应神经心理发展的综合表现，与家庭环境、育儿方式、儿童性别、年龄、性格密切相关。

（1）独立能力：应在日常生活中培养婴幼儿的独立能力，如自行进食、控制大小便、独自睡觉、自己穿衣鞋等。年长儿则应培养其独立分析、解决问题的能力。

（2）控制情绪：儿童控制情绪的能力与语言、思维的发展和父母的教育有关。婴幼儿的生活需要依靠成人的帮助，父母及时应答儿童的需要有助于儿童心理的正常发育。儿童常因要求不能满足而不能控制自己的情绪，或发脾气、或发生侵犯行为，故成人对儿童的要求与行为应按社会标准或予以满足、或加以约束、或预见性的处理问题，减少儿童产生消极行为的机会。用诱导方法而不用强制方法处理儿童的行为问题可以减少对立情绪。

（3）意志：在日常生活、游戏、学习中应该有意识培养儿童克服困难的意志，增强其自觉、坚持、果断和自制的能力。

（4）社交能力：从小给予儿童积极愉快的感受，如：喂奶时不断抚摸孩子；与孩子眼对眼微笑说话；抱孩子，和其说话、唱歌；孩子会走后，常与孩子做游戏、讲故事，这些都会增强孩子与周围环境和谐一致的生活能力。注意培养儿童之间互相友爱，鼓励孩子帮助朋友，倡导善良的品德。在游戏中学习遵守规则，团结友爱，互相谦让，学习与人相处。

（5）创造能力：人的创造能力与想象能力密切有关。启发式地向儿童提问题，引导儿童

自己去发现问题和探索问题，可促进儿童思维能力的发展。通过游戏、讲故事、绘画、听音乐、表演、自制小玩具等可以培养想象力和创造能力。

3．父母和家庭对儿童心理健康的作用　父母的教养方式和态度、与儿童的亲密程度等与儿童个性的形成和社会适应能力的发展密切相关。从小与父母建立相依感情的儿童，日后会有良好的社交能力和人际关系；父母对婴儿的咿呀学语作出及时的应答可促进儿童的语言和社会性应答能力的发展；婴儿期与母亲接触密切的儿童，其语言和智能发育较好。父母采取民主方式教育的儿童善与人交往，机灵、大胆而有分析思考能力；反之，如父母常打骂儿童，则儿童缺乏自信心、自尊心，他们的戒备心理往往使他们对他人的行为和意图产生误解。父母过于溺爱的儿童缺乏独立性、任性，且情绪不稳定。父母是孩子的第一任老师，应提高自身的素质，言行一致，以身作则教育儿童。

（五）定期健康检查

0～6岁的散居儿童和托幼机构的集体儿童应进行定期的健康检查，系统观察小儿的生长发育、营养状况，及早发现异常、采取相应干预措施。

1．新生儿访视　于新生儿出生28天内家访3～4次，高危儿应适当增加家访次数，主要由社区卫生服务中心的妇幼保健人员实施。家访的目的是早期发现问题，及时指导处理，降低新生儿的发病率或减轻发病的程度。家访内容包括：①了解新生儿出生情况；②回家后的生活情况；③预防接种情况；④喂养与护理指导；⑤体重测量；⑥体格检查，重点应注意有无产伤、黄疸、畸形、皮肤与脐部感染等。⑦咨询及指导。如在访视中发现严重问题应立即转医院诊治。

2．儿童保健门诊　应按照各年龄期保健需要，定期到固定的社区卫生服务中心儿童保健科进行健康检查，通过连续的纵向观察可获得个体儿童的体格生长和社会心理发育趋势，以早期发现问题、给予正确的健康指导。定期检查的频度：6 月以内婴儿每月1次，7～12个月婴儿则2～3月检查一次，高危儿、体弱儿宜适当增加检查次数。定期检查的内容包括：①体格测量及评价，3岁后每年测视力、血压一次；②全身各系统体格检查。③常见病的定期实验室检查，如缺铁性贫血、寄生虫病等，对临床可疑佝偻病、微量元素缺乏、发育迟缓等疾病应作相应的进一步检查。

（六）体格锻炼

1．户外活动　一年四季均可进行户外活动。户外活动可增加儿童对冷空气的适应能力，提高机体免疫力；接受日光直接照射还能预防佝偻病。带婴儿到人少、空气新鲜的地方，开始户外活动时间由每日1～2次，每次10～15分钟，逐渐延长到1～2小时；冬季户外活动时仅暴露面、手部，注意身体保暖。年长儿除恶劣气候外，鼓励多在户外玩耍。

2．皮肤锻炼

（1）婴儿皮肤按摩：按摩时可用少量婴儿润肤霜使之润滑，在婴儿面部、胸部、腹部、背部及四肢有规律的轻柔与捏握，每日早晚进行，每次15 分钟以上。按摩可刺激皮肤，有益于循环、呼吸、消化、肢体肌肉的放松与活动；同时也是父母与婴儿之间最好的情感交流方式之一。

（2）温水浴：温水浴可提高皮肤适应冷热变化的能力，还可促进新陈代谢，增加食欲。冬季应注意室温、水温，作好温水浴前的准备工作，减少体表热能散发。

（3）擦浴：7～8 个月以后的婴儿可进行身体擦浴。水温32～33℃，待婴儿适应后，水温可逐渐降至26℃。先用毛巾浸入温水，拧至半干，然后在婴儿四肢做向心性擦浴，擦毕再用干毛巾擦至皮肤微红。

（4）淋浴：适用于3岁以上儿童，效果比擦浴更好。每日一次，每次冲淋身体20～40秒钟，

水温35～36℃，浴后用干毛巾擦至全身皮肤微红。待儿童适应后，可逐渐将水温降至26～28℃。

3．体育运动

（1）婴儿被动操：被动操是指由成人给婴儿做四肢伸屈运动，可促进婴儿大运动的发育、改善全身血液循环，适用于2～6个月的婴儿，每日1～2次为宜。

（2）婴儿主动操：7～12个月婴儿大运动开始发育，可训练婴儿爬、坐、仰卧起身、扶站、扶走、双手取物等动作。

（3）幼儿体操：12～18 个月幼儿学走尚不稳时，在成人的扶持下，帮助婴儿进行有节奏的活动。18个月～3岁幼儿可配合音乐，做模仿操。

（4）儿童体操：如广播体操、健美操，以增进动作协调性，有益于肌肉骨骼的发育。

（5）游戏、田径与球类：年长儿可利用器械进行锻炼，如木马、滑梯，还可进行各种田径、球类、舞蹈、跳绳等活动。

（七）儿童伤害预防

儿童意外伤害是5岁以下儿童死亡的首位原因，但是可以预防的。

1．窒息与异物吸入　3个月以内的婴儿应注意防止因被褥、母亲的身体、吐出的奶液等造成的窒息；较大婴幼儿应防止食物、果核、果冻、钮扣、硬币等异物吸人气管。

2．中毒　保证儿童食物的清洁卫生，防止食物在制作、储备、出售过程中处理不当所致的细菌性食物中毒。避免食用有毒的食物，如毒蘑菇、含氰果仁（苦杏仁、桃仁、李仁等）、白果仁（白果二酸）、河豚、鱼苦胆等。药物应放置儿童拿不到的地方；儿童内、外用药应分开放置，防止误服外用药造成的伤害。

3．外伤　婴幼儿居室的窗户、楼梯、阳台、睡床等都应置有栏杆，防止从高处跌落。妥善放置开水、高温的油和汤等，以免造成烫伤。教育儿童不可随意玩火柴、煤气等危险物品。室内电器、电源应有防止触电的安全装置。

4．溺水与交通事故　教育儿童不可独自或与小朋友去无安全措施的江河、池塘玩水。教育儿童遵守交通规则。

5．教会孩子自救　如家中发生火灾拨打119，遭受外来人的侵犯拨打110，意外伤害急救拨打120电话。

（沈晓明）

第四章 儿科疾病诊治原则

第一节 儿科病史采集和体格检查

儿科的病史采集、记录和体格检查在内容、程序、方法以及分析判断等方面具有自身的特点，故在要求上与成人有一定差别。熟练掌握与此有关的方法和技巧，是开展儿科临床诊疗工作的基础。

医学的进步以及整体诊疗水平的提高，对医生运用系统医学知识、临床基本技能及正确的临床系统思维提出了更高的要求、熟练而规范地采集病史和进行体格检查并正规书写病历对培养临床综合能力和确立疾病的诊断十分重要。临床实验室的发展和医疗诊断设备的更新，为疾病的诊断提供了更多、更精确的手段，但准确的病史资料的采集和体格检查永远是正确诊断疾病的重要的基础。病历记录则是最重要的医疗证据。

一、病史采集和记录

病史采集要准确。其要点是认真听、重点问，关键是从家长或监护人提供的信息中发现对病情诊断有用的线索。在病史询问过程中态度要和蔼亲切，语言要通俗易懂，要注重与家长的沟通，要让家长感觉到医护人员对孩子的关爱，以取得家长和孩子的信任，同时要尊重家长和孩子的隐私，并为其保密。切不可先入为主，尤其不能用暗示的言语或语气来诱导家长主观期望的回答，这样会给诊断造成困难。病史采集内容包括：

1．一般内容 正确记录患儿的姓名、性别、年龄（采用实际年龄：新生儿记录天数、婴儿记录月数、1岁以上记录几岁几个月）、种族、父母或抚养人的姓名、职业、年龄、文化程度、家庭住址及（或）其它联系方式（如电话）、病史叙述者与病儿的关系以及病史的可靠程度。

2．主诉 用病史提供者的语言概括主要症状或体征及其时间。例如："间歇腹痛3天"、"持续发烧5天"。

3．现病史 为病历的主要部分。详细描述此次患病的情况，包括主要症状、病情发展和诊治经过。要特别注意以下几点：①主要症状要仔细询问，要注意症状的特征，如咳嗽的询问应包括：持续性还是间断性、剧烈还是轻咳、单声或连续性、阵发性咳嗽、有无鸡鸣样吼声、有无痰及其性状，咳嗽在一日中何时较重，有无任何伴随症状及诱因等；②有鉴别意义的有关症状包括阴性症状，也要询问并记录在病史中；③病后小儿的一般情况，如精神状态、吃奶或食欲情况、大小便、睡眠等以及其他系统的症状；④已经做过的检查和结果；⑤已经进行治疗的病人要询问用药的情况，如药物名称、剂量、给药方法、时间、治疗的效果及有无不良反应等。

4．个人史 包括出生史、喂养史、生长发育史，根据不同的年龄和不同的疾病在询问时各有侧重详略。

（1）出生史：母孕期的情况；第几胎第几产，出生体重；分娩时是否足月、早产或过期产；生产方式，出生时有无窒息或产伤，Apgar评分情况等。新生儿和小婴儿疑有中枢神经系统发育不全或智力发育迟缓等患儿，更应详细了解围生期有关的情况。

（2）喂养史：母乳喂养还是人工喂养或部分母乳喂养，以何种乳品为主，配制方法，喂哺次数及量，断奶时间，添加辅食的时间、品种及数量，进食及大、小便情况。年长儿还应注意了解有无挑食、偏食及吃零食的习惯。了解喂养情况对患有营养性或消化系统疾病的儿童尤为重要。

（3）生长发育史：常用的生长发育指标有：体重和身高以及增长情况，前囟关闭及乳牙萌出的时间等；发育过程中何时能抬头、会笑、独坐、站立和走路；何时会有意识地叫爸爸、妈妈。学龄儿童还应询问在校学习成绩和行为表现等。

5．既往史 包括以往疾病史和预防接种史。

（1）既往患病史：需详细询问既往患过的疾病、患病时间和治疗结果。应着重了解传染病史，如过去曾患过麻疹而此次有发热、皮疹的患儿，在综合分析时应多考虑其他发热出疹性疾病；认真了解有无药物或食物过敏史，并详细记录，以供治疗时参考。在年长儿或病程较长的疑难病例，应对各系统进行系统回顾。

（2）预防接种史：对常规接种的疫苗均应逐一询问。何时接受过何种预防接种，具体次数，有无反应。接种非常规的疫苗也应记录。

6．家族史 家族中有无遗传性、过敏性或急、慢性传染病患者；如有，则应详细了解与患儿接触的情况。父母是否近亲结婚、母亲分娩情况、同胞的健康情况（死亡者应了解原因和死亡年龄）。必要时要询问家庭成员及亲戚的健康状况、家庭经济情况、居住环境、父母对患儿的关爱程度和对患儿所患疾病的认识等。

7．传染病接触史 疑为传染性疾病者，应详细了解可疑的接触史，包括患儿与疑诊或确诊传染病者的关系、该患者的治疗经过和归转、患儿与该患者的接触方式和时间等。了解父母对传染病的认识和基本知识也有助于诊断。

二、体格检查

为了获得准确无误的体格检查资料，在采集病史时要创造一种自然轻松的气氛，以尽可能取得患儿的合作，而医生的表现是决定母亲和孩子合作程度的主要因素。

（一）体格检查的注意事项

1．询问病史时就应该开始和患儿建立良好的关系。微笑、呼患儿的名字或小名、乳名、用表扬语言鼓励患儿或用手轻轻抚摸他，可以使患儿消除紧张心理；也可用听诊器或其他玩具逗患儿玩耍以消除或减少恐惧，取得患儿的信任和合作；并同时观察患儿的精神状态、对外界的反应及智力情况。

2．为增加患儿的安全感，检查时应尽量让孩子与亲人在一起，婴幼儿可坐或躺在家长的怀里检查，检查者顺应患儿的体位。

3．检查的顺序可根据患儿当时的情况灵活掌握。由于婴幼儿注意力集中时间短，因此在体格检查时应特别记住以下要点：安静时先检查心、肺听诊、心率、呼吸次数或腹部触诊等易受哭闹影响的项目，一般在患儿开始接受检查时进行；容易观察的部位随时查，如四肢、躯干、骨骼、全身浅表淋巴结等；对患儿有刺激而患儿不易接受的部位最后查，如口腔、咽部等，有疼痛的部位也应放在最后检查。

4．检查时态度和蔼，动作轻柔，冬天时双手及所用听诊器胸件要温暖。检查过程中既要全面仔细，又要注意保暖，不要过多暴露身体部位以免着凉。对年长儿还要照顾他（她）们的害羞心理和自尊心。

5．对急症或危重抢救病例，应先重点检查生命体征或与疾病有关的部位，全面的体检最好在病情稍稳定后进行，也可边抢救边检查。

6．小儿免疫功能差，为防止交叉感染，检查前后均应清洗双手，使用一次性或消毒后的压舌板；检查者的工作衣和听诊器要勤消毒。

（二）检查方法

1．一般状况 询问病史的过程中，留心观察小儿的营养发育情况、神志、表情、对周围事物的反应、皮肤颜色、体位、行走姿势和孩子的语言能力等，由此得到的资料较为真实，可供正确判断一般情况。

2．一般测量 包括体温、呼吸、脉搏、血压，还有身长、体重、头围、胸围等。

（1）体温：可根据小儿的年龄和病情选用测温的方法：①腋下测温法：最常用，也最安全、方便，但测量的时间偏长。将消毒的体温表水银头放在小儿腋窝中，将上臂紧压腋窝，保持5～10分钟，36～37℃为正常。②口腔测温法：准确、方便，保持3分钟，37℃为正常，用于神志清楚而且配合的6岁以上的小儿。③肛门内测温法：测温时间短、准确。小儿取侧卧位，下肢屈曲，将已涂满润滑油的肛表水银头轻轻插入肛门内3～4cm，测温3～5分钟，36.5～37.5℃为正常，1岁以内小儿、不合作的儿童以及昏迷、休克患儿可采用此方法。④耳内测温法：准确、快速，不会造成交叉感染，但仪器贵。目前临床比较少用。

（2）呼吸、脉搏：应在小儿安静时进行。小儿呼吸频率可通过听诊或观察腹部起伏而得，也可将棉花少许置于小儿鼻孔边缘，观察棉花纤维的摆动而得。要同时观察呼吸的节律和深浅。对年长儿一般选择较浅的动脉如桡动脉来检查脉搏，婴幼儿亦可检查股动脉或通过心脏听诊来检测。要注意脉搏的速率、节律、强弱及紧张度。各年龄组小儿呼吸脉搏正常值见表4-1。

表4-1 各年龄小儿呼吸、脉搏（次数/分）

年龄	呼吸	脉搏	呼吸：脉搏
新生儿	40～45	120～140	1：3
＜1岁	30～40	110～130	1：3～1：4
1～3岁	25～30	100～120	1：3～1：4
4～7岁	20～25	80～100	1：4
8～14岁	18～20	70～90	1：4

（3）血压：测量血压时应根据不同的年龄选择不同宽度的袖带，袖带的宽度通常应为上臂长度的1/2～2/3。袖带过宽时测得的血压值较实际值偏低，过窄时则较实际值为高。新生儿多采用多普勒超声监听仪或心电监护仪测定血压，也可用简易潮红法测量：测量时使患婴仰卧位，将气带包裹于腕部（或踝部）以上，然后用加压绑带从肢体远端指（趾）尖向上，连续包裹至气带处，打气使压力达200mmHg或收缩压正常高限以上，将压力绑带去除，只见手或足的皮肤均泛白，然后以每秒种降低5mmHg的速度放气，当气带远端手（或足）的皮肤刚出现潮红时，即为平均压；若有严重贫血、水肿及明显低温，则可影响观察结果。年龄越小，血压越低。不同年龄小儿血压的正常值可用公式推算：收缩压（mmHg）=80+（年龄×2）；舒张压应该为收缩压的2/3。mmHg与kPa的换算为：mmHg测定值÷7.5＝kPa值。

3．皮肤和皮下组织：应在自然光线下仔细观察才准确。在保暖的前提下仔细观察身体各部位皮肤的颜色，有无苍白、黄染、发绀、潮红、皮疹、瘀点（斑）、脱屑、色素沉着，毛发有无异常，触摸皮肤的弹性、皮下组织及脂肪的厚度，有无水肿及水肿的性质。

4．淋巴结：包括淋巴结的大小、数目、活动度、质地、有无粘连和（或）压痛等。颈部、耳后、枕部、腹股沟等部位尤其要认真检查，正常情况下在这些部位可触及单个质软的黄豆大小的淋巴结，活动，无压痛。

5．头部：

（1）头颅：观察大小、形状，必要时测量头围；前囟大小及紧张度、有无凹陷或隆起；

小婴儿要观察有无枕秃和颅骨软化、血肿或颅骨缺损等。

（2）面部：有无特殊面容，眼距宽窄，鼻梁高低，注意双耳位置和形状等。

（3）眼、耳、鼻：有无眼睑浮肿、下垂、眼球突出、斜视、结膜充血、眼分泌物、角膜混浊、瞳孔大小、形状、对光反射。检查双外耳道有无分泌物、局部红肿及外耳牵拉痛；若怀疑有中耳炎时应用耳镜检查鼓膜情况。观察鼻形，注意有无鼻翼扇动、鼻腔分泌物及通气情况。

（4）口腔：口唇色泽有无苍白、紫绀、干燥、口角糜烂、疱疹。口腔内颊粘膜、牙龈、硬腭有无充血、溃疡、黏膜斑、鹅口疮，腮腺开口处有无红肿及分泌物，牙齿数目及龋齿数，舌质、舌苔颜色。咽部检查放在体格检查最后进行，医生一手固定小儿头部使其面对光源，一手持压舌板，在小儿张口时进入口腔，压住舌后根部，利用小儿反射性将口张大暴露咽部的短暂时间，迅速观察双扁桃体是否肿大，有无充血、分泌物、脓点、伪膜及咽部有无溃疡、充血、滤泡增生、咽后壁脓肿等情况。

6．颈部：颈部是否软，有无斜颈、短颈或颈蹼等畸形，颈椎活动情况；甲状腺有无肿大，气管位置；颈静脉充盈及搏动情况，有无颈肌张力增高或弛缓等。

7．胸部：

（1）胸廓：注意有无鸡胸、漏斗胸、肋骨串珠、肋膈沟、肋缘外翻等佝偻病的体征；胸廓两侧是否对称，心前区有无隆起，有无桶状胸，肋间隙饱满、凹陷、增宽或变窄等。

（2）肺：视诊应注意呼吸频率和节律有无异常，有无呼吸困难和呼吸深浅改变；吸气性呼吸困难时可出现“三凹征”，即胸骨上窝、肋间隙和剑突下在吸气时向内凹陷；呼气性呼吸困难时可出现呼气延长。触诊在年幼儿可利用啼哭或说话时进行。因小儿胸壁薄，叩诊反响比成人轻，故叩诊时用力要轻或可用直接叩诊法，用两个手指直接叩击胸壁。听诊时正常小儿呼吸音较成人响，呈支气管肺泡呼吸音，应注意听腋下、肩胛间区及肩胛下区有无异常，因肺炎时这些部位较易听到湿性啰音。听诊时尽量保持小儿安静，小儿啼哭后深吸气时容易闻及细湿啰音。

（3）心：视诊时观察心前区是否隆起，心尖搏动强弱和搏动范围，正常小儿心尖搏动范围在2～3cm^2之内，肥胖小儿不易看到心尖搏动。触诊主要检查心尖搏动的位置及有无震颤，并应注意出现的部位和性质（收缩期、舒张期或连续性）。通过叩心界可估计心脏大小、形状及其在胸腔的位置，叩诊心界时用力要轻才易分辨清、浊音界线，3岁以内婴幼儿一般只叩心脏左右界；叩左界时从心尖搏动点左侧起向右叩，听到浊音改变即为左界，记录为第几肋间左乳线外或内几厘米；叩右界时先叩出肝浊音界，然后在其上一肋间自右向左叩，有浊音改变时即为右界，以右胸骨线（胸骨右缘）外几厘米记录。各年龄小儿心界参考表4-2。小儿心脏听诊应在安静环境中进行，听诊器的胸件要小。小婴儿第一心音与第二心音响度几乎相等；随年龄的增长，心尖部第一心音较第二音响，而心底部第二音超过第一音。小儿时期肺动脉瓣区第二音比主动脉瓣区第二音响（$P_2>A_2$），有时可出现吸气性第二心音分裂。学龄前期及学龄儿童常于肺动脉瓣区或心尖部听到生理性收缩期杂音或窦性心律不齐。

表4-2　各年龄小儿心界

年龄	左界	右界
＜1岁	左乳线外1～2cm	沿右胸骨旁线
1～4岁	左乳线外1cm	右胸骨旁线与右胸骨之间
5～12岁	左乳线上或乳线内0.5～1cm	接近右胸骨线
12岁	左乳线内0.5～1cm	右胸骨线

8．腹部　视诊在新生儿或消瘦小儿常可见到肠型或肠蠕动波，新生儿应注意脐部有无分泌物、出血、炎症，脐疝大小。触诊应尽量争取小儿的合作，可让其躺在母亲怀里或在哺乳时进行，检查者的手应温暖、动作轻柔，如小儿哭闹不止，可利用其吸气时作快速扪诊。检查有无压痛主要观察小儿表情反应，不能完全依靠小儿回答。正常婴幼儿肝脏可在肋缘下1～2cm处扪及，柔软无压痛；6～7岁后不应在肋下触及。小婴儿偶可触及脾脏边缘。叩诊可采用直接叩诊或间接叩诊法，其检查内容与成人相同。小儿腹部听诊有时可闻及肠鸣音亢进，如有血管杂音时应注意杂音性质、强弱及部位。

9．脊柱和四肢　注意有无畸形、躯干与四肢比例和佝偻病体征，如“O”型或“X”型腿、手镯、脚镯样变、脊柱侧弯等；观察手、足指（趾）有无杵状指、多指（趾）畸形等。

10．会阴肛门和外生殖器　观察有无畸形（如先天性无肛、尿道下裂、两性畸形）、肛裂；女孩有无阴道分泌物、畸形；男孩有无隐睾、包皮过长、过紧、鞘膜积液和腹股沟疝等。

11．神经系统　根据病种、病情、年龄等选择必要的检查。

（1）一般检查：观察小儿的神志、精神状态、面部表情、反应灵敏度、动作语言能力、有无异常行为等。

（2）神经反射：新生儿期特有的反射如吸吮反射、拥抱反射、握持反射是否存在。有些神经反射有其年龄特点，如新生儿和小婴儿期提睾反射、腹壁反射较弱或不能引出，但跟腱反射亢进，并可出现踝阵挛；2岁以下的小儿Babinski征可呈阳性，但一侧阳性，另一侧阴性则有临床意义。

（3）脑膜刺激征：如颈部有无抵抗、Kernig征和Brudzinski征是否阳性，检查方法同成人。如小儿不配合，要反复检查才能正确判定。正常小婴儿由于在胎内时屈肌占优势，故生后头几个月Kernig征和Brudzinski征也可阳性。因此，在解释检查结果意义时一定要根据病情、结合年龄特点全面考虑。

（三）体格检查记录方法

体格检查项目虽然在检查时无一定顺序，但结果记录应按上述顺序书写；不仅阳性体征应记录，重要的阴性体征结果也要记录。

第二节 儿科疾病治疗原则

儿童阶段是一个生长发育的连续过程，不同年龄阶段的小儿在生理、病理和心理特点上各异，在发病原因、疾病过程和转归等方面与成年人更有不同之处，因此在疾病的治疗和处理上须充分考虑年龄因素。不同年龄小儿的表达能力不同，更增加了儿科医护人员在治疗过程中观察和判断的难度。由于小儿起病急，变化快，容易并发一个甚至多个器官或系统病变，故治疗措施既要适时、全面，又要仔细、突出重点；且在疾病的治疗过程中较成年人更需要爱心、耐心和精湛的医术，任何一个不恰当的处理方法或方式，都可能对小儿生理和心理等方面产生较长久甚至终身的不良影响，要求儿科临床工作者必须熟练掌握护理、饮食、用药和心理等各方面的治疗技术，使患儿身心顺利康复。

一、护理的原则

在疾病治疗过程中，儿科护理是极为重要的一个环节，许多治疗操作均通过护理工作来实施。良好的护理在促进患儿康复中起着很大的作用。护理工作不仅是护士的工作，儿科医师应关心和熟悉护理工作，医护密切协作，以提高治疗效果。

1．细致的临床观察　临床所观察到的患儿不典型的或细微的表现，都应考虑其可能存在的病理基础。如婴儿哭闹可以是正常的生理要求，也可能是疾病的表现，细致的观察是鉴别两者的关键。

2．合理的病室安排　病室要整齐、清洁、安静、舒适，空气新鲜、流通，温度适宜。为提高治疗和护理的质量，可按年龄、病种、病情轻重和护理要求合理安排病房及病区：①按年龄分病区：如新生儿和早产儿病室、年长儿病室、小婴儿病室等；②按病种分病区：将同类病儿集中管理，传染病则按病种隔离；③按病情分病房：重危者收住抢救监护病室，恢复期病儿可集中一室。

3．规律的病房生活　保证充足的睡眠和休息很重要，观察病情应尽量不影响患儿的睡眠，尽可能集中时间进行治疗和诊断操作，定时进餐。

4．预防医源性疾病等 ① 防止交叉感染：医护人员在接触患儿之前、后均应洗手，病室要定时清扫、消毒；② 防止医源性感染：正确、规范地应用导尿、穿刺等各种治疗方法，定时检查消毒设备，防止感染的发生；③ 防止意外的发生：医护人员检查、处理完毕后要及时拉好床栏，所用物品如体温表、药杯等用毕即拿走，以免小儿玩耍误伤，喂药、喂奶要将婴儿抱起，避免呛咳、呕吐引起窒息。

二、饮食治疗原则

根据病情选择适当的饮食有助于治疗和康复；不当的饮食可使病情加重，甚至危及生命。母乳喂养儿应继续喂以母乳。

1. 乳品 ①稀释乳：供新生儿、早产儿食用；②脱脂奶：半脱脂或全脱脂奶，脂肪含量低. 只供腹泻时或消化功能差者短期食用；③酸奶：牛乳加酸或经乳酸杆菌发酵成酸奶，其蛋白凝块小、易消化，供腹泻及消化力弱的病儿食用；④豆奶：适用于乳糖不耐受和牛乳过敏的小儿；⑤无乳糖奶粉（不含乳糖，含蔗糖、葡萄糖聚合体、麦芽糖糊精、玉米糖浆）：长期腹泻、有乳糖不耐受的婴儿应使用无乳糖奶粉；⑥低苯丙氨酸奶粉：用于确诊为苯丙酮尿症的婴儿。

2．一般膳食 ①普通饮食：采用易消化、营养丰富、热能充足的食物；②软食：将食物烹调得细、软、烂，介于普通饮食和半流质饮食之间，如稠粥、烂饭、面条、馒头、肉末、鱼羹等，使之易于消化，适用于消化功能尚未完全恢复或咀嚼能力弱的病儿；③半流质饮食：呈半流体状或羹状，介于软食和流质饮食之间，由牛乳、豆浆、稀粥、烂面、蒸蛋羹等组成，可另加少量饼干、面包，适用于消化功能尚弱，不能咀嚼吞咽大块固体食物的病儿；④流质饮食：全部为液体，如牛乳、豆浆、米汤、蛋花汤、藕粉、果汁、牛肉汤等，不需咀嚼就能吞咽，且易于消化吸收，适用于高热、消化系统疾病、急性感染、胃肠道手术后病儿，亦用于鼻饲。流质饮食供热能与营养素均低，只能短期应用。

3．特殊膳食　①少渣饮食：纤维素含量少，对胃肠刺激性小，易消化，适用于胃肠感染、肠炎病儿；②无盐及少盐饮食：无盐饮食每日食物中含盐量在3g以下，烹调膳食不另加食盐；少盐饮食则每天额外供给1g氯化钠，供心力衰竭和肝、肾疾病导致的水肿患儿食用；③贫血饮食：每日增加含铁食物，如动物血、动物肝、各种肉类等；④高蛋白膳食：在一日三餐中添加富含蛋白质的食物，如鸡蛋、鸡、瘦肉、肝或豆制品等，适用于营养不良、消耗性疾病患儿；⑤低脂肪饮食：膳食中不用或禁用油脂、肥肉等，适用于肝病患儿；⑥低蛋白饮食：膳食中减少蛋白质含量，以糖类如马铃薯、甜薯、水果等补充热量，用于尿毒症、肝昏迷和急性肾炎的少尿期患儿；⑦低热能饮食：一日三餐的普通饮食中减少脂肪和糖类的含量，又要保证蛋白质和维生素的需要量，可选用鱼、蛋、豆类、蔬菜和瘦肉等，用于单纯性肥胖症的小儿；⑧代谢

病专用饮食：如不含乳糖食物用于半乳糖血症病儿，低苯丙氨酸奶用于苯丙酮尿症小儿，糖尿病饮食等。

4．检查前饮食 在进行某些化验检查前对饮食有特别的要求，如：①潜血膳食：连续3天食用不含肉类、动物肝脏、血和绿叶蔬菜等的饮食，用于消化道出血的检查；②胆囊造影膳食：用高蛋白、高脂肪膳食如油煎荷包蛋等使胆囊排空，以检查胆囊和胆管功能；③干膳食：食用米饭、馒头、鱼、肉等含水分少的食物，以利于尿浓缩功能试验和12小时尿细胞计数等检查。

5．禁食 因消化道出血或术后等原因不能进食小儿，应注意静脉供给热量，并注意水、电解质平衡。

三、药物治疗原则

药物是治疗疾病的一个重要手段，而药物的过敏反应、副作用和毒性作用常对机体产生不良影响。生长发育中的小儿因器官功能发育尚不够成熟、健全，对药物的毒、副作用较成年人更为敏感。小儿疾病多变，选择药物须慎重，更要求剂量恰当，因此必须充分了解小儿药物治疗的特点，掌握药物性能、作用机制、毒副作用、适应证和禁忌证，以及精确的剂量计算和适当的用药方法。

（一）儿科药物治疗的特点

由于药物在体内的分布受体液的pH值、细胞膜的通透性、药物与蛋白质的结合程度、药物在肝脏内的代谢和肾脏排泄等因素的影响，小儿期的药物治疗具有下述特点。

1．药物在组织内的分布因年龄而异　如巴比妥类、吗啡、四环素在幼儿脑浓度明显高于年长儿。

2．小儿对药物的反应因年龄而异　吗啡对新生儿呼吸中枢的抑制作用明显高于年长儿，麻黄碱使血压升高的作用在未成熟儿却低得多。

3．肝脏解毒功能不足　特别是新生儿和早产儿，肝脏酶系统发育不成熟，对某些药物的代谢延长，药物的半衰期延长，增加了药物的血浓度和毒性作用。

4．肾脏排泄功能不足　新生儿特别是未成熟儿的肾功能尚不成熟，药物及其分解产物在体内滞留的时间延长，增加了药物的毒副作用。

5．先天遗传因素　要考虑家族中有遗传病史的患儿对某些药物的先天性异常反应；对家族中有药物过敏史者要慎用某些药物。

（二）药物选择

选择用药的主要依据是小儿年龄、病种和病情，同时要考虑小儿对药物的特殊反应和药物的远期影响。

1．抗生素　小儿容易患感染性疾病，故常用抗生素等抗感染药物。儿科工作者既要掌握抗生素的药理作用和用药指征，更要重视其毒、副作用的一面。对个体而言，除抗生素本身的毒副作用而外，过量使用抗生素还容易引起肠道菌群失衡，使体内微生态紊乱，引起真菌或耐药菌感染；对群体和社会来讲，广泛、长时间地滥用广谱抗生素，容易产生微生物对药物的耐受性、进而对人们的健康产生极为有害的影响。临床应用某些抗生素时必须注意其毒副作用，如肾毒性、对造血功能的抑制作用等。

2．肾上腺皮质激素　短疗程常用于过敏性疾病、重症感染性疾病等；长疗程则用于治疗肾病综合征、某些血液病、自身免疫性疾病等。哮喘、某些皮肤病则提倡局部用药。在使用中必须重视其副作用：① 短期大量使用可掩盖病情，故诊断未明确时一般不用；② 较长期使用可抑制骨骼生长，影响水、电解质、蛋白质、脂肪代谢，也可引起血压增高和库欣综合征；③

长期使用除以上副作用以外，尚可导致肾上腺皮质萎缩，可降低免疫力使病灶扩散；④ 水痘患儿禁用激素，以防加重病情。

3．退热药　一般使用对乙酰氨基酚和布洛芬，剂量不宜过大，可反复使用。

4．镇静止惊药　在患儿高热、烦躁不安、剧咳不止等情况下可考虑给予镇静药。发生惊厥时可用苯巴比妥、水合氯醛、地西泮等镇静止惊药。婴儿不宜使用阿司匹林，以免发生Reye综合征。

5．镇咳止喘药　婴幼儿一般不用镇咳药，多用祛痰药口服或雾化吸入，使分泌物稀释、易于咳出。哮喘病儿提倡局部吸入β_2受体激动剂类药物，必要时也可用茶碱类，但新生儿、小婴儿慎用。

6．止泻药与泻药　对腹泻患儿慎用止泻药，除用口服补液疗法防治脱水和电解质紊乱外，可适当使用保护肠黏膜的药物，或辅以含双歧杆菌或乳酸杆菌的制剂以调节肠道的微生态环境。小儿便秘一般不用泻药，多采用调整饮食和松软大便的通便法。

7．乳母用药　阿托品、苯巴比妥、水杨酸盐等药物可经母乳影响哺乳婴儿，应慎用。

8，新生儿、早产儿用药　幼小婴儿的肝、肾等代谢功能均不成熟，不少药物易引起毒副反应，如磺胺类药、维生素K_3可引起高胆红素血症，氯霉素引起“灰婴综合征”等，故应慎重。

（三）给药方法

根据年龄、疾病及病情选择给药途径、药物剂型和用药次数，以保证药效和尽量减少对病儿的不良影响。在选择给药途径时，应尽量选用患儿和患儿家长可以接受的方式给药。

1．口服法　是最常用的给药方法。幼儿用糖浆、水剂、冲剂等较合适，也可将药片捣碎后加糖水吞服，年长儿可用片剂或药丸。小婴儿喂药时最好将小儿抱起或头略抬高，以免呛咳时将药吐出。病情需要时可采用鼻饲给药。

2．注射法　注射法比口服法奏效快，但对小儿刺激大，肌肉注射次数过多还可造成臀肌挛缩、影响下肢功能，故非病情必需不宜采用。肌肉注射部位多选择臀大肌外上方；静脉推注多在抢救时应用；静脉滴注应根据年龄大小、病情严重程度控制滴速。

在抗生素应用时间较长时，提倡使用续贯疗法，以提高疗效和减少抗生素的副作用。

3．外用药　以软膏为多，也可用水剂、混悬剂、粉剂等。要注意小儿用手抓摸药物，误入眼、口引起意外。

4．其他方法　雾化吸入常用；灌肠法小儿采用不多，可用缓释栓剂；含剂、漱剂很少用于小龄儿，年长儿可采用。

（四）药物剂量计算

儿科用药剂量较成人更须准确。可按以下方法计算：

1．按体重计算　是最常用、最基本的计算方法，可算出每日或每次需用量：每日（次）剂量＝病儿体重（kg）× 每日（次）每千克体重所需药量。须连续应用数日的药，如抗生素、维生素等，都按每日剂量计算，再分2～3 次服用；而临时对症用药如退热、催眠药等，常按每次剂量计算。病儿体重应以实际测得值为准。年长儿按体重计算如已超过成人量则以成人量为上限。

2．按体表面积计算　此法较按年龄、体重计算更为准确，因其与基础代谢、肾小球滤过率等生理活动的关系更为密切。小儿体表面积计算公式为：

如体重≤30 kg，小儿的体表面积（m^2）＝体重（kg）×0.035+0.1；

如体重≥30 kg，小儿的体表面积（m^2）＝（体重kg－30）×0.02+1.05。

3．按年龄计算　剂量幅度大、不需十分精确的药物，如营养类药物等可按年龄计算，比

较简单易行。

4．从成人剂量折算　小儿剂量＝成人剂量×小儿体重（kg）/50，此法仅用于未提供小儿剂量的药物，所得剂量一般都偏小，故不常用。

采用上述任何方法计算的剂量，还必须与病儿具体情况相结合，才能得出比较确切的药物用量，如：新生儿或小婴儿肾功能较差，一般药物剂量宜偏小；但对新生儿耐受较强的药物如苯巴比妥，则可适当增大用量；重症患儿用药剂量宜比轻症患儿大；须通过血脑屏障发挥作用的药物，如治疗化脓性脑膜炎的磺胺类药或青霉素类药物剂量也应相应增大。用药目的不同，剂量也不同，如阿托品用于抢救中毒性休克时的剂量要比常规剂量大几倍到几十倍。

四、心理治疗原则

儿童心理治疗是指根据传统的和现代的心理分析与治疗理论而建立的系统治疗儿童精神问题的方法，可分为个体心理治疗、群体治疗和家庭治疗等；包括儿童心理、情绪和行为问题，精神性疾病和心身性疾病等。

随着医学模式的转变，对小儿的心理治疗或心理干预不再仅仅是儿童心理学家和儿童精神病学家的工作，而应该贯穿于疾病的诊治过程中。由于心理因素在儿科疾病的治疗、康复中的重要性和普遍性越来越明显，要求儿科工作者在疾病的治疗中重视各种心理因素，学习儿童心理学的基本原理，掌握临床心理治疗和心理护理的基本方法。

儿童的心理、情绪障碍，如焦虑、退缩、抑郁和恐怖等，常常发生在一些亚急性、慢性非感染性疾病的病程中，尤其是在神经系统、内分泌系统、消化系统、循环和泌尿系统等疾病，在门诊及住院治疗的过程中容易发生心理和情绪障碍。心理和情绪障碍既是疾病的后果，又可能是使病情加重或是使治疗效果不佳的原因之一。心身性疾患产生的一些突出症状，如慢性头痛、腹痛、腹泻等常与器质性病变相交织，使已经存在的疾患变得更加顽固和复杂。

常用的心理治疗包括支持疗法、行为疗法、疏泄法等，对初次治疗者要细心了解、观察，不强求儿童改变其行为以适合治疗者的意愿，要尊重儿童有自我改善的潜在能力，以暗示和循循善诱帮助儿童疏泄其内心郁积的压抑，激发其情绪释放，以减轻其心理和精神障碍的程度，促进原发病的康复。

患病使小儿产生心理负担，又进入陌生的医院环境，容易焦虑、紧张甚至恐怖。常见的症状为出现哭闹或沉默寡言、闷闷不乐，有的患儿拒谈、拒绝治疗或整夜不眠。安静、舒适和整洁的环境，亲切的语言、轻柔的动作、和蔼的面孔和周到的服务是改善患儿症状的关键。护理人员应通过细致的观察使心理护理个体化，获得患儿的信任和配合，促进疾病的痊愈和身心的康复。

（毛萌）

第三节　小儿液体平衡的特点和液体疗法

一、小儿液体平衡的特点

体液是人体的重要组成部分，保持其生理平衡是维持生命的重要条件。体液中水、电解质、酸碱度、渗透压等的动态平衡依赖于神经、内分泌、肺，特别是肾脏等系统的正常调节功能。儿童的水、电解质、酸碱及食物成分按单位体重的进出量大，尤其是婴儿在生后数月内肾功能不如成人健全，常不能抵御及纠正水或酸碱平衡紊乱，其调节功能极易受疾病和外界环境的影

响而失调。由于这些生理特点，水、电解质和酸碱平衡紊乱在儿科临床中极为常见。

（一）体液的总量与分布

体液的总量分布于血浆、间质及细胞内，前两者合称为细胞外液。年龄愈小，体液总量相对愈多，这主要是间质液的比例较高，而血浆和细胞内液量的比例则与成人相近。在妊娠早期，胎儿单位体重水的比例相当大，随着妊娠的进程，胎儿体内实质部分逐渐增加，水的比例逐渐下降。在胎儿期，25周时体液占体重的85%，其中细胞外液占60%；28周时占体重的80%；在足月儿，体液总量占体重的72～78%。在新生儿早期，常有体液的迅速丢失，可达体重的5%或更多，即所谓的生理性体重下降，此时婴儿逐渐适应子宫外的环境。经此调节后，体液约占体重的65%，在8岁时体液占体重的60%，达到成人水平。体液占体重的比例在婴儿及儿童时期相对保持恒定。在青春期，开始出现因性别不同所致的体内成分不同。正常性成熟男性肌肉总量较多而脂肪较少，而女性则有较多的脂肪、较少的肌肉组织。由于体内脂肪在男、女性别间的差异，体液总量在男性占体重的60%，而在女性为55%。不同年龄的体液分布见表4-3。

表4-3　不同年龄的体液分布（占体重的%）

年龄	细胞外液			细胞内液
	总量	血浆	间质液	
足月新生儿	78	6	37	35
1岁	70	5	25	40
2～14岁	65	5	20	40
成人	55～60	2	10～15	40～45

（二）体液的电解质组成

细胞内液和细胞外液的电解质组成有显著的差别。细胞外液的电解质成分能通过血浆精确地测定。正常血浆阳离子主要为Na^{+}、K^{+}、Ca^{2+}，和Mg^{2+}，其中Na^{+}含量占该区阳离子总量的90%以上，对维持细胞外液的渗透压起主要作用。血浆主要阴离子为Cl^{-}、HCO_3^{-}和蛋白质，这3种阴离子的总电荷与总阴离子电位差称为未确定阴离子（undetermined anion，UA），主要由无机硫和无机磷、有机酸如乳酸、酮体等组成。组织间液的电解质组成除Ca^{2+}含量较血浆低一半外，其余电解质组成与血浆相同。细胞内液的电解质测定较为困难，且不同的组织间有很大的差异。细胞内液阳离子以K^{+}、Ca^{2+}、Mg^{2+}、和Na^{+}为主，其中K^{+}占78%。阴离子以蛋白质、HCO_3^{-}、HPO_4^{2-}和Cl^{-}等离子为主。

（三）儿童水的代谢特点

健康小儿尽管每天的水和电解质摄入量有很大的波动，但体内液体和电解质的含量保持相当的稳定，即水的摄入量大致等于排泄量。

1．水的生理需要量　水的需要量与新陈代谢、摄入热量、食物性质、经肾排出溶质量、不显性失水、活动量及环境温度有关。儿童水的需要量大，交换率快，其主要原因为小儿生长发育快；活动量大、机体新陈代谢旺盛；摄入热量、蛋白质和经肾排出的溶质量均较高；体表面积相对大、呼吸频率快，使不显性失水较成人多。细胞组织增长时需积蓄水分也可增加水的摄入，但以每天计算，其量是很少的。按体重计算，年龄愈小，每日需水量愈多。不同年龄小儿每日所需水量见表4-4。早期新生儿每日需液量见新生儿章节。

表4-4　小儿每日水的需要量

年龄	需水量（ml/kg）
＜1岁	120～160
1～3岁	100～140
4～9岁	70～110
10～14岁	50～90

2．水的排出 机体主要通过肾（尿）途径排出水分，其次为经皮肤和肺的不显性失水和消化道（粪）排水，另有极少量的水贮存体内供新生组织增长。正常情况下，水通过皮肤和肺的蒸发，即不显性失水，主要用于调节体温。汗液属显性失水，也是调节体温的重要机制，与环境温度及机体的散热机制有关。不显性失水常不被引起注意，但在较小的早产儿其量是相当可观的。每天人体产生热量的1/4左右是通过皮肤和肺蒸发水分而丧失的，且往往是失去纯水，不含电解质。小婴儿尤其是新生儿和早产儿要特别重视不显性失水量，新生儿成熟度愈低、体表面积愈大、呼吸频率愈快、体温及环境温度愈高、环境的水蒸气压愈小以及活动量愈大，不显性失水量就愈多。不显性失水量不受体内水分多少的影响，即使长期不进水，机体也会动用组织氧化产生和组织中本身含有的水分来抵偿，故在供给水分时应将其考虑在常规补液的总量内。不同年龄小儿的不显性失水量见表4-5。

表4-5　不同年龄小儿的不显性失水量

不同年龄或体重	不显性失水量（ml/kg.d）
早产儿或足月新生儿	
750～1000g	82
1001～1250g	56
1251～1500g	46
>1500g	26
婴儿	19～24
幼儿	14～17
儿童	12～14

小儿排泄水的速度较成人快，年龄愈小，出入量相对愈多。婴儿每日水的交换量为细胞外液量的1/2，而成人仅为1/7，故婴儿体内水的交换率比成人快3～4倍。因婴儿对缺水的耐受力差，在病理情况下如进水不足同时又有水分继续丢失时，由于肾脏的浓缩功能有限，将比成人更易脱水。

3．水平衡的调节 肾脏是唯一能通过其调节来控制细胞外液容量与成分的重要器官。蛋白质的代谢产物尿素、盐类（主要为钠盐）是肾脏主要的溶质负荷，必须有足够的尿量使其排出。肾脏水的排出与抗利尿激素（ADH）分泌及肾小管上皮细胞对ADH的反应性有密切关系。正常引起ADH分泌的血浆渗透压阈值为280mOsm/L，血浆渗透压变化1～2%即可影响ADH的分泌。当液体丢失达总量的8%或以上时，ADH分泌即显著增加，严重脱水使ADH增加呈指数变化。

小儿的体液调节功能相对不成熟。正常情况下水分排出的多少主要靠肾脏的浓缩和稀释功能调节。肾功能正常时，水分摄入多，尿量就多；水分入量少或有额外的体液丢失（如大量出汗、呕吐、腹泻）而液体补充不足时，机体即通过调节肾功能，以提高尿比重、减少尿量的方式来排泄体内的代谢废物，最终使水的丢失减少。小儿年龄愈小，肾脏的浓缩和稀释功能愈不

成熟。新生儿和幼婴由于肾小管重吸收功能发育尚不够完善，其最大的浓缩能力只能使尿液渗透压浓缩到约700 mOsm/L（比重1.020），在排出l mmol溶质时需带出1.0～2.0ml水；而成人的浓缩能力可使渗透压达到1400 mOsm/L（比重1.035），只需0.7ml水即可排出l mmol溶质，因此小儿在排泄同等量溶质时所需水量较成人为多，尿量相对较多。当入水量不足或失水量增加时，易超过肾脏浓缩能力的限度，发生代谢产物滞留和高渗性脱水。另一方面，正常成人可使尿液稀释到50～l00 mOsm/L（比重1.003），新生儿出生一周后肾脏稀释能力虽可达成人水平，但由于肾小球滤过率低，水的排泄速度较慢，若摄入水量过多又易致水肿和低钠血症。年龄愈小，肾脏排钠、排酸、产氨能力也愈差，因而也容易发生高钠血症和酸中毒。

二、水与电解质平衡失调

（一）脱水

脱水是指水分摄入不足或丢失过多所引起的体液总量尤其是细胞外液量的减少，脱水时除丧失水分外，尚有钠、钾和其他电解质的丢失。体液和电解质的丢失的严重程度取决于丢失的速度及幅度，而丢失体液和电解质的种类反映了水和电解质（主要是钠）的相对丢失率。

1．脱水的程度　脱水的程度常以丢失液体量占体重的百分比来表示，体重的下降常是体液和电解质的丢失而非身体实质部分的减少。因病人常有液体丢失的病史及脱水体征，在临床如病人无近期的体重记录，体重下降的百分比常可通过体检及询问病史估计。一般根据前囟、眼窝的凹陷与否、皮肤弹性、循环情况和尿量等临床表现综合分析判断。常将脱水程度分为三度：

（1）轻度脱水：表示有3～5％体重或相当于30～50ml/kg体液的减少；

（2）中度脱水：表示有5～10％的体重减少或相当于体液丢失50～l00ml/kg；

（3）重度脱水：表示有10％以上的体重减少或相当于体液丢失100～120ml/kg。中度与重度脱水的临床体征常有重叠，有时使估计单位体重的液体丢失难以精确计算。

2．脱水的性质 脱水的性质常常反映了水和电解质的相对丢失量，临床常根据血清钠及血浆渗透压水平对其进行评估。血清电解质与血浆渗透压常相互关联，因为渗透压在很大的程度上取决于血清阳离子，即钠离子。低渗性脱水时血清钠低于130mmol/L；等渗性脱水时血清钠在130～150mmol/L；高渗性脱水时血清钠大于150mmol/L。但在某些情况下，如发生在糖尿病病人存在酮症酸中毒时因血糖过高或在病人应用甘露醇后，血浆渗透压异常增高，此时的高渗性脱水也可发生在血清钠水平低于150mmol/L。临床上以等渗性脱水最为常见，其次为低渗性脱水，高渗性脱水少见。

脱水的不同性质与病理生理、治疗及预后均有密切的关系。详细的病史常能提供估计失水性质与程度的信息，故应详细询问病人的摄入量与排出量、体重变化、排尿次数及频率、一般状况及儿童的性情改变。当患儿有腹泻数天，摄入水量正常而摄入钠盐极少时，常表现为低渗性脱水；当高热数天而摄入水很少时，将配方奶不正确地配成高渗或使用高渗性液体时，可出现高钠血症；当使用利尿剂、有肾脏失盐因素存在而摄入又不足时，可出现低钠血症。但是，当患儿有原发性或继发性肾源性尿崩症而水的摄入受限时，也可能发生高渗性脱水。一般腹泻的大便呈低渗，随着低渗液体的部分口服补充，使最终的脱水呈等渗性。

3．临床表现 在等渗性脱水，细胞内外无渗透压梯度，细胞内容量保持原状，临床表现视脱水的轻重而异，临床表现在很大程度上取决于细胞外容量的丢失量。应注意在严重营养不良儿往往对脱水程度估计过重。眼窝凹陷常被家长发现，其恢复往往是补液后最早改善的体征之

一。

（1）轻度脱水：患儿精神稍差，略有烦躁不安。体检时见皮肤稍干燥，弹性尚可，眼窝和前囟稍凹陷；哭时有泪，口唇黏膜略干，尿量稍减少。

（2）中度脱水：患儿精神萎靡或烦躁不安。皮肤苍白、干燥、弹性较差；眼窝和前囟明显凹陷，哭时泪少，口唇黏膜干燥；四肢稍凉，尿量明显减少。

（3）重度脱水：患儿呈重病容，精神极度萎靡，表情淡漠，昏睡甚至昏迷。皮肤发灰或有花纹、弹性极差；眼窝和前囟深凹陷，眼闭不合，两眼凝视，哭时无泪；口唇黏膜极干燥。因血容量明显减少可出现休克症状，如心音低钝、脉搏细速、血压下降、四肢厥冷、尿极少甚至无尿。

低渗性脱水时，水从细胞外进入细胞内，使循环容量在体外丢失的情况下，因水向细胞内转移更进一步减少，严重者可发生血压下降，进展至休克。由于血压下降，内脏血管发生反射性收缩，肾血流量减少，肾小球滤过率减低，尿量减少，而出现氮质血症。肾小球滤过率降低的另一后果是进入肾小管内的钠离子减少，因而钠几乎全部被重吸收，加之血浆容量缩减引起醛固酮分泌增加，钠的回吸收更为完全，故尿中钠、氯离子极度减少，尿比重降低。若继续补充非电解质溶液，则可产生水中毒、脑水肿等严重后果。由于低渗性脱水时细胞外液的减少程度相对较其他两种脱水明显，故临床表现多较严重。初期可无口渴的症状，除一般脱水现象如皮肤弹性降低、眼窝和前囟凹陷外，多有四肢厥冷、皮肤花斑、血压下降、尿量减少等休克症状。由于循环血量减少和组织缺氧，严重低血钠者可发生脑细胞水肿，因此多有嗜睡等神经系统症状，甚至发生惊厥和昏迷。当伴有酸中毒时常有深大呼吸；伴低血钾时可出现无力、腹胀、肠梗阻或心律失常；当伴有低血钙、低血镁时可出现肌肉抽搐、惊厥和心电图异常等。

在高渗性脱水，水从细胞内转移至细胞外，使细胞内、外的渗透压达到平衡，其结果是细胞内容量降低。而此时因细胞外液得到了细胞内液体的补充，使临床脱水体征并不明显，皮肤常温暖、有揉面感；神经系统可表现为嗜睡，但肌张力较高，反射活跃。由于细胞外液钠浓度过高，渗透压增高，使体内抗利尿激素分泌增多，肾脏回吸收较多的水分，结果使尿量减少。细胞外液渗透压增高后，水由细胞内渗出以调节细胞内、外的渗透压，结果使细胞内液减少。因细胞外液减少并不严重，故循环衰竭和肾小球滤过率减少都较其他两种脱水轻。由于细胞内缺水，患儿常有剧烈口渴、高热、烦躁不安、肌张力增高等表现，甚至发生惊厥。由于脱水后肾脏负担明显增加，既要尽量回吸收水分，同时又要将体内废物排出体外，如果脱水继续加重，最终将出现氮质血症。

（二）钾代谢异常

人体内钾主要存在于细胞内，细胞内钾约为150mmol/L细胞液。正常血清钾维持在3.5～5.0mmol/L，它在调节细胞的各种功能中起重要作用。

1．低钾血症 当血清钾浓度低于3.5mmol/L 时称为低钾血症。

（1）病因：低钾血症在临床较为多见，其发生的主要原因有：①钾的摄入量不足。②由消化道丢失过多；如呕吐、腹泻、各种引流或频繁灌肠而又未及时补充钾。③肾脏排出过多；如酸中毒等所致的钾从细胞内释出，随即大量地由肾脏排出。临床常遇到重症脱水、酸中毒病儿血清钾多在正常范围，缺钾的症状也不明显，当输入不含钾的溶液后，由于血浆被稀释，钾随尿量的增加而排出；酸中毒纠正后钾则向细胞内转移；糖原合成时可消耗钾。由于上述原因，使血清钾下降，并出现低钾症状。此外有肾上腺皮质激素分泌过多如Cushing综合征、原发性醛固酮增多症、糖尿病酮症酸中毒、甲状腺功能亢进、低镁、大量利尿、碳酸酐酶抑制剂的应用

和原发性肾脏失钾性疾病如肾小管性酸中毒等也可引起低钾。④钾在体内分布异常：如在家族性周期性麻痹，病人由于钾由细胞外液迅速地移入细胞内而产生低钾血症。⑤各种原因的碱中毒。

（2）临床表现：低钾血症的临床表现不仅决定于血钾的浓度，而更重要的是缺钾发生的速度。当血清钾下降lmmol/L时，体内总钾下降已达10～30％。此时大多数患儿能耐受；起病缓慢者，体内缺钾虽达到严重的程度，而临床症状不一定很重。一般当血清钾低于3mmol/L时即可出现症状。包括：①神经肌肉：神经肌肉兴奋性降低，表现为骨骼肌、平滑肌及心肌功能的改变，如肌肉软弱无力，重者出现呼吸肌麻痹或麻痹性肠梗阻、胃扩张；膝反射、腹壁反射减弱或消失；②心血管：出现心律失常、心肌收缩力降低、血压降低、甚至发生心力衰竭；心电图表现为T波低宽、出现U波、QT间期延长，T波倒置以及ST段下降等；③肾损害：低血钾使肾脏浓缩功能下降，出现多尿，重者有碱中毒症状；长期低血钾可致肾单位硬化、间质纤维化，在病理上与慢性肾盂肾炎很难区分。此外，慢性低血钾可使生长激素分泌减少。

（3）低钾血症的治疗：低钾的治疗主要为补钾。一般每天可给钾3mmol/kg，严重低钾者可给4～6mmol/kg。补钾常以静脉输入，但如病人情况允许，口服缓慢补钾更安全。应积极治疗原发病，控制钾的进一步丢失。静脉补钾时应精确计算补充的速度与浓度。因细胞对钾的恢复速率有一定的限制，即使在严重低钾病人快速补钾也有潜在危险，包括引起致死性的心律失常。肾功能障碍无尿时影响钾的排出，此时应见尿才能补钾。在补钾时应多次监测血清钾水平，有条件者给予心电监护。一般补钾的输注速度应小于每小时0.3mmol/kg，浓度小于40mmol/L（0.3％）。当低钾伴有碱中毒时，常伴有低血氯，故采用氯化钾液补充可能是最佳策略。

2．高钾血症 血清钾浓度≥5.5mmol/L时称为高钾血症。

（1）病因：①肾功能衰竭、肾小管性酸中毒、肾上腺皮质功能低下等使排钾减少；②休克、重度溶血以及严重挤压伤等使钾分布异常；③由于输入含钾溶液速度过快或浓度过高等。

（2）临床表现：高钾血症的主要表现为：①心电图异常与心律紊乱：高钾血症时心率减慢而不规则，可出现室性早搏和心室颤动，甚至心搏停止。心电图可出现高耸的T波、P波消失或QRS波群增宽，心室颤动及心脏停搏等。心电图的异常与否对决定是否需治疗有很大帮助。②神经、肌肉症状：高钾血症时患儿精神萎靡，嗜睡，手足感觉异常，腱反射减弱或消失，严重者出现弛缓性瘫痪、尿潴留甚至呼吸麻痹。

（3）治疗：高血钾时，所有的含钾补液及口服补钾必须终止，其它隐性的钾来源，如抗生素、肠道外营养等也应注意。高血钾的治疗包括：快速静脉应用碳酸氢钠1～3mmol/kg，或葡萄糖加胰岛素（葡萄糖0.5～1.0g /kg，每3g葡萄糖加1单位胰岛素），促使钾进入细胞内，使血清钾降低。沙丁胺醇（Salbutamol）5μg /kg，经15分钟静脉应用，或以2.5～5mg雾化吸入常能有效地降低血钾，并能持续2～4小时。10％葡萄糖酸钙0.5ml/kg在数分钟内缓慢静脉应用，可对抗高钾的心脏毒性作用，但同时必须监测心电图。上述方法都只是短暂的措施，体内总钾并未显著减少，如采用离子交换树脂、血液或腹膜透析则较有效。此外，对于假性醛固酮增多症引起的高血钾，应用氢氯噻嗪常有效。

（三）酸碱平衡紊乱

正常儿童血pH值与成人一样，均为7.4，但其范围稍宽，即7.35～7.45。人体调节pH值在较稳定的水平取决于两个机理：①理化或缓冲机制：作为保护过多的酸或碱丢失；②生理机制：主要为肾脏和肺直接作用于缓冲机制，使其非常有效地发挥作用。血液及其它体液的缓冲系统主要包括两个方面：碳酸/碳酸氢盐系统和非碳酸氢盐系统。在血液非碳酸氢盐系统，主要为血

红蛋白、有机及无机磷，血浆蛋白占较少部分。在间质液几乎无非碳酸氢盐缓冲系统。在细胞内液，碳酸/碳酸氢盐及非碳酸盐缓冲系统均起作用，后者主要由有机磷蛋白及其它成分组成。

酸碱平衡是指正常体液保持一定的H^+浓度。机体在代谢过程中不断产生酸性和碱性物质，必须通过体内缓冲系统以及肺、肾的调节作用使体液pH维持在7.40（7.35～7.45），以保证机体的正常代谢和生理功能。细胞外液的pH主要取决于血液中最重要的一对缓冲物质，即HCO_3^-和H_2CO_3两者含量的比值。正常HCO_3^-和H_2CO_3比值保持在20/1。当某种因素促使两者比值发生改变或体内代偿功能不全时，体液pH值即发生改变，超出7.35～7.45的正常范围，出现酸碱平衡紊乱。肺通过排出或保留CO_2来调节血液中碳酸的浓度，肾负责排酸保钠。肺的调节作用较肾为快，但两者的功能均有一定限度。当肺呼吸功能障碍使CO_2排出过少或过多、使血浆中H_2CO_3的量增加或减少所引起的酸碱平衡紊乱，称为呼吸性酸中毒或碱中毒。若因代谢紊乱使血浆中H_2CO_3的量增加或减少而引起的酸碱平衡紊乱，则称为代谢性酸中毒或碱中毒。出现酸碱平衡紊乱后，机体可通过肺、肾调节使$[HCO_3^-]/[H_2CO_3]$的比值维持在20/1，即pH维持在正常范围内，称为代偿性代谢性（或呼吸性）酸中毒（或碱中毒）；如果$[HCO_3^-]/[H_2CO_3]$的比值不能维持在20/1，即pH低于或高于正常范围，则称为失代偿性代谢性（或呼吸性）酸中毒（或碱中毒）。常见的酸碱失衡为单纯型（呼吸性酸中毒、呼吸性碱中毒、代谢性酸中毒、代谢性碱中毒）；有时亦出现混合型。

1．代谢性酸中毒 所有代谢性酸中毒都有下列两种可能之一：①细胞外液酸的产生过多；②细胞外液碳酸氢盐的丢失。前者常见有酮症酸中毒，肾衰竭时磷酸、硫酸及组织低氧时产生的乳酸增多。后者代谢酸中毒是由于碳酸氢盐从肾脏或小肠液的丢失，常发生于腹泻、小肠瘘管的引流等。腹泻大便常呈酸性，这是由于小肠液在肠道经细菌发酵作用，产生有机酸，后者与碱性肠液中和，使最终大便仍以酸性为主。在霍乱病人，由于短期内大量肠液产生，大便呈碱性。代谢性酸中毒时主要的缓冲成分是碳酸氢盐，也可通过呼吸代偿使$PaCO_2$降低，但通过呼吸代偿很少能使血液pH值完全达到正常。呼吸代偿只是改善pH的下降（部分代偿），完全代偿取决于肾脏酸化尿液，使血碳酸氢盐水平达到正常，再通过呼吸的重新调节，最终才能使血酸碱平衡达到正常。

代谢性酸中毒的治疗：①积极治疗缺氧、组织低灌注、腹泻等原发疾病；②采用碳酸氢钠或乳酸钠等碱性药物增加碱储备、中和H^+。

一般主张当血气分析的pH值<7.30时用碱性药物。所需补充的碱性溶液mmol数＝剩余柱（BE）负值×0.3×体重（kg），因5％碳酸氢钠lml=0.6mmol，故所需5％碳酸氢钠量（m1）=（-BE）×0.5×体重（kg）。一般将碳酸氢钠稀释成1.4％的溶液输入；先给以计算量的1/2，复查血气后调整剂量。纠酸后钾离子进入细胞内使血清钾降低，游离钙也减少，故应注意补钾、补钙。

2．阴离子间隙（anion gap, AG） 在诊断单纯或混合性酸中毒时，阴离子间隙常有很大的帮助。阴离子间隙是主要测得阳离子与阴离子的差值。测得的阳离子为钠和钾，可测得的阴离子为氯和碳酸氢根。因钾离子浓度相对较低，在计算阴离子间隙时常忽略不计。

阴离子间隙＝$[Na^+]-([Cl^-]+[HCO_3^-])$，正常为12mmol/L（范围：8～16mmol/L）

由于阴离子蛋白、硫酸根和其他常规不测定的阴离子的存在，正常阴离子间隙为12 mmol/L±4mmol/L。AG的增加几乎总是由于代谢性酸中毒所致。但是，不是所有的代谢性酸中毒均有AG增高。AG增高见于代谢性酸中毒伴有常规不测定的阴离子如乳酸、酮体等增加。代谢性酸中毒不伴有常规不测定的阴离子增高时AG不增高，称为高氯性代谢性酸中毒。在高氯性代谢性酸中毒，碳酸氢根的降低被氯离子所替代，而后者可通过血清电解质的测量获得。计算阴离子间隙可发现常规不测定的阴离子或阳离子的异常增高。

当代谢性酸中毒由肾小管酸中毒或大便碳酸氢盐丢失引起时，阴离子间隙可以正常。当血浆碳酸氢根水平降低时，氯离子作为伴随钠在肾小管重吸收的主要阴离子，其吸收率、血浆氯增高，使总阴离子保持不变。

肾功能衰竭时血磷、硫等有机阴离子的增加；糖尿病人的酮症酸中毒、乳酸性酸中毒、高血糖非酮症性昏迷、未定名的有机酸血症、氨代谢障碍等均可使阴离子间隙增加。阴离子间隙增加也见于大量青霉素应用后、水杨酸中毒等。

阴离子间隙降低在临床上较少见。可见于肾病综合征，此时血清白蛋白降低，而白蛋白在pH7.4时属阴离子；多发性骨髓瘤时由于阴离子蛋白的产生增加，也可使阴离子间隙降低。阴离子间隙增加及正常阴离子间隙代谢性酸中毒原因见表4-6。

表4-6　阴离子间隙增加及正常阴离子间隙代谢性酸中毒原因

阴离子间隙增加（AG＞16mmol/L）
慢性肾功能不全
糖尿病酮症酸中毒
静脉营养
遗传性氨基酸尿症
乳酸性酸中毒
中毒：水杨酸等
饥饿
正常阴离子间隙（AG＝8～16mmol/L）
近端、远端肾小管性酸中毒，伴有高钾血症的肾小管性酸中毒
腹泻
碱的摄入

3．代谢性碱中毒　代谢性碱中毒的原发因素是细胞外液强碱或碳酸氢盐的增加。主要原因有：①过度的氢离子的丢失，如呕吐或胃液引流导致的氢和氯的丢失，最常见为先天性肥厚性幽门狭窄；②摄入或输入过多的碳酸氢盐；③由于血钾降低，肾脏碳酸氢盐的重吸收增加，原发性醛固酮增多症、Cushing综合症等；④呼吸性酸中毒时，肾脏代偿性分泌H^+，增加HCO_3^-重吸收，使酸中毒得到代偿，当应用机械通气后，血$PaCO_2$能迅速恢复正常，而血浆[HCO_3^-]含量仍高，导致代谢性碱中毒；⑤细胞外液减少及近端肾小管HCO_3^-的重吸收增加。

代谢性碱中毒时，为减少血pH的变化，会出现一定程度的呼吸抑制，以$PaCO_2$略升高作为代偿，但这种代偿很有限，因为呼吸抑制时可出现低氧症状，后者又能刺激呼吸。通过肾脏排出HCO_3^-使血pH降低，此时常见有碱性尿（pH可达8.5～9）；当临床上同时存在低血钾和低血容量时，除非给予纠正，碱中毒常较难治疗。

代谢性碱中毒无特征性临床表现。轻度代谢性碱中毒可无明显症状，重症者表现为呼吸抑制，精神萎靡。当因碱中毒致游离钙降低时，可引起抽搐；有低血钾时，可出现相应的临床症状。血气分析见血浆pH值增高，$PaCO_2$和[HCO_3^-]增高，常见低血氯和低血钾。典型的病例尿呈碱性，但在严重低钾时尿液pH也可很低。

代谢性碱中毒的治疗包括：①去除病因；②停用碱性药物，纠正水、电解质平衡失调；③

静脉滴注生理盐水；④重症者给以氯化铵静脉滴注；⑤碱中毒时如同时存在的低钠、低钾和低氯血症常阻碍其纠正，故必须在纠正碱中毒时同时纠正这些离子的紊乱。

4．呼吸性酸中毒 呼吸性酸中毒是原发于呼吸系统紊乱，引起肺泡PCO_2增加所致。临床上许多情况可导致血二氧化碳分压增加，包括呼吸系统本身疾病，如肺炎、肺气肿、呼吸道阻塞（如异物、黏稠分泌物、羊水堵塞、喉头痉挛水肿）、支气管哮喘、肺水肿、肺不张、肺萎陷、呼吸窘迫综合征等；胸部疾病所致呼吸受限，如气胸、胸腔积液、创伤和手术等；神经-肌肉疾病，如重症肌无力、急性感染性多发性神经根炎、脊髓灰质炎等；中枢神经系统疾病如头颅损伤、麻醉药中毒以及人工呼吸机使用不当、吸入CO_2过多等。呼吸性酸中毒时通过肾脏代偿使血碳酸氢盐增加，同时伴有肾脏因酸化尿液、氯分泌增加（Cl^-与NH_3^-交换）而致的血氯降低。在血$PaCO_2$<60mmHg时常可通过代偿使pH维持正常。呼吸性酸中毒时常伴有低氧血症及呼吸困难。高碳酸血症可引起血管扩张，颅内血流增加，致头痛及颅内压增高，严重高碳酸血症可出现中枢抑制，血pH降低。

呼吸性酸中毒治疗主要应针对原发病，必要是应用人工辅助通气。

5．呼吸性碱中毒 呼吸性碱中毒是由于肺泡通气过度增加致血二氧化碳分压降低。其原发病因可为心理因素所致的呼吸过度、机械通气时每分通气量太大，也可见于水杨酸中毒所致的呼吸中枢过度刺激、对CO_2的敏感性太高所致的呼吸增加。低氧、贫血、CO中毒时呼吸加快，也可使$PaCO_2$降低出现碱中毒。

呼吸性碱中毒临床主要出现原发疾病所致的相应症状及体征。急性低碳酸血症可使神经肌肉兴奋性增加和因低血钙所致的肢体感觉异常。血气分析见pH值增加、$PaCO_2$降低、血HCO_3^-浓度降低、尿液常呈酸性。

呼吸性碱中毒的治疗主要针对原发病。

6．混合性酸碱平衡紊乱 当有两种或以上的酸碱紊乱分别同时作用于呼吸或代谢系统称为混合性酸碱平衡紊乱。当代偿能力在预计范围之外时，就应考虑存在混合性酸碱平衡紊乱。例如糖尿病酮症酸中毒病人同时存在肺气肿，呼吸窘迫综合征（RDS）病人有呼吸性酸中毒与代谢性酸中毒同时存在时。呼吸系统本身的疾病存在阻碍了以通过降低$PaCO_2$的代偿机制，结果使pH值下降显著。当慢性呼吸性酸中毒伴有充血性心力衰竭时，如过度使用利尿剂可出现代谢性碱中毒，此时血浆[HCO_3^-]水平和pH值将高于单纯的慢性呼吸性酸中毒。肝功能衰竭时可出现代谢性酸中毒与呼吸性碱中毒，此时pH值可能变化不大，但血浆[HCO_3^-]和$PaCO_2$显著降低。

混合性酸碱平衡紊乱的治疗包括：①积极治疗原发病，保持呼吸道通畅，必要时给以人工辅助通气，使pH正常。②对高AG性代谢性酸中毒，以纠正缺氧、控制感染和改善循环为主；经机械通气改善肺氧合功能后，代谢性酸中毒亦可减轻或纠正，仅少数病人需补碱性药物；碱性药物应在保证通气的前提下使用。pH值明显低下时应立即用碱性药物。

7．临床酸碱平衡状态的评估 临床上酸碱平衡状态常通过血pH，$PaCO_2$及[HCO_3^-]三项指标来评估。pH与$PaCO_2$可直接测定，[HCO_3^-]虽能直接测定，但常常用血清总二氧化碳含量，通过算图估计。应该指出的是一般血气分析仪只含测定pH、$PaCO_2$和PaO_2三项指标的电极，[HCO_3^-]是按Henderson-Hasselbalch方程计算的。$PaCO_2$、[HCO_3^-]变化与pH值的关系可从表4-7分析、判断。判断单纯的酸碱平衡紊乱并不困难，pH值的变化取决于$PaCO_2$与[HCO_3^-]的比值变化。在临床判断时，首先应确定是酸中毒还是碱中毒；其次是引起的原发因素是代谢性还是呼吸性；第三，如是代谢性酸中毒，其阴离子间隙是高还是低；第四，分析呼吸或代谢代偿是否充分。

表4-7 酸碱紊乱的分析方法

动脉血气测定			
酸中毒（pH<7.40）		碱中毒（pH>7.40）	
↓$[HCO_3^-]$	↑$PaCO_2$	↑$[HCO_3^-]$	↓$PaCO_2$
代谢性酸中毒	呼吸性酸中毒	代谢性碱中毒	呼吸性碱中毒
↓$PaCO_2$代偿	↑$[HCO_3^-]$代偿	↑$PaCO_2$代偿	↓$[HCO_3^-]$代偿
呼吸代偿	肾脏代偿	呼吸代偿	肾脏代偿
临床举例：酮症酸中毒；乳酸酸中毒；腹泻、肠液丢失；肾小管性酸中毒等	临床举例： 中枢呼吸抑制；神经肌肉疾病；肺实质性疾病等	临床举例：呕吐引起的H^+、Cl^-丢失；外源性HCO_3^-摄入或输入过多等	临床举例：由于精神因素或药物（如水杨酸）中毒所致的呼吸增快
代偿效果：每↓$PaCO_2$ 1.2 mmHg 可代偿 1 mmol/L的$[HCO_3^-]$↓	代偿效果：每↑$[HCO_3^-]$ 3.5mmol/L可代偿10mmHg的$PaCO_2$↑	代偿效果：每↑$PaCO_2$ 0.7mmHg可代偿1mmol/L 的$[HCO_3^-]$↑	代偿效果：每↓$[HCO_3^-]$ 5mmol/L可代偿10mmHg的$PaCO_2$↑

三、液体疗法时常用补液溶液

常用液体包括非电解质和电解质溶液。其中非电解质溶液常用5％或10％葡萄糖液，因葡萄糖输入体内将被氧化成水，故属无张力溶液。电解质溶液包括氯化钠、氯化钾、乳酸钠、碳酸氢钠和氯化铵等以及它们的不同配制液，详见表4-8。

表4-8 常用溶液成分

溶液	每100ml含溶质或液量	Na^+	K^+	Cl^-	HCO_3^-或乳酸根	Na^+/Cl^-	渗透压或相对于血浆的张力
血浆		142	5	103	24	3：2	300mOsm/L
① 0.9%氯化钠	0.9g	154		154		1：1	等张
② 5%或10%葡萄糖	5g或10g						
③ 5%碳酸氢钠	5g	595			595		3.5张
④ 1.4%碳酸氢钠	1.4g	167			167		等张
⑤ 11.2%乳酸钠	11.2g	1000			1000		6张
⑥ 1.87%乳酸钠	1.87g	167			167		等张
⑦ 10%氯化钾	10g		1342	1342			8.9张
⑧ 0.9%氯化铵	0.9g	NH^+167		167			等张
1：1含钠液	①50ml,②50ml	77		77			1/2张
1：2含钠液	①35ml,②65ml	54		54			1/3张
1：4含钠液	①20ml,②80ml	30		30			1/5张
2：1含钠液	①65ml,④或⑥35ml	158		100	58	3：2	等张
2：3：1含钠液	①33ml，②50ml ④或⑥17ml	79		51	28	3：2	1/2张
4：3：2含钠液	①45ml，②33ml ④或⑥22ml	106		69	37	3：2	2/3张

【附】口服补液盐（oral rehydration salts，ORS）

ORS是世界卫生组织（WHO）推荐用以治疗急性腹泻合并脱水的一种溶液，经临床应用取得了良好效果，对发展中国家尤其适用。其理论基础是基于小肠的Na^+-葡萄糖偶联转运吸收机

制，即小肠上皮细胞刷状缘的膜上存在着Na^{+}-葡萄糖共同载体，此载体上有Na^{+}和葡萄糖两个结合位点，当Na^{+}-葡萄糖同时与结合位点相结合时即能运转、并显著增加钠和水的吸收。

目前有多种ORS配方。世界卫生组织（WHO）2002年推荐的低渗透压口服补液盐配方与传统的配方比较同样有效，但更为安全。该配方中各种电解质浓度为$[Na^{+}]$ 75 mmol/L，$[K^{+}]$ 20 mmol/L，$[Cl^{-}]$ 65 mmol/L,，枸橼酸根10 mmol/L，葡萄糖75 mmol/L。可用NaCl 2.6g、枸橼酸2.9 g、氯化钾1.5g、葡萄糖13.5g，加水到1000ml配成。总渗透压为245mOsm/L。ORS一般适用于轻度或中度脱水无严重呕吐者，在用于补充继续损失量和生理需要量时需适当稀释。

四、液体疗法

液体疗法是儿科医学的重要组成部分，其目的是维持或恢复正常的体液容量和成分，以保持正常的生理功能。液体疗法包括了补充生理需要量、累积损失量及继续丢失量。上述每一部分都可独立地进行计算和补充。例如，对于空腹将接受外科手术的儿童，可能只需补充生理需要量和相应的电解质；而对于腹泻病人则需补充生理需要液、累积损失量和继续丢失量。由于体液失衡的原因和性质非常复杂，在制定补液方案时必须全面掌握病史、体格检查和实验室检查资料及患儿的个体差异，分析三部分液体的不同需求，制定合理、正确的输液量、速度、成分及顺序。一般情况下，肾脏、肺、心血管及内分泌系统对体内液体平衡有较强的调节作用，故补液成分及量如基本合适，机体就能充分调整，以恢复体液的正常平衡；但如上述脏器存在功能不全，则应较严格地选择液体的成分，根据其病理生理特点选择补液量及速度，并根据病情变化而调整。

1．生理需要量　生理需要量涉及热量、水和电解质。维持液量和电解质直接与代谢率相关，代谢率的变化可通过糖类、脂肪和蛋白质氧化影响内生水的产生。肾脏的溶质排出可影响水的排出。由于25％的水是通过不显性失水丢失的，能量的产生必然会影响到水的丢失，故正常生理需要量的估计可按能量需求计算，一般按每代谢100kcal能量需100～150ml水；年龄越小需水相对越多，故也可按简易计算表计算（见表4-9）。

表4-9　生理需要量简易计算

体重	每天需要量（ml）
～10kg	100ml/kg
11～20kg	1000+超过10 kg体重数×50ml/kg
>20kg	1500+超过20 kg体重数×20ml/kg

生理需要量的需求取决于尿量、大便丢失及不显性失水。大便丢失常可忽略不计，不显性失水占液体丢失的约1/3，在发热时增加（体温每增加1℃，不显性失水增加12％），肺不显性失水在过度通气，如哮喘、酮症酸中毒时增加，在有湿化功能的人工呼吸机应用时肺不显性失水降低。在极低体重儿，不显性失水可多达每天100ml/kg以上。

电解质的需求包括每日出汗、正常大小便、生理消耗的电解质等，变化很大。平均钾、钠、氯的消耗量约2～3mmol/100kcal。生理需要量应尽可能口服补充，不能口服或不足者可以静脉滴注1/4～1/5张含钠液，同时给予生理需要量的钾。发热、呼吸加快的患儿应适当增加进液量；营养不良者应注意能量和蛋白质补充；必要时用部分或全静脉营养。

2．补充累积损失量　根据脱水程度及性质补充：即轻度脱水约30～50ml/kg（体重）；中度为50～100ml/kg；重度为100～120ml/kg。通常对低渗性脱水补2/3张含钠液；等渗性脱水补1/2

张含钠液；高渗性脱水补1/3～1/5张含钠液，如临床上判断脱水性质有困难，可先按等渗性脱水处理。补液的速度取决于脱水程度，原则上应先快后慢。对伴有循环不良和休克的重度脱水患儿，开始应快速输入等渗含钠液（生理盐水或2：1 液）按20ml/kg于30分钟～1小时输入。其余累积损失量补充常在8～12h内完成。在循环改善出现排尿后应及时补钾。酸碱平衡紊乱及其它电解质异常的纠正见本节（酸碱平衡紊乱）。对于高渗性脱水，需缓慢纠正高钠血症（每24小时血钠下降<10mmol/L），也可在数天内纠正。有时需用张力较高甚至等张液体，以防血钠迅速下降出现脑水肿。

3．补充继续丢失量　在开始补充累积损失量后，腹泻、呕吐、胃肠引流等损失大多继续存在，以致体液继续丢失，如不予以补充将又成为新的累积损失。此种丢失量依原发病而异，且每日可有变化，对此必须进行评估，根据实际损失量用类似的溶液补充。各种体液丢失的性质见表4-10。

表4-10　各种体液损失成分表

体液	Na^+（mmol/L）	K^+（mmol/L）	Cl^-（mmol/L）	蛋白质（g/dl）
胃液	20～80	5～20	100～150	—
胰液	120～140	5～15	90～120	—
小肠液	100～140	5～15	90～130	—
胆汁液	120～140	5～15	50～120	—
回肠造瘘口损失液	45～135	5～15	20～115	—
腹泻液	10～90	10～80	10～110	—
正常出汗	10～30	3～10	10～25	—
烫伤	140	2	110	3～5

（杜立中）

第五章 营养和营养障碍疾病

第一节 儿童营养基础

一、营养素与膳食营养素参考摄入量

营养（nutrition）是指人体获得和利用食物维持生命活动的整个过程。食物中经过消化、吸收和代谢能够维持生命活动的物质称为营养素（nutrients）。膳食营养素参考摄入量（dietary reference intakes，DRIs）包括4项内容：平均需要量（estimated average requirement，EAR）是某一特定性别、年龄及生理状况群体中对某营养素需要量的平均值，摄入量达到EAR水平时可以满足群体中50%个体对该营养素的需要；对个体可以满足自身50%需要，缺乏的可能性为50%。推荐摄入量（recommended nutrient intake，RNI）可以满足某一特定性别、年龄及生理状况群体中绝大多数（97％～98％）人体的需要；适宜摄入量（adequate intake，AI）是通过观察或实验获得的健康人群某种营养素的摄入量，可能高于RNI，不如RNI精确；可耐受最高摄入量（tolerable upper intake level，UL）是平均每日可以摄入该营养素的最高量。当摄入量超过UL时，发生毒副作用的危险性增加。

营养素分为：能量；宏量营养素（蛋白质、脂类、碳水化合物）；微量营养素（矿物质，包括常量元素和微量元素；维生素）；其他膳食成分（膳食纤维、水）。

儿童由于生长发育快对营养需求高，而自身消化吸收功能尚不完善，正确的膳食行为有待建立，处理好这些矛盾对儿童健康成长十分重要。

（一）儿童能量代谢

人体能量代谢的最佳状态是达到能量消耗与能量的摄入的平衡，能量缺乏和过剩都对身体健康不利。儿童总能量消耗量包括基础代谢率、食物的热力作用、生长、活动和排泄5个方面。能量单位是千卡（kcal），或以千焦耳（kJ）为单位，lkcal＝4.184kJ，或1kJ＝0.239kcal。

1．基础代谢率（BMR） 小儿基础代谢的能量需要量较成人高，随年龄增长逐渐减少。如婴儿的BMR约为55kcal（230.12kJ）/（kg.d），7岁时BMR为44kcal（184.10kJ）/（kg.d），12岁时每日约需30kcal（125.52kJ）/（kg.d），成人时为25kcal（104.6kJ）～30kcal（125.52KJ）/（kg.d）。

2.食物热力作用（thermic effect of food，TEF） 是指由于进餐后几小时内发生的超过BMR的能量消耗，主要用于体内营养素的代谢。与食物成分有关：糖类食物的食物热力作用为本身产生能量的6%，脂肪为4%，蛋白质为30%。婴儿食物含蛋白质多，食物热力作用占总能量的7%～8%，年长儿的膳食为混合食物，其食物热力作用为5％。

3．活动消耗（physical activity） 儿童活动所需能量与身体大小、活动强度、活动持续时间、活动类型有关。故活动所需能量个体波动较大，并随年龄增加而增加。当能量摄入不足时，儿童首先表现活动减少。

4．排泄消耗（excreta） 正常情况下未经消化吸收的食物的损失约占总能量的10％，腹泻时增加。

5．生长所需（growth） 组织生长合成消耗能量为儿童特有，生长所需能量与儿童生长的速度成正比，即随年龄增长逐渐减少。

一般认为基础代谢占能量的50%，排泄消耗占能量的10%，生长和运动所需能量占32%～35%，食物的TEF占7%～8%（图5-1）。婴儿能量RNI为95kcal（397.48kJ）/（kg.d），1岁后以每岁计算。

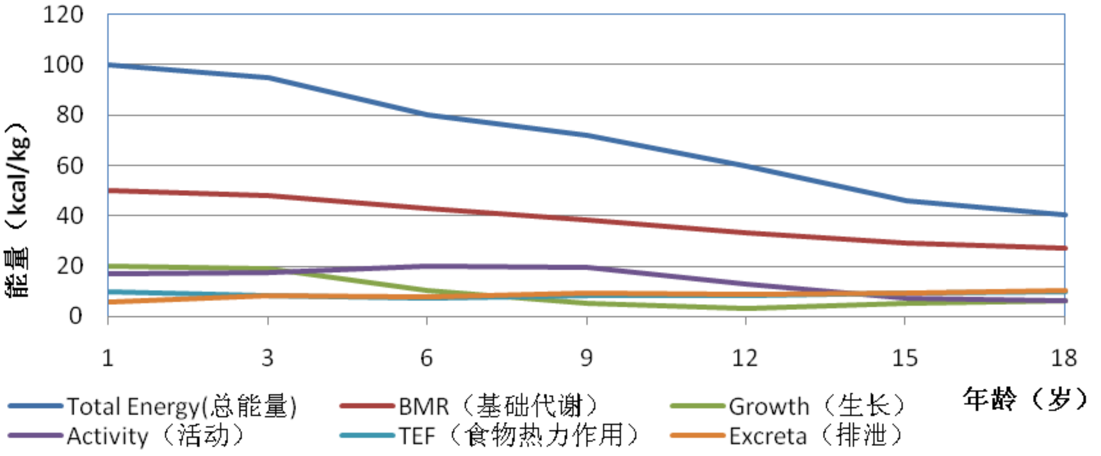

图5-1 能量分布与年龄的关系

（二）宏量营养素

1．糖类 为供能的主要来源。常用可提供能量的百分比来表示糖类的适宜摄入量。2 岁以上儿童膳食中，糖类所产的能量应占总能量的55%～65%。保证充分糖类摄入，提供合适比例的能量来源是重要的，如糖类产能>80%或<40%都不利于健康。糖类主要来源于粮谷类和薯类食物。

2．脂类 为脂肪（甘油三酯）和类脂，是机体的第二供能营养素。人体不能合成、必须由食物供给的脂肪酸称为必需脂肪酸，如亚油酸（$C_{18:2}$）、亚麻酸（$C_{18:3}$，）。亚油酸是n-6系的脂肪酸，可衍生多种n-6不饱和脂肪酸，如花生四烯酸。亚油酸在体内可转变成亚麻酸和花生四烯酸，故亚油酸是最重要的必需脂肪酸。α-亚麻酸（n-3）也属必需脂肪酸，可衍生多种n-3不饱和脂肪酸，包括二十碳五烯酸（EPA，$C_{20:5}$）和二十二碳六烯酸（DHA，$C_{22:6}$）。这些必需脂肪酸对细胞膜功能、基因表达、防治心脑血管疾病和生长发育都有重要作用。n-3多不饱和脂肪酸对脑、视网膜、皮肤和肾功能的健全十分重要。

必需脂肪酸来源：主要来源于植物，亚油酸主要存在于植物油、坚果类（核桃、花生）；亚麻酸主要存在于绿叶蔬菜、鱼类脂肪及坚果类。母乳含有丰富的必需脂肪酸。

脂肪供能占总能量的百分比（AI）：6个月以下占婴儿总能量的45%～50%，6个月～2岁以下为35%～40%,2～7岁以下为30%～35%，7岁以上为25%～30%（附录二）。膳食亚油酸占膳食能量的3%～5%，亚麻酸占膳食能量的0.5%～1%。EPA、DHA占总能量的0.5%。

3．蛋白质 除需要有与成人相同的8种必需氨基酸外，组氨酸是婴儿所需的必需氨基酸；胱氨酸、酪氨酸、精氨酸、牛磺酸对早产儿可能也必需。蛋白质氨基酸的模式与人体蛋白质氨基酸模式接近的食物，生物利用率就高，称为优质蛋白质。优质蛋白质主要来源于动物和大豆蛋白质。

蛋白质主要功能是构成机体组织和器官的重要成分，次要功能是供能，占总能量的8%～15%。1岁内婴儿蛋白质的RNI为1.5～3g/（kg.d）。婴幼儿生长旺盛，保证优质蛋白质供给非常重要，优质蛋白质应占50%以上。食物的合理氨基酸及加工可达到蛋白质互补，提高食物的生物价值。例如小麦、米、玉米等赖氨酸含量低，蛋氨酸含量高，而豆类则相反，如两者搭配可

互相弥补不足。如豆制品的制作可使蛋白质与纤维素分开，利于消化

为满足儿童生长发育的需要，应首先保证能量供给，其次是蛋白质。宏量营养素应供给平衡，比例适当，否则易发生代谢紊乱。如儿童能量摄入不足，机体会动用自身的能量储备甚至消耗组织以满足生命活动能量的需要。相反，如能量摄入过剩，则能量在体内的储备增加，造成异常的脂肪堆积，与成年期慢性疾病和代谢综合征有关，是当前要特别重视的问题。

（三）微量营养素

1．矿物质

（1）常量元素：在矿物质中，人体含量大于体重的0.01%的各种元素称为常量元素，如钙、钠、磷、钾等。常量元素中钙的问题最多，婴儿期钙的沉积高于生命的任何时期，2岁以下每日钙在骨骼增加约200mg，非常重要。乳类是钙的最好来源，大豆是钙的较好来源。钙的AI：母乳喂养婴儿为300mg，牛乳喂养婴儿为500mg，幼儿为600mg，4岁及以上为800mg，钙摄入过量可能造成一定危害，需特别注意钙的补充控制在UL（2g/d）以下。

（2）微量元素：在体内含量很低，含量绝大多数小于人体重的0.01%，需通过食物摄入具有十分重要的生理功能，如碘、锌、硒、铜、钼、铬、钴、铁、镁等，其中铁、碘、锌缺乏症是全球最主要的微量营养素缺乏病。必需微量元素是酶、维生素必需的活性因子；构成或参与激素的作用；参与核酸代谢。

2．维生素　维生素是维持人体正常生理功能所必需的一类有机物质，在体内含量极微，但在机体的代谢、生长发育等过程中起重要作用。一般不能在体内合成（维生素D、部分B属及K例外）或合成量太少，必须由食物供给。分为脂溶性和水溶性两大类。对儿童来说维生素A、D、C、B_1是容易缺乏的维生素。

常见维生素和矿物质的作用及来源见表5-1。常见维生素和矿物质的每日推荐摄入量见附录二。

表5-1　常见维生素和矿物质的作用及来源

种类	作用	来源
维生素A	促进生长发育和维持上皮组织的完整性，为形成视紫质所必需的成分，与铁代谢、免疫功能有关	肝、牛乳、奶油、鱼肝油；有色蔬菜中的胡萝卜素
维生素B_1（硫胺素）	是构成脱羧辅酶的主要成分，为糖类代谢所必需，维持神经、心肌活动功能，调节胃肠蠕动，促进生长发育	米糠、麦麸、豆、花生；瘦肉、内脏；肠内细菌和酵母可合成一部分
维生素B_2（核黄素）	为辅黄酶主要成分，参与体内氧化过程	肝、蛋、鱼、乳类、蔬菜、酵母
维生素PP（烟酸、尼克酸）	是辅酶Ⅰ及Ⅱ的组成成分，为体内氧化过程所必需；维持皮肤、黏膜和神经的健康，防止癞皮病，促进消化系统的功能	肝、肉、谷类、花生、酵母
维生素B_6	为转氨酶和氨基酸脱羧酶的组成成分，参与神经、氨基酸及脂肪代谢	各种食物中，亦由肠内细菌合成一部分
维生素B_{12}	参与核酸的合成、促进四氢叶酸的形成等，促进细胞及细胞核的成熟，对生血和神经组织的代谢有重要作用	动物性食物
叶酸	叶酸的活性形式四氢叶酸是体内转移“一碳基团”的辅酶，参与核苷酸的合成，特别是胸腺嘧啶核苷酸的合成，有生血作用；胎儿期缺乏引起神经管畸形	绿叶蔬菜、肝、肾、酵母较丰富，肉、鱼、乳类次之，羊乳中含量甚少
维生素C	参与人体的羟化和还原过程，对胶原蛋白、细胞间黏合质、神经递质（如去甲肾上腺素等）的合成，类固醇的羟化，氨	各种水果及新鲜蔬菜

种类	作用	来源
	基酸代谢，抗体及红细胞的生成等均有重要作用	
维生素D	调节钙磷代谢，促进肠道对钙的吸收，维持血液钙浓度，有利骨骼矿化	鱼肝油、肝、蛋黄；人皮肤日光照射合成
维生素K	由肝脏利用、合成凝血酶原	肝、蛋、豆类、青菜、部分维生素K由肠内细菌合成
钙	为凝血因子，能降低神经、肌肉的兴奋性，是构成骨骼、牙齿的主要成分	乳类、豆类、绿色蔬菜
磷	是骨骼、牙齿、细胞核蛋白、各种酶的主要成分，协助糖、脂肪和蛋白质的代谢，参与缓冲系统，维持酸碱平衡	乳类、肉类、豆类和五谷类
铁	是血红蛋白、肌红蛋白、细胞色素和其他酶系统的主要成分，帮助氧的运输	肝、血、豆类、肉类、绿色蔬菜、杏、桃
锌	为多种酶的成分	鱼、蛋、肉、禽、全谷、麦胚、豆、酵母等
镁	构成骨骼和牙齿成分，激活糖代谢酶，与肌肉神经兴奋性有关，为细胞内阳离子，参与细胞代谢过程	谷类、豆类、干果、肉、乳类
碘	为甲状腺素主要成分	海产品中含量丰富

（四）其他膳食成分

1．膳食纤维　膳食纤维主要来自植物的细胞壁，为不被小肠酶消化的非淀粉多糖。功能：吸收大肠水分，软化大便，增加大便体积，促进肠蠕动等功能。膳食纤维在大肠被细菌分解，产生短链脂肪酸，降解胆固醇，改善肝代谢，防止肠萎缩。婴幼儿可从谷类、新鲜蔬菜、水果中获得一定量的膳食纤维。

2．水　儿童水的需要量与能量摄入、食物种类、肾功能成熟度、年龄等因素有关。婴儿新陈代谢旺盛，水的需要量相对较多，为150ml/（kg.d），以后每3岁减少约25ml/（kg.d）。

二、小儿消化系统功能发育与营养关系

儿科医生掌握与了解小儿消化系统解剖发育知识非常重要，如吸吮、吞咽的机理、食管运动、肠道运动发育、消化酶的发育水平等，可正确指导家长喂养婴儿，包括喂养的方法、食物的量以及比例等。

（一）消化酶的成熟与宏量营养素的消化、吸收

1．蛋白质　出生时新生儿消化蛋白质能力较好。胃蛋白酶可凝结乳类，出生时活性低，3个月后活性增加，18个月时达成人水平。生后1周胰蛋白酶活性增加，1个月时已达成人水平。

生后几个月小肠上皮细胞渗透性高，有利于母乳中的免疫球蛋白吸收，但也会增加异体蛋白（如牛奶蛋白、鸡蛋白蛋白）、毒素、微生物以及未完全分解的代谢产物吸收机会，产生过敏或肠道感染。因此，对婴儿，特别是新生儿，食物的蛋白质应有一定限制。

2．脂肪　新生儿胃脂肪酶发育较好；而胰脂酶几乎无法测定，2～3岁后达成人水平。母乳的脂肪酶可补偿胰脂酶的不足。故婴儿吸收脂肪和能力随年龄增加而提高，28～34周的早产儿脂肪的吸收率为65%～75%；足月儿脂肪的吸收率为90%；生后6个月婴儿脂肪的吸收率达95%以上。

3．糖类　0～6个月婴儿食物中的糖类主要是乳糖，其次为蔗糖和少量淀粉。肠双糖酶发育好，消化乳糖好。胰淀粉酶发育较差，3个月后活性逐渐增高，2岁达成人水平，故婴儿生后

几个月消化淀粉能力较差，不宜过早添加淀粉类食物。

（二）与进食技能有关的消化道发育

1．　食物接受的模式发展　婴儿除受先天的甜、酸、苦等基本味觉反射约束外，通过后天学习形成味觉感知。味觉感知是食物营养价值的提示，对食物接受的模式发展具有重要作用。婴儿对能量密度较高的食物和感官好的食物易接受，一旦对能量味觉的指示被开启后再调节摄入是很困难的，这可能是肥胖发生的原因之一。儿童对食物接受的模式源于对多种食物刺激的经验和后天食物经历对基础味觉反应的修饰，这说明学习和经历对儿童饮食行为建立具有重要意义。

2．挤压反射　新生儿至3～4月婴儿对固体食物出现舌体抬高、舌向前吐出的挤压反射。婴儿最初的这种对固体食物的抵抗可被认为是一种保护性反射，其生理意义是防止吞入固体食物到气管发生窒息，在转乳期用勺添加新的泥状食物时注意尝试8～10次才能成功。

3．咀嚼　咀嚼和吞咽是先天就会的生理功能，咀嚼功能发育需要适时的生理刺激，需要后天学习训练。换奶期及时添加泥状食物是促进咀嚼功能发育的适宜刺激，咀嚼发育完善对语言的发育也有直接影响。后天咀嚼行为的学习敏感期在4～6个月。有意训练7个月左右婴儿咬嚼指状食物、从杯中咂水，9个月始学用勺自喂，1岁学用杯喝奶，均有利于儿童口腔发育成熟。

第二节 婴儿喂养方法

一、母乳喂养

(一)人乳的特点

人乳是满足婴儿生理和心理发育的天然最好食物，对婴儿的健康生长发育有不可替代作用。一个健康的母亲可提供足月儿正常生长到6个月所需要的营养素、能量、液体量。哺乳不仅供给婴儿营养，同时还提供一些可供婴儿利用的现成物质，如脂肪酶、SIgA等，直到婴儿体内可自己合成。

1．营养丰富　人乳营养生物效价高，易被婴儿利用。人乳含必需氨基酸比例适宜；人乳所含酪蛋白的为β-酪蛋白，含磷少，凝块小；人乳所含白蛋白为乳清蛋白，促乳糖蛋白形成；人乳中酪蛋白与乳清蛋白的比例为1：4，与牛乳（4：1）有明显差别，易被消化吸收。人乳中宏量营养素产能比例适宜（表5-2）。人乳喂养的婴儿很少产生过敏。

表5-2　人乳与牛乳宏量营养素产能比（100ml）

	人乳	牛乳	理想标准
糖　类	41%（6.9g）	29%（5.0g）	40%～50%
脂　肪	50%（3.7g）	52%（4.0g）	50%
蛋白质	9%（1.5g）	19%（3.3g）	11%
能　量	67kcal	69kcal	

人乳中乙型乳糖（β-双糖）含量丰富，利于脑发育；利于双歧杆菌、乳酸杆菌生长，并产生B族维生素；利于促进肠蠕动；乳糖在小肠远端与钙形成螯合物，降低钠在钙吸收时的抑制作用，避免了钙在肠腔内沉淀，同时乳酸使肠腔内pH下降，有利小肠钙的吸收。

人乳含不饱和脂肪酸较多，初乳中更高，有利于脑发育。人乳的脂肪酶使脂肪颗粒易于消化吸收。

人乳中电解质浓度低、蛋白质分子小，适宜婴儿不成熟的肾发育水平。人乳矿物质易被婴儿吸收，如人乳中钙、磷比例适当（2：1），含乳糖多，钙吸收好；人乳中含低分子量的锌结合因子-配体，易吸收，锌利用率高；人乳中铁含量为0.05mg/dl与牛奶（0.05mg/dl）相似但人乳中铁吸收率（49%）高于牛奶（4%）。

人乳中维生素D含量较低，母乳喂养的婴儿应补充维生素D，并鼓励家长让婴儿生后尽早户外活动，促进维生素D皮肤的光照合成；人乳中维生素K含量亦较低，除鼓励乳母合理膳食多吃蔬菜、水果以外，乳母应适当补充维生素K，以提高乳汁中维生素K的含量。

2．生物作用

（1）缓冲力小：人乳pH为3.6（牛奶pH 5.3），对酸碱的缓冲力小，不影响胃液酸度（胃酸pH0.9～1.6），利于酶发挥作用。

（2）含不可替代的免疫成分（营养性被动免疫）：初乳含丰富的SIgA，早产儿母亲乳汁的SIgA高于足月儿。人乳中的SIgA在胃中稳定，不被消化，可在肠道发挥作用。SIgA黏附于肠黏膜上皮细胞表面，封闭病原体，阻止病原体吸附于肠道表面，使其繁殖受抑制，保护消化道黏膜，抗多种病毒、细菌。

人乳中含有大量免疫活性细胞，初乳中更多，其中85%～90%为巨噬细胞，10%～15%为淋巴细胞；免疫活性细胞释放多种细胞因子而发挥免疫调节作用。人乳中的催乳素也是一种有免疫调节作用的活性物质，可促进新生儿免疫功能的成熟。

人乳含较多乳铁蛋白，初乳含量更丰富（可达1741mg/L），是人乳中重要的非特异性防御因子。人乳的乳铁蛋白对铁有强大的螯合能力，能夺走大肠杆菌、大多数需氧菌和白色念珠菌赖以生长的铁，从而抑制细菌的生长。

人乳中的溶菌酶能水解革兰阳性细菌胞壁中的乙酰基多糖，使之破坏并增强抗体的杀菌效能。人乳的补体及双歧因子含量也远远多于牛乳。双歧因子促乳酸杆菌生长，使肠道pH达4～5，抑制大肠杆菌、痢疾杆菌、酵母菌等生长。

低聚糖是人乳所特有的。人乳中低聚糖与肠粘膜上皮细胞的细胞黏附抗体的结构相似，可阻止细菌黏附于肠粘膜；促使乳酸杆菌及双歧杆菌的生长。

（3）生长调节因子：为一组对细胞增殖、发育有重要作用的因子，如牛磺酸、激素样蛋白（上皮生长因子、神经生长因子），以及某些酶和干扰素。

3．其他　母乳喂养还有经济（仅1/5人工喂养费用）、方便、温度适宜、有利于婴儿心理健康的优点。母亲哺乳可加快乳母产后子宫复原，减少再受孕的机会。

（二）人乳的成分变化

1．各期人乳成分　初乳为孕后期与分娩4～5日以内的乳汁；5～14日为过渡乳；14日以后的乳汁为成熟乳。人乳中的脂肪、水溶性维生素、维生素A、铁等营养素与乳母饮食有关，而维生素D、E、K不易由血进入乳汁，故与乳母饮食成分关系不大（表5-3）。

表5-3　各期人乳成分（g/L）

	初乳	过渡乳	成熟乳
蛋白质	22.5	15.6	11.5
脂肪	28.5	43.7	32.6
糖类	75.9	77.4	75.0
矿物质	3.08	2.41	2.06
钙	0.33	0.29	0.35
磷	0.18	0.18	0.15

初乳量少，淡黄色，碱性，比重1.040～1.060（成熟乳1.030），每日量约15ml～45ml；初乳含脂肪较少而蛋白质较多（主要为免疫球蛋白）；初乳中维生素A、牛磺酸和矿物质的含量颇丰富，并含有初乳小球（充满脂肪颗粒的巨噬细胞及其他免疫活性细胞），对新生儿的生长发育和抗感染能力十分重要。随哺乳时间的延长，蛋白质与矿物质含量逐渐减少。各期乳汁中乳糖的含量较恒定。

2．哺乳过程的乳汁成分变化　每次哺乳过程乳汁的成分亦随时间而变化。如将哺乳过程分为三部分，即第一部分分泌的乳汁脂肪低而蛋白质高，第二部分乳汁脂肪含量逐渐增加而蛋白质含量逐渐降低，第三部分乳汁中脂肪含量最高（表5-4）。

表5-4　各部分乳汁成分变化（g/L）

	I	II	III
蛋白质	11.8	9.4	7.1
脂肪	17.1	27.7	55.1

3．乳量　正常乳母平均每天泌乳量随时间而逐渐增加，成熟乳量可达700～1000ml。一般产后6 个月后乳母泌乳量与乳汁的营养成分逐渐下降。判断奶量是否充足是以婴儿体重增长情况、尿量多少与睡眠状况等综合判断。劝告母亲不要轻易放弃哺乳。

（三）建立良好的母乳喂养

成功的母乳喂养应当是母子双方都积极参与并感到满足。当母亲喂养能力提高，婴儿的摄乳量也将提高。因此，建立良好的母乳喂养有三个条件，一是孕母能分泌充足的乳汁；二是哺乳时出现有效的射乳反射；三是婴儿有力的吸吮。世界卫生组织（WHO）和我国卫生部制定的《婴幼儿喂养策略》建议生生6个月内完成接受母乳喂养。

1．产前准备　大多数健康的孕妇都具有哺乳的能力，但真正成功的哺乳则需孕妇身、心两方面的准备和积极的措施。保证孕母合理营养，孕期体重增加适当（12～14kg），母体可贮存足够脂肪，供哺乳能量的消耗。

2．乳头保健　孕母在妊娠后期每日用清水（忌用肥皂或酒精之类）擦洗乳头；乳头内陷者用两手拇指从不同的角度按捺乳头两侧并向周围牵拉，每日1至数次；哺乳后可挤出少许乳汁均匀地涂在乳头上，乳汁中丰富的蛋白质和抑菌物质对乳头表皮有保护作用。这些方法可防止因出现乳头皲裂及乳头内陷而中止哺乳。

3．尽早开奶、按需哺乳　吸吮对乳头的刺激可反射性地促进泌乳。0～2月的小婴儿每日多次、按需哺乳，使吸吮有力，乳头得到多次刺激，乳汁分泌增加。有力的吸吮是促进乳汁分泌的重要因素，使催乳素在血中维持较高的浓度，产后2周乳晕的传入神经特别敏感，诱导缩

宫素分泌的条件反射易于建立，是建立母乳喂养的关键时期。吸吮是主要的条件刺激，应尽早开奶（产后15分钟～2小时内）。尽早开奶可减轻婴儿生理性黄疸，同时还可减轻生理性体重下降、低血糖的发生。

4．促进乳房分泌　吸乳前让母亲先湿热敷乳房，促进乳房循环流量。2～3分钟后，从外侧边缘向乳晕方向轻拍或按摩乳房，促进乳房感觉神经的传导和泌乳。两侧乳房应先后交替进行哺乳。若一侧乳房奶量已能满足婴儿需要，则可每次轮流哺喂一侧乳房，并将另一侧的乳汁用吸奶器吸出。每次哺乳应让乳汁排空。

5．正确的喂哺技巧　正确的母、儿喂哺姿势可刺激婴儿的口腔动力，有利于吸吮。正确的喂哺技巧还包括如何唤起婴儿的最佳进奶状态，如哺乳前让婴儿用鼻推压或舔母亲的乳房，哺乳时婴儿的气味、身体的接触都可刺激乳母的射乳反射；等待哺乳的婴儿应是清醒状态、有饥饿感、已更换干净的尿布。

6．乳母心情愉快　因与泌乳有关的多种激素都直接或间接地受下丘脑的调节，下丘脑功能与情绪有关，故泌乳受情绪的影响很大，心情压抑可以刺激肾上腺素分泌，使乳腺血流量减少，阻碍营养物质和有关激素进入乳房，从而使乳汁分泌减少。刻板地规定哺乳时间也可造成精神紧张，故在婴儿早期应采取按需哺乳的方式并保证孕妇和乳母的身心愉快和充足的睡眠，避免精神紧张，可促进泌乳。

（四）不宜哺乳的情况

凡是母亲感染HIV、患有严重疾病应停止哺乳，如慢性肾炎、糖尿病、恶性肿瘤、精神病、癫痫或心功能不全等。乳母患急性传染病时，可将乳汁挤出，经消毒后哺喂。乙型肝炎的母婴传播主要发生在临产或分娩时，是通过胎盘或血液传递的，因此乙型肝炎病毒携带者并非哺乳的禁忌证。母亲感染结核病，但无临床症状时可继续哺乳。

二、部分母乳喂养

同时采用母乳与配方奶或兽乳喂养婴儿为部分母乳喂养，有两种情况。

1．补授法　母乳喂养的婴儿体重增长不满意时，提示母乳不足。此时用配方奶或兽乳补充母乳喂养为补授法，适宜4～6月内的婴儿。补授时，母乳哺喂次数一般不变，每次先哺母乳，将两侧乳房吸空后再以配方奶或兽乳补足母乳不足部分。这样有利于刺激母乳分泌。补授的乳量由小儿食欲及母乳量多少而定，即“缺多少补多少”。

2．代授法 用配方奶或兽乳替代一次母乳量，为代授法。母乳喂养婴儿至4～6 月龄时，为断离母乳开始引入配方奶或兽乳时宜采用代授法。即在某一次母乳哺喂时，有意减少哺喂母乳量，增加配方奶量或兽乳，逐渐替代此次母乳量。依次类推直到完全替代所有的母乳。

三、人工喂养

4～6个月以内的婴儿由于各种原因不能进行母乳喂养时，完全采用配方奶或其他兽乳，如牛乳、羊乳、马乳等喂哺婴儿，称为人工喂养。

（一）兽乳的特点（以牛乳为例）

人工喂养时常用牛乳，但成分不适合婴儿。

1．乳糖含量低　牛乳的乳糖含量低于人乳，主要为甲型乳糖，有利大肠杆菌的生长。

2．宏量营养素比例不当　牛乳蛋白质含量较人乳为高，且以酪蛋白为主，酪蛋白易在胃中形成较大的凝块；牛乳的氨基酸比例不当；牛乳脂肪颗滴大，而且缺乏脂肪酶，较难消化；牛乳不饱和脂肪酸（亚麻酸）（2％）低于人乳（8％）。牛乳含磷高，磷易与酪蛋白结合，影响

钙的吸收。

3．肾负荷重 牛乳含矿物质比人乳多3～3.5倍，增加婴儿肾脏的溶质负荷，对婴儿肾脏有潜在的损害。

4．缺乏免疫因子 牛乳缺乏各种免疫因子是与人乳的最大区别，故牛乳喂养的婴儿患感染性疾病的机会较多。

羊乳的营养价值与牛乳大致相同，蛋白质凝块较牛奶细而软，脂肪颗粒大小与人乳相仿。但羊乳中叶酸含量很少，长期哺给羊乳易致巨幼红细胞性贫血。马乳的蛋白质和脂肪含量少，能量亦低，故不宜长期哺用。

（二）牛乳的改造

由于种类的差异，兽乳所含的营养素不适合人类的婴儿。故一般人工喂养和婴儿断离母乳时应首选配方奶。

1．配方奶粉 是以牛乳为基础改造的奶制品，使宏量营养素成分尽量“接近”于人乳，使之适合于婴儿的消化能力和肾功能，如降低其酪蛋白、无机盐的含量等；添加一些重要的营养素，如乳清蛋白、不饱和脂肪酸、乳糖；强化婴儿生长时所需要的微量营养素如核苷酸、维生素A、D、β胡萝卜素和微量元素铁、锌等。使用时按年龄选用。

合理的奶粉调配在保证婴儿营养摄入中至关重要。一般市售配方奶粉配有统一规格的专用小勺。如盛4.4g奶粉的专用小勺，一勺宜加入30ml温开水；盛8.8g奶粉的专用小勺，一勺宜加入60ml温开水（重量比均为1：7）。

2．全牛乳的家庭改建 若无条件选用配方奶而采用兽乳喂养婴儿时，必须改造，不宜直接采用兽乳喂养婴儿。

（1）加热：煮沸可达到灭菌的要求，且能使奶中的蛋白质变性，使之在胃中不易凝成大块。

（2）加糖：婴儿食用全牛乳应加糖。这不是为增加牛乳甜味，或增加能量（因牛乳与母乳能量相近），而是改变牛乳中宏量营养素的比例，利于吸收，软化大便（表5-5）。一般每100ml牛奶中可加蔗糖5～8g。加糖过多或过少均不利于婴儿营养。

表5-5 三种乳类宏量营养素产能比较（%）

	人乳	8%糖牛奶	牛奶
蛋白质	9	13	19
脂肪	50	36	52
糖类	41	51	29
总能量（kcal）	67	99	67

（3）加水：降低牛奶矿物质、蛋白质浓度，减轻婴儿消化道、肾负荷。稀释奶仅用于新生儿，生后不满2周者可采用2：1奶（即2份牛奶加1份水）；以后逐渐过渡到3：1或4：1奶；满月后即可用全奶。

（三）奶量摄入的估计（6个月以内）

实际工作中为正确指导家长或评价婴儿的营养状况，常常需要估计婴儿奶量的摄入量。婴儿的体重、RNIs以及奶制品规格是估计婴儿奶量的必备资料。

1．配方奶粉摄入量估计 一般市售婴儿配方奶粉100g供能约500kcal（2029kJ），婴儿能量需要量约为100kcal/（kg.d）[418.4kJ/（kg.d）]，故需婴儿配方奶粉20g/（kg.d）可满足需要。按规定调配的配方奶蛋白质与矿物质浓度接近人乳，只要奶量适当，总液量亦可满足需要。

2．全牛奶摄入量估计　100ml全牛奶67kcal（280.33kJ），8%糖牛乳100ml供能约100kcal（418.4kJ），婴儿的能量需要量为100kcal/（kg.d）（418.4kJ）/（kg.d），婴儿需8%糖牛乳100ml/（kg.d）。全牛奶喂养时，因蛋白质与矿物质浓度较高，应两次喂哺之间加水，使奶与水量（总液量）达150ml/（kg.d）。

（四）正确的喂哺技巧

同母乳喂养一样，人工喂养喂哺婴儿亦需要有正确的喂哺技巧，包括正确的喂哺姿势、婴儿完全觉醒状态。还应注意选用适宜的奶嘴和奶瓶、奶液的温度、喂哺时奶瓶的位置。喂养时婴儿的眼睛尽量能与父母（或喂养者）对视。

四、婴儿食物转换

婴儿期随着生长发育的逐渐成熟，需要进入到由出生时的纯乳类向固体食物转换的换乳期。换乳期的泥状食物是人类生态学发展中不可逾越的食物形态，它不仅提供营养素，对儿童功能发育和能力获得还有重要促进作用，应引起儿科医师重视。

（一）不同喂养方式婴儿的食物转换

婴儿喂养的食物转换过程是让婴儿逐渐适应各种食物的味道、培养婴儿对其他食物感兴趣、逐渐由乳类为主要食物转换为进食固体为主的过程。母乳喂养婴儿的食物转换问题是帮助婴儿逐渐用配方奶或兽乳完全替代母乳，同时引入其他食物；部分母乳喂养和人工喂养婴儿的食物转换是逐渐引入其他食物。

（二）转乳期食物

是除母乳或配方奶（兽乳）外，为过渡到成人固体食物所添加的富含能量和各种营养素的泥状食物（半固体食物）（表5-6）。给婴儿引入食物的时间和过程应适合婴儿的接受能力，保证食物的结构、风味等能够被婴儿接受。

表5-6　过渡期食物的引入

月龄	食物性状	种类	餐数		进食技能
			主要营养源	辅助食品	
4～6月	泥状食物	菜泥、水果泥、含铁配方米粉、配方奶	6次奶（断夜间奶）	逐渐至1次	用勺喂
7～9月	末状食物	稀（软）饭、烂面菜末、蛋、鱼泥、豆腐、肉末、肝泥、水果	4次奶	1餐饭1次水果	学用杯
10～12月	碎食物	软饭、烂面碎肉、碎菜、蛋、鱼肉、豆制品、水果	3次奶1次水果	2餐饭	断奶瓶手抓食自用勺

应根据婴儿发育状况决定引入其他食物。一般应在婴儿体重达6.5～7kg，此时年龄多为4～6月龄。

给婴儿首先选择的其他食物应易于吸收、能满足生长需要、又不易产生食物过敏。首先添加的是含强化铁的米粉，其次引入的食物是根块茎蔬菜、水果，可补充维生素、矿物质营养；7～8月龄后逐渐引入动物性食物，如鱼类、蛋类、肉类、和豆制品。引入的食物制作应以当地食物为基础，注意食物的质地、营养密度、卫生、制作多样性。此期仍应保证600～800ml乳类，为婴儿营养的主要来源。

婴儿最初的对新食物的抵抗可通过多次体验改变。因此，婴儿食物转变期有一对其他食物的习惯过程。此期让婴儿熟悉多种食物，特别是蔬菜类，有利于儿童期完成食物转换。因此食

物加入应由少到多，即在哺乳后立即给予婴儿少量强化铁的米粉（1勺→2勺→多勺），6～7月龄后可代替1～2次乳量；一种到多种，如蔬菜的引入，应每种菜泥（茸）尝2次/日，直至3～4日婴儿习惯后再换另一种，以刺激味觉的发育。单一食物引入的方法可帮助了解婴儿是否出现食物过敏。为训练婴儿的进食能力应注意引入的方法和食物的质地。如用勺、杯进食可帮助口腔动作协调，学习主动吞咽；7～9个月后食物的质地从泥（茸）状过渡到碎末状可帮助学习咀嚼，增加食物的能量密度。此期还应注意婴儿神经心理发育对食物转变的作用，如允许手抓食物，既可增加婴儿进食的兴趣，又有利于眼手动作协调和培养独立能力。

（三）婴儿期易出现的问题

1．溢乳　15％的婴儿常出现溢乳，可因过度喂养、不成熟的胃肠运动类型、不稳定的进食时间造成。同时，婴儿胃呈水平位置，韧带松弛，易折叠；贲门括约肌松弛，幽门括约肌发育好的消化道的解剖生理特点使6个月内的小婴儿常常出见胃食管反流（gastroesophageal reflux，GER）。此外，喂养方法不当，如奶头过大、吞入气体过多时，婴儿也往往出现溢乳。

2．食物引入时间不当　过早引入半固体食物影响母乳铁吸收，增加食物过敏、肠道感染的机会；过晚引入其他食物，错过味觉、咀嚼功能发育关键年龄，造成进食行为异常，断离母乳困难，以致婴儿营养不足。引入半固体食物时采用奶瓶喂养，导致孩子不会主动咀嚼、吞咽饭菜。

3．能量及营养素摄入不足　8～9个月的婴儿已可接受能量密度较高的成人固体食物。如经常食用能量密度低的食物，或摄入液量过多，婴儿可表现进食后不满足，体重增长不足、下降，或在安睡后常于夜间醒来要求进食。

婴儿后期消化功能发育较成熟，应注意逐渐增加婴儿6个月后的半固体食物能量密度比，满足生长需要。避免给婴儿过多液量影响进食。

4．进餐频繁　胃的排空与否与消化能力密切相关。婴儿进餐频繁（超过7～8次/日），或延迟停止夜间进食，使胃排空不足，影响婴儿食欲。一般，安排婴儿一日6餐有利于形成饥饿的生物循环。

5．喂养困难　难以适应环境、过度敏感气质的婴儿常常有不稳定的进食时间，常常表现喂养困难。

第三节 幼儿营养与膳食安排

一、幼儿进食特点

1．生长速度减慢　1岁后儿童生长逐渐平稳。因此，幼儿进食相对稳定，较婴儿期旺盛的食欲相对略有下降。

2，心理行为影响　幼儿神经心理发育迅速，对周围世界充满好奇心，表现出探索性行为，进食时也表现出强烈的自我进食欲望。成人如忽略了儿童的要求，仍按小婴儿的方法抚养，儿童可表示不合作与违拗心理；而且儿童注意力易被分散，儿童进食时玩玩具、看电视等做法都会降低对食物的注意力，进食下降。应允许儿童参与进食，满足其自我进食欲望，培养独立进食能力。

3．家庭成员的影响　家庭成员进食的行为和对食物的反应可作为小儿的榜样。由于学习与社会的作用，小儿的进食过程形成了以后接受食物的类型。如给小儿食物是在积极的社会情况下（如奖励，或与愉快的社会行为有关），则小儿对食物的偏爱会增加；相反，强迫进食可使小儿不喜欢有营养的食物。

4．进食技能发育状况　幼儿的进食技能发育状况与婴儿期的训练有关，错过训练吞咽、咀嚼的关键期，长期食物过细，幼儿期会表现不愿吃固体食物，或“包在嘴中不吞”。

5．食欲波动 幼儿有准确的判断能量摄入的能力。这种能力不但是一餐中表现出来，连续几餐都可被证实。幼儿可能一日早餐吃很多，次日早餐什么也没吃；一天中吃得少的早餐，可能会有吃较多的中餐和较少的晚餐。变化的进食行为提示幼儿有调节进食的能力。研究显示幼儿餐间摄入的差别可达40％，但一日的能量摄入比较一致，只有10％的变化。

二、幼儿膳食安排

幼儿膳食中各种营养素和能量的摄入需满足该年龄阶段儿童的生理需要，蛋白质每日40g左右，其中优质蛋白（动物性蛋白质和豆类蛋白质）应占总蛋白的1/2。蛋白质、脂肪和糖类产能之比约为10％～15％：30％～35％：50％～60％。但膳食安排需合理，四餐（奶类2，主食2）二点为宜。频繁进食、夜间进食、过多饮水均会影响小儿的食欲。

第四节 营养状况评价的原则

儿童营养状况评价包括临床表现、体格发育评价、膳食调查以及实验室检查四方面进行综合。

（一）体格检查

除常规体格检查外，注意有关营养素缺乏体征。

（二）体格发育评价

见有关章节。

（三）膳食调查

按工作要求选择不同方法。

（1）称重法：实际称量各餐进食量，以生／熟比例计算实际摄入量。查“食物成分表”得出今日主要营养素的量（人均量）。通常应按季节、食物供给不同每季度测一次。调查需准备表格、食物成分表、计算器、秤（食物、器皿重）。称重法的优点是准确，但较复杂，调查时间较长（3～4日）。多应用集体儿童膳食调查，也可据调查目的选择个人进行膳食调查。

常以平均数法分析结果，即从每日摄入食物种类、数量计算各种食物中某营养素的总量，用日人数算出人平均摄入量。日人数为三餐人数的平均数。

（注：如三餐就餐儿童数相差太大，应按日人数计算出人平均摄入量。日人数＝早餐主食量／早餐人数＋中餐主食量／中餐人数+晚餐主食量／晚餐人数）

（2）询问法：多用于个人膳食调查，采用询问对象刚刚吃过的食物或过去一段时间吃过的食物。询问法又分24小时回忆法、膳食史法和食物频度法了解膳食习惯。询问法简单，易于临床使用，但因结果受被调查对象报告情况或调查者对市场供应情况以及器具熟悉程度的影响而不准确。多种方法结合可增加准确性。采用24小时回忆法一般至少要调查2～3次，计算与结果分析同称重法。

（3）记帐法：多用于集体儿童膳食调查，以食物记出入库的量算。记帐法简单，但结果不准确，要求记录时间较长。计算与结果分析同称重法。

2．膳食评价：将膳食调查结果与DRIs比较。

（1）营养素摄入：对个体而言：计算出的摄入量低于EAR时摄入不足的几率高达50%，必须提高摄入；摄入量在EAR和RNI之间也可能需要改变，因为摄入不足的几率大于2%～3%，只

有多天的观测达到或超过RNI时，或虽系几天的观测但结果远高于RNI时才可以有把握的认为摄入量是充足的。

对群体而言：计算出的摄入量低于EAR时在群体中占的百分比数即为摄入不足的比例数；摄入量等于或高于RNI，人群摄入不足的几率较小；不宜用RNI和AI作为切点来评估人群摄入不足，也不宜用食物频数来评估人群摄入不足。

当能量摄入>EAR时，显示能量摄入足够，反之说明能量摄入不足；当蛋白质摄入大于或等于RNI或AI时，显示蛋白质摄入足够，反之说明蛋白质摄入不足，优质蛋白质占膳食中蛋白质总量的1/2以上；矿物质、维生素摄入应大于或等于RNI或AI。

（2）宏量营养素供能比例：膳食中宏量营养素比例应适当，即蛋白质产能应占总能量的10％～15％，7岁以上脂类占总能量的25％～30％（其余年龄见附录二），糖类占总能量的50％～60％。

（3）膳食能量分布：每日三餐食物供能亦应适当，即早餐供能应占一日总能量的25％～30％，中餐应占总能量的35％～45％，点心占总能量的10％，晚餐应占总能量的25％～30％。

（四）实验室检查

了解机体某种营养素贮存、缺乏水平。通过实验方法测定小儿体液或排泄物中各种营养素及其代谢产物或其他有关的化学成分，了解食物中营养素的吸收利用情况。

（李廷玉）

第五节 维生素营养障碍

一、维生素A缺乏病

维生素A缺乏病（vitamin A ddficiency disorder）目前仍是不发达国家中威胁人类健康，尤其是儿童的主要疾病之一。其临床表现除了皮肤黏膜改变（如毛囊角化、角膜软化等）和影响视网膜上视紫红质更新引起夜盲外，还能在此之前出现免疫功能损伤，导致易感性上升，这种“亚临床状态维生素A缺乏”现象已日益引起人们的重视。我国儿童中维生素A缺乏病的发生率已明显下降，但在边远农村地区仍有群体流行，亚临床状态缺乏现象还相当普遍。

【吸收与代谢】

维生素A的化学名为视黄醇，在动物性食物如乳类、蛋类和动物内脏中含量丰富，在不发达地区由于此类食物供应较少，往往要依靠以植物来源的胡萝卜素作为维生素A的重要供应来源。胡萝卜素在深色蔬菜中含量较高，其中最具有维生素A生物活性的是β-胡萝卜素，但其在人类肠道中的吸收利用率很低，大约仅为维生素A的1/6，其他胡萝卜素的吸收率更低。无论胡萝卜素还是维生素A，在小肠细胞中转化成棕榈酸酯后均与乳糜微粒结合通过淋巴系统入血行然后转运到肝脏。在肝脏中再酯化为棕榈酸酯后储存。当周围靶组织需要维生素A时，肝脏中的维生素A棕榈酸酯经酯酶水解为醇式后，与视黄醇结合蛋白结合，再与前白蛋白结合，形成复合体后释放入血，经血行转运至靶组织。维生素A在体内氧化后转变为视黄酸，视黄酸是维生素A在体内发生多种生物作用的重要活性形式，如维持上皮细胞活性、调节淋巴细胞功能等。

【生理功能和病理改变】

维生素A缺乏会影响很多生理功能和产生不少病理变化。

1．维持皮肤粘膜层的完整性　维生素A是调节糖蛋白合成的一种辅酶，对上皮细胞的细胞膜起稳定作用，维持上皮细胞的形态完整和功能健全。维生素A缺乏的初期病理改变是上皮组织干燥，继而使正常柱状上皮细胞转变为角状复层扁平上皮，形成过度角化变性和腺体分泌减少。这种变化累及全身上皮组织，最早受影响的是眼睛的结膜和角膜，表现为结膜和角膜干燥、软化甚至穿孔，以及泪腺分泌减少。皮肤改变则为毛囊角化，皮脂腺、汗腺萎缩。消化道表现为舌味蕾上皮角化，肠道黏膜分泌减少，食欲减退等。呼吸道黏膜上皮萎缩、干燥，纤毛减少，抗病能力减退。消化道和呼吸道感染性疾病的危险性提高，且感染常迁延不愈。泌尿和生殖系统上皮细胞也有同样改变，影响其功能。

2．构成视觉细胞内的感光物质　视网膜上对暗光敏感的杆状细胞含有感光物质视紫红质，由11-顺式视黄醛与视蛋白结合而成，为暗视觉的必需物质。经光照漂白后，11-顺式视黄醇转变为全反式视黄醛并与视蛋白分离。此过程产生电能刺激视神经形成视觉。全反式视黄醛还原为全反式视黄醇，再经酶的作用重新转化为11-顺式视黄醛，可在暗光下与视蛋白结合再次形成视紫红质。在此过程中，除了消耗能量和酶外，还有部分视黄醛变成视黄醇被排泄，所以必须不断地补充维生素A，才能维持视紫红质的合成和整个暗光视觉过程。

3．促进生长发育和维护生殖功能　维生素A参与细胞的RNA、DNA合成，对细胞分化、组织更新有一定影响。参与软骨内成骨，缺乏时长骨形成和牙齿发育均受障碍。维生素A缺乏时还会导致男性睾丸萎缩，精子数量减少、活力下降，也可影响胎盘发育。

4．维持和促进免疫功能　目前已经明确，维生素A对许多细胞功能活动的维持和促进作用是通过其在细胞核内的特异性受体－视黄酸受体实现的。视黄酸受体可以形成异源性二聚体或同源性二聚体与视黄酸反应元件结合从而调控靶细胞基因的相应区域。这种对基因调控结果可以促进免疫细胞产生抗体的能力，也可以促进细胞免疫功能，以及促进T淋巴细胞产生某些细胞因子。维生素A缺乏时，免疫细胞内视黄酸受体的表达相应下降，因此影响机体的免疫功能。

【病因】

1．原发性因素　儿童维生素A缺乏在4岁以下儿童的发生率远高于成人，其主要原因是维生素A和胡萝卜素都很难通过胎盘进入胎儿体内，因此新生儿血清和肝脏中的维生素A水平明显低于母体，如在出生后不能得到充足的维生素A补充则极易出现维生素A缺乏病。

血浆中视黄醇结合蛋白的水平低下会导致血浆维生素A的下降，引起维生素A缺乏。新生儿的血浆视黄醇结合蛋白只有成人的一半左右，要到青春期才逐步达到成人水平。这也是小年龄儿童容易招致维生素A缺乏的原因之一。

2．消化吸收影响因素　维生素A为脂溶性维生素，它和胡萝卜素在小肠的消化吸收都依靠胆盐的帮助，膳食中脂肪含量与它们的吸收有密切联系。膳食中脂肪含量过低，胰腺炎或胆石症引起胆汁和胰腺酶分泌减少，一些消化道疾病如急性肠炎、粥样泻等造成胃肠功能紊乱都可以影响维生素A和胡萝卜素的消化和吸收。

3．储存利用影响因素　任何影响肝脏功能的疾病都会影响维生素A在体内储存量，造成维生素A缺乏。一些消耗性传染病，尤其是儿童中的麻疹、猩红热、肺炎和结核病等都会使体内的维生素A存储消耗殆尽，摄入量则往往因食欲不振或消化功能紊乱而明显减少，两者的综合结果势必导致维生素A缺乏病发生。

【临床表现】

1．眼部表现　眼部的症状和体征是维生素A缺乏病的早期表现。夜盲或暗光中视物不清最早出现，但往往不被重视，婴幼儿也常常不会叙述。上述暗适应力减退的现象持续数周后，开始出现干眼症的表现，外观眼结膜、角膜干燥，失去光泽，自觉痒感，泪减少，眼部检查可见结膜近角膜边缘处干燥起皱褶，角化上皮堆积形成泡沫状白斑，称结膜干燥斑或毕脱斑（Bitot's spots）。继而角膜发生干燥、浑浊、软化，自觉畏光、眼痛，常用手揉搓眼部导致感染。严重时可发生角膜溃疡、坏死引起穿孔，虹膜、晶状体脱出，导致失明。这些表现多见于小年龄儿童罹患消耗性感染性疾病如麻疹、疟疾等之后，多数为双侧同时发病。

2．皮肤表现　开始时仅感皮肤干燥、易脱屑，有痒感，渐至上皮角化增生，汗液减少，角化物充塞毛囊形成毛囊丘疹。检查触摸皮肤时有粗砂样感觉，以四肢伸面、肩部为多，可发展至颈、背部甚至面部。毛囊角化引起毛发干燥，失去光泽，易脱落，指（趾）甲变脆易折、多纹等。

3．生长发育障碍　维生素A缺乏会影响儿童的生长发育，主要是骨骼系统的生长发育。表现为长骨增长迟滞，同时齿龈发生增生和角化，影响成釉质细胞发育。临床表现为身高落后，牙齿釉质易剥落，失去光泽，易发生龋齿。由于颅骨、脊椎骨发育受阻而神经系统发育照常，使两者不相称，引起脑和脊髓组织受压，导致颅内压增高和脊神经萎缩。

4．易感性增高　在维生素A缺乏早期甚或亚临床状态缺乏时，免疫功能低下就已经可能存在，表现为消化道和呼吸道感染性疾病发生率增高，且易迁延不愈。

5．其他　维生素A有促进肝脏中储存铁释放入血后的转运，使铁能正常地被红细胞摄入利用。因此维生素A缺乏时会出现贫血，其表现类似缺铁性贫血，血红蛋白、红细胞比容和血清铁水平降低，血清铁蛋白正常，肝脏和骨髓储存铁反而增加。维生素A缺乏能使泌尿器官的上皮发生角化脱屑，并形成一个中心病灶，钙化物以此为中心不断沉淀而形成尿系道结石。

【诊断】

1．临床诊断　长期动物性食物摄入不足，有各种消化道疾病或慢性消耗性疾病史，急性传染病史等情况下应高度警惕维生素A缺乏病。如出现夜盲或眼干燥症等眼部特异性表现，以及皮肤的症状和体征时，诊断本病困难不大。为了进一步早期确诊，应根据当地条件进行实验室检查。

2．实验室诊断

（1）血浆维生素A测定：婴幼儿血浆正常水平为300～500μg /L，年长儿和成人为300～800μg /L，低于200μg /L 可诊断为维生素A缺乏，200～300μg /L 为亚临床状态缺乏可疑。血浆维生素A水平并不能完全反映全身组织维生素A营养状态，因此在高度怀疑时可以使用相对剂量反应试验（RDR）进一步确定。其方法是在空腹时采取静脉血（A_0），然后口服视黄醇制剂450μg，5小时后再次采取静脉血（A_5），测定两次血浆中维生素A的水平并按公式（如下）计算RDR值，如RDR值大于20％为阳性，表示存在亚临床状态维生素A缺乏。

$$RDR\% = \frac{A_5 - A_0}{A_5} \times 100$$

（2）血浆视黄醇结合蛋白测定：血浆视黄醇结合蛋白（RBP）水平能比较敏感地反映体内维生素A的营养状态，低于正常范围有维生素A缺乏的可能。

（3）尿液脱落细胞检查：加1％甲紫于新鲜中段尿中，摇匀计数尿中上皮细胞，如无泌尿

道感染，超过3个/mm^3为异常，有助于维生素A缺乏诊断，找到角化上皮细胞具有诊断意义。

（4）暗适应检查：用暗适应计和视网膜电流变化检查，如发现暗光视觉异常，有助诊断。

有明确摄入不足或消耗增加的病史，以及明显的维生素A缺乏的临床表现者即可做出临床诊断，进行治疗。实验室检查结果表明血清维生素A低于正常水平则有助于确认和疗效随访。亚临床状态的维生素A缺乏往往没有明显的临床表现，其诊断主要依靠实验室检查。

【预防】

平时注意膳食的营养平衡，经常食用富含维生素A的动物性食物和深色蔬菜，一般不会发生维生素A缺乏。小年龄儿童是预防维生素A缺乏的主要对象，孕妇和乳母应多食上述食物，以保证新生儿和乳儿有充足的维生素A摄入。母乳喂养优于人工喂养，人工喂养婴儿应尽量选择维生素A强化的乳方，每日推荐供应量婴幼儿为400μg视黄醇当量（RE），5岁以上儿童为750μg RE，少年和成人为800μg RE，孕妇为1000μg RE，乳母为1200μg RE（1IU维生素A＝0.3μg RE=6μgβ胡萝卜素）。在维生素A缺乏的高发地区，可以采取每隔半年给予一次口服维生素A 的预防措施，大于1岁的儿童每次66000μg RE（20万IU），6～12个月的婴儿每次33000μg RE（10万IU），小于6个月的小婴儿每次17000μg RE（5万IU）。对患感染性疾病如麻疹、疟疾和结核病等，以及慢性消耗性疾病的病人应及早补充维生素A制剂。有慢性腹泻等维生素A吸收不良者可短期内肌注维生素A，数日后再改为口服，或采用水溶性维生素A制剂。采用大剂量维生素A作预防时应注意避免过量造成中毒。

【治疗】

无论临床症状严重与否，甚或是无明显症状的亚临床状态维生素A缺乏，都应该尽早进行维生素A的补充治疗，因为多数病理改变经治疗后都可能逆转而恢复。

1．调整饮食、去除病因　提供富含维生素A的动物性食物或含胡萝卜素较多的深色蔬菜，有条件的地方也可以采用维生素A强化的食品如婴儿的配方奶粉和辅食等。此外，应重视原发病的治疗。

2．维生素A制剂治疗　轻症维生素A缺乏病及消化吸收功能良好者可以每日口服维生素A制剂7500μg～15000μg（相当于2.5万～5万IU，浓维生素A丸含2.5万IU/丸），分2～3次服用，2天后减量为每天口服1500μg（4500IU）。如有慢性腹泻或肠道吸收障碍者或重症患者，可先采用深部肌注维生素AD注射剂（每支含维生素A 7500μg和维生素D 62.5μg）0.5～lml，每日1次。3～5天后，病情好转即改口服。经维生素A治疗后临床症状好转迅速，夜盲常于2～3天后明显改善，干眼症状3～5日消失，结膜干燥、毕脱氏斑1～2周后消失，角膜病变也渐好转，皮肤过度角化需1～2月方痊愈。

3．眼局部治疗　除全身治疗外，对比较严重的维生素A缺乏病患者常有眼部的局部治疗。为预防结膜和角膜发生继发感染，可采用抗生素眼药水（如0.25％氯霉素）或眼膏（如0.5％红霉素或金霉素）治疗，每日3～4次，可减轻结膜和角膜干燥不适。如果角膜出现软化和溃疡时，可采用抗生素眼药水与消毒鱼肝油交替滴眼，约1小时一次，每日不少于20次。治疗时动作要轻柔，勿压迫眼球，以免角膜穿孔，虹膜、晶状体脱出。

【附】 维生素A过多症和胡萝卜素血症

维生素A摄入过多可以引起维生素A过多症，分为急性和慢性两种。维生素A过量会降低细胞膜和溶酶体膜的稳定性，导致细胞膜受损，组织酶释放，引起皮肤、骨骼、脑、肝等多种脏

器组织病变。脑受损可使颅压增高；骨组织变性引起骨质吸收、变形、骨膜下新骨形成、血钙和尿钙都上升。肝组织受损则引起肝脏肿大，肝功能改变。

1．急性维生素A过多症　成人一次剂量超过30万～100万IU，儿童一次剂量超过30万IU即可能发生急性中毒。从曾发生的急性维生素A过多症病史看，成人多为食用大量富含维生素A的食物如北极熊、鲨鱼和鳕鱼等的肝而发生中毒，儿童则多因意外服用大量维生素A、D制剂引起。

临床表现在摄入后6～8小时，至多在1～2天内出现。主要有嗜睡或过度兴奋，头痛、呕吐等高颅压症状，12～20小时后出现皮肤红肿，继尔脱皮，以手掌、脚底等厚处最为明显，数周后方恢复正常。婴幼儿以高颅压为主要临床特征，囟门未闭者可出现前囟隆起。脑脊液检查压力增高，细胞数正常，蛋白质量偏低，糖正常。血浆维生素A水平剧增，可达500μg / L以上（正常成人100～300μg /L）。

2．慢性维生素A过多症　多因不遵医嘱长期摄入过量维生素A制剂引起。从已发生的病案看，成人每天摄入8万～10万IU，持续半年；或每天3万～4万IU，超过8年可引起慢性中毒。婴幼儿每天摄入5万～10万IU，超过6个月即可引起慢性中毒；也有报道每天仅服2.5万IU，1个月即出现中毒症状者。这种情况常见于采用口服鱼肝油制剂治疗维生素D缺乏性佝偻病时，由于鱼肝油制剂既含有维生素D又有维生素A，当口服途径使用较大治疗剂量的维生素D时极易造成维生素A的过量。

临床表现不似急性维生素A过多症那样迅速出现高颅压和皮肤损害的症状及体征。成人慢性维生素A过多症首先出现的常是胃纳减退，体重下降，继尔有皮肤干燥、脱屑、皲裂、毛发干枯、脱发、齿龈红肿、唇干裂和鼻出血等皮肤黏膜损伤现象，以及长骨肌肉连接处疼痛伴肿胀，体格检查可见贫血、肝脾肿大。X线检查长骨可见骨皮质增生，骨膜增厚。脑脊液检查可有压力增高。肝功能检查可出现转氨酶升高，严重者可出现肝硬化表现。有时可见血钙和尿钙升高。

根据过量摄入维生素A的病史，临床表现，血浆维生素A浓度明显升高以及X线检查等其他实验室检查结果，对于急、慢性维生素A过多症的诊断并不困难。惟慢性维生素A过多症的早期临床表现可能只是个别症状或体征，容易误诊，应注意同佝偻病、坏血病等鉴别。

维生素A 过多症一旦确诊，应立即停止服用维生素A制剂和含维生素A的食物。急性维生素A过多症的症状一般在1～2周内消失，骨骼改变也逐渐恢复，但较缓慢，约需2～3个月。一般不需其他治疗。高颅压引起的反复呕吐以及因此发生的水和电解质紊乱应给予对症治疗。本病预后良好，个别病程长、病情严重者可留下身材矮小后遗症。

3，胡萝卜素血症 因摄入富含胡萝卜素的食物（如胡萝卜、南瓜、橘子等）过多，以致大量胡萝卜素不能充分迅速在小肠黏膜细胞中转化为维生素A而引起。虽然摄入的β-胡萝卜素在体内可转化为维生素A，但其吸收率只有1/3，而吸收的胡萝卜素只有一半可以转化为维生素A，所以胡萝卜素摄入量最后仅有1/6发挥维生素A的作用，故大量摄入胡萝卜素一般不会引起维生素A过多症，但可以使血中胡萝卜素水平增高，发生胡萝卜素血症。血清胡萝卜素含量明显升高，可达4.7～9.3μmol/L（正常为1.9～2.7μmol/L），致使黄色素沉着在皮肤内和皮下组织内，表现为皮肤黄染，以鼻尖、鼻唇皱襞、前额、手掌和足底部位明显，但巩膜无黄染。停止大量食入富含胡萝卜素的食物后，胡萝卜素血症可在2～6周内逐渐消退，一般没有生命危险。不需特殊治疗。

（王卫平）

二、营养性维生素D缺乏

（一）营养性维生素D缺乏性佝偻病

营养性维生素D缺乏佝偻病（rickets of vitamin D deficiency）是由于儿童体内维生素D不足使钙、磷代谢紊乱，产生的一种以骨骼病变为特征的全身慢性营养性疾病。典型的表现是生长着的长骨干骺端和骨组织矿化不全，维生素D不足使成熟骨矿化不全，则表现为骨质软化症（Osteomalacia）。

婴幼儿特别是小婴儿是高危人群，北方佝偻病患病率高于南方。近年来，随社会经济文化水平的提高，我国营养性维生素D缺乏性佝偻病发病率逐年降低，病情也趋于轻度。

【维生素D的生理功能与代谢】

1．维生素D的体内活化　维生素D是一组具有生物活性的脂溶性类固醇衍生物（secosteroids），包括维生素D_2（麦角骨化醇，ergocalcifero1）和维生素D_3（胆骨化醇，cholecalciferol），前者存在于植物中，后者系由人体或动物皮肤中的7-脱氢胆固醇经日光中紫外线的光化学作用转变而成。食物中的维生素D_2在胆汁的作用下，在小肠刷状缘经淋巴管吸。皮肤合成的维生素D_3直接吸收入血。维生素D_2和D_3在人体内都没有生物活性，它们被摄入血循环后即与血浆中的维生素D结合蛋白（DBP）相结合后被转运到肝脏。维生素D在体内必须经过两次羟化作用后始能发挥生物效应。首先经肝细胞发生第一次羟化，生成25-羟维生素D_3 [25-(OH)D_3]，这个过程受饮食维生素D、25-(OH)D_3和1,25-(OH)D_3的负调节。25-(OH)D_3是循环中维生素D的主要形式。循环中的25-(OH)D_3与α-球蛋白结合被运载到肾脏，在近端肾小管上皮细胞线粒体中的1-α羟化酶的作用下再次羟化，生成有很强生物活性的1，25-二羟维生素D，即1,25-(OH)D_3。

2．维生素D 的生理功能　从肝脏释放入血循环中的25-(OH)D_3浓度较稳定，可反映体内维生素D的营养状况，正常含量为11～60ng/ml。25-(OH)D_3虽有一定的生物活性，但在生理浓度范围时，作用较弱，可动员骨钙入血，抗佝偻病的生物活性较低。

正常情况下，血循环中的1,25-(OH)D_3主要与DBP 相结合，对靶细胞发挥其生物较应。1,25-(OH)D_3是维持钙、磷代谢平衡的主要激素之一，主要通过作用于靶器官（肠、肾、骨）而发挥其抗佝偻病的生理功能：①促小肠黏膜细胞合成一种特殊的钙结合蛋白（CaBP），增加肠道钙的吸收，磷也伴之吸收增加，1,25-(OH)D_3可能有直接促进磷转运的作用；②增加肾近曲小管对钙、磷的重吸收，特别是磷的重吸收，提高血磷浓度，有利于骨的矿化作用。③对骨骼钙的动员：与甲状旁腺协同使破骨细胞成熟，促进骨重吸收，旧骨中钙盐释放入血；另一方面刺激成骨细胞促进骨样组织成熟和钙盐沉积。

目前研究进展认为1,25-(OH)D_3不仅是一个重要的营养成分，也是激素前体，其核受体除存在于骨骼、肾、肠道外，还广泛存在于其他组织如胎盘、甲状旁腺、胰腺、胃、脑和包括与钙平衡及免疫、内分泌、生殖、皮肤和肿瘤等密切相关的多种细胞，1,25-(OH)D_3参与多种细胞的增殖、分化和免疫功能的调控过程，对人体有很多其他重要作用。

3．维生素D 代谢的调节

（1）自身反馈作用：正常情况下维生素D的合成与分泌是据机体需要受血中25-(OH)D_3的浓度自行调节，即生成的1,25-(OH)D_3的量达到一定水平时，可抑制25-(OH)D_3在肝内的羟化、1,25-(OH)D_3在肾脏羟化过程。

（2）血钙、磷浓度与甲状旁腺、降钙素调节：肾脏生成1,25-(OH)D_3间接受血钙浓度调节。当血钙过低时，甲状旁腺（PTH）分泌增加，PTH 刺激肾脏1,25-(OH)D_3合成增多；PTH与

1,25-$(OH)D_3$共同作用于骨组织，使破骨细胞活性增加，降低成骨细胞活性，骨重吸收增加，骨钙释放入血，使血钙升高，以维持正常生理功能。血钙过高时，降钙素（CT）分泌，抑制肾小管羟化生成1,25-$(OH)D_3$。血磷降低可直接促肾脏内1,25-$(OH)D_3$的增加，高血磷则抑制其合成。

【维生素D的来源】

婴幼儿体内维生素D来源有三个途径。

1．母体-胎儿的转运　胎儿可通过胎盘从母体获得维生素D，胎儿体内25-$(OH)D_3$的贮存可满足生后一段时间的生长需要。早期新生儿体内维生素D的量与母体的维生素D的营养状况及胎龄有关。

2．食物中的维生素D　天然食物中含维生素D很少，母乳含维生素D少，谷物、蔬菜、水果不含维生素D，肉和白鱼含量很少。但配方奶粉和米粉摄入足够量，婴幼儿可从这些强化维生素D的食物中获得充足的维生素D。

3．皮肤的光照合成　是人类维生素D的主要来源。人类皮肤中的7-脱氢胆骨化醇（7-DHC），是维生素D生物合成的前体，经日光中紫外线照射（290～320nm波长），变为胆骨化醇，即内源性维生素D_3。皮肤产生维生素D_3的量与日照时间、波长、暴露皮肤的面积有关。

【病因】

1．围生期维生素D不足　母亲妊娠期，特别是妊娠后期维生素D营养不足，如母亲严重营养不良、肝肾疾病、慢性腹泻，以及早产、双胎均可使婴儿的体内贮存不足。

2．日照不足　因紫外线不能通过玻璃窗，婴幼儿被长期过多的留在室内活动，使内源性维生素D生成不足。大城市高大建筑可阻挡日光照射，大气污染如烟雾、尘埃可吸收部分紫外线。气候的影响，如冬季日照短，紫外线较弱，亦可影响部分内源性维生素D的生成。

3．生长速度快，需要增加　如早产及双胎婴儿生后生长发育快，需要维生素D多，且体内贮存的维生素D不足。婴儿早期生长速度较快，也易发生佝偻病。重度营养不良婴儿生长迟缓，发生佝偻病者不多。

4．食物中补充维生素D不足　因天然食物中含维生素D少，即使纯母乳喂养，婴儿若户外活动少亦易患佝偻病。

5．疾病影响　胃肠道或肝胆疾病影响维生素D吸收，如婴儿肝炎综合征、慢性腹泻等，肝、肾严重损害可致维生素D羟化障碍，1,25-$(OH)D_3$生成不足而引起佝偻病。长期服用抗惊厥药物可使体内维生素D不足，如苯妥英钠、苯巴比妥，可刺激肝细胞微粒体的氧化酶系统活性增加，使维生素D和25-$(OH)D_3$加速分解为无活性的代谢产物。糖皮质激素有对抗维生素D对钙的转运作用。

【发病机理】

维生素D缺乏性佝偻病可以看成是机体为维持血钙水平而对骨骼造成的损害。长期严重维生素D缺乏造成肠道吸收钙、磷减少和低血钙症，以致甲状旁腺功能代偿性亢进，PTH分泌增加以动员骨钙释出使血清钙浓度维持在正常或接近正常的水平；但PTH同时也抑制肾小管重吸收磷，继发机体严重钙、磷代谢失调，特别是严重低血磷的结果（图5-2）。细胞外液钙、磷浓度不足破坏了软骨细胞正常增殖、分化和凋亡的程序；钙化管排列紊乱，使长骨钙化带消失、骺板失去正常的形态，参差不齐；骨基质不能正常矿化，成骨细胞代偿增生，碱性磷酸酶分泌增加，骨样组织堆积于干骺端，骺端增厚，向两侧膨出形成“串珠”，“手足镯”。骨膜下骨矿化不全，成骨异常，骨皮质被骨样组织替代，骨膜增厚，骨皮质变薄，骨质疏松；负重出现弯曲；颅骨骨化障碍而颅骨软化，颅骨骨样组织堆积出现“方颅”。临床即出现一系列佝偻病症状和血生化改变。

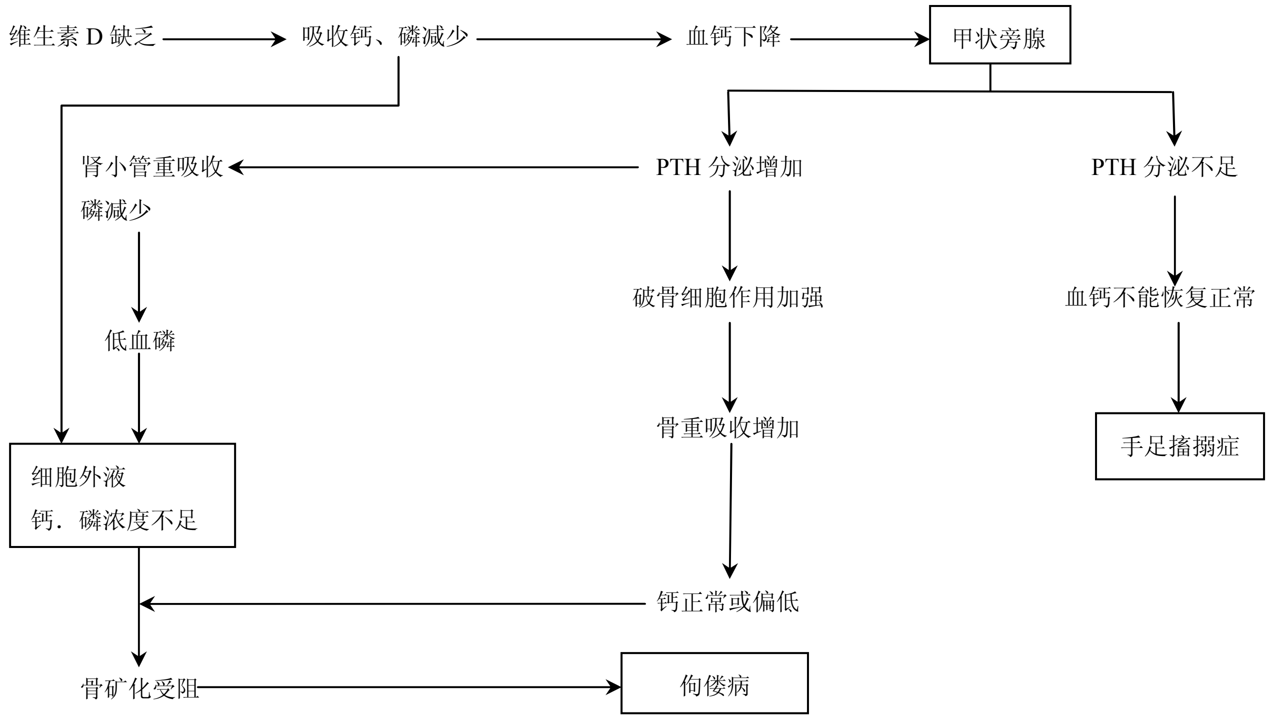

图5-2　维生素D缺乏性佝偻病和手足搐搦症的发病机制

【临床表现】

多见于婴幼儿，特别是小婴儿。主要表现为生长最快部位的骨骼改变，并可影响肌肉发育及神经兴奋性的改变。因此年龄不同，临床表现不同。佝偻病的骨骼改变常在维生素D缺乏数月后出现，围生期维生素D不足的婴儿佝偻病出现较早。儿童期发生佝偻病的较少。重症佝偻病患儿还可有消化和心肺功能障碍，并可影响行为发育和免疫功能。本病在临床上可分期如下：

1．初期（早期）　多见6个月以内，特别是3个月以内小婴儿。多为神经兴奋性增高的表现，如易激惹、烦闹、汗多刺激头皮而摇头等。但这些并非佝偻病的特异症状，仅作为临床早期诊断的参考依据。此期常无骨骼病变，骨骼X线可正常，或钙化带稍模糊；血清25-(OH)D_3下降，PTH升高，血钙下降，血磷降低，碱性磷酸酶正常或稍高。

2．活动期（激期）　早期维生素D缺乏的婴儿未经治疗，继续加重，出现PTH功能亢进和钙、磷代谢失常的典型骨骼改变。

6月龄以内婴儿的佝偻病以颅骨改变为主，前囟边较软，颅骨薄，检查者用双手固定婴儿头部，指尖稍用力压迫枕骨或顶骨的后部，可有压乒乓球样的感觉。6月龄以后，尽管病情仍在进展，但颅骨软化消失。正常婴儿的骨缝周围亦可有乒乓球样感觉。额骨和顶骨中心部分常常逐渐增厚，至7～8个月时，变成“方盒样”头型即方头（从上向下看），头围也较正常增大。骨骺端因骨样组织堆积而膨大，沿肋骨方向于肋骨与肋软骨交界处可扪及圆形隆起，从上至下如串珠样突起，以第7～10肋骨最明显，称佝偻病串珠（rachitic rosary）；手腕、足踝部亦可形成钝圆形环状隆起，称手、足镯。1岁左右的小儿可见到胸骨和邻近的软骨向前突起，形成“鸡胸样”畸形；严重佝偻病小儿胸廓的下缘形成一水平凹陷，即肋膈沟或郝氏沟（Harrison’s groove）。由于骨质软化与肌肉关节松弛，小儿开始站立与行走后双下肢负重，可出现股骨、胫骨、腓骨弯曲，形成严重膝内翻（“O”形）或膝外翻（“X”形），有时有“K”形样下肢畸形。

患儿会坐与站立后，因韧带松弛可致脊柱畸形。严重低血磷使肌肉糖代谢障碍，使全身肌

肉松弛，肌张力降低和肌力减弱。

此期血生化除血清钙稍低外，其余指标改变更加显著。

X线显示长骨钙化带消失，干骺端呈毛刷样、杯口状改变；骨骺软骨盘增宽（>2mm）；骨质稀疏，骨皮质变薄；可有骨干弯曲畸形或青枝骨折，骨折可无临床症状（图5-3）。

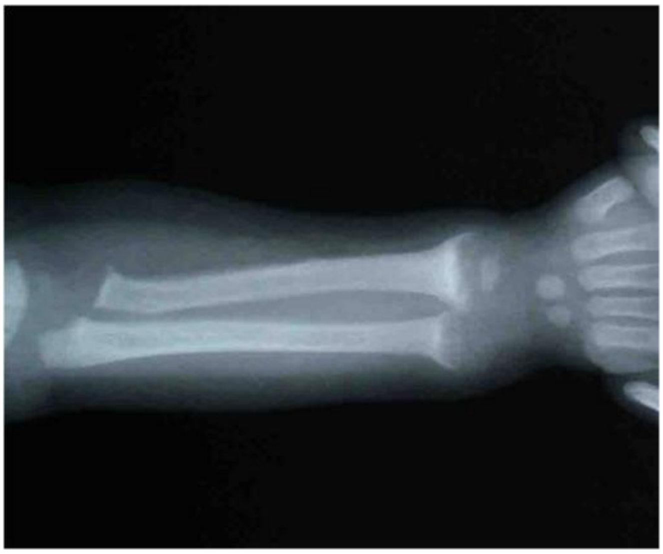

图 5-3　佝偻病时骨骼 X 线改变

3．恢复期　以上任何期经治疗或日光照射后，临床症状和体征逐渐减轻或消失。血钙、 磷逐渐恢复正常，碱性磷酸酶约需1～2月降至正常水平。治疗2～3周后骨骼X线改变有所改善，出现不规则的钙化线，以后钙化带致密增厚，骨骺软骨盘<2mm，逐渐恢复正常。

4．后遗症期　多见于2岁以后的儿童。因婴幼儿期严重佝偻病，残留不同程度的骨骼畸形。无任何临床症状，血生化正常，X线检查骨骼干骺端病变消失。

【诊断】

要解决是否有佝偻病、如有属于哪个期、是否需要治疗。正确的诊断必须依据维生素D缺乏的病因、临床表现、血生化及骨骼X线检查。应注意早期的神经兴奋性增高的症状无特异性，如多汗、烦闹等，仅据临床表现的诊断准确率较低；骨骼的改变可行；血清25-(OH)D_3水平为最可靠的诊断标准，但很多单位不能检测。血生化与骨骼X线的检查为诊断的“金标准”。

【鉴别诊断】

1．与佝偻病的体征的鉴别

（1）粘多糖病：粘多糖代谢异常时，常多器官受累，可出现多发性骨发育不全，如头大、头型异常、脊柱畸形、胸廓扁平等体征。此病除临床表现外，主要依据骨骼的X线变化及尿中粘多糖的测定作出诊断。

（2）软骨营养不良：是一遗传性软骨发育障碍，出生时即可见四肢短、头大、前额突出、腰椎前突、臀部后凸。根据特殊的体态（短肢型矮小）及骨骼X线作出诊断。

（3）脑积水：生后数月起病者，头围与前囟进行性增大。因颅内压增高，可见前囟饱满紧张，骨缝分离，颅骨叩诊有破壶声，严重时两眼向下呈落日状。头颅B超、CT检查可做出诊断。

2．与佝偻病体征相同而病因不同的鉴别

（1）低血磷抗生素D佝偻病：本病多为性连锁遗传，亦可为常染色体显性或隐性遗传，也有散发病例。为肾小管重吸收磷及肠道吸收磷的原发性缺陷所致。佝偻病的症状多发生于1岁以后，因而2～3岁后仍有活动性佝偻病表现；血钙多正常，血磷明显降低，尿磷增加。对用一般治疗剂量维生素D治疗佝偻病无效时应与本病鉴别。

（2）远端肾小管性酸中毒：为远曲小管泌氢不足，从尿中丢失大量钠、钾、钙，继发甲状旁腺功能亢进，骨质脱钙，出现佝偻病体征。患儿骨骼畸形显著，身材矮小，有代谢性酸中毒，多尿，碱性尿，除低血钙、低血磷之外，血钾亦低，血氨增高，并常有低血钾症状。

（3）维生素D依赖性佝偻病：为常染色体隐性遗传，可分二型：Ⅰ型为肾脏1-羟化酶缺陷，使25-(OH)D_3转变为1,25-(OH)D_3发生障碍，血中25-(OH)D_3浓度正常；Ⅱ型为靶器官1,25-(OH)D_3

受体缺陷，血中$1,25\text{-}(OH)D_3$浓度增高。两型临床均有严重的佝偻病体征，低钙血症、低磷血症，碱性磷酸酶明显升高及继发性甲状旁腺功能亢进，Ⅰ型患儿可有高氨基酸尿症；Ⅱ型患儿的一个重要特征为脱发。

（4）肾性佝偻病：由于先天或后天原因所致的慢性肾功能障碍，导致钙磷代谢紊乱，血钙低，血磷高，甲状旁腺继发性功能亢进，骨质普遍脱钙，骨骼呈佝偻病改变。多于幼儿后期症状逐渐明显，形成侏儒状态。

（5）肝性佝偻病：肝功能不良可能使$25\text{-}(OH)D_3$生成障碍。若伴有胆道阻塞，不仅影响维生素D吸收，而且由于钙皂形成，进一步抑制钙的吸收。急性肝炎、先天性肝外胆管缺乏或其它肝脏疾病时，循环中$25\text{-}(OH)D_3$可明显降低，出现低血钙性、抽搐和佝偻病的体征。

各型佝偻病（活动期）的实验室检查见表5-7。

表5-7　各型佝偻病（活动期）的实验室检查

病名	血清						氨基酸尿	其他
	钙	磷	碱性磷酸酶	$25\text{-}(OH)D_3$	$1,25\text{-}(OH)D_3$	甲状旁腺素		
维生素D缺乏性佝偻病	正常(↓)	↓	↑	↓	↓	↑	(-)	尿磷↑
家族性低磷血症	正常	↓	↑	正常(↑)	正常(↓)	正常	(-)	尿磷↑
远瑞肾小管性酸中毒	正常(↓)	↓	↑	正常(↑)	正常(↓)	正常(↑)	(-)	碱性尿、高血氯、低血钾
维生素D依赖性佝偻病								
Ⅰ型	↓	↓	↑	↑	↓	↑	(+)	
Ⅱ型	↓	↓	↑	正常	↑	↑	(+)	
肾性佝偻病	↓	↑	正常	正常	↓	↑	(-)	等渗尿、氮质血症酸中毒

【治疗】

目的在于控制活动期，防止骨骼畸形。治疗的原则应以口服为主，一般剂量为每日50μg~100μg（2000IU～4000IU），或$1,25\text{-}(OH)D_3$0.5μg～2.0μg，一月后改预防量400IU/日。大剂量维生素D与治疗效果无正比例关系，不缩短疗程，与临床分期无关；且采用大剂量治疗佝偻病的方法缺乏可靠的指标来评价血中维生素D代谢产物浓度、维生素D的毒性、高血钙症的发生以及远期后果。因此大剂量治疗应有严格的适应症。当重症佝偻病有并发症或无法口服者可大剂量肌肉注射维生素D20万IU～30万IU一次，3个月后改预防量。治疗1个月后应复查，如临床表现、血生化与骨骼X线改变无恢复征象，应与抗维生素D佝偻病鉴别。

除采用维生素D治疗外，应注意加强营养，保证足够奶量，及时添加转乳期食品，坚持每日户外活动。

【预防】

营养性维生素D缺乏性佝偻病是自限性疾病，一旦婴幼儿有足够时间户外活动，可以自愈。有研究证实日光照射和生理剂量的维生素D（400IU）可治疗佝偻病。因此，现认为确保儿童每日获得维生素D400IU是预防和治疗的关键。

1．围生期　孕母应多户外活动，食用富含钙、磷、维生素D以及其他营养素的食物。妊娠

后期适量补充维生素D（800IU/日）有益于胎儿贮存充足维生素D，以满足生后一段时间生长发育的需要。

2．婴幼儿期　预防的关键在日光浴与适量维生素D的补充。出生1个月后可让婴儿逐渐坚持户外活动，冬季也要注意保证每日1～2小时户外活动时间。有研究显示，每周让母乳喂养的婴儿户外活动2小时，仅暴露面部和手部，可维持婴儿血25-(OH)D_3浓度在正常范围的低值（>11ng／dl）。

早产儿、低出生体重儿、双胎儿生后1周开始补充维生素D800IU/日，3个月后改预防量；足月儿生后2周开始补充维生素D400IU/日，均补充至2岁。夏季阳光充足，可在上午和傍晚户外活动，暂停或减量服用维生素D。

一般可不加服钙剂，但乳类摄入不足和营养欠佳时可适当补充微量营养素和钙剂。

（二）维生素D缺乏性手足搐搦症

维生素D缺乏性手足搐搦症（tetany of vitamin D deficiency）是维生素D缺乏性佝偻病的伴发症状之一，多见6个月以内的小婴儿。目前因预防维生素D缺乏工作的普遍开展，维生素D缺乏性手足搐搦症已较少发生。

【病因和发病机理】

维生素D缺乏时，血钙下降而甲状旁腺不能代偿性分泌物增加；血钙继续降低，当总血钙低于1.75～1.8mmol/L（<7mg/dl～7.5mg/dl），或离子钙低于1.0mmol/L（4mg/dl）时可引起神经肌肉兴奋性增高，出现抽搐见图5-2。为什么维生素D缺乏时机体出现甲状旁腺功能低下的原因尚不清楚，推测当婴儿体内钙营养状况较差时，维生素D缺乏的早期甲状旁腺急剧代偿分泌增加，以维持血钙正常；当维生素D继续缺乏，甲状旁腺功能反应过度而疲惫，以致出现血钙降低。因此维生素D缺乏性手足搐搦症的患儿，同时存在甲状旁腺功能亢进所产生的佝偻病的表现和甲状旁腺功能低下的低血钙所致的临床表现。

【临床表现】

主要为惊厥、喉痉挛和手足搐搦，并有程度不等的活动期佝偻病的表现。

1．隐匿型　血清钙多在1.75～1.88mmol/L，没有典型发作的症状，但可通过刺激神经肌肉而引出下列体征。①面神经征（Chvostek sign）：以手指尖或叩诊锤骤击患儿颧弓与口角间的面颊部（第7脑神经孔处），引起眼睑和口角抽动为面神经征阳性，新生儿期可呈假阳性；②腓反射（peroneal reflex）：以叩诊锤骤击膝下外侧腓骨小头上腓神经处，引起足向外侧收缩者即为腓反射阳性；③陶瑟征（Trousseau sign）：以血压计袖带包裹上臂，使血压维持在收缩压与舒张压之间，5分钟之内该手出现痉挛症状属陶瑟征阳性。

2．典型发作　血清钙低于1.75mmol/L时可出现惊厥、喉痉挛和手足搐搦。①惊厥：突然发生四肢抽动，两眼上窜，面肌颤动，神志不清，发作时间可短至数秒钟，或长达数分钟以上，发作时间长者可伴口周发绀。发作停止后，意识恢复，精神萎靡而入睡，醒后活泼如常，发作次数可数日1次或1日数次，甚至多至1日数十次。一般不发热，发作轻时仅有短暂的眼球上窜和面肌抽动，神志清楚；②手足搐搦：可见于较大婴儿、幼儿，突发手足痉挛呈弓状，双手呈腕部屈曲状，手指伸直，拇指内收掌心，强直痉挛；足部踝关节伸直，足趾同时向下弯曲。③喉痉挛：婴儿见多，喉部肌肉及声门突发痉挛，呼吸困难，有时可突然发生窒息，严重缺氧甚至死亡。三种症状以无热惊厥为最常见。

【诊断与鉴别诊断】

突发无热惊厥，且反复发作，发作后神志清醒无神经系统体征，同时有佝偻病存在，总血钙低于1.75mmol/L，离子钙低于1.0mmol/L。应与下列疾病鉴别：

1．其他无热惊厥性疾病

（1）低血糖症：常发生于清晨空腹时，有进食不足或腹泻史，重症病例惊厥后转入昏迷，一般口服或静脉注射葡萄液后立即恢复，血糖常低于2.2mmol/L。

（2）低镁血症：常见于新生儿或年幼婴儿，常有触觉、听觉过敏，引起肌肉颤动，甚至惊厥、手足搐搦，血镁常低于0.58mmol/L（1.4mg/dl）。

（3）婴儿痉挛症：为癫痫的一种表现。起病于1岁以内，呈突然发作，头及躯干、上肢均屈曲，手握拳，下肢弯曲至腹部，呈点头哈腰状抽搦和意识障碍，发作数秒至数十秒自停，伴智能异常，脑电图有特征性的高辐异常节律波出现。

（4）原发性甲状旁腺功能减退：表现为间歇性惊厥或手足搐搦，间隔几天或数周发作1次，血磷升高>3.2mmol/L（10mg/d），血钙降至1.75mmol/L（7mg/dl）以下，碱性磷酸酶正常或稍低，颅骨X线可见基底核钙化灶。

2．中枢神经系统感染　脑膜炎、脑炎、脑脓肿等大多伴有发热和感染中毒症状，精神萎靡，食欲差等。体弱年幼儿反应差，有时可不发热。有颅内压增高体征及脑脊液改变。

3．急性喉炎 大多伴有上呼吸道感染症状，也可突然发作，声音嘶哑伴犬吠样咳嗽及吸气困难，无低血钙症状，钙剂治疗无效。

【治疗】

1．急救处理

（1）氧气吸入：惊厥期应立即吸氧，喉痉挛者须立即将舌头拉出口外，并进行口对口呼吸或加压给氧，必要对作气管插管以保证呼吸道通畅。

（2）迅速控制惊厥或喉痉挛：可用10％水合氯醛，每次40～50mg/kg，保留灌肠；或地西泮每次0.1～0.3mg/kg肌肉或静脉注射。

2．钙剂治疗　尽快给予10％葡萄糖酸钙5～l0ml加入10％葡萄糖液5～20ml，缓慢静脉注射或滴注，迅速提高血钙浓度，惊厥停止后口服钙剂，不可皮下或肌肉注射钙剂以免造成局部坏死。

3．维生素D治疗　急诊情况控制后，按维生素D缺乏性佝偻病补充维生素D治疗。

【附】　维生素D中毒

近年来屡有因维生素D摄入过量引起中毒的报道，应引起儿科医师的重视。维生素D中毒多因以下原因所致：（1）短期内多次给以大剂量维生素D治疗佝偻病；（2）预防量过大，每日摄入维生素D过多，或大剂量维生素D数月内反复肌注；（3）误将其他骨骼代谢性疾病或内分泌疾病诊为佝偻病而长期大剂量摄入维生素D。维生素D中毒剂量的个体差异大。一般小儿每日服用500～1250μg（2万～5万IU），或每日50μg /kg（2000IU/kg），连续数周或数月即可发生中毒。敏感小儿每日100μg（4000IU），连续1～3月即可中毒。

【机制】

当机体大量摄入维生素D，使体内维生素D反馈作用失调，血清1,25-$(OH)_2D_3$的浓度增加，肠吸收钙与磷增加，血钙浓度过高，降钙素（CT）调节使血钙沉积于骨与其他器官组织，影响其功能。如钙盐沉积于肾脏可产生肾小管坏死和肾钙化，严重时可发生肾萎缩、慢性肾功能损害；钙盐沉积于小支气管与肺泡，损坏呼吸道上皮细胞引起溃疡或钙化灶；如在中枢神经系统、心血管等重要器官组织出现较多钙化灶，可产生不可逆的严重损害。

【临床表现】

早期症状为厌食、恶心、倦怠、烦躁不安、低热、呕吐、顽固性便秘，体重下降。重症可出现惊厥、血压升高、心律不齐、烦渴、尿频、夜尿、甚至脱水、酸中毒；尿中出现蛋白质、

红细胞、管型等改变，继而发生慢性肾衰竭。

【诊断】

有维生素D过量的病史。因早期症状无特异性，且与早期佝偻病的症状有重叠，如烦躁不安、多汗等，应仔细询问病史加以鉴别。

早期血钙升高>3mmol/L（12mg/d1），尿钙强阳性（Sulkowitch反应），尿常规检查示尿蛋白阳性，严重时可见红细胞、白细胞、管型。X线检查可见长骨干骺端钙化带增宽（>1mm），致密，骨干皮质增厚，骨质疏松或骨硬化；颅骨增厚，呈现环形密度增深带；重症时大脑、心、肾、大血管、皮肤有钙化灶。可出现氮质血症、脱水和电解质紊乱。肾脏B超示肾萎缩。

【治疗】

疑维生素D过量中毒即应停服维生素D，如血钙过高应限制钙的摄入，包括减少富含钙的食物摄入。加速钙的排泄，口服氢氧化铝或依地酸二钠减少肠钙的吸收，使钙从肠道排出；口服泼尼松抑制肠内钙结合蛋白的生成而降低肠钙的吸收；亦可试用降钙素。注意保持水、电解质的平衡。

（李廷玉）

第六节 蛋白质-能量营养障碍

一、蛋白质-能量营养不良

蛋白质-能量营养不良（proteln-energy malnutrition，PEM）是由于缺乏能量和（或）蛋白质所致的一种营养缺乏症，主要见于3岁以下婴幼儿。临床上以体重明显减轻、皮下脂肪减少和皮下水肿为特征，常伴有各器官系统的功能紊乱。急性发病者常伴有水、电解质紊乱，慢性者常有多种营养素缺乏。临床常见三种类型：能量供应不足为主的消瘦型；以蛋白质供应不足为主的浮肿型以及介于两者之间的消瘦-浮肿型。

【病因】

1．摄入不足　小儿处于生长发育的阶段，对营养素尤其是蛋白质的需要相对较多，喂养不当是导致营养不良的重要原因，如：母乳不足而未及时添加其他富含蛋白质的食品；奶粉配制过稀；突然停奶而未及时添加辅食；长期以淀粉类食品（粥、米粉、奶糕）喂养等。较大儿童的营养不良多为婴儿期营养不良的继续，或因不良的饮食习惯如偏食、挑食、吃零食过多、不吃早餐等引起。

2．消化吸收不良　消化吸收障碍，如消化系统解剖或功能上的异常（包括唇裂、腭裂、幽门梗阻等）、迁延性腹泻、过敏性肠炎、肠吸收不良综合征等均可影响食物的消化和吸收。

3．需要量增加　急、慢性传染病（如麻疹、伤寒、肝炎、结核）的恢复期、生长发育快速阶段等均可因需要量增多而造成营养相对缺乏；糖尿病、大量蛋白尿、发热性疾病、甲状腺功能亢进、恶性肿瘤等均可使营养素的消耗量增多而导致营养不足。先天不足和生理功能低下如早产、双胎因追赶生长而需要量增加可引起营养不良。

【病理生理】

1．新陈代谢异常

（1）蛋白质：由于蛋白质摄入不足或蛋白质丢失过多，使体内蛋白质代谢处于负平衡。当血清总蛋白浓度<40g/L、白蛋白<20g/L时，便可发生低蛋白性水肿。

（2）脂肪：能量摄入不足时，体内脂肪大量消耗以维持生命活动的需要，故血清胆固醇浓度下降。肝脏是脂肪代谢的主要器官，当体内脂肪消耗过多，超过肝脏的代谢能力时可造成

肝脏脂肪浸润及变性。

（3）糖类：由于摄入不足和消耗增多，故糖原不足和血糖偏低，轻度时症状并不明显，重者可引起低血糖昏迷甚至猝死。

（4）水、盐代谢：由于脂肪大量消耗，故细胞外液容量增加，低蛋白血症可进一步加剧而呈现浮肿；PEM时ATP合成减少可影响细胞膜上钠-钾-ATP酶的运转，钠在细胞内潴留，细胞外液一般为低渗状态，易出现低渗性脱水、酸中毒、低血钾、低血钠、低血钙和低镁血症。

（5）体温调节能力下降：营养不良儿体温偏低，可能与热能摄入不足；皮下脂肪菲薄，散热快；血糖降低；氧耗量低、脉率和周围血循环量减少等有关。

2．各系统功能低下

（1）消化系统：由于消化液和酶的分泌减少、酶活力降低，肠蠕动减弱，菌群失调，致消化功能低下，易发生腹泻。

（2）循环系统：心脏收缩力减弱，心排出量减少，血压偏低，脉细弱。

（3）泌尿系统：肾小管重吸收功能减低，尿量增多而尿比重下降。

（4）神经系统：精神抑郁但时有烦躁不安、表情淡漠、反应迟钝、记忆力减退、条件反射不易建立。

（5）免疫功能：非特异性（如皮肤黏膜屏障功能、白细胞吞噬功能、补体功能）和特异性免疫功能均明显降低。患儿结核菌素等迟发性皮肤反应可呈阴性，常伴IgG亚类缺陷和T细胞亚群比例失调等。由于免疫功能全面低下，患儿极易并发各种感染。

【临床表现】

体重不增是营养不良的早期表现。随营养失调日久加重，体重逐渐下降，患儿主要表现为消瘦，皮下脂肪逐渐减少以至消失，皮肤干燥、苍白、皮肤逐渐失去弹性、额部出现皱纹如老人状、肌张力逐渐降低、肌肉松弛、肌肉萎缩呈“皮包骨”时、四肢可有挛缩。皮下脂肪层消耗的顺序首先是腹部，其次为躯干、臀部、四肢、最后为面颊。皮下脂肪层厚度是判断营养不良程度的重要指标之一。营养不良初期，身高并无影响，但随着病情加重，骨骼生长减慢，身高亦低于正常。轻度营养不良，精神状态正常，但重度可有精神萎靡，反应差，体温偏低，脉细无力，无食欲，腹泻、便秘交替。合并血浆白蛋白明显下降时，可有凹陷性浮肿、皮肤发亮，严重时可破溃、感染形成慢性溃疡。重度营养不良可有重要脏器功能损害，如心脏功能下降，可有心音低钝、血压偏低、脉搏变缓、呼吸浅表等。

常见的并发症有营养性贫血，以小细胞低色素性贫血最为常见，贫血与缺乏铁、叶酸、维生素B_{12}、蛋白质等造血原料有关。营养不良可有多种维生素缺乏，尤以脂溶性维生素A、D 缺乏常见。在营养不良时，维生素D缺乏的症状不明显，在恢复期生长发育加快时症状比较突出。约有3/4的病儿伴有锌缺乏，由于免疫功能低下，故易患各种感染，如反复呼吸道感染、鹅口疮、肺炎、结核病、中耳炎、尿路感染等；婴儿腹泻常迁延不愈加重营养不良，形成恶性循环。

营养不良可并发自发性低血糖，患儿可突然表现为面色灰白、神志不清、脉搏减慢、呼吸暂停、体温不升，但一般无抽搐，若不及时诊治，可致死亡。

【实验室检查】

血清白蛋白浓度降低是最重要的改变，但其半衰期较长（19～21天）故不够灵敏。视黄醇结合蛋白（半衰期10小时）、前白蛋白（半衰期1.9天），甲状腺结合前白蛋白（半衰期2天）和转铁蛋白（半衰期3天）等代谢周期较短的血浆蛋白质具有早期诊断价值。胰岛素样生长因子1（IGF1）不仅反应灵敏且受其他因素影响较小，是诊断蛋白质营养不良的较好指标。营养不良小儿牛磺酸和必需氨基酸浓度降低，而非必需氨基酸变化不大；血清淀粉酶、脂肪酶、胆碱酯

酶、转氨酶、碱性磷酸酶、胰酶和黄嘌呤氧化酶等活力均下降，经治疗后可迅速恢复正常；胆固醇，各种电解质及微量元素浓度皆可下降；生长激素水平升高。

【诊断】

根据小儿年龄及喂养史，有体重下降、皮下脂肪减少、全身各系统功能紊乱及其他营养素缺乏的临床症状和体征，典型病例的诊断并不困难。轻度患儿易被忽略，需通过定期生长监测、随访才能发现。确诊后还需详细询问病史和进一步检查，以确定病因。诊断营养不良的基本测量指标为身长和体重。5岁以下营养不良的体格测量指标的分型和分度如下。

1．体重低下（underweight）　体重低于同年龄、同性别参照人群值的均值减2SD以下为体重低下。如低于同年龄、同性别参照人群值的均值减2SD～3SD为中度；在均值减3SD以下为重度。该项指标主要反映慢性或急性营养不良。

2．生长迟缓（stunting）　其身长低于同年龄、同性别参照人群值的均值减2SD为生长迟缓。如低于同年龄、同性别参照人群均值减2SD～3SD为中度；低于均值减3SD以下为重度。此指标主要反映慢性长期营养不良。

3．消瘦（wasting）　体重低于同性别、同身高参照人群值的均值减2SD为消瘦。如低于同性别、同身高参照人群值的均值减2SD～3SD为中度；低于均值减3SD为重度。此项指标主要反映近期、急性营养不良。

临床常综合应用以上指标来判断患儿营养不良的类型和严重程度。以上三项判断营养不良的指标可以同时存在，也可仅符合其中一项。符合一项即可进行营养不良的诊断。

【治疗】

营养不良的治疗原则是积极处理各种危及生命的合并症、祛除病因、调整饮食、促进消化功能。

1．处理危及生命的并发症　严重营养不良常发生危及生命的并发症，如腹泻时的严重脱水和电解质紊乱、酸中毒、休克、肾功能衰竭、自发性低血糖、继发感染及维生素A缺乏所致的眼部损害等。有真菌感染的患儿，除积极给予支持治疗外，要及时进行抗真菌治疗及其他相应的处理。

2．祛除病因　在查明病因的基础上，积极治疗原发病，如纠正消化道畸形，控制感染性疾病、根治各种消耗性疾病、改进喂养方法等。

3．调整饮食　营养不良患儿的消化道因长期摄入过少，已适应低营养的摄入，过快增加摄食量易出现消化不良、腹泻，故饮食调整的量和内容应根据实际的消化能力和病情逐步完成，不能操之过急。轻度营养不良可从每日250～330kJ/kg（60～80kcal/kg）开始，中、重度可参考原来的饮食情况，从每日165～230kJ/kg（40～55kcal/kg）开始，逐步少量增加；若消化吸收能力较好，可逐渐加到每日500～727kJ/kg（120～170kcal/kg），并按实际体重计算热能需要。母乳喂养儿可根据患儿的食欲哺乳，按需哺喂；人工喂养儿从给予稀释奶开始，适应后逐渐增加奶量和浓度。除乳制品外，可给予蛋类、肝泥、肉末、鱼粉等高蛋白食物，必要时也可添加酪蛋白水解物、氨基酸混合液或要素饮食。蛋白质摄入量从每日1.5～2.0g/kg开始，逐步增加到3.0～4.5g/kg，过早给予高蛋白食物可引起腹胀和肝肿大。食物中应含有丰富的维生素和微量元素。

4．促进消化　其目的是改善消化功能。

（1）药物：可给予B族维生素和胃蛋白酶、胰酶等以助消化。蛋白质同化类固醇制剂如苯丙酸诺龙能促进蛋白质合成，并能增加食欲，每次肌注10～25mg，每周1～2次，连续2～3周，用药期间应供给充足的热量和蛋白质。对食欲差的患儿可给予胰岛素注射，降低血糖，增加饥

饿感以提高食欲，通常每日一次皮下注射正规胰岛素2～3U，注射前先服葡萄糖20～30g，每1～2周为一疗程。锌制剂可提高味觉敏感度，有增加食欲的作用，每日可口服元素锌0.5～lmg/kg。

（2）中医治疗：中药参苓白术散能调整脾胃功能，改善食欲；针灸、推拿、抚触、捏脊等也有一定疗效。

5．其他　病情严重、伴明显低蛋白血症或严重贫血者，可考虑成分输血。静脉点滴高能量脂肪乳剂、多种氨基酸、葡萄糖等也可酌情选用。此外，充足的睡眠、适当的户外活动、纠正不良的饮食习惯和良好的护理亦极为重要。

【预后和预防】

预后取决于营养不良的发生年龄、持续时间及其程度，其中尤以发病年龄最为重要，年龄愈小，其远期影响愈大，尤其是认知能力和抽象思维能力易发生缺陷。本病的预防应采取综合措施。

1．合理喂养　大力提倡母乳喂养，对母乳不足或不宜母乳喂养者应及时给予指导，采用混合喂养或人工喂养并及时添加辅助食品；纠正偏食、挑食、吃零食的不良习惯，小学生早餐要吃饱，午餐应保证供给足够的能量和蛋白质。

2．合理安排生活作息制度　坚持户外活动，保证充足睡眠，纠正不良的卫生习惯。

3．防治传染病和先天畸形　按时进行预防接种；对患有唇裂、腭裂及幽门狭窄等先天畸形者应及时手术治疗。

4．推广应用生长发育监测图　定期测量体重，并将体重值标在生长发育监测图上，如发现体重增长缓慢或不增，应尽快查明原因，及时予以纠正。

二、小儿单纯性肥胖

小儿单纯性肥胖（obesity）是由于长期能量摄入超过人体的消耗，使体内脂肪过度积聚、体重超过一定范围的一种营养障碍性疾病。体重超过同性别、同身高参照人群均值的20％即可称为肥胖。小儿单纯性肥胖症在我国呈逐步增多的趋势，目前约占5％～8％。肥胖不仅影响儿童的健康，且儿童期肥胖可延续至成人，容易引起高血压、糖尿病、冠心病、胆石症、痛风等疾病，对本病的防治应引起社会及家庭的重视。

【病因】

单纯性肥胖占肥胖的95％～97％，不伴有明显的内分泌和代谢性疾病。

1．能量摄入过多　摄入的营养超过机体代谢需要，多余的能量便转化为脂肪贮存体内、导致肥胖。

2．活动量过少　活动过少和缺乏适当的体育锻炼是发生肥胖症的重要因素，即使摄食不多，也可引起肥胖。肥胖儿童大多不喜爱运动，形成恶性循环。

3．遗传因素　肥胖有高度的遗传性，目前认为肥胖的家族性与多基因遗传有关。肥胖双亲的后代发生肥胖者高达70％～80％；双亲之一肥胖者，后代肥胖发生率约为40％～50％；双亲正常的后代发生肥胖者仅10％～14％。

4．其他　如进食过快，或饱食中枢和饥饿中枢调节失衡以致多食；精神创伤（如亲人病故或学习成绩低下）以及心理异常等因素亦可致儿童过量进食。

【病理生理】

引起肥胖的原因为脂肪细胞数目增多或体积增大。人体脂肪细胞数量的增多主要在出生前3个月、生后第1年和11～13岁三个阶段，若肥胖发生在这三个时期，即可引起脂肪细胞数目增多性肥胖，治疗较困难且易复发；而不在此脂肪细胞增殖时期发生的肥胖，脂肪细胞体积增大而数目正常，治疗较易奏效。肥胖患儿可有下列代谢及内分泌改变。

1．体温调节与能量代谢　肥胖儿对外界体温的变化反应较不敏感，用于产热的能量消耗较正常儿少，使肥胖儿有低体温倾向。

2．脂类代谢　肥胖儿常伴有血浆甘油三酯、胆固醇、极低密度脂蛋白（VLDL）及游离脂肪酸增加，但高密度脂蛋白（HDL）减少。故以后易并发动脉硬化、冠心病、高血压、胆石症等疾病。

3．蛋白质代谢　肥胖者嘌呤代谢异常，血尿酸水平增高，易发生痛风症。

4．内分泌变化　内分泌变化在肥胖小儿较常见。

（1）甲状腺功能的变化：总T_4、游离T_4、总T_3、游离T_3、反T_3、蛋白结合碘、吸^{131}碘率等均正常，下丘脑-垂体-甲状腺轴也正常，但发现T_3受体减少，被认为是产热减少的原因。

（2）甲状旁腺激素及维生素D代谢：肥胖儿血清PTH水平升高，25-(OH)D_3及24，25-$(OH)_2D_3$水平也增高，可能与肥胖的骨质病变有关。

（3）生长激素水平的变化：肥胖儿血浆生长激素减少；睡眠时生长激素分泌高峰消失；在低血糖或精氨酸刺激下，生长激素分泌反应迟钝。但肥胖儿IGFl分泌正常，胰岛素分泌增加，对生长激素的减少起到了代偿作用，故患儿无明显生长发育障碍。

（4）性激素的变化：女性肥胖患者雌激素水平增高，可有月经不调和不孕；男性患者因体内脂肪将雄激素芳香化转变为雌激素，雌激素水平增高，可有轻度性功能低下、阳痿，但不影响睾丸发育和精子形成。

（5）糖皮质激素：肥胖患儿尿17-羟类固醇、17-酮类固醇及皮质醇均可增加，但血浆皮质醇正常或轻度增加，昼夜规律存在。

（6）胰岛素与糖代谢的变化：肥胖者有高胰岛素血症的同时又存在胰岛素抵抗，致糖代谢异常，可出现糖耐量减低或糖尿病。

【临床表现】

肥胖可发生于任何年龄，但最常见于婴儿期、5～6岁和青春期。患儿食欲旺盛且喜吃甜食和高脂肪食物。明显肥胖儿童常有疲劳感，用力时气短或腿痛。严重肥胖者由于脂肪的过度堆积限制了胸廓和膈肌运动，使肺通气量不足、呼吸浅快，故肺泡换气量减少，造成低氧血症、气急、紫绀、红细胞增多、心脏扩大或出现充血性心力衰竭甚至死亡，称肥胖-换氧不良综合征（Pickwickian syndrome）。

体格检查可见患儿皮下脂肪丰满，但分布均匀，腹部膨隆下垂，严重肥胖者可因皮下脂肪过多，使胸腹、臀部及大腿皮肤出现皮纹；因体重过重，走路时两下肢负荷过重可致膝外翻和扁平足。女孩胸部脂肪堆积应与乳房发育相鉴别，后者可触到乳腺组织硬结。男性肥胖儿因大腿内侧和会阴部脂肪堆积，阴茎可隐匿在阴阜脂肪垫中而被误诊为阴茎发育不良。

肥胖小儿性发育常较早，故最终身高常略低于正常小儿。由于怕被别人讥笑而不愿与其他小儿交往，故常有心理上的障碍，如自卑、胆怯、孤独等。

【实验室检查】

肥胖儿甘油三酯、胆固醇大多增高，严重患者血清β白蛋白也增高；常有高胰岛素血症，血生长激素水平减低，生长激素刺激试验的峰值也较正常小儿为低。肝脏超声波检查常有脂肪肝。

【诊断】

小儿体重为同性别、同身高参照人群均值10％～19％者为超重；超过20％以上者便可诊断为肥胖症；20％～29％者为轻度肥胖；30％～49％者为中度肥胖；超过50％者为重度肥胖。确诊时须与可引起继发性肥胖的疾病鉴别。

体质指数（body mass index，BMI）是评价肥胖的另一种指标。BMI 是指体重（kg）/身长的平方（m^2），小儿BMI随年龄性别而有差异，评价时可查阅图表，如BMI值在P_{85}～P_{95}为超重，超过P_{95}为肥胖。

【鉴别诊断】

1．伴肥胖的遗传性疾病

（1）Prader-Willi综合征：呈周围型肥胖体态、身材矮小、智能低下、手脚小、肌张力低、外生殖器发育不良。本病可能与位于15q12的SNRPN基因缺陷有关。

（2）Laurence-Moon-Biedl综合征：周围型肥胖、智能轻度低下、视网膜色素沉着、多指趾、性功能减低。

（3）Alstrom综合征：中央型肥胖、视网膜色素变性、失明、神经性耳聋、糖尿病。

2．伴肥胖的内分泌疾病

（1）肥胖生殖无能症（Fröhlich syndrome）：本症继发于下丘脑及垂体病变，其体脂主要分布在颈、颏下、乳房、下肢、会阴及臀部，手指、足趾显得纤细、身材矮小，第二性征延迟或不出现。

（2）其他内分泌疾病：如肾上腺皮质增生症、甲状腺功能减低症、生长激素缺乏症等虽有皮脂增多的表现，但均各有其特点，故不难鉴别。

【治疗】

肥胖症的治疗原则是减少产热能性食物的摄入和增加机体对热能的消耗，使体内脂肪不断减少，体重逐步下降。饮食疗法和运动疗法是两项最主要的措施，药物治疗效果不很肯定，外科手术治疗的并发症严重，不宜用于小儿。

1．饮食疗法　鉴于小儿正处于生长发育阶段以及肥胖治疗的长期性，故多推荐低脂肪、低糖类和高蛋白食谱。低脂饮食可迫使机体消耗自身的脂肪储备，但也会使蛋白质分解，故需同时供应优质蛋白质。糖类分解成葡萄糖后会强烈刺激胰岛素分泌，从而促进脂肪合成，故必须适量限制。食物的体积在一定程度上会使患儿产生饱腹感，故应鼓励其多吃体积大而热能低的蔬菜类食品，其纤维还可减少糖类的吸收和胰岛素的分泌，并能阻止胆盐的肠肝循环，促进胆固醇排泄，且有一定的通便作用。萝卜、胡萝卜、青菜、黄瓜、番茄、莴苣、苹果、柑橘、竹笋等均可选择。

良好的饮食习惯对减肥具有重要作用，如避免晚餐过饱，不吃夜宵，不吃零食，少吃多餐，减慢进食速度、细嚼慢咽等。平时不要让患儿看到美味食品，以免引起食欲中枢兴奋。

2．运动疗法　适当的运动能促使脂肪分解，减少胰岛素分泌，使脂肪合成减少，蛋白质合成增加，促进肌肉发育。肥胖小儿常因动作笨拙和活动后易累而不愿锻炼，可鼓励和选择患儿喜欢和有效易于坚持的运动，如晨间跑步、散步、做操等，每天坚持至少运动30分钟，活动量以运动后轻松愉快、不感到疲劳为原则。运动要循序渐进，不要求之过急。如果运动后疲惫不堪，心慌气促以及食欲大增均提示活动过度。

3．药物治疗　苯丙胺类和马吲哚类等食欲抑制剂以及甲状腺素等增加消耗类药物对儿童均应慎用。

【预防】

孕妇在妊娠后期要适当减少摄入脂肪类食物，防止胎儿体重增加过重；要宣传肥胖儿不是健康儿的观点，使家长摒弃“越胖越健康”的陈旧观念；父母肥胖者更应定期监测小儿体重，以免小儿发生肥胖症。

（毛　萌）

第七节 微量元素障碍

人类必需微量元素缺乏包括铁、碘、氟、锌、铬、硒、镁、钼和铜等，除铁外，锌和碘缺乏也是儿童时期较为常见的疾病。

一、锌缺乏

锌为人体必需微量元素之一，主要存在于骨、牙齿、毛发、皮肤、肝脏和肌肉中，为100多种酶的关键组成成分，参与DNA、RNA和蛋白质的合成。儿童缺锌的主要表现为食欲不振、生长发育减慢、免疫机能低下、味觉减退和夜盲；青春期缺锌可致性成熟障碍。

【病因】

1．摄入不足　动物性食物不仅含锌丰富而且易于吸收，坚果类（核桃、板栗、花生等）含锌也不低，其他植物性食物则含锌少，故素食者容易缺锌。全胃肠道外营养如未加锌也可致锌缺乏。

2．吸收障碍　各种原因所致的腹泻皆可妨碍锌的吸收。谷类食物中含大量植酸和粗纤维，这些均可与锌结合而妨碍其吸收。牛乳含锌量与母乳相似，约45.9～53.5μmol/L（300～350μg/dl），但牛乳锌的吸收率（39％）远低于母乳锌（65％），故长期纯牛乳喂养也可致缺锌。肠病性肢端皮炎（acrodermatitis enteropathica）是一种常染色体隐性遗传病，因小肠缺乏吸收锌的载体，故可表现为严重缺锌。

3．需要量增加　在生长发育迅速阶段的婴儿，或组织修复过程中、或营养不良恢复期等状态下，机体对锌需要量增多，如未及时补充，可发生锌缺乏。

4．丢失过多　如反复出血、溶血、大面积灼伤、慢性肾脏疾病、长期透析、蛋白尿以及应用金属螯合剂（如青霉胺）等均可因锌丢失过多而导致锌缺乏。

【临床表现】

正常人体含锌2～2.5g，缺锌可影响核酸和蛋白质的合成及其他生理功能。

1．消化功能减退　缺锌影响味蕾细胞更新和唾液磷酸酶的活性，使舌黏膜增生、角化不全，以致味觉敏感度下降，发生食欲不振、厌食、异嗜癖。

2．生长发青落后　缺锌可妨碍生长激素轴功能以及性腺轴的成熟，表现为生长发育迟缓、体格矮小、性发育延迟和性腺功能减退。

3．免疫机能降低　缺锌可导致T淋巴细胞功能损伤而容易发生感染。

4．智能发育延迟　缺锌可使脑DNA和蛋白质合成障碍，脑内谷氨酸浓度降低，从而引起智能迟缓。

5．其他　如脱发、皮肤粗糙、皮炎、地图舌、反复口腔溃疡、伤口愈合延迟、视黄醛结合蛋白减少而出现夜盲、贫血等。

【实验室检查】

1．血清锌测定 正常最低值为11.47μmol/L（75μg /dl）。

2．餐后血清锌浓度反应试验（PICR）　测空腹血清锌浓度（A_0）作为基础水平，然后给予标准饮食（按全天总热量的20％计算，其中蛋白质为10％～15％，脂肪为30％～35％，糖类为50％～60％），2小时后复查血清锌（A_2），按公式PICR＝（A_0－A_2）/A_0×100%计算，若PICR>15％提示缺锌。

3．发锌测定　不同部位的头发和不同的洗涤方法均可影响测定结果，轻度缺锌时发锌浓

度降低，严重时头发生长减慢，发锌值反而增高，故发锌不能反映近期体内的锌营养状况。

【诊断】

根据缺锌的病史和临床表现，血清锌<11.47μmol/L；PICR>15％；锌剂治疗有显效等即可诊断。

【治疗】

1．针对病因　治疗原发病。

2．饮食治疗　鼓励多进食富含锌的动物性食物如肝、鱼、瘦肉、禽蛋、牡蛎等。初乳含锌丰富。

3．补充锌剂　常用葡萄糖酸锌，每日剂量为锌元素0.5～1.0mg/kg，相当于葡萄糖酸锌3.5~7mg/kg，疗程一般为2～3个月。长期静脉输入高能量者，每日锌用量为：早产儿0.3mg/kg；足月儿～5岁0.1mg/kg；>5岁2.5~4mg/d。

锌剂的毒性较小，但剂量过大也可引起胃部不适、恶心、呕吐、腹泻等消化道刺激症状，甚至脱水和电解质紊乱。锌中毒可干扰铜代谢，引起低铜血症、贫血、中性粒细胞减少、、肝细胞中细胞色素氧化酶活力降低等中毒表现。

【预防】

元素锌每日推荐摄入量为：6个月以下1.5mg，6个月～1岁以下8mg，1～4岁以下12mg，4～7岁以下13.5mg。提倡母乳喂养。坚持平衡膳食是预防缺锌的主要措施，戒绝挑食、偏食、吃零食的习惯。对可能发生缺锌的情况如早产儿、人工喂养者、营养不良儿、长期腹泻、大面积烧伤等，均应适当补锌。

二、碘缺乏

碘为人体必需微量元素之一，体内含量约为2.5mg，主要存在于甲状腺内，是甲状腺素和三碘甲腺原氨酸合成的底物。全球约有38%的人口生活在碘缺乏地区，碘缺乏可导致碘缺乏病（Iodine Deficiency Disorders, IDD），以前我国是全球IDD流行最严重的国家之一。

【病因】

食物和饮水中缺碘是其根本原因，缺碘使甲状腺素合成障碍，影响体格生长和脑发育。

【临床表现】

缺碘的主要危害是影响脑发育，导致儿童智力损害和体格发育障碍，表现为以智能障碍为主要特征的精神-神经-甲状腺合成不足，可引起甲状腺功能低下。胎儿期缺碘可致流产、死胎、早产和先天畸形；新生儿期则表现为甲状腺功能低下；胎儿期和婴儿期严重缺碘可造成克汀病；儿童和青春期则引起地方性甲状腺肿、甲状腺功能低下、智能低下。

儿童长期轻度缺碘则可出现亚临床型甲状腺功能减低症（亚临床型克汀病），常伴有体格生长落后。

【实验室检查】

1．血清T_3、T_4、TSH测定　血清总T_3、T_4或游离T_3、T_4明显降低，而TSH增高。

2．尿碘测定　尿碘测定是判断个体或群体碘营养状况的一项简便而又有效的方法。尿碘中位数值低于100μg /L意味着碘摄入量不足，50～99μg /L为轻度缺碘，20～49μg /L为中度缺碘，<20μg /L为重度缺碘。

【诊断】

亚临床型甲状腺功能减低症的诊断标准。

1．必备条件

（1）出生、居住于低碘地方性甲状腺肿病流行区。

（2）有智能发育障碍，主要表现轻度智能迟缓。

2．辅助条件

（1）神经系统障碍主要表现为：①轻度听力障碍（电测听高频或低频异常）；②极轻度语言障碍；③精神运动发育障碍。

（2）甲状腺功能障碍主要表现有：①极轻度的体格发育障碍；②极轻度的骨龄发育落后；③甲状腺功能低下（T_3、T_4降低，TSH升高）。

具有上述必备条件，以及辅助条件中神经系统障碍或甲状腺功能低下中任何1项或1项以上，并能排除其他原因如营养不良、锌缺乏、中耳炎影响便可作出诊断。

【治疗】

1．碘剂　主要用于缺碘所引起的弥漫型重度甲状腺肿大且病程短者。复方碘溶液每日1～2滴（约含碘3.5mg），或碘化钾（钠）每日10～15mg，连服2周为1疗程，两个疗程之间停药3个月，反复治疗1年。长期大量服用碘剂应注意甲状腺机能亢进的发生。

除了过敏以外，一般人均能耐受大剂量的碘。但对缺碘并伴有结节性甲状腺肿的患者进行补碘，则有发生碘性甲状腺机能亢进症的危险，其临床表现如食欲亢进、体重减轻、肌无力、畏热等均较轻微，突眼也不明显，但如果患者原有器质性心脏病，就有一定的危险性。

2．甲状腺素制剂　参见甲状腺功能减低症。

【预防】

IDD是一种可以预防的造成智力、精神发育和脑损伤的疾病。食盐加碘是预防IDD最有效的措施，自20世纪90年代实施全民食盐加碘（USI）干预措施以来，我国现在已经为世界上碘营养适宜的国家。

所谓碘化食盐，即将可溶性碘化物按1：2万～1：5万比例加入食盐，我国碘化盐中碘添加剂为KIO3。推广碘化食盐可使广大人群、特别是小儿免受缺碘所带来的种种危害；对缺碘较重地区可定期开展碘油强化补碘。

补碘后最常见的并发症是碘性甲状腺功能亢进，故补碘宜适度。我国每日碘推荐摄入量为：4岁以下50μg，4～11岁以下为90μg，11～13岁以下为120μg，14岁以上为150μg。

（何庆南）

第六章　青春期健康与疾病

青春期（adolescence, puberty）是儿童到成人的过渡阶段，也是儿童发育过程的特殊时期。这一时期生理变化的特点是生长发育突增、第二性征开始出现到体格发育完全及性成熟。青春期前（prepuberty）的生长突增（growth spurt），发生在第二性征出现之前，可樗着青春期的开始；随着体格快速生长、第二性征出现，生殖系统寻事发育；到骨骺完全融合、身高停止生长、性发育成熟，至此青春期结束。在此年龄阶段所发生的一系列形态、生理、生化以及心理和行为的改变程度，对每一个体来说，都是一生中其他年龄阶段所不能比拟的。由于生理上很快成熟即将进入成人，但心理、行为和社会学方面的发育相对滞后，造成青春期发育过程中一些特有的问题。青春期问题是全球问题，尽管不同国家、地区和民族的社会背景、文化及生活方式等存在差异，但都具有一定的共性，应给予充分的认识和注意。

第一节　青春期发育有关问题

青春发育期，各种与生长发育有关的激素不仅保证了机体各个器官与组织的生长、发育及成熟过程的顺利进行，促进生殖器官和生殖细胞的发育与成熟，还可调节中枢神经系统与自主神经系统的功能，从而影响学习、记忆与行为等。在青春期，生长激素、促肾上腺皮质激素、促甲状腺素、促性腺素等的分泌都达到新的水平。生长激素直接作用于全身的组织细胞，可以增加细胞的体积和数量，促进个体生长。促甲状腺素分泌增加引起体内甲状腺素水平的增高，可以增进全身的代谢过程。促性腺素有两种，一种是卵泡刺激素，刺激卵巢中滤泡的发育和睾丸中精子的生成；一种是黄体生成素，促进卵巢黄体的生成和刺激睾丸中间隙细胞的功能。促肾上腺皮质激素刺激肾上腺皮质主要产生糖皮质类固醇和性激素。这些激素水平的高低主要是受下丘脑-垂体系统的调节，并直接与青春期的改变有关，同时可能导致一些青春期常见的生理或病理变化。

青春期儿童进入生殖系统和性征发育时期。由于丘脑下部-垂体-性器官的发育渐趋成熟，女孩体中雌激素的水平增高，雌激素主要来自卵巢，以雌二醇的生物活性最强。雌激素的生理功能主要是促进女性内外生殖器及乳房的发育，促进月经初潮来临。雌激素也有促进体格生长、促进骨骺愈合的作用。青春期的女性身体及内、外生殖器（即第一性征）发育极快，第一性征的发育包括卵巢增大、子宫增大、输卵管变粗、阴道长度及宽度增加等；第二性征显著，包括声调变高，乳房丰满而隆起，腋毛、阴毛出现，骨盆进一步宽大，皮下脂肪增多等；月经开始来潮。月经初潮时卵巢尚未发育完全，因此可能出现月经不规律。青春期后，卵巢功能逐步完善，月经周期也正常。月经对女性心理、情绪和身体各系统生理功能都有影响。直接促使男性性成熟的主要器官是睾丸。睾丸可分泌雄激素，其中以睾酮作用最强。睾酮的主要作用是促进蛋白质的合成，使骨骼肌肉发育，肌肉力量增加。青春期男性随着生殖器官发育，出现第二性征如毛发（阴毛、腋毛及胡须）生长、变声及出现喉结等。阴毛最先出现，其次是腋毛，然后长出胡须。喉结的突出是男性特有的第二性征。外生殖器在睾酮的作用下迅速发育，并产生了遗精。男性首次遗精年龄平均为14～16岁，比女性月经初潮平均年龄约晚2年。初期的精液里可能没有成熟的精子。首次遗精发生后体格发育渐趋缓慢，而睾丸、附睾及阴茎却在迅速发育，达到成人水平。青春期发育的有关问题常见的有：

1．青春期甲状腺肿大　甲状腺的发育在青春期达人一生中之高峰。甲状腺分泌甲状腺素，有兴奋神经、调节新陈代谢、促进生长发育的功能。青春期间，为了满足生长发育的需要，机体需要摄入充足的碘来合成甲状腺素，对碘的需求量猛增，若摄取量不足，可发生甲状腺代偿性肥大。在非缺碘地区的青春期少男少女，也有可能出现不同程度的甲状腺肿大，以女孩多见。为两侧甲状腺腺体弥漫性肿大，质地柔软，一般摸不到结节。过了青春期以后甲状腺肿大可以自行消退。防治青春期甲状腺肿大的措施主要是补碘。多吃含碘丰富的食物，如海带、海蜇皮、紫菜及各种海鱼等，食用碘盐也是补碘的一种途径。

2．痤疮（acne）　又称粉刺，是青春期常见的毛囊皮脂腺的慢性炎症性皮肤病，不影响健康，但因影响面容美观，往往使青少年十分苦恼。痤疮有多种发病因素，其发病机制目前还不十分明了。内分泌因素、皮脂的作用、毛囊内微生物是痤疮发病的主要因素。近年来有人认为本病与免疫有关。此外，遗传也是本病的发生的一个重要因素。多吃动物脂肪及糖类食物，消化不良或便秘等胃肠障碍，某些微量元素如锌缺乏，精神紧张，湿热气候等因素对痤疮病人可以有不利的影响，矿物油类的接触或碘化物、溴化物及某些其他药的内服也可加剧痤疮的恶化。痤疮的皮损主要发生于面部，也可发生在胸背上部及肩部，偶尔发生于其他部位。开始时多有黑头粉刺及油性皮脂溢出，还常有丘疹、结节、脓疱、脓肿、窦道或瘢痕。多无自觉症状，如炎症明显时，则可引起疼痛和触痛，症状时轻时重。青春期后大多数病人均能自然痊愈或症状减轻。饮食调节有助于防治痤疮，多吃富含纤维和维生素的食物，少吃动物性脂肪、甜食和刺激性食物。经常保持皮肤清洁是防治痤疮的有效措施，要常用温水或其他去脂消炎的香皂洗涤患处。不要用手抠或挤压，不要用油脂类化妆品擦脸，以免阻塞毛囊口和皮脂腺开口，症状。以感染为主的痤疮应选用抗生素，也可选用复合维生素B、维生素A、锌制剂等，或进行局部理疗，减轻皮损。

3．青春期高血压　青春期高血压的特点是收缩压升高，可达140～150mmHg（18.7～20kPa），而舒张压不高或升高不明显。平时没有什么不良感觉，仅在过度疲劳或剧烈运动时才有头晕、胸闷等症状。引起青春期高血压的主要原因是由于青春期身体各器官系统迅速发育，心脏也随着发育，心收缩力大大提高，但此时血管发育却往往落后于心脏，导致血压增高。另外，青春发育时期内分泌腺发育增强，激素分泌增多，神经系统兴奋性提高，自主神经调节功能不平衡，也会产生血压增高。青春期高血压的发生是暂时性的，过了青春期，心血管系统发育迅速趋于平衡，血压就会恢复正常。因此一般不主张过早应用降压药物，但必须通过建立良好健康的生活方式来达到使血压恢复正常的目的。养成良好的饮食习惯，少吃咸食、甜食及含脂肪高的食物，多吃新鲜蔬菜和水果，不吸烟、不酗酒，保持情绪愉快，减少心理紧张和心理压力。定期测量血压及检查，以便及时发现，进一步确诊，并查明原因，及时治疗。

4．月经不调和经前期综合征　月经不调是青春期女性的一种常见疾病，表现为月经周期紊乱，出血期延长或缩短，出血量增多或减少，甚至月经闭止。卵巢功能失调、全身性疾病或其他内分泌疾病影响卵巢功能者都可能引起月经失调。月经失调主要是心理原因造成的，如果精神压力过重，引起情绪上的忧思焦虑，严重的甚至闭经。在经前期，约有1/3的女生会出现经前期综合征，其主要表现是头痛、眩晕、恶心、呕吐、心悸等。这些症状也会引起心理变化。如有的女孩子易怒、好攻击、对周围的人苛求、易与人发生口角；有些人烦躁、事事不如意、坐卧不安；有些人孤僻、多愁善感、多疑、好猜、好哭。此外，还有些人感到乳房胀痛、失眠、记忆力减退、注意力涣散等等。一般月经过后，症状即减弱或消失。经前期综合征是由于神经-内分泌功能失调造成的，心理因素在发病中占有重要地位。情绪抑郁愁闷，心理矛盾得不到适当的解决，都可能引起神经内分泌功能失调而引起本症。因此，保持乐观而稳定的情绪，将有

助于减少和消除经前期综合征。

5．乳房发育问题　女性到达青春期的第一个信息就是乳房发育。在发育过程中，有可能出现乳房过小或过大、双侧乳房发育不均、乳房不发育、乳房畸形以及乳房包块等现象。若发现这些情况，一是可通过健美运动促进胸肌发达，使乳房显得丰满；二是在医生指导下进行适当治疗。少女要到身体发育定型、性完全成熟才能确定乳房是否发育不良，不要过早下结论。

6．遗精（spermatorrhoea）　在没有性交或手淫的情况下射精，称为遗精。遗精多发生于夜间睡眠中，也可在清醒状态下发生。遗精是男性中学生中常见的一种正常生理现象。因为男性到了青春发育期，睾丸不断分泌大量的雄激素，同时产生大量精子，精子与精浆共同组成精液。精液不断产生并不断积聚在输精管内，当达到一定饱和状态时，便会通过遗精的方式排出体外。遗精虽然是一种正常的生理现象，但是它的间隔日期没有规律，一个月遗精在7～8次内均属正常。遗精次数过于频繁，尤其是梦遗，可能会扰乱睡眠，引起心理紧张、头痛、头晕、无精打采、胃纳不佳、浑身无力等症状。

7．手淫（masturbation）　手淫在青春期是一个极为普遍的性行为问题，是指通过自我抚弄或刺激性器官而产生性兴奋或性高潮的一种行为，这种刺激可以通过手或是某种物体，甚至两腿夹挤生殖器产生。手淫在青春期男、女均可发生，以男性更多见。许多研究材料表明，手淫可以起到缓解性心理和性生理的紧张的作用。但青少年对手淫问题有许多不正确的看法，这些看法在不同程度上影响了他们的身心健康。有过手淫问题的青少年常常对此感到内疚，手淫后感到情绪低落、担心、困惑、害怕、痛苦。因此，手淫并不是值得提倡的行为。应当设法引导学生将这方面的精力转移到学习或其他活动中去。

第二节　常见心理行为问题

由于青春期身体处于加速发育阶段，尤其是生殖系统在此期迅速发育而达到性成熟，而心理和社会适应能力发展的相对推迟，容易在心理上引起骚扰和波动，形成了复杂的青春期心理卫生问题。大多数青少年在青春期发育的某个阶段和某个方面会经历一些情绪或行为上的困难，被称为心理社会发育障碍（distrubed psychosocial development），如焦虑、抑郁、不良习惯等。这些问题绝大多数是暂时现象，只要得到适当的引导和帮助便能得到解决；但若不及时解决，持续时间长，问题可能会变得复杂、严重，造成心理缺陷，甚至影响一生的健康、学习、工作和行为，严重者还可能危及家庭和社会。

一、青春期综合征

青春期综合征是青少年特有的生理失衡和由此引发的心理失衡病症。青春期生理与心理发育不同步，心理发育相对滞后、过度用脑和不良习惯是形成青春期综合征的重要原因。主要表现为：①脑神经功能失衡：记忆力下降，注意力涣散，上课听不进，思维迟钝，意识模糊，学习成绩下降；白天精神萎靡，上课易瞌睡，大脑昏沉；夜晚大脑兴奋，浮想联翩，难以入眠，乱梦纷纭，醒后大脑特别，提不起精神。②性神经功能失衡：性冲动频繁，形成不良性习惯过度手淫，并且难以用毅力克服，由于频繁手淫、卫生不洁使生殖器出现红、肿、痒、臭等炎症，甚至性器官发育不良。③心理功能失衡：由于上述种种生理失衡症状困扰着青少年，造成青少年心理失衡，表现为心理状态欠佳、自卑自责、忧虑抑郁、烦躁消极、敏感多疑、缺乏学习兴趣、冷漠、忧伤、恐惧、自暴自弃、厌学、逃学、离家出走，甚至自虐、轻生。

尽管青春期综合征不属于严重的心理异常的范畴，可其对青少年心理的良好发展和人格的

健全却是十分有害的，如果不能迅速地走出这种心理误区，则有可能导致较为严重的心理障碍。因此要引起足够重视，应引导和教育青少年正确对待并正确评价自我，了解生理卫生知识，正确处理性方面可能出现的问题。用情感战胜理智，用顽强的意志力去克服自己的不良行为，使自己健康平稳地度过青春期。

二、青春期焦虑症

焦虑症（anxiety disorder） 即焦虑性神经症，是由一组情绪反应组成的综合征，患者以焦虑情绪反应为主要病态，同时伴有明显的自主神经系统功能紊乱。青春期是焦虑症的易发期，这个时期个体的发育回忆，身心变化处于一个转折点。随着第二性征的出现，个体对自己在体态、生理和心理等方面的变化，会产生一种神秘感，甚至不知所措。诸如女孩由于乳房发育而不敢挺胸、月经初潮而紧张不安；男孩出现性冲动、遗精、手淫后的追悔自责等，这些都将对青少年的心理、情绪及行为带来很大影响。往往由于好奇和不理解会出现恐惧、紧张、羞涩、孤独、自卑和烦恼，还可能伴发头晕头痛、失眠多梦、眩晕乏力、口干厌食、心慌气促、神经过敏、情绪不稳、体重下降和焦虑不安等症状。患者常因此而长期辗转于内科、神经科求诊，而经反复检查并没有发现任何器质性病变，这类病症在精神科常被诊断为青春期焦虑症。青春期焦虑症会严重危害青少年的身心健康，因此必须及时予以合理治疗。一般是心心理治疗为主，配合药物治疗。

三、青春期抑郁症

青春期的情绪改变是对身体改变、社会角色和各种关系变化的一种适应，其特点是反应强度大且易变化，情感变化复杂，容易狂喜、愤怒，也容易极度悲伤和恐惧。因外界不利环境如家长和老师的忽视、压制和不公平，学习压力和对性发育的困惑等而引起烦恼、焦虑和抑郁等情绪不稳现象并不少见。由于性的成熟，学习的紧张，神经系统承受的压力更大，尤其是在遇到挫折和烦恼的情况下，神经系统的功能很容易失调。如果反应异乎寻常的强烈和低落，可以出现持续性的、焦虑、抑郁、内疚、恐慌等状态，以致发生抑郁症（depression）。

抑郁是指情绪低落、思维迟钝、动作和语言减少，伴有焦虑、身体不适和睡眠障碍。情绪抑郁如果每星期发生3次，每次持续至少3小时或更多者被认为是持续性抑郁。青春期抑郁症的发病率为0.4～8.3%，女性是男性的2～3倍。

青春期抑郁症的表现多种多样，主要有以下几种：

1．自暴自弃　自责，自怨自艾；认为自己笨拙、愚蠢、丑陋和无价值。

2．多动　男性多见，表面淡漠，但内心孤独和空虚。有的则用多动、挑衅斗殴、逃学、破坏公物等方式发泄情感郁闷。

3．冷漠　整天心情不畅、郁郁寡欢，感觉周围一切都是灰暗的。

各种类型的抑郁症均有轻重程度不同。青春期轻者占大多数，严重的抑郁症对身心健康的影响明显，对学习毫无热情，注意力不能集中，学习成绩急剧下降；对前途和未来悲观失望，有轻生念头；人际关系差；对病无自知力，不愿求治。重度患者若无积极治疗，常导致严重后果。所以防治青春期抑郁症是青少年保健工作的重点内容。

四、饮食障碍

1．神经性厌食症（anorexia nervosa） 神经性厌食症是一种由不良心理社会因素引起的饮食障碍，早期为主动性节食、厌食，进而缺乏食欲、消瘦、内分泌代谢紊乱。近年来发病率有

所增加，女性多见。有的青春期女性盲目追求体型美，为了身材“苗条”而节食，甚至不吃动物性食品和主食，把人体所需的许多营养素都排队在食谱之外，食欲日趋降低，看见食物就恶心，最后发展到拒食。抑郁症伴饮食紊乱者也可出现神经性厌食。此外，家庭氛围不良、家长教养方法不当以及神经内分泌异常等也可能成为病因。神经性厌食往往伴随体重明显下降、身体虚弱、心率变缓、血压下降、皮肤粗糙和闭经等症状，还可能出现一些精神症状和行为失常，如不及时治疗将会导致严重后果。本病尚无系统性治疗方法，以心理治疗为主，结合行为调节、营养康复。对抑郁症伴饮食紊乱的患者可采用抗抑郁药物治疗。对因减肥导致神经性厌食者应耐心劝说，精心护理；鼓励少吃多餐，吃营养丰富的食物；引导青春期女性树立正确的审美观念，提倡健康美。

2．神经性贪食症（bulimia nervosa） 神经性贪食症的临床特征为反复发作和不可抗拒的摄食欲望及暴食行为，病人有担心发胖的恐惧心理，常采用引吐、导泻、禁食等方法以消除暴食引起的发胖。可与神经性厌食交替出现，两者具有相似的病理心理机制及性别、年龄分布。多数病人是神经性厌食的延续者，发病年龄较神经性厌食晚。贪食发作是选择的大多为高热量且易消化的食品，呕吐行为相当常见，而且往往是通过手指刺激咽喉部而完成，但也有些人可以随意地将胃内容物吐出。很多人在每次贪食发作之后就会产生情绪抑郁。人为导泻的病人常常出现各种并发症，如低钾血症、低氯性碱中毒等。反复呕吐则出现食管以及胃部的撕裂伤。暴食周期性地发生，常由心理紧张激发，有时可多至一日数次，患者的暴食和自我诱吐常常是秘密进行的。尽管贪食症患者表现出对肥胖的担忧，而且有些患者也的确很胖，但大多数人的体征仍在正常标准上下波动。治疗方法有心理治疗（认知-行为或人际治疗）和应用抗抑郁药物治疗。心理治疗可以有短期和长期的效果。即使没有抑郁症状，应用抗抑郁药也有一定好处，但前者的长期效果比抗抑郁药要好。

五、其他

1．网瘾 网瘾是指上网者由于长时间地和习惯性地沉浸在网络时空当中，对互联网产生强烈的依赖以至于达到了痴迷的程度而难以自我摆脱的行为状态和心理状态。其判断的基本标准主要包括四个方面：①行为和心理上的依赖感；②行为的自我约束和自我控制能力基本丧失；③工作和生活的正常秩序被打乱；④身心健康受到较严重的损害。一上网就不能控制时间，当网络被掐断或由于其他原因不能上网时还会感到烦躁不安、情绪低落或无所适从，觉得在网上比在现实生活中更快乐或更能实现自我等等。现在有不少青少年沉迷于电子游戏、电脑游戏或上网而不能自拔，长时间上网的青少年会出现情绪不稳定、注意力不集中、情绪低落、思维迟缓、孤独、焦虑、自主神经功能紊乱和睡眠障碍等现象，严重危害了青少年的身心健康，部分青少年因交网友甚至走向吸毒、偷窃等违法犯罪之路。互联网的飞速发展正迅速地改变着人们的生产和生活方式，学习网络、掌握网络、使用网络是进入信息时代的必由之路。对于青少年，我们要注意其生理和心理所处的特殊阶段，对他们上网不能一味采取封堵禁止的方法，教师和家长的配合是戒掉网瘾不可或缺的一环。要多与他们交流沟通，正确地引导他们上网，使其能真正利用网上的丰富资源促进自身发展。

2．物质滥用（substance abuse) 物质滥用是指反复、大量地使用与医疗目的无关且具有依赖性的一类有害物质。包括烟、酒、某些药物如镇静药、镇痛药、鸦片类、大麻、可卡因、幻觉剂、有同化作用的激素类药等。由于青春期的心理特点、现代社会复杂性增加及各种药物的广泛可得，使得越来越多的青少年滥用这些物质。对青少年和成人一项大范围的社区调查中发现，15～24岁的人有不同程度的物质依赖，程度因所滥用的物质性质不同而改变。许多儿童和

青少年的物质滥用常常未被发现，因此也未接受治疗。物质滥用造成青少年身心损伤，已成为全世界一大公害。

滥用物质种类的发生率随年龄、性别、地区、种族和地理因素不同而异。青少年中常见的滥用物质及其损害有：

（1）酒精：酒精的危害主要是中枢神经系统损伤。作为中枢神经系统抑制剂可以产生欣快、头昏眼花、多语和短期记忆障碍等。血清乙醇水平很高时可以出现呼吸抑制。饮酒最常见的胃肠道并发症是急性腐蚀性胃炎，表现为上腹疼痛、食欲不振、呕吐和大便隐血阳性。长期大量滥用可致酒精性肝炎、肝硬化。青少年每天饮酒数周后即对酒精产生生理性依赖。

（2）烟草：吸烟是导致心血管病、慢性支气管炎、肺气肿、肺癌、喉癌、咽癌、口腔癌等多种癌症及呼吸道和胃溃疡等疾病的主要危险因素。动脉硬化的严重程度与吸烟期限有关，自青春期开始吸烟者动脉硬化的危险则增加。吸烟对健康的不良影响可在青春期就出现，如慢性咳嗽和喘鸣等。烟草中的主要成分尼古丁刺激神经兴奋，使人产生依赖性。

（3）致幻剂：也称拟精神病药，包括大麻、麦角酰二乙胺。使用此类药物后产生类似精神病病人的表现，如生动的幻觉、片段的妄想及相应情绪、行为的改变。

（4）镇静催眠药：包括巴比妥类和苯二氮草类。这类药物的主要药理作用是中枢抑制，临床上主要用于镇静催眠和抗焦虑。由于应用范围甚广，极易形成滥用。

（5）兴奋剂：包括可卡因、咖啡因、苯丙胺及利他林等中枢神经系统兴奋药物。临床主要应用于振奋精神，可致欣快感。此类药物反复使用甚易形成心理依赖。此外，合成类固醇药物作为兴奋剂也有滥用现象。

（6）鸦片类：包括吗啡、可待因类罂粟碱等。吗啡是鸦片中主要的有效成分，医疗上主要用于镇静、止痛。吗啡及其衍生物包括海洛因（二醋吗啡）、可待因是当今世界成瘾问题最严重的毒品之一。吗啡样镇痛作用的人工合成镇痛药物，如哌替啶、美沙酮等药物使用也会成瘾。

预防青春期物质滥用的有效方法是加强青春期对抵制滥用物质的宣传和教育，积极和努力对青少年的心理疏导和精神帮助。

对物质滥用的青少年成功的长期处理方法是，在生理解毒后进行连续的医学随访和提供适宜的社会和心理支持。

3．青少年伤害　青少年是生命力最旺盛，死亡率最低的时期，但伤害是威胁青少年健康的严重卫生问题。全球每天死于伤害人数高达16000，即全年超过500万，中国有70万，其中青少年约占半数，伤害是我国1～19岁的首位死亡原因。

伤害（injury）是指因为能量（机械能、热能、电能等）的传递或干扰超过人体的耐受性造成组织损伤，窒息导致缺氧和刺激引起精神创伤。因此，伤害不只限于躯体组织的损伤或机能障碍，还可导致精神创伤或心理障碍。伤害可以分为非故意伤害和故意伤害两大类。伤害死因顺位依次为意外跌落、车祸、自杀、意外中毒和溺水，在15～19岁，其顺位为自杀、车祸、意外中毒、溺水、意外跌落和他杀。

伤害发生的个人因素包括个体状态差异如生理节律、神经系统成熟度、智力及事故倾向性等；性格与行为如病态性格、社会心理失衡等；生活与遭遇如紧张、压力等。儿童的身体和心理发育水平及状态是影响儿童意外伤害发生的重要因素。环境因素包括家庭因素和社会因素等。

常见伤害有：

（1）自杀：自杀是指自愿的、自己动手让自己死亡的行为。是一种自我惩罚和毁灭的行

为。自杀的原因有：

1）遗传因素：有自杀行为的青少年有时可有家族自杀行为倾向，其父母往往有自杀企图的历史。单卵双生子有一个自杀的，发生双生同胞自杀的可能性增大。

2）心理障碍：精神疾患如抑郁症、厌世症、边缘人格、攻击性行为等与青少年自杀有密切关系。

3）环境因素：父母不和睦、有不良行为，亲子关系紧张可使青少年产生自杀。学校课程负担重、考试失败是近年来自杀的重要因素。其他如失恋、性行为问题、物质滥用等与自杀也有密切关系。

对有自杀企图的青少年需要心理专家的咨询，最好能够住院帮助解决存在的冲突及提供安全场所。在自杀发生前，常有许多心理与行为的改变，这些改变或征候可被父母、同学或同伴发现，应早期采取措施，防止自杀的发生。

（2）暴力：指一种威胁或身体力量对某人或一群人造成伤害或死亡。在美国，青少年枪杀在逐年增加。青少年暴力行为与发生在家庭内外的暴力有关，由于在儿童期受虐待和忽视，目击暴力，青少年性乱和体罚、遭受暴力和攻击可使青少年今后发生暴力行为和犯罪。

对有暴力行为的青少年需要识别和干预。有进攻行为的青少年常伴有精神发育迟滞、学习困难、中重度语言障碍和心理障碍如注意缺陷多动综合征等。

预防暴力需通过改变个人行为、改善家庭环境、提高社区和全社会整体环境的共同作用。

（3）车祸：即道路交通伤害，是指车辆如汽车、摩托车、自行车等交通工具在公用道路上行驶过程中，因违章行为或过失发生碰撞、颠覆等造成人身伤亡或经济损失的事故。车祸的原因包括：

1）内源性因素：缺乏经验，未察觉到危险，不遵守交通规则和青少年的冒险行为。某些因素可改变心理状况，促发车祸发生，如紧张性情绪、酒精或药物的使用等。

2）环境因素：如道路设计和质量问题；车辆安全因素如刹车的制动性不好，没有防护设施（如头盔）；气候因素：如雨、雪、雾等不利气候条件下，车祸发生增加。

伤害的预防与控制与控制措施包括伤害监测、伤害干预措施研究、改善环境、加强安全防范措施、开展健康教育和各种宣传以及建立相关法律和法规，如制订有毒物品包装、易燃物管理及儿童乘车安全的法规等。

（毛萌）

第七章 新生儿与新生儿疾病

第一节 概 述

新生儿（neonate，newborn）系指从脐带结扎到生后28天内的婴儿。新生儿学（neonatology）是研究新生儿生理、病理、疾病防治及保健等方面的学科。新生儿学原属儿科学范畴，近数十年来发展十分迅速，现已渐形成独立的学科。新生儿是胎儿的继续，与产科密切相关，因此，又是围生医学（perinatology）的一部分。

围生医学是研究胎儿出生前后影响胎儿和新生儿健康的一门学科，涉及产科、新生儿科和相关的遗传、生化、免疫、生物医学工程等领域，是一门边缘学科，并与提高人口素质、降低围产儿死亡率密切相关。围生期（perinatal period）是指产前、产时和产后的一个特定时期。由于各国医疗保健水平差异很大，其定义有所不同。目前国际上有四种定义：①自妊娠28周（此时胎儿体重约1000克）至生后7天；②自妊娠20周（此时胎儿体重约500克）至生后28天；③妊娠28周至生后28天；④自胚胎形成至生后7天。我国目前采用第一种定义。围生期的婴儿称围生儿，由于经历了宫内迅速生长、发育，以及从宫内向宫外环境转换阶段，因此，其死亡率和发病率均居于人的一生之首，尤其是生后24小时内。

【新生儿分类】

新生儿分类有不同的方法，分别根据胎龄、出生体重、出生体重和胎龄的关系及出生后周龄等。

1．根据胎龄分类　胎龄（gestational age，GA）是从最后1次正常月经第1天起至分娩时为止，通常以周表示。①足月儿（full term infant）：37周≤GA<42周（259～293天）的新生儿；②早产儿（preterm infant）：GA<37周（<259天）的新生儿；③过期产儿（post-term infant）：GA≥42周（≥294天）的新生儿。

2．根据出生体重分类　出生体重（birth weight，BW）指出生1小时内的体重。①低出生体重 [(low birth weight，LBW)儿：BW<2500g的新生儿，其中BW<1500g称极低出生体重（very low birth weight，VLBW）儿，BW<1000g称超低出生体重（extremely low birth weight，ELBW）儿。LBW儿中大多是早产儿，也有足月或过期小于胎龄儿；②正常出生体重（normal birth weight，NBW）儿：BW≥2500g并≤4000g的新生儿；③巨大（macrosomia）儿：BW>4000g的新生儿。

3．根据出生体重和胎龄的关系分类见（图7-1）①小于胎龄（small for gestational age，SGA）儿：婴儿的BW在同胎龄儿平均出生体重的第10百分位以下；②适于胎龄（appropriate for gestational age，AGA）儿：婴儿的BW在同胎龄儿平均出生体重的第10至90百分位之间；③大于（1arge for gestational age，LGA）儿：婴儿的BW在同胎龄儿平均出生体重的第90百分位以上。我国15城市不同胎龄新生儿出生体重值见表7-1。

4．根据出生后周龄分类 ①早期新生儿（early newbom）：生后1周以内的新生儿，也属于围生儿。其发病率和死亡率在整个新生儿期最高，需要加强监护和护理；②晚期新生儿（late newborn）：出生后第2周至第4周末的新生儿。

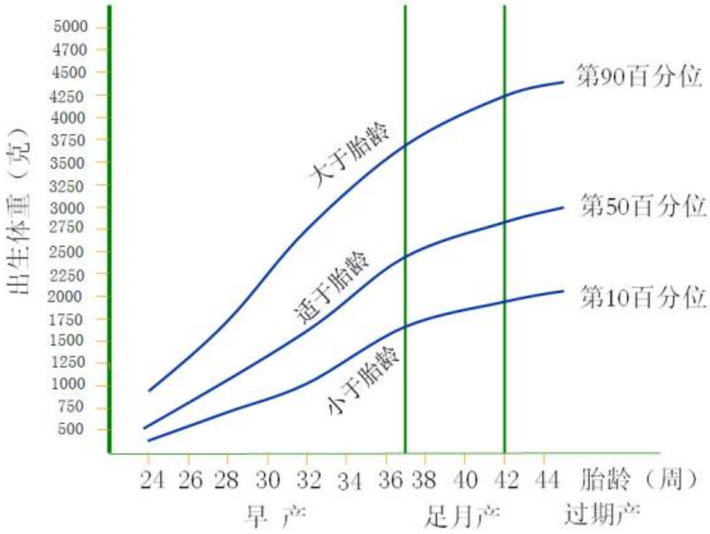

图7-1 新生儿胎龄与出生体重的百分位曲线

表7-1 我国15城市不同胎龄新生儿出生体重值

胎龄（周）	平均值(g)	标准差(g)	第3百分位数(g)	第10百分位数(g)	第90百分位数(g)	第97百分位数(g)
28	1389	302	923	972	1799	2071
29	1475	331	963	1057	2034	2329
30	1715	400	1044	1175	2255	2563
31	1943	512	1158	1321	2464	2775
32	1970	438	1299	1488	2660	2968
33	2133	434	1461	1670	2843	3142
34	2363	449	1635	1860	3013	3299
35	2560	414	1815	2051	3169	3442
36	2708	401	1995	2238	3312	3572
37	2922	368	2166	2413	3442	3690
38	3086	376	2322	2569	3558	3798
39	3197	371	2457	2701	3660	3899
40	3277	392	2562	2802	3749	3993
41	3347	396	2632	2865	3824	4083
42	3382	413	2659	2884	3885	4170
43	3359	448	2636	2852	3932	4256
44	3303	418	2557	2762	3965	4342

*摘自中国15城市新生儿体格发育科研协作组资料（中华儿科杂志1989，27：316）

5．高危儿（high risk infant） 指已发生或可能发生危重疾病而需要监护的新生儿。常见于以下情况：①母亲疾病史：母有糖尿病、感染、慢性心肺疾患、吸烟、吸毒或酗酒史，母亲为Rh 阴性血型，过去有死胎、死产或性传播病史等；②母孕史：母年龄>40岁或<16岁，孕期有阴道流血、妊娠高血压、先兆子痫、子痫、羊膜早破、胎盘早剥、前置胎盘等；③分娩史：难产、手术产、急产、产程延长、分娩过程中使用镇静和止痛药物史等；④新生儿：窒息、多胎儿、早产儿、小于胎龄儿、巨大儿、宫内感染和先天畸形等。

【新生儿病房分级】

1．根据医护水平及设备条件将新生儿病房分为三级：①I级新生儿病房（1evel I nursery）：即普通婴儿室，适于健康新生儿，主要任务是指导父母护理技能和方法，以及对常见遗传代谢疾病进行筛查。母婴应同室，以利于母乳喂养及建立母婴相依感情，促进婴儿身心健康。②Ⅱ级新生儿病房（1evel Ⅱ nursery）：即普通新生儿病房，适于胎龄>32周、出生体重≥1500g（发达国家为胎龄>30周、出生体重≥1200g）的早产儿及有各种疾病而又无需循环或呼吸支持、监护的婴儿。③Ⅲ级新生儿病房（1evel Ⅲ nursery）:即新生儿重症监护室（neonatal intensive care unit，NICU），是集中治疗I、Ⅱ级新生儿病房转来的危重新生儿的病室。应具备高水平的新生儿急救医护人员及新生儿急救转运系统，一般应设立在医学院校的附属医院或较大的儿童医院。

2．收治对象　包括：①应用辅助勇气及拔管后24小时内的新生儿；②重度围生期窒息儿；③严重心肺疾病或呼吸暂停儿；④外科大手术术后（尤其是24小时内）；⑤出生体重＜1500g的小早产儿；⑥接受全胃肠外营养或需换血术者；⑦顽固性惊厥者；⑧多器官功能衰竭（如休克、DIC、心力衰竭、肾衰竭等）者。

3．设备　监护室应配备完善的监护治疗设备及报警系统，以进行各种生命体重的监测。①心电监护：主要监测患儿的心率、节律和心电波形变化。②呼吸监护：主要监测患儿的呼吸频率、节律变化及呼吸暂停。③血压监护：有直接测压法（创伤性）和间接测压法（无创性）两种。前者经动脉（多为脐动脉）插入导管直接连续测量血压，其测量值准确，但损伤复杂，并发症多，临床仅在周围灌注不良时应用；后者是将袖带束于患儿上臂间接间断测量，自动显示收缩压、舒张压和平均动脉压，其测量值准确性不及直接测压法，但方法简便，无并发症，是目前国内NICU最常用的血压测量方法。④体温监测：置婴儿于热辐射式抢救台上或暖箱内，将体温监测仪传感器分别置于腹壁皮肤和肛门内，其腹壁皮肤温度、核心温度和环境温度自动连续显示。⑤血气监测：包括经皮氧分压（$Tc\text{-}PO_2$）、二氧化碳分压（$TcPCO_2$）及脉搏氧饱和度监护（transcutaneous oxygen saturation, $TcSO_2$）。具有无创、连续、自动、操作简便并能较好的反映自身血气变化的趋势等优点，但测量值较动脉血气值有一定差距，尤其在周围血液循环灌注不良时，其准确性更差，因此，应定期检测动脉血气。由于$TcSO_2$相对较准确，故是目前NICU中血氧动态监测的常用手段。

近数十年来，由于NICU的普遍建立，新生儿病死率和远期发病率已明显下降。

第二节 正常足月儿和早产儿的特点与护理

正常足月儿（normalterminfant）是指胎龄≥37周并<42周，出生体重≥2500g并≤4000g，无畸形或疾病的活产婴儿。早产儿又称未成熟儿（preterm infant；premature infant）。近年来我国早产儿的发生率呈逐年上升趋势：1985年为4.5%，1998年为5.87%，而2002～2003年上升至7.76%，即我国每年约有256万早产儿出生。其死亡率约为12.7％～20.8％，且胎龄愈小，体重愈轻，死亡率愈高。根据美国的资料，出生体重500～600g和1250～1500g的婴儿存活率分别为近20%和90%以上；出生体重在1500g以下者死亡人数占整个新生儿死亡人数的50%以上，而占婴儿的50%，是医学和伦理学领域面临的最大挑战之一。因此，预防早产对于降低新生儿死亡率，减少儿童的伤残率均具有重要意义。母孕期感染、吸烟、酗酒、吸毒、外伤、生殖器畸形、过度劳累及多胎等是引起早产的原因。另外，种族和遗传因素与早产也有一定的关系。

1．正常足月儿和早产儿外观特点　不同胎龄的正常足月儿与早产儿在外观上各具特点（见表7-2），因此可根据初生婴儿的体格特征和神经发育成熟度来评定其胎龄。目前国际上有数种评分方法，最常用的是Ballard评分法。

表7-2　足月儿与早产儿外观特点

	早产儿	足月儿
皮肤	绛红、水肿和毳毛多	红润、皮下脂肪丰满和毳毛少
头	头更大（占全身比例1/3）	头大（占全身比例1/4）
头发	细而乱	分条清楚
耳壳	软、缺乏软骨、耳舟不清楚	软骨发育好、耳舟成形、直挺
乳腺	无结节或结节＜4mm	结节＞4mm，平均7mm
外生殖器		
男婴	睾丸未降或未全降	睾丸已降至阴囊
女婴	大阴唇不能遮盖小阴唇	大阴唇遮盖小阴唇
指、趾甲	未达指、趾端	达到或超过指、趾端
跖纹	足底纹理少	足纹遍及整个足底

2．正常足月儿和早产儿生理特点

（1）呼吸系统：胎儿肺内充满液体，分娩时儿茶酚胺释放使肺液分泌减少，足月儿约30～35ml/kg，出生时经产道挤压，约1/3肺液由口鼻排出，其余在建立呼吸后由肺间质内毛细血管和淋巴管吸收，如吸收延迟，则出现湿肺症状。呼吸频率较快，安静时约为40次/分左右，如持续超过60～70次/分称呼吸急促，常由呼吸或其他系统疾病所致。胸廓呈圆桶状，肋间肌薄弱，呼吸主要靠膈肌的升降，呈腹式呼吸。呼吸道管腔狭窄，黏膜柔嫩，血管丰富，纤毛运动差，易致气道阻塞、感染、呼吸困难及拒乳。

早产儿由于：①呼吸中枢及呼吸器官发育不成熟；②红细胞内缺乏碳酸酐酶，碳酸分解为二氧化碳的数量减少，因而不能有效地刺激呼吸中枢；③肺泡数量少，呼吸道黏膜上皮细胞呈扁平立方形，毛细血管与肺泡间距离较大，气体交换率低；④呼吸肌发育不全，咳嗽反射弱。因此，早产儿呼吸浅快不规则，易出现周期性呼吸及呼吸暂停（apnea）或青紫。呼吸暂停是指呼吸停止>20秒，伴心率<100次/分及发绀。其发生率与胎龄有关，胎龄愈小、发生率愈高，且常于生后第1天出现。因肺泡表面活性物质少，易发生呼吸窘迫综合征。由于肺发育不成熟，易感高气道压力、高容量、高浓度氧以及炎性损伤而致支气管肺发育不良（bronchopulmonary dysplasia, BPD），即慢性肺疾病（chronic lung disease，CLD）。

（2）循环系统：出生后血液循环动力学发生重大变化：①胎盘-脐血循环终止；②肺循环阻力下降，肺血流增加；③回流至左心房血量明显增多，体循环压力上升；④卵圆孔、动脉导管功能上关闭。严重肺炎、酸中毒、低氧血症时，肺血管压力升高，当压力等于或超过体循环时，可致卵圆孔、动脉导管重新开放，出现右向左分流，称持续胎儿循环（persistent fetal circulation，PFC），即新生儿持续肺动脉高压（persistent pulmonary hypertension of newborn，PPHN）。新生儿心率波动范围较大，通常为90～160次/分。足月儿血压平均为70/50mmHg（9.3/6.7kPa）。

早产儿心率偏快，血压较低，部分可伴有动脉导管开放。

（3）消化系统：足月儿出生时吞咽功能已经完善，但食管下部括约肌松弛，胃呈水平位，幽门括约肌较发达，易溢乳甚至呕吐。消化道面积相对较大，管壁薄、通透性高，有利于大量的流质及乳汁中营养物质的吸收，但肠腔内毒素和消化不全产物也容易进入血循环，引起中毒症状。除淀粉酶外，消化道已能分泌充足的消化酶，因此不宜过早喂淀粉类食物。胎便由胎儿

肠道分泌物、胆汁及咽下的羊水等组成，呈糊状，为墨绿色。足月儿在生后24小时内排胎便，约2～3天排完。若生后24小时仍不排胎便，应排除肛门闭锁或其它消化道畸形。肝内尿苷二磷酸葡萄糖醛酸基转移酶的量及活力不足，是生理性黄疸的主要原因，同时对多种药物处理能力(葡萄糖醛酸化）低下，易发生药物中毒。

早产儿吸吮力差，吞咽反射弱，胃容量小，常出现哺乳困难或乳汁吸入引起吸入性肺炎。消化酶含量接近足月儿，但胆酸分泌少，脂肪的消化吸收较差。缺氧或喂养不当等不利因素易引起坏死性小肠结肠炎。由于胎粪形成较少及肠蠕动差，胎粪排出常延迟。肝功能更不成熟，生理性黄疸程度较足月儿重，持续时间更长，且易发生胆红素脑病。肝脏合成蛋白能力差，糖原储备少，易发生低蛋白血症、水肿和低血糖。

（4）泌尿系统：足月儿出生时肾结构发育已完成，但功能仍不成熟。肾稀释功能虽与成人相似，但其肾小球滤过率低，浓缩功能差，故不能迅速有效地处理过多的水和溶质，易发生水肿或脱水。新生儿一般在生后24小时内开始排尿，少数在48小时内排尿，一周内每日排尿可达20次。

早产儿肾浓缩功能更差，排钠分数高，肾小管对醛固酮反应低下，易出现低钠血症。葡萄糖阈值低，易发生糖尿。碳酸氢根阈值极低和肾小管排酸能力差，由于普通牛乳中蛋白质含量及酪蛋白比例均高，喂养时可使内源性氢离子增加，超过肾小管排泄能力，引起晚期代谢性酸中毒，表现为面色苍白、反应差、体重不增和代谢性酸中毒。因此人工喂养的早产儿应采用早产儿配方奶粉。

（5）血液系统：足月儿出生时血红蛋白为170g/L（140～200g/L），由于刚出生时入量少、不显性失水等原因使血液浓缩，血红蛋白值上升，生后24小时达峰值，约于第1周末恢复至出生时水平，以后逐渐下降。生后2周内静脉血血红蛋白≤130g/L或毛细管血红蛋白≤145g/L定义为新生儿贫血。血红蛋白中胎儿血红蛋白占70％～80％，5周后降至55％，随后逐渐被成人型血红蛋白取代。网织红细胞数初生3天内为0.04～0.06，4～7天迅速降至0.005～0.015，4～6周回升至0.02～0.08。血容量为85～l00ml/kg，与脐带结扎时间有关，脐带结扎延迟可从胎盘多获得35％的血容量。白细胞数生后第1天为(15～20)×10^9/L，3天后明显下降，5天后接近婴儿值；分类中以中性粒细胞为主，4～6天与淋巴细胞相近，以后淋巴细胞占优势。血小板数与成人相似。由于胎儿肝脏维生素K储存量少，凝血因子Ⅱ、Ⅶ、Ⅸ、Ⅹ活性较低。

早产儿血容量为85～110ml/kg，周围血中有核红细胞较多，白细胞和血小板稍低于足月儿。大多数早产儿第3周末嗜酸性细胞增多，并持续2周左右。由于早产儿红细胞生成素水平低下、先天性铁储备少、血容量迅速增加，“生理性贫血”出现早，而且胎龄越小，贫血持续时间越长，程度越严重。

（6）神经系统：新生儿脑相对大，但脑沟、脑回仍未完全形成。新生儿出生后头围生长速率约为1.1cm，至生后40周左右逐渐减缓。脊髓相对长，其末端约在3、4腰椎下缘，故腰穿时应在第4、5腰椎间隙进针。足月儿大脑皮层兴奋性低，睡眠时间长，觉醒时间一昼夜仅为2～3小时，大脑对下级中枢抑制较弱，且锥体束、纹状体发育不全，常出现不自主和不协调动作。出生时已具备多种暂时性原始反射。临床上常用的原始反射如下：①觅食反射（rooting reflex）：用左手托婴儿呈半卧位，右手食指触其一侧面颊，婴儿反射性地转头向该侧；②吸吮反射（sucking reflex）：将乳头或奶嘴放入婴儿口内，会出现有力的吸吮动作；③握持反射（grasp reflex）：将物品或手指置入婴儿手心中，婴儿立即将其握紧；④拥抱反射（Moro reflex）：新生儿仰卧位，从背部托起婴儿，一手托住婴儿颈及背部，另一手托着枕部，然后托住枕部的手突然下移数厘米（不是放手）使婴儿头及颈部“后倾”数厘米。正常可见两上肢外展并伸直，手指

张开，然后上肢屈曲回缩。早产儿往往出现不安全的反应，即上肢不屈曲回缩。

正常情况下，上述反射生后数月自然消失。新生儿期如这些反射减弱或消失，或数月后仍不消失，常提示有神经系统疾病。此外，正常足月儿也可出现年长儿的病理性反射如克氏征（Kernig 征）、巴宾斯基征（Babinski征）和佛斯特征（Chvostek征）等，腹壁和提睾反射不稳定，偶可出现阵发性踝阵挛。

早产儿神经系统成熟度与胎龄有关，胎龄愈小，原始反射愈难引出或反射不完全。此外，早产儿尤其极低出生体重儿脑室管膜下存在着发达的胚胎生发层组织，易发生脑室周围-脑室内出血及脑室周围白质软化。

（7）体温：新生儿体温调节中枢功能尚不完善，皮下脂肪薄，体表面积相对较大，皮肤表皮角化层差，易散热，早产儿尤甚。寒冷时无寒战反应而靠棕色脂肪化学产热。生后环境温度显著低于宫内温度，散热增加，如不及时保温，可发生低体温、低氧血症、低血糖和代谢性酸中毒或寒冷损伤。中性温度（neutral temperature）是指使机体维持体温正常所需的代谢率和耗氧量最低时的最适环境温度。出生体重、生后日龄不同，中性温度也不同（表7-3）。新生儿正常体表温度为36.0～36.5℃，正常核心（直肠）温度为36.5～37.5℃。不显性失水过多可增加热的消耗，适宜的环境湿度为50%～60%。环境温度过高、进水少及散热不足，可使体温增高，发生脱水热。

早产儿棕色脂肪少，产热能力差，寒冷时更易发生低体温，甚至硬肿症。汗腺发育差，环境温度过高时体温亦易升高。

表7-3　不同出生体重新生儿的中性温度

出生体重（kg）	中性温度			
	35℃	34℃	33℃	32℃
1.0	初生10天内	10天以后	3周以后	5周以后
1.5		初生10天内	10天以后	4周以后
2.0		初生2天内	2天以后	3周以后
>2.5			初生2天内	2天以后

（8）能量及体液代谢：新生儿基础热量消耗为209kJ/kg（50kcal/kg），每日总热量约需418～502kJ/kg（100～120kcal/kg）。早产儿吸吮力弱，消化功能差，在生后数周内常不能达到上述需要量，因此需肠道外营养。

初生婴儿体内含水量占体重的70～80%，且与出生体重及日龄有关，出生体重越低、日龄越小、含水量越高，故新生儿需水量因出生体重、胎龄、日龄及临床情况而异。生后第1天需水量为每日60～100ml/kg，以后每日增加30ml/kg，直至每日150～180ml/kg。生后由于体内水分丢失较多、进入量少、胎脂脱落、胎粪排出等使体重下降，约1周末降至最低点（小于出生体重的10%，早产儿为15%～20%），10天左右恢复到出生体重，称生理性体重下降。早产儿体重恢复的速度较足月儿慢。

足月儿钠需要量为1～2mmol/（kg·d），<32周早产儿为3～4mmol／（kg·d）；初生婴儿10天内一般不需补钾，以后需要量为1～2mmol／（kg·d）。

（9）免疫系统：新生儿非特异性和特异性免疫功能均不成熟。皮肤粘膜薄嫩易损伤；脐残端未完全闭合，离血管近，细菌易进入血液；呼吸道纤毛运动差，胃酸、胆酸少，杀菌力差，同时分泌型IgA缺乏，易发生呼吸道和消化道感染。血-脑屏障发育未完善，易患细菌性脑膜炎。血浆中补体水平低，调理素活性低，多形核白细胞产生及储备均少，且趋化性及吞噬能力低下，

早产儿尤甚。免疫球蛋白IgG虽可通过胎盘，但与胎龄相关，胎龄愈小，IgG含量愈低；IgA和IgM不能通过胎盘，因此易患细菌感染，尤其是革兰阴性杆菌感染。抗体免疫答低下或迟缓，尤其是对多糖类疫苗和荚膜类细菌。T细胞免疫功能低下是新生儿免疫应答无能的主要原因，早产儿更差。随着生后不断接触抗原，T细胞渐趋成熟。

（10）常见的几种特殊生理状态：①生理性黄疸：参见本章第九节；②“马牙”和“螳螂嘴”：在口腔上腭中线和齿龈部位，有黄白色、米粒大小的小颗粒，是由上皮细胞堆积或粘液腺分泌物积留形成，俗称“马牙”，数周后可自然消退；两侧颊部各有一隆起的脂肪垫，有利于吸吮乳汁。两者均属正常现象，不可挑破，以免发生感染。少数初生婴儿在下切齿或其他部位有早熟齿，称新生儿齿或诞生牙，多数活动易脱落而致吸入呼吸道，故需拔除。③乳腺肿大和假月经：男女新生儿生后4～7天均可有乳腺增大，如蚕豆或核桃大小，2～3周消退，切忌挤压，以免感染；部分女婴生后5～7天阴道流出少许血性分泌物，或大量非脓性分泌物，可持续1周。上述现象均由于来自母体的雌激素中断所致。④新生儿红斑及粟粒疹：生后1～2天，在头部、躯干及四肢常出现大小不等的多形性斑丘疹，称为“新生儿红斑”，1～2天后自然消失。也可因皮脂腺堆积在鼻尖、鼻翼、颜面部形成小米粒大小黄白色皮疹，称为“新生儿粟粒疹”，脱皮后自然消失。

3．足月儿及早产儿护理

（1）保暖：生后应立即用预热的毛巾擦干新生儿，并采取各种保暖措施，使婴儿处于中性温度中。早产儿、尤其出生体重<2000g或低体温者，应置于自温箱中，并根据体重、日龄选择中性环境温度。温箱中的湿化装置容易滋生“水生菌”，故应每日换水，并加1：10000硝酸银2ml。无条件者可采取其他保暖措施，如用热水袋（应注意避免烫伤）等。因新生儿头部表面积大，散热量多，寒冷季节应戴绒布帽。

（2）喂养：正常足月儿生后半小时即可抱至母亲处哺乳，以促进乳汁分泌，提倡按需哺乳。无母乳者可给配方乳。详见第五章第二节。

早产儿也应酌情尽早母乳喂养。与足月人乳相比，早产儿的母乳含有更多的蛋白质、必需脂肪酸、能量、矿物质、微量元素和IgA，可使早产儿在较短期恢复到出生体重。对吸吮能力差、吞咽功能不协调的小早产儿、或有病者可由母亲挤出乳汁经管饲喂养。也可暂行人工喂养，但应用早产儿配方奶。哺乳量应因人而异，原则上是胎龄愈小，出生体重愈低，每次哺乳量愈少，喂奶间隔时间也愈短，并且根据奶后有无腹胀、呕吐、胃内残留（管饲喂养）及体重增长情况（理想的每天增长为10～15g/kg）进行调整。对于出生体重＜1500g的小早产儿可试行微量肠道喂养，哺乳量不能满足所需热能者应辅以静脉营养。

足月儿生后应肌注1次维生素K_1 0.5～lmg，早产儿连用3天。生后第4天加维生素C 50～100mg/d，10天后加维生素A 500～1000IU/d，维生素D 400～1000IU/d，4周后应注意铁的摄入量，足月儿每日给元素铁 2mg/kg，极低出生体重儿每日给3～4mg/kg，并同时加用维生素E 25IU和叶酸2.5mg，每周2次。极低出生体重儿出生后可给予重组人类红细胞生成素，每周600～750IU/kg，皮下注射，分3次给药，可减少输血需要。

（3）呼吸管理：保持呼吸道通畅，早产儿仰卧时可在肩下放置软垫，避免颈部弯曲。低氧血症时予以吸氧，应以维持动脉血氧分压6.7～9.3kPa（50～70mmHg）或经皮血氧饱和度90%～95%为宜。切忌给早产儿常规吸氧，以防吸入高浓度氧或吸氧时间过长导致早产儿视网膜病（retinopathy of prematurity，ROP）和BPD。呼吸暂停者可经弹、拍打足底或托背等恢复呼吸，可同时给予氨茶碱静脉注入，负荷量为4～6mg/kg，12小时后给予维持量2～4mg/（kg.d），分2～4次给药。继发性呼吸暂停应病因治疗。

（4）预防感染：婴儿室工作人员应严格遵守消毒隔离制度。接触新生儿前应严格洗手；护理和操作时应注意无菌；工作人员或新生儿如患感染性疾病应立即隔离，防止交叉感染；避免过分拥挤，防止空气污染和杜绝乳制品污染。

（5）皮肤黏膜护理：①勤洗澡，保持皮肤清洁。每次大便后用温水清洗臀部，勤换尿布防止红臀或尿布疹发生。②保持脐带残端清洁和干燥。一般生后3～7天残端脱落，脱落后如有黏液或渗血，应用碘伏消毒或重新结扎；如有肉芽组织，可用硝酸银烧灼局部；如有化脓感染，用过氧化氢溶液或碘酒消毒。③口腔黏膜不宜擦洗。④衣服宜宽大，质软，不用钮扣。应选用柔软、吸水性强的尿布。

（6）预防接种：①卡介苗：生后3天接种，目前新生儿接种卡介苗有皮上划痕和皮内注射两种方法。皮内接种后2～3周出现红肿硬结，约10mm×10mm，中间逐渐形成白色小脓疱，自行穿破后呈溃疡，最后结痂脱落并留下一永久性圆形疤痕。皮上接种1～2周即出现红肿，3～4周化脓结痂，1～2个月脱落痊愈，并留下一凹陷的划痕疤痕。早产儿、有皮肤病变或发热等其他疾病者应暂缓接种；对疑有先天性免疫缺陷的新生儿，应绝对禁忌接种卡介苗，以免发生全身感染而危及生命。②乙肝疫苗：生后第1天、1个月、6个月时应各注射重组乙肝病毒疫苗1次，如母亲为乙肝病毒携带者或乙肝患者，婴儿出生后应立即肌注高价乙肝免疫球蛋白（HBIg）0.5ml，同时换部位注射重组乙肝病毒疫苗。

（7）新生儿筛查：应开展先天性甲状腺功能减低症及苯丙酮尿症等先天性代谢缺陷病的筛查。

第三节 小于胎龄儿和大于胎龄儿

一、小于胎龄儿和宫内生长迟缓

小于胎龄儿（SGA）与宫内生长迟缓（intrauterine growth restriction/retardation, IUGR）相关，但两者并非同义词。SGA是描述出生体重低于人群正常值或低于某一体重阈值，通常是指出生体重在同胎龄儿出生体重的第10百分位以下的新生儿，有早产、足月、过期产小于胎龄儿之分。其原因可能是病理因素，如IUGR的婴儿；也可能是非病理性，如虽小，但健康。IUGR是指由于各种不利因素导致胎儿在宫内生长模式偏离或低于预期的生长模式，但并非一定是SGA婴儿，也可能是AGA婴儿，但其出生体重低于其兄弟、家族以及自身生长势能的预期生长模式。大部分SGA和IUGR婴儿是正常的，但从整体上来看，其围生期死亡率及其远期发病率均明显高于适于胎龄儿，尤其是出生体重在第3个百分位以下者。

【病因】

1．母亲因素　①孕母年龄过大或过小、身材矮小；②孕母营养不良（尤其发生在孕晚期时对出生体重影响最明显）、严重贫血、微量元素缺乏等，是发展中国家常见的IUGR原因；③缺氧或血供障碍：如原发性或妊娠高血压、晚期糖尿病、慢性肺、肾疾患、居住在海拔较高处等，均可因子宫、胎盘血流减少而影响胎儿生长；④孕母吸烟，在发达国家常作为单一的、最重要的致IUGR因素，其他的有吸毒、应用对胎儿有损伤的药物、接触放射线等。

2．胎儿因素　①双胎或多胎；②染色体疾病：染色体缺失或不平衡可引起胎儿生长减慢；③先天性畸形；④慢性宫内感染（如TORCH感染），尤其当感染发生在孕早期，正值胎儿器官形成期，可引起细胞破坏或数目减少；⑤性别、胎次：女婴、第一胎平均出生体重通常低于男婴和以后儿胎；种族或人种不同，出生体重也有差异。

3．胎盘和脐带因素　胎儿通过胎盘从母体摄取营养，胎儿大小与胎盘生长直接相关。扫

母亲有血管性疾病，子宫异常（解剖异常、子宫平滑肌瘤），胎盘功能不全如小胎盘、胎盘绒毛广泛损伤或胎盘血管异常、慢性胎盘早剥等，另外，脐带附着异常、双血管脐带等均可影响胎儿生长。

4．内分泌因素　任何一种激素先天性缺陷均可致胎儿生长迟缓，如胰岛素样生长因子（insulin-like growth factor，IGFs），尤其是IGF-Ⅰ（主要调节孕后期胎儿及新生儿生后早期的生长）、IGF-Ⅱ（主要调节胚胎的生长），胰岛素样生长因子结合蛋白（insulin like growth factor binding protein, IGFBPs，尤其是IGFBP-3）以及营养物质（葡萄糖）-胰岛素-胰岛素样生长因子代谢轴等均是调节胎儿生长的中心环节。

【临床分型】

1．匀称型　常由染色体异常、遗传代谢性疾病、先天性感染所致，损伤发生在孕早期，患儿出生时头围、身长、体重成比例下降，体型匀称。

2．非匀称型　常由孕母营养因素、血管性疾病所致，损伤发生在妊娠晚期，胎儿体重下降与身长、头围降低不成比例，即体重小于预期的胎龄，而身长及头围与预期的胎龄相符，大脑发育常不受影响。

【并发症】

1．围生期窒息　小于胎龄儿在宫内常处于慢性缺氧环境中，故常并发围生期窒息，且多留有不同程度的神经系统后遗症。

2．先天性畸形　染色体畸变和慢性宫内感染可引起各种先天性畸形。

3．低血糖　由于：①肝糖原贮存减少；②糖异生底物如脂肪酸和蛋白质缺乏，糖异生酶活力低下；③胰岛素水平相对较高，而儿茶酚胺水平较低；④游离脂肪酸和甘油三酯氧化减少，使能源系统中各种物质间转化受到限制；⑤出生时如有缺氧情况，使糖原贮存更趋于耗竭，极易发生低血糖。非匀称型由于脑与肝之比相对较大，更易发生低血糖。

4．红细胞增多症-高黏滞度综合征　胎儿宫内慢性缺氧，引起红细胞生成素水平增加、红细胞增多，后者可引起血黏稠度增高而影响组织正常灌注，导致全身各器官受损而出现一临床症状和体征，如呼吸窘迫、青紫、低血糖、心脏扩大、肝大、黄疸、坏死性小肠结肠炎等，并且进一步加重了低血糖和脑损伤。

5．胎粪吸入综合征：宫内缺氧、肠蠕动增加和肛门括约肌松弛，常有胎便排入羊水，胎儿如在产前或产程中吸入污染胎粪的羊水，则引起胎粪吸入综合征。

【治疗】

1．有围生期窒息者生后立即进行复苏。

2．注意保暖。有条件者置入暖箱中，维持体温在正常范围，减少能量消耗。

3．尽早开奶，预防低血糖。注意监测血糖，及时发现低血糖，并给予治疗（详见本章第十五节）。能量不足者可给予部分静脉营养。

4．对有症状的红细胞增多症-高黏滞度综合征，并且静脉血红细胞比容＞0.7（70％）者可进行部分换血以降低血细胞比容，从而降低血黏滞度。换血量计算方法如下：

$$换血量(ml)=\frac{实际血细胞比容-预期血细胞比容}{实际血细胞比容}\times 体重(kg)\times 85(新生儿血容量为85ml/kg)$$

预期血细胞比容以0.60为宜，换出血量代以补充生理盐水或新鲜血浆等其他血制品。

【预后】

1．长期预后与病因、宫内受损发生的时间及持续时间及出生后营养状况和环境有关。其围生期死亡率是适于胎龄儿的10～20倍，围生期窒息和合并致命性先天性畸形是引起死亡的两

个首要因素。

2．大部分小于胎龄儿出生后体重增长呈追赶趋势，随后身长也出现快速增长阶段，生后第2年末达到正常水平，体格、智力发育正常。

3．约8％出生体重或身长小于第3个百分位者出现终身生长落后。宫内感染、染色体疾病等所致严重宫内生长迟缓者可能会出现终身生长、发育迟缓和不同程度的神经系统后遗症，如学习、认知能力低下，运动功能障碍、甚至脑瘫等。

4．成年后胰岛素抵抗性糖尿病、脂质代谢病及心血管疾病等发病率高。

【预防】

1．加强孕妇保健，避免一切不利于胎儿宫内生长的因素。

2．加强胎儿宫内监护，及时发现胎儿宫内生长迟缓，并对孕母进行治疗。

3．如有宫内窘迫，应立即行剖宫产。

二、大于胎龄儿

大于胎龄儿（LGA）是指出生体重大于同胎龄平均体重第90百分位以上的新生儿。出生体重＞4kg者称巨大儿，其中有些是健康儿。

【病因】

1．生理性因素　如父母体格高大，或母孕期食量较大、摄入大量蛋白质等。

2．病理性因素　如①母患有未控制的糖尿病；②胰岛细胞增生症；③胎儿患有Rh血型不合溶血症；④先天性心脏病（大血管错位）；⑤Beckwith综合征等。

【临床表现】

1．由于体格较大，易发生难产而引起窒息、颅内出血或各种产伤，如颈丛和臂丛神经损伤、膈神经损伤、锁骨骨折、肝破裂以及头面部挤压伤等。

2．原发疾病的临床表现　①Rh血型不合者有重度高胆红素血症、贫血、水肿、肝脾大；②大血管错位者常有气促、发绀及低氧血症；③糖尿病母亲分娩的婴儿常有早产史，易发生一过性低血糖、肺透明膜病、高胆红素血症、红细胞增多症等；④胰岛细胞增生症有持续性高胰岛素血症及顽固性低血糖；⑤Beckwith综合征患儿面容特殊，如突眼、大舌、面部扩张的血管痣、耳有裂纹等，另外还有内脏大、脐疝、低血糖症等。

3．远期并发症　肥胖、Ⅱ型糖尿病发生率远高于适于胎龄儿。

【治疗】

1．预防难产和窒息。

2．治疗各种原发疾病及其并发症。

第四节　新生儿窒息

新生儿窒息（asphyxia of newborn）是指婴儿出生后无自主呼吸或呼吸抑制而导致低氧血症和混合性酸中毒，是引起新生儿死亡和儿童伤残的重要原因之一。由于诊断标准未完全统一，国内文献报道的发病率差异很大。

【病因】

窒息的本质是缺氧，凡是影响胎盘或肺气体交换的因素均可引起窒息。可出现于妊娠期，但绝大多数出现于产程开始后。新生儿窒息多为胎儿窒息（宫内窘迫）的延续。

1．孕母因素　①孕母有慢性或严重疾病，如心、肺功能不全、严重贫血、糖尿病、高血压等；②妊娠并发症：妊娠高血压综合征；③孕妇吸毒、吸烟或被动吸烟、年龄≥35岁或<16岁及多胎妊娠等。

2．胎盘因素　前置胎盘、胎盘早剥和胎盘老化等。

3．脐带因素　脐带脱垂、绕颈、打结、过短或牵拉等。

4．胎儿因素　①早产儿或巨大儿；②先天性畸形：如食道闭锁、喉蹼、肺发育不全、先天性心脏病等；③宫内感染；④呼吸道阻塞：羊水、黏液或胎粪吸入等。

5．分娩因素　头盆不称、宫缩乏力、臀位，使用高位产钳、胎头吸引、臀位抽出术，产程中麻醉药、镇痛药或催产药使用不当等。

【病理生理】

1．窒息时胎儿向新生儿呼吸、循环的转变受阻　正常胎儿向新生儿呼吸、循环系统转变的特征为：①胎儿肺液从肺中清除；②表面活性物质分泌；③肺泡功能残气量建立；④肺循环阻力下降，体循环阻力增加，导致动脉导管和卵圆孔功能性关闭。窒息时新生儿呼吸停止或抑制，致使肺泡不能扩张，肺液不能清除；缺氧、酸中毒引起表面活性物质产生减少、活性降低，以及肺血管阻力增加，胎儿循环重新开放、持续性肺动脉高压。后者可进一步造成组织严重缺氧、缺血、酸中毒，最后导致不可逆器官损伤。

2．窒息时各器官缺血缺氧改变　窒息开始时，缺氧和酸中毒引起机体产生经典的"潜水"反射，即体内血液重新分布，肺、肠、肾、肌肉和皮肤等非生命器官血管收缩，血流量减少，以保证脑、心和肾上腺等生命器官的血流量。同时血浆中促肾上腺皮质激素、糖皮质激素、儿茶酚胺、精氨酸加压素、肾素、心钠素等分泌增加，使心肌收缩力增强，心率增快，心排出量增加，以及外周血压轻度上升，心、脑血流灌注得以维持。如低氧血症持续存在，无氧代谢使代谢性酸中毒进一步加重，体内储存糖原耗尽，脑、心肌和肾上腺的血流量也减少，心肌功能受损，心率和动脉血压下降，生命器官供血减少，脑损伤发生。非生命器官血流量则进一步减少而导致各脏器受损。

3．呼吸改变

(1)原发性呼吸暂停（primary apnea）：胎儿或新生儿缺氧初期，呼吸代偿性加深加快，如缺氧未及时纠正，随即转为呼吸停止、心率减慢，即原发性呼吸暂停。此时患儿肌张力存在，血压稍升高，伴有紫绀。此阶段若病因解除，经清理呼吸道和物理刺激即可恢复自主呼吸。

（2）继发性呼吸暂停（secondary apnea）：若缺氧持续存在，则出现几次喘息样呼吸，继而出现呼吸停止，即继发性呼吸暂停。此时肌张力消失，苍白，心率和血压持续下降，此阶段需正压通气方可恢复自主呼吸，否则将死亡。

临床上有时难以区分原发性和继发性呼吸暂停，为不延误抢救，均可按继发性呼吸暂停处理。

4．血液生化和代谢改变

（1）PaO_2↓、 pH↓及混合性酸中毒：为缺氧后无氧代谢、气道阻塞所致。

（2）糖代谢紊乱：窒息早期儿茶酚胺及高血糖素释放增加，血糖正常或增高，继之糖原耗竭而出现低血糖。

（3）高胆红素血症：酸中毒抑制胆红素与白蛋白结合，降低肝脏酶活力，使未结合胆红素增加。

（4）低钠血症和低钙血症：由于心钠素和抗利尿激素分泌异常，发生稀释性低钠血症；钙通道开放、钙泵失灵、钙内流引起低钙血症。

【临床诊断及窒息诊断】

1．胎儿宫内窒息 早期有胎动增加，胎心率≥160次/分；晚期则胎动减少，甚至消失，胎心率<100 次份；羊水胎粪污染。

2．新生儿窒息　Apgar评分系统在1953年由麻醉科医生Apgar博士提出，是一种简易的、临床上评价刚出生婴儿情况和复苏是否有效的可靠指标。内容包括皮肤颜色（appearance）、心率（pulse）、对刺激的反的反应（grimace）、肌张力（activity）和呼吸（respiration）五项指标；每项0～2分，总共10分（表7-4）。分别于生后1分钟、5分钟和10分钟进行，如婴儿需复苏，15、20分钟仍需评分。以往把Apgar评分作为判断窒息的标准，即8～10分为正常，4～7分为轻度窒息，0～3分为重度窒息。但近年来，国内外许多学者认为，单独的Apgar评分不应作为评估低氧或产时窒息以及神经系统预后的唯一指标，尤其是早产儿或存在其他严重疾病时。因此，美国儿科学会（AAP）和妇产科学会（ACOG）1996年共同制订了以下窒息诊断标准：①脐动脉血显示严重代谢性或混合性酸中毒，pH＜7；②Apgar评分0～3分，并且持续时间＞5分钟；③有神经系统表现，如惊厥、昏迷或肌张力低；④多脏器受损。

表7-4　新生儿Apgar评分标准

体征	评分标准			评分	
	0	1	2	1分钟	5分钟
皮肤颜色	青紫或苍白	身体红，四肢青紫	全身红		
心率（次/分）	无	＜100	＞100		
弹足底或插鼻管反应	无反应	有些动作，如皱眉	哭，喷嚏		
肌张力	松弛	四肢略屈曲	四肢活动		
呼吸	无	慢，不规则	正常，哭声响		

3．多脏器受损症状　缺氧缺血可造成多器官受损，但不同组织细胞对缺氧的易感性各异，其中脑细胞最敏感，其次为为心肌、肝和肾上腺；而纤维、上皮及骨骼肌细胞耐受性较高，因此各器官损伤发生的频率和程度则有差异。①中枢神经系统：缺氧缺血性脑病和颅内出血；②呼吸系统：羊水或胎粪吸入综合征、肺出血以及急性肺损伤或急性呼吸窘迫综合征等；③心血管系统：持续性肺动脉高压、缺氧缺血性心肌损害，后者表现为心律紊乱、心力衰竭、心源性休克等；④泌尿系统：肾功能不全、衰竭及肾静脉血栓形成等；⑤代谢方面：低血糖或高血糖、低血钙及低钠血症等；⑥消化系统：应激性溃疡、坏死性小肠结肠炎及黄疸加重或时间延长等；⑦血液系统：DIC（常在生后数小时或数天内出现）；血小板减少（骨髓缺血性损伤可致骨髓抑制5～7天后可逐渐恢复）。上述疾病的临床表现详见相关章节。

【辅助检查】

对宫内缺氧胎儿，可通过羊膜镜了解羊水胎粪污染程度或胎头露出宫口时取头皮血行血气分析，以评估宫内缺氧程度；生后应检测动脉血气、血糖、电解质、血尿素氮和肌酐等生化指标。

【治疗与预防】

生后应立即进行复苏及评估，而不应延迟至1分钟Apgar评分后进行，并由产、儿科医生、助产士（师）及麻醉师共同协作进行。

1．复苏方案　采用国际公认的ABCDE复苏方案。①A（airway）清理呼吸道；②B（breathing）建立呼吸；③C（circulation）维持正常循环；④D（drugs）药物治疗；⑤E（evaluation）评估。前三项最重要，其中A是根本，B是关键，评估贯穿于整个复苏过程中。呼吸、心率和皮肤颜色是窒息复苏评估的三大指标，并遵循：评估→决策→措施程序，如此循环往复，直到完成复苏。

应严格按照A→B→C→D步骤进行复苏，其步骤不能颠倒。大多数经过A和B步骤即可复苏，少数则需要A、B及C步骤，仅极少数需A、B、C及D步骤才可复苏。

2．复苏步骤和程序（图7-2）　根据ABCDE复苏原则，具体复苏步骤和程序如下：

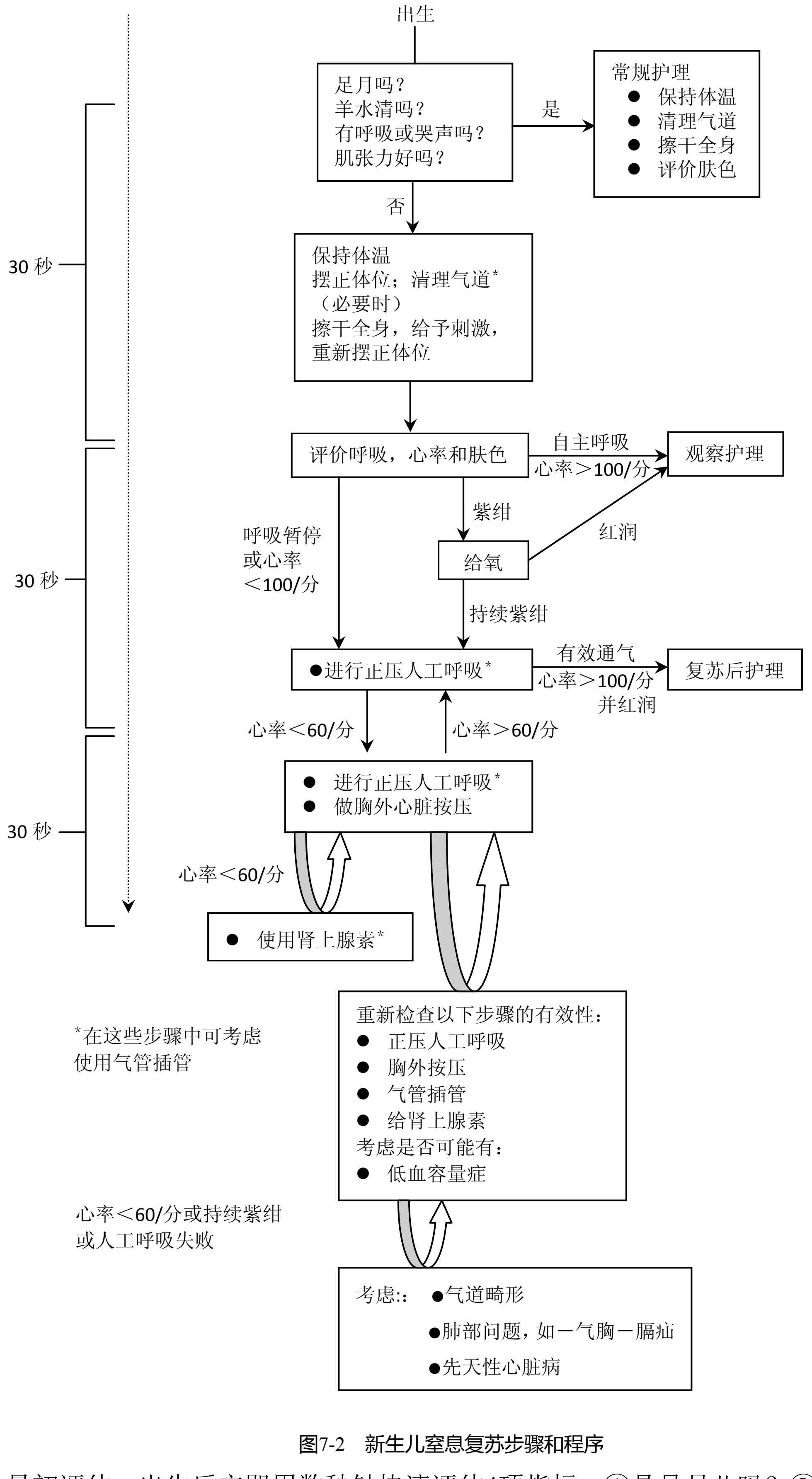

图7-2　新生儿窒息复苏步骤和程序

（1）最初评估：出生后立即用数秒钟快速评估4项指标：①是足月儿吗？②羊水清吗？③有呼吸或哭声吗？④肌张力好吗？如以上任何1项为“否”，则进行以下初步复苏。

（2）初步复苏步骤：①保暖：新生儿娩出后立即置于预热的开放式抢救台上，设置腹壁温度为36.5℃；②摆好体位：置新生儿头轻微伸仰位(图7-3)；③清理呼吸道：肩娩出前助产者

用手挤捏新生儿的面、颏部，排出其口、咽、鼻中的分泌物。新生儿娩出后，立即用吸球或吸管，先口咽，后鼻腔，吸净口、咽和鼻腔的黏液。如羊水混有较多胎粪，且新生儿无活力，在婴儿呼吸前，应做气管插管，将胎粪吸出。如羊水清或羊水污染但新生儿有活力（有活力的定义：呼吸规则、肌张力好及心率＞100次/分），则可以不进行气管内吸引。④擦干：用温热干毛巾快速揩干全身。⑤刺激：用手拍打或手指弹患儿的足底或摩擦背部2次以诱发自主呼吸。以上步骤应在30秒内完成。

（3）气囊面罩正压人工呼吸（图7-4、图7-5和图7-6）：如新生儿仍呼吸暂停或抽泣样呼吸：心率＜100次/分；或持续性中心性青紫，应立即应用100%的氧进行正压通气（目前证据还不足以证明空气复苏与100%氧同样成功）。最初的几次正压人工呼吸需要30～40cm H_2O（1cm H_2O＝0.098 kPa），以后维持在20cm H_2O；频率40～60次/分（胸外按压时为30次/分）；以心率增加接近正常、胸廓起伏、听诊呼吸音正常为宜。经30秒充分正压人工呼吸后，如有自主呼吸，再评估心率，如心率＞100次/分，可逐步减少并停止正压人工呼吸。如自主呼吸不充分，或心率＜100次/分，须继续用气囊面罩或气管插管正压通气。

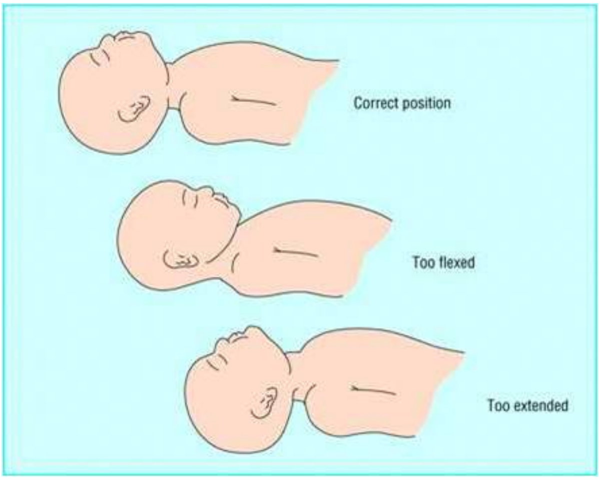

图 7-3　摆好体位

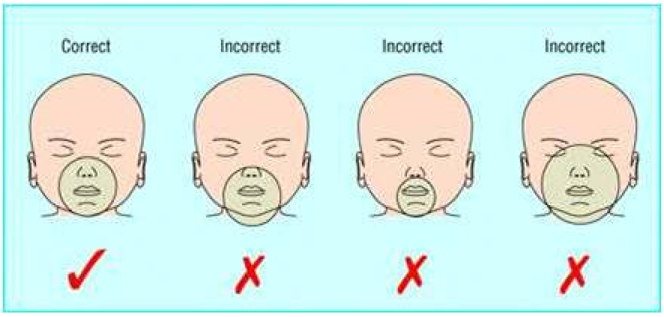

图 7-4　面罩正压通气

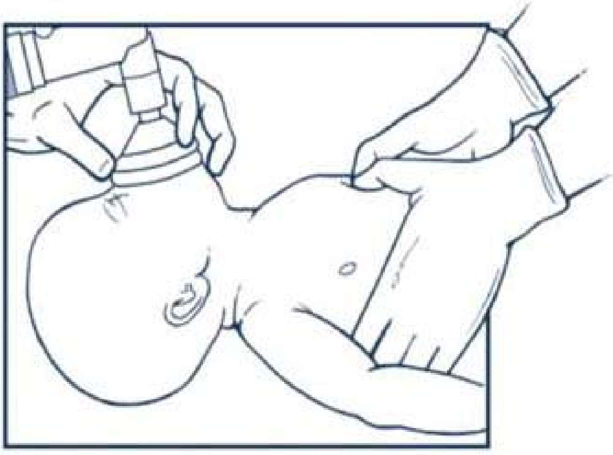

图 7-5　复苏气囊面罩正压通气，双拇指胸外心脏按压

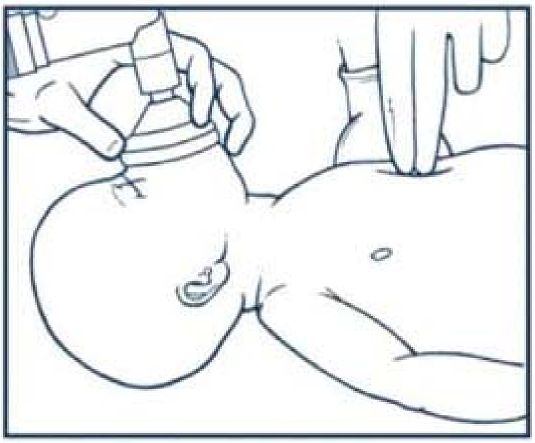

图 7-6　复苏气囊面罩正压通气，中、示指胸外心脏按压

（4）胸外心脏按压：如无心率或气管插管正压通气30秒后心率持续＜60次/分，应同时进行胸外心脏按压。用双拇指见图7-5，或中示指见图7-6，按压胸骨体下1/3处，频率为90次/分（每按压3次，正压通气1次），按压为深度为胸廓前后径的1/3。

（5）药物治疗：①肾上腺素：经100%氧充分正压人工呼吸、同时胸外按压30秒后，心率仍<60次/分，应立即给予1：10000肾上腺素0.1～0.3ml/kg，脐静脉导管内注入或气管导管内注

入，剂量为0.3～1ml/kg，5分钟后可重复一次。②扩容剂：给药30秒后，如心率<100次/分，并有血容量不足表现时，给予生理盐水，剂量为每次l0ml/kg，于10分钟以上静脉缓慢输注。大量失血需输入与新生儿交叉配血阴性的同型血。③碳酸氢钠：在复苏过程中一般不鼓励使用碳酸氢钠，如经上述处理无效，且确定有严重代谢性酸中毒，可给予5％碳酸氢钠3～5ml/kg，加等量5％葡萄糖液，缓慢静脉推注(>5～10分)。④纳洛酮（naloxone）：仅用于正压人工呼吸使心率和肤色恢复正常后仍出现严重的呼吸抑制，并且其母产前4～6小时有注射麻醉药史的新生儿，每次0.1mg/kg，静脉或气管内注入，间隔0.5～1小时可重复1～2次（注意：母亲疑有吸毒者或持续使用美沙酮的新生儿不可用纳洛酮，否则会导致新生儿严重惊厥）。

3．复苏后监护与转运　复苏后仍需监测体温、呼吸、心率、血压、尿量、肤色及窒息引起的多器官损伤。如并发症严重，需转运到NICU治疗，转运中需注意保温、监护生命指标和予以必要的治疗。

【预后】

窒息持续时间对婴儿预后起关键的作用。因此，慢性宫内窒息、重度窒息复苏不及时或方法不当者预后可能不良。

【预防】

1．加强围产期保健，及时处理高危妊娠。

2．加强胎儿监护，避免宫内胎儿缺氧。

3．推广ABCDE复苏技术，培训产、儿科医护人员。

4．各级医院产房内需配备复苏设备。

5．每个分娩都应有掌握复苏技术的人员在场。

第五节 新生儿缺氧缺血性脑病

新生儿缺氧缺血性脑病（hypoxic-ischemic encephalopathy，HIE）是指各种围生期窒息引起的部分或完全缺氧、脑血流减少或暂停而导致胎儿或新生儿脑损伤。早产儿发生率明显高于足月儿，但由于足月儿在活产新生儿中占绝大多数，故以足月儿多见。HIE 是引起新生儿急性死亡和慢性神经系统损伤的主要原因之一。

【病因】

缺氧是发病的核心，其中围生期窒息是最主要的病因。另外，出生后肺部疾患，心脏病变及严重失血或贫血也可引起脑损伤。

【发病机制】

1．脑血流改变　当缺氧缺血为部分性或慢性时，体内血液出现代偿性重新分配，以保证小脑的血液供应。随着缺氧时间延长，这种代偿机制丧失，脑血流最终因心功能受损、全身血压下降而锐减，遂出现第2次血流重新分配，大脑半球血流减少，以保证代谢最旺盛部位，如基底神经节、脑干、丘脑及小脑的血液供应。而大脑皮层矢状旁区及其下部的白质（大脑前、中、后动脉的边缘带）最易受损。如窒息为急性完全性，则上述代偿机制不会发生，脑损伤可发生在基底神经节等代谢最旺盛的部位，而大脑皮层不受影响，甚至其他器官也不会发生缺血损伤。这种由于脑组织内在特性的不同而具有对损害特有的高危性称选择性易损区（selective vulnerability），足月儿的易损区在大脑矢状旁区的脑组织；早产儿的易损区则位于脑室周围的白质区。缺氧和高碳酸血症还可导致脑血管自主调节功能障碍，形成“压力被动性脑血流”，即脑血流灌注完全随全身血压的变化而波动。当血压高时，脑血流过度灌注可致颅内血管破裂出血；当血压下降、脑血流减少，则引起缺血性脑损伤。

2．脑组织代谢改变　葡萄糖是人类脑组织能量的最主要来源。但脑组织储存糖原很少。在正常情况下，85％～95％脑组织能量由葡萄糖氧化而来，仅5％～15％的葡萄糖通过无氧酵解转化为乳酸。有氧代谢时每分子葡萄糖产能是无氧酵解时的19倍。缺氧时，由于脑组织无氧酵解增加，组织中乳酸堆积、能量产生急剧减少，最终因能量衰竭，出现一系列使损害进一步恶化而导致脑细胞死亡的瀑布样反应：①细胞膜上钠-钾泵、钙泵功能不足，使Na^{+}、水进入细胞内，造成细胞毒性脑水肿；②Ca^{2+}通道开启异常，大量Ca^{2+}进入细胞内导致脑细胞不可逆的损害，同时还可激活某些受其调节的酶，引起胞浆膜磷脂成分分解，从而进一步破坏脑细胞膜的完整性及通透性；③当脑组织缺血时，ATP降解，腺苷转变为次黄嘌呤，当脑血流再灌注期重新供氧，次黄嘌呤在次黄嘌呤氧化酶作用下产生氧自由基；④能量持续衰竭时，兴奋性氨基酸尤其是谷氨酸在细胞外聚积产生毒性作用，进一步诱发上述生化反应，引起细胞内Ca^{2+}超载，自由基生成增多，以及脑血流调节障碍等陆续发生，最终导致细胞水肿、凋亡和坏死。

【病理】

病变的范围和分布主要取决于损伤时脑成熟度、严重程度及持续时间。①脑水肿：为其早期主要的病理改变；②选择性神经元死亡〖包括凋亡和坏死〗及梗死：足月儿主要病变在脑灰质，包括脑皮质（呈层状坏死）、海马、基底节、丘脑、脑干和小脑半球，后期表现为软化、多囊性变或瘢痕形成；③出血：包括脑室、原发性蛛网膜下腔、脑实质出血；④早产儿主要表现为脑室周围白质软化和脑室周围室管膜下-脑室内出血。

【临床表现】

根据意识、肌张力、原始反射改变、有无惊厥、病程及预后等，临床上分为轻、中、重度(表7-5)。

急性损伤、病变在两侧大脑半球者，症状常发生在生后24小时内，其中50％～70％可发生惊厥，特别是足月儿。惊厥最常见的表现形式为轻微发作型或多灶性阵挛型，严重者为强直型，同时有前囟隆起等脑水肿症状体征。病变在脑干、丘脑者，可出现中枢性呼吸衰竭、瞳孔缩小或扩大、顽固性惊厥等脑干症状，常在24～72小时病情恶化或死亡。少数患儿在宫内已发生缺血缺氧性脑损伤，出生时Apagar评分可正常，多脏器受损不明显，但生后数周或数月逐渐出现神经系统受损症状。

表7-5　HIE临床分度

临床表现	分度		
	轻度	中度	重度
意识	兴奋	嗜睡	昏迷
肌张力	正常	减低	松软
原始反射			
拥抱反射	活跃	不完全	消失
吸吮反射	正常	减弱	消失
惊厥	可有肌阵挛	常有	多见，频繁发作
中枢性呼吸衰竭	无	有	严重
瞳孔改变	正常或扩大	常缩小、对光反射迟钝	不对称或扩大
前囟张力	正常	正常或稍饱满	饱满、紧张
病程及预后	症状在72小时内消失，预后好	症状在14天内消失，可能有后遗症	症状可持续数周，病死率高，存活者多有后遗症

【辅助检查】

1．血清肌酸磷酸激酶同工酶（creatine kinase，CPK-BB） 正常值<10U/L，脑组织受损时升高。

2，神经元特异性烯醇化酶（neuron-specific enolase，NSE） 正常值<6μg /L，神经元受损时血浆中此酶活性升高。

3．腰椎穿刺 无围生期窒息史，需要排除其他疾病引起的脑病时可行腰椎穿刺，应行脑脊液常规、生化及脑特异性肌酸激酶检测。

4．B 超 具有无创、价廉、可在床边操作和进行动态随访等优点，对基底神经节、脑室及其周围出血具有较高的特异性，但对皮质损伤不敏感。

5．CT扫描 有助于了解颅内出血范围和类型，但对于HIE的诊断仅作为参考，尤其是后颅凹病变。最适宜检查时间为生后2～5 天。

6．核磁共振(MRl) 对脑灰、白质的分辨率异常清晰，且轴位、矢状位及冠状位三维成像，能清晰显示B超或CT不易探及的部位，对于足月儿和早产儿脑损伤的判断均有较强的敏感性。弥散加权磁共振（diffusion weighted imaging，DWI）对显示脑梗死则具有较高的敏感性和特异性。

7．氢质子磁共振波谱（^{1}HMRS） 可在活体上直接检测脑内代谢产物的变化，有助于早产儿和足月儿脑损伤的早期诊断。

8．脑电图 可客观地反应脑损害程度、判断预后，以及有助于惊厥的诊断。在生后1周内检查，表现为脑电活动延迟、异常放电、背景活动异常（以低电压和爆发抑制为主）等。

【诊断】

由中华医学会儿科学会新生儿学组制定的足月儿HIE诊断标准如下：①有明确的可导致胎儿宫内窘迫的异常产科病史，以及严重的胎儿宫内窘迫表现（胎心率＜100次/分，持续5分钟以上和（或）羊水Ⅲ度污染），或在分娩过程中有明显窒息史；②出生时有重度窒息，指Apgar评分1分钟≤3分，并延续至5分钟时仍≤5分；或出生时脐动脉血气pH≤7；③出生后不久出现神经系统症状，并持续24小时以上；④排除电解质紊乱、颅内出血和产伤等原因引起的抽搐，以及宫内感染、遗传代谢性疾病和其他先天性疾病所引起的脑损伤。同时具备以上4条者可确诊，第4条暂时不能确定者可作为拟诊病例。目前尚无早产儿HIE诊断标准。

【治疗】

1．支持疗法 ①维持良好的通气功能是支持疗法的核心，保持PaO_2>7.98～10.64kPa（60～80mmHg）、$PaCO_2$和pH在正常范围。可酌情予以不同方式的氧疗，严重者可用机械通气、NO吸入，但应避免PaO_2过高或$PaCO_2$过低。②维持脑和全身良好的血液灌注是支持疗法的关键措施，避免脑灌注过低或过高。低血压可用多巴胺，也可同时加用多巴酚丁胺。③维持血糖在正常高值（4.16～5.55mmol/L，75～100mg/dl），以提供神经细胞代谢所需能源。

2．控制惊厥 首选苯巴比妥，负荷量20mg/kg，于15～30分钟静脉滴入，若不能控制惊厥，1小时后可加10mg/kg。12～24小时后给维持量，每日3～5mg/kg。肝功能不良者改用苯妥英钠；顽固性抽搐者加用地西泮，每次0.1～0.3mg/kg静脉滴注；或加用水合氯醛50mg/kg。

3．治疗脑水肿 避免输液过量是预防和治疗脑水肿的基础，每日液体总量不超过60～80ml/kg。颅内压增高时，首选利尿剂呋塞米，每次0.5～lmg/kg，静注；严重者可用20％甘露

醇，每次0.25～0.5g/kg，静注，每4～6小时1次，连用3～5天。一般不主张使用糖皮质激素。

4．新生儿期后治疗　病情稳定后尽早智能和何勇的康复训练，有利于促进脑功能恢复，减少后遗症。

【预后和预防】

本病预后与病情严重程度、抢救是否正确及时有关。病情严重，惊厥、意识障碍、脑干症状持续时间超过1周，血清CPK-BB和脑电图持续异常者预后差。幸存者常留有不同程度的运动和智力障碍、癫痫等后遗症。积极推广新法复苏，防止围生期窒息是预防本病的主要方法。

第六节　新生儿颅内出血

新生儿颅内出血（intracranial haemorrhage of the newborn）是新生儿期最严重的脑损伤，早产儿多见，病死率高，存活者常留有神经系统后遗症。

【病因与发病机制】

1．早产 胎龄32周以下的早产儿，在脑室周围的室管膜下及小脑软脑膜下的颗粒层均留存胚胎生发基质（germinal matrix，GM）。该组织是一未成熟的毛细血管网，其血管壁仅有一层内皮细胞、缺少胶原和弹力纤维支撑。当动脉压突然升高时可导致毛细血管破裂引起室管膜下出血；出血向内可穿破室管膜进入脑室内引起脑室内出血；血液外渗可扩散至脑室周围的白质。GM层血管壁内皮细胞富含线粒体，耗氧量大，对缺氧十分敏感，易引起血管壁破坏出血。此处小静脉系统呈“U”字形走向汇于Galen静脉，由于这种特殊走向，易发生血流动力学的变化而致出血及出血性脑梗死。胎龄32周以后GM层逐步退化形成神经胶质细胞，构成生后脑白质的基础。

2．缺血缺氧窒息时低氧血症、高碳酸血症可损害脑血流的自主调节功能，形成压力被动性脑血流。当动脉压力升高时，脑血流量增加，可引起毛细血管破裂出血；当动脉压力降低时，脑血流量减少，引起毛细血管缺血性损伤而出血；低氧、高碳酸血症还可引起脑血管扩张，血管内压增加，毛细血管破裂出血；或静脉淤滞、血栓形成，脑静脉血管破裂出血。

3．外伤　主要为产伤所致。如胎位不正、胎儿过大、产程延长等使胎儿头部过分受压，或使用高位产钳、胎头吸引器、急产、臀牵引等机械性损伤均可使天幕、大脑镰撕裂和脑表浅静脉破裂而导致硬膜下出血。其他如头皮静脉穿刺、吸痰、搬动、气管插管等频繁操作或机械通气时呼吸机参数设置不当等可造成头部过分受压、脑血流动力学突然改变和脑血流自主调节受损引起毛细血管破裂而出血。

4．其他　新生儿肝功能不成熟，凝血因子不足或患其他出血性疾病，如母亲患原发性血小板减少性紫癜或孕期使用苯妥英钠、苯巴比妥、利福平等药物可引起新生儿血小板或凝血因子减少；不适当地输入碳酸氢钠、葡萄糖酸钙、甘露醇等高渗溶液，可导致毛细血管破裂。

【临床表现】

主要与出血部位和出血量有关，轻者可无症状，大量出血者可在短期内死亡。常见的症状与体征有：①神志改变：激惹、嗜睡或昏迷；②呼吸改变：增快或减慢，不规则或暂停；③颅内压力增高：前囟隆起，血压增高，抽搐，角弓反张，脑性尖叫；④眼征：凝视、斜视、眼球上转困难、眼球震颤等；⑤瞳孔对光反应消失；⑥肌张力：增高、减弱或消失；⑦其他：不明原因的苍白、贫血和黄疸。

根据出血部位不同，临床上分为以下几型：

1．脑室周围-脑室内出血（periventricular-intraventricular haemorrhage，PVH-IVH） 是新生儿颅内出血中常见的一种类型。主要见于胎龄小于32周、体重低于1500g的早产儿，其发病率可达40％～50％，胎龄愈小，发病率愈高，是引起早产儿死亡的主要原因之一。2％～3％的足月儿也可发生PVH-IVH，其中50％～60％出血来自室管膜下GM，其余则源于脉络丛。根据头颅B超或CT检查分为4级：Ⅰ级：室管膜下出血；Ⅱ级：脑室内出血但无脑室扩大；Ⅲ级：脑室内出血伴脑室扩大；Ⅳ级：脑室内出血伴脑实质出血。出血发生的时间50％在出生后第1天，90％发生在出生后72小时内，仅少数发病会更晚。Ⅰ～Ⅱ级出血绝大部分存活；Ⅲ～Ⅳ级出血者50％以上死亡，幸存者半数以上遗留神经系统后遗症。

2．原发性蛛网膜下腔出血（primary subarachoid haemorrhage，SAH） 出血原发部位在蛛网膜下腔内，不包括硬膜下、脑室内或小脑等部位出血后向蛛网膜下腔扩展。此种出血类型在新生儿十分常见，尤其是早产儿。SAH与缺氧、酸中毒、产伤有关。由于出血原因常为缺氧引起蛛网膜下的毛细血管内血液外渗，而非静脉破裂，故大多数出血量少，无临床症状，预后良好。部分典型病例表现为生后第2天抽搐，但发作间歇表现正常；极少数病例大量出血常于短期内死亡。主要的后遗症为交通性或阻塞性脑积水。

3．脑实质出血（intraparenchymal haemorrhage，IPH） 多因小静脉栓塞后使毛细血管压力增高、破裂而出血。由于出血部位和量不同，临床症状有很大差异。如出血部位在脑干，早期可发生瞳孔变化、呼吸不规则和心动过缓等，前囟张力可不高。主要后遗症为脑性瘫痪、癫痫和精神发育迟缓。由于支配下肢的神经传导束邻近侧脑室，向外依次为躯干、上肢、面部神经的传导束，因此下肢运动障碍较多见。出血部位可液化形成囊肿，如囊肿与脑室相通称之为脑穿通性囊肿（porencephalic cysts）。

4．硬膜下出血（subdural hemorrhage，SDH） 是产伤性颅内出血最常见的类型，多见于足月巨大儿。近年来由于产科技术提高，其发生率已明显下降。出血量少者可无症状；出血明显者一般在出生24小时后出现惊厥、偏瘫和斜视等神经系统症状。严重的天幕、大脑镰撕裂和大脑表浅静脉破裂可在出生后数小时内死亡。也有在新生儿期症状不明显，而至数月后发生慢性硬脑膜下积液。

5．小脑出血（eerebellar hemorrhage，CH） 包括原发性小脑出血，脑室内或蛛网膜下腔出血扩散至小脑，静脉出血性梗死，及产伤引起小脑撕裂4种类型。多见于胎龄小于32周、体重低于1500g的早产儿，或有产伤史的足月儿。严重者除一般神经系统症状外主要表现为脑干症状，如频繁呼吸暂停、心动过缓等，可在短时间内死亡。预后较差，尤其是早产儿。

【诊断】

病史、症状体征可提供诊断线索，但确诊需靠头颅影像学检查。头颅B超对颅脑中心部位病变分辨率高，因此成为PVH-IVH的特异性诊断手段，应为首选，并在生后3～7天进行，1周后动态监测。但蛛网膜下腔、后颅窝和硬膜外等部位出血B超不易发现，需CT、MRI确诊。脑脊液检查可与其他引起中枢神经系统症状的疾病鉴别。颅内出血时显微镜下可见皱缩红细胞，蛋白含量明显升高，严重者在出血后24小时内脑脊液糖含量降低，5～10天最明显，同时乳酸含量低。

【治疗】

1．支持疗法 保持患儿安静，尽可能避免搬动、刺激性操作，维持正常的PaO_2、$PaCO_2$、pH、渗透压及灌注压。

2．止血 可选择使用维生素K_1、酚磺乙胺（ethamsylate）、立止血（reptilase）等。

3．控制惊厥 见缺血缺氧性脑病节。

4．降低颅内压　有颅内压力增高症状者可用呋塞米，每次0.5～lmg/kg，每日2～3次静注。对中枢性呼吸衰竭者可用小剂量甘露醇，每次0.25～0.5g/kg，每6～8小时1次，静注。

5．脑积水　乙酰唑胺（acetazolamide）　可减少脑脊液的产生，每日50～l00mg/kg，分3～4次口服；对脑室内或蛛网膜下腔出血可于病情稳定后（生后2周左右）连续腰椎穿刺，每日或隔日1次，防止粘连和脑积水，但对此法尚存在争议。梗阻性脑积水上述治疗多无效，可行脑室-腹腔分流术。

【预后】

主要与出血部位、出血量、胎龄及其他围生期因素有关。早产儿、Ⅲ～Ⅳ级PVH-IVH、慢性缺氧、顶枕部脑实质出血预后差，幸存者常留有神经系统后遗症。

【预防】

1．做好孕妇保健工作，避免早产；提高产科技术，减少新生儿窒息和产伤；对患有出血性疾病的孕妇及时给予治疗。

2．提高医护质量，避免各种可能导致医源性颅内出血的因素发生。

（常立文）

第八节 胎粪吸入综合征

胎粪吸入综合征（meconium aspiration syndrome，MAS）是由胎儿在宫内或产时吸入混有胎粪的羊水，而导致以呼吸道机械性阻塞及化学性炎症为主要病理特征，以生后出现呼吸窘迫为主要表现的临床综合征。多见于足月儿或过期产儿。据文献报道，分娩时羊水混胎粪的发生率约为5％～15％，但仅其中5%～10%发生MAS；而MAS中10%～20%患儿并发气胸，5%患儿可死亡。

【病因和病理生理】

1．胎粪吸入　胎儿在宫内或分娩过程中出现缺氧，其肠道及皮肤血液量减少，继之迷走神经兴奋，最终导致肠壁缺血痉挛，肠蠕动增加，肛门括约肌松弛而排出胎粪。同时缺氧使胎儿产生呼吸运动（喘息），将胎粪吸入气管内或肺内，或在胎儿娩出建立有效呼吸后，使其吸入肺内。也有学者根据早产儿很少发生羊水混有胎粪，而过期产儿发生率高于35%这一现象，推断羊水混有胎粪也可能是胎儿成熟的标志之一。

2．不均匀气道阻塞和化学性炎症　MAS的主要病理变化是由于胎粪的机械性阻塞所致。①肺不张：部分肺泡因其小气道被较大胎粪颗粒完全阻塞，其远端肺泡内气体吸收，引起肺不张，使肺泡通气/血流降低，导致肺内分流增加，从而发生低氧血症。②肺气肿：黏稠胎粪颗粒不完全阻塞部分肺泡的小气道，则形成“活瓣”，吸气时小气道扩张，使气体能进入肺泡，呼气时因小气道阻塞，气体不能完全呼出，导致肺气肿，致使肺泡通气量下降，引起CO_2潴留。若气肿的肺泡破裂则发生肺气漏，如间质气肿、纵隔气肿或气胸等。③正常肺泡：部分肺泡的小气道可无胎粪，但该部分肺泡的通换气功能均可代偿性增强。由此可见，MAS的病理特征为不均匀气道阻塞，即肺不张、肺气肿及正常肺泡同时存在，其各自所占的比例决定患儿临床表现的轻重。

因胆盐是胎粪组成之一，故胎粪吸入除引起呼吸道的机械性阻塞外，也可刺激局部引起化学性炎症，进一步加重通换气功能障碍。胎粪尚有利于细菌生长，故MAS也可继发细菌感染。此外，近年来有文献报道，MAS时Ⅱ型肺胞上皮细胞受损和肺表面活性物质减少，但其结论尚

需进一步研究证实。

3．肺动脉高压　严重缺氧和混合性酸中毒导致肺小动脉痉挛，甚至血管平滑肌肥厚（长期低氧血症），导致肺动脉阻力增加，右心压力增加，发生卵圆孔水平右向左分流；肺血管阻力的持续增加，使肺动脉压超过体循环动脉压，从而导致已功能性关闭或尚未关闭的动脉导管发生导管水平的右向左分流，即新生儿持续肺动脉高压（persistent pulmonary hypertension of newborn，PPHN）。上述变化将进一步加重低氧血症及混合性酸中毒，并形成恶性循环。

【临床表现】

1．吸入混胎粪的羊水　是诊断MAS的前提。①分娩时可见羊水混胎粪；②患儿皮肤、脐带和指、趾甲床留有胎粪污染的痕迹；③口、鼻腔吸引物中含有胎粪；④气管插管时声门处或气管内吸引物中可见胎粪（即可确诊）。

2．呼吸系统表现　患儿症状轻重与吸入羊水的物理性状（混悬液或块状胎粪等）和量的多少密切相关。若吸入少量或混合均匀的羊水，可无症状或症状轻微；若吸入大量混有黏稠胎粪羊水者，可致死胎或生后不久死亡。常于生后数小时出现呼吸急促（>60次/分）、发绀、鼻翼扇动和吸气性三凹征等呼吸窘迫表现，少数患儿也可出现呼气性呻吟。体格检查可见胸廓前后径增加，早期两肺有鼾音或粗湿啰音，以后出现中、细湿啰音。如呼吸窘迫突然加重，并伴有呼吸音明显减弱，应怀疑气胸的发生。

3．PPHN　多发生于足月儿，在有文献报道的PPHN患儿中，约75%其原发病是MAS。重症MAS患儿多伴有PPHN。主要表现为严重的发绀，其特点为：当FiO_2＞0.6时，发绀仍不缓解；哭闹、哺乳或躁动时发绀加重；发绀程度与肺部体征不平行（发绀重，体征轻）。部分患儿在胸骨左缘第2肋间可闻及收缩期杂音，严重者可出现休克和心力衰竭。

尽管发绀PPHN的主要临床表现，但常需与青紫型先天性心脏病或严重肺部疾病所导致的发绀相鉴别，故应做如下试验：①高氧试验（hyperoxia test）：吸入纯氧15分钟，如动脉氧分压（PaO_2）或经皮血氧饱和度（$TcSO_2$）较前明显增加，提示为肺实质病变；PPHN和青紫型先心病则无明显增加。②动脉导管前、后血氧差异试验：比较动脉导管前（右桡或颞动脉）和动脉导管后（左桡、脐或下肢动脉）的PaO_2或$TcSO_2$，若动脉导管前、后PaO_2差值＞2kPa（15mmHg）或$TcSO_2$差值＞4%，表明动脉导管水平有右至左分流。若无差值也不能除外PPHN，因为也可有卵圆孔水平的右至左分流。③高氧-高通气试验（hyperoxic hyperventilation test）：应用气管插管纯氧抱球通气，频率60～80次/分，通气10～15分钟，使动脉二氧化碳分压（PaO_2）下降和血pH值上升，若PaO_2较通气前升高>4kPa（30mmHg）或$TcSO_2$升高>8％，则提示PPHN存在。

严重MAS可并发红细胞增多症、低血糖、低钙血症、HIE、多器官功能障碍及肺出血等。

【辅助检查】

1．实验室检查　血气分析：pH值及PaO_2降低，$PaCO_2$增高；血常规、血糖、血钙和相应血生化检查；气管内吸引物及血液的培养。

2．X线检查　两肺透亮度增强伴有节段性或小叶肺不张，也可仅有弥漫性浸润影或并发纵隔气肿、气胸等（图7-7、图7-8）。临床统计尚发现：部分MAS患儿胸片改变不与临床表现成正比，即胸片严重异常者症状却很轻，胸片轻度异常甚或基本正常，症状反而很重。

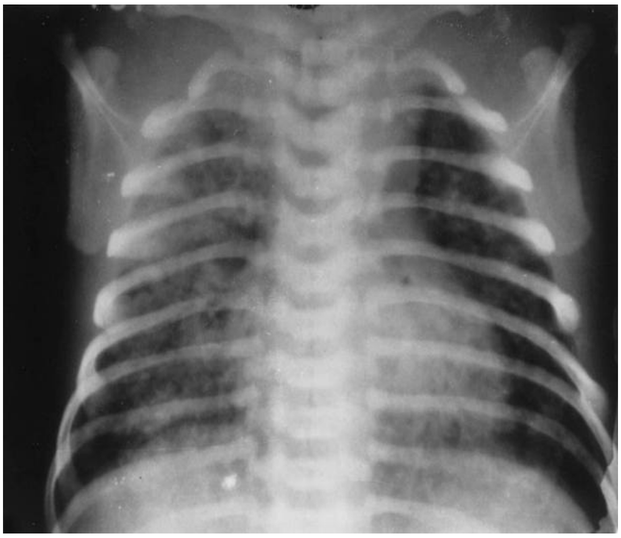

图 7-7　MAS 患儿胸片
双肺纹理增强、模糊，见模糊小斑片影，双肺野透光过度增高

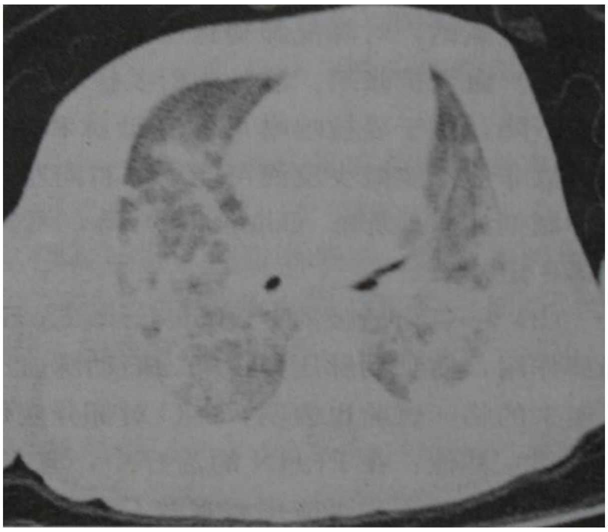

图 7-8　MAS 患儿的肺 CT
双肺纹理增强、模糊，双肺见沿纹理走行散在斑片模糊高密度影，以双肺下叶明显

3．超声波检查　彩色Doppler超声检查有助于PPHN的诊断。

【治疗】

1．促进气管内胎粪排出　为促进气管内胎粪排出，可采用体位引流、拍叩和震动胸部等方法。对病情较重且生后不久的MAS患儿，可气管插管后进行吸引，胎粪黏稠者也可气管内注入0.5ml生理盐水后再行吸引，以减轻MAS的病变程度及预防PPHN发生。此外，动物实验结果表明，即使胎粪进入气道4小时后，仍可将部分胎粪吸出。

2．对症治疗

（1）氧疗：当PaO_2＜8.0kPa（60mmHg）或$TcSO_2$＜90%是，应依据患儿缺氧程度选用鼻导管、面罩或氧气涵等吸氧方式，以维持PaO_2 8.0～10.6kPa（60～80mmHg）或$TcSO_2$ 90％～95％为宜。若患儿已符合上机标准，应尽早机械通气治疗（见本章第八节）。

（2）纠正酸中毒：①纠正呼吸性酸中毒：可经口、鼻或气管插管吸引，保持气道通畅，必要是进行正压通气；②纠正代谢性酸中毒：纠正缺氧，改善循环，当血气结果中碱剩余为-6～-10是地，应在保证通气的前提下予碱性药物。

（3）维持正常循环：出现低体温、苍白和低血压等休克表现者，应用血浆、全血、5％白蛋白或生理盐水等进行扩容，同时静脉点滴多巴胺和（或）多巴酚丁胺等。

（4）其他：①限制液体入量：严重者常伴有脑水肿，肺水肿或心力衰竭，应适当限制液体入量；②抗生素：不主张预防性应用抗生素，但对有继发细菌感染者，根据血、气管内吸引物细菌培养及药敏结果应用抗生素；③肺表面活性物质：目前有应用其治疗MAS的临床报道，但病例数较少，确切疗效尚有待证实；④预防肺气漏：需机械通气病例，PIP和PEEP不宜过高，以免引起气胸等；⑤气胸治疗：应紧急胸腔穿刺抽气，可立即改善症状，然后根据胸腔内气体的多少，可反复胸腔穿刺抽气或行胸腔闭式引流；⑥其他：保温、镇静，满足热卡需要，维持血糖和血钙正常等。

3．PPHN治疗　去除病因至关重要。

（1）碱化血液：是治疗PPHN经典而有效的方法之一。采用人工呼吸机进行高通气，以维持动脉血气：pH值7.45～7.55，$PaCO_2$ 3.3～4.7kPa（25～35 mmHg），PaO_2 10.6～13.3kPa（80～

100mmHg）或$TcSO_2$ 96％～98％，从而降低肺动脉压力。

但应注意，低碳酸血症可减少心搏量和脑血流量，特别是早产儿增加了脑室周围白质软化的发生机会，帮PPHN治疗中应避免造成过度的低$PaCO_2$。此外，静脉应用碱性药物如碳酸氢钠，对降低肺动脉压也有一定疗效。

（2）血管扩张剂：静脉注射妥拉唑啉虽能降低肺动脉压，但也引起体循环压相应或更严重下降，鉴于妥拉唑啉可使肺动脉和体循环压同时下降，其压力差较前无明显改变甚或加大，帮非但不能减少反而可能增加右向左分流，目前目前临床已很少应用。近年来，磷酸二酯酶抑制剂如西地那非（sildenafil）等，可选择性扩张肺血管，被试用于新生儿PPHN，也取得一定疗效。

（3）一氧化氮吸入（inhaled nitric oxide，iNO）：NO是血管舒张因子，由于iNO的局部作用，使肺动脉压力下降，而动脉血压不影响，故不乏是PPHN治疗的选择之一。近年来的临床试验也表明，iNO对部分病例有较好疗效。

（4）其他：在PPHN的治疗中，有报道肺表面活性物质能使肺泡均匀扩张，降低肺血管阻力；关于是否应用激素及CPAP治疗尚存在争议；液体通所尚在试验中；高频震荡通气取得一定效果；体外膜肺（ECMO）对严重MAS（并发PPHN）疗效较好，但人格昂贵，人员及设备要求高。

【预防】

积极防治胎儿宫内窘迫和产时窒息；尽量避免过期产；及时纠正低氧血症和混合性酸中毒对预防PPHN至关重要。

第八节　呼吸窘迫综合征

新生儿呼吸窘迫综合征（respiratory distress syndrome，RDS）又称肺透明膜病（hyaline membrane disease，HMD），是由肺表面活性物质（pulmonary surfactant，PS）缺乏而导致，以生后不久出现呼吸窘迫并呈进行性加重的临床综合征。多见于早产儿，其胎龄愈小，发病率愈高：胎龄36周者仅5％，32周者为25％，28周者达70％，24周者超过80%。

【PS成分与产生】

PS是由Ⅱ型肺泡上皮细胞合成并分泌的一种磷脂蛋白复合物，磷脂约占80%，其中磷脂酰胆碱即卵磷脂（phosphatidyl cholin，PC即lecithin）是起表面活性作用的重要物质。孕18～20周开始产生，继之缓慢上升，35～36周迅速增加达肺成熟水平。其次是磷脂酰甘油（phosphatidylglycerol，PG），26～30周前浓度很低，而后与PC平行升高，36周达高峰，随之下降，足月时约为高峰值的1/2。此外尚有其他磷脂，其中鞘磷脂（sphingomyelin）的含量较恒定，只在28～30周出现小高峰，故羊水或气管吸引物中L/S（lecithin/sphingomyelin）值可作为评价胎儿或新生儿肺成熟度的重要指标。PS中蛋白质约占13%，其中能与PS结合的蛋白质称为表面活性物质蛋白（surfactant protein，SP），包括SP-A、SP-B、SP-C和SP-D等，可与磷脂结合，增加其表面活性作用。PS覆盖在肺泡表面，降低其表面张力，防止呼气末肺泡萎陷，以保持功能残气量（functional residual capacity，FRC），稳定肺泡内压和减少液体自毛细血管向肺泡渗出。

【病因与发病机制】

早产儿胎龄愈小，功能肺泡愈少，气体交换功能愈差；呼吸膜愈厚，气体弥散功能愈差；气管软骨少，气道阻力大；胸廓支撑力差，肺泡不易张开。因此，对于肺解剖结构尚未完善的

早产儿，其胎龄愈小，PS的量也愈低，肺泡表面张力增加，呼气末FRC降低，肺泡趋于萎陷。故其肺功能异常主要表现为肺顺应性下降，气道阻力增加，通气/血流值降低，气体弥散障碍及呼吸功增加，从而导致缺氧和因其所致的代谢性酸中毒及通气功能障碍所致的呼吸性酸中毒。由于缺氧及酸中毒使肺毛细血管通透性增高，液体漏出，使肺间质水肿和纤维蛋白沉着于肺泡表面形成嗜伊红透明膜，进一步加重气体弥散障碍，加重缺氧和酸中毒，并抑制PS合成，形成恶性循环。此外，严重缺氧及混合性酸中毒也可导致PPHN的发生。

糖尿病母亲所娩的婴儿（infant of diabetic mother，IDM）也易发生此病，是由于其血中高浓度胰岛素能拮抗肾上腺皮质激素对PS合成的促进作用，故IDM的RDS发生率比正常增加5～6倍。PS的合成还受体液pH值、体温和肺血流量的影响，因此，围生期窒息、低体温、前置胎盘、胎盘早剥和母亲低血压等所致的胎儿血容量减少，均可诱发RDS。此外，剖宫产儿、双胎的第二婴和男婴，RDS的发生率也较高。

【临床表现】

生后6小时内出现呼吸窘迫，主要表现为：呼吸急促（>60/分）是为增加肺泡通气量，代偿潮气量的减少；鼻扇为增加气道横截面积，减少气流阻力；、吸气性三凹征和明显的呼气呻吟是因呼气时声门不完全开放，使肺内气体潴留产生正压，防止肺泡萎陷；吸气性三凹征是呼吸辅助肌参与的结果，以满足增加的肺扩张压；发绀是由于氧合成不足，常提示动脉血中还原血红蛋白＞50g/L。呼吸窘迫呈进行性加重是本病特点。严重时表现为呼吸浅表，呼吸节律不整、呼吸暂停及四肢松弛。由于呼气时肺泡萎陷，体格检查可见胸廓扁平；因潮气量小而听诊呼吸音减低，肺泡有渗出时可闻及细湿啰音。

随着病情的逐渐好转，由于肺顺应性的改善，肺动脉压力下降，约有30%患儿于恢复期出现动脉导管重新开放。故恢复期的RDS患儿，其原发病已明显好转，突然出现对氧气的需求量增加、难以矫正和解释的代谢性酸中毒、喂养困难、呼吸暂停、周身发凉发花及肝脏在短时间内进行性增大，应注意本病。若同时具备脉压增大，水冲脉，心率增快或减慢，心前区增强，胸骨左缘第2肋间可听到收缩期或连续性杂音，则应确诊本病。

RDS通常于生后第2、3天病情严重，72小时后明显好转。但新生儿的出生体重、肺病变的严重程度、表面活性物质的治疗有否感染的存在及动脉导管的开放等均会对患儿的病程有不同程度的影响。若出生12小时后出现呼吸窘迫，一般不考虑本病。

【辅助检查】

1．实验室检查　①泡沫试验（foam test）：取患儿胃液1 ml加95％酒精1 ml，振荡15秒，静置15分钟后沿管壁有多层泡沫形成则可除外RDS。若无泡沫可考虑为RDS，两者之间为可疑。其机理是由于PS利于泡沫的形成和稳定，而酒精则起抑制作用。②用肺成熟度的判定：测定羊水或患儿气管吸引物中L/S，若≥2提示“肺成熟”，1.5～2为可疑、<1.5提示“肺未成熟”；PS中其他磷脂成分的测定也有助于诊断。③血气分析：pH值和动脉氧分压（PaO_2）降低，动脉二氧化碳分压（$PaCO_2$）增高，碳酸氢根减低是RDS常见改变。

2．X线检查　是目前确诊RDS的最佳手段。①毛玻璃样（ground glass）改变：两肺呈普遍性的透过度降低，可见弥漫性均匀一致的细颗粒网状影（图7-9）；②支气管充气征（air bronchogram）：在弥漫性不张肺泡（白色）的背景下，可见清晰充气的树枝状支气管（黑色）影（图7-11）；③白肺（white out）：严重时双肺野均呈白色，肺肝界及肺心界均消失（图7-10、图7-11）；④肺容量减少（非CPAP或机械通气条件下）。尽管典型病例的胸片有其特异性表现，但动态拍摄X线胸片更有助于鉴别诊断、病情判定、呼吸机参数调整及治疗效果（如应用肺表面活性物质）的评价。

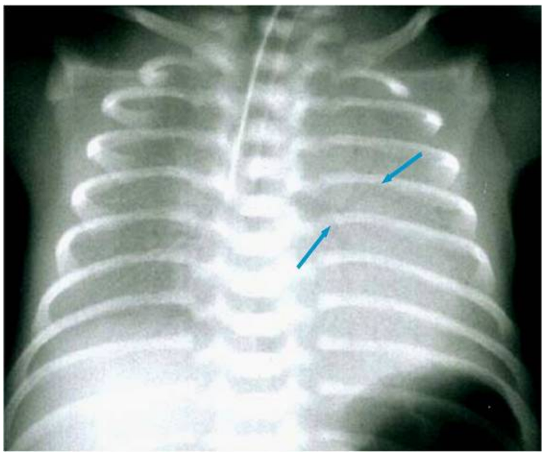

图 7-9 RDS 患儿胸片
双肺野透过度明显降低，呈毛玻璃样改变，
双肺下野仅肺门处见充气支气管

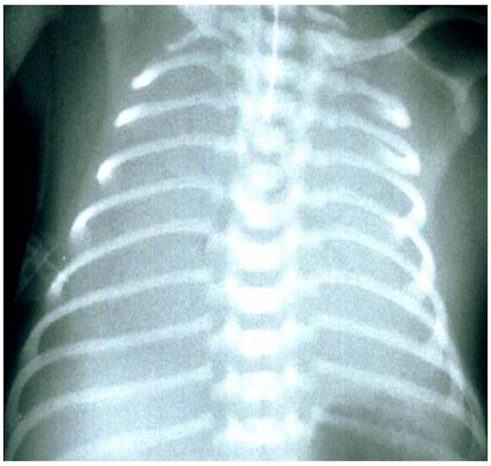

图 7-10 RDS 患儿胸片
双肺野透过度均匀一致性降低，未见正常肺纹理，其内可见含气支气管影。双侧心缘、膈肌及膈角均显示不清

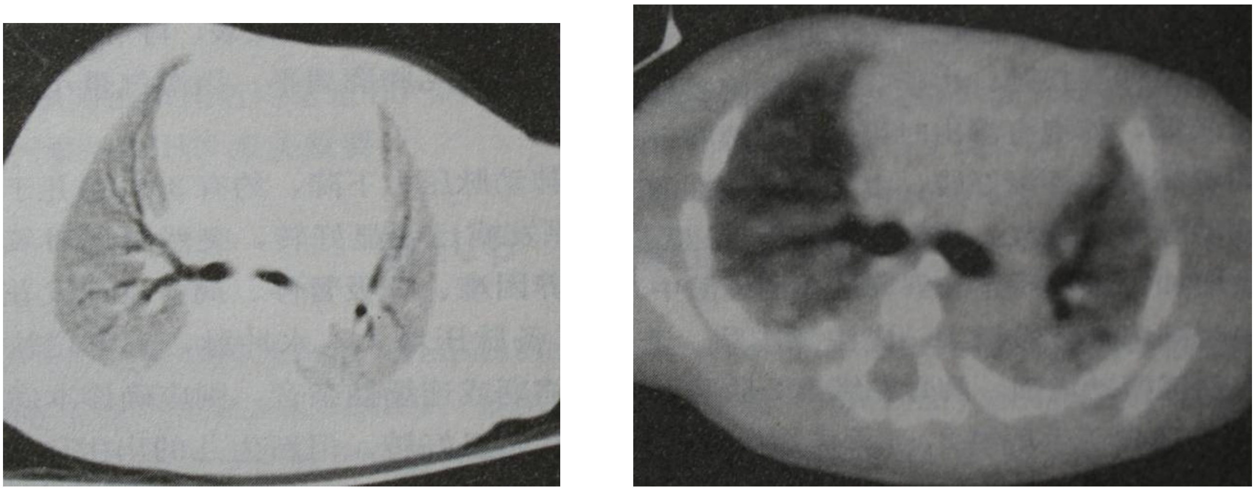

图 7-11 RDS 患儿肺 CT
双肺透过度明显降低，肺组织呈毛玻璃样改变，内含支气管气像，肺纹理模糊

3．超声波检查 彩色Doppler超声有助于动脉导管开放确定和PPHN的诊断。

【诊断和鉴别诊断】

1．湿肺（wet lung） 亦称新生儿暂时性呼吸增快（transient tachypnea of newborn，TTN）。多见于足月儿，为自限性疾病。系肺淋巴或（和）静脉吸收肺液功能暂时低下，使其积留于淋巴管、静脉、间质、叶间胸膜和肺泡等处，影响气体交换。生后数小时内出现呼吸增快（>60～80次/分），但吃奶佳、哭声响亮及反应好，重者也可有发绀及呻吟等。听诊呼吸音减低，可闻及湿啰音。X线胸片显示肺气肿、肺门纹理增粗和斑点状云雾影，常见毛发线（叶间积液）。对症治疗即可。一般2～3天症状缓解消失。

2．B组链球菌肺炎（group B streptococcal pneumonia） 是由B组链球菌败血症所致的宫内感染性肺炎。其临床及X线所见有时与RDS难以鉴别。但前者母亲妊娠晚期多有感染、胎膜前破或羊水有臭味史；母血或宫颈拭子培养有B组链球菌生长；机械通气时所需参数较低；病程与RDS不同。

3．膈疝（diaphragrnatic hernia） 表现为阵发性呼吸急促及发绀。腹部凹陷，患侧胸部呼吸音减弱甚至消失，可闻及肠鸣音；X线胸片可见患侧胸部有充气的肠曲或胃泡影及肺不张，

纵隔向对侧移位。

【治疗】

目的是保证通换气功能正常，待自身PS产生增加，RDS得以恢复。机械通气和PS是治疗的重要手段。

1．一般治疗　①保温：放置在自控式暖箱内或辐射式抢救台上，保持皮肤温度在36.5℃。②监测：体温、呼吸、心率、血压和血气。③保证液体和营养供应：第1天5％或10％葡萄糖液65～75ml/(kg.d)，以后逐渐增加到120～150ml/(kg.d)，并适当补充电解质。病情好转后改为经口喂养，热能不足时辅以部分静脉营养。④纠正酸中毒。⑤抗生素：原则上不主张用，但若合并感染，应依据细菌培养和药敏结果选择相应抗生素。

2．氧疗（oxygen therapy）和辅助通气

（1）吸氧：。轻症可选用鼻导管、面罩、氧气涵或鼻塞吸氧，维持PaO_2 6.7～9.3 kPa（50～70mmHg）和$TcSO_2$ 85％～93％为宜。

（2）持续气道正压（continuous positive airway pressure, CAPA）：①目的是使有自主呼吸的患儿在整个呼吸周期中都接受高于大气压的气体，以增加FRC，防止呼气时肺泡萎陷，以改善肺氧合及减少肺内分流；②指征：吸入氧分数（fraction of inspiratory oxygen, FiO_2）>0.4，PaO_2<50 mmHg或$TcSO_2$<90%；③方法：可经鼻塞、面罩或气管插管进行；④参数：压力4～10cm H_2O，气体流量最低为患儿每分通气量的3倍或5L/min。

CPAP多适用于轻、中度RDS患儿，若其$TcSO_2$或PaO_2已符合上呼吸机指征者，还应尽早给予机械通气治疗。

（3）常频机械通气（conventional mechanical ventilation, CMV）：新生儿最好使用持续气流、时间转换、压力限制型呼吸机。

1）指征：①当FiO_2=0.6，PaO_2<50mmHg或$TcSO_2$<85%（发绀型先心病除外）；②PaO_2>60～70 mmHg伴pH<7.25；③严重或药物治疗无效的呼吸暂停。具备上述一项者即可行机械通气。

2）初始参数：吸气峰压（PIP）20～30cmH_2O；呼气末正压（PEEP）4～6cmH_2O；呼吸频率（RR）20～60bpm；吸气时间（TI）0.3～0.5秒；流量（FR）8～12L/min。15～30分钟后检测动脉血气，依据其结果决定是否需调整参数。

3）并发症：①肺气漏：由于CMV的压力过高所致，包括肺间质气肿、气胸及纵隔积气等；②慢性肺疾病（chronic lung disease, CLD）：又称支气管肺发育不良（BPB），即生后28天或纠正胎龄达36周仍需吸氧并伴有胸片异常者，多因长时间吸入高浓度氧或高送气压力等因素所致；③早产儿视网膜病（retinopathy of prematurity, ROP）：也与长时间吸入高浓度氧有关，重者可失明；④呼吸机相关性肺炎：是由气管插管和使用机后引起的继发感染，常见致病菌有铜绿假单胞菌、克雷伯杆菌、不动杆菌及肠杆菌等。

此外，近年来大样本、多中心的研究表明，当常频机械通气治疗难以奏效时，改用高频震荡呼吸机，已取得较好疗效。

3．PS替代疗法　可明显降低RDS病死率及气胸发生率，同时可改善肺顺应性和通换气功能，降低呼吸机参数。

（1）应用指征：已确诊的RDS或产房同防止RDS的预防性应用。

（2）临床常用的PS：①Survanta：从牛肺中提取，脱脂后加入棕榈酸、PC、甘油三酯而制成，内含SP-B和SP-C；②Exosurf：是人工合成的PS，含有二软脂酰磷酰胆碱（DPPC）、16烷醇和四丁酚醛，前者起表面活性作用，后两者可改善PS在肺泡表面的分布。此外，目前临床应用的PS还有从猪肺提取的Curosurf、来自牛肺的Infasurf以及人造肺扩张剂（artificial lung

expanding compound, ALEC）等。

（3）使用方法：一旦确诊，国争生后24小时内经气管插管注入肺内。根据所用PS的不同，其剂量及重复给药的间隔时间（6小时或12小时）亦不相同。视病情轻重，可给予2～4次。

（4）注意事项：①因表面活性物质的粘滞可发生气道阻塞，故在PS从呼吸道扩散到肺泡内之前，应适当增加机械通气的压力；②应用PS后，当潮气量迅速增加时，应及时下调PIP，以免发生肺气漏；③预防性应用PS时，应尽管避免因气管插管时间过长而发生低氧血症，甚至导致早产儿脑损伤。

4．关闭动脉导管　如出现动脉导管开放表现，应采取以下措施。

（1）限制入液量，并给予利尿剂：尽可能减少液体的摄入，减少血液从降主动脉分流到肺动脉，以减少肺内液体的积聚。此外，利尿剂尚有利于减轻心脏的前负荷。

（2）吲哚美辛：为前列腺素合成酶抑制剂。前列腺素E是胎儿及生后初期维持动脉导管开放的重要物质，而前列腺素合成酶抑制剂（吲哚美辛）可减少前列腺素E的合成，故有助于导管关闭。剂量为每次0.2mg/kg，静脉用药，首次用药后12、36小时可再重复1次，共3次。此外，国外有学者提倡对RDS早产儿生后预防性给予，不但减少RDS恢复期PDA的开放，且其PDA永久性关闭率更高。其缺点是40%患儿可能根本不发生PDA，因而不需要预防性治疗。

（3）布洛芬：为非选择性环氧化酶抑制剂。有研究显示，布洛芬治疗PDA与吲哚美辛同样疗效，且不发生使用吲哚美辛的一些并发症，如减少肠系膜及肾血流，对肾脏的副作用更小。首次剂量10mg/kg口服，24小时和48小时后再重复1次，剂量5mg/kg。但对胎龄＜27周的早产儿用药应慎重。

【预防】

1．预防早产　加强高危妊娠和分娩的监护及治疗；对欲行剖宫产或提前分娩者，应准确测双顶径和羊水中L/S值，以判定胎儿大小和胎肺成熟度。

2．促进胎肺成熟　对孕24～34周需提前分娩或有早产迹象的胎儿，出生48小时前给孕母肌注地塞米松或倍他米松，可明显降低RDS的发病率和病死率，临床上多在分娩前1周应用。也有报道产前应用糖皮质激素可降低IVH、动脉导管开放和坏死性小肠结肠炎发生率。

3．PS　对胎龄<24～34周的早产儿，力争生后30分钟内常规应用，若条件不允许也应争取24小时内应用。

第九节　新生儿黄疸

新生儿黄疸（neonatal jaundice）是因胆红素在体内积聚引起的皮肤或其他器官黄染。若新生儿血中胆红素超过5～7mg/dl（成人超过2mg/dl），即可出现肉眼可见的黄疸。部分高未结合胆红素血症患儿可发生胆红素脑病（核黄疸），一般多留有不同程度的神经系统后遗症，重者甚至死亡。

【新生儿胆红素代谢特点】

1．胆红素生成过多　新生儿胆红素是血红素的分解产物，约80％来源于血红蛋白，约20％来源于肝脏和其他组织中的血红素及骨髓中红细胞前体。新生儿每日生成的胆红素明显高于成人（新生儿8.8mg/kg，成人为3.8mg/kg），其主要原因是：①胎儿血氧分压低，其红细胞数量代偿性增加，出生后血氧分压升高，大量红细胞破坏；②新生儿红细胞寿命短（早产儿低于70天，

足月儿约80天，成人为120天），且血红蛋白的分解速度是成人2倍；③肝脏和其他组织中的血红素及骨髓红细胞前体较多。

2．血浆白蛋白联结胆红素的能力差　单核-吞噬细胞系统的胆红素进入血循环，与血浆中白蛋白联结后，运送到肝脏进行代谢。与白蛋白联结的胆红素不能透过细胞膜或血脑屏障，故不引起细胞和脑组织损伤。刚娩出的新生儿常有不同程度的酸中毒，可减少胆红素与白蛋白联结；早产儿胎龄越小，白蛋白含量越低，其联结胆红素的量也越少。

3．肝细胞处理胆红素能力差　未结合胆红素（unconjugated bilirubin）进入肝细胞后，与Y、Z蛋白结合，在滑面内质网，主要通过尿苷二磷酸葡萄糖醛酸基转移酶（UDPGT）的催化，形成水溶性、不能透过半透膜的结合胆红素（conjugated bilirubin），经胆汁排泄至肠道。新生儿出生时肝细胞内Y蛋白含量极微（生后5～10天达正常），UDPGT含量低（生后1周接近正常）且活性不足（仅为正常的0%～30%），故生成结合胆红素的量较少（即未结合胆红素水平高）；出生时肝细胞将结合胆红素排泄到肠道的能力暂时低下，早产儿更为明显，可出现暂时性肝内胆汁淤积（即结合胆红素水平高）。

4．肠肝循环（enterohepatic circulation）增加　在成人，肠道内的结合胆红素被细菌还原成尿胆原及其氧化产物，其中大部分随粪便排除，小部分被结肠吸收后，极少量由肾脏排泄，余下的经门静脉至肝脏重新转变为结合胆红素，再经胆道排泄，即胆红素的“肠肝循环”。出生时肠腔内有β-葡萄糖醛酸苷酶，可将结合胆红素转变成未结合胆红素，加之肠道内缺乏细菌，导致未结合胆红素的产生和吸收增加。此外，胎粪约含胆红素80～180mg，若排泄延迟，可使其重吸收增加。

当患儿饥饿或伴有缺氧、脱水、酸中毒、头颅血肿及颅内出血时，则更易发生黄疸或使原有黄疸加重。

【新生儿黄疸分类】

1．生理性黄疸（physiologicaljaundice）　由于新生儿胆红素代谢特点，约50%～60%的足月儿和80%的早产儿出现生理性黄疸，其特点为：①一般情况良好。②足月儿生后2～3天出现黄疸，4～5天达高峰，5～7天消退，但最迟不超过2周；早产儿黄疸多于生后3～5天出现，5～7天达高峰，7～9天消退，最长可延迟到3～4周。③每日血清胆红素升高<85μmol/L（5mg/dl）。

以往规定足月儿血清胆红素的上限值为205μmol/L（12mg/dl），但国内、外研究资料表明此值偏低，故国外将血清胆红素：足月儿＜221μmol/L（12.9mg/dl）和早产儿＜257μmol/L（15mg/dl）定为生理性黄疸的界限。但有资料表明：亚洲足月儿生理性黄疸的血清胆红素值高于西方足月儿；也有小早产儿血清胆红素＜171μmol/L（10mg/dl）即可发生胆红素脑病（bilirubin encephalopathy）的报道。因此，有关足月儿和早产儿生理性黄疸的上限值，尚需进一步研究。但需注意，生理性黄疸始终是一除外性诊断，必须排除引起病理性黄疸的各种原因后方可确定。

2．病理性黄疸（pathologic jaundice）　①生后24小时内出现黄疸；②血清胆红素足月儿>221μmol/L（12.9mg/dl）、早产儿>257μmol/L（15mg/dl），或每日上升>85μmol/L（5mg/dl）；③黄疸持续时间足月儿>2周，早产儿>4周；④黄疸退而复现；⑤血清结合胆红素>34μmol/L（2mg/dl）。若具备上述任何一项者均可诊断为病理性黄疸。

【病因】

病理性黄疸根据其发病原因分为如下三类。

1．胆红素生成过多　因过多红细胞的破坏及肠肝循环增加，使血清未结合胆红素升高。

（1）红细胞增多症：即静脉血红细胞>6×10^{12}/L，血红蛋白>220g/L，红细胞比容>65%。常见于母-胎或胎-胎间输血、脐带结扎延迟、青紫型先天性心脏病及糖尿病母亲所分娩出的婴

儿等。

（2）血管外溶血：如较大的头颅血肿、皮下血肿、颅内出血、肺出血和其他部位出血。

（3）同族免疫性溶血：见于血型不合如ABO或Rh血型不合等，我国以ABO溶血病较为多见。

（4）感染：细菌、病毒、螺旋体、衣原体、支原体和原虫等引起的重症感染皆可致溶血，以金黄色葡萄球菌及大肠杆菌引起的败血症多见。

（5）肠肝循环增加：先天性肠道闭锁、先天性幽门肥厚、巨结肠、饥饿和喂养延迟等均可使胎粪排泄延迟，使胆红素吸收增加；母乳性黄疸，可能与母乳中的β-葡萄糖醛酸苷酶进入患儿肠内，使肠道内未结合胆红素生成增加有关，见于母乳喂养儿，黄疸于生后3～8天出现，1～3周达高峰，6～12周消退，停喂母乳3～5天，黄疸明显减轻或消退有助于诊断。

（6）红细胞酶缺陷：葡萄糖-6-磷酸脱氢酶（G-6-PD）、丙酮酸激酶和己糖激酶缺陷均可影响红细胞正常代谢，使红细胞膜僵硬，变形能力减弱，滞留和破坏于单核－吞噬细胞系统。

（7）红细胞形态异常：遗传性球形红细胞增多症、遗传性椭圆形红细胞增多症、遗传性口形红细胞增多症、婴儿固缩红细胞增多症等均由于红细胞膜结构异常使红细胞在脾脏破坏增加。

（8）血红蛋白病：α地中海贫血，血红蛋白F-Poole和血红蛋白Hasharon等，由于血红蛋白肽链数量和质量缺陷而引起溶血。

（9）其他：维生素E缺乏和低锌血症等，使红细胞膜结构改变导致溶血。

2．肝脏胆红素代谢障碍　由于肝细胞摄取和结合胆红素的功能低下，使血清未结合胆红素升高。

（1）缺氧和感染：如窒息和心力衰竭等，均可抑制肝脏UDPGT的活性。

（2）Crigler-Najjar综合征：即先天性UDPGT缺乏。Ⅰ型属常染色体隐性遗传，酶完全缺乏，酶诱导剂治疗无效，很难存活；Ⅱ型属常染色体显性遗传，酶活性低下，酶诱导剂治疗有效。

（3）Gilbert综合征：即先天性非溶血性未结合胆红素增高症，属常染色体显性遗传，是由于肝细胞摄取胆红素功能障碍，黄疸较轻。也可同时伴有UDPGT活性降低，此时黄疸较重，酶诱导剂治疗有效。预后良好。

（4）Lucey-Driscoll综合征：即家族性暂时性新生儿黄疸，由于妊娠后期孕妇血清中存在一种孕激素，抑制UDPGT活性所致。本病有家族史，新生儿早期黄疸重，2～3周自然消退。

（5）药物：某些药物如磺胺、水杨酸盐、维生素K_3、吲哚美辛、毛花苷丙等，可与胆红素竞争Y、Z蛋白的结合位点。

（6）其他：先天性甲状腺功能低下、垂体功能低下和21-三体综合征等常伴有血胆红素升高或黄疸消退延迟。

3．胆汁排泄障碍　肝细胞排泄结合胆红素障碍或胆管受阻，可致高结合胆红素血症，但如同时伴有肝细胞功能受损，也可有未结合胆红素增高。

（1）新生儿肝炎：多由病毒引起的宫内感染所致。常见有乙型肝炎病毒、巨细胞病毒、风疹病毒、单纯疱疹病毒、肠道病毒及EB病毒等。

（2）先天性代谢缺陷病：α_1-抗胰蛋白酶缺乏症、半乳糖血症、果糖不耐受症、酪氨酸血症、糖原累积病Ⅳ型及脂质累积病（尼曼匹克病、戈谢病）等可有肝细胞损害。

（3）Dubin-Johnson综合征：即先天性非溶血性结合胆红素增高症，是由肝细胞分泌和排泄结合胆红素障碍所致。

（4）胆管阻塞：先天性胆道闭锁和先天性胆总管囊肿，使肝内或肝外胆管阻塞，结合胆红素排泄障碍，是新生儿期阻塞性黄疸的常见原因；胆汁黏稠综合征是由于胆汁淤积在小胆管中，使结合胆红素排泄障碍，见于严重的新生儿溶血病；肝和胆道的肿瘤也可压迫胆管造成阻塞。

【鉴别黄疸的实验室检查】

由于新生儿黄疸常见、产生原因较多并且发病机制复杂，除要详细询问病史、全面体格检查和必要的组织和影像学检查外，按照一定步骤选择适当的实验室检查对黄疸的诊断和鉴别诊断甚为重要（图7-12）。

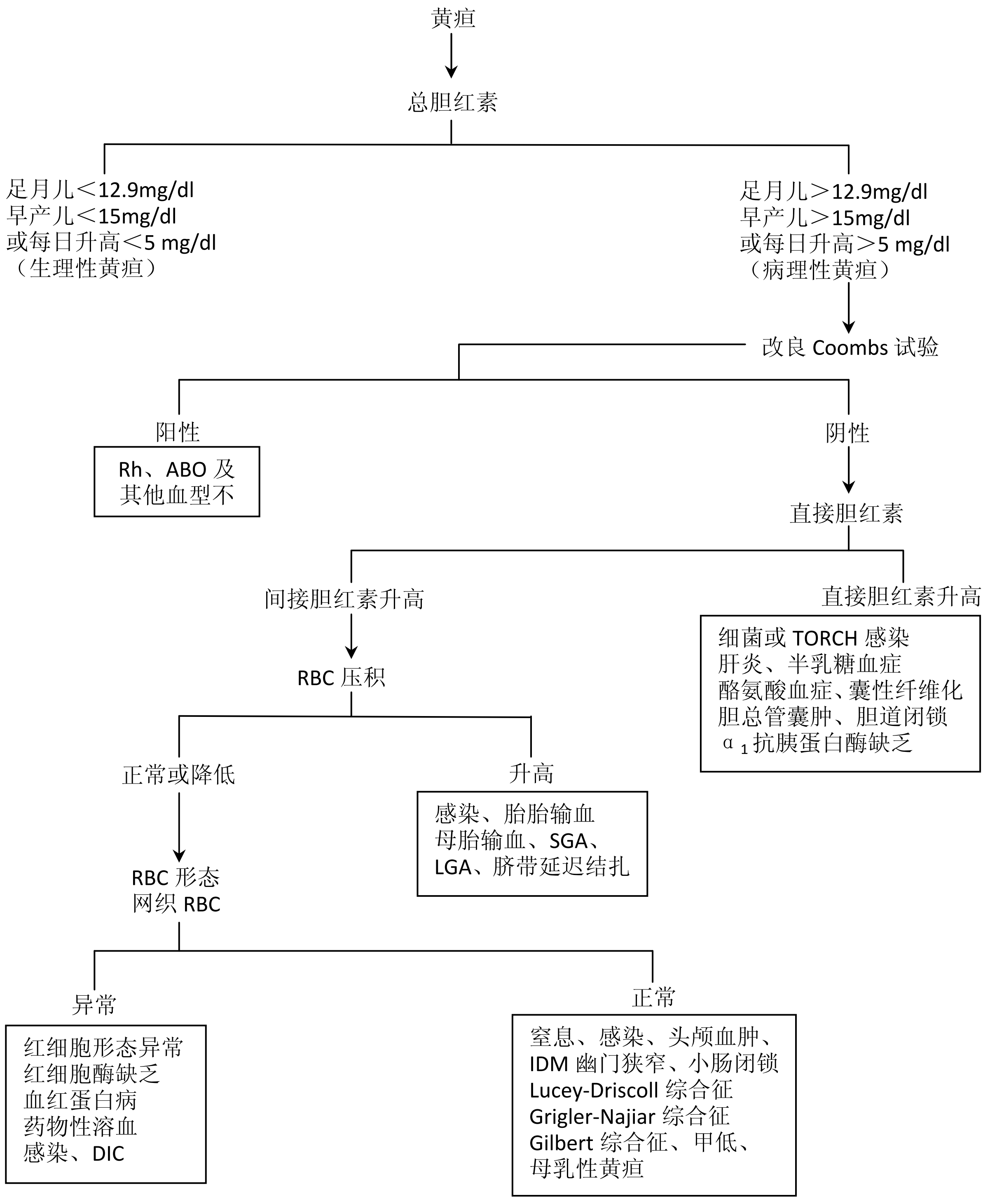

图 7-12　新生儿黄疸的诊断步骤

第十节 新生儿溶血病

新生儿溶血病（hemolytic disease of newborn, HDN）系指母、子血型不合引起的同族免疫性溶血。在已发现的人类26个血型系统中，以ABO血型不合最常见，其次Rh血型不合。有报道新生儿溶血病中，ABO溶血病占85.3％，Rh溶血病占14.6％，MN溶血病仅占0.1％。

【病因和发病机制】

由父亲遗传而母亲所不具有的显性胎儿红细胞血型抗原，通过胎盘进入母体，刺激母体产生相应的血型抗体，当不完全抗体（IgG）进入胎儿血循环后，与红细胞的相应抗原结合（致敏红细胞），在单核-吞噬细胞系统内被破坏，引起溶血。若母婴血型不合的胎儿红细胞在分娩时进入母血，则母亲产生的抗体不使这一胎发病，而可能使下一胎发病（血型与上一胎相同）。

1．ABO溶血　主要发生在母亲O型而胎儿A型或B型，如母亲AB型或婴儿“O”型，则不发生ABO溶血病。

（1）40%～50%的ABO溶血病发生在第一胎。其原因是：O型血的母亲在首次妊娠前，已受到自然界A或B血型物质（某些植物、寄生虫、伤寒疫苗、破伤风及白喉类毒素等）的刺激，产生了抗A或抗B抗体（IgG）。

（2）在母子ABO血型不合中，仅1/5新生儿发生ABO溶血病。其原因为：①胎儿红细胞抗原性的强弱不同，导致抗体产生量的多少各异；②血浆及组织中存在的A和B血型物质，可与来自母体的抗体结合，使血中抗体减少。

2．Rh溶血　Rh血型系统有6种抗原，即D、E、C、c、d、e（d抗原未测出只是推测），其抗原性强弱依次为D>E>C>c>e，故Rh溶血病中以RhD溶血病最常见，其次为RhE，由于e抗原性最弱，故Rhe溶血病罕见。传统上红细胞缺乏D抗原称为Rh阴性，而具有D抗原称为Rh阳性，中国人绝大多数为Rh阳性。但由于母亲Rh阳性（有D抗原），也可缺乏Rh系统其他抗原如E，若胎儿有该抗原时，也可发生Rh溶血病，故本节将缺少Rh血型系统中任一抗原者均称之为Rh阴性，反之称之为Rh阳性。

（1）Rh溶血病一般不发生在第一胎，是因为自然界无Rh血型物质，Rh抗体只能由人类红细胞Rh抗原刺激产生。Rh阴性母亲首次妊娠，于妊娠末期或胎盘剥离（包括流产及刮宫）时，Rh阳性的胎儿血（>0.5～lml）进入母血中，约经过8～9周产生IgM抗体（初发免疫反应），此抗体不能通过胎盘，以后虽可产生少量IgG抗体，但胎儿已经娩出。如母亲再次妊娠（与第一胎Rh血型相同），怀孕期可有少量（0.05～0.1ml）胎儿血进入母体循环，于几天内便可产生大量IgG抗体（次发免疫反应），该抗体通过胎盘引起胎儿溶血。

（2）既往输过Rh阳性血的Rh阴性母亲，其第一胎可发病。极少数Rh阴性母亲虽未接触过Rh阳性血，但其第一胎也发生Rh溶血病，这可能是由于Rh阴性孕妇的母亲为Rh阳性，其母怀孕时已使孕妇致敏，故其第一胎发病（外祖母学说）。

（3）抗原性最强的RhD血型不合者，也仅有1/20发病，主要由于母亲对胎儿红细胞Rh抗原的敏感性不同。另外，母亲为RhD阴性，如父亲的RhD血型基因为杂合子，则胎儿为RhD阳性的可能性为50%，如为纯合子则为100%，其他Rh血型也一样。

【病理生理】

ABO溶血除引起黄疸外，其他变化不明显。Rh溶血可引起胎儿重度贫血，甚至心力衰竭。由于重度贫血、低蛋白血症和心力衰竭可导致全身水肿（胎儿水肿）。贫血时，髓外造血增强，可出现肝、脾肿大。胎儿血中的胆红素经胎盘入母亲肝脏进行代谢，故娩出时黄疸往往不明显。

出生后，由于新生儿胆红素的能力较差，因而出现黄疸。若血清未结合胆红素过高，则可透过血-脑屏障，使基底核等处的神经细胞黄染、坏死，发生胆红素脑病（bilirubin encephalopathy）。

【临床表现】

症状轻重与溶血程度基本一致。多数ABO溶血病患儿除黄疸外，无其他明显异常。Rh溶血病症状较重，严重者甚至死胎。

1．黄疸　大多数Rh溶血病患儿生后24小时内出现黄疸并迅速加重，而多数ABO溶血病在生后第2～3天出现。血清胆红素以未结合型为主，但如溶血严重，造成胆汁淤积，结合胆红素也可升高。

2．贫血　程度不一。重症Rh溶血，生后即可有严重贫血、胎儿水肿或伴有心力衰竭。部分患儿因其抗体持续存在，也可于生后3～6周发生晚期贫血。

3．肝脾大　Rh溶血病患儿多有不同程度的肝脾增大，ABO溶血病患儿则不明显。

【并发症】

胆红素脑病为新生儿溶血病的最严重并发症，早产儿更易发生。多于生后4～7天出现症状，临床将其分为4期。

1．警告期　表现为嗜睡、反应低下、吮吸无力、拥抱反射减弱、肌张力减低等，偶有尖叫和呕吐。持续约12～24小时。

2．痉挛期　出现抽搐、角弓反张和发热（多与抽搐同时发生）。轻者仅有双眼凝视，重者出现肌张力增高、呼吸暂停、双手紧握、双臂伸直内旋，甚至角弓反张。此期约持续12～48小时。

3．恢复期　吃奶及反应好转，抽搐次数减少，角弓反张逐渐消失，肌张力逐渐恢复，此期约持续2周。

4．后遗症期　胆红素脑病四联症：①手足徐动：经常出现不自主、无目的和不协调的动作；②眼球运动障碍：眼球向上转动障碍，形成落日眼。③听觉障碍：耳聋，对高频音失听。④牙釉质发育不良：牙呈绿色或深褐色。此外，也可留有脑瘫、智能落后、抽搐、抬头无力和流涎等后遗症。

典型病例依据病史及临床表现不难诊断，但患儿的头部MRI检查则更有助于该病的诊断（图7-13、图7-14）。

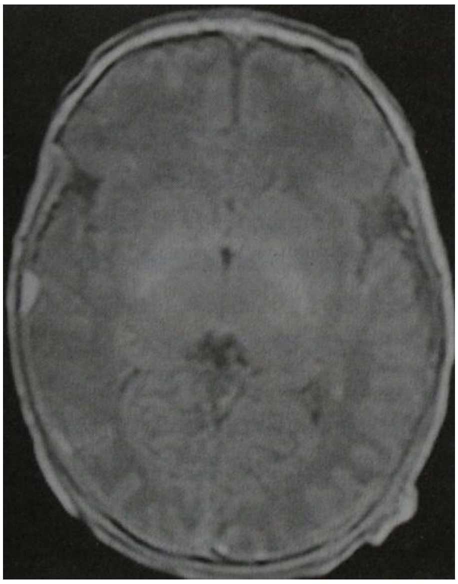

图 7-13　胆红素脑病患儿头 MRI（T1WI）
双侧苍白球可见对称性短 T1 信号（高信号）

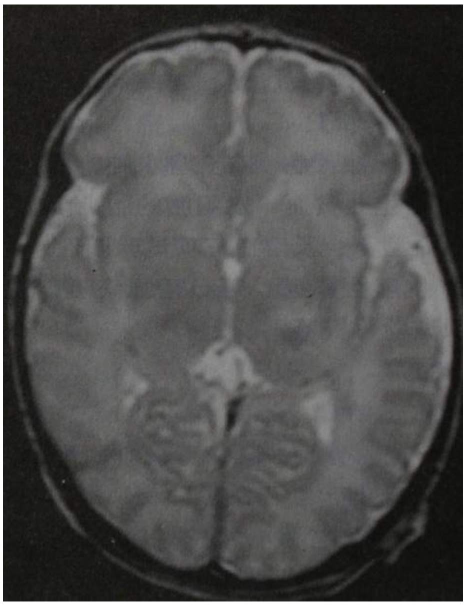

图 7-14　胆红素脑病患儿头 MRI（T2WI）
双侧苍白球未见明显信号异常

【辅助检查】

1．检查母子血型　检查母子ABO和Rh血型，证实有血型不合存在。

2．确定有无溶血　溶血时红细胞和血红蛋白减少，早期新生儿血红蛋白<145g/L可诊断为贫血；网织红细胞增高（>6%）；血涂片有核红细胞增多（>10/100个白细胞）；血清总胆红素和未结合胆红素明显增加。

3．致敏红细胞和血型抗体测定

（1）改良直接抗人球蛋白试验：即改良Coombs试验（既往常用的Coombs试验已淘汰），是用“最适稀释度”的抗人球蛋白血清与充分洗涤后的受检红细胞盐水悬液混合，如有红细胞凝聚为阳性，表明红细胞已致敏。Rh溶血病其阳性率高而ABO溶血病阳性率低。该项为该新生儿溶血病的确诊试验。

（2）抗体释放试验（antibody release test）：通过加热使患儿血中致敏红细胞的血型抗体释放于释放液中，将与患儿相同血型的成人红细胞（ABO系统）或O型标准红细胞（Rh系统）致敏，再加入抗人球蛋白血清，如有红细胞凝聚为阳性。Rh和ABO溶血病一般均为阳性。该项是检测致敏红细胞的敏感试验，故也为新生儿溶血病的确诊实验。

（3）游离抗体试验（free antibody test）：在患儿血清中加入与其相同血型的成人红细胞（ABO系统）或O型标准红细胞（Rh系统）致敏，再加入抗人球蛋白血清，如有红细胞凝聚为阳性。表明血清中存在游离的ABO或Rh血型抗体，并可能与红细胞结合引起溶血。该项实验有助于估计是否继续溶血或换血后的效果评价，但不是新生儿溶血病的确诊试验。

【诊断】

1．产前诊断　凡既往有不明原因的死胎、流产、新生儿重度黄疸史的孕妇及其丈夫均应进行ABO、Rh血型检查，不合者进行孕妇血清中抗体检测。孕妇血清中IgG抗A或抗B＞1：64，提示有可能发生ABO溶血病。Rh阴性孕妇在妊娠16周时应检测血中Rh血型抗体作为基础值，以后每2～4周检测一次，当抗体效价上升，则提示可能发生Rh溶血病。

2．生后诊断　新生儿娩出后黄疸出现早、且进行性加重，有母子血型不合，改良Coombs或抗体释放试验中有一项阳性者即可确诊。

【鉴别诊断】

1．先天性肾病　有全身水肿、低蛋白血症和蛋白尿，但无病理性黄疸和肝脾大。

2．新生儿贫血　双胞胎的胎-胎间输血，或胎-母间输血可引起新生儿贫血，但无重度黄疸、血型不合及溶血三项试验阳性。

3．生理性黄疸　ABO溶血病可仅表现为黄疸，易与生理性黄疸混淆，血型不合及溶血三项试验可资鉴别。

【治疗】

1．产前治疗

（1）提前分娩：既往有输血、死胎、流产和分娩史的Rh阴性孕妇，本次妊娠Rh抗体效价逐渐升至1：32或1：64以上，用分光光度计测定羊水胆红素增高，且羊水L/S＞2者，提示胎肺已发育成熟，应考虑提前分娩。

（2）血浆置换：对血Rh抗体效价明显增高，但又不宜提前分娩的孕妇，进行血浆置换，以换出抗体，减少胎儿溶血。

（3）宫内输血：对胎儿水肿或胎儿Hb<80g/L，而肺尚未成熟者，可直接将与孕妇血清不凝集的浓缩红细胞在B超下注入脐血管或胎儿腹腔内，以纠正贫血。

（4）苯巴比妥：孕妇于预产期前1～2周口服苯巴比妥，可诱导胎儿UDPGT产生增加，以减轻新生儿黄疸。

2．新生儿治疗

（1）光照疗法（phototherapy）：简称光疗，是降低血清未结合胆红素简单而有效的方法。

1）原理：未结合胆红素在光的作用下，转变成水溶性异构体，经胆汁和尿液排出。波长425～475nm的蓝光和波长510～530nm的绿光效果较好，日光灯或太阳光也有一定疗效。光疗主要作用于皮肤浅层组织，因此皮肤黄疸消退并不一定表明血清未结合胆红素已降至正常。

2）设备：主要有光疗箱、光疗灯和光疗毯等。光疗箱以单面光160W、双面光320W为宜，双面光优于单面光；上、下灯管距床面距离分别为40cm和20cm；蓝光灯管使用300小时其能量减少20％，900小时减少35％，2000小时减少45％。光照时，婴儿双眼用黑色眼罩保护，以免损伤视网膜，除会阴、肛门部用尿布遮盖外，其余均裸露。照射时间以不超过4天为宜。

3）副作用：可出现发热、腹泻和皮疹，但多不严重，可继续光疗；蓝光可分解体内核黄素，光疗超过24小时可引起核黄素减少，并进而降低红细胞谷胱甘肽还原酶活性而加重溶血，故光疗时应补充核黄素（光疗时每日3次，5mg/次；光疗后每日1次，连服3日）。当血清结合胆红素>68μmol/L（4mg/dl），并且血清谷丙转氨酶和碱性磷酸酶增高时，光疗可使皮肤呈青铜色即青铜症，此时应停止光疗，青铜症可自行消退。此外，光疗时应适当补充水分及钙剂。

4）指征：①血清总胆红素>205μmol/L（12mg/dl）；②已诊断新生儿溶血病，若生后血清胆红素>85μmol/L（5mg/dl）便可光疗；③超低出生体重儿（ELBW）的血清胆红素>85μmol/L（5mg/dl），极低出生体重儿（VLBW）的血清胆红素>103μmol/L（6mg/dl）（因小早产儿易发生胆红素脑病）。此外，也有学者主张，对所有高危儿进行预防性光疗。

（2）药物治疗：①供给白蛋白：输血浆每次10～20ml/kg或白蛋白1g/kg，以增加其与未结合胆红素的联结，减少胆红素脑病的发生。②纠正代谢性酸中毒：应用5％碳酸氢钠提高血pH值，以利于未结合胆红素与白蛋白联结。③肝酶诱导剂：能增加UDPGT的生成和肝脏摄取未结合胆红素能力。常用苯巴比妥每日5mg/kg，分2～3次口服，共4～5日；也可加用尼可刹米每日100mg/kg，分2～3次口服，共4～5日。④静脉用免疫球蛋白：可阻断单核-吞噬细胞系统Fc受体，抑制吞噬细胞破坏致敏红细胞，用法为lg/kg，于6～8小时内静脉滴入，早期应用临床效果较好。

（3）换血疗法（exchange transfusion）

1）作用：①换出部分血中游离抗体和致敏红细胞，减轻溶血；②换出血中大量胆红素，防止发生胆红素脑病；③纠正贫血，改善携氧，防止心力衰竭。

2）指征：大部分Rh溶血病和个别严重ABO溶血病。符合下列条件之一者即应换血：①产前已明确诊断，出生时脐血总胆红素>68μmol/L（4mg/dl），血红蛋白低于120g/L，伴水肿、肝脾大和心力衰竭者；②生后12小时内胆红素每小时上升>12μmol/L（0.7mg/dl）者；③总胆红素已达到342μmol/L（20mg/dl）者；④不论血清胆红素水平高低，已有胆红素脑病的早期表现者；⑤小早产儿、合并缺氧、酸中毒者或上一胎溶血严重者，应适当放宽指征。

3）方法：①血源：Rh溶血病应选用Rh系统与母亲同型，ABO系统与患儿同型的血液，紧急或找不到血源时也可选用O型血；母O型、子A或B型的ABO溶血病，最好用AB型血浆和O型红细胞的混合血，也可用抗A或抗B效价不高的O型血或患儿同型血；有明显贫血和心力衰竭者，可用血浆减半的浓缩血。②换血量：一般为患儿血量的2倍（约150～180ml/kg），大约可换出85％

的致敏红细胞和60%的胆红素及抗体。也有人主张用3倍血，以换出更多致敏红细胞、胆红素及抗体，但所需时间较长并对患儿循环影响较大。③途径：一般选用脐静脉或其他较大静脉进行换血，也可选用动、静脉或外周动、静脉进行同步换血。

（4）其他治疗：防止低血糖、低体温，纠正缺氧、贫血、水肿和心力衰竭等。

【预防】

Rh阴性妇女在流产或分娩Rh阳性胎儿后，应尽早注射相应的抗Rh免疫球蛋白，以中和进入母血的Rh抗原。目前临床常用的预防方法，是对RhD阴性妇女在流产或分娩RhD阳性胎儿后，72小时内肌注抗D球蛋白300μg，并起到了较满意的预防效果。

（薛辛东）

第十一节 新生儿感染性疾病

近年来感染性疾病已有逐年减少的趋势，但目前我国该病的发病率和病死率仍占新生儿疾病首位。细菌和病毒是最常见的病原体，其次为霉菌、原虫、螺旋体等。TORCH是弓形虫（toxoplasma）、其他（other）、风疹病毒（rubella virus，RV）、巨细胞病毒（cytomegalovirus，CMV）和单纯疱疹病毒（herpes simplex virus，HSV）英文字头的简称，是引起宫内感染的常见病原体。近年来，梅毒螺旋体、乙型肝炎病毒、细小病毒B_{19}（parovirus B_{19}）、解脲脲支原体（ureaplasma urealyticum）、人类免疫缺陷病毒等感染逐渐增多，也成为宫内感染的常见病原体，应做好母亲的产前检查。

新生儿感染可发生在出生前、出生时或出生后。①出生前感染：病原体经母亲血液透过胎盘感染胎儿是最常见的途径，又称宫内感染。TORCH是宫内感染的常见病原体，可导致流产、死胎、死产、胎儿宫内发育迟缓、先天性畸形及婴儿出生后肝、脾肿大、黄疸、贫血、血小板减少以及神经系统受损等多器官损害，即“宫内感染综合征”。此外，母亲生殖道病原体上行性感染羊膜囊，胎儿吸入污染的羊水，或取绒毛标本、羊膜囊穿刺、脐带取血等有创性操作而又消毒不严时也可导致胎儿感染。②出生时感染：胎儿吸入产道中污染的分泌物或血液中的病原体；胎膜早破、产程延长、分娩时消毒不严或经阴道采胎儿头皮血、产钳助产损伤等均可使胎儿感染。③出生后感染：较上述两种感染更常见，病原体可通过皮肤黏膜创面、脐残端创面、呼吸道、消化道及带菌的家庭成员或医护人员接触传播。其中，与携带病毒的母亲密切接触是新生儿生后病毒感染最重要的途径（表7-6）。另外，消毒不严的各种导管和仪器也可造成医源性感染。

表7-6 新生儿主要感染病原体、传播时间及途径

感染途径		病毒	细菌	其他
妊娠期间子宫内感染	经胎盘	水痘-带状疱疹病毒、柯萨奇病毒、微小病毒、巨细胞病毒、人类免疫缺陷病毒、风疹病毒	李斯特菌	弓形虫、疟原虫、梅毒螺旋体
	上行性	单纯疱疹病毒	B族链球菌	沙眼衣原体
分娩时感染	经产道	乙型肝炎病毒、人类免疫缺陷病毒、单纯疱疹病毒	结核杆菌、B族链球菌、淋球菌	

一、新生儿败血症

新生儿败血症（neonatal septicemia）是指病原体侵入新生儿血液循环，并在其中生长、繁殖、产生毒素并发生全身炎症性反应综合征。常见的病原体为细菌，也可为真菌、病毒或原虫等。本节按阐述细菌性败血症（bacterial sepsis），其发生率占活产儿的1‰～10‰，病死率为13%～50%。

【病因和发病机制】

1．病原菌　因不同地区和年代而异，我国多年来一直以葡萄球菌最多见，其次为大肠杆菌等G^{-}杆菌。近年来随着NICU的发展，静脉留置针、气管插管和广谱抗生素的广泛应用以及极低出生体重儿存活率明显提高，表皮葡萄球菌、铜绿假单胞菌、克雷伯杆菌、肠杆菌等机会致病菌，产气荚膜梭菌、厌氧菌以及耐药菌株所致的感染有增加趋势。空肠弯曲菌、幽门螺杆菌等已成为新的致病菌。B组溶血性链球菌（group B streptococcus, GBS）和李斯特菌虽然为欧美等发达国家新生儿感染常见的致病菌，但我国及发展中国家少见。

2．非特异性免疫功能　①屏障功能差：皮肤角质层薄、黏膜柔嫩易损伤；脐残端未完全闭合，细菌易进入血液；呼吸道纤毛运动差，胃液酸度低，胆酸少，杀菌力弱，肠黏膜通透性高，同时分泌型IgA缺乏，易发生呼吸道和消化道感染，有利于细菌侵入血循环；血-脑屏障功能不全，易患细菌性脑膜炎。②淋巴结发育不全，缺乏吞噬细菌的过滤作用，不能将感染局限在局部淋巴结。③经典及替代补体途径的部分成分（C3、C5、调理素等）含量低，机体对某些细菌抗原的调理作用差；④中性粒细胞产生及储备均少，趋化性及黏附性低下，备解素、纤维结合蛋白、溶菌酶含量低，吞噬和杀菌能力不足，早产儿尤甚；⑤单核细胞产生粒细胞-集落刺激因子（G-CSF）、白介素8（IL-8）等细胞因子的能力低下。

3．特异性免疫功能　①新生儿体内IgG主要来自母体，且与胎龄相关，胎龄愈小，IgG含量愈低，因此早产儿更易感染；②IgM和IgA分子量较大，不能通过胎盘，新生儿体内含量很低，因此对G^{-}杆菌易感；③由于未曾接触特异性抗原，T细胞处于初始状态，产生细胞因子低下，对外来特异性抗原应答差；④巨噬细胞、自然杀伤细胞活性低。

【临床表现】

1．根据发病时间分早发型和晚发型

（1）早发型：①生后7天内起病；②感染发生在出生前或出生时，与围生因素有关，常由母亲垂直传播引起，病原菌以大肠杆菌等G^{-}杆菌为主；③常呈暴发性多器官受累，尤以呼吸系统的症状最明显，病生率高。

（2）晚发型：①出生7天后起病；②感染发生在出生时或出生后，由水平传播引起，病原菌以葡萄球菌、机会致病菌为主；③常有脐炎、肺炎或脑膜炎等局灶性感染，病死率较早发型低。

2．早期症状、体征常不典型　一般表现为反应差、嗜睡、发热或体温不升、不吃、不哭、体重不增等症状。出现以下表现时应高度怀疑败血症：①黄疸：有时是败血症的唯一表现，表现为黄疸迅速加重、消退延迟或退而复现；②肝脾大：出现较晚，一般为轻至中度大；③出血倾向：皮肤黏膜瘀点、瘀斑、针眼处渗血不止，消化道出血、肺出血等，严重时发生DIC；④休克：面色苍灰，皮肤呈大理石样花纹，血压下降，尿少或无尿，硬肿症出现常提示预后不良；⑤其他：呕吐、腹胀、中毒性肠麻痹、呼吸窘迫或暂停、青紫；⑥可合并肺炎、脑膜炎、坏死性小肠结肠炎、化脓性关节炎和骨髓炎等。

【辅助检查】

1．外周血象　白细胞总数$<5\times10^9$/L或$>20\times10^9$/L、中性粒细胞杆状核细胞所占比例≥0.20、出现中毒颗粒或空泡、血小板计数$<100\times10^9$/L有诊断价值。

2．病原学检查

（1）细菌培养：①血培养：应在使用抗生素之前作，抽血时必须严格消毒；同时作L型细菌和厌氧菌培养可提高阳性率。②脑脊液、尿培养：脑脊液除培养外，还应涂片找细菌；尿培养最好从耻骨上膀胱穿刺取尿液，以免污染，尿培养阳性有助于诊断。③其他：可酌情行胃液、外耳道分泌物、咽拭子、皮肤拭子、脐残端、肺泡灌洗液（气管插管患儿）等细菌培养，阳性仅证实有细菌定植但不能确立败血症的诊断。

（2）病原菌抗原检测：①采用对流免疫电泳（CIE）、酶联免疫吸附试验（ELISA）、乳胶颗粒凝集（LA）等方法用于血、脑脊液和尿中致病菌抗原检测。②基因诊断方法：应用质粒（plasmid）分析、限制性内切酶分析（restriction endonuclease analysis，REA）、核酸杂交（nucleic acid hybridization）、聚合酶链式反应（polymerase chain reaction，PCR）等方法用于鉴别病原菌的生物型和血清型，有利于寻找感染源。

3．急相蛋白　C反应蛋白（C-reactive protein，CRP）、触珠蛋白（Hp）、α_1-酸性糖蛋白（α_1-AG）、α_1-抗胰蛋白酶（α_1-AT）等在急性感染早期即可增加，其中CRP反应最灵敏，在感染6～8小时内即上升，8～60小时达高峰，可超过正常值的数百倍以上，感染控制后可迅速下降。

4．鲎试验　用于检测血和体液中细菌内毒素，阳性提示有革兰阴性细菌感染。

【诊断】

根据病史中有高危因素、临床症状体征、周围血象改变、CRP增高等可考虑本病诊断，确诊有赖于病原菌或病原菌抗原的检出。

【治疗】

1．抗生素治疗　用药原则：①早用药：对于临床上怀疑败血症的新生儿，不必等待血培养结果即应使用抗生素。②静脉、联合给药：病原菌未明确前可结合当地菌种流行病学特点和耐药菌株情况选择两种抗生素联合使用；病原菌明确后可根据药敏试验选择用药（表7-7）；药敏不敏感但临床有效者可暂不换药。③疗程足：血培养阴性，经抗生素治疗后病情好转时应继续治疗5～7天；血培养阳性，疗程至少需10～14天；有并发症者应治疗3周以上。④注意药物毒副作用：1周以内的新生儿，尤其是早产儿肝肾功能不成熟，给药次数宜减少，每12～24小时给药1次，1周后每8～12小时给药1次。氨基糖苷类抗生素因可能产生耳毒性目前已不主张在新生儿期使用。

表7-7　新生儿抗菌药物选择和使用方法

抗菌药物	每次剂量（mg/kg）	每日次数		主要病原
		＜7天	＞7天	
青霉素G	5万～10万U	2	3	肺炎链球菌，链球菌，对青霉素敏感的葡萄球菌，G^-球菌
氨苄西林	50	2	3	嗜血流感杆菌，G^-杆菌，G^+球菌
苯唑西林	25～50	2	3～4	耐青霉素葡萄球菌
羧苄西林	100	2	3～4	铜绿假单胞菌，变形杆菌，多数大肠杆菌，沙门菌
呱拉西林	50～100	2	3	铜绿假单胞菌，变形杆菌，大肠杆菌，肺炎链球菌

抗菌药物	每次剂量（mg/kg）	每日次数		主要病原
		＜7天	＞7天	
头孢呋辛	50	2	3	G^-杆菌，G^+球菌
头孢噻肟	50	2	3	G^-杆菌，G^+球菌，需氧菌，厌氧菌
头孢曲松	50～100	1	1	G^-杆菌，耐青霉素葡萄球菌
头孢他啶	30～50	2	3	铜绿假单胞菌，脑膜炎球菌，G^-杆菌，G^+厌氧球菌
红霉素	10～15	2	3	G^+菌，衣原体，支原体，螺旋体，立克次体
万古霉素（稳可信）	10～15	2	3	金葡菌，链球菌
亚胺培南/西司他丁（泰能）	20～30	2	2	对绝大多数G^-、G^+需氧和厌氧菌有强大杀菌作用
甲硝唑	7.5	2	2	厌氧菌

2．处理严重并发症 ①休克时输新鲜血浆或全血，每次10ml/kg；应用多巴胺或多巴酚丁胺（见本章第五节）；②纠正酸中毒和低氧血症；③减轻脑水肿。

3．清除感染灶。

4．支持疗法　注意保温，供给足够热能和液体，维持血糖和血电解质在正常水平。

5．免疫疗法　①静注免疫球蛋白，每日300～500mg/kg，连用3～5日；②重症患儿可行交换输血，换血量100～150ml/kg。③中性粒细胞明显减少者可输粒细胞1×10^9/kg；④血小板减低者输血小板1～2U/5kg。

二、新生儿感染性肺炎

感染性肺炎（infectious pneumonia）是新生儿常见疾病，也是引起新生儿死亡的重要病因。据统计，围生期感染性肺炎病死率约为5％～20％。可发生在宫内、分娩过程中或生后，由细菌、病毒、衣原体、真菌等不同的病原体引起。

【病因】

1．宫内感染性肺炎（又称先天性肺炎）　主要的病原体为病毒，如风疹病毒、巨细胞病毒、单纯疱疹病毒等，病原体经血行通过胎盘感染胎儿；孕母阴道内的细菌（大肠杆菌、克雷伯杆菌、李斯特菌）、支原体等感染也可经胎盘感染胎儿，但较少见；胎儿吸入污染的羊水可产生肺炎。

2．分娩过程中感染性肺炎　①胎膜早破24小时以上或孕母产道内病原体上行感染羊膜，引起羊膜绒毛膜炎，胎儿吸入污染的羊水，发生感染性肺炎；②胎儿分娩时通过产道吸入污染的羊水或母亲的宫颈分泌物。常见病原体为大肠杆菌、肺炎链球菌、克雷伯菌、李斯特菌和B组溶血性链球菌等，也有病毒、支原体。早产、滞产、产道检查过多更易诱发感染。

3．出生后感染性肺炎　①呼吸道途径：与呼吸道感染患者接触；②血行感染：常为败血症的一部分；③医源性途径：由于医用器械如吸痰器、雾化器、供氧面罩、气管插管等消毒不严，或呼吸机使用时间过长，或通过医务人员手传播等引起感染性肺炎。病原体以金黄色葡萄球菌、大肠杆菌多见。近年来机会致病菌如克雷伯杆菌、假单胞菌、表皮葡萄球菌、枸橼酸杆菌等感染增多。病毒则以呼吸道合胞病毒、腺病毒、巨细胞病毒多见；其他的病原菌如沙眼衣原体、解脲脲支原体等亦应引起重视。广谱抗生素使用过久易发生念珠菌肺炎。

【临床表现】

1．宫内感染性肺炎　临床表现差异很大。多在生后24小时内发病，出生时常有窒息史，复苏后可有气促、呻吟、呼吸困难，体温不稳定，反应差。肺部听诊呼吸音可为粗糙、减低或闻及湿啰音。严重者可出现呼吸衰竭、心力衰竭、DIC、休克或持续肺动脉高压。血行感染者常缺乏肺部体征，而表现为黄疸、肝脾大和脑膜炎等多系统受累。也有生后数月进展为慢性肺炎。周围血象白细胞大多正常，也可减少或增加。脐血IgM>200mg/L或特异性IgM增高者对产前感染有诊断意义。X线胸片常显示为间质性肺炎改变，细菌性肺炎则为支气管肺炎表现。

2．分娩过程中感染性肺炎　发病时间因不同病原体而异，一般在出生数日至数周后发病，细菌性感染在生后3～5天发病，Ⅱ型疱疹病毒感染多在生后5～10天发病，而衣原体感染潜伏期则长达3～12周。生后立即进行胃液涂片找白细胞和病原体，或取血标本、气管分泌物等进行涂片、培养和对流免疫电泳等检测有助于病原学诊断。

3．产后感染性肺炎　表现为发热或体温不升、气促、鼻翼扇动、发绀、吐沫、三凹征等。肺部体征早期常不明显，病程中可出现双肺细湿啰音。呼吸道合胞病毒肺炎可表现为喘息，肺部听诊可闻哮鸣音。鼻咽部分泌物细菌培养、病毒分离和荧光抗体，血清特异性抗体检查有助于病原学诊断。金黄色葡萄球菌肺炎易合并脓气胸，X线检查可见肺大泡。

【治疗】

1．呼吸道管理　雾化吸入，体位引流，定期翻身、拍背，及时吸净口鼻分泌物，保持呼吸道通畅。

2．供氧　有低氧血症时可用鼻导管、面罩、头罩或鼻塞CPAP给氧，呼吸衰竭时可行机械通气，使动脉血PaO_2维持在6.65～10.7kPa（50～80mmHg）。

3．抗病原体治疗　细菌性肺炎者可参照败血症选用抗生素。李斯特菌肺炎可用氨苄西林；衣原体肺炎首选红霉素；单纯疱疹病毒性肺炎可用阿昔洛韦；巨细胞病毒肺炎可用更昔洛韦。

4．支持疗法　纠正循环障碍和水、电解质及酸碱平衡紊乱，每日输液总量60～100ml/kg，输液速率应慢，以免发生心力衰竭及肺水肿；保证充足的能量和营养供给，酌情静脉输注血浆、白蛋白和免疫球蛋白，以提高机体免疫功能。

三、新生儿破伤风

新生儿破伤风（neonatal tetanus）是指破伤风梭状杆菌侵入脐部、并产生痉挛毒素而引起以牙关紧闭和全身肌肉强直性痉挛为特征的急性感染性疾病。随着我国城乡新法接生技术的应用和推广，本病发病率已明显降低。

【病因和发病机制】

破伤风杆菌为革兰阳性厌氧菌，其芽胞抵抗力强，普通消毒剂无效。破伤风杆菌广泛存在于土壤、尘埃和粪便中，当用该菌污染的器械断脐或包扎时破伤风杆菌即进入脐部，包扎引起的缺氧环境更有利于破伤风杆菌繁殖。其产生的痉挛毒素沿神经干、淋巴液等传至脊髓和脑干运动神经核，与中枢神经组织中神经节苷脂结合，使后者不能释放抑制性神经介质（甘氨酸、氨基丁酸），引起全身肌肉强烈持续收缩。此毒素也可兴奋交感神经，引起心动过速、血压升高、多汗等。

【临床表现】

潜伏期3～14天，常于生后多为4～7天发病，故本病又有“七日风”的俗称。潜伏期愈短、病情愈重、病死率也愈高。早期症状为哭闹、口张不大、吃奶困难，如用压舌板压舌时，用力愈

大、张口愈困难，有助于早期诊断。随后发展为牙关紧闭、面肌紧张、口角上牵、呈“苦笑”面容，伴有阵发性双拳紧握，上肢过度屈曲，下肢伸直，呈角弓反张状。呼吸肌和喉肌痉挛可引起青紫、窒息。痉挛发作时患儿神志清楚为本病的特点，任何轻微刺激即可诱发痉挛发作。经合理治疗1～4周后痉挛逐渐减轻，发作间隔时间延长，能吮乳，完全恢复约需2～3个月。病程中常并发肺炎和败血症。

【治疗】

1．护理　将患儿置于安静、避光的环境，尽量减少刺激以减少痉挛发作。痉挛期应暂禁食，禁食期间可通过静脉供给营养，症状减轻后试用胃管喂养。脐部用3％过氧化氢清洗，涂抹碘酒、酒精。

2．抗毒素　只能中和游离破伤风毒素，对已与神经节苷脂结合的毒素无效，因此愈早用愈好。破伤风抗毒素（TAT）1万～2万IU肌注或静脉滴注，3000IU脐周注射，用前须做皮肤过敏试验；或破伤风免疫球蛋白（TIG）500IU肌注，TIG血浓度高，半衰期长达30天，且不会发生过敏反应，但价格较昂贵。

3．止痉药　控制痉挛是治疗成功的关键。

（1）地西泮：首选，每次0.3～0.5mg/kg，缓慢静脉注射，5分钟内即可达有效浓度，但半衰期短，不适合作维持治疗，4～8小时1次。

（2）苯巴比妥钠：首次负荷量为15～20mg/kg，缓慢静注；维持量为每日5mg/kg，分4～8小时1次，静注。可与地西泮交替使用。

（3）10％水合氯醛：剂量每次0.5ml/kg，胃管注入或灌肠，常作为发作时临时用药。

4．抗生素　青霉素每日20万U/kg，或头孢菌素、甲硝唑，静脉滴注，7～10天，可杀灭破伤风杆菌。

【预防】

严格执行新法接生完全可预防本病。一旦接生时未严格消毒，须在24小时内将患儿脐带远端剪去一段，并重新结扎、消毒脐带处，同时肌注TAT l500～3000IU，或注射TIG 75~250IU。

四、新生儿巨细胞病毒感染

巨细胞病毒感染（cytomegalovirus infection）是由人类巨细胞病毒（human cytomegalovirus，HCMV）引起。巨细胞病毒属于疱疹病毒，普遍存在于自然界，感染的发生与地区、环境、居住条件、经济状况、性别、年龄等有关。我国是CMV感染的高发地区，孕妇抗体阳性率高达95％左右。母孕期初次感染（原发感染）或再发感染时病毒通过胎盘感染胎儿称先天性感染，再发感染包括母孕期潜伏感染重新激活（复燃）和不同抗原的CMV再感染。新生儿出生时经产道吸入含CMV的分泌物或出生后不久接触母亲含有CMV的唾液、尿液、摄入带病毒的母乳、输血引起的感染称围生期感染。由于母乳中CMV排毒率约20％～70％，因此，摄入带病毒的母乳是生后感染的重要途径。

【临床表现】

1．先天性感染（宫内感染）　①新生儿出生2周内有病毒排出。②母为原发感染时，30％～40％胎儿被感染，但仅5％～10％新生儿出生时出现多器官、多系统受损症状，其中20％～30％于新生儿期死亡，主要死于DIC、肝功能衰竭或继发严重细菌感染；其余大部分有后遗症。90％出生时无症状者中，10％～15％以后将出现后遗症。③母为再发感染时，仅1％的胎儿被感染，且新生儿出生时无症状。④常见的临床症状有早产、宫内发育迟缓、黄疸、肝脾肿大、肝功能

损害、皮肤瘀斑、血小板减少、贫血、脉络膜视网膜炎、脑钙化、腹股沟疝等，极低出生体重儿CMV肺炎可引起慢性肺部疾病。⑤常见的后遗症有智力低下、运动障碍、癫痫、牙釉质钙化不全，尤为突出的是感觉神经性耳聋，多在1岁左右出现，常为双侧性，并呈进行性加重。

2．围生期感染　婴儿在生后3～12周排病毒，多数无症状，新生儿期主要表现为肝炎和间质性肺炎，足月儿常呈自限性经过，预后一般良好。早产儿还可表现为单核细胞增多症、血液系统损害、心肌炎等，死亡率高达20％。输血传播可引起致命的后果。

【实验室检查】

1．病毒分离　此法最可靠、特异性最强，尿标本中病毒量高，且排病毒持续时间可达数月至数年，但排病毒为间歇性，多次尿培养分离可提高阳性率；此外，脑脊液、唾液等也可行病毒分离。

2．CMV标志物检测　在各种组织或脱落细胞中可检测出典型的包涵体、病毒抗原、或基因等CMV标志物，其中特异性高、敏感的方法是采用DNA杂交试验检测患儿样本中的CMV；或采用PCR技术体外扩增特异性CMV基因片段检出微量病毒。取新鲜晨尿或脑沉渣涂片，在光镜下找典型病变细胞或核内包涵体。此法特异性高，但阳性率低，有时需多次采样才获阳性结果。

3．用ELISA方法检测血清CMV-IgG、IgM抗体　IgM抗体不能通过胎盘，因此，脐血或新生儿生后2周内血清中检出IgM抗体是先天性感染的标志。但其水平低，故阳性率也低。IgG可通过胎盘，从母体获得的IgG在生后逐渐下降，6～8周降至最低点，若血清IgG滴度升高持续6个月以上，提示宫内感染。

4．胎儿超声波图像　可见宫内发育迟缓、脑室扩大、小头畸形、颅内钙化、肝脾大。

【治疗】

更昔洛韦（丙氧鸟苷，ganciclovir）有一定疗效，剂量为每日5～6mg/kg，每12小时1次静脉滴注，疗程6周。副作用主要有白细胞和血小板减少、肝功能损害和脉络膜视网膜炎等；尚可静脉输注丙种球蛋白。对于CMV-IgM阳性的母亲，建议停哺母乳。

五、先天性弓形虫感染

弓形虫病（toxoplasmosis）由刚地弓形虫（toxoplasma gondii）引起，猫科动物是其唯一的终宿主。世界各地感染以欧美国家为主，其中法国人群阳性率高达80％左右，我国在8％以下。成人弓形虫感染大多不发病。母亲对弓形虫有抗体者，胎儿感染罕见。经胎盘传播引起胎儿先天性弓形虫感染者，其孕母几乎均为原发性感染。母亲感染后通过血行传播，引起胎盘感染，从而引起胎儿宫内弓形虫感染。经胎盘传播率约40％，且传播率随胎龄增大而增加，但胎儿感染严重程度随胎龄增大而减轻。据统计，北京地区母亲弓形虫感染、其婴儿感染率为12.6％，是引起小儿中枢神经系统先天畸形及精神发育障碍的重要病因之一。

【临床表现】

中枢神经系统受损和眼症状最突出，脉络膜视网膜炎、脑积水、脑钙化灶是先天性弓形虫病常见的三联症。先天性弓形虫感染中2/3患儿出生时无明显症状，但其中1/3患儿已有亚临床改变。未治疗者于生后数周或数月逐渐出现症状。症状有轻、中、重之分，主要表现为：①全身症状：早产、宫内生长迟缓、黄疸、肝脾大、皮肤紫癜、皮疹、发热或体温不稳、肺炎、心肌炎、肾炎、淋巴结肿大等。②中枢神经系统：可出现脑膜脑炎的症状和体征，如前囟隆起、抽搐、角弓反张、昏迷等。脑脊液常有异常，表现为淋巴细胞增多，蛋白质增高，糖减少，以

及阻塞性脑积水、脑皮层钙化等。脑积水有时是先天性弓形虫感染的唯一表现，可发生在出生时，或出生后逐渐发生；③眼部病变：脉络膜视网膜炎最常见，一侧或双侧眼球受累，还可见小眼球、无眼球等。仅有10％病例出生时症状明显，幸存者大部分遗留中枢神经系统后遗症，如智力发育迟缓、惊厥、脑瘫、视力障碍等。出生时有症状者中30％～70％可发现脑钙化，如不治疗，病灶可增大增多；但若经治疗，其中75％钙化灶可在1岁时减小或消失。

【实验室检查】

1．ELISA检测血清弓形虫IgG、IgM　IgG和IgM双阳性提示急性感染的可能性大。

2．取血或体液直接涂片找病原体。

3．易感动物（鼠、兔）接种或组织细胞培养分离病原体。

4．弓形虫DNA检测　弓形虫特异性DNA探针技术及聚合酶链反应（PCR）技术已用于弓形虫感染的诊断。

妊娠初期感染弓形虫者应终止妊娠，中后期感染者应予治疗。

【治疗】

1．磺胺嘧啶（sulfadiazine）　每日50～l00mg/kg，分4 次口服。

2．乙胺嘧啶（pyrimethamine）　每日lmg/kg，每12小时1次，2～4日后减半。疗程4～6周，用3～4个疗程，每疗程间隔1个月。两药合用是目前治疗此病的最常用方法，但可引起骨髓抑制和叶酸缺乏，用药期间应定期观察血象并服用叶酸5mg，每日3次。

3．螺旋霉素（spiramycin）　在胎盘组织中浓度高，不影响胎儿，适用于弓形虫感染的孕妇及先天性弓形虫病。成人每日2～4g，儿童每日100mg/kg，分2～4次服用，连服3周，间隔1周重复1疗程。

【预后】

轻型或亚临床型预后良好，新生儿期出现症状者约25％死亡。母孕20周前感染者应终止妊娠。

六、新生儿衣原体感染

新生儿衣原体感染（chlamydial infection）是由沙眼衣原体（chlamydia trachomatis，CT）引起。可引起包涵体结膜炎及沙眼衣原体肺炎。在我国发病率颇高。衣原体是必须在活细胞内生长、增殖的一类独立微生物群，包括4个种族，其中与新生儿感染有关的主要是CT。本病主要通过性传播，是西方社会最常见的性传播疾病。新生儿CT感染主要是在分娩时通过产道获得，剖宫产出生的婴儿受染的可能性很小，多由胎膜早破病原体上行而致。

新生儿衣原体感染以结膜炎、肺炎最常见，其他包括中耳炎、鼻咽炎及女婴阴道炎。①衣原体结膜炎：CT是新生儿期结膜炎中的最常见病原菌，暴露于病原体者有1/3发病，潜伏期通常为5～14天，很少超过19天。分泌物初为浆液性，很快变成脓性，眼睑水肿明显，结膜充血、略增厚。由于新生儿缺乏淋巴样组织，故无沙眼典型的滤泡增生，但可有假膜形成。病变以下穹窿和下睑结膜明显。角膜可见微血管翳，但失明罕见。②衣原体肺炎：系结膜炎或定植于鼻咽部CT下行感染所致。多在生后2～4周发病，早期表现为上呼吸道感染症状，不发热或有低热。严重者可见阵发断续性咳嗽、气促或呼吸暂停，肺部可闻及捻发音。如不治闻，病程常迁延数周至数月。胸部X线表现较临床症状为重，主要表现为两肺充气过度、伴双侧广泛间质和肺泡浸润，支气管周围炎，以及散在分布的局灶性肺不张。X线改变一般持续数周至数月消散。白细胞计数一般正常，50％～70％肺炎患儿嗜酸性粒细胞>300×10^{6}/L。

根据典型的结膜炎和肺炎症状，结合胸片，并行下列实验室检测，可明确诊断。①眼下穹隆、下睑结膜刮片行吉姆萨或碘染色找胞浆内包涵体；②从刮片标本接种组织细胞培养中分离CT；取肺炎患儿气管深部分泌物、或鼻咽部抽吸物培养可提高阳性率；③直接荧光抗体（DFA）法、酶免疫测定（EIA）检测CT抗原；④免疫荧光法检测特异性IgM抗体，效价≥1：64有诊断意义；因为CT感染时机体多数不产生IgM，而母亲传给胎儿的特异性IgG抗体可持续数周，故第2次复查抗体滴度升高4倍以上才有诊断价值。

CT结膜炎和肺炎治疗均首选红霉素，每日20～50mg/kg，分3～4次口服，疗程14天；阿奇霉素（azithromycin）具有吸收好，易进入细胞内，不良反应少等优点，剂量为每日10mg/kg，1次服用，连服3日。衣原体结膜炎局部用0.1％利福平眼药水或10％磺胺醋酰钠眼药水滴眼，每日4次，共2周。

七、先天性梅毒

先天性梅毒（congenital syphilis）是指梅毒螺旋体由母体经胎盘进入胎儿血循环所致的感染。近年来，我国先天性梅毒发病率已有明显上升趋势。

在妊娠的任何阶段梅毒螺旋体都可能通过胎盘感染胎儿，多发生在妊娠4个月后。胎儿感染与母亲梅毒的病程及妊娠期是否治疗有关。孕母早期梅毒且未经治疗时，无论是原发或继发感染，其胎儿几乎均会受累，其中50％胎儿发生流产、早产、死胎或在新生儿期死亡。存活者在出生后不同的年龄出现临床症状，其中2岁以内发病者为早期梅毒，主要是感染和炎症的直接结果；2岁后为晚期梅毒，主要为早期感染遗留的畸形或慢性损害。

2/3的新生儿在出生时没有临床感染征象，可由常规产前筛查检出，常于生后2～3周逐渐出现症状。常见的症状有：①肝脾及全身淋巴结肿大：几乎所有患儿均有肝大，其中1/3伴有梅毒性肝炎，出现黄疸、肝功能受损，可持续数月至半年之久；50%患儿有全身淋巴结肿大，无触痛，滑车上淋巴结肿大有诊断价值。②皮肤、黏膜损害：发生率为15％～60％，常见的为梅毒性鼻炎，鼻炎为早期特征，于生后1周出现，可持续3个月之久，表现为鼻塞，分泌物早期清，继之呈脓性、血性，含大量病原体，极具传染性，当鼻黏膜溃疡累及鼻软骨时形成“鞍鼻”，累及喉部引起声嘶。皮疹常于生后2～3周出现，初为粉红、红色多形性斑丘疹，以后变为棕褐色，并有细小脱屑，掌、跖部还可见梅毒性天疱疮。其分布比形态更具特征性，最常见于口周、鼻翼和肛周，皮损数月后呈放射状皲裂。③骨损害：约占80％～90％，但多数无临床体征，少数可因剧痛而致“假瘫”。X线表现为骨、软骨骨膜炎改变，上肢最易受累，且以单侧为主。④血液系统：表现为贫血，白细胞减少或增多，血小板减少，及Coombs试验阴性的溶血性贫血。⑤其他：多为早受累症状，表现为急性化脓性脑膜炎样症状，脑脊液淋巴细胞增高，蛋白增高，糖正常；生后2～3个月时尚可见以肾小球病变为主的肾损伤等。

晚期梅毒表现为性梅毒疹和梅毒瘤、实质性角膜炎、神经性耳聋、楔状齿（Hutchinson齿）。其他有马鞍鼻、马刀胫、惊厥、智能低下等。

诊断主要根据母亲病史、临床表现及实验室检查及X线检查进行综合分析。确诊可根据：①取胎盘、羊水、皮损等易感部位标本，在暗视野显微镜下查找梅毒螺旋体；②性病实验试剂盒（venereal disease research laboratories，VDRL）：简便、快速，敏感性极高，但有假阳性，可作为筛查试验；③荧光螺旋体抗体吸附试验（f1uorescent treponema antibody-absorption，FTA-ABS）：特异性强，常用于确诊。

治疗包括药物治疗及床旁隔离。药物首选青霉素，因梅毒螺旋体对青霉素极度敏感，青霉

素能使梅毒螺旋体自溶酶造成的细胞壁破坏持续进行，直至死亡而不能修复。为避免大剂量青霉素杀死螺旋体而释放出的异性蛋白质所致不良反应，应从小剂量开始，每次5万U/kg，每12小时1次，静脉滴注，共7天，以后改为每8小时1次，共10～14天。或用普鲁卡因青霉素，每日5万U/kg，肌注，共10～14天。青霉素过敏者，可用红霉素每日15mg/kg，连用12～15日，口服或注射。疗程结束后应在2、4、6、9、12个月时追踪监测VDRL试验，直至其滴度持续下降，最终阴性。

及时、正规治疗孕妇梅毒，是减少先天性梅毒发病率的最有效措施。

（姚笠）

第十二节　新生儿寒冷损伤综合征

新生儿寒冷损伤综合征（neonatal cold injury syndrome）简称新生儿冷伤，因多有皮肤硬肿，故又称新生儿硬肿症（sclerema neonatorum）。系由于寒冷或（和）多种疾病所致，以低体温和皮肤硬肿为主要临床表现，重症可并发多器官功能衰竭。

【病因和病理生理】

1．寒冷保温不足　新生儿，尤其是早产儿，易发生低体温和皮肤硬肿的原因较多。①体温调节中枢不成熟。环境温度低时，其增加产热和减少散热的调节功能差，使体温降低。②体表面积相对较大，皮下脂肪少，皮肤薄，血管丰富，易于失热。寒冷时散热增加，导致低体温。③躯体小，总液体含量少，体内储存热量少，对失热的耐受能力差，寒冷时即使有少量热量丢失，体温便可降低。④新生儿由于缺乏寒战反应，寒冷时主要靠棕色脂肪（brown fat）代偿产热，但其代偿能力有限；早产儿由于其储存少（胎龄越小储存越少），代偿产热能力更差；因此，寒冷时易出现低体温。棕色脂肪分布在颈、肩胛间、腋下、中心动脉、肾和肾上腺周围。⑤皮下脂肪（白色脂肪）中，饱和脂肪酸含量高（为成人3倍），由于其熔点高，低体温时易于凝固出现皮肤硬肿。

2．某些疾病　严重感染、缺氧、心力衰竭和休克等使能源物质消耗增加、热卡摄入不足，加之缺氧又使能源物质的氧化产能发生障碍，故产热能力不足，即使在正常散热的条件下，也可出现低体温和皮肤硬肿。严重的颅脑疾病也可抑制尚未成熟的体温调节中枢，其调节功能进一步降低，使散热大于产热，出现低体温，甚至皮肤硬肿。

3．多器官损害　低体温及皮肤硬肿，可使局部血液循环淤滞，引起缺氧和代谢性酸中毒，导致皮肤毛细血管壁通透性增加，出现水肿。如低体温持续存在和（或）硬肿面积扩大，缺氧和代谢性酸中毒进一步加重，可引起多器官功能损害。

【临床表现】

主要发生在寒冷季节或重症感染时。多于生后1周内发病，早产儿多见。低体温和皮肤硬肿是本病的主要特点。

1．一般表现　反应低下，吮乳差或拒乳、哭声低弱或不哭，活动减少，也可出现呼吸暂停等。

2．低体温　新生儿低体温是指体温<35℃。轻症为30～35℃；重度<30℃，可出现四肢甚或全身冰冷。

3．皮肤硬肿　即皮肤紧贴皮下组织，不能移动，按之似橡皮样感，呈暗红色或青紫色，伴水肿者有指压凹陷。硬肿常呈对称性，其发生顺序依次为：下肢→臀部→面颊→上肢→全身。硬肿的面积可按头颈部20％、双上肢18％、前胸及腹部14％、背部及腰骶部14％、臀部8％及

双下肢26％进行计算。严重硬肿可妨碍关节活动，胸部受累可致呼吸困难。

4．多器官功能损害　重症可出现休克、DIC、急性肾衰竭和肺出血等多器官功能衰竭。

【辅助检查】

根据病情需要，检测血常规、动脉血气和血电解质、血糖、尿素氮、肌酐、DIC筛查试验。必要时可做ECG及X光胸片等。

【诊断】

有寒冷季节，不幸温度低和保温不足，或患有可诱发本病的疾病；有体温降低，皮肤硬肿，即可诊断。临床依据体温及皮肤硬肿范围可分为：①轻度：体温≥35℃、皮肤硬肿范围<20％；②中度：体温<35℃，皮肤硬肿范围20%～50％；③重度：体温＜30℃，皮肤硬肿范围＞50%，常伴有器官功能障碍。

【鉴别诊断】

应与新生儿水肿和新生儿皮下坏疽相鉴别。

1．新生儿水肿　①局限性水肿：常发生于女婴会阴部，数日内可自愈；②早产儿水肿：下肢常见凹陷性水肿，有时延及手背、眼睑或头皮，大多数可自行消退；③新生儿Rh溶血病或先天性肾病：水肿较严重，并有其各自的临床特点。

2．新生儿皮下坏疽　常由金黄色葡萄球菌感染所致。多见于寒冷季节。有难产或产钳分娩史。常发生于身体受压部位（枕、背、臀部等）或受损（如产钳）部位。表现为局部皮肤变硬、略肿、发红、边界不清楚并迅速蔓延，病变中央初期较硬以后软化，先呈暗红色逐渐变为黑色，重者可伴有出血和溃疡，亦可融合成大片坏疽。

【治疗】

1．复温（rewarming）　其目的是在体内产热不足的情况下，通过提高环境温度（减少失热或外加热），以恢复和保持正常体温。新生儿由于腋窝部皮下含有较多棕色脂肪，寒冷时氧化产热，使局部温度升高，此时腋温高于或等于肛温（核心温度）。正常状态下，棕色脂肪不产热，T_{A-R}＜0℃；重症新生儿冷伤，因棕色脂肪耗尽，故T_{A-R}也＜0℃；新生儿冷伤初期，棕色脂肪代偿产热增加，则T_{A-R}≥0℃。因此，腋温-肛温差（T_{A-R}）可作为判断棕色脂肪产热状态的指标。

（1）若肛温>30℃，T_{A-R}≥0，提示体温虽低，但棕色脂肪产热较好，此时可通过减少散热，使体温回升。将患儿置于已预热至中性温度的暖箱中，一般在6～12小时内可恢复正常体温。

（2）当肛温<30℃时，多数患儿T_{A-R}<0，提示体温很低，棕色脂肪被耗尽，虽少数患儿T_{A-R}≥0，但体温过低，靠棕色脂肪自身产热难以恢复正常体温，且易造成多器官功能损害，故若肛温<30℃，一般均应将患儿置于箱温比肛温高1～2℃的暖箱中进行外加温。每小时提高箱温0.5～1℃（箱温不超过34℃），在12～24小时内恢复正常体温。然后根据患儿体温调整暖箱温度。在肛温>30℃，T_{A-R}<0时，仍提示棕色脂肪不产热，故此时也应采用外加温使体温回升。

若无上述条件，也可采用温水浴、热水袋、火炕、电热毯或母亲将患儿抱在怀中等加热方法。

2．热量和液体补充　供给充足的热量有助于复温和维持正常体温。热量供给从每日210kJ/kg（50kcal/kg）开始，逐渐增加至每日419～502 kJ/kg（100～120kcal/kg）。喂养困难者可给予部分或完全静脉营养。液体量按0.24ml/kJ（1ml/kcal）计算，有明显心、肾功能损害者，应严格控制输液速度及液体入量。

3．控制感染　根据血培养和药敏结果应用抗生素。

4．纠正器官功能紊乱　对并发心力衰竭、休克、凝血机制障碍、弥散性血管内凝血、肾

衰竭和肺出血等，应给以相应治疗。

【预防】

1．做好围生期保健工作，宣传预防新生儿冷伤的知识。

2．避免早产、产伤和窒息等，及时治疗诱发冷伤的各种疾病。

3．尽早开始喂养，保证充足的热量供应。

4．注意保暖，产房温度不宜低于24℃，生后应立即擦干皮肤，用预热的被毯包裹，有条件者放置暖箱中数小时，待体温稳定后再放入婴儿床中，若室温低于24℃，应增加包被。小早产儿生后应一直在暖箱中保温，箱温设为中性温度，待体重>1800g或室温下体温稳定时，方可放置于婴儿床中。在转院过程中应注意保暖。

第十三节 新生儿坏死性小肠结肠炎

新生儿坏死性小肠结肠炎（neonatal necrotizing enterocolitis，NEC）是以腹胀、呕吐和便血为主要临床表现，以肠壁囊样积气和门静脉充气征为为X线特征的新生儿肠道疾病。90％发生于早产儿，病情严重，其病死率高达50％左右。

【病因和发病机制】

目前有关其确切机制尚不清楚，多认为与下列因素有关。

1．早产儿胃肠道功能不成熟　胃酸分泌少，胃肠动力差，消化酶活力不足，消化道黏膜通透性高，消化吸收能力及局部免疫反应低下。故不适当的喂养、感染及肠壁缺氧缺血等诸因素，均可导致肠道损伤而引发NEC。

2．肠黏膜缺氧缺血　机体缺氧缺血时将重新分配全身血液，以保证心、脑等重要脏器的血液供应，而此时肠系膜血管收缩、肠道血流可减少至正常的35％～50％，若肠黏膜缺血持续存在或缺血后再灌注发生，均可导致肠黏膜损伤而发生NEC。如围生期窒息、严重呼吸暂停、严重心肺疾病、休克、脐动脉插管、低体温、红细胞增多症等。

3．感染　败血症或肠道感染时，细菌及其毒素可直接损伤肠道黏膜，或通过激活免疫细胞产生多种细胞因子，如血小板活化因子、白介素及肿瘤坏死因子等，从而介导肠黏膜的损伤。此外，因肠道内细菌的过度繁殖而造成的肠管胀气也导致肠道黏膜损伤。较常见的细菌有大肠杆菌、梭状芽胞杆菌、铜绿假单胞菌、沙门菌、克雷伯杆菌、产气荚膜杆菌等。病毒和真菌也可引起本病。

4．其他　摄入渗透压过高（＞460mmol/L）的配方乳、渗透压较高的药物如维生素E、茶碱、吲哚美辛等，使大量液体由血管渗入肠腔，减少肠黏膜的血流灌注。此外高渗乳或高渗液也可直接损伤尚未发育成熟的肠黏膜。

【病理】

好发部位为回肠远端及近端升结肠。肠道病变范围轻重悬殊，轻者仅数厘米，重者可累及全胃肠道，但十二指肠较少受累。主要病理变化是肠腔充气，黏膜呈斑片状或大片坏死，肠壁有不同程度的积气、出血及坏死。严重时整个肠壁全层坏死并伴发穿孔。

【临床表现】

本病多见于早产儿。大多在生后2周内（2～12天）发病，极低出生体重儿可延迟至2个月。病初可表现为体温不升、呼吸暂停、心动过缓、拒乳及嗜睡等，同时或继之出现不同程度的胃潴留、腹胀、呕吐、腹泻及血便等。体格检查可见腹壁发红、肠型、腹部压痛，肠鸣音减弱或消失。严重者常并发败血症、肠穿孔和腹膜炎等。最后发展为呼吸衰竭、休克、DIC而死亡。

【辅助检查】

腹部X线摄片对诊断本病有重要意义。主要表现为麻痹性肠梗阻、肠壁间隔增宽、肠壁积气、门静脉充气征，重者肠袢固定（肠坏死）、腹水（腹膜炎）和气腹（肠穿孔）。肠壁积气和门静脉充气征为本病的特征性表现。严重者常伴有外周血中性粒细胞及血小板的减少，代谢性酸中毒和（或）呼吸性酸中毒，休克及DIC等，故血气分析、血常规、C-反应蛋白、血培养及DIC的监测对判定病情尤为重要。此外，大便潜血及培养也不容忽视。

【诊断】

若同时具备以下三项者，即可确诊：①全身感染中毒表现：体温不升、面色苍白、呼吸不规则及心动过缓等；②胃肠道表现：胃潴留、呕吐、肉眼血便、腹胀及肠鸣音消失；③腹部X线摄片表现：肠梗阻和肠壁积气。

【治疗】

1．禁食　疑似患儿禁食3天，确诊病例7～10天，重症14天或更长。待其临床表现好转，腹胀消失，大便潜血转阴后可逐渐恢复进乳。恢复喂养要从水开始，再试喂糖水、稀释奶、以后根据病情逐步增加稀释奶浓度。

2．胃肠减压　禁食期间需进行胃肠减压。

3．抗感染　依据细菌培养及药敏试验结果选择敏感抗生素。若细菌不明时可用氨苄青霉素、哌拉西林钠或第3代头孢菌素，如为厌氧菌首选甲硝唑。疗程7～10天，重症14天或更长。

4．支持疗法和其他治疗　禁食期间应予以静脉营养维持水、电解质平衡及能量需求，液体量120～150ml/kg，热能从209kJ/kg（50kcal/kg）开始，逐渐增加至418～503kJ/kg（100～120kcal/kg）。并注意补充必需氨基酸、必需脂肪酸和维生素。有凝血机制障碍时可输新鲜冰冻血浆或冷沉淀。出现休克时给予抗休克治疗。

5．外科治疗　明显腹膜炎时可考虑手术，肠穿孔时应立即手术。

（薛辛东）

第十四节 新生儿出血症

新生儿出血症（hemorrhagic disease of the newborn，HDN）是由于维生素K缺乏而导致体内某些维生素K依赖凝血因子活性降低的自限性出血性疾病。近年来，由于对初生婴儿出生时常规注射维生素K_1，此病发生率已明显下降。

【病因和发病机理】

Ⅱ、Ⅶ、Ⅸ、Ⅹ等凝血因子主要在肝微粒体内合成，在此过程中须维生素K参与，这些凝血因子前体蛋白的谷氨酸残基才能γ-羧基化，羧基型蛋白具有更多的钙离子结合位点，然后方具凝血的生物活性。当维生素K缺乏时，上述维生素K依赖因子不能羧化，只是无功能的蛋白质，因此不能参与凝血过程而致出血。

本病与下列因素有关：①肝脏储存量低：母体维生素K经胎盘通透性很低，仅1/10的量到达胎儿体内；母亲产前应用抗惊厥药、抗凝药、抗结核药等，干扰维生素K的储存或功能。②合成少：新生儿刚出生时肠道尚无细菌，或使用广谱抗生素抑制肠道正常菌群，均使维生素K合成不足。③摄入少：母乳中维生素K含量（15μg/L）明显低于牛乳（60μg/L），因此纯母乳喂养的婴儿多见；刚出生时摄入少、获得的维生素K量亦少。④吸收少：有先天性肝胆疾病、慢性腹泻等可影响维生素K的吸收。

【临床表现】

根据发病时间分为3型。

1．早发型　生后24小时之内发病，多与母亲产前服用干扰维生素K代谢的药物有关，少数原因不明。轻重程度不一，轻者仅有皮肤少量出血或脐残端渗血；严重者表现为皮肤、消化道、脑等多部位、多器官出血，颅内出血常是致命的。

2．经典型　生后第2～5天发病，早产儿可迟至生后2周发病。表现为皮肤瘀斑、脐残端渗血、胃肠道出血等，而婴儿一般情况好，出血呈自限性。

3．晚发型　生后1～3个月发病，多见于纯母乳喂养、慢性腹泻、营养不良、长期接受全静脉营养者。除其他部位出血外，几乎均有颅内出血，死亡率高，幸存者遗留神经系统后遗症。

【辅助检查】

1．凝血酶原时间和部分凝血活酶时间均延长（为对照的2倍以上意义更大），出血时间、血小板计数正常。

2．活性Ⅱ因子与Ⅱ因子总量比值　两者比值小于1时提示维生素K缺乏。

3．PIVKA Ⅱ法（protein induced in vitamin K absence）　用免疫学方法或电泳法直接直接测定无活性的凝血酶原，阳性提示维生素K缺乏。

4．维生素K测定　高压液相层析法直接测定血中维生素K含量。因需血量大，限制了其在临床上应用。

【诊断与鉴别诊断】

根据有高危病史、发病时间、临床表现、实验室检查及维生素K治疗有效即可诊断，需与以下疾病鉴别。

1．新生儿咽下综合征　婴儿在分娩过程中咽下母血，生后不久即呕血和（或）便血。但本病：①无其他部位出血及贫血；②血红蛋白和凝血机制正常；③经1％碳酸氢钠洗胃1～2次后呕血停止；④Apt试验可鉴别呕吐物中之血是否来自母体：取1份呕吐物加5份水，搅匀，离心（2000r/min）10分钟后取上清液4ml，加入1％氢氧化钠lml，1～2分钟后，如上清液变为棕色提示为母血，不变色（粉红色）为婴儿血。

2．新生儿消化道出血　坏死性小肠结肠炎、应激性溃疡、先天性胃穿孔等可出现呕血或便血。但患儿常有窒息、感染或使用糖皮质激素等原发病史，一般情况较差，腹部体征明显，易与新生儿出血症鉴别。

3．新生儿其他出血性疾病　血小板减少性紫癜有血小板明显降低；DIC常伴有严重原发疾病，纤维蛋白原和血小板减少；血友病患儿以男性多见，且多有家族史，主要表现为外伤后出血不止。

【治疗】

出血者可给予维生素$K_1$1～2mg静脉滴注，出血可迅速停止；通常2小时内凝血因子水平和功能上升，＜24小时完全纠正。严重者可输新鲜冰冻血浆10～20ml/kg，以提高血浆中有活性的凝血因子水平。纠正低血压和贫血。

【预防】

母孕期服用干扰维生素K代谢的药物者，应在妊娠最后3个月期间及分娩前各肌注1次维生素$K_1$10mg。纯母乳喂养者，母亲应口服维生素$K_1$20mg/次，每周2次。所有新生儿出生后应立即给予维生素$K_1$0.5～lmg肌注1次（早产儿连用3天），以预防晚发性维生素K_1缺乏。早产儿、有肝胆疾病、慢性腹泻、长期全静脉营养等高危儿应每周静脉注射1次维生素$K_1$0.5～lmg。

第十五节 新生儿低血糖和高血糖

一、新生儿低血糖

【定义】

新生儿出生后血糖浓度有一自然下降继而上升的过程，并且许多低血糖的新生儿并无任何临床症状和体征，因此，长期以来低血糖的定义一直未完全统一。目前多数学者认为，全血血糖<2.2mmol/L（40mg/dl）应诊断为新生儿低血糖（neonatal hypoglycemia），而不考虑出生体重、胎龄和生后日龄。

【病因和发病机制】

新生儿低血糖有暂时性或持续性之分。

1．暂时性低血糖　指低血糖持续时间较短、不超过新生儿期。

（1）葡萄糖储存不足：主要见于①早产儿：肝糖原储存主要发生在妊娠的最后3个月，因此，胎龄越小，糖原储存越少；②围生期窒息：低氧、酸中毒时儿茶酚胺分泌增多，刺激肝糖原分解增加，加之无氧酵解使葡萄糖利用增多；③小于胎龄儿：除糖原储存少外，糖异生途径中的酶活力也低；④其他：如低体温、败血症、先天性心脏病等，常由于热卡摄入不足，而葡萄糖利用增加所致。

（2）葡萄糖利用增加（即高胰岛素血症）：主要见于①糖尿病母亲娩出的婴儿：由于胎儿在宫内高胰岛素血症，而出生后母亲血糖供给突然中断所致；②Rh溶血病：红细胞破坏致谷胱甘肽释放，刺激胰岛素浓度增加。

2．持续性低血糖　指低血糖持续至婴儿或儿童期。

（1）高胰岛血症：主要见于胰岛细胞增生症、Beckwith综合征、胰岛细胞腺瘤。

（2）内分泌缺陷：如先天性垂体功能不全、皮质醇缺乏、高糖素缺乏、生长激素缺乏等。

（3）遗传代谢性疾病：①糖类疾病：如糖原累积病Ⅰ型、III型；②脂肪酸代谢性疾病：如中链酰基辅酶A 脱氢酶缺乏；③氨基酸代谢缺陷：如支链氨基酸代谢障碍、亮氨酸代谢缺陷等。

【临床表现】

大多数低血糖者无临床症状；少数可出现喂养困难、嗜睡、青紫、哭声异常、颤抖、震颤，甚至惊厥等非特异性症状，经静脉注射葡萄糖后上述症状消失，血糖恢复正常，称“症状性低血糖”。

【辅助检查】

1．血糖测定　高危儿应在生后4小时内反复监测血糖，以后每隔4小时复查，直至血糖浓度稳定。由于纸片法检测简便、快速、无创，可作为高危儿的筛查，但确诊需依据化学法（如葡萄糖氧化酶）测定的血清葡萄糖值（相差10%（1）。须注意：①取标本后应及时测定，因室温下红细胞糖酵解增加，血糖值每小时可下降15～20mg/dl；②由于新生儿红细胞多，且其中还原型谷胱甘肽含量高，红细胞糖酵解增加，故全血糖值较血清糖低10%～15％，当血糖值<30mg/dl时，这种差异更大。

2．持续性低血糖者应酌情选测血胰岛素、高糖素、T_4、TSH、生长激素、皮质醇、血、尿氨基酸及有机酸等。

3．高胰岛素血症时可作胰腺B超或CT检查；疑有糖原累积病时可行肝活体组织检查测定肝糖原和酶活力。

【治疗】

由于并不能确定引起脑损伤的低血糖阈值，因此不管有无症状，低血糖者均应及时治疗。

1．无症状性低血糖并能进食者　可先进食，并密切监测血糖，低血糖不能纠正者可静脉输注葡萄糖，按6～8mg/（kg·min）速率输注，4～6小时后根据血糖测定结果调节输糖速率，稳定24小时后逐渐停用。

2．症状性低血糖：可先给予一次剂量的10％葡萄糖200mg/kg（2ml/kg），按每分钟1.0ml静注；以后改为6～8mg/（kg·min）维持，以防低血糖反跳。每4～6小时监测血糖一次，并根据血糖值调节输糖速率，正常24小时后逐渐减慢输注速率，48～72小时停用。低血糖持续时间较长者可加用氢化可的松5mg/kg，静脉注射，每12小时一次；或泼尼松1～2mg/（kg·d），口服，共3～5天，可诱导糖异生酶活性增高。极低出生体重早产儿对糖耐受性差，输糖速率>6～8mg/（kg·min）易致高血糖症。

3．持续性低血糖　葡萄糖输注速率常需提高至20～30mg/（kg·min）以上才能维持血糖浓度在正常范围。还可①静脉注射胰高血糖素0.02mg/kg，间断给药；或10μg/（kg·h）静脉维持；②高胰岛素血症可用二氮嗪（diazoxide），每日l0mg/kg（最大剂量<25mg/kg），分3次口服。胰岛细胞增生症则须作胰腺次全切除，先天性代谢缺陷患儿应给予特殊饮食疗法。

【预防】

1．避免可预防的高危因素（如寒冷损伤），高危儿定期监测血糖。

2．生后能进食者宜早期喂养。

3．不能经胃肠道喂养者可给10％葡萄糖静脉滴注，足月适于胎龄儿按3～5mg/（kg·min）、早产适于胎龄儿以4～6mg/（kg·min）、小于胎龄儿以6～8mg/（kg·min）速率输注，可达到近似内源性肝糖原产生率。

二、新生儿高血糖

【定义】

新生儿全血血糖>7.0mmol/L（125mg/dl），或血浆葡萄糖水平>8.40mmol/L（150mg/dl）为新生儿高血糖（neonatal hyperglycemia）诊断标准。

【病因和发病机制】

1．应激状态　是新生儿尤其是极低出生体重儿高血糖的最常见原因。当窒息、寒冷损伤、严重感染、外科手术等危重疾病时，血中儿茶酚胺、皮质醇、高血糖素浓度显著升高，提示高血糖与应激有关。同时新生儿本身胰岛β细胞对高血糖的反应迟钝，胰岛素对葡萄糖负荷反应低下；以及存在相对性胰岛素抵抗，引起肝脏产生葡萄糖和胰岛素的浓度及输出之间失衡，是新生儿高血糖的内在因素，尤其是极低出生体重儿。据统计，出生体重1000g以下的早产儿高血糖的危险性是出生体重大于2000g婴儿的18倍。

2．医源性　输注高浓度的葡萄糖或脂肪乳，可引起高血糖。但新生儿对葡萄糖的耐受个体差异很大，极低体重儿即使输糖速率在4～6mg/（kg.min）时亦易发生高血糖。胎龄越小、体重越轻、对糖的耐受越差。

3．药物　氨茶碱能引起cAMP浓度升高，激活肝葡萄糖输出，引起高血糖；其他的药物还有咖啡因、皮质类固醇、苯妥英钠等。

4．新生儿糖尿病　十分罕见，可以是①暂时性（持续3～4周）；②暂时性以后复发；③永久性糖尿病，约1/3患儿有糖尿病家族史，多见于SGA儿。

【临床表现】

轻者可无症状；血糖增高显著者表现为脱水、多尿、体重下降，严重者可因高渗血症致脑室内出血。新生儿糖尿病可出现尿糖阳性、尿酮体阴性或阳性。

【治疗】

极低体重儿用5%的葡萄糖；轻度、短暂（24～48小时）高血糖可通过减慢葡萄糖输注速率纠正；治疗原发病、纠正脱水及电解质紊乱；高血糖不易控制者给胰岛素，开始每小时0.01U/kg，逐渐增至0.05～0.1U/kg输注，但应密切监测血糖，以防低血糖发生，血糖正常后停用。

（常立文）

第十六节 新生儿低钙血症

新生儿低钙血症（neonatalhypocalcemia）是新生儿惊厥的常见原因之一，主要与暂时的生理性甲状旁腺功能低下有关。

【病因和发病机制】

胎盘能主动向胎儿转运钙，故胎儿通常血钙不低。妊娠晚期母血甲状旁腺激素（PTH）水平高，分娩时脐血总钙和游离钙均高于母血水平（早产儿血钙水平低），故使胎儿及新生儿甲状旁腺功能暂时受到抑制（即PTH水平较低）。出生后因源于母亲钙的供应中断，而外源性钙的摄入又不足，加之新生儿PTH水平较低，骨质中钙不能入血，故导致低钙血症。

1．早期低血钙　是指发生于生后72小时内，多见于早产儿、小于胎龄儿、IDM及母亲患妊娠高血压综合征所生婴儿。若有难产、窒息、感染及产伤史者，也易发生低钙血症，其原因可能与细胞大量破坏，导致的的高血磷有关。

2．晚期低血钙　是指发生于生后72小时后，多见于牛乳喂养的足月儿。主要是由于牛乳中磷含量高（900～1000mg/L，人乳150mg/L），钙磷比例不适宜（牛乳1.35：1，人乳2.25：1），故不利于钙的吸收。同时新生儿肾小球滤过率低，而肾小管对磷的重吸收能力较强，导致血磷过高、血钙沉积于骨，发生低钙血症。

3．其他　因碳酸氢钠等碱性药物可使血中游离钙变为结合钙，换血时抗凝剂枸橼酸钠可结合血中游离钙，故补充碱性药物或换血时可使血中游离钙降低。此外，若低血钙持续时间长或反复出现，应注意有无下述疾病。

（1）母甲状旁腺功能亢进：多见于母亲甲状旁腺瘤。由于母血PTH持续在高水平，故孕妇和胎儿高血钙，使胎儿的甲状旁腺功能严重抑制，从而生后出现顽固而持久的低钙血症，并可伴发低镁血症。血磷通常＞2.6mmol/L（8.0mg/dl），应用钙剂可使抽搐缓解，疗程常需持续数周之久。

（2）暂时性先天性特发性甲状旁腺功能不全：属良性自限性疾病，母甲状旁腺功能正常。除应用钙剂外，尚需配伍使用适量的维生素D治疗数月。

（3）先天性永久性甲状旁腺功能不全：系由于新生儿甲状旁腺先天缺如或发育不全所致，为X连锁隐性遗传。具有持久的甲状旁腺功能低下和高磷酸盐血症。如同时合并胸腺缺如、免疫缺陷、小颌畸形和主动脉弓异常者，则应诊断为DiGeorge综合征。

【临床表现】

症状多出现于生后5～10天。主要表现为烦躁不安、肌肉抽动及震颤，可有惊跳及惊厥等，手足搦搐和喉痉挛少见。惊厥发作时常伴有呼吸暂停和发绀；发作间期一般情况良好，但肌张力稍高，腱反射亢进，踝阵挛可呈阳性。早产儿生后3天内易出现血钙降低，其降低程度一般与胎龄成反比，通常无明显体征，可能与其发育不完善、血浆蛋白低和酸中毒时血清游离钙相对较高等有关。

【辅助检查】

血清总钙<1.75mmol/L（7mg/dl），血清游离钙<0.9mmol/L（3.5mg/dl），血清磷>2.6mmol/L（8mg/d1），碱性磷酸酶多正常。必要时还应检测母血钙、磷和PTH水平。心电图QT间期延长（早产儿>0.2秒，足月儿>0.19秒）提示低钙血症。

【治疗】

1．抗惊厥　静脉补充钙剂对低钙惊厥疗效明显。惊厥发作时应立即静脉推注10％葡萄糖酸钙，若抽搐仍不缓解，应加用镇静剂。①使用方法：10％葡萄糖酸钙2ml/（kg·次），以5％葡萄糖液稀释1倍后静脉推注，其速度为lml/min。必要时可间隔6～8小时再给药1次，每日最大剂量为6ml/kg（每日最大元素钙量50～60mg/kg；10％葡萄糖酸钙含元素钙量为9mg/ml）。②注意事项：因血钙浓度升高可抑制窦房结引起心动过缓，甚至心脏停搏，故静脉推注时应保持心率>80次/分。同时应避免药液外溢至血管外，发生组织坏死。③疗程：惊厥停止后可口服葡萄糖酸钙或氯化钙1～2g/d维持治疗，病程长者可口服钙盐2～4周，以维持血钙在2～2.3mmol/L（8.0～9.0mg/dl）为宜。

2．补充镁剂　使用钙剂后，惊厥仍不能控制，应检查血镁。若血镁<1.2mEq/L（1.4mg/dl），可肌内注射25％硫酸镁，按每次0.4ml/（kg．次）。

3．减少肠道内磷的吸收　可服用10％氢氧化铝3～6ml/次，因为氢氧化铝可结合牛乳中的磷，从而减少磷在肠道的吸收。

4．调节饮食　因母乳中钙磷比例适当，利于肠道钙的吸收，故应尽量母乳喂养或应用钙磷比例适当的配方乳。

5．甲状旁腺功能不全者需长期口服钙剂，同时给予维生素$D_2$10000～25000IU/d或二氢速变固醇0.05～0.1mg/d或$1,25(OH)_2D_3$ 0.25～0.5μg/d。治疗过程中应定期监测血钙水平，调整维生素D的剂量。

（薛辛东）

第十七节 新生儿脐部疾病

一、脐 炎

脐炎（omphalitis）是指细菌入侵脐残端，并且在其繁殖所引起的急性炎症。金黄色葡萄球菌是最常见的病原菌，其次为大肠杆菌、铜绿假单胞菌、溶血性链球菌等。轻者脐轮与脐周皮肤轻度红肿，或伴有少量浆液脓性分泌物。重者脐部和脐周明显红肿发硬，分泌物呈脓性且量多，常有臭味。可向周围皮肤或组织扩散，引起腹壁蜂窝织炎、皮下坏疽、腹膜炎、败血症、门静脉炎，甚至以后可发展为门静脉高压症、肝硬化。正常新生儿生后12小时脐部除金黄色葡萄球菌外，还可有表皮葡萄球菌、大肠杆菌、链球菌集落生长，局部分泌物培养阳性并不表示存在感染，必须具有脐部的炎症表现，应予鉴别。轻者局部用2％碘酒及75％酒精清洗，每日2～3次；重者需选用适当的抗生素静脉注射；如有脓肿形成，则需行切开引流。

二、脐 疝

由于脐环关闭不全或薄弱，腹腔脏器由脐环处向外突出到皮下，形成脐疝（umbilical hernia）。疝囊为腹膜及其外层的皮下组织和皮肤，囊内为大网膜和小肠肠曲，与囊壁一般无粘连。疝囊大小不一，直径多为1cm左右，偶有超过3～4cm者。多见于低出生体重儿，体重低于

1500g者75％有脐疝。通常哭闹时脐疝外凸明显，安静时用手指压迫脐囊可回纳，不易发生嵌顿。出生后1年内腹肌逐渐发达，多数疝环逐渐狭窄缩小，自然闭合，预后良好。疝囊较大、4岁以上仍未愈合者可手术修补。

三、脐肉芽肿

脐肉芽肿（umbilical granuloma）是指断脐后脐孔创面受异物刺激（如爽身粉、血痂）或感染，在局部形成小的肉芽组织增生。脐肉芽组织表面湿润，有少许黏液或黏液脓性渗出物，可用酒精一日数次清洁肉芽组织表面，预后良好。顽固肉芽组织增生者，呈灰红色，表面有脓血性分泌物，可用硝酸银烧灼或搔刮局部。

第十八节 新生儿产伤性疾病

新生儿产伤（birth injury）是指分娩过程中因机械因素对胎儿或新生儿造成的损伤。近年来由于加强了产前检查及产科技术提高，产伤发生率已明显下降，但仍是引起新生儿死亡及远期致残的原因之一，尤其是在基层单位。

一、头颅血肿

头颅血肿（cephalhematoma）是由于产伤导致骨膜下血管破裂、血液积留在骨膜下所致。常由胎位不正、头盆不称、胎头吸引术或产钳助产引起。

【临床表现】

血肿部位以头顶部多见，枕、颞、额部少见，常为一侧性，少数为双侧。血肿在生后数小时至数天逐渐增大，因颅缝处骨膜与骨粘连紧密，故血肿不超越骨缝，边界清楚，触之有波动感，其表面皮肤颜色正常。如由产钳牵拉或胎头吸引所致，皮肤常有溃破或呈紫红色。血肿机化后变硬，常需6～8周始吸收。血肿大者常致黄疸加重及贫血，严重者甚至可发生胆红素脑病。应注意与下列疾病鉴别：①先锋头（caput succedaneum)，又称产瘤，是由于分娩时头皮循环受压，血管通透性改变及淋巴回流受阻引起的皮下水肿，多发生在头先露部位，出生时即可发现，肿块边界不清、不受骨缝限制，头皮红肿、柔软、压之凹陷、无波动感，出生2～3天即消失。有时与血肿并存，待头皮水肿消退后才显出血肿。②帽状腱膜下出血（subaponeurotic hemorrhage)：出血发生在头颅帽状腱膜与骨膜之间的疏松组织内，因无骨缝限制，故出血量多，易于扩散。头颅外观呈广泛性肿胀，有波动感，但可超过骨缝。出血量大者，眼睑、耳后和颈部皮下可见紫红色瘀斑，常伴有高胆红素血症、贫血、甚至休克。

【治疗】

血肿小者不需治疗；大血肿伴中度以上高胆红素血症者，应在严格无菌操作下抽吸血肿，并加压包扎2～3天，以避免胆红素脑病发生。同时每日肌注1次维生素K_1lmg，共3次。帽状腱膜下出血伴严重贫血者应给予输血治疗。

二、锁骨骨折

锁骨骨折（fracture of clavicle）是产伤性骨折中最常见的一种，与分娩方式、胎儿娩出方位和出生体重有关。难产、胎儿转位幅度大、巨大儿发生率高。骨折多发生在锁骨中段外1/3处，此处锁骨较细，无肌肉附着，当胎儿肩娩出受阻时，S 形锁骨凹面正好卡在母亲耻骨弓下，容易折断。大部分患儿无明显症状，故极易漏诊，多因其他情况摄胸片时发现。但仔细观察可发现患儿患侧上臂活动减少或被动活动时哭闹，对锁骨进行常规触诊可发现双侧锁骨不对称，

病侧有增厚模糊感，局部软组织肿胀、有压痛、骨摩擦音，甚至可扪及骨痂硬块，患侧拥抱反射减弱或消失，X摄片可确诊。青枝骨折一般不需治疗；对于完全性骨折，有学者也认为无需处理，随着小儿生长发育，肩部增宽，错位及畸形均自行消失；也可在患侧腋下置一软垫，患肢以绷带固定于胸前，2周可愈合。

三、臂丛神经麻痹

臂丛神经麻痹（brachial plexus palsy）是新生儿周围神经损伤中最常见的一种。由于难产、臀位、肩娩出困难等因素使臂丛神经过度牵拉受损，足月、大于胎龄儿多见。按受损部位不同可分为：①上臂型：又称Duchenne-Erb麻痹，由于第5、6颈神经根最易受损，故此型临床最多见。患侧整个上肢下垂、内收，不能外展及外转。肘关节表现为前臂内收，伸直，不能旋后或弯曲。腕、指关节屈曲，拥抱反射不对称。②中臂型：颈7神经根损伤，桡神经所支配的肌肉麻痹，前臂、腕、手的伸展动作丧失或减弱，而肱三头肌、拇指伸肌为不完全麻痹。③下臂型（Klumpke paralysis）：颈8至胸1神经根受累，腕部屈肌及手肌无力，握持反射弱，临床上较少见。如第1胸椎根的交感神经纤维受损，可引起Horner综合征，表现为瞳孔缩小，睑裂变狭等。磁共振可确定病变部位，肌电图检查及神经传导试验也有助于诊断。预后取决于受损程度，若损伤为神经功能性麻痹，数周内可完全恢复。生后第1周开始作按摩及被动运动，大部分病例可于治疗后2～3个月内获得改善和治愈，如为神经撕裂则留有永久麻痹。

四、面神经麻痹

面神经麻痹（facial nerve palsy）常由于胎头在产道下降时母亲骶骨压迫或产钳助产受损所致。面瘫部位与胎位有密切关系，常为一侧、周围性，眼不能闭合、不能皱眉，哭闹时面部不对称，患侧鼻唇沟浅、口角向健侧歪斜。治疗主要是注意保护角膜，多数患儿在生后1个月能自行恢复，个别因神经撕裂持续未恢复者需行神经移植或神经转移术治疗。

（常立文）

第八章　遗传性疾病

遗传性疾病是由于遗传物质结构或功能改变所导致的疾病，简称遗传病（genetic disease）。科学和社会的进步，医疗卫生水平的提高，使急性感染性疾病得到了有效的控制，人类的疾病谱发生了很大的改变，遗传病所占的地位越来越重要。遗传病种类繁多，涉及全身各个系统，分散在临床各专业，导致畸形、代谢异常、神经和肌肉功能障碍，病死率和残疾率均较高。由于多数疾病无有效治疗方法，存活患儿常伴有智力低下和体格残疾，因此疾病的预防极为重要。遗传病的种类和数量统计结果见表8-1。

表8-1　人类孟德尔遗传遗传病在线（online Mendelian inheritance in man, OMIM）网站对遗传性疾病的统计（2007年1月）

常染色体疾病	16288种
X伴性连锁疾病	974种
Y连锁疾病	56种
线粒体病	63种
总计	17370种*

*其中基因定位明确的疾病有11234种

第一节　概　　述

【染色体与基因】

遗传物质包括细胞中的染色体及其基因。人类细胞染色体数为23对（46条），其中22对男性和女性都一样，称常染色体（autosome），1对染色体男女不同，是决定性别的，称性染色体（sex chromosome），男性为XY，女性为XX。正常男性的染色体核型为46，XY；正常女性的染色体核型为46，XX。而正常人每一个配子（卵子和精子）含有22条常染色体和一条性染色体（X或Y），即22+X或22+Y的一个染色体组称为单倍体（haploid），人类体细胞染色体数目为双倍体（diploid），即2n=46。

细胞的遗传信息几乎都储存在染色体的DNA分子长链上，DNA分子是由两条多核苷酸链依靠核苷酸碱基之间的氢键相连接而成的双螺旋结构。其中一条核苷酸链的腺嘌呤（A）、鸟嘌呤（G）必定分别与另一条上的胸腺嘧啶（T）、胞嘧啶（C）连接，互补成对的A和T、G和C即称为互补碱基对。在DNA长链上，每3个相邻的核苷酸碱基组成的特定顺序（密码子）即代表一种氨基酸，即DNA分子贮存的遗传信息。

基因是遗传的基本功能单位，是DNA双螺旋链上的一段负载一定遗传信息，并在特定条件下表达，产生特定生理功能的DNA片段。基因是编码蛋白质肽链和RNA所必需的核苷酸顺序，人类细胞中的全部基因称为基因组（genome），由30亿个碱基对组成，约有3万个基因。每个基因在染色体上都有自己特定的位置，称为基因位点（locus），二倍体同一对染色体上同一位点的基因及其变异叫等位基因，等位基因中一个异常，一个正常，称为病态杂合子，两个异常者称为病态纯合子。如果致病基因位于常染色体上，杂合状态下发病的称为常染色体显性（AD）遗传病；杂合状态下不发病，纯合状态下才发病的称常染色体隐性（AR）遗传病。如果致病基

因位于X染色体上，依传递方式不同，可分为X-连锁显性或隐性遗传病。

线粒体为细胞的运动、收缩、生物合成、主动运输、信号传导等耗能的过程提供能源。线粒体作为细胞的供能装置，将细胞氧化还原产生的能量以高能磷酸键形式暂时储存起来，是糖、脂肪和蛋白质代谢的最终通路。线粒体基因组（mitochondrial genome, mtDNA）是独立于细胞核染色体外的基因组，具有自我复制、转录和编码功能。线粒体中所含的DNA为环状双链结构的DNA分子（mtDNA），编码多种与细胞氧化磷酸化有关的酶，是独立于细胞核染色体外的遗传物质，这些基因突变所导致的疾病称线粒体基因病。现发现60余种疾病与线粒体基因突变或结构异常有关。

人体基因除以上结构基因之外还存在有一定结构特征的其他序列。最为演出的是含有很多重复序列，例如卫星DNA，可作为基因组的一种多态性标记。另外，目前发现基因组的单核苷酸多态性（single nucleotide polymorphism, SNP）发布广泛，数量达数百万，在分子遗传学连锁分析、种群多样性研究、亲子鉴定以及功能研究等领域中具有重要意义。

【遗传性疾病的分类】

根据遗传物质的结构和功能改变的不同，可将遗传性疾病分为五类：

1．染色体病（chromosomal disorders）　指染色体数目或结构异常，造成许多基因物质的丢失而引起的疾病，已经明确的染色体畸变综合征有100多种。

2．单基因遗传病（single gene disease）　疾病种类极多，在一对基因中只要有1个致病基因存在就能表现性状称显性基因，一对基因需2个基因同时存在病变时才能表现性状称隐性基因。单基因遗传病可进一步分为以下5类遗传方式：①常染色体显性遗传（autosomal dominant inheritance）：致病基因在常染色体上，亲代只要有1个显性致病基因传递给子代，子代就会表现性状。例如软骨发育不全、成骨不全。家系特点是患者为杂合子型，亲代中有1人患病；父母一方有病，子女有50%风险率；父母双方有病，子女有75%风险率；男女发病机会均等；父母的同胞或上代有病，父母无病，子女一般无病。②常染色体隐性遗传（autosomal recessive inheritance）：致病基因在常染色体上，为一对隐性基因。只带1个致病隐性基因的个体不发病，为致病基因携带者。多数遗传性代谢病为常染色体隐性遗传，如苯丙酮尿症、白化病等。家系特点：父母均为健康者，患者为纯合子，同胞中25%发病，25%正常，50%为携带者。近亲婚配发病率增高。③X连锁隐性遗传（X-linked recessive inheritance）：疾病随X染色体传递，女性带有1个隐性致病基因，为表型正常的致病基因携带者。男性只有1条X染色体，即使是隐性基因，也会发病，如血友病等。家系特点是男性患者与正常女性婚配，男性都正常，女性都是携带者；女性携带者与正常男性婚配，男性50%是患者，女性50%为携带者。④X连锁显性遗传（X-linked dominant inheritance）：X连锁显性遗传致病基因在X染色体上。家系特点是患者双亲之一是患者，男性患者后代中女性都是患者，男性都正常；女性患者所生子女，50%为患者。女性患者病情较轻，如抗佝偻病。⑤Y连锁显性遗传（Y-linked inheritance）：Y连锁遗传致病基因位于Y染色体上，只有男性出现症状，由父传子。

3．多基因遗传病（multifactorial diseases）　疾病由多对基因共同作用，每对基因作用微小，但有积累效应，积累到一定数量就发病。这些微效基因的总和加上环境因素的影响，就决定了个体的性状。例如2型糖尿病、高血压、神经管缺陷、唇裂等都属多基因遗传病。

4．线粒体病（mitochondrial diseases）　人类细胞中有一部分DNA存在于细胞浆内，称为线粒体DNA，按母系遗传，含37上基因。基因突变为一组较为独特的遗传病，例如脂肪酸氧化障碍、呼吸链酶缺陷、特殊类型的糖尿病等。

5．基因组印记（genomic imprinting）　基因根据来源亲代的不同而有不同的表达，活性

随亲源而改变，两条染色体如皆来自父源则有不同的表现形式。例如，Prader-Willi综合征和Angelman综合征都是15q11-13缺失，Prader-Willi综合征是父源性15q11-13缺失，Angelman综合征为母源性15q11-13缺失。

【遗传性疾病的诊断和预防】

遗传病的诊断是开展遗传咨询和防治的基础，遗传病的诊断要注意收集以下资料。

1．病史

（1）对有先天畸形、生长发育障碍、智能发育落后、性发育异常或有遗传病家族史者应做全身检查，并且做详细的家系调查和家谱分析，了解其他成员健康情况，了解死产、流产和血缘关系。新生儿期出现黄疸不退、腹泻、持续呕吐、肝大、惊厥、低血糖、酸中毒、高氨血症、电解质异常以及尿中有持续臭味，应疑为遗传性代谢病，并做进一步检查。

（2）记录母亲妊娠史，如胎儿发育情况、母亲有无糖尿病、羊水过少等。糖尿病母亲婴儿畸形发生率高，羊水过多和常有畸形。

（3）应详细询问母亲孕期用药史及疾病史，母孕期患风疹及巨细胞病毒感染能造成胎儿器官畸形，但有感染病史不一定与畸形有因果关系。虽然回顾性流行病学调查认为一些药物与畸形有关，但真正能证实的致畸因素为数很少。

2．体格检查

（1）头面部注意头围，有无小头畸形、小下颌畸形，耳的大小，耳位高低，眼距，眼裂，鼻翼发育，有无唇裂、腭裂和高腭弓，毛发稀疏和颜色。

（2）注意上身长与下身长的比例、指距、手指长度、乳状距离、皮肤和毛发色素、手纹、外生殖器等。注意黄疸、肝、脾大和神经系统症状。嗅到一些不正常的汗味或尿味等，可提示某些遗传病可能，主要见于氨基酸代谢病。

3．实验室检查

（1）染色体核型分析：将一个处于有丝分裂中期的细胞中全部染色体按大小及形态特征有秩序地配对排列，观察有无染色体数目或结构异常。

（2）生物化学检查：测定血、尿、红细胞、白细胞、皮肤成纤维细胞中酶和蛋白质或中间代谢产物。近年在国内逐步开展的遗传性代谢病串联质谱检测技术（MS/MS）、气相色谱-质谱技术（GC/MS）已逐步成为遗传性代谢病诊断的常规检测工具，特别串联质谱技术可诊断多种氨基酸代谢病、有机酸代谢紊乱、脂肪酸和肉碱代谢紊乱等疾病，在临床上发挥着重要作用。

（3）基因诊断：基因诊断是在DNA水平上对受检者的某一特定致病基因进行分析和检测，从而达到对疾病进行特异性分子诊断。

4．预防　由于遗传病多数无法治疗，目前防治的重点主要贯彻预防为主的方针，做好三级预防，防止和减少有遗传病的患儿出生，避免有遗传病患儿生后发病。

（1）一级预防：防止遗传病的发生。近亲结婚所生子女患智能低下的比例比非近亲婚配的要高150部，畸形率也要高3倍多。国家法律禁止直系血缘和三代以内的旁系血缘结婚。凡本人或家族成员有遗传病或先天畸形史、多次在家族中出现或生育过智力低下儿或反复自然流产者，应进行遗传咨询，找出病因，明确诊断，制定合理的婚姻和生育计划。

（2）二级预防：减少遗传病患儿出生。对高危孕妇要进行必要的产前诊断检查，减少遗传病患儿的出生。常用产前诊断方法有早期绒毛活体组织检查、羊膜囊穿刺取样、B型超声检查、甲胎蛋白测定和21-三体综合征的产前筛查等。

（3）三级预防：遗传病出生后的治疗。新生儿疑有遗传病，出生后即尽可能利用血生化检查或染色体分析，作出早期诊断。新生儿疾病筛查是提高人口素质的重要措施之一，通过快

速、敏感的检验方法，对一些先天性和遗传性疾病进行群体筛检，从而使患儿在临床上尚未出现疾病表现，而其体内生化、代谢或者功能已有变化时就作出早期诊断，并且结合有效治疗，避免患儿重要脏器出现不可逆性的损害，保障儿童正常的体格发育和智能发育。目前全国各地主要筛查先天性甲状腺功能减低症和苯丙酮尿症两种导致智能发育障碍的疾病，广东及广西两省根据当地疾病谱的特点增加了葡萄糖-6-磷酸脱氢酶缺乏症筛查。根据我国580万例新生儿筛查统计，苯丙酮尿症发病率为1：11000，先天性甲状腺功能减低症发病率为1：3000。

第二节　染色体病

染色体病是由于各种原因引起的染色体数目或（和）结构异常的疾病，常造成机体多发畸形、智力低下、生长发育迟缓和多系统的功能障碍，故又称之为染色体畸变综合征（chromosomal aberration syndrome）。将一个细胞的全部染色体按标准配对排列进行分析诊断，即是核型分析。

【染色体畸变】

染色体的畸变包括染色体数目异常和结构异常两大类。

1．染色体数目异常　是由于染色体在减数分裂或有丝分裂时不分离，而使46条染色体固有数目增加或减少。如果是整个染色体组增减，产生整倍体变异，含有3个或3个以上染色体组的细胞称多倍体（polyploid），按多倍体的染色体组数，可称为三倍体（69，XXX、69，XXY）和四倍体（92，XXXX、92，XXYY）。多倍体多在胚胎期死亡而流产，临床上罕见。如果是个别染色体的增减，产生非整倍体变异，形成非整倍体（aneuploid）。临床上常见的是在二倍体基础上，少数染色体的增加形成超二倍体（hyperdiploid）或减少形成亚二倍体（hylmdiploid）。亚二倍体中比二倍体染色体数（2n）少一条染色体，称染色体为单体（monosomy），由于基因组的严重失衡，机体难以存活。染色体单体生存的唯一例证是Turner综合征，核型为45，X。超二倍体中比二倍体染色体数增加一条染色体，称染色体为三体（trisomy），是最常见的染色体数目畸变的类型。

如果同一个体的细胞存在两种不同的染色体核型，即体内存在两种或两种以上的细胞系，称为嵌合体（mosaic）。嵌合体中各种细胞系的类型及比例取决于发生染色体不分离时期的早晚，发生得越晚，体内正常二倍体细胞所占比例愈大，临床症状也较轻。

2．染色体结构异常　是由于各种原因造成染色体断裂所引起，断裂后断端富有黏着性，能与其他断端再结合，发生结构重排而导致缺失、倒位、易位、等臂、环形染色体等改变。无论是哪一种结构异常，均可使携载的基因在数量上或排列顺序上发生改变而导致疾病。断裂的片段形成易位后，基因没有缺失或增加的称平衡易位（balanced translocation），临床无症状，但这种平衡易位染色体携带者的子代易患染色体病。

【染色体畸变的原因】

能导致染色体畸变的原因见表8-2。

表8-2　能导致染色体畸变的原因

染色体畸变原因	描述
1．物理因素	放射线能诱发染色体畸变，畸变率随射线剂量的增高而增高，孕母接触放射线后，其子代发生染色体畸变的危险性增高
2．化学因素	许多化学药物（如抗代谢药物、抗癫痫药物等）和农药、毒物（如苯、甲苯、砷等）可致染色体畸变增加
3．生物因素	一些病毒例如风疹病毒、巨细胞病毒、麻疹病毒、腮腺炎病毒的感染可引起胎儿染色体断裂
4．孕妇年龄	孕妇年龄大是引起21-三体综合征和其他三体型的主要原因之一，可能与生殖细胞的老化有关
5．遗传因素	染色体异常的父母可能传给下一代，最明显的例子是一些平衡易位的携带者

【染色体病的临床特征】

1．常染色体病　即常染色体数目异常或结构畸变所产生的综合征，其共同的特征为：①生长发育迟缓；②智能发育落后；③多发性先天畸形：内脏畸形、骨骼畸形、特殊面容、皮肤纹理改变。最常见的是21-三体综合征，其次是18-三体综合征、13-三体综合征及$5P^-$综合征等。

2．性染色体病　即性染色体X或Y数目异常或结构的畸变。一般没有常染色体病严重，常伴有性征发育障碍或异常，最常见的是Turner综合征、Klinefelter综合征，其次尚有XYY、多X等综合征。

【染色体核型分析的指征】

在临床上，若患者出现以下情况则需考虑进行染色体核型分析检查：①怀疑患有染色体病者；②有多种先天性畸形；③有明显生长发育障碍或智能发育障碍；④性发育异常或不全；⑤孕母年龄过大、不孕或多次自然流产史；⑥有染色体畸变家族史。

一、21-三体综合征

21-三体综合征又称Down's综合征，以前也称先天愚型，是人类最早被确定的染色体病，在活产婴儿中发生率约为1：600～1：1000，母亲年龄愈大，发生率愈高。

【遗传学基础】

细胞遗传学特征是第21号染色体呈三体征（trisomy 21），其发生主要是由于生殖细胞在减数分裂形成配子时，或受精卵在有丝分裂时，21号染色体发生不分离，使胚胎体细胞内存在一条额外的21号染色体。

【临床表现】

本病主要特征为智能落后、特殊面容和生长发育迟缓，并可伴有多种畸形。

1．智能落后　绝大部分患儿都有不同程度的智能发育障碍，随年龄的增长日益明显。嵌合体型患儿若正常细胞比例较大则智能障碍较轻。

2．生长发育迟缓　患儿出生的身长和体重均较正常儿低，生后体格发育、动作发育均迟缓，身材矮小，骨龄落后于实际年龄，出牙迟且顺序异常；四肢短，韧带松弛，关节可过度弯曲；肌张力低下，腹膨隆，可伴有脐疝；手指粗短，小指尤短，中间指骨短宽且向内弯曲。

3．特殊面容　出生时即有明显的特殊面容，表情呆滞。眼裂小，眼距宽，双眼外眦上斜，可有内眦赘皮；鼻梁低平，外耳小；硬腭窄小，常张口伸舌，流涎多；头小而圆，前囟大且关闭延迟；颈短而宽。

4．皮纹特点　可有通贯手和特殊皮纹。

5．伴发畸形　约50％患儿伴有先天性心脏病，其次是消化道畸形。先天性甲状腺功能减低症和急性淋巴细胞性白血病的发生率明显高于正常人群。免疫功能低下，易患感染性疾病。

【实验室检查】

1．细胞遗传学检查　根据核型分析可分为三型：

（1）标准型：约占患儿总数95％，患儿体细胞染色体为47条，有一条额外的21号染色体，核型为47，XY（或XY），+21。

（2）易位型：约占2.5％～5％，染色体总数为46条，其中一条是额外的21号染色体的长臂与一条近端着丝粒染色体长臂形成的易位染色体，即发生于近着丝粒染色体的相互易位，称罗伯逊易位（Robertsonian translocation），亦称着丝粒融合。以14号染色体为主，少数为15号或13号染色体，最常见核型为46，XY（或XX），－14，＋t（14q21q）。

（3）嵌合体型：此型约占2%～4%，由于受精卵在早期分裂过程中发生了21号染色体不分离，患儿体内存在两种细胞系，一种为正常细胞，另一种为21-三体细胞，形成嵌合体，其核型为46，XY（或XX）／47，XY（或XX），+21。此型患儿临床表现的严重程度与异常细所占百分比有关。

2．荧光原位杂交　以21号染色体的相应片段序列作探针，与外周血中的淋巴细胞或羊水细胞进行原位杂交，可快速、准确进行诊断。在本病患者的细胞中呈现3个21号染色体的荧光信号。

【诊断与鉴别诊断】

典型病例根据特殊面容、智能与生长发育落后、皮纹特点等不难作出临床诊断，但应作染色体核型分析以确诊。新生儿或症状不典型者更需核型分析确诊。

本病应与先天性甲状腺功能减低症鉴别，后者有颜面黏液性水肿、头发干燥、皮肤粗糙、喂养困难、便秘、腹胀等症状，可测血清TSH、T4和染色体核型分析进行鉴别。

【遗传咨询】

标准型21-三体综合征的再发风险为1%，孕母年龄愈大，风险率愈高。少数有生育能力的女性患者，其子代发病概率为50%。在易位型中，再发风险为4%～10%，若母亲为21q22q平衡易位携带者，子代发病风险率为100%。

【产前筛查】

对高危孕妇可作羊水细胞或绒毛膜细胞染色体检查进行产前诊断。目前还可在孕中期筛查相关血清标记物，采用测定孕妇血清绒毛膜促性腺激素（HCG）、甲胎蛋白（AFP）、游离雌三醇（FE_3），结合孕母年龄，可计算其本病的危险度。采用这一方法可以检出大约60%～80%的21-三体综合征胎儿。此外，通过B超测量胎儿颈项皮肤厚度也是诊断21-三体综合征的重要指标。

【治疗】

目前尚无有效的治疗方法。要采用综合措施，包括医疗和社会服务，对患者进行长期耐心的教育。要训练弱智儿掌握一定的工作技能。对患儿宜注意预防感染，如伴有先天性心脏病、胃肠道或其他畸形，可考虑手术矫治。

二、先天性卵巢发育不全综合征

本病由Turner于1938年首先报道，故称为Turner综合征。1959年证实该病因性染色体X呈单体性所致。Turner综合征的表型是女性，在活产女婴中约占0.3‰～0.4‰，其发生率低是因为X单体的胚胎不易存活，约99%的病例发生流产。该病也是人类唯一能生存的单体综合征。

【遗传学基础】

Turner综合征是由于细胞内X染色体缺失或结构发生改变所致，可能的机制为：①亲代生殖细胞的减数分裂发生不分离；②在有丝分裂过程中X染色体的部分丢失。

【临床表现】

典型的Turner综合征患者在出生时即呈现身高、体重落后，在新生儿期可见颈后皮肤过度折叠以及手、足背发生水肿等特殊症状。

患者为女性表型，生长缓慢，身材矮小，成年期身高约135～140cm；颈短，50%有颈蹼，后发际低；两乳头距离增宽，随年龄增长乳头色素变深；皮肤多痣，有肘外翻。青春期无性征发育、原发性闭经、外生殖器呈幼稚型，婚后不育。患者常伴有其他先天畸形，如主动脉缩窄、肾脏畸形（马蹄肾、易位肾等）、指（趾）甲发育不良、第4、5掌骨较短等。大多数患儿智能

正常或稍低。患者多因身材矮小、青春期无性征发育、原发性闭经等而就诊。

【实验室检查】

1．外周血细胞染色体核型分析　用外周血淋巴细胞培养技术进行核型分析，先天性卵巢发育不全综合征的异常核型有以下几种类型：①单体型：45，X是最多见的一种，约占60%。这种核型的个体绝大部分在妊娠早期自然流产，其余存活的个体具有典型的临床症状。②嵌合型：核型为45，X／46，XX，约占该病的25%。细胞类型以46，XX为主的个体临床症状较轻，约20％的患者可有月经来潮，部分可有生育能力。若患者以45，X细胞为主，其表型与单体型相似。③X染色体结构异常：46，Xdel（Xq）或者46，Xdel（Xp），即一条X染色体长臂或短臂缺失，同时伴有X染色体易位等。

2．血促性腺激素　FSH、LH明确升高，E_2降低，提示卵巢功能衰竭。

3．腹部B超　显示子宫、卵巢发育不良，严重者呈纤维条索状。

【治疗】

本病的治疗以改善其成人期最终身高和性征发育，保证患儿心理健康为目的。重组人生长激素对Turner综合征患儿身高改善有作用，每晚临睡前皮下注射0.15U/kg。开始治疗年龄越小，效果越好。

在青春期可用雌激素进行替代疗法，一般从12～14岁开始，先用小剂量炔雌醇12.5μg/d治疗6～12个月，以促使乳房及外阴发育，保障患儿心理健康，对预防骨质疏松有一定作用。然后进行周期性的雌激素-孕激素疗法（人工周期治疗）。每月1～20日连续口服炔雌醇，第21天停药，在服药第10天后加甲羟孕酮2～4mg/d，并与炔雌醇同时停药，停药后可引起子宫撤退性出血。采用倍美盈（premelle cycle）进行人工周期治疗效果亦较好。

极少数嵌合型患者可能有生育能力，但其流产或者死胎率极高，30%后代患有染色体畸变。

三、先天生睾丸发育不全综合征

先天性睾丸发育不全综合征又称Klinefelter综合征（Klinefelter syndrome），是一种发病率较高的性染色体疾病，由于性染色体异常导致睾丸发育不全、不育和智能低下。是男性不育的常见原因之一。

【临床表现】

男性表型，体格较高，间距大于身高。乳房女性化约占40%。青春期发育常延缓，由于无精子，一般不能生育（偶有例外）。体格检查发现男性第二性征不明显，无胡须，无喉结，皮肤白皙，睾丸小，阴茎亦小，可有隐睾或尿道下裂，阴毛发育差。

患者可有性格孤僻、腼腆、胆小，缺乏男孩性格。在标准型47，XXY核型中，约有25%显示中等度智能发育落后，表现为语言和学习障碍。

【实验室检查】

1．外周血细胞染色体核型分析　该病性染色体标准型为三体型47，XXY，也可有性染色体四体型或者五体型，例如48，XXXY；48，XXYY；49，XXXXY；49，XXXYY，不同类型的嵌合体也较常见。

2．生化检验　患者血清中睾酮降低，垂体促性腺激素、黄体生成激素（LH）、卵泡刺激素（FSH）升高。

3．其他检验　患者精液中一般无精子生成，病理检查见曲细精管玻璃样变，其睾丸间质细胞（Leydig细胞）虽有增生，但内分泌活力不足。

【治疗】

本病需尽早确诊，自幼开始强化教育和训练，促进智能发育及正常性格形成。患者自11～12岁开始，应进行雄激素疗法。一般可采用长效睾酮制剂，如庚酸睾酮治疗，开始每次肌注50mg，每3周1次，每隔6～9个月增加剂量50mg，直至达到成人剂量（每3周200mg）。

第三节　遗传性代谢病

遗传性代谢病（inborn erroers of metabolism, IEM）于1908年首次被提出，是遗传性生化代谢缺陷的总称，是由于基因突变，引起蛋白质分子在结构和功能上发生改变，导致酶、受体、载体等的缺陷，使机体的生化反应和代谢出现异常，反应底物或者中间代谢产物在体内大量蓄积，引起一繁殖临床表现的一大类疾病。

遗传性代谢病种类繁多，目前已达数千种，常见有400～500种，单一病种患病率较低，但是总体发病率较高、危害严重，是临床的疑难杂症。患者若得不到及时诊治，常可致残，甚至危及生命，给社会和家庭带来沉重负担。近几十年来，随着生化测定和基因诊断技术的不断发展，遗传性代谢病的诊治和预防水平也不断在提高。

【遗传性代谢病的种类】

遗传性代谢病可根据先天性缺陷所累及的生化物质进行分类（表8-3）。约80%属常染色体隐性遗传，其余为X连锁遗传、常染色体显性遗传或线粒体遗传。

表8-3　遗传性代谢病的分类及主要疾病

氨基酸代谢病
苯丙酮尿症、枫糖尿病、同型胱氨酸血症、高甲硫氨酸血症、白化病黑酸尿症、酪氨酸血症、高鸟氨酸血症、瓜氨酸血症、精氨酸酶缺乏症等
糖代谢病
半乳糖血症、葡萄糖-6-磷酸脱氢酶缺乏症、果糖不耐受症、糖原累积病、磷酸烯醇丙酮酸羧化酶缺陷等
脂肪酸氧化障碍
肉碱转动障碍、肉碱棕榈酰转移酶缺乏症、短链酰基辅酶A脱氢酶缺乏症、中链酰基辅酶A脱氢酶缺乏症、极长链酰基辅酶A脱氢酶缺乏症
尿素循环障碍及高氨血症
氨甲酰磷酸合成酶缺陷、鸟氨酸氨甲酰转移酶缺陷、瓜氨酸血症、精氨酸琥珀酸血症、精氨酸酶缺陷、N-乙酰谷氨酸合成酶缺陷等
谷氨酸合成酶缺陷等
有机酸代谢病
甲基丙二酸血症、丙酸血症、异戊酸血症、多种辅酶A羧化酶缺乏症、戊二酸血症等
溶酶体蓄积症
戈谢病、黏多糖病、GM1神经节苷脂蓄积症、尼曼-皮克病等
线粒体代谢异常
Leigh综合征、高乳酸血症、线粒体脑病、线粒体肌病
核酸代谢异常
着色性干皮病、次黄嘌呤鸟嘌呤磷酸核糖转移酶缺陷症
金属元素代谢异常
肝豆状核变性（Wilson病）、Menkes病
内分泌代谢异常
先天性肾上腺皮质增生症（21-羟化酶缺乏症、11-羟化酶缺乏症、17-羟化酶缺乏症）
其他
卟啉病、α_1-抗胰蛋白酶缺乏，囊性纤维变性、葡萄糖醛酸转移酶缺乏症等

【遗传性代谢病的代谢紊乱】

由于酶的生理功能是催化底物转变为产物，因此几乎所有因酶代谢缺陷所引起的病理改变都直接或间接地与底物的堆积、产物的缺乏有关，在病理情况下堆积之常常循旁路代谢途径产生大量旁路代谢产物，也可造成病理性损害。例如在苯丙酮尿症时，苯丙氨酸羟化酶缺乏，导致底物苯丙氨酸增高，代谢旁路开放，代谢产物苯乙酸、苯乳酸增高，这些物质的毒性作用造成了神经系统的损害。在21-羟化酶缺乏时，造成产物皮质醇、醛固酮缺乏，导致临床水、电解质紊乱和休克。这是基因突变导致先天性代谢缺陷发病的基本机制。当然在不同的疾病类型中常以某一种情况、或底物堆积、或产物缺乏、或旁路代谢产物产生为主，产生病理损害。

【遗传性代谢病常见的症状与体征】

遗传性代谢病可在婴幼儿期、儿童期、青少年期发病，其临床表现有急性危象期、缓解期和缓慢进展期。急性症状和检验异常包括急性代谢性脑病、高氨血症、代谢性酸中毒、低血糖等，随年龄不同有差异，全身各器官均可受累，以神经系统以及消化系统的表现较为突出，有些有容貌异常，毛发、皮肤色素改变。部分遗传性代谢病在婴儿早期即可有临床表现（表8-4）。

表8-4　遗传性代谢病在新生儿期主要临床表现

喂养困难、食欲差、呕吐、体重不增
嗜睡、惊厥、昏迷
呼吸困难、酸中毒、过度换气
肌张力异常
肝大
皮肤病变、毛发异常
特殊尿味、汗味
黄疸
脱水、持续呕吐、电解质异常

【遗传性代谢病的诊断】

遗传性代谢病的诊断需要依赖实验室检查，如尿液三氯化铁试验、尿液二硝基苯肼（DNPH）试验和乙酸试验、尿液硝普盐试验（Brand反应）、甲苯胺蓝试验等可以对某些疾病进行初步筛查。血、尿常规分析、生化检测如血糖、血气分析、肝功能、胆红素、血氨、乳酸、酮体、丙酮酸、肌酐、尿素、电解质、钙、磷测定，有助于对遗传性代谢病作出初步的诊断或者缩小诊断范围。

遗传性代谢病的确诊需根据疾病进行氨基酸分析、铜蓝蛋白、17-羟孕酮等特异性底物或者产物的测定。串联质谱技术（tandem mass, MS/MS）已成为遗传性代谢病的常规诊断工具，能对一个标本一次进行30多种氨基酸、有机酸、脂肪酸代谢性疾病的检测，气相色谱-质谱联用仪（gas chromatography mass spectrometry GC/MS）的应用对诊断有机酸尿症和某些疾病有重要意义。基因诊断对遗传病的确定和准确分型越来越重要。对于怀疑遗传性代谢现濒临死亡的婴儿，应留取适当的标本，以便进行分析，明确病因，为遗传咨询和产前诊断提供依据。

目前根据国家“母婴保健法”的规定，新生儿疾病筛查正在全国逐步推广，除了对先天性甲状腺功能减低症、苯丙酮尿症新生儿期筛查外，有的地区开展了G-6-PD缺乏症、先天性肾上腺皮质增生症筛查，个别城市已经开展了串联质谱新技术的遗传性代谢病筛查，大大扩大了筛查的疾病谱。通过尽早确诊和积极治疗，可大大降低遗传性代谢病的危害性。

一、苯丙酮尿症

苯丙酮尿症（phenylketonuria, PKU）是一种常染色体隐性遗传疾病，是先天性氨基酸代谢障碍中较为常见的一种，因患儿尿液中排出大量苯丙酮酸代谢产物而得名。发病率随种族不同而异，我国的发病率总体为1：11000，北方人群高于南方人群。

【发病机制】

苯丙氨酸（phenylalanine, Phe）是人体必需氨基酸之一，食入体内的Phe一部分用于蛋白质的合成，另一部分通过苯丙氨酸羟化酶（phenylalanine hydroxylase, PAH）作用转变为酪氨酸，仅有少量的Phe经过次要代谢途径在转氨酶的作用下转变成苯丙酮酸，其代谢途径见图8-1。

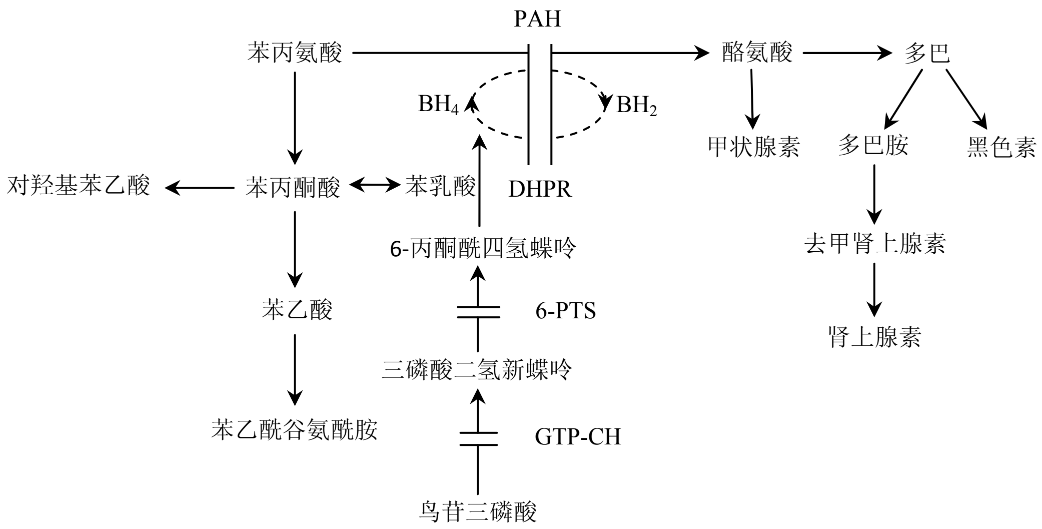

图8-1 苯丙氨酸主要代谢图

PKU是由于患儿肝脏缺乏苯丙氨酸羟化酶活性，不能将苯丙氨酸转化为酪氨酸，导致苯丙氨酸在血液、脑脊液、各种组织中的浓度极度增高，通过旁路代谢产生大量苯丙酮酸、苯乙酸、苯乳酸和对羟基苯乙酸。高浓度的Phe及其代谢产物能导致脑组织损伤。

人类苯丙氨酸羟化酶基因位于第12号染色体上（12q22～12q24），基因全长约90kb，有13个外显了和12个内含子，成熟的mRNA约2.4kb，编码451个氨基酸。通过对PKU患者进行基因分析，在中国人群中已经发现了70种以上基因突变。

苯丙氨酸的代谢，除了需要有苯丙氨酸羟化酶的作用外，还必须要有辅酶四氢生物蝶呤（tetrabiopterin, BH_4）参与，人体内的BH_4来源于三磷酸鸟苷（GTP-CH）、6-丙酮酰四氢蝶呤合成酶（PTPS）和二氢生物蝶呤还原酶（DHPR）的催化。PAH、GTPGH、PTS、DHPR等酶的编码基因缺陷都有可能造成相关酶的活力缺陷，导致血苯丙氨酸升高。BH_4是苯丙氨酸、酪氨酸和色氨酸等芳香氨基酸在催化过程中所必须的共同的辅酶，缺乏时不仅苯丙氨酸不能氧化成酪氨酸，而且造成多巴胺、5-羟色胺等重要神经递质的合成受阻，加重了神经系统的功能损害。

根据统计，在新生儿筛查中发现的高苯丙氨酸血症，大多数为PKU，约10%～15%为BH_4缺乏症，国内目前发现全部是PTPS缺乏类型。

【临床表现】

患儿出生时正常，通常在3～6个月时始出现症状，1岁时症状明显，表现为：

1．神经系统　智能发育落后最为突出，智商常低于正常。有行为异常，如兴奋不安、忧郁、多动、孤僻等。可有癫痫小发作，少数呈现肌张力增高和腱反射亢进。

2．皮肤　患儿在出生数月后因黑色素合成不足，头发由黑变黄，皮肤白皙。皮肤湿疹较常见。

3．体味　由于尿和汗液中排出较多苯乙酸，可有明显鼠尿臭味。

【实验室检查】

1．新生儿疾病筛查　新生儿哺乳3天后，针刺足跟采集外周血，滴于专用采血滤纸上，晾干后即寄送至筛查实验室，进行苯丙氨酸浓度测定。如Phe浓度大于切割值，进一步检查和确诊。

2．苯丙氨酸浓度测定　正常浓度小于120μmol/L（2mg/dl），经典型PKU＞1200μmol/L。

3．尿三氯化铁（$FeCl_3$）及2,4-二硝基苯肼试验（DNPH）　一般用于较大儿童的初筛。新生儿PKU因苯丙氨酸代谢旁路尚未健全，患者尿液测定为阴性。

4．尿蝶呤图谱分析　主要用于PKU的鉴别诊断。尿蝶呤谱分析应用高压液相层析（HPLC）测定尿液中新蝶呤（N）和生物蝶呤（B）的含量。如因6-丙酮酰四氢蝶呤合成酶缺乏所致的BH_4缺乏症，尿中新蝶呤明显增加，生物蝶呤下降，N/B增高，比值（B/B＋N%）＜10%。尿蝶呤谱分析显示异常者需进一步作口服BH_4负荷试验，以助确诊。

5．DNA分析　目前对苯丙氨酸羟化酶、6-丙酮酰四氢蝶呤合成酶、二氢生物蝶呤还原酶等基因缺陷都可用DNA分析方法进行基因突变检测和诊断，可进行产前诊断。

【诊断与鉴别诊断】

根据智能落后、头发由黑变黄、特殊体味和血苯丙氨酸升高可以确诊。本病应力求早期诊断与治疗，以避免神经系统的损伤。

PKU需与以下疾病鉴别：

1．暂时性高苯丙氨酸血症　见于新生儿或早产儿，可能为苯丙氨酸羟化酶成熟延迟所致。生后数月苯丙氨酸可逐渐恢复正常。

2．四氢生物蝶呤缺乏症　又称非经典型PKU，由于PAH辅助因子BH_4缺乏所致。患儿除了有典型PKU表现外，神经系统表现较为突出，如肌张力异常、不自主运动、震颤、阵发性角弓反张、惊厥发作等。该病的发生率占PKU的10%左右，诊断主要依靠HPLC测定尿蝶呤谱。

【治疗】

1．疾病一旦确诊，应立即治疗。开始治疗的年龄愈小，预后越好。

2．患儿主要采用低苯丙氨酸奶方治疗，待血浓度降到理想浓度时，可逐渐少量添加天然饮食，其中首选母乳，因母乳中苯丙氨酸含量仅为牛奶的1/3（表8-5）。较大婴儿及儿童可加入牛奶、粥、面、蛋等，添加食品应以低蛋白、低苯丙氨酸食物为原则，其量和次数随血苯丙氨酸浓度而定。Phe浓度过高或者过低都将影响生长发育。

3．由于每个患儿对苯丙氨酸的耐受量不同，故在饮食治疗中，仍需定期测定血苯丙氨酸，根据患儿具体情况调整食谱。低苯丙氨酸饮食治疗至少持续到青春期，终生治疗对患者更有益。

4．成年女性患者在怀孕前应重新开始饮食控制，血苯丙氨酸应该在300μmol/L以下，直至分娩，以免高苯丙氨酸血症影响胎儿。

5．对有本病家族史的夫妇及先证者可进行DNA分析，对其胎儿进行产前诊断。

6．对诊断BH_4缺乏症患者，治疗需补充BH_4、5-羟色胺和L-DOPA，一般不需饮食治疗。

表8-5　不同年龄血苯丙氨酸理想控制范围

年龄	血苯丙氨酸浓度（μmol/L）
0～3岁	120～240
3～9岁	180～360
9～12岁	180～480
12～16岁	180～600
＞16岁	180～900

二、肝豆状核变性

肝豆状核变性（hepatolenticular degeneration, HLD）又称Wilson病，是一种常染色体隐性遗传性疾病，因P型ATP7B基因异常，导致铜在体内储积。临床上以肝硬化、眼角膜K-F环和锥体外系症状及体征三大表现为特征。发病率约为1：30000，我国南方沿海地区的发病数相对较内地多。

【发病机制】

铜（Cu）是人体所必需的微量元素之一，是体内氧化还原酶的辅助因子。肝脏是进行铜代谢的主要器官，铜蓝蛋白由肝细胞合成。铜的摄入主要来源于食物中，以Cu^{2+}的形式参与代谢。细胞膜内外Cu^{2+}的转动体是P型ATP酶，即ATP7A和ATP7B两种酶。ATP7A酶将主动吸收的铜与血中的蛋白结合，运至肝脏进一步代谢，缺乏ATP7A酶将导致铜缺乏，即Menkes病。ATP7B酶主要将Cu^{2+}递交给铜蓝蛋白并使多余的铜经胆汁排泄。肝豆状核变性主要因ATP7B基因突变，铜蓝蛋白和铜氧化酶活性降低，铜自胆汁中排出锐减，但由于患者肠道吸收铜功能正常，因此大量铜蓄积在体内重要脏器组织，影响细胞的正常功能。

ATP7B基因定位于染色体12q14.3-21.1区域，含21个外显子，cDNA全长约7.5kb，编码1411个氨基酸。目前已经发现各种类型的ATP7B基因突变达150种以上。ATP7B基因突变类型在不同种族地区存在明显差异，中国人的突变以外显子8较高，其中R778L突变最常见。

【病理】

肝细胞最初呈现脂肪浸润改变，以门静脉区周围为显著。溶酶体内含有脂质颗粒，过氧化酶体形态不一，且其基质呈颗粒状或絮状。随病程进展，肝组织出现纤维化和肝硬化改变。脑的病变主要位于神经节的豆状核及尾状核，脑胶质细胞内及毛细血管周围可见铜沉积。肾脏可见肾小管上皮细胞变性，细胞质内有铜沉积。角膜铜颗粒主要沉积于周边部分，形成环状，称K-F环（Kayser-Fleisher ring）。

【临床表现】

从出生开始到发病前为无症状期，随着体内铜积量的增加，患儿逐渐出现器官受损症状，发病年龄以7～12岁最多见。

临床表现以肝脏损害最常见，可呈慢性或者急性发病。肝脏病损表现轻重不一，可表现有肝硬化、慢性活动性肝炎、急性或亚急性肝炎和爆发性肝炎等，有时初诊就发现有肝硬化。严重者出现肝、脾质地坚硬、腹水、食管静脉曲张、脾功能亢进、出血倾向和肝功能不全的表现。

神经系统的症状也较为常见，较多在10岁以后出现，症状轻时不易发现，当家长察觉时疾病已进入中后期，患者可出现程度不等的锥体外系症状，如腱反射亢进、病理反射等，有肌张

力改变、精细动作困难、肢体震颤、面无表情、构音及书写困难等。

其他伴发的症状可有溶血性贫血、血尿或蛋白尿、精神心理异常等。

眼睛角膜早期可正常，晚期患者在眼角膜出现K-F环。

【实验室检查】

1．血清铜蓝蛋白测定　小儿正常值为200～400mg/L，患者通常低于200mg/L。

2．血清铜氧化酶活性　铜氧化酶吸光度正常值为0.17～0.57，患者明显降低。

3．24小时尿铜排出量测定　正常＜40μg，患儿可高达100～1000μg。

4．K-F环检查　在角膜边缘可看见形成的呈棕灰、棕绿或棕黄色的色素环，色素环宽约1～3mm。K-F环自角膜上缘开始出现，然后成为环状。早期需在眼科裂隙灯下检查，以后肉眼亦可见到。

5．X线检查　常见骨质疏松、关节间隙变窄或骨赘生等病变。

【诊断】

根据肝脏和神经系统症状、体征和实验室检查结果，特别是角膜K-F环阳性，血清铜蓝蛋白低于200mg/L，铜氧化酶吸光度低于0.17可确立诊断。

【治疗】

治疗目的是防止或减少铜在组织内蓄积，患者应终身治疗。开始治疗越早，预后越好。早期治疗可使症状消失。

1．促进铜排泄的药物主要有

（1）青霉胺（penicillamine）：剂量为每日20mg/kg，分2～3次饭前半小时口服。首次服用应作青霉素皮内试验，阴性才能使用，阳性者酌情脱敏试验后服用。青霉胺还可引起维生素B_6缺乏，每日应补充维生素B_6 10～20mg，每日3次。服用青霉胺期间应定期检查血、尿常规和24小时尿铜等变化。

（2）盐酸三乙撑四胺（triethylene-tetramine dihydrochlorate）及连四硫代钼酸胺（TTM）：这两种铜络合剂适用于不能使用青霉胺者。

2．减少铜吸收的药物　常用锌制剂，服后大便排铜增加，减少体内铜的蓄积。常用制剂为硫酸锌，儿童用量每次0.1～0.2g，每日2～3次口服。年长儿可增至每次0.3g，每日3次。服药后1小时内禁食以避免影响锌吸收。重症患者不宜首选。

青霉胺与锌盐联合治疗可减少青霉胺用量，青霉胺每日7～10mg/kg，4～6个月后仅用锌作维持治疗。轻症者单用锌盐也可改善症状。两药合用时最好间隔2～3小时，以免影响疗效。

3．低铜饮食　避免食用含铜量高的食物，如肝、贝壳类、蘑菇、蚕豆、豌豆、玉米和巧克力等。

三、糖原累积病

糖原累积病（glycogen storage disease, GSD）是一组由于先天性酶缺陷所造成的糖原代谢障碍性疾病。这类疾病的共同特征是糖原代谢异常，多数疾病可见到糖原在肝脏、肌肉、肾脏等组织中储积量增加。有的类型以肝脏病变为主，有时以肌肉组织受损为主。

GSD依其所缺陷的酶可分为12型，多数属分解代谢上的缺陷，使糖原异常堆积。除GSD Ⅸb型为X连锁隐性遗传外，其余都是常染色体隐性遗传性疾病。表8-6为部分糖原累积病的酶缺陷与主要临床表现。

表8-6　部分糖原累积病的酶缺陷和主要临床表现

型号和病名	酶缺陷	主要临床表现
0型	糖原合成酶	酮症低血糖，智能低下
Ⅰa型 Von Gierke病	葡萄糖-6-磷酸酶	矮小，肝大，低血糖
Ⅱ型 Pompe病	α-1，4-葡萄糖苷酶	肌张力低下，心脏扩大
Ⅲ型 Cori病	脱支酶	低血糖，惊厥，肝大
Ⅳ型 Andersen病	分支酶	肝大，进行性肝硬化
Ⅴ型 McArdle病	肌磷酸化酶	疼痛性肌痉挛，血红蛋白尿，继发性肾衰竭
Ⅵ型 Hers病	肝磷酸化酶	轻度低血糖，生长迟缓，肝大
Ⅶ型 Tarui病	肌磷酸果糖激酶	肌痉挛，肌红蛋白尿
Ⅸ型	肝磷酸化酶激酶	肝大

糖原累积病Ⅰa型

【发病机制】

糖原累积病Ⅰa型是由于葡萄糖-6-磷酸酶（G6Pase）缺陷所导致的常染色体隐性遗传的代谢性疾病，其活产儿发病率为1/100000，在各型中最为多见。

G6Pase基因位于17号染色体长臂2区1带，约有12.5kb，包含5个外显子。G6Pase蛋白为细胞内质网膜蛋白，包含357个氨基酸。迄今为止，G6Pase基因编码区已发现100余种突变。不同的种族和不同地区的人群有不同的突变类型，白种人中以R83C和Q347X突变为最常见，我国和日本以727g＞t突变最为常见。

【临床表现】

患儿临床表现轻重不一，呈娃娃脸，肌张力低下，智能发育多数正常。重症在新生儿期即可出现严重低血糖、酸中毒、呼吸困难和肝大等症状和体征，少数可出现低血糖惊厥。患儿有高乳酸血症、高尿酸血症。

轻者在幼儿期表现为生长落后、身材矮小、低血糖、肝大、易感染，也可出现高脂血症，一些患儿尽管血糖很低，但无明显的低血糖症状，往往因肝大就诊，经生化检查才发现低血糖。

患者可出现骨质疏松，由于血小板功能不良，患儿常有鼻出血等出血倾向，可并发肾病或肾功能异常。

【实验室检查】

1．血液生化测定　低血糖、酮症酸中毒、乳酸血症，血脂及尿酸升高，肝功能多数正常。

2．肾上腺素试验　皮下注射1：1000肾上腺素0.02ml/kg，注射前、后10分钟中、20分钟、30分钟、40分钟、50分钟、60分钟测定血糖，正常者血糖上升40%～60%，患者血糖无明显上升。

3．胰高血糖素试验　肌注胰高血糖素30μg/kg（最大量1mg），于注射后0分钟、15分钟、30分钟、45分钟、60分钟、90分钟、120分钟取血测血糖。正常时在15～45分钟内血糖可升高1.5～2.8mmol/L，患者血糖升高不明显。

4．肝组织活体检查和酶活力测定　肝组织糖原染色见糖原增多，特异性酶活性降低。

5．外周血白细胞DNA分析，进行基因诊断。

【诊断】

根据病史、体征和血生化检测结果可作出临床诊断，肾上腺素或胰高血糖素等试验可辅助

诊断。准确分型需进行酶学测定和基因诊断。

【治疗】

1．无病因治疗，可做一些对症处理。

2．在严重低血糖时，静脉给予葡萄糖0.5g/（kg.h），根据血糖进行调整。日间少量多次喂给糖类食物和夜间使用鼻饲点滴葡萄糖（10mg/（kg.min）维持），维持血糖4～5mmol/L为宜。

3．一岁后中用生玉米淀粉治疗，每4～6小时喂给1.75～2.0g/（kg.次），以防治低血糖和乳酸血症。

4．包含治疗需注意补充各种微量元素和矿物质。

5．家庭中未发病的同胞兄妹，应定期检查，以便作出早期诊断。家庭如需生育第二胎，可进行遗传咨询，进行产前基因诊断。

四、黏多糖病

黏多糖病（mucopolysaccharidosis, MPS）是一组先天性遗传病，因黏多糖降解酶缺乏使酸性黏多糖不能完全降解，导致黏多糖积聚在机体的不同组织，产生骨骼畸形、智能障碍等一繁殖临床症状和体征。

【发病机制】

黏多糖是结缔组织细胞间的主要成分，广泛存在于各种细胞内。黏多糖是带阴性电荷的多聚物重要的黏多糖有硫酸皮肤素（dermatan sulfate, DS）、硫酸类肝素（heparan sulfate, HS）、硫酸角质素（keratan sulfate, KS）、硫酸软骨素（chondroitin sulfate, CS）、透明质酸（hyaluronic acid, HA）等，前3种是黏多糖病的主要病理性黏多糖。这些黏多糖都是直链杂多糖，由不同的双糖单位连接而成，包括N-乙酰氨基已糖和糖醛酸或者已糖组成。每个氨基聚糖直链约由50～100个分子组成，许多直链又同时与一条蛋白质肽链结合，形成更大分子量的聚合体。结缔组织便是由这类聚合体所形成。这种多糖酶的降解必须在溶酶体中进行。正常溶酶体中含有许多种糖苷酶、硫酸酯酶和乙酸转移酶，不同的黏多糖需不同的溶酶体酶进行降解。已知有10种溶酶体酶参与其降解过程，其中任何一种酶的缺陷都会造成氨基葡聚糖链分解障碍，在溶酶体内积聚，尿中排出增加。患儿缺陷的酶活性常仅及正常人的1%～10%。

【临床表现】

1．体格发育障碍　患者一般出生时正常，随年龄增大，临床症状逐渐明显，其共同特征是在出生1年左右出现生长落后，主要表现为矮小、面容较丑陋，例如表情淡漠、头大、眼裂小、眼距宽、鼻梁低平、鼻孔大、唇厚、前额和双颧突出、毛发多而发际低、颈短等。有的类型有角膜混浊、关节进行性畸变、胸廓畸形、脊柱后凸或侧凸、膝外翻、爪形手、早期出现肝、脾大、耳聋、心脏增大等。

2．智能发育落后　患儿精神神经发育在周岁后逐渐迟缓，除ⅠS、Ⅳ型和Ⅵ型外，患者都伴有智能落后。

黏多糖病除Ⅱ型为X连锁隐性遗传外，其余均属常染色体隐性遗传病。各型黏多糖病的酶缺陷见表8-7。

【实验室检查】

1．尿黏多糖测定　通常用甲苯胺蓝法做定性试验，患者尿液呈阳性反应。醋酸纤维薄膜电泳可以区分尿中排出黏多糖的种类，进行分型参考。

表8-7　各型黏多糖病的分型、酶的缺陷和临床特征

型别综合征名	酶缺陷	尿中排出	智能低下	丑陋面容	骨骼病变	肝脾肿大	心血管病变	眼病变	耳聋
Ⅰ H型<br>Hurler	α-L-艾杜糖酶	DS，HS	＋＋＋	＋＋＋	＋＋＋	＋＋→<br>＋＋＋	＋＋→<br>＋＋＋	＋＋＋	＋＋
Ⅰ S型<br>Scheie	α-L-艾杜糖酶	DS，HS	－	＋	＋	＋/－	＋	＋＋＋	－
Ⅰ H/S型<br>Hurler Scheie	α-L-艾杜糖酶	DS，HS	＋	＋＋	＋＋	＋	＋＋	＋＋＋	＋/－
Ⅱ型<br>Hunter	艾杜糖醛酸硫酸酯酶	DS，HS	＋＋＋	＋＋	＋＋→<br>＋＋＋	＋＋→<br>＋＋＋	＋＋→<br>＋＋＋	＋/－	＋＋
ⅢA型*<br>Sanfilippo A	类肝素N-硫酸酸酯酶	HS	＋＋＋	＋	＋	＋→<br>＋＋	－	－	＋
ⅣA型**<br>Morquio A	半乳糖胺-6-硫酸酯酶	KS，CS	－	＋/－	＋＋＋	＋/－	＋	＋	＋
Ⅵ型<br>Maroteaux-Lamy	芳基硫酸酯酶	DS，HS	－	＋＋＋	＋＋＋	＋＋	＋＋	＋＋＋	＋
Ⅶ型<br>Sly型	β-葡萄糖醛酸酶	HS，DS，<br>CS	＋→<br>＋＋	＋＋	＋＋	＋＋	＋	＋	－

*ⅢB、ⅢC、ⅢD型分别为N乙酰-α-D氨基葡萄糖苷酶，乙酰辅酶A：α-氨基葡萄糖苷-N-乙酰转移酶，N-乙酰-α-D氨基葡糖苷-6-硫酸酯酶缺陷，临床上不易区别

**ⅣB型为β-半乳糖苷酶缺陷，临床上不易区别

2．骨骼X线检查　骨质较疏松，骨皮质变薄；颅骨增大，蝶鞍增大；脊柱后凸或侧凸，椎体呈楔形或扁平，胸、腹椎体前下缘呈鱼唇样前突或呈鸟嘴突；肋骨脊柱端细小，胸骨端增宽，呈飘带状；掌骨短粗，基底变尖，指骨远端窄圆，腕骨骨化成熟延迟。

3．酶学分析　根据测定白细胞、成纤维细胞中的特异性酶活性，可对黏多糖病分型。

4．DNA分析　参与黏多糖代谢的各种酶的编码基因都已定位，并且在患者中发现了相应的基因突变，有条件可进行基因诊断。

【诊断】

1．根据临床特殊面容和体征、X线片表现以及尿黏多糖阳性，可以作出临床诊断。

2．家族史中有黏多糖病患者对早期诊断有帮助。

本病应与佝偻病、先天性甲状腺功能减低症、黏脂累积病各型、甘露糖累积病、GM1神经节苷脂沉积病等鉴别，这些疾病临床表现与黏多糖病相似，但尿中黏多糖排量不增加。

【治疗】

以往对各型黏多糖病无病因治疗方法，近年基因工程生产的特异性酶的问世，使黏多糖病的酶替代治疗开始在临床上应用，黏多糖病Ⅰ型、Ⅵ型的替代治疗取得了较好的临床疗效。酶学替代治疗的主要问题是对有中枢神经系统症状者疗效差，原因是酶无法穿透血-脑屏障，另一问题是酶替代治疗价格目前极其昂贵，尚不能推广。

家庭如需生育第二胎，应进行遗传咨询，有条件可做产前基因诊断。

（顾学范）

第九章　免疫性疾病

第一节　概　　述

免疫（immunity）是机体的生理性保护机制，其本质为识别自身，排除异己；具体功能包括防御感染，清除衰老、损伤或死亡的细胞，识别和清除突变细胞。免疫功能失调可致异常免疫反应，既变态反应、自身免疫反应、免疫缺陷和发生恶性肿瘤。

【小儿免疫系统发育特点】

小儿免疫状况与成人明显不同，导致儿童疾病的特殊性。传统认为小儿时期，特别是新生儿期免疫系统不成熟。实际上，出生时免疫器官和免疫细胞均已相当成熟，免疫功能低下可能为未接触抗原，尚未建立免疫记忆之故。

1．单核／巨噬细胞　新生儿单核细胞发育已完善，但因缺乏辅助因子，其趋化、黏附、吞噬、氧化杀菌、产生G-CSF、IL-8、IL-6、IFN-γ、IL-12和抗原提呈能力均较成人差。新生儿期接触抗原或过敏原的类型和剂量不同直接影响单核／巨噬细胞，特别是DC的免疫调节功能，将影响新生儿日后的免疫状态。

2．中性粒细胞　受分娩的刺激，出生后12小时外周血中性粒细胞计数较高，72小时后渐下降，继后逐渐上升达成人水平。由于储藏库空虚，严重新生儿败血症易发生中性粒细胞减少。新生儿趋化和黏附分子Mac-1（CD11b／CD18、CD10、CD13和CD33）表达不足，以未成熟儿和剖宫产者为著。未成熟儿中性粒细胞FcRⅢ表达下降，出生后2周才达到成人水平。中性粒细胞功能暂时性低下是易发生化脓性感染的原因。

3．T淋巴细胞及细胞因子

（1）成熟T细胞占外周血淋巴细胞的80％，因此外周血淋巴细胞计数可反映T细胞数量。出生时淋巴细胞数目较少，6～7个月时超过中性粒细胞的百分率，6～7岁时两者相当；此后随年龄增长，逐渐降至老年的低水平。

（2）T细胞表型和功能：绝大多数脐血T细胞（97％）为$CD45RA^+$“初始”（“naive"）T细胞（成人外周血为50％)，而$CD45RO^+$记忆性T细胞极少。新生儿T细胞表达CD25和CD40配体较成人弱，辅助B细胞合成和转换Ig、促进吞噬细胞和CTL的能力差。

（3）T_H亚群：新生儿T_H2细胞功能较T_Hl细胞占优势，有利于避免母子免疫排斥反应。

（4）细胞因子：新生儿T细胞产生TNF和GM-CSF仅为成人的50％，IFN-ɣ、IL-10 和IL-4为10%～20％。随抗原反复刺激，各种细胞因子水平逐渐升高。如IFN-ɣ于生后175天即达到成人水平。

（5）NK和ADCC：NK的表面标记CD56于出生时几乎不表达，整个新生儿期亦很低，NK活性于生后1～5个月时达成人水平。ADCC功能仅为成人的50％，于1岁时达到成人水平。

4．B淋巴细胞及Ig

（1）B细胞表型和功能：胎儿和新生儿有产生IgM的B细胞，但无产生IgG和IgA的B细胞。分泌IgG的B细胞于2岁时，分泌IgA的B细胞于5岁时达成人水平。由于T_H细胞功能不足，B细胞不能产生荚膜多糖细菌抗体。

（2）IgG：是唯一能通过胎盘的Ig类别，其转运过程为主动性。大量IgG通过胎盘发生在孕娠后期。胎龄小于32周的胎儿或未成熟儿的血清IgG浓度低于400mg/dl，而足月新生儿血清IgG高于其母体5%～10%。新生儿自身合成的IgG比IgM慢，生后3个月血清IgG降至最低点，至10～12个月时体内IgG均为自身产生，8～10岁时达成人水平。IgG亚类随年龄增长而逐渐上升，IgG_2代表细菌多糖的抗体，其上升速度在2岁内很慢，在此年龄阶段易患荚膜细菌感染。

（3）IgM：胎儿期已能产生IgM，出生后更快，男孩于3岁时，女孩于6岁时达到成人血清水平。脐血IgM水平增高，提示宫内感染。

（4）IgA：发育最迟，至青春后期或成人期才达成人水平。分泌型IgA于新生儿期不能测出，2个月时唾液中可测到，2～4岁时达成人水平。Ig的个体发育见图9-1，不同年龄儿童血清IgG、IgA和IgM正常值见表9-1。

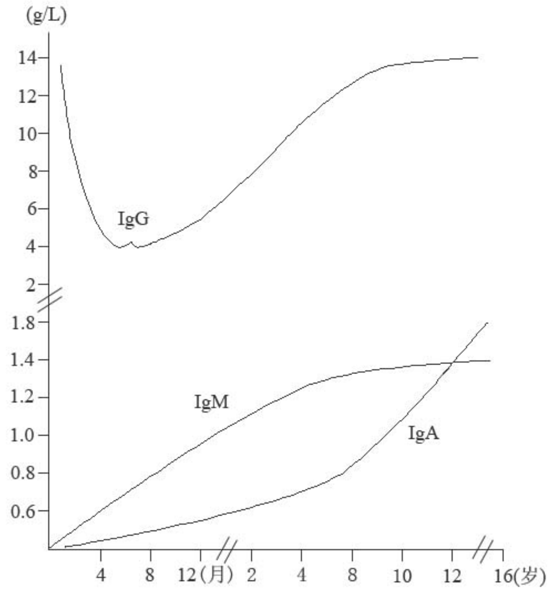

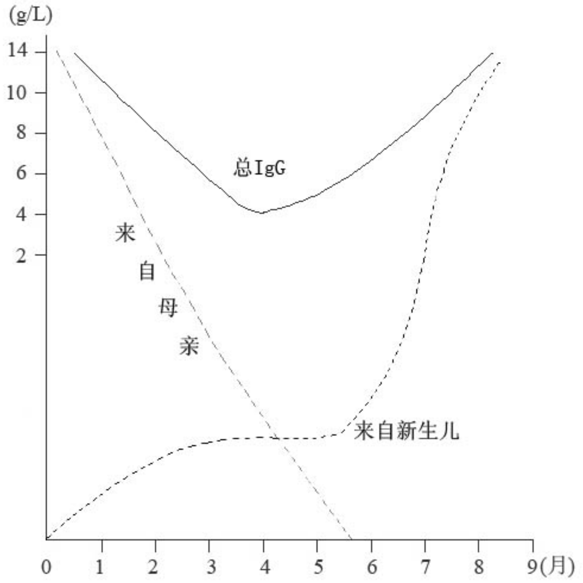

图9-1 免疫球蛋白的个体发育

注：A：IgG、IgM和IgA个体发育，由于母体IgG能通过胎盘，使出生时婴儿血清IgG水平甚高，随母体IgG消失，于生后3～5月降至最低点，婴儿自身的IgG逐渐产生，大约于8～10岁时达成人水平。IgM和lgA出生时几乎为零，IgM发育最快，于6～8岁时达成人水平；IgA于11～12岁时接近成人浓度。B：出生后9月内婴儿血清IgG动态变化。

表9-1 健康儿童血清免疫球蛋白含量（g/L）

年龄组	测定人数	IgG	IgA	IgM
新生儿	7	5.190-10.790（8.490）	0.001-0.018（0.009）	0.018-0.120（0.069）
4个月～	11	3.050-6.870（4.970）	0.110-0.450（0.280）	0.310-0.850（0.580）
7个月～	20	4.090-7.030（5.560）	0.210-0.470（0.340）	0.330-0.730（0.530）
1岁～	60	5.090-10.090（7.590）	0.310-0.670（0.490）	0.980-1.780（1.380）
3岁～	85	6.600-10.390（8.240）	0.580-1.000（0.790）	1.100-1.800（1.450）
7岁～	50	7.910-13.070（10.720）	0.850-1.710（1.280）	1.200-2.260（1.730）
12岁～	30	8.270-14.170（11.220）	0.860-1.920（1.390）	1.220-2.560（1.890）

注：表内数字为均值±2SD，括弧内为均值。本表摘自“小儿内科学”第3版 人民卫生出版社 1995，413页

5．补体和其他免疫分子

（1）补体：母体的补体不转输给胎儿，新生儿补体经典途径（CH50、C3、C4和C5）活性是其母亲的50%～60%，生后3～6个月达到成人水平。旁路途径的各种成分发育更为落后，B

因子和备解素仅分别为成人的35%～60%和35%～70%。未成熟儿补体经典和旁路途径均低于成熟儿。

（2）其他免疫分子：新生儿血浆纤连蛋白浓度仅为成人的1/3～1/2，未成熟儿则更低。未成熟儿甘露糖结合血凝素（mannose binding lectin，MBL）较成人为低，生后10～20周达到足月新生儿水平。

第二节　原发性免疫缺陷病

免疫缺陷病（immunodeficiency，ID）是指因免疫细胞（淋巴细胞、吞噬细胞和中性粒细胞）和免疫分子（可溶性因子白细胞介素、补体和免疫球蛋白和细胞膜表面分子）发生缺陷引起的机体抗感染免疫功能低下的一组临床综合征。免疫缺陷病可为遗传性，即相关基因突变或缺失所致，称为原发性免疫缺陷病（primary immunodeficiency，PID）；也可为出生后环境因素影响免疫系统，如感染、营养紊乱和某些疾病状态所致，称为继发性免疫缺陷病（secondary immunodeficiency, SID）；因其程度较轻，又称为免疫功能低下（immuno-compromise）。由人类免疫缺陷病毒（human immunodeficiency virus，HIV）感染所致者，称为获得性免疫缺陷综合征（acquired immunodeficiency syndrome，AIDS）。

【原发性免疫缺陷病的分类和发病率】

自1952年发现首例原发性免疫缺陷病X-连锁无丙种球蛋白血症（XLA）以来，每年都有新的病种发现。至2002年已发现120个病种。PID的确切发病率尚不清楚，估计总发病率为1：10000（未包括无症状的选择性IgA缺乏症和甘露聚糖结合蛋白缺陷病）。按此计算，我国每年2500万新生儿中，将会增加新的病例2500例；累计存活病例至少有3万～6万例。

各种免疫缺陷病的相对发生率为：单纯Ig或抗体缺陷占50%（其中可能包括因T细胞辅助功能缺乏而致B细胞产生抗体能力下降的病例）。细胞免疫缺陷占10%，联合免疫缺陷20%，吞噬细胞缺陷（包括吞噬细胞、中性粒细胞缺陷）占18%，补体缺陷2%（图9-2）。

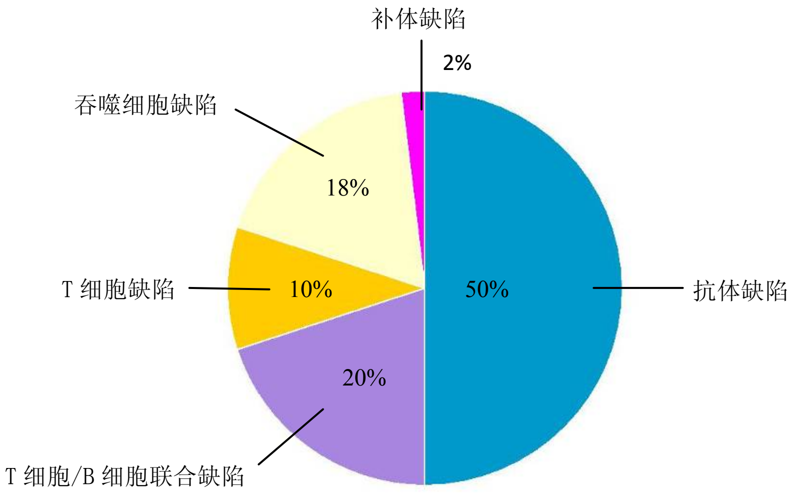

图9-4 原发性免疫缺陷病的相对发病率

细胞免疫缺陷和联合免疫缺陷时，因T辅助细胞不能提供足够的信息协助B细胞合成Ig，而发生不同程度的抗体缺陷。因此全部原发性免疫缺陷病中，约80%存在Ig和（或）抗体缺陷。

PID的病因复杂，尚无统一的分类，按国际免疫协会PID专家委员会1999年以分子学发病机理为基础的分类原则，分为①特异性免疫缺陷病（包括联合免疫缺陷病、抗体缺陷为主的免疫缺陷病、T细胞缺陷为主的免疫缺陷病、伴有其他特征的免疫缺陷病）；②免疫缺陷合并其他先

天性疾病；③补体缺陷病；④吞噬细胞缺陷病。

1．联合免疫缺陷病（combined immunodeficiency，CID）（表9-2） 该组疾病中T和B细胞均有明显缺陷，临床表现为婴儿期致死性感染，细胞免疫和抗体反应均缺陷；外周血淋巴细胞减少，尤以T细胞为著。

表9-2 联合免疫缺陷病

	血清Ig	B细胞	T细胞	病因	遗传	其他表现
1．严重型（SCID）						
(1)T^-B^+						
① X-连锁	↓	→/↑	↓↓	IL-2,4,7,9	XL	
				15R突变		
② AR	↓	→/↑	↓↓	Jak3突变	AR	
(2)T^-B^-						
① RAG1/2缺陷	↓	↓↓	↓↓	RAG1/2基因突变	AR	
② ADA缺陷	↓	↘	↘	dATP毒性	AR	
③ 网状发育不良	↓	↓↓	↓↓	干细胞缺陷	AR	全血减少
2．高IgM综合征	IgM/D↑→	IgA, G↓↓	→	CD40配体基因突变	XL	血PMNs↓
	IgG, A, E↓↓					血小板↓
						溶血性贫血
						胃肠及肝脏受累
3．嘌呤核苷磷酸化	→/↓	→	↘	dGTP毒性	AR	自身免疫性溶血，
酶(PNP)缺陷						神经系统障碍
4．MHC II 缺陷	→/↓	→	→	转录因子C II T或	AR	
			CD4↓	RFX-5突变		
5．CD3γ/CD3ε	→	→	→	CD3γ/CD3ε转录缺陷	AR	
6．ZAP-70缺陷	→	→	→	ZAP激酶基因突变	AR	
7．TAP-2缺陷	→	→	CD8↓	TAP-2基因突变	AR	

注：↓下降，→正常，↘逐渐下降，XL X-连锁遗传，AR 常染色体隐性遗传，ADA 腺苷脱氨酶，RAG 重组活化基因

（1）严重联合免疫缺陷病（severe combined immunodeficiency，SCID）：

1）T细胞缺陷，B细胞正常（T^-B^+SCID）：以X-连锁遗传最常见，其病因为IL-2、IL-4、IL-7、IL-9和IL-15的共有受体γ链（γc）基因突变所致。生后不久即发生严重细菌或病毒感染，多数病例于婴儿期死亡。

2）T和B细胞均缺如（T^-B^-SCID）：均为常染色体隐性遗传。①RAG-1/-2 缺陷：RAG-1或RAG-2基因突变，外周血T和B细胞计数均明显下降，于婴儿期发病。②腺苷脱氨酶（ADA）缺陷：ADA基因突变使ADA的毒性中间代谢产物累积，抑制T、B细胞增殖和分化。多数病例早年发生感染，极少数轻症在年长儿或成人发病。③网状发育不良（reticular dysgenesis）：为淋巴干细胞和髓前体细胞发育成熟障碍，外周血淋巴细胞、中性粒细胞和血小板均严重减少，常死于婴儿期。

（2）高IgM综合征（hyper IgM Syndrome）：70％为X-连锁遗传，病因为T细胞CD40配体基因突变，其余为常染色体隐性遗传。特点为B细胞内Ig转换障碍，不能从IgM向下游Ig类别转

化，使IgM正常或增高，而IgG、IgA 和IgE均减少或缺如。外周血IgM^+IgD^+B细胞正常或增多，IgG^+和IgA^+B细胞缺如。

（3）其他联合免疫缺陷病：如嘌呤核苷酸磷酸化酶（PNP）缺陷、MHC Ⅱ类抗原缺陷、CD3、ZAP-70和TAP2转移因子缺陷。

2．以抗体缺陷为主的免疫缺陷病（表9-3）　抗体缺陷可能是B细胞本身发育障碍，也可能是缺陷的T_H细胞不能向B细胞提供协同信号所致。主要临床表现是化脓性感染。

表9-3　以抗体缺陷为主的免疫缺陷病

	Ig	B	病因	遗传	其他表现
1．XLA	↓↓	↓↓	btk突变	XL	
2．非XL高IgM	IgM/D↑<br>其他↓↓	IgM/D→<br>其他↓↓		AR	PMN↓，血小板↓<br>溶血性贫血<br>胃肠道和肝脏受累
3．Ig重链缺失	IgG亚类↓<br>IgA2，E↓	→/↓	14q32缺失	AR	
4．κ链缺失	Igk↓<br>抗体反应→↓	→/$κ^+$↓	2p11点突变	AR	
5．选择性IgG亚类缺陷	IgG亚类↓	→/不成熟	同种型分化障碍	不明	
6．抗体缺陷（Ig正常）	→	→	不明	不明	
7．CVID	↓↓	→/↓	各异/不明	不明	
8．IgA缺陷	IgA/IgA2↓	→/$sIgA^+$↓	IgA^+B细胞分化障碍	各异	自身免疫性或过敏性疾病
9．婴儿暂时性低IgG血症	IgG/A2↓→		分化障碍；<br>辅助功能成熟延迟	不明	家族中常有PID患者
10．AR无丙种球蛋白血症	↓↓	↓↓	前B→B细胞分化障碍	AR	

注：XLA　X连锁我丙种球蛋白血症，CVID常见变异型免疫缺陷病，Igk免疫球蛋白κ链

（1）X连锁无丙种球蛋白血症（X-Linked agammaglobulinaemia，XLA）：IgM、IgG和IgA均明显下降或缺如，外周血B细胞极少或缺如。淋巴器官生发中心缺如，T细胞数量和功能正常。B细胞浆内Bruton酪氨酸激酶基因（btk）突变为其病因。感染症状轻重不一，易发生化脓性和肠道病毒感染。

（2）选择性IgG亚类缺陷：血清1～2种IgG亚类浓度低于同龄儿童2SD时可考虑IgG亚类缺陷。我国儿童IgG亚类缺陷以IgG3为主，可无症状，也可表现为反复呼吸道感染。当IgG2和IgG4联合缺陷时，易患荚膜细菌感染。多数IgG亚类缺陷患儿随年龄增长可自行消失。该病病因可能与T细胞功能障碍有关。

（3）常见变异型免疫缺陷病（commonvariable immunodeficiency，CVID）　为一组病因不明，遗传方式不定，表现为Ig缺如的综合征。临床表现为年长儿或青年人反复呼吸道感染，包括鼻窦炎、肺炎和支气管扩张，也易患胃肠道感染和肠病毒性脑膜炎。外周淋巴结肿大和脾肿大，淋巴系统、胃肠道恶性肿瘤和自身免疫性疾病的发生率很高。血清IgG和IgA低下，IgM正常或降低，诊断依赖于排除其他原发性免疫缺陷病。B细胞数量可能减少，T细胞功能异常可能是致病的关键，如$CD4^+$ / $CD8^+$细胞比率、IL-2、IL-5和IFNγ活性下降。

（4）婴儿暂时性低丙种球蛋白血症：因不能及时产生IgG，故血清IgG水平持续低下。约3岁后才逐渐回升。其机理不明。

3．T淋巴细胞缺陷为主的免疫缺陷病　是一组新近才发现的，其分子遗传学和病因学尚不清楚的疾病，包括CD4、CD7、IL-2、IL-5、T细胞信息传递障碍和钙内流机制失调。

4．伴有其他特征的免疫缺陷病　这类疾病除免疫缺陷外，尚有其他突出的临床表现（表9-4）。

表9-4　伴有其他特征的免疫缺陷病

	Ig	B	T	病因	遗传	其他表现
1．湿疹血小板减少免疫缺陷	IgM↓↓<br>抗多糖抗体↓<br>IgA/E↑	→	↘	WASP基因突变，细胞骨架功能缺陷	XL	血小板↓，小血小板，湿疹，淋巴瘤，自身免疫病
2．共济失调毛细血管扩张症	IgA/E↓<br>IgG亚类↓<br>IgM↑<br>抗体缺陷	→	↓	ATM基因突变，细胞周期异常所致染色体不稳定	AR	共济失调，毛细血管扩张，甲胎蛋白↑，淋巴系统增生，恶性肿瘤放射性敏感性增强
3．胸腺发育不全	→/↓	→	↓/→	持续基因缺陷	AD	低钙血症，颈面畸形

注：↓下降，→正常，↘逐渐下降，XL X-连锁遗传，AR 常染色体隐性遗传，AD 常染色体显性遗传

（1）湿疹血小板减少伴免疫缺陷（Wiskott-Aldrich syndrome，WAS）：发病于婴幼儿期，临床表现为湿疹、反复感染和血小板减少。血小板体积小，血小板和白细胞膜表面唾液糖蛋白、CD43和gpⅠb不稳定。扫描电镜示淋巴细胞呈“光秃”状；T细胞和血小板细胞骨架异常，肌动蛋白成束障碍。免疫功能呈进行性降低：IgM下降，多糖抗原特异性抗体反应差，外周血淋巴细胞减少和细胞免疫功能障碍。淋巴瘤和自身免疫性血管炎发生率高。位于X染色体短臂的WAS蛋白（WASP）基因突变是本病的病因。

（2）共济失调毛细血管扩张综合征（ataxia-telangiectasia，AT）：为常染色体隐性遗传疾病。进行性小脑共济失调和毛细血管扩张为其特点，后者以耳垂和球结合膜尤为突出。血清甲胎蛋白增高。早期免疫缺陷不明显，后期约70%病例免疫功能异常，出现反复呼吸道感染。血清IgG2、IgG4、IgA和IgE下降或缺如，抗体反应下降，T细胞数量和功能均下降。DNA对放射线非常敏感，且不易修复，易患恶性肿瘤。atm（AT突变）基因的蛋白质产物ATM是AT的病因。

（3）胸腺发育不全（DiGeorge anormaly，DA）：染色体22q11-ter持续基因缺失引起心脏畸形、面部异常、胸腺发育不良、颚裂和低钙血症。部分缺失者上述表现仅部分出现，称为不全性胸腺发育不全。约20%的病例出现T细胞功能异常；存活的婴儿随年龄增长，受损的T细胞功能可自然恢复。

5．补体缺陷　补体由9个活性成分（C1～C9）和5个调节蛋白（C1抑制物、C4结合蛋白、备解素、H因子和I因子）组成。C1由3个亚单位组成：Clq、Clr和C1s。D、I、H和B因子参与补体旁路系统，上述成分均可发生缺陷。除C1抑制物为常染色体显性遗传和备解素为X-连锁遗传外，其他补体成分缺陷均为常染色体隐性遗传。奈瑟菌感染、系统性红斑狼疮样综合征和其他化脓性感染是补体系统缺陷的共同临床表现，C1抑制物缺乏者伴有遗传性血管性水肿。

6．吞噬细胞数量和功能缺陷（表9-5）

表9-5　吞噬细胞数量和功能缺陷

	受累细胞	功能缺陷	遗传	表现
1. 严重先天性中性粒细胞减少	PMN	—	AR	G-CSF受体亚类突变，部分患者发生骨髓功能衰竭或急粒
2. 周期性粒细胞减少	主要为PMN	—	AR	网状细胞，血小板和其他白细胞可受累
3. 白细胞黏附分子缺陷1	PMN，Mφ	趋化、黏附、吞饮功能↓	AR	脐带脱落迟缓，慢性皮肤溃疡，牙龈炎，白细胞增多，T，NK细胞功能↓
4. 白细胞黏附分子缺陷2	主要PMN	趋化性	AR	伤口不易康复，慢性皮肤溃疡，牙龈炎，白细胞增多，短臂，智力低下，Bomboy血型
5. Chediak-Higashi综合征	主要PMN，Mφ，NK	趋化性	AR	眼-皮肤白斑，有核细胞巨大颗粒，嗜血综合征
6. 特异性颗粒缺乏	PMN	趋化性	AR	双叶核PMN
7. Schwachman综合征	PMN	趋化性	AR	贫血，血小板减少，胰腺功能不全，软骨发育不良，低Ig血症
8. 慢性肉芽肿病	PMN，Mφ	杀伤力	XL/AR	慢性化脓性感染，肉芽肿形成
9. 中性粒细胞G6PD缺乏	PMN，Mφ	杀伤力	XL	贫血
10. 髓过氧化物酶缺陷	PMN	杀伤力	AR	化脓性感染
11. IFN-γ受体缺陷	PMN，Mφ，L，NK	杀伤力	AR	分枝杆菌感染

注：↓下降，XL X-连锁遗传，AR 常染色体隐性遗传，PMN 中性粒细胞，Mφ吞噬细胞，NK 自然杀伤细胞，L 淋巴细胞，T T淋巴细胞

（1）周期性中性粒细胞减少：外周血中性粒细胞呈周期性缺如或降低，一个周期大约为3周。外周血中性粒细胞极度下降时可致感染。

（2）白细胞黏附分子缺陷（1eukocyte adhesion defects，LAD）：为常染色体隐性遗传，黏附分子CD18（包括CD11b、CD11c和CD11a）缺陷者称为LAD1；Sialyl-LewisX配体合成障碍者称为LAD2。患儿易发生皮肤感染、牙周炎、小肠或肛周瘘、新生儿脐炎、脐带延迟脱落、脓毒血症，外周血白细胞增高可达30×10^9/L。LAD2患儿尚有矮身材和智力发育迟缓。

（3）慢性肉芽肿病（chronic granulomatous，CGD）：吞噬细胞细胞色素（NADPH氧化酶成分）基因突变，致使不能产生超氧根、单态氧和H_2O_2，其杀伤功能减弱，导致慢性化脓性感染，形成肉芽肿，尤见于淋巴结、肝、肺和胃肠道。病原菌为葡萄球菌、大肠杆菌、沙雷菌、奴卡菌和曲霉菌。CGD可为X-连锁遗传：细胞色素b558中的91kD链（$qp91^{phox}$）基因突变；也可为常染色体隐性遗传：细胞色素16基因$p22^{phox}$缺陷，或NADPH氧化酶$p67^{phox}$或$p47^{phox}$缺陷。

【原发性免疫缺陷病的共同临床表现】

原发性免疫缺陷病的临床表现由于病因不同而极为复杂，但其共同的表现却非常一致，即反复感染、易患肿瘤和自身免疫性疾病。多数原发性免疫缺陷病有明显家族史。

1．反复和慢性感染　免疫缺陷最常见的表现是感染，表现为反复、严重、持久的感染。不常见和致病力低的细菌常为感染原。许多患儿需要持续使用抗菌药物预防感染。

（1）感染发生的年龄：起病年龄40％于1岁以内，1～5岁占40％，6～16岁占15％，仅5％

发病于成人。T细胞缺陷和联合免疫缺陷病发病于出生后不久，以抗体缺陷为主者，因存在母体抗体，在生后6～12个月才发生感染。成人期发病者多为普通变异型免疫缺陷病（CVID）。

（2）感染的部位：以呼吸道最常见，如复发性或慢性中耳炎、鼻窦炎、结合膜炎、支气管炎或肺炎；其次为胃肠道，如慢性肠炎。皮肤感染可为脓疖、脓肿或肉芽肿。也可为全身性感染，如败血症、脓毒血症、脑膜炎和骨关节感染。

（3）感染的病原体：一般而言，抗体缺陷易发生化脓性感染。T细胞缺陷则易发生病毒、结核杆菌和沙门菌属等细胞内病原体感染；此外，也易于真菌和原虫感染。补体成分缺陷好发生奈瑟菌属感染。中性粒细胞功能缺陷时的病原体常为金黄色葡萄球菌。发生感染的病原体的毒力可能并不很强，常呈机会感染。

（4）感染的过程：常反复发作或迁延不愈，治疗效果欠佳，尤其是抑菌剂疗效更差，必需使用杀菌剂，剂量偏大，疗程较长才有一定疗效。

一些非免疫性因素也可能造成感染易感性，在考虑原发性免疫缺陷病时，应排除这些因素。

2．肿瘤和自身免疫性疾病　未因严重感染而致死亡者，随年龄增长易发生自身免疫性疾病和肿瘤，尤其是淋巴系统肿瘤。其发生率较正常人群高数10倍乃至100倍以上。淋巴瘤最常见，以B细胞淋巴瘤多见（50％），淋巴细胞白血病（12.6％）、T细胞淋巴瘤和霍奇金病（8.6％），腺癌（9.2％）和其他肿瘤（19.2％）也可发生。

原发性免疫缺陷病伴发的自身免疫性疾病包括溶血性贫血、血小板减少性紫癜、系统性血管炎、系统性红斑狼疮、皮肌炎、免疫复合物性肾炎、Ⅰ型糖尿病、免疫性甲状腺功能低下和关节炎等。

3．其他临床表现　除反复感染外，尚可有其他的临床特征。了解这些特征有助于临床诊断。如WAS的湿疹和出血倾向，胸腺发育不全的特殊面容、先天性心脏病和难以控制的低钙惊厥等。

【原发性免疫缺陷病的诊断】

1．病史和体检

（1）过去史：脐带延迟脱落是LADl的重要线索。严重麻疹或水痘病程提示细胞免疫缺陷。了解有无引起继发性免疫缺陷病的因素、有无输血、血制品和移植物抗宿主反应（GVHR）史。详细记录预防注射，特别是灰髓炎活疫苗接种后有无麻痹发生。

（2）家族史：约1/4患儿家族能发现因感染致早年死亡的成员。应对患儿家族进行家系调查。原发性免疫缺陷病现证者可为基因突变的开始者，而无阳性家族史。了解有无过敏性疾病、自身免疫性疾病和肿瘤患者，有助于对现证者的评估。

2．体格检查　严重或反复感染可致体重下降、发育滞后现象、营养不良、轻-中度贫血和肝脾肿大。B细胞缺陷者的周围淋巴组织如扁桃体和淋巴结变小或缺如。X-连锁淋巴组织增生症则出现全身淋巴结肿大。可存在皮肤疖肿、口腔炎、牙周炎和鹅口疮等感染证据。某些特殊综合征则有相应的体征，如胸腺发育不全、WAS和AT等疾病。

3．实验室检查　反复不明原因的感染和阳性家族史提示原发性免疫缺陷病的可能性，确诊该病必须有相应的实验室检查依据，明确免疫缺陷的性质。不可能测定全部免疫功能，一些实验技术仅在研究中心才能进行。为此，在作该病的实验室检查时，可分为3个层次进行，即①初筛试验；②进一步检查；③特殊或研究性实验（表9-6）。其中初筛试验在疾病的初期筛查过程中尤其重要。

表9-6　免疫缺陷病的实验室检查

初筛试验	进一步检查	特殊/研究性实验
－B细胞缺陷		
IgG、M、A水平	B细胞计数（CD19或CD20）	进一步B细胞表型分析
同族凝集素	IgG亚类水平	淋巴结活检
嗜异凝集素	IgD和IgE水平	抗体反应（φx174，KLH）
抗链球菌溶血素O抗体	抗体反应（破伤风、白喉、风疹、流感杆菌疫苗）	体内Ig半衰期
分泌型IgA水平		体外Ig合成
	抗体反应（伤寒、肺炎球菌疫苗）	B细胞活化增殖功能
	侧位X线片咽部腺样体影	基因突变分析
－T细胞缺陷		进一步T细胞表型分析
外周淋巴细胞计数及形态	T细胞亚群计数（CD3，CD4，CD8）	细胞因子及其受体测定（如IL-2，IFN-γ，TN-α）
胸部X片胸腺影	丝裂原增殖反应或混合淋巴细胞培养，	细胞毒细胞功能（NK，CTL，ADCC）
迟发皮肤过敏试验（腮腺炎、念珠菌、破伤风类毒素、毛霉菌素、结核菌素或纯衍生物）	HLA配型染色体分析	酶测定：ADA，PNP
		皮肤，胸腺活检，胸腺素，细胞活化增殖功能，基因突变分析
－吞噬细胞		
计数	化学发光试验	黏附分子测定（CD11b/CD18，选择素配体）
WBC及形态学	WBC动力观察	移动和趋化性、变形性、黏附和凝集功能测定
NBT试验	特殊形态学	氧化代谢功能测定
IgE水平	吞噬功能测定	酶测定（MPO，G6PD，NADPH氧化酶）
	杀菌功能测定	基因突变分析
－补体缺陷		
CH50活性	调理素测定	补体旁路测定
C3水平	各补体成分测定	补体功能测定（趋化因子，免疫黏附）
C4水平	补体活化成分测定（C3a，C4a，C4d，C5a）	同种异体分析

注：ADA：腺苷脱氨酶，ADCC：抗体依赖性杀伤细胞，CTL：细胞毒性T细胞，G6PD：葡萄糖-6-磷酸脱氢酶，KLH：锁孔虫戚血兰素，MPO：髓过氧化酶，NADPH：烟酰胺腺苷2核苷磷酸，NBT：四唑氮兰，NK：自然杀伤细胞，PNP嘌呤核苷磷酸酶，φx：嗜菌体

（1）Ig测定：包括血清IgG、IgM、IgA 和IgE。一般而言，年长儿和成人总Ig>6g/L属正常，<4g/L或IgG<2g/L提示抗体缺陷。总Ig为4～6g/L或IgG2～4g/L者为可疑的抗体缺陷，应作进一步抗体反应试验或IgG亚类测定。IgE增高见于某些吞噬细胞功能异常，特别是趋化功能缺陷。

（2）抗A和抗B同族凝集素：代表IgM类抗体功能，正常情况下，生后6个月婴儿抗A、抗B滴度至少为1：8。WAS患儿伴有低IgM血症时同族凝集素滴度下降或测不出。

（3）抗链球菌溶血素O（ASO）和嗜异凝集素滴度：由于广泛接触诱发自然抗体的抗原，故一般人群嗜异凝集素滴度均大于1：10，代表IgG类抗体。我国人群由于广泛接受抗菌药物，ASO效价一般较低，若血清ASO在12岁后仍低于50单位可提示IgG抗体反应缺陷。

（4）分泌型IgA水平：分泌型IgA缺乏常伴有选择性IgA缺乏症。一般测定唾液、泪、鼻分泌物和胃液中分泌型IgA。

（5）外周血淋巴细胞绝对计数：外周血淋巴细胞80％为T细胞，因此外周血淋巴细胞绝对计数可代表T细胞数量，正常值为2×10^9/L～6×10^9/L；<2×10^9/L为可疑T细胞减少，<1.5×10^9/L则可确诊。若持续性淋巴细胞数量减少，且其体积变小者，方可定为细胞数量减少。应了解有无贫血、血小板和中性粒细胞数量、红细胞形态和大小等。中性粒细胞内巨大空泡见于Chediak-Higashi综合征。

（6）胸部X线片：婴幼儿期缺乏胸腺影者提示T细胞功能缺陷，但胸腺可因深藏于纵膈中而无法看到，应予注意。

（7）迟发皮肤过敏试验（DCH）：DCH代表T_H1细胞功能。抗原皮内注射24～72小时后观察局部反应，出现红斑及硬结为阳性结果，提示T_H1细胞功能正常。常用的抗原为腮腺炎病毒疫苗、旧结核菌类或结核菌纯蛋白衍化物（PPD）、毛霉菌素，白色念珠菌素、白喉类毒素。2岁以内正常儿童可因未曾致敏，而出现阴性反应，故应同时进行5种以上抗原皮试，只要一种抗原皮试阳性，即说明T_H1功能正常。

（8）四唑氮兰染料（NBT）试验：NBT为淡黄色可溶性染料，还原后变成蓝黑色甲臜颗粒。内毒素刺激中性粒细胞后，还原率>90％，慢性肉芽肿病患者<1％。疾病携带者则呈嵌合体。

（9）补体CH50活性、C3和C4水平：总补体CH50活性法测定的正常值为50～100U/ml。C3正常值新生儿期为570～1160mg/L，1～3月为530～1310mg/L，3个月～1岁为620～1800mg/L，1～10岁为770～1950mg/L。C4正常值新生儿期为70～230mg/L，1～3月为70～270mg/L，3～10岁为70～400mg/L。

（10）基因突变分析和产前诊断：多数PID为单基因遗传，对疾病编码基因的序列分析可发现突变位点和形式，用于确诊及进行家系调查。基因突变分析也是产前诊断最好的手段，其他用于产前诊断的方法如测定绒毛膜标本酶（ADA）活性等。

【原发性免疫缺陷病的治疗】

1．一般治疗　患儿应得到特别的儿科护理，包括预防和治疗感染，应有适当的隔离措施，注重营养，加强家庭宣教以增强父母和患儿对抗疾病的信心等。应鼓励经治疗后的患儿尽可能参加正常生活。一旦发现感染灶应及时治疗，有时需用长期抗感染药物预防性给药。下呼吸道慢性感染者，应定期作肺功能试验。

T细胞缺陷患儿，不宜输血或新鲜血制品，以防发生GVHR。若必需输血或新鲜血制品时，应先将血液进行放射照射，剂量为2000～3000rad。供血者应作CMV筛查。最好不作扁桃体和淋巴结切除术，脾切除术视为禁忌。

若患儿尚有一定抗体合成能力，可接种死疫苗，如百-白-破三联疫苗。严重免疫缺陷患者禁用活疫菌，以防发生疫苗诱导的感染。

家庭成员中已确诊免疫缺陷者，应接受遗传学咨询，妊娠期应作产前筛查，必要时终止孕妊。

2．替代治疗

（1）静脉注射丙种球蛋白（IVIG）：治疗指针仅限于低IgG血症。抗体缺陷病儿经IVIG治疗后，可使症状完全缓解，获得正常生长发育。剂量为每月1次静注IVIG 100～600mg/kg，持续终身。治疗剂量应个体化，以能控制感染为尺度。

（2）高效价免疫血清球蛋白（Special immune serum globulins，SIG）：包括水痘-带状疱疹、狂犬病、破伤风和乙肝SIG，用于预防高危患儿。

（3）血浆：除有IgG外，尚含有IgM、IgA、补体和其他免疫活性成分，剂量为20ml/kg，

必要时可加大剂量。

（4）其他替代治疗：

1）新鲜白细胞：吞噬细胞缺陷患者伴严重感染时。由于白细胞在体内存活时间短，反复使用会发生不良免疫反应，故仅用于严重感染时，而不作常规替代治疗。

2）细胞因子治疗：如胸腺素类、转移因子、IFN-γ、IL-2等。

3）酶替代治疗：腺苷脱氨酶（ADA）缺陷者，可输注红细胞（其中富含ADA）或牛ADA-多聚乙二烯糖结合物肌注，效果优于红细胞输注。

3．免疫重建　免疫重建是采用正常细胞或基因片段植入患者体内，使之发挥其功能，以持久地纠正免疫缺陷病。

（1）胸腺组织移植：包括胎儿胸腺组织移植和胸腺上皮细胞移植，其疗效不肯定，且约1/10接受胸腺移植的患者发生淋巴瘤，目前已较少使用。

（2）干细胞移植

1）胎肝移植：一些患儿接受胎肝移植后出现嵌合体，表明移植成功，此法目前已很少使用。

2）骨髓移植（BMT）：已有超过1000例原发性免疫缺陷病儿接受BMT。

3）脐血干细胞移植：脐血富含造血干细胞，可作为免疫重建的干细胞重要来源。脐血干细胞移植后GVHR较无点供体配型骨髓（matched unrelated marrow donor, MUD）移植为轻。

4）外周血干细胞移植目前尚处于实验阶段。

4．基因治疗　许多原发性免疫缺陷病的突变基因已被克隆，其突变位点已经确立。这给基因治疗打下了基础：将正常的目的基因片段整合到患者干细胞基因组内（基因转化），这些被目的基因转化的细胞经有丝分裂，使转化的基因片段能在患者体内复制而持续存在。

基因治疗原发性免疫缺陷病尝试已经历多年，取得一定成效，总的来说基因治疗尚处于探索和临床验证阶段。

第三节　继发性免疫缺陷病

【概述】

1．病因　继发性免疫缺陷病（SID）是出生后因不利的环境因素导致免疫系统暂时性功能障碍，一旦不利因素被纠正，免疫功能既可恢复正常。人的一生中，在某一特定的时期或环境下均可能发生一过性SID。SID的发病率远高于PID，且为可逆性，因此及早确诊，并找到其诱因，及时予以纠正，显得尤为重要。引起SID的常见因素见表9-7。

营养紊乱是儿童时期最常见的SID的原因．包括蛋白质-热能营养不良（PCM）、亚临床微量元素锌和铁缺乏、亚临床维生素A、维生素B族和维生素D缺乏、脂肪和糖类摄入过多等。

表9-7 导致继发性免疫缺陷病的因素

1．营养紊乱	蛋白质-热能营养不良，铁缺乏症，锌缺乏症，维生素A缺乏症，肥胖症
2．免疫抑制剂	放射线，抗体，糖皮质激素，环孢素，细胞毒性药物，抗惊厥药物
3．遗传性疾病	染色体异常，染色体不稳定综合征，酶缺陷，血红蛋白病，张力性肌萎缩症，先天性无脾症，骨骼发育不良
4．肿瘤和血液病	组织细胞增生症，类肉瘤病，淋巴系统肿瘤，白血病，霍奇金病，淋巴组织增生性疾病，再生障碍性贫血
5．新生儿	
6．感染	细菌感染，真菌感染，病毒感染，寄生虫感染
7．其他	糖尿病，蛋白质丢失性肠病，肾病综合征，尿毒症，外科手术和外伤

2．临床表现和处理　最常见的SID的临床表现为反复呼吸道感染，包括反复上呼吸道感染、支气管炎和肺炎，亦有胃肠道感染者，一般症状较轻，但反复发作。反复感染尤其是胃肠道感染可引起更严重的营养吸收障碍而加重营养不良；感染本身也可直接引起免疫功能的进一步恶化。如此，形成“营养不良-免疫功能下降-感染-加重营养不良”的恶性循环，构成了儿童时期重要的疾病谱。SID的治疗原则是治疗原发性疾病，去除诱发因素。

获得性免疫缺陷综合征（艾滋病）

获得性免疫缺陷综合征（acquired immunodeficiency syndrome，AIDS，即艾滋病）是由人类免疫缺陷病毒（human immunodeficiency virus，HIV）所引起的一种传播迅速、病死率极高的感染疾病。

【病因】

HIV属RNA反转录病毒，直径100～200nm，目前已知HIV有两个型，即HIV-Ⅰ和HIV-Ⅱ。两者均能引起AIDS，但HIV-Ⅱ致病性较HIV-Ⅰ弱。HIV-Ⅰ共有A、B、C、D、E、F、G、H、O等9种亚型，以B型最常见。本病毒为圆形或椭圆形，外层为类脂包膜，表面有锯齿样突起，内有圆柱状核心，含Mg^{2+}依赖性反转录酶。病毒包括结构蛋白P19、核心蛋白P24和P15、反转录酶蛋白P66和P51、外膜蛋白gpl20和跨膜蛋白gp41等。病毒对热敏感，56℃ 30分钟能灭活，50％浓度的酒精、0.3％的过氧化氢、0.2％次氯酸钠及10％漂白粉，经10分钟能灭活病毒，但对甲醛溶液、紫外线和γ射线不敏感。

【流行病学】

小儿患病自成人传播而来。1982年报道了首例儿童HIV感染，估计全球每天有1000例HIV感染的新生儿出生。2001年联合国艾滋病联合规划署宣布，在过去的20年，累计的HIV感染者有5600万，其中2200万人已经死于艾滋病及相关疾病，包括430万儿童。据世界卫生组织（WHO）估计，2003年全球有63万儿童感染了HIV，其中撒哈拉以南的非洲有50多万，亚洲有5万。如果这种感染速率持续下去，未来的10年全球将有500万～1000万儿童成为HIV感染者，其中90%以上在发展中国家。1995年我国首次发现经母婴途径传播的HIV感染者。感染HIV的新生儿中约有1/4会在1岁前死亡，2/3在2岁前死亡，绝大多数死亡都发生在5岁以前。

1．传染源　患者和无症状病毒携带者是本病的传染源，特别是后者。病毒主要存在于血液、精子、子宫和阴道分泌物中。其他体液如唾液、眼泪和乳汁亦含有病毒，均具有传染性。

2．儿童HIV感染的传播方式

（1）母婴传播：是儿童感染的主要途径。感染本病的孕妇可以通过胎盘、产程中及产后血性分泌物或喂奶等方式传播给婴儿。

（2）血源传播：如输血、注射、器官移植等。

（3）其他途径：如性接触传播、人工授精等，主要发生在成年人。

目前尚未证实空气、昆虫、水及食物或与AIDS患者的一般接触，如握手、公共游泳、被褥等会造成感染，亦未见到偶然接触发病的报告。

【发病机制】

HIV产生的逆向转录酶能以病毒RNA为模板，使逆向转录而产生cDNA，然后整入宿主细胞DNA链中，随着宿主细胞DNA的复制而得以繁殖。病毒感染靶细胞后1～2周内芽生脱落而离开原细胞侵入新的靶细胞，使得人体$CD4^+$ T淋巴细胞遭受破坏。近年研究发现HIV侵入$CD4^+$ T淋巴细胞时，必须借助融合素（fusin），可使$CD4^+$ T淋巴细胞融合在一起，使未受HIV侵犯的

$CD4^+$ T淋巴细胞与受害的$CD4^+$ T淋巴细胞融合而直接遭受破坏。由于$CD4^+$ T淋巴细胞被大量破坏，丧失辅助B淋巴细胞分化的能力，使体液免疫功能亦出现异常，表现为高免疫球蛋白血症、出现自身抗体和对新抗原反应性降低。抗体反应缺陷，使患儿易患严重化脓性病变；细胞免疫功能低或衰竭，引起各种机会性感染，如结核菌、卡氏肺囊虫、李斯特菌、巨细胞病毒等感染，常是致死的原因。

【病理】

HIV感染后可见淋巴结和胸腺等免疫器官病变。淋巴结呈反应性病变和肿瘤性病变两种。早期表现是淋巴组织反应性增生，随后可出现类血管免疫母细胞淋巴结病，继之淋巴结内淋巴细胞稀少，生发中心空虚，脾脏小动脉周围T细胞区和脾小结淋巴细胞稀少，无生发中心或完全丧失淋巴成分。胸腺上皮严重萎缩，缺少胸腺小体。艾滋病患儿往往发生严重的机会性感染，其病理改变因病原体不同而异。

HIV常侵犯中枢神经系统，病变包括胶质细胞增生，灶性坏死，血管周围炎性浸润，多核巨细胞形成和脱髓现象。

【临床表现】

1．AIDS的分类　患儿症状和体征的发生与发展和免疫系统受损程度及病儿机体器官功能状态相关，1994年美国疾病控制中心根据临床表现和免疫状态将HIV感染进行分类，根据临床表现分为：无临床表现（N），轻度临床表现（A），中度临床表现（B）和严重临床表现（C）。结合免疫学状况又可分为：无免疫学抑制（N1，A1，B1和C1），中度免疫学抑制（N2，A2，B2和C2）和严重免疫学抑制（N3，A3，B3和C3）。

（1）无临床表现期（N）：儿童无任何感染的症状和体征，或仅有轻微临床表现中的一个情况。

（2）轻微临床表现期（A）：儿童具有下列2个或更多的表现，但无中度和严重临床表现期的情况：淋巴结病（>0.5cm，发生在2个部位以上，双侧对称分布）；肝大；脾大；皮炎；腮腺炎；反复或持续性上呼吸道感染、鼻窦炎或中耳炎。

（3）中度临床表现期（B）：除A类的表现外，尚有以下表现：

1）贫血（Hb<80g/L），中性粒细胞减少（$<1\times10^9/L$），或血小板减少（$<100\times10^9/L$），持续30天。

2）细菌性脑膜炎、肺炎或败血症（纯培养）。

3）6个月婴儿持续2个月以上的口腔念珠菌病。

4）心肌病。

5）发生于出生后1个月内的巨细胞病毒感染，反复和慢性腹泻，肝炎。

6）单纯疱疹病毒性口腔炎，1年内发作2次以上；单纯疱疹病毒性毛细支气管炎、肺炎或食道炎发生于出生1个月内；

7）带状疱疹至少发作2次或不同皮损部位。

8）平滑肌肉瘤伴有EB病毒感染。淋巴样间质性肺炎或肺淋巴样增生综合征。

9）肾病。

10）诺卡菌属感染，持续发热1个月以上。

11）弓形虫感染发生于出生1个月内。

12）播散性水痘。

（4）严重临床表现期（C）：包括以下情况：

1）严重反复和多发性细菌感染，如脓毒血症、肺炎、脑膜炎、骨关节感染和深部脓肿，

不包括中耳炎、皮肤黏膜脓肿和导管插入引起的感染。

2）念珠菌感染累及食管、气管、支气管和肺；深部真菌感染，呈播散性（肺、肺门和颈淋巴结以外的区域）。

3）隐球菌感染伴持续腹泻1个月以上。

4）巨细胞病毒感染发生于出生1个月内，累及肝、脾和淋巴结以外的区域。

5）脑病：以下表现之一，至少持续2个月，找不到其它原因者：①发育滞后或倒退，智能倒退；②脑发育受损；头围测定证实为后天性小头畸形或CT / MRI证实为脑萎缩；③后天性系统性运动功能障碍：瘫痪、病理性反射征、共济失调和敏捷运动失调，具有其中2项者。

6）单纯疱疹病毒性黏膜溃疡持续1个月以上，或单纯疱疹病毒性支气管炎、肺炎或食管炎发生于出生1个月以后；

7）组织胞浆菌病累及肺、肺门和颈淋巴结以外的区域。

8）卡波西肉瘤：淋巴瘤（Burkitt淋巴瘤或免疫母细胞性、B细胞性、大细胞性或免疫学表型不明性）。

9）结核病，肺外播散型。

10）卡氏肺囊虫性肺炎。

11）进行性多发性白质性脑病。

12）沙门菌属（非伤寒）脓毒血症，反复发作。

13）脑弓形虫感染发生于出生1个月以后。

14）消耗综合征：①体重持续丧失基线的10%，或②大于1岁者的体重-年龄曲线下降25个百分位，或③出生1个月后体重-身高曲线下降5个百分位；同时伴有①慢性腹泻（每天至少2次经稀便持续1个月以上），或②发热1个月以上（持续或间歇性）。

【实验室检查】

1．病原学诊断

（1）病毒抗体检测：是初筛试验的主要手段，包括：

1）初筛试验：血清或尿的酶联免疫吸附试验，血快速试验。

2）确认试验：蛋白印迹试验或免疫荧光检测试验。病毒抗体检查对小于18个月龄小儿的诊断存在局限性。

（2）病毒分离：目前常采用的方法是将受检者周围血单个核细胞（PBMCs）与经植物血凝素（PHA）激活3天的正常人PBMCs共同培养（加入IL-2 10U/ml）。3周后观察细胞病变，检测反转录酶或P24抗原或病毒核酸（PCR），确定有无HIV。目前一般只用于实验研究，不作为诊断指标。

（3）抗原检测：主要是检测病毒核心抗原P24，一般在感染后1～2周内即可检出。

（4）病毒核酸检测：利用PCR或连接酶链反应（LCR）技术，可检出微量病毒核酸。

2．免疫缺陷的实验诊断

（1）血淋巴细胞亚群分析$CD4^+/CD8^+$倒置，自然杀伤细胞活性降低，皮肤迟发性变态反应减退或消失，抗淋巴细胞抗体和抗精子抗体、抗核抗体阳性。β_2微球蛋白增高，尿中新蝶呤升高。

（2）各种机会性感染病原的检诊：应尽早进行，以便及时明确感染病原，实施针对性治疗。

【诊断】

2002年中华医学会儿科学分会感染学组与免疫学组共同制定了小儿HIV感染和AIDS的诊

断标准。

1．小儿无症状HIV感染

（1）流行病史：①HIV感染母亲所生的婴儿；②输入未经HIV抗体检测的血液或血液制品史。

（2）临床表现：无任何症状、体征。

（3）实验室检查：≥18个月儿童，HIV抗体阳性，经确认试验证实者；患儿血浆中HIV RNA阳性。

（4）确诊标准：①≥18个月小儿，具有相关流行病史，实验室检查中任何一项阳性可确诊。②<18个月小儿，具备相关流行病学史，2次不同时间的血浆样本HIV RNA阳性可确诊。

2．小儿AIDS

（1）流行病史同无症状HIV感染。

（2）临床表现：不明原因的持续性全身淋巴结肿大（直径>1cm）、肝脾肿大、腮腺炎；不明原因的持续发热超过1个月；慢性反复发作性腹泻；生长发育迟缓；体重下降明显（3个月下降>基线10%）；迁延难愈的间质性肺炎和口腔霉菌感染；常发生各种机会感染等。与成人AIDS相比，小儿AIDS的特点为：①HIV感染后，潜伏期短，起病较急，进展快；②偏离正常生长曲线的生长停滞是小儿HIV感染的一种特殊表现；③易发生反复的细菌感染，特别是对多糖夹膜细菌更易感染；④慢性腮腺炎和淋巴细胞性间质性肺炎常见；⑤婴幼儿易发生脑病综合征，且发病早、进展快、预后差。

（3）实验室检查：HIV抗体阳性并经确认试验证实，患儿血浆中HIV RNA阳性；外周血$CD4^+$ T淋巴细胞总数减少，$CD4^+$ T细胞占淋巴细胞数百分比减少（表9-8）。

（4）确诊标准：患儿具有一项或多项临床表现，≥18个月患儿HIV抗体阳性（经确认实验证实）或HIV RNA阳性者；<18个月患儿2次不同时间的样本HIV RNA阳性者均可确诊。有条件者应做$CD4^+$ T细胞计数和百分比以评估免疫状况（表9-8）。

表9-8　AIDS患儿$CD4^+$细胞计数和$CD4^+$ T细胞百分率与免疫状况分类

免疫学分类	小于1岁（%）	1～5岁（%）	6～12岁（%）
无抑制	≥1500/mm^3(≥25)	≥1000/mm^3(≥25)	≥500/mm^3(≥25)
中度抑制	750～1499/mm^3(15～24)	500～999/mm^3(15～24)	200～499/mm^3(15～24)
重度抑制	<750/mm^3(<15)	<500/mm^3(<15)	<200/mm^3(<15)

【治疗】

1. 抗逆转录病毒治疗的指征　最近对HIV感染发病机制的了解和新的抗逆转录病毒药物的出现，使HIV感染的治疗已发生很大变化。所有抗逆转录病毒药物均可用于儿童病例，目前使用抗病毒药物的指征为：HIV感染的临床症状，包括临床表现A、B或C。$CD4^+$ T细胞绝对数或百分率下降，达到中度或严重免疫抑制；年龄在1岁以内的患儿，无论其临床、免疫学或病毒负荷状况；年龄大于1岁的病儿，无临床症状者，除非能明确其临床疾病进展的危险性极低或存在其它需延期治疗的因素，也主张早期治疗。应严密监测未开始治疗的病例的临床、免疫学和病毒负荷状态。

一旦发现以下情况即开始治疗：HIV RNA复制物数量极高或进行性增高；$CD4^+$ T细胞绝对数或百分率很快下降，达到中度免疫学抑制；出现临床症状。

2．抗病毒治疗

（1）核苷类反转录酶抑制剂：如齐多夫定（zidovudine，AZT）、二脱氧肌苷（DDI）、拉

米夫定（1amivudine，STC）和司他夫定（stavudius，d4T），此类药物能选择性与HIV反转录酶结合，并渗入正在延长的DNA链中，使DNA链中止，从而抑制HIV的复制和转录。

（2）非核苷类反转录酶抑制剂：如奈韦拉平（nevirapine，NVP），delavirdine（DLR）其主要作用于HIV反转录酶的某个位点，使其失去活性，从而抑制HIV复制。

（3）蛋白酶抑制剂：如沙奎那韦（saquinavir）、佳息患（indinavir，IDV）、奈非即韦（rifonavir）和利托那韦（ritonavir），其机制通过抑制蛋白酶即阻断HIV复制和成熟过程中所必须的蛋白质合成，从而抑制HIV的复制。

单用一种药物治疗效果差，目前提倡2种以上药物联合治疗，但药物最佳搭配并无定论。已确诊的AIDS患儿应转入指定医院接受治疗。

3．免疫学治疗　基因重组IL-2与抗病毒药物同时应用对改善免疫功能是有益的，IL-12是另一个有治疗价值的细胞因子，体外实验表明IL-12能增强免疫细胞杀伤被HIV感染细胞的能力。

4．支持及对症治疗　包括输血及营养支持疗法，补充维生素特别是维生素B_{12}和叶酸。

5．抗感染和抗肿瘤治疗　发生感染或肿瘤时，应给予相应的治疗。

【预防】

儿童AIDS病的预防应特别注意以下几点：①普及艾滋病知识，减少育龄期女性感染HIV；②HIV感染者避免妊娠，HIV感染或AIDS孕妇应规劝其终止妊娠或尽量进行剖宫产；③严格禁止高危人群献血，在供血员中必须除外HIV抗体阳性者；④HIV抗体阳性母亲及其新生儿应服用AZT，以降低母婴传播；⑤严格控制血液及各种血制品的质量；⑥疫苗预防：目前正在美国和泰国等地进行美国Vax Gen公司研制的AIDS VAX疫苗是用基因重组技术，针对HIV-1的糖蛋白gpl20为靶位点，目前正在进行三期临床试验。

第四节　风湿性疾病概述

自身免疫性反应是由于不同原因（包括物理、化学和生物学因子）诱导的宿主异常免疫反应，将自身组织和细胞作为靶向。若此种自身免疫反应非常强烈，引起组织严重和持久的结构和功能破坏，出现临床症状，则称为自身免疫性疾病。

风湿性疾病（rheumatic diseases）是一组病因不明的自身免疫性疾病，因主要累及不同脏器的结缔组织和胶原纤维，故曾称为结缔组织病。虽然其病因不明，但一般认为几乎所有风湿性疾病的发病机理均有其共同规律，即感染原刺激具有遗传学背景（多基因遗传）的个体，发生异常的自身免疫反应。

除经典的风湿性疾病（如风湿热、系统性红斑狼疮、皮肌炎、硬皮病、类风湿性关节炎等）外，许多以往病因不明的血管炎性综合征，如过敏性紫癜和川崎病等，现已明确为自身免疫性疾病，并纳入风湿性疾病的范畴。另一些病因不明的疾病，现也确认其发病机制为自身免疫性反应所致，如肾小球肾炎、Ⅰ型糖尿病、自身免疫性甲状腺炎、重症肌无力、格林-巴利综合征、克罗恩病和原发性血小板减少性紫癜等，未归入自身免疫性疾病中，仍分类于各系统性疾病里。

虽然风湿热发病率近年已明显下降，但仍是儿童时期最常见的风湿性疾病之一。川崎病、过敏性紫癜和幼年类风湿性关节炎是常见的儿童时期风湿性疾病。

儿童风湿性疾病的临床特点有别于成人。一些儿童风湿性疾病的全身症状较成人明显，如全身性起病型幼年类风湿性关节炎。儿童系统性红斑狼疮病程较急，预后较成人差。与多数成人风湿性疾病的慢性过程不同，川崎病和过敏性紫癜很少复发。

第五节　风　湿　热

风湿热（rheumatic fever）是常见的风湿性疾病，主要表现为心脏炎、游走性关节炎、舞蹈病、环形红斑和皮下小结，可反复发作。心脏炎是最严重的表现，急性期可危及患儿生命，反复发作可致永久性心脏瓣膜病变，影响日后劳动力。本病3岁以下少见，好发年龄为6～15岁；一年四季均可发病，以冬春多见；无性别差异。

总体来看风湿热的发病率已有明显下降，病情也明显减轻，但在发展中国家，风湿热和风湿性心脏病仍常见和严重。我国各地发病情况不一，风湿热总发病率约为22/10万，其中风湿性心脏病患病率为0.22‰，虽低于其他发展中国家，仍明显高于西方发达国家。我国农村和边远地区发病率仍然很高，且近年来风湿热发病率有回升趋势，应值得重视。

【病因和发病机理】

1．病因　风湿热是A组乙型溶血性链球菌咽峡炎后的晚期并发症。约0.3％～3％因该菌引起的咽峡炎患儿于1～4周后发生风湿热。皮肤及其他部位A组乙型溶血性链球菌感染不会引起风湿热。影响本病发生的因素有：①链球菌在咽峡部存在时间愈长，发病的机会愈大；②特殊的致风湿热A溶血性链球菌株，如M血清型（甲组1～48型）和黏液样菌株；③患儿的遗传学背景，一些人群具有明显的易感性。

2．发病机理

（1）分子模拟：A组乙型溶血性链球菌的抗原性很复杂，各种抗原分子结构与机体器官抗原存在同源性，机体的抗链球菌免疫反应可与人体组织产生免疫交叉反应，导致器官损害，是风湿热发病的主要机制。这些交叉抗原包括：

1）荚膜由透明质酸组成，与人体关节、滑膜有共同抗原。

2）细胞壁外层蛋白质中M蛋白和M相关蛋白、中层多糖中N-乙酰葡糖胺和鼠李糖均与人体心肌和心瓣膜有共同抗原。

3）细胞膜的脂蛋白与人体心肌肌膜和丘脑下核、尾状核之间有共同抗原。

（2）自身免疫反应：人体组织与链球菌的分子模拟导致的自身免疫反应包括：

1）免疫复合物病：与链球菌抗原模拟的自身抗原与抗链球菌抗体可形成循环免疫复合物沉积于人体关节滑膜、心肌、心瓣膜，激活补体成分产生炎性病变。

2）细胞免疫反应异常：①周围血淋巴细胞对链球菌抗原的增殖反应增强、患儿T淋巴细胞具有对心肌细胞的细胞毒作用；②患者外周血对链球菌抗原诱导的白细胞移动抑制试验增强，淋巴细胞母细胞化和增殖反应降低，自然杀伤细胞功能增加；③患者扁桃体单核细胞对链球菌抗原的免疫反应异常。

（3）遗传背景：有人发现HLA-B35，HLA-DR2，HLA-DR4和淋巴细胞表面标记$D8/17^{+}$等与发病有关，但还应进一步进行多中心研究才能证实该病是否为多基因遗传病和相应的相关基因。

（4）毒素：A组链球菌还可产生多种外毒素和酶类直接对人体心肌和关节有毒性作用，但并未得到确认。

【病理】

1．急性渗出期　受累部位如心脏、关节、皮肤等结缔组织变性和水肿，淋巴细胞和浆细胞浸润；心包膜纤维素性渗出，关节腔内浆液性渗出。本期持续约1个月。

2．增生期　主要发生于心肌和心内膜（包括心瓣膜），特点为形成风湿小体（Aschoff小体），小体中央为胶原纤维素样坏死物质，外周有淋巴细胞、浆细胞和巨大的多核细胞（风湿细胞）。

风湿细胞呈圆形或椭圆形，含有丰富的嗜碱性胞浆，胞核有明显的核仁。此外，风湿小体还可分布于肌肉及结缔组织，好发部位为关节处皮下组织和腱鞘，形成皮下小结，是诊断风湿热的病理依据，表示风湿活动。本期持续约3～4个月。

3．硬化期　风湿小体中央变性和坏死物质被吸收，炎症细胞减少，纤维组织增生和疤痕形成。心瓣膜边缘可有嗜伊红性疣状物，瓣膜增厚，形成疤痕。二尖瓣最常受累，其次为主动脉瓣，很少累及三尖瓣。此期约持续2～3个月。

此外，大脑皮层、小脑、基底核可见散在非特异性细胞变性和小血管透明变性。

【临床表现】

急性风湿热发生前1～5周有链球菌咽峡炎病史。如未经治疗，一次急性风湿热发作一般不超过6个月；未进行预防的患者常反复发作。风湿热多呈急性起病，亦可为隐匿性进程。临床主要表现为心脏炎、关节炎、舞蹈病、皮下小结和环形红斑，发热和关节炎是最常见的主诉。

1．一般表现　急性起病者发热在38～40℃间，无一定热型，1～2周后转为低热。隐匿起病者仅为低热或无发热。其他表现有精神不振、疲倦、胃纳不佳、面色苍白、多汗、鼻出血、关节痛和腹痛等，个别有胸膜炎和肺炎。

2．心脏炎　约40％～50％的风湿热患者累及心脏，是风湿热唯一的持续性器官损害。首次风湿热发作时，一般于起病1～2周内出现心脏炎的症状。初次发作时以心肌炎和心内膜炎最多见，同时累及心肌、心内膜和心包膜者，称为全心炎。

（1）心肌炎：轻者可无症状，重者可伴不同程度的心力衰竭；安静时心动过速，与体温升高不成比例；心脏扩大，心尖搏动弥散；心音低钝，可闻奔马律；心尖部轻度收缩期吹风样杂音，75％的初发患儿主动脉瓣区可闻舒张中期杂音。X线检查心脏扩大，心脏搏动减弱；心电图示P-R间期延长，伴有T波低平和ST段异常，或有心律失常。

（2）心内膜炎：主要侵犯二尖瓣和（或）主动脉瓣，造成关闭不全；二尖瓣关闭不全表现为心尖部2～3/6级吹风样全收缩期杂音，向腋下传导，有时可闻二尖瓣相对狭窄所致舒张中期杂音；主动脉瓣关闭不全时胸骨左缘第三肋间可闻舒张期叹气样杂音。急性期瓣膜损害多为充血水肿，恢复期可渐消失。多次复发可造成心瓣膜永久性瘢痕形成，导致风湿性心瓣膜病。超声心动图检查能更敏感地发现临床听诊无异常的隐匿性心瓣膜炎。

（3）心包炎：积液量很少时，临床上难以发现，可有心前区疼痛，有时于心底部听到心包摩擦音。积液量多时心前区搏动消失，心音遥远，有颈静脉怒张、肝大等心包填塞表现。X线检查心影向两侧扩大呈烧瓶形；心电图示低电压，早期ST段抬高，随后ST段回到等电线，并出现T波改变；超声心动图可确诊少量心包积液。临床上有心包炎表现者，提示心脏炎严重，易发生心力衰竭。

风湿性心脏炎初次发作约有5％～10％患儿发生充血性心力衰竭，再发时发生率更高。风湿性心脏瓣膜病患儿伴有心力衰竭者，提示有活动性心脏炎存在。

3．关节炎　约占急性风湿热总数的50％～60％，典型病例为游走性多关节炎，以膝、踝、肘、腕等大关节为主。表现为关节红、肿、热、痛，活动受限。每个受累关节持续数日后自行消退，愈后不留畸形，但此起彼伏，可延续3～4周。

4．舞蹈病　占风湿热患儿的3%～10％，也称Sydenham舞蹈病。表现为全身或部分肌肉的无目的不自主快速运动，如伸舌歪嘴、挤眉弄眼、耸肩缩颈、语言障碍、书写困难、细微动作不协调等，兴奋或注意力集中时加剧，入睡后即消失。患儿常伴肌无力和情绪不稳定。舞蹈病常在其他症状出现后数周至数月出现；如风湿热其他症状较轻，舞蹈病可能为首发症状。舞蹈病病程1～3个月，个别病例在1～2年内反复发作。少数病儿遗留不同程度神经精神后遗症，如

性格改变、偏头痛、细微运动不协调等。

5．皮肤症状

（1）环形红斑：较少见，环形或半环形边界明显的淡色红斑，大小不等，中心苍白，出现在躯干和四肢近端，呈一过性，或时隐时现呈迁延性，可持续数周。

（2）皮下小结：见于5％的风湿热患儿，常伴有严重心脏炎，呈坚硬无痛结节，与皮肤不粘连，直径0.1～1 cm，出现于肘、膝、腕、踝等关节伸面，或枕部、前额头皮以及胸、腰椎脊突的突起部位，约经2～4周消失。

【辅助检查】

1．链球菌感染证据　咽拭子培养可发现A组乙型溶血性链球菌，链球菌感染一周后血清抗链球菌溶血素O（ASO）滴度开始上升，两个月后逐渐下降。80％风湿热患儿ASO升高，同时测定抗脱氧核糖核酸酶B（Anti-Dnase B）、抗链球菌激酶（ASK）、抗透明质酸酶（AH）则阳性率可提高到95％。

2．风湿热活动指标　包括白细胞计数和中性粒细胞增高、血沉增快、C-反应蛋白阳性、α_2球蛋白和黏蛋白增高等，但仅能反映疾病的活动情况，对诊断本病并无特异性。

【诊断和鉴别诊断】

1．Jones诊断标准　风湿热的诊断有赖于临床表现和实验室检查的综合分析。1992年修改的Jones诊断标准包括3个部分：①主要指标；②次要指标；③链球菌感染的证据。在确定链球菌感染证据的前提下，有两项主要表现或一项主要表现伴两项次要表现即可作出诊断（表9-9）。由于近年风湿热不典型和轻症病例增多，硬性按照Jones标准，易造成诊断失误。因此，应进行综合判断，必要时需追踪观察，方能提高确诊率。

表9-9 风湿热的诊断标准

主要表现	次要表现	链球菌感染证据
心脏炎	发热	咽拭子培养阳性或快速链球菌抗原试验阳性
多关节炎	关节痛	抗链球菌抗体滴度升高
舞蹈病	血沉增高	
环形红斑	CRP阳性	
皮下小结	P-R间期延长	

注：主要表现为关节炎者，关节痛不再作为次要表现；主要表现为心脏炎者，P-R间期延长不再作为次要表现。在有链球菌感染证据的前提下，存在以下3项之一者亦应考虑风湿热：①排除其他原因的舞蹈病；②无其他原因可解释的隐匿性心脏炎；③以往已确诊为风湿热，存在一项主要表现，或有发热和关节痛，或急性期反应物质增高，提示风湿热复发。

确诊风湿热后，应尽可能明确发病类型，特别应了解是否存在心脏损害。以往有风湿热病史者，应明确是否有风湿热活动。

2．鉴别诊断　风湿热需与下列疾病进行鉴别：

（1）与风湿性关节炎的鉴别：

1）幼年类风湿性关节炎：多于3岁以下起病，常侵犯指趾小关节，关节炎无游走性特点。反复发作后遗留关节畸形，X线骨关节摄片可见关节面破坏、关节间隙变窄和邻近骨骼骨质疏松。

2）急性化脓性关节炎：为全身脓毒血症的局部表现，中毒症状重，好累及大关节，血培养阳性，常为金黄色葡萄球菌。

3）急性白血病：除发热、骨关节疼痛外，有贫血、出血倾向、肝、脾及淋巴结肿大。周围血片可见幼稚白细胞，骨髓检查可予鉴别。

4）非特异性肢痛：又名“生长痛”，多发生于下肢，夜间或入睡尤甚，喜按摩，局部无红

肿。

（2）与风湿性心脏炎的鉴别诊断

1）感染性心内膜炎：先天性心脏病或风湿性心脏病合并感染性心内膜炎时，易与风湿性心脏病伴风湿活动相混淆，贫血、脾大、皮肤瘀斑或其他栓塞症状有助诊断，血培养可获阳性结果，超声心动图可看到心瓣膜或心内膜有赘生物。

2）病毒性心肌炎：近年单纯风湿性心肌炎病例日渐增多，与病毒性心肌炎难以区别。一般而言，病毒性心肌炎杂音不明显，较少发生心内膜炎，较多出现过早搏动等心率失常，实验室检查可发现病毒感染证据。

【治疗】

1．休息　卧床休息的期限取决于心脏受累程度和心功能状态。急性期无心脏炎患儿卧床休息2周，随后逐渐恢复活动，于2周后达正常活动水平；心脏炎无心力衰竭患儿卧床休息4周，随后于4周内逐渐恢复活动；心脏炎伴充血性心力衰竭患儿则需卧床休息至少8周，在以后2～3个月内逐渐增加活动量。

2．清除链球菌感染　应用青霉素80万单位肌注，每日2次，持续2周，以彻底清除链球菌感染。青霉素过敏者可改用其他有效抗生素如红霉素等。

3．抗风湿热治疗　心脏炎时宜早期使用糖皮质激素，泼尼松每日2 mg/kg，最大量≤60 mg/d，分次口服，2～4周后减量，总疗程8～12周。无心脏炎的患儿可用阿司匹林，每日100 mg/kg，最大量≤3 g/d，分次服用，2周后逐渐减量，疗程4～8周。

4．其他治疗　有充血性心力衰竭时应视为心脏炎复发，及时给予大剂量静脉注射糖皮质激素，如氢化可的松或甲基泼尼松龙每日1次10～30 mg/kg，共1～3次。多数情况在用药后2～3天即可控制心力衰竭，应慎用或不用洋地黄制剂，以免发生洋地黄中毒。应予以低盐饮食，必要时氧气吸入、给予利尿剂和血管扩张剂。舞蹈病时可用苯巴比妥、地西泮等镇静剂。关节肿痛时应予制动。

【预防和预后】

风湿热预后主要取决于心脏炎的严重程度、首次发作是否得到正确抗风湿热治疗以及是否正规抗链球菌治疗。心脏炎者易于复发，预后较差，尤以严重心脏炎伴充血性心力衰竭的患儿为甚。

每3～4周肌内注射苄星青霉素（长效青霉素，Benzathine penicilline）120万单位，预防注射期限至少5年，最好持续至25岁；有风湿性心脏病者，宜作终身药物预防。对青霉素过敏者可改用红霉素类药物口服，每月口服6～7天，持续时间同前。

风湿热或风湿性心脏病患儿，当拔牙或行其他手术时，术前、术后应用抗生素以预防感染性心内膜炎。

第六节　幼年特发性关节炎

幼年特发性关节炎（juvenile idiopathic arthritis, JIA）是儿童时期常见的风湿性疾病，以慢性关节滑膜炎为主要特征，伴全身多脏器功能损害。是小儿时期残疾或失明的重要原因。该病命名繁多，如幼年类风湿性关节炎（juvenile rheumatoid arthritis, JRA）、Still’s病、幼年慢性关节炎（juvenile chronic arthritis, JCA）、幼年型关节炎（juvenile arthritis, JA）等。为了便于国际间协作组对这类疾病的遗传学、流行病学、转归和治疗方案实施等方面进行研究，近10多年国际风湿病联盟儿科委员会专家组经过多次讨论，将“儿童时期（16岁以下）不明原因关节肿胀，持续6周以上者”，命名为幼年特发性关节炎（JIA）。各地分类的比较见表9-10。

表9-10　幼年特发性关节炎分类与美国和欧洲分类的比较

美国风湿病学会（ACR）	欧洲风湿病联盟（EULAR）	国际风湿病联盟（ILAR）
幼年类风湿关节炎（JRA）	幼年慢性关节炎（JCA）	幼年特发性关节炎（JIA）
	全身型	全身型
全身型	多关节炎型JCA	多关节炎型（RF阴性）
多关节炎型	幼年类风湿关节炎	多关节炎型（RF阳性）
少关节炎型	少关节炎型	少关节炎型
		持续型
		扩展型
	银屑病性关节炎（JpsA）	银屑病性关节炎
	幼年强直性脊柱炎（JAS）	与附着点炎症相关的关节炎
		其他关节炎

【病因和发病机理】

病因至今尚不清楚，可能与多种因素有关。

1．感染因素　虽有许多关于细菌（链球菌、耶尔森菌、志贺菌、空肠弯曲菌和沙门菌属等）、病毒（细小病毒B19、风疹和EB病毒等）、支原体和衣原体感染与本病有关的报道，但都不能证实是诱导本病的直接原因。

2．遗传因素　很多资料证实JIA具有遗传学背景，研究最多的是人类白细胞抗原（HLA），具有HLA-DR4（特别是DRl*0401）、DR8（特别是DRBl*0801）和DR5（特别是DRl*1104）位点者是JIA的易发病人群。其他与JIA发病有关的HLA位点为HLA-DR6，HLA-A2 等。也发现另外一些HLA位点与抗JIA发病有关。

3．免疫学因素　有许多证明证实JIA为自身免疫性疾病：①部分患儿血清和关节滑膜液中存在类风湿因子（RF，抗变性IgG抗体）和抗核抗体（ANA）等自身抗体；②关节滑膜液中有IgG包涵体和类风湿因子的吞噬细胞（类风湿性关节炎细胞，RAC）；③多数患儿的血清IgG、IgM和IgA上升；④外周血CD^{4+}T细胞克隆扩增；⑤血清炎症性细胞因子明显增高。

综上所述，JIA的发病机制可能为：各种感染性微生物的特殊成分作为外来抗原，作用于具有遗传学背景的人群，激活免疫细胞，通过直接损伤或分泌细胞因子、自身抗体触发异常免疫反应，引起自身组织的损害和变性。尤其是某些细菌、病毒的特殊成分（如HSP）可作为超抗原，直接与具有特殊可变区β链（Vβ）结构的T细胞受体（TCR）结合而激活T细胞，激发免疫损伤。自身组织变性成分（内源性抗原）如变性IgG或变性的胶原蛋白，也可作为抗原引发针对自身组织成分的免疫反应，进一步加重免疫损伤。

【JIA的分类及临床表现】

1．幼年特发性关节炎分类

2．各型幼年特发性关节炎的定义及临床特点

（1）全身型关节炎（systemic JIA）：任何年龄皆可发病，但大部分起病于5岁以前：

1）定义：每日发热至少2周以上，伴有关节炎，同时伴随以下2）～5）项中的一项或更多症状。

2）短暂的、非固定的红斑样皮疹。

3）淋巴结肿大。

4）肝脾大。

5）浆膜炎：如胸膜炎及心包炎。

6）应排除下列情况：①银屑病患者；②8岁以上HLA-B27阳性的男性关节炎患儿；③家族史中一级亲属有HLA-B27相关的疾病（强直性脊柱炎、与附着点炎症相关的关节炎、急性前色素膜炎或骶髂关节炎）；④两次类风湿因子阳性，两次间隔为3个月。

本型的发热呈弛张高热，每天体温波动在36～40℃之间。其皮疹特点为随体温升降而出现或消退。关节症状主要是关节痛或关节炎，发生率在80%以上，为多关节炎或少关节炎，常在发热时加剧，热退后减轻或缓解。关节症状既可首发，又可在急性发病数月或数年后才出现。部分有神经系统症状。

（2）多关节型，类风湿因子阴性（polyarticular JIA, RF negative）：

1）应排除下列情况：①银屑病患者；②8岁以上HLA-B27阳性的男性关节炎患儿；③家族史中一级亲属有HLA-B27相关的疾病（强直性脊柱炎、与附着点炎症相关的关节炎、急性前色素膜炎或骶髂关节炎）；④两次类风湿因子阳性，两次间隔为3个月；⑤全身型JIA。

（3）多关节型，类风湿因子阳性（polyarticular JIA,RF positive）：

1）定义：发热最初6个月有5个关节受累，类风湿因子阳性。

2）应排除下列情况：①银屑病患者；②8岁以上HLA-B27阳性的男性关节炎患儿；③家族史中一级亲属有HLA-B27相关的疾病（强直性脊柱炎、与附着点炎症相关的关节炎、急性前色素膜炎或骶髂关节炎）；④全身型JIA。

本型发病亦以女孩多见。多于儿童后期起病，本型临床表现基本上与成人RA相同。关节症状较类风湿因子阴性组为重，后期可侵犯髋关节，最终约半数以上发生关节强直变形而影响关节功能。除关节炎表现外，可出现类风湿结节。

（4）少关节型（oligoarticular JIA）

1）定义：发病最初6个月有1～4个关节受累。疾病又分为两个亚型①持续型少关节型JIA：整个疾病过程中关节受累均在4个以下；②扩展型少关节型JIA：在疾病发病后6个月发展成关节受累≥5个，约20%患儿有此情况。

2）应排除下列情况：①银屑病患者；②8岁以上HLA-B27阳性的男性关节炎患儿；③家族史中一级亲属有HLA-B27相关的疾病（强直性脊柱炎、与附着点炎症相关的关节炎、急性前色素膜炎或骶髂关节炎）；④两次类风湿因子阳性，两次间隔为3个月；⑤全身型JIA。

本型女孩多见，起病多在5岁以前。多为大关节受累，膝、踝、肘或腕等大关节为好发部位，常为非对称性。虽然关节炎反复发作，但很少致残。约20%～30%患儿发生慢性虹膜睫状体炎而造成视力障碍、甚至失明。

（5）与附着点炎症相关的关节炎（enthesitis related JIA, ERA）：

1）定义：关节炎合并附着点炎症或关节炎或附着点炎症，伴有以下情况中至少2项：①骶髂关节压痛或炎症性腰骶部及脊柱疼痛，而不局限在颈椎；②HLA-B27阳性；③8岁以上的男性患儿；④家族史中一级亲属有HLA-B27相关的疾病（强直性脊柱炎、与附着点炎症相关的关节炎、急性前色素膜炎或骶髂关节炎）。

2）应排除下列情况：①银屑病患者；②两次类风湿因子阳性，两次间隔为3个月；③全身型JIA。

本型以男孩多见，多于8岁以上起病。四肢关节炎常为首发症状，但以下肢大关节如髋、膝、踝关节受累为多见，表现为肿、痛和活动受限。

骶髂关节病变可于病初发生，但多数于起病数月至数年后才出现。典型症状为下腰部疼痛，初为间歇性，数月或数年后转为持续性，疼痛可放射至臀部，甚至大腿。直接按压骶髂关节时

有压痛。随着病情发展，腰椎受累时可致腰部活动受限，严重者病变可波及胸椎和颈椎，使整个脊柱呈强直状态。在儿童常只有骶髂关节炎的X线改变，而无症状和体征。

患儿还可有反复发作的急性虹膜睫状体炎和足跟疼痛，这是由于跟腱及足底筋膜与跟骨附着处炎症所致。本型HLA-B27阳性者占90%，多有家族史。

（6）银屑病性关节炎（psoriatic JIA）：

1）定义：1个或更多的关节炎合并银屑病，或关节炎合并以下任何2项：①指（趾）炎；②指甲凹陷或指甲脱离；③家族史中一级亲属有银屑病。

2）应排除下列情况：①8岁以上HLA-B27阳性的男性关节炎患儿；②家族史中一级亲属有HLA-B27相关的疾病（强直性脊柱炎、与附着点炎症相关的关节炎、急性前色素膜炎或骶髂关节炎）；③两次类风湿因子阳性，两次间隔为3个月；④全身型JIA。

本型儿童时期罕见。发病以女性占多数。女与男之比为2.5：1。表现为一个或几个关节受累，常为不对称性。大约有半数以上患儿有远端指间关节受累及指甲凹陷。关节炎可发生于银屑病发病之前或数月、数年后。40%患者有银屑病家族史。发生骶髂关节炎或强直性脊柱炎者，HLA-B27阳性。

（7）未定类的幼年特发性关节炎（undefined JIA）：不符合上述任何一项或符合上述两项以上类别的关节炎。

【诊断与鉴别诊断】

1．辅助诊断　实验室检查的任何项目都不具备确诊价值，但可帮助了解疾病程度和除外其他疾病。

（1）炎症反应的证据：血沉明显加快，但少关节型患者的血沉结果多数正常。在多关节型和全身型患者中急性期反应物（C反应蛋白、IL-1和IL-6等）增高，有助于随访时了解病程。

（2）自身抗体：

1）类风湿因子（RF）：RF阳性提示严重关节病变及有类风湿结节。RF阴性中约75%患儿能检出隐匿型RF，对JIA患者的诊断有一定帮助。

2）抗核抗体（ANA）：40%的患儿出现低中滴度的ANA。

（3）其他检查：

1）关节液分析和滑膜组织学检查：可鉴别化脓性关节炎、结核性关节炎、类肉瘤病、滑膜肿瘤等。

2）血常规：常见轻-中度贫血，外周血白细胞总数和中性粒细胞增高，可伴类白血病反应。

3）X线检查：早期（病程1年左右）X线仅显示软组织肿胀，关节周围骨质疏松，关节附近呈现骨膜炎。晚期才能见到关节面骨破坏，以手腕关节多见。

4）其他影像学检查：骨放射性核素扫描、超声波和MRI均有助于发现骨关节损害。

2．诊断依据　JIA的诊断主要依靠临床表现，采用排除诊断法。

（1）定义：16岁以下儿童不明原因关节肿胀，持续6周以上者，诊断为幼年特发性关节炎。必须除外下列鉴别诊断中的疾病。

（2）分类：参考上述各型幼年特发性关节炎的分类定义。

3．鉴别诊断

（1）以高热、皮疹等全身症状为主者应与以下疾病相鉴别：

1）全身感染：败血症、结核、病毒感染。

2）恶性病：白血病、淋巴瘤、恶性组织细胞病、其他恶性肿瘤。

（2）以外周关节受累为主者：应与风湿热、化脓性关节炎、关节结核、创伤性关节炎鉴

别。

（3）与其他风湿性疾病合并关节炎相鉴别：SLE、MCTD、血管炎综合征（过敏性紫癜、川崎病）。

（4）JIA需与以下疾病相鉴别：脊髓肿瘤、腰椎感染、椎间盘病变、先天性髋关节病变以及溃疡性结肠炎、局限性小肠炎、银屑病和Reiter’s syndrome（瑞特综合征）合并脊柱炎。

【治疗】

JIA的治疗原则是：控制病变的活动度，减轻或消除关节疼痛和肿胀；预防感染和关节炎症的加重；预防关节功能不全和残废；恢复关节功能及生活与劳动能力。

1．一般治疗　除急性发热外，不主张过多地卧床休息。宜鼓励患儿参加适当的运动，尽可能像正常儿童一样生活。定期进行裂隙灯检查以发现虹膜睫状体炎。心理治疗也重要，应克服患儿因慢性疾病或残疾造成的自卑心理，鼓励参加正常活动和上学；取得家长配合，增强他们战胜疾病的信心，使患儿的身心健康成长。

2．药物治疗

（1）非甾体抗炎药（non-steroidal anti-inflammatory drugs, NSAIDs）：以肠溶阿斯匹林（ASP）为代表，推荐剂量为每天60～90 mg/kg，分4～6次口服。有效血浓度为20～30 mg/dl，约1～4周内见效，病情缓解后逐渐减量，最后以最低临床有效剂量维持，可持续数月至数年。不良反应包括胃肠道反应，肝、肾功能损害，过敏反应等。近年由于发现ASP的不良反应较多，其他NSAID的使用逐渐增多，如萘普生（每天10～15 mg/kg，分2次）、布洛芬（每日50 mg/kg，分2～3次）、双氯芬酸钠或尼美舒利（nimesulide）等。

（2）缓解病情抗风湿药（disease modifying anti-rheumatic drugs, DMARDs）：即二线药物，因为应用这类药物至出现临床疗效之间所需时间较长，故又称慢作用抗风湿药（slow acting anti-rheumatic drugs, SAARDs）。近年来认为，在患者尚未发生骨侵蚀或关节破坏时及早使用本组药物，可以控制病情加重。

1）羟氯喹（hydroxychloroquine）：剂量为每日5～6 mg/(kg.d)，不超过0.25 g/d，分1～2次服用。疗程3个月至1年。不良反应可有视网膜炎、白细胞减少、肌无力和肝功能损害。

2）柳氮磺胺吡啶（sulfasalazine）：剂量为50 mg/(kg.d)，服药1～2个月即可起效。副作用包括恶心、呕吐、皮疹、哮喘、贫血、溶血、骨髓抑制、中毒性肝炎和不育症。

3）其他：包括青霉胺（d-penicillamine）、金制剂（gold）如硫代苹果酸金钠（myochrysine）。

（3）肾上腺皮质激素：虽可减轻JIA关节炎症状，但不能阻止关节破坏，长期使用不良反应太大，而一旦停药将会严重复发。因此，糖皮质激素不作为首选或单独使用的药物，应严格掌握指征。临床应用适应证：

1）多关节型：对NSAIDs和DMARDs未能控制的严重病儿，加用小剂量泼尼松隔日顿服，可使原来不能起床或被迫坐轮椅者症状减轻，过着基本正常的生活。

2）全身型：非甾体类抗炎药物或其他治疗无效的全身型可加服泼尼松0.5～1 mg/(kg.d)（≤40 mg/d），一次顿服或分次服用。一旦体温得到控制时即逐渐减量至停药。

3）少关节型：不主张用肾上腺皮质激素全身治疗，可酌情在单个病变关节腔内抽液后，注入醋酸氢化可的松混悬剂局部治疗。

4）虹膜睫状体炎：轻者可用扩瞳剂及肾上腺皮质激素类眼药水点眼。对严重影响视力患者，除局部注射肾上腺皮质激素外需加用泼尼松口服。虹膜睫状体炎对泼尼松很敏感，无需大剂量。

对银屑病性关节炎不主张用肾上腺皮质激素。

（4）免疫抑制剂：

1）甲氨蝶呤（methotraxate，MTX）：剂量为10 mg/m^2，每周1次顿服。服药3～12周即可起效。MTX不良反应较轻，有不同程度胃肠道反应、一过性转氨酶升高、胃炎和口腔溃疡、贫血和粒细胞减少。长期使用可能发生B细胞淋巴瘤。对多关节型安全有效。

2）其他免疫抑制剂：可选择使用环孢素A、环磷酰胺（CTX）、来氟米特和硫唑嘌呤、雷公藤多苷。但其治疗JIA的有效性与安全性尚需慎重评价。

（5）其他：大剂量IVIG治疗难治性全身发病型JIA的疗效尚未能得到确认。抗肿瘤坏死因子（TNF）-α单克隆抗体对多关节型JIA有一定疗效。

（6）中药制剂等。

3．理疗（physical therapy） 对保持关节活动、肌力强度是极为重要的。尽早开始保护关节活动及维持肌肉强度的锻炼，有利于防止发生或纠正关节残废。

【预后】

JIA总体预后较好，给予适当处理后75%的患者不会严重致残。并发症主要是关节功能丧失和虹膜睫状体炎所致的视力障碍。但就个例而言预后难测，有些人在历经数年缓解后在成人期偶尔也会出现复发。有研究认为IgM型RF阳性滴度越高预后越差。另外，目前有报道JIA可能发生严重并发症，即巨噬细胞活化综合征（macrophage activation syndrom, MAS），这种疾病常急性发作，多见于男性患者，临床表现为快速进展的肝功能衰竭、脑病、全血细胞减低、紫癜、瘀斑、黏膜出血，甚至可死亡。主要认为是由于T淋巴细胞和巨噬细胞的活化和不可遏制的增生，导致细胞因子过度产生所致。

第七节　过敏性紫癜

过敏性紫癜（anaphylactoid purpura）又称亨-舒综合征（henoch-schonlein syndrome，henoch-schonlein purpura, HSP），是以小血管炎为主要病变的系统性血管炎。临床特点为血小板不减少性紫癜，常伴关节肿痛、腹痛、便血、血尿和蛋白尿。多发生于2～8岁的儿童，男孩多于女孩；一年四季均有发病，以春秋二季居多。

【病因】

本病的病因尚未明确，虽然食物过敏（蛋类、乳类、豆类等），药物（阿司匹林、抗生素等）、微生物（细菌、病毒、寄生虫等）、疫苗接种、麻醉、恶性病变等与过敏性紫癜发病有关，但均无确切证据。

近年关于链球菌感染导致过敏性紫癜的报道较多。约50％过敏性紫癜患儿有链球菌性呼吸道感染史，但随后研究发现链球菌性呼吸道感染史者在过敏性紫癜患儿和健康儿童间并无差别。另有报道30％过敏性紫癜肾炎患儿肾小球系膜有A组溶血性链球菌抗原（肾炎相关性血浆素受体，NAP1r）沉积；而非过敏性紫癜肾炎的NAP 1 r沉积率仅为3％。表明A组溶血性链球菌感染是诱发过敏性紫癜的重要原因。

【发病机理】

B淋巴细胞多克隆活化为其特征，患儿T淋巴细胞和单核细胞CD40配体（CD40L）过度表达，促进B淋巴细胞分泌大量IgA和IgE。30％～50％患儿血清IgA浓度升高，急性期外周血IgA^+B淋巴细胞数、IgA类免疫复合物或冷球蛋白均增高。IgA、补体C3和纤维蛋白沉积于肾小球系膜、皮肤和肠道毛细血管，提示本病为IgA免疫复合物疾病。血清肿瘤坏死因子-α和IL-6等前炎症因

子升高。

本病家族中可同时发病，同胞中可同时或先后发病，有一定遗传倾向，部分患儿为HLA-DW35遗传标志或C2补体成分缺乏者。

综上所述，过敏性紫癜的发病机理可能为：各种刺激因子，包括感染原和过敏原作用于具有遗传背景的个体，激发B细胞克隆扩增，导致IgA介导的系统性血管炎。

【病理】

过敏性紫癜的病理变化为广泛的白细胞碎裂性小血管炎，以毛细血管炎为主，亦可波及小静脉和小动脉。血管壁可见胶原纤维肿胀和坏死，中性粒细胞浸润，周围散在核碎片。间质水肿，有浆液性渗出，同时可见渗出的红细胞。内皮细胞肿胀，可有血栓形成。病变累及皮肤、肾脏、关节及胃肠道，少数涉及心、肺等脏器。在皮肤和肾脏荧光显微镜下可见IgA为主的免疫复合物沉积。过敏性紫癜肾炎的病理改变：轻者可为轻度系膜增生、微小病变、局灶性肾炎，重者为弥漫增殖性肾炎伴新月体形成。肾小球IgA性免疫复合物沉积也见于IgA肾病，但过敏性紫癜和IgA肾病的病程全然不同，不似同一疾病。

【临床表现】

多为急性起病，各种症状可以不同组合，出现先后不一，首发症状以皮肤紫癜为主，少数病例以腹痛、关节炎或肾脏症状首先出现。起病前1～3周常有上呼吸道感染史，可伴有低热、食欲不振、乏力等全身症状。

1．皮肤紫癜　反复出现皮肤紫癜为本病特征，多见于四肢及臀部，对称分布，伸侧较多，分批出现，面部及躯干较少。初起呈紫红色斑丘疹，高出皮面，压之不褪色，数日后转为暗紫色，最终呈棕褐色而消退。少数重症患儿紫癜可融合成大疱伴出血性坏死。部分病例可伴有荨麻疹和血管神经性水肿。皮肤紫癜一般在4～6周后消退，部分患儿间隔数周、数月后又复发。

2．胃肠道症状　约见于2/3病例。由血管炎引起的肠壁水肿、出血、坏死或穿孔是产生肠道症状及严重并发症的主要原因。一般以阵发性剧烈腹痛为主，常位于脐周或下腹部，疼痛，可伴呕吐，但呕血少见。部分患儿可有黑便或血便，偶见并发肠套叠、肠梗阻或肠穿孔者。

3．关节症状　约1/3病例可出现膝、踝、肘、腕等大关节肿痛，活动受限。关节腔有浆液性积液，但一般无出血，可在数日内消失，不留后遗症。

4．肾脏症状　30％～60％病例有肾脏受损的临床表现。肾脏症状多发生于起病1个月内，亦可在病程更晚期，于其他症状消失后发生，少数则以肾炎作为首发症状。症状轻重不一，与肾外症状的严重度无一致性关系。多数患儿出现血尿、蛋白尿和管型尿，伴血压增高及浮肿，称为紫癜性肾炎；少数呈肾病综合征表现。虽然有些患儿的血尿，蛋血尿持续数月甚至数年，但大多数都能完全恢复，少数发展为慢性肾炎，死于慢性肾功能衰竭。

5．其他表现　偶可发生颅内出血，导致惊厥、瘫痪、昏迷、失语。出血倾向包括鼻出血、牙龈出血、咯血、睾丸出血等。偶而累及循环系统发生心肌炎和心包炎，累及呼吸系统发生喉头水肿，哮喘、肺出血等。

【辅助检查】

尚无特异性诊断试验，以下试验有助于了解病程和并发症。

1．周围血象　白细胞正常或增加，中性粒细胞和嗜酸性粒细胞可增高；除非严重出血，一般无贫血。血小板计数正常甚至升高，出血和凝血时间正常，血块退缩试验正常，部分患儿毛细血管脆性试验阳性。

2．尿常规　可有红细胞、蛋白、管型，重症有肉眼血尿。

3．大便隐血试验阳性。

4．血沉轻度增快；血清IgA升高，IgG和IgM正常，亦可轻度升高；C3、C4正常或升高；抗核抗体及类风湿因子阴性；重症血浆黏度增高。

5.腹部超声波检查有利于早期诊断肠套叠，头颅MRI对有中枢神经系统症状患儿可予确诊，肾脏症状较重和迁延者可行肾穿刺以了解病情给予相应治疗。

【诊断和鉴别诊断】

典型病例诊断不难，若临床表现不典型，皮肤紫癜未出现时，容易误诊为其他疾病，需与特发性血小板减少性紫癜、风湿性关节炎、败血症、其他肾脏疾病和外科急腹症等鉴别。

【治疗】

1．一般治疗　卧床休息，积极寻找和去除致病因素，如控制感染，补充维生素。有荨麻疹或血管神经性水肿时，应用抗组胺药物和钙剂。腹痛时应用解痉剂，消化道出血时应禁食，可静脉滴注西咪替丁每日20～40mg/kg，必要时输血。

2．糖皮质激素和免疫抑制剂　急性期对腹痛和关节痛可予缓解，但不能预防肾脏损害的发生，亦不能影响预后。泼尼松每日1～2mg/kg，分次口服，或用地塞米松、甲基泼尼松龙每日（5～10mg/kg）静脉滴注，症状缓解后即可停用。重症过敏性紫癜肾炎可加用免疫抑制剂如环磷酰胺、硫唑嘌呤或雷公藤多苷片。

3．抗凝治疗

（1）阻止血小板聚集和血栓形成的药物：阿司匹林每日3～5 mg/kg，或每日25～50 mg，每天一次服用；双嘧达莫每日3～5 mg / kg，分次服用。

（2）肝素：每次0.5～1 mg/kg，首日3次，次日2次，以后每日1次，持续7天。

（3）尿激酶：每日1000～3000 u/kg静脉滴注。

（4）其他　钙通道拮抗剂如硝苯地平每日0.5～1.0 mg/kg，分次服用，非甾体抗炎药如吲哚美辛每日2～3 mg/kg，分次服用，均有利于血管炎的恢复。中成药如贞芪扶正冲剂、复方丹参片、银杏叶片，口服3～6个月，可补肾益气和活血化淤。

【预后】

本病预后一般良好，除少数重症患儿可死于肠出血、肠套叠、肠坏死或神经系统损害外，大多痊愈。病程一般约1～2周至1～2个月，少数可长达数月或一年以上。肾脏病变常较迁延，可持续数月或数年，少数病例（1％）发展为持续性肾脏疾病，极个别病例（0.1％）发生肾功能不全。

第八节　川　崎　病

川崎病（Kawasaki disease，KD）于1967年由日本川崎富作首先报告，曾称为黏膜皮肤淋巴结综合征（mucocutaneous lymphnode syndrome，MCLS），约15％～20％未经治疗的患儿发生冠状动脉损害。自1970年以来，世界各国均有发生，以亚裔人发病率为高。本病呈散发或小流行，四季均可发病。发病年龄以婴幼儿多见，80％在5岁以下。男：女为1.5：1。

【病因和发病机理】

1．病因　病因不明，流行病学资料提示立克次体、丙酸杆菌、葡萄球菌、链球菌、反转录病毒、支原体感染为其病因，但均未能证实。

2．发病机理　本病的发病机理尚不清楚。推测感染原的特殊成分，如超抗原（热休克蛋白65，HSP_{65}等）可不经过单核／巨噬细胞，直接通过与T细胞抗原受体（TCR）Vβ 片段结合，激活$CD30^{+}$T细胞和CD40配体表达。在T细胞的诱导下，B淋巴细胞多克隆活化和凋亡减少，产生大量免疫球蛋白（IgG、IgM、IgA、IgE）和细胞因子（IL-1，IL-2，IL-6，TNF-α）。抗中性粒细胞胞浆抗体（ANCA）、抗内皮细胞抗体和细胞因子损伤血管内皮细胞，使其表达细胞间黏附分子-1（ICAM-1）和内皮细胞性白细胞黏附分子-1（ELAM-1）等黏附分子，导致血管壁进一步损伤。

【病理】

本病病理变化为全身性血管炎，好发于冠状动脉。病理过程可分为四期，各期变化如下：

Ⅰ期　约1～9天，小动脉周围炎症，冠状动脉主要分支血管壁上的小营养动脉和静脉受到侵犯。心包、心肌间质及心内膜炎症浸润，包括中性粒细胞、嗜酸性粒细胞及淋巴细胞。

Ⅱ期　约12～25天，冠状动脉主要分支全层血管炎，血管内皮水肿、血管壁平滑肌层及外膜炎性细胞浸润。弹力纤维和肌层断裂，可形成血栓和动脉瘤。

Ⅲ期　约28～31天，动脉炎症渐消退，血栓和肉芽形成，纤维组织增生，内膜明显增厚，导致冠状动脉部分或完全阻塞。

Ⅳ期　数月至数年，病变逐渐愈合，心肌疤痕形成，阻塞的动脉可能再通。

【临床表现】

1．主要表现

（1）发热：39～40℃，持续7～14天或更长，呈稽留或弛张热型，抗生素治疗无效。

（2）球结合膜充血：于起病3～4天出现，无脓性分泌物，热退后消散。

（3）唇及口腔表现：唇充血皲裂，口腔黏膜弥漫充血，舌乳头突起、充血呈草莓舌。

（4）手足症状：急性期手足硬性水肿和掌跖红斑，恢复期指、趾端甲下和皮肤交界处出现膜状脱皮，指、趾甲有横沟，重者指、趾甲亦可脱落。

（5）皮肤表现：多形性皮斑和猩红热样皮疹，常在第一周出现。肛周皮肤发红、脱皮。

（6）颈淋巴结肿大：单侧或双侧，坚硬有触痛，但表面不红，无化脓。病初出现，热退时消散。

2．心脏表现　于疾病1～6周可出现心包炎、心肌炎、心内膜炎、心律失常。发生冠状动脉瘤或狭窄者，可无临床表现，少数可有心肌梗塞的症状。冠状动脉损害多发生于病程2～4周，但也可于疾病恢复期。心肌梗塞和冠状动脉瘤破裂可致心源性休克甚至猝死。

3．其他　可有间质性肺炎、无菌性脑膜炎、消化系统症状（腹痛、呕吐、腹泻、麻痹性肠梗阻、肝大、黄疸等）、关节痛和关节炎。

【辅助检查】

1．血液检查　周围血白细胞增高，以中性粒细胞为主，伴核左移。轻度贫血，血小板早期正常，第2～3周时增多。血沉增快，C-反应蛋白等急相蛋白、血浆纤维蛋白原和血浆黏度增高，血清转氨酶升高。

2．免疫学检查　血清IgG、IgM、IgA、IgE和血循环免疫复合物升高；TH_2类细胞因子如IL-6明显增高，总补体和C3正常或增高。

3．心电图　早期示非特异性ST-T变化；心包炎时可有广泛ST段抬高和低电压；心肌梗死时ST段明显抬高、T波倒置及异常Q波。

4．胸部平片　可示肺部纹理增多、模糊或有片状阴影，心影可扩大。

5．超声心动图　急性期可见心包积液，左室内径增大，二尖瓣、主动脉瓣或三尖瓣返流；

可有冠状动脉异常，如冠状动脉扩张（直径>3 mm，≤4 mm为轻度；4～7 mm为中度）、冠状动脉瘤（≥8 mm）、冠状动脉狭窄。

6．冠状动脉造影　超声波检查有多发性冠状动脉瘤、或心电图有心肌缺血表现者，应进行冠状动脉造影，以观察冠状动脉病变程度，指导治疗。

【诊断和鉴别诊断】

1．诊断标准（表9-11）

表9-11　川崎病的诊断标准

发热5天以上，伴下列5项临床表现中4项者，排除其他疾病后，即可诊断为川崎病：
（1）四肢变化：急性期掌跖红斑，手足硬性水肿；恢复期指趾端膜状脱皮
（2）多形性红斑
（3）眼结合膜充血，非化脓性
（4）唇充血皲裂，口腔黏膜弥漫充血，舌乳头突起、充血呈草莓舌
（5）颈部淋巴结肿大

注：如 5 项临床表现中不足 4 项，但超声心动图有冠状动脉损害，亦可确诊为川崎病。

2．IVIG非敏感型KD　目前对该病诊断尚无统一定义，还有“IVIG无反应型KD”、“IVIG耐药型KD”、“难治性KD”等多种表述。多数认为，KD患儿在发病10天内接受IVIG2 g/kg治疗，无论一次或分次输注48小时后体温仍高于38℃，或给药2～7天甚至2周）后再次发热，并符合至少一项KD诊断标准者，可考虑为IVIG非敏感型KD。

3．鉴别诊断　本病需与渗出性多形红斑、幼年特发性关节炎全身型、败血症和猩红热相鉴别。

【治疗】

1．阿司匹林　每日30～50 mg/kg，分2～3次服用，热退后3天逐渐减量，约2周左右减至每日3～5mg/kg，维持6～8周。如有冠状动脉病变时，应延长用药时间，直至冠状动脉恢复正常。

2．静脉注射丙种球蛋白（IVIG）　剂量为1～2 g/kg于8～12小时静脉缓慢输入，宜于发病早期（10天以内）应用，可迅速退热，预防冠状动脉病变发生。应同时合并应用阿司匹林，剂量和疗程同上。部分患儿对IVIG效果不好，可重复使用1～2次，但约1%～2%的病例仍然无效。应用过IVIG的患儿在 9 个月内不宜进行麻疹、风疹、腮腺炎等疫苗预防接种。

3．糖皮质激素　因可促进血栓形成，易发生冠状动脉瘤和影响冠脉病变修复，故不宜单独应用。IVIG治疗无效的患儿可考虑使用糖皮质激素，亦可与阿司匹林和双嘧达莫合并应用。剂量为每日2 mg/kg，用药2～4周。

4．其他治疗

（1）抗血小板聚集：除阿司匹林外可加用双嘧达莫，每日3～5 mg/kg。

（2）对症治疗：根据病情给予对症及支持疗法，如补充液体、保护肝脏、控制心力衰竭、纠正心律失常等，有心肌梗死时应及时进行溶栓治疗。

（3）心脏手术：严重的冠状动脉病变需要进行冠状动脉搭桥术。

5．IVIG非敏感型KD的治疗

（1）继续IVIG治疗：首剂IVIG后仍发热者，应尽早再次应用IVIG，可有效预防CAL，若治疗过晚，则不能预防冠状动脉损伤。建议再次使用剂量为2 g/kg一次性输注。

（2）糖皮质激素联用阿司匹林治疗：有学者建议IVIG非敏感型KD可以在IVIG使用基础上联合使用糖皮质激素加阿司匹林。

【预后】

川崎病为自限性疾病，多数预后良好。复发见于1%～2%的患儿。无冠状动脉病变患儿于出院后1、3、6月及1～2年进行一次全面检查（包括体检、心电图和超声心动图等）。未经有效治疗的患儿，15%～25%发生冠状动脉瘤，更应长期密切随访，每6～12月一次。冠状动脉瘤多于病后2年内自行消失，但常遗留管壁增厚和弹性减弱等功能异常。大的动脉瘤常不易完全消失，常致血栓形成或管腔狭窄。

（李　秋）

第十章　感染性疾病

第一节　病　毒　感　染

一、麻疹

麻疹（measles）是由麻疹病毒引起的已知最具传染性的呼吸道疾病之一，如果接触了麻疹病毒，几乎所有未接受免疫的儿童都将感染麻疹。病后大多可获得终身免疫。临床上以发热、上呼吸道炎、结膜炎、口腔麻疹黏膜斑（又称柯氏斑koplik’s spots）、全身斑丘疹及疹退后遗留色素沉着伴糠麸样脱屑为特征。死亡主要是由于肺炎等导致的严重并发症。世界卫生组织（WHO）发起的全球免疫活动已使最近5年间麻疹的死亡率减少了48%，一些国家和地区已经消灭了麻疹。

【病原学】

麻疹病毒属副黏病毒科，球形颗粒，有6种结构蛋白。仅存在一种血清型，抗原性稳定。人是唯一宿主。病后可产生持久的免疫力，大多可达到终身免疫。病毒在外界生存力弱，不耐热，对紫外线和消毒剂均敏感。随飞沫排出的病毒在室内可存活32小时，但在流通的空气中或阳光下半小时即失去活力。

【流行病学】

麻疹患者是唯一的传染源。感染早期病毒在患者呼吸道大量繁殖，含有病毒的分泌物经过患者的呼吸、咳嗽、喷嚏排出体外并悬浮于空气中，通过呼吸道进行传播。密切接触者亦可经污染病毒的手传播。麻疹患者出疹前后的5天均有传染性，有并发症的患者传染性可延长至出疹后10天。以冬春季发病为多。

【发病机制】

麻疹病毒通过鼻咽部进入人体，在呼吸道上皮细胞和局部淋巴组织中繁殖并侵入血液，通过血液的单核细胞向其他器官传播，如脾、胸腺、肺、肝脏、肾脏、消化道黏膜、结膜和皮肤，引起广泛损伤而出现一系列临床表现。由于免疫反应受到抑制，常并发喉炎、支气管肺炎或导致结核病复燃，特别是营养不良或免疫功能缺陷的儿童，可发生重型麻疹或因严重肺炎、腹泻、脑炎等并发症而导致死亡。

【病理】

病变部位广泛的单核细胞浸润、增生及形成多核巨细胞（华-佛细胞Warthin-Finkeldey giant cell）是麻疹的病理特征。基本病变主要见于皮肤、淋巴组织、呼吸道和肠道黏膜及结膜。毛细血管周围有严重的渗出，单核细胞增生，形成的多核巨细胞大小不一，内含多个核，核内外均有病毒集落（嗜酸性包涵体）。真皮和黏膜下层毛细血管内皮细胞充血、水肿、增生、单核细胞浸润并有浆液性渗出而形成麻疹皮疹和麻疹黏膜斑。由于皮疹处红细胞裂解，疹退后形成棕色色素沉着。麻疹病毒引起的间质性肺炎为Hecht巨细胞肺炎，继发细菌感染则引起支气管肺炎。亚急性硬化性全脑炎（subacute sclerosing panencephalitis, SSPE）患者有皮质和白质变性，细胞核及细胞浆内均见包涵体。

【临床表现】

近年来，由于疫苗的应用，麻疹的临床表现变得不十分规律，临床上可以见到以下几种情况。

1．典型表现

（1）潜伏期：大多为6～18天(平均10天左右)。潜伏期末可有低热、全身不适。

（2）前驱期：也称出疹前期，常持续3～4天。主要表现为：①发热：多为中度以上，热型不一。②在发热同时出现咳嗽、喷嚏、咽部充血等上呼吸道感染症状，特别是流涕、结膜充血、眼睑水肿、畏光、流泪等明显的眼、鼻卡他症状是本病特点。③麻疹黏膜斑（Koplik斑）：是麻疹早期具有特征性的体征，一般在出疹前1～2天出现。开始时见于下磨牙相对的颊黏膜上，为直径约0.5～1.0 mm的灰白色小点，周围有红晕，常在1～2天内迅速增多，可累及整个颊黏膜并蔓延至唇部黏膜，于出疹后逐渐消失，可留有暗红色小点。④部分病例可有一些非特异症状，如全身不适、食欲减退、精神不振等。婴儿可有呕吐、腹泻等消化系统症状。偶见皮肤荨麻疹、隐约斑疹或猩红热样皮疹，在出现典型皮疹时消失。

（3）出疹期：多在发热3～4天后出皮疹，此时全身中毒症状加重，体温可突然高达40～40.5℃，咳嗽加剧，伴嗜睡或烦躁不安，重者有谵妄、抽搐。皮疹先出现于耳后、发际，渐及额、面、颈部，自上而下蔓延至躯干、四肢，最后达手掌与足底。皮疹初为红色斑丘疹，呈充血性，疹间可见正常皮肤，不伴痒感。以后部分融合成片，色加深呈暗红。此期肺部可闻干、湿性啰音，X线检查可见肺纹理增多或轻重不等弥漫性肺部浸润。

（4）恢复期：若无并发症发生，出疹3～4天后发热开始减退，食欲、精神等全身症状逐渐好转，皮疹按出疹的先后顺序开始消退，疹退后皮肤有棕色色素沉着伴糠麸样脱屑，一般7～10天痊愈。

2．非典型麻疹

（1）轻型麻疹：多见于有部分免疫者，如潜伏期内接受过丙种球蛋白或8个月以下有母亲被动抗体的婴儿。主要临床特点为一过性低热，轻度眼、鼻卡他症状，全身情况良好，可无麻疹黏膜斑，皮疹稀疏、色淡，消失快，疹退后无色素沉着或脱屑，无并发症。常需要靠流行病学资料和麻疹病毒血清学检查确诊。

（2）重型麻疹：主要见于营养不良，免疫力低下继发严重感染者。体温持续40℃以上，中毒症状重，伴惊厥，昏迷。皮疹密集融合，呈紫蓝色出血性皮疹者常伴有黏膜和消化道出血，或咯血、血尿、血小板减少等，称为黑麻疹，可能是弥散性血管内凝血的一种形式。部分患者疹出不透、色暗淡，或皮疹骤退、四肢冰冷、血压下降出现循环衰竭表现。此型患儿常有肺炎、心力衰竭等并发症，死亡率高。

（3）异型麻疹：主要见于接种过麻疹灭活疫苗而再次感染麻疹野病毒株者。典型症状是持续高热、乏力、肌痛、头痛或伴四肢浮肿，皮疹不典型，呈多样性，出疹顺序可从四肢远端开始延及躯干、面部。易发生肺炎。本型少见，临床诊断较困难，麻疹病毒血清这检查有助诊断。

【并发症】

1．肺炎　是麻疹最常见的并发症，占麻疹患儿死因的90％以上。多见于5岁以下小儿。由麻疹病毒本身引起的间质性肺炎多不严重，常在出疹及体温下降后消退。继发性肺炎病原体多为细菌性，常见金黄色葡萄球菌、肺炎链球菌、流感嗜血杆菌等故易并发脓胸和脓气胸。部分为病毒性，多见腺病毒。也可为多种病原体混合感染。主要见于重度营养不良或免疫功能低下的小儿，临床症状较重、体征明显，预后较差。

2．喉炎　由于麻疹病毒本身可导致整个呼吸道炎症，故麻疹患儿常有轻度喉炎表现。如并发细菌感染时喉部组织明显水肿，分泌物增多，临床出现声音嘶哑、犬吠样咳嗽、吸气性呼吸困难及三凹征，严重者因喉梗阻而窒息死亡。

3．心肌炎　常见于营养不良和并发肺炎的小儿。轻者仅有心音低钝、心率增快和一过性心电图改变，重者可出现心力衰竭、心源性休克。

4．神经系统

（1）麻疹脑炎：发病率约为1‰～2‰，患儿常在出疹后的2～6天再次发热，临床表现和脑脊液改变与病毒性脑炎相似。脑炎的轻重与麻疹轻重无关。病死率约为15%，存活者中智能障碍、瘫痪、癫痫等后遗症的发生率可达20%以上。

（2）亚急性硬化性全脑炎：是少见的麻疹远期并发症，发病率约为1/100万～4/100万。病理变化主要为脑组织慢性退行性病变。大多在患麻疹2～17年后发病，开始时症状隐匿，可仅为行为和情绪的改变，以后出现进行性智能减退，病情逐渐恶化，出现共济失调、视、听障碍、肌阵挛等表现。晚期因昏迷、强直性瘫痪而死亡。患者血清或脑脊液中麻疹病毒IgG抗体持续强阳性。

5．结核病恶化　麻疹患儿因免疫反应受到暂时抑制，可使体内原有潜伏的结核病灶重趋活动恶化，甚至播散而致粟粒性肺结核或结核性脑膜炎。

6．营养不良与维生素A缺乏症　由于麻疹病程中持续高热、食欲不振或护理不当，可致营养不良和维生素缺乏，常见维生素A缺乏，可引起干眼症，重者出现视力障碍，甚至角膜穿孔、失明。

【实验室检查】

1．血常规　血白细胞总数减少，淋巴细胞相对增多。

2．多核巨细胞检查　于出疹前2天至出疹后1天，取患者鼻、咽分泌物或尿沉渣涂片，瑞氏染色后直接镜检，可见多核巨细胞或包涵体细胞，阳性率较高。

3．血清学检查　多采用酶联免疫吸附试验（ELISA法）进行麻疹病毒特异性IgM抗体检测，敏感性和特异性均好，出疹早期即可出现阳性。

4．病毒抗原检测　用免疫荧光法检测鼻咽部分泌物或尿沉渣脱落细胞中麻疹病毒抗原，可早期快速帮助诊断。也可采用PCR法检测麻疹病毒RNA。

5．病毒分离　前驱期或出疹初期取血、尿或鼻咽分泌物接种人胚肾细胞或羊膜细胞进行麻疹病毒分离。出疹晚期则较难分离到病毒。

【诊断和鉴别诊断】

根据流行病学资料、麻疹接触史、急性发热、上呼吸道卡他症状、口腔麻疹黏膜斑、皮疹形态和出现顺序以及疹退后皮肤脱屑及色素沉着等特点，较易做出临床诊断。麻疹病毒血清IgM抗体阳性或分离到麻疹病毒可确诊。

鉴别诊断包括各种发热、出疹性疾病，（表10-1）。

表10-1　小儿出疹性疾病的鉴别诊断

	病原	全身症状及其他特征	皮疹特点	发热与皮疹关系
麻疹	麻疹病毒	呼吸道卡他性炎症，结膜炎，发热第2～3天口腔麻疹黏膜斑	红色斑丘疹，自头面部→颈→躯干→四肢，退疹后有色素沉着及细小脱屑	发热3～4天后出疹，出疹期为发热的高峰期
风疹	风疹病毒	全身症状轻，耳后、枕部淋巴结肿大并触痛	面部→躯干→四肢，斑丘疹，疹间有正常皮肤，退后无色素沉着及脱屑	发热半天至1天后出疹

	病原	全身症状及其他特征	皮疹特点	发热与皮疹关系
幼儿急疹	人疱疹病毒6型	一般情况好，高热时可有惊厥，耳后枕部淋巴结亦可肿大，常伴有轻度腹泻	红色细小密集斑丘疹，头面颈及躯干部多见，四肢较少，一天出齐，次日开始消退	高热3～5天，热退疹出
猩红热	乙型溶血性链球菌	高热，中毒症状重，咽峡炎，杨梅舌，环口苍白圈，扁桃体炎	皮肤弥漫充血，上有密集针尖大小丘疹，持续2～3天退疹，疹退后伴大片状脱皮	发热1～2天出疹，出疹时高热
肠道病毒感染	埃可病毒<br>柯萨奇病毒	发热、咽痛、流涕、结膜炎、腹泻、全身或颈、枕后淋巴结肿大	散在斑疹或斑丘疹，很少融合，1～3天消退，不脱屑，有时可呈紫癜样或水疱样皮疹	发热时或热退后出疹
药物疹		原发病症状	皮疹痒感，摩擦及受压部位多，与用药有关，斑丘疹、疱疹、猩红热样皮疹、荨麻疹	发热多为原发病引起，服药史

【治疗】

现在还没有特效的药物治疗麻疹，主要为对症治疗、加强护理和预防并发症。

1．一般治疗　卧床休息，保持室内适当的温度、湿度和空气流通，避免强光刺激。注意皮肤和眼、鼻、口腔清洁。鼓励多饮水，给予易消化和营养丰富的食物。

2．对症治疗　高热时可酌情使用上量退热剂，但应避免急骤退热，特别是在出疹期。糊口可适当给予镇静剂。频繁剧咳可用镇咳剂或雾化吸入。继发细菌感染可给抗生素。世界卫生组织（WHO）推荐给予麻疹患儿补充维生素A　20万～40万单位，每日1次口服，连服2剂可减少并发症的发生，有利于疾病的恢复。

3．并发症的治疗　有并发症者给予相应治疗。

【预防】

提高人群免疫力，减少麻疹易感人群是消除麻疹的关键。其他国家的经验表表，要实现消灭麻疹的目标，人群麻疹免疫力要达到并保持在95%的水平。

1．主动免疫　采用麻疹减毒活疫苗预防接种。我国儿童计划免疫程序规定出生8个月为麻疹疫苗的初种年龄，7岁儿童要完成第2次接种。此外，根据麻疹流行病学情况，在一定范围、短时间内对高发人群开展强化免疫接种。

2．被动免疫　接触麻疹后5天内立即给予免疫血清球蛋白0.25 ml/kg可预防发病。如果是使用量不足或接触麻疹5天以后使用，仅可减轻症状。被动免疫只能维持3～8周，以后应采取主动免疫。

3．控制传染源　对麻疹患者要做到早发现、早报告、早隔离、早治疗。一般隔离至出疹后5天，合并肺炎者延长至出疹后10天。对接触麻疹的易感儿应隔离检疫3周，并给予被动免疫。

4．切断传播途径　流行期间易感儿童避免到人群密集的场所去。患者停留过的房间应通风并用紫外线照射消毒，患者衣物应在阳光下暴晒。无并发症的轻症患儿可在家中隔离，以减少传播和继发医院内感染。

5．加强麻疹的监测管理　麻疹监测的目的是了解麻疹的流行病学特征、评价免疫等预防控制措施的效果、为制定有效的麻疹控制策略提供依据。对麻疹疑似病例要注意进行流行病学调查和必要的实验室检查，及时报告并采取针对性措施进行隔离观察，预防和控制疫情的发生和蔓延。

二、脊髓灰质炎

脊髓灰质炎（poliomyelitis）又称小儿麻痹症，是由脊髓灰质炎病毒（poliovirus）引起的严重危害儿童健康的急性传染病，是小儿致残的主要疾病之一。多发生在5岁以下的小儿，3岁以下占88%。本病无特效治疗方法，但可以应用疫苗有效预防。2000年10月世界卫生组织（WHO）宣布包括我国在内的本太平洋区域为无脊髓灰质炎地区，这是世界上继美洲区以外的第二个无脊髓灰质炎地区。

【病原与流行病学】

脊髓灰质炎病毒属于小RNA病毒科的肠道病毒，为20面体球形、无包膜的裸体颗粒。有三个血清型，各型间较少交叉免疫。该病毒体外生存力强，耐酸，耐乙醚、氯仿等有机溶剂，低温环境中能长期存活；高温、紫外线照射、含氯消毒剂、氧化剂等可将其灭活。人是自然界唯一宿主。

粪-口感染为本病的主要传播方式。感染之初患者的鼻咽分泌物也排出病毒，故亦可通过飞沫传播，但为时短暂。急性期患者和健康带病毒者的粪便是重要的病毒来源，其中隐性感染者（占90%以上）和轻型无麻痹患者是最危险的传染源。病程的潜伏期末和瘫痪前期传染性最大，热退后传染性减少。患儿粪便中脊髓灰质为病毒存在时间可长达2个月，但以发病2周内排出最多。一般以40天作为本病的隔离期。人群普遍易感，感染后获得对同型病毒株的持久免疫力。

【发病机制】

病毒经口进入人体，在咽部和肠壁的淋巴组织中增殖，同时向外排出病毒，如机体抵抗力强，患儿可无临床症状，形成隐性感染；少数患者病毒可侵入血液引起病毒血症，并侵犯呼吸道、消化道等组织引起前驱症状。此时如机体免疫系统能清除病毒则形成顿挫型感染；否则病毒可继续扩散到全身淋巴组织中大量增殖，并再次入血形成第二次病毒血症。如侵犯神经系统，轻者不发生瘫痪，称无瘫痪型；重者发生瘫痪，称瘫痪型。在此期间，任何使机体抵抗力降低的因素如劳累、感染、局部刺激（如外伤、肌内注射）、手术等均可使病情加重并促进瘫痪的发生。

【病理】

脊髓灰质炎病毒为嗜神经病毒，主要侵犯中枢神经系统的运动神经细胞，以脊髓前角运动神经元损害为主，尤其是颈段和腰段受损多见，脑干及其他部位受累次之。病灶特点为多发、散在且不对称。可见神经细胞胞质内染色体溶解，周围组织充血、水肿和血管周围为性细胞浸润。早期病变呈可逆性，病变严重者则因神经细胞坏死、瘢痕形成而造成持久性瘫痪。偶见局灶性心肌炎、间质性肺炎、肝、肾等其他器官病变。

【临床表现】

潜伏期一般为5～14天。临床表现轻重悬殊，有无症状型，又称隐性感染（占90%以上），顿挫型（约占4%～8%），无瘫痪型和瘫痪型。其中瘫痪型为本病的典型表现，可分为以下各期。

1．前驱期　主要表现为发热、全身不适、食欲不振、多汗、咽痛、咳嗽、流涕等上呼吸道感染症状。亦可见恶心、呕吐、腹痛、腹泻等消化道症状。持续1～4天，如病情不再发展而痊愈，即为顿挫型。

2．瘫痪前期　多数患者由前驱期进入本期，少数于前驱期症状消失数天后再次发热至本期，亦可无前驱期症状而从本期开始发病。患儿出现高热、头痛、颈、背、四肢肌肉疼痛，活动或变换体位时加重。同时有多汗、皮肤发红、烦躁不安等兴奋状态和颈强直、脑膜刺激征阳性等中枢神经系统感染的症状和体征。小婴儿拒抱，较大患儿体检可见：①三角架征（tripod

sign）：患儿坐起时需用两臂向后撑在床上使身体形似三角架以支持体位；②吻膝试验（kiss-the-knee test）阳性：小儿坐起后不能自如地弯颈使下颌抵膝；③头下垂征（head drop sign）：将手置于患者腋下，抬起期躯干时，头与躯干不能平行。此时脑脊液已出现异常，呈现细胞蛋白分离现象。若3～5天后热退，症状消失则为无瘫痪型；如病情继续发展，浅反射和深腱反射逐渐减弱至消失，可能发生瘫痪。

3．瘫痪期　临床上无法将此期与瘫痪前期截然分开，一般于起病后的2～7天或第二次发热后1～2天出现不对称性弛缓性瘫痪，随发热而加重，热退后瘫痪不再进展。无感觉障碍，大小便功能障碍少见。根据病变部位分为以下类型：

（1）脊髓型：最常见。多表现为不对称的单侧下肢弛缓性瘫痪，近端肌群瘫痪程度重于远端。如累及颈背肌、膈肌、肋间肌时，可出现抬头及坐起困难、呼吸运动受限、矛盾呼吸等表现。腹肌、肠肌瘫痪则可引起肠麻痹、顽固性便秘；膀胱肌瘫痪时出现尿潴留或尿失禁。

（2）延髓型：病毒侵犯延髓呼吸中枢、循环中枢及脑神经的运动神经核，病情大多严重，可见脑神经麻痹及呼吸、循环受损的表现。常与脊髓型同时发生。

（3）脑型：较少见。呈弥漫性或局灶性脑炎，临床表现与其他病毒性脑炎无异。可有上运动神经元瘫痪。

（4）混合型：同时存在上述两种或两种以上类型的表现。

4．恢复期　一般在瘫痪后1～2周瘫痪的肌肉开始恢复，常从肢体远端的手指、足趾开始，继之近端大肌群，并逐渐上升至腰部。轻症1～3个月恢复，重症需更长时间。

5．后遗症期　因运动神经元严重受损而形成持久性瘫痪，1～2年内仍不能恢复则为后遗症。受累肌群萎缩，形成马蹄足内翻或外翻、脊柱弯曲等畸形。

【并发症】

呼吸肌麻痹者可继发吸入性肺炎、肺不张；尿潴留易并发尿路感染；长期卧床可致褥疮、肌萎缩、骨质脱钙、尿路结石和肾衰竭等。

【实验室检查】

1．脑脊液　瘫痪前期及瘫痪早期可见细胞数增多，蛋白增加不明显，呈细胞蛋白分离现象，对诊断有一定参考价值。至瘫痪第3周，细胞数多已恢复正常，而蛋白质仍继续增高，4～6周后方恢复正常。

2．血清学检查　病后未再服用过脊髓灰质炎疫苗的患者，发病一个月内用ELISA法检测患者血液及脑脊液中抗脊髓灰质炎病毒特异性IgM抗体，阳性可早期帮助诊断；恢复期患者血清中特异性IgG抗体滴定度较急性期有4倍以上，对诊断有一定意义。

3．病毒分离　粪便病毒分离是本病最重要的确诊性检查。对发病两周内、病后未再服过脊髓灰质炎减毒活疫苗的患者，间隔24～48小时，收集双份粪便标本（重量≥5 g），及时冷藏4℃以下送各级疾控中心脊灰实验室检测。发病1周内，从患儿鼻咽部、血、脑脊液中也可分离出病毒。

【诊断与鉴别诊断】

脊髓灰质炎出现典型瘫痪症状时，诊断并不困难。瘫痪出现前多不易确立诊断。血清学检查和大便病毒分离阳性可确诊。需与其他急性弛缓性麻痹（AFP）相鉴别。

1．　急性感染性多发性神经根神经炎（Guillain-Barre综合征）　起病前1～2周常有呼吸道或消化道感染史，一般不发热，由远端开始的上行性、对称性、弛缓性肢体瘫痪，多有感觉障碍。面神经、舌咽神经可受累，病情严重者常有呼吸肌麻痹。脑脊液呈蛋白细胞分离现象。血清学检查和大便分离阴性（表10-2）。

表10-2 脊髓灰质炎（瘫痪型）与感染性多发性神经根神经炎的鉴别要点

	脊髓灰质炎	感染性多发性神经根神经炎
发病早期	多有发热	很少有发热
瘫痪肢体	不对称弛缓性瘫痪，且近端重于远端	对称性弛缓性瘫痪，且远端重于近端
感觉过敏	有	无
感觉障碍	无	有
早期脑脊液变化	呈细胞蛋白分离	呈蛋白细胞分离
遗留后遗症	多有	多无

2．家族性周期性麻痹 较少见，常有家族史及周期性发作史，突然起病，发展迅速，对称性四肢弛缓性瘫痪。发作时血钾降低，补钾后迅速恢复。

3．周围神经炎 臀部注射时位置不当、维生素C缺乏、白喉后神经病变等引起的瘫痪，可根据病史、感觉检查和有关临床特征鉴别。

4．假性瘫痪 婴儿如有先天性髋关节脱位、骨折、骨髓炎、骨膜下血肿时可见假性瘫痪。详细询问病史、体格检查，必要时经X线检查容易确诊。

5．其他原因所致弛缓性麻痹应进行病原学检查来确诊。

【治疗】

目前尚无药物可控制瘫痪的发生和发展，主要是对症处理和支持治疗。

1．前驱期和瘫痪前期 卧床休息，隔离40天。避免劳累、肌注及手术等刺激。肌肉痉挛疼痛可予热敷或口服镇痛剂。静脉滴注高渗葡萄糖及维生素C，可减轻神经组织水肿。有条件可静脉输注丙种球蛋白400 mg/(kg·d)，连用2～3天，有减轻病情的作用。早期应用α-干扰素有抑制病毒复制和免疫调节作用，100万 U /d肌注，14天为一疗程。

2．瘫痪期 瘫痪肢体置功能位置，防止畸形。地巴唑0.1～0.2 mg/(kg·d) 顿服，10天为一疗程，有兴奋脊髓和扩张血管的作用；加兰他敏能促进神经传导，0.05～0.1 mg/(kg·d)肌注，20～40天为一疗程；$VitB_{12}$能促进神经细胞的代谢，0.1 mg/d肌注。呼吸肌麻痹者及早使用呼吸机；吞咽困难者用胃管保证营养；继发感染者选用适宜抗生素治疗。

3．恢复期及后遗症期 尽早开始主动和被动锻炼，防止肌肉萎缩。也可采用针灸、按摩及理疗等，促进肌肉功能恢复，严重肢体畸形可手术矫正。

【预防】

1．主动免疫 口服脊髓灰质炎减毒活疫苗糖丸，基础免疫自出生后2个月龄婴儿开始，连服3次，每次间隔1个月，4岁时加强免疫一次。还可根据需要对5岁以下儿童实施基础免疫外的强化补充免疫接种。

2．被动免疫 未服用疫苗而与患者有密切接触的5岁以下小儿或有先天性免疫缺陷的儿童应及早注射丙种球蛋白0.3～0.5 ml/(kg·次)，每日1次，连用2日，可防止发病或减轻症状。

【监测】

为进一步落实《2003～2010年全国保持无脊髓灰质炎状态行动计划》，应做了对急性弛缓性麻痹（AFP）病例的主动监测。发现急性弛缓性麻痹的患者或疑似患者，要在24小时内向当地疾病控制中心进行报告，并及时隔离患者，自发病之日起至少隔离40天。对有密切接触史的易感者要进行医学观察20天。所有AFP病例均应按标准采集双份大便标本用于病毒分离，并尽可能进行血清学检测。

三、水痘

水痘（chichenpox, varicella）是一种传染性极强的儿童期出疹性疾病，与带状疱疹（herpeszoster）为同一病毒所引起的两种不同表现的临床病症。水痘为原发感染。经过飞沫或接触传播，感染后可获得持久的免疫力，但以后可以发生带状疱疹。其临床特点为皮肤黏膜相继出现和同时存在斑疹、丘疹、疱疹和结痂等各类皮疹，全身症状轻微。冬春季节多发。

【病原与流行病学】

病原体为水痘-带状疱疹病毒（varicella-zoster virus, VZV），属疱疹病毒科α亚科。只有一个血清型，但与单纯疱疹病毒（HSV）抗原有部分交叉免疫。人是唯一宿主。该病毒在体外抵抗力弱，对热、酸和各种有机溶剂敏感，不能在痂皮中存活。

水痘患者为本病的传染源。主要通过空气飞沫经呼吸道传染，也可通过接触患者疱疹浆液而感染。传染期从出疹前1～2天至病损结痂，约7～8天。人群普遍易感，主要见于儿童，以2～6岁为高峰。20岁以后发病者占2%以下。孕妇分娩前6天患水痘可感染胎儿，出生后10天内发病。

【发病机制】

病毒经上呼吸道或眼结合膜侵入人体，在局部黏膜及淋巴组织内繁殖，然后侵入血液，形成病毒血症，如患者的免疫能力不能清除病毒，则病毒可到达单核-巨噬细胞系统内再次增殖后入血，引起各器官病变。主要损害部位在皮肤和黏膜，偶尔累及内脏。皮疹分批出现与间隙性病毒血症有关。皮疹出现1～4天后，产生特异性细胞免疫和抗体，病毒血症消失，症状随之缓解。

【病理】

水痘病变主要发生在皮肤和黏膜，皮肤真皮层毛细管内皮细胞肿胀，表皮棘状细胞层上皮细胞水肿变性，液化后形成水疱，内含大量病毒，以后液体吸收、结痂。有时疱疹破裂，留下浅表溃疡，很快愈合。黏膜病变与皮疹类似。免疫功能低下的小儿可发生全身性水痘，病变可波及肺、肝、脾、胰、肾、肠等，受累器官可有局灶性坏死、充血水肿和出血。并发脑炎者，可有脑水肿、充血和点状出血等。

【临床表现】

1．典型水痘　出疹前1天可出现前驱症状，如低热、不适、厌食等，次日出现皮疹。皮疹特点：①首发于头、面和躯干，继而扩展到四肢，末端稀少，呈向心性分布；②最初的皮疹为红色斑疹和丘疹，继之变为透明饱满的水疱，24小时后水疱内容物变混浊并中央凹陷，水疱易破溃，2～3天迅速结痂；③皮疹陆续分批出现，伴明显痒感，在疾病高峰期可见到斑疹、丘疹、疱疹和结痂同时存在；④黏膜皮疹还可出现在口腔、眼结膜、生殖器等处，易破溃形成浅溃疡，轻型水痘多为自限性疾病，10天左右痊愈，全身症状和皮疹较轻。皮疹结痂后一般不留瘢痕。

2．重症水痘　多发生在恶性疾病或免疫功能低下患儿。持续高热和全身中毒症状明显，皮疹多、且易融合成大疱型或出血性，可继发感染或伴血小板减少而发生暴发性紫癜。

3．先天性水痘　母亲在妊娠早期感染水痘可导致胎儿多发性先天畸形；若发生水痘数天后分娩可导致新生儿水痘，病死率25%～30%。新生儿水痘的皮疹有时酷似带状疱疹的皮疹。

【并发症】

最常见为皮肤继发感染如脓疱疮、丹毒、蜂窝织炎，甚至由此导致败血症等；水痘肺炎主要发生在免疫缺陷儿和新生儿中，其他年龄儿童很少见；神经系统可见水痘后脑炎、横贯性脊

髓炎、面神经瘫痪、Reye综合征等；其他少数病例可发生心肌炎、肝炎、肾炎、关节炎等。

【实验室检查】

1．外周血白细胞计数　白细胞总数正常或稍低。

2．疱疹刮片　刮取新鲜疱疹基底组织和疱疹液涂片，瑞氏染色见多核巨细胞；苏木素-伊红染色可查到细胞核内包涵体；或疱疹液直接荧光抗体染色查病毒抗原简捷有效。

3．病毒分离　取水痘疱疹液、涸部分泌物或血液作病毒分离。

4．血清学检查　血清水痘病毒特异性IgM抗体检测，可早期帮助诊断；份血清特异性IgG抗体滴度4倍以上增高也有助诊断。

【诊断和鉴别诊断】

典型水痘临床诊断不难。对非典型病例可选用实验室检查帮助确诊。水痘的鉴别诊断包括丘疹性荨麻疹以及能引起疱疹性皮肤损害的疾病，如肠道病毒或金黄色葡萄球菌感染、药物和接触性皮炎等。

【治疗】

水痘是自限性疾病，无合并症时以一般治疗和对症处理为主。加强护理，如勤换内衣、剪短患儿指甲、戴手套以防抓伤和减少继发感染等。保持空气流通，供给足够水分和易消化食物。皮肤瘙痒可局部使用炉甘石洗剂，必要时可给少量镇静剂。抗病毒药物首选阿昔洛韦，应尽早使用，一般应在皮疹出现的48小时内开始。口服2 mg/(kg·次)，每日4次；重症患者需静脉给药，10～20 mg/(kg·次)，每8小时1次。此外，早期使用α-干扰素能较快抑制皮疹发展，加速病情恢复。继发细菌感染时给抗生素治疗。皮质激素对水痘病程有不利影响，可导致病毒，一般不宜用。

【预防】

控制传染源，隔离患儿至皮疹全部结痂为止；对已接触的易患儿，应检疫3周。水痘减毒活疫苗能有效预防易患小儿发生水痘，其保护率可达85%～95%，并可持续10年以上。对正在使用大剂量糖皮质激素、免疫功能受损、恶性病患者、接触过患者的孕妇以及患水痘母亲的新生儿，在接触水痘72小时内肌注水痘-带状疱疹免疫球蛋白125～625 U/kg，可起到预防作用。

四、传染性单核细胞增多症

传染性单核细胞增多症(infectious mononucleosis, IM)是由EB病毒(Epstein-Barr virus, EBV)感染所导致的急性感染性疾病，主要侵犯儿童和青少年，临床上以发热、咽喉痛、肝脾和淋巴结肿大、外周血中淋巴细胞增多并出现单核样异型淋巴细胞等为其特征。由于其症状、体征的多样化和不典型病例在临床上逐渐增多，给诊断治疗带来了一定困难。

【病原学】

EBV是本病的病原体。1964年由Epstein和Barr首先从患恶性淋巴瘤(Burkitt Lymphoma)非洲儿童的瘤组织中发现，1968年由Henle等报道为本病的病原体，并在此后众多的研究中得到证实。EBV属于疱疹病毒，是一种嗜淋巴细胞的DNA病毒，主要侵犯B淋巴细胞（B淋巴细胞表面的CD21受体，与EB病毒受体相同)。电镜下病毒呈球形，直径约150～180nm；EBV基因组呈线状，但在受染细胞内，病毒DNA存在两种形式，一是线状DNA整合到窑主细胞染色体DNA中；另一种是以环状的游离体游离于宿主细胞DNA之外。这两种形式的DNA，因不同的宿主细胞而可独立或并存。

EBV有5种抗原成分，均能产生各自相应的抗体：①衣壳抗原(Viral capsid antigen, VCA)：可产生IgM和IgG抗体，VCA-IgG出现稍迟于前者，可持续多年或终生，故不能区别新近感染与既往感染。②早期抗原(early antigen, EA)：是EBV进入增殖性周期初期形成的一种抗原，其中EA-D成分是EBV活跃增殖的标志。EA-IgG抗体于病后3～4周达高峰，持续3～6个月。③核心抗原(nuclear antigen, EBNA)：EBNA-IgG于病后3～4周出现，持续终生，是既往感染的标志。④淋巴细胞决定的膜抗原(Lymphocyte determinant membrane antigen, LYDMA)：带有LYDMA的B细胞是细胞毒性T(T_c)细胞攻击的靶细胞，其抗原为补体结合抗体，出现和持续时间与EBNA-IgG相同，也是既往感染的标志。⑤膜抗原(membrane antigen，MA)：是中和性抗原，可产生相应中和抗体，其出现和持续时间与EBNA-IgG相同。

【流行病学】

本病世界各地均有发生，多呈散发性，但也不时出现一定规模的流行。全年均有发病，以秋末至初春为多。病后可获得较稳固的免疫力，再次发病者极少。患者和隐性感染者是传染源。病毒大量存在于唾液腺及唾液中，可持续或间断排毒达数周、数月甚至数年之久。由于病毒主要在口腔分泌物中，因此口-口传播是重要的传播途径，飞沫传播虽有可能但并不重要，偶可经输血传播。虽然也在妇女生殖道内发现EBV，但垂直传播问题尚有争议。本病主要见于儿童和青少年，性别差异不大。6岁以下小儿得病后大多表现为隐性或轻型感染，15岁以上感染者则后呈典型症状。超过35岁的患者少见。

【发病机制】

本病的发病机制尚未完全阐明。由于B淋巴细胞EBV表面有EBV受体，故EBV进入口腔后，可能首先感染咽扁桃体中的B淋巴细胞和口腔上皮细胞，并在细胞中进行增殖，导致细胞破坏，引起扁桃体炎和咽炎症状，局部淋巴结受累肿大。病毒还可在腮腺和其他唾液腺上皮细胞中繁殖，并可长期或间歇性向唾液中排放，然后进入血液，通过病毒血症或受感染的B淋巴细胞进行播撒，继而累及周身淋巴系统。受感染的B淋巴细胞表面抗原发生改变，引起T淋巴细胞的强烈免疫应答而转化为细胞毒性T细胞（主要是$CD8^+$ T细胞，T_c）。T_c细胞在免疫病理损伤形成中起着非常重要的作用，它一方面杀伤感染EBV的B细胞，另一方面侵犯许多组织器官而产生一系列的临床表现。患者血中的大量异常淋巴细胞（又称异型细胞）就是这种具有杀伤能力的T细胞。此外，本病发病机制除主要是由B、T细胞间的交互作用外，还有免疫复合物的沉积以及病毒对细胞的直接损害等因素。婴幼儿时期典型病例很少，主要是因为不能对EBV产生充分的免疫应答。

【病理】

淋巴细胞的良性增生是本病的基本病理特征。病理所见非化脓性淋巴结肿大，淋巴细胞及单核-吞噬细胞高度增生。肝、心、肾、肾上腺、肺、皮肤、中枢神经系统等重要脏器均可有淋巴细胞（包括成熟淋巴细胞、单核细胞及异型淋巴细胞）浸润及局限性坏死病灶。脾脏充满异型淋巴细胞，水肿，致脾脏质脆、易出血，甚至破裂。

【临床表现】

潜伏期5～15天。起病急缓不一。症状呈多样性，多数患者有乏力、头痛、畏寒、鼻塞、恶心、食欲减退、轻度腹泻等前驱症状。发病期典型表现有：

1．发热　一般均有发热，体温38.5～40℃不等，无固定热型，热程大多1～2周，少数可达数月。中毒症状多不严重。

2．咽峡炎　咽部、扁桃体、腭垂充血肿胀，可见出血点，伴有咽痛，少数有溃疡或假膜形成。咽部肿胀严重者可出现呼吸及吞咽困难。

3．淋巴结肿大　大多数患者有浅表淋巴结肿大，在病程第1周就可出现。全身淋巴结均可受累，以颈部最为常见。肘部滑车淋巴结肿大常提示有本病可能。肿大淋巴结直径很少超过3 cm，中等硬度，无明显压痛和粘连，常在热退后数周才消退。肠系膜淋巴结肿大时，可有腹痛。

4．肝、脾大　肝大者约占20％～62％，大多数在肋下2 cm以内，可出现肝功能异常，并伴有急性肝炎的上消化道症状，部分有轻度黄疸。约半数患者有轻度脾大，伴疼痛及压痛，偶可发生脾破裂。

5．皮疹　部分患者在病程中出现多形性皮疹，如丘疹、斑丘疹、荨麻疹、猩红热样斑疹、出血性皮疹等。多见于躯干。皮疹大多在4～6日出现，持续1周左右消退。

本病病程一般为2～3周，也可长至数月。偶有复发，但病程短，病情轻。婴幼儿感染常无典型表现，但血清EBV抗体可阳性。

【实验室检查】

1．血常规　外周血象改变是本病的重要特征。早期白细胞总数可正常或偏低，以后逐渐升高> 10×10^9/L，高者可达30×10^9～50×10^9/L。白细胞分类早期中性粒细胞增多，以后淋巴细胞数可达60％以上，并出现异型淋巴细胞。异型淋巴细胞超过10％或其绝对值超过1.0×10^9/L时，具有诊断意义。血小板计数常见减少，可能与病毒直接损伤或免疫复合物作用有关。

2．血清嗜异凝集试验（heterophil agglutination test, HAT）　患者血清中出现IgM嗜异性抗体，能凝集绵羊或马红细胞，阳性率达80％～90％。凝集效价在1：64以上，经豚鼠肾吸收后仍阳性者，具有诊断意义。5岁以下小儿试验多为阴性。

3．EBV特异性抗体检测　间接免疫荧光法和酶联免疫吸附法检测血清中VCA-IgM和EA-IgG。VCA-IgM阳性是新近EBV感染的标志，EA-IgG一过性升高是近期感染或EBV复制活跃的标志，均具有诊断价值。

4．EBV-DNA检测　采用聚合酶链反应（PCR）方法能快速、敏感、特异的检测患儿血清中含有高浓度EBV-DNA，提示存在病毒血症。

【诊断和鉴别诊断】

根据流行情况、典型临床表现（发热、咽痛、肝脾及淋巴结肿大），外周血异型淋巴细胞>10％、嗜异凝集试验阳性和EB病毒特异性抗体（VCA-IgM、EA-IgG）检测可做出临床诊断，特别是VCA-IgM阳性或急性期及恢复期双份血清VCA-IgG抗体效价呈 4 倍以上增高是诊断EBV急性感染最特异和最有价值的血清学试验，阳性可以确诊。

本病需与巨细胞病毒、腺病毒、肺炎支原体、甲肝病毒、风疹等感染所致的淋巴细胞和单核细胞增多相鉴别。其中巨细胞病毒所致者最常见，有人认为在嗜异性抗体阴性的类传染性单核细胞增多症中，几乎半数与CMV有关。

【治疗】

本病系自限性疾病，若无并发症，预后大多良好。临床上无特效的治疗方法，主要采取对症治疗。由于轻微的腹部创伤就有可能导致脾破裂，因此有脾大的患者2～3周内应避免与腹部接触的运动。抗菌药物对本病无效，仅在继发细菌感染时应用。抗病毒治疗可用阿昔洛韦800 mg/d，分 4 次口服，连服5天，有一定的疗效；更昔洛韦10 mg/(kg・d)，分2次静脉注射，亦可改善病情。静脉注射丙种球蛋白400 mg/(kg・d)，每日1次，连用4～5次，可使临床症状改善，缩短病程，早期给药效果更好。α-干扰素亦有一定治疗作用。重型患者短疗程应用肾上腺皮质激素可明显减轻症状。发生脾破裂时，应立即输血，并作手术治疗。

【预防】

由于除了传染性单核细胞增多症以外，一些恶性疾病包括鼻咽癌、霍奇金病等也与EB病毒感染有关。因此近年来，国内外正在研制EB病毒疫苗，除可用以预防本病外，尚考虑用于EBV感染相关的儿童恶性淋巴瘤和鼻咽癌的免疫预防。

五、流行性腮腺炎

流行性腮腺炎(mumps，epidemic parotitis)是由腮腺炎病毒引起的急性呼吸道传染病，常在幼儿园和学校中感染流行。以5~15岁患者较为多见，2岁以下、40岁以上很少发病。一次感染后可获得终身免疫，但个别抗体水平低下者亦可再次感染。临床上以腮腺肿大及疼痛为特征，各种唾液腺体及器官均可受累。

【病因和流行病学】

腮腺炎病毒属于副黏病毒科的单股RNA病毒。只有一个血清型。病毒颗粒呈圆形，大小悬殊，约100~200 nm，有包膜。对物理和化学因素敏感，来苏、福尔马林等均能在2~5分钟内将其灭活，紫外线照射也可将其杀灭，加热至56℃、20分钟即失去活力。人是病毒的唯一宿主。腮腺炎患者和健康带病毒者是本病的传染源，患者在腮腺肿大前6天到发病后5天或更长的时间均可排出病毒。主要通过呼吸道飞沫传播，亦可因唾液污染食具和玩具，通过直接接触而感染。全年均可发生感染流行，但以冬春季发病较多。

【发病机制】

病毒通过口、鼻侵入人体后，在上呼吸道黏膜上皮组织中生长增殖，导致局部炎症和免疫反应，并进入血液引起病毒血症，进而扩散到腮腺和全身各器官，亦可经口腔沿腮腺管传播到腮腺。由于病毒对腺体组织和神经组织具有高度亲和性，可使多种腺体(腮腺、舌下腺、颌下腺、胰腺、生殖腺等)发生炎症改变，如侵犯神经系统，可导致脑膜脑炎等严重病变。

【病理】

受侵犯的腺体出现非化脓性炎症为本病的病理特征，间质充血、水肿、点状出血、淋巴细胞浸润和腺泡坏死等。腺体导管细胞肿胀，管腔中充满坏死细胞及渗出物，使腺体分泌排出受阻，唾液中的淀粉酶经淋巴系统进入血液，使血、尿淀粉酶增高。

【临床表现】

潜伏期14~25天，平均18天。大多无前驱期症状，常以腮腺肿大为首发体症。常先见一侧，然后另一侧也相继肿大，2~3日内达高峰，面部一侧或双侧因肿大而变形，局部疼痛、过敏，开口咀嚼或吃酸性食物时胀痛加剧。肿大的腮腺以耳 垂为中心，向前、后、下发展，边缘不清，表面发热但多不红，触之有弹性感并有触痛。腮腺肿大可持续5日左右，以后逐渐消退。腮腺管口（位于上颌第二磨牙对面黏膜上）在早期可见红肿，有助于诊断。在腮腺肿胀时，常波及邻近的颌下腺和舌下腺。颌下腺肿大时颈前下颌处明显肿胀，可触及椭圆形腺体。舌下腺肿大时可见舌下及颈前下颌肿胀。病程中患者可有不同程度发热，持续时间不一，短者1~2天，多为5~7天，亦有体温始终正常者。可伴有头痛、乏力、令人减退等。

由于腮腺炎病毒有嗜腺体和嗜神经性，常侵人中枢神经系统和其他腺体、器官而出现以下并发症：

1．脑膜脑炎 较常见，常在腮腺炎高峰时出现，也可出现在腺腮肿大前或腮腺肿大消失以后。表现为发热、头痛、呕吐、颈项强直、克氏征阳性等，脑脊液的改变与其他病毒性脑炎相似。脑电图可有改变但不似其他病毒性脑炎明显，以脑膜受累为主，预后大多良好，常在2周内恢复正常，多无后遗症。如侵犯脑实质，可出现嗜睡、甚至昏迷等，并可能有神经系统后遗症甚至死亡。

2．睾丸炎 是男孩最常见的并发症，多为单侧。常发生在腮腺炎起病后的4~5天，肿大有

腮腺开始消退时。开始为睾丸疼痛，随之肿胀伴剧烈触痛，可并发附睾炎、鞘膜积液和阴囊水肿。大多数患者有严重的全身反应，突发高热、寒战等。一般10天左右消退，约1/3~1/2的病例发生不同程度的睾丸萎缩，如双侧萎缩可导致不育症。

3．卵巢炎 约5%~7%的青春期后女性患者可并发卵巢炎，症状多较轻，可出现下腹痛及压痛、月经不调等，不影响受孕。

4．胰腺炎 严重的急性胰腺炎较少见。常发生于腮腺肿大数日后，表现为上腹部剧痛和触痛，伴发热、寒战、反复呕吐等。由于单纯腮腺炎即可引起血、尿淀粉酶增高，因此淀粉酶升高不能作为诊断胰腺炎的证据，需作脂肪酶检查，有助于诊断。

5．耳聋 为听神经受累所致，发病率不高，大多为单侧性，不易及时发现，治疗困难，可成为永久性耳聋。

6．其他并发症 心肌炎较常见，而肾炎、乳腺炎、胸腺炎、甲状腺炎、泪 腺炎、角膜炎、血小板减少及关节炎等偶可发生。

【实验室检查】

1．血、尿淀粉酶测定 90%患者发病早期血清和尿淀粉酶有轻至中度增高，2周左右恢复正常。血脂酶增高有助于胰腺炎的诊断。

2．血清学检查 近年来大多采用ELISA法检测患者血清中腮腺炎病毒特异性IgM抗体，可以早期快速诊断（前提是1个月内未接种过腮腺炎减毒活疫苗）。双份血清特异性IgG抗体效价有4倍或4倍以上提高为阳性。亦可用PCR技术检测腮腺炎病毒RNA，有很高的敏感性。

3．病毒分离 在发病早期取患者唾液、尿液、脑脊液或血液标本，及时接种鸡胚或人胚肾细胞进行病毒分离实验，阳性标本采用红细胞吸附抑制试验呈血凝抑制试验进行鉴定，阳性者可以确诊。

【诊断与鉴别诊断】

根据流行病学史、接触史以及发热、腮腺和邻近腺体肿大疼痛等症状，临床诊断较容易。对可疑病例可进行血清学检查及病毒分离以确诊。鉴别诊断包括化脓性腮腺炎、其他病毒性腮腺炎以及其他原因引起的腮腺肿大如白血病、淋巴瘤、口眼干燥关节综合征或罕见的腮腺肿瘤等。

【治疗】

无特殊治疗，以对症处理为主。

注意保持口腔清洁，清淡包含，忌酸性食物，多饮水。对高热、头痛和并发睾丸炎者给予解热止痛药物。睾丸肿痛时可用丁字带托起。中药治疗多用清热解毒、软坚消痛方法，常用普济消毒饮加减内服和青黛散调醋局部外敷等。发病早期可使用利巴韦林15 mg/(kg·d)静滴，疗程5~7天。也可使用干扰素治疗，有加速消肿、缩短热程的效果。对重症患者可短期使用肾上腺素激素治疗，疗程3~5天。脑膜脑炎、胰腺炎等的治疗见相关章节。

【预防】

及早隔离患者直至腮腺肿胀完全消退为止。集体机构的接触儿童应检疫3周。保护易感儿可接种腮腺炎减毒活疫苗，除皮下接种外，采用喷喉、喷鼻或气雾吸入等，同样取得非常好的效果。国外应用麻疹-风疹-腮腺炎三联疫苗接种，也取得良好的保护作用。

（夏晓玲）

第二节 细菌感染

一、败血症

败血症（septicemia）过去的定义系指致病菌进入血循环并在其中繁殖，产生毒素而引起的全身性严重感染。败血症患者出现低灌注和脏器功能失调者称为重症败血症。近年来，对败血症的研究越来越重视机体对微生物及其毒素所产生的全身反应，并将宿主对微生物感染的全身反应称为脓毒血症（sepsis）。将人体对各种损害，包括细菌感染所引起的全身性炎症反应称为全身炎症反应综合征（systemic inflammatory response syndrome, SIRS）。新的败血症的定义是指微生物进入血循环并在其中繁殖，产生毒素，并发生SIRS。

【病因】

各种致病菌都可引起败血症。G^+球菌主要为葡萄球菌、肠球菌和链球菌；G^-细菌主要为大肠埃希菌、肺炎克雷伯杆菌、假单胞菌属、变形杆菌、克雷白菌属等；厌氧菌以脆弱类杆菌、梭状芽胞杆菌及消化道链状菌为多见。败血症致病菌种类可因不同年龄、性别、感染灶、原发病、免疫功能、感染场所和不同地区有一定差别。自抗生素应用以来，特别是随着新型抗生素的不断问世和广泛应用于临床，使G^+球菌感染有所下降，G^-菌及耐药菌株感染逐年上升。由于糖皮质激素等免疫抑制剂及抗肿瘤药物的广泛应用，机体防御功能受损，致使一些既往认为不致病或致病力弱的条件致病菌引起的败血症亦有所增加。

【发病机制】

侵入人体的病原微生物能否引起败血症，不仅与微生物的毒力及数量有关，更重要的是取决于人体的免疫防御功能。当人体的抵抗力因各种慢性疾病，皮肤黏膜屏障破坏、免疫抑制受到削弱时，致病微生物可自局部侵入血循环，细菌进入血循环后，在生长、增殖的同时产生了大量毒素，造成机体组织受损，进而激活TNF、IL-1、IL-6、IL-8、IFN-γ等细胞因子，发生SIRS，激活补体系统、凝血系统、血管舒缓素、激肽系统等，造成广汽的内皮细胞损伤、凝血及纤溶过程改变，血管张力丧失及心肌抑制，引发感染性休克、DIC和多器官功能衰竭（multiple organ failure, MOF）（图10-1）。

【病理】

败血症患者共同的和最显著的病理变化是毒血症引起的中毒改变。组织器官细胞变性、微血管栓塞、组织坏死、出血及炎症细胞浸润。除肺、肠、肝、肾、肾上腺等具有上述病变外，心、脾也常被波及。

【临床表现】

1．原发感染灶 多数败血症患者都有轻重不等的原发感染灶。原发感染灶的特点为所在部位红、肿、热、痛和功能障碍。

2．感染中毒症状 大多起病较急，突然发热或先有畏冷或寒战，继之高热，弛张热或稽留热，间歇或不定型。体弱、重症营养不良和小婴儿可不发热，甚至体温低于正常。精神萎靡或烦躁不安，面色苍白或青灰、头痛、肌肉、关节酸痛、软弱无力、不思饮食、气急、脉速、甚至呼吸困难。少数患者可有恶心、呕吐、腹痛、腹泻等胃肠道症状。重者可出现中毒性脑病、

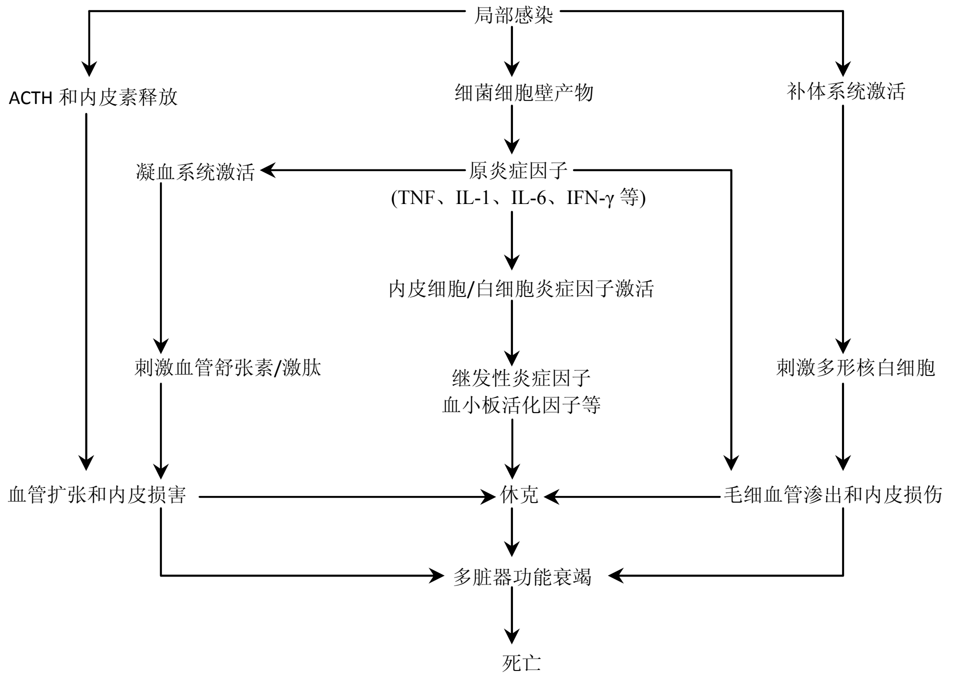

图 10-1 败血症的病理过程

中毒性心肌炎、肝炎、肠麻痹、感染性休克、DIC等。

3．皮疹 可有出血点、斑疹、丘疹或荨麻疹等。金黄色葡萄球菌败血症可出现猩红热样皮疹、荨麻疹；脑膜炎双球菌败血症常有大小不等的瘀点、瘀斑；坏死性皮疹可见于铜绿假单胞菌败血症。

4．肝脾大 一般仅轻度增大，当发生中毒性肝炎或肝脓肿时则肝增大显著且伴明显压痛，并可出现黄疸。

5．迁徙性病灶 随病原菌而不同，常见的迁徙性病灶有皮下及深部肌肉脓肿、肺炎、渗出性胸膜炎、肺脓肿、脓胸、感染性心内膜炎、化脓性心包炎、脑脓肿、骨髓炎等。

【实验室检查】

1．外周血象 白细胞总数以及中性粒细胞增加，核左称，细胞质中出现中毒颗粒。重症或衰弱者白细胞总数减少，红细胞以及血红蛋白常降低，重症者血小板减少。

2．病原学检查 可送血及骨髓培养、原发病灶及迁徙病灶的脓液培养及涂片和瘀点涂片寻找病原菌。为提高病原菌检出率，尽量于早期、抗菌药物治疗之前多次于发热和寒战发作期间采血，连续两次或同时从不同部位取双份标本以便能分清是污染还是致病菌。必要时应同时做厌氧菌、L型菌和真菌培养。

3．其他检查 聚合酶链反应（PCR）可用于检测病原菌DNA，方法快速，敏感性强，但易出现假阳性。对流免疫电泳、乳胶凝集试验用于检测病原菌抗原，有辅助诊断价值。

【诊断和鉴别诊断】

凡急性发热、外周血白细胞及中性粒细胞明显增高，而无局限于某一系统的急性感染时，都应考虑有败血症的可能。凡新近有皮肤感染、外伤，特别是有挤压疮疖史者，或者呼吸道、尿路等感染病灶或局灶感染虽经有效抗菌药物治疗但体温仍未控制且感染中毒症状明显，应高度怀疑败血症的可能。血培养和（或）骨髓培养阳性为败血症确诊的依据，但一次血培养阴性不能否定败血症的诊断。

败血症应与伤寒、粟粒性肺结核、恶性组织细胞病、结缔组织病如幼年特发性关节炎（全身型）等相鉴别。

【治疗】

1．一般治疗 患儿宜卧床休息，加强护理，供给营养丰富食品及足够液体，注意电解质平衡及维生素补充，防止褥疮等发生。感染中毒症状严重者可在足量应用有效抗生素的同时给予小剂量糖皮质激素治疗5~7天。

2．抗菌治疗 应尽早使用抗生素，在未获得病原学结果之前应根据情况给予抗菌药物经验治疗，以后再根据病原菌各类和药敏试验结果调整给药方案。常选用二联或三联杀菌性抗生素联合静脉给药，2~3周病情稳定后改用肌注或口服。疗程需持续到症状改善，退热后2~3周，或血培养转阴后1~2周或连续2~3次血培养阴性后方可停药。

针对革兰阳性球菌，可用青霉素加氨基糖苷类（阿米卡星或庆大霉素）；金黄色葡萄球菌而药菌株可用万古霉素；耐药性革兰阴性菌可用头孢三代抗生素。抗生素宜用足量或大剂量静脉给药，无尿或少尿者不宜用对肾脏有毒副作用的药物。

3．并发症的防治

（1）感染性休克，详见有关章节。

（2）原发炎症及迁徙性化脓性炎症或脓肿，应及时进行处理，有效引流。

（3） 基础病的治疗：败血症易发生在某些有基础疾病的患者，如糖尿病、肝硬化、慢性肾炎、恶性肿瘤等。对这些基础疾病仍应继续治疗。

二、感染性休克

感染性休克(septic shock)是发生在严重感染的基础上，由致病微生物及其产物所引起的急性循环障碍，有效循环血容量减少，组织血流灌注不足而致的复杂综合病征。

【病因】

多种病原微生物感染均可伴发感染性休克，其中尤以G^-菌所致者最多见。常见病原菌为：痢疾杆菌、脑膜炎球菌、铜绿假单胞菌、大肠杆菌、克雷白杆菌、沙门菌属及变形杆菌等。因G^-细菌能分泌内毒素，极易引起内毒素休克。严重G^+细菌感染亦能引起感染性休克。本病多见于暴发型流行性脑膜炎、中毒型细菌性痢疾、G^-杆菌败血症、大叶性肺炎、出血性坏死性肠炎等病患儿。另外，在有全身免疫功能缺陷时，如患有慢性病、白血病、淋巴瘤等，器官移植，长期应用免疫抑制剂，抗癌药物、放射治疗和放置静脉导管和导尿管等，极易诱发G^-菌感染而导致感染性休克。

【发病机制】

感现在认为休克是外因、内因和医源性因素构成致病网络作用下，机体由全身炎症反应综合征(SIRS)、严重败血症发展为多脏器功能不全综合征过程中的急性循环衰竭。

1．微循环障碍 在休克发生发展过程中，微血管经历痉挛、扩张和麻痹三个阶段。有效循环血量减少，回心血量进一步降低，血压明显下降。缺氧和酸中毒更明显。

2．免疫炎症反应失控 全身或局部感染时，病原体刺激机体细胞（主要是血管内皮细胞、中性粒细胞和单核-巨噬细胞）产生多种促炎和抗炎介质，由于促炎/抗炎平衡失调， 产生SIRS或代偿性抗炎反应综合征(Compensated anti-inflammatory response syndrome，CARS)。

3．神经体液、内分泌机制和其他体液介质。

【临床表现】

感染性休克的临床分期

1．休克代偿期 以脏器低灌注为主要表现。患者神志尚清，但烦躁焦虑，面色和皮肤苍白，口唇和甲床轻度发绀，肢端湿冷。呼吸、心率代偿性增快，血压正常或略低。

2．休克失代偿期 脏器低灌注进一步加重，患者烦躁或意识不清，面色青灰，四肢厥冷，唇、指（趾）端明显发绀，皮肤毛细血管再充盈时间>3秒，心音低钝，血压下降。

3．休克不可逆期 患儿表现为血压明显下降，心音极度低钝，常合并肺水肿或ARDS、DIC、肾衰竭、脑水肿和胃肠功能衰竭等多脏器功能衰竭（诊断标准见表10-3）。

【实验室检查】

1．外周血象 白细胞计数大多增高，在（10～30）$\times 10^9$/L之间；中性粒细胞增多伴核左移现象。血细胞压积和血红蛋白增高为血液浓缩的标志。

2．病原学检查 在抗菌药物治疗前常规进行血或其他体液、渗出液、脓液培养（包括厌氧菌培养）。分离得到致病菌后作药敏试验。

3．尿常规和肾功能检查 发生肾衰竭时，尿比重由初期的偏高转为低而固定（1.010左右）；尿/血肌酐比值>15，尿/血毫渗量之比<1.5；尿钠排泄量>40 mmol/L。

4．血液生化及血气分析 血清电解质测定：血钠偏低，血钾高低不一，取决于肾功能状况。血清酶的测定：血清丙氨酸氨基转移酶(ALT)，肌酸磷酸激酶(CPK)，乳酸脱氢酶同工酶的测定可反映组织脏器的损害情况。

5．血液流变学和有关DIC的检查 发生DIC时，血小板计数进行性降低，凝血酶原时间及凝血活酶时间延长，纤维蛋白原减少，纤维蛋白降解产物增多；凝血酶时间延长，血浆鱼精蛋白副凝试验（3P试验）阳性。

6．其他 心电图、X线检查等可按需进行。

【诊断】

中华急诊医学分会儿科组和中华儿科分会急诊组于2006年制订了儿科感染性休克（脓毒性休克）诊疗推荐方案。

1．感染性休克代偿期（早期） 临床表现符合下列6项中3项。

（1）意识改变：烦躁不安或萎靡，表情淡漠。意识模糊，甚至昏迷、惊厥。

（2）皮肤改变面色苍白发灰，唇周、指趾发绀，皮肤花纹，四肢凉。如有面色潮红，四肢温暖、皮肤干燥为暖休克。

（3）心率脉搏：外周动脉搏动细弱，心率、脉搏增快。

（4）毛细血管再充盈时间≥3秒（需除外环境温度影响）。

（5）尿量<1 ml/(kg·h)。

（6）代谢性酸中毒（除外其他缺血缺氧及代谢因素）。

2．感染性休克失代偿期 代偿期临床表现加重伴血压下降，收缩压<该年龄组第5百分位，或<该年龄组平均值减2个标准差。即:1~12个月<70 mmHg，1~10岁<70 mmHg+[2×年龄（岁）]，≥10岁<90 mmHg。

3．临床表现分型

（1）暖休克：为高动力性休克早期，可有意识改变、尿量减少或代谢性酸中毒等，但面色潮红、四肢温暖、脉搏无明显减弱，毛细血管再充盈时间无明显延长。此期容易漏诊，且可很快转为冷休克。心率快，血压低，过度通气，中心静脉压高，心排出量低多为失代偿表现。

（2）冷休克：为低动力性休克，皮肤苍白、花纹，四肢凉，脉搏快、细弱，毛细血管再充盈时间延长。儿科以冷休克为多。

【治疗】

1．液体复苏 充分液体复苏是逆转病情、降低病死率最关键的措施。需迅速建立2条静脉或骨髓输液通道。条件允许应放置中心静脉导管。

（1）第1小时快速输液：常用0.9%氯化钠，首剂20 ml/kg，10~20分钟静脉推注。然后评估循环与组织灌注情况（心率、血压、脉搏、毛细血管再充盈时间等）。若循环无明显改善，可再予第2剂、第3剂，每次均为10~20 ml/kg。总量最多可达40~60 ml/kg。第1小时输液既要重视液量不足，又要注意心肺功能（如肺部啰音、奔马律、肝大、呼吸做功增加等）。条件允许应做中心静脉压监测。第1小时液体复苏不用含糖液，血糖应控制在正常范围，若有低血糖可用葡萄糖0.5~1 g/kg纠正；当血糖大于200 mg/dl时，用胰岛素0.05 U/(kg・h)，称强化胰岛素治疗。

（2）继续和维持输液：由于血液重新分配及毛细血管渗漏等，感染性休克的液体丢失和持续低血容量可能持续数日，因此要继续补液和维持补液。继续输液可用1/2~2/3张液体，可根据血电解质测定结果进行调整，6~8小时内输液速度5~10 ml/(kg・h)。维持输液用1/3张液体，24小时内输液速度2~4 ml/ (kg・h)，24小时后根据情况进行调整。在保证通气前提下，根据血气分析结果给予碳酸氢钠，使pH达7.25即可。可适当补充胶体液，如血浆等。一般不输血，若HCT＜30%，应酌情输红细胞悬液或鲜血，使Hb>100 g/L。继续及维持补液阶段也要动态观察循环状态，评估液体量是否恰当，随时调整输液方案。

2．血管活性药物 在液体复苏基础上休克难以纠正，血压仍低或仍有明显灌注不良表现，可考虑使用血管活性药物以提高血压、改善脏器灌流。

（1）多巴胺：5~10 μg/(kg・min)持续静脉泵注，根据血压监测调整剂量，最大不宜超过20 μg/(kg・min)。

（2）肾上腺素：0.05~0.3 μg/(kg・min)持续静脉泵注，冷休克或有多巴胺抵抗时首选。

（3）去甲肾上腺素：0.05~0.3 μg/(kg・min)持续静脉泵注，暖休克或有多巴胺抵抗时首选。对儿茶酚胺反应的个体差异很大，用药要注意个体化原则。若有α受体敏感性下调，出现对去甲肾上腺素抵抗，有条件可试用血管紧张素或精氨酸血管加压素，此类药物发挥作用不受α受体影响。

（4）莨菪类药物：主要有阿托品、山莨菪碱(654-2)、东莨菪碱。

（5）正性肌力药物：伴有心功能障碍，疗效不佳时可使用正性肌力药物。常用多巴酚丁胺5~10 μg/(kg・min)持续静脉泵注，根据血压调整剂量，最大不宜超过20 μg/(kg・min)。对多巴酚丁胺抵抗，可用肾上腺素。若存在儿茶酚胺抵抗，可选用磷酸二酯酶抑制剂氨力农、米力农。

（6）硝普钠：心功能障碍严重且又存在高外周阻力的患儿，在液体复苏及应用正性肌力药物基础上可使用半衰期短的血管扩张剂，如硝普钠0.5~8 μg/(kg・min)，应从小剂量开始，避光使用。

在治疗过程中进行动态评估，适时调整药物剂量及药物种类，使血流动力学指标达到治疗目标。切勿突然停药，应逐渐减少用药剂量，必要时小剂量可持续数天。

3．积极控制感染和清除病灶 病原未明确前联合使用广谱高效抗生素静点，同时注意保护肾脏功能并及时清除病灶。

4．肾上腺皮质激素 对重症休克疑有肾上腺皮质功能低下（如流脑）、ARDS、长期使用肾上腺皮质激素或出现儿茶酚胺抵抗性休克时可以使用。目前主张小剂量、中疗程。氢化可的松3~5 mg/(kg・d)或甲泼尼龙2~3 mg/(kg・d)，分2~3次给予。

5．纠正凝血障碍 早期可给予小剂量肝素5~10 IU /kg皮下注射或静脉输注（注意肝素钠不能皮下注射），每6小时1次。若已明确有DIC，则应按DIC常规治疗。

6．其他治疗 ①保证氧供及通气，充分发挥呼吸代偿作用。可应用NCPAP，必要时小婴儿更需积极气管插管及机械通气，以免呼吸肌疲劳。儿童肺保护策略与成人相似。②注意各脏器功能支持，维持内环境稳定。③保证能量营养供给，注意监测血糖、血电解质。

【效果评价】

治疗目标是维持正常心肺功能，恢复正常灌注及血压。①毛细血管再充盈时间<2秒；②外周及中央动脉搏动均正常；③四肢温暖；④意识状态良好；⑤血压正常；⑥尿量>1 ml/(kg・h)。

表10-3 婴儿及儿童系统脏器功能衰竭的诊断标准的建议（1995年5月于太原）

心血管系统

1．血压（收缩压）：婴儿<40 mmHg，儿童<50 mmHg或需持续静脉输入药物如多巴胺>5 μg/(kg·min)可以维持上述血压。

2．心率：体温正常，安静状态，连续测定1分钟，婴儿<50次/分或>200次/分；儿童<50次/分或>180次/分。

3．心搏骤停。

4．血清pH<7.2（$PaCO_2$不高于正常值）

呼吸系统

1．呼吸频率：体温正常，安静状态，连续测定1分钟，婴儿<15次/分或>90次/分；儿童<10次/分或>70次/分。

2．$PaCO_2$>65 mmHg。

3．PaO_2<40 mmHg（不吸氧，除外青紫型心脏病）。

4．需机械通气（不包括手术后24小时内的患儿）。

5．PaO_2/FiO_2<200 mmHg（除外青紫型心脏病）。

神经系统

1．Glasgow昏迷评分≤7.

2．瞳孔固定、散大（除外药物影响）。

血液系统

1．急性贫血危象：Hb<50 g/L。

2．白细胞计数$<2\times10^9$/L。

3．血小板计数$<20\times10^9$/L。

肾脏系统

1．血清BUN>35.7 mmol/L（100 mg/dl）。

2．血清肌酐>176.8 μmol/L（2.0 mg/dl）。

3．因肾功能不良需透析者。

胃肠系统

1．应激性溃疡出血需输血。

2．出现中毒性肠麻痹，高度腹胀。

肝脏系统

总胆红素>85.5 μmol/L（5 mg/dl）及AST或LDH为正常的2倍以上（无溶血）。

三、中毒型细菌性痢疾

中毒型细菌性痢疾(bacillary dysentery，toxic type)以下简称中毒型菌痢，是急性细菌性痢疾的危重型。起病急骤，突然高热、反复惊厥、嗜睡、迅速发生休克、昏迷。本型多见于2~7岁健壮儿童，病死率高，必须积极抢救。

【病因及发病机制】

病原是痢疾杆菌，属于肠杆菌的志贺菌属，分A、B、C、D 四群（志贺菌、福氏菌、鲍氏菌、宋内氏菌），我国以福氏志贺菌多见。志贺菌内毒素从肠壁吸收入血后，引起发热、毒血症及急性微循环障碍。中毒性菌痢可发生脑水肿甚至脑疝，出现昏迷、抽搐及呼吸衰竭，是中毒性菌痢死亡的主要原因。

【病理】

中毒性菌痢肠道病变轻微，多见充血水肿，个别病例结肠有浅表溃疡，但全身病变重，多脏器的微血管痉挛及通透性增加，突出的病理改变为大脑及脑干水肿，神经细胞变性及点状出血，肾小管上皮细胞变性坏死，部分病例肾上腺充血、皮质出血和萎缩。

【临床表现】

潜伏期多数为1~2天，短者数小时。起病急，发展快，高热可>40℃(少数不高)，迅速发生呼吸衰竭、休克或昏迷，肠道症状多不明显甚至无腹痛与腹泻，也有在发热、排便后2~3天始发展为中毒型。根据其主要表现又可分为以下三型。

1．休克型（皮肤内脏微循环障碍型） 主要表现为感染性休克。见感染性休克章节。

2．脑型（脑微循环障碍型） 因脑缺氧、水肿而发生反复惊厥、昏迷和呼吸衰竭。早期有嗜睡、呕吐、头痛、血压偏高，心率相对缓慢。随病情进展很快进入昏迷、频繁或持续惊厥。瞳孔大小不等、对光反射消失，呼吸深浅不匀、节律不整、甚至呼吸停止。此型较严重，病死率高。

3．肺型（肺微循环障碍型） 又称呼吸窘迫综合征，以肺微循环障碍为主，常在中毒性痢疾脑型或休克型基础上发展而来，病情危重，病死率高。

4．混合型 上述两型或三型同时或先后出现，是最为凶险的一型，病死率很高。

严重病例常合并DIC、肾衰竭，偶可合并溶血尿毒综合征。

【实验室检查】

1．大便常规 病初可正常，以后出现脓血黏液便，镜检有成堆脓细胞、红细胞和吞噬细胞。

2．大便培养 可分离出痢疾杆菌。

3．外周血象 白细胞总数多增高至(10~20)×10^9/L以上。中性粒细胞为主，并可见核左移。当有DIC时，血小板明显减少。

4．免疫学检测 目前已有应用荧光物质标记的痢疾杆菌特异性多价抗体来检测大便标本中的致病菌，方法各异，都较快速，但特异性有待进一步提高。

5．特异性核酸检测 采用核酸杂交或PCR可直接检查粪便中的痢疾杆菌核酸，具有灵敏度高、特异性强、快速简便等优点。

【诊断与鉴别诊断】

2~7岁健壮儿童，夏秋季节突起高热，伴反复惊厥、脑病和（或）休克表现者，均应考虑中毒型菌痢，可用肛拭子或灌肠取粪便镜检有大量脓细胞或红细胞可初步确诊。本病应注意与高热惊厥、流行性乙型脑炎等疾病相鉴别。

【治疗】

病情凶险，必须及时抢救。

1．降温止惊 可综合使用物理、药物降温或亚冬眠疗法。惊厥不止者，可用地西泮0.3 mg/kg肌内注射或静脉注射（每次最大剂量≤10 mg）；或用水合氯醛40～60 mg/kg保留灌肠；或肌注苯巴比妥钠每次5～10 mg/kg。

2．感染性休克的治疗 参照感染性休克章节。

3．防治脑水肿和呼吸衰竭 保持呼吸道通畅，给氧。首选20%甘露醇降颅压，剂量为0.5～1 g/(kg·次)静注，每6~8小时一次，疗程3~5天，或与利尿剂交替使用，可短期静脉推注地塞米松。若出现呼吸衰竭应及早使用呼吸机。

4．抗菌治疗 为迅速控制感染，通常选用两种痢疾杆菌敏感的抗生素静脉滴注。因近年来痢疾杆菌对氨苄西林、庆大毒素等耐药菌株日益增多，故可选用阿米卡星、头孢噻肟钠或头孢曲松钠等药物。

第三节 结核病

一、概述

结核病(tuberculosis)是由结核杆菌引起的慢性感染性疾病。全身各个脏器均可受累，但以肺结核最常见。近年来，结核病的发病率有上升的趋势。多药耐药性结核菌株(MDR-TB)的产生，已成为防治结核病的严重问题。

【病因】

结核菌属于分板杆菌属，具有抗酸性，为需氧菌，革兰染色阳性，抗酸染色呈红色。分裂系列缓慢，在固体培养基上需4~6周才出现菌落。结核杆菌可分为4型：人型、牛型、鸟型和鼠型，对人类致病的主要为人型和牛型，其中人型是人类结核病的主要病原体。

【流行病学】

1．传染源 开放性肺结核（open pulmonary tuberculosis）患者是主要传染源，正规化疗2~4周后，随着痰菌排量减少而传染性降低。

2．传播途径 呼吸道为主要传染途径，小儿吸入带结核菌的飞沫或尘埃后即可引起感染，形成肺部原发病灶。少数经消化道传染者，产生咽部或肠道原发病灶；经皮肤或胎盘传染者少见。

3．易感人群 生活贫困、居住拥挤、营养不良、社会经济落后等是人群结核病高发的原因。新生儿对结核菌非常易感。儿童发病与否主要取决于：

（1）结核菌的毒力及数量。

（2）机体抵抗力的强弱：患麻疹、百日咳及白血病、淋巴瘤或艾滋病等小儿免疫功能受抑制和接受免疫抑制剂治疗者尤其好发结核病。

（3）遗传因素：与本病的发生有一定关系。单卵双胎儿结核病的一致性明显高于双卵双胎儿；亚洲人种（主要为菲律宾）发病率最高，白人最低；身材瘦长者较矮胖者易感。另外，经研究发现组织相容性抗原（HLA）与结核病密切相关，特别是有HLA-BW35抗原者发生结核病的危险性比一般小儿高7倍。

【发病机制】

小儿初次接触结核杆菌后是否发展为结核病，主要与机体的免疫力，细菌的毒力和数量有关，尤其与细胞免疫力强弱相关。机体在感染结核菌后，在产生免疫力的同时，也产生变态反应，均为致敏T细胞介导的，是同一细胞免疫过程的两种不同表现。

1．细胞介导的免疫反应 巨噬细胞吞噬和消化结核杆菌，并将特异性抗原传递给辅助T淋巴细胞（$CD4^+$细胞），巨噬细胞（主要为树突状细胞）分泌IL-12，诱导$CD4^+$细胞向TH1细胞极化，分泌和释放IFN-γ。IFN-γ增强细胞毒性T淋巴细胞（CTL、$CB8^+$细胞）和自然杀伤（NK）细胞的活性。上述细胞免疫反应，可最终消灭结核杆菌，但亦可导致宿主细胞和组织破坏。当细胞免疫反应不足以杀灭结核杆菌时，结核杆菌尚可通过巨噬细胞经淋巴管扩散到淋巴结。

2．迟发型变态反应 是宿主对结核菌及其产物的超常免疫反应，亦由T细胞介导，以巨噬细胞为效应细胞。由于迟发型变态反应直接和间接作用，引起细胞坏死及干酪样改变。甚至形成空洞。

感染结核杆菌后机体可获得免疫力，90%可终生不发病；5%因免疫力低下当即发病，即为原发性肺结核。另5%仅于日后机体免疫力降低时才发病，称为继发性肺结核，是成人肺结核的主要类型。初染结核杆菌除潜匿于胸部淋巴结外，亦可随感染初期菌血症转到其他脏器，并长期潜伏，成为肺外结核(extra-pulmonary tuberculosis)发病的来源。

【诊断】

力求早期诊断。包括发现病灶，决定其性质、范围和是否排菌，并确定其是否活动，以作为预防和治疗的根据。

1．病史

（1）中毒症状：有无长期低热、轻咳、盗汗、乏力、食欲减退、消瘦等。

（2）结核病接触史：应特别注意家庭病史，肯定的开放性结核病接触史对诊断有重要意义，年龄愈小，意义愈大。

（3）接种史：接种卡介苗可能提高对结核病的抵抗力，应仔细检查患儿左上臂有无卡介苗接种后疤痕。

（4）有无急性传染病史：特别是麻疹、百日咳等可使机体免疫功能暂时降低，致使体内隐伏的结核病灶活动、恶化，或成为感染结核病的诱因。

（5）无结核过敏表现：如结节性红斑、疱疹性结膜炎等。

2．结核菌素试验

（1）结核菌素试验：小儿受结核感染4～8周后，作结核菌素试验即呈阳性反应。结素反应属于迟发型变态反应。硬结平均直径不足5 mm为阴性，5~9 mm为阳性（+）；10~19 mm为中度阳性（++），≥20 mm为强阳性（+++），局部除硬结外，还有水疱、破溃、淋巴管炎及双圈反应等为极强阳性反应（++++）。

若患儿结核变态反应强烈，如患疱疹性结膜炎、结节性红斑或一过性多发性结核过敏性关节炎等，宜用1个结核菌素单位的PPD试验，以防局部的过度反应及可能的病灶反应。

（2）临床意义：

1）阳性反应见于：①接种卡介苗后。②年长儿无明显临床症状仅呈一般阳性反应，表示曾感染过结核杆菌。③婴幼儿尤其是未接种卡介苗者，阳性反应多表示体内有新的结核病灶。年龄愈小，活动性结核可能性愈大。④强阳性反应者，表示体内有活动性结核病。⑤由阴性反应转为阳性反应，或反应强度由原来小于10 mm增至大于10 mm，且增幅超过6 mm时，表示新近有感染。

接种卡介苗后与自然感染阳性反应的主要区别见表10-4。此外，非结核分支杆菌感染也可致PPD皮试阳性。

表10-4 接种卡介苗与自然感染阳性反应的主要区别

	接种卡介苗后	自然感染
硬结直径	多为5~9 mm	多为10~15 mm
硬结颜色	浅红	深红
硬结质地	较软、边缘不整	较硬、边缘清楚
阳性反应持续时间	较短，2~3天即消失	较长，可达7~10天以上
阳性反应的变化	有较明显的逐年减弱倾向，一般于3~5年内逐渐消失	短时间内反应无减弱倾向，可持续若干年，甚至终身

2）阴性反应见于：①未感染过结核；②结核迟发性变态反应前期（初次感染后4~8周内）；③假阴性反应，由于机体免疫功能低下或受抑制所致，如部分危重结核病；急性传染病如麻疹、水痘、风疹、百日咳等；体质极度衰弱者如重度营养不良、重度脱水、重度水肿等，应用糖皮质激素或其他免疫抑制剂治疗时；原发或继发免疫缺陷病；④技术误差或结核菌素失效。

3．实验室检查

（1）结核杆菌检查：从痰、胃液（婴幼儿可抽取空腹胃液）、脑脊液、浆膜腔液中找到结核杆菌是重要的确诊手段。

（2）免疫学诊断及分子生物学诊断

1）酶联免疫吸附试验(ELISA)：用于检测结核患者血清、浆膜腔液、脑脊液等的抗结核杆菌抗体。

2）分子生物学方法检测：如核酸杂交、聚合酶链反应（PCR）能快速检测标本中结核杆菌核酸物质。

3）血沉：多增快，反应结核病的活动性。

4．结核病影像学诊断

（1）X线检查：除正前位胸片外，同时应拍侧位片。可检出结核病灶的范围、性质、类型、活动或进展情况。重复检查有助于结核与非结核疾患的鉴别，亦可观察治疗效果。

（2）计算机断层扫描：胸部CT检查对肺结核的诊断及鉴别诊断很有意义，有利于发现隐蔽区病灶。特别是高分辨薄切CT可显示早期（2周内）粟粒性肺结核，≥4 mm的肺门纵隔淋巴结。淋巴结的钙化显示率也高于X线放射学检查。

5．其他辅助检查

（1）纤维支气管镜检查：有助于支气管内膜结核及支气管淋巴结结核的诊断。

（2）周围淋巴结穿刺液涂片检查：可发现特异性结核改变，如结核结节或干酪性坏死，有助于结核病的诊断和鉴别诊断。

（3）肺穿刺活体组织检查或胸腔镜取肺活体组织检查：病理和病原学检查，对特殊疑难病例确诊有帮助。

【治疗】

1．一般治疗 注意营养，选用富含蛋白质和维生素的食物。有明显结核中毒症状及高度衰弱者应卧床休息。居住环境应阳光充足，空气流通。避免传染麻疹、百日咳等疾病。一般原发型结核病可在门诊治疗，但要填报疫情，治疗过程中应定期复查随诊。

2．抗结核药物 治疗目的是：①杀灭病灶中的结核菌；②防止血行播散。治疗原则为：①早期治疗；②适宜剂量；③联合用药；④规律用药；⑤坚持全程；⑥分段治疗。

（1）目前常用的抗结核药物可分为两类：

1）杀菌药物：①全杀菌药：如异烟肼（isoniazid，INH）和利福平（rifampin，RFP）；②半杀菌药：如链霉素（streptomycin，SM）和吡嗪酰胺（pyrazinamide，PZA）。

2）抑菌药物：常用者有乙胺丁醇（ethambutol，ENB）及乙硫异烟胺（ethionamide，ETH）。

（2）针对耐药菌株的几种新型抗结核药：

1）老药的复合剂型：如rifamate（内含INH 150mg和RFP 300mg）；rifater（内含INH、RFP和PZA）等。

2）老药的衍生物：如rifapentine。

3）新的化学制剂：如力排肺疾（dipasic）。

（3）抗结核药的使用见表10-5：

表10-5 小儿抗结核药物

药物	剂量	给药途径	主要副作用
异烟肼（INH或H）	10 mg(≤300 mg/d)	口服（可肌注，静点）	肝毒性，末梢神经炎，过敏，皮疹和发热
利福平（RFP或R）	10 mg(≤450 mg/d)	口服	肝毒性，恶心、呕吐和流感样症状
链霉素（SM或S）	20~30 mg(≤0.75 g/d)	肌注	Ⅷ脑神经损害，肾毒性，过敏，皮疹和发热
吡嗪酰胺（PZA或Z）	20~30 mg(≤0.75 g/d)	口服	肝毒性，高尿酸血症，关节痛，过敏和发热
乙胺丁醇（EMB或E）	15~25 mg	口服	皮疹，视神经炎
乙硫异烟胺（ETH）丙硫异烟胺	10~15 mg	口服	胃肠道反应，肝毒性，末梢神经炎，过敏，皮疹，发热
卡那霉素	15~20 mg	肌注	肾毒性，Ⅷ脑神经损害
对氨柳酸	150~200 mg	口服	胃肠道反应，肝毒性，过敏，皮疹和发热

（4）化疗方案

1）标准疗法：一般用于无明显自觉症状的原发型肺结核。每日服用INH，RFP和（或）EMB，疗程9～12个月。

2）两阶段疗法：用于活动性原发型肺结核、急性粟粒性结核病及结核性脑膜炎。①强化治疗阶段：联用3~4种杀菌药物。目的在于迅速杀灭敏感菌及生长繁殖活跃的细菌与代谢低下的细菌，防止或减少耐药菌株的产生，为化疗的关键阶段。在长程化疗时，此阶段一般需3~4个月；短程疗法时一般为2个月。②巩固治疗阶段：联用2种抗结核药物，目的在于杀灭持续存在的细菌以巩固疗效，防止复发。在长程疗法时，此阶段可长达12～18个月；短程疗法时，一般为4个月。

3）短程疗法：为结核病现代疗法的重大进展，直接监督下服药与短程化疗是世界卫生组织（WHO）治愈结核病人的重要策略。短程化疗的作用机制是快速杀灭机体内处于不同繁殖速度的细胞内、外结核菌，使痰菌早期转阴并持久阴性，且病变吸收消散快，远期复发少。可选用以下几种6~9个月短程化疗方案：①2HRZ/4HR（数字为月数，以下同)；②2SHRZ/4HR；③2EHRZ/4HR。若无PZA则将疗程延长至9个月。

【预防】

1．控制传染源 结核菌涂片阳性病人是小儿结核病的主要传染源，早期发现及合理治疗结核菌涂片阳性病人，是预防小儿结核病的根本措施。

2．普及卡介菌接种 卡介苗接种是预防小儿结核病的有效措施。目前我国计划免疫要求在全国城乡普及新生儿卡介苗接种。

下列情况禁止接种卡介苗：①先天性胸腺发育不全症或严重联合免疫缺陷病患者；②急性传染病恢复期；③注射局部有湿疹或患全身性皮肤病；④结核菌素试验阳性。

3．预防性化疗

（1）目的：①预防儿童活动性肺结核；②预防肺外结核病发生；③预防青春期结核病复燃。

（2）适应证：①密切接触家庭内开放性肺结核者；②3岁以下婴幼儿未接种卡介苗而结核菌素试验阳性者；③结核菌素试验新近由阴性转为阳性者；④结核菌素试验阳性伴结核中毒症状者；⑤结核菌素试验阳性，新患麻疹或百日咳小儿；⑥结核菌素试验阳性小儿需较长期使用糖皮质激素或其它免疫抑制剂者。

（3）方法：INH每日l0 mg/kg(≤300 mg/d)，疗程6~9个月；或INH每日10 mg/kg(≤300 mg/d)联合RFP每日10 mg/kg(≤300 mg/d)，疗程3个月。

二、原发型肺结核

原发型肺结核（primary pulmonary tuberculosis）是原发性结核病中最常见者，为结核杆菌初次侵入肺部后发生的原发感染，是小儿肺结核的主要类型，占儿童各型肺结核总数的85.3％。原发型肺结核包括原发综合征（primary complex）和支气管淋巴结结核。前者由肺原发病灶、局部淋巴结病变和两者相连的淋巴管炎组成；后者以胸腔内肿大淋巴结为主。肺部原发病灶或因其范围较小，或被纵隔影掩盖，X线片无法查出，或原发病灶已经吸收，仅遗留局部肿大的淋巴结，故在临床上诊断为支气管淋巴结核。此两者并为一型，即原发型肺结核。

【病理】

肺部原发病灶多位于右侧，肺上叶底部和下叶的上部，近胸膜处。基本病变为渗出、增殖、坏死。渗出性病变以炎症细胞、单核细胞及纤维蛋白为主要成分；增殖性改变以结核结节及结核性肉芽肿为主；坏死的特征性改变为干酪样改变，常出现于渗出性病变中。结核性炎症的主要特征是上皮样细胞结节及郎格汉斯细胞。

典型的原发综合征呈“双极”病变，即一端为原发病灶，一端为肿大的肺门淋巴结、纵隔淋巴结。由于小儿机体处于高度过敏状态，使病灶周围炎症甚广泛，原发病灶范围扩大到一个肺段甚至一叶。小儿年龄愈小，此种大片性病变愈明显。引流淋巴结肿大多为单侧，但亦有对侧淋巴结受累者。

【临床表现】

症状轻重不一。轻者可无症状，一般起病缓慢，可有低热、食欲不振、疲乏、盗汗等结核中毒症状，多见于年龄较大儿童。婴幼儿及症状较重者可急性起病，高热可达39～40℃，但一般情况尚好，与发热不相称，持续2～3周后转为低热，并伴结核中毒症状，干咳和轻度呼吸困难是最常见的症状。婴儿可表现为体重不增或生长发育障碍。部分高度过敏状态小儿可出现眼疱疹性结膜炎，皮肤结节性红斑及（或）多发性一过性关节炎。当胸内淋巴结高度肿大时，可产生一系列压迫症状：压迫气管分叉处可出现类似百日咳样痉挛性咳嗽；压迫支气管使其部分阻塞时可引起喘鸣；压迫喉返神经可致声嘶；压迫静脉可致胸部一侧或双侧静脉怒张。

体格检查可见周围淋巴结不同程度肿大。肺部体征可不明显，与肺内病变不一致。胸片呈中到重度肺结核病变者，50％以上可无体征。如原发病灶较大，叩诊呈浊音，听诊呼吸音减低或有少许干湿啰音。婴儿可伴肝脏肿大。

【诊断和鉴别诊断】

应结合病史、临床表现、实验室检查及肺部影像学进行综合分析。

1．原发综合征：肺内原发灶大小不一。局部炎性淋巴结相对较大而肺部的初染灶相对较小是原发性肺结核的特征。婴幼儿病灶范围较广，可占据一肺段甚至一肺叶；年长儿病灶周围炎症较轻，阴影范围不大，多呈小圆形或小片状影。部分病例可见局部胸膜病变。小儿原发型肺结核在X线胸片上呈现典型哑铃状双极影者已少见。

2．支气管淋巴结结核：是小儿原发型肺结核X线胸片最为常见者。分三种类型：①炎症型：呈现从肺门向外扩展的密度增高阴影，边缘模糊，此为肺门部肿大淋巴结阴影；②结节型：表现为肺门区域圆形或卵圆形致密阴影，边缘清楚，突向肺野；③微小型：其特点是肺纹理紊乱，肺门形态异常，肺门周围呈小结节状及小点片状模糊阴影。

3．CT扫描 在显示小的原发灶、淋巴结肿大、胸膜改变和空洞方面优于X线检查。对疑诊

原发综合征但胸部平片正常的病例有助于诊断。也可发现由于肿大淋巴结压迫或淋巴结-支气管瘘引起的气管或支气管狭窄、扭曲、肺不张。增强扫描后淋巴结周围有环型强化，中心因干酪性坏死呈低密度。

4．纤维支气管镜检查 结核病变蔓延至支气管内造成支气管结核，纤维支气管镜检查可见到以下病变：①肿大淋巴结压迫支气管致管腔狭窄，或与支气管壁粘连固定，以致活动受限；②黏膜充血、水肿、溃疡或肉芽肿；③在淋巴结穿孔前期，可见突入支气管腔的肿块；④淋巴结穿孔形成淋巴结-支气管瘘，穿孔口呈火山样突起，色泽红而有干酪样物质排出。

本病应与上呼吸道感染、支气管炎、百日咳、风湿热、伤寒、各种肺炎、支气管异物、支气管扩张、纵隔良恶性肿瘤相鉴别。

【治疗】

一般治疗及治疗原则见总论。抗结核药物的应用如下：

1．无明显症状的原发型肺结核 选用标准疗法，每日服用INH、RFP和（或）EMB，疗程9~12个月。

2．活动性原发型肺结核 宜采用直接督导下短程化疗（DOTS）。强化治疗阶段宜用3~4种杀菌药：INH、RFP、PZA或SM，2～3个月后以INH，RFP或EMB巩固维持治疗。常用方案为2HRZ/4HR。

三、急性粟粒性肺结核

急性粟粒性肺结核（acute military tuberculosis of the lungs）或称急性血行播散性肺结核，是结核杆菌经血行播散而引起的肺结核，常是原发综合征发展的后果，主要见于小儿时期，尤其是婴幼儿。据北京儿童医院1966年对235例急性粟粒性肺结核患儿分析，3岁以下占59.1%，而1岁以内者占30.6%。年龄幼小，患麻疹、百日咳或营养不良时，机体免疫力低下，特别是HIV感染，易诱发本病。婴幼儿和儿童常并发结核性脑膜炎。

【病理】

多在原发感染后3～6个月以内发生。由于婴幼儿免疫功能低下，机体处于高度敏感状态，感染结核后，易形成结核杆菌血症。当原发病灶或淋巴结干酪样坏死发生溃破时，则大量细菌由此侵入血液而引起急性全身粟粒性结核病，可累及肺、脑膜、脑、肝、脾、肾、心、肾上腺、肠、腹膜、肠系膜淋巴结等。播散到上述脏器中的结核菌，在间质组织中形成细小结节。在肺脏中的结核结节分布于上肺部者多于下肺部，为灰白色半透明或淡黄色不透明的结节，如针尖或粟粒一般，约1～2 mm大小。显微镜检查示结核结节由类上皮细胞、淋巴细胞和朗格汉斯细胞加上中心干酪坏死性病灶组成。

【临床表现】

起病多急骤，婴幼儿多突然高热（39～40℃），呈稽留热或弛张热，部分病例体温可不太高，呈规则或不规则发热，常持续数周或数月，多伴有寒战、盗汗、食欲不振、咳嗽、面色苍白、气促和发绀等。肺部可听到细湿啰音而被误诊为肺炎。约50%以上的患儿在起病时就出现脑膜炎征象。部分患儿伴有肝脾以及浅表淋巴结肿大等。

6个月以下婴儿粟粒性结核的特点为发病急、症状重而不典型，累及器官多，特别是伴发结核性脑膜炎者居多，病程进展快，病死率高。

全身性粟粒性结核患者的眼底检查可发现脉络膜结核结节，后者分布于视网膜中心动脉分支周围。

【诊断和鉴别诊断】

诊断主要根据结核接触史、临床表现、肝脾大及结核菌素试验阳性，可疑者应进行细菌学检查、血清抗结核菌抗体检测与胸部X线摄片。胸部X线摄片常对诊断起决定性作用，早期因粟粒阴影细小而不易查出。至少在起病后2～3周后胸部摄片方可发现大小一致、分布均匀的粟粒状阴影，密布于两侧肺野。肺部CT扫描可见肺影显示大小、密度、分布一致粟粒影，部分病灶有融合。

临床上应与肺炎、伤寒、败血症、朗格罕组织细胞增生症、肺含铁血黄素沉着症及特发性肺间质疾病等相鉴别。

【治疗】

一般支持疗法见原发型肺结核。早期抗结核治疗甚为重要。

1．抗结核药物 目前主张将抗结核治疗的全疗程分为两个阶段进行，即强化抗结核治疗阶段及维持治疗阶段，此方案可提高疗效。前者于治疗开始时即给予强有力的四联杀菌药物如INH、RFP、PZA及SM。开始治疗越早杀灭细菌的效果越好，以后产生耐药菌的机会越小，此法对原发耐药病例亦有效。

2．糖皮质激素 有严重中毒症状及呼吸困难者，在应用足量抗结核药物的同时，可用泼尼松1～2 mg/(kg·d)，疗程1～2个月。

【预后】

病情多急重，但若能早期诊断和彻底治疗仍可治愈。如延误诊断和治疗，则可导致死亡。

四、结核性脑膜炎

结核性脑膜炎（tuberculous meningitis）简称结脑，是小儿结核病中最严重的类型。常在结核原发感染后1年以内发生，尤其在初染结核3～6个月最易发生。多见于3岁以内婴幼儿，约占60%。自普及卡介苗接种和有效抗结核药物应用以来，本病的发病率较过去明显降低，预后有很大改进，但若诊断不及时和治疗不当，病死率及后遗症的发生率仍较高，故早期诊断和合理治疗是改善本病预后的关键。

【发病机制】

结脑常为全身性粟粒性结核病的一部分，通过血行播散而来。婴幼儿中枢神经系统发育不成熟、血-脑屏障功能不完善、免疫功能低下与本病的发生密切相关。结脑亦可由脑实质或脑膜的结核病灶破溃，结核菌进入蛛网膜下腔及脑脊液中所致。偶见脊椎、颅骨或中耳与乳突的结核灶直接蔓延侵犯脑膜。

【病理】

1．脑膜病变 软脑膜弥漫充血、水肿、炎性渗出，并形成许多结核结节。蛛网膜下腔大量炎性渗出物积聚，因重力关系、脑底池腔大、脑底血管神经周围的毛细血管吸附作用等，使炎性渗出物易在脑底诸池聚集。渗出物中可见上皮样细胞、郎格汉斯细胞及干酪坏死。

2．脑神经损害 浆液纤维蛋白渗出物波及脑神经鞘，包围挤压颅神经引起脑神经损害，常见面神经、舌下神经、动眼神经、展神经障碍的临床症状。

3．脑部血管病变 在早期主要为急性动脉炎，病程较长者，增生性结核病变较明显，可见栓塞性动脉内膜炎，严重者可引起脑组织梗死、缺血、软化而致偏瘫。

4．脑实质病变 炎症可蔓延至脑实质，或脑实质原已有结核病变，可致结核性脑膜脑炎。少数病例脑实质内有结核瘤。

5．脑积水及室管炎 室管膜及脉络丛受累，出现脑室管膜炎。如室管膜或脉络丛结核病变使一侧或双侧室间孔粘连狭窄，可出现一侧或双侧脑室扩张。脑底部渗出物机化、粘连、堵塞使脑脊液循环受阻可导致脑积水。

6．脊髓病变 有时炎症蔓延至脊膜、脊髓及脊神经根，脊膜肿胀、充血、水肿和粘连，蛛网膜下腔完全闭塞。

【临床表现】

典型结脑起病多较缓慢。根据临床表现，病程大致可分为3期。

1．早期（前驱期） 约1～2周，主要症状为小儿性格改变，如少言、懒动、易倦、烦躁、易怒等。可有发热、食欲不振、盗汗、消瘦、呕吐、便秘（婴儿可为腹泻）等。年长儿可自诉头痛，多轻微或非持续性；婴儿则表现为蹙眉皱额，或凝视、嗜睡，或发育迟滞等。

2．中期（脑膜刺激期） 约1～2周，因颅内压增高致剧烈头痛、喷射性呕吐、嗜睡或烦躁不安、惊厥等。出现明显脑膜刺激征。幼婴则表现为前囟膨隆、颅缝裂开。此期可出现脑神经障碍，最常见者为面神经瘫痪，其次为动眼神经和外展神经瘫痪。部分患儿出现脑炎体征，如定向、运动及（或）语言障碍。眼底检查可见视乳头水肿、视神经炎或脉络膜粟粒状结核结节。

3．晚期（昏迷期） 约1～3周，以上症状逐渐加重，由意识朦胧，半昏迷继而昏迷。阵挛性或强直性惊厥频繁发作。患儿极度消瘦，呈舟状腹。常出现水、电解质代谢紊乱。最终因颅内压急剧增高导致脑疝致使呼吸及心血管运动中枢麻痹而死亡。

不典型结脑表现为：①婴幼儿起病急，进展较快，有时仅以惊厥为主诉；②早期出现脑实质损害者，可表现为舞蹈症或精神障碍；③早期出现脑血管损害者，可表现为肢体瘫痪；④合并脑结核瘤者可似颅内肿瘤表现；⑤当颅外结核病变极端严重时，可将脑膜炎表现掩盖而不易识别；⑥在抗结核治疗过程中发生脑膜炎时，常表现为顿挫型。

【诊断】

早期诊断主要依靠详细的病史询问，周密的临床观察及对本病高度的警惕性，综合资料全面分析，最可靠的诊断依据是脑脊液中查见结核杆菌。

1．病史 ①结核接触史：大多数结脑患儿有结核接触史，特别是家庭内开放性肺结核患者接触史，对小婴儿的诊断尤有意义；②卡介苗接种史：约大多数患儿未接种过卡介苗；③既往结核病史：尤其是1年内发现结核病又未经治疗者，对诊断颇有帮助；④近期急性传染病史：如麻疹、百日咳等常为结核病恶化的诱因。

2．临床表现 凡有上述病史的患儿出现性格改变、头痛、不明原因的呕吐、嗜睡或烦躁不安相交替及顽固性便秘时，即应考虑本病的可能。眼底检查发现有脉络膜粟粒结节对诊断有帮助。

3．脑脊液检查 对本病的诊断极为重要

常规检查：脑脊液压力增高，外观无色透明或呈毛玻璃样，蛛网膜下腔阻塞时，可呈黄色，静置12~24小时后，脑脊液中可有蜘蛛网状薄膜形成，取之涂片作抗酸染色，结核杆菌检出率较高。白细胞数多为（50～500）$\times 10^6$/L，分类以淋巴细胞为主，但急性进展期，脑膜新病灶或结核瘤破溃时，白细胞数可>1000$\times 10^6$/L，其中1/3病例分类以中性粒细胞为主。糖和氯化物均降低为结脑的典型改变。蛋白量增高，一般多为1.0～3.0 g/L，椎管阻塞时可高达40～50 g/L。对脑脊液改变不典型者，需重复化验，动态观察变化。脑脊液（5～10 m1）沉淀物涂片抗酸染色镜检阳性率可达30％。

4．其它检查

（1）结核菌抗原检测：以ELISA法检测脑脊液结核菌抗原，是敏感、快速诊断结脑的辅助方法。

（2）抗结核抗体测定：以ELISA法检测结脑患儿脑脊液PPD-IgM抗体和PPD-IgG抗体，其水平常高于血清中的水平。PPD-IgM抗体于病后2～4天开始出现，2周达高峰，至8周时基本降

至正常，为早期诊断依据之一；而PPD-IgG抗体于病后2周起逐渐上升，至6周达高峰，约在12周时降至正常。

（3）腺苷脱氨酶（adenosine deaminase，ADA）活性测定：ADA主要存在于T细胞中，有63％～100％结脑患者脑脊液ADA增高（>9 μ/L），ADA在结脑发病1个月内明显增高，治疗3个月后明显降低，为一简单可靠的早期诊断方法。

（4）结核菌素试验：阳性对诊断有帮助，但高达50％的患儿可呈阴性反应。

（5）脑脊液结核菌培养：是诊断结脑可靠的依据。

（6）聚合酶链反应（PCR）：应用PCR技术在结脑患儿脑脊液中扩增出结核菌所特有的DNA片段，能使脑脊液中极微量结核菌体DNA被准确地检测。

5．X线检查、CT 扫描或磁共振（MRl） 约85％结核性脑膜炎患儿的胸片有结核病改变，其中90％为活动性病变，呈粟粒型肺结核者占48％。胸片证明有血行播散性结核病对确诊结脑很有意义。脑CT在疾病早期可正常，随着病情进展可出现基底节阴影增强，脑池密度增高、模糊、钙化、脑室扩大、脑水肿或早期局灶性梗塞症。

【鉴别诊断】

应与化脓性脑膜炎、病毒性脑膜炎、隐球菌脑膜炎、脑肿瘤进行鉴别。

【并发症及后遗症】

最常见的并发症为脑积水、脑实质损害、脑出血及脑神经障碍。其中前3者是导致结脑死亡的常见原因。严重后遗症为脑积水、肢体瘫痪、智力低下、失明、失语、癫痫及尿崩症等。晚期结脑发生后遗症者约占2/3，而早期结脑后遗症甚少。

【治疗】

应抓住抗结核治疗和降低颅高压两个重点环节。

1．一般疗法 应卧床休息，细心护理，对昏迷患者可予鼻饲或胃肠外营养，以保证足够热量，应经常变换体位，以防止褥疮和坠积性肺炎。做好眼睛、口腔、皮肤的清洁护理。

2．抗结核治疗 联合应用易透过血-脑屏障的抗结核杀菌药物，分阶段治疗。

（1）强化治疗阶段：联合使用INH、RFP、PZA及SM。疗程3～4个月，其中INH每日15～25 mg/kg，RFP每日10～15 mg/kg（<450mg/d），PZA每日20～30 mg/kg（<750 mg/d），SM每日15～20 mg/kg（<750 mg/d）。开始治疗的1～2周，将INH全日量的一半加入10％葡萄糖中静脉滴注，余量口服，待病情好转后改为全日量口服。

（2）巩固治疗阶段：继用INH、RFP或EMB。RFP或EMB 9～12个月。抗结核药物总疗程不少于12个月，或待脑脊液恢复正常后继续治疗6个月。早期患者可采用9个月短程治疗方案（3HRZS/6HR）有效。

3．降低颅高压 最早于10天即可出现，故应及时控制颅内压，措施如下：

（1）脱水剂：常用20％甘露醇，一般剂量为每次0.5～1.0 g/kg，于30分钟内快速静脉注入。4～6小时一次，脑疝时可加大剂量至每次2 g/kg。2～3日后逐渐减量，7～10日停用。

（2）利尿剂：乙酰唑胺（diamox）一般于停用甘露醇前1～2天加用该药，每日20～40 mg/kg（<0.75 g/d）口服，根据颅内压情况，可服用1～3个月或更长，每日服或间歇服（服4日，停3日）。

（3）侧脑室穿刺引流：适用于急性脑积水而其它降颅压措施无效或疑有脑疝形成时。引流量根据脑积水严重程度而定，一般每日50～200 ml，持续引流时间为1～3周。有室管膜炎时可予侧脑室内注药。特别注意防止继发感染。

（4）腰椎穿刺减压及鞘内注药：适应证为：①颅内压较高，应用肾上腺皮质激素及甘露

醇效果不明显，但不急需作侧脑室引流或没有作侧脑室引流的条件者；②脑膜炎症控制不好以致颅内压难于控制者；③脑脊液蛋白量>3.0 g/L以上。方法为：根据颅内压情况，适当放出一定量脑脊液以减轻颅内压；3 岁以上每次注入INH20～50 mg及地塞米松2 mg，3岁以下剂量减半，开始为每日1次，1周后酌情改为隔日1次、1周2次及1周1次。2～4周为1疗程。

（5）分流手术：若由于脑底脑膜粘连梗阻发生梗阻性脑积水时，经侧脑室引流等难以奏效，而脑脊液检查已恢复正常，为彻底解决颅高压问题，可考虑作侧脑室小脑延髓池分流术。

4．糖皮质激素 能抑制炎症渗出从而降低颅内压，可减轻中毒症状及脑膜刺激症状，有利于脑脊液循环，并可减少粘连，从而减轻或防止脑积水的发生。是抗结核药物有效的辅助疗法，早期使用效果好。一般使用泼尼松，每日1～2 mg/kg（<45 mg/d），1个月后逐渐减量，疗程8～12周。

5．对症治疗

（1）惊厥的处理：见第16章。

（2）水、电解质紊乱的处理：①稀释性低钠血症：由于丘脑下部视上核和室旁核受结核炎症渗出物刺激，使垂体分泌抗利尿激素增多，导致远端肾小管回吸收水增加，造成稀释性低钠血症。如水潴留过多，可致水中毒，出现尿少、头痛、频繁呕吐、反复惊厥甚至昏迷。治疗宜用3％氯化钠液静滴，每次6～12 ml/kg，可提高血钠5～l0 mmol/L，同时控制入水量。②脑性失盐综合征：结脑患儿可因间脑或中脑发生损害，调节醛固酮的中枢失灵，使醛固酮分泌减少；或因促尿钠排泄激素过多，大量Na^+由肾排出，同时带出大量水分，造成脑性失盐综合征。应检测血钠、尿钠，以便及时发现，可用2：1等张含钠液补充部分失去的体液后，酌情补以3％氯化钠液以提高血钠浓度。③低钾血症：宜用含0.2％氯化钾的等张溶液静滴，或口服补钾。

6．随访观察 复发病例全部发生在停药后4年内，绝大多数在2～3年内。停药后随访观察至少3～5年，凡临床症状消失，脑脊液正常，疗程结束后2年无复发者，方可认为治愈。

【预后】

与下列因素有关：①治疗早晚：治疗愈晚病死率愈高，早期病例无死亡，中期病死率为3.3％，晚期病死率高达24.9％；②年龄：年龄愈小，脑膜炎症发展愈快，愈严重，病死率愈高；③病期和病型：早期、浆液型预后好，晚期、脑膜脑炎型预后差；④结核杆菌耐药性：原发耐药菌株已成为影响结脑预后的重要因素；⑤治疗方法：剂量不足或方法不当时可使病程迁延，易出现并发症。

五、潜伏结核感染

由结核杆菌感染引起的结核菌素试验阳性，除外卡介苗接种后反应，X线胸片或临床无活动性结核病证据者，称潜伏结核感染（1atent tuberculosis infection）。

【诊断要点】

1．病史 多有结核病接触史。

2．临床表现 有或无结核中毒症状，体格检查可无阳性发现。

3．胸部X线检查正常

4．结核菌素试验阳性。

5．应注意与慢性扁桃体炎、反复上呼吸道感染、泌尿道感染及风湿热相鉴别。

【治疗】

下列情况按预防性抗结核感染治疗：①接种过卡介苗，但结核菌素试验最近2年内硬结直

径增大≥l0 mm者可认定为自然感染；②结核菌素试验反应新近由阴性转为阳性的自然感染者；③结核菌素试验呈强阳性反应的婴幼儿和少年；④结核菌素试验阳性并有早期结核中毒症状者；⑤结核菌素试验阳性而同时因其他疾病需用糖皮质激素或其它免疫抑制剂者；⑥结核菌素试验阳性，新患麻疹或百日咳小儿；⑦结核菌素试验阳性的艾滋病毒感染者及艾滋病患儿。

第四节 深部真菌病

深部真菌病（deep mycosis）是指致病真菌不仅侵犯皮肤、黏膜而且侵犯深部组织和内脏所致的疾病。真菌广泛分布于自然界，某些真菌可以感染人体而致病。致病真菌分为两大类：发病原菌：如组织胞浆菌、球孢子菌、新型隐球菌、芽生菌等；②条件致病菌：如念珠菌、曲霉菌、毛霉菌等。深部真菌病常为继发感染，多在糖尿病、血液病、恶性肿瘤、大面积烧伤、严重营养不良或其他慢性消耗性疾病的基础上发病；或长期应用抗生素、糖皮质激素、免疫抑制剂，使机体内菌群失调或抑制了机体的免疫反应而诱发。

一、念珠菌病

念珠菌病（candidiasis）是由数种念珠菌引起的疾病。本病多见于儿童，有的自婴儿发病后，长期潜伏至成人时再发病。最常引起人类疾病的念珠菌是白色念珠菌。

【病因和发病机制】

白色念珠菌（candida albicans）是一种假丝酵母菌，菌体呈圆形或椭圆形，主要以出芽方式繁殖，产生芽生孢子和假菌丝，革兰染色阳性。白色念珠菌属于条件致病菌，通常存在于正常人皮肤、口腔、上呼吸道、肠道及阴道等处，健康小儿带菌率达5%～30%。

【病理】

黏膜病变以其坏死组织、纤维素及大量菌丝和芽孢形成假膜，假膜脱落后形成灶性糜烂和出血性溃疡；内脏病变多呈肉芽肿改变；急性播散型病灶显示灰白色的微小脓肿。病灶内可找到孢子及假菌丝，外围有中性粒细胞及组织细胞浸润。血管受累呈急、慢性血管炎改变，易破裂出血，亦可见微血管内血栓形成。严重免疫抑制者炎症反应较轻，仅见念珠菌及坏死组织形成的脓肿。

【临床表现】

本病分为皮肤黏膜型和内脏型，可呈急性、亚急性或慢性。

1．皮肤黏膜型 好发于新生儿和小婴儿，尤其是肥胖多汗者。在新生儿期肛周、臀部、外阴及腹股沟等尿布包裹区最易受损，其次为腋窝、颈前及下颌。以擦伤最常见，皮肤皱褶处可见皮肤潮红、糜烂，边界清楚，上有灰白色脱屑，周围见散在的红色丘疹、小水泡或脓疱。如患者有免疫缺陷，皮肤可呈肉芽肿改变。播散型可见全身性粟粒疹。黏膜受损以鹅口疮（thrush）最多见，在颊、齿龈、上下腭黏膜表面出现白色乳凝块样物，不易擦去，强行剥削后可见鲜红色糜烂面，可有溢血。免疫功能低下时，黏膜病变由舌、颊粘膜蔓延至咽喉、气管和食道。

2．内脏型

（1）消化道念珠菌病（gastrointestinal candidiasis)：最常见为念珠菌肠炎（candida enteritis)，常伴低热，发生在腹泻病基础上，大便为稀便、水样便或豆腐渣样便，多泡沫，有发酵气味，每日3～10余次不等。严重者形成肠黏膜溃疡而出现便血。

念珠菌食管炎的主要症状为恶心、呕吐、拒食、吞咽困难、流涎。年长儿诉胸骨下疼痛、烧灼感和吞咽痛。X线检查见食道狭窄，蠕动改变。食道镜检可见白色厚膜。

（2）呼吸道念珠菌病（respiratory candidiasis）：以念珠菌性肺炎（candida pneumonia）多见，由于呼吸道柱状上皮细胞具有对真菌侵袭的自然抵抗力，原发念珠菌性肺炎罕见，大多继发于婴幼儿细菌性肺炎、肺结核及血液病，亦可从口腔直接蔓延或经血行播散。起病缓慢，临床表现支气管肺炎的症状体征，常咳出无色胶冻样痰，有时带血丝，可闻及中小湿啰音，当病灶融合时可出现相应肺实变体征。X线表现与支气管肺炎相似。抗生素治疗无效，病程迁延。

（3）泌尿道念珠菌病（urinary tractc andidiasis）：全身性念珠菌病患者常见肾内病灶，多为白色念珠菌经血行播散所致，肾皮质和髓质均可见小脓肿。轻者临床症状不明显，重者出现尿频、尿急、尿痛及肾功能改变。

（4）播散性念珠菌病综合征和念珠菌菌血症（syndrome of disseminated candidiasis and candidemia）：主要表现为长期发热，在原发病（白血病、恶性肿瘤等）的基础上体温增高，症状加重，全身状况恶化。念珠菌播散时往往侵犯多个器官，常见心肌炎、心内膜炎、心包炎、肾小脓肿、脑膜炎、骨髓炎、眼炎和肺炎等。念珠菌心内膜炎的赘生物较大且易发生栓塞；亦可经血行播散引起脑膜炎、脑脓肿，病死率高。

【诊断】

1．真菌检查 因念珠菌是常驻菌，从皮肤、黏膜、痰、粪中等标本中查到孢子不能肯定其为致病菌，必须在镜下见到出芽的酵母菌与假菌丝，结合临床表现才能确定念珠菌病的诊断。①病灶组织或假膜、渗液等标本显微镜检查，可见厚膜孢子及假菌丝，多次显微镜检查阳性有诊断意义；②标本真菌培养1周内出现乳白色光滑菌落，菌落数大于50%，有诊断意义。

2．病理诊断 病理组织中发现真菌和相应病理改变即可确诊。

3．眼底检查 念珠菌菌血症患者视网膜和脉络膜上可见白色云雾状或棉球样病灶。

二、隐球菌

隐球菌病（cryptococcosis）是一种侵袭性真菌疾病，由单相荚膜酵母菌引起。新型隐球菌（cryptococcus neoformans）是人类主要的致病菌，主要侵袭中枢神经系统，亦可播散至肺部、皮肤、黏膜、骨骼、关节和其他内脏，呈急性或慢性病程，各年龄均可发病。

【病因和发病机制】

新型隐球菌属酵母菌，在脑脊液、痰液或病灶组织中呈圆形或半圆形，直径约5～20 μm，四周包围肥厚的胶质样夹膜。该菌以芽生方式繁殖，不生成假菌丝，芽生孢子成熟后脱落成独立个体。新型隐球菌广泛分布于自然界，存在土壤、干鸽粪、水果、蔬菜、正常人皮肤和粪便中。在干燥鸽粪中可以生存达数年之久，是人的主要传染源。一般认为该菌可经呼吸道或皮肤粘膜破损处侵入人体，血行播散至脑、骨骼和皮肤。有80%病例中枢神经系统受损，可能为隐球菌从鼻腔沿嗅神经及淋巴管传至脑膜所致。正常人血清中存在可溶性抗隐球菌因子，而脑脊液中缺乏，故利于隐球菌生长繁殖。

【病理】

基本病理变化有两种：早期为弥漫性浸润渗出性改变，晚期为肉芽肿形成。在早期病灶组织中有大量的新型隐球菌集聚，因菌体周围包绕胶样荚膜，使菌体与组织没有直接接触，故组织炎症反应不明显。肉芽肿的形成常在感染数月后，可见巨细胞、巨噬细胞及成纤维细胞的增生、淋巴细胞和浆细胞浸润，偶见坏死灶及小空洞形成。

【临床表现】

1．隐球菌脑膜炎（cryptococcal meningitis） 是真菌性脑膜炎中最常见的类型。起病缓慢，不同程度发热、阵发性头痛并逐渐加重、恶心、呕吐、晕眩。数周或数月后可出现颅内压增高

症状及脑神经受累的表现，常伴有眼底渗出和视网膜渗出性改变。有时出现精神症状：抑郁、淡漠、易激动。晚期可出现偏瘫、共济失调、抽搐、昏迷等。临床表现颇似结核性脑膜炎，但有间歇性自然缓解。如隐球菌肉芽肿局限于脑某一部位，临床表现与脑脓肿或脑肿瘤相似。

2．肺隐球菌病（pulmonary cryptococcosis） 常与中枢神经系统感染并存，亦可单独发生。起病缓慢，常无明显症状而被忽略。如出现症状，则与肺结核不易区分，如低热、乏力、轻咳、盗汗、体重减轻等，多趋自愈。少数患儿呈急性肺炎的表现，如病灶延及胸膜，可有胸痛和胸膜渗出。X线片可显示单侧或双侧块状病变，亦可为广泛性浸润、支气管周围浸润或栗粒状病变，但不侵犯肺门或纵隔淋巴结。肺部感染一般预后良好。

3．皮肤黏膜隐球菌病（mucocutaneous cryptococcosis） 皮肤黏膜隐球菌病很少单独发生，常为全身性隐球菌病的局部表现，可能由脑膜、肺部或其它病灶播散所致。皮肤隐球菌病主要表现为痤疮样皮疹、丘疹、硬结、肉芽肿等，中央可见坏死，形成溃疡、瘘管等。黏膜损害见于口腔、鼻咽部，表现为结节、溃疡和肉芽肿样，表面覆盖粘性渗出性薄膜。

【诊断】

1．病原体检查 ①墨汁染色法：是迅速、简便、可靠的方法，根据受损部位不同取所需检查的新鲜标本，如脑脊液、痰液、病灶组织或渗液等，置于玻片上，加墨汁1滴，覆以盖玻片在显微镜暗视野下找隐球菌，可见圆形菌体，外周有一圈透明的肥厚荚膜，内有反光孢子但无菌丝。反复多次查找阳性率高。脑脊液应离心后取沉淀涂片。②真菌培养：取标本少许置于沙氏培养基中，在室温或37℃；培养3～4天可见菌落长出。

2．血清学检查 由于病人血清中可测到的抗体不多，因此检测抗体阳性率不高，特异性不强，仅作辅导诊断。通常检测新型隐球菌荚膜多醣体抗原，以乳胶凝集试验（latex agglutination test）灵敏而特异，且有估计预后和疗效的作用。

三、曲霉菌病

曲霉菌病（aspergil1osis）是由致病曲霉菌（aspegillus）所引起的疾病。致病菌主要经呼吸道吸入侵犯肺部，也可侵犯皮肤、黏膜。严重者可发生败血症，使其他组织和系统受累。近年来证明一些曲霉菌可致癌。

【病因和发病机理】

曲霉菌属丝状真菌，是一种常见的条件致病性真菌。引起人类疾病常见的有烟曲霉菌（aspergillus fumigatus）和黄曲霉菌（aspergillus flavus）。曲霉菌广布自然界，存在土壤、空气、植物、野生或家禽动物及飞鸟的皮毛，也常见于农田、马棚、牛栏、谷仓等处。可寄生于正常人的皮肤和上呼吸道，为条件致病菌。过敏体质者吸人曲霉菌孢子可触发IgE介导的变态反应而引起支气管痉挛。

【病理】

曲霉菌最常侵犯支气管和肺，亦可侵犯鼻窦、外耳道、眼和皮肤，或经血行播散至全身各器官。病变早期为弥漫性浸润渗出性改变；晚期为坏死、化脓和肉芽肿形成。病灶内可找到大量菌丝。菌丝穿透血管可引起血管炎、血管周围炎、血栓形成等，血栓形成又使组织缺血、坏死。

【临床表现】

1．肺曲霉菌病（pulmonary aspergillosis） 最常见，多发生在慢性肺部疾病基础上。临床表现分两型：①曲霉菌性支气管-肺炎（aspergil1us bronchopnelmlonia），大量曲霉孢子被吸入后引起急性支气管炎，若菌丝侵袭肺组织，则引起广泛的浸润性肺炎或局限性肉芽肿，也可引

起坏死、化脓，形成多发性小脓肿。急性起病者高热或不规则发热、咳嗽、气促、咯绿色脓痰；慢性者见反复咳嗽、咯血等类似肺结核症状。肺部体征不明显或闻及粗湿啰音。X线检查见肺纹理增多，肺部可见弥漫性斑片状模糊阴影。②球型肺曲霉菌病（aspergilloma，fumgusball）：常在支气管扩张、肺结核等慢性肺疾患基础上发生，菌丝体在肺内空腔中繁殖、聚集并与纤维蛋白和黏膜细胞形成球形肿物，不侵犯其他肺组织。多数患者无症状或表现原发病症状，或出现发热、咳嗽、气急、咯黏液脓痰，其中含绿色颗粒。由于菌球周围有丰富的血管网，可反复咯血。肺部X线检查可见圆形曲霉球悬在空洞内，形成一个新月体透亮区，有重要诊断价值。

2．变态反应性曲霉菌病（allegic asperigillosis） 过敏体质者吸入大量含有曲霉孢子的尘埃，引起过敏性鼻炎、支气管哮喘、支气管炎或变应性肺曲霉菌病。吸入后数小时出现喘息、咳嗽和咳痰，可伴发热。大多数患者3～4天缓解，如再吸入又复发上述症状。痰中可检出大量嗜酸性粒细胞和菌丝，培养见烟熏色曲霉菌生长。血嗜酸性粒细胞增多（$>1.0\times10^9$/L），血清IgE>1000ng/ml。

3．全身性曲霉菌病（disseminated aspergillosis） 多见于原发性或继发性免疫缺陷者。曲霉菌多由肺部病灶进入血循环，播散至全身多个脏器。白血病、恶性淋巴瘤、肿瘤、慢性肺部疾患、长期使用抗生素和皮质激素等是发生本病的诱因。其临床表现随所侵犯的脏器而异，临床上以发热、全身中毒症状和栓塞最常见。累及心内膜、心肌或心包，引起化脓、坏死和肉芽肿。中枢神经系统受累引起脑膜炎和脑脓肿。消化系统以肝受累多见。

【诊断】

1．病原体检查 取自患处的标本作直接涂片或培养，涂片可见菌丝或曲霉菌孢子，培养见曲霉菌生长。曲霉菌是实验室常见的污染菌，必须反复涂片或培养，多次阳性且为同一菌种才有诊断价值。

2．病理组织检查 取受损组织或淋巴结活检，可根据真菌形态确诊。尤其对播散性曲霉菌病，可及时作出诊断。

四、组织胞浆菌病

组织胞浆菌病（histoplasmosis）是由荚膜组织胞浆菌（histoplasma capsulatum）引起的一种传染性很强的真菌病，以侵犯单核-吞噬系统或肺部为主，可累及全身各脏器。本病半数患者为儿童，以6个月～2岁发病率最高，且多为播散型。

【病因和发病机制】

荚膜组织胞浆菌是一种双相真菌，在自然界它以菌丝形态存在，在人体组织由以酵母菌形态出现，以出芽方式繁殖。本菌存在于被蝙蝠、鸡粪等污染的土壤中，在污染严重的地区可见组织胞浆菌病的区域性爆发和流行。人类感染主要途径是经呼吸道吸入小分生孢子，分生孢子芽增殖成酵母菌，引起肺部感染，经血源播散到单核-巨噬细胞系统。细胞介导的免疫使病变局限，形成肉芽肿，不治自愈，临床上无症状。免疫功能低下者的肺部病灶可经淋巴和血液将组织浆菌病播散到全身各脏器，引起广泛病变。目前认为，Ⅱ型和Ⅳ型变态反应参与了肺组织胞浆菌病的发病。

【病理】

典型的病理变化是由于单核-巨噬细胞系统的组织细胞和吞噬细胞吞噬组织胞浆菌以后，在肺、肝、脾、肾上腺和其他组织器官形成上皮样或组织细胞样肉芽肿、结核样结节、干酪样坏死及钙化，部分形成空洞，很少化脓。播散型除软骨和骨皮质外，身体任何部位均可被侵犯。由于组织细胞明显浸润和增生，常破坏受累器官的正常结构，50％患者发生肾上腺皮质坏死。

【临床表现】

一般分为3型：

1．急性肺组织胞浆菌病（acute pulmonary histoplasmosis） 起病急，发热、寒战、咳嗽、胸痛、呼吸困难，肺部可闻湿啰音，肝脾大，胸部X线检查可见弥漫性与多个浸润区，愈后再检查可见多个大小分布一致的钙化点，为本病特征。

2．慢性肺组织胞浆菌病（chronic pulmonary histoplasmosis） 可由肺部原发病灶蔓延而致，亦可为二重感染。病程长，肺部呈进行性、退化性病变。任何年龄均可发病，2岁以下婴幼儿最多见，病死率高。临床表现很似肺结核，发热、咳嗽、盗汗、乏力、体重下降。胸部X线检查见肺实变，以单或双侧上肺多见，部分患者肺尖形成空洞。病情进行性加重，最终导致肺纤维化和肺功能减退。

3．播散性组织胞浆菌病（disseminated histoplasmosis） 多数患者免疫功能低下，1/3发生于婴幼儿。起病急缓不一，全身症状明显，发热、寒战、咳嗽、呼吸困难、头痛、胸痛、腹痛、腹泻、便血、肝脾及淋巴结肿大、低色素性贫血、白细胞减少、血小板减少等。

【诊断】

1．病原体检查 痰、尿、血、骨髓和分泌物涂片或培养分离出组织胞浆菌，或病理切片发现酵母型真菌即可确诊。播散型患者周围血涂片瑞氏染色在中性粒细胞和单核细胞内、外见典型芽状的酵母型组织胞浆菌。

2．组织胞浆菌素皮肤试验 皮试后48～72小时看结果，以红肿硬结≥5 mm为阳性。阳性提示过去或现在有感染。

3．组织胞浆菌抗体检测 ①补体结合试验：检测抗体敏感性高、特异性强，抗体滴度≥1：8或近期升高4倍以上为阳性；②酶联免疫吸附试验：简便易行，滴度≥1：16为阳性。免疫功能低下者可呈假阴性。

4．组织胞浆菌抗原检测 从血清、尿液、脑脊液中可检出抗原，阳性示活动性感染。对免疫缺陷的患者更具诊断意义。

【鉴别诊断】

儿童患者的临床表现颇似血液病或肺结核等，须加鉴别。

五、深部真菌病的治疗

（一）一般治疗

1．积极治疗原发病，去除病因。

2．严格掌握抗生素、糖皮质激素和免疫抑制剂的用药指征，尽可能少用或不用这些药物。

3．加强护理和支持疗法，补充维生素和微量元素。

（二）抗真菌治疗

1．制霉菌素（nystatin）

（1）局部用药：可制成油剂、霜剂、粉剂、溶液等，浓度为含制霉菌素10万U/g或/ml基质，依患者具体情况选用一种剂型局部涂擦，每日2～4次。

（2）口服：肠道念珠菌病可给予制霉菌素口服，新生儿每日20～40万U，2岁以下每日40～80万U，2岁以上每日100～200万U，分3～4次饭前服用，疗程7～10日。口服不易吸收，全部由粪便排出。不良反应有恶心、呕吐、轻泻。

（3）雾化吸入：适用于呼吸系统念珠菌病，制霉菌素5万U溶于2 ml 0.9％氯化钠溶液中雾化吸同样。

2．二性霉素B（amphotericin B） 为多烯类抗生素，与真菌胞膜上的固醇类结合，改变膜的通透性，使菌体破坏，起杀菌作用。是目前治疗隐球菌病、组织胞浆菌病和全身念珠菌病的首选药物，对曲霉菌病效果较差。

（1）静脉滴注：开始宜用小量，每日0.1 mg/kg，如无不良反应，渐增至每日1～1.5 mg/kg，疗程1～3个月。静注时用5％葡萄糖液稀释，浓度不超过0.05～0.1 mg/ml，缓慢静脉滴注，每剂不少于6小时滴完。浓度过高易引起静脉炎，滴速过快可发生抽搐、心律失常、血压骤降，甚至心跳停搏。

（2）椎管内注射或脑室内注射：限于治疗隐球菌性脑膜炎的病情严重或静脉滴注失败的病例。儿童鞘内注射，首次0.01 mg，用蒸馏水（不用0.9％氯化钠溶液）稀释，浓度不超过0.25 mg/ml（偏稀为宜）或将药物与腰穿时引流出的脑脊液3～5 ml混合后一并缓慢注入。以后每日一次，剂量渐增，约1周内增至每次0.1 mg，以后每隔1～3日增加0.1 mg，直至每次0.5 mg为止，不超过0.7 mg。疗程一般约30次，如有副作用可减量或暂停用药。脑脊液内药物过多可引起蛛网膜炎而致脑脊液细胞增多、暂时性神经根炎、感觉消失、尿潴留，甚至瘫痪、抽搐。如及早停药，大多能缓解。

（3）两性霉素的副作用：恶心、呕吐、腹痛、发热、寒战、头痛、头晕、贫血、血小板减少、血栓性静脉炎等，对肝、肾、造血系统有一定毒性。为减轻副作用，可于治疗前半小时及治疗后3小时给阿司匹林，严重者可用静脉滴注氢化可地松或地塞米松。用药期间，应每隔3～7天检查血、尿常规及肝、肾功能，血清肌酐>2.5 mg/dl时用药应减量。尿素氮>40 mg/d应停药，停药2～5周恢复正常，再从小剂量开始给药。注射部位易发生血栓性静脉炎，最初输液部位宜先从四肢远端小静脉开始。

3．5-氟胞嘧啶（5-fluorocytosine） 是一种口服系统性抗真菌化学药物，对隐球菌和白色念珠菌有良好抑制作用。可与两性霉素B合用，治疗全身性隐球菌病，剂量为每日50～150 mg/kg，分4次口服，疗程4～6周。婴儿剂量酌减。口服吸收良好，血清浓度高，脑脊液浓度可达血清的64%～88％。容易产生耐药性，副作用有恶心、呕吐、皮疹、中性粒细胞和血小板减少，肝肾损伤。与两性霉素B合用时可减少耐药性、药量可稍减，毒性反应可减轻，可缩短疗程。

4．克霉唑（clotrimazole） 为广谱抗真菌药，1％～5％软膏皮肤外用。口服易吸收，剂量每日20～60 mg/kg，分3次口服。全身性深部真菌感染可与两性霉菌B联合使用。副作用有胃肠症状、兴奋失眠、荨麻疹、白细胞减少、ALT升高等。

5．酮康唑（Ketoconazole） 合成的口服咪唑类抗真菌药，系咪唑类衍生物。通过抑制麦角甾醇的合成，改变真菌细胞的通透性，导致真菌死亡。抗菌谱广，口服体内吸收良好，毒性反应低，对念珠菌病、曲霉菌病、组织胞浆菌病等疗效均显著。开始剂量：体重30 kg以下者每日口服100 mg；30kg以上每日口服200～400 mg；1～4岁者每日口服50 mg；5～12岁者每日口服100mg。如小儿每日达400 mg高剂量时，可有恶心、呕吐、一过性的低胆固醇血症和肝功能异常。

6．氟康唑（Flueonazoi） 双三唑类抗真菌药，作用机制和抗菌谱与酮康唑相似，体内抗真菌活性比酮康唑强，生物利用度高，口服吸收好，对念珠菌、新型隐球菌等有抑制作用，可在脑脊液中达到有效治疗浓度。>3岁每日3～6 mg/kg，一次顿服或静滴。不良反应有胃肠反应、皮疹，偶致肝功能异常。

（申昆玲）

第五节 寄生虫病

寄生虫病（parasitic disease）是儿童时期最常见的一类病，对儿童的健康危害大，轻者出现消化不良、营养不良等症状，重者可致生长发育障碍，甚至致残或致命。人体寄生虫病对全球人类健康危害严重，广大发展中国家，特别是在热带和亚热带地区寄生虫病广泛流行；在经济发达的国家，寄生虫病也是公共卫生的重要问题。1988～1992年在我国首次寄生虫病流行病学调查显示：我国寄生虫平均感染率为62.5％，0～15岁儿童寄生虫感染率为55.3％～73.3％，说明我国广大儿童的寄生虫病是一个不可忽视的重要问题。

一、蛔虫病

人蛔虫亦称似蛔线虫（ascaris lmnbricoides linnaeus），简称蛔虫，蛔虫病是儿童最常见的寄生虫病之王。成虫寄生于人体小肠，可引起蛔虫病（ascariasis），幼虫能在人体内移行引起内脏移行症（visceral larva migrans）或眼幼虫移行症（ocular larva migrans）。儿童由于食入感染期虫卵而被感染，轻者多无明显症状，异位寄生虫可导致胆道蛔虫病、肠梗阻等严重并发症，严重者可危及生命。

【病因和流行病学】

蛔虫是寄生人体肠道内最大的线虫，成虫呈圆柱形，雌雄异体，形似蚯蚓，活虫略带粉红色或微黄色，体表可见横纹和两条明显的侧线，一般长15～35 cm，横径0.2～0.6 cm。成虫寄生于人体小肠，以肠内容物为食物，雌虫每天排卵可多达20万个，蛔虫卵随粪便排出体外，在适宜环境条件下5～10天发育成熟即具感染性。虫卵被吞食后，虫卵中的胚幼破卵而出，穿入肠壁通过门静脉系统循环移行至肝脏、经右心进入肺泡腔，沿支气管、气管到咽部又重新被吞咽至小肠并逐步发育成熟为成虫。在移行过程中幼虫也可随血流到达其他器官，一般不发育为成虫，但可造成器官损害。成虫有向别处移行和钻孔的习性，在人体不适（发热、胃肠病变等）或大量食入辛辣食物和服用驱虫药物剂量不当等因素刺激下，蛔虫钻入开口肠壁的各种管道，不仅可引起胆道蛔虫症、蛔虫性肠梗阻，而且上窜阻塞气管、支气管造成窒息死亡，亦可能钻入阑尾或胰管引起炎症。自人体感染到雌虫产卵约需60～75天，雌虫寿命为1～2年。

蛔虫病患者是主要的传染源，由于雌虫产卵量极大和虫卵对外界理化因素抵抗力强，虫卵可在泥土中生存数月，在5～10℃可生存2年仍具感染力，因此是构成蛔虫易于传播的重要因素。生吃未经洗净且附有感染性虫卵的食物或用感染的手取食是主要的传染途径，虫卵亦可随飞扬的尘土被吸入咽下。

人蛔虫病是世界上流行最广的人类蠕虫病，据世界卫生组织（WHO）估计全球有13亿患者，儿童特别是学龄前儿童感染率高。世界各地均有蛔虫病，气候温暖的国家感染者更多，在温暖、潮湿和卫生条件差的地区感染较普遍。感染率农村高于城市，儿童高于成年人。蛔虫是国内感染率最高、分布最广的寄生虫，我国约有5.31亿人感染，平均感染率为46.99％，最高达71.12％。由于在全国学校贯彻肠道感染综合防治方案，近年来感染率逐渐下降。

【临床表现】

1．幼虫移行引起的症状 ①蛔虫卵移行至肺可引起蛔幼性肺炎或蛔虫性嗜酸性细胞性肺炎（Loffler综合征），表现为咳嗽、胸闷、血丝痰或哮喘样症状，血嗜酸性细胞增多，肺部体征不明显，X线胸片可见肺部点状、片状或絮状阴影，病灶易变或很快消失。症状1～2周消失。②

严重感染时，幼虫可侵入脑、肝、脾、肾、甲状腺和眼，引起相应的临床表现，如脑膜炎、癫痫、肝大、肝功能异常、视网膜炎、眼睑水肿及尿的改变等。

2．成虫引起的症状 成虫寄生于肠道，以肠腔内半消化食物为食。临床表现与蛔虫多少、寄生部位有关。轻者无任何症状，大量蛔虫感染可引起食欲不振或多食易饥，异食癖；常腹痛，位于脐周，喜按揉，不剧烈；部分病人烦躁易惊或萎靡、磨牙；虫体的异种蛋白可引起荨麻疹、哮喘等过敏症状。感染严重者可造成营养不良，影响生长发育。

3．并发症

（1）胆道蛔虫症（biliary ascariasis）：是最常见的并发症典型表现为阵发性上腹剧烈绞痛、屈体弯腰、哭叫打滚、恶心呕吐，可吐出胆汁或蛔虫。腹部检查无明显阳性体征或仅有右上腹压痛。当发生胆道感染时，患儿可出现发热、黄疸、外周血白细胞数增高。个别患儿，蛔虫可直接窜入肝脏引起出血、脓肿或虫体钙化。其他还包括胆道大出血、胆结石、胆囊破裂、胆汁性腹膜炎、急性出血性坏死性胰腺炎、肠穿孔等。

（2）蛔虫性肠梗阻：多见于10岁以下的儿童，其中2岁以下发病率最高。蛔虫在肠道内扭结成团，部分或完全梗阻肠道，造成肠梗阻，多见于回肠下段。表现为起病急骤、脐周或右下腹阵发性剧痛、呕吐、腹胀、肠鸣音亢进、可见肠型和蠕动波、可扪及条索状包块。腹部X线检查可见肠充气和液平面。

（3）肠穿孔及腹膜炎：表现为突发全腹的剧烈腹痛、伴恶心呕吐、进行性腹胀。体检可见明显的腹膜刺激症状，腹部X线检查见膈下游离气体。

【诊断】

根据临床症状和体征、有排蛔虫或呕吐蛔虫史、粪便涂片查到蛔虫卵即可确诊。血中嗜酸性粒细胞增高，有助于诊断。若出现上述并发症时，需与其它外科急腹症鉴别。

【治疗】

1．驱虫治疗

（1）甲苯咪唑（mebendazole）：是治疗蛔虫病的首选药物之一，为广谱驱虫药，能杀灭蛔虫、蛲虫、钩虫、鞭虫等，可直接抑制虫体对葡萄糖的摄入，导致糖原和ATP生成减少，使虫体无法生存。在杀灭幼虫、抑制虫卵发育方面亦起作用。>2岁驱蛔剂量为每次100 mg，每日2次，或每日200 mg顿服，连服3天。虫卵转阴率90%～100%。不良反应轻微，偶见胃肠不适、腹泻、呕吐、头痛、头昏、皮疹、发热等。复方甲苯咪唑（mebendazole compound）每片含甲苯米唑100 mg和左旋咪唑25 mg，剂量同前。

（2）枸橼酸哌嗪（piperazine citrate）：是安全有效的抗蛔虫和蛲虫药物。能阻断虫体神经肌肉接头冲动传递，使虫体不能吸附在肠壁而随粪便排出体外，麻痹前不兴奋虫体，适用于有并发症的患儿。每日剂量150 mg/kg（最大剂量不超过3 g），睡前顿服，连服2日。不良反应轻微，大量时偶有恶心、呕吐、腹痛、荨麻疹、震颤、共济失调等，肝肾功能不良及癫痫患儿禁用。在肠梗阻时，最好不用，以免引起虫体骚动。

（3）左旋咪唑（1evamisole） 为广谱驱肠虫药，可选择性抑制虫体肌肉中琥珀酸脱氢酶，抑制无氧代谢，减少能量产生，使虫体肌肉麻痹随粪便排出。口服吸收快，由肠道排泄，无蓄积中毒。驱蛔效果达90％～100％，对钩虫、蛲虫也有效，同时也是一种免疫调节剂，可恢复细胞免疫功能。驱蛔虫每日剂量2～3 mg/kg，睡前1次顿服或空腹顿服。不良反应轻微，可有头痛、呕吐、恶心、腹痛，偶有白细胞减少、肝功损害、皮疹等，肝肾功能不良者慎用。

（4）阿苯达唑（albendazole）：是广谱杀虫剂。能抑制虫全对葡萄糖的摄取，导致糖原和ATP生成减少，使虫体失去能量供应而死亡，能有效地抑制虫卵发育。>2岁驱蛔虫剂量为400 mg，

睡前1次顿服。治愈率可达96%，如需要，10日后重复1次。不良反应轻微，可有口干、乏力、头晕、头痛、食欲减退、恶心、腹痛、腹胀等。<2岁者慎用。

2．并发症的治疗

（1）胆道蛔虫症：治疗原则为解痉止痛、驱虫、控制感染及纠正脱水、酸中毒及电解质紊乱。驱虫最好选用虫体肌肉麻痹驱虫药。内科治疗持久不缓解者，必要时可手术治疗。

（2）蛔虫性肠梗阻：不完全性肠梗阻可采用禁食、胃肠减压、输液、解痉、止痛等处理，疼痛缓解后可予驱虫治疗。完全性肠梗阻时应即时手术治疗。

（3）蛔虫性阑尾炎或腹膜炎：一旦诊断明确，应及早手术治疗。

【预防】

普及卫生知识，注意饮食卫生和个人卫生，做好粪便管理，不随地大小便。广泛给易感人群投药以降低感染是比较可行的方法，但蛔虫病的感染率极高，应隔3～6月再给药。最重要的是人粪便必须进行无害化处理后再当肥料使用和提供对污水处理的卫生设施才是长期预防蛔虫病的最有效措施。

二、蛲虫病

蛲虫又称蠕形住肠线虫（enterobius vermicularis）。蛲虫病（enterobiasis）是由蛲虫寄生于体小肠末端、盲肠和结肠所引起的一种常见寄生虫病，尤以幼儿期多见，临床上以夜间会阴部和肛门附近瘙痒为主要特征。

【病因和流行病学】

蛲虫的成虫细小，乳白色线头状。雄虫长0.2～0.5 cm，雌虫长0.8～1.3 cm。虫卵为不对称椭圆形。雌雄异体，交配后雄虫很快死亡。成虫寄生于人体的盲肠、结肠及回肠下段，在人体内存活2～4周，一般不超过2个月。雌虫向肠腔下段移行，当入睡时，肛门括约肌较松弛，雌虫从肛门爬出，受温度、湿度改变和空气的刺激大量排卵，然后大多数死亡，少数雌虫可再进入肛门、阴道、尿道等处，引起异位损害。虫卵在肛周约6小时发育成为感染性卵。当虫卵污染患儿手指，再经口食入而自身感染。感染性卵抵抗力强，在室内一般可存活3周，虫卵可散落在衣裤、被褥或玩具、食物上，经吞食或空气吸入等方式传播。蛲虫患者是唯一的传染源，蛲虫病常在集体儿童机构和家庭中传播流行。

蛲虫感染呈世界性分布，国内感染也较普遍。感染率一般城市高于农村，儿童高于成人，尤其集体生活的儿童感染率更高。据卫生部2001～2004年组织调查全国31省、市36万余名12岁以下儿童的蛲虫感染率为10.28％。

【临床表现】

蛲虫感染可引起局部和全身症状，最常见的症状是肛门和会阴皮肤强烈搔痒和睡眠不安。局部皮肤可因瘙损而发生皮炎和继发感染。全身症状有胃肠激惹现象，如恶心、呕吐、腹痛、腹泻、食欲不振，还可见焦虑不安、失眠、夜惊、易激动、注意力不集中等精神症状。偶可见异位寄生其他器官和侵入临近器官引起阑尾炎、阴道炎、盆腔炎和腹膜炎等。外周血见嗜酸性粒细胞增多。

【诊断】

主要依靠临床症状，同时检出虫卵或成虫以确定诊断。因蛲虫一般不在肠内产卵，故粪便直接涂片法不易检出虫卵，必须从肛门周围皮肤皱襞处直接采集标本。可于这个问题患儿入睡后1～3小时观察肛周皮肤皱襞处有无白色小线虫；或凌晨用透明胶纸紧压肛周部位粘取虫卵，

然后在显微镜下观察虫卵，需多次检查可提高阳性率。

【治疗】

1．驱虫治疗

（1）恩波维铵（pryvinium embonate）：是治疗蛲虫感染的首选药物。可干扰虫体的呼吸酶系统，抑制呼吸，并阻碍虫体对葡萄糖的吸收。剂量为5 mg/kg（最大量0.25 g），睡前1次顿服，2～3周后重复治疗1次。不良反应轻微，少数有腹痛、腹泻、恶心、呕吐，偶有感觉过敏、肌肉痉挛。口服本品可将粪便染成红色，不必惊慌。

（2）噻嘧啶（pyrantel pamoate）：为广谱高效驱虫药，可抑制虫体胆碱脂酶，阻断虫体神经肌肉接头冲动传递，麻痹虫体，安全排出体外。口服很少吸收，剂量为11 mg/kg（最大量1g），睡前1次顿服，2周后重复一次。不良反应轻微，有恶心、眩晕、腹痛等，严重溃疡病者慎用。

（3）甲苯咪唑：剂量和用法与驱蛔虫治疗相同，2周后重复一次。

2．局部用药 每晚睡前清洗会阴和肛周，局部涂擦蛲虫软膏（含百部浸膏30％、甲紫0.2％）杀虫止痒；或用噻嘧啶栓剂塞肛，连用3～5日。

【预防】

应强调预防为主，培养良好的卫生习惯，饭前便后洗手，纠正吮手指习惯，勤剪指甲，婴幼儿尽早穿满裆裤，玩具、用具、被褥要常清洗和消毒。

三、钩虫病

钩虫病（ancylostomiasis）是由钩虫科线虫（hookworm）寄生于人体小肠所引起的肠道寄生虫病。寄生人体的钩虫常见有十二指肠钩虫（ancylostoma duodenale）和美洲钩虫（necator americanus）。轻者无症状表现，仅在粪便中发现虫卵，称为钩虫感染（hookworm infection）。典型临床主要表现为贫血、营养不良有、胃肠功能失调，严重者可出现心功能不全和生长发育障碍。

【病因和流行病学】

成虫半透明灰白或米黄色，长约1 cm，雌雄异体，寄生人体小肠上段，以其口囊咬吸在肠黏膜上，摄取血液及组织液。成熟十二指肠钩虫雌虫每日产卵1～3万个；美洲钩虫雌虫每日产卵5千～1万个。虫卵随粪便排出，在温暖、潮湿、疏松土壤中孵育成杆状蚴，1～2周后，经过二次蜕皮后发育为丝状蚴，即感染期蚴。丝状蚴通过毛囊、汗腺口或皮肤破损处钻入人体进入血管和淋巴管，随血流经右心至肺，穿过肺微血管进入肺泡，向上移行至咽部，被吞咽入胃，达小肠发育为成虫。成虫在人体内一般可存活3年左右，最长可达15年。

钩虫病患者为主要传染源。皮肤接触污染的土壤是主要感染途径；进食污染的食物也是感染途径之一；婴幼儿可因尿布、衣服晾晒在或落在沾有钩蚴的土地上而感染，或因坐地、爬玩而感染。

钩虫感染遍及全球，全世界约有十亿人感染钩虫，在热带、亚热带和温带地区特别流行。在我国除少数气候干燥、寒冷的地区外，其他地区均有不同程度流行，尤以四川、浙江、湖南、福建、广东、广西等地较严重。在华东和华北地区以十二指肠钩虫为主；在华南和西南地区以美洲钩虫为主，大多属混合感染。其感染率农村高于城市，成人高于儿童。小儿年龄越大，感染率越高。1988～1992年全国寄生虫病调查结果显示，我国钩虫感染人数为19405万人，平均感染率为17.17％，海南省的感染率（60.90％）为最高，其次是四川省（40.88％）和广西壮族自治区（37.85％），东北、华北和西北10个省（区）的感染率则低于1％。5～9岁感染率最高，

10～14岁次之。

【临床表现】

1．钩蚴引起的症状

（1）钩蚴皮炎：钩蚴入侵的皮肤处多见于足趾或手指间皮肤较薄处及其他部位暴露的皮肤，可出现红色点状丘疹或小疱疹，烧灼、针刺感，奇痒，数日内消失。搔抓破后常继发感染，形成脓疱，并可引起发热和淋巴结炎。

（2）呼吸道症状：感染后3～7天，幼虫移行至肺部可引起喉咙发痒、咳嗽、发热、气急和哮喘，痰中带血丝，甚至大咯血。胸部X线检查见肺有短暂的浸润性病变，血嗜酸性粒细胞增高。病程数日或数周。

2．成虫引起的症状

（1）贫血：失血性贫血是主要症状。表现为不同程度的贫血、皮肤黏膜苍白、乏力、眩晕，影响小儿体格和智能发育。严重者可发生贫血性心脏病。

（2）消化道症状：初期表现为贪食、多食易饥，但体重下降。后期食欲下降，胃肠功能紊乱，腹胀不适，异食癖，营养不良等，严重者可出现便血。

3．婴儿钩虫病 临床表现为急性便血性腹泻，大便黑色或柏油样，胃肠功能紊乱，面色苍白，发热，心尖部可闻及明显收缩期杂音，肝、脾大，生长发育迟缓，严重贫血，血红蛋白低于50 g/L，大多数患儿周围血白细胞总数增高，嗜酸性粒细胞显著增高，有时呈类白血病样反应。发病多在5～12个月，亦有新生儿发病的报道。

【诊断】

1．病原体检查 在流行区，对有贫血、胃肠功能紊乱、异食癖、营养不良、生长发育迟缓的小儿应考虑钩虫病的可能。粪便中检出钩虫卵或孵化出钩蚴是确诊的依据。粪便饱和盐水漂浮法简便易行，钩蚴培养法检出率较高。当咳嗽时痰中找到钩蚴亦可确诊。

2．免疫学诊断 适用于大规模普查。用钩虫虫体抗原作皮内试验，阳性者结合流行病学及临床特点，可作出早期诊断。

【治疗】

1．驱虫治疗

（1）苯咪唑类药物：是一类广谱驱肠线虫药，具有杀死成虫和虫卵的作用。因为能选择性及不可逆地抑制寄生虫对葡萄糖的利用，影响虫体能量代谢而达驱虫。但驱虫作用缓慢，治疗3～4天才排钩虫。常用剂型有：①甲苯咪唑（甲苯达唑 mebendazole）：不分年龄，每次l00 mg，每日2次，连服3日。治愈率90％以上。不良反应轻而短暂，少数患者有头痛、恶心、腹痛等，严重肝、肾疾病者及<2岁儿童慎用。②阿苯达唑（albendazole）：单剂有效，儿童每次200 mg，10日后可重复1次。严重心功能不全、活动性溃疡病患儿慎用。

（2）噻嘧啶（pyrantel pamoate）：也是一类广谱驱肠线虫药，为神经肌肉阻滞剂，使虫体麻痹而被排出。驱虫作用快，服药1～2天排虫。常用剂量为ll mg/kg（最大量1g），每日1次，睡前顿服，连服2～3日。不良反应轻，可见恶心、腹痛、腹泻等。急性肝炎、肾炎者暂缓给药。

（3）左旋咪唑（levamisole）：是广谱驱肠虫药，剂量为1.5～2.5 mg/kg，睡前1次顿服，连用3日为1疗程。不良反应轻微，可有头痛、呕吐、恶心、腹痛，偶有白细胞减少、肝功能损害、皮疹等。肝肾功能不良者慎用。

（4）联合用药：左旋咪唑和噻嘧啶合用可提高疗效。

2．对症治疗 纠正贫血，给予铁剂和充足营养，严重贫血可少量多次输血。

【预防】

加强卫生宣教，注意饮食卫生，不随地大便，加强粪便无害化管理。在流行区定期普查普治，加强个人防护，防止感染。

四、绦虫病

绦虫病（taeniasis）是由绦虫寄生在人体肠道引起的疾病。常见的有猪肉绦虫病和牛肉绦虫病，系因进食含有活囊尾蚴的猪或牛肉而感染。

【病因和流行病学】

绦虫（cestode）又称带虫（tapeworm），成虫扁长如带，长约2～4米，乳白色，雌雄同体。成虫寄生于人的小肠，虫体分为头节、颈节和体节三部分：头节具有固着器官，上有吸盘和小钩；颈节具有生发功能，节片由此向后连续长出；体节靠近颈节部分因其生殖器官未发育成熟称为未成熟节，中间部分节片因生殖器官发育成熟称为成熟节，后部节片中存满虫卵称为孕节，每一孕节含卵8～10万个，虫卵或孕节随粪便排出体外，当虫卵被猪、牛等中间宿主吞食后，卵内的六钩蚴虫在其小肠内逸出，钻进肠壁血管或淋巴管随血循环或淋巴循环到达全身，主要在运动较多的肌肉组织中发育成为囊尾蚴，囊尾蚴如黄豆大，内有白色米粒大小的囊尾蚴头节。这种含有囊尾蚴的肉（俗称米猪肉）未经煮熟而被人摄入后即在人小肠中经8~10周发育为成虫而致病，成虫的寿命可达20~30年甚至更长。人也可以成为猪绦虫的中间宿主，即由于吞食的虫卵或孕节在人体内发育成囊尾蚴所造成，称为囊虫病（cysticercosis），但这种囊尾蚴不能在人体内继续发育为成虫。寄生在人体的绦虫除大量掠夺宿主的营养外，其固有器官吸盘和小钩对宿主肠道亦造成机械刺激和损伤。囊尾蚴在人体内寄生的危害性比绦虫病更大，其程度因囊尾蚴寄生的部位和数量而不同，其中以脑囊虫病最为严重。大脑是对包囊最敏感的器官，当入侵大脑的包囊数目多或其阻塞脑脊液通路时，可导致症状。包囊死亡分解后，可完全吸收或钙化。

绦虫在全世界分布很广，主要流行于欧洲、中美一些国家和东南亚等国。我国各地都有发生，以内蒙古、新疆、广西、云南、贵州、四川等地多见，呈局限性流行或散在发生。在绦虫病严重流行区，居民有爱吃生的或未煮熟的猪、牛肉的习惯，对本病的传播起着决定的作用。生熟砧板不分，易造成交叉污染，而致感染。患者农村多于城市，以青壮年为主，儿童受感染者也不少。

【临床表现】

1．成虫引起的症状 潜伏期为2～3个月。除腹部隐痛不适外，很少引起临床症状。腹痛常见于中上腹和脐部，进食后腹痛缓解为其特征。部分患儿有恶心、呕吐、腹泻、食欲不振或亢进、体重减轻等。大便中常发现白色虫体节片，单节脱落后可由肛门排出。

2．囊尾蚴寄生的症状 因囊虫寄生的部位和数量不同而异。

（1）脑囊虫病：症状极为复杂多样，从全无症状到猝死不等。癫痫发作、颅内压增高和精神症状是三个主要的症状。癫痫发作是最突出的症状，一般在排虫后或皮下囊包出现后半年发生。脑脊液检查多属正常，少数病例可见细胞数和蛋白轻度增加。依颅内寄生部位分为皮质型、脑室型、蛛网膜下腔型或颅底型而产生不同的症状，也有寄生于椎管压迫脊髓。

（2）肌肉与皮下组织囊虫病：囊尾蚴侵入肌肉和皮下组织形成圆形或卵圆形结节，微隆起或不隆起于皮肤表面，如黄豆或蚕豆，大小相近，硬而有压痛，无炎症反应，1～2个至数百、数千个不等，头和躯干较多，蚴虫死后发生钙化。

（3）眼囊虫病 可发生在眼的任何部位，以玻璃体和视网膜多见。轻者视力障得，重者失

明，以单眼多见。眼底检查在玻璃体内可见大小不等的圆形或椭圆形的浅灰色包囊，周围有红晕光环。

【诊断】

有生食或进食半生的牛、猪肉史、粪便中发现绦虫节片或检出虫卵即可确诊。囊虫病的诊断依据：①有猪绦虫病史，或粪便中发现有绦虫卵或妊娠节片。②皮下结节病理检查见囊尾蚴。③免疫试验：囊尾蚴抗原皮内试验、补体结合试验阳性，用囊尾蚴液纯化抗原与患者脑脊液进行酶联免疫吸附试验阳性。免疫金银染色（ICSS）是近10年发展的高敏感性的方法。④患病时间较长者，囊虫已死亡而有钙化者（一般显示需5年以上），可拍头颅X线片或脑室造影帮助诊断。

【治疗】

1．驱虫治疗

（1）氯硝柳胺（niclosamide）：能破坏绦虫的角质膜，麻痹神经和肌肉，可杀死绦虫的头节和近段虫体。口服不吸收，在肠中保持高浓度。小儿剂量：<2岁每日0.5 g，2～6岁每日1 g，>6岁每日2 g，均分2次空腹服，2次之间间隔1小时，服时应将药片嚼碎后吞下，服后2小时服硫酸镁导泻。不良反应轻微，偶有乏力、头晕、胸闷、胃及腹部不适等。

（2）吡喹酮（praziquantel）：为广谱抗寄生虫药，能作用于虫体细胞膜，影响其通透性，虫体表破坏而挛缩。治疗绦虫病和囊虫病均有效，疗效高于氯硝柳胺，治愈率可达100%。剂量为10～15 mg/kg，顿服。治疗脑囊虫病的剂量为每日20 mg/kg，分3次服，9日为一疗程，疗程间隔3～4个月。不良反应较重，因虫体死亡炎症反应和水肿加重，颅内高压明显，原有症状加重，个别病例因脑疝死亡，应高度警惕，必要时先降颅内压再行治疗。

（3）槟榔与南瓜子：槟榔对绦虫的头节和前段有瘫痪作用，南瓜子能使绦虫中、后段节片瘫痪，两者合用可使虫体变软，借小肠蠕动作用随粪便排出体外。驱猪绦虫服35％槟榔煎剂60～120 ml，清晨顿服。驱牛绦虫，先服炒熟去皮南瓜子30～60 g，2小时后服上述剂量的槟榔煎剂。一般服药后3小时内有完整虫体排出。槟榔有胃肠痉挛和剧烈腹痛的不良反应，婴儿不宜应用。

驱绦虫治疗的注意事项：①无论用何种药驱绦虫，在排便时应坐在盛有水温与体温相同的生理盐水中排便，以免虫体遇冷收缩而不能全部排出；②留集24小时粪便寻找头节；③治疗3个月无虫卵和节片排出为治愈。

2．手术治疗 眼囊虫病目前主张以手术摘除为宜。颅内、尤其脑室内单个囊虫也可行手术治疗。

【预防】

加强卫生宣传，改变不良饮食习惯。加强肉品检验，不吃生的或未煮熟的猪、牛肉；仔细清洗蔬菜与水果；应区分生、熟食品的砧板；彻底治疗绦虫病患者。

（孙立荣）

第十一章 消化系统疾病

第一节 儿童消化系统解剖生理特点

（一）口腔

口腔是消化道的起端，具有吸吮、吞咽、咀嚼、消化、味觉、感觉和语言等功能。足月新生儿出生时已具有较好的吸吮及吞咽功能。新生儿及婴幼儿口腔黏膜薄嫩，血管丰富，唾液腺不够发达，口腔黏膜干燥，因此易受损伤和局部感染；3～4个月时唾液分泌开始增加。婴儿口底浅，尚不能及时吞咽所分泌的全部唾液，因此常发生生理性流涎。

（二）食管

食管长度在新生儿为8～10 cm，1岁时为12 cm，5岁时为16 cm，学龄儿童为20～25 cm，成人为25～30 cm。食管横径婴儿为0.6～0.8 cm，幼儿为1 cm，学龄儿童为1.2～1.5 cm。食管pH通常在5.0～6.8。新生儿和婴儿的食管呈漏斗状，黏膜纤弱、腺体缺乏、弹力组织及肌层尚不发达，食管下段括约肌发育不成熟，控制能力差，常发生胃食管反流。婴儿吸奶时常吞咽过多空气，易发生溢奶。

（三）胃

胃容量在新生儿约为30～60 ml，1～3个月时90～150 ml，1岁时250～300 ml，5岁时为700 ml～850 ml，成人约为2000 ml，故年龄愈小每天喂养的次数愈多。哺乳后不久幽门即开放，胃内容物陆续进入十二指肠，故实际胃容量不受上述容量限制。婴儿胃略呈水平位，当开始行走时其位置变为垂直。胃平滑肌发育尚未完善，在充满液体食物后易使胃扩张。由于贲门和胃底部肌张力低，幽门括约肌发育较好，故易发生幽门痉挛而出现呕吐。胃排空时间随食物种类不同而异，稠厚含凝乳块的乳汁排空慢；水的排空时间为1.5～2小时；母乳2～3小时；牛乳3～4小时；早产儿胃排空更慢，易发生胃潴留。

（四）肠

儿童肠管相对比成人长，一般为身长的5～7倍，或为坐高的10倍。小肠的主要功能包括运动（蠕动、摆动、分节运动）、消化、吸收及免疫保护。大肠的主要功能是贮存食物残渣、进一步吸收水分以及形成粪便。婴幼儿肠黏膜肌层发育差，肠系膜柔软而长，结肠无明显结肠带与脂肪垂，升结肠与后壁固定差，易发生肠扭转和肠套叠。肠壁薄、通透性高，屏障功能差，加之口服耐受机制尚不完善，肠内毒素、消化不全产物和过敏原等可经肠黏膜进入体内，引起全身感染和变态反应性疾病。由于婴幼儿大脑皮质功能发育不完善，进食时常引起胃-结肠反射，产生便意，所以大便次数多于成人。

（五）肝

年龄愈小，肝脏相对愈大。婴儿肝脏结缔组织发育较差，肝细胞再生能力强，不易发生肝硬变，但易受各种不利因素的影响，如缺氧、感染、药物中毒等均可使肝细胞发生肿胀、脂肪浸润、变性、坏死、纤维增生而肿大，影响其正常功能。婴儿时期胆汁分泌较少，故对脂肪的消化，吸收功能较差。

（六）胰腺

出生后3～4个月时胰腺发育较快，胰液分泌量也随之增多。出生后一年，胰腺外分泌部生长迅速，为出生时的3倍。胰液分泌量随年龄生长而增加，至成人每日可分泌1～2L。酶类出现的顺序为：胰蛋白酶最先，而后是糜蛋白酶、羧基肽酶、脂肪酶，最后是淀粉酶。新生儿胰液

所含脂肪酶活性不高，直到2～3岁时才接近成人水平。婴幼儿时期胰液及其消化酶的分泌易受炎热天气和各种疾病的影响而被抑制，容易发生消化不良。

（七）肠道细菌

在母体内，胎儿肠道是无菌的，生后数小时细菌即侵入肠道，主要分布在结肠和直肠。肠道菌群受食物成分影响，单纯母乳喂养儿以双岐杆菌占绝对优势，人工喂养和混合喂养儿肠内的大肠杆菌、嗜酸杆菌、双歧杆菌及肠球菌所占比例几乎相等。正常肠道常驻菌群对侵入肠道的致病菌有一定的拮抗作用。婴幼儿肠道正常菌群脆弱，易受许多内外界因素影响而致菌群失调，引起消化功能紊乱。

（八）健康儿童粪便

食物进入消化道至粪便排出时间因年龄而异：母乳喂养的婴儿平均为13小时，人工喂养者平均为15小时，成人平均为18～24小时。

1．母乳喂养儿粪便 为黄色或金黄色，多为均匀膏状或带少许黄色粪便颗粒，或较稀薄，绿色、不臭，呈酸性反应（pH 4.7～5.1）。平均每日排便2～4次，一般在添加辅食后次数即减少。

2．人工喂养儿粪便 人工喂养的婴儿粪便为淡黄色或灰黄色，较干稠，呈中性或碱性反应（pH 6～8）。因牛乳含蛋白质较多，粪便有明显的蛋白质分解产物的臭味，有时可混有白色酪蛋白凝块。大便1～2次/日，易发生便秘。

3．混合喂养儿粪便 喂食母乳加牛乳的婴儿粪便与喂牛乳者相似，但较软、黄色，添加淀粉类食物可使大便增多，稠度稍减，稍呈暗褐色，臭味加重。添加各类蔬菜、水果等辅食时大便外观与成人粪便相似，初加菜泥时，常有小量绿色便排出。便次每日1次左右。

第二节 口　　炎

口炎（stomatitis）是指口腔黏膜由于各种感染引起的炎症，若病变限于局部如舌、齿龈、口角亦可称为舌炎，齿龈炎或口角炎等。本病多见于婴幼儿。可单独发生，亦可继发于全身疾病如急性感染、腹泻、营养不良、久病体弱和维生素B、C缺乏等。感染常由病毒、真菌、细菌引起。不注意食具及口腔卫生或各种疾病导致机体抵抗力下降等因素均可导致口炎的发生。目前细菌感染性口炎已经很少见，病毒及真菌感染所致的口炎仍经常见到。

一、鹅口疮

鹅口疮（thrush，oral candidiasis）又称雪口病，为白色念珠菌感染在黏膜表面形成白色斑膜的疾病。多见于新生儿和婴幼儿，营养不良、腹泻、长期使用广谱抗生素或类固醇激素的患儿常有此症。新生儿多由产道感染或因哺乳时奶头不洁及从污染的乳具获得感染。

【临床表现】

口腔黏膜表面覆盖白色乳凝块样小点或小片状物，可逐渐融合成大片，不易擦去，周围无炎症反应，强行剥离后局部黏膜潮红、粗糙、可有溢血，不痛，不流涎，一般不影响吃奶，无全身症状。重症则整个口腔均被白色斑膜覆盖，甚至可蔓延到咽、喉头、食管、气管、肺等处，此时可危及生命。重症患儿可伴低热、拒食、吞咽困难。取白膜少许放玻片上加10％氢氧化钠一滴，在显微镜下可见真菌的菌丝和孢子。使用抗生素可加重病情，促其蔓延。

【治疗】

一般不需静脉或口服抗真菌药物。可用2%碳酸氢钠溶液于哺乳前后清洁口腔，或局部涂抹10万～20万U/ml制霉菌素鱼肝油混悬溶液，每日2～3次。亦可口服肠道微生态制剂，纠正肠道菌群失调，抑制真菌生长。预防应注意哺乳卫生，加强营养，适当增加维生素B_2和C。

二、疱疹性口腔炎

疱疹性口腔炎（herptic stomatitis）为单纯疱疹病毒Ⅰ型感染所致。多见于1～3岁小儿，发病无明显季节差异。从患者的唾液、皮肤病变和大小便中均能分离出病毒。

【临床表现】

常好发于颊粘膜、齿龈、舌、唇内和唇黏膜及邻近口周皮肤。起病时发热可达38～40℃，1～2天后，上述各部位口腔黏膜出现单个或成簇的小疱疹，直径约2mm，周围有红晕，迅速破溃后形成溃疡，有黄白色纤维素性分泌物覆盖，多个溃疡可融合成不规则的大溃疡，有时累及软腭、舌和咽部。由于疼痛剧烈，患儿可表现拒食、流涎、烦躁，常因拒食啼哭才被发现。所属淋巴结常肿大和压痛，可持续2～3周。体温在3～5天后恢复正常，病程约1～2周。

本病应与疱疹性咽峡炎鉴别，后者大都由柯萨奇病毒所引起，疱疹主要发生在咽部和软腭，有时见于舌但不累及齿龈和颊黏膜，此点与疱疹性口腔炎迥异。

【治疗】

保持口腔清洁，多饮水。食物以微温或凉的流质为宜，避免刺激性食物。局部可涂疱疹净抑制病毒，亦可喷撒西瓜霜，锡类散等。为预防继发感染可涂2.5%～5%金霉素鱼肝油。疼痛严重者可在餐前用2%利多卡因涂抹局部。发热时可用退热剂，可行全身抗病毒治疗，抗生素不能缩短病程，仅用于有继发感染者。

第三节 胃食管反流及反流性食管炎

胃食管反流（gastroesophageal reflux，GER）是指胃内容物，包括从十二指肠流入胃的胆盐和胰酶等反流入食管甚至口咽部，分生理性和病理性两种。生理情况下，由于小婴儿食管下端括约肌（lower esophageal sphincter，LES）发育不成熟或神经肌肉协调功能差，可出现反流，往往出现于日间餐时或餐后，又称“溢乳”。病理性反流是由于LES的功能障碍和（或）与其功能有关的组织结构异常，以致LES压力低下而出现的反流，常常发生于睡眠、仰卧位及空腹时，引起一系列临床症状和并发症，即胃食管反流病（GERD）。随着直立体位时间和固体饮食的增多，到2岁时60%患儿的症状可自行缓解，部分患儿症状可持续到4岁以后。脑性瘫痪、21-三体综合征以及其他原因引起的发育迟缓患儿，有较高的GER发生率。

【病因和发病机制】

1．抗反流屏障功能低下 ①LES压力降低：是引起GER的主要原因。正常吞咽时LES反射性松弛，压力下降，通过食管蠕动推动食物进入胃内，然后压力又恢复到正常水平，并出现一个反应性的压力增高以防止食物反流。当胃内压和腹内压升高时，LES会发生反应性主动收缩使其压力超过增高的胃内压，起到抗反流作用。如因某种因素使上述正常功能发生紊乱时，LES短暂性松弛即可导致胃内容物反流入食管。②LES周围组织薄弱或缺陷：例如缺少腹腔段食管，致使腹内压增高时不能将其传导至LES使之收缩达到抗反流的作用；小婴儿食管角（由食管和胃贲门形成的夹角，即His角）较大（正常为30°～50°）；膈肌食管裂孔钳夹作用减弱；膈食管韧带和食管下端黏膜瓣解剖结构存在器质性或功能性病变时以及胃内压、腹内压增高等，均

可破坏正常的抗反流功能。

2．食管廓清能力降低 正常情况下，食管廓清能力是依靠食管的推动性蠕动、唾液的冲洗、对酸的中和作用、食丸的重力和食管黏膜细胞分泌的碳酸氢盐等多种因素完成其对反流物的清除作用，以缩短反流物和食管黏膜的接触时间。当食管蠕动减弱或消失、或出现病理性蠕动时，食管清除反流物的能力下降，这样就延长了有害的反流物质在食管内停留时间，增加了对黏膜的损伤。

3．食管黏膜的屏障功能破坏 屏障作用是由黏液层、细胞内的缓冲液、细胞代谢及血液供应共同构成。反流物中的某些物质，如胃酸、胃蛋白酶、以及从十二指肠反流入胃的胆盐和胰酶使食管黏膜的屏障功能受损，引起食管黏膜炎症。

4．胃、十二指肠功能失常 胃排空能力低下，使胃内容物及其压力增加，当胃内压增高超过LES压力时可使LES开放。胃容量增加又导致胃扩张，致使贲门食管段缩短，使其抗反流屏障功能降低。十二指肠病变时，幽门括约肌关闭不全则导致十二指肠胃反流。

【临床表现】

食管上皮细胞暴露于反流的胃内容物中，是产生症状和体征的原因。

1．呕吐 新生儿和婴幼儿以呕吐为主要表现。85％患儿于生后第1周即出现呕吐，另有10％患儿于生后6周内出现症状。呕吐程度轻重不一，多数发生在进食后，有时在夜间或空腹时，严重者呈喷射状。呕吐物为胃内容物，有时含少量胆汁，也有表现为漾奶、反刍或吐泡沫。年长儿以反胃、反酸、嗳气等症状多见。

2．反流性食管炎 常见症状有①烧灼感：见于有表达能力的年长儿，位于胸骨下端，饮用酸性饮料可使症状加重，服用抗酸剂症状减轻；②咽下疼痛：婴幼儿表现为喂奶困难、烦躁、拒食，年长儿诉咽下疼痛，如并发食管狭窄则出现严重呕吐和持续性咽下困难；③呕血和便血：食管炎严重者可发生糜烂或溃疡，出现呕血或黑便症状。严重的反流性食管炎可发生缺铁性贫血。

3．Barrette食管 由于慢性GER，食管下端的鳞状上皮被增生的柱状上皮所替代，抗酸能力增强，但更易发生食管溃疡、狭窄和腺癌。溃疡较深者可发生食管气管瘘。

4．其他全身症状

（1）与GERD相关的呼吸系统疾病：①呼吸道感染：反流物直接或间接引发反复呼吸道感染。②哮喘：反流物刺激食管黏膜感受器反射性地引起支气管痉挛而出现哮喘。部分病例发病早、抗哮喘治疗无效，无特异体质家族史的哮喘患儿更可能由GERD引起。③窒息和呼吸暂停：多见于小婴儿和早产儿，为反流物所致喉痉挛或呼吸道梗阻，表现为面色青紫或苍白、心动过缓，甚至发生婴儿猝死综合征。

（2）营养不良：见于80％左右的患儿，因呕吐及食管炎引起喂养困难而摄食不足所致。主要表现为体重不增和生长发育迟缓，贫血等。

（3）其它：如声音嘶哑、中耳炎、鼻窦炎、反复口腔溃疡、龋齿等。部分患儿可出现精神经症状：①Sandifer综合征：是指病理性GER患儿呈现类似斜颈样的一种特殊“公鸡头样”的姿势。此为一种保护性机制，以期保持气道通畅或减轻酸反流所致的疼痛，同时伴有杵状指、蛋白丢失性肠病及贫血；②婴儿哭吵综合征：表现为易激惹、夜惊、进食时哭闹等。

【辅助检查】

1，食管钡餐造影 可对食管的形态、运动状况、造影剂的反流和食管与胃连接部的组织结构做出判断，并能观察到是否存在食管裂孔疝等先天性疾患，以及严重病例的食管黏膜炎症改变。

2．食管pH值动态监测 经鼻孔将微电极放置在食管括约肌的上方，24小时连续监测食管下端pH，如有酸性GER发生则pH下降。通过计算机软件分析可反映GER的发生频率、时间、反流物在食管内停留的状况，以及反流与起居活动、临床症状之间的关系，借助一些评分标准，可区分生理性和病理性反流，是目前最可靠的诊断方法。特别是用于一些症状不典型的患者，或用于查找一些症状如咳嗽、哽噎、喘鸣、阵发性青紫、呼吸暂停的原因。还可以同时检测食管、胃双pH，以判断食管下端pH不下降时的碱性GER和十二指肠胃食管反流。

3．食管胆汁反流动态监测 应用便携式24小时胆红素监测仪，将监测探头经鼻孔插入，放置在食管括约肌上方，监测24小时，记录平卧、直立、进餐及症状发生的时间，数据以专用软件处理，可提示胆汁反流至食管的十二指肠胃食管反流（DGER）。

4．食管动力功能检查 应用低顺应性灌注导管系统和腔内微型传感器导管系统等测压设备，了解食管运动情况及LES功能。对于LES压力正常患儿应连续测压，动态观察食管运动功能。

5．食管内镜检查及黏膜活体组织检查 内镜下食管病变诊断及分级标准：0级：食管黏膜无异常；Ⅰ级：黏膜点状或条状发红、糜烂、无融合现象；Ⅱ级：黏膜有条状发红、糜烂并有融合，但小于周径的2/3；Ⅲ级：黏膜广泛发红、糜烂融合成全周性或有溃疡。食管黏膜组织活体组织检查可发现鳞状上皮基底层细胞增生、肥厚，黏膜固有层乳头延伸进入上皮，上皮层内中性粒细胞、嗜酸性粒细胞、淋巴细胞浸润，甚至黏膜糜烂、溃疡、肉芽组织形成和（或）纤维化。Barrette食管：鳞状上皮由柱状上皮取代，出现杯状细胞的肠上皮化生。

6．胃-食管放射性核素烁扫描 口服或胃管内注入含有^{99m}Tc标记的液体，应用γ照像机测定食管反流量，可了解食管运动功能，明确呼吸道症状与GER的关系。

【诊断】

GER临床表现复杂且缺乏特异性，仅凭临床症状有时难以与其它引起呕吐的疾病相鉴别，即使是GER也难以区分是生理性或病理性。凡临床发现不明原因反复呕吐、咽下困难、反复发作的慢性呼吸道感染、难治性哮喘、生长发育迟缓、营养不良、贫血、反复出现窒息、呼吸暂停等症状时都应考虑到GER的可能，针对不同情况，选择必要的辅助检查以明确诊断。

【鉴别诊断】

1．贲门失弛缓症 又称贲门痉挛，是指食管下括约肌松弛障碍导致的食管功能性梗阻。婴幼儿表现喂养困难、呕吐，重症可伴有营养不良、生长发育迟缓。年长儿诉胸痛和烧灼感、反胃。通过X线钡餐造影、内镜和食管测压等可确诊。

2．以呕吐为主要表现的新生儿、小婴儿应排除消化道器质性病变，如：先天性幽门肥厚性狭窄、胃扭转、肠旋转不良、环状胰腺、胎粪性腹膜炎等。

3．对反流性食管炎伴并发症的患儿，必须排除由于物理性、化学性、生物性等致病因素所引起组织损伤而出现的类似症状。

【治疗】

凡诊断为GER的患儿，特别是有合并症或影响生长发育者必须及时进行治疗。包括体位、饮食、药物和手术治疗。

1．体位治疗 将床头抬高30°，小婴儿的最佳体位为前倾俯卧位，但为防止婴儿猝死综合征的发生，睡眠时应采取仰卧位及左侧卧位。儿童在清醒状态下最佳体位为直立位和坐位，睡眠时保持左侧卧位及上体抬高，减少反流频率及反流物误吸。

2．饮食疗法 以稠厚饮食为主，少量多餐，婴儿增加喂奶次数，缩短喂奶间隔时间，人工喂养儿可在牛奶中加入淀粉类或进食谷类食品。年长儿亦应少量多餐，以高蛋白低脂肪饮食为

主，睡前2小时不予进食，保持胃处于非充盈状态，避免食用降低LES张力和增加胃酸分泌的食物，如酸性饮料、碳酸及咖啡因饮料、高脂饮食、巧克力和辛辣食品。此外，应控制肥胖，避免被动吸烟。

3．药物治疗 主要基于降低胃内容物酸度和促进上消化道动力，包括促胃肠动力药、抗酸或抑酸药、黏膜保护剂等，但使用时应注意药物的适用年龄及不良反应。

（1）促胃肠动力药（prokinetic agents）：能提高LES张力，增加食管和胃蠕动，提高食管廓清能力，促进胃排空，从而减少反流和反流物在食管内的停留时间。①多巴胺受体拮抗剂：多潘立酮（domperidone，吗叮啉）为选择性、周围性多巴胺D_2受体拮抗剂，可增强食管蠕动和LES张力，增加胃窦和十二指肠运动，协调幽门收缩，促进胃排空。常用剂量为每次0.2～0.3 mg/kg，每日3次，饭前半小时及睡前口服。②通过乙酰胆碱起作用的药物：西沙必利（cisapride, 普瑞博思）主要作用于肠肌层神经丛运动神经原的5-羟色胺（5-HT_4）受体，增加乙酰胆碱释放，从而促进胃排空和增加LES压力。常用剂量为每次0.1～0.2mg/kg, 每日3次口服。

（2）抗酸和抑酸药：主要作用为抑制酸分泌、中和胃酸以减少反流物对食管黏膜的损伤，提高LES张力。①抑酸药：H_2受体拮抗剂（H_2 –receptor blockers）如西咪替丁（cimetidine）、雷尼替丁（ranitidine）、法莫替丁（famotidine）、尼扎替丁（nizatidine）；质子泵抑制剂（proton pump inhibitors，PPI）如奥美拉唑（omeprazol，洛赛克）、兰索拉唑（lansoprazole）、埃索美拉唑（esomeprazole）等。②中和胃酸药：如氢氧化铝凝胶，多用于年长儿。 '

（3）黏膜保护剂（mucosa protector）：硫醣铝、硅酸铝盐、磷酸铝等。

（2）、（3）两项药物治疗见本章第四节消化性溃疡治疗。

4．外科治疗 及时采用体位、包含、药物等治疗方法后，大多数患儿症状能明显改善和痊愈。具有下列指征可考虑外科手术：①内科治疗6～8周无效，有严重并发症（消化道出血、营养不良、生长发育迟缓）；②严重食管炎伴溃疡、狭窄或发现有解剖异常如食管裂孔疝等；③有严重的呼吸道并发症，如呼吸道梗阻、反复发作吸入性肺炎或窒息、伴支气管肺发育不良者；④合并严重神经系统疾病。

第四节 胃炎和消化性溃疡

一、胃炎

胃炎（gastritis）是指由各种物理性、化学性或生物性有害因子引起的胃黏膜或胃壁炎性改变的一种疾病。根据病程分急性和慢性两种，后者发病率高。

【病因和发病机制】

1．急性胃炎 多为继发性，可由严重感染、休克、缺血缺氧、颅内损伤、严重烧伤、呼吸衰竭和其它危重疾病所致的应激反应（又称胃肠功能障碍/衰竭）引起。误服毒性物质和腐蚀剂，摄入由细菌及其毒素污染的食物，服用对胃黏膜有损害的药物（如阿司匹林等非甾体类抗炎药），食物过敏，胃内异物，情绪波动、精神紧张和各种因素所致的变态反应等均能引起胃黏膜的急性炎症。

2．慢性胃炎 是有害因子长期反复作用于胃黏膜引起损伤的结果，小儿慢性胃炎中以浅表性胃炎最常见，约占90％～95％，萎缩性胃炎极少。病因迄今尚未完全明确，可能与下列因素

有关。

（1）感染：已证实幽门螺杆菌（helicobater pylori，Hp）所致的胃内感染是胃炎的主要病因，在活动性、重度胃炎中Hp检出率很高。慢性胃炎亦有家族聚集倾向也表明了Hp在家族成员间的传播。

（2）胆汁反流：各种原因引起胃肠道动力异常，胃窦内容物滞留或十二指肠胃反流，反流的胆盐刺激减低了胃黏膜对离子通透的屏障功能，使得胃液中氢离子得以反弥散进入胃黏膜引起炎症。

（3）长期服用刺激性食物和药物：如粗糙、过硬、过冷、过热、辛辣的食品，经常暴饮、暴食、饮浓茶、咖啡，阿司匹林等非甾体抗炎药及类固醇激素类药物。

（4）精神神经因素：持续精神紧张、压力过大，可使消化道激素分泌异常。

（5）全身慢性疾病影响：如慢性肾炎、尿毒症、重症糖尿病、肝胆系统疾病、类风湿性关节炎、系统性红斑狼疮等。

（6）其它因素：如环境、遗传、免疫、营养等因素均与发病有关。

【临床表现】

1．急性胃炎 发病急骤，轻者仅有食欲不振、腹痛、恶心、呕吐，严重者可出现呕血、黑便、脱水、电解质及酸碱平衡紊乱。有感染者常伴有发热等全身中毒症状。

2．慢性胃炎 常见症状为反复发作、无规律性的腹痛，疼痛经常出现于进食过程中或餐后，多数位于上腹部、脐周，部分患儿部位不固定，轻者为间歇性隐痛或钝痛，严重者为剧烈绞痛。常伴有食欲不振、恶心、呕吐、腹胀，继而影响营养状况及生长发育。胃黏膜糜烂出血者伴呕血、黑便。

【实验室检查】

1．胃镜检查 为最有价值、安全、可靠的诊断手段。可直接观察胃黏膜病变及其程度，可见黏膜广泛充血、水肿、糜烂、出血，有时可见黏膜表面的黏液斑或反流的胆汁。Hp感染胃炎时，还可见到胃黏膜微小结节形成（又称胃窦小结节或淋巴细胞样小结节增生）。同时可取病变部位组织进行幽门螺杆菌和病理学检查。

2．幽门螺杆菌检测

（1）胃黏膜组织切片染色与培养：Hp培养需在微氧环境下用特殊培养基进行，3～5天可出结果，是最准确的诊断方法。

（2）尿素酶试验：尿素酶试剂中含有尿素和酚红，Hp产生的酶可分解其中的尿素产生氨，后者使试剂中的pH值上升，从而使酚红由棕黄色变成红色。将活检胃黏膜放入上述试剂（滤纸片）中，如胃黏膜含有Hp则试剂变为红色，此法快速、简单，特异性和敏感性可达90％以上。

（3）血清学检测抗Hp抗体：但是IgM抗体可在清除了Hp几个月后仍保持阳性，限制了其诊断意义。

（4）核素标记尿素呼吸试验：让患儿口服一定量同位素^{13}C标记的尿素，如果患儿消化道内含有Hp，则Hp产生的尿素酶可将尿素分解产生CO_2，由肺呼出。通过测定呼出气体中^{13}C含量即可判断胃内Hp感染程度，其特异性和敏感性均达90％以上。

【病理】

1．急性胃炎 表现为上皮细胞变性、坏死，固有膜大量中性粒细胞浸润，无或极少有淋巴细胞、浆细胞，腺体细胞呈不同程度变性坏死。

2．慢性胃炎 浅表性胃炎见上皮细胞变性，小凹上皮细胞增生，固有膜炎症细胞主要为淋巴细胞、浆细胞浸润。萎缩性胃炎主要为固有腺体萎缩，肠腺化生及炎症细胞浸润。

【诊断和鉴别诊断】

根据病史、体检、临床表现、胃镜和病理学检查，基本可以确诊。由于引起儿童腹痛的病因很多，急性发作的腹痛必须注意与外科急腹症、肝、胆、胰、肠等腹内脏器的器质性疾病，以及腹型过敏性紫癜相鉴别。慢性反复发作性腹痛应与肠道寄生虫、肠痉挛等疾病鉴别。

1．肠蛔虫症 常有不固定腹痛、偏食、异食癖、恶心、呕吐等消化功能紊乱症状，有时出现全身过敏症状，驱虫治疗有效等可协助诊断。有吐、排虫史，粪便查找虫卵可以确诊。随着卫生条件的改善，肠蛔虫症在我国已经大为减少。

2．肠痉挛 婴儿多见，可出现反复发作的阵发性腹痛，腹部无异常体征，排气、排便后可缓解。

3．心理因素所致功能性（再发性）腹痛 是一种常见的儿童期身心疾病。原因不明，与情绪改变、生活事件、家庭成员过度焦虑等有关。表现为发作性腹痛，持续数十分钟或数小时而自行缓解，可以伴有恶心、呕吐等症状。临床和辅助检查往往无阳性发现。

【治疗】

1．急性胃炎 去除病因，积极治疗原发病，避免服用一切刺激性食物和药物，及时纠正水、电解质紊乱。有上消化道出血者应卧床休息，保持安静，监测生命体征及呕吐与黑便情况。静滴H_2受体拮抗剂，口服胃黏膜保护剂，可用局部黏膜止血的方法。细菌感染者应用有效抗生素。

2．慢性胃炎

（1）去除病因，积极治疗原发病。

（2）饮食治疗：养成良好的饮食习惯和生活规律。饮食定时定量，避免服用刺激性食品和对胃黏膜有损害的药物。

（3）药物治疗：①黏膜保护剂：如次碳酸铋、硫糖铝、蒙脱石粉剂等；②H_2受体拮抗剂：常用西咪替丁、雷尼替丁、法莫替丁等；③胃肠动力药：腹胀、呕吐或胆汁反流者加用多潘立酮、西沙必利；④有幽门螺杆菌感染者应进行规范的抗Hp治疗（见消化性溃疡病治疗）。药物治疗时间视病情而定。

二、消化性溃疡

消化性溃疡（peptic ulcer）是指胃和十二指肠的慢性溃疡，也可发生在与酸性胃液相接触的其他胃肠道部位。各年龄儿童均可发病，以学龄儿童多见。婴幼儿多为急性、继发性溃疡，常有明确的原发疾病，胃溃疡和十二指肠溃疡发病率相近；年长儿多为慢性、原发性溃疡，以十二指肠溃疡多见，男孩多于女孩，可有明显的家族史。

【病因和发病机制】

原发性消化性溃疡的病因与诸多因素有关，确切发病机理至今尚未完全阐明，目前认为溃疡的形成是由于对胃和十二指肠黏膜有损害作用的侵袭因子（酸、胃蛋白酶、胆盐、药物、微生物及其它有害物质）与黏膜自身的防御因素（黏膜屏障、黏液重碳酸盐屏障、黏膜血流量、细胞更新、前列腺素等）之间失去平衡的结果。一般认为，与酸有关侵袭因素对十二指肠溃疡的意义较大，而组织防御因素对胃溃疡有更重要的意义。

1．胃酸和胃蛋白酶的侵袭 酸和胃蛋白酶是对胃和十二指肠黏膜有侵袭作用的主要因素。十二指肠溃疡患者基础胃酸、壁细胞数量及壁细胞对刺激物质的敏感性均高于正常人，且胃酸分泌的正常反馈抑制机制亦发生缺陷，故酸度增高是形成溃疡的重要原因。新生儿生后1～2天胃酸分泌高，与成人相同，4～5天时下降，以后又逐渐增高，故生后2～3天亦可发生原发性消

化性溃疡。因胃酸分泌随年龄而增加，所以年长儿原发性溃疡的发病率较婴幼儿高。

2．胃和十二指肠黏膜的防御功能 决定胃黏膜抵抗损伤能力的因素包括黏膜血流、上皮细胞的再生、黏液分泌和黏膜屏障的完整性。在各种攻击因子的作用下，黏膜血循环及上皮细胞的分泌与更新受到影响，屏障功能受损，发生黏膜缺血、坏死而形成溃疡。

3．幽门螺杆菌感染 儿童十二指肠溃疡Hp检出率约为60％，Hp被根除后溃疡的复发率即下降，说明Hp在溃疡病发病机制中起重要作用。

4．遗传因素 消化性溃疡的发生具有遗传因素的证据，部分患儿可以有家族史，单卵双胎发生溃疡的一致性也较高。但其家族史也可能与Hp感染的家族聚集倾向有关。O型血的人十二指肠溃疡发病率较其它血型的人高；2/3的十二指肠溃疡患者的家族成员血清胃蛋白酶原升高。

5．其它 精神创伤、中枢神经系统病变、外伤、手术后、饮食习惯不当如暴饮暴食，过冷、油炸食品、气候因素、对胃黏膜有刺激性的药物如非甾体抗炎药、类固醇激素等均可降低胃黏膜的防御能力，引起胃黏膜损伤。

继发性溃疡是由于全身疾病引起的胃、十二指肠黏膜局部损害。见于各种危重疾病所致的应激反应（参见急性胃炎病因）。

【病理】

十二指肠溃疡好发于球部，偶尔位于球后以下的部位称球后溃疡。多为单发，也可多发。胃溃疡多发生在胃窦、胃体交界的小弯侧，少数可发生在胃窦、胃体、幽门前方或幽门管内。溃疡大小不等，深浅不一，胃镜下观察呈圆形、不规则圆形或线形，底部有灰白苔，周围黏膜充血、水肿。球部因黏膜充血、水肿，或因多次复发后纤维组织增生和收缩而导致球部变形，有时出现假憩室。胃和十二指肠同时有溃疡时称复合溃疡。

【临床表现】

由于溃疡在各年龄阶段的好发部位、类型和演变过程不同，临床症状和体征也有所不同，年龄愈小，症状愈不典型，不同年龄患者的临床表现有各自的特点。

1．新生儿 继发性溃疡多见，常见原发病有：早产儿、出生窒息等缺血缺氧、败血症、低血糖、呼吸窘迫综合征和中枢神经系统疾病等。常表现急性起病，呕血、黑便。生后2～3天亦可发生原发性溃疡。

2．婴儿期 继发性溃疡多见，发病急，首发症状可为消化道出血和穿孔。原发性以胃溃疡多见，表现为食欲差、呕吐、进食后啼哭、腹胀、生长发育迟缓，也可表现为呕血、黑便。

3．幼儿期 胃和十二指肠溃疡发病率相等，常见进食后呕吐，间歇发作脐周及上腹部疼痛，烧灼感少见，夜间及清晨痛醒，可发生呕血、黑便甚至穿孔。

4．学龄前及学龄期 以原发性十二指肠溃疡多见，主要表现为反复发作脐周及上腹部胀痛、烧灼感，饥饿时或夜间多发。严重者可出现呕血、便血、贫血。并发穿孔时疼痛剧烈并放射至背部或左右上腹部。也有仅表现为贫血、粪便隐血试验阳性。

【并发症】

主要为出血、穿孔和幽门梗阻，常可伴发缺铁性贫血。重症可出现失血性休克。如溃疡穿孔至腹腔或邻近器官，可出现腹膜炎、胰腺炎等。如炎症和水肿较广泛，可出现急、慢性梗阻。

【辅助检查】

1．上消化道内镜检查 是诊断溃疡病准确率最高的方法。内镜观察不仅能准确诊断溃疡、观察病灶大小、周围炎症的轻重、溃疡表面有无血管暴露，同时又可采取黏膜活体组织作病理

组织学和细菌学检查，还可以在内镜下控制活动性出血。内镜下溃疡可分为活动期（A）、愈合期（H）和瘢痕期（S），其中每个病期又可分为1～2个阶段。

2．胃肠X 线钡餐造影 虽然应用较广泛，但此诊断手段不够敏感和特异。

（1）直接征象：发现胃和十二指肠壁龛影可确诊。

（2）间接征象：溃疡对侧切迹，十二指肠球部痉挛、畸形对本病有诊断参考价值。因儿童溃疡浅表，钡餐通过快，检出率较成人为低，且假阳性率较高，气、钡双重对比造影效果较佳。

3．幽门螺杆菌检测（见慢性胃炎节）。

【诊断和鉴别诊断】

由于儿童消化性溃疡的症状和体征不如成人典型，常易误诊和漏诊，故对出现剑突下有烧灼感或饥饿痛；反复发作、进食后缓解的上腹痛，夜间及清晨症状明显；与饮食有关的呕吐；粪便隐血试验阳性的贫血患儿；反复胃肠不适，且有溃疡病尤其是十二指肠溃疡家族史者；原因不明的呕血、便血者等，均应警惕消化性溃疡病的可能性，及时进行内镜检查，尽早明确诊断。以下症状应与其他疾病鉴别：

1．腹痛 应与肠痉挛、蛔虫症、腹内脏器感染、结石、腹型过敏性紫癜等疾病鉴别。

2．呕血 新生儿和小婴儿呕血可见于新生儿自然出血症、食管裂孔疝等；年长儿需与肝硬化致食管静脉曲张破裂及全身出血性疾病鉴别，有时还应与咯血相鉴别。

3．便血 消化性溃疡出血多为柏油样便，鲜红色便仅见于大量出血者。应与肠套叠、梅克尔憩室、息肉、腹型过敏性紫癜及血液病所致出血鉴别。

【治疗】

目的是缓解和消除症状，促进溃疡愈合，防止复发，并预防并发症。

1．一般治疗 培养良好的生活习惯，饮食定时定量，避免过度疲劳及精神紧张，消除有害因素如避免食用刺激性、对胃黏膜有损害的食物和药物。如有出血时，应积极监护治疗，以防止失血性休克。应监测生命体征如血压、心率及末梢循环。禁食同时注意补充足够血容量。应积极进行消化道局部止血（如喷药、胃镜下硬化、电凝治疗）及全身止血。如失血严重时应及时输血。

2．药物治疗 原则为抑制胃酸分泌和中和胃酸，强化黏膜防御能力，抗幽门螺杆菌治疗。

（1）抑制胃酸治疗：是消除侵袭因素的主要途径。①H_2受体拮抗剂（H_2RI）：可直接抑制组织胺、阻滞乙酰胆碱分泌，达到抑酸和加速溃疡愈合的目的。常用西米替丁，每日10～15 mg/kg，分4次于饭前10分钟至30分钟口服，或1～2次/日静脉滴注，疗程为4～8周；雷尼替丁，每日3～5 mg/kg，每晚一次口服，或分2～3次/日静脉滴注，疗程均为4～8周；法莫替丁，0.9 mg/kg，睡前一次口服，或1次/日静脉滴注，疗程为2～4周；尼扎替丁，每日5～10 mg/kg，静滴或口服。②质子泵抑制剂（PPI）：作用于胃黏膜壁细胞，降低壁细胞中的H^+-K^+-ATP酶活性，阻抑H^+从细胞浆内转移到胃腔而抑制胃酸分泌。常用奥美拉唑，剂量为每日0.6～0.8 mg/kg，清晨顿服。疗程2～4 周。③中和胃酸的抗酸剂：起缓解症状和促进溃疡愈合的作用。常用碳酸钙、氢氧化铝、氢氧化镁等。

（2）胃黏膜保护剂：①硫糖铝：在酸性胃液中与蛋白形成大分子复合物，凝聚成糊状物覆盖于溃疡表面起保护作用，亦可增强内源性前列腺素合成，促进溃疡愈合。常用剂量为每日10～25 mg/kg，分4次口服，疗程4～8周。②枸橼酸铋钾：在酸性环境中沉淀，与溃疡面的蛋白质结合，覆盖其上形成一层凝固的隔离屏障。促进前列腺素分泌，铋剂还具抗幽门螺杆菌的作用。剂量每日6～8 mg/kg，分3次口服，疗程4～6周。本药有导致神经系统不可逆损害和急性肾

功能衰竭等副作用，长期大剂量应用时应谨慎，最好有血铋监测。③蒙脱石粉、麦滋林-S颗粒剂：亦有保护胃黏膜、促进溃疡愈合的作用。④米索前列醇：即前列腺素样作用，其作用机制可能与刺激黏液和碳酸氢盐分泌，或直接保护胃黏膜上皮的完整性有关。但因其副作用临床应用较少，罕见儿科应用。

（3）抗幽门螺杆菌治疗：有Hp感染的消化性溃疡，需用抗菌药物治疗。临床常用的药物有：枸橼酸铋钾6～8 mg/（kg・d）；阿莫西林50 mg/（kg・d）；克拉霉素15～30 mg/（kg・d）；甲硝唑25～30mg/（kg・d）；呋喃唑酮5～10 mg/（kg・d），分3次口服。目前多主张联合用药，以下方案可供参考：即以PPI为中心的“三联”药物方案： PPI+上述抗生素中的2种，持续1～2周；以铋剂为中心的“三联”“四联”治疗方案：枸橼酸铋钾4～6周+2种抗生素（阿莫西林4周、克拉霉素2周、甲硝唑2周、呋喃唑酮2周），或同时+H_2RI 4～8周。

3．消化性溃疡一般不需手术治疗。但如有以下情况，应根据个体情况考虑手术治疗：①溃疡合并穿孔；②难以控制的出血，失血量大，48小时内失血量超过血容量的30％；③幽门完全梗阻，经胃肠减压等保守治疗72小时仍无改善；④慢性难治性疼痛。

第五节 先天性肥厚性幽门狭窄

先天性肥厚性幽门狭窄（congenital hypertrophic pyloric stenosis）是由于幽门环肌增生肥厚，使幽门管腔狭窄而引起的上消化道不完全梗阻性疾病。发病率约为1/1000～1/3000。第一胎多见，男性多见，男女发病率之比约为5：1，患儿多为足月儿，未成熟儿较少见。

【病因和发病机制】

尚未完全清楚，一般认为与下列两种因素有关。

1．遗传因素 本病为多基因遗传病，父或母有本病史者，其子代发病率可高达7％左右；母亲有本病史的子代发病机会比父亲有本病史者为高。

2．胃肠激素及其他生物活性物质紊乱 研究注意到：患儿幽门环肌中的脑啡肽、P物质和血管活性肠肽有不同程度的减少；血清促胃液素升高、前列腺素水平增高；使用外源性前列腺素E维持动脉导管开放时容易发生本病；幽门组织一氧化氮合酶减少等。

【病理】

幽门肌全层增生、肥厚，以环肌更为明显。幽门明显增大呈橄榄形，颜色苍白，表面光滑，质地如硬橡皮。肿块随日龄而逐渐增大。肥厚的肌层渐向胃壁移行，胃窦部界限不明显，十二指肠端则界限分明，肥厚组织突然终止于十二指肠始端，因胃强烈蠕动使幽门管部分被推入十二指肠，使十二指肠粘膜反折呈子宫颈样。幽门管腔狭窄造成食物潴留致使胃扩大、胃壁增厚，黏膜充血、水肿，可有炎症和溃疡。

【临床表现】

典型症状和体征为无胆汁的喷射性呕吐，胃蠕动波和右上腹肿块。

1．呕吐 为本病主要症状，一般在出生后2～4周，少数于生后1周发病，也有迟至生后2～3个月发病。开始为溢乳，逐日加重呈喷射性呕吐，几乎每次吃奶后均吐，多于喂奶后不到半小时即吐，自口鼻涌出。吐出物为带凝块的奶汁，不含胆汁，少数患儿因呕吐频繁使胃黏膜毛细血管破裂出血，吐出物可含咖啡样物或血。患儿食欲旺盛，呕吐后即饥饿欲食。呕吐严重时，大部食物被吐出，致使大便次数减少和少尿。

2．胃蠕动波 常见，但非特有体征。蠕动波从左季肋下向右上腹部移动，到幽门即消失。在喂奶时或呕吐前容易见到，轻拍上腹部常可引出。

3．右上腹肿块 为本病特有体征，具有诊断意义，临床检出率可达60%～80%。用指端在右季肋下腹直肌外缘处轻轻向深部按扪，可触到橄榄形、光滑、质较硬的肿块，可以移动。

4．黄疸 约1%～2%患儿伴有黄疸，间接胆红素增高，致使个别患儿呕吐物亦可能黄染，手术后数日即消失。可能与饥饿和肝功能不成熟，葡萄糖醛酸基转移酶活性不足，以及大便排出少，胆红素肝肠循环增加有关。

5．消瘦、脱水及电解质紊乱 因反复呕吐，营养物质及水摄人不足，并有胃液H^+和$C1^-$的大量丢失，患儿体重不增或下降，逐渐出现营养不良、脱水、低氯性碱中毒等，晚期脱水加重导致组织缺氧，产生乳酸血症、低钾血症。肾功能损害时，可合并代谢性酸中毒。

【辅助检查】

1．腹部B型超声检查 可发现幽门肥厚肌层为一环形低回声区，相应的黏膜层为高密度回声，并可测量肥厚肌层的厚度、幽门直径和幽门管长度，如果幽门肌厚度≥4mm、幽门前后径≥13mm、幽门管长≥17mm，即可诊断为本病。

2．X线钡餐检查 透视下可见胃扩张，钡剂通过幽门排出时间延长。仔细观察可见幽门管延长，向头侧弯曲，幽门胃窦呈鸟嘴状改变，管腔狭窄如线状等为诊断本病特有的X线征象。

【诊断和鉴别诊断】

凡具有典型的呕吐病史者，应疑及本病。若于右上腹部扪及橄榄状肿块，辅以影像学检查，即可确诊。对疑似病例应与下列疾病鉴别 。

1．喂养不当 由于喂奶过多、过急，或人工喂养时将奶瓶内气体吸入胃内，或喂奶后体位放置不当等，均为新生儿呕吐的常见原因。如系喂养不当引起的呕吐，应防止喂奶过多过急，食后抱起小儿，轻拍后背使积存在胃内的气体排出，呕吐即可停止。

2．幽门痉挛 与本病临床症状相似，但多在生后即出现间歇性不规则呕吐，非喷射性，量不多，无进行性加重，偶见胃蠕动波，但右上腹摸不到肿块。一般状况较好，无明显脱水、营养不良，B超检查无幽门肌层肥厚，用阿托品、氯丙嗪等解痉镇静剂治疗，效果良好。

3．胃食管反流 呕吐为非喷射性，上腹无蠕动波，无右上腹橄榄样肿块。采用体位疗法和稠厚食物喂养治疗可减轻呕吐。X线钡餐检查、食管24小时pH监测等可协助确诊。

4．胃扭转 生后数周内出现呕吐，移动体位时呕吐加剧。X线钡餐检查可见：①食管与胃黏膜有交叉现象；②胃大弯位于小弯之上；③幽门窦的位置高于十二指肠球部；④双胃泡、双液平面；⑤食管腹段延长，且开口于胃下方。胃镜检查亦可达到诊断和治疗（胃镜下整复）的目的。

5．其他先天性消化道畸形 如肠旋转不良、环状胰腺及肠梗阻型胎粪性腹膜炎等。根据畸形所造成的肠梗阻程度不同，症状出现早晚不一。一般于生后不久出现胆汁性呕吐，同时排便减少或消失。腹部平片显示胃及十二指肠不同程度扩张，表现为“双气泡”或“三气泡”等十二指肠梗阻的影像；环状胰腺时十二指肠降段呈现内陷、线形狭窄或节段性缩窄。肠旋转不良时钡剂灌肠可显示出结肠框及回盲部充满钡剂位于右上腹部或上腹中部。肠梗阻型胎粪性腹膜炎可见腹腔钙化斑。

【治疗】

确诊后应及早进行幽门环肌切开术，手术方法简便，效果良好。

第六节 肠 套 叠

肠套叠（intussusception）系指部分肠管及其肠系膜套入邻近肠腔所致的一种绞窄性肠梗阻，是婴幼儿时期最常见的急腹症之一，也是3个月至6岁期间引起肠梗阻的最常见原因。60％本病患儿的年龄在1岁以内，但新生儿罕见。80％患儿年龄在2岁以内，男孩发病率多于女孩，约为4：1。健康肥胖儿多见，发病季节与胃肠道病毒感染流行相一致，以春秋季多见。常伴发于胃肠炎和上呼吸道感染。

【病因和发病机制】

肠套叠分原发和继发两种。95％为原发性，多为婴幼儿，有人认为婴儿回盲部系膜尚未完全固定、活动度较大是容易发生肠套叠的结构上因素。5％继发性病例多为年长儿，发生套叠的肠管多有明显的机械原因，如梅克尔憩室翻入回肠腔内，成为肠套叠的起点。肠息肉、肠肿瘤、肠重复畸形、腹型紫癜致肠壁血肿等均可牵引肠壁而发生肠套叠。

有些促发因素可导致肠蠕动的节律发生紊乱，从而诱发肠套叠，如饮食改变、病毒感染及其腹泻等。有研究表明病毒感染可引起末段回肠集合淋巴结增生，局部肠壁增厚，甚至凸入肠腔，构成套叠起点，加之肠道受病毒感染后蠕动增强而导致肠套叠发生。

【病理】

肠套叠多为近端肠管套入远端肠腔内，依据其套入部位不同分为：①回盲型：回盲瓣是肠套叠头部，带领回肠末端进入升结肠，盲肠、阑尾也随着翻入结肠内，此型最常见，约占总数的50％～60％；②回结型：回肠从距回盲瓣几厘米处起，套入回肠最末端，穿过回盲瓣进入结肠，约占30％；③回回结型：回肠先套入远端回肠内，然后整个再套入结肠内，约占10％； ④小肠型：小肠套入小肠，少见；⑤结肠型：结肠套入结肠，少见；⑥多发型：回结肠套叠和小肠套叠合并存在。肠套叠多为顺行性套叠，与肠蠕动方向相一致。套入部随着肠蠕动不断继续前进，该段肠管及其肠系膜也一并套入鞘内，颈部束紧不能自动退出。

由于鞘层肠管持续痉挛，致使套入部肠管发生循环障碍，初期静脉回流受阻，组织充血水肿，静脉曲张，黏膜细胞分泌大量黏液，进入肠腔内，与血液及粪质混合成果酱样胶冻状排出，肠壁水肿、静脉回流障碍加重及动脉供血不足，导致肠壁坏死并出现全身中毒症状，严重者可并发肠穿孔和腹膜炎。

【临床表现】

1．急性肠套叠

（1）腹痛：既往健康的孩子突然发作剧烈的阵发性肠绞痛，哭闹不安，屈膝缩腹、面色苍白、拒食、出汗，持续数分钟或更长时间后，腹痛缓解，安静或入睡，间歇10～20分钟又反复发作。阵发性腹痛系由于肠系膜受牵拉和套叠鞘部强烈收缩所致。

（2）呕吐：初为乳汁，乳块和食物残渣，后可含胆汁，晚期可吐粪便样液体，说明有肠管梗阻。

（3）血便：为重要症状。出现症状的最初几小时大便可正常，以后大便少或无便。约85％病例在发病后6～12小时排出果酱样黏液血便，或作直肠指检时发现血便。

（4）腹部包块：多数病例在右上腹季肋下可触及有轻微触痛的套叠肿块，呈腊肠样，光滑不太软，稍可移动。晚期发生肠坏死或腹膜炎时，出现腹胀、腹水、腹肌紧张和压痛，不易扪及肿块，有时腹部扪诊和直肠指检双合检查可触及肿块。

（5）全身情况 患儿在早期一般情况尚好，体温正常，无全身中毒症状。随着病程延长，病情加重，并发肠坏死或腹膜炎时，全身情况恶化，常有严重脱水、高热、嗜睡、昏迷及休克

等中毒症状。

2．慢性肠套叠 年龄愈大，发病过程愈缓慢。主要表现为阵发性腹痛，腹痛时上腹或脐周可触及肿块，不痛时腹部平坦柔软无包块，病程有时长达十余日。由于年长儿肠腔较宽阔可无梗阻现象，肠管亦不易坏死。呕吐少见，便血发生也较晚。

【辅助检查】

1．腹部B超检查 在套叠部位横断扫描可见同心圆或靶环状肿块图像，纵断扫描可见“套筒征”。

2．B超监视下水压灌肠 经肛门插入Foley管并将气囊充气20～40ml。将“T”形管一端接Foley管，侧管接血压计监测注水压力，另一端为注水口，注入37～40℃ 等渗盐水匀速推入肠内，可见靶环状块影退至回盲部，“半岛征”由大到小，最后消失，诊断治疗同时完成。

3．空气灌肠 由肛门注入气体，在X线透视下可见杯口阴影，能清楚看见套叠头的块影，并可同时进行复位治疗。

4．钡剂灌肠 可见套叠部位充盈缺损和钡剂前端的杯口影，以及钡剂进入鞘部与套入部之间呈现的线条状或弹簧状阴影。只用于慢性肠套叠疑难病例。

【诊断和鉴别诊断】

凡健康婴幼儿突然发生阵发性腹痛或阵发性哭闹、呕吐、便血和腹部扪及腊肠样肿块时可确诊。肠套叠早期在未排出血便前应做直肠指检。本病应与下列疾病鉴别。

1．细菌性痢疾 夏季发病多，大便含黏液、脓血，里急后重，多伴有高热等感染中毒症状。粪便检查可见成堆脓细胞，细菌培养阳性。但必须注意细菌性痢疾偶尔亦可引起肠套叠，两种疾病可同时存在或肠套叠继发于细菌性痢疾后。

2．梅克尔憩室出血 大量血便，常为无痛性，亦可并发肠套叠。

3．过敏性紫癜 有阵发性腹痛，呕吐、便血，由于肠管有水肿、出血、增厚，有时左右下腹可触及肿块，但绝大多数患儿有出血性皮疹、关节肿痛，部分病例有肾脏病变。该病由于肠蠕动功能紊乱和肠壁血肿，也可并发肠套叠。

【治疗】

急性肠套叠是一种危及生命的急症，其复位是一个紧急的治疗过程，一旦确诊需立即进行。

1．非手术疗法

（1）灌肠疗法的适应证：肠套叠在48小时内，全身情况良好，腹部不胀，无明显脱水及电解质紊乱。

（2）禁忌证：①病程已超过48小时，全身情况差，有脱水、精神萎靡、高热、休克等症状者，对3个月以下婴儿更应注意；②高度腹胀，腹部有腹膜刺激征者；③X线腹部平片可见多数液平面者；④套叠头部已达脾曲，肿物硬而且张力大者；⑤多次复发疑有器质性病变者；⑥小肠型肠套叠。

（3）方法：包括①B超监视下水压灌肠；②空气灌肠；③钡剂灌肠复位三种。

（4）灌肠复位成功的表现：①拔出肛管后排出大量带臭味的黏液血便和黄色粪水；②患儿很快入睡，不再哭闹及呕吐；③腹部平软，触不到原有的包块；④灌肠复位后给予0.5～1 g活性炭口服，6～8小时后应有炭末排出，表示复位成功。

2．手术治疗 肠套叠超过48～72小时，或虽时间不长但病情严重疑有肠坏死或穿孔者以及小肠型肠套叠均需手术治疗。根据患儿全身情况及套叠肠管的病理变化程度选择进行肠套叠手法复位、肠切除吻合术或肠造瘘术等。

5％～8％患儿可有肠套叠复发。灌肠复位比手术复位的复发率高。

第七节 先天性巨结肠

先天性巨结肠（congenital megacolon）又称先天性无神经节细胞症（aganglionosis） 或赫什朋病（Hirschsprung disease，HD），是由于直肠或结肠远端的肠管持续痉挛，粪便淤滞在近端结肠，使该肠管肥厚、扩张。本病是婴儿常见的先天性肠道畸形，发病率为1/2000～1/5000，男女之比3～4：1，有遗传倾向。

【病因和病理生理】

该病发生是多基因遗传和环境因素共同作用的结果。其基本病理变化是肠壁肌间和黏膜下神经丛内缺乏神经节细胞，在形态学上可分为痉挛段、移行段和扩张段三部分。除形成巨结肠外，其他病理生理变化还有排便反射消失等。根据病变肠管痉挛段的长度，本病可分为：①常见型（约占85％）；②短段型（10％左右）；③长段型（4％左右）；④全结肠型（1％左右）。

【临床表现】

1．胎便排出延迟、顽固性便秘和腹胀 生后48小时内多无胎便或仅有少量胎便排出，可于生后2～3天出现低位肠梗阻症状。以后即有顽固性便秘，3～7天以至于1～2周排便一次。严重者发展成不灌肠不排便。痉挛段愈长，出现便秘时间愈早，愈严重。腹胀逐渐加重，腹壁紧张发亮，有静脉扩张，可见肠型及蠕动波，肠鸣音增强，膈肌上升引起呼吸困难。

2．呕吐、营养不良和发育迟缓 由于功能性肠梗阻，可出现呕吐，量不多，呕吐物含少量胆汁，严重者可见粪样液，加上长期腹胀、便秘使患儿食欲下降，影响营养物质吸收致发育迟缓、消瘦、贫血或有低蛋白血症伴水肿。

3．直肠指检 直肠壶腹部空虚，拔指后由于近端肠管内积存多量粪便，可排出恶臭气体及大便。

【并发症】

1．小肠结肠炎 为本病的常见并发症，可见于任何年龄尤其是新生儿期。由于远端肠梗阻使结肠高度扩张，肠腔内压增高导致肠黏膜缺血，降低了黏膜的屏障作用，使粪便的代谢产物、细菌、毒素进入血循环，患儿出现高热、高度腹胀、呕吐、排出恶臭并带血的稀便。肠黏膜缺血处可产生水肿、溃疡，引起血便及肠穿孔。重者炎症侵犯肌层，出现浆膜充血、水肿、增厚，导致渗出性腹膜炎。由于吐泻及扩张肠管内大量肠液的积存，迅速出现脱水和酸中毒，死亡率极高。

2．肠穿孔 多见于新生儿，常见的穿孔部位为乙状结肠和盲肠。

3．继发感染 如败血症、肺炎等。

【辅助检查】

1．X线检查

（1）腹部立位平片：多显示低位结肠梗阻，近端结肠扩张，盆腔无气体。

（2）钡剂灌肠检查：其诊断率在90％左右，可显示痉挛段及其上方的扩张肠管，排钡功能差。若黏膜皱襞变粗（锯齿状变化），提示伴有小肠结肠炎。

2．直肠、肛门测压检查 测定直肠、肛门括约肌的反射性压力变化，患儿压力升高。2周内新生儿可出现假阴性，故不适用。

3．直肠黏膜活检 HE染色判断神经节细胞的有无。组化方法测定乙酰胆碱含量和胆碱酯酶活性，患儿两者均较正常儿高出5～6 倍，但对新生儿诊断率较低。还可用免疫组织化学方法检测神经元特异性稀醇化酶等。

4．直肠肌层活体组织检查 从直肠壁取肌层组织作活体组织检查，计数神经节细胞数量。患儿缺乏神经节细胞，而无髓鞘的神经纤维增殖。

5．肌电图检查 患儿直肠和乙状结肠远端的肌电图波形低矮，频率低，不规则，峰波消失。

【诊断和鉴别诊断】

凡新生儿生后胎粪排出延迟或不排胎粪，伴有腹胀、呕吐应考虑本病。婴幼儿有长期便秘史和腹胀等体征者即应进行特殊检查。本病应与以下疾病鉴别。

1．新生儿期

（1）胎粪栓综合征（胎粪便秘）：由于胎粪浓缩稠厚可出现一过性低位肠梗阻症状，经灌肠排出胎粪后，即正常排便且不再复发。

（2）先天性肠闭锁：新生儿回肠或结肠闭锁，表现为低位肠梗阻症状，直肠指检仅见少量灰白色胶冻样便，用盐水灌肠亦不能排便。腹部直立位平片可见整个下腹部无气，钡剂灌肠X线造影可明确诊断。

（3）新生儿坏死性小肠结肠炎：与先天性巨结肠伴发小肠结肠炎者很难鉴别。本病多为早产儿，围产期多有窒息、缺氧、感染、休克的病史，且有便血。X线平片示肠壁有气囊肿和（或）门静脉积气。

2．婴儿和儿童期

（1）继发性巨结肠：肛门、直肠末端有器质性病变，如先天性肛门狭窄、术后疤痕狭窄或直肠外肿瘤压迫等使排便不畅，粪便滞留，结肠继发扩张。经肛诊检查可以确诊。

（2）特发性巨结肠：该症与排便训练不当有关，特点是患儿直、结肠有正常的神经节细胞。表现为无新生儿期便秘史，2～3岁出现症状，慢性便秘常伴肛门污便，便前常有腹痛。肛诊感觉除直肠扩张积便外，括约肌处于紧张状态，直肠肛门测压有正常反射。

3．功能性便秘：是一种原因不明的慢性便秘，分为慢传输型、出口梗阻型及混合型。表现为排便次数少、排便费力、粪质较硬或呈球状、排便不尽感，有时需借助人工方式（手抠）来协助排便。诊断需钡剂灌肠或肠镜检查排除器质性疾病。

【治疗】

应进行根治手术切除无神经节细胞肠段和部分扩张结肠。先天性巨结肠许多并发症发生在生后2个月内，故要特别重视此期间的治疗。

1．保守治疗 ①口服缓泻剂、润滑剂，帮助排便；②使用开塞露、扩肛等刺激括约肌，诱发排便；③灌肠：肛管插入深度要超过狭窄段，每日一次注入生理盐水，揉腹后使灌肠水与粪水排出，反复数次，逐渐使积存的粪便排出。

2．手术治疗 包括结肠造瘘术和根治术。凡合并小肠结肠炎不能控制者；合并有营养不良、高热、贫血、腹胀、不能耐受根治术者；或保守治疗无效、腹胀明显影响呼吸者，均应及时行结肠造瘘术。现多主张早期进行根治手术，一般认为体重在3 kg以上，周身情况良好即可行根治术。

第八节 腹 泻 病

小儿腹泻（diarrhea）是一组由多病原、多因素引起的以大便次数增多和大便性状改变为特点的消化道综合征。是我国婴幼儿最常见的疾病之一。6个月～2岁婴幼儿发病率高，一岁以内约占半数，是造成小儿营养不良、生长发育障碍的主要原因之一。

婴幼儿容易患腹泻病，主要与下列易感因素有关。

1．婴幼儿消化系统发育尚未成熟，胃酸和消化酶分泌少，酶活力偏低，不能适应食物质和量的较大变化。婴幼儿水代谢旺盛，对缺水的耐受力差，一旦失水容易发生体液紊乱。婴儿时期神经、内分泌、循环、肝、肾功能发育不成熟，容易发生消化道功能紊乱。

2．生长发育快，所需营养物质相对较多，且婴儿食物以液体为主，进入量较多，胃肠道负担重。

3．机体防御功能差 ①婴儿胃酸偏低，胃排空较快，对进入胃内的细菌杀灭能力较弱； ②血清免疫球蛋白（尤其是IgM、IgA）和胃肠道分泌型IgA（SIgA）均较低，肠黏膜免疫的防御反应及口服耐受机制均不完善。

4．肠道菌群失调 正常肠道菌群（normal bacteria flora）对入侵的致病微生物有拮抗作用，新生儿生后尚未建立正常肠道菌群、改变饮食使肠道内环境变化或滥用广谱抗生素，均可使肠道正常菌群平衡失调而患肠道感染。同时，维生素Kraft合成有赖于肠道正常菌群的参与，故肠道菌群失调时除易患腹泻外，还可有呕吐物或大便中带血。

5．人工喂养 母乳中含有大量体液因子（SIgA、乳铁蛋白）、巨噬细胞和粒细胞、溶菌酶、溶酶体，有很强的抗肠道感染作用。家畜乳中虽有某些上述成分，但在加热过程中被破坏，而且人工喂养的食物和食具易受污染，故人工喂养儿肠道感染发生率明显高于母乳喂养儿。

【病因】

引起儿童腹泻病的病因分为感染性及非感染性两种。

1．感染因素 肠道内感染可由病毒、细菌、真菌、寄生虫引起，以前两者多见，尤其是病毒。

（1）病毒感染：寒冷季节的婴幼儿腹泻80％由病毒感染引起。病毒性肠炎主要病原为轮状病毒（rotavirus），其次有星状病毒（astrovirus），杯状病毒（calicivirus）科的诺如病毒属（Norovirus）如诺沃克病毒（Norwalk virus），札如病毒（sapovirus）；此外，肠道病毒包括柯萨奇病毒（coxsackie virus）、埃可病毒（echo virus）、肠道腺病毒（enteric adenovirus）等，冠状病毒（coronavirus）科的环曲病毒（torovirus）等。

（2）细菌感染（不包括法定传染病）

1）致腹泻大肠杆菌：根据引起腹泻的大肠杆菌不同致病毒性和发病机制，已知菌株可分为5大组。①致病性大肠杆菌（enteropathogenic E. coli，EPEC）：为最早发现的致腹泻大肠杆菌。EPEC侵入肠道后，粘附在肠黏膜上皮细胞，引起肠黏膜微绒毛破坏，皱襞萎缩变平，黏膜充血、水肿而致腹泻，可累及全肠道。②产毒性大肠杆菌（enterotoxigenic E. coli，ETEC）可粘附在小肠上皮刷状缘，在细胞外繁殖，产生不耐热肠毒素（1abile toxin，LT）和耐热肠毒素（stable toxin，ST）而引起腹泻。③侵袭性大肠杆菌（enteroinvasive E. coli，EIEC）：可直接侵入小肠黏膜引起炎症反应，也可粘附和侵入结肠黏膜，导致肠上皮细胞炎症和坏死，引起痢疾样腹泻。该菌与志贺菌相似，两者O抗原有交叉反应。④出血性大肠杆菌（enterohemorrhagic E. coli，EGEC）：粘附于结肠产生与志贺杆菌相似的肠毒素（vero毒素），引起肠黏膜坏死和肠液分泌，致出血性肠炎。⑤粘附-集聚性大肠杆菌（enteroadherent-aggregative E. coli，EAEC）：以集聚方式粘附于下段小肠和结肠黏膜致病，不产生肠毒素，亦不引起组织损伤。

2）空肠弯曲菌（campylobacter jejuni）：与肠炎有关的弯曲菌有空肠型、结肠型和胎儿亚型3种，95％～99％弯曲菌肠炎是由胎儿弯曲菌空肠亚种（简称空肠弯曲菌）所引起。致病菌直接侵入空肠、回肠和结肠黏膜，引起侵袭性腹泻，某些菌株亦能产生肠毒素。

3）耶尔森菌（Yersinia）：除侵袭小肠、结肠黏膜外，还可产生肠毒素，引起侵袭性和分泌性腹泻。

4）其它：沙门菌（salmonella）（主要为鼠伤寒和其它非伤寒、副伤寒沙门菌）、嗜水气单胞菌（aeromonas hydrophila）、难辨梭状芽胞杆菌（clostridium difficile）、金黄色葡萄球菌（staphylococcal aureus）、绿脓杆菌（bacillus pyoeyaneus）、变形杆菌（bacillus proteus）等均可引起腹泻。

（3）真菌：致腹泻的真菌有念珠菌、曲菌、毛霉菌，婴儿以白色念珠菌（candida albicans）多见。

（4）寄生虫：常见为蓝氏贾第鞭毛虫、阿米巴原虫和隐孢子虫等。

肠道外感染：有时亦可产生腹泻症状，如患中耳炎、上呼吸道感染、肺炎、泌尿系感染、皮肤感染或急性传染病时，可由于发热、感染原释放的毒素、抗生素治疗、直肠局部激惹（膀胱感染）作用而并发腹泻。有时病原体（主要是病毒）可同时感染肠道。

使用抗生素引起的腹泻：除了一些抗生素可降低糖类转运和乳糖酶水平之外，肠道外感染时长期、大量地使用广谱抗生素可引起肠道菌群紊乱，肠道正常菌群减少，耐药性金黄色葡萄球菌、变形杆菌、绿脓杆菌、难辨梭状芽胞杆菌或白色念珠菌等可大量繁殖，引起药物较难控制的肠炎，称之为抗生素相关性腹泻（antibiotic-associated diarrhea，AAD）。

2．非感染因素

（1）饮食因素 ①喂养不当可引起腹泻，多为人工喂养儿，原因为：喂养不定时，饮食量不当，突然改变食物品种，或过早喂给大量淀粉或脂肪类食品；果汁，特别是那些含高果糖或山梨醇的果汁，可产生高渗性腹泻；肠道刺激物（调料、富含纤维素的食物）也可引起腹泻。②过敏性腹泻，如对牛奶或大豆等食物过敏而引起腹泻。③原发性或继发性双糖酶（主要为乳糖酶）缺乏或活性降低，肠道对糖的消化吸收不良而引起腹泻。

（2）气候因素：气候突然变化、腹部受凉使肠蠕动增加；天气过热消化液分泌减少或由于口渴饮奶过多等都可能诱发消化功能紊乱致腹泻。

【发病机制】

导致腹泻的机制有：肠腔内存在大量不能吸收的具有渗透活性的物质——“渗透性”腹泻；肠腔内电解质分泌过多——“分泌性”腹泻；炎症所致的液体大量渗出——“渗出性”腹泻；及肠道运动功能异常——“肠道功能异常”性腹泻等。但在临床上不少腹泻并非由某种单一机制引起，而是在多种机制共同作用下发生的。

1．感染性腹泻 病原微生物多随污染的食物或饮水进入消化道，亦可通过污染的日用品、手、玩具或带菌者传播。病原微生物能否引起肠道感染，决定于宿主防御机能的强弱、感染菌量的多少及微生物的毒力。

（1）病毒性肠炎：各种病毒侵入肠道后，在小肠绒毛顶端的柱状上皮细胞上复制，使细胞发生空泡变性和坏死，其微绒毛肿胀，排列紊乱和变短，受累的肠黏膜上皮细胞脱落，遗留不规则的裸露病变，致使小肠黏膜回吸收水分和电解质的能力受损，肠液在肠腔内大量积聚而引起腹泻。同时，发生病变的肠黏膜细胞分泌双糖酶不足且活性降低，使食物中糖类消化不全而积滞在肠腔内，并被细菌分解成小分子的短链有机酸，使肠液的渗透压增高。微绒毛破坏亦造成载体减少，上皮细胞钠转运功能障碍，水和电解质进一步丧失（图11-1）。新近的研究表明：轮状病毒的非结构蛋白4（NSP4）与发病机制关系密切。NSP4是具有多种功能的液体分泌诱导剂，可以通过以下方式发挥作用：作用于固有层细胞，激活Cl^-分泌和水的外流；改变上皮细胞的完整性，从而影响细胞膜的通透性；本身可能形成一个通道或是激活一种潜在的Ca^{2+}激活通道，导致分泌增加；通过旁分泌效应作用于未感染的细胞，扩大了被感染黏膜上皮细胞的感染效应；直接作用于肠道神经系统（ENS），产生类似于霍乱毒素引起的腹泻。

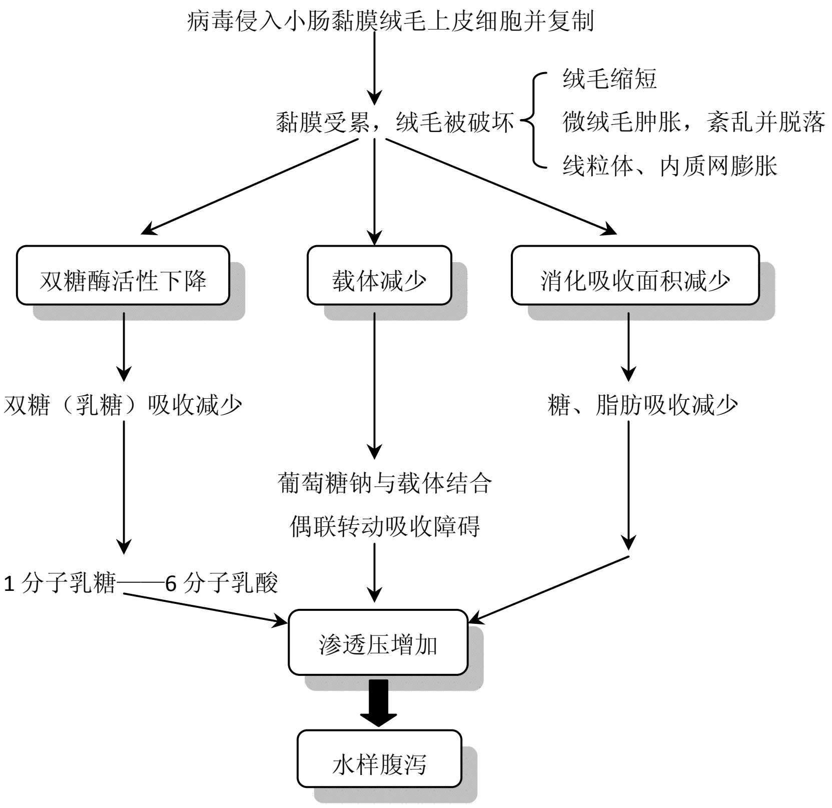

图 11-1 病毒性肠炎发病机制

（2）细菌性肠炎：肠道感染的病原菌不同，发病机理亦不同。

1）肠毒素性肠炎：各种产生肠毒素的细菌可引起分泌性腹泻，如霍乱弧菌、产肠毒素性大肠杆菌等，如图11-2所示。病原体侵入肠道后，一般仅在肠腔内繁殖，粘附在肠上皮细胞刷状缘，不侵入肠黏膜。细菌在肠腔释放2种肠毒素，即不耐热肠毒素（LT）和耐热肠毒素（ST），LT与小肠上皮细胞膜上的受体结合后激活腺苷酸环化酶，致使三磷酸腺苷（ATP）转变为环磷酸腺苷（cAMP），cAMP增多后即抑制小肠绒毛上皮细胞吸收Na^+、$C1^-$和水，并促进肠腺分泌$C1^-$；ST则通过激活鸟苷酸环化酶，使三磷酸鸟苷（GTP）转变为环磷酸鸟苷（cGMP），cGMP 增多后亦使肠上皮细胞减少Na^+和水的吸收、促进Cl^-分泌。两者均使小肠液液总量增多，超过结肠的吸收限度而发生腹泻，排出大量水样便，导致患儿脱水和电解质紊乱。

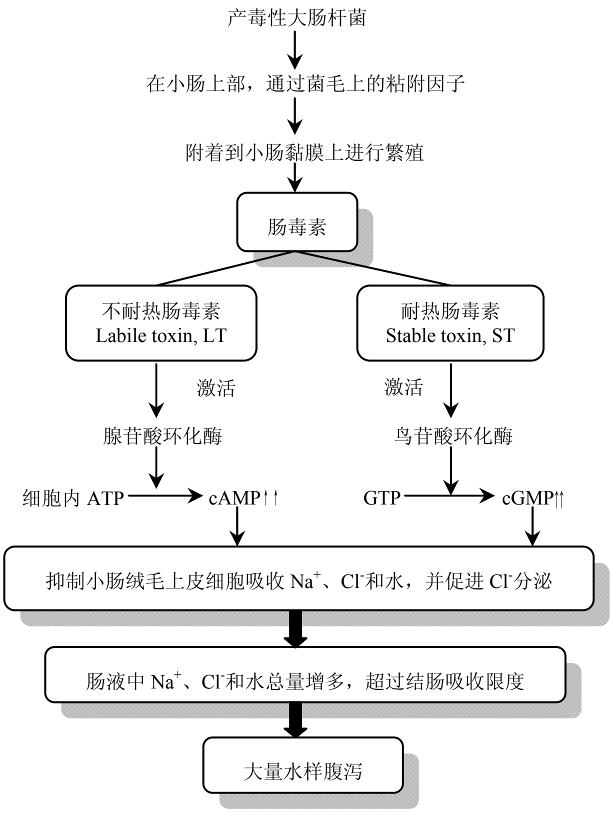

图 11-2 肠毒素引起的肠炎发病机制-以产毒性大肠杆菌为例

（2）侵袭性肠炎：各种侵袭性细菌感染可引起渗出性腹泻，如志贺菌属、沙门菌属、侵袭性大肠杆菌、空肠弯曲菌、耶尔森菌和金黄色葡萄球菌等均可直接侵袭小肠或结肠肠壁，使黏膜充血、水肿，炎症细胞浸润引起渗出和溃疡

等病变。患儿排出含有大量白细胞和红细胞的菌痢样粪便。结肠由于炎症病变而不能充分吸收来自小肠的液体，并且某些致病菌还会产生肠毒素，故亦可发生水样腹泻。

2．非感染性腹泻 主要是由饮食不当引起，如图11-3所示。当进食过量或食物成分不恰当时，消化过程发生障碍，食物不能被充分消化和吸收而积滞在小肠上部，使肠腔内酸度降低，有利于肠道下部的细菌上移和繁殖；食物发酵和腐败，分解产生的短链有机酸使肠腔内渗透压增高，腐败性毒性产物刺激肠壁使肠蠕动增加导致腹泻，进而发生脱水和电解质紊乱。

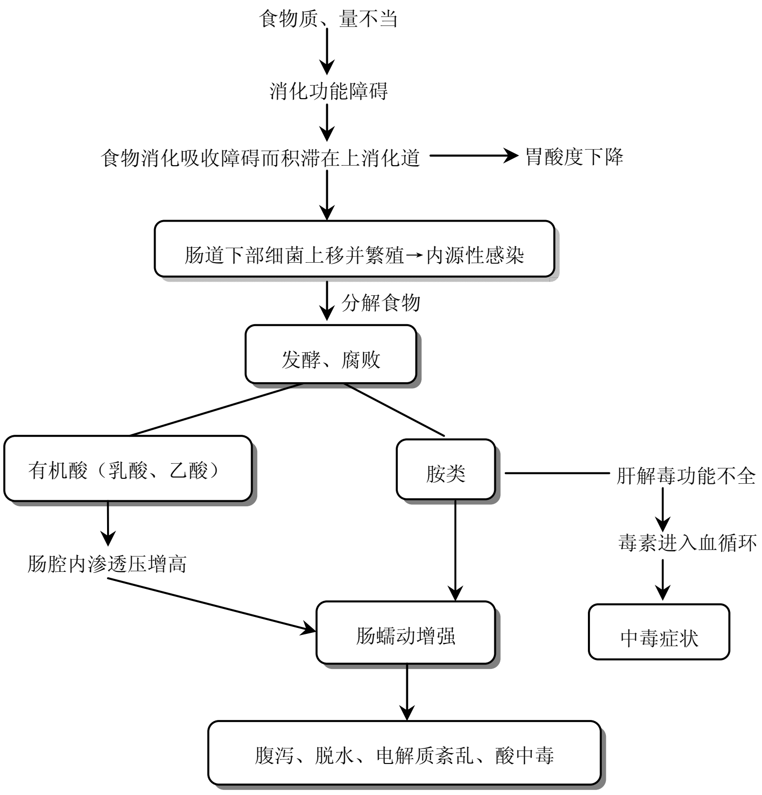

图 11-3 饮食不当引起腹泻发生机制

【临床表现】

不同病因引起的腹泻常各具临床特点和不同临床过程。故在临床诊断中常包括病程、严重程度及估计可能的病原。连续病程在2周以内的腹泻为急性腹泻，病程2周～2个月为迁延性腹泻，慢性腹泻的病程为2个月以上。国外学者亦有将病程持续2周以上的腹泻统称为慢性腹泻或难治性腹泻。

1．急性腹泻

（1）腹泻的共同临床表现

1）轻型：常由饮食因素及肠道外感染引起。起病可急可缓，以胃肠道症状为主，食欲不振，偶有溢乳或呕吐，大便次数增多，但每次大便量不多，稀薄或带水，呈黄色或黄绿色，有酸味，常见白色或黄白色奶瓣和泡沫。无脱水及全身中毒症状，多在数日内痊愈。

2）重型：多由肠道内感染引起。常急性起病，也可由轻型逐渐加重、转变而来，除有较重的胃肠道症状外，还有较明显的脱水、电解质紊乱和全身感染中毒症状，如发热、精神烦躁或萎靡、嗜睡，甚至昏迷、休克。

胃肠道症状食欲低下，常有呕吐，严重者可吐咖啡色液体；腹泻频繁，大便每日10余次至数10次，多为黄色水样或蛋花样便，含有少量黏液，少数患儿也可有少量血便。

水、电解质及酸碱平衡紊乱：由于吐泻丢失体液和摄入量不足，使体液总量尤其是细胞外

液量减少，导致不同程度（轻、中、重）脱水。由于腹泻患儿丧失的水和电解质的比例不尽相同，可造成等渗、低渗或高渗性脱水，以前两者多见。出现眼窝、囟门凹陷，尿少泪少，皮肤黏膜干燥、弹性下降，甚至血容量不足引起的末梢循环的改变（图11-4）。

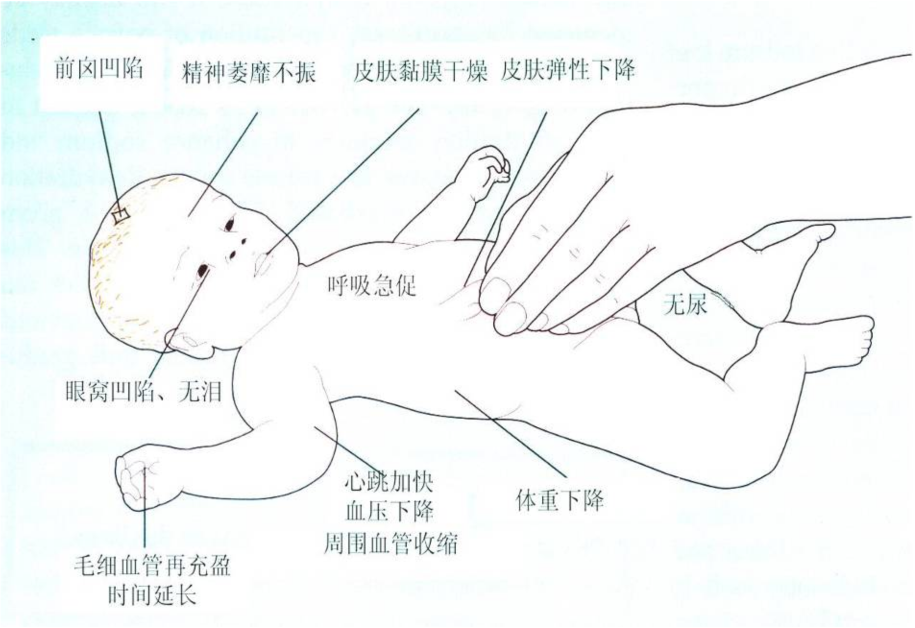

图11-4 婴幼儿脱水时的特征性症状、体征

代谢性酸中毒：其发生原因有：①腹泻丢失大量碱性物质；②进食少，肠吸收不良，热能不足使机体得不到正常能量供应导致脂肪分解增加，产生大量酮体；③脱水时血容量减少，血液浓缩使血流缓慢，组织缺氧导致无氧酵解增多而使乳酸堆积；④脱水使肾血流量亦不足，其排酸、保钠功能低下使酸性代谢产物滞留体内。患儿可出现精神不振、口唇樱红、呼吸深大、呼出气凉有丙酮味等症状，但小婴儿症状可以很不典型。

低钾血症：胃肠液中含钾较多，呕吐和腹泻丢失大量钾盐；进食少，钾的摄入量不足；肾脏保钾功能比保钠差，缺钾时仍有一定量钾继续排出，所以腹泻病时常有体内缺钾。但在脱水未纠正前，由于血液浓缩，酸中毒时钾由细胞内向细胞外转移，尿少而致钾排出量减少等原因，体内钾总量虽然减少，但血清钾多数正常。随着脱水、酸中毒被纠正、排尿后钾排出增加、大便继续失钾以及输入葡萄糖合成糖原时使钾从细胞外进入细胞内等因素使血钾迅速下降，出现不同程度的缺钾症状，如精神不振、无力、腹胀、心律紊乱、碱中毒等。

低钙血症和低镁血症：腹泻患儿进食少，吸收不良，从大便丢失钙、镁，可使体内钙、镁减少，活动性佝偻病和营养不良患儿中更多见。但是脱水、酸中毒时由于血液浓缩、离子钙增多等原因，不出现低血钙的症状，待脱水、酸中毒纠正后则出现低钙症状（手足搐搦和惊厥）。极少数久泻和营养不良患儿输液后出现震颤、抽搐，用钙治疗无效时应考虑有低镁血症可能。

（2）几种常见类型肠炎的临床特点

1）轮状病毒肠炎：是秋、冬季婴幼儿腹泻最常见的病原，故曾被称为秋季腹泻。呈散发或小流行，经粪-口传播，也可通过气溶胶形式经呼吸道感染而致病。潜伏期1～3天，多发生在6～24个月婴幼儿，4 岁以上者少见。起病急，常伴发热和上呼吸道感染症状，无明显感染中毒症状。病初1～2天常发生呕吐，随后出现腹泻。大便次数多、量多、水分多，黄色水样或蛋花样便带少量黏液，无腥臭味。常并发脱水、酸中毒及电解质紊乱。近年报道，轮状病毒感染亦可侵犯多个脏器，可产生神经系统症状，如惊厥等；有的患儿表现为血清心肌酶谱异常，提示心肌受累。本病为自限性疾病，数日后呕吐渐停，腹泻减轻，不喂乳类的患儿恢复更快，自然病程约3～8天，少数较长。大便显微镜检查偶有少量白细胞，感染后1～3天即有大量病毒自

大便中排出，最长可达6天。血清抗体一般在感染后3周上升。病毒较难分离，有条件可直接用电镜检测病毒，或用ELISA法检测病毒抗原和抗体，或PCR及核酸探针技术检测病毒抗原。

2）诺沃克病毒性肠炎：主要发病季节为9月～4月，多见于年长儿和成人。潜伏期1～2天，起病急慢不一。可有发热、呼吸道症状。腹泻和呕吐轻重不等，大便量中等，为稀便或水样便，伴有腹痛。病情重者体温较高，伴有乏力、头痛、肌肉痛等。本病为自限性疾病，症状持续1～3天。粪便及周围血象检查一般无特殊发现。

3）产毒性细菌引起的肠炎：多发生在夏季。潜伏期1～2天，起病较急。轻症仅大便次数稍增，性状轻微改变。重症腹泻频繁，量多，呈水样或蛋花样混有黏液，镜检无白细胞。伴呕吐，常发生脱水、电解质和酸碱平衡紊乱。自限性疾病，自然病程3～7天，亦可较长。

4）侵袭性细菌（包括侵袭性大肠杆菌、空肠弯曲菌、耶尔森菌、鼠伤寒杆菌等）引起的肠炎：全年均可发病，多见于夏季。潜伏期长短不等。常引起志贺杆菌性痢疾样病变。起病急，高热甚至可以发生热惊厥。腹泻频繁，大便呈黏液状，带脓血，有腥臭味。常伴恶心、呕吐、腹痛和里急后重，可出现严重的中毒症状如高热、意识改变，甚至感染性休克。大便显微镜检查有大量白细胞及数量不等的红细胞。粪便细菌培养可找到相应的致病菌。其中空肠弯曲菌常侵犯空肠和回肠，且有脓血便，腹痛甚剧烈，易误诊为阑尾炎，亦可并发严重的小肠结肠炎、败血症、肺炎、脑膜炎、心内膜炎和心包炎等。另有研究表明格林-巴利综合征与空肠弯曲菌感染有关。耶尔森菌小肠结肠炎，多发生在冬季和早春，可引起淋巴结肿大，亦可产生肠系膜淋巴结炎，症状可与阑尾炎相似，也可引起咽痛和颈淋巴结炎。鼠伤寒沙门菌小肠结肠炎，有胃肠炎型和败血症型，新生儿和<1 岁婴儿尤易感染，新生儿多为败血症型，常引起暴发流行，可排深绿色黏液脓便或白色胶冻样便。

5）出血性大肠杆菌肠炎：大便次数增多，开始为黄色水样便，后转为血水便，有特殊臭味。大便显微镜检查有大量红细胞，常无白细胞。伴腹痛，个别病例可伴发溶血尿毒综合征和血小板减少性紫癜。

6）抗生素诱发的肠炎：①金黄色葡萄球菌肠炎：多继发于使用大量抗生素后，病程与症状常与菌群失调的程度有关，有时继发于慢性疾病的基础上。表现为发热、呕吐、腹泻、不同程度中毒症状、脱水和电解质紊乱，甚至发生休克。典型大便为暗绿色，量多带黏液，少数为血便。大便显微镜检查有大量脓细胞和成簇的革兰阳性球菌，培养有葡萄球菌生长，凝固酶阳性。②伪膜性小肠结肠炎：由难辨梭状芽胞杆菌引起。除万古霉素和胃肠道外用的氨基糖苷类抗生素外，几乎各种抗生素均可诱发本病。可在用药1周内或迟至停药后4～6周发病。亦见于外科手术后或患有肠梗阻、肠套叠、巨结肠等病的体弱患者。此菌大量繁殖，产生毒素A（肠毒素）和毒素B（细胞毒素）致病。表现为腹泻，轻症大便每日数次，停用抗生素后很快痊愈。重症频泻，黄绿色水样便，可有假膜排出，为坏死毒素致肠黏膜坏死所形成的假膜。黏膜下出血可引起大便带血，可出现脱水、电解质紊乱和酸中毒。伴有腹痛、腹胀和全身中毒症状，甚至发生休克。对可疑病例可行结肠镜检查。大便厌氧菌培养、组织培养法检测细胞毒素可协助确诊。③真菌性肠炎：多为白色念珠菌所致，2岁以下婴儿多见。常并发于其它感染，或肠道菌群失调时。病程迁延，常伴鹅口疮。大便次数增多，黄色稀便，泡沫较多带黏液，有时可见豆腐渣样细块（菌落）。大便显微镜检查有真菌孢子和菌丝，如芽胞数量不多，应进一步以沙氏培养基作真菌培养确诊。

2．迁延性和慢性腹泻 病因复杂，感染、物质过敏、酶缺陷、免疫缺陷、药物因素、先天性畸形等均可引起。以急性腹泻未彻底治疗或治疗不当、迁延不愈最为常见。人工喂养、营养不良婴幼儿患病率高，其原因为：①重症营养不良时胃黏膜萎缩，胃液酸度降低，使胃杀菌屏

障作用明显减弱，有利于胃液和十二指肠液中的细菌和酵母菌大量繁殖。②营养不良时十二指肠、空肠黏膜变薄，肠绒毛萎缩、变性，细胞脱落增加，双糖酶尤其是乳糖酶活性以及刷状缘肽酶活性降低，小肠有效吸收面积减少，引起各种营养物质的消化吸收不良。③重症营养不良患儿腹泻时小肠上段细菌显著增多，十二指肠内厌氧菌和酵母菌过度繁殖，由于大量细菌对胆酸的降解作用，使游离胆酸浓度增高，损害小肠细胞，同时阻碍脂肪微粒形成。④营养不良患儿常有肠动力的改变。⑤长期滥用抗生素引起肠道菌群失调。⑥重症营养不良患儿免疫功能缺陷，抗革兰阴性杆菌有效的IgM抗体、起黏膜保护作用的分泌型IgA抗体、吞噬细胞功能和补体水平均降低，因而增加了对病原的易感性，同时降低了对食物蛋白抗原的口服耐受（oral tolerance）。故营养不良婴儿患腹泻时易迁延不愈，持续腹泻又加重了营养不良，两者互为因果，最终引起免疫功能愈发低下，继发感染，形成恶性循环，导致多脏器功能异常。

对于迁延性、慢性腹泻的病因诊断，必须详细询问病史，全面体格检查，正确选用有效的辅助检查，如①粪便常规、肠道菌群分析、大便酸度、还原糖和细菌培养；②十二指肠液检查，分析pH值、胰蛋白酶、糜蛋白酶、肠激酶及血清胰蛋白酶原以判断蛋白质的消化吸收能力，测定十二指肠液的脂酶、胆盐浓度以了解脂肪的消化吸收状况，还可进行寄生虫抗原和寄生虫卵的检测；③小肠黏膜活体组织检查是了解慢性腹泻病理生理变化的最可靠方法。必要时还可做蛋白质、糖类和脂肪的吸收功能试验、X线、结肠镜等检查综合分析判断。

【诊断和鉴别诊断】

根据发病季节、病史（包括喂养史和流行病学资料）、临床表现和大便性状可以作出临床诊断。必须判定有无脱水（程度和性质）、电解质紊乱和酸碱失衡。注意寻找病因，从临床诊断和治疗需要考虑，可先根据大便常规有无白细胞将腹泻分为两组：

1．大便无或偶见少量白细胞者 为侵袭性细菌以外的病因（如病毒、非侵袭性细菌、寄生虫等肠道内、外感染或喂养不当）引起的腹泻，多为水泻，有时伴脱水症状，应与下列疾病鉴别。

（1）“生理性腹泻”：多见于6个月以内婴儿，外观虚胖，常有湿疹，生后不久即出现腹泻，除大便次数增多外，无其他症状，食欲好，不影响生长发育。近年来发现此类腹泻可能为乳糖不耐受的一种特殊类型，添加辅食后，大便即逐渐转为正常。

（2）导致小肠消化吸收功能障碍的各种疾病：如乳糖酶缺乏、葡萄糖-半乳糖吸收不良、失氯性腹泻、原发性胆酸吸收不良、过敏性腹泻等，可根据各病特点进行粪便酸度、还原糖试验、食物过敏原（特异性免疫球蛋白）等检查方法加以鉴别。

2．大便有较多的白细胞者 表明结肠和回肠末端有侵袭性炎症病变，常由各种侵袭性细菌感染所致，仅凭临床表现难以区别，必要时应进行大便细菌培养，细菌血清型和毒性检测，尚需与下列疾病鉴别。

（1）细菌性痢疾：常有流行病学病史，起病急，全身症状重。大便次数多，量少，排脓血便伴里急后重，大便显微镜检查有较多脓细胞、红细胞和吞噬细胞，大便细菌培养有痢疾杆菌生长可确诊。

（2）坏死性肠炎：中毒症状较严重，腹痛、腹胀、频繁呕吐、高热，大便暗红色糊状，渐出现典型的赤豆汤样血便，常伴休克。腹部立、卧位X线摄片呈小肠局限性充气扩张，肠间隙增宽，肠壁积气等。

【治疗】

治疗原则为：调整饮食，预防和纠正脱水，合理用药，加强护理，预防并发症。不同时期的腹泻病治疗重点各有侧重，急性腹泻多注意维持水、电解质平衡及抗感染；迁延及慢性腹泻

则应注意肠道菌群失调及饮食疗法。

1．急性腹泻的治疗

（1）饮食疗法：腹泻时进食和吸收减少，而肠黏膜损伤的恢复，发热时代谢旺盛，侵袭性肠炎丢失蛋白等因素使得营养需要量增加，如限制饮食过严或禁食过久常造成营养不良，并发酸中毒，以致病情迁延不愈影响生长发育。故应强调继续饮食，满足生理需要，补充疾病消耗，以缩短腹泻后的康复时间。有严重呕吐者可暂时禁食4～6 小时（不禁水），好转后继续喂食，由少到多，由稀到稠。病毒性肠炎多有继发性双糖酶（主要是乳糖酶）缺乏，对疑似病例可暂停乳类喂养，改为豆灰、淀粉代乳品，或发酵奶，或去乳糖配方奶粉以减轻腹泻，缩短病程。腹泻停止后逐渐恢复营养丰富的饮食，并每日加餐一次，共2周。

（2）纠正水、电解质紊乱及酸碱失衡：参见第四章第三节。

1）口服补液：口服补液盐（ORS）可用于腹泻时预防脱水及纠正轻、中度脱水。轻度脱水口服液量约50～80 ml/kg，中度脱水约80～100 ml/kg，于8～12 小时内将累积损失量补足。脱水纠正后，可将ORS用等量水稀释按病情需要随意口服。新生儿和有明显呕吐、腹胀、休克、心肾功能不全或其他严重并发症的患儿不宜采用口服补液。

2）静脉补液：适用于中度以上脱水、吐泻严重或腹胀的患儿。输用溶液的成分、量和滴注持续时间必须根据不同的脱水程度和性质决定，同时要注意个体化，结合年龄、营养状况、自身调节功能而灵活掌握。

第1天补液：①总量：包括补充累积损失量、继续损失量和生理需要量，一般轻度脱水约为90～120 ml/kg、中度脱水约为120～150 ml/kg、重度脱水约为150～180 ml/kg，对少数合并营养不良，肺炎、心、肾功能不全的患儿尚应根据具体病情分别作较详细的计算。②溶液种类：溶液中电解质溶液与非电解质溶液的比例应根据脱水性质（等渗性、低渗性、高渗性）分别选用，一般等渗性脱水用1/2 张含钠液、低渗性脱水用2/3张含钠液、高渗性脱水用1/3张含钠液。若临床判断脱水性质有困难时，可先按等渗性脱水处理。③输液速度：主要取决于脱水程度和继续损失的量和速度，对重度脱水有明显周围循环障碍者应先快速扩容，20 ml/kg等渗含钠液，30～60 分钟内快速输入。累积损失量（扣除扩容液量）一般在8～12 小时内补完，约每小时8～10 ml/kg。脱水纠正后，补充继续损失量和生理需要量时速度宜减慢，于12～16 小时内补完，约每小时5 ml/kg。若吐泻缓解，可酌情减少补液量或改为口服补液。④纠正酸中毒：因输入的混合溶液中已含有一部分碱性溶液，输液后循环和肾功能改善，酸中毒即可纠正。也可根据临床症状结合血气测定结果，另加碱性液纠正。对重度酸中毒可用1.4％碳酸氢钠扩容，兼有扩充血容量及纠正酸中毒的作用。⑤纠正低血钾：有尿或来院前6 小时内有尿即应及时补钾；浓度不应超过0.3％；每日静脉补钾时间，不应少于8 小时；切忌将钾盐静脉推入，否则导致高钾血症，危及生命。细胞内的钾浓度恢复正常要有一个过程，因此纠正低钾血症需要有一定时间，一般静脉补钾要持续4～6 天。能口服时可改为口服补充。⑥纠正低血钙、 低血镁：出现低钙症状时可用10％葡萄糖酸钙（每次1～2 ml/kg，最大量≤10 ml）加葡萄糖稀释后静注。低血镁者用25％硫酸镁按每次0.1 mg/kg深部肌肉注射，每6 小时一次，每日3～4 次， 症状缓解后停用。

第二天及以后的补液：经第1天补液后，脱水和电解质紊乱已基本纠正，第2天及以后主要是补充继续损失量（防止发生新的累积损失）和生理需要量，继续补钾，供给热量。一般可改为口服补液。若腹泻仍频繁或口服量不足者，仍需静脉补液。补液量需根据吐泻和进食情况估算，并供给足够的生理需要量，用1/3～1/5 张含钠液补充。继续损失量是按“丢多少补多少”“随时丢随时补”的原则，用1/2～1/3 张含钠溶液补充。将这两部分相加于12～24 小时内均

匀静滴。仍要注意继续补钾和纠正酸中毒的问题。

（3）药物治疗：

1）控制感染 ：①水样便腹泻患者（约占70％）多为病毒及非侵袭性细菌所致，一般不用抗生素，应合理使用液体疗法，选用微生态制剂和黏膜保护剂。如伴有明显中毒症状不能用脱水解释者，尤其是对重症患儿、新生儿、小婴儿和衰弱患儿（免疫功能低下）应选用抗生素治疗。②黏液、脓血便患者（约占30％）多为侵袭性细菌感染，应根据临床特点，针对病原经验性选用抗菌药物，再根据大便细菌培养和药敏试验结果进行调整。大肠杆菌、空肠弯曲菌、耶尔森菌、鼠伤寒沙门菌所致感染常选用抗G^-杆菌抗生素以及大环内酯类抗生素。金黄色葡萄球菌肠炎、假膜性肠炎、真菌性肠炎应立即停用原使用的抗生素，根据症状可选用新青霉素、万古霉素、利福平、甲硝唑或抗真菌药物治疗。

2）肠道微生态疗法：有助于恢复肠道正常菌群的生态平衡，抑制病原菌定植和侵袭，控制腹泻。常用双岐杆菌、嗜酸乳杆菌、粪链球菌、需氧芽胞杆菌、蜡样芽胞杆菌等制剂。

3）肠黏膜保护剂：能吸附病原体和毒素，维持肠细胞的吸收和分泌功能，与肠道黏液糖蛋白相互作用可增强其屏障功能，阻止病原微生物的攻击，如蒙脱石粉。

4）避免用止泻剂，如洛哌丁醇，因为它有抑制胃肠动力的作用，增加细菌繁殖和毒素的吸收，对于感染性腹泻有时是很危险的。

5）补锌治疗：世界卫生组织（WHO）/联合国儿童基金会最近建议，对于急性腹泻患儿，应每日给予元素锌20 mg（>6 个月），疗程10～14 天，6 个月以下婴儿每日10 mg，可缩短病程。

2．迁延性和慢性腹泻治疗 因迁延性和慢性腹泻常伴有营养不良和其它并发症，病情较为复杂，必须采取综合治疗措施。积极寻找引起病程迁延的原因，针对病因进行治疗，切忌滥用抗生素，避免顽固的肠道菌群失调。预防和治疗脱水，纠正电解质及酸碱平衡紊乱。营养治疗，继续喂养对促进疾病恢复，如肠黏膜损伤的修复、 胰腺功能的恢复、微绒毛上皮细胞双糖酶的产生等是必要的治疗措施。

（1）调整饮食：应继续母乳喂养。人工喂养儿应调整饮食，保证足够热能。

（2）双糖不耐受患儿由于有不同程度的原发性或继发性双糖酶缺乏，食用含双糖（包括蔗糖、乳糖、麦芽糖）的饮食可使腹泻加重，其中以乳糖不耐受最多见，治疗宜采用去双糖饮食，如采用豆浆或去乳糖配方奶粉。

（3）过敏性腹泻的治疗：如果在应用无双糖饮食后腹泻仍不改善时，需考虑食物过敏（如对牛奶或大豆蛋白过敏）的可能性，应改用其它饮食或水解蛋白配方饮食。

（4）要素饮食：是肠黏膜受损伤患儿最理想的食物，系由氨基酸、葡萄糖、中链甘油三酯、多种维生素和微量元素组合而成。应用时的浓度和量视患儿临床状态而定。

（5）静脉营养：少数患儿不能耐受口服营养物质者，可采用静脉高营养。推荐方案为：脂肪乳剂每日2～3 g/kg，复方氨基酸每日2～2.5 g/kg，葡萄糖每日12～15 g/kg，电解质及多种微量元素适量，液体每日120～150 ml/kg，热卡每日50～90 cal/kg。病情好转后改为口服。

（6）药物治疗：抗生素仅用于分离出特异病原的感染患儿，并根据药物敏感试验选用。补充微量元素和维生素：如锌、铁、烟酸、维生素A、B_{12}、B_1、C和叶酸等，有助于肠黏膜的修复。应用微生态调节剂和肠黏膜保护剂。

（7）中医辨证论治有良好疗效，并可配合中药、推拿、捏脊、针灸和磁疗等。

【预防】

1．合理喂养，提倡母乳喂养，及时添加辅助食品，每次限一种，逐步增加，适时断奶。

人工喂养者应根据具体情况选择合适的代乳品。

2．对于生理性腹泻的婴儿应避免不适当的药物治疗、不要由于婴儿便次多而怀疑其消化能力，而不按时添加辅食。

3．养成良好的卫生习惯，注意乳品的保存和奶具、食具、便器、玩具和设备的定期消毒。

4．感染性腹泻患儿，尤其是大肠杆菌、鼠伤寒沙门菌、轮状病毒肠炎的传染性强，集体机构如有流行，应积极治疗患者，做好消毒隔离工作，防止交叉感染。

5．避免长期滥用广谱抗生素，对于即使无消化道症状的婴幼儿，在因败血症、肺炎等肠道外感染必须使用抗生素，特别是广谱抗生素时，亦应加用微生态制剂，防止由于难治性肠道菌群失调所致的腹泻。

6．轮状病毒疫苗接种为预防轮状病毒肠炎的理想方法，口服疫苗已见诸报道，保护率在80％以上，但持久性尚待研究。

第九节 婴儿肝炎综合征

婴儿肝炎综合征（infantile hepatitis syndrome）系指一组于婴儿期（包括新生儿期）起病、具有肝细胞性黄疸、肝脏病理体征（肝大、质地异常）和肝功能损伤（主要为血清谷丙转氨酶升高）的临床症候群。病因复杂，主要有宫内和围生期感染、先天性遗传代谢病、肝内胆管发育异常等，由环境、遗传等因素单独或共同造成病变。这类疾病在明确病因之前统称为婴儿肝炎综合征，一旦病因明确，即按原发病因诊断。

【病因及发病机制】

婴儿肝炎综合征的原因包括：

1．感染 包括肝脏的原发性感染和全身感染累及肝脏。临床上所谓的TORCH综合征包括了主要的感染病原，即弓形虫（toxoplasma）、风疹病毒（rubella virus）、巨细胞病毒（cytomegalovirus，CMV）、单纯疱疹病毒（herpes simplex virus，HSV）以及嗜肝病毒、EB病毒、柯萨奇病毒B组、埃可病毒、腺病毒等。细菌感染如金黄色葡萄球菌、大肠杆菌、沙门菌、厌氧菌、肺炎球菌、链球菌等，以及一些条件致病菌，往往在全身感染时累及肝脏。近年来梅毒螺旋体引起肝炎综合征病例有所增加，人类免疫缺陷病毒（HIV）等新的病原体的母婴传播引起肝炎综合征亦应引起注意（参见新生儿感染章节）。

2．先天性代谢异常 先天性代谢异常可以累及肝脏，但只有少数会引起严重的、持续的肝损害。一般来说，有代谢性累积病变都伴有显著的肝大，而有肝损伤者往往为中等度肝大。按其种类包括：

（1）糖类代谢异常：如遗传性果糖不耐受症、半乳糖血症、糖原累积症等。其中与婴儿肝炎综合征相关的糖原累积症主要有Ⅰ、Ⅲ、Ⅳ型（参见遗传代谢病章节）。

（2）氨基酸及蛋白质代谢异常：酶缺陷使正常代谢途径发生阻滞，其中遗传性酪氨酸血症等可以造成持续性肝脏损伤。

（3）脂质代谢异常：系一组遗传性疾病，由于类脂质代谢过程中某些酶的遗传性缺陷，使得原本能被该酶分解的某些类脂质沉积在单核-巨噬细胞系统及其他组织内，呈现充脂性组织细胞增殖。如戈谢病、尼曼-匹克病、Wolman’s病等。

（4）胆汁酸代谢异常：如进行性家族性肝内胆汁淤积症（PFIC）、肝动脉发育不良、Zellweger’s综合征（脑-肝-肾综合征）等。

（5）抗胰蛋白酶缺乏症：是由于抗胰蛋白酶缺乏，中和白细胞弹性蛋白凝固酶等抗蛋白酶作用减弱，使自体组织遭到破坏而致病。可造成肝细胞损伤、汇管区纤维化伴胆管增生以及胆管发育不良等类型改变。

3．先天性胆管闭锁、胆管扩张和肝内胆管发育不良

（1）先天性胆管闭锁：是发生于胎儿后期、生后早期及新生儿期的一种进行性病变，由于某种原因导致肝内和肝外胆管的阻塞，使胆汁排泄的通道梗阻，并逐步形成不同程度的胆道闭锁。多数学者认为围生期感染（特别是病毒感染）所致的炎症病变是导致本病的重要因素，因胆道炎症原因造成先天性胆道闭锁的约占80%，而因先天性胆管发育不良造成胆道闭锁者仅占10%。

（2）先天性胆管扩张症：又称先天性胆总管囊肿，是一种由于多种因素参与的先天性发育畸形。胚胎时期胰胆分化异常，胆总管和胰管未能正常分离，胰液反流入胆管，胆总管远端狭窄，胆道内压力增高，Oddi括约肌神经肌肉功能失调，是本病的综合致病因素。

（3）Caroli病：又称先天性肝内胆管扩张症，为常染色体隐性遗传，以男性多见，一般以复发性胆管炎为主要特点。可伴有先天性肝纤维化，肝外胆管扩张或其他纤维囊性病。

4．其他原因包括肝内占位病变，累及肝脏的全身恶性疾病等。

部分病例病因不明。

【病理】

病因虽多，但主要病理改变为非特异性的多核巨细胞形成。胆汁淤积、肝间质和门脉区有炎症细胞浸润，程度与病情轻重有关。轻者肝小叶结构正常，重者可紊乱失常，肝细胞点状或片状坏死，库普弗细胞和小胆管增生，病情进展门静脉周围可有纤维化。

【临床表现】

主要表现为黄疸。往往因为生理性黄疸持续不退或退而复现就诊。母孕期可有感染（主要是孕早期病毒感染）、服用药物、或有早产、胎膜早破、胎儿宫内发育迟缓等病史。患儿生后可有感染如脐炎、臀炎、皮肤脓疱疮、口腔、呼吸道或消化道感染等。亦可出现其他症状如发热、呕吐、腹胀等。尿色较深，大便由黄转为淡黄，亦可能发白。可有家族肝病史或遗传疾病史。体格检查有肝脾大。多数在3～4 个月内黄疸缓慢消退，也可并发干眼病、低血钙性抽搐、出血和腹泻。少数重症者病程较长可致肝硬化、肝功能衰竭。可伴发其他先天畸形（脐疝、腹股沟疝、先天性心脏病、幽门肥厚等）及生长发育障碍。此外，还有与本综合征有关的原发疾病的临床表现，如消化及神经系统症状及体征。

【辅助检查】

1．全血常规 CMV感染时，可有单个核细胞增多、血小板减少、贫血、溶血等改变。

2．肝功能 结合胆红素和未结合胆红素均有不同程度的增高；谷丙转氨酶升高；甲胎蛋白持续增高则提示肝细胞有破坏，再生增加；血清谷氨酰转肽酶、碱性磷酸酶、5’-核苷酸酶等反映胆管性胆汁淤积的指标增高；反映肝细胞合成功能的指标，如凝血因子和纤维蛋白原、血清白蛋白等降低。

3．病原学检查 病毒感染标记物和相应的病毒学、血清学检查，如肝炎病毒、CMV、EBV、HIV、HSV、风疹病毒等；弓形虫、梅毒螺旋体检查；血、中段尿细菌培养等可提示相应的感染原。

4．疑似遗传代谢、内分泌疾病时，可行血糖测定、尿糖层析、T_3、T_4、TSH、抗胰蛋白酶、尿有机酸、血、尿氨基酸测定、血气分析以及特异性酶学、染色体、基因检查等。

5．影像学检查 做肝、胆、脾B超、肝脏CT或肝胆磁共振胆管成像（MRCP）检查，可显

示相应的畸形或占位病变。

6．肝胆放射性核素扫描 正常^{99m}Tc-EHIDA静脉注射后迅速被肝细胞摄取，3～5 分钟肝脏即清晰显影，左右肝管于5～10 分钟可显影，15～30 分钟胆囊、总胆管及十二指肠开始出现放射性。充盈的胆囊于脂餐后迅速收缩，肝影于12～20 分钟逐渐明显消退，在正常情况下，胆囊及肠道显影均不迟于60 分钟。先天性胆道闭锁时肠道内始终无放射性出现。

7．胆汁引流 可做动态持续十二指肠引流查胆汁常规、细菌培养、胆汁中胆红素、胆汁酸检查。

8．肝活体组织病理检查 可经皮肝穿刺或腹腔镜检查。

【治疗】

婴儿肝炎综合征在查明原因后，应按原发疾病的治疗原则进行治疗，但大多数病例在疾病早期病因较难确定，临床上往往以对症治疗为主。主要包括利胆退黄、护肝、改善肝细胞功能和必要的支持疗法。

1．利胆退黄 苯巴比妥口服具有改善与提高酶活力及促进胆汁排泄作用。也可以用中药利胆治疗（茵陈、山栀、大黄等）。

2．护肝、改善肝细胞功能 ATP、辅酶A有保护肝细胞、促进肝细胞新陈代谢的作用，也可辅以B族维生素及维生素C。还可应用促进肝细胞增生的肝细胞生长因子、保肝解毒的葡醛内酯、促进肝脏解毒与合成功能的还原型谷胱甘肽、降酶作用显著的联苯双酯、甘草酸二铵及补充肠道微生态制剂等。

3．其他处理 低蛋白血症时可用白蛋白制剂；凝血因子缺乏时可用维生素K_1或凝血酶原复合物；有丙种球蛋白低下及反复感染时可用静脉丙种球蛋白；可应用维生素D制剂和钙剂治疗低血钙惊厥和佝偻病；有感染时可适当选用抗生素、抗病毒制剂如更昔洛韦、干扰素等。

4．胆汁分流术及肝移植 如为胆道闭锁及其他原因所致的胆汁淤积、肝纤维化，则应行胆汁分流手术，待条件允许行肝移植术。

（孙 梅）

第十二章 呼吸系统疾病

小儿呼吸道疾病包括上、下呼吸道急慢性感染性疾病，呼吸道变态反应性疾病，胸膜疾病，呼吸道异物，呼吸系统先天畸形及肺部肿瘤等。其中急性呼吸道感染最为常见，约占儿科门诊的 60%以上，在住院患儿中，上、下呼吸道感染占 60%以上，绝大部分为肺炎，且仍是全国 5 岁以下儿童第一位的死亡原因。因此需积极采取措施，降低呼吸道感染的发病率和死亡率。

本章仅介绍小儿呼吸系统解剖、生理特点，急性上、下呼吸道感染性疾病，支气管哮喘。

第一节　小儿呼吸系统解剖生理特点和检查方法

小儿呼吸系统的解剖、生理、免疫特点与小儿时期易患呼吸道疾病密切相关。呼吸系统以环状软骨下缘为界，分为上、下呼吸道。上呼吸道包括鼻、鼻窦、咽、咽鼓管、会厌及喉；下呼吸道包括气管、支气管、毛细支气管、呼吸性细支气管、肺泡管及肺泡。

【解剖特点】

1．上呼吸道

（1）鼻：鼻腔相对短小，鼻道狭窄。婴幼儿鼻黏膜柔嫩并富于血管，感染时黏膜肿胀，易造成堵塞，导致呼吸困难或张口呼吸。

（2）鼻窦：新生儿上颌窦和筛窦极小，2 岁以后迅速增大，至12 岁才充分发育。额窦2～3 岁开始出现，12～13 岁时才发育。蝶窦3 岁时才与鼻腔相通，6 岁时很快增大。由于鼻窦黏膜与鼻腔黏膜相连续，鼻窦口相对大，故急性鼻炎常累及鼻窦，易发生鼻窦炎。

（3）鼻泪管和咽鼓管：婴幼儿鼻泪管短，开口接近于内眦部，且瓣膜发育不全，故鼻腔感染常易侵入结膜引起炎症。婴儿咽鼓管较宽，且直而短，呈水平位，故鼻咽炎时易致中耳炎。

（4）咽部：咽部较狭窄且垂直。扁桃体包括腭扁桃体及咽扁桃体，腭扁桃体1 岁末才逐渐增大，4～10 岁发育达高峰，14～15 岁时渐退化，故扁桃体炎常见于年长儿，婴儿则少见。咽扁桃体又称腺样体，6 个月已发育，位于鼻咽顶部与后壁交界处，严重的腺样体肥大是小儿阻塞性睡眠呼吸暂停综合征的重要原因。

（5）喉：以环状软骨下缘为标志。喉部呈漏斗形，喉腔较窄，声门狭小，软骨柔软，黏膜柔嫩而富有血管及淋巴组织，故轻微炎症即可引起声音嘶哑和吸气性呼吸困难。

2．下呼吸道

（1）气管、支气管：婴幼儿的气管、支气管较成人短且较狭窄，黏膜柔嫩，血管丰富，软骨柔软，因缺乏弹力组织而支撑作用差，因黏液腺分泌不足而气道较干燥，因纤毛运动较差而清除能力差。故婴幼儿容易发生呼吸道感染，一旦感染则易于发生充血、水肿导致呼吸道不畅。左支气管细长，由气管向侧方伸出，而右支气管短而粗，为气管直接延伸，故异物较易进入右支气管。毛细支气管平滑肌在生后5 个月以前薄而少，3 岁以后才明显发育，故小婴儿呼吸道梗阻主要是黏膜肿胀和分泌物堵塞引起。

（2）肺：肺泡数量较少且面积小、弹力纤维发育较差，血管丰富，间质发育旺盛，致肺含血量多而含气量少，易于感染。感染时易致黏液阻塞，引起间质炎症、肺气肿和肺不张等。

3．胸廓 婴幼儿胸廓较短，前后径相对较长，呈桶状；肋骨呈水平位，膈肌位置较高，胸

腔小而肺脏相对较大；呼吸肌发育差。因此在呼吸时，肺的扩张受到限制，尤以肺的后下部受限理甚，不能充分换气，故当肺部病变时，容易出现呼吸困难。小儿纵隔体积相对较大，周围组织松软，在胸腔积液或气胸时易致纵隔移位。

【生理特点】

1．呼吸频率与节律 小儿呼吸频率快，年龄越小，频率越快。新生儿40～44 次/分，～1 岁30 次/分，～3 岁24 次/分，3～7 岁22 次/分，～14 岁20 次/分，～18岁16～18 /分。新生儿及生后数月的婴儿，呼吸极不稳定，可出现深、浅呼吸交替，或呼吸节律不整、间歇、暂停等现象。

2．呼吸型 婴幼儿呼吸肌发育不全，胸廓活动范围小，呼吸时肺主要向膈方向扩张而呈腹膈式呼吸（abdominal respiration）。随年龄增长，膈肌和腹腔脏器下降，肋骨由水平位变为斜位，逐渐转化为胸腹式呼吸（thoracic abdominal respiration）。7岁以后以混合式呼吸为主。

3．呼吸功能特点

（1）肺活量（vital capacity）：小儿肺活量约为50～70 ml/kg。在安静情况下，年长儿仅用肺活量的12.5%来呼吸，而婴幼儿则需用30%左右，说明婴幼儿呼吸功能储备量较小。小儿发生呼吸障碍时其代偿呼吸量最大不超过正常的2.5 倍，而成人可达10 倍，因此易发生呼吸衰竭。

（2）潮气量（tidal volume）：小儿潮气量约为6～10 ml/kg，年龄越小，潮气量越小；死腔/潮气量比值大于成人。

（3）每分钟通气量和气体弥散量：前者按体表面积计算与成人相近；后者按单位肺容积计算与成人相近。

（4）气道阻力：由于气道管径细小，小儿气道阻力大于成人，因此小儿发生喘息的机会较多。随年龄增大气道管径逐渐增大，从而阻力递减。

【呼吸道免疫特点】

小儿呼吸道的非特异性和特异性免疫功能均较差。如咳嗽反射及纤毛运动功能差，难以有效清除吸入的尘埃和异物颗粒。肺泡吞噬细胞功能不足，婴幼儿辅助性T细胞功能暂时性低下，使分泌型IgA、IgG，尤其是IgG_2亚类含量低微。此外，乳铁蛋白、溶菌酶、干扰素及补体等的数量和活性不足，故易患呼吸道感染。

【检查方法】

1．体格检查

（1）视诊：①呼吸频率改变：呼吸困难的第一征象为呼吸频率增快，年龄越小越明显。呼吸频率减慢或节律不规则也是危险征象。②发绀（cyanosis）：肢端发绀为末梢性发绀，舌、黏膜的发绀为中心性发绀。中心性发绀较末梢性发绀发生晚，但更有意义。③吸气时胸廓软组织凹陷：上呼吸道梗阻或严重肺病变时，胸骨上、下，锁骨上窝及肋间隙软组织凹陷，称为“三凹征”（three depressions sign）。④其他：小婴儿呼吸困难时常有呻吟、鼻扇和口吐泡沫等表现。

（2）吸气喘鸣（inspiratory stridor）和呼气喘息（expiratory wheeze）：吸气时出现喘鸣音，同时伴吸气延长，是上呼吸道梗阻的表现。呼气时出现喘鸣音，同时伴呼气延长，是下呼吸道梗阻的表现。

（3）肺部听诊：哮鸣音常于呼气相明显，提示细小支气管梗阻。不固定的中、粗湿啰音常来自支气管的分泌物。于吸气相，特别是深吸气末，听到固定不变的细湿啰音提示肺泡内存在分泌物，常见于肺泡炎。小婴儿因呼吸浅快，啰音可不明显，刺激其啼哭方可在吸气末闻及。

2．血气分析 反映气体交换和血液的酸碱平衡状态，为诊断和治疗提供依据。小儿血气分析正常值见表12-1。

表12-1 小儿血液气体分析正常值

项　目	新　生　儿	~2 岁	>2 岁
pH值	7.35～7.45	7.35～7.45	7.35～7.45
PaO_2（kPa）	8～12	10.6～13.3	10.6～13.3
$PaCO_2$（kPa）	4.00～4.67	4.00～4.67	4.67～6.00
HCO_3^-（mmol/L）	20～22	20～22	22～24
BE（mmol/L）	-6～+2	-6～+2	-4～+2
SaO_2（%）	90～97	95～97	96～98

当动脉血氧分压（PaO_2）<50 mmHg（6.67 kPa），动脉二氧化碳分压（$PaCO_2$）>50 mmHg（6.67 kPa），动脉血氧饱和度（Sa O_2）<85%时为呼吸衰竭。

3．肺脏影像学 胸部平片仍为呼吸系统疾病影像学诊断的基础，可基本满足70%以上的临床需要。胸透对儿童生长发育影响较大，目前已经不用于儿童常规检查。CT特别是高分辨率CT（HRCT）和螺旋CT（spiral CT）的发展，小儿呼吸系统疾病的诊断率已大为提高。

4．儿童纤维支气管镜检查 利用纤维支气管镜和电子支气管镜不仅能直视气管和支气管内的各种病变，还能利用黏膜刷检技术、活体组织检查技术和肺泡灌洗技术提高对儿童呼吸系统疾病的诊断率。

5．肺功能检查 5 岁以上儿童可作较全面的肺功能检查。脉冲振荡技术的优点是受试者可以自由呼吸，无需配合，无创作性，特别适用于儿童和重症患者的肺功能检查。应用潮气-流速容量曲线（TFV）技术使婴幼儿肺功能检查成为可能。

第二节 急性上呼吸道感染

急性上呼吸道感染（acute upper respiratory infection，AURI）系由各种病原引起的上呼吸道的急性感染（简称上感），俗称“感冒”，是小儿最常见的疾病。该病主要侵犯鼻、鼻咽和咽部，根据主要感染部位的不同可诊断为急性鼻炎、急性咽炎、急性扁桃体炎等。可见，急性上呼吸道感染就是上呼吸道局部感染的说法并不确切。

【病因】

各种病毒和细菌均可引起急性上呼吸道感染，但90%以上为病毒，主要有鼻病毒（rhinovirus，RV）、呼吸道合胞病毒（respiratory syncytial virus，RSV）、流感病毒（influenza virus）、副流感病毒（parainfluenza virus）、腺病毒（adenovirus，ADV）、冠状病毒（coronal virus）等。病毒感染后可继发细菌感染，最常见为溶血性链球菌，其次为肺炎链球菌\流感嗜血杆菌等。肺炎支原体（mycoplasma pneumoniae）不仅可引起肺炎，也可引起上呼吸道感染。

婴幼儿时期由于上呼吸道的解剖和免疫特点而易患本病。营养障碍性疾病，如维生素D缺乏性佝偻病、亚临床维生素A、锌或铁缺乏症等，或免疫缺陷病、被动吸烟、护理不当、气候改变和环境不良等因素，则易发生反复上呼吸道感染或使病程迁延。

【临床表现】

由于年龄大小、体质强弱及病变部位的不同，病情的缓急、轻重程度也不同。年长儿症状

较轻，婴幼儿则较重。

1．一般类型上感

（1）症状：

1）局部症状：鼻塞、流涕、喷嚏、干咳、咽部不适和咽痛等，多于3～4 天内自然痊愈。

2）全身症状：发热、烦躁不安、头痛、全身不适、乏力等。部分患儿有食欲不振、呕吐、腹泻、腹痛等消化道症状。腹痛多为脐周阵发性疼痛，无压痛，可能为肠痉挛所致；如腹痛持续存在，多为并发急性肠系膜淋巴结炎。

婴幼儿起病急，全身症状为主，常有消化道症状，局部症状较轻。多有发热，体温可高达39～40℃，热程2～3 天至1 周左右，起病1～2 天可因高热引起惊厥。

（2）体征：体格检查可见咽部充血，扁桃体肿大。有时可见下颌和颈淋巴结肿大。肺部听诊一般正常。肠道病毒感染者可见不同形态的皮疹。

2．两种特殊类型上感

（1）疱疹性咽峡炎（herpangina）：病原体为柯萨奇A组病毒。好发于夏秋季。起病急骤，临床表现为高热、咽痛、流涎、厌食、呕吐等。体格检查可发现咽部充血，在咽腭弓、软腭、悬雍垂的黏膜上可见数个至十数个2～4 mm大小灰白色的疱疹，周围有红晕，1～2 日后破溃形成小溃疡，疱疹也可发生于口腔的其他部位。病程为1 周左右。

（2）咽结合膜热（pharyngo-cojunctival fever）：病原体为腺病毒3、7型。以发热、咽炎、结膜炎为特征。好发于春夏季，散发或发生小流行。临床表现为高热、咽痛、眼部刺痛，有时伴消化道症状。体检发现咽部充血、可见白色点块状分泌物，周边无红晕，易于剥离；一侧或双侧滤泡性眼结合膜炎，可伴球结合膜出血；颈及耳后淋巴结增大。病程1～2 周。

【并发症】

以婴幼儿多见，病变若向邻近器官组织蔓延可引起中耳炎、鼻窦炎、咽后壁脓肿、扁桃体周围脓肿、颈淋巴结炎、喉炎、支气管炎及肺炎等。年长儿若患A组溶血性链球菌咽峡炎，以后可引起急性肾小球肾炎和风湿热，其他病原体也可引起类风湿病等结缔组织病。

【实验室检查】

病毒感染者外周血白细胞计数正常或偏低，中性粒细胞减少，淋巴细胞计数相对增高。病毒分离和血清学检查可明确病原。近年来免疫荧光、免疫酶及分子生物学技术可做出早期诊断。

细菌感染者外周血白细胞可增高，中性粒细胞增高，在使用抗菌药物前行咽拭子培养可发现致病菌。C反应蛋白（CRP）和前降钙素原（PCT）有助于鉴别细菌感染。

【诊断和鉴别诊断】

根据临床表现一般不难诊断，但需与以下疾病鉴别：

1．流行性感冒 简称流感，由流感病毒、副流感病毒引起。有明显的流行病史，局部症状较轻，全身症状较重。常有高热、头痛、四肢肌肉酸痛等，病程较长。

2．急性传染病早期 上感常为各种传染病的前驱症状，如麻疹、流行性脑脊髓膜炎、百日咳、猩红热等，应结合流行病史、临床表现及实验室资料等综合分析，并观察病情演变加以鉴别。

3．急性阑尾炎 伴腹痛者应注意与急性阑尾炎鉴别。本病腹痛常先于发热，腹痛部位以右下腹为主，呈持续性，有固定压痛点、反跳痛及腹肌紧张、腰大肌试验阳性等体征，白细胞及中性粒细胞增高。

4．过敏性鼻炎 某些学龄前或学龄儿童“感冒”症状如流涕、打喷嚏持续超过2 周或反复发作，而全身症状较轻，则应考虑过敏性鼻炎的可能，鼻拭子涂片嗜酸性粒细胞增多有助于诊

断。

在排除上述疾病后，尚应对上呼吸道感染的病因进行鉴别，以便指导治疗。

【治疗】

1．一般治疗 病毒性上呼吸道感染者，应告诉患儿家长该病的自限性和治疗的目的，防止交叉感染及并发症。注意休息、保持良好的周围环境、多饮水和补充大量维生素C等。

2．抗感染治疗

（1）抗病毒药物：大多数上呼吸道感染由病毒引起，可试用利巴韦林（病毒唑，virazole），剂量为10～15mg/（kg·d），口服或静脉点滴，或2 mg含服，每2 小时一次，每日6 次，3～5 日为一疗程。若为流感病毒感染，可用磷酸奥司他韦口服。合并结膜炎者，可用0.1%阿昔洛韦滴眼液滴眼。

（2）抗生素：细菌性上呼吸道感染或病毒性上呼吸道感染继发细菌感染者可选用抗生素治疗，常选用青霉素类、头孢菌素类、复方新诺明及大环内酯类抗生素。咽拭子培养阳性结果有助于指导抗菌治疗。若证实为链球菌感染，或既往有风湿热、肾炎病史者，青霉素疗程应为10～14 日。

3．对症治疗

（1）高热可口服对乙酰氨基酚或布洛芬，亦可用冷敷、温湿敷或酒精浴降温。

（2）发生高热惊厥者可予以镇静、止惊等处理。

（3）咽痛可含服咽喉片。

（4）中成药亦有较好的效果。

【预防】

主要靠加强体格锻炼以增强抵抗力；提倡母乳喂养；避免被动吸烟；防治佝偻病及营养不良；避免去人多拥挤的公共场所。

第三节 急性感染性喉炎

急性感染性喉炎（acute infectious laryngitis）是指喉部黏膜急性弥漫性炎症。以犬吠样咳嗽、声嘶、喉鸣、吸气性呼吸困难为临床特征。冬春季节多发，且多见于婴幼儿。

【病因】

由病毒或细菌感染引起，亦可并发于麻疹、百日咳和流感等急性传染病。常见的病毒为副流感病毒、流感病毒和腺病毒，常见的细菌为金黄色葡萄球菌、链球菌和肺炎链球菌。由于小儿喉部解剖特点，炎症时易充血、水肿而出现喉梗阻。

【临床表现】

起病急、症状重。可有发热、犬吠样咳嗽、声嘶、吸气性喉鸣和三凹征。严重时可出现发绀、烦躁不安、面色苍白、心率加快。咽部充血，间接喉镜检查可见喉部、声带有不同程度的充血、水肿。一般白天症状轻，夜间入睡后加重，喉梗阻者若不及时抢救，可窒息死亡。

按吸气性呼吸困难的轻重，将喉梗阻分为四度：Ⅰ 度：患者仅于活动后出现吸气性喉鸣和呼吸困难，肺部听诊呼吸音及心率无改变；Ⅱ度：于安静时亦出现喉鸣和吸气性呼吸困难，肺部听诊可闻喉传导音或管状呼吸音，心率加快；Ⅲ度：除上述喉梗阻症状外，患儿因缺氧而出现烦躁不安，口唇及指趾发绀，双眼圆睁，惊恐万状，头面部出汗，肺部呼吸音明显降低，

心率快，心音低钝；Ⅳ度：患儿渐显衰竭、昏睡状态，由于无力呼吸，三凹征可不明显，面色苍白发灰，肺部听诊呼吸音几乎消失，仅有气管传导音，心律不齐，心音钝、弱。

【诊断和鉴别诊断】

根据急起犬吠样咳嗽、声嘶、喉鸣、吸气性呼吸困难等临床表现不难诊断，但应与白喉、急性会厌炎、喉痉挛、喉或气管异物、喉先天畸形等所致的喉梗阻鉴别。

【治疗】

1．保持呼吸道通畅 可用1％～3％麻黄素和吸入型糖皮质激素如丁地去炎松溶液雾化吸入，促进黏膜水肿消退。

2．控制感染 及时静脉输入足量抗生素，一般给予青霉素、大环内酯类或头孢菌素类等，严重者予以两种以上抗生素。

3．糖皮质激素 有抗炎和抑制变态反应等作用，能及时减轻喉头水肿，缓解喉梗阻。病情较轻者可口服泼尼松，Ⅱ度喉梗阻以上的患儿应给予静点地塞米松、氢化可的松或甲泼尼龙。

4．对症治疗 缺氧者予以吸氧；烦躁不安者可用异丙嗪，除镇静外还有减轻喉头水肿的作用；痰多者可选用祛痰剂，必要时直接喉镜吸痰；不宜使用氯丙嗪和吗啡。

5．气管切开 经上述处理仍有严重缺氧征象或有Ⅲ度以上喉梗阻者，应及时行气管切开术。

第四节 急性支气管炎

急性支气管炎（acute bronchitis）是指由于各种致病原引起的支气管黏膜炎症，由于气管常同时受累，故称为急性气管支气管炎（acute tracheobronchitis）。常继发于上呼吸道感染或为急性传染病的一种表现。是儿童时期常见的呼吸道疾病，婴幼儿多见。

【病因】

病原为各种病毒或细菌，或为混合感染。能引起上呼吸道感染的病原体都可引起支气管炎。免疫功能低下、特应性体质、营养障碍、佝偻病和支气管局部结构异常等均为本病的危险因素。

【临床表现】

大多先有上呼吸道感染症状，之后以咳嗽为主要症状，开始为干咳，以后有痰。婴幼儿症状较重，常有发热、呕吐及腹泻等。一般无全身症状。双肺呼吸音粗糙，可有不固定的散在的干啰音和粗中湿啰音。婴幼儿有痰常不易咳出，可在咽喉部或肺部闻及痰鸣音。

婴幼儿期伴有喘息的支气管炎，如伴有湿疹或其他过敏史者，少数可发展为哮喘。

【治疗】

1．一般治疗 同上呼吸道感染，经常变换体位，多饮水，使呼吸道分泌物易于咳出。

2．控制感染 由于病原体多为病毒，一般不采用抗生素。怀疑有细菌感染者则可用β-内酰胺类抗生素，如系支原体感染，则应予以大环内酯类抗生素。

3．对症治疗 应使痰易于咳出，故不用镇咳剂。①祛痰药：如N-乙酰半胱氨酸、氨溴索、愈创木酚甘油醚和一些中药制剂等；②止喘：对喘憋严重者，可雾化吸入沙丁胺醇等β_2受体激动剂，或用氨茶碱口服或静脉给药。喘息严重者可短期使用糖皮质激素，如口服泼尼松3～5 天；③抗过敏：可选用马来酸氯苯那敏和盐酸异丙嗪等抗过敏药物。

第五节 毛细支气管炎

毛细支气血管炎（bronchiolitis）是一种婴幼儿较常见的下呼吸道感染，多见于1～6 个月的小婴儿，以喘憋、三凹征和气促为主要临床特点。临床上较难发现未累及肺泡与肺泡间壁的纯粹毛细支气管炎，故国内认为是一种特殊类型的肺炎，有人称之为喘憋性肺炎。

【病因】

主要由呼吸道合胞病毒（RSV）引起，副流感病毒、鼻病毒、人类偏肺病毒（human meta-pneumovirus，hMPV）、某些腺病毒及肺炎支原体也可引起本病。

【发病机制】

研究较多的是免疫学机制，几个事实可以表明在RSV引起的毛细支气管炎的发病机制中存在免疫损害：①恢复期的毛细支气管炎婴儿的分泌物中发现有抗RSV IgE抗体；②近来对感染RSV的婴儿与动物模型的研究表明，在RSV感染时有大量的可溶性因子的释放（包括白介素、白三烯、趋化因子）导致炎症与组织破坏；③经胃肠道外获得高抗原性、非活化的RSV疫苗的儿童，在接触野毒株RSV时比对照组更容易发生严重的毛细支气管炎。

目前认为具有特应质或过敏体质（atopy）者，发生RSV或其他病毒感染时，更易于引起毛细支气管炎。毛细支气管炎患者日后发生反复喘息发作，甚至形成哮喘的机制尚不完全清楚。

【病理】

病变主要侵犯直径75～300μm的毛细支气管，表现为上皮细胞坏死和周围淋巴细胞浸润，黏膜下充血、水肿和腺体增生、黏液分泌增多。病变会造成毛细支气管腔狭窄甚至堵塞，导致肺气肿和肺不张。炎症还可波及肺泡、肺泡壁及肺间质，出现通气和换气功能障碍。

【临床表现】

本病发生于2 岁以下小儿，多数在6 个月以内，常为首次发作。喘憋和肺部哮鸣音为其突出表现。主要表现为下呼吸道梗阻症状，出现呼气性呼吸困难，呼气相延长伴喘鸣。呼吸困难可呈阵发性，间歇期呼气性哮鸣消失。严重发作者，可见面色苍白、烦躁不安，口周和口唇发绀。全身中毒症状较轻，可无热、低热、中度发热，少见高热。体格检查发现呼吸浅而快，60～80 次/分，甚至100 次/分，伴鼻翼扇动和三凹征；心率加快，可达150～200 次/分。肺部体征主要为呼气相哮鸣音，亦可闻及中、细湿啰音，叩诊可呈鼓音。肝脾可由于肺气肿而推向肋缘下，因此可触及肝脏和脾脏。重度喘憋者可有PaO_2降低，$PaCO_2$升高。本病高峰期在呼吸困难发生后的48～72 小时，病程一般约为1～2 周。

【辅助检查】

外周血白细胞总数及分类大多在正常范围内。采集鼻咽拭子或分泌物使用免疫荧光技术、免疫酶技术及分子生物学技术可明确病原。

X线胸部检查可见不同程度肺气肿或肺不张，也可以见到支气管周围炎及肺纹理增粗。血气分析可了解患儿缺氧和CO_2潴留程度。

【诊断与鉴别诊断】

根据本病发生在小婴儿，具有典型的喘憋及喘鸣音，一般诊断不难，但须与以下疾病鉴别。

1．儿童哮喘 婴儿的第一次感染性喘息发作，即为毛细支气管炎，但若三次以上，则应考虑为婴幼儿哮喘的可能。毛细支气管炎发展为哮喘的危险因素包括过敏体质、哮喘家庭史、抗RSV-IgE升高、先天性小气道、被动吸烟等。

2．原发型肺结核 常伴有喘息，可闻及哮鸣音，可根据结核接触史、结核中毒症状、结核菌素试验和胸部X线改变予以鉴别。

3．其他疾病 如纵隔占位、充血性心力衰竭、心内膜弹力纤维增生症、异物吸入及先天性气管支气管畸形等均可发生喘息，应结合病史和体征及必要的检查作出鉴别。

【治疗】

毛细支气管炎的治疗主要为氧疗、控制喘憋、病原治疗及免疫疗法。

1．氧疗 重症患儿可采用不同方式吸氧，如鼻前庭导管给氧、面罩或氧帐等。

2．控制喘憋 重症患儿可用沙丁胺醇喷射雾化吸入。糖皮质激素用于严重的喘憋发作或其他治疗不能控制者，琥珀酸氢化可的松5～10 mg/（kg·d）或甲泼尼松龙1～2mg/（kg·d），数小时内静脉滴入。也可采用喷射雾化吸入吸入型糖皮质激素（如丁地去炎松等）。

3．抗病原体药物治疗 如系病毒感染所致，可用利巴韦林静脉滴注或雾化吸入；亦可试用α-干扰素肌注，但其疗效均不肯定。支原体感染者可应用大环内酯类抗生素，有细菌感染者应用适当的抗生素。

4．生物制品治疗 静脉注射免疫球蛋白（IVIG）400rng/（kg·d），连续3～5 天，可缓解临床症状，减少患儿排毒量和缩短排毒期限。静脉注射抗合胞病毒免疫球蛋白（RSV-IVIG）的疗效与IVIG 相当，抗RSV 单克隆抗体（Palivizumab®）对高危婴儿（早产儿、支气管肺发育不良、先天性心脏病、免疫缺陷病）和毛细支气管炎后反复喘息发作者的预防效果确切，但容易导致RSV发生基因突变，而对该单克隆抗体产生抗性。

5．其他 保持呼吸道通畅，保证液体摄入量、纠正酸中毒，并及时发现和处理呼吸衰竭及其他生命体征危象，具体参见支气管肺炎治疗内容。

第六节 支气管哮喘

支气管哮喘（bronchial asthma）简称哮喘，是儿童期最常见的慢性呼吸道疾病。哮喘是由多种细胞（如嗜酸性粒细胞、肥大细胞、T淋巴细胞、中性粒细胞及气道上皮细胞等）和细胞组分共同参与的气道慢性炎症性疾病，这种慢性炎症导致气道反应性的增加，通常出现广泛多变的可逆性气流受限，并引起反复发作性喘息、气促、胸闷或咳嗽等症状，常在夜间和（或）清晨发作或加剧，多数患儿可经治疗缓解或自行缓解。全球约有1.6亿患者，各国患病率在1%～13%不等，发达国家高于发展中国家，城市高于农村。2000年中国城区儿童哮喘病率调查显示儿童哮喘患病率为1.97%，2 年现患率为1.54%。70%～80%的儿童哮喘发病于5岁以前，约20%的患者有家族史，特应质或过敏体质（atopy）对本病的形成关系很大，多数患者有婴儿湿疹、过敏性鼻炎和（或）食物（药物）过敏史。儿童哮喘如诊治不及时，随病程的延长可产生气道不可逆性狭窄和气道重塑。因此，早期防治至关重要。为此，世界卫生组织（WHO）与美国国立卫生研究院心肺血液研究所制定了全球哮喘防治创议（Global Initiative For Asthma，GINA）方案，该方案不断更新，目前已成为防治哮喘的重要指南。

【发病机理】

哮喘的发病机理极为复杂，尚未完全清楚，与免疫、神经、精神、内分泌因素和遗传学背景密切有关。

1．免疫因素 气道慢性炎症被认为是哮喘的本质。自19世纪90年代以来，通过大量临床病理研究发现，无论病程长短、病情轻重，哮喘患者均存在气道慢性炎症性改变。新近的研究表明哮喘的免疫学发病机制为：Ⅰ型树突状细胞（DCⅠ）成熟障碍，分泌IL-12不足，使T_H0不能

向T_H1细胞分化；在IL-4诱导下CDⅡ促进T_H0 细胞向T_H2发育，导致T_H1（分泌IFN-γ减少）/T_H2（分泌IL-4增高）细胞功能失衡。T_H2细胞促进B细胞产生大量IgE（包括抗原特异性IgE）和分泌炎症性细胞因子（包括黏附分子）刺激其他细胞（如上皮细胞、内皮细胞、嗜碱细胞、肥大细胞和嗜酸细胞等）产生一系列炎症介质（如白三烯、内皮素、前列腺素和血栓素A_2等），最终诱发速发型（IgE增高）变态反应和慢性气道炎症。

2．神经、精神和内分泌因素 哮喘患儿的β-肾上腺素能受体功能低下和迷走神经张力亢进，或同时伴有α-肾上腺能神经反应性增强，从而发生气道高反应性（airway hyperresponsiveness，AHR）。气道的自主神经系统除肾上腺素能和胆碱能神经系统外，尚存在第三类神经，即非肾上腺素能非胆碱能（nonadreneergic noncholinergic，NANC）神经系统。NANC神经系统又分为抑制性NANC神经系统（i-NANC）及兴奋性NANC神经系统（e-NANC），两者平衡失调，则可引起支气管平滑肌收缩。

3．遗传学背景 哮喘具有明显遗传倾向，患儿及其家庭成员患过敏性疾病和特应性体质者明显高于正常人群。哮喘为多基因遗传性疾病，已发现许多与哮喘发病有关的基因（疾病相关基因），如IgE、IL-4、IL-13、T细胞抗原受体（TCR）等基因多态性。但是，哮喘发病率在近30 年来明显增高，不能单纯以基因变异来解释。

【危险因素】

1．吸入过敏原（室内：尘螨、动物毛屑及排泄物、蟑螂、真菌等；室外：花粉、真菌等）。

2．食入过敏原（牛奶、鱼、虾、鸡蛋和花生等）。

3．呼吸道感染（尤其是病毒及支原体感染）。

4．强烈的情绪变化。

5．运动和过度通气。

6．冷空气。

7．药物（如阿司匹林等）。

8．职业粉尘及气体。

以上为诱发哮喘症状的常见危险因素，有些因素只引起支气管痉挛，如运动及冷空气。有些因素可以突然引起哮喘的致死性发作，如药物及职业性化学物质。

【病理和病理生理】

哮喘死亡患儿的肺组织呈肺气肿，大、小气道内填满黏液栓。黏液栓由黏液、血清蛋白、炎症细胞和细胞碎片组成。显微镜显示支气管和毛细支气管上皮细胞脱落，管壁嗜酸性粒细胞和单核细胞浸润，血管扩张和微血管渗漏，基底膜增厚，平滑肌增生肥厚，杯状细胞和黏膜下腺体增生。

气流受阻是哮喘病理生理改变的核心，支气管痉挛、管壁炎症性肿胀、黏液栓形成和气道重塑均是造成患儿气道受阻的原因。

1．支气管痉挛 急性支气管痉挛为速发型哮喘反应，是IgE依赖型介质释放所致（Ⅰ型变态反应），包括肥大细胞释放组胺、前列腺素和白三烯等。

2．管壁炎症性肿胀 抗原对气道刺激后6～24 小时发生的气道直径减小，是微血管通透性和漏出物增加导致气道黏膜增厚和肿胀所致。伴随或不伴随平滑肌收缩，为迟发型哮喘反应。

3．黏液栓形成 主要发生于迟发型哮喘，黏液分泌增多，形成黏液栓，重症病例黏液栓广泛阻塞细小支气管，引起严重呼吸困难，甚至发生呼吸衰竭。

4．气道重塑 因慢性和反复的炎症损害，可以导致气道重塑（airway remodelling），表现为

气道壁增厚和基质沉积、胶原沉积，上皮下纤维化，平滑肌增生和肥大，肌成纤维细胞增殖及黏液腺杯状细胞化生及增生，上皮下网状层增厚，微血管生成。

气道高反应（airway hyperresponsiveness，AHR）是哮喘的基本特征之一，指气道对多种刺激因素，如过敏原、理化因素、运动和药物等呈现高度敏感状态，在一定程度上反映了气道炎症的严重性。气道炎症通过气道上皮损伤、细胞因子和炎症介质的作用引起AHR。

【临床表现】

咳嗽和喘息呈阵发性发作，以夜间和清晨为重。发作前可有流涕、打喷嚏和胸闷，发作时呼吸困难，呼气相延长伴有喘鸣声。严重病例呈端坐呼吸，恐惧不安，大汗淋漓，面色青灰。

体格检查可见桶状胸、三凹症，肺部满布哮鸣音，严重者气道广泛堵塞，哮鸣音反可消失，称“闭锁肺”（silent lung），是哮喘最危险的体征。肺部粗湿啰音时现时隐，在剧烈咳嗽后或体位变化时可消失，提示湿啰音的产生是位于气管内的分泌物所致。在发作间歇期可无任何症状和体征，有些病例在用力时才可听到哮鸣音。此外在体格检查时还应注意鼻炎、鼻窦炎和湿疹。

哮喘发作在合理应用常规缓解药物治疗后，仍有严重或进行性呼吸困难者，称为哮喘危重状态（哮喘持续状态，status asthmaticus）。表现为哮喘急性发作，出现咳嗽、喘息、呼吸困难、大汗淋漓和烦躁不安，甚至表现出端坐呼吸、语言不连贯、严重发绀、意识障碍及心肺功能不全的征象。

【辅助检查】

1．肺功能检查 肺功能检查主要用于5 岁以上的患儿，采用FEV_1/用力肺活量（FVC）比率、呼气峰流速（PEF）了解有无气流受阻。FEV_1/FVC<70％～75％提示气流受阻，吸入支气管扩张剂15～20 分钟后增加15％或更多表明为可逆性气流受阻，是诊断哮喘的有利依据。PEF的日间变异率是诊断哮喘和反映哮喘严重程度的重要指标。如日间变异率>20%、使用支气管扩张剂后变异率增加20%可以诊断为哮喘。也可用组胺或乙酰甲胆碱激发试验。

2．胸部X线检查 急性期胸片正常或呈间质性改变，可有肺气肿或肺不张。胸片还可排除肺部其他疾病，如肺炎、肺结核、气管支气管异物和先天性畸形等。

3．过敏原测试 用多种吸入性过敏原或食物性过敏原提取液所做的过敏原皮肤试验是诊断变态反应的首要工具，提示患者对该过敏原过敏与否。目前常用皮肤点刺试验法和皮内试验法。血清特异性IgE测定也很有价值，血清总IgE测定只能反映是否存在特应质。

【诊断和鉴别诊断】

1．诊断 根据GINA 2002 版方案并结合我国国情，中华医学会儿科学分会呼吸学组于2003年制定了我国的“儿童支气管哮喘防治常规（试行）”，修订了儿童哮喘和咳嗽变异性哮喘的诊断标准。

（1）儿童哮喘诊断标准：

1）反复发作的喘息、气促、胸闷或咳嗽，多与接触变应原、冷空气、物理或化学性刺激、病毒性上、下呼吸道感染、运动等有关。

2）发作时双肺可闻及散在或弥漫性以呼气相为主的哮鸣音，呼气相延长。

3）支气管舒张剂有显著疗效。

4）除外其他疾病所引起的喘息、气促、胸闷或咳嗽。

5）对于症状不典型的患儿，同时在肺部闻及哮鸣音者，可酌情采用以下任何1项支气管舒张试验协助诊断，若阳性可诊断为哮喘：①速效β_2受体激动剂雾化溶液或气雾剂吸入；②以0.1%

肾上腺素0.01 ml/kg皮下注射（最大不超过0.3 ml/次）。在进行以上任何1种试验后的15～30 min内，如果喘息明显缓解，哮鸣音明显减少者为阳性。5岁以上患儿若有条件可在治疗前后测呼气峰流速（PEF）或第1秒用力呼气容积（FEV_1），治疗后上升≥15%者为阳性。如果肺部未闻及哮鸣音，且FEV_1>75%者，可做支气管激发试验，若阳性可诊断为哮喘。

（2）咳嗽变异型哮喘标准：

1）持续咳嗽>1 个月,常在夜间和（或）清晨发作，运动、遇冷空气或嗅到特殊气味后加重，痰少，临床上无感染征象，或经较长时间抗生素治疗无效。

2）支气管舒张剂诊断性治疗可使咳嗽发作缓解（基本诊断条件）。

3）有个人或家族过敏史、家族哮喘病史，过敏原检测阳性可作辅助诊断。

4）排除其他原因引起的慢性咳嗽。

由于年幼儿患哮喘其临床特点、治疗及其预后均有别于年长儿，中华儿科学会呼吸学组1988 年提出婴幼儿哮喘诊断标准，从最初的8 项评分到1992 年的5 项评分，直至1998 年的不评分诊断。婴幼儿哮喘诊断的提出对我国儿童哮喘的早期诊断和防治起到了积极的作用。但是根据GINA方案以及美国、英国等许多国家的儿童哮喘诊疗指南，哮喘可以发生于儿童的各个年龄段，所以儿童哮喘的诊断不应以年龄诊断，2003 年我国儿童哮喘防治常规中没有单独列出婴幼儿哮喘。尽管不以年龄命名诊断哮喘，仍需要强调在哮喘诊断、鉴别诊断、检查、治疗等方面，儿童不同年龄段存在的不同特点。

2．哮喘的分期与病情的评价 哮喘可分为急性发作期（exacerbation）、慢性持续期（persistent）和临床缓解期（remission）。急性发作期指患者出现以喘息为主的各种症状，其发作持续的时间和程度不尽相同，哮喘急性发作时严重程度评估见表12-2。慢性持续期指许多患者即使没有急

表12-2 哮喘急性发作期病情严重程度的分级

临床特点	轻　度	中　度	重　度	急性呼吸暂停
呼吸急促	走路时	稍事活动时	休息时	
体位	可平卧	喜坐位	前弓位	
讲话能力	能成句	成短句	说单字	难以说话
精神意识	可	时有焦虑、烦躁	焦虑、烦躁	嗜睡、意识模糊
出汗	无	轻微	大汗淋漓	
呼吸频率	轻度增加	增加	明显增加	减缓或暂停
辅助呼吸肌活动及三凹征	一般没有	通常有	通常有	胸腹矛盾运动
哮鸣音	散在，呼吸末期出现	响亮、弥漫	响亮、弥漫	减弱乃至消失
脉率（次/分）（>8岁）	<100	100～120	≥120	减慢，不规则
吸入速效$β_2$激动剂后PEF占正常预计值或本人最佳值百分比（%）	>80	60～80	≤60或$β_2$激动剂作用持续时间<2 小时	
PaO2（吸空气，kPa）	正常	>8.0	<8.0 可能有呼吸衰竭	
SaO2（吸空气，%）	>95	91～95	≤90	
pH				降低

注：多个参数可同时出现，但不一定全部均有；1 kPa=7.5 mmHg。

性发作，但在相当长的时间内总是不同频度和（或）不同程度地出现症状（喘息、咳嗽和胸闷），因此需要依据就诊前日间症状、夜间症状和肺功能情况对其病情进行评价，分成4 级（表12-3）。临床缓解期指经过治疗或未经治疗症状和体征消失，肺功能（FEV_1或PEF）≥80%预计值，并维持4 周以上。

表12-3 哮喘慢性持续期病情严重程度分级

级　　别	日间症状	夜间症状	PEF或FEV_1占预计值（%）	PEF变异率（%）
一级（轻度间歇）	<1 次/周，发作间歇无症状	≤2 次/月	≥80	<20
二级（轻度持续）	≥1 次/周，<1 次/天，发作时可能影响活动	>2 次/月	≥80	20～30
三级（中度持续）	每日有症状，影响活动	>1 次/周	60～80	>30
四级（重度持续）	持续有症状，体力活动受限	频繁	≤60	>30

注：①患儿只要具有某级严重程度的一个特点，就可将其列为该级别，即严重程度按最严重一项来确定。
②患儿属于任何一级，甚至间歇发作，都可以有严重的哮喘发作。

3．鉴别诊断 以喘息为主要症状的儿童哮喘应注意与毛细支气管炎、肺结核、气道异物、先天性气管支气管畸形和先天性心血管疾病相鉴别，咳嗽变异型哮喘（CVA）应注意与支气管炎、鼻窦炎、胃食管反流和嗜酸性粒细胞支气管炎等疾病相鉴别。

【治疗】

哮喘的治疗目标：①有效控制急性发作症状，并维持最轻的症状，甚至无症状；②防止症状加重或反复；③尽可能将肺功能维持在正常或接近正常水平；④防止发生不可逆的气流受限；⑤保持正常活动（包括运动）能力；⑥避免药物的不良反应；⑦防止因哮喘死亡。

治疗原则为长期、持续、规范和个体化治疗。急性发作期治疗重点为抗炎、平喘，以便快速缓解症状；慢性缓解期应坚持长期抗炎，降低气道反应性，防止气道重塑，避免危险因素和自我保健。

治疗哮喘的药物包括缓解药物和控制药物。缓解药物能快速缓解支气管收缩及其他伴随的急性症状，用于哮喘急性发作期，包括：①吸入型速效β_2受体激动剂；②全身性糖皮质激素；③抗胆碱能药物；④口服短效β_2受体激动剂；⑤短效茶碱等。控制药物是抑制气道炎症需长期使用的药物，用于哮喘慢性持续期，包括：①吸入型糖皮质激素；②白三烯调节剂；③缓释茶碱；④长效β_2受体激动剂；⑤肥大细胞膜稳定剂；⑥全身性糖皮质激素等。

1. 哮喘急性发作期治疗

（1）β_2受体激动剂：β_2受体激动剂是目前临床应用最广的支气管舒张剂。根据起作用的快慢分为速效和缓慢起效两大类，根据维持时间的长短分为短效和长效两大类。吸入型速效β_2受体激动剂疗效可维持4～6 小时，是缓解哮喘急性症状的首选药物，严重哮喘发作时第1 小时可每20 分钟吸入1 次，以后每2～4 小时可重复吸入。药物剂量：每次沙丁胺醇2.5～5.0 mg或特布他林2.5～5.0 mg。急性发作病情相对较轻时也可选择短期口服短效β_2受体激动剂如沙丁胺醇片和特布他林片等。

（2）全身性糖皮质激素：病情较重的急性病例应给予口服泼尼松短程治疗（1～7 天），每日1～2 mg/kg，分2～3 次。一般不主张长期使用口服糖皮质激素治疗儿童哮喘。严重哮喘发作时应静脉给予甲基泼尼松龙，每日2～6 mg/kg，分2～3 次输注，或琥珀酸氢化可的松或氢化

可的松，每次5～10 mg/kg。必要时可加大剂量。一般静脉糖皮质激素使用1～7 天，症状缓解后即停止静脉用药，若需持续使用糖皮质激素者，可改为口服泼尼松。

（3）抗胆碱能药物：吸入型抗胆碱能药物如溴化异丙托品舒张支气管的作用比β_2受体激动剂弱，起效也较慢，但长期使用不易产生耐药，不良反应少。

（4）短效茶碱：短效茶碱可作为缓解药物用于哮喘急性发作的治疗，主张将其作为哮喘综合治疗方案中的一部分，而不单独应用治疗哮喘。需注意其不良反应，长时间使用者，最好监测茶碱的血药浓度。

2．哮喘慢性持续期治疗

（1）吸入型糖皮质激素：吸入型糖皮质激素（ICS）是哮喘长期控制的首选药物，也是目前最有效的抗炎药物，优点是通过吸入，药物直接作用于气道黏膜，局部抗炎作用强，全身不良反应少。通常需要长期、规范吸入1～3 年才能起预防作用。目前临床上常用的吸入型糖皮质激素有布地奈德、丙酸氟替卡松和丙酸倍氯米松。每3 个月应评估病情，以决定升级治疗、维持目前治疗或降级治疗。

（2）白三烯调节剂：分为白三烯合成酶抑制剂和白三烯受体拮抗剂，该药耐受性好，副作用少，服用方便。白三烯受体拮抗剂包括孟鲁司特和扎鲁司特。

（3）缓释茶碱：缓释茶碱用于长期控制时，主要协助ICS抗炎，每日分1～2 次服用，以维持昼夜的稳定血药浓度。

（4）长效β_2受体激动剂：药物包括福莫特罗、沙美特罗、班布特罗及丙卡特罗等。

（5）肥大细胞膜稳定剂：肥大细胞膜稳定剂色甘酸钠，常用于预防运动及其他刺激诱发的哮喘，治疗儿童哮喘效果较好，副作用小，在美国等国家应用较多。

（6）全身性糖皮质激素：在哮喘慢性持续期控制哮喘发作过程中，全身性糖皮质激素仅短期在慢性持续期分级为重度持续患儿，长期使用高剂量ICS加吸入型长效β_2受体激动剂及其也控制药物疗效欠佳的情况下使用。

（7）联合治疗：对病情严重度分级为重度持续和单用ICS病情控制不佳的中度持续的哮喘提倡长期联合治疗，如ICS联合吸入型长效β_2受体激动剂、ICS联合白三烯调节剂和ICS联合缓释茶碱。

3．哮喘持续状态的处理

（1）氧疗：所有危重哮喘患儿均存在低氧血症，需用密闭面罩或双鼻导管提供高浓度湿化氧气，初始吸氧浓度以40%为宜，流量4～5 L/min。

（2）补液、纠正酸中毒：注意维持水、电解质平衡，纠正酸碱紊乱。

（3）糖皮质激素：全身应用糖皮质激素作为儿童危重哮喘治疗的一线药物，应尽早使用。病情严重时不能以吸入治疗替代全身糖皮质激素治疗，以免延误病情。

（4）支气管扩张剂的使用：可用：①吸入型速效β_2受体激动剂；②氨茶碱静脉滴注；③抗胆碱能药物；④肾上腺素皮下注射，药物剂量：每次皮下注射1：1000肾上腺素0.01 ml/kg，儿童最大不超过0.3 ml。必要时可每20 分钟使用1 次，不能超过3 次。

（5）镇静剂：可用水合氯醛灌肠，慎用或禁用其他镇静剂；在插管条件下，亦可用地西泮镇静，剂量为每次0.3～0.5 mg/kg。

（6）抗生素酌情使用：儿童哮喘发作主要由病毒引发，抗生素不作为常规应用，如同时发生下呼吸道细菌感染则选用病原体敏感的抗菌药物。

（7）辅助机械通气指征：指征为：①持续严重的呼吸困难；②呼吸音减低或几乎听不到哮鸣音及呼吸音；③因过度通气和呼吸肌疲劳而使胸廓运动受限；④意识障碍、烦躁或抑制，

甚至昏迷；⑤吸氧状态下发绀进行性加重；⑥PaO_2≥65 mmHg。

【预防复发及教育管理】

1．避免危险因素 应避免接触过敏原，积极治疗和清除感染灶，去除各种诱发因素（吸烟、呼吸道感染和气候变化等）。

2．特异性免疫治疗 在无法避免接触过敏原或药物治疗无效时，可考虑针对过敏原的特异性免疫治疗，需要在有抢救措施的医院进行。对其远期疗效和安全性尚待进一步研究和评价，且过敏原制备的标准化及纯化也有待加强及规范。特异性免疫治疗应与抗炎及平喘药物子将奚先，坚持足够疗程。

3．哮喘的教育与管理 哮喘患儿的教育与管理是提高疗效、减少复发、提高患儿生活质量的重要措施。通过对患儿及家长进行哮喘基本防治知识的教育，调动其对哮喘防治的主观能动性，提高依从性，避免各种危险因素，巩固治疗效果，提高生活质量。

【预后】

儿童哮喘的预后较成人好，病死率约为2/10万～4/10万，约70％～80％年长后症状不再反复，但仍可能存在不同程度气道炎症和高反应性，30％～60％的患儿可完全治愈。

（李昌崇）

第七节 肺炎的分类

肺炎（pneumonia）是指不同病原体或其他因素（如吸入羊水、油类或过敏反应）等所引起的肺部炎症。主要临床表现为发热、咳嗽、气促、呼吸困难和肺部固定性中、细湿啰音。重症患者可累及循环、神经及消化系统而出现相应的临床症状，如心力衰竭、中毒性脑病及中毒性肠麻痹等。

肺炎为婴儿时期重要的常见病，是我国住院小儿死亡的第一位原因，严重威胁小儿健康，被卫生部列为小儿四病防治之一，故加强对本病的防治十分重要。

【分类】

无统一分类，目前常用的有以下几种分类法。

1．病理分类 大叶性肺炎、支气管肺炎和间质性肺炎。

2．病因分类

（1）病毒性肺炎：呼吸道合胞病毒（RSV）占首位，其次为腺病毒（ADV）3、7、11、21 型，流感病毒、副流感病毒1、2、3 型，巨细胞病毒和肠道病毒等。

（2）细菌性肺炎：肺炎链球菌、金黄色葡萄球菌、肺炎杆菌、流感嗜血杆菌、大肠杆菌、军团菌等。

（3）支原体肺炎：由肺炎支原体所致。

（4）衣原体肺炎：由沙眼衣原体（CT）、肺炎衣原体（CP）和鹦鹉热衣原体引起，以CT和CPu多见。

（5）原虫性肺炎：卡氏肺囊虫（卡氏肺孢子虫）肺炎，免疫缺陷病患者为易感人群。

（6）真菌性肺炎：由白色念珠菌、肺曲菌、组织胞浆菌、毛霉菌、球孢子菌等引起的肺炎，多见于免疫缺陷病及长期使用抗生素者。

（7）非感染病因引起的肺炎：如吸入性肺炎、坠积性肺炎、嗜酸性粒细胞性肺炎（过敏性肺炎）等。

3．病程分类 ①急性肺炎：病程<1 个月；②迁延性肺炎：病程1～3 个月；③慢性肺炎：

病程>3 个月。

4．病情分类 ①轻症：除呼吸系统外，其他系统仅轻微受累，无全身中毒症状；②重症：除呼吸系统外，其他系统亦受累，出现其他系统表现，全身中毒症状明显，甚至危及生命。

5．临床表现典型与否分类 ①典型性肺炎：肺炎链球菌、金黄色葡萄球菌（金葡菌）、肺炎杆菌、流感嗜血杆菌、大肠杆菌等引起的肺炎；②非典型性肺炎：肺炎支原体、衣原体、军团菌、病毒性肺炎等。2002 年冬季和2003 年春季在我国发生一种传染性非典型肺炎（infectious atypical pneumonia），世界卫生组织（WHO）将其命名为严重急性呼吸道综合征（severe acute respiratory syndrome，简称SARS），为新型冠状病毒（coronavirus）引起，以肺间质病变为主，传染性强，病死率较高；儿童患者临床表现较成人轻，病死率亦较低。还有近年来发生的禽流感病毒所致的肺炎。

6．发生肺炎的地区进行分类 ①社区获得性肺炎（community acquired pneumonia，CAP）指无明显免疫抑制的患儿在院外或住院48 小时内发生的肺炎；②院内获得性肺炎（hospital acquired pneumonia，HAP）指住院48 小时后发生的肺炎。

另外，新生儿患肺炎则称之为新生儿肺炎。

临床上如果病原体明确，则按病因分类，有助于指导治疗，否则按病理或其他方法分类。

第八节 支气管肺炎

支气管肺炎（bronchopneumonia）是累及支气管壁和肺泡的炎症，为小儿时期最常见的肺炎，2 岁以内儿童多发。一年四季均可发病，北方多发生于冬春寒冷季节及气候骤变时。室内居住拥挤、通风不良、空气污浊，致病微生物增多，易发生肺炎。此外有营养不良、维生素D缺乏性佝偻病、先天性以及病等并存症及低出生体重儿、免疫缺陷者均易发生本病。

【病因】

最常为细菌和病毒，也可由病毒、细菌“混合感染”。发达国家小儿肺炎病原以病毒为主，主要有RSV、ADV、流感及副流感病毒等；发展中国家则以细菌为主。细菌感染仍以肺炎链球菌多见，近年来肺炎支原体、衣原体和流感嗜血杆菌有增加趋势。病原体常由呼吸道入侵，少数经血行入肺。

【病理】

病理变化以肺组织充血、水肿、炎性细胞浸润为主。肺泡内充满渗出物，经肺泡壁通道（kohn孔）向周围组织蔓延，呈点片状炎症灶。若病变融合成片，可累及多个肺小叶或更广泛。当小支气管、毛细支气管发生炎症时，可导致管腔部分或完全阻塞引起肺气肿或肺不张。

不同的病原造成的肺炎病理改变亦有不同：细菌性肺炎以肺实质受累为主；而病毒性肺炎则以间质受累为主，亦可累及肺泡。临床上支气管肺炎与间质性肺炎常同时并存。

【病理生理】

主要变化是由于支气管、肺泡炎症引起通气和换气障碍，导致缺氧和二氧化碳潴留，从而造成一系列病理生理改变(图12-1)。

1．呼吸功能不全 由于通气和换气障碍，氧进入肺泡以及氧自肺泡弥散至血液和二氧化碳排出均发生障碍，血液含氧量下降，动脉血氧分压（PaO_2）和动脉血氧饱和度（SaO_2）均降低，致低氧血症；血CO_2浓度升高。当SaO_2<85％，还原血红蛋白>50 g/L时，则出现发绀。肺炎的早期，仅有缺氧，无明显CO_2潴留。为代偿缺氧，呼吸和心率加快以增加每分钟通气量和改善通气血流比。随着病情的进展，通气和换气功能严重障碍，在缺氧的基础上出现CO_2潴留，此

时PaO_2和SaO_2下降，$PaCO_2$升高，当PaO_2<50 mmHg（6.67 kPa）和（或）$PaCO_2$>50 mmHg（6.67 kPa）时即为呼吸衰竭。为增加呼吸深度，以吸进更多的氧，呼吸辅助肌也参加活动，因而出现鼻翼扇动和三凹征。

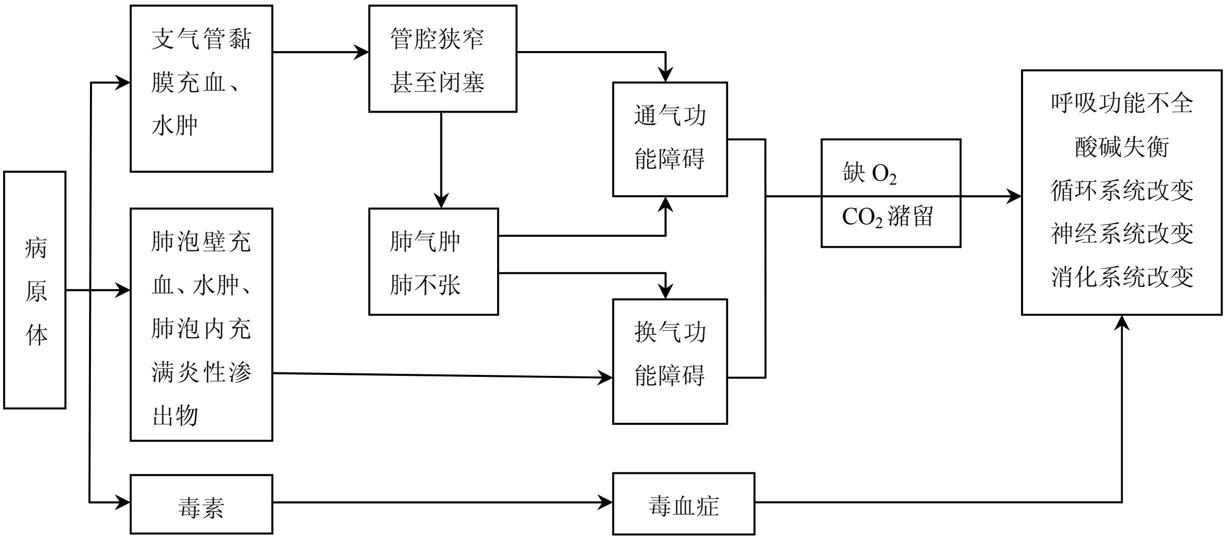

图12-1 支气管肺炎的病理生理

2．酸碱平衡失调及电解质紊乱 严重缺氧时，体内需氧代谢发生障碍，无氧酵解增加，酸性代谢产物增加，加上高热、进食少、脂肪分解等因素，常引起代谢性酸中毒；同时由于二氧化碳排出受阻，可产生呼吸性酸中毒，因此，严重者存在不同程度的混合性酸中毒。6 个月以上的小儿，因呼吸代偿功能稍强，通过加深呼吸，加快排出二氧化碳，可致呼吸性碱中毒，血pH变化不大，影响较小；而6 个月以下的小儿，代偿能力较差，二氧化碳潴留往往明显，甚至发生呼吸衰竭。缺氧和二氧化碳潴留导致肾小动脉痉挛而引起水钠潴留，且重症肺炎缺氧时常有抗利尿激素（ADH）分泌增加，加上缺氧使细胞膜通透性改变、钠泵功能失调，使Na^+进入细胞内，造成低钠血症。

3．循环系统 病原体和毒素侵袭心肌，引起心肌炎；缺氧使肺小动脉反射性收缩，肺循环压力增高，使右心负荷增加。肺动脉高压和中毒性心肌炎是诱发心衰的主要原因。重症患儿常出现微循环障碍、休克甚至弥散性血管内凝血（DIC）。

4．神经系统 严重缺O_2和CO_2潴留使血与脑脊液pH值降低，高碳酸血症使脑血管扩张、血流减慢、血管通透性增加，致使颅内压增加。严重缺氧使脑细胞无氧代谢增加，造成乳酸堆积、ATP生成减少和Na^+-K^+离子泵转运功能障碍，引起脑细胞内钠、水潴留，形成脑水肿。病原体毒素作用亦可引起脑水肿。

5．胃肠道功能紊乱 低氧血症和病原体毒素可使胃肠黏膜糜烂、出血、上皮细胞坏死脱落，导致黏膜屏障功能破坏，使胃肠功能紊乱，出现腹泻、呕吐，甚至发生中毒性肠麻痹。毛细血管通透性增高，可致消化道出血。

【临床表现】

2 岁以下的婴幼儿多见，起病多数较急，发病前数日多先有上呼吸道感染，主要临床表现为发热、咳嗽、气促、肺部固定性的中、细湿啰音。

1．主要症状 ①发热：热型不定，多为不规则发热，亦可为弛张热或稽留热。值得注意的是新生儿、重度营养不良患儿体温可不升或低于正常。②咳嗽：较频繁，在早期为刺激性干咳，极期咳嗽反而减轻，恢复期咳嗽有痰。③气促：多在发热、咳嗽后出现。④全身症状：精神不振、食欲减退、烦躁不安，轻度腹泻或呕吐。

2．体征 ①呼吸增快：40～80 次/分，并可见鼻翼扇动和三凹征。②发绀：口周、鼻唇沟和指趾端发绀，轻症病儿可无发绀。③肺部啰音：早期不明显，可有呼吸音粗糙、减低，以后可闻及较固定的中、细湿啰音，以背部两侧下方及脊柱两旁较多，于深吸气末更为明显。肺部叩诊多正常，病灶融合时，可出现实变体征。

3．重症肺炎的表现 重症肺炎由于严重的缺氧及毒血症，除呼吸系统改变外，可发生循环、神经和消化等系统功能障碍。

（1）循环系统：可发生心肌炎、心力衰竭。肺炎合并心衰的表现：①呼吸突然加快>60 次/分。②心率突然>180 次/分。③突然极度烦躁不安，明显发绀，面色苍白或发灰，指（趾）甲微血管再充盈时间延长。以上三项不能用发热、肺炎本身和其他合并症解释者。④心音低钝、奔马律，颈静脉怒张。⑤肝脏迅速增大。⑥尿少或无尿，眼睑或双下肢水肿。具备前5 项即可诊断为肺炎合并心力衰竭。此症过去多、现在少；北方多、南方少。亦有学者认为不存在此症，只是肺炎本身的一些表现。

（2）神经系统：肺炎并发中毒性脑病至今尚无可靠的诊断方法，在确认肺炎后出现下列症状与体征者，可考虑为中毒性脑病：①烦躁、嗜睡，眼球上窜、凝视；②球结膜水肿，前囟隆起；③昏睡、昏迷、惊厥；④瞳孔改变：对光反应迟钝或消失；⑤呼吸节律不整，呼吸心跳解离（有心跳，无呼吸）；⑥有脑膜刺激征，脑脊液检查除压力增高外，其他均正常。在肺炎的基础上，除外高热惊厥、低血糖、低血钙及中枢神经系统感染（脑炎、脑膜炎），如有①～②项提示脑水肿，伴其他一项以上者可确诊。

（3）消化系统：一般为食欲减退、呕吐和腹泻。发生中毒性肠麻痹时表现为严重腹胀，膈肌升高，加重了呼吸困难，听诊肠鸣音消失。重症患儿还可呕吐咖啡样物，大便潜血阳性或柏油样便。

（4）抗利尿激素异常分泌综合征（syndrome of inappropriate secretion of antidiuretic hormone， SIADH）：①血钠≤130 mmol/L，血渗透压<270 mmol/L；②肾脏排钠增加，尿钠≥20 mmol/L；③临床上无血容量不足，皮肤弹性正常；④尿渗透克分子浓度高于血渗透克分子浓度；⑤肾功能正常；⑥肾上腺皮质功能正常；⑦ADH升高。若ADH不升高，则可能为稀释性低钠血症。SIAHD与中毒性脑病有时表现类似，但治疗却完全不同。

（5）DIC：可表现为血压下降，四肢凉，脉速而弱，皮肤、黏膜及胃肠道出血。

【并发症】

早期合理治疗者并发症少见。若延误诊断或病原体致病力强者可引起并发症，如脓胸、脓气胸、肺大泡等。

1．脓胸（empyema）临床表现为：高热不退；呼吸困难加重；患侧呼吸运动受限；语颤减弱；叩诊呈浊音；听诊呼吸音减弱，其上方有时可听到管性呼吸音。当积脓较多时，患侧肋间隙饱满，纵隔和气管向健侧移位。胸部X线（立位）示患侧肋膈角变钝，或呈反抛物线阴影。胸腔穿刺可抽出脓液。

2．脓气胸（pyopneumothorax） 肺脏边缘的脓肿破裂与肺泡或小支气管相通即造成脓气胸。表现为突然呼吸困难加剧，剧烈咳嗽，烦躁不安，面色发绀。胸部叩诊积液上方呈鼓音，听诊呼吸音减弱或消失。若支气管破裂处形成活瓣，气体只进不出，形成张力性气胸，可危及生命，必须积极抢救。立位X线检查可见液气面。

3．肺大泡（pneumatocele） 由于细支气管形成活瓣性部分阻塞，气体进的多、出的少或只进不出，肺泡扩大，破裂而形成肺大泡，可一个亦可多个。体积小者无症状，体积大者可引起呼吸困难。X线可见薄壁空洞。

以上三种并发症多见于金黄色葡萄球菌肺炎和某些革兰阴性杆菌肺炎。

【辅助检查】

1．外周血检查

（1）白细胞检查：细菌性肺炎白细胞升高，中性粒细胞增多，并有核左移现象，胞浆可有中毒颗粒。病毒性肺炎的白细胞计数大多正常或偏低，亦有少数升高者，时有淋巴细胞增高或出现变异型淋巴细胞。

（2）C反应蛋白（CRP）：细菌感染时血清CRP值多上升，而非细菌感染时则上升不明显。

2．病原学检查

（1）细菌学检查：

1）细菌培养和涂片：采取气管吸取物、肺泡灌洗液、胸水、脓液和血标本作细菌培养和鉴定，同时进行药物敏感试验对明确细菌性致病菌和治疗有指导性意义。亦可作涂片染色镜检，进行初筛试验。

2）其他检查：已用于临床的有对流免疫电泳法测定肺炎球菌多糖抗原和葡萄球菌磷壁酸抗体（滴度≥1：4 为阳性，特异性高，准确率为94.6％）。试管凝集试验对军团菌的诊断为目前首选的简易方法，双份血清抗体滴度≥4倍升高或单份血清抗体滴度≥1：320为阳性。鲎珠溶解物试验用于检测革兰阴性菌内毒素。

（2）病毒学检查

1）病毒分离和血清学试验：取气管吸取物、肺泡灌洗液接种于敏感的细胞株，进行病毒分离是诊断病毒性病原体的好方法。于急性期和恢复期（14 天后）采取双份血清测定特异性IgG抗体水平，若抗体升高≥4 倍为阳性。传统的病毒分离和检测双份血清滴度的结果可靠，但由于费时太长，往往只能作为回顾性诊断和其他方法的对照，限制了其临床实际应用。

2）快速诊断：①检测抗原：采取咽拭子、鼻咽分泌物、气管吸取物或肺泡灌洗液涂片，或快速培养后使用病毒特异性抗体（包括单克隆抗体）免疫荧光技术、免疫酶法或放射免疫法可发现特异性病毒抗原；②检测抗体：血清中IgM特异性病毒抗体出现较早，消失较快，若病毒特异性IgM抗体阳性说明是新近感染；分直接ELISA-IgM和IgM抗体捕获试验（MCA-IgM）；③其他快速诊断方法：如核酸分子杂交技术或聚合酶链反应（PCR）技术的敏感性很高，但易于污染而出现假阳性，要求较高的实验室条件方可防止污染的发生。

（3）其他病原学检查

1）肺炎支原体（mycoplasma pneumoniae，MP）：①冷凝集试验≥1：64 有很大参考价值，该试验为非特异性，可作为过筛试验；②特异性诊断：包括MP 分离培养或特异性IgM和IgG抗体测定。补体结合抗体检测是诊断MP的常用方法；基因探针及PCR技术检测MP的特异性强和敏感性高，但应避免发生污染。

2）衣原体：能引起肺炎的衣原体为沙眼衣原体（chlamydia tracomatis，CT）、肺炎衣原体（chlamydia pneumoniae，CP）和鹦鹉热衣原体。细胞培养用于诊断CT和CP。直接免疫荧光或吉姆萨染色法可检查CT。其他方法有酶联免疫吸附试验、放射免疫电泳法检测双份血清特异性抗体或抗原、核酸探针及PCR技术检测抗原。

3．X线检查 早期肺纹理增强，透光度减低，以后两肺下野、中内带出现大小不等的点状或小斑片状影，或融合成片状阴影，甚至波及节段。可有肺气肿、肺不张。伴发脓胸时，早期患侧肋膈角变钝；积液较多时，可呈反抛物线状阴影，纵隔、以及向健侧移位。并发脓气胸时，患侧胸腔可见液平面。肺大泡时则见完整薄壁、无液平面的大泡。

【诊断】

支气管肺炎的诊断比较简单，一般有发热、咳嗽、呼吸急促的症状，肺部听到中、细湿啰音或X线有肺炎的改变均可诊断为支气管肺炎。

确诊支气管肺炎后应进一步了解引起肺炎的可能病原体和病情的轻重。若为反复发作者，还应尽可能明确导致反复感染的原发疾病或诱因，如原发性或继发性免疫缺陷病、呼吸道局部畸形或结构异常、支气管异物、先天性心脏病、营养性障碍和环境因素等。此外，还要注意是否有并发症。

【鉴别诊断】

1．急性支气管炎 一般不发热或低热，全身状况好，以咳嗽为主要症状，肺部可闻及干湿啰音，多不固定，随咳嗽而改变。X线示肺纹理增多、排列紊乱。若鉴别困难，则按肺炎处理。

2．支气管异物 有异物吸入史，突然出现呛咳，可有肺不张和肺气肿，可资鉴别。但有的病程迁延，有继发感染则类似肺炎或合并肺炎，需注意鉴别。

3．支气管哮喘 儿童哮喘可无明显喘息发作，主要表现为持续性咳嗽，X线示肺纹理增多、排列紊乱和肺气肿，易与本病混淆。患儿具有过敏体质，肺功能检查及激发和舒张试验有助于鉴别。

4．肺结核 一般有结核接触史，结核菌素试验阳性，X线示肺部有结核病灶可资鉴别。粟粒性肺结核可有气急和发绀，从而与肺炎极其相似，但肺部啰音不明显。

【治疗】

采用综合治疗，原则为控制炎症、改善通气功能、对症治疗、防止和治疗并发症。

1．一般治疗及护理 室内空气要流通，以温度18～20℃、湿度60％为宜。给予营养丰富的饮食，重症患儿进食困难者，可给予肠道外营养。经常变换体位，以减少肺部淤血，促进炎症吸收。注意隔离，以防交叉感染。

注意水和电解质的补充，纠正酸中毒和电解质紊乱，适当的液体补充还有助于气道的湿化。但要注意输液速度，过快可加重心脏负担。

2．抗感染治疗

（1）抗生素治疗：明确为细菌感染或病毒感染继发细菌感染者应使用抗生素。

1）原则：①根据病原菌选用敏感药物：在使用抗菌药物前应采集合适的呼吸道分泌物进行细菌培养和药物敏感试验，以便指导治疗；在未获培养结果前，可根据经验选择敏感的药物；②选用的药物在肺组织中应有较高的浓度；③早期用药；④联合用药；⑤足量、足疗程。重者患儿宜静脉联合用药。

2）根据不同病原选择抗生素：①肺炎链球菌：青霉素敏感者首选青霉素或阿莫西林；青霉素低度耐药者仍可首选青霉素，但剂量要加大，青霉素过敏者选用大环内酯类抗生素如红霉素等；②金黄色葡萄球菌：甲氧西林敏感者首选苯唑西林钠或氯唑西林钠，耐药者选用万古霉素或联用利福平；③流感嗜血杆菌：首选阿莫西林加克拉维酸（或加舒巴坦）；④大肠杆菌和肺炎杆菌：首选头孢曲松或头孢噻肟，铜绿假单胞菌（绿脓杆菌）首选替卡西林加克拉维酸；⑤卡他莫拉菌：首选阿莫西林加克拉维酸；⑥肺炎支原体和衣原体：首选大环内酯类抗生素如红霉素、罗红霉素及阿奇霉素。

3）用药时间：一般应持续至体温正常后5～7 天，症状、体征消失后3 天停药。支原体肺炎至少使用抗菌药物2～3 周。葡萄球菌肺炎在体温正常后2～3 周可停药，一般总疗程≥6 周。

（2）抗病毒治疗：①利巴韦林（病毒唑）：可滴鼻、雾化吸入、肌注和静脉点滴，肌注和静点的剂量为10～15 mg/（kg·d），可抑制多种RNA和DNA病毒；②α-干扰素（interferon-α，IFN-α）：5～7 天为一疗程，亦可雾化吸入。

3．对症治疗

（1）氧疗：有缺氧表现，如烦躁、口周发绀时需吸氧，多用鼻前庭导管给氧，经湿化的氧气的流量为0.5～1 L/min，氧浓度不超过40％。新生儿或婴幼儿可用面罩、氧帐、鼻塞给氧，面罩给氧流量为2～4 L/min，氧浓度为50％～60％。

（2）气道管理：及时清除鼻痂、鼻腔分泌物和吸痰，以保持呼吸道通畅，改善通气功能。气道的湿化非常重要，有利于痰液的排出。雾化吸入有助于解除支气管痉挛和水肿。分泌物堆积于下呼吸道，经湿化和雾化仍不能排除，使呼吸衰竭加重时，应行气管插管以利于清除痰液。严重病例宜短期使用机械通气（人工呼吸机）。接受机械通气者尤应注意气道湿化、变换体位和拍背，保持气道湿度和通畅。

（3）腹胀的治疗：低钾血症者，应补充钾盐。中毒性肠麻痹时，应禁食和胃肠减压，亦可使用酚妥拉明（Regitine）0.3～0.5 mg/（kg·次）加5%葡萄糖20 ml静脉滴注，最大量≤10 mg/次。

（4）其他：高热患儿可用物理降温，如35％酒精擦浴；冷敷，冰袋放在腋窝、腹股沟及头部；口服对乙酰胺基酚或布洛芬等。若伴烦躁不安可给予氯丙嗪、异丙嗪各0.5～1．0mg/（kg·次）肌注，或苯巴比妥5 mg/（kg·次）肌注。

4．糖皮质激素 糖皮质激素可减少炎症渗出，解除支气管痉挛，改善血管通透性和微循环，降低颅内压。使用指征为：①严重憋喘或呼吸衰竭；②全身中毒症状明显；③合并感染中毒性休克；④出现脑水肿。上述情况可短期应用激素。可用琥珀酸氢化可的松5～10 mg/（kg·d）或用地塞米松0.1～0.3mg/（kg·d）加入瓶中静脉点滴，疗程3～5 天。

5．并发症及并存症的治疗

（1）肺炎合并心力衰竭的治疗：吸氧、镇静、利尿、强心、血管活性药物。①利尿：可用呋塞米、依他尼酸，剂量为1 mg/（kg·次），稀释成2 mg/ml，静注或加滴壶中静点；亦可口服呋塞米、依他尼酸或双氢克尿噻等。②强心药：可使用地高辛或毛花苷丙静脉注射。③血管活性药物：常用酚妥拉明0.5～1.0 mg/（kg·次），最大剂量不超过10 mg/次，肌注或静注，必要时隔1～4 小时重复使用；亦可用巯甲丙脯酸和硝普钠。

（2）肺炎合并中毒性脑病的治疗：脱水疗法、改善通气、扩血管、止痉、糖皮质激素、促进脑细胞恢复。①脱水疗法：主要使用甘露醇，根据病情轻重每次0.25～0.5～1.0 g/kg，每6 小时1 次。②改善通气：必要时应予人工辅助通气、间歇正压通气，疗效明显且稳定后应及时改为正常通气。③扩血管药物：可缓解脑血管痉挛、改善脑微循环，从而减轻脑水肿，常用酚妥拉明、654-2。酚妥拉明0.5～1.0 mg/（kg·次），新生儿每次≤3 mg，婴幼儿每次≤10 mg，静脉快速滴注，每2～4小时一次，也可静脉滴注维持。④止痉：一般选用地西泮0.2～0.3 mg/（kg·次），静脉注射，1～2 小时可重复一次；也可采用人工冬眠疗法。⑤糖皮质激素的使用：可非特异性抗炎、减少血管与血-脑屏障的通透性，故可用于治疗脑水肿。常用地塞米松0.25 mg/（kg·次），静脉滴注，每6 小时一次，2～3天后逐渐减量或停药。⑥促进脑细胞恢复的药物：常用的有三磷酸腺苷（ATP）、胞磷胆碱、维生素B_1和维生素B_6等。

（3）SIADH的治疗：与肺炎合并稀释性低钠血症治疗是相同的。原则为限制水入量，补充高渗盐水。当血钠为120～130 mmol/L，无明显症状时，主要措施是限制水的摄入量，以缓解低渗状态。如血钠<120 mmol/L，有明显低钠血症症状时，按3%氯化钠12 ml/kg，可提高血钠10 mmol/L计算，先给予1/2 量，在2～4小时内静脉点滴，必要时4 小时后可重复一次。

（4）脓胸和脓气胸者应及时进行穿刺引流，若脓液黏稠，经反复穿刺抽脓不畅或发生张力性气胸时，宜考虑胸腔闭式引流。

（5）对并存佝偻病、贫血、营养不良者，应给予相应治疗。

6．生物制剂 血浆和静脉注射用丙种球蛋白（IVIG）含有特异性抗体，如RSV-IgG抗体，可用于重症患儿，IVIG 400 mg/（kg・d），3～5天为一疗程。

第九节 几种不同病原体所致肺炎的特点

一、病毒性肺炎

（一）呼吸道合胞病毒肺炎（respiratory syncytial virus pneumonia）

简称合胞病毒（RSV）肺炎，是最常见的病毒性肺炎。RSV只有一个血清型，但有A、B两个亚型，我国以A亚型为主。本病多见于婴幼儿，尤多见于1 岁以内小儿。一般认为其发病机制是RSV对肺的直接侵害，引起间质性炎症，而非变态反应所致，与RSV毛细支气管炎不同。临床上轻症患者发热、呼吸困难等症状不重；中、重症者有较明显的呼吸困难、喘憋、口唇发绀、鼻扇及三凹症。发热可为低、中度热和高热。肺部听诊多有中、细湿啰音。X线表现为两肺可见小点片状、斑片状阴影，部分病儿有不同程度的肺气肿。外周血白细胞总数大多正常。

（二）腺病毒肺炎（adenovirus pneumonia）

腺病毒肺炎为腺病毒（ADV）感染所致，ADV共有49 个血清型，引起小儿肺炎最常见的为3、7型，其次为11、21型，1、2、5、6、14型亦可见到。ADV肺炎曾是我国小儿患病率和死亡率最高的病毒性肺炎，占20 世纪70 年代前病毒性肺炎的第一位，死亡率最高曾达33％，发病率现在被RSV肺炎取代为第一位。7型ADV有15 个基因型，其中7b所致肺炎的临床表现典型而严重。本病多见于6 个月～2 岁小儿，冬春季节多发。临床特点为起病急骤、高热持续时间长、中毒症状重、啰音出现较晚、X线改变较肺部体征出现早，易合并心肌炎和多器官衰竭。症状表现为：①发热：可达39℃以上，呈稽留高热或弛张热，热程长，可持续2～3 周；②中毒症状重：面色苍白或发灰，精神不振，嗜睡与烦躁交替；③呼吸道症状：咳嗽频繁，呈阵发性喘憋，轻重不等的呼吸困难和发绀；④消化系统症状：腹泻、呕吐和消化道出血；⑤可因脑水肿而致嗜睡、昏迷或惊厥发作。体格检查发现：①肺部啰音出现较迟，多于高热3～7 天后才出现，肺部病变融合时可出现实变体征；②肝脾增大，由于单核-吞噬细胞系统反应较强所致；③麻疹样皮疹；④出现心率加速、心音低钝等心肌炎表现；亦可有脑膜刺激征等中枢神经系统体征。X线特点：①肺部X线改变较肺部啰音出现早，故强调早期摄片；②大小不等的片状阴影或融合成大病灶，甚至一个大叶；③病灶吸收较慢，需数周或数月。

从20 世纪80 年代后期至今，ADV的7b型已渐被7d型取代，而7d型引起的肺炎相对较轻且不典型。

ADV肺炎易继发细菌感染。继发细菌感染者表现为：持续高热不退；症状恶化或一度好转又恶化；痰液由白色转为黄色脓样；外周血白细胞明显升高，有核左移；胸部X线见病变增多或发现新的病灶。

二、细菌性肺炎

（一）金黄色葡萄球菌肺炎（staphylococcal aureus pneumonia）

病原为金黄色葡萄球菌（简称金葡菌）。由呼吸道入侵或经血行播散入肺。新生儿、婴幼儿发病率高，近年来由于滥用抗生素致耐药性金葡菌株明显增加，加上小儿免疫功能低下，故易发生。病理改变以肺组织广泛出血性坏死和多发性小脓肿形成为特点。由于病变发展迅速，

组织破坏严重，故易形成肺脓肿、脓胸、脓气胸、肺大泡、皮下气肿、纵隔气肿。并可引起败血症及其他器官的迁徙性化脓灶，如化脓性心包炎、脑膜炎、肝脓肿、肝脓肿、皮肤脓肿、骨髓炎和关节炎。临床特点为起病急、病情严重、进展快，全身中毒症状明显。发热多呈弛张热型，但早产儿和体弱儿有时可无发热或仅有低热；患者面色苍白、烦躁不安、咳嗽、呻吟，呼吸浅快和发绀；重症者可发生休克；消化系统症状有呕吐、腹泻和腹胀。肺部体征出现较早，两肺有散在中、细湿啰音，发生脓胸、脓气胸和皮下气肿时则有相应体征。发生纵隔气肿时呼吸困难加重。可有各种类型皮疹，如荨麻疹或猩红热样皮疹等。

X线检查：胸部X线可有小片状影，病变发展迅速，甚至数小时内可出现小脓肿、肺大泡或胸腔积液，因此在短期内应重复摄片。病变吸收较一般细菌性肺炎缓慢，重症病例在2 个月时可能还未完全消失。

外周血白细胞多数明显增高，中性粒细胞增高伴核左移并有中毒颗粒。婴幼儿和重症患者可出现外周血白细胞减少，但中性粒细胞百分比仍较高。

（二）革兰阴性杆菌肺炎（Gram-negative bacillary pneumonia，GNBP）

目前有增多趋势，病原菌以流感嗜血杆菌和肺炎杆菌为多，伴有免疫缺陷者常发生铜绿假单胞菌肺炎，新生儿时期易患大肠杆菌肺炎。革兰阴性杆菌肺炎的病情较重，治疗困难，预后较差。病理改变以肺内浸润、实变、出血性坏死为主。大多先有数日呼吸道感染症状，病情呈亚急性，但全身中毒症状明显，表现为发热，精神萎靡、嗜睡、咳嗽、呼吸困难、面色苍白、口唇发绀，病重者甚至休克。肺部听诊可听到湿啰音，病变融合有实变体征。

肺部X线改变多种多样，如肺炎杆菌肺炎可为肺段或大叶性致密实变阴影，其边缘往往膨胀凸出；绿脓杆菌肺炎显示结节状浸润阴影及细小脓肿，后可融合成大脓肿；流感嗜血杆菌肺炎可呈粟粒状阴影。但基本改变为支气管肺炎征象，或呈一叶或多叶节段性或大叶性炎症阴影，易见胸腔积液。

三、其他微生物所致肺炎

（一）肺炎支原体肺炎（mycoplasma pneumoniae pneumonia）

是学龄儿童及青年常见的一种肺炎，婴幼儿亦不少见。本病全年均可发生，占小儿肺炎的10％～20％，流行年分可达30％。病原体为肺炎支原体（MP），是一种介于细菌和病毒之间的微生物，无细胞壁结构。

起病缓慢，潜伏期约2～3周，病初有全身不适、乏力、头痛。2～3 天后出现发热，体温常达39℃左右，可持续1～3 周，可伴有咽痛和肌肉酸痛。

咳嗽为本病突出的症状，一般于病后2～3 天开始，初为干咳，后转为顽固性剧咳，常有黏稠痰液，偶带血丝，少数病例可类似百日咳样阵咳，可持续1～4 周。肺部体征多不明显，甚至全无。少数可听到干、湿啰音，但多很快消失，故体征与剧咳及发热等临床表现不一致，为本病特点之一。婴幼儿起病急，病程长，病情较重，表现为呼吸困难、喘憋、喘鸣音较为突出；肺部啰音比年长儿多。

部分患儿可有溶血性贫血、脑膜炎、心肌炎、肾炎、格林-巴利综合征等肺外表现。

X线检查：本病的重要诊断依据为肺部X线改变。特点为：①支气管肺炎；②间质性肺炎；③均匀一致的片状阴影似大叶性肺炎改变；④肺门阴影增浓。上述改变可相互转化，有时一处消散，而另一处又出现新的病变，即所谓游走性浸润；有时呈薄薄的云雾状浸润影。亦可有胸腔积液。体征轻而X线改变明显是它的又一特点。

（二）衣原体肺炎（chlamydial pneumonia）

是由衣原体引起的肺炎，衣原体有沙眼衣原体（CT）、肺炎衣原体（CP）、鹦鹉热衣原体

和家畜衣原体。与人类关系密切的为CT和CP，偶见鹦鹉热衣原体肺炎。

1．沙眼衣原体肺炎 CT肺炎主要通过母婴垂直传播而感染。①主要见于婴儿，多为1～3 个月婴儿。②起病缓慢，多不发热或仅有低热，一般状态良好。③开始可有鼻塞、流涕等上呼吸道感染症状，1/2患儿有结膜炎。④呼吸系统主要表现为呼吸增快和具有特征性的明显的阵发性不连贯的咳嗽，一阵急促的咳嗽后继以一短促的吸气，但无百日咳样回声。阵咳可引起发绀和呕吐，亦可有呼吸暂停。⑤肺部偶闻及干、湿啰音，甚至捻发音和哮鸣音。⑥X线可显示双侧间质性或小片状浸润，双肺过度充气。CT肺炎也可急性发病，迅速加重，造成死亡，有报告89例CT肺炎中猝死3 例。

2．肺炎衣原体肺炎 ①多见于学龄儿童；②大部分为轻症，发病常隐匿；③无特异性临床表现，早期多为上呼吸道感染的症状，咽痛、声音嘶哑；④呼吸系统最多见的症状是咳嗽，1～2 周后上呼吸道感染症状逐渐消退而咳嗽逐渐加重，并出现下呼吸道感染征象，如未经有效治疗，则咳嗽可持续1～2 个月或更长；⑤肺部偶闻及干、湿啰音或哮鸣音；⑥X 线可见到肺炎病灶，多为单侧下叶浸润，也可为广泛单侧或双侧性病灶。

（鲁继荣）

第十三章 心血管系统疾病

第一节 正常心血管解剖生理

一、心脏的胚胎发育

胚胎早期22 天左右由胚胎腹面两侧的原始基所形成的两个血管源性管状结构在胚胎中轴两侧向中线融合，形成原始心管。胎龄22～24 天，在一系列基因的调控下，由头至尾，形成了动脉干、心球、心室、心房与静脉窦等结构，与此同时心管发生扭转，心球转至右尾侧位，心管逐渐扭曲旋转，心室的扩展和伸张较快，因此渐渐向腹面突出，这样使出自心球、原来处于心管前后两端的动脉总干和静脉窦都位于心脏的前端。心脏的流入及排出孔道并列在一端，四组瓣膜环也连在一起，组成纤维支架。

至胚胎29 天左右，心脏外形基本形成，但此时心脏仍为单一的管道，由静脉窦流入的血液由动脉干流出。房和室的最早划分为房室交界的背面和腹面长出心内膜垫，背侧内膜垫与腹侧内膜垫相互融合成为中间的分隔结构，将房室分隔开。心房的左右之分起始于第3 周末，在心房腔的前背部长出一镰状隔，为第一房间隔，其下缘向心内膜垫生长，暂时未长合时所留孔道名第一房间孔。在第一房间孔未闭合前，第一房间隔的上部形成另一孔，名第二房间孔，这样使左右心房仍保持相通。至胚胎第5、6 周，于第一房间隔右侧又长出一镰状隔，名第二房间隔，此隔在向心内膜垫延伸过程中，其游离缘留下一孔道，名卵圆孔，此孔与第一房间隔的第二房间孔上下相对。随着心脏继续成长，第一房间隔与第二房间隔渐渐接近而黏合，第二房间孔被第二房间隔完全掩盖，即卵圆孔处第一房间隔紧贴着作为此孔的幕帘，血流可由右侧推开幕帘流向左侧，反向时幕帘遮盖卵圆孔而阻止血液自左房流向右房（图13-1）。心房内分隔形

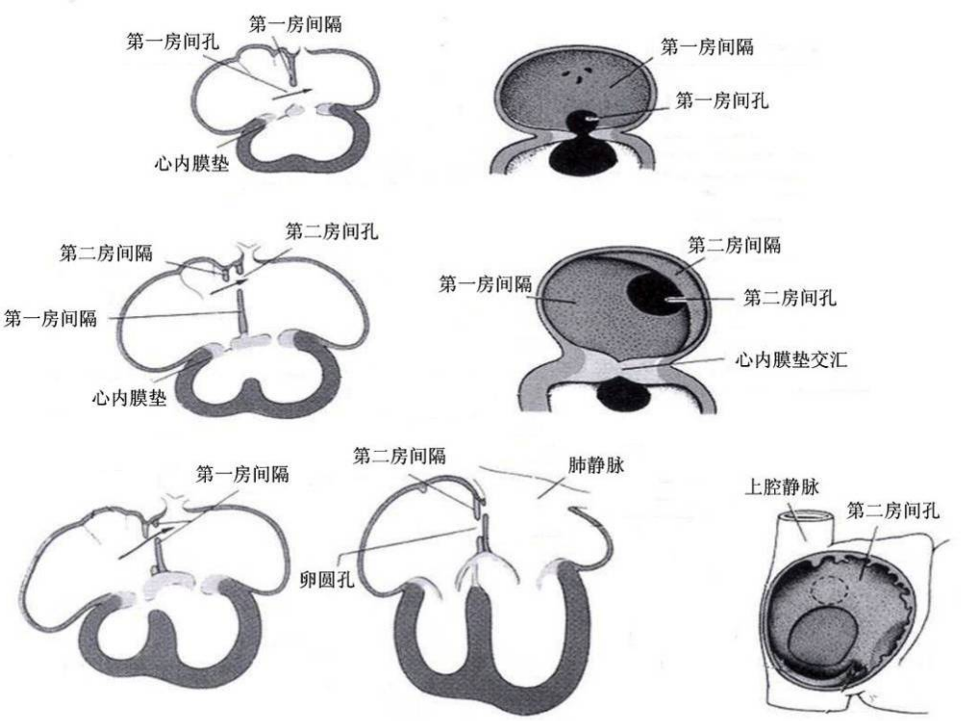

图13-1 人类胚胎30天左右房房间隔的发育过程

成时，由心室底部突出室间隔基胚并向房室管方向生长，使心室分成左右两半，至胚胎第7 周时室间隔上缘的结缔组织、漏斗部及心内膜垫融合成膜部室间隔使室间孔完全闭合。心室间隔的形成有三个来源：①肌膈，由原始心室底壁向上生长，部分地将左右二室分开；②心内膜垫向下生长与肌膈相合，完成室间隔；③小部分为动脉总干及心球分化成主动脉与肺动脉时的中隔向下延伸的部分。后两部分形成室间隔的膜部。室间隔发育过程中任何部分出现异常即可出现室间隔缺损，其中以室间隔膜周部缺损最常见。二尖瓣、三尖瓣分别由房室交界的左右侧及腹背侧心内膜垫及圆锥隔所组成（图13-2）。

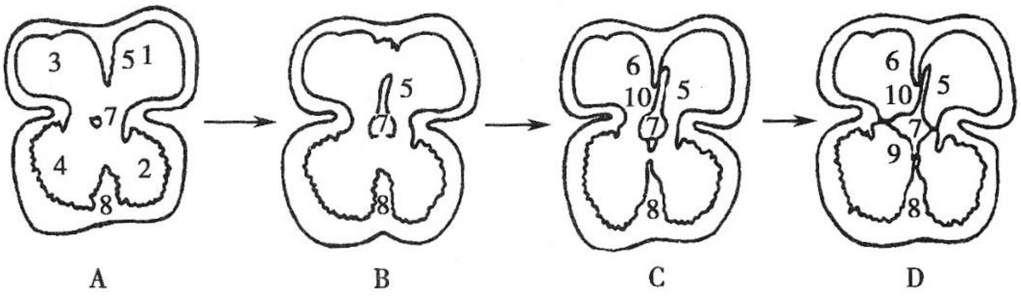

图13-2 人类室间隔的发育

1．左心房 2．左心室 3．右心房 4．右心室 5．第一房间隔 6．第二房间隔
7．心内膜垫 8．室隔肌部 9．室隔膜部 10．卵圆孔

原始的心脏出口是一根动脉总干，在总干的内层对侧各长出一纵嵴，两者在中央轴相连，将总干分为主动脉与肺动脉。由于该纵隔自总干分支处成螺旋形向心室生长，使肺动脉向前、向右旋转与右心室连接，主动脉向左、向后旋转与左心室连接。如该纵隔发育遇障碍，分隔发生偏差或扭转不全，则可造成主动脉骑跨或大动脉错位等畸形。

原始心脏于胚胎第2 周开始形成后，约于第4 周起有循环作用，至第8 周房室间隔已完全长成，即成为四腔心脏。先天性心脏畸形的形成主要就是在这一时期。

二、胎儿新生儿循环转换

（一）正常胎儿循环

胎儿时期的营养和气体代谢是通过脐血管和胎盘与母体之间以弥散方式而进行交换的。由胎盘来的动脉血经脐静脉进入胎儿体内，至肝脏下缘，约50％血流入肝与门静脉血流汇合，另一部分经静脉导管入下腔静脉，与来自下半身的静脉血混合，共同流入右心房。由于下腔静脉瓣的阻隔，使来自下腔静脉的混合血（以动脉血为主）入右心房后，约三分之一经卵圆孔入左心房，再经左心室流入升主动脉，主要供应心脏、脑及上肢；其余的流入右心室。从上腔静脉回流的、来自上半身的静脉血，入右心房后绝大部分流入右心室，与来自下腔静脉的血一起进入肺动脉。由于胎儿肺脏处于压缩状态，故肺动脉的血只有少量流入肺脏经肺静脉回到左心房，而约80％的血液经动脉导管与来自升主动脉的血汇合后，进入降主动脉（以静脉血为主），供应腹腔器官及下肢，同时经过脐动脉回至胎盘，换取营养及氧气。故胎儿期供应脑、心、肝及上肢的血氧量远远较下半

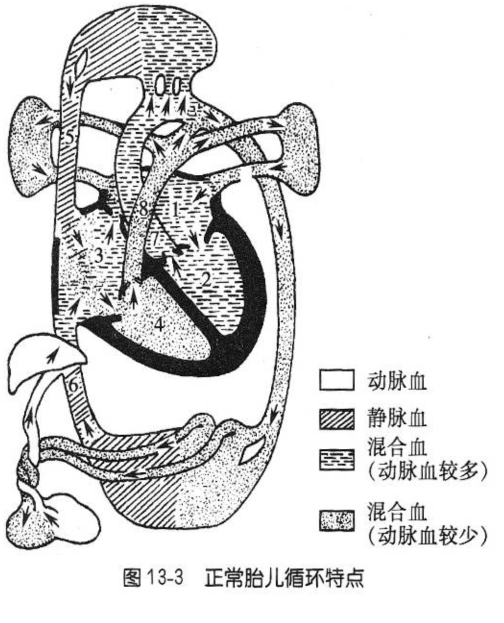

图13-3 正常胎儿循环特点

身为高（图13-3）。右心室在胎儿期不仅要克服体循环的阻力，同时承担着远较左心室多的容量负荷。

（二）出生后血循环的改变

出生后脐血管被阻断，呼吸建立，肺泡扩张，肺小动脉管壁肌层逐渐退化，管壁变薄并扩张，肺循环压力下降。从右心经肺动脉流入肺脏的血液增多，使肺静脉回流至左心房的血量也增多，左心房压力因而增高。当左心房压力超过右心房时，卵圆孔瓣膜先在功能上关闭，到出生后5～7 月，解剖上大多闭合。自主呼吸使血氧增高，动脉导管壁平滑肌受到刺激后收缩，同时，低阻力的胎盘循环由于脐带结扎而终止，体循环阻力增高，动脉导管处逆转为左向右分流，高的动脉氧分压加上出生后体内前列腺素的减少，使导管逐渐收缩、闭塞，最后血流停止，成为动脉韧带。足月儿约80％在生后10～15 小时形成功能性关闭。约80％婴儿于生后3 个月、95％婴儿于生后一年内形成解剖上关闭。若动脉导管持续未闭，可认为有畸形存在。脐血管则在血流停止后6～8 周完全闭锁，形成韧带。

第二节 儿童心血管病检查方法

一、病史和体格检查

在小儿心血管病的诊断中，尽管有多种影像学检查手段，病史和体格检查仍具有不容忽视的价值。仔细的病史询问和体格检查，可以对许多心血管病作出大致判断，缩小鉴别诊断的范围，使进一步的影像学检查更具针对性。

（一）病史询问

小儿时期，尤其是3 岁以内婴幼儿的心血管疾患以先天性心脏病（先心病）最常见。心脏杂音、青紫及心功能不全是先心病患者最常见的就诊原因，其出现时间及演变对疾病的诊断、治疗决策、预后判断有重要意义。反复的肺炎、心功能不全、生长发育迟缓是大量左向右分流的证据；左房或肺动脉扩张压迫喉返神经可引起声音嘶哑。婴幼儿的心功能不全以呼吸浅促、喂养困难、易出汗更突出。有青紫者应注意排除呼吸系统疾病，还要询问有无蹲踞、缺氧发作。一些后天获得性心血管疾病如川崎病主要见于3 岁以下小儿，临床上的皮肤、黏膜、淋巴结等的表现独特。风湿性心脏病多见于年长儿，注意有无咽痛、游走性关节痛、舞蹈病等病史。对胸闷、心悸、心前区疼痛者应注意心律失常、心肌疾病。病史询问中还要注意母孕早期有无病毒感染、放射线接触、有害药物应用史及有无家族遗传病史。许多先心病与遗传性疾病有关，肥厚性心肌病常有阳性家族史。

（二）体格检查

1．全身检查 评价生长发育，注意特殊面容及全身合并畸形、精神状态、体位和呼吸频率。检查口唇、鼻尖、指（趾）端等毛细血管丰富部位有无发绀，青紫6 个月～1 年后，可出现杵状指（趾）。皮肤黏膜瘀点是感染性心内膜炎血管栓塞的表现；皮下小结、环形红斑是风湿热的主要表现之一。注意颈动脉搏动，肝颈静脉回流征，肝脾大小、质地及有无触痛，下肢有无浮肿。

2．心脏检查

（1）视诊：心前区有无隆起，心尖搏动的位置、强弱及范围。心前区隆起者多示有心脏扩大，应注意与佝偻病引起的鸡胸相鉴别。正常<2 岁的小儿，心尖搏动见于左第四肋间，其左侧最远点可达锁骨中线外1 cm，5～6 岁时在左第五肋间，锁骨中线上。正常的心尖搏动范围不超过2～3 cm^2，若心尖搏动强烈、范围扩大提示心室肥大。左心室肥大时，心尖搏动最强

点向左下偏移；右心室肥大时，心尖搏动弥散，有时扩散至剑突下。心尖搏动减弱见于心包积液和心肌收缩力减弱。右位心的心尖搏动则见于右侧。消瘦者心尖搏动易见，而肥胖者相反。

（2）触诊：进一步确定心尖搏动的位置、强弱及范围，心前区有无抬举冲动感及震颤。左第5～6 肋间锁骨中线外的抬举感为左室肥大的佐证，胸骨左缘第3～4 肋间和剑突下的抬举感提示右室肥大。震颤的位置有助于判断杂音的来源。

（3）叩诊：可粗略估计心脏的位置及大小。

（4）听诊：注意心率的快慢、节律是否整齐，第一、二心音的强弱，是亢进、减弱还是消失，有无分裂，特别是肺动脉瓣区第二音（P_2）意义更大。P_2亢进提示肺动脉高压，而减弱则支持肺动脉狭窄的诊断；正常儿童在吸气时可有生理性P_2分裂，P_2固定性分裂是房间隔缺损的独特体征。杂音对鉴别先天性心脏病的类型有重要意义，需注意其位置、性质、响度、时相及传导方向。

3．周围血管征 比较四肢脉搏及血压，如股动脉搏动减弱或消失，下肢血压低于上肢，提示主动脉缩窄。脉压增宽，伴有毛细血管搏动和股动脉枪击音，提示动脉导管未闭或主动脉瓣关闭不全等。

二、特殊检查

（一）普通X线检查

包括透视和摄片，透视可动态地观察心脏和大血管的搏动、位置、形态以及肺血管的粗细、分布，但不能观察细微病变。摄片可弥补这一缺点，并留下永久记录，常规拍摄正位片，必要时辅以心脏三位片。分析心脏病X线片时，应注意以下几点：

1．摄片质量要求 理想的胸片应为吸气相拍摄，显示肺纹理清晰，对比良好，心影轮廓清晰，心影后的胸椎及椎间隙可见。

2．测量心胸比值 年长儿应小于50％，婴幼儿小于55％，呼气相及卧位时心胸比值增大。

3．肺血管阴影，是充血还是缺血，有无侧支血管形成。

4．心脏的形态、位置及各房室有无增大，血管有无异位，肺动脉段是突出还是凹陷，主动脉结是增大还是缩小。

5.确定有无内脏异位症 注意肝脏、胃泡及横膈的位置，必要时可摄增高电压（100～140kV）的高kV胸片，观察支气管的形态。

（二）心电图

心电图对心脏病的诊断有一定的帮助，特别对各种心律失常，心电图是确诊的手段。对心室肥厚、心房扩大、心脏位置及心肌病变有重要参考价值，24 小时动态心电图及各种负荷心电图可提供更多的信息。有些先天性心脏病有特征性的心电图，如房间隔缺损的V_1导联常呈不完全性右束支阻滞。在分析小儿心电图时应注意年龄的影响：

1．年龄越小，心率愈快，各间期及各波时限较短，有些指标的正常值与成人有差别。

2．QRS综合波以右室占优势，尤其在新生儿及婴幼儿，随着年龄增长逐渐转为左室占优势。

3．右胸前导联的T波在不同年龄有一定改变，如生后第1天，V_1导联T波，4～5 天后T波转为倒置或双向。

（三）超声心动图

超声心动图是一种无创检查技术，不仅可以提供详细的心脏解剖结构信息，还能提供心脏功能及部分血流动力学信息，有以下几种。

1．M型超声心动图 能显示心脏各层结构，特别是瓣膜的活动，常用于测量心腔、血管内径，结合同步记录的心电图和心音图可计算多种心功能指标。

2．二维超声心动图 是目前各种超声心动图的基础，可实时地显示心脏和大血管各解剖结构的活动情况，以及它们的空间毗邻关系。经食道超声使解剖结构显示更清晰，已用于心脏手术和介入性导管术中，进行监护及评估手术效果。

3．多普勒超声 有脉冲波多普勒、连续波多普勒及彩色多普勒血流显像三种，可以检测血流的方向及速度，并换算成压力阶差，可用于评估瓣膜、血管的狭窄程度，估算分流量及肺动脉压力，评价心功能等。

4．三维超声心动图 成像直观、立体感强、易于识别，还可对图像进行任意切割，充分显示感兴趣区，为外科医师模拟手术进程与切口途径选择提供了丰富的信息。

超声心动图检查已经能为绝大多数的先心病作出准确的诊断并为外科手术提供足够的信息，已部分取代了心脏导管及造影术，而且能在胎儿期作出部分先心病的诊断。

（四）心导管检查

是先天性心脏病进一步明确诊断和决定手术前的一项重要检查方法之一，根据检查部位不同分为右心导管、左心导管检查两种。右心导管检查系经皮穿刺股静脉，插入不透X线的导管，经下腔静脉、右心房、右心室至肺动脉；左心导管检查时，导管经股动脉、降主动脉逆行至左心室。检查时可探查异常通道，测定不同部位的心腔、大血管的血氧饱和度、压力，进一步计算心排出量、分流量及血管阻力。通过肺小动脉楔入压测定可以评价肺高压患者的肺血管床状态，对左心房入口及出口病变、左心室功能等有一定意义。连续压力测定可评价瓣膜或血管等狭窄的部位、类型、程度。此外经心导管还可进行心内膜活体组织检查、电生理测定。

（五）心血管造影

心导管检查时，根据诊断需要将导管顶端送到选择的心腔或大血管，并根据观察不同部位病损的要求，采用轴向（成角）造影，同时进行快速摄片或电影摄影，以明确心血管的解剖畸形，尤其对复杂性先天性心脏病及血管畸形，心血管造影仍是主要检查手段。数字减影造影技术（DSA）的发展及新一代造影剂的出现降低了心血管造影对人体的伤害，使诊断更精确。

（六）放射性核素心血管造影

常用的放射性核素为^{99m}锝，静脉注射后，应用γ闪烁照相机将放射性核素释放的γ射线最终转换为点脉冲，所有的数据均由计算机记录、存储，并进行图像重组及分析。常用的心脏造影有初次循环心脏造影及平衡心脏血池造影。主要用于左向右分流及心功能检查。

（七）磁共振成像

磁共振成像（MRl）具有无电离辐射损伤、多剖面成像能力等特点，有多种技术选择，包括自旋回波技术（SE）、电影MRI、磁共振血管造影（MRA）及磁共振三维成像技术等。常用于诊断主动脉弓等流出道畸形的诊断，并已经成为复杂畸形诊断的重要补充手段。

（八）计算机断层扫描

电子束计算机断层扫描（EBCT）和螺旋型CT已应用于心血管领域。对下列心脏疾病有较高的诊断价值：大血管及其分支的病变；心脏瓣膜、心包和血管壁钙化，心腔内血栓和肿块；心包缩窄、心肌病等。

第三节 先天性心脏病概述

先天性心脏病（congenital heart disease，CHD，先心病）是胎儿期心脏及大血管发育异常

而致的先天畸形，是小儿最常见的心脏病。流行病学调查资料提示，先天性心脏病的发病率在活产婴儿中为4.05～12.3/‰；若包括出生前已死亡的胎儿，本病的发病率更高。国内对上海市两个区调查了2万多名活产婴儿，发现本病在生后第一年的发病率为6.9‰。估计我国每年约出生15 万患有先天性心脏病的新生儿，如未经治疗，约1/3的患儿在生后1 年内可因病情严重和复杂畸形而死亡。据国内外资料统计，新生儿期死亡的病例以大动脉转位为最多，其次是左心发育不良综合征。各类先天性心脏病的发病情况以室间隔缺损最多，其次为房间隔缺损、动脉导管未闭和肺动脉瓣狭窄。法洛四联症则是存活的发绀型先天性心脏病中最常见者。

近年来随着科学技术的不断发展，先天性心脏病介入导管关闭动脉导管、房间隔缺损和室间隔缺损，应用球囊导管扩张狭窄的瓣膜（如肺动脉瓣狭窄）和血管等技术的发展为先天性心脏病的治疗开辟了崭新的途径。心脏外科手术方面，体外循环、深低温麻醉下心脏直视手术的发展以及带瓣管道的使用不仅使大多数常见先天性心脏病根治手术效果大为提高，而且对某些复杂心脏畸形亦能在婴儿期、甚至新生儿期进行手术，因此先天性心脏病的预后已大为改观。

【病因和预防】

在胎儿心脏发育阶段，若有任何因素影响了心脏胚胎发育，使心脏某一部分发育停顿或异常，即可造成先天性心脏畸形。这类有关因素很多，可分为内因和外因两类，以后者为多见。

内在因素主要与遗传有关，可为染色体异常或多基因突变引起。如：房室间隔缺损和动脉干畸形等与第21 号染色体长臂某些区带的过度复制和22 对染色体部分片段缺失有关。第7、12、15、和22 号染色体上也有与形成心血管畸形有关的基因。据统计，大约有315 种临床综合征伴有先天性心脏病，同一家庭中可有数人同患某一种先天性心脏病也说明其与遗传因素有关。

外在因素中较重要的为宫内感染，特别是母孕早期患病毒感染如风疹、流行性感冒、流行性腮腺炎和柯萨奇病毒感染等，其他如孕母缺乏叶酸，接触放射线，服用药物（抗癌药，抗癫痫药等），代谢性疾病（糖尿病、高钙血症、苯丙酮尿症等），宫内缺氧等均可能与发病有关。

虽然如此，绝大多数先天性心脏病患者的病因尚不清楚，目前认为85%以上先天性心脏病的发生可能是胎儿周围环境因素与遗传因素相互作用的结果。因此，加强孕妇的保健特别是在妊娠早期适量补充叶酸，积极预防风疹、流感等病毒性疾病，以及避免与发病有关的因素接触，保持健康的生活方式等都对预防先天性心脏病具有积极的意义。

【分类】

先天性心脏病的种类很多，且可有两种以上畸形并存，可根据左、右两侧及大血管之间有无分流分为三大类。

1．左向右分流型（潜伏青紫型） 正常情况下由于体循环压力高于肺循环，故平时血液从左向右分流而不出现青紫。当剧哭、屏气或任何病理情况下致使肺动脉或右心室压力增高并超过左心压力时，则可使血液自右向左分流而出现暂时性青紫，如室间隔缺损、动脉导管未闭和房间隔缺损等。

2．右向左分流型（青紫型） 某些原因（如右心室流出道狭窄）致使右心压力增高并超过左心，使血流经常从右向左分流时，或因大动脉起源异常，使大量静脉血流入体循环，均可出现持续性青紫，如法洛四联症和大动脉转位等。

3．无分流型（无青紫型） 即心脏左、右两侧或动、静脉之间无异常通路或分流，如肺动脉狭窄和主动脉缩窄等。

第四节 常见先天性心脏病

一、房间隔缺损

房间隔缺损（atrial septal defect）是小儿时期常见的先天性心脏病，该病的发病率约为活产婴儿的1/1500，占先天性心脏病发病总数的5％～10％。是房间隔在胚胎发育过程中发育不良所致。女性较多见，男女性别比例为1：2。

【病理解剖】

根据胚胎发生，房间隔缺损可分为以下四个类型：

1．原发孔型房间隔缺损 也称为Ⅰ孔型房间隔缺损，约占15％，缺损位于心内膜垫与房间隔交接处。常合并二尖瓣或三尖瓣隔瓣裂，此时称为部分型房室间隔缺损。

2．继发孔型房间隔缺损 最为常见，约占75％。缺损位于房间隔中心卵圆窝部位，亦称为中央型。

3．静脉窦型房间隔缺损 约占5％，分上腔型和下腔型。上腔静脉窦型的缺损位于上腔静脉入口处，右上肺静脉常经此缺损异位引流入右心房。下腔静脉型缺损位于下腔静脉入口处，常合并右下肺静脉异位引流入右心房，此种情况常见于弯刀综合征（scimitar syndrome）。

4．冠状静脉窦型房间隔缺损 约占2％，缺损位于冠状静脉窦上端与左心房间，造成左心房血流经冠状静脉窦缺口分流入右心房。此型缺损常合并左侧上腔静脉残存、左、右侧房室瓣狭窄或闭锁、完全性房室间隔缺损、无脾综合征、多脾综合征等。部分性冠状静脉窦隔缺损，可单发或多发。

【病理生理】

出生后左心房压高于右心房，房间隔缺损时则出现左向右分流，分流量与缺损大小、两侧心房压力差及心室的顺应性有关。生后初期左、右心室壁厚度相似，顺应性也相近故分流量不多。随年龄增长，肺血管阻力及右心室压力下降，右心室壁较左心室壁薄，右心室充盈阻力也较左心室低，故分流量增加。由于右心血流量增加，舒张期负荷加重，故右房、右心室增大（图13-4）。肺循环血量增加，压力增高，晚期可导致肺小动脉肌层及内膜增厚，管腔狭窄，引起肺动脉高压，使左向右分流减少，甚至出现右向左分流，临床出现发绀。

【临床表现】

房间隔缺损的症状随缺损大小而有所不同。缺损小的可无症状，仅在体格检查时发现胸骨左缘2～3 肋间有收缩期杂音。缺损较大时分流量也大，导致肺充血、体循环血流量不足表现为体形瘦长、面色苍白、乏力、多汗、活动后气促和生长发育迟缓。由于肺循环血流增多而易反复呼吸道感染，严重者早期发生心力衰竭。

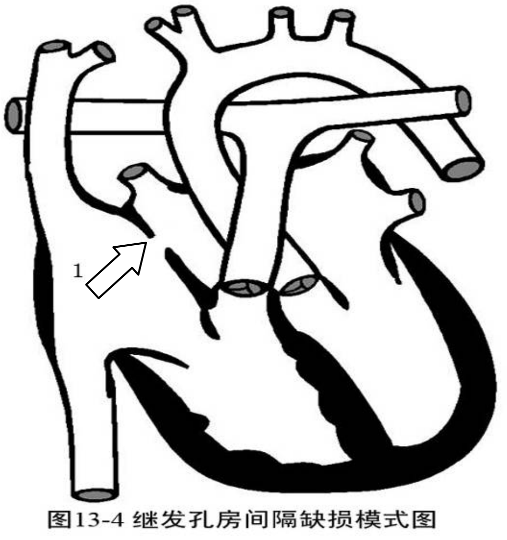

图13-4 继发孔房间隔缺损模式图
1. 继发孔房间隔缺损

多数患儿在婴幼儿期无明显体征，2～3 岁后心脏增大，前胸隆起，触诊心前区有抬举冲动感，一般无震颤，少数大缺损分流量大者可出现震颤。听诊有以下四个特点：①第一心音亢进，肺动脉第二心音增强。②由于右心室容量增加，收缩时喷射血流时间延长，肺动脉瓣关闭更落后于主动脉瓣，出现不受呼吸影响的第二心音固定分裂。③由于右心室增大，大量的血流通过正常肺动

脉瓣时（形成相对狭窄）在左第二肋间近胸骨旁可闻及2～3 级喷射性收缩期杂音。④当肺循环血流量超过体循环达1 倍以上时，则在胸骨左下第4～5 肋间隙处可出现三尖瓣相对狭窄的短促与低频的舒张早中期杂音，吸气时更响，呼气时减弱。随着肺动脉高压的进展，左向右分流逐渐减少，第二心音增强，固定性分裂消失，收缩期杂音缩短，舒张期杂音消失，但可出现肺动脉瓣及三尖瓣关闭不全的杂音。

【辅助检查】

1．X线表现 对分流较大的房间隔缺损具有诊断价值。心脏外形轻至中度增大，以右心房及右心室为主，心胸比大于0.5。肺动脉段突出，肺叶充血明显，主动脉影缩小。透视下可见肺动脉总干及分支随心脏搏动而一明一暗的“肺门舞蹈”征，心影略呈梨形（图13-5）。原发孔型房缺伴二尖瓣裂缺者，左心房及左心室增大。

2．心电图 电轴右偏，平均额面电轴在十95°～+170°之间。右心房和右心室肥大。P-R间期延长，V_1及V_{3R}导联呈rSr'或rsR'等不完全性右束支传导阻滞的图形。分流量较大患者R波可出现切迹。原发孔型房缺的病例常见电轴左偏及左心室肥大。一般为窦性心律，年龄较大者可出现交界性心律或室上性心律失常。

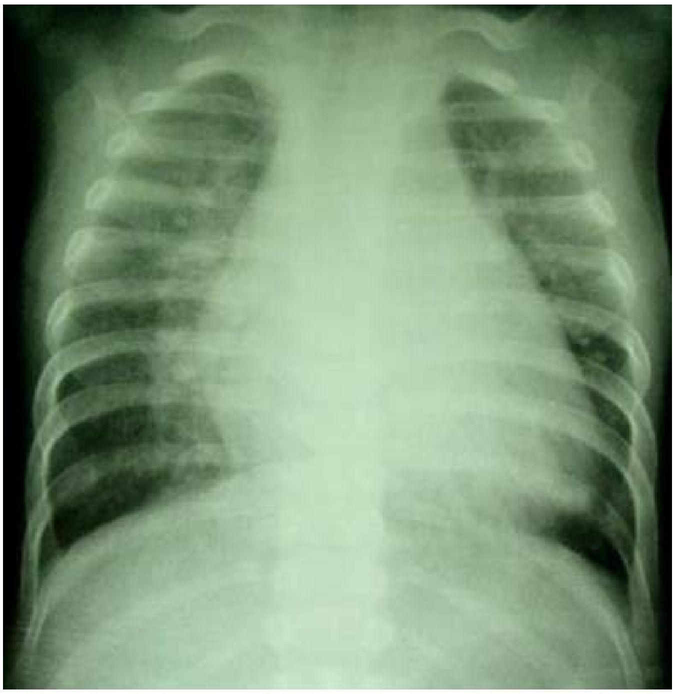

图13-5 房间隔缺损的典型X线特征

3．超声心动图 M型超声心动图可以显示右心房、右心室增大及室间隔的矛盾运动。二维超声可以显示房间隔缺损的位置及大小，结合彩色多普勒超声可以提高诊断的可靠性并能判断分流的方向，应用多普勒超声可以估测分流量的大小，估测右心室收缩压及肺动脉压力。年龄较大的肥胖患者经胸超声透声较差者，可选用经食管超声心动图进行诊断。而动态三位超声心动图可以从左心房侧或右心房侧直接观察到缺损的整体形态，观察缺损与毗邻结构的立体关系及其随心动周期的动态变化，有助于提高诊断的正确率。

4．磁共振 年龄较大患者，剑突下超声透声窗受限，图像不够清晰。磁共振可以清晰地显示缺损的位置、大小及其肺静脉回流情况而建立诊断。

5．心导管检查 一般不需要做心导管检查，当合并肺动脉高压、肺动脉瓣狭窄或肺静脉异位引流时可行右心导管检查。右心导管检查时导管易通过缺损由右心房进入左心房，右心房血氧含量高于腔静脉血氧含量，右心室和肺动脉压力正常或轻度增高，并按所得数据可计算出肺动脉阻力和分流量大小。合并肺静脉异位引流者应探查异位引流的肺静脉。

6．心血管造影 一般不做心血管造影。造影剂注入右上肺静脉，可见其通过房间隔缺损迅速由左心房进入右心房。

【治疗】

小于3 mm的房间隔缺损多在3 个月内自然闭合，大于8 mm的房间隔缺损一般不会自然闭合。房缺分流量较大时一般可在3～5 岁时选择体外循环下手术治疗。反复呼吸道感染、发生心力衰竭或合并肺动脉高压者应尽早手术治疗。房间隔缺损也可通过介入性心导管术，应用双面蘑菇伞（Amplatzer装置）关闭缺损，适应证为：①继发孔型房缺；②直径小于30 mm；③房

间隔缺损边缘距肺静脉、腔静脉、二尖瓣口及冠状静脉窦口的距离大于5 mm；④房间隔的伸展径要大于房间隔缺损直径14 mm以上等。

二、室间隔缺损

室间隔缺损（ventricular septal defect）（简称室缺）由胚胎期室间隔（流入道、小梁部和流出道）发育不全所致，是最常见的先天性心脏病，约占我国先心病的50%。单独存在者约占25%，其他近2/3多为复杂性先天性心脏病合并室间隔缺损。室缺分类的种类很多，但趋向于与外科手术切口结合起来，更具实用性及直观性。最多见为膜周部缺损，占60%～70%，位于主动脉下，由膜部向与之接触的三个区域（流入道、流出道或小梁肌部）延伸而成。肌部缺损，占20%～30%，又分为窦部肌肉缺损（即肌部流入道）、漏斗隔肌肉缺损（过去统称为嵴上型或干下型）及肌部小梁部缺损。

【病理生理】

正常人右室的收缩压仅及左室的1/4～1/6，肺循环阻力为体循环的1/10左右，若存在室缺，左心房血液进入左心室后，一部分从正常途径即左心室到主动脉至体循环，为有效循环，另一部分则自左心室经室缺分流入右心室到肺动脉至肺循环，为无效循环（图13-6）。此时两个循环量不再相等，肺循环血流量大于体循环血流量，从肺动脉瓣或二尖瓣血流量中减去主动脉瓣或三尖瓣血流量即分流量。分流量多少取决于缺损面积、心室间压差及肺小动脉阻力，缺损大致可分为3种类型：

1．小型室缺（Roger病） 缺损直径小于5 mm或缺损面积<0.5 cm^2/m^2体表面积。缺损小，心室水平左向右分流量少，血流动力学变化不大，可无症状。

2．中型室缺 缺损直径5～15 mm或缺损面积0.5～1.0 cm^2/m^2体表面积。缺损较大，分流量较多，肺循环血流量可达体循环的1.5～3.0 倍以上，但因肺血管床有很丰富的后备容受量，肺动脉收缩压和肺血管阻力可在较长时期不增高。

3．大型室间隔缺损 缺损直径大于15 mm或缺损面积>1.0 cm^2/m^2体表面积。缺损巨大，缺损口本身对左向右分流量不构成阻力，血液在两心室自由交通，即非限制性室缺。大量左向右分流量使肺循环血流量增加，当超过肺血管床的容量限度时，出现容量性肺动脉高压，肺小动脉痉挛，肺小动脉中层和内膜层渐增厚，管腔变小、梗阻。随着肺血管病变进行性发展则渐变为不可逆的阻力性肺动脉高压。当右心室收缩压超过左心室收缩压时，左向右分流逆转为双向分流或右向左分流，出现发绀，即艾森曼格（Eisenmenger）综合征。

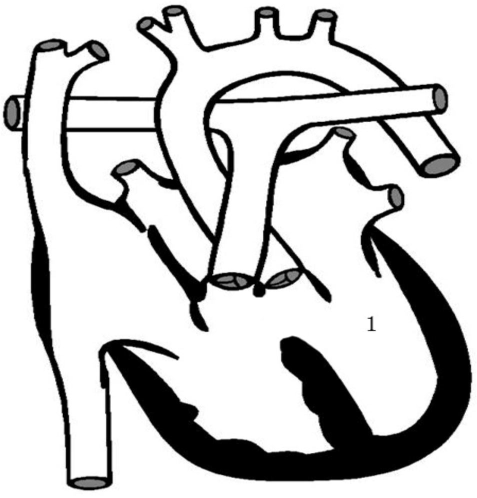

图13-6 室间隔缺损的模式图
1. 膜部室间隔缺损

【临床表现】

临床表现决定于缺损大小和心室间压差，小型缺损可无症状，一般活动不受限制，生长发育不受影响。仅体格检查时听到胸骨左缘第3、4肋间响亮的全收缩期杂音，常伴震颤，肺动脉第二音正常或稍增强。缺损较大时左向右分流量多，体循环血流量相应减少，患儿多生长迟缓，体重不增，有消瘦、喂养困难、活动后乏力、气短、多汗、易患反复呼吸道感染，易导致充血性心力衰竭等。有时因扩张的肺动脉压迫喉返神经，引起声音嘶哑。体格检查发现心界扩大，搏动活跃，胸骨左缘第3、4肋间可闻及Ⅲ～Ⅳ粗糙的全收缩期杂音，向四周广泛传导，可扪及收缩期震颤。分流量大时在心尖区可闻及二尖瓣相对狭窄的较柔和舒张中期杂音。大型缺损伴有明显肺动脉高压时（多见于儿童或青少年期），右心室压力显著升高，逆转为右向左分流，

出现青紫，并逐渐加重，此时心脏杂音较轻而肺动脉第二音显著亢进。继发漏斗部肥厚时，则肺动脉第二音降低。

室间隔缺损易并发支气管炎、充血性心力衰竭、肺水肿及感染性心内膜炎。20%～50%的膜周部和肌部小梁部缺损在5 岁以内有自然闭合的可能，但大多发生于1 岁内。肺动脉下或双动脉下的漏斗隔缺损很少能闭合，且易发生主动脉脱垂致主动脉瓣关闭不全时，应早期处理。

【辅助检查】

1．X线检查 小型室间隔缺损心肺X线检查无明显改变，或肺动脉段延长或轻微突出，肺野轻度充血。中型缺损心影轻度到中度增大，左、右心室增大，以左室增大为主，主动脉弓影较小，肺动脉段扩张，肺野充血（图13-7）。大型缺损心影中度以上增大，呈二尖瓣型，左、右心室增大，多以右心室增大为主，肺动脉段明显突出，肺野明显充血。当肺动脉高压转为双向或右向左分流时，出现艾森曼格综合征，主要特点为肺动脉主支增粗，而肺外周血管影很少，宛如枯萎的秃枝，心影可基本正常或轻度增大。

2．心电图 小型缺损心电图可正常或表现为轻度左心室肥大；中型缺损主要为左心室舒张期负荷增加表现，RV_5、V_6升高伴深Q波，T波直立高尖对称，以左心室肥厚为主；大型缺损为双心室肥厚或右心室肥厚。症状严重、出现心力衰竭时，可伴有心肌劳损。

3．超声心动图 可解剖定位和测量大小，但<2 mm的缺损可能不被发现。二维超声可从多个切面显示缺损直接征象——回声中断的部位、时相、数目与大小等。彩色多普勒超声可显示分流束的起源、部位、数目、大小及方向。频谱多普勒超声可测量分流速度，计算跨隔压差和右室收缩压，估测肺动脉压。还可通过测定肺动脉瓣口和二尖瓣口血流量计算肺循环血流量（Qp）；测定主动脉瓣口和三尖瓣口血流量计算体循环血流量（Qs），正常时Qp/Qs≈1，此值增高≥1.5 提示为中等量左向右分流，≥2.0 为大量左向右分流。

4．心导管检查 进一步证实诊断及进行血流动力学检查，评价肺动脉高压程度、计算肺血管阻力及体肺分流量等。造影可示心腔形态、大小及心室水平分流束的起源、部位、时相、数目与大小，除外其他并发畸形等（图13-8）。

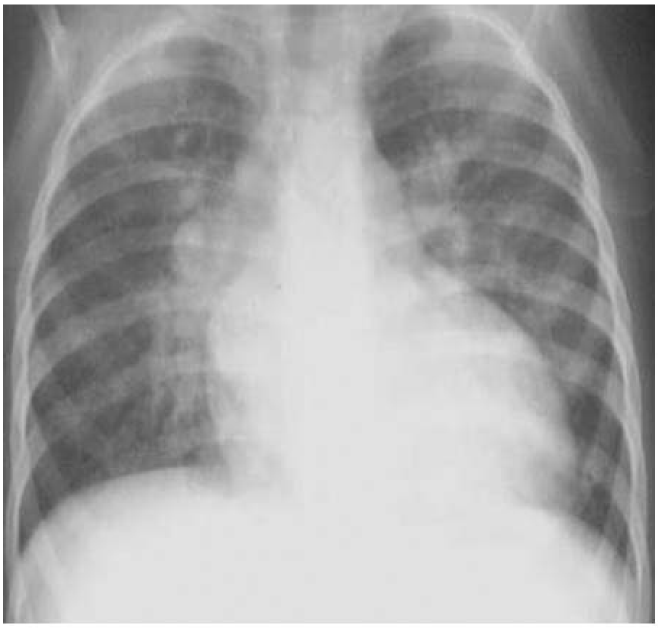

图13-7 室间隔缺损的典型X线特征

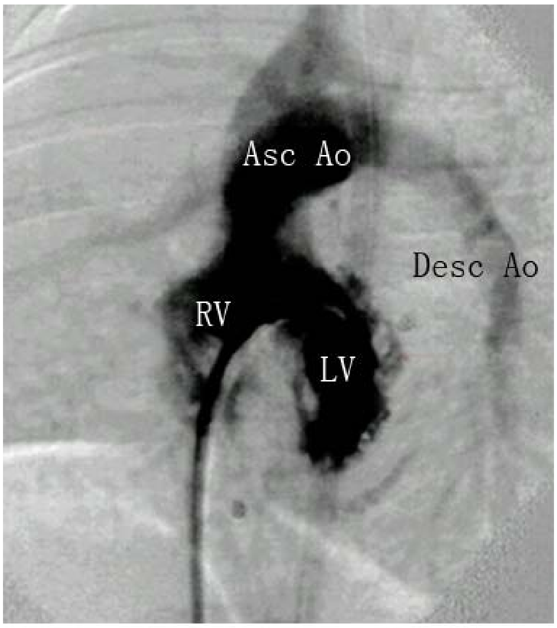

图13-8 室间隔缺损的心血管造影影像

RV：右心室；LV：左心室；Asc Ao：升主动脉；Desc Ao：降主动脉

【治疗】

室间隔缺损有自然闭合可能，中小型缺损可先在门诊随访至学龄前期，有临床症状如反复呼吸道感染和充血性心力衰竭时进行抗感染、强心、利尿、扩血管等内科处理。大中型缺损有难以控制的充血性心力衰竭者，肺动脉压力持续升高超过体循环压的1/2或肺循环/体循环量之比大于2：1 时，应及时处理。室间隔缺损治疗过去只能依靠外科体外循环下直视手术修补，随着介入医学的发展，应用可自动张开和自动置入的Amplatzer等装置经心导管堵塞进行非开胸的介入治疗，初步应用表明该方法对关闭肌部、部分膜部室缺是安全有效的，但远期疗效有待进一步的临床实践和研究证实。

三、动脉导管未闭

动脉导管未闭（patent ductus arteriosus）为小儿先天性心脏病常见类型之一，占先天性心脏病发病总数的15％。胎儿期动脉导管被动开放是血液循环的重要通道，出生后，大约15 小时即发生功能性关闭，80％在生后3 个月解剖性关闭。到出生后1 年，在解剖学上应完全关闭。若持续开放，并产生病理、生理改变，即称动脉导管未闭。

【病理分型及血流动力学】

1．病理分型 未闭的动脉导管的大小、长短和形态不一，一般分为三型：①管型：导管长度多在1 cm左右，直径粗细不等；②漏斗型：长度与管型相似，但其近主动脉端粗大，向肺动脉端逐渐变窄；③窗型：肺动脉与主动脉紧贴，两者之间为一孔道，直径往往较大。

2．血流动力学 出生后动脉导管关闭的机制包括多种因素。在组织结构方面，动脉导管的肌层丰富，含有大量凹凸不平的螺旋状弹性纤维组织，易于收缩闭塞。而出生后体循环中氧分压的增高，强烈刺激动脉导管平滑肌收缩。此外自主神经系统的化学介质（如激肽类）的释放也能使动脉导管收缩。

未成熟儿动脉导管平滑肌发育不良，更由于其平滑肌对氧分压的反应低于成熟儿，故早产儿动脉导管未闭发病率高，占早产儿的20％，且伴呼吸窘迫综合征发病率很高。

动脉导管未闭引起的病理生理学改变主要是通过导管引起的分流。分流量的大小与导管的粗细及主、肺动脉的压差有关。由于主动脉在收缩期和舒张期的压力均超过肺动脉，因而通过未闭动脉导管的左向右分流的血液连续不断，使肺循环及左心房、左心室、升主动脉的血流量明显增加，左心负荷加重，其排血量达正常时的2～4 倍（图13-9），部分患者左心室搏出量的70％可通过大型动脉导管进入肺动脉，导致左心房扩大，左心室肥厚扩大，甚至发生充血性心力衰竭。长期大量血流向肺循环的冲击，肺小动脉可有反应性痉挛，形成动力性肺动脉高压；继之管壁增厚硬化导致梗阻性肺动脉高压，此时右心室收缩期负荷过重，右心室肥厚甚至衰竭。当肺动脉压力超过主动脉压时，左向右分流明显减少或停止，产生肺动脉血流逆向分流入主动脉，患儿呈现差异性紫绀（differential cyanosis），下半身青紫，左上肢有轻度青紫，右上肢正常。

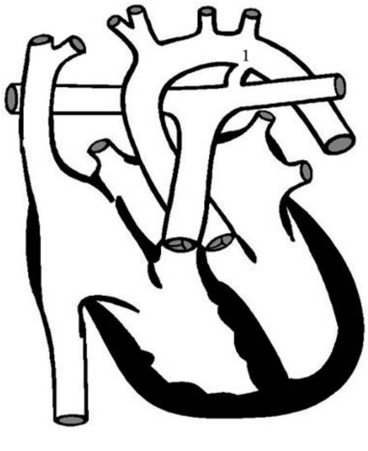

图 13-9 动脉导管未闭的模式图
1.动脉导管未闭

动脉导管未闭大都单独存在，但有10％的病例合并其他心脏畸形，如主动脉缩窄、室间隔缺损、肺动脉狭窄。

【临床表现】

1．症状 动脉导管细小者临床上可无症状。导管粗大者可有咳嗽、气急、喂养困难及生长发育落后等。

2．体征 胸骨左缘上方有一连续性“机器”样杂音，占整个收缩期与舒张期，于收缩末期最响，杂音向左锁骨下、颈部和背部传导，当肺血管阻力增高时，杂音的舒张期成分可能减弱或消失。分流量大者因相对性二尖瓣狭窄而在心尖部可闻及较短的舒张期杂音。肺动脉瓣区第二音增强，婴幼儿期因肺动脉压力较高，主、肺动脉压力差在舒张期不显著，因而往往仅听到收缩期杂音，当合并肺动脉高压或心力衰竭时，多仅有收缩期杂音。由于舒张压降低，脉压差增宽，并可出现周围血管体征，如水冲脉、指甲床毛细血管搏动等。

早产儿动脉导管未闭时，出现周围动脉搏动宏大，锁骨下或肩胛间闻及收缩期杂音（偶闻及连续性杂音），心前区搏动明显，肝脏增大，气促，并易发生呼吸衰竭而依赖机械辅助通气。

【辅助检查】

1．心电图 分流量大者可有不同程度的左心室肥大，偶有左心房肥大，肺动脉压力显著增高者，左、右心室肥厚，严重者甚至仅见右心室肥厚。

2．X线检查 动脉导管细者心血管影可正常。大分流量者心胸比率增大，左心室增大，心尖向下扩张，左心房亦轻度增大。肺血增多，肺动脉段突出，肺门血管影增粗（图13-10）。当婴儿有心力衰竭时，可见肺淤血表现，透视下左心室和主动脉搏动增强。肺动脉高压时，肺门处肺动脉总干及其分支扩大，而远端肺野肺小动脉狭小，左心室有扩大肥厚征象。主动脉结正常或凸出。

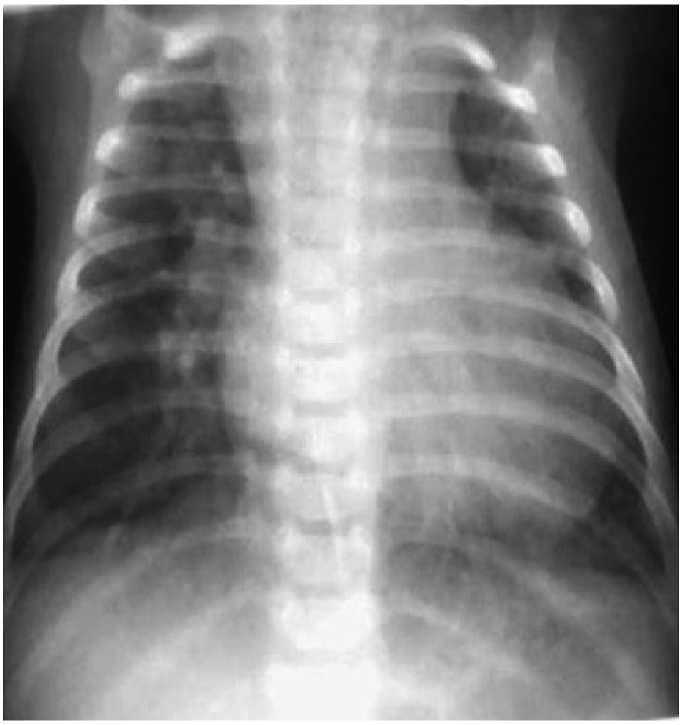

图 13-10 动脉导管未闭 X 线表现

3．超声心动图 对诊断极有帮助。二维超声心动图可以直接探查到未闭合的动脉导管，常选用胸骨旁肺动脉长轴观或胸骨上主动脉长轴观。脉冲多普勒在动脉导管开口处可探测到典型的收缩期与舒张期连续性湍流频谱。叠加彩色多普勒可见红色流柱出自降主动脉，通过未闭导管沿肺动脉外测壁流动；在重度肺动脉高压时，当肺动脉压超过主动脉时，可见蓝色流注自肺动脉经未闭导管进入降主动脉。

4．心导管检查 当肺血管阻力增加或疑有其他合并畸形时有必要施行心导管检查，它可发现肺动脉血氧含量较右心室为高。有时心导管可以从肺动脉通过未闭导管插入降主动脉。

5．心血管造影 逆行主动脉造影对复杂病例的诊断有重要价值，在主动脉根部注入造影剂可见主动脉与肺动脉同时显影，未闭动脉导管也能显影。

【并发症】

感染性动脉炎、充血性心力衰竭、心内膜炎等是常见的并发症。少见的并发症有肺动脉和动脉导管瘤样扩张、动脉导管钙化及血栓形成。

【治疗原则】

为防止心内膜炎，有效治疗和控制心功能不全和肺动脉高压，不同年龄、不同大小的动脉

导管均应手术或经介入方法予以关闭。早产儿动脉导管未闭的处理视分流大小、呼吸窘迫综合征情况而定。症状明显者，需抗心力衰竭治疗，生后一周内使用吲哚美辛治疗，但仍有10%的患者需手术治疗。采用介入疗法，选择弹簧圈（coil）、蘑菇伞（Amplatzer）等关闭动脉导管。

但在有些病例中，如完全性大血管转位、肺动脉闭锁、三尖瓣闭锁、严重的肺动脉狭窄中，动脉导管为依赖性者，对维持患婴生命至关重要，此时应该应用前列腺素E_2以维持动脉导管开放。

四、肺动脉瓣狭窄

肺动脉瓣狭窄（pulmonary stenosis，PS）是一种常见的先天性心脏病，单纯性肺动脉瓣狭窄约占先心病的10%，约有20%的先心病合并肺动脉瓣狭窄。

【病理解剖】

正常肺动脉瓣叶为三个半月瓣，瓣叶交界处完全分离，瓣环与右室漏斗部肌肉相连。肺动脉瓣狭窄根据病变累及的部位不同，分为两种类型：

1．典型肺动脉瓣狭窄 肺动脉瓣三个瓣叶交界处互相融合，使瓣膜开放受限，瓣口狭窄；只有两个瓣叶的交界处融合为肺动脉瓣二瓣化畸形；瓣叶无交界处仅中心部留一小孔，为单瓣化畸形。瓣叶结构完整，瓣环正常，肺动脉干呈狭窄后扩张，有时可延伸到左肺动脉，但扩张的程度与狭窄的严重性并不完全成比例。

2．发育不良型肺动脉瓣狭窄 肺动脉瓣叶形态不规则且明显增厚或呈结节状，瓣叶间无粘连，瓣叶启闭不灵活，瓣环发育不良，肺动脉干不扩张或发育不良。此病常有家族史，Noonan综合征大多合并此病变。

肺动脉瓣狭窄的继发性改变为右室向心性肥厚，狭窄严重者，心室腔小，心内膜下心肌可有缺血性改变。右心房有继发性增大，心房壁增厚，卵圆孔开放，或伴有房间隔缺损。

【病理生理】

右心室向肺动脉射血遇到瓣口狭窄的困阻，右室必须提高收缩压方能向肺动脉泵血，其收缩压提高的程度与狭窄的严重性成比例。因室间隔无缺损，所以严重狭窄时右心室的压力高度可以超过左心室。右心室的血流进入肺脏虽有困难，但全身所有静脉血仍必须完全进入肺脏。但如狭窄严重，右心室壁极度增厚使心肌供血不足，可导致右心衰竭（图13-11）。

在胎内，肺动脉瓣狭窄使右心室的心肌肥厚，右心室输出量仍可维持正常，对胎儿循环无多大影响；如狭窄很重，右心室输出量大减，腔静脉血回右心房后大多通过卵圆孔或房间隔缺损流入左心房、左心室，而右心室则偏小。临床上有一少见的肺动脉狭窄类型为右心室先天发育不良，三尖瓣也偏小，往往伴有大型房间隔缺缺，于是产生大量右向左分流，左心室偏大，青紫明显。

大多数患轻、中度肺动脉瓣狭窄的婴儿与儿童生长发育正常，因此体肺循环血流量随年龄而增长。如狭窄的肺动脉瓣不能相应生长，右心室收缩压必须明显增加以维持心输出量。此外，由于婴儿的正常静态心率高于年长儿，随着心率的下降，每搏量将相应增加，因而越过狭窄瓣膜的收缩期血流也将相应增加。

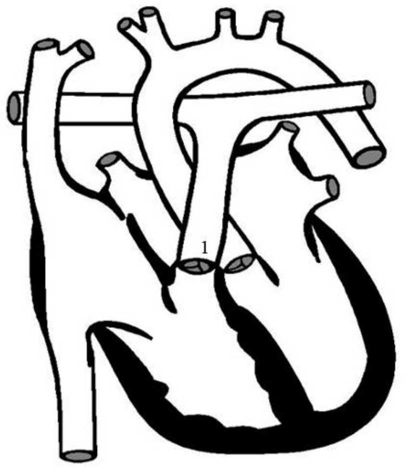

图 13-11 肺动脉瓣狭窄的模式图

1.肺动脉瓣狭窄

【临床表现】

1．症状 轻度狭窄可完全无症状；中度狭窄在2～3 岁内

无症状，但年长后劳力时即感易疲乏及气促；严重狭窄者中度体力劳动亦可呼吸困难和乏力，突有昏厥甚至猝死。亦有患者活动时感胸痛或上腹痛，可能由于心排出量不能相应提高，致使心肌供血不足或心律失常所致，提示预后不良，应着手准备手术。

生长发育多正常，半数患儿面容硕圆，大多无青紫，面颊和指端可能暗红；狭窄严重者可有青紫，大多由于卵圆孔的右向左分流所致，如伴有大型房间隔缺损可有严重青紫，并有杵状指趾及红细胞增多，但有蹲踞者很少见。

颈静脉有明显的搏动者提示狭窄严重，该收缩期前的搏动在肝区亦可扪及。

2．体征 心前区可较饱满，有严重狭窄伴有心力衰竭时心脏扩大；左侧胸骨旁可摸得右心室的抬举搏动，在心前区搏动弥散，甚至可延伸到腋前线。胸骨左缘第2、3 肋间可及收缩期震颤并可向胸骨上窝及胸骨左缘下部传导；新生儿患者可无震颤。听诊时胸骨左缘上部有洪亮的Ⅳ/Ⅵ级以上喷射性收缩杂音，向左上胸、心前区、颈部、腋下及背面传导。第一心音正常，轻度和中度狭窄者可听到收缩早期喀喇音，狭窄越重，喀喇音出现越早，甚至与第一音相重，使第一音呈金属样的声音。喀喇音系由于增厚但仍具弹性的瓣膜在开始收缩时突然绷紧所致。第二心音分裂，分裂程度与狭窄严重程度成比例。

【辅助检查】

1．X线检查 轻、中度狭窄时心脏大小正常，重度狭窄时如心功能尚可，心脏仅轻度增大；如有心力衰竭，心脏则明显增大，主要为右心室和右心房扩大。狭窄后的肺动脉扩张为本病特征性的改变，有时扩张延伸到左肺动脉，但在婴儿期扩张多不明显。

2．心电图 心电图将显示右房扩大、P波高耸。心电图还可显示右心室肥大、电轴右偏，其程度依赖于狭窄的严重程度。右胸前导联将显示R波高耸，狭窄严重时出现T波倒置、ST段压低。

3．超声心动图 二维超声心动图可显示肺动脉瓣的厚度、收缩时的开启情况及狭窄后的扩张。多普勒超声可检查心房水平有无分流，更重要的是可较可靠地估测肺动脉瓣狭窄的严重程度。

4．心导管检查 右心室压力明显增高，可与体循环压力相等，而肺动脉压力明显降低，心导管从肺动脉向右心室退出时的连续曲线显示明显的无过渡区的压力阶差。

5．心血管造影 右心室造影可见明显的“射流征”，同时可显示肺动脉瓣叶增厚和（或）发育不良及肺动脉总干的狭窄后扩张。

【治疗】

严重肺动脉瓣狭窄（右室收缩压超过体循环压力）患儿应接受球囊瓣膜成形术，如无该术适应证，则应接受外科瓣膜切开术。大多数严重肺动脉瓣狭窄伴有漏斗部狭窄，在大多数患儿，一旦肺动脉瓣狭窄解除，漏斗部肥厚将自行消退。轻度肺动脉瓣狭窄（右心室收缩压低于体循环收缩压）患儿的手术标准目前尚未确定，一般认为如右心室收缩压超过50 mmHg，则有可能导致心肌损害。因此可推荐行狭窄解除手术。球囊瓣膜成形术是大多数患儿的首选治疗方法。

五、法洛四联症

法洛四联症（tetralogy of Fallot，TOF）是婴儿期后最常见的青紫型先天性心脏病，约占所有先天性心脏病的10％。1888 年法国医生Etienne Fallot详细描述了该病的病理改变及临床表现，故而得名。

【病理解剖】

法洛四联症由4种畸形组成：

1．右室流出道梗阻 狭窄范围可自右心室漏斗部入口至左、右肺动脉分支。可为漏斗部狭窄、动脉瓣狭窄或两者同时存在。常有肺动脉瓣环、肺动脉总干的发育不良和肺动脉分支的非对称性狭窄。狭窄的严重程度差异较大。

2．室间隔缺损 缺损为膜部周围型缺损并向流出道延伸，多位于主动脉下，有时可向肺动脉下方延伸，称对位不良型室间隔缺损。

3．主动脉骑跨 主动脉根部粗大且顺钟向旋转右移并骑跨在室间隔缺损上，骑跨范围在15％～95％。

4．右心室肥厚 属继发性病变。

以上四种畸形中室间隔缺损必须足够大使左右心室的压力相等，右心室流出道狭窄是决定患儿的病理生理、病情严重程度及预后的主要因素。狭窄可随时间推移而逐渐加重。

本病可合并其他心血管畸形，如25％的四联症患儿为右位型主动脉弓；其他如左上腔静脉残留、冠状动脉异常、房间隔缺损、动脉导管未闭、肺动脉瓣缺如等。

【病理生理】

由于室间隔缺损为非限制性，左右心室压力基本相等。右心室流出道狭窄程度的不同，心室水平可出现左向右、双向甚至右向左分流。肺动脉狭窄较轻至中度者，可有左向右分流，此时患者可无明显的青紫；肺动脉狭窄严重时，出现明显的右向左分流，临床出现明显的青紫（青紫型法洛四联症）。临床上的杂音由右心室流出道梗阻所致而非室间隔缺损。右心室流出道的梗阻使右心室后负荷加重，引起右心室的代偿性肥厚。

由于主动脉骑跨于两心室之上，主动脉除接受左心室的血液外，还直接接受一部分来自右心室的静脉血，输送到全身各部，因而出现青紫；同时因肺动脉狭窄，肺循环进行气体交换的血流减少，更加重了青紫的程度。此外，由于进入肺动脉的血流减少，增粗的支气管动脉与肺血管之间形成侧支循环（图13-12）。

在动脉导管关闭前，肺循环血流量减少程度较轻，青紫可不明显，随着动脉导管的关闭和漏斗部狭窄的逐渐加重，青紫日益明显，并出现杵状指（趾）。由于缺氧，刺激骨髓代偿性产生过多的红细胞，血液黏稠度高，血流缓慢，可引起脑血栓，若为细菌性血栓，则易形成脑脓肿。

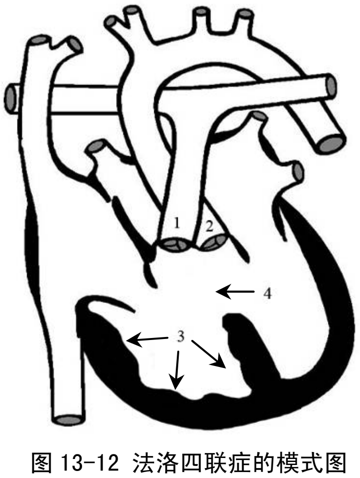

图 13-12 法洛四联症的模式图
1．右室漏斗部及肺动脉瓣狭窄；2．主动脉骑跨；3．右室肥厚；4．室间隔缺损

【临床表现】

1．青紫 为其主要表现，其程度和出现的早晚与肺动脉狭窄程度有关。多见于毛细血管丰富的浅表部位，如唇、指（趾）甲床、球结合膜等。因血氧含量下降，活动耐力差，稍一活动如啼哭、情绪激动、体力劳动、寒冷等，即可出现气急及青紫加重。

2．蹲踞症状 患儿多有蹲踞症状，每于行走、游戏时，常主动下蹲片刻。蹲踞时下肢屈曲，使静脉回心血量减少，减轻了心脏负荷，同时下肢动脉受压，体循环阻力增加，使右向左分流量减少，从而缺氧症状暂时得以缓解。不会行走的小婴儿，常喜欢大人抱起，双下肢屈曲状。

3．杵状指（趾） 患儿长期处于缺氧环境中，可使指、趾端毛细血管扩张增生，局部软组织和骨组织也增生肥大，表现为指（趾）端膨大如鼓槌状。

4．阵发性缺氧发作 多见于婴儿，发生的诱因为吃奶、哭闹、情绪激动、贫血、感染等。表现为阵发性呼吸困难，严重者可引起突然昏厥、抽搐，甚至死亡。其原因是由于在肺动脉漏斗部狭窄的基础上，突然发生该处肌部痉挛，引起一时性肺动脉梗阻，使脑缺氧加重所致。年

长儿常诉头痛、头昏。

体格检查时，患儿生长发育一般均较迟缓，智能发育亦可能稍落后于正常儿。心前区略隆起，胸骨左缘第2、3、4 助间可闻及Ⅱ～Ⅲ级粗糙喷射性收缩期杂音，此为肺动脉狭窄所致，一般无收缩期震颤。肺动脉第二音减弱。部分患儿可听到亢进的第二心音，乃由右跨之主动脉传来。狭窄极严重者或在阵发性呼吸困难发作时，可听不到杂音。有时可听到侧支循环的连续性杂音。发绀持续6 个月以上，出现柱状指（趾）。

常见的并发症为脑血栓、脑脓肿及感染性心内膜炎。

【辅助检查】

1. 血液检查 周围血红细胞计数和血红蛋白浓度明显增高，红细胞可达（5.0～8.0）$\times 10^{12}$/L，血红蛋白170～200 g/L，红细胞比容也增高，为53 vol%～80 vol%。血小板降低，凝血酶原时间延长。

2．X 线检查 心脏大小一般正常或稍增大，典型者前后位心影呈“靴状”，即心尖圆钝上翘，肺动脉段凹陷，上纵隔较宽，肺门血管影缩小，两侧肺纹理减少，透亮度增加，年长儿可因侧支循环形成，肺野呈网状纹理，25%的患儿可见到右位主动脉弓阴影（图13-13）。

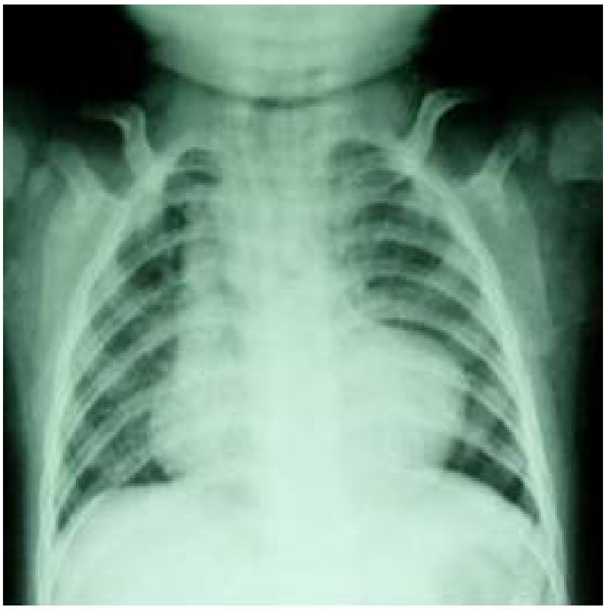

图 13-13 法洛四联症的典型 X 线特征

3．心电图 典型病例示电轴右偏，右心室肥大，狭窄严重者往往出现心肌劳损，可见右心房肥大。

4．超声心动图 二维超声左心室长轴切面可见到主动脉内径增宽，骑跨于室间隔之上，室间隔中断，并可判断主动脉骑跨的程度；大动脉短轴切面可见到右心室流出道及肺动脉狭窄。此外，右心室、右心房内径增大，左心室内径缩小，彩色多普勒血流显像可见右心室直接将血液注入骑跨的主动脉内。

5. 心导管检查 右心室压力明显增高，可与体循环压力相等，而肺动脉压力明显降低，心导管从肺动脉向右心室退出时的连续曲线显示明显的压力阶差。可根据连续曲线的形态来判断狭窄的类型，心导管较容易从右心室进入主动脉或左心室，说明主动脉右跨与室间隔缺损的存在。导管不易进入肺动脉，说明肺动脉狭窄较重。股动脉血氧饱和度降低，常小于89%，说明右向左分流的存在。

6．心血管造影 典型表现是造影剂注入右心室后可见到主动脉与肺动脉几乎同时显影。通过造影剂能见到室间隔缺损的位置，增粗的主动脉阴影，且位置偏前、稍偏右。了解肺动脉狭窄的部位和程度以及肺动脉分支的形态。选择性左心室及主动脉造影可进一步了解左心室发育的情况及冠状动脉的走向。此外，通过造影可发现伴随的畸形，这对制订手术方案和估侧预后至关重要。

【治疗】

1． 内科治疗

（1）一般护理：平时应经常饮水，预防感染，及时补液，防治脱水和并发症。婴幼儿则需特别注意护理，以免引起阵发性缺氧发作。

（2）缺氧发作的治疗：发作轻者使其取胸膝位即可缓解，重者应立即吸氧，给予去氧肾

上腺素每次0.05 mg/kg静注，或普萘洛尔每次0.1 mg/kg。必要时也可皮下注射吗啡每次0.1～0.2 mg/kg，纠正酸中毒，给予5％碳酸氢钠1.5～5.0 ml/kg静注。以往有缺氧发作者，可口服普萘洛尔1～3 mg/（kg・d）。平时应去除引起缺氧发作的诱因如贫血、感染，尽量保持患儿安静，经上述处理后仍不能有效控制发作者，应考虑急症外科手术修补。

2．外科治疗 近年来外科手术不断的进展，本病根治术的死亡率在不断下降。轻症患者可考虑于5～9 岁行一期根治手术，但临床症状明显者应在生后6～12 个月行根治术。对重症患儿也可先行姑息手术，待一般情况改善，肺血管发育好转后，再作根治术。目前常用的姑息手术有：锁骨下动脉-肺动脉吻合术（Blalock-Taussig手术），上腔静脉-右肺动脉吻合术（Glenn手术）等。

六、完全性大动脉转位

完全性大动脉转位（complete transposition of the great arteries，c-TGA）是新生儿期最常见的发绀型先天性心脏病，发病率为0.2‰～0.3‰，占先天性心脏病总数的5％～7％，居发绀型先心病的第二位，男女患病之比为2～4：1。患有糖尿病孕妇分娩的婴儿患本病的发病率较正常母体高达11.4 倍，妊娠初期使用过激素及抗惊厥药物的孕妇分娩的婴儿患本病的发病率较高。若不治疗，约90％的患者在1 岁内死亡。

【病理解剖】

正常情况下，肺动脉瓣下圆锥发育，肺动脉位于左前上方；主动脉瓣下圆锥萎缩，主动脉位于右后下方。大动脉转位时，主动脉瓣下圆锥发达，未被吸收，主动脉位于右前上方；肺动脉瓣下圆锥萎缩，肺动脉位于左后下方。这样使肺动脉向后连接左心室，主动脉向前连接右心室；主动脉瓣下因有圆锥存在，与三尖瓣间呈肌性连接；肺动脉瓣下无圆锥结构存在，与二尖瓣呈纤维连接。常见的合并畸形有：房间隔缺损或卵圆孔未闭、室间隔缺损、动脉导管未闭、肺动脉狭窄等。

【病理生理】

完全性大动脉转位若不伴其它畸形，则形成两个并行循环。上、下腔静脉回流的静脉血通过右心射至转位的主动脉供应全身，而肺静脉回流的氧合血则通过左心射入转位的肺动脉到达肺部。患者必须依靠心内交通（卵圆孔未闭、房间隔缺损、室间隔缺损）或心外交通（动脉导管未闭、侧支血管）进行血流混合（图13-14）。本病血液动力学改变取决于是否伴同其他畸形，左右心血液沟通混合程度及肺动脉是否狭窄。根据是否合并室间隔缺损及肺动脉狭窄可将完全性大动脉转位分为三大类：

1．完全性大动脉转位而室间隔完整 右心室负荷增加而扩大肥厚，随正常的肺血管阻力下降，左心室压力降低，室间隔常偏向左心室。二者仅靠未闭的卵圆孔及动脉导管沟通混合，故青紫、缺氧严重。

2．完全性大动脉转位合并室间隔缺损 完全性大动脉转位伴室间隔缺损可使左右心血液沟通混合较多，使青紫减轻，但肺血流量增加可导致心力衰竭。

3．完全性大动脉转位合并室间隔缺损及肺动脉狭窄 血液动力学改变类似法洛四联症。

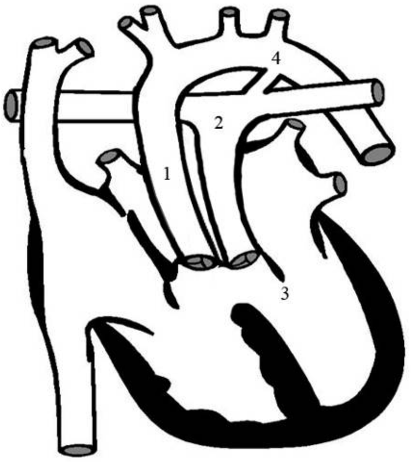

图 13-14 完全性大动脉转位的模式图

1．主动脉；2．肺动脉；3．室间隔缺损；4．动脉导管未闭

【临床表现】

1．青紫 出现早，半数出生时即存在，绝大多数始于1 个

月内。随着年龄增长及活动量增加，青紫逐渐加重。青紫为全身性，若同时合并动脉导管未闭，则出现差异性发绀，上肢青紫较下肢重。

2．充血性心力衰竭 生后3～4 周婴儿出现喂养困难、多汗、气促、肝大和肺部细湿啰音等进行性充血性心力衰竭等症状。患儿常发育不良。

3．体检发现 早期出现杵状指、趾。生后心脏可无明显杂音，但有单一的响亮的第二心音，是出自靠近胸壁的主动脉瓣关闭音。若伴有大的室隔缺损或大的动脉导管或肺动脉狭窄等，则可听到相应畸形所产生的杂音。如合并动脉导管未闭，可在胸骨左缘第2 肋间听到连续性杂音。合并室间隔缺损，可在胸骨左缘第3、4 肋间听到全收缩期杂音。合并肺动脉狭窄，可在胸骨左缘上方听到收缩期喷射性杂音。杂音较响时，常伴有震颤。一般伴有大型室隔缺损者早期出现心力衰竭伴肺动脉高压；但伴有肺动脉狭窄者则发绀明显，而心力衰竭少见。

【辅助检查】

1．X线检查 主要表现为：①由于主、肺动脉干常呈前后位排列，因此正位片见大动脉阴影狭小，肺动脉略凹陷，心蒂小而心影呈“蛋形”；②心影进行性增大；③大多数患者肺纹理增多，若合并肺动脉狭窄者肺纹理减少。

2．心电图 新生儿期可无特殊改变。婴儿期示电轴右偏，右心室肥大，有时尚有右心房肥大。肺血流量明显增加时则可出现电轴正常或左偏，左右心室肥大等。合并房室通道型室间隔缺损时电轴左偏，双心室肥大。

3．超声心动图 是诊断完全性大动脉转位的常用方法。若二维超声显示房室连接正常，心室大动脉连接不一致，则可建立诊断。主动脉常位于右前，发自右心室；肺动脉位于左后，发自左心室。彩色及频谱多普勒超声检查有助于心内分流方向、大小的判定及合并畸形的检出。

4．心导管检查 导管可从右心室直接插入主动脉，右心室压力与主动脉相等。也有可能通过卵圆孔或房间隔缺损到左心腔再入肺动脉，肺动脉血氧饱和度高于主动脉。

5．心血管造影 选择性右心室造影时可见主动脉发自右心室，左心室造影可见肺动脉发自左心室。选择性升主动脉造影可显示大动脉的位置关系，判断是否合并冠状动脉畸形。

【治疗】

诊断后首先纠正低氧血症和代谢性酸中毒等。

1．姑息性治疗方法

（1）球囊房隔成形术（Rashkind procedure）：缺氧严重而又不能进行根治手术时可行球囊房隔造漏或房间隔缺损扩大术，使血液在心房水平大量混合，提高动脉血氧饱和度，使患儿存活至适合根治手术。

（2）肺动脉环缩术：完全性大动脉转位伴大型室间隔缺损者，可在6 个月内作肺动脉环缩术，预防充血性心力衰竭及肺动脉高压引起的肺血管病变。

2．根治性手术

（1）生理纠治术（Senning或Mustard手术）：可在生后1～12 个月内进行，即用心包膜及心房壁在心房内建成板障，将体循环的静脉血导向二尖瓣口而入左心室，并将肺静脉的回流血导向三尖瓣口而入右心室，形成房室连接不一致及心室大血管连接不一致，以达到生理上的纠治。

（2）解剖纠正手术（switch手术）：可在生后4 周内进行，即主动脉与肺动脉互换及冠状动脉再植，达到解剖关系上的纠正。手术条件为：左/右心室压力比> 0.85，左心室射血分数> 0.45，左心室舒张末期容量>正常的90 %，左心室后壁厚度>4 ～4.5 mm，室壁张力<12000 达

因/cm。

第五节 病毒性心肌炎

心肌炎由各种感染性、中毒性、结缔组织性过程侵犯心肌所至。最常见的是病毒性心肌炎（viral myocarditis），其病理特征为心肌细胞的坏死或变性，有时病变也可累及心包或心内膜。儿童期的发病率尚不确切。国外资料显示在因意外事故死亡的年轻人尸体解剖中检出率为4%～5%。

【病因】

引起儿童心肌炎的常见病毒有柯萨奇病毒（B 组和A 组）、埃可病毒、脊髓灰质炎病毒、腺病毒、传染性肝炎病毒、流感和副流感病毒、麻疹病毒及单纯疱疹病毒以及流行性腮腺炎病毒等。值得注意的是新生儿期柯萨奇病毒B组感染可导致群体流行，其死亡率可高达50%以上。

【发病机制】

本病的发病机理尚不完全清楚。但随着分子病毒学、分子免疫学的发展，揭示出病毒性心肌炎发病机制涉及到病毒对被感染的心肌细胞直接损害和病毒触发人体自身免疫反应而引起心肌损害。病毒性心肌炎急性期，柯萨奇病毒和腺病毒通过心肌细胞的相关受体侵入心肌细胞，在细胞内复制，并直接损害心肌细胞，导致变性、坏死和溶解。机体受病毒的刺激，激活细胞和体液免疫反应，产生抗心肌抗体、白细胞介素-Ⅰα、肿瘤坏死因子α和γ干扰素等诱导产生细胞黏附因子，促使细胞毒性T细胞（$CD8^{+}$）有选择地向损害心肌组织黏附、浸润和攻击。

【临床表现】

1．症状 表现轻重不一，取决于年龄和感染的急性或慢性过程。预后大多良好，部分患者起病隐匿，有乏力、活动受限、心悸、胸痛症状，少数重症患者可发生心力衰竭并发严重心律紊乱、心源性休克，甚至猝死。部分患者呈慢性进程，演变为扩张性心肌病。新生儿患病时病情进展快，常见高热、反应低下、呼吸困难和发绀，常有神经、肝脏和肺的并发症。

2．体征 心脏有轻度扩大，伴心动过速、心音低钝及奔马律，可导致心力衰竭及昏厥等。反复心衰者，心脏明显扩大，肺部出现湿啰音及肝、脾肿大，呼吸急促和紫绀，重症患者可突然发生心源性休克，脉搏细弱，血压下降。

【辅助检查】

1．心电图 可见严重心律失常：包括各种期前收缩，室上性和室性心动过速，房颤和室颤，Ⅱ度或Ⅲ度房室传导阻滞。心肌受累明显时可见T波降低、ST-T段的改变，但是心电图缺乏特异性，强调动态观察的重要性。

2．心肌损害血生化指标

（1）磷酸激酶（CPK）：在早期多有增高，其中以来自心肌的同工酶（CK-MB）为主。血清乳酸脱氢酶（SLDH）同工酶增高在心肌炎早期诊断有提示意义。

（2）近年来通过随访观察发现心肌肌钙蛋白（cTnI或cTnT）的变化对心肌炎诊断的特异性更强。

3．超声心动图检查 可显示心房、心室的扩大，心室收缩功能受损程度，探查有无心包积液以及瓣膜功能。

4．病毒学诊断 疾病早期可从咽拭子、咽冲洗液、粪便、血液中分离出病毒，但需结合血清抗体测定才更有意义。恢复期血清抗体滴度比急性期有4 倍以上增高、病程早期血中特异性IgM抗体滴度在1：128以上，利用聚合酶链反应或病毒核酸探针原位杂交自血液或心肌组织中

查到病毒核酸可作为某一型病毒存在的依据。

5．心肌活体组织检查 仍被认为是诊断的金标准，但由于取样部位的局限性，阳性率仍然不高。

【诊断】

病毒性心肌炎诊断标准（中华医学会儿科学分会心血管学组中华儿科杂志编辑委员会，1999 年）：

1．临床诊断依据

（1）心功能不全、心源性休克或心脑综合征。

（2）心脏扩大（X线、超声心动图检查具有表现之一）。

（3）心电图改变：以R波为主的2 个或2 个以上主要导联（Ⅰ，Ⅱ，aVF，V_5）的ST-T改变持续4 天以上伴动态变化，窦房、房室传导阻滞，完全右或左束支传导阻滞，成联律、多型、多源、成对或并行期前收缩，非房室结及房室折返引起的异位性心动过速，低电压（新生儿除外）及异常Q波。

（4）CK-MB升高或心肌肌钙蛋白（cTnI或cTnT）阳性。

2．病原学诊断依据

（1）确准指标：自心内膜、心肌、心包（活体组织检查、病理）或心包穿刺液检查发现以下之一者可确诊。①分离到病毒；②用病毒核酸探针查到病毒核酸；③特异性病毒抗体阳性。

（2）参考依据：有以下之一者结合临床表现可考虑心肌炎由病毒引起。①自粪便、咽试子或血液中分离到病毒，且恢复期血清同型抗体滴度较第一份血清升高或降低4 倍以上；②病程早期血中特异性IgM抗体阳性；③用病毒核酸探针自患儿血中查到病毒核酸。

（3）确诊依据：具备临床诊断依据两项，可临床诊断。发病同时或发病前1～3 周有病毒感染的证据支持诊断者：①同时具备病原学确诊依据之一者，可确诊为病毒性心肌炎；②具备病原学参考依据之一者，可临床诊断为病毒性心肌炎；③凡不具备确诊依据，应给予必要的治疗或随诊，根据病情变化，确诊或除外心肌炎。应除外风湿性心肌炎、中毒性心肌炎、先天性心脏病、由风湿性疾病以及代谢性疾病（如甲状腺功能亢进症）引起的心肌损害、原发性心肌病、原发性心内膜弹力纤维增生症、先天性房室传导阻滞、心脏自主神经功能异常、β受体功能亢进及药物引起的心电图改变。

【治疗】

1．休息 急性期需卧床休息，减轻心脏负荷。

2．药物治疗

（1）对于仍处于病毒血症阶段的早期患者，可选用抗病毒治疗，但疗效不确定。

（2）改善心肌营养：1, 6 二磷酸果糖有益改善心肌能量代谢，促进受损细胞的修复，常用剂量为100～250 mg/kg，静脉滴注，疗程10～14 天。同时可选用大剂量VitC、泛醌（CoQ10）、VitE和VitBco。中药生脉饮、黄芪口服液等。

（3）大剂量丙种球蛋白：通过免疫调节作用减轻心肌细胞损害，剂量2 g/kg，2～3 天内分次静脉滴注。

（4）皮质激素：通常不使用。对重型患者合并心源性休克、致死性心律紊乱（Ⅲ度房室传导阻滞、室性心动过速）、心肌活体组织检查证实慢性自身免疫性心肌炎症反应者应足量、早期应用。

（5）心律紊乱治疗：参见本章第六节。

（6）其他治疗：可根据病情联合应用利尿剂、洋地黄和血管活性药物，应特别注意用洋

地黄时饱和量应较常规剂量减少，并注意补充氯化钾，以避免洋地黄中毒。

第六节 心内膜弹力纤维增生症

心内膜弹力纤维增生症（endocardial fibroelastosis）的主要病理改变为心内膜下弹力纤维及胶原纤维增生，病变以左心室为主。多数于1 岁以内发病。原因尚未完全明确，部分病例可能由病毒性心肌炎发展而来；心内膜供血不足及缺氧亦很可能为发病的原因，约9％病人有遗传倾向。原发性心内膜弹力纤维增生症没有明显瓣膜损害和其他先天性心脏畸形；而继发性心内膜弹力纤维增生症有左心梗阻型的先天性心脏病如：严重主动脉缩窄、左心发育不良综合征、主动脉瓣闭锁或狭窄。

【临床表现】

主要表现为充血性心力衰竭，按症状的轻重缓急，可分为三型。

1．暴发型 起病急骤，突然出现呼吸困难、口唇发绀、面色苍白、烦躁不安、心动过速、心音减低，可听到奔马律，肺部常听到干、湿啰音，肝脏增大，少数出现心源性休克，甚至于数小时内猝死。此型多见于6 个月内的婴儿。

2．急性型 起病亦较快，但心力衰竭发展不如暴发型急剧。常并发支气管炎，肺部出现细湿啰音。部分患者因心腔内附壁血栓的脱落而发生脑栓塞。此型发病年龄同暴发型。如不及时治疗，多数死于心力衰竭。

3．慢性型 症状同急性型，但进展缓慢。患儿生长发育多较落后。经适当治疗可获得缓解，存活至成年期，但仍可因反复发生心力衰竭而死亡。

【诊断】

除发病年龄特点和临床表现以充血性心力衰竭为主以外，实验室检查亦有其特点：心电图多呈左心室肥大，少数表现右心室肥大或左、右心室合并肥大，可同时出现ST段、T波改变以及房室传导阻滞。X线改变以左心室肥大为明显，左心缘搏动多减弱，肺纹理增多。必要时可作左心导管检查，左室舒张压增高，其波形具有诊断意义。选择性造影则可见左心室增大，室壁增厚及排空延迟。

【治疗】

主要应用洋地黄控制心力衰竭，一般反应较好，需长期服用，直到症状消失，X线、心电图恢复正常后1～2 年方可停药。合并肺部感染时，应给予抗生素等治疗。

本病如不治疗，大多于2 岁前死亡。对洋地黄反应良好而又能长期坚持治疗者，预后较好，且有痊愈可能。

第七节 感染性心内膜炎

心内膜炎（endocarditis）指各种原因引起的心内膜炎症病变，常累及心脏瓣膜，也可累及室间隔缺损处、心内壁内膜或未闭动脉导管、动静脉瘘等处，按原因可分为感染性和非感染性两大类。非感染性心内膜炎包括：风湿性心内膜炎、类风湿性心内膜炎、系统性红斑狼疮性心内膜炎、新生儿急性症状性心内膜炎等，本节主要阐述感染性心内膜炎。

感染性心内膜炎（infective endocarditis）在过去常分为急性和亚急性两个类型，急性者多发生于原无心脏病的患儿，侵入细菌毒力较强，起病急骤，进展迅速，病程在6 周以内。亚急性者多在原有心脏病的基础上感染毒力较弱的细菌，起病潜隐，进展相对缓慢，病程超过6 周。由于抗生素的广泛应用，本病的病程已延长，临床急性和亚急性难以截然划分。致病微生物除

了最常见的细菌外，尚有真菌、衣原体、立克次体及病毒等。近年来随着新型抗生素的不断出现，外科手术的进步，感染性心内膜炎死亡率已显著下降，但由于致病微生物的变迁，心脏手术和心导管检查的广泛开展，长期静脉插管输液的增多等因素，本病的发病率并无显著下降。

【病因】

1．心脏的原发病变 92％的感染性心内膜炎患者均有原发心脏病变，其中以先天性心脏病最为多见，约占78％，室间隔缺损最易合并感染性心内膜炎，其他依次为法洛四联症、动脉导管未闭、肺动脉瓣狭窄、主动脉瓣狭窄、主动脉瓣二叶畸形，房间隔缺损等；后天性心脏病如风湿性瓣膜病、二尖瓣脱垂综合征等也可并发感染性心内膜炎。随着小儿心脏外科技术的发展，越来越多的小儿心脏病得以纠正、根治，但因此而留置在心腔内的装置或材料（如心内补片、人造心脏瓣等）是近年感染性心内膜炎常见的易患因素。

2．病原体 几乎所有细菌均可导致感染性心内膜炎，草绿色链球菌仍为最常见的致病菌，但所占比例已显著下降。近年金黄色葡萄球菌、白色葡萄球菌，以及肠球菌、产气杆菌等革兰阴性杆菌引起的感染性心内膜炎显著增多。真菌性心内膜炎极少见，多有其他致病因素如长期应用抗生素、糖皮质激素或免疫抑制剂等。立克次体及病毒感染所致的心内膜炎甚罕见。少数情况下，感染性心内膜炎由一种以上的病原体引起，常见于人工瓣膜手术者。

3．诱发因素 约1/3的患儿在病史中可找到诱发因素，常见的诱发因素为纠治牙病和扁桃体摘除术。近年心导管检查和介入性治疗、人工瓣膜置换、心内直视手术的广泛开展，也是感染性心内膜炎的重要诱发因素之一，其他诱发因素如长期使用抗生素、糖皮质激素和免疫抑制剂等。

【病理和病理生理】

正常人口腔和上呼吸道常聚集一些细菌，一般不会致病，只有在机体防御功能低下时可侵入血流，特别是口腔感染、拔牙、扁桃体摘除术时易侵入血流，当心腔内膜，特别是心瓣膜存在病理改变或先天性缺损时，细菌易在心瓣膜、心内膜和动脉内膜表面粘着、繁殖，从而形成心内膜炎。但尚需存在双侧心室或大血管间较大的压力差，能够产生高速的血流，冲击心内膜面，使之损伤并暴露心内膜下胶原组织，与血小板和纤维蛋白聚积形成无菌性赘生物。当有菌血症时，细菌易在上述部位黏附、定植和繁殖，形成有菌赘生物。

受累部位多在压力低的一侧，如室间隔缺损感染性赘生物常见于缺损的右缘、三尖瓣的隔叶及肺动脉瓣；动脉导管在肺动脉侧；主动脉瓣关闭不全在左心室等。狭窄瓣孔及异常通道两侧心室或管腔之间的压力差越大、湍流越明显，压力低的一侧越易形成血栓和赘生物。房间隔缺损、大型室间隔缺损并发心力衰竭时，由于异常通道两侧压力差减小，血流速度减慢，湍流相对不明显，一般较少并发感染性心内膜炎。

基本病理改变是心瓣膜、心内膜及大血管内膜面附着疣状感染性赘生物。赘生物由血小板、白细胞、红细胞、纤维蛋白、胶原纤维和致病微生物等组成。心脏瓣膜的赘生物可致瓣膜溃疡、穿孔；若累及腱索和乳头肌，可使腱索缩短及断裂。累及瓣环和心肌，可致心肌脓肿、室间隔穿孔和动脉瘤，大的或多量的赘生物可堵塞瓣膜口或肺动脉，致急性循环障碍。

赘生物受高速血流冲击可有血栓脱落，随血流散布到全身血管导致器官栓塞。右心的栓子引起肺栓塞；左心的栓子引起肾、脑、脾、四肢、肠系膜等动脉栓塞。微小栓子栓塞毛细血管产生皮肤瘀点，即欧氏小结（Osler’s node）。肾栓塞时可致梗塞、局灶性肾炎或弥漫性肾小球肾炎。脑栓塞时可发生脑膜、脑实质、脊髓、颅神经等弥漫性炎症，产生出血、水肿、脑软化、脑脓肿、颅内动脉瘤破裂等病变。后者破裂可引起颅内各部位的出血如脑出血、蛛蛛膜下腔出血。

【临床表现】

起病缓慢，症状多种多样。大多数患者有器质性心脏病，部分患者发病前有龋齿、扁桃体炎、静脉插管、介入治疗或心内手术史。

1．感染症状 发热是最常见的症状，几乎所有的病例都有过不同程度的发热，热型不规则，热程较长，个别病例无发热。此外患者有疲乏、盗汗、食欲减退、体重减轻、关节痛、皮肤苍白等表现，病情进展较慢。

2．心脏方面的症状 原有的心脏杂音可因心脏瓣膜的赘生物而发生改变，出现粗糙、响亮、呈海鸥鸣样或音乐样的杂音。原无心脏杂音者可出现音乐样杂音，约一半患儿由于心瓣膜病变、中毒性心肌炎等导致充血性心力衰竭，出现心音低钝、奔马律等。

3．栓塞症状 视栓塞部位的不同而出现不同的临床表现，一般发生于病程后期，但约1/3的患者为首发症状，皮肤栓塞可见散在的小瘀点，指趾屈面可有隆起的紫红色小结节，略有触痛，此即欧氏小结；内脏栓塞可致脾大、腹痛、血尿、便血，有时脾大很显著；肺栓塞可有胸痛、咳嗽、咯血和肺部啰音；脑动脉栓塞则有头痛、呕吐、偏瘫、失语、抽搐甚至昏迷等。病程久者可见杵状指、趾，但无发绀。

同时具有以上三方面症状的典型患者不多，尤其2 岁以下婴儿往往以全身感染症状为主，仅少数患儿有栓塞症状和（或）心脏杂音。

【实验室检查】

1．血培养 血细菌培养阳性是确诊感染性心内膜炎的重要依据，凡原因未明的发热、体温持续在1 周以上，且原有心脏病者，均应反复多次进行血培养，以提高阳性率。若血培养阳性，尚应做药物敏感试验。

2．超声心动图 超声心动图检查能够检出直径大于2 mm以上的赘生物，因此对诊断感染性心内膜炎很有帮助，此外在治疗过程中超声心动图还可动态观察赘生物大小、形态、活动和瓣膜功能状态，了解瓣膜损害程度，对决定是否做换瓣手术有参考价值。该检查还可发现原有的心脏病。

3．CT检查 对怀疑有颅内病变者应及时做CT，了解病变部位和范围。

4．其他 血常规可见进行性贫血，多为正细胞性贫血，白细胞数增高和中性粒细胞升高，血沉快，C反应蛋白阳性，血清球蛋白常常增多，免疫球蛋白升高，循环免疫复合物及类风湿因子阳性，尿常规有红细胞，发热期可出现蛋白尿。

【诊断】

小儿感染性心内膜炎的诊断标准（2004 年试行草案，中华医学会儿科学会心血管学组）：

1．临床指标

（1）主要指标：

1）血培养阳性：分别2 次血培养有相同的感染性心内膜炎常见的微生物（如草绿色链球菌，金黄色葡萄球菌，肠球菌等）。

2）心内膜受累证据：应用超声心动图检查心内膜受累证据，有以下超声心动图征象之一：①附着于瓣膜或瓣膜装置，或心脏、大血管内膜、或置植入人工材料上的赘生物；②心内脓肿；③瓣膜穿孔、人工瓣膜或缺损补片有新的分裂。

3）血管征象：重要动脉栓塞，脓毒性肺梗死或感染性动脉瘤。

（2）次要指标：

1）易感染条件：基础心脏疾病，心脏手术，心导管术，或中心静脉内插管。

2）较长时间的发热（≥38℃），伴贫血。

3）原有心脏杂音加重，出现新的返流杂音，或心功能不全。

4）血管征象：瘀斑，脾大，颅内出血，结膜出血，镜下血尿，或Janeway斑。

5）免疫学征象：肾小球肾炎，Osler结，Roth斑，或类风湿因子阳性。

6）微生物学证据：血培养阳性，但未符合主要指际中的要求。

2．病理学指标

（1）赘生物（包括已形成的栓塞）或心内脓肿经培养或镜检发现微生物。

（2）存在赘生物或心内脓肿，并经病理检查证实伴活动性心内膜炎。

3．诊断依据

（1）具备以下①～⑤项任何之一者可诊断为感染性心内膜炎：①临床主要指标2项；②临床主要指标1 项和次要指标3 项；③心内膜受累证据和次要指标2 项；④临床次要指标5 项；⑤病理学指标1 项。

（2）有以下情况时可排除感染性心内膜炎诊断：有明确的其他诊断解释临床表现；经抗生素治疗≤4 天临床表现消除；抗生素治疗≤4 天、手术或尸体检查无感染性心内膜炎的病理证据。

（3）临床考虑感染性心内膜炎，但不具备确诊依据时仍应进行治疗，根据临床观察及进一步的检查结果确诊或排除感染性心内膜炎。

【治疗】

总的原则是积极抗感染、加强支持疗法，但在应用抗生素之前必须先做几次血培养和药物敏感试验，以期对选用抗生素及剂量提供指导。

1．抗生素 应用原则是早期、联合应用、剂量足、选用敏感的杀菌药、疗程要长。在具体应用时，对不同的病原菌感染选用不同的抗生素。抗感染药物应连用4～8 周，用至体温正常，栓塞现象消失，周围血象、血沉恢复正常，血培养阴性后逐渐停药。

2．一般治疗 包括细心护理，保证患者充足的热量供应，可少量多次输新鲜血或血浆，也可输注丙种球蛋白。

3．手术治疗 近年早期外科治疗感染性心内膜炎取得了良好效果。对心脏赘生物和污染的人造代用品清创、修复或置换损害的瓣膜，挽救了严重患者，提高了治愈率。手术指征为：①瓣膜功能不全引起的中重度心力衰竭；②赘生物阻塞瓣膜；③反复发生栓塞；④真菌感染；⑤经最佳抗生素治疗无效；⑥新发生的心脏传导阻滞。

【预后和预防】

在应用抗生素治疗前本病的死亡率几乎为100％。经合理应用抗生素治疗以来，近年病死率已下降为20％～25％。约有半数患儿可出现各种并发症如充血性心力衰竭、脑栓塞、肺栓塞、心脏瓣膜破坏、腱索断裂、动脉瘤形成等。残留严重瓣膜损伤者，需进行瓣膜修复或置换术。因此预防感染性心内膜炎发生显得极为重要。有先天性或风湿性心脏病患儿平时应注意口腔卫生，防止齿龈炎、龋齿；预防感染；若施行口腔手术、扁桃体摘除术、心导管和心脏手术时，可于术前1～2 小时及术后48 小时内肌注青霉素80 万U/d，或长效青霉素120 万UI剂。青霉素过敏者，可选用头孢菌素类或万古霉素静脉注射一次，然后改口服红霉素30 mg/（kg．d），分4 次服用，连续2 天。

第八节 小儿心律失常

儿童时期如果心脏的心肌细胞兴奋性、传导性和自律性等电生理发生改变，都可构成心律失常（cardiac arrhythmia）。儿科的心律失常可以是先天性的，也可以是获得性的：如风湿热、

心肌炎；毒物、毒素；药物或心脏手术后。心律失常的主要危险是由此产生的严重心动过缓或心动过速可导致心搏出量的降低，并可能引起晕厥或猝死。但大多数心律失常并无生命危险，如单纯房性、室性期前收缩可存在正常儿童中，准确判断心律失常是否对生命构成威胁非常重要。

一、期前收缩

期前收缩（premature beat）是由心脏异位兴奋灶发放的冲动所引起，为小儿时期最常见的心律失常。异位起搏点可位于心房、房室交界或心室组织。分别引起房性、交界性及室性期前收缩，其中以室性期前收缩为多见。

【病因】

常见于无器质性心脏病的小儿。可由疲劳、精神紧张、自主神经功能不稳定等所引起，但也可发生于心肌炎、先天性心脏病或风湿性心脏病。另外，药物如拟交感胺类、洋地黄、奎尼丁中毒及缺氧、酸碱平衡失常、电解质紊乱（低血钾）、心导管检查、心脏手术等均可引起期前收缩。健康学龄儿童中约1％～2％有期前收缩。

【临床表现】

小儿症状较成人为轻，常缺乏主诉。个别年长儿可述心悸、胸闷、不适。期前收缩次数因人而异，同一患儿在不同时间亦可有较大出入。某些患儿于运动后心率增快时期前收缩减少，但也有反而增多者。后者提示可能同时有器质性心脏病存在的可能。为了明确诊断，了解期前收缩的性质，必须作心电图检查。根据心电图有无P’波的存在、P’波的形态、P-R间期长短以及QRS波的形态来判断期前收缩属于何种类型。

【辅助检查】

1．房性期前收缩的心电图特征 ①P’波提前，可与前一心动的T波重叠；②P’-R间期在正常范围；③期前收缩后代偿间隙不完全；④如伴有变形的QRS波则为心室内差异传导所致（图13-15）。

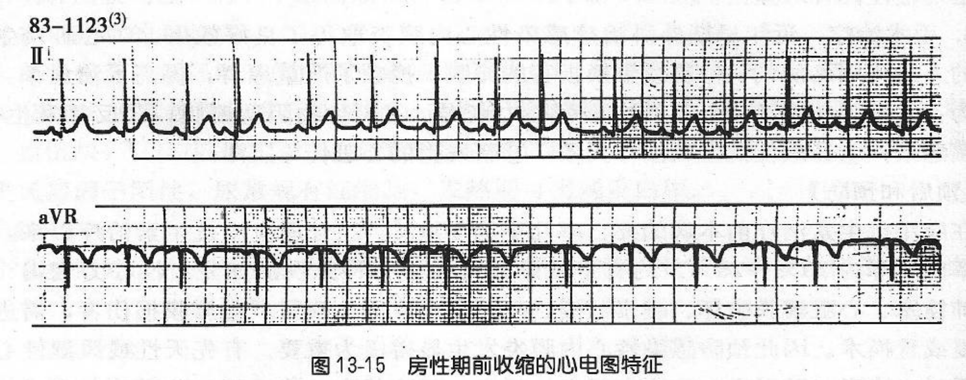

图13-15 房性期前收缩的心电图特征

2．交界性期前收缩的心电图特征 ①QRS波提前，形态、时限与正常窦性基本相同。②期前收缩所产生的QRS波前或后有逆行P’波，P’-R<0.10 s。有时P’波可与QRS波重叠，而辨认不清。③代偿间歇往往不完全（图13-16）。

3．室性期前收缩（ventricular premature beat）的心电图特征：①QRS波提前，其前无异位P波；②QRS波宽大、畸形，T波与主波方向相反；③期前收缩后多伴有完全代偿间歇（图13-17）。

【治疗】

必须针对基本病因治疗原发病。一般认为若期前收缩次数不多，无自觉症状，或期前收缩虽频发呈联律性，但形态一致，活动后减少或消失则不需无特需用药治疗。有些患者期前收缩可持续多年，但不少病人最终自行消退。对在器质性心脏病基础上出现的期前收缩或有自觉症

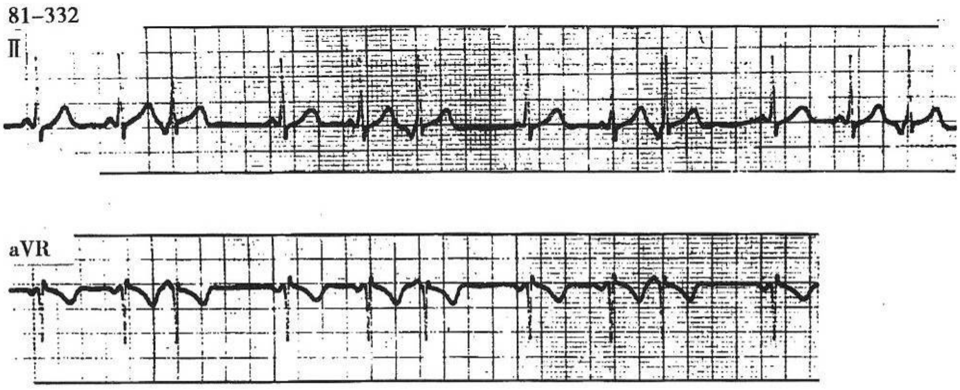

图 13-16 交界性期前收缩的心电图特征

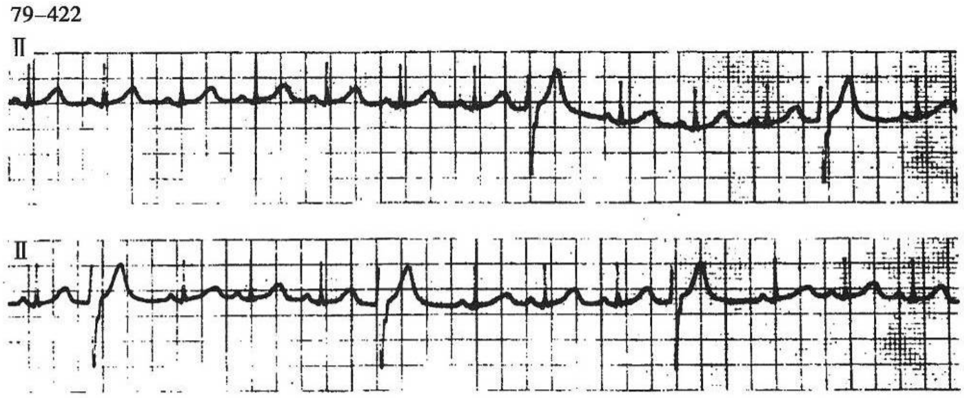

图 13-17 室性期前收缩的心电图特征

状、心电图上呈多源性者，则应予以抗心律紊乱药物治疗。根据期前收缩的不同类型选用药物。可服用普罗帕酮或普萘洛尔等β受体阻滞剂。房性期前收缩若用之无效可改用洋地黄类。室性期前收缩必要时可选用利多卡因、美西律和莫雷西嗪等。

二、阵发性室上性心动过速

阵发性室上性心动过速（paroxysmal superventricular tachycardia）是小儿最常见的异位快速心律失常。是指异位激动在希氏束以上的心动过速。主要由折返机制造成，少数为自律性增高或平行心律。本病对药物反应良好的儿科急症之一，若不及时治疗易致心力衰竭。本病可发生于任何年龄，容易反复发作，但初次发病以婴儿时期多见。

【病因】

可发生于先天性心脏病、预激综合征、心肌炎、心内膜弹力纤维增生症等疾病基础上。但多数患儿无器质性心脏疾患。感染为常见诱因，但也可因疲劳、精神紧张、过度换气、心脏手术时和手术后、心导管检查等诱发。

【临床表现】

小儿常突然烦躁不安，面色青灰，皮肤湿冷，呼吸增快，脉搏细弱常伴有干咳，有时呕吐。年长儿还可自诉心悸、心前区不适、头晕等。发作时心率突然增快在160～300 次/min之间，一次发作可持续数秒钟至数日。发作停止时心率突然减慢，恢复正常。此外，听诊时第一心音强度完全一致，发作时心率较固定而规则等为本病的特征。发作持续超过24 小时者，易引发心力衰竭。

【辅助检查】

1．X线检查 取决于原来有无心脏器质性病变和心力衰竭。透视下见心脏搏动减弱。

2．心电图检查 P波形态异常，往往较正常时小，常与前一心动的T波重叠，以致无法辨认。QRS波形态同窦性（图13-18）。发作持续时间较久者，可有暂时性ST段及T波改变。部分患儿在发作间歇期可有预激综合征表现。有时需与窦性心动过速及室性心动过速相鉴别。

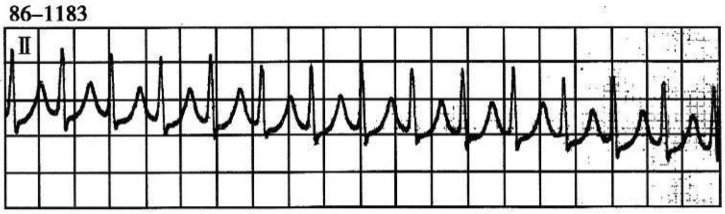

图13-18 阵发性室上性心动过速

【治疗】

1．兴奋迷走神经终止发作 对无器质性心脏病，无明显心衰者可先用此方法刺激咽部，以压舌板或手指刺激患儿咽部使之产生恶心、呕吐及使患儿深吸气后屏气。如无效时可试用压迫颈动脉窦法、潜水反射法。

2．以上方法无效或当即有效但很快复发时，可考虑下列药物治疗。

（1）洋地黄类药物：适用于病情较重，发作持续24 小时以上，有心力衰竭表现者。室性心动过速或洋地黄中毒引起的室上性心动过速禁用此药。低血钾、心肌炎、阵发性室上性心动过速伴房室传导阻滞或肾功能减退者慎用。

（2）β受体阻滞剂：可试用普萘洛尔静注。重度房室传导阻滞，伴有哮喘症及心力衰竭者禁用。

（3）维拉帕米：此药为选择性钙离子拮抗剂。抑制钙离子进入细胞内，疗效显著。不良反应为血压下降，并具有明显的负性肌力作用，加重房室传导阻滞，1 岁内婴儿禁用。

（4）普罗帕酮：为较强的钠通道阻滞剂，可有效终止室上性心动过速，具有良好的效果，而且副作用较少见。

3．电学治疗 对个别药物疗效不佳者，尤其是血流动力学不稳定者，除洋地黄中毒外可考虑用直流电同步电击转律。有条件者，可使用经食管心房调搏或经静脉右心房内调搏终止室上性心动过速。

4．射频消融术（radiofrequency ablation） 药物治疗无效，发作频繁，逆传型房室折返型可考虑使用此方法。

三、室性心动过速

室性心动过速（ventricular tachycardia）是指起源于希氏束分叉处以下的3～5 个宽大畸形QRS波组成的心动过速。

【病因】

可由心脏手术、心导管检查、严重心肌炎、先天性心脏病、感染、缺氧、电解质紊乱等原因引起。但不少病例其病因不易确定。

【临床表现】

与阵发性室上性心动过速相似，但症状比较严重。小儿烦躁不安、苍白、呼吸急促。年长儿可主诉心悸、心前区疼痛，严重病例可有晕厥、休克、充血性心力衰竭等。发作短暂者血液

动力学的改变较轻；发作持续24 小时以上者则可发生显著的血液动力学改变。体格检查发现心率增快，常在150 次/分以上，节律整齐，心音可有强弱不等现象。

【辅助检查】

心电图特征 ①心室率常在150～250 次/分之间，QRS波宽大畸形，时限增宽；②T波方向与QRS波主波相反。P波与QRS波之间无固定关系；③Q-T间期多正常，可伴有Q-T间期延长，多见于多形性室速（图13-19）；④心房率较心室率缓慢，有时可见到室性融合波或心室夺获。

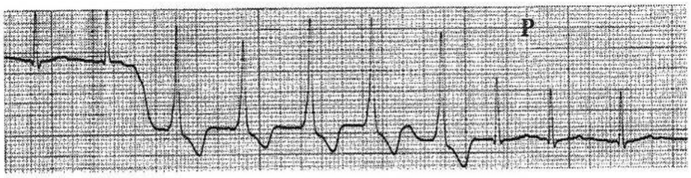

图 13-19 室性心动过速的心电图

心电图是诊断室性心动过速的重要手段，但有时与室上性心动过速伴心室差异传导的鉴别比较困难，必须综合临床病史、体格检查、心电图特点、对治疗措施的反应等仔细加以区别。

【治疗】

室性心动过速是一种严重的快速心律失常，可发展成心室颤动，致心脏性猝死。同时有心脏病存在者病死率可达50％以上，所以必须及时诊断，予以适当处理。药物可选用利多卡因0.5～1.0 mg/kg静脉滴注或缓慢推注。必要时可每隔10～30 分钟重复，总量不超过5 mg/kg。此药能控制心动过速，但作用时间很短，剂量过大能引起惊厥、传导阻滞等毒性反应。伴有血压下降或心力衰竭者首选同步直流电击复律（1～2 J/ kg），转复后再用利多卡因维持。预防复发可用口服美西律、普罗帕酮、莫雷西嗪。

对多型性室速伴Q-T间期延长者，如为先天性因素，则首选β受体阻滞剂，禁忌Ⅰa、Ⅰc及Ⅲ类药物和异丙基肾上腺素。而后天性因素所至者，可选用异丙剂肾上腺素，必要时可试用利多卡因。

四、房室传导阻滞

房室传导阻滞（AV block）是指由于房室传导系统某部位的不应期异常延长，激动心房向心室传播过程中传导延缓或部分甚至全部不能下传的现象，临床上将房室传导阻滞分为三度。

1．Ⅰ度房室传导阻滞 房室传导时间延长，心电图表现为P-R间期超过正常范围，但每个心房激动都能下传到心室（图13-20）。

2．Ⅱ度房室传导阻滞 Ⅱ度房室传导阻滞时窦房结的冲动不能全部传达心室因而造成不同程度的漏搏。通常又可分为两型。

（1）莫氏Ⅰ型：又称为文氏现象。特点是P-R间期逐步延长，最终P波后不出现QRS波，在P-R间期延长的同时，R-R间期往往逐步缩短，且脱漏的前后两个R波的距离小于最短的R-R间期的两倍（图13-21）。

（2）莫氏Ⅱ型：此型特点为P-R间期固定不变，心房搏动部分不能下传到心室，发生间歇性心室脱漏。且常伴有QRS波的增宽（图13-22）。

3．Ⅲ度房室传导阻滞 此时，房室传导组织有效不应期极度延长，使P波全部落在了有效不应期内，完全不能下传到心室，心房与心室各自独立活动，彼此无关。心室率较心房率慢（图13-23）。

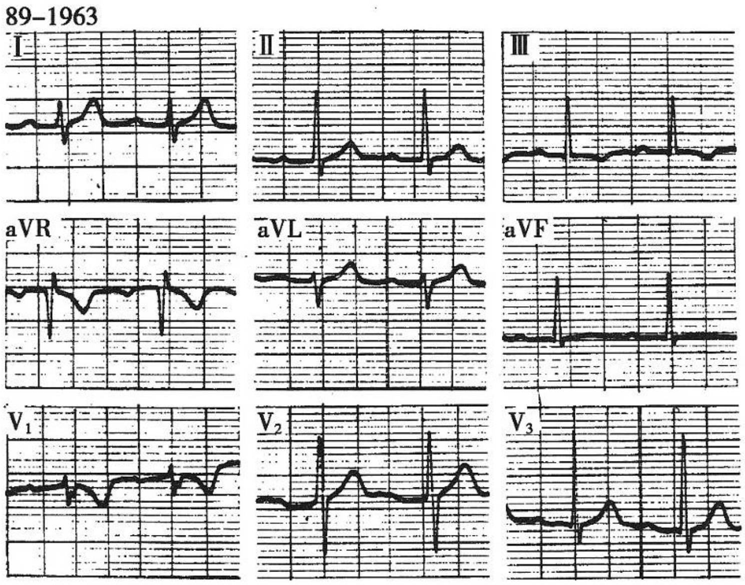

图 13-20　Ⅰ度房室传导阻滞

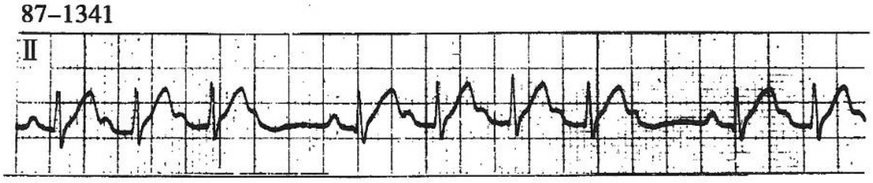

图 13-21　Ⅱ度房室传导阻滞（莫氏Ⅰ型）

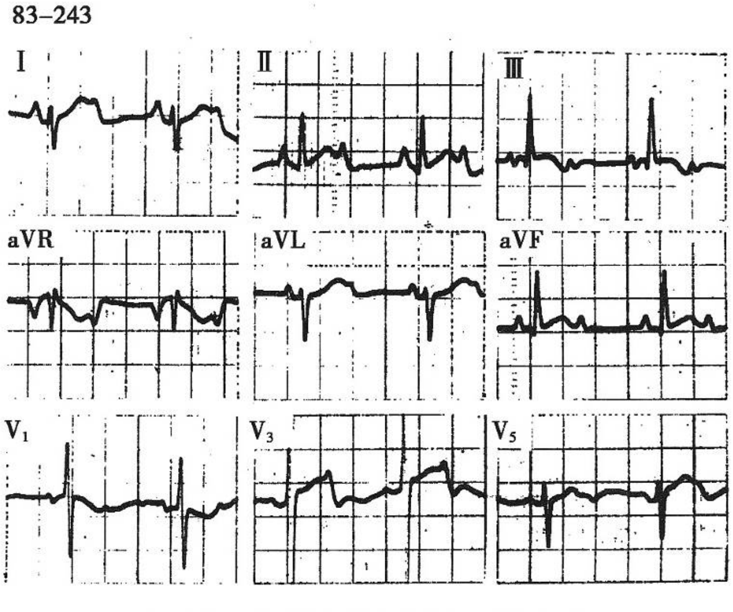

图 13-22　Ⅱ度房室传导阻滞（莫氏Ⅱ型）

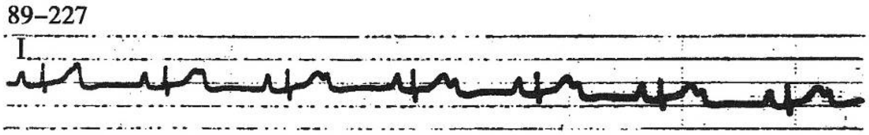

图 13-23　Ⅲ度房室传导阻滞

【病因】

Ⅰ度房室传导阻滞可见于正常健康儿童，也可由风湿性心脏炎、病毒性心肌炎、发热、肾炎、先天性心脏病引起。在应用洋地黄时也能延长**P-R**间期。Ⅱ度房室传导阻滞产生原因有风湿

性心脏病、各种原因引起的心肌炎、严重缺氧、心脏手术后及先天性心脏病（尤其是大动脉错位）等。Ⅲ度房室传导阻滞，又称完全性房室传导阻滞，小儿较少见。病因可分为先天性与获得性两种。前者中约有50％患儿的心脏并无形态学改变，部分患儿合并先天性心脏病或心内膜弹力纤维增生症等。后者以心脏手术引起的最为常见，其次为病毒性心肌炎，新生儿低血钙与酸中毒也可引起暂时性第三度房室传导阻滞。

【临床表现】

Ⅰ度房室传导阻滞本身对血液动力学并无不良影响。临床听诊，除第一心音较低钝外，并无其他特殊体征。诊断主要通过心电图检查。

Ⅱ度房室传导阻滞临床表现取决于基本心脏病变以及由传导阻滞而引起的血液动力学改变。当心室率过缓时可引起胸闷、心悸，甚至产生眩晕和晕厥。听诊时除原有心脏疾患所产生的听诊改变外，尚可发现心律不齐，脱漏搏动。莫氏Ⅰ型比Ⅱ型为常见，但Ⅱ型的预后则比较严重，容易发展为完全性房室传导阻滞，导致发生阿-斯综合征。

Ⅲ度房室传导阻滞临床上部分小儿并无主诉，重者因心搏出量减少而自觉乏力、眩晕、活动时气短。最严重的表现为阿-斯综合征发作，知觉丧失，甚至发生死亡。某些小儿则表现为心力衰竭以及对应激状态的耐受能力降低。体格检查时脉率缓慢而规则。第一心音强弱不一，有时可闻及第三心音或第四心音。绝大多数患儿心底部可听到Ⅰ～Ⅱ级喷射性杂音，为心脏每次搏出量增加引起的半月瓣相对狭窄所致。由于经过房室瓣的血量也增加，所以可闻及舒张中期杂音。X线检查发现不伴有其他心脏疾患的Ⅲ度房室传导阻滞者中，60％患儿亦有心脏增大。

【治疗】

1．Ⅰ度房室传导阻滞应着重病因治疗，基本上不需特殊治疗，预后较好。

2．Ⅱ度房室传导阻滞的治疗应针对原发疾病。当心室率过缓、心脏搏出量减少时可用阿托品、异丙肾上腺素治疗。预后与心脏的基本病变有关。

3．Ⅲ度房室传导阻滞有心功能不全症状或阿-斯综合征表现者需积极治疗。纠正缺氧与酸中毒可改善心脏传导功能。由心肌炎或手术暂时性损伤引起者，肾上腺皮质激素可消除局部水肿。可口服阿托晶、麻黄碱，或异丙基肾上腺素舌下含服，重症者应用阿托品皮下或静脉注射，或异丙肾上腺素1 mg溶于5％～10％葡萄糖溶液250 ml中，持续静脉滴注，速度为0.05～2 μg/（kg．min），然后根据心率调整速度。

安装起搏器的指征为：反复发生阿-斯综合征，药物治疗无效或伴心力衰竭者。一般先安装临时起搏器，经临床治疗可望恢复正常，若观察4 周左右仍未恢复者，考虑安置永久起搏器。

第九节 心力衰竭

充血性心力衰竭（congestive heart failure，以下简称心衰）是指心脏工作能力（心肌收缩或缩张功能）下降，即心排血量绝对或相对不足，不能满足全身组织代谢的需要的病理状态。心力衰竭是儿童时期危重症之一。

【病因】

小儿时期心衰以1 岁以内发病率最高，其中尤以先天性心脏病引起者最多见。先天性心脏病中，流出道狭窄即可导致后负荷即压力负荷增加，某些流入道狭窄引起相同作用。而左向右分流和瓣膜反流则导致前负荷（容量负荷）的增加。心力衰竭也可继发于病毒性心肌炎、川崎病、心肌病、心内膜弹力纤维增生症等。儿童时期以风湿性心脏病和急性肾炎所致的心衰最为多见。另外，贫血、营养不良、电解质紊乱、严重感染、心律紊乱和心脏负荷过重等都是儿童

心衰发生的诱因。

【病理生理】

心脏功能从正常发展到心力衰竭，经过一段称为代偿过程，心脏出现心肌肥厚，心脏扩大和心率增快。由于心肌纤维伸长和增厚使收缩力增强，排血量增多。如基本病因持续存在，则代偿性改变相应发展，心肌能量消耗增多，冠状动脉血供相对不足，心肌收缩速度减慢和收缩力减弱。心率增快超过一定限度时，舒张期缩短，心排血量反而减少。心排血量通过代偿不能满足身体代谢需要时，即出现心力衰竭。

心力衰竭时心排血量一般均减少到低于正常休息时的心排血量，故称为低输血量心力衰竭。但由甲状腺机能亢进、组织缺氧、严重贫血、动静脉瘘等引进的心力衰竭，体循环量增多，静脉回流量和心排血量高于正常；心力衰竭发生后，心排血量减少，但仍可超过正常休息时的心排血量，故称为高输出血量心力衰竭。

心力衰竭时由于心室收缩期排血量减少，心室内残余血量增多。舒张期充盈压力增高，可同时出现组织缺氧以及心房和静脉淤血。组织缺氧通过交感神经活性增加，引起皮肤内脏血管收缩，血液重新分布，以保证重要器官的血供。肾血管收缩后肾血流量减少，肾小球滤过率降低，肾素分泌增加，继而醛固酮分泌增多，使近端和远端肾曲小管对钠的再吸收增多，体内水钠潴留，引起血容量增多，组织间隙等处体液淤积。近年来对神经内分泌在心衰发生发展中的调节作用有了新的认识。心衰时心排出量减少，可通过交感神经激活肾素-血管紧张素-醛固酮系统，从而引起β受体-腺苷酸环化酶系统调节紊乱。使外周血管收缩，水钠潴留。以致加剧心室重塑，促进心衰恶化。

心室负荷过重可分为容量负荷过重和压力负荷过重。前者在轻度或中度时心肌代偿能力较后者好些，例如房间隔缺损虽然有时分流量很大，但属舒张期负荷过重，在儿童期很少发生心力衰竭，肺动脉瓣狭窄属收缩期负荷过重，心衰出现更早些；主动脉瓣缩窄伴动脉导管未闭则兼有收缩和舒张期负荷过重，故在新生儿时期可致死。

【临床表现】

年长儿心衰的症状与成人相似，主要表现为乏力、活动后气急、食欲减低、腹痛和咳嗽。安静时心率增快，呼吸浅表、增速，颈静脉怒张，肝增大、有压痛，肝颈反流试验阳性。病情较重者尚有端坐呼吸、肺底部可听到湿啰音，并出现浮肿，尿量明显减少。心脏听诊除原有疾病产生的心脏杂音和异常心音外，常可听到心尖区第一心音减低和奔马律。

婴幼儿心衰的临床表现有一定特点。常见症状为呼吸快速、表浅、频率可达50～100 次/分，喂养困难，体重增长缓慢，烦躁多汗，哭声低弱，肺部可闻及干啰音或哮鸣音。浮肿首先见于颜面、眼睑等部位，严重时鼻唇三角区呈现青紫。

【诊断】

1．临床诊断依据 ①安静时心率增快，婴儿>180 次份，幼儿>160 次/分，不能用发热或缺氧解释者；②呼吸困难，青紫突然加重，安静时呼吸达60 次/分以上；③肝大达肋下3cm 以上，或在密切观察下短时间内较前增大，而不能以横膈下移等原因解释者；④心音明显低钝，或出现奔马律；⑤突然烦躁不安，面色苍白或发灰，而不能用原有疾病解释；⑥尿少、下肢浮肿，以除外营养不良，肾炎、维生素B_1缺乏等原因所造成者。

2．其他检查 上述前四项为临床诊断的主要依据。尚可结合其他几项以及下列1～2 项检查进行综合分析。

（1）胸部X线检查：心影多呈普遍性扩大，搏动减弱，肺纹理增多，肺门或肺门附近阴影增加，肺部淤血。

（2）心电图检查：不能表明有无心衰，但有助于病因诊断及指导洋地黄的应用；

（3）超声心动图检查：可见心室和心房腔扩大，M型超声心动图显示心室收缩时间期延长，喷血分数降低。心脏舒张功能不全时，二维超声心动图对诊断和引起心衰的病因判断有帮助。

【治疗】

应重视病因治疗，先天性心脏病患者的内科治疗往往是术前的准备，而且手术后亦需继续治疗一个时期；心肌病患者，内科治疗可使病人症状获得暂时的缓解；如心衰由甲状腺功能亢进、重度贫血或维生素B_1缺乏、病毒性或中毒性心肌炎等引起者需及时治疗原发疾病。心力衰竭的内科治疗有下列几方面：

1．一般治疗 充分的休息和睡眠可减轻心脏负担，平卧或取半卧位，尽力避免患儿烦躁、哭闹，必要时可适当应用镇静剂，苯巴比妥、吗啡（0.05 mg/kg）皮下或肌肉注射常能取得满意效果，但需警惕抑制呼吸。供氧往往是需要的。心力衰竭时，患者易发生酸中毒、低血糖和低血钙，新生儿时期更是如此。因此一旦发生以上情况，应予及时纠正。应给予容易消化及富有营养的食品，一般饮食中钠盐应减少，很少需要严格的极度低钠饮食。

2．洋地黄类药物 迄今为止洋地黄仍是儿科临床上广泛使用的强心药物之一。洋地黄作用于心肌细胞上的Na^+-K^+-ATP酶，抑制其活性，使细胞内Na^+浓度升高，通过Na^+-Ca^{2+}交换使细胞内Ca^{2+}升高，从而加强心肌收缩力，使心室排空完全，心室舒张终末期压力明显下降，从而静脉淤血症状减轻。近年，更认识到它对神经内分泌和压力感受器的影响。洋地黄能直接抑制过度的神经内分泌活性（主要抑制交感神经活性作用）。除正性肌力作用外，洋地黄还具有负性传导、负性心率等作用。洋地黄对左心瓣膜反流、心内膜弹力纤维增生症、扩张型心肌病和某些先心病等所致的充血性心力衰竭均有效。尤其是合并心率增快、房扑、房颤者更有效。而对贫血、心肌炎引起者疗效较差。

小儿时期常用的洋地黄制剂为地高辛（digoxin），可口服和静脉注射，作用时间较快，排泄亦较迅速，因此剂量容易调节，药物中毒时处理也比较容易。地高辛酏剂口服吸收率更高。早产儿对洋地黄比足月儿敏感，后者又比婴儿敏感。婴儿的有效浓度为2～4 ng/ml，大年龄儿童为1～2 ng/ml。由于洋地黄的剂量和疗效的关系受到多种因素的影响，所以洋地黄的剂量要个体化。常用剂量和用法见表13-1。

表13-1 洋地黄药物的临床应用

洋地黄制剂	给药法	洋地黄化总量（mg/kg）	每日平均维持量	效力开始时间	效力最大时间	中毒作用消失时间	效力完全消失时间
地高辛	口服	<2 岁 0.05~0.06<br>>2 岁 0.03~0.05<br>（总量不超过1.5mg）	1/5 洋地黄化量，分2次	2 小时	4~8 小时	1~2 天	4~7 天
	静脉	口服量的1/2~2/3		10 分钟	1~2 小时		
毛花苷丙（西地兰）	静脉	<2 岁 0.03~0.04<br>>2 岁 0.02~0.03		15~30 分钟	1~2 小时	1 天	2~4 天

（1）洋地黄化法：如病情较重或不能口服者，可选用毛花甙丙或地高辛静注，首次给洋地黄化总量的1/2，余量分2 次，每隔4～6 小时给予，多数患儿可于8～12 小时内达到洋地黄化；能口服的患者开始给予口服地高辛，首次给洋地黄化总量的1/3 或1/2，余量分2 次，每隔6～8 小时给予。

（2）维持量：洋地黄化后12 小时可开始给予维持量。维持量的疗程视病情而定：急性肾炎合并心衰者往往不需用维持量或仅需短期应用；短期难以去除病因者如心内膜弹力纤维增生症或风湿性心瓣膜病等，则应注意随患儿体重增长及时调整剂量，以维持小儿血清地高辛的有

效浓度。

（3）使用洋地黄注意事项：用药前应了解患儿在2～3 周内的洋地黄使用情况，以防药物过量引起中毒。各种病因引起的心肌炎患儿对洋地黄耐受性差，一般按常规剂量减去1/3，且饱和时间不宜过快。未成熟儿和<2 周的新生儿因肝肾功能尚不完善，易引起中毒，洋地黄化剂量应偏小，可按婴儿剂量减少1/2～1/3。钙剂对洋地黄有协同作用，故用洋地黄类药物时应避免用钙剂。此外，低血钾可促使洋地黄中毒，应予注意。

（4）洋地黄毒性反应：心力衰竭愈重、心功能愈差者，其治疗量和中毒量愈接近，故易发生中毒。肝肾功能障碍、电解质紊乱、低钾、高钙、心肌炎和大剂量利尿之后的患儿均易发生洋地黄中毒。小儿洋地黄中毒最常见的表现为心律失常，如房室传导阻滞、室性期前收缩和阵发性心动过速等；其次为恶心、呕吐等胃肠道症状；神经系统症状，如嗜睡、头昏、色视等较少见。

洋地黄中毒时应立即停用洋地黄和利尿剂，同时补充钾盐。小剂量钾盐能控制洋地黄引起的室性期前收缩和阵发性心动过速。轻者每日用氯化钾0.075～0.1 g/kg，分次口服；严重者每小时0.03～0.04 g/kg 静脉滴注，总量不超过0.15 g/kg，滴注时用10%葡萄糖稀释成0.3%浓度。肾功能不全和合并房室传导阻滞时忌用静脉给钾。钾盐治疗无效或并发其他心律失常时的治疗参见心律失常节。

3．利尿剂 钠、水潴留为心力衰竭的一个重要病理生理改变，故合理应用利尿剂为治疗心力衰竭的一项重要措施。当使用洋地黄类药物而心衰仍未完全控制，或伴有显著水肿者，宜加用利尿剂（表13-2）。对急性心衰或肺水肿者可选用快速强效利尿剂如呋塞米或依他尼酸，其作用快而强，可排除较多的Na^+，而K^+的损失相对较少。慢性心衰一般联合使用噻嗪类与保钾利尿剂，并采用间歇疗法维持治疗，防止电解质紊乱。

表13-2 各种利尿剂的临床应用

药 名	剂量和方法	作用时间	并发症及注意事项	作用强弱
碱性利尿剂：				
依他尼酸<br>25 mg/支、20 mg/片<br>呋塞米<br>25 mg/支、20 mg/片	静注：每次l mg/kg，稀释成2 mg/ml，5～10 分钟缓推，必要时8～12 小时可重复。口服：2～3 mg/（kg. d），分2～3 次	静注后15 分钟，口服30 分钟开始起作用。1～2 小时为利尿高峰	可引起脱水，低血钾，低血氯，碱中毒。肾衰竭者用利依他尼酸有耳聋危险，婴儿慎用	++++
噻嗪类：				
氢氯噻嗪 25 mg/片	口服：1～5 mg/（kg • d），分2～3 次，维持治疗服4 天停3 天，<6 月者，0.5～0.75 mg/（kg • d），分2～3 次	1 小时开始，4～6 小时达高峰，持续12 小时	常用可致电解质紊乱（低血钾，低血氯）及心律紊乱，粒细胞减少	+++
保钾利尿剂：				
螺内脂 20 mg/粒	口服：1～2 mg/（kg • d），分2～3 次	8～12 小时开始，3～4 小时达高峰，持续2～3 天	有保血钾、保血氯作用，和噻嗪类合用，可增强疗效	+
氨苯蝶啶 50 mg/片	口服：2～4 mg/（kg • d），分2～3 次	1 小时开始，4～6 小时达高峰，持续12 小时		+

4．血管扩张剂 近年来应用血管扩张剂治疗顽固性心衰取得一定疗效。小动脉的扩张使心脏后负荷降低，从而可能增加心搏出量，同时静脉的扩张使前负荷降低，心室充盈压下降，肺充血的症状亦可能得到缓解，对左室舒张压增高的患者更为适用。

（1）血管紧张素转换酶抑制剂：通过血管紧张素转换酶的抑制，减少循环中血管紧张素Ⅱ的浓度发挥效应。通过国际大规模多中心的随机对照的临床试验证明该药能有效缓解心衰的临床症状，改善左室的收缩功能，防止心肌的重构，逆转心室肥厚，降低心衰患者的死亡率。儿科临床的中、长期疗效还有待观察。卡托普利（巯甲丙脯酸）初始剂量为每日0.5 mg/kg，以后根据病情逐渐加量，每周递增1 次，每次增加0.3 mg/（kg．d），最大耐受量为5 mg/（kg．d），分3～4 次口服。依那普利（苯脂丙脯酸）剂量为每日0.05～0.1 mg/kg，一次口服。

（2）硝普钠：硝普钠能释放NO，使cGMP升高而松弛血管的平滑肌，扩张小动脉、静脉的血管平滑肌，作用强，生效快和持续时间短。硝普钠对急性心衰（尤其是急性左心衰、肺水肿）伴周围血管阻力明显增加者效果显著。在治疗体外循环心脏手术后的低心排综合征时联合多巴胺效果更佳。应在动脉压力监护下进行。剂量为每分钟0.2 μg/kg，以5％葡萄糖稀释后点滴，以后每隔5 分钟，可每分钟增加0.1～0.2 μg/kg，直到获得疗效或血压有所降低。最大剂量不超过每分钟3～5 μg/kg。

（3）酚妥拉明（苄胺唑啉）：α受体阻滞剂，以扩张小动脉为主，兼有扩张静脉的作用。剂量为每分钟2～6 μg/kg，以5％葡萄糖稀释后静滴。

5．其他药物治疗 心衰伴有血压下降时可应用多巴胺，每分钟5～10 μg/kg。必要时剂量可适量增加，一般不超过每分钟30 μg/kg。如血压显著下降，以给予肾上腺素每分钟0.1～1.0 μg/kg持续静脉滴注，这有助于增加心搏出量、提高血压而心率不一定明显增快。

（桂永浩）

第十四章 泌尿系统疾病

第一节 儿童泌尿系统解剖生理特点

一、解剖特点

（一）肾脏

儿童年龄愈小，肾脏相对愈重，新生儿两肾重量约为体重的1/125，而成人两肾重量约为体重的1/220。婴儿肾脏位置较低，其下极可低至髂嵴以下第4腰椎水平，2 岁以后始达髂嵴以上。右肾位置稍低于左肾。2 岁以内健康儿童腹部触诊时容易扪及肾脏。婴儿肾脏表面呈分叶状，至2～4 岁时，分叶完全消失。

（二）输尿管

婴幼儿输尿管长而弯曲，管壁肌肉和弹力纤维发育不良，容易受压及扭曲而导致梗阻，易发生尿潴留而诱发感染。

（三）膀胱

婴儿膀胱位置比年长儿高，尿液充盈时，膀胱顶部常在耻骨联合之上，顶入腹腔而容易触到，随年龄增长逐渐下降至盆腔内。

（四）尿道

新生女婴尿道长仅1 cm（性成熟期3～5 cm），且外口暴露而又接近肛门，易受细菌污染。男婴尿道虽较长，但常有包茎，尿垢积聚时也易引起上行性细菌感染。

二、生理特点

肾脏有许多重要功能：①排泄体内代谢终末产物如尿素、有机酸等；②调节机体水、电解质、酸碱平衡，维持内环境相对稳定；③内分泌功能，产生激素和生物活性物质如促红细胞生成素、肾素、前列腺素等。肾脏完成其生理活动，主要通过肾小球滤过和肾小管重吸收、分泌及排泄。儿童肾脏虽具备大部分成人肾的功能，但其发育是由未成熟逐渐趋向成熟。在胎龄36周时肾单位数量（每肾85 万～100 万）已达成人水平，出生后上述功能已基本具备，但调节能力较弱，贮备能力差，一般至1～2 岁时接近成人水平。

（一）胎儿肾功能

胎儿于12 周末，由于近曲小管刷状缘的分化及小管上皮细胞开始运转，已能形成尿液。但此时主要通过胎盘来完成机体的排泄和调节内环境稳定，故无肾的胎儿仍可存活和发育。

（二）肾小球滤过率（GFR） 。

新生儿出生时肾小球滤过率比较低，为成人的1/4，早产儿更低，3～6 个月为成人1/2，6～12个月时为成人的3/4，2 岁时达成人水平，故不能有效地排出过多的水分和溶质。血肌酐作为反映肾小球滤过功能的常用指标，由于身高和肌肉发育等影响，不同年龄有不同的正常参考值（表14-1、表14-2）。

表14-1 足月和极低出生体重新生儿最初几周血清肌酐平均值

体重（g）	血清肌酐（μmol/L）生后时间（天）			
	1～2	8～9	15～16	22～23
1001～1500	95	64	49	35
1501～2000	90	58	50	30
2001～2500	83	47	38	30
足月	66	40	30	27

摘自 Avner ED, Harmon WE, Niaudet P. Pediatric Nephrology 5th Edition. Lippincott Williams & Wilkins, 2003, 409

表14-2 儿童血清肌酐参考值

年龄（岁）	血清肌酐 μmol/L	mg/dl
<2	35～40	0.4～0.5
2～8	40～60	0.5～0.7
9～18	50～80	0.6～0.9

摘自Garcia-Nieto V, Santos F. Pruebas funcionals renales in: Garcia-Nieto V, Santos F, eds, Nefrologia pediatrica. Madrid: Aula Media, 2000; and Garcia-Nieto V, Santos F, eds. Grupo aula medica. Madrid: Aula Medica, 2000; 15-26.

（三）肾小管重吸收及排泄功能

新生儿期在葡萄糖肾阈、排钠能力、醛固酮分泌等方面都有其特点，详见新生儿章节。

（四）浓缩和稀释功能

新生儿及幼婴由于髓袢短，尿素形成量少（婴儿蛋白合成代谢旺盛）以及抗利尿激素分泌不足，使浓缩尿液功能不足，在应激状态下保留水分的能力低于年长儿和成人。婴儿每由尿中排出1 mmol 溶质时需水分1.4～2.4 mL，成人仅需0.7 ml。脱水时幼婴尿渗透压最高不超过700 mmol/L，而成人可达1400 mmol/L，故入量不足时易发生脱水甚至诱发急性肾功能不全。新生儿及幼婴尿稀释功能接近成人，可将尿稀释至40 mmol/L，但因GFR较低，大量水负荷或输液过快时易出现水肿。

（五）酸碱平衡

新生儿及婴幼儿易发生酸中毒，主要原因有：①肾保留HCO_3^-的能力差，碳酸氢盐的肾阈低，仅为19～22 mmol/L；②泌NH_3和泌H^+的能力低；③尿中排磷酸盐量少，故排出可滴定酸的能力受限。

（六）肾脏的内分泌功能

新生儿的肾脏已具有内分泌功能，其血浆肾素、血管紧张素和醛固酮均等于或高于成人，生后数周内逐渐降低。新生儿肾血流量低，因而前列腺素合成速率较低。由于胎儿血氧分压较低，故胚肾合成促红细胞生成素较多，生后随着血氧分压的增高，促红细胞生成素合成减少。婴儿血清$1,25(OH)_2D_3$水平高于儿童期。

（七）小儿排尿及尿液特点

1．排尿次数 93％新生儿在生后24 小时内，99％在48 小时内排尿。生后头几天内，因摄

入量少，每日排尿仅4～5 次；1 周后因新陈代谢旺盛，进水量较多而膀胱容量小，排尿突增至每日20～25 次；1 岁时每日排尿15～16 次，至学龄前和学龄期每日6～7 次。

2. 排尿控制 正常排尿机制在婴儿期由脊髓反射完成，以后建立脑干-大脑皮层控制，至3 岁已能控制排尿。在1.5～3 岁之间，儿童主要通过控制尿道外括约肌和会阴肌控制排尿，若3 岁后仍保持这种排尿机制，不能控制膀胱逼尿肌收缩，则出现不稳定膀胱，表现为白天尿频尿急，偶然尿失禁和夜间遗尿。

3. 每日尿量 儿童尿量个体差异较大，新生儿生后48 小时正常尿量一般每小时为1～3 ml/kg，2 天内平均尿量为30～60 ml/d，3～10 天为100～300 ml/d，～2 个月为250～400 ml/d，～1 岁为400～500 ml/d，～3 岁为500～600 ml/d，～5 岁为600～700 ml/d，～8 岁为600～1000 ml/d，～14 岁为800～1400 ml/d，>14 岁为1000～1600 ml/d。若新生儿尿量每小时<1.0 ml/kg为少尿，每小时<0.5 ml/kg为无尿。学龄儿童每日排尿量少于400 ml，学龄前儿童少于300 ml，婴幼儿少于200 ml时为少尿；每日尿量少于50 ml为无尿。

4. 尿的性质

（1）尿色：生后头2～3 天尿色深，稍混浊，放置后有红褐色沉淀，此为尿酸盐结晶。数日后尿色变淡。正常婴幼儿尿液淡黄透明，但在寒冷季节放置后可有盐类结晶析出而变混，尿酸盐加热后，磷酸盐加酸后可溶解，尿液变清，可与脓尿或乳糜尿鉴别。

（2）酸碱度：生后头几天因尿内含尿酸盐多而呈强酸性，以后接近中性或弱酸性，pH多为5～7。

（3）尿渗透压和尿比重：新生儿尿渗透压平均为240 mmol/L，尿比重为1.006～1.008，随年龄增长逐渐增高；婴儿尿渗透压为50～600 mmol/L，1 岁后接近成人水平；儿童通常为500～800 mmol/L，尿比重范围为1.003～1.030，通常为1.011～1.025。

（4）尿蛋白：正常小儿尿中仅含微量蛋白，通常≤100 mg/（m^2·24h），定性为阴性，一次随意尿的尿蛋白（mg/dL）/尿肌酐（mg/dL）≤0.2。若尿蛋白含量>150 mg/d 或>4 mg/（m^2·h），或>100 mg/L，定性检查阳性为异常。尿蛋白主要来自血浆蛋白，2/3 为白蛋白，1/3为Tamm-Horsfall蛋白和球蛋白。

（5）尿细胞和管型：正常新鲜尿液离心后沉渣显微镜检查，红细胞<3 个/HP，白细胞<5 个/HP，偶见透明管型。12 小时尿细胞计数（Addis count）：红细胞<50 万、白细胞<100 万、管型<5000 个为正常。

第二节 儿童肾小球疾病的临床分类

中华医学会儿科分会肾脏病学组于2000 年11 月珠海会议对1981 年修订的关于小儿肾小球疾病临床分类再次修订如下：

（一）原发性肾小球疾病（primary glomerular diseases）

1. 肾小球肾炎（glomerulonephritis）

（1）急性肾小球肾炎（acute glomerulonephritis，AGN）：急性起病，多有前驱感染，以血尿为主，伴不同程度蛋白尿，可有水肿、高血压或肾功能不全，病程多在1 年内。可分为：①急性链球菌感染后肾小球肾炎（acute poststreptococcal glomerulonephritis，APSGN）：有链球菌感染的血清学证据，起病6～8 周内有血补体低下；②非链球菌感染后肾小球肾炎（non-poststreptococcal glomerulonephritis）。

（2）急进性肾小球肾炎（rapidly progressive glomerulonephritis，RPGN）：起病急，有尿改

变（血尿、蛋白尿、管型尿）、高血压、水肿，并常有持续性少尿或无尿，进行性肾功能减退。若缺乏积极有效的治疗措施，预后严重。

（3）迁延性肾小球肾炎（persistent glomerulonephritis）：有明确急性肾炎病史，血尿和（或）蛋白尿迁延达1 年以上；或没有明确急性肾炎病史，但血尿和蛋白尿超过半年，不伴肾功能不全或高血压。

（4）慢性肾小球肾炎（chronic glomerulonephritis）：病程超过1 年，或隐匿起病，有不同程度的肾功能不全或肾性高血压的肾小球肾炎。

2．肾病综合征（nephrotic syndrome，NS）诊断标准：大量蛋白尿（尿蛋白+++～++++，1 周内3 次，24 小时尿蛋白定量≥50 mg/kg）；血浆白蛋白低于30 g/L；血浆胆固醇高于5.7 mmol/L；不同程度的水肿。以上四项中以大量蛋白尿和低白蛋白血症为必要条件。

（1）依临床表现分为两型：单纯型肾病（simple type NS）和肾炎型肾病（nephritic type NS）。

凡具有以下四项之一或多项者属于肾炎型肾病：①2 周内分别3 次以上离心尿检查RBC≥10 个/HPF，并证实为肾小球源性血尿者；②反复或持续高血压，学龄儿童≥130/90 mmHg，学龄前儿童≥120/80 mmHg。并除外糖皮质激素等原因所致；③肾功能不全，并排除由于血容量不足等所致；④持续低补体血症。

（2）按糖皮质激素反应分为：①激素敏感型肾病（steroid-responsive NS）：以泼尼松足量治疗≤8 周尿蛋白转阴者；②激素耐药型肾病（steroid-resistant NS）：以泼尼松足量治疗8 周尿蛋白仍阳性者；③激素依赖型肾病（steroid-dependent NS）：对激素敏感，但减量或停药1 个月内复发，重复2 次以上者；④肾病复发与频复发（relapse and frequently relapse）：复发（包括反复）。是指尿蛋白由阴转阳>2 周；频复发是指肾病病程中半年内复发≥2 次；或1 年内复发≥3 次。

3．孤立性血尿或蛋白尿（isolated hematuria or proteinuria）指仅有血尿或蛋白尿，而无其他临床症状，化验改变及肾功能改变者。

（1）孤立性血尿（isolated hematuria）：指肾小球源性血尿，分为持续性和再发性；

（2）孤立性蛋白尿（isolated proteinuria）：分为体位性和非体位性。

（二）继发性肾小球疾病（secondary glomerular diseases）

1．紫癜性肾炎（purpura nephritis）

2．狼疮性肾炎（lupus nephritis）

3．乙肝病毒相关性肾炎（HBV-associated glomerulonephritis）

4．其他 毒物、药物中毒或其他全身性疾患所致的肾炎及相关性肾炎。

（三）遗传性肾小球疾病（hereditary glomerular diseases）

1．先天性肾病综合征（congenital nephritic syndrome）指生后3 个月内发病，临床表现符合肾病综合征，可除外继发所致者（如TORCH或先天性梅毒等），分为：

（1）遗传性：芬兰型，法国型（弥漫性系膜硬化）。

（2）原发性：指生后早期发生的原发性肾病综合征。

2．遗传性进行性肾炎（Alport综合征）。

3．家族性再发性血尿（familial recurrent hematuria）。

4．其他，如甲-膑综合征。

第三节 急性肾小球肾炎

急性肾小球肾炎（简称急性肾炎），是指一组病因不一，临床表现为急性起病，多有前驱

感染，以血尿为主，伴不同程度蛋白尿，可有水肿、高血压，或肾功能不全等特点的肾小球疾患。急性肾炎可分为急性链球菌感染后肾小球肾炎和非链球菌感染后肾小球肾炎，本节急性肾炎主要是指前者。

1982 年全国105 所医院的调查结果为急性肾炎患儿占同期泌尿系统疾病的53.7%。本病多见于儿童和青少年，以5～14 岁多见，小于2 岁少见，男女之比为2：1。

【病因】

尽管本病有多种病因，但绝大多数的病例属A组β溶血性链球菌急性感染后引起的免疫复合性肾小球肾炎。溶血性链球菌感染后，肾炎的发生率一般在0%～20%。1982 年全国105 所医院儿科泌尿系统疾病住院病人调查，急性肾炎患儿抗“O”升高者占61.2%。我国各地区均以上呼吸道感染或扁桃体炎最常见，占51%，脓皮病或皮肤感染次之占25.8%。

除A组β溶血性链球菌之外，其他细菌如绿色链球菌、肺炎球菌、金黄色葡萄球菌、伤寒杆菌、流感杆菌等，病毒如柯萨基病毒B4 型、ECHO 病毒9 型、麻疹病毒、腮腺炎病毒、乙型肝炎病毒、巨细胞病毒、EB病毒、流感病毒等，还有疟原虫、肺炎支原体、白色念珠菌、丝虫、钩虫、血吸虫、弓形虫、梅毒螺旋体、钩端螺旋体等也可导致急性肾炎。

【发病机制】

目前认为急性肾炎主要与A组溶血性链球菌中的致肾炎菌株感染有关，所有致肾炎菌株均有共同的致肾炎抗原性，包括菌壁上的M蛋白内链球菌素（endostretocin）和“肾炎菌株协同蛋白”（nephritis strain associated protein，NSAP）。主要发病机制为抗原抗体免疫复合物引起肾小球毛细血管炎症病变，包括循环免疫复合物和原位免疫复合物形成学说。此外，某些链球菌株可通过神经氨酸苷酶的作用或其产物如某些菌株产生的唾液酸酶，与机体的免疫球蛋白（1gG）结合，改变其免疫原性，产生自身抗体和免疫复合物而致病。另有人认为链球菌抗原与肾小球基膜糖蛋白间具有交叉抗原性，可使少数病例呈现抗肾抗体型肾炎。急性链球菌感染后肾炎的发病机制见图14-1。

【病理】

在疾病早期，肾脏病变典型，呈毛细血管内增生性肾小球肾炎改变。光镜下肾小球表现为程度不等的弥漫性增生性炎症及渗出性病变。肾小球增大、肿胀，内皮细胞和系膜细胞增生，炎性细胞浸润。毛细血管腔狭窄甚或闭锁、塌陷。肾小球囊内可见红细胞、球囊上皮细胞增生。部分患者中可见到新月体。肾小管病变较轻，呈上皮细胞变性，间质水肿及炎症细胞浸润。

电镜检查可见内皮细胞胞浆肿胀呈连拱状改变，使内皮孔消失。电子致密物在上皮细胞下沉积，呈散在的圆顶状驼峰样分布。基膜有局部裂隙或中断。

免疫荧光检查在急性期可见弥漫一致性纤细或粗颗粒状的IgG、C3 和备解素沉积，主要分布于肾小球毛细血管袢和系膜区，也可见到IgM和IgA沉积。系膜区或肾小球囊腔内可见纤维蛋白原和纤维蛋白沉积。

【临床表现】

急性肾炎临床表现轻重悬殊，轻者全无临床症状仅发现镜下血尿，重者可呈急进性过程，短期内出现肾功能不全。

1．前驱感染 90%病例有链球菌的前驱感染，以呼吸道及皮肤感染为主。在前驱感染后经1～3 周无症状的间歇期而急性起病。咽炎为诱因者病前6～12 天（平均10 天）多有发热、颈淋巴结大及咽部渗出。皮肤感染见于病前14～28 天（平均20 天）。

2．典型表现 急性期常有全身不适、乏力、食欲不振、发热、头痛、头晕、咳嗽、气急、恶心、呕吐、腹痛及鼻出血等。

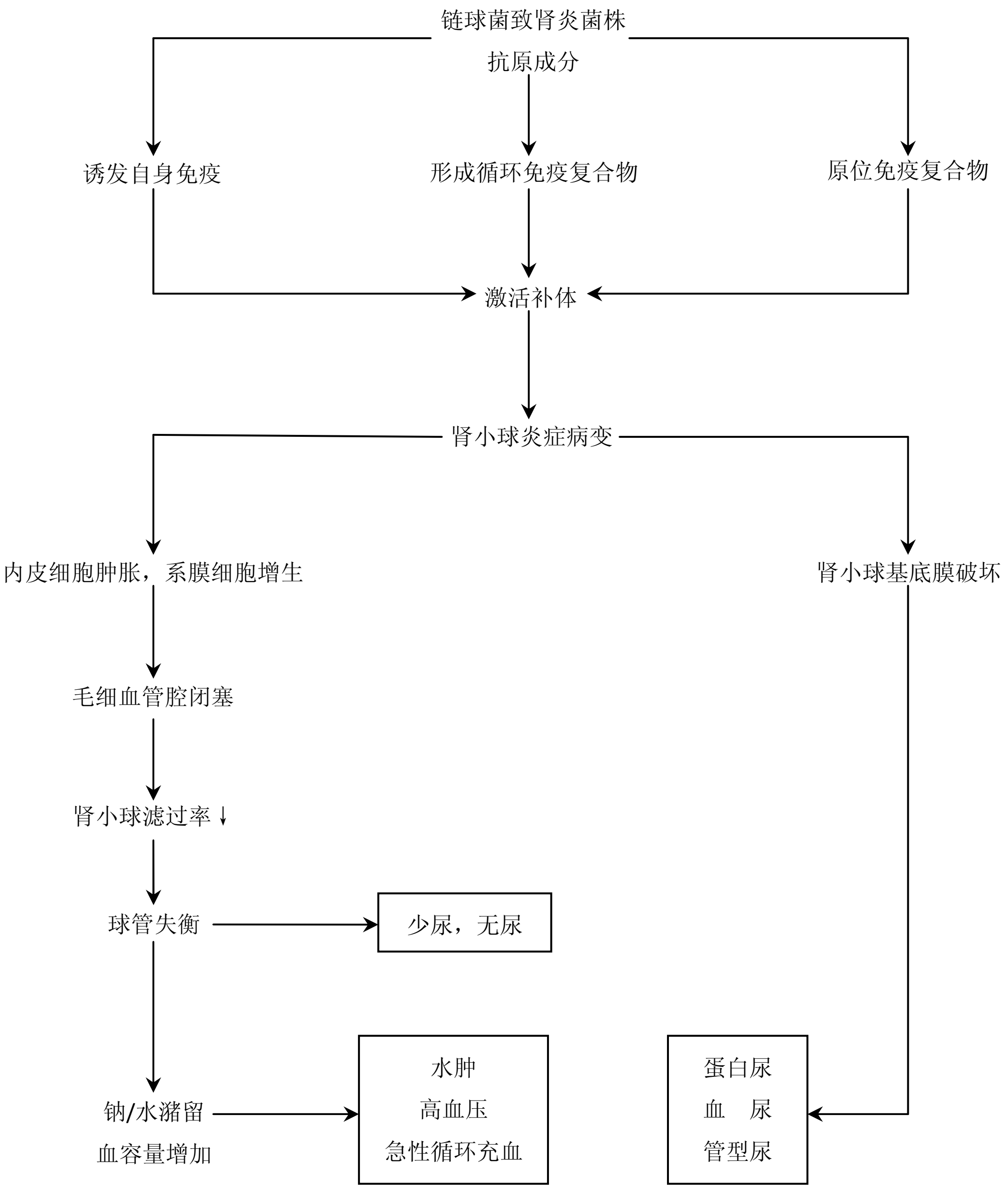

图14-1 急性链球菌感染后肾炎发病机制示意图

（1）水肿：70％的病例有水肿，一般仅累及眼睑及颜面部，重者2～3 天遍及全身，呈非凹陷性。

（2）血尿：50％～70％患者有肉眼血尿，持续1～2 周即转显微镜下血尿。

（3）蛋白尿：程度不等。有20％可达肾病水平。蛋白尿患者病理上常呈严重系膜增生。

（4）高血压：30％～80％病例有血压增高。

（5）尿量减少：肉眼血尿严重者可伴有排尿困难。

3．严重表现 少数患儿在疾病早期（2 周之内）可出现下列严重症状：

（1）严重循环充血：常发生在起病1 周内，由于水、钠潴留，血浆容量增加而出现循环充血。当肾炎患儿出现呼吸急促和肺部有湿啰音时，应警惕循环充血的可能性，严重者可出现呼吸困难、端坐呼吸、颈静脉怒张、频咳、吐粉红色泡沫痰、两肺满布湿啰音、心脏扩大、甚至出现奔马律、肝大而硬、水肿加剧。少数可突然发生，病情急剧恶化。

（2）高血压脑病：由于脑血管痉挛，导致缺血、缺氧、血管渗透性增高而发生脑水肿。

也有人认为是由脑血管扩张所致。常发生在疾病早期，血压突然上升之后，血压往往在150～160 mmHg/100～110 mmHg以上。年长儿会主诉剧烈头痛、呕吐、复视或一过性失明，严重者突然出现惊厥、昏迷。

（3）急性肾功能不全：常发生于疾病初期，出现尿少、尿闭等症状，引起暂时性氮质血症、电解质紊乱和代谢性酸中毒，一般持续3～5 日，不超过10 天。

4．非典型表现

（1）无症状性急性肾炎：为亚临床病例，患儿仅有显微镜下血尿或仅有血C3 降低而无其他临床表现。

（2）肾外症状性急性肾炎：有的患儿水肿、高血压明显，甚至有严重循环充血及高血压脑病，此时尿改变轻微或尿常规检查正常，但有链球菌前驱感染和血C3 水平明显降低。

（3）以肾病综合征表现的急性肾炎：少数病儿以急性肾炎起病，但水肿和蛋白尿突出，伴轻度高胆固醇血症和低白蛋白血症，临床表现似肾病综合征。

【实验室检查】

尿蛋白可在+～+++之间，且与血尿的程度相平行，尿显微镜下检查除多少不等的红细胞外，可有透明、颗粒或红细胞管型，疾病早期可见较多的白细胞和上皮细胞，并非感染。外周血白细胞一般轻度升高或正常，血沉加快。前驱期为咽炎病例抗链球菌溶血素O（ASO）往往增加，10～14 天开始升高，3～5 周时达高峰，3～6 个月后恢复正常。另外咽炎后APSGN者抗双磷酸吡啶核苷酸酶（ADPNase）滴度升高。皮肤感染后APSGN者ASO升高者不多，抗脱氧核糖核酸酶B（DANase-B）和抗透明质酸酶（HAase）滴度升高。80％～90％的病人血清C3 下降，至第8 周94％的病例恢复正常。明显少尿时血尿素氮和肌酐可升高。肾小管功能正常。持续少尿、无尿者，血肌酐升高，内生肌酐清除率降低，尿浓缩功能也受损。

【诊断及鉴别诊断】

往往有前期链球菌感染史，急性起病，具备血尿、蛋白尿和管型尿、水肿及高血压等特点，急性期血清ASO滴度升高，C3 浓度降低，均可临床诊断急性肾炎。作出APSGN 等诊断多不困难，肾穿刺活体组织检查只在考虑有急进性肾炎或临床、化验不典型或病情迁延者才进行以确定诊断。急性肾炎必须注意和以下疾病鉴别。

1．其他病原体感染的肾小球肾炎 多种病原体可引起急性肾炎，可从原发感染灶及各自临床特点相区别。

2．IgA肾病 以血尿为主要症状，表现为反复发作性肉眼血尿，多在上呼吸道感染后24～48 小时出现血尿，多无水肿、高血压、血清C3 正常。确诊靠肾活体组织检查免疫病理诊断。

3．慢性肾炎急性发作 既往肾炎史不详，无明显前期感染，除有肾炎症状外，常有贫血，肾功能异常，低比重尿或固定低比重尿，尿改变以蛋白增多为主。

4．原发性肾病综合征 具有肾病综合征表现的急性肾炎需与原发性肾病综合征鉴别。若患儿呈急性起病，有明确的链球菌感染的证据，血清C3 降低，肾活体组织检查病理为毛细血管内增生性肾炎者有助于急性肾炎的诊断。

5．其他 还应与急进性肾炎或其他系统性疾病引起的肾炎如紫癜性肾炎、狼疮性肾炎等相鉴别。

【治疗】

本病无特异治疗。

1．休息 急性期需卧床2～3 周，直到肉眼血尿消失，水肿减退，血压正常，即可下床作轻微活动。血沉正常可上学，但应避免重体力活动。尿沉渣细胞绝对计数正常后方可恢复体力

活动。

2．饮食 对有水肿、高血压者应限食盐及水。食盐以60mg/（kg·d）为宜。水分一般以不显性失水加尿量计算。有氮质血症者应限蛋白，可给优质动物蛋白0.5 g/（kg·d）。

3．抗感染 有感染灶时用青霉素10～14 天。

4．对症治疗

（1）利尿：经控制水、盐入量仍水肿、少尿者可用氢氯噻嗪1～2 mg/（kg·d），分2～3 次口服。无效时需用呋噻米，口服剂量2～5 mg/（kg·d），注射剂量1～2 mg/（kg·次），每日1～2次，静脉注射剂量过大时可有一过性耳聋。

（2）降血压：凡经休息，控制水、盐摄入、利尿而血压仍高者均应给予降压药。①硝苯地平：系钙通道阻滞剂。开始剂量为0.25 mg/（kg·d），最大剂量1 mg/（kg·d），分3 次口服。在成人此药有增加心肌梗死发生率和死亡率的危险，一般不单独使用。②卡托普利：系血管紧张素转换酶抑制剂。初始剂量为0.3～0.5 mg/（kg·d），最大剂量5～6 mg/（kg·d），分3 次口服，与硝苯地平交替使用降压效果更佳。

5．严重循环充血的治疗

（1）矫正水、钠潴留，恢复正常血容量，可使用呋噻米注射。

（2）表现有肺水肿者除一般对症治疗外可加用硝普钠，5～20 mg加入5％葡萄糖液100 ml中，以1μg/（kg·min）速度静滴，用药时严密监测血压，随时调节药液滴速，每分钟不宜超过8 μg/kg，以防发生低血压。滴注时针筒、输液管等须用黑纸覆盖，以免药物遇光分解。

（3）对难治病例可采用腹膜透析或血液滤过治疗。

6．高血压脑病的治疗 原则为选用降血压效力强而迅速的药物。首选硝普钠，用法同上。有惊厥者应及时止痉。

7．急性肾衰竭的治疗（见急性肾衰竭节）

【预后和预防】

急性肾炎急性期预后好。95％APSGN病例能完全恢复，小于5％的病例可有持续尿异常，死亡病例在1％以下，主要死因是急性肾衰竭。

防治感染是预防急性肾炎的根本。减少呼吸道及皮肤感染，对急性扁桃体炎、猩红热及脓疱患儿应尽早、彻底地用青霉素或其他敏感抗生素治疗。A组溶血性链球菌感染后1～3 周内应定期检查尿常规，及时发现和治疗本病。

第四节 肾病综合征

肾病综合征（nephrotic syndrome，NS）是一组由多种原因引起的肾小球基底膜通透性增加，导致血浆内大量蛋白质从尿中丢失的临床综合征。临床有以下四大特点：①大量蛋白尿；②低白蛋白血症；③高脂血症；④明显水肿。以上第①、②两项为必备条件。

肾病综合征在儿童肾脏疾病中发病率仅次于急性肾炎。1982 年我国的调查结果显示肾病综合征占同期住院泌尿系疾病患儿的21％。男女比例为3.7：1。发病年龄多为学龄前儿童，3～5 岁为发病高峰。肾病综合征按病因可分为原发性、继发性和先天性三种类型。本节主要叙述原发性肾病综合征（primary nephritic syndrome，PNS）。

[病因及发病机制]

原发性肾病综合征约占儿童时期肾病综合征总数的90％。原发性肾脏损害使肾小球通透性增加导致蛋白尿，而低蛋白血症、水肿和高胆固醇血症是继发的病理生理改变。

原发性肾病综合征的病因及发病机制目前尚不明确。近年研究已证实下列事实：①肾小球

毛细血管壁结构或电化学改变可导致蛋白尿。实验动物模型及人类肾病的研究看到微小病变时肾小球滤过膜多阴离子丢失，致静电屏障破坏，使大量带阴电荷的中分子血浆白蛋白滤出，形成高选择性蛋白尿。也可因分子滤过屏障损伤，尿中丢失大中分子量的多种蛋白，形成低选择性蛋白尿。②非微小病变型常见免疫球蛋白和（或）补体成分肾内沉积，局部免疫病理过程可损伤滤过膜正常屏障作用而发生蛋白尿。③微小病变型肾小球未见以上沉积，其滤过膜静电屏障损伤原因可能与细胞免疫失调有关。④患者外周血淋巴细胞培养上清液经尾静脉注射可致小鼠发生大量蛋白尿和肾病综合征的病理改变，表明T淋巴细胞异常参与本病的发病。

肾病综合征的发病具有遗传基础。国内报道糖皮质激素敏感肾病综合征患儿HLA-DR7抗原频率高达38％，频复发肾病综合征患儿则与HLA-DR9相关。另外肾病综合征还有家族性表现，且绝大多数是同胞患病。流行病学调查发现，黑人患肾病综合征症状表现重，对糖皮质激素反应差，提示肾病综合征发病与人种及环境有关。

【病理生理】

大量蛋白尿可引起以下病理生理改变。

1．低蛋白血症 血浆蛋白由尿中大量丢失和从肾小球滤出后被肾小管吸收分解是造成肾病综合征低蛋白血症的主要原因；肝脏合成蛋白的速度和蛋白分解代谢率的改变也使血浆蛋白降低。患儿胃肠道也可有少量蛋白丢失，但并非低蛋白血症的主要原因。

2．高脂血症 患儿血清总胆固醇、甘油三酯和低密度、极低密度脂蛋白增高，其主要机制是低蛋白血症促进肝脏合成脂蛋白增加，其中的大分子脂蛋白难以从肾脏排出而蓄积于体内，导致了高脂血症。血中胆固醇和低密度脂蛋白，尤其α脂蛋白持续升高，而高密度脂蛋白却正常或降低，促进了动脉硬化的形成；持续高脂血症，脂质从肾小球滤出，可导致肾小球硬化和肾间质纤维化。

3．水肿 水肿的发生与下列因素有关：①低蛋白血症降低血浆胶体渗透压，当血浆白蛋白低于25 g/L时，液体将在间质区潴留；低于15 g/L则可有腹水或胸水形成；②血浆胶体渗透压降低使血容量减少，刺激了渗透压和容量感受器，促使抗利尿激素和肾素-血管紧张素-醛固酮分泌、心钠素减少，最终使远端肾小管钠、水吸收增加，导致钠、水潴留；③低血容量使交感神经兴奋性增高，近端肾小管Na^+吸收增加；④某些肾内因子改变了肾小管管周体液平衡机制，使近曲小管Na^+吸收增加。

4．其他 患儿体液免疫功能降低与血清IgG和补体系统B、D因子从尿中大量丢失有关，也与T淋巴细胞抑制B淋巴细胞IgG合成转换有关。抗凝血酶Ⅲ丢失，而Ⅳ、Ⅴ、Ⅶ因子和纤维蛋白原增多，使患儿处于高凝状态。由于钙结合蛋白降低，血清结合钙可以降低；当25(OH)D_3结合蛋白同时丢失时，使游离钙也降低。另一些结合蛋白降低，可使结合型甲状腺素（T_3、T_4）、血清铁、锌和铜等微量元素降低，转铁蛋白减少则可发生低色素小细胞性贫血。

【病理】

原发性肾病综合征可见于各种病理类型。根据国际儿童肾脏病研究组（1979）对521 例儿童原发性肾病综合征的病理观察有以下类型：微小病变（76.4％），局灶性节段性肾小球硬化（6.9％），膜性增生性肾小球肾炎（7.5％），单纯系膜增生（2.3％），增生性肾小球肾炎（2.3％），局灶性球性硬化（1.7％），膜性肾病（1.5％），其他（1.4％）。儿童肾病综合征最主要的病理变化是微小病变型。

【临床表现】

水肿最常见，开始见于眼睑，以后逐渐遍及全身，呈凹陷，严重者可有腹水或胸腔积液。一般起病隐匿，常无明显诱因。大约30％有病毒感染或细菌感染发病史，70％肾病复发与病毒

感染有关。常伴有尿量减少，颜色变深，无并发症的患者无肉眼血尿，而短暂的镜下血尿可见于大约15%的病人。大多数血压正常，但轻度高血压也见于约15%的患者，严重的高血压通常不支持微小病变型肾病综合征的诊断。约30%病例因血容量减少而出现短暂肌酐清除率下降，一般肾功能正常，急性肾衰竭少见。部分病例晚期可有肾小管功能障碍，出现低血磷性佝偻病、肾性糖尿、氨基酸尿和酸中毒等。

【并发症】

1．感染 肾病患儿极易罹患各种感染。常见为呼吸道、皮肤、泌尿道感染和原发性腹膜炎等，其中尤以上呼吸道感染最多见，占50%以上。呼吸道感染中病毒感染常见。细菌感染中以肺炎链球菌为主，结核杆菌感染亦应引起重视。另外肾病患儿的医院内感染不容忽视，以呼吸道感染和泌尿道感染最多见，致病菌以条件致病菌为主。

2．电解质紊乱和低血容量 常见的电解质紊乱有低钠、低钾、低钙血症。患儿不恰当长期禁用食盐或长期食用不含钠的食盐代用品、过多使用利尿剂以及感染、呕吐、腹泻等因素均可致低钠血症。其临床表现可有厌食、乏力、懒言、嗜睡、血压下降甚至出现休克、抽搐等。另外由于低蛋白血症、血浆胶体渗透压下降、显著水肿、而常有血容量不足，尤在各种诱因引起低钠血症时易出现低血容量性休克。

3．血栓形成 肾病综合征高凝状态易致各种动、静脉血栓形成，以肾静脉血栓形成常见，表现为突发腰痛、出现血尿或血尿加重，少尿甚至发生肾衰竭。但临床以不同部位血管血栓形成的亚临床型则更多见。除肾静脉血栓形成外，可出现：①两侧肢体水肿程度差别固定，不随体位改变而变化，多见有下肢深静脉血栓形成；②皮肤突发紫斑并迅速扩大；③阴囊水肿呈紫色；④顽固性腹水；⑤下肢疼痛伴足背动脉搏动消失等症状及体征时，应考虑下肢动脉血栓形成；⑥股动脉血栓形成是儿童肾病综合征并发的急症之一，如不及时溶栓治疗可导致肢端坏死而需截肢；⑦不明原因的咳嗽、咯血或呼吸困难而无肺部阳性体征时要警惕肺栓塞，其半数可无临床症状；⑧突发的偏瘫、面瘫、失语、或神志改变等神经系统症状在排除高血压脑病、颅内感染性疾病时要考虑脑栓塞。血栓缓慢形成者其临床症状多不明显。

4．急性肾衰竭 5%微小病变型肾病可并发急性肾衰竭。

5．肾小管功能障碍 除原有肾小球的基础病可引起肾小管功能损害外，由于大量尿蛋白的重吸收，可导致肾小管（主要是近曲小管）功能损害。可出现肾性糖尿或氨基酸尿，严重者呈Fanconi综合征。

【实验室检查】

1．尿液分析

（1）常规检查：尿蛋白定性多在+++，约15%有短暂显微镜下血尿，大多可见透明管型、颗粒管型和卵圆脂肪小体。

（2）蛋白定量：24 小时尿蛋白定量检查超过40mg/（h·m^2）或>50mg/（kg·d）为肾病范围的蛋白尿。尿蛋白/尿肌酐（mg/mg），正常儿童上限为0.2，肾病>3.5。

2．血清蛋白、胆固醇和肾功能测定 血清白蛋白浓度为30 g/L（或更少）可诊断为肾病综合征的低白蛋白血症。由于肝脏合成增加，α_2、β球蛋白浓度增高，IgG减低，IgM、IgE可增加。胆固醇>5.7 μmol/L和三酰甘油升高，LDL和VLDL增高，HDL多正常。BUN、Cr在肾炎性肾病综合征可升高，晚期可有肾小管功能损害。

3．血清补体测定 微小病变型肾病综合征或单纯性肾病综合征患儿血清补体水平正常，肾炎性肾病综合征患儿补体可下降。

4．系统性疾病的血清学检查 对新诊断的肾病患者需检测抗核抗体（ANA），抗-ds-DNA

抗体，Smith抗体等。对具有血尿、补体减少并有临床表现的患者尤其重要。

5．高凝状态和血栓形成的检查 多数原发性肾病患儿都存在不同程度的高凝状态，血小板增多，血小板聚集率增加，血浆纤维蛋白原增加，尿纤维蛋白裂解产物（FDP）增高。对疑及血栓形成者可行彩色多普勒B型超声检查以明确诊断，有条件者可行数字减影血管造影（DSA）。

6．经皮肾穿刺组织病理学检查 多数儿童肾病综合征不需要进行诊断性肾活体组织检查。肾病综合征肾活体组织检查指征：①对糖皮质激素治疗耐药或频繁复发者；②对临床或实验室证据支持肾炎性肾病或继发性肾病综合征者。

【诊断与鉴别诊断】

临床上根据有无血尿、高血压、氮质血症和低补体血症，将原发性肾病综合征分为单纯性和肾炎性肾病综合征（见第二节）。

原发性肾病综合征还需与继发于全身性疾病的肾病综合征鉴别。部分非典型链球菌感染后肾炎、系统性红斑狼疮性肾炎、过敏性紫癜性肾炎、乙型肝炎病毒相关性肾炎及药源性肾炎等均可有肾病综合征样表现。临床上须排除继发性肾病综合征后方可诊断原发性肾病综合征。有条件的医疗单位应开展肾活体组织检查以确定病理诊断。

【治疗】

1．一般治疗

（1）休息：除水肿显著或并发感染，或严重高血压外，一般不需卧床休息。病情缓解后逐渐增加活动量。

（2）饮食：显著水肿和严重高血压时应短期限制水钠摄入，病情缓解后不必继续限盐。活动期病例供盐1～2 g/d。蛋白质摄入1.5～2 g/（kg·d），以高生物价的动物蛋白（乳、鱼、蛋、禽、牛肉等）为宜。在应用糖皮质激素过程中每日应给予维生素D 400 u及适量钙剂。

（3）防治感染。

（4）利尿：对糖皮质激素耐药或未使用糖皮质激素，而水肿较重伴尿少者可配合使用利尿剂，但需密切观察出入水量、体重变化及电解质紊乱。

（5）对家属的教育：应使父母及患儿很好地了解肾病的有关知识，积极配合随访和治疗。

2．糖皮质激素

（1）初治病例诊断确定后应尽早选用泼尼松治疗。

1）短程疗法：泼尼松2 mg/（kg·d）（按身高标准体重，以下同），最大量60 mg/d，分次服用，共4 周。4 周后不管效应如何，均改为泼尼松1.5 mg/kg 隔日晨顿服，共4 周，全疗程共8 周，然后骤然停药。短程疗法易于复发，国内少用。

2）中、长期疗法：可用于各种类型的肾病综合征。先以泼尼松2 mg/（kg·d），最大量60 mg/d，分次服用。若4 周内尿蛋白转阴，则自转阴后至少巩固2 周方始减量，以后改为隔日2 mg/ kg早餐后顿服，继用4 周，以后每2～4 周减总量2.5～5 mg，直至停药。疗程必须达6 个月（中程疗法）。开始治疗后4 周尿蛋白未转阴者可继服至尿蛋白阴转后2 周，一般不超过8 周。以后再改为隔日2 mg/kg早餐后顿服，继用4 周，以后每2～4 周减量一次，直至停药，疗程9 个月（长程疗法）。

（2）复发和糖皮质激素依赖性肾病的其他激素治疗

1）调整糖皮质激素的剂量和疗程：糖皮质激素治疗后或在减量过程中复发者，原则上再次恢复到初始疗效剂量或上一个疗效剂量。或改隔日疗法为每日疗法，或将激素减量的速度放慢，延长疗程。同时注意查找患儿有无感染或影响糖皮质激素疗效的其他因素存在。

2）更换糖皮质激素制剂：对泼尼松疗效较差的病例，可换用其它糖皮质激素制剂，如：

阿赛松（triamcinolone，曲安西龙）、康宁克通A（kenacort A）等。

3）甲基泼尼松龙冲击治疗：慎用，宜在肾脏病理基础上，选择适应证。

（3）激素治疗的副作用：长期超生理剂量使用糖皮质激素可见以下副作用：①代谢紊乱，可出现明显柯兴貌、肌肉萎缩无力、伤口愈合不良、蛋白质营养不良、高血糖、尿糖、水钠潴留、高血压、尿中失钾、高尿钙和骨质疏松；②消化性溃疡和精神欣快感、兴奋、失眠甚至呈精神病、癫痫发作等；还可发生白内障、无菌性股骨头坏死、高凝状态，生长停滞等；③易发生感染或诱发结核灶的活动；④急性肾上腺皮质功能不全，戒断综合征。

3．免疫抑制剂 主要用于肾病综合征频繁复发，糖皮质激素依赖、耐药或出现严重副作用者。在小剂量糖皮质激素隔日使用的同时可选用下列免疫抑制剂。

（1）环磷酰胺：一般剂量2.0～2.5 mg/（kg・d），分3 次口服，疗程8～12 周，总量不超过200 mg/kg。或用环磷酰胺冲击治疗，剂量10～12 mg/（kg・d），加入5％葡萄糖盐水100～200 ml内静滴1～2 小时，连续2 天为一疗程。用药日嘱多饮水，每2 周重复一疗程，累积量<150~200 mg/kg。副作用有：白细胞减少，秃发，肝功能损害，出血性膀胱炎等，少数可发生肺纤维化。注意远期性腺损害。病情需要者可小剂量、短疗程、间断用药，避免青春期前和青春期用药。

（2）其他免疫抑制剂：可根据病例需要选用苯丁酸氮芥、环孢素A、硫唑嘌呤、霉酚酸酯及雷公藤多甙片等。

4．抗凝及纤溶药物疗法 由于肾病往往存在高凝状态和纤溶障碍，易并发血栓形成，需加用抗凝和溶栓治疗。

（1）肝素钠 1 mg/（kg・d），加入10％葡萄糖液50～100 ml中静脉点滴，每日1 次，2～4 周为一疗程。亦可选用低分子肝素。病情好转后改口服抗凝药维持治疗。

（2）尿激酶：有直接激活纤溶酶溶解血栓的作用。一般剂量3 万U～6 万U/d，加入10％葡萄糖液100～200 ml中静脉滴注，1～2 周为一疗程。

（3）口服抗凝药：双嘧达莫5～10 mg/（kg・d），分3 次饭后服，6 个月为一疗程。

5．免疫调节剂 一般作为糖皮质激素辅助治疗，适用于常伴感染、频复发或糖皮质激素依赖者。左旋咪唑2.5 mg/kg，隔日用药，疗程6 个月。副作用可有胃肠不适，流感样症状、皮疹、周围血液中性粒细胞下降，停药即可恢复。

6．血管紧张素转换酶抑制剂（ACEI） 对改善肾小球局部血流动力学，减少尿蛋白，延缓肾小球硬化有良好作用。尤其适用于伴有高血压的肾病综合征。常用制剂有卡托普利（captopril）、依那普利（enalapril）、福辛普利（fosinopril）等。

7．中医药治疗 肾病综合征属中医“水肿”、“阴水”、“虚劳”的范畴。可根据辨证施治原则进行治疗。

【预后】

肾病综合征的预后转归与其病理变化和对糖皮质激素治疗反应关系密切。微小病变型预后最好，局灶节段性肾小球硬化预后最差。微小病变型90％～95％的病儿对首次应用糖皮质激素有效。其中85％可有复发，复发在第一年比以后更常见。3～4 年未复发者，其后有95％的机会不复发。微小病变型预后较好，但要注意严重感染或糖皮质激素的严重副作用。局灶节段性肾小球硬化者如对糖皮质激素敏感，则预后可改善。

【附】 先天性肾病综合征

先天性肾病综合征（congenital nephritic syndrome）通常指生后3 个月内发病，临床表现符合肾病综合征，并除外继发所致者（如TORCH或先天性梅毒感染所致等）。其中包括典型的芬兰型肾病综合征、弥漫性系膜硬化（DMS）和生后早期发生的原发性肾病综合征。

【病因及发病机制】

本病为常染色隐性遗传性疾病，本病的确切发病机制仍然不清。

【病理】

本病患儿肾脏体积及重量是正常肾脏的2～3 倍，肾单位也明显增多。光镜下没有特异性的病变。生后1 个月肾脏可出现皮质小管囊性改变和增生性肾脏损害；最终小囊中的上皮细胞扁平，刷状缘结构消失，小管萎缩。晚期可见终末期肾病病理改变。

【临床表现】

多数患儿生后3 个月已表现出典型的肾病综合征。常有早产史或胎儿窘迫史，常见臀位，大胎盘（胎盘重量>胎儿体重的25％）。患儿出生时即有明显蛋白尿，镜下血尿也常见。几乎所有患儿出生后2 个月内出现水肿，伴有腹胀和腹水。血清白蛋白很低，血浆胆固醇正常或升高。部分病例可发生缺铁性贫血、生长障碍、骨化延迟和甲状腺功能低下等。血清尿素氮和肌酐最初大多数正常，一般在1 岁后进入肾衰竭。母亲孕期常合并妊娠中毒症。

【实验室检查】

除大量蛋白尿外，常有显微镜下血尿。可见轻度氨基酸尿和糖尿。血浆蛋白降低，血浆胆固醇可高或不高。血清C3正常或下降。母血和羊水中甲胎蛋白阳性。

【诊断与鉴别诊断】

诊断本病主要依据阳性家族史，大量蛋白尿，巨大胎盘，出生6 个月内肾功能正常，必要时应行肾穿刺活组织检查。

临床上需与下列类型先天性肾病综合征鉴别：

1．弥漫性系膜硬化

2．婴儿肾病综合征继发于全身疾病 ①先天性梅毒伴肾病综合征；②伴有生殖器畸形的肾病综合征；③肾胚胎瘤及肾静脉栓塞。

3．其他类型肾病综合征

【治疗】

糖皮质激素和免疫抑制剂治疗无效，需定期输注白蛋白，肾移植是最佳选择。

【预后】

本病预后差，如不能及时行肾移植病死率高。

第五节 乙型肝炎病毒相关性肾炎

乙型肝炎病毒相关性肾炎（hepatitis B virus associated glomerulonephritis，HBV-GN）简称乙肝肾炎，是指HBV感染人体后通过免疫反应形成免疫复合物损伤肾小球或HBV直接侵袭肾组织引起的肾小球肾炎。临床表现为蛋白尿、血尿或肾病综合征，典型病理改变为膜性肾病。HBV感染伴肾小球肾炎的发病率约为6.8％～20.0％。儿童多见。

【发病机制】

乙型肝炎病毒相关性肾炎的发病机制尚未完全清楚，可能有几种方式致病。

1．HBV抗原-抗体复合物沉积于肾小球导致免疫损伤，可以两种形式致病。

（1）循环免疫复合物：HBV感染人体后，与其血清抗体可能在血循环中形成免疫复合物，沉积于肾小球毛细血管袢，激活补体造成免疫损伤。HBsAg与HBcAg的分子量较大，且带有负电荷，因此它们形成的免疫复合物很难穿透肾小球基膜而进入上皮下，主要沉积于内皮下及系

膜区引起系膜毛细血管性肾炎或系膜增生性肾炎。HBeAg分子量小，其所形成的免疫复合物的分子量也较小，HBeAg虽也带负电荷，但其抗体HBeAb却带有强大的正电荷，因此此种复合物可透过基膜沉积于上皮下而引起膜性肾病。

（2）原位免疫复合物：分子量较小的HBeAg可以穿过基膜与先植入上皮下的HBeAb结合形成原位免疫复合物，沉积于肾小球上皮下而致病。

2．病毒直接感染肾脏细胞 无论动物实验还是人体研究，均在肾组织中找到了HBV DNA，提示HBV有直接感染肾脏的可能。

3. HBV感染导致自身抗体和细胞免疫损伤 HBV感染刺激机体产生多种自身抗体如抗DNA抗体、抗细胞骨架成分抗体、抗肝细胞膜特异脂蛋白抗体、抗肾小管刷状缘抗体等。HBV感染靶细胞后引起细胞毒性T细胞对靶细胞免疫杀伤，改变靶细胞膜的抗原决定簇，引起自身免疫反应。自身免疫损伤的发生可能与免疫调节功能缺陷密切相关。

【病理】

主要表现为膜性肾病，其次为系膜毛细血管性肾炎、系膜增生性肾炎、局灶节段硬化性肾炎，毛细血管内增生性肾炎偶见。

HBV相关膜性肾病是HBV相关性肾炎的主要病理表现，与特发性膜性肾炎有所不同。HBV相关膜性肾病大多数肾小球毛细血管壁呈弥漫性一致的增厚，银染色基膜见多数“钉突”，有时见系膜区轻度扩大，系膜细胞轻度增生，电镜下见增生的系膜细胞有插入。上皮下及膜内见大量团块状电子致密物沉积，基膜增厚。免疫组织化学法检测可见HBeAg 和（或）HBsAg 呈颗粒状沿肾小球毛细血管袢沉积，少数有间质及小管沉积，伴IgG（100％）、C3 （75％）沉积，少数有IgA、IgM沉积。

【临床表现】

起病年龄多为儿童及青少年。男性居多，临床表现多样。

1．肾脏症状 所有患者均出现镜下血尿或蛋白尿，起病隐匿，多在查尿时发现。部分患者可以肾炎综合征或肾病综合征起病。表现为肾病综合征者，伴有不同程度水肿，可有大量腹水。表现为系膜毛细血管性肾炎者，40％有血压升高，20％有肾功能不全。表现为膜性肾病者，无血压升高和肾功能不全。

2．肝脏症状 大多数无肝炎病史和肝炎的临床表现，部分病人可有肝脏增大或肝功能异常。

【实验室检查】

蛋白尿明显，可伴不同程度镜下血尿和管型尿。表现为肾病综合征者，有大量蛋白尿和低蛋白血症。肾功能多数正常，部分系膜毛细血管性肾炎者可有肾功能不全。

几乎全部病人血HBsAg阳性，60％～80％病例HBeAg阳性。血清C3、C4 减低，冷球蛋白增多，白蛋白减少，胆固醇轻度增高，谷丙转氨酶及谷草转氨酶可增高。有人认为球蛋白增多是HBV-GN的主要特征，血IgG、IgA增高，提示病变处于活动期。

【诊断】

中华儿科学会肾脏病学组于2000 年11 月珠海会议制定：①血清HBV标志物持续阳性; ②患肾小球肾炎并可除外其他继发性肾小球疾病；③肾组织切片中找到HBV抗原或HBV DNA; ④肾组织病理为膜性肾病。凡符合第①、②、③条可确诊，不论其肾组织病理为何；符合第①、②、④条时，尽管其肾组织切片中未查到HBV抗原或HBV DNA，可作为拟诊。

【治疗】

1．一般治疗 合理的生活制度，恰当的营养，定期的医疗随诊很重要。表现为肾病综合征者，可用优质蛋白、低盐饮食，予以利尿剂或静脉补充白蛋白等非特异治疗。

2．抗病毒治疗：①α-干扰素具有抗病毒作用，通过与细胞表面受体特异性结合阻断病毒的繁殖和复制，但不能进入宿主细胞直接杀灭病毒。可使用重组人类α干扰素（α-IFN）100 万～300 万IU肌注，每周3 次，6 个月为一疗程。主要副作用为发热、流感样症状、嗜睡和乏力，少数患者发生多形红斑。个别病例出现精神症状或原有神经症状加重，应及时减量或停药。②阿糖腺苷（Ara-A）：在体内转化能抑制DNA多聚酶和还原酶，从而抑制病毒复制。剂量15 mg/（kg・d）静脉点滴，2 周为一疗程，联合应用α-IFN可取得较好效果。③胸腺肽α：具有免疫调节作用，与α-IFN合用时HBV转阴率较单用α-IFN明显提高。④核苷（酸）类似物：可以直接抑制乙肝病毒的复制，从而控制和改善临床症状。小年龄儿童慎用。

糖皮质激素短程试用，可减轻或消除蛋白尿，但因有促进HBV在细胞内复制的潜在危险，使病理改变迁徙不愈或加重，不可单独使用，要与抗病毒治疗同时使用。

【预后】

HBV-GN的预后与病理类型有关。膜性肾病50％可自发缓解，当血清HBeAg转阴而出现HBeAb时，尿和肝功能异常也相继改善。而病理表现为系膜毛细血管性肾炎、局灶节段硬化性肾炎者预后较差，可渐进至肾功能不全。

第六节 泌尿道感染

泌尿道感染（urinary tract infection，UTI）是指病原体直接侵入尿路，在尿液中生长繁殖，并侵犯尿路黏膜或组织而引起损伤。按病原体侵袭的部位不同，分为肾盂肾炎（pyelonephritis）、膀胱炎（cystitis）、尿道炎（urethritis）。肾盂肾炎又称上尿路感染；膀胱炎和尿道炎合称下尿路感染。由于儿童时期感染局限在尿路某一部位者较少，且临床上又难以准确定位，故常不加区别统称为泌尿道感染。可根据有无临床症状，分为症状性泌尿道感染（symptomatic urinary tract infection）和无症状性菌尿（asymptomatic bacteriuria）。

据我国1982 年全国调查显示，尿路感染占本系统疾病的8.5％；1987 年全国21 省市儿童尿过筛检查统计，泌尿道感染占儿童泌尿系疾病的12.5％。无论成人或儿童，女性泌尿道感染的发病率普遍高于男性，但新生儿或婴幼儿早期，男性发病率却高于女性。

无症状性菌尿是儿童泌尿道感染的一个重要组成部分，见于各年龄、性别儿童，甚至3 个月以下的小婴儿，但以学龄女孩更常见。

【病因】

任何致病菌均可引起泌尿道感染，但绝大多数为革兰阴性杆菌，如大肠杆菌、副大肠杆菌、变形杆菌、克雷伯杆菌、绿脓杆菌，少数为肠球菌和葡萄球菌。大肠杆菌是泌尿道感染中最常见的致病菌，约占60％～80％。初次患泌尿道感染的新生儿、所有年龄的女孩和1 岁以下的男孩，主要的致病菌仍是大肠杆菌；而在1 岁以上男孩主要致病菌多是变形杆菌。对于10～16 岁的女孩，白色葡萄球菌亦常见；克雷伯杆菌和肠球菌多见于新生儿泌尿道感染。

【发病机制】

细菌引起泌尿道感染的发病机制错综复杂，是宿主内在因素与细菌致病性相互作用的结果。

1．感染途径

（1）血源性感染：经血源途径侵袭尿路的致病菌主要是金黄色葡萄球菌。

（2）上行性感染：致病菌从尿道口上行并进入膀胱，引起膀胱炎，膀胱内的致病菌再经输尿管移行至肾脏，引起肾盂肾炎，这是泌尿道感染最主要的途径。引起上行性感染的致病菌

主要是大肠杆菌，其次是变形杆菌或其他肠杆菌。膀胱输尿管反流（vesicoureteral reflux，VUR）常是细菌上行性感染的直接通道。

（3）淋巴感染和直接蔓延：结肠内的细菌和盆腔感染可通过淋巴管感染肾脏，肾脏周围邻近器官和组织的感染也可直接蔓延。

2．宿主内在因素

（1）尿道周围菌种的改变及尿液性状的变化，为致病菌入侵和繁殖创造了条件。

（2）细菌黏附于尿路上皮细胞（定植）是其在泌尿道增殖引起泌尿道感染的先决条件。

（3）泌尿道感染患者分泌型IgA的产生存在缺陷，使尿中分泌型IgA浓度减低，增加发生泌尿道感染的机会。

（4）先天性或获得性尿路畸形，增加尿路感染的危险性。

（5）新生儿和小婴儿抗感染能力差，易患泌尿道感染。尿布、尿道口常受细菌污染，且局部防卫能力差，易致上行感染。

（6）糖尿病、高钙血症、高血压、慢性肾脏疾病、镰刀状细胞贫血及长期使用糖皮质激素或免疫抑制剂的患儿，其泌尿道感染的发病率可增高。

3．细菌毒力 宿主无特殊易感染内在因素，如泌尿系结构异常者，微生物的毒力是决定细菌能否引起上行性感染的主要因素。

【临床表现】

1．急性泌尿道感染 临床症状随患儿年龄组的不同存在着较大差异。

（1）新生儿：临床症状极不典型，多以全身症状为主，如发热或体温不升、苍白、吃奶差、呕吐、腹泻等。许多患儿有生长发育停滞，体重增长缓慢或不增，伴有黄疸者较多见。部分患儿可有嗜睡、烦躁甚至惊厥等神经系统症状。新生儿泌尿道感染常伴有败血症，但其局部排尿刺激症状多不明显，30％的患儿血和尿培养出的致病菌一致。

（2）婴幼儿：临床症状也不典型，常以发热最突出。拒食、呕吐、腹泻等全身症状也较明显。局部排尿刺激症状可不明显，但细心观察可发现有排尿时哭闹不安，尿布有臭味和顽固性尿布疹等。

（3）年长儿：以发热、寒战、腹痛等全身症状突出，常伴有腰痛和肾区叩击痛，肋脊角压痛等。同时尿路刺激症状明显，患儿可出现尿频、尿急、尿痛、尿液浑浊，偶见肉眼血尿。

2．慢性泌尿道感染 是指病程迁延或反复发作伴有贫血、消瘦、生长迟缓、高血压或肾功能不全者。

3．症状性菌尿 在常规的尿过筛检查中，可以发现健康儿童存在着有意义的菌尿，但无任何尿路感染症状。这种现象可见于各年龄组，在儿童中以学龄女孩常见。无症状性菌尿患儿常同时伴有尿路畸形和既往症状尿路感染史。病原体多数是大肠杆菌。

【实验室检查】

1．尿常规检查及尿细胞计数 ①尿常规检查：如清洁中段尿离心沉渣中白细胞>10 个/HPF，即可怀疑为尿路感染。血尿也很常见。肾盂肾炎患者有中等蛋白尿、白细胞管型尿及晨尿的比重和渗透压减低。②1 小时尿白细胞排泄率测定：白细胞数>30×10^4/h为阳性，可怀疑尿路感染；<20×10^4/h为阴性，可排除尿路感染。

2．尿培养细菌学检查 尿细菌培养及菌落计数是诊断尿路感染的主要依据。通常认为中段尿培养菌落数>10^5/ml可确诊。10^4～10^5/ml为可疑，<10^4/ml系污染。但结果分析应结合患儿性别、有无症状、细菌种类及繁殖力综合评价临床意义。由于粪链球菌一个链含有32 个细菌，

一般认为菌落数在10^3～10^4/ml之间即可诊断。已通过耻骨上膀胱穿刺获取的尿培养，只要发现有细菌生长，即有诊断意义。至于伴有严重尿路刺激症状的女孩，如果尿中有较多白细胞，中段尿细菌定量培养≥10^2/ml，且致病菌为大肠杆菌类或腐物寄生球菌等，也可诊断为泌尿道感染。临床高度怀疑泌尿道感染而尿普通细菌培养阴性的，应作L-型细菌和厌氧菌培养。

3．尿液直接涂片法找细菌 油镜下如每个视野都能找到一个细菌，表明尿内细菌数> 10^5/ml以上。

4．亚硝酸盐试纸条试验（Griess试验） 大肠杆菌、副大肠杆菌和克雷伯杆菌呈阳性，产气、变形、绿脓和葡萄球菌为弱阳性，粪链球菌、结核菌阴性。如采用晨尿，可提高其阳性率。

5．其他 如尿沉渣找闪光细胞（甲紫沙黄染色）2 万个～4 万个/小时可确诊。新生儿上尿路感染血培养可阳性。

【影像学检查】

影像学检查目的在于：①检查泌尿系有无先天性或获得性畸形；②了解以前由于漏诊或治疗不当所引起的慢性肾损害或疤痕进展情况；③辅助上尿路感染的诊断。常用的影像学检查有B型超声检查、静脉肾盂造影加断层摄片（检查肾疤痕形成）、排泄性膀胱尿路造影（检查膀胱输尿管反流）、动态、静态肾核素造影、CT扫描等。

【诊断与鉴别诊断】

年长儿泌尿道感染症状与成人相似，尿路刺激症状明显，常是就诊的主诉。如能结合实验室检查，可立即得以确诊。但对于婴幼儿、特别是新生儿，由于排尿刺激症状不明显或缺如，而常以全身表现较为突出，易致漏诊。故对病因不明的发热患儿都应反复作尿液检查，争取在用抗生素治疗前进行尿培养、菌落计数和药敏试验。凡具有真性菌尿者，即清洁中段尿定量培养菌落数≥10^5/ml或球菌≥10^3/ml，或耻骨上膀胱穿刺尿定性培养有细菌生长，即可确立诊断。

完整的泌尿道感染的诊断除了评定泌尿系被细菌感染外，还应包括以下内容：①本次感染系初染、复发或再感；②确定致病菌的类型并做药敏试验；③有无尿路畸形如膀胱输尿管反流、尿路梗阻等，如有膀胱输尿管反流，还要进一步了解“反流”的严重程度和有无肾脏疤痕形成；④感染的定位诊断，即上尿路感染或下尿路感染。

泌尿道感染需与肾小球肾炎、肾结核及急性尿道综合征鉴别。急性尿道综合征的临床表现为尿频、尿急、尿痛、排尿困难等尿路刺激症状，但清洁中段尿培养无细菌生长或为无意义性菌尿。

【治疗】

治疗目的是控制症状，根除病原体，去除诱发因素，预防再发。

1．一般处理

（1）急性期需卧床休息，鼓励患儿多饮水以增加尿量，女孩还应注意外阴部的清洁卫生。

（2）鼓励患儿进食，供给足够的热能、丰富的蛋白质和维生素，以增强机体的抵抗力。

（3）对症治疗：对高热、头痛、腰痛的患儿应给予解热镇痛剂缓解症状。对尿路刺激症状明显者，可用阿托品、山莨菪碱等抗胆碱药物治疗或口服碳酸氢钠碱化尿液。以减轻尿路刺激症状。

2．抗菌药物治疗 选用抗生素的原则：①感染部位：对肾盂肾炎应选择血浓度高的药物，对膀胱炎应选择尿浓度高的药物。②感染途径：对上行性感染，首选磺胺类药物治疗。如发热等全身症状明显或属血源性感染，多选用青霉素类、氨基糖甙类或头孢菌素类单独或联合治疗。

③根据尿培养及药敏试验结果，同时结合临床疗效选用抗生素。④药物在肾组织、尿液、血液中都应有较高的浓度。⑤选用的药物抗菌能力强，抗菌谱广，最好能用强效杀菌剂，且不易使细菌产生耐药菌株。⑥对肾功能损害小的药物。

（1）症状性泌尿道感染的治疗：对单纯性泌尿道感染，在进行尿细菌培养后，初治首选复方磺胺异恶唑（SMZ Co），按SMZ 50 mg/（kg・d），TMP l0 mg/（kg・d）计算，分2 次口服，连用7～10 天。待尿细菌培养结果出来后药敏试验结果选用抗菌药物。

对上尿路感染或有尿路畸形患儿，在进行尿细菌培养后，一般选用两种抗菌药物。新生儿和婴儿用氨苄西林75～l00 mg/（kg・d）静注，加头孢噻肟钠50～l00 mg/（kg・d）静注，连用10～14天；1 岁后儿童用氨苄西林100～200 mg/（kg・d）分3 次滴注，或用头孢噻肟钠，也可用头孢曲松钠50～75 mg/（kg・d）静脉缓慢滴注，疗程共10～14 天。治疗开始后应连续3 天送尿细菌培养，若24 小时后尿培养阴转，表示所用药物有效，否则按尿培养药敏试验结果调整用药。停药1 周后再作尿培养一次。

（2）无症状菌尿的治疗：单纯无症状菌尿一般无需治疗。但若合并尿路梗阻、膀胱输尿管反流或存在其他尿路畸形，或既往感染使肾脏留有陈旧性疤痕者，则应积极选用上述抗菌药物治疗。疗程7～14 天，继之给予小剂量抗菌药物预防，直至尿路畸形被矫治为止。

（3）再发泌尿道感染的治疗：再发泌尿道感染有两种类型，即复发和再感染。复发是使原来感染的细菌未完全杀灭，在适宜的环境下细菌再度滋生繁殖。绝大多数患儿复发多在治疗后1 月内发生。再感染是指上次感染已治愈，本次是由不同细菌或菌株再次引发泌尿道感染。再感染多见于女孩。多在停药后6 个月内发生。

再发泌尿道感染的治疗在进行尿细菌培养后选用2 种抗菌药物治疗，疗程10～14 天为宜，然后予以小剂量药物维持，以防再发。

3．积极矫治尿路畸形。

4．泌尿道感染的局部治疗 常采用膀胱内药液灌注治疗，主要治疗经全身给药治疗无效的顽固性慢性膀胱炎经全身。

【预后】

急性泌尿道感染经合理抗菌治疗，多数于数日内症状消失、治愈，但有近50％患者可复发或再感染。再发病例多伴有尿路畸形，其中以膀胱输尿管反流最常见。膀胱输尿管反流与肾瘢痕关系密切，肾瘢痕的形成是影响儿童泌尿道感染预后的最重要因素。肾瘢痕在学龄期儿童最易形成，10 岁后进展不明显。一旦肾瘢痕引起高血压，如不能被有效控制，最终发展为慢性肾衰竭。

【预防】

泌尿道感染的预防包括：①注意个人卫生，不穿紧身内裤，勤洗外阴以防止细菌入侵；②及时发现和处理男孩包茎、女孩处女膜伞、蛲虫感染等；③及时矫治尿路畸形，防止尿路梗阻和肾瘢痕形成。

【附】膀胱输尿管反流和反流性肾病

【病因及分类】

导致膀胱输尿管反流的主要机制是膀胱输尿管连接部异常。按发生原因可分以下两类：

1．原发性 最常见，为先天性膀胱输尿管瓣膜机制不全。53％的病例为膀胱逼尿肌功能异常所致反流。

2．继发性 导致Waldeyer’s鞘功能紊乱的因素有泌尿道感染、膀胱颈及下尿路梗阻、创伤

等，儿童泌尿道感染并发反流者高达30％～50％。

【发病机制】

反流性肾病（reflux nephropathy，RN）的发病机制目前仍未阐明，膀胱输尿管反流引起肾损害可能是多因素所致：①菌尿；②尿动力学改变；③尿液漏入肾组织；④肾内血管狭窄；⑤肾小球硬化；⑥遗传因素。

【病理】

有反流的乳头管、集合管明显扩张，管壁周围间质充血水肿，淋巴细胞及中性粒细胞浸润，继之肾小管萎缩，局灶性及肾小球周围纤维化。肾盏、肾盂扩张、肾实质变薄，重度膀胱输尿管反流伴反复泌尿道感染者瘢痕广泛，一般肾上、下极突出（即极性分布倾向）。小动脉可有增厚狭窄。

【临床表现】

反流性肾病最常见的临床表现为反复发作的泌尿道感染。膀胱刺激症状仅在泌尿道感染急性期出现。

1．无症状性反流 无任何症状体征，仅在因其他原因作B超或排尿性膀胱造影时才被发现。

2．泌尿系感染 膀胱输尿管反流常合并泌尿道感染，且易反复。

3．反流性肾病 蛋白尿可为反流性肾病的首发症状，亦可在严重疤痕形成数年后才出现。

4．其他 夜尿、多尿，尿淋漓不尽，在儿童可以遗尿作为首发症状。

【辅助检查】

1．实验室检查 泌尿道感染时尿常规检查有脓尿，尿细菌培养阳性。

2．超声检查 通过B超可观察输尿管扩张、蠕动及膀胱基底部的连续性；观察肾盂、肾脏形态及实质改变情况。

3．X线检查

（1）排尿性膀胱尿路造影：此为常用的确诊膀胱输尿管反流的基本方法及分级的“金标准”。

（2）静脉肾盂造影（IVP）：可进一步确诊有无肾萎缩及肾瘢痕形成。

4．放射性核素检查

（1）放射性核素膀胱显像：分直接测定法和间接测定法，用于测定膀胱输尿管反流。

（2）DMSA扫描技术：用于尿无菌的患者，对诊断儿童反流性肾病是唯一的“金标准”。

【诊断】

由于临床诊断膀胱输尿管反流时症状多不明显或仅有非特异性表现，故确诊需依赖影像学检查。

1．下列情况应考虑反流存在可能性 ①反复复发和迁延的泌尿道感染；②长期尿频、尿淋漓或遗尿；③年龄较小（<2 岁）和（或）男孩泌尿道感染；④中段尿培养持续阳性；⑤泌尿道感染伴尿路畸形；⑥家族一级亲属有膀胱输尿管反流、反流性肾病患者；⑦胎儿或婴儿期有肾盂积水。

2．反流性肾病的诊断 确诊依赖影像学检查，临床表现和肾活体组织检查病理改变有助诊断。

【治疗】

主要是制止尿液反流和控制感染，防止肾功能进一步损害。

1．内科治疗 按膀胱输尿管反流的不同分级采用治疗措施。

（1）Ⅰ、Ⅱ级：治疗感染和长期服药预防。

（2）Ⅲ级：处理同Ⅰ、Ⅱ级，但须每隔6 个月检查一次反流，每年做静脉肾盂造影。

（3）Ⅳ、Ⅴ级：应在预防性服药后手术矫整。

2．外科治疗 膀胱输尿管反流外科治疗方法多为整形手术。

【预后】

原发性膀胱输尿管反流为先天性疾病，随着年龄逐渐增大和发育的逐渐成熟，膀胱输尿管反流逐渐消失。很多生长中的小儿Ⅰ～Ⅲ级反流可自愈，Ⅴ级则难自愈。

第七节 肾小管酸中毒

肾小管酸中毒（renal tubular acidosis，RTA）是由于近端肾小管对HCO_3^-重吸收障碍和（或）远端肾小管排泌H^+障碍所致的一组临床综合征。其主要表现为：①慢性高氯性酸中毒；②电解质紊乱；③肾性骨病；④尿路症状等。特发性者为先天缺陷，多有家族史，早期无肾小球功能障碍；继发性者可见于许多肾脏和全身疾病。

肾小管酸中毒一般分为4 个临床类型：①远端肾小管酸中毒（RTA－Ⅰ）；②近端肾小管酸中毒（RTA-Ⅱ）；③混合型或Ⅲ型肾小管酸中毒（RTA-Ⅲ）；④高钾型肾小管酸中毒（RTA-Ⅳ）。

一、远端肾小管酸中毒（Ⅰ型）

远端肾小管酸中毒（distal renal tubular acidosis，dRTA）是由于远端肾小管排泌H^+障碍，尿NH_4^+及可滴定酸排出减少所致。

【病因】

Ⅰ型肾小管酸中毒有原发性和继发性，原发者见于先天性肾小管功能缺陷，多为常染色体显性遗传，也有隐性遗传和特发病例。继发者可见于很多疾病，如肾盂肾炎、特发性高γ-球蛋白血症、干燥综合征、原发性胆汁性肝硬化、系统性红斑狼疮、纤维素性肺泡炎、甲状旁腺机能亢进、甲状腺机能亢进、维生素D中毒、特发性高钙尿症、肝豆状核变性、药物性或中毒性肾病、肾髓质囊性病、珠蛋白生成障碍性贫血、碳酸酐酶缺乏症等。

【发病机制】

由于原发性或继发性原因导致远端肾小管排泌H^+和维持小管腔液-管周间H^+梯度功能障碍，使尿液酸化功能障碍，尿pH>6，净酸排泄减少。正常情况下远曲小管HCO_3^-重吸收很少，排泌的H^+主要与管腔液中Na_2HPO_4交换Na^+，形成NaH_2PO_4，与NH_3结合形成NH_4^+。$H_2PO_4^-$与NH_4^+不能弥散至细胞内，因此产生较陡峭的小管腔液-管周间H^+梯度。Ⅰ型肾小管酸中毒患者不能形成或维持这个梯度，故使H^+蓄积，而体内HCO_3^-储备下降，血液中Cl^-代偿性增高，发生高氯性酸中毒。由于泌H^+障碍，Na^+-H^+交换减少，必然导致Na^+-K^+交换增加，大量K^+、Na^+被排出体外，造成低钾、低钠血症，患者由于长期处于酸中毒状态，致使骨质脱钙、骨骼软化而变形，骨质游离出的钙可导致肾钙化或尿路结石。

【临床表现】

1．原发性病例 可在出生后即有临床表现。

2．慢性代谢性酸中毒 患儿表现为厌食、恶心、呕吐、腹泻、便秘、生长发育迟缓。尿pH >6。

3．电解质紊乱 主要为高氯血症和低钾血症，患者出现全身肌无力和周期性瘫痪。

4．骨病 常表现为软骨病或佝偻病，出牙延迟或牙齿早脱，维生素D治疗效果差。患者常有骨痛和骨折，小儿可有骨畸形和侏儒等。

5．尿路症状 由于肾结石和肾钙化，患儿可有血尿、尿痛等表现，易导致继发感染与梗阻性肾病。肾脏浓缩功能受损时，患者还常有多饮、多尿、烦渴等症状。

【实验室检查】

1．血液生化检查 ①血浆pH值、[HCO_3^-]或CO_2结合力降低；②血氯升高，血钾、血钠降低，血钙和血磷偏低，阴离子间隙正常；③血ALP升高。

2．尿液检查 ①尿比重低；②尿pH>6；③尿钠、钾、钙、磷增加；④尿氨显著减少。

3．HCO_3^-排泄分数（FE HCO_3^-）正常值<5％。方法：从每日口服碳酸氢钠2～10 mmol/kg起，逐日增加剂量至酸中毒纠正，然后测定血和尿中[HCO_3^-]和肌酐（Cr），按下列公式计算：

$$FE\ HCO_3^- =（尿[HCO_3^-]/血[HCO_3^-]）÷（尿Cr/血Cr）\times 100$$

4．NH_4Cl负荷试验： 口服NH_4Cl 0.1 g/kg，1 小时内服完，3～8 小时内收集血和尿液，测量血[HCO_3^-]和尿pH值，当血[HCO_3^-]降至20 mmol/L以下时，尿pH>6，具有诊断价值。尿pH<5.5，则可排除本病。NH_4Cl负荷试验对明显酸中毒者不宜应用。

5．肾功能检查 早期为肾小管功能降低。待肾结石、肾钙化导致梗阻性肾病时，可出现肾小球滤过率下降，血肌酐和BUN升高。

6．X线检查 骨骼显示骨密度普遍降低和佝偻病表现，可见陈旧性骨折。腹部平片可见泌尿系结石影和肾钙化。

【诊断与鉴别诊断】

根据以上典型临床表现，排除其他原因所致的代谢性酸中毒，尿pH>6 者，即可诊断远端肾小管酸中毒，确定诊断应具有：①即使在严重酸中毒时，尿pH也不会低于5.5；②有显著的钙、磷代谢紊乱及骨骼改变；③尿铵显著降低；④FE HCO_3^-<5％；⑤氯化铵负荷试验阳性。

应与各种继发性远端肾小管酸中毒相鉴别。

【治疗】

1．纠正酸中毒 儿童有6％～15％的碳酸氢盐从肾脏丢失（在成人<5％），故可给予2.5～7 mmol/（kg·d）的碱性药物。常用口服碳酸氢钠或用复方枸橼酸溶液（Shohl 液，含枸橼酸140 g，枸橼酸钠98 g，加水1000 ml），每1 ml Shohl液相当于1 mmol的碳酸氢钠盐。开始剂量2～4 mmol/（kg·d），最大可用至5～14 mmol/（kg·d），直至酸中毒纠正。

2．纠正电解质紊乱 低钾血症可服10％枸橼酸钾0.5～1 mmol/（kg·d），每日3 次。不宜用氯化钾，以免加重高氯血症。

3．肾性骨病的治疗 可用维生素D、钙剂。维生素D剂量5000～10000 IU/d。但应注意：①从小剂量开始，缓慢增量；②监测血药浓度及血钙、尿钙浓度及时调整剂量，防止高钙血症的发生。

4．利尿剂 噻嗪类利尿剂可减少尿钙排泄，促进钙回吸收，防止钙在肾内沉积。如氢氯噻嗪1～3 mg/（kg·d），分3 次口服。

5．补充营养，保证入量，控制感染及原发疾病的治疗均为非常重要的措施。

【预后】

如早期发现，长期治疗，防止肾钙化及骨骼畸形的发生，预后良好，甚至可达正常的生长发育水平。有些患者可自行缓解，但也有部分患者可发展为慢性肾衰竭而死亡。

二、近端肾小管酸中毒（Ⅱ型）

近端肾小管酸中毒（proximal renal tubular acidosis，pRTA）是由于近端肾小管重吸收HCO_3^-

功能障碍所致。

【病因】

Ⅱ型肾小管酸中毒病因亦可分为原发性和继发性。①原发性多为常染色体显性遗传，亦可与隐性遗传和X-连锁遗传有关，多见于男性，部分为散发性病例；②继发性者可继发于重金属盐中毒、过期四环素中毒、甲状旁腺功能亢进、高球蛋白血症、半乳糖血症、胱氨酸尿症、肝豆状核变性、干燥综合征、肾髓质囊性病变、多发性骨髓瘤等。

【发病机制】

HCO_3^-回吸收障碍的机制尚未明确，可能与下列因素有关：①近端肾小管管腔中碳酸酐酶功能障碍，影响HCO_3^-分解成CO_2和H_2O，从而使近端肾小管分泌的H^+与腔液中HCO_3^-结合减少；②氢离子分泌泵障碍；③近端肾小管H^+排泌的调节异常；④H^+-K^+-ATP酶缺陷。

患儿肾小管HCO_3^-阈值一般为15～18 mmol/L（正常21～25 mmol/L），显著低于正常阈值，故即使血液HCO_3^-浓度低于21 mmol/L，亦有大量的HCO_3^-由尿中丢失，此时患儿产生酸中毒而其尿液呈碱性。由于其远端肾小管泌H^+功能正常，故当患儿HCO_3^-下降至15～18 mmol/L，尿HCO_3^-丢失减少，尿液酸化正常，故尿pH 可低于5.5。补碱后尿中排出大量碳酸氢盐。远端肾小管K^+-Na^+交换增多，可导致低钾血症。

【临床表现】

本型多见于男性。症状与Ⅰ型肾小管酸中毒相似，但较轻，其特点为：①生长发育落后，但大多数无严重的骨骼畸形，肾结石、肾钙化少见；②明显的低钾表现；③高氯性代谢性酸中毒；④可同时有其他近端肾小管功能障碍的表现，患儿常有多尿、脱水、烦渴症状；⑤少数病例只有尿的表现，而无代谢性酸中毒，即呈不完全型，但可进一步发展为完全型。

【实验検查】

1．血液生化检查 ①血pH值、HCO_3^-或CO_2结合力降低；②血氯显著升高，血钾显著降低，阴离子间隙可正常。

2．尿液检查 ①尿比重和渗透压降低；②尿pH>6，当酸中毒加重，血HCO_3^-<16 mmol/L时，尿pH<5.5。

3．HCO_3^-排泄分数（FE HCO_3^-）>15％。

4．氯化铵负荷试验：尿pH值<5.5。

【诊断与鉴别诊断】

在临床上具有多饮、多尿、恶心、呕吐和生长迟缓，血液检查具有持续性低钾高氯性代谢性酸中毒特征者应考虑近端肾小管酸中毒，确定诊断应具有：①当血[HCO_3^-]<16 mmol/L时，尿pH<5.5；②FE HCO_3^->15％；③尿钙不高，临床无明显骨骼畸形、肾结石和肾钙化；④氯化铵试验阴性。

当患儿伴有其他近端肾小管功能障碍时须注意与下列疾病相鉴别：①原发性Fanconi综合征；②胱氨酸尿；③肝豆状核变性；④毒物或药物中毒等引起的继发性肾小管酸中毒。

【治疗】

1．纠正酸中毒 因儿童肾HCO_3^-阈值比成人低，故患儿尿中HCO_3^-丢失更多，治疗所需碱较远端肾小管酸中毒为大，其剂量约10～15 mmol/（kg・d），给予碳酸氢钠或复方枸橼酸溶液口服。

2．纠正低钾血症。

3．重症者可予低钠饮食并加用氢氯噻嗪，可减少尿HCO_3^-排出，促进HCO_3^-重吸收。

【预后】

本型预后较好，多数患儿能随年龄增长而自行缓解。

（徐　虹）

第八节 溶血尿毒综合征

溶血尿毒综合征（hemolytic uremic syndrome，HUS），是由多种病因引起血管内溶血的微血管病，临床以溶血性贫血、血小板减少和肾衰竭为特点。本病好发于婴幼儿和学龄儿童，是小儿急性肾衰竭常见的原因之一。本病可分为典型和非典型两型，典型病例常有前驱胃肠道症状，非典型病例多有家族史，且易复发。本病死亡率高，近年来采用血浆转换和透析等综合疗法，病死率已明显下降。

【病因及分型】

本病的确切病因尚不清楚，目前较公认的分型有：

1．腹泻后溶血尿毒综合征（post-diarrhea HUS，D+HUS） 占全部病例的90％左右，又称典型溶血尿毒综合征。本病与产生螺旋毒素（verotoxin，VT）的细菌有关，如致病性大肠杆菌O_{157}：H_7、O_{26}、O_{121}、O_{145}等株及志贺痢疾杆菌Ⅰ型有关。75%的病例与致病性大肠杆菌O_{157}：H_7感染有关。该病菌寄生于家畜的肠道，常通过未熟的肉类和未经巴氏消毒的牛奶播散。

2．无腹泻溶血尿毒综合征（non-diarrhea HUS，D-HUS）约占10％的病例，又称非典型溶血尿毒综合征。常与以下因素有关：

（1）感染：包括细菌感染（肺炎球菌、空肠弯曲菌、伤寒杆菌、假单胞菌属、耶辛那菌、类杆菌等）和病毒感染（流感、EB病毒、柯萨奇病毒、埃可病毒、人类免疫缺陷病毒等）。

（2）药物：使用环孢菌素、丝裂菌素、光辉霉素、干扰素诱导剂等。

（3）其他：系统性红斑狼疮、肿瘤、恶性高血压、器官移植等。有家族中同患溶血尿毒综合征的报道，为常染色体隐性或显性遗传。

【发病机制】

各种有害因素（包括螺旋毒素、神经氨酸酶、内毒素、细胞黏附因子、活性氧反应物质等）引起的血管内皮损伤是发病的始动因素。血小板内皮损伤引起的级联反应包括：中性粒细胞介导的炎症反应、内皮细胞受损释放的von willebrand因子介导血小板聚集、受损的内皮细胞合成前列环素（prostacyclin，PGI_2）减少、血小板聚集释放血栓素引起血管收缩、血管内微血栓形成。

上述病理过程中，血小板大量消耗，临床上出现血小板减少；小血管腔内血栓形成，红细胞通过病变部位时受机械变形作用发生溶血性贫血；肾脏入球小动脉和肾小球毛细血管内皮细胞受累，导致内皮细胞肿胀、血管腔狭窄、血小板聚集、纤维素沉积、血栓形成，最终导致肾小球滤过率下降，临床出现少尿、无尿、急性肾衰竭等一系列表现。

【病理】

以多脏器微血管病变，微血栓形成为特点。肾脏是主要的受累器官。急性期肾小球内皮细胞肿胀，内皮下纤维素沉积，毛细血管壁增厚，肿胀的内皮细胞与基底膜分离可呈双轨样改变。毛细血管腔狭窄，可见红细胞碎片、血小板及微血栓形成。系膜区纤维蛋白沉积，系膜区扩大，系膜细胞无明显增生。严重者可见小动脉血栓形成、肾皮质坏死、系膜溶解、肾小球缺血样改

变。偶有新月体形成。肾小管腔内常见透明管型和红细胞管型，可出现小管上皮坏死、萎缩。免疫荧光检查可见纤维蛋白原沿肾小球毛细血管壁及系膜区沉积，也可见IgM、补体C3、C1q、备解素沉积。电镜下可见内皮细胞增生、肿胀，内皮和基底膜之间分离形成内皮下间隙，其间充以细微纤维、脂质红细胞碎片、血小板，沿内皮细胞侧可见新形成的薄层基底膜，上皮细胞足突融合。

【临床表现】

主要发生于婴幼儿和儿童，男性多见。散发多见，少数地区呈暴发流行，国内以晚春及初夏为高峰。典型临床表现为：

1．前驱症状 近90%的患者有前驱症状，大多为胃肠炎表现，如腹痛、腹泻、呕吐及食欲不振，伴中度发热。腹泻可为严重血便，极似溃疡性结肠炎，少数病例以呼吸道感染症状为前驱症。前驱期约持续数天至2 周，其后常有一无症状间歇期。

2．溶血性贫血 在前驱期后5～10 天（可迟至数周）突然发病，以溶血性贫血和出血为突出表现。患儿突然面色苍白、黄疸（约占15%～30%），头昏乏力，皮肤黏膜出血、呕血、便血或血尿，常有部分患者出现贫血性心力衰竭及水肿，可有肝脾大、皮肤瘀斑及皮下血肿等症。

3．急性肾衰竭 与贫血几乎同时发生，少尿或无尿，水肿，血压增高，出现尿毒症症状、水电解质紊乱和酸中毒。

4．其它 尚可有中枢神经系统症状，如头痛、嗜睡、性格异常、抽搐、昏迷、共济失调等。

【实验室检查】

1．血液学改变 血红蛋白下降明显，可低至30～50 g/L，末梢血网织红细胞明显增高，血涂片可见红细胞形态异常，呈三角形、芒刺形、盔甲形及红细胞碎片等。外周血白细胞数大多增高，可达（20～30）×10^9/L，血小板减少见于90%的患者，可低至10×10^9/L，持续1～2 周后逐渐升高。骨髓检查见巨核细胞数目增多、形态正常，未能测出血小板抗体，Coomb试验阴性。

2．凝血与纤溶 早期纤维蛋白原稍降低、纤维蛋白降解产物增加，因子Ⅱ、Ⅷ、Ⅸ及Ⅹ减少，凝血酶原时间延长，一般数天内恢复正常，后期纤维蛋白原略升高。弥散性血管内凝血（DIC）表现者罕见。

3．血液生化改变 血清总胆红素增高，以间接胆红素升高为主，血浆结合珠蛋白降低，血浆乳酸脱氢酶（LDH）及其同工酶（丙酮酸脱氢酶）均升高。超氧化物歧化酶（SOD）降低及红细胞膜脂质过氧化产物丙乙醛（MDA）增高提示自身红细胞抗氧化能力降低。少尿期血尿素氮、肌酐增高、血钾增高等电解质紊乱及代谢性酸中毒，血尿酸增高。

4．尿常规 可见不同程度的血尿、红细胞碎片，严重溶血者可有血红蛋白尿，还可有不同程度的蛋白尿、白细胞及管型。

5．肾活体组织检查 有助于明确诊断并可估计预后，因为急性期有血小板减少和出血倾向，宜在急性期过后病情缓解时进行。肾活体组织病理检查表现为肾脏微血管病变、微血管栓塞。

【诊断和鉴别诊断】

典型溶血尿毒综合征病例诊断不难，凡有前驱症状后突然出现溶血性贫血、血小板减少及急性肾衰竭三大特征者应考虑本病的诊断。症状不典型者可做肾活体组织检查，如发现显著的小血管病变和血栓形成有助诊断。本病应与血栓性血小板减少性紫癜（thromboic thrombocytopenic purpura，TTP）相鉴别。HUS伴有发热及中枢神经系统症状者不易与TTP相鉴别，后者中枢

神经系统损害较HUS多见且较重，而肾损害较HUS轻，TTP主要见于成年女性，而HUS主要见于小儿，特别是婴幼儿。另外，还需与免疫性溶血性贫血、特发性血小板减少症、败血症、阵发性睡眠性血红蛋白尿（paroximal nocturnal hemoglobinuria，PNH）、急性肾小球肾炎、各种原因所致的急性肾衰竭等相鉴别。

【治疗】

本病无特殊治疗，主要是早期诊断，及时纠正水、电解质平衡紊乱，控制高血压，尽早进行腹膜透析和血液透析是治疗的关键。

1．一般治疗 包括抗感染、补充营养、维持水电解质平衡等。

2．急性肾衰竭的治疗 治疗原则与方法与一般急性肾衰竭治疗相似（详见急性肾衰竭节），除强调严格控制入水量、积极治疗高血压及补充营养、维持水电解质平衡外，提倡尽早进行透析治疗。

3．纠正贫血 一般主张尽可能少输血，以免加重微血管内凝血。当血红蛋白低于60 g/L是时，应输新鲜洗涤红细胞2.5～5 ml/（kg・次），于2～4 小时内缓慢输入。必要时可隔6～12 小时重复输入。

4．抗凝治疗 仅适用于早期有高凝状态的严重病例。包括肝素、双嘧达莫、阿司匹林等。

5．输注新鲜冻血浆 以恢复前列环素（PGI_2）活性。开始剂量为每次30～40 ml/kg，以后改为每次15~20 ml/kg，直到血小板数升至正常或>150×10^9/L，溶血停止。因肺炎链球菌产生的唾液酸酶可使红细胞膜、血小板膜和肾小球内皮细胞膜上的T-F（Thomsen-Friedenreich）抗原暴露，正常成人血浆中含有抗T-F的抗体，会与暴露的T-F抗原发生反应，导致红细胞溶解、血小板减少和血栓性微血管病，因此，由肺炎球菌所致的溶血尿毒综合征患者禁输血浆。

6．血浆置换疗法 与新鲜冰冻血浆联合使用，疗效较好，可用于严重病例，以补充刺激PGI_2生成所需的血浆因子或去除血浆中抑制PGI_2的物质。

7．去纤维肽 系一种多脱氧核糖核酸盐，具有抗血栓形成和纤维蛋白溶解活性，促进PGI_2合成，用药后可迅速改善甚至消除神经症状、凝血异常现象，高血压得到有效控制，肾功能也可部分或完全恢复。用法：10 mg/（kg・d）静脉滴入1～2 周后，可酌情改口服维持1～6 个月。

8．肾移植 部分患者对上述治疗反应不佳，而逐渐出现慢性肾衰竭，此时可考虑行肾脏移植手术，但肾移植后可再发本病。

【预后】

20 世纪60 年代本病的急性期病死率达50％以上，近几年随着治疗方法的改进，病死率可降至5％～10％以下。溶血尿毒综合征的预后主要取决于肾脏损伤的程度，偶可由于神经系统严重损害或因少尿、严重贫血、电解质紊乱、高血压诱发充血性心力衰竭、心跳骤停而致死。影响预后的因素包括：①年龄及性别：婴幼儿预后好，男性较女性预后好；②类型：流行型较散发型为好；③肾损害重者预后差；④伴中枢神经系统受累者预后差；⑤反复发作者及有家族倾向者预后差；⑥血红蛋白值和白细胞计数：高Hb水平（约100 g/L），白细胞数大于20.0×10^9/L者预后不佳；⑦治疗方法：早期诊断，正确治疗、及早进行血浆置换和透析是降低急性期溶血尿毒综合征病死率的关键。部分溶血尿毒综合征患者可在病情缓解后演变为慢性肾功能不全甚至需长期透析维持生命。

第九节 血 尿

血尿（hematuria）是儿科泌尿系统疾病常见的症状。正常人尿中红细胞仅为0～2 个/HPF，

血尿是指尿液中红细胞数超过正常，分为镜下血尿和肉眼血尿，前者仅在显微镜下发现红细胞增多。取新鲜清洁中段尿（以清晨为好）10 ml，以1500转/分离心沉淀5 分钟，弃上清液，将管底沉渣0.2 ml混匀后涂片镜检，高倍镜下RBC>3 个/HPF、或尿沉渣红细胞计数>8×10^6/L（8000 个/ml）即为镜下血尿。肉眼即能见尿呈“洗肉水”色或血样称为“肉眼血尿”。一般当尿红细胞>2.5×10^9/L,（1000 ml尿中含0.5 ml 血液）即可出现肉眼血尿，肉眼血尿的颜色与尿液的酸碱度有关，中性或弱碱性尿颜色鲜红或呈洗肉水样，酸性尿呈浓茶样或烟灰水样。

目前常用尿液分析仪（试纸法）检测血尿，其原理是利用血红蛋白的氧化性与试纸的呈色反应来进行半定量分析，但当尿中存在还原物质（如维生素C>50 mg/L），可呈假阴性。而尿中存在游离血红蛋白、肌红蛋白和过氧化酶等物质时可呈假阳性。健康儿童尿分析可有潜血阳性，且尿潜血与镜检往往不平行，诊断血尿应以镜检为准。

【病因与临床分类】

引起血尿的原因很多，各种致病因素引起的肾小球基膜完整性受损或通透性增加、尿道黏膜的损伤、肾小球毛细血管腔内压增高、全身凝血机制障碍等均可导致血尿。

1．肾脏疾病

（1）各种原发性肾小球病：急、慢性肾小球肾炎，遗传性肾炎，薄基膜肾病，IgA肾病，肺出血-肾炎综合征等。

（2）感染：肾结核，肾盂肾炎。

（3）畸形：肾血管畸形，先天性多囊肾，游走肾，肾下垂，肾盂积水等。

（4）肿瘤：肾胚胎瘤，肾盏血管肿瘤等。

（5）肾血管病变：肾静脉血栓形成，左肾静脉受压综合征（胡桃夹现象）。

（6）损伤：肾挫伤及其他损伤。

（7）药物：肾毒性药物如氨基糖甙类抗生素、杆菌肽、水杨酸制剂、磺胺类、苯妥英钠、环磷酰胺等均可引起肾损害产生血尿。

2．尿路疾病

（1）感染：膀胱炎，尿道炎，结核。

（2）结石：输尿管结石，膀胱结石。

（3）肿瘤，息肉，憩室，异物等。

3．全身性疾病

（1）出血性疾病：弥散性血管内凝血，血小板减少性紫癜，血友病，新生儿自然出血症，再生障碍性贫血，白血病等。

（2）心血管疾病：充血性心力衰竭，感染性心内膜炎。

（3）感染性疾病：猩红热，伤寒，流行性出血热，传染性单核细胞增多症，暴发型流脑以及肺炎支原体、结核杆菌、肝炎病毒、钩端螺旋体等所致感染后肾炎。

（4）风湿性疾病：系统性红斑狼疮，过敏性紫癜，结节性多动脉炎，风湿性肾炎。

（5）营养性疾病：维生素C缺乏症，维生素K缺乏症。

（6）过敏性疾病：饮食过敏如牛奶或菠萝过敏。

（7）其他疾病：如遗传性毛细血管扩张症，剧烈运动引起的一过性血尿，特发性高钙尿症等。

【诊断与鉴别诊断】

1．真性血尿与假性血尿 血尿的诊断首先要排除以下能产生假性血尿的情况：①摄入含大量人造色素（如苯胺）的食物、蜂蜜或药物（如大黄、利福平、苯妥因钠）等引起红色尿；②

血红蛋白尿或肌红蛋白尿；③卟啉尿；④初生新生儿尿内之尿酸盐可使尿布呈红色。但以上尿检查均无红细胞可资鉴别；⑤血便或月经血污染。

2．肾小球性与非肾小球性血尿 血尿确定后，首先判断血尿的来源，然后确定原发病因。目前常用方法有：①尿沉渣红细胞形态学检查：若以异形红细胞为主则提示为肾小球性血尿（相差显微镜下>30％）。以均一形为主者则提示非肾小球性血尿，血尿来源于肾盂、肾盏、输尿管、膀胱或尿道，多见于泌尿道感染、结石、结核、肿瘤、创伤等。影响尿红细胞形态的因素有：年龄、尿比重，尿pH，利尿剂的应用，泌尿系感染，肉眼血尿发作。②尿中红细胞平均体积测定：若MCV<72 fl且呈小细胞分布，则说明血尿来源于肾小球，此法敏感性为95％，特异性为96％，且可克服检测者主观的误差。③尿沉渣检查见到红细胞管型和肾小管上皮细胞，表明血尿为肾实质性。若镜下血尿时，尿蛋白定量>500 mg/24h；肉眼血尿时，尿蛋白>990 mg/24h，或>660 mg/L，则多提示肾小球疾病。④尿红细胞电泳：肾小球性者为20.64 秒±1.72 秒，非肾小球性者为27.27 秒±1.66 秒。

3．肾小球性血尿诊断步骤

（1）临床资料分析：肾小球性血尿的鉴别诊断应注意特别详细地询问血尿的伴随症状及体征。①伴水肿、高血压，尿液中发现管型和蛋白尿，应考虑原发性或继发性肾小球疾病；②新近有皮肤感染、咽喉炎后出现血尿，首先要考虑急性链球菌感染后肾小球肾炎，其次为IgA肾病；③伴有夜尿增多，贫血显著时应考虑慢性肾小球肾炎；④伴有听力异常，应考虑Alport综合征；⑤有血尿家族史，应考虑薄基膜病；⑥伴感觉异常，应考虑Fabry病；⑦伴肺出血应想到肺出血-肾炎综合征；⑧伴有紫癜，应考虑紫癜性肾炎；⑨伴有高度水肿和大量蛋白尿应考虑肾病综合征。

（2）血和尿生化分析：①血ASO升高伴有C3下降应考虑急性链球菌感染后肾炎；②伴血HBsAg（+）和（或）HBeAg（+），肾组织中有乙肝病毒抗原沉积，可诊断为乙肝病毒相关性肾炎；③血清补体持续性下降，考虑原发性膜增生性肾炎、狼疮性肾炎、乙肝病毒相关性肾炎、慢性肾小球肾炎；④ANA、Anti-dsDNA、ANCA等阳性应考虑狼疮性肾炎；⑤血清IgA增高，提示有IgA肾病可能；IgG、IgM、IgA均增高，可见于狼疮性肾炎、慢性肾炎；⑥尿蛋白成分分析中以大分子蛋白尿为主，多见于急、慢性肾小球肾炎及肾病综合征；小分子蛋白尿为主，提示间质性肾炎。

（3）肾活体组织检查分析：肾活体组织病理检查对血尿的病因诊断具有极为重要价值，如IgA肾病、薄基膜病、局灶节段性肾小球硬化、狼疮性肾炎、肝炎病毒相关性肾炎、Alport综合征等。

4．非肾小球性血尿诊断步骤

（1）尿三杯试验：第一杯红细胞增多为前尿道出血；第三杯红细胞增多则为膀胱基底部、前列腺、后尿道或精囊出血；三杯均有出血，则为膀胱颈以上部位出血。上尿路出血多呈暗棕色尿，无膀胱刺激征，有时可见血块。尿中出现血块通常为非肾小球性疾病。

（2）临床资料分析：①伴有尿频、尿急、尿痛，应考虑泌尿道感染，其次为肾结核；②伴有低热、盗汗、消瘦应考虑肾结核；③伴有皮肤黏膜出血应考虑出血性疾病；④伴有出血、溶血、循环障碍及血栓症状，应考虑DIC或溶血尿毒综合征；⑤伴有肾绞痛或活动后腰痛应考虑肾结石；⑥伴有外伤史应考虑泌尿系统外伤；⑦伴有肾区肿块应考虑肾肿瘤或肾静脉栓塞；⑧近期使用肾毒性药物，应考虑急性间质性肾炎；⑨无明显伴随症状时，应考虑左肾静脉受压综合征、特发性高钙尿症、肾微结石、肾盏乳头炎、肾小血管病及肾盂、尿路息肉、憩室。

（3）辅助检查分析：①两次尿培养阳性，尿菌落计数>10^5/ml，可诊断泌尿道感染。②尿

培养检出结核杆菌，对诊断肾结核有重要价值，并可通过3 次以上晨尿沉渣找抗酸杆菌，其阳性率为80％～90％，24 小时尿沉渣找抗酸杆菌，阳性率为70％。③全尿路X线平片检查在非肾小球性血尿病因诊断中非常重要，可及时发现泌尿系结石。对于尿酸结石，X线检查阴性者可采用B超检查。④对于怀疑上尿路病变者，可行静脉肾盂造影（IVP），IVP阴性而持续血尿者，应行B超或CT检查，以排除小的肾肿瘤、小结石、肾囊肿以及肾静脉血栓形成。若仍阴性者，可行肾活体组织检查。⑤左肾静脉受压综合征是非肾小球性血尿的常见原因，彩色Doppler检查可以确诊。⑥儿童特发性高钙尿症也是非肾小球性血尿的常见原因，24 小时尿钙测定>4 mg/kg或尿钙/尿肌酐>0.2，即可诊断。

第十节 急性肾衰竭

急性肾衰竭（acute renal failure，ARF）是多种原因引起的肾生理功能在短期内急剧下降或丧失的临床综合征，患儿体内代谢产物堆积，出现氮质血症、水及电解质紊乱和代谢性酸中毒。

【病因】

急性肾衰竭常见的病因可分为肾前性、肾实质性和肾后性三类。

1．肾前性肾衰竭 系指任何原因引起有效循环血容量降低，致使肾血流量不足、肾小球滤过率（GFR）显著降低所。

常见的原因包括：呕吐、腹泻和胃肠减压等胃肠道液体的大量丢失、大面积烧伤、手术或创伤出血等引起的绝对血容量不足；休克、低蛋白血症、严重心律失常、心包填塞和心力衰竭等引起的相对血容量不足。

2．肾实质性肾衰竭 亦称为肾性肾衰竭，系指各种肾实质病变所导致的肾衰竭，或由于肾前性肾衰竭未能及时去除病因、病情进一步发展所致。常见的原因包括：急性肾小管坏死（acute tubular necrosis，ATN）、急性肾小球肾炎、急性间质性肾炎、肾血管病变（血管炎、血管栓塞和弥散性血管内栓塞）、以及慢性肾脏疾患在某些诱因刺激下肾功能急剧衰退。

3．肾后性肾衰竭 各种原因所致的泌尿道梗阻引起的急性肾衰竭，如输尿管肾盂连接处狭窄、肾结石、肿瘤压迫、血块堵塞等。

【发病机制】

急性肾衰竭的发病机制十分复杂，本章着重讨论急性肾小管坏死的主要发病机制。

1．肾小管损伤 肾缺血或肾中毒时引起肾小管急性严重的损伤，小管上皮细胞变性、坏死和脱落、肾小管基膜断裂，一方面脱落的上皮细胞引起肾小管堵塞，造成管内压升高和小管扩张，致使肾小球有效滤过压降低和少尿；另一方面肾小管上皮细胞受损引起肾小管液回漏，导致肾间质水肿。

2．肾血流动力学改变 肾缺血和肾毒素能使肾素-血管紧张素系统活化，肾素和血管紧张素Ⅱ分泌增多、儿茶酚胺大量释放、TXA_2/PGI_2比例增加以及内皮素水平升高，均可导致肾血管持续收缩和肾小球入球动脉痉挛，引起肾缺血缺氧、肾小球毛细血管内皮细胞肿胀致使毛细血管腔变窄，肾血流量减少，GFR降低而导致急性肾衰竭。

3．缺血-再灌注肾损伤 肾缺血再灌注时，细胞内钙通道开放，钙离子内流造成细胞内钙超负荷；同时局部产生大量的氧自由基，可使肾小管细胞的损伤发展为不可逆性损伤。

4．非少尿型急性肾小管坏死的发病机制 非少尿型急性肾小管坏死的发生主要是由于肾单位受损轻重不一所致。另外，非少尿型急性肾小管坏死不同的肾单位肾血流灌注相差很大，部分肾单位血液灌注量几乎正常，无明显的血管收缩，血管阻力亦不高，而一些肾单位灌注量明

显减少，血管收缩和阻力增大。

【病理】

急性肾小管坏死肾脏病理改变：①肉眼检查肾脏体积增大、苍白色，剖面皮质肿胀、髓质呈暗红色；②光镜检查主要部位在近端小管直段，早期小管上皮细胞肿胀，脂肪变性和空泡变性；晚期小管上皮细胞可呈融合样坏死，细胞核浓缩，细胞破裂或溶解，形成裂隙和剥脱区基膜暴露或断裂，间质充血、水肿和炎性细胞浸润，有时可见肾小管上皮细胞再生，肾小球和肾小动脉则多无显著变化。近端肾小管刷状缘弥漫性消失、变薄和远端肾单位节段性管腔内管型形成是缺血型急性肾小管坏死常见的特征性病理改变。近端肾小管及远端肾单位局灶节段性斑块坏死和细胞脱落是中毒型急性肾小管坏死的病理特征。

【临床表现】

根据尿量减少与否，急性肾衰竭可分为少尿型和非少尿型。急性肾衰竭伴少尿或无尿表现者称为少尿型。非少尿型系指血尿素氮、血肌酐迅速升高，肌酐清除率迅速降低，而不伴有少尿表现。临床常见少尿型急性肾衰竭，临床过程分为三期：

1．少尿期 少尿期一般持续1～2 周，长者可达4～6 周，持续时间越长，肾损害越重。持续少尿大于15 天，或无尿大于10 天者，预后不良。少尿期的系统症状有：

（1）水钠潴留：患儿可表现为全身水肿、高血压、肺水肿、脑水肿和心力衰竭，有时因水潴留可出现稀释性低钠血症。

（2）电解质紊乱：常见高钾、低钠、低钙、高镁、高磷和低氯血症。

（3）代谢性酸中毒：表现为恶心、呕吐、疲乏、嗜睡、呼吸深快、食欲不振、甚至昏迷，血pH值降低。

（4）尿毒症：因肾排泄障碍使各种毒性物质在体内积聚所致。可出现全身各系统中毒症状。其严重程度与血中尿素氮及肌酐增高的浓度相一致。

1）消化系统：表现为食欲不振、恶心、呕吐和腹泻等，严重者出现消化道出血或黄疸，而消化道出血可加重氮质血症。

2）心血管系统：主要因水钠潴留所致，表现为高血压和心力衰竭，还可发生心律失常、心包炎等。

3）神经系统症状：可有嗜睡、神志混乱、焦虑不安、抽搐、昏迷和自主神经功能紊乱如多汗或皮肤干燥，还可表现为意识、行为、记忆、感觉、情感等多种功能障碍。

4）血液系统：急性肾衰竭常伴有正细胞正色素性贫血，贫血随肾功能恶化而加重，系由于红细胞生成减少、血管外溶血、血液稀释和消化道出血等原因所致。出血倾向（牙龈出血、鼻出血、皮肤瘀点及消化道出血）多因血小板减少、血小板功能异常和DIC引起。急性肾衰早期白细胞总数常增高，中性粒细胞比例也增高。

（5）感染：感染是急性肾衰竭最为常见的并发症，以呼吸道和尿路感染多见，致病菌以金黄色葡萄球菌和革兰阴性杆菌最多见。

2．利尿期 当急性肾衰竭患儿尿量逐渐增多，全身水肿减轻，24 小时尿量达250 ml/m^2以上时，即为利尿期。一般持续1～2 周（长者可达1 个月），此期由于大量排尿，可出现脱水、低钠和低钾血症。早期氮质血症持续甚至加重，后期肾功能逐渐恢复。

3．恢复期 利尿期后，肾功能改善，尿量恢复正常，血尿素氮和肌酐逐渐恢复正常，而肾浓缩功能需要数月才能恢复正常，少数患者遗留不可逆性的肾功能损害。此期患儿可表现为虚弱无力、消瘦、营养不良、贫血和免疫功能低下。

药物所致的急性肾小管坏死多为非少尿型急性肾衰竭，临床表现较少尿型急性肾衰症状

轻、并发症少、病死率低。

【实验室检查】

1．尿液检查 尿液检查有助于鉴别肾前性急性肾衰竭和肾实质性急性肾衰竭，详见表14-3。

2． 血生化检查 应注意监测电解质浓度变化及血肌酐和尿素氮。

3．肾影像学检查 多采用腹平片、超声波、CT、磁共振等检查有助于了解肾脏的大小、形态，血管及输尿管、膀胱有无梗阻，也可了解肾血流量、肾小球和肾小管的功能，使用造影剂可能加重肾损害，须慎用。

4． 肾活检 是对原因不明的急性肾衰竭可靠的诊断手段，可帮助诊断和评估预后。

【诊断和鉴别诊断】

当患儿尿量急剧减少、肾功能急剧恶化时，均应考虑急性肾衰竭的可能，而急性肾衰竭诊断一旦确定，须进一步鉴别是肾前性、肾性还是肾后性急性肾衰竭。

1． 诊断依据 ①尿量显著减少：出现少尿（每日尿量<250 ml/m^2）或无尿（每日尿量<50 ml/m^2）；②氮质血症：血清肌酐≥176 μmol/L，血尿素氮≥15 mmol/L，或每日血肌酐增加≥44 μmol/L，或血尿素氮增加≥3.57 mmol/L，有条件者测肾小球滤过率（如内生肌酐清除率）常每分钟≤30 ml/1.73m^2；③有酸中毒、水电解质紊乱等表现。无尿量减少为非少尿型急性肾衰竭。

2． 临床分期 如前所述。

3． 病因诊断

（1）肾前性和肾实质性急性肾衰竭的鉴别见表14-3。

（2）肾后性急性肾衰竭：泌尿系统影像学检查有助于发现导致尿路梗阻的病因。

表14-3 肾前性和肾性急性肾衰竭的鉴别

指　　标	肾　前　性	肾　　性
脱水征	有	无或有
尿沉渣	偶见透明管型、细颗粒管型	粗颗粒管型或红细胞管型
尿比重	>1.020	<1.010
尿渗透压	>500 mOsm/L	<350 mOsm/L
尿肌酐/血肌酐	>40	<20（常<5）
肾衰指数*	<1	>1
尿钠	<20 mmol/L	>40 mmol/L
滤过钠排泄分数△	<1%	>1%
中心静脉压	<50 mmH_2O	正常或增高
补液试验□	尿量增加	无效
利尿试验+	有效	无效

$$*肾衰指数 = \frac{尿钠(mmol/L)\times 血浆肌酐\ (\mu mol/L)}{尿肌酐\ (\mu mol/L)}$$

$$△滤过钠排泄分数 = \frac{尿钠(mmol/L)\times 血浆肌酐\ (\mu mol/L)}{血清钠\ (mmol/L)\times 尿肌酐\ (\mu mmol/L)}\times 100\%$$

□补液试验：用2：1等张液15～20 ml/kg快速输入（半小时内输完），2 小时尿量增加至6～10 ml/kg为肾前性少尿，尿量无增加刚可能为肾性肾衰。

+利尿试验：如补液后无反应可使用20%甘露醇0.2～0.3 mg/kg，在20～30 分钟内推注，2 小时尿量增加至6～10 ml/kg为有效，需继续补液改善循环；无反应者给呋塞米1～2 mg/kg，2 小时尿量增加至6～10 ml/kg为有效，若仍无改善，为肾性肾衰竭。对已有循环充血者，慎用甘露醇。

【治疗】

治疗原则是去除病因，积极治疗原发病，减轻症状，改善肾功能，防止并发症的发生。

1．少尿期的治疗

（1）去除病因和治疗原发病：肾前性急性肾衰竭应注意及时纠正全身循环血流动力学障碍，包括补液、输注血浆和白蛋白、控制感染等。避免接触肾毒性物质，严格掌握肾毒性抗生素的用药指征，并根据肾功能调节用药剂量，密切监测尿量和肾功能变化。

（2）饮食和营养：应选择高糖、低蛋白、富含维生素的食物，尽可能供给足够的能量。供给热量210～250 J/（kg •d），蛋白质0.5 g/（kg •d），应选择优质动物蛋白，脂肪占总热量30%～40％。

（3）控制水和钠摄入：坚持“量入为出”的原则，严格限制水、钠摄入，有透析支持则可适当放宽液体入量。每日液体量控制在：尿量+显性失水（呕吐、大便、引流量）+不显性失水-内生水。无发热患儿每日不显性失水为300 ml/m^2，体温每升高1℃，不显性失水增加75 ml/m^2；内生水在非高分解代谢状态为250～350 ml/m^2。所用液体均为非电解质液。髓袢利尿剂（呋塞米）对少尿型急性肾衰竭可短期试用。

（4）纠正代谢性酸中毒：轻、中度代谢性酸中毒一般无须处理。当血浆HCO_3^-<12 mmol/L或动脉血pH<7.2，可补充5％碳酸氢钠5 ml/kg，提高CO_2CP 5 mmol/L。纠正酸中毒时宜注意防治低钙性抽搐。

（5）纠正电解质紊乱：包括高钾血症、低钠血症、低钙血症和高磷血症的处理。

（6）透析治疗：凡上述保守治疗无效者，均应尽早进行透析。透析的指征：①严重水潴留，有肺水肿、脑水肿的倾向；②血钾≥6.5 mmol/L或心电图有高钾表现；③严重酸中毒，血浆HCO_3^-<12 mmol/L 或动脉血pH<7.2；④严重氮质血症，血浆尿素氮>28.6 mmol/L，或血浆肌酐>707.2 μmol/L，特别是高分解代谢的患儿。目前透析指征有放宽的趋势。透析的方法包括腹膜透析、血液透析和连续动静脉血液滤过三种技术，儿童、尤其是婴幼儿以腹膜透析为常用。

2．利尿期的治疗 利尿期早期，肾小管功能和GFR尚未恢复，血肌酐、尿素氮、血钾和酸中毒仍继续升高，伴随着多尿，还可出现低钾和低钠血症等电解质紊乱，故应注意监测尿量、电解质和血压变化，及时纠正水、电解质紊乱，当血浆肌酐接近正常水平时，应增加饮食中蛋白质摄入量。

3．恢复期的治疗 此期肾功能日趋恢复正常，但可遗留营养不良、贫血和免疫力低下，少数患者遗留不可逆性肾功能损害，应注意休息和加强营养，防治感染。

【预后】

随着透析的广泛开展，急性肾衰竭的病死率已有明显降低。急性肾衰竭的预后与原发病性质、肾脏损害程度、少尿持续时间长短、早期诊断和早期治疗与否、透析与否和有无并发症等有直接关系。

（黄松明）

第十五章 造血系统疾病

第一节 小儿造血和血象特点

一、造血特点

（一）胚胎期造血

根据造血组织发育和造血部位发生的先后，可将此期分为三个不同的阶段。

1．中胚叶造血期 在胚胎第3 周开始出现卵黄囊造血，之后在中胚叶组织中出现广泛的原始造血成分，其中主要是原始的有核红细胞。在胚胎第6 周后，中胚叶造血开始减退。

2．肝脾造血期 在胚胎第6～8 周时，肝脏出现活动的造血组织，并成为胎儿中期的主要造血部位。胎儿期4～5 个月时达高峰，6 个月后逐渐减退。胎肝造血主要产生有核红细胞，也可产生少量粒细胞和巨核细胞。

约于胚胎第8 周脾脏开始造血，以生成红细胞占优势，稍后粒系造血也相当活跃，至12 周时出现淋巴细胞和单核细胞。胎儿5 个月之后，脾脏造红细胞和粒细胞的功能逐渐减退，至出生时成为终生造血淋巴器官。

胸腺是中枢淋巴器官，胚胎第6～7 周已出现胸腺，并开始生成淋巴细胞。来源于卵黄囊、肝脏或骨髓的淋巴干细胞在胸腺中经包括胸腺素在内的微环境中诱导分化为具有细胞免疫功能的前T细胞和成熟的T淋巴细胞，并迁移至周围淋巴组织，在相应的微环境中分化为不同的亚群，这种功能维持终生。此外，胚胎期胸腺还有短暂的生成红细胞和粒细胞功能。

自胚胎第11 周淋巴结开始生成淋巴细胞，从此，淋巴结成为终生造淋巴细胞和浆细胞的器官。胎儿期淋巴结亦有短暂的红系造血功能。

3．骨髓造血期 胚胎第6 周开始出现骨髓，但至胎儿4 个月时才开始造血活动，并迅速成为主要的造血器官，直至出生2～5 周后成为唯一的造血场所。

（二）生后造血

1．骨髓造血 出生后主要是骨髓造血。婴幼儿期所有骨髓均为红骨髓，全部参与造血，以满足生长发育的需要。5～7 岁开始，脂肪组织（黄髓）逐渐代替长骨中的造血组织，因此年长儿和成人红骨髓仅限于肋骨、胸骨、脊椎、骨盆、颅骨、锁骨和肩胛骨，但黄髓仍有潜在的造血功能，当造血需要增加时，它可转变为红髓而恢复造血功能。小儿在出生后头几年缺少黄髓，故造血代偿潜力小，如果造血需要增加，就会出现髓外造血。

2．骨髓外造血 在正常情况下，骨髓外造血极少。出生后，尤其在婴儿期，当发生感染性贫血或溶血性贫血等造血需要增加时，肝、脾和淋巴结可随时适应需要，恢复到胎儿时的造血状态，出现肝、脾、淋巴结肿大。同时外周血中可出现有核红细胞或（和）幼稚中性粒细胞。这是小儿造血器官的一种特殊反应，称为“骨髓外造血”，感染及贫血纠正后即恢复正常。

二、血象特点

不同年龄小儿的血象有所不同。

（一）红细胞数和血红蛋白量

由于胎儿期处于相对缺氧状态，红细胞生成素合成增加，故红细胞数和血红蛋白量较高，出生时红细胞数约5.0～7.0×10^{12}/L，血红蛋白量约150～220 g/L。未成熟儿与足月儿基本相等，少数可稍低。生后6～12 小时因进食较少和不显性失水，其红细胞数和血红蛋白量往往比出生时高些。生后随着自主呼吸的建立，血氧含量增加，红细胞生成素减少，骨髓造血功能暂时性降低，网织红细胞减少；胎儿红细胞寿命较短，且破坏较多（生理性溶血）；婴儿生长发育迅速，循环血量迅速增加等因素，红细胞数和血红蛋白量逐渐降低，至2～3 个月时（早产儿较早）红细胞数降至3.0×10^{12}/L、血红蛋白量降至100 g/L左右，出现轻度贫血，称为“生理性贫血”。“生理性贫血”呈自限性，3 个月以后，红细胞数和血红蛋白量又缓慢增加，于12 岁时达成人水平。此外，初生时外周血中可见到少量有核红细胞，生后1 周内消失。

网织红细胞数在初生3 天内约为0.04～0.06 ，于生后第7 天迅速下降至0.02 以下，并维持在较低水平，约0.003 ，以后随生理性贫血恢复而短暂上升，婴儿期以后约与成人相同。

（二）白细胞数与分类

初生时白细胞总数15～20×10^9/L，生后6～12 小时达21～28×10^9/L，然后逐渐下降，1 周时平均为12×10^9/L，婴儿期白细胞数维持在10×10^9/L左右，8 岁以后接近成人水平。

白细胞分类主要是中性粒细胞与淋巴细胞比例的变化。出生时中性粒细胞约占0.65，淋巴细胞约占0.30。随着白细胞总数的下降，中性粒细胞比例也相应下降，生后4～6 天时两者比例约相等；至1～2岁时淋巴细胞约占0.60，中性粒细胞约占0.35，之后中性粒细胞比例逐渐上升，至4～6 岁时两者比例又相等；以后白细胞分类与成人相似。此外，初生儿外周血中也可出现少量幼稚中性粒细胞，但在数天内即消失。

（三）血小板数与分类

血小板数与成人相似，约为150～300×10^9U/L。

（四）血红蛋白种类

血红蛋白分子由两对多肽链组成，构成血红蛋白分子的多肽链共有6 种，分别称为α、β、γ、δ、ε和ζ链，不同的血红蛋白分子是由不同的多肽链组成。正常情况下可有6 种不同的血红蛋白分子：胚胎期的血红蛋白为Gower1（$\zeta_2\varepsilon_2$）、Gower2（$\alpha_2\varepsilon_2$）和Portland（$\zeta_2\gamma_2$）；胎儿期的胎儿血红蛋白（HbF，$\alpha_2\gamma_2$）；成人血红蛋白分为HbA（$\alpha_2\beta_2$）和HbA_2（$\alpha_2\gamma_2$）两种。

血红蛋白Gowerl、Gower2 和Portland在胚胎12 周时消失，并为HbF所代替。胎儿6 个月时HbF占0.90，而HbA仅占0.05～0.10；以后HbA合成逐渐增加，至出生时HbF占0.70，HbA约占0.30，HbA_2<0.01。出生后HbF迅速为HbA所代替，1 岁时HbF不超过0.05，2 岁时HbF不超过0.02。成人的HbA约占0.95，HbA_2占0.02～0.03，HbF不超过0.02。

（五）血容量

小儿血容量相对较成人多，新生儿血容量约占体重的10％，平均300 ml；儿童约占体重的8％～10％；成人血容量约占体重的6％～8％。

（盛光耀）

第二节 小儿贫血概述

贫血是指外周血中单位容积内的红细胞数、血红蛋白量或红细胞压积低于正常。婴儿和儿

童的红细胞数和血红蛋白量随年龄不同而有差异，根据世界卫生组织（WHO）的资料，血红蛋白的低限值在6 个月～6 岁者为110 g/L，6～14 岁为120 g/L，海拔每升高1000 米，血红蛋白上升4%；低于此值者为贫血。6 个月以下的婴儿由于生理性贫血等因素，血红蛋白值变化较大，目前尚无统一标准。我国小儿血液会议（1989 年）暂定：血红蛋白在新生儿期<145 g/L，1～4 月时<90 g/L，4～6 个月时<100 g/L者为贫血。

一、贫血的分类

（一）贫血程度分类

根据外周血血红蛋白含量或红细胞数可分为四度：①血红蛋白（Hb）从正常下限～90 g/L者为轻度；②～60 g/L者为中度；③～30 g/L者为重度；④<30 g/L者为极重度。新生儿Hb为144～120 g/L者为轻度，～9 0g/L者为中度，～60 g/L者为重度，<60 g/L者为极重度。

（二）病因分类

根据造成贫血的原因将其分为红细胞或血红蛋白生成不足、溶血性和失血性三类。

1．红细胞和血红蛋白生成不足

（1）造血物质缺乏：如缺铁性贫血（铁缺乏）、巨幼心红细胞性贫血（维生素B_{12}、叶酸缺乏）、维生素B_6缺乏性贫血、铜缺乏、维生素C缺乏、蛋白质缺乏等。

（2）骨髓造血功能障碍：如再生障碍性贫血，单纯红细胞再生障碍性贫血。

（3）其他：感染性及炎症性贫血，慢性肾病所致贫血，铅中毒，癌症性贫血等。

2．溶血性贫血 可由红细胞内在异常或红细胞外在因素引起。

（1）红细胞内在异常：①红细胞膜结构缺陷：如遗传性球形红细胞增多症、遗传性椭圆形红细胞增多症、棘状红细胞增多、阵发性睡眠性血红蛋白尿等；②红细胞酶缺乏：如葡萄糖-6-磷酸脱氢酶（G-6-PD）缺乏、丙酮酸激酶（PK）缺乏症等；③血红蛋白合成或结构异常：如地中海贫血、血红蛋白病等。

（2）红细胞外在因素：①免疫因素：体内存在破坏红细胞的抗体，如新生儿溶血症、自身免疫性溶血性贫血、药物所致的免疫性溶血性贫血等；②非免疫因素：如感染、物理化学因素、毒素、脾功能亢进、弥散性血管内凝血等。

3．失血性贫血 包括急性失血和慢性失血引起的贫血。

（三）形态分类

根据红细胞数、血红蛋白量和血细胞比容计算红细胞平均容积（MCV）、红细胞平均血红蛋白（MCH）和红细胞平均血红蛋白浓度（MCHC）的结果而将贫血分为四类（表15-1）。

表15-1 贫血的细胞形态分类

	MCV（fl）	MCH（pg）	MCHC(%)
正常值	80～94	28～32	32～38
大细胞性	>94	>32	32～38
正细胞性	80～94	28～32	32～38
单纯小细胞性	<80	<28	32～38
小细胞低色素性	<80	<28	<32

二、临床表现

贫血的临床表现与其病因、程度轻重、发生急慢等因素有关。急性贫血如急性失血或溶血，虽贫血程度轻，亦可引起严重症状甚至休克；慢性贫血，若机体各器官的代偿功能较好，可无症状或症状较轻，当代偿不全时才逐渐出现症状。红细胞的主要功能是携带氧气，故贫血时组织与器官缺氧而产生一系列症状。

（一）一般表现

皮肤、黏膜苍白为突出表现。贫血时皮肤（面、耳轮、手掌等）、黏膜（睑结膜、口腔黏膜）及甲床呈苍白色；重度贫血时皮肤往往呈蜡黄色，易误诊为轻度黄疸；相反，伴有黄疸、青紫或其他皮肤色素改变时可掩盖贫血的表现。此外，病程较长的患儿还常有易疲倦、毛发干枯、营养低下、体格发育迟缓等症状。

（二）造血器官反应

婴幼儿期的骨髓几乎全是红髓，贫血时，骨髓不能进一步代偿而出现骨髓外造血，表现为肝脾和淋巴结肿大，外周血中可出现有核红细胞、幼稚粒细胞。

（三）各系统症状

1．循环和呼吸系统 贫血时可出现呼吸加速、心率加快、脉搏加强、动脉压增高，有时可见毛细血管搏动。重度贫血失代偿时，则出现心脏扩大，心前区收缩期杂音，甚至发生充血性心力衰竭。

2．消化系统 胃肠蠕动及消化酶分泌功能均受影响，出现食欲减退、恶心、腹胀或便秘等。偶有舌炎、舌乳头萎缩等。

3．神经系统 常表现精神不振，注意力不集中，情绪易激动等。年长儿可有头痛、昏眩、眼前有黑点或耳鸣等。

三、诊断要点

贫血是综合征，必须找出其贫血的原因，才能进行合理和有效的治疗。因此，详细询问病史、全面的体格检查和必要的实验室检查是作出贫血病因诊断的重要依据。

（一）病史

1．发病年龄 可提供诊断线索。不同年龄发生贫血的病因不同：出生后即有严重贫血者要考虑产前或产时失血；生后48 小时内出现贫血伴有黄疸者，以新生儿溶血症可能性大；婴儿期发病者多考虑营养缺乏性贫血、遗传性溶血性贫血；儿童期发病者多考虑慢性失血性贫血、再生障碍性贫血、其他造血系统疾病、全身性疾病引起的贫血。

2．病程经过和伴随症状 起病快、病程短者，提示急性溶血或急性失血；起病缓慢者，提示营养性贫血、慢性失血、慢性溶血等。如伴有黄疸和血红蛋白尿提示溶血；伴有呕血、便血、血尿、瘀斑等提示出血性疾病；伴有神经和精神症状如嗜睡、震颤等提示维生素B_{12}缺乏；伴有骨病提示骨髓浸润性病变，肿瘤性疾病多伴有发热、肝脾及淋巴结肿大。

3．喂养史：详细了解婴幼儿的喂养方法及饮食的质与量对诊断和病因分析有重要意义。单纯乳类喂养未及时添加辅食的婴儿，易患营养性缺铁性贫血或巨幼细胞性贫血；幼儿及年长儿饮食质量差或搭配不合理者，可能为缺铁性贫血。

4．过去史：询问有无寄生虫病特别是钩虫病史；询问其他系统疾病，包括消化系统疾病、慢性肾病、严重结核、慢性炎症性疾病如类风湿病等可引起贫血的有关疾病。此外，还要询问是否服用对造血系统有不良影响的药物如氯霉素、磺胺等。

5．家族史：与遗传有关的贫血，如遗传性球形红细胞增多症、G-6-PD缺乏、地中海贫血等，家族（或近亲）中常有同样患者。

（二）体格检查

1．生长发育 慢性贫血往往有生长发育障碍。某些遗传性溶血性贫血，特别是重型β地中海贫血，除发育障碍外还表现有特殊面貌，如颧、额突出，眼距宽，鼻梁低，下颌骨较大等。

2．营养状况 营养不良常伴有慢性贫血。

3．皮肤、黏膜 皮肤和黏膜苍白的程度一般与贫血程度成正比。小儿因自主神经功能不稳定，故面颊的潮红与苍白有时不一定能正确反映有无贫血，观察甲床、结合膜及唇黏膜的颜色比较可靠。长期慢性贫血者皮肤呈苍黄，甚至呈古铜色；反复输血者皮肤常有色素沉着。如贫血伴有皮肤、黏膜出血点或瘀斑，要注意排除出血性疾病和白血病。伴有黄疸时提示溶血性贫血。

4．指甲和毛发 缺铁性贫血的患儿指甲菲薄、脆弱，严重者扁平甚至呈匙形反甲。巨幼红细胞性贫血患儿头发细黄、干稀、无光泽，有时呈绒毛状。

5．肝脾和淋巴结肿大 这是婴幼儿贫血常见的体征。肝脾轻度肿大多提示髓外造血；如肝脾明显肿大且以脾大为主者，多提示遗传性溶血性贫血。贫血伴有明显淋巴结肿大者，应考虑造血系统恶性病变（如白血病、恶性淋巴瘤）。

除上述病史与体检资料外，还应注意贫血对各系统的影响，如心脏扩大和心尖部收缩期杂音等，以及各系统可能的其他损害与贫血的因果关系。

（三）实验室检查

血液检查是贫血的诊断和鉴别诊断不可缺少的措施，临床上应由简而繁进行。一般根据病史、体征和初步的实验室检查资料，通过综合分析，对大多数贫血可做出初步诊断或确定诊断；对一些病情复杂暂时不能明确诊断者，亦可根据初步线索进一步选择必要的检查。

1．外周血象 这是一项简单而又重要的检查方法。根据红细胞和血红蛋白量可判断有无贫血及其程度，并可根据形态分类协助病因分析。仔细观察血涂片中红细胞大小、形态及染色情况，对贫血的病因诊断有帮助。如红细胞较小、染色浅、中央淡染色区扩大，多提示缺铁性贫血；红细胞呈球形，染色深提示遗传性球形红细胞增多症；红细胞大小不等，染色浅并有异形、靶形和碎片者，多提示地中海贫血；红细胞形态正常则见于急性溶血或骨髓造血功能障碍。白细胞和血小板计数以及观察血涂片中白细胞和血小板的质和量的改变，对判断贫血的原因也有帮助。

网织红细胞计数可反映骨髓造红细胞的功能。增多提示骨髓造血功能活跃，可见于急慢性溶血或失血性贫血；减少提示造血功能低下，可见于再生障碍性贫血、营养性贫血等。此外在治疗过程中定期检查网织红细胞计数，有助于判断疗效，如缺铁性贫血经合理的治疗后，网织红细胞在1 周左右即开始增加。

2．骨髓检查 骨髓涂片检查可直接了解骨髓造血细胞生成的质和量的变化，对某些贫血的诊断具有决定性意义（如白血病、再生障碍性贫血、营养性巨幼红细胞性贫血）。骨髓活体组织检查对白血病、转移瘤等骨髓病变具有诊断价值。

3．血红蛋白分析检查 如血红蛋白碱变性试验、血红蛋白电泳、包涵体生成试验等，对地中海贫血和异常血红蛋白病的诊断有重要意义。

4．红细胞脆性试验 脆性增高见于遗传性球形红细胞增多症；减低则见于地中海贫血。

5．特殊检查 红细胞酶活力测定对先天性红细胞酶缺陷所致的溶血性贫血有诊断意义；抗人球蛋白试验可以协助诊断自身免疫性溶血；血清铁、铁蛋白、红细胞游离原卟啉等检查可以

分析体内铁代谢情况，以协助诊断缺铁性贫血；核素^{51}铬可以测定红细胞寿命；基因分析方法对遗传性溶血性贫血不但有诊断意义，还有产前诊断价值。

四、治疗原则

（一）去除病因

这是治疗贫血的关键，有些贫血在病因去除后，很快可以治愈。对一些贫血原因暂时未明的，应积极寻找病因，予以去除。

（二）一般治疗

加强护理，预防感染，改善饮食质量和搭配等。

（三）药物治疗

针对贫血的病因，选择有效药物给予治疗，如铁剂治疗缺铁性贫血，维生素B_{12}和叶酸治疗巨幼红细胞性贫血，肾上腺皮质激素治疗自身免疫性溶血性贫血和先天性纯红细胞再生障碍性贫血；“强化”免疫抑制（抗胸腺球蛋白、环孢素A等）治疗再生障碍性贫血等。

（四）输红细胞

当贫血引起心功能不全时，输红细胞是抢救措施。对长期慢性贫血者，若代偿功能良好，可不必输红细胞；必需输注时应注意量和速度，贫血愈严重，一次输注量愈少且速度宜慢。一般选用浓缩红细胞，每次5～l0 ml/kg，速度不宜过快，以免引起心力衰竭和肺水肿。对于贫血合并肺炎的患儿，每次输红细胞量更应减少，速度减慢。

（五）造血干细胞移植

这是目前根治严重遗传性溶血性贫血和再生障碍性贫血的有效方法，但受HLA相配的造血干细胞来源的限制。

（六）并发症治疗

婴幼儿贫血易合并急、慢性感染，营养不良，消化功能紊乱等，应予积极治疗。同时还应考虑贫血与合并症的相互影响的特点，如贫血患儿在消化功能紊乱时对于体液失衡的调节能力较无贫血的小儿差，在输液治疗时应予注意。

第三节 营养性贫血

一、缺铁性贫血

缺铁性贫血（iron deficiency anemia，IDA）是由于体内铁缺乏导致血红蛋白合成减少，临床上以小细胞低色素性贫血、血清铁蛋白减少和铁剂治疗有效为特点的贫血症。本病以婴幼儿发病率最高，严重危害小儿健康，是我国重点防治的小儿常见病之一。

【铁的代谢】

1．人体内铁元素的含量及其分布 正常人体内的含铁总量随着年龄、体重、性别和血红蛋白水平的不同而异。正常成人男性体内总铁量约为50 mg/kg，女性约为35 mg/kg，新生儿约为75 mg/kg。总铁量中约64％用于合成血红蛋白，32％以铁蛋白及含铁血黄素形式贮存于骨髓、肝和脾内，3.2％用于合成肌红蛋白， <1％存在于含铁酶内和以运转铁形式存在于血浆中。

2．铁的来源 铁的来源主要有二：

（1）外源性铁：主要来自食物，占人体铁摄入量的1/3；分为血红素铁和非血红素铁，前者吸收率高于后者。动物性食物含铁高且为血红素铁，吸收率达10％～25％；母乳与牛乳含铁量均低；但母乳的铁吸收率比牛乳高2～3 倍。植物性食物中的铁是非血红素铁，吸收率约1.7％～7.9％。

（2）内源性铁：体内红细胞衰老或破坏所释放的血红蛋白铁，占人体铁摄入量的2/3，几乎全部被再利用。

3．铁的吸收和运转 食物中的铁主要以Fe^{2+} 形式在十二指肠和空肠上段被吸收。进入肠黏膜细胞的Fe^{2+} 被氧化成Fe^{3+} ，一部分与细胞内的去铁蛋白（apoferritin）结合形成铁蛋白（ferritin），暂时保存在肠黏膜细胞中；另一部分与细胞浆中载体蛋白结合后移出胞外进入血液，与血浆中的转铁蛋白（transferrin，Tf）结合，随血液循环将铁运送到需铁和贮铁组织，供给机体利用。红细胞破坏后释放出的铁，也同样通过与Tf 结合后运送到骨髓等组织，被利用或贮存。

肠黏膜细胞调节铁的吸收，这种调节作用又通过体内贮存铁和转铁蛋白受体（TfR）来调控。当体内贮存铁充足时，转铁蛋白受体（TfR）与铁复合物合成减少，铁蛋白合成增加，肠黏膜细胞内的铁大部分以铁蛋白形式贮存在该细胞内，随肠黏膜细胞的脱落而被排出体外，因而吸收减少；当体内缺铁时，TfR合成增加，铁蛋白合成减少，肠黏膜细胞内TfR-铁复合物进入血流，铁的吸收增加。

正常的情况下，血浆中的转铁蛋白仅1/3与铁结合，此结合的铁称为血清铁（serum iron，SI）；其余2/3的转铁蛋白仍具有与铁结合的能力，在体外加入一定量的铁可使其成饱和状态，所加的铁量即为未饱和铁结合力。血清铁与未饱和铁结合力之和称之为血清总铁结合力（totoal iron binding capacity，TIBC）。血清铁在总铁结合力中所占的百分比称之为转铁蛋白饱和度（transferin saturation，TS）。

4．铁的利用与储存 铁到达骨髓造血组织后即进入幼红细胞，在线粒体中与原卟啉结合形成血红素，血红素与珠蛋白结合形成血红蛋白。此外，铁参与肌红蛋白和某些酶（如细胞色素C、单胺氧化酶、核糖核酸还原酶、琥珀酸脱氢酶等）的合成。在体内未被利用的铁以铁蛋白及含铁血黄素的形式贮存。在机体需要铁时，这两种铁均可被利用，通过还原酶的作用，使铁蛋白中的Fe^{2+} 释放，然后被氧化酶氧化成Fe^{3+} ，与转铁蛋白结合后被转运到需铁的组织。

5．铁的排泄 正常情况下每日仅有极少量的铁排出体外。小儿每日排出量约为15 μg/kg，约2/3随脱落的肠黏膜细胞、红细胞、胆汁由肠道排出，其他经肾脏和汗腺排出，表皮细胞脱落也失去极微量的铁。

6．铁的需要量 小儿由于生长发育的需要，每日需摄入的铁量相对较成人为多。成熟儿自生后4 个月至3 岁每天约需铁 1mg/kg；早产儿约达2 mg/kg；各年龄小儿每天摄入总量不宜超过15 mg。

7．出生前、后铁代谢特点

（1）胎儿期铁代谢特点：胎儿通过胎盘从母体获得铁，以孕后期3 个月获铁量最多，平均每日约4 mg。故足月儿从母体所获得的铁足够其生后4～5 个月内之需；而未成熟儿从母体所获得的铁较少，容易发生缺铁。当孕母严重缺铁，由于母体TfR的代偿性增加和胎盘摄铁能力的下降，可影响胎儿获取铁量。

（2）婴幼儿期铁代谢的特点：足月新生儿体内总铁约75 mg/kg，其中25％为贮存铁。生后由于“生理性溶血”释放的铁较多，随后是“生理性贫血”期造血相对较低下，加之从母体获取的铁一般能满足4 个月之需，故婴儿早期不易发生缺铁。但早产儿从母体获取铁少，且生长发育更迅速，可较早发生缺铁。约4 月龄以后，从母体获取的铁逐渐耗尽，加上此期生长发育迅速，造血活跃，因此对膳食铁的需要增加，而婴儿主食人乳和牛乳的铁含量均低，不能满足机体之需，贮存铁耗竭后即发生缺铁，故6 个月～2 岁的小儿缺铁性贫血发生率高。

（3）儿童期和青春期铁代谢特点：儿童期一般较少缺铁，此期缺铁的主要原因是偏食使

摄取的铁不足，或是食物搭配不合理使铁的吸收受抑制；肠道慢性失血也是此期缺铁的原因。青春期由于生长发育迅速而对铁的需要量增加，初潮以后少女如月经过多造成铁的丢失也是此期缺铁的原因。

【病因】

1．先天储铁不足 胎儿从母体获得的铁以妊娠最后3 个月最多，故早产、双胎或多胎、胎儿失血和孕母严重缺铁等均可使胎儿储铁减少。

2．铁摄入量不足 这是缺铁性贫血的主要原因。人乳、牛乳、谷物中含铁量均低，如不及时添加含铁较多的辅食，容易发生缺铁性贫血。

3．生长发育因素 婴儿期生长发育较快，5 个月时和1 岁时体重分别为出生时的2 倍和3 倍；随着体重增加，血容量也增加较快，1 岁时血循环中的血红蛋白增加2 倍；未成熟儿的体重及血红蛋白增加倍数更高；如不及时添加含铁丰富的食物，则易致缺铁。

4．铁的吸收障碍 食物搭配不合理可影响铁的吸收。慢性腹泻不仅铁的吸收不良，而且铁的排泄也增加。

5．铁的丢失过多 正常婴儿每天排泄铁量相对比成人多。每1 ml血约含铁0.5 mg，长期慢性失血可致缺铁，如肠息肉、梅克尔憩室、膈疝、钩虫病等可致慢性失血，用不经加热处理的鲜牛奶喂养的婴儿可因对牛奶过敏而致肠出血（每天失血约0.7 ml）。

【发病机制】

1．缺铁对血液系统的影响 铁是合成血红蛋白的原料，缺铁时血红素生成不足，进而血红蛋白合成也减少，导致新生的红细胞内血红蛋白含量不足，细胞浆减少，细胞变小；而缺铁对细胞的分裂、增殖影响较小，故红细胞数量减少程度不如血红蛋白减少明显，从而形成小细胞低色素性贫血。缺铁通常经过以下三个阶段才发生贫血：①铁减少期（iron depletion，ID）：此阶段体内储存铁已减少，但供红细胞合成血红蛋白的铁尚未减少；②红细胞生成缺铁期（iron deficient erythropoiesis，IDE）：此期储存铁进一步耗竭，红细胞生成所需的铁亦不足，但循环中血红蛋白的量尚未减少；③缺铁性贫血期（iron deficiency anemia，IDA）：此期出现小细胞低色素性贫血，还有一些非造血系统的症状。

2．缺铁对其他系统的影响 缺铁可影响肌红蛋白的合成，并可使多种含铁酶（如细胞色素C、单胺氧化酶、核糖核苷酸还原酶、琥珀酸脱氢酶等）的活性减低。由于这些含铁酶与生物氧化、组织呼吸、神经递质分解与合成有关，故铁缺乏时造成细胞功能紊乱，尤其是单胺氧化酶的活性降低，造成重要的神经介质如5-羟色胺、去甲肾上腺素、肾上腺素及多巴胺发生明显变化，不能正常发挥功能，因而产生一些非造血系统的表现，如体力减弱、易疲劳、表情淡漠、注意力难于集中、注意力减退和智力减低等。缺铁还可引起组织器官的异常，如口腔黏膜异常角化、舌炎、胃酸分泌减少、脂肪吸收不良和反甲等。此外，缺铁还可引起细胞免疫功能降低，易患感染性疾病。

【临床表现】

任何年龄均可发病，以6 个月至2 岁最多见。发病缓慢，其临床表现随病情轻重而有不同。

1．一般表现 皮肤、黏膜逐渐苍白，以唇、口腔黏膜及甲床较明显。易疲乏，不爱活动。年长儿可诉头晕、眼前发黑、耳鸣等。

2．髓外造血表现 由于髓外造血，肝、脾可轻度肿大；年龄愈小、病程愈久、贫血愈重，肝脾肿大愈明显。

3．非造血系统症状

（1）消化系统症状：食欲减退，少数有异食癖（如嗜食泥土、墙皮、煤渣等）；可有呕吐、腹泻；可出现口腔炎、舌炎或舌乳头萎缩；重者可出现萎缩性胃炎或吸收不良综合征。

（2）神经系统症状：表现为烦躁不安或萎靡不振，精神不集中、记忆力减退，智力多数低于同龄儿。

（3）心血管系统症状：明显贫血时心率增快，严重者心脏扩大甚至发生心力衰竭。

（4）其他：因细胞免疫功能降低，常合并感染。可因上皮组织异常而出现反甲。

【实验室检查】

1．外周血象 血红蛋白降低比红细胞数减少明显，呈小细胞低色素性贫血。外周血涂片可见红细胞大小不等，以小细胞为多，中央淡染区扩大。平均红细胞容积（MCV）<80 fl，平均红细胞血红蛋白量（MCH）<26 pg，平均红细胞血红蛋白浓度（MCHC）<0.31。网织红细胞数正常或轻度减少。白细胞、血小板一般无改变。

2．骨髓象 呈增生活跃，以中、晚幼红细胞增生为主。各期红细胞均较小，胞浆少，染色偏蓝，显示胞浆成熟程度落后于胞核。粒细胞和巨核细胞系一般无明显异常。

3．有关铁代谢的检查

（1）血清铁蛋白（serum ferritin，SF）：可较敏感地反映体内贮存铁情况，因而是诊断缺铁ID期的敏感指标。其放射免疫法测定的正常值：<3 个月婴儿为194～238 μg/L，3 个月后为18～91 μg/L；低于12 μg/L，提示缺铁。由于感染、肿瘤、肝脏和心脏疾病时SF明显升高，故当缺铁合并这些疾病时其SF值可不降低，此时测定红细胞内碱性铁蛋白有助诊断。

（2）红细胞游离原卟啉（free erythrocyte protoporphyin，FEP）：红细胞内缺铁时FEP不能完全与铁结合成血红素，血红素减少又反馈性地使FEP合成增多，未被利用的FEP在红细胞内堆积，导致FEP值增高，当FEP>0.9 μmol/L（500 μg/dl）即提示细胞内缺铁。如SF值降低、FEP升高而未出现贫血，这是IDE期的典型表现。FEP增高还见于铅中毒、慢性炎症和先天性原卟啉增多症。

（3）血清铁（SI）、总铁结合力（TIBC）和转铁蛋白饱和度（TS）：这三项检查是反映血浆中铁含量，通常在IDA期才出现异常：即SI和TS降低，TIBC升高。SI正常值为12.8～31.3 μmol/L（75～175 μg/ dl），<9.0～10.7 μmol/L（50～60 μg/ dl）有意义，但其生理变异大，并且在感染、恶性肿瘤、类风湿性关节炎等疾病时也可降低。TIBC>62.7 μmol/L（350 ug/ dl）有意义；其生理变异较小，在病毒性肝炎时可增高。TS<15％有诊断意义。

4．骨髓可染铁 骨髓涂片用普鲁士蓝染色显微镜检查，缺铁时细胞外铁减少。观察红细胞内铁粒细胞数，如<15％提示储存铁减少（细胞内铁减少），这是一项反映体内贮存铁的敏感而可靠的指标。

【诊断】

根据病史特别是喂养史、临床表现和血象特点，一般可作出初步诊断。进一步作有关铁代谢的生化检查有确诊意义。必要时可作骨髓检查。用铁剂治疗有效可证实诊断。

地中海贫血、异常血红蛋白病、维生素B_6缺乏性贫血、铁粒幼红细胞性贫血等亦表现为小细胞低色素性贫血，应根据各病临床特点和实验室检查特征加以鉴别。

【治疗】

主要原则为去除病因和补充铁剂。

1．一般治疗 加强护理，保证充足睡眠；避免感染，如伴有感染者应积极控制感染；重度

贫血者注意保护心脏功能。根据患儿消化能力，适当增加含铁质丰富的食物，注意饮食的合理搭配，以增加铁的吸收。

2．去除病因 对饮食不当者应纠正不合理的饮食习惯和食物组成，有偏食习惯者应予纠正。如有慢性失血性疾病，如钩虫病、肠道畸形等，应予及时治疗。

3．铁剂治疗

（1）口服铁剂：剂是治疗缺铁性贫血的特效药，若无特殊原因，应采用口服法给药；二价铁盐容易吸收，故临床均选用二价铁盐制剂。常用的口服铁剂有硫酸亚铁（含元素铁20％）、富马酸亚铁（含元素铁33％）、葡萄糖酸亚铁（含元素铁12％）、琥珀酸亚铁（含元素铁35％）等。口服铁剂的剂量为元素铁每日4～6 mg/kg，分3 次口服，一次量不应超过元素铁1.5～2 mg/kg；以两餐之间口服为宜，为减少胃肠副反应，可从小剂量开始，如无不良反应，可在1～2 日内加至足量。同时服用维生素C，可增加铁的吸收。牛奶、茶、咖啡及抗酸药等与铁剂同服均可影响铁的吸收。

（2）注射铁剂：注射铁剂较容易发生不良反应，甚至可发生过敏性反应致死，故应慎用。其适应证是：①诊断肯定但口服铁剂后无治疗反应者；②口服后胃肠反应严重，虽改变制剂种类、剂量及给药时间仍无改善者；③由于胃肠疾病胃肠手术后不能应用口服铁剂或口服铁剂吸收不良者。常用注射铁剂有：山梨醇枸橼酸铁复合物，专供肌肉注射用；右旋糖酐铁复合物，为氢氧化铁与右旋糖酐铁复合物，可供肌肉注射或静脉注射；葡萄糖氧化铁，供静脉注射用。

补充铁剂12～24 小时后，细胞内含铁酶开始恢复，烦躁等精神症状减轻，食欲增加。网织红细胞于服药2～3 天后开始上升，5～7 日达高峰，2～3 周后下降至正常。治疗1～2 周后血红蛋白逐渐上升，通常于治疗3～4 周达到正常。如3 周内血红蛋白上升不足20 g/L，注意寻找原因。如治疗反应满意，血红蛋白恢复正常后再继续服用铁剂6～8 周，以增加铁储存。

4．输红细胞 一般不必输红细胞，输注红细胞的适应证是：①贫血严重，尤其是发生心力衰竭者；②合并感染者；③急需外科手术者。贫血愈严重，每次输注量应愈少。Hb 在30 g/L以下者，应采用等量换血方法；Hb 在30～60 g/L者，每次可输注浓缩红细胞4～6 ml/kg；Hb 在60 g/L以上者，不必输红细胞。

【预防】

做好卫生宣教工作，使全社会尤其是家长认识到缺铁对小儿的危害性及做好预防工作的重要性，使之成为儿童保健工作中的重要内容。主要预防措施包括：①提倡母乳喂养，因母乳中铁的吸收利用率较高；②做好喂养指导，无论是母乳或人工喂养的婴儿，均应及时添加含铁丰富且铁吸收率高的辅助食品，如精肉、血、内脏、鱼等，并注意膳食合理搭配，婴儿如以鲜牛乳喂养，必须加热处理以减少牛奶过敏所致肠道失血；③婴幼儿食品（谷类制品、牛奶制品等）应加入适量铁剂加以强化；④对早产儿，尤其是非常低体重的早产儿宜自2 个月左右给予铁剂预防。

二、营养性巨幼细胞贫血

营养性巨幼细胞贫血（nutritional megaloblastic anemia）是由于维生素B_{12}或（和）叶酸缺乏所致的一种大细胞性贫血。主要临床特点是贫血、神经精神症状、红细胞的胞体变大、骨髓中出现巨幼细胞、用维生素B_{12}或（和）叶酸治疗有效。

【病因】

1．摄入量不足 单纯母乳喂养而未及时添加辅食的婴儿、人工喂养不当及严重偏食的婴幼儿，其饮食中缺乏肉类、动物肝、肾及蔬菜，可致维生素B_{12}和叶酸缺乏。羊乳中叶酸量很低，

单纯以羊奶喂养者，可致叶酸缺乏。

2．需要量增加 婴儿生长发育较快，对叶酸、维生素B_{12}的需要量也增加，严重感染者维生素B_{12}的消耗量增加，需要量相应增加。

3．吸收和运输障碍 食物中维生素B_{12}必须与胃底部壁细胞分泌的糖蛋白结合成复合物才能由末端回肠黏膜吸收，进入血循环后再与转钴蛋白（transcobalamin）结合，运送到肝脏。慢性腹泻影响叶酸吸收，先天性叶酸代谢障碍（如不肠吸收叶酸缺陷及叶酸转运功能障碍）也可致叶酸缺乏。

【发病机制】

叶酸经叶酸还原酶的还原作用和维生素B_{12}的催化作用后变成四氢叶酸，后者是DNA合成过程中必需的辅酶。当维生素B_{12}或叶酸缺乏后，使四氢叶酸减少，导致DNA合成减少。幼稚红细胞内的DNA合成减少使其分裂和增殖时间延长，出现细胞核的发育落后于细胞质的发育而血红蛋白的合成不受影响，使红细胞的胞体变大，形成巨幼红细胞。由于红细胞生成速度慢，巨幼红细胞在骨髓内易被破坏，进入血循环的红细胞寿命也较短，从而出现贫血。

DNA合成不足也导致粒细胞核成熟障碍，出现巨大幼稚粒细胞和中性粒细胞分叶过多现象。而且，亦可使巨核细胞的核发育障碍而致巨大血小板。

维生素B_{12}能促使脂肪代谢产生的甲基丙二酸转变成琥珀酸而参与三羧酸循环，此作用与神经髓鞘中脂蛋白形成有关，因而能保持中枢和外周髓鞘神经纤维的功能完整性；当其缺乏时，可导致中枢和外周神经髓鞘受损，因而出现神经精神症状。叶酸缺乏主要引起情感改变，偶见深感觉障碍，其机制尚未明了。

维生素B_{12}缺乏还可使中性粒细胞和巨噬细胞吞噬细菌后的杀灭细菌作用减弱，使组织、血浆及尿液中甲基丙二酸堆积，后者是结核杆菌细胞壁成分的原料，有利于结核杆菌生长，故维生素B_{12}缺乏者易伴结核病。

【临床表现】

以6 个月～2 岁多见，起病缓慢。

1．一般表现 多呈虚胖或颜面轻度浮肿，毛发纤细稀疏、黄色，严重者皮肤有出血点或瘀斑。

2．贫血表现 皮肤常呈现腊黄色，睑结膜、口唇、指甲等处苍白，偶有轻度黄疸；疲乏无力，常伴有肝、脾肿大。

3．精神、神经症状 可出现烦躁不安、易怒等症状。维生素B_{12}缺乏者表现为表情呆滞、目光发呆、对周围反应迟钝，嗜睡、不认亲人，少哭不笑，智力、动作发育落后甚至退步。重症病例可出现不规则性震颤，手足无意识运动，甚至抽搐、感觉异常、共济失调、踝阵挛和Barbinski征阳性等。叶酸缺乏不发生神经系统症状，但可导致神经精神异常。

4．消化系统症状 常出现较早，如厌食、恶心、呕吐、腹泻和舌炎等。

【实验室检查】

1．外周血象 呈大细胞性贫血，MCV>94 fl，MCH>32 pg。血涂片可见红细胞大小不等，以大细胞为多，易见嗜多色性和嗜碱点彩红细胞，可见巨幼变的有核红细胞，中性粒细胞呈分叶过多现象。网织红细胞、白细胞、血小板计数常减少。

2．骨髓象 增生明显活跃，以红细胞系增生为主，粒、红系统均出现巨幼变，表现为胞体变大、核染色质粗而松、副染色质明显。中性粒细胞的胞浆空泡形成，核分叶过多。巨核细胞的核有过度分叶现象，巨大血小板。

3．血清维生素B_{12}和叶酸测定 血清维生素B_{12}正常值为200～800 ng/L，<100 ng/L为缺乏。

血清叶酸水平正常值为5～6 μg/L，<3 μg/L为缺乏。

【诊断】

根据临床表现、血象和骨髓象可诊断为巨幼红细胞性贫血。在此基础上，如精神神经症状明显，则考虑为维生素B_{12}缺乏所致。有条件时测定血清维生素B_{12}或叶酸水平可进一步协助确诊。

【治疗】

1．一般治疗　注意营养，及时添加辅食；加强护理，防止感染。

2．去除病因　对引起维生素B_{12}和叶酸缺乏的原因应予去除。

3．维生素B_{12}和叶酸治疗　有精神神经症状者，应以维生素B_{12}治疗为主，如单用叶酸反而有加重症状的可能。维生素B_{12} 500～1000 μg一次肌注；或每次肌注100 μg，每周2～3 次，连用数周，直至临床症状好转、血象恢复正常为止。当有神经系统受累表现时，可予每日1 mg，连续肌注2 周以上；由于维生素B_{12}吸收缺陷所致的患者，每月肌注1 mg，长期应用。用维生素B_{12}治疗后6～7 小时骨髓内巨幼红细胞可转为正常幼红细胞；一般精神症状2～4 天后好转；网织红细胞2～4 天开始增加，6～7 天达高峰，2 周后降至正常；精神神经症状恢复较慢。

叶酸口服剂量为每次5 mg，每日3 次，连续数周至临床症状好转、血象恢复正常为止。同时口服维生素C有助叶酸的吸收。服叶酸1～2 天后食欲好转，骨髓中巨幼红细胞转为正常；2～4 天网织红细胞增加，4～7 天达高峰；2～6 周红细胞和血红蛋白恢复正常。因使用抗叶酸代谢药物而致病者，可用亚叶酸钙（calc-leucovorin）治疗。先天性叶酸吸收障碍者，口服叶酸剂量应增至每日15～50 mg才有效。

【预防】

改善哺乳母亲的营养，婴儿应及时添加辅食，注意饮食均衡，及时治疗肠道疾病，注意合理应用抗叶酸代谢药物。

第四节　溶血性贫血

一、遗传性球形红细胞增多症

遗传性球形红细胞增多症（hereditary spherocytosis，HS）是由红细胞膜先天性缺陷而引起的溶血性贫血，以不同程度贫血、反复出现黄疸、脾大、球形红细胞增多及红细胞渗透脆性增加为特征。

【病因和发病机制】

本病大多数为常染色体显性遗传，少数为常染色体隐性遗传。正常红细胞膜由双层脂质和膜蛋白组成。本病由于调控红细胞膜蛋白的基因突变造成膜骨架蛋白（膜收缩蛋白、锚蛋白）单独或联合缺陷。这些缺陷造成红细胞的病理生理改变：①红细胞膜双层脂质不稳定以出芽形式形成囊状而丢失，使红细胞表面积减少，表面积与体积比值下降，红细胞变成球形；②红细胞膜阳离子通透增加，钠和水进入胞内而钾透出胞外，为了维持红细胞内外钠离子平衡，钠泵作用加强致ATP缺乏，钙-ATP酶受抑，致细胞内钙离子浓度升高并沉积在红细胞膜上；③红细胞膜蛋白磷酸化功能下降，过氧化酶增加，与膜结合的血红蛋白增加，导致红细胞变形性下降。球形红细胞的细胞膜变形性能和柔韧性能减弱，少量水分进入胞内即易胀破而溶血，红细胞通过脾时易被破坏而溶解，发生血管外溶血。

【临床表现】

贫血、黄疸、脾大是本病三大特征，而且在慢性溶血性贫血的过程中易出现急性溶血发作。发病年龄越小，症状越重。新生儿期起病者出现急性溶血性贫血和高胆红素血症；婴儿和儿童患者贫血的程度差异较大，大多为轻至中度贫血。黄疸可见于大部分患者，多为轻度，呈间歇性。几乎所有患者有脾大，且随年龄增长而逐渐显著，溶血危象时肿大明显。肝脏多为轻度肿大。未行脾切除人年长儿可并发色素性胆石症。偶见踝部溃疡。

在慢性病程中，常因感染、劳累或情绪紧张等因素诱发“溶血危象”：贫血和黄疸突然加重，伴有发热、寒战、呕吐，脾大显著并有疼痛。也可出现“再生障碍危象”：表现为以红系造血受抑为主的骨髓造血功能暂时性抑制，出现严重贫血，可有不同程度的白细胞和血小板减少。后者与微小病毒（parvovirus）感染有关，呈自限性过程，持续数天或1～2 周缓解。

【实验室检查】

1. 外周血象 贫血多为轻至中度，发生危象时可呈重度；网织红细胞升高；MCV和MCH 多正常，MCHC 可增加；白细胞及血小板多正常。外周血涂片可见胞体小、染色深、中心浅染区消失的球形红细胞增多，是本病的特征，约占红细胞数的0.2～0.4 。仅少数患者球形红细胞数量少或红细胞形态改变不明显。

2．红细胞渗透脆性试验 大多数病例红细胞渗透脆性增加，0.5％～0.75％盐水开始溶血，0.40％完全溶血。24 小时孵育脆性试验则100％病例阳性。

3．其他 溶血的证据如血清间接胆红素和游离血红蛋白增高，结合珠蛋白降低，尿中尿胆原增加。红细胞自身溶血试验阳性，加入葡萄糖或ATP可以纠正。骨髓象示红细胞系统明显增生，但有核红细胞形态无异常。酸化甘油试验阳性。采用十二磺酸钠聚丙烯酰胺凝胶电泳或放射免疫法测定膜蛋白含量有助于判断膜蛋白的缺陷。分子生物学方法可确定基因突变位点。

【诊断和鉴别诊断】

根据贫血、黄疸、脾大等临床表现，球形红细胞增多，红细胞渗透脆性增加即可作出诊断；阳性家族史更有助于确诊。对于球形红细胞数量不多者，可作孵育后红细胞渗透脆性试验和自身溶血试验，如为阳性有诊断意义。须注意铁缺乏时红细胞渗透脆性可降低，当本病合并缺铁时，红细胞渗透脆性可能正常。自身免疫性溶血患者既有溶血的表现，球形红细胞亦明显增多，易与本病混淆，Coombs试验阳性，肾上腺皮质激素治疗有效等可资鉴别。轻型HS溶血发作时可误为黄疸型肝炎，应注意鉴别。

【治疗】

1．一般治疗 注意防治感染，避免劳累和情绪紧张。适当补充叶酸。

2．防治高胆红素血症 见于新生儿发病者（参阅新生儿黄疸节）。

3．输注红细胞 贫血轻者无需输红细胞，重度贫血或发生溶血危象时应输红细胞。发生再生障碍危象时除输红细胞外，必要时予输血小板。

4．脾切除或大部分脾栓塞 脾切除对常染色体显性遗传病例有显著疗效，术后黄疸消失、贫血纠正，不再发生溶血危象和再生障碍危象，红细胞寿命延长，但不能根除先天缺陷。手术应于5 岁以后进行，因过早切脾可降低机体免疫功能，易发生严重感染。若反复再生障碍危象或重度溶血性贫血致生长发育迟缓时，则手术年龄可提早。切脾时注意有无副脾，如有应同时切除。为防止术后感染，应在术前1～2 周注射多价肺炎球菌疫苗，术后应用长效青霉素预防治疗1 年。脾切除术后血小板数于短期内升高，如PLT>800×10^9/L，应予抗血小板凝集药物如双嘧达莫等。有报告开展大部分脾栓塞或腹腔镜脾切除术治疗HS，近期疗效良好，远期疗效有待进一步观察。

二、红细胞葡萄糖-6-磷酸脱氢酶缺乏症

红细胞葡萄糖-6-磷酸脱氢酶（G-6-PD）缺乏症是一种性连锁不完全显性红细胞酶缺陷病。本病分布遍及世界各地，估计全世界有2 亿以上的人患有G-6-PD缺陷。在我国，此病主要见于长江流域及其以南各省，以云南、海南、广东、广西、福建、四川、江西、贵州等省（自治区）的发病率较高，北方地区较为少见。

【遗传学】

本病是由于调控G-6-PD 的基因突变所致。G-6-PD基因定位于X染色体长臂2区8带（Xq28），全长约18.5Kb，含13 个外显子，编码515 个氨基酸。男性半合子和女性纯合子均表现为G6PD显著缺乏；女性杂合子发病与否，取决于其G-6-PD缺乏的细胞数量在细胞群中所占的比例，在临床上有不同的表现度，故称为不完全显性。

迄今，G-6-PD基因的突变已达122种以上；中国人（含海外华裔）的G-6-PD基因突变型即有17 种，其中最常见的是nt1376G→T（占57.6%）、nt1388G→A（占14.9%），其他突变有nt95A→G、nt493A→G、nt1024G→T等。同一地区的不同民族其基因突变型相似，而分布在不同地区的同一民族其基因突变型则差异很大。

【发病机制】

目前认为服用氧化性药物（如伯氨喹啉）诱发溶血的机制为：G-6-PD在磷酸戊糖旁路中是6-磷酸葡萄糖（G-6-P）转变为6-磷酸葡萄糖酸（G-6-PG）反应中必需的酶。G-6-PD缺乏时，使还原型三磷酸吡啶核苷（NADPH）减少，不能维持生理浓度的还原型谷胱甘肽（GSH）。从而使红细胞膜蛋白和酶蛋白中的巯基遭受氧化，破坏了红细胞膜 的完整性。NAGPH减少后，使高铁血红蛋白（MHb）不能转变为氧合血红蛋白，MHb增加致红细胞内还可溶性变性珠蛋白小体（Heinz body）形成明显增加，红细胞膜变硬，通过脾脏时被破坏，导致溶血。新生的红细胞G-6-PD活性较高，对氧化剂药物有较强的“抵抗性”，当衰老红细胞酶活性过低而被破坏后，新生红细胞即代偿性增加，故不再发生溶血，呈“自限性”。蚕豆诱发溶血的机理未明，蚕豆浸液中含有多巴、多巴胺、蚕豆嘧啶类、异脲咪等类似氧化剂物质，可能与蚕豆病的发病有关，但很多G-6-PD缺乏者在进食蚕豆后并不一定发病，故认为还有其它因素参与，尚有待进一步研究。

【临床表现】

根据诱发溶血的不同原因，可分为以下5 种临床类型。

1．伯氨喹啉型药物性溶血性贫血 是由于服用某些具有氧化特性的药物而引起的急性溶血。此类药物包括：抗疟药（伯氨喹啉、奎宁等），镇痛退热药（阿司匹林、安替比林等），硝基呋喃类，磺胺类药，砜类药，萘苯胺，大剂量维生素K，丙磺舒，川莲，腊梅花等。常于服药后1～3 天出现急性血管内溶血。有头晕、厌食、恶心、呕吐、疲乏等症状，继而出现黄疸、血红蛋白尿，溶血严重者可出现少尿、无尿、酸中毒和急性肾衰竭。溶血过程呈自限性是本病的重要特点，轻症的溶血持续1～2 天或1 周左右临床症状逐渐改善而自愈。

2．蚕豆病 常见于10 岁以下小儿，男孩多见，常在蚕豆成熟季节流行，进食蚕豆或蚕豆制品（如粉丝）均可致病，母亲食蚕豆后哺乳可使婴儿发病。通常于进食蚕豆或其制品后24～48 小时内发病，表现为急性血管内溶血，其临床表现与伯氨喹啉型药物性溶血相似。

3．新生儿黄疸 在G-6-PD缺乏症高发地区由G-6-PD缺乏引起的新生儿黄疸并不少见。感染、病理产、缺氧、给新生儿哺乳的母亲服用氧化剂药物、或新生儿穿戴有樟脑丸气味的衣服等均可诱发溶血，但也有不少病例无诱因可查。黄疸大多于出生2～4 天后达高峰，半数患儿可有肝脾肿大，贫血大多数为轻度或中度，重者可致胆红素脑病。

4．感染诱发的溶血 细菌、病毒感染可诱发G-6-PD缺乏者发生溶血，一般于感染后几天之内突然发生溶血，程度大多较轻，黄疸多不显著。

5．先天性非球形细胞性溶血性贫血（CNSHA） 在无诱因情况下出现慢性溶血，常于婴儿期发病，表现为贫血、黄疸、脾大，可因感染或服药而诱发急性溶血。约有半数病例在新生儿期以高胆红素血症起病。

【实验室检查】

1．红细胞G-6-PD缺乏的筛选试验 常用3 种方法：

（1）高铁血红蛋白还原实验：正常还原率>0.75；中间型为0.74～0.31；显著缺乏者<0.30。此试验可出现假阳性或假阴性，故应配合其他有关实验室检查。

（2）荧光斑点试验：正常10 分钟内出现荧光；中间型者10～30 分钟出现荧光；严重缺乏者30 分钟仍不出现荧光。本试验敏感性和特异性均较高。

（3）硝基四氮唑蓝（NBT）纸片法：正常滤纸片呈紫蓝色，中间型呈淡蓝色，显著缺乏者呈红色。

2．红细胞G-6-PD活性测定 这是特异性的直接诊断方法，正常值随测定方法而不同：

（1）世界卫生组织（WHO）推荐的Zinkham法为12.1 IU/gHb±2.09 IU/gHb。

（2）国际血液学标准化委员会（SICSH）推荐的Clock与Mclean法为8.34 IU/gHb±1.59 IU/gHb。

（3）NBT 定量法为13.1～30.0 BNT单位。

（4）近年开展的G-6-PD/6-PGD比值测定，可进一步提高杂合子检出率，正常值为成人1.0～1.67，脐带血1.1～2.3，低于此值为G-6=PD缺乏。

3．变性珠蛋白小体生成试验 在溶血时阳性细胞>0.05，溶血停止时呈阴性。不稳定血红蛋白病患者此试验亦可为阳性。

【诊断】

阳性家族史或过去病史均有助于临床诊断。病史中有急性溶血特征，并有食蚕豆或服药物史，或新生儿黄疸，或自幼即出现原因未明的慢性溶血者，均应考虑本病。结合实验室检查即可确诊。

【治疗】

对急性溶血者，应去除诱因。在溶血期应供给足够水份，注意纠正电解质失衡，口服碳酸氢钠，使尿液保持碱性，以防止血红蛋白在肾小管内沉积。贫血较轻者不需要输血，去除诱因后溶血大多于1 周内自行停止。严重贫血时，可输给G-6-PD正常的红细胞1～2 次。应密切注意肾功能，如出现肾功能衰竭，应及时采取有效措施。

新生儿黄疸可用蓝光治疗，个别严重者应考虑换血疗法，以防止胆红素脑病的发生。

【预防】

在G-6-PD缺陷高发地区，应进行群体G-6-PD缺乏症的普查；已知为G-6-PD缺乏者应避免进食蚕豆及其制品，忌服有氧化作用的药物，并加强对各种感染的预防。

三、地中海贫血

地中海贫血又称海洋性贫血（thalassemia）、珠蛋白生成障碍性贫血，是遗传性溶血性贫血的一组疾病。其共同特点是珠蛋白基因的缺陷使一种或几种珠蛋白肽链合成减少或不能合成，导致血红蛋白的组成成分改变。本组疾病的临床症状轻重不一。

本病以地中海沿岸国家和东南亚各国多见，我国长江以南各省均有报道，以广东、广西、海南、四川、重庆等省区发病率较高，在北方较为少见。

【病因和发病机制】

正常人血红蛋白（Hb）中的珠蛋白含四种肽链，即α 、β 、γ 和δ 。根据珠蛋白肽链组合的不同形成三种血红蛋白，即HbA（$\alpha_2\beta_2$）、HbA_2（$\alpha_2\delta_2$）和HbF（$\alpha_2\gamma_2$）。当遗传缺陷时，珠蛋白基因缺失或点突变后，珠蛋白肽链合成障碍，从而出现慢性溶血性贫血。根据肽链合成障碍的不同，分别称为α 、β 、δβ 和δ 等地中海贫血。其中以β 和α 地中海贫血较为常见。

1．β地中海贫血 人类β珠蛋白基因簇位于第11号染色体短臂1区2节（11p1.2）。β地中海贫血（简称β地贫）的病因主要是由于该基因的点突变，少数为基因缺失。基因缺失和有些点突变可致β链的生成完全受抑制，称为β^0 地贫；有些点突变或缺失使β链的生成部分受抑制，则称为β^+地贫。染色体上的二个等位基因突变点相同者称为纯合子；同源染色体上只有一个突变点者称为杂合子；等位基因的突变点不同者称为双重杂合子。

重型β 地贫是β^0 或β^+ 地贫的纯合子或β^0 与β^+ 地贫双重杂合子状态。因β链生成完全或几乎完全受到抑制，以致含有β 链的HbA合成减少或消失，而多余的α 链则与γ 链结合而成为HbF（$\alpha_2\gamma_2$），使HbF明显增加。由于HbF的氧亲合力高，致患者组织缺氧。过剩的α 链沉积于幼红细胞和红细胞中，形成α 链包涵体附着于红细胞膜上而使其变僵硬，在骨髓内大多被破坏而导致“无效造血”。部分含有包涵体的红细胞虽能成熟并被释放至外周血，但当它们通过微循环时就容易被破坏；这种包涵体还影响红细胞膜的通透性，从而导致红细胞的寿命缩短。所以，患儿在临床上呈慢性溶血性贫血。贫血和缺氧刺激红细胞生成素的分泌量增加，促使骨髓增加造血，因而引起骨骼的改变。贫血使肠道对铁的吸收增加，加上在治疗过程中的反复输血，使铁在组织中大量贮存，导致含铁血黄素沉着症。

轻型地贫是β^0 或β^+ 地贫的杂合子状态，β 链的合成仅轻度减少，故其病理生理改变极轻微。中间型β 地贫是一些β^+地贫的双重杂合子和某些地贫变异型的纯合子或双重杂合子状态，其病理生理改变介于重型和轻型之间。

2．α 地中海贫血 人类α珠蛋白基因簇位于第16号染色体短臂末端（16p13.3）。每条染色体各有2个α 珠蛋白基因，一对染色体共有4 个α珠蛋白基因。大多数α 地中海贫血（简称α 地贫）是由于α 珠蛋白基因的缺失所致，少数由基因点突变造成。若仅是一条染色体上的一个α 基因缺失或缺陷，则α 链的合成部分受抑制，称为α^+地贫；若每一条染色体上的2 个α 基因均缺失或缺陷，则无α 链合成，称为α^0地贫。

重型α 地贫是α^0 地贫的纯合子状态，其4个α 珠蛋白基因均缺失或缺陷，以致完全无α 链生成，因而含有α 链的HbA、HhA_2和HbF的合成均减少。患者在胎儿期即发生大量γ 链合成γ_4（HbBart’s）。HbBart’s对氧的亲合力极高，造成组织缺氧而引起胎儿水肿综合征。中间型α 地贫是α^0 和α^+ 地贫的双重杂合子状态，是由3 个α 珠蛋白基因缺失或缺陷所造成，患者仅能合成少量α 链，其多余的β链即合成HbH（β_4）。HbH对氧亲合力较高，又是一种不稳定血红蛋白，容易在红细胞内变性沉淀而形成包涵体，造成红细胞膜僵硬而使红细胞寿命缩短。

轻型α 地贫是α^+ 地贫纯合子或α^0地贫杂合子状态，它仅有2 个α 珠蛋白基因缺失或缺陷，故有相当数量的α 链合成，病理生理改变轻微。静止型α 地贫是α^+ 地贫杂合子状态，它仅有一个α基因缺失或缺陷，α 链的合成略为减少，病理生理改变非常轻微。

【临床表现和实验室检查】

1．β 地中海贫血 根据病情轻重的不同，分为以下3型。

（1）重型：又称Cooley贫血。患儿出生时无症状，至3～12个月开始发病，呈慢性进行性贫血，面色苍白，肝脾大，发育不良，常有轻度黄疸，症状随年龄增长而日益明显。由于骨髓代偿性增生导致骨骼变大、髓腔增宽，先发生于掌骨，以后为长骨和肋骨；1 岁后颅骨改变明

显，表现为头颅变大、额部隆起、颧高、鼻梁塌陷，两眼距增宽，形成地中海贫血特殊面容。患儿常并发支气管炎或肺炎。当并发含铁血黄素沉着症时，因过多的铁沉着于心肌和其它脏器如肝、胰腺、垂体等而引起该脏器损害的相应症状，其中最严重的是心力衰竭，它是贫血和铁沉着造成心肌损害的结果，是导致患儿死亡的重要原因之一。本病如不治疗，多于5 岁前死亡。

实验室检查：外周血象呈小细胞低色素性贫血，红细胞大小不等，中央浅染区扩大，出现异形、靶形、碎片红细胞和有核红细胞、点彩红细胞、嗜多染性红细胞、豪-周氏小体等；网织红细胞正常或增高。骨髓象呈红细胞系统增生明显活跃，以中、晚幼红细胞占多数，成熟红细胞改变与外周血相同。红细胞渗透脆性明显减低。HbF含量明显增高，大多>0.40，这是诊断重型β 地贫的重要依据。颅骨X线片可见颅骨内外板变薄，板障增宽，在骨皮质间出现垂直短发样骨刺。

（2）轻型：患者无症状或轻度贫血，脾不大或轻度大。病程经过良好，能存活至老年。本病易被忽略，多在重型患者家族调查时被发现。

实验室检查：成熟红细胞有轻度形态改变，红细胞渗透脆性正常或减低，血红蛋白电泳显示HbA_2含量增高（0.035～0.060），这是本型的特点。HbF含量正常。

（3）中间型：多于幼童期出现症状，其临床表现介于轻型和重型之间，中度贫血，脾脏轻或中度大，黄疸可有可无，骨骼改变较轻。

实验室检查：外周血象和骨髓象的改变如重型，红细胞渗透脆性减低，HbF含量约为0.40～0.80，HbA_2含量正常或增高。

2． α 地中海贫血

（1）静止型：患者无症状。红细胞形态正常，出生时脐带血中Hb Bart's含量为0.01～0.02，但3 个月后即消失。

（2）轻型 ：患者无症状。红细胞形态有轻度改变，如大小不等、中央浅染、异形等；红细胞渗透脆性降低；变性珠蛋白小体阳性；HbA_2和HbF含量正常或稍低。患儿脐血Hb Bart's含量为0.034～0.140，于生后6 个月时完全消失。

（3）中间型：又称血红蛋白H病。患儿出生时无明显症状；婴儿期以后逐渐出现贫血、疲乏无力、肝脾大、轻度黄疸；年龄较大患者可出现类似重型β 地贫的特殊面容。合并呼吸道感染或服用氧化性药物、抗疟药物等可诱发急性溶血而加重贫血，甚至发生溶血危象。

实验室检查：外周血象和骨髓象的改变类似重型β 地贫；红细胞渗透脆性减低；变性珠蛋白小体阳性；HbA_2及HbF含量正常。出生时血液中含有约0.25 Hb Bart's及少量HbH；随年龄增长，HbH逐渐取代Hb Bart's，其含量约为0.024～0.44。包涵体生成试验阳性。

（4）重型：又称Hb Bart's胎儿水肿综合征。胎儿常于30～40 周时流产、死胎或娩出后半小时内死亡，胎儿呈重度贫血、黄疸、水肿、肝脾肿大、腹水、胸水。胎盘巨大且质脆。

实验室检查：外周血成熟红细胞形态改变如重型β地贫，有核红细胞和网织红细胞明显增高。血红蛋白中几乎全是Hb Bart's或同时有少量HbH，无HbA、HbA_2和HbF。

【诊断与鉴别诊断】

根据临床特点和实验室检查，结合阳性家族史，一般可作出诊断。有条件时，可作基因诊断。本病须与下列疾病鉴别。

1．缺铁性贫血 轻型地中海贫血的临床表现和红细胞的形态改变与缺铁性贫血有相似之处，故易被误诊。但缺铁性贫血常有缺铁诱因，血清铁蛋白含量减低，骨髓外铁粒幼红细胞减少，红细胞游离原卟啉升高，铁剂治疗有效等可资鉴别。对可疑病例可借助于血红蛋白碱变性

试验和血红蛋白电泳。

2．遗传性球形细胞增多症 见本节遗传性球形细胞增多症。

3．传染性肝炎或肝硬化 因HbH病贫血较轻，还伴有肝脾肿大、黄疸，少数病例还可有肝功能损害，故易被误诊为黄疸型肝炎或肝硬化。但通过病史询问、家族调查以及红细胞形态观察、血红蛋白电泳检查即可鉴别。

【治疗】

轻型地贫无需特殊治疗。中间型和重型地贫应采取下列一种或数种方法给予治疗。

1．一般治疗 注意休息和营养，积极预防感染。适当补充叶酸和维生素E。

2．输血和去铁治疗 此法在目前仍是重要治疗方法之一。

（1）红细胞输注：少量输注法仅适用于中间型α 和β 地贫，不主张用于重型β 地贫。对于重型β 地贫应从早期开始给予中、高量输血，以使患儿生长发育接近正常和防止骨骼病变。其方法是：先反复输注浓缩红细胞，使患儿血红蛋白含量达120～150 g/L；然后每隔2～4 周输注浓缩红细胞10～15 ml/kg，使血红蛋白含量维持在90～105 g/L以上。但本法容易导致含铁血黄素沉着症，故应同时给予铁螯合剂治疗。

（2）铁螯合剂：常用去铁胺（deferoxamine），可以增加铁从尿液和粪便排出，但不能阻止胃肠道对铁的吸收。通常在规则输注红细胞1 年或10～20 单位后进行铁负荷评估，如有铁超负荷（例如SF>1000μg/L），则开始应用铁螯合剂。去铁胺每日25～50 mg/kg，每晚1 次连续皮下注射12 小时，或加入等渗葡萄糖液中静滴8～12 小时；每周5～7 天，长期应用。或加入红细胞悬液中缓慢输注。去铁胺副作用不大，偶见过敏反应，长期使用偶可致白内障和长骨发育障碍，剂量过大可引起视力和听觉减退。维生素C与螯合剂联合应用可加强去铁胺从尿中排铁的作用，剂量为200 mg/d。

3．脾切除 脾切除对血红蛋白H病和中间型β 地贫的疗效较好，对重型β地贫效果差。脾切除可致免疫功能减弱，应在5～6 岁以后施行并严格掌握适应证。

4．造血干细胞移植 异基因造血干细胞移植是目前能根治重型β 地贫的方法。如有HLA相配的造血干细胞供者，应作为治疗重型β 地贫的首选方法。

5．基因活化治疗 应用化学药物可增加γ 基因表达或减少α 基因表达，以改善β 地贫的症状，已用于临床的药物有羟基脲、5-氮杂胞苷（5-AZC）、阿糖胞苷、白消安、异烟肼等，目前正在探索之中。

【预防】

开展人群普查和遗传咨询、作好婚前指导以避免地贫基因携带者之间联姻，对预防本病有重要意义。采用基因分析法进行产前诊断，可在妊娠早期对重型β 和α 地贫胎儿作出诊断并及时中止妊娠，以避免胎儿水肿综合征的发生和重型β 地贫患者出生，是目前预防本病行之有效的方法。

（方建培）

第五节 出血性疾病

一、特发性血小板减少性紫癜

特发性血小板减少性紫癜（idiopathic thrombocytopenic purpura，ITP）又称自身免疫性血小

板减少性紫癜（immune thrombocytopenic purpura，ITP），是小儿最常见的出血性疾病。其主要临床特点是：皮肤、黏膜自发性出血和束臂实验阳性，血小板减少、出血时间延长和血块收缩不良。

【病因与发病机制】

患儿在发病前常有病毒感染史。目前认为病毒感染不是导致血小板减少的直接原因，而是由于病毒感染后使机体产生相应的抗体，这类抗体可与血小板膜发生交叉反应，使血小板受到损伤而被单核-巨噬细胞系统所清除。此外，在病毒感染后，体内形成的抗原-抗体复合物可附着于血小板表面，使血小板易被单核-巨噬细胞系统吞噬和破坏，使血小板的寿命缩短，导致血小板减少。患者血清中血小板相关抗体（PAIgG）含量多增高。研究证实，辅助性T细胞（Th）和细胞毒T细胞（CTL）的活化及相关细胞因子紊乱是导致本病慢性化过程的重要原因。现已知道，血小板和巨核细胞有共同抗原性，抗血小板抗体同样作用于骨髓中巨核细胞，导致巨核细胞成熟障碍，巨核细胞生成和释放均受到严重影响，使血小板进一步减少。

【临床表现】

本病见于各年龄时期小儿，多见于1～5 岁小儿，男女发病数无差异，春季发病数较高。急性型患儿于发病前1～3 周常有急性病毒感染史，如上呼吸道感染、流行性腮腺炎、水痘、风疹、麻疹、传染性单核细胞增多症等，亦偶见于免疫接种之后。大多数患儿发疹前无任何症状，部分可有发热。以自发性皮肤和黏膜出血为突出表现，多为针尖大小的皮内或皮下出血点，或为瘀斑和紫癜，少见皮肤出血斑和血肿。分布不均，通常以四肢为多，在易于碰撞的部位更多见。常伴有鼻出血或齿龈出血，胃肠道大出血少见，偶见肉眼血尿。青春期女性患者可有月经过多。少数患者可有结膜下和视网膜出血。颅内出血少见，如一旦发生，则预后不良。出血严重者可致贫血，肝脾偶见轻度肿大，淋巴结不肿大。

大约80％～90％的患儿于发病后1～6 个月内痊愈，10％～20％的患儿呈慢性病程。其中部分患者经正规糖皮质激素、脾切除和一般免疫抑制剂治疗无效称为难治性ITP。病死率约为0.5％～1％，主要致死原因为颅内出血。

【实验室检查】

1．外周血象 血小板计数<100×10^9/L，出血轻重与血小板数多少有关，血小板<50×10^9/L时可见自发性出血，<20×10^9/L时出血明显，<10×10^9/L时出血严重。慢性型者可见血小板大小不等，染色较浅。失血较多时可致贫血，白细胞数正常。出血时间延长，凝血时间正常，血块收缩不良。血清凝血酶原消耗不良。

2．骨髓象 急性骨髓巨核细胞数增多或正常。慢性型巨核细胞显著增多，幼稚巨核浆细胞增多，核分叶减少，核-浆发育不平衡，产生血小板的巨核细胞明显减少，其细胞质中有空泡形成、颗粒减少和量少等现象。

3．血小板抗体测定 主要是PAIgG增高，但PAIgG增高并非ITP的特异性改变，其它免疫性疾病亦可增高。如同时检测PAIgM和PAIgA，以及结合在血小板表面的糖蛋白、血小板内的抗GPⅡb/Ⅲa自身抗体和GPⅠb/Ⅸ自身抗体等可提高临床诊断的敏感性和特异性。

4．血小板寿命测定 经放射性核素^{51}Cr或^{111}In标记血小板测定其寿命，发现患者血小板存活时间明显缩短，甚至只有数小时（正常为8～10 天），一般不作为常规检查。

5．其他 束臂试验阳性，慢性ITP患者的血小板黏附和聚集功能可以异常。

【诊断与鉴别诊断】

根据病史、临床表现和实验室检查，即可作出诊断。临床上主要根据病程的长短将本症分

为两型：≤6 个月为急性型，>6 个月为慢性型，两者的鉴别见表15-2。

表15-2 急性与慢性特发性血小板减少性紫癜的鉴别

	急 性 型	慢 性 型
发病年龄	1～5岁多见	学龄期多见
起病	较急	较缓
出血程度	较重	较轻
病程	≤6 个月	>6 个月
血小板数	大多<20×10^9/L	一般（30～80）$\times10^9$/L
骨髓巨核细胞	计数正常或增多，胞体大小不一，以小型为多，幼稚巨核细胞比例正常或稍高，产血小板巨核细胞减少	计数明显增多，核浆发育不平衡，细胞浆出现空泡变性，产血小板巨核细胞明显减少

本症还需与下列疾病相鉴别：

1．急性白血病 外周血白细胞不增高的急性白血病易与ITP相混淆，通过血涂片和骨髓检查见到白血病细胞即可确诊。

2．再生障碍性贫血 患者表现为发热、贫血和出血，肝、脾和淋巴结不肿大，与ITP合并贫血者相似。但再生障碍性贫血时贫血较重，外周血白细胞数和中性粒细胞数减少，骨髓造血功能减低，巨核细胞减少有助于诊断。

3．过敏性紫癜 为出血性斑丘疹，对称分布，成批出现，多见于下肢和臀部，血小板数正常，一般易于鉴别。

4．继发性血小板减少性紫癜 严重细菌感染和病毒血症均可引起血小板减少，化学药物、脾功能亢进、部分自身免疫性疾病（如系统性红斑狼疮等）、恶性肿瘤侵犯骨髓和某些溶血性贫血等均可导致血小板减少，应注意鉴别。

【治疗】

1．一般治疗 在急性出血期间以住院治疗为宜，尽量减少活动，避免外伤，明显出血时应卧床休息。应积极预防及控制感染，避免服用影响血小板功能的药物（如阿司匹林等）。

2．糖皮质激素 其主要药理作用是：降低毛细血管通透性；抑制血小板抗体产生；抑制单核-巨噬细胞系统破坏有抗体吸附的血小板。常用泼尼松，剂量为每日1.5～2 mg/kg，分3 次口服。出血严重者可用冲击疗法：地塞米松每日0.5～2 mg/kg，或甲基泼尼松龙每日20～30 mg/kg，静脉滴注，连用3 天，症状缓解后改口服泼尼松。用药至血小板数回升至接近正常水平即可逐渐减量，疗程一般不超过4 周。停药后如有复发，可再用泼尼松治疗。

3．大剂量静脉丙种球蛋白 其主要作用是：①封闭巨噬细胞受体，抑制巨噬细胞对血小板的结合与吞噬，从而干扰单核-巨噬细胞吞噬血小板的作用；②在血小板上形成保护膜抑制血浆中的IgG或免疫复合物与血小板结合，从而使血小板免受吞噬细胞破坏；③抑制自身免疫反应，使抗血小板抗体减少。单独应用大剂量静脉丙种球蛋白的升血小板效果与激素相似，常用剂量为每日0.4～0.5 g/kg，连续5 天静脉滴注；或每次1 g/kg静脉滴注，必要时次日可再用1 次；以后每3～4 周1 次。副作用少，偶有过敏反应。

4．血小板输注 因患儿血循环中含有大量抗血小板抗体，输入血小板很快被破坏，故通常不主张输血小板；只有在发生颅内出血或急性内脏大出血危及生命时才输注血小板，并需同时予以大剂量肾上腺皮质激素，以减少输入血小板被破坏。

5．抗-D免疫球蛋白（anti-D immunoglobulin） 又称抗Rh球蛋白，其作用机制尚未完全清楚，主要作用是封闭网状内皮细胞的Fc受体。其升高血小板作用较糖皮质激素和大剂量丙种球蛋白慢，但持续时间长。常用剂量为每日25～50 μg/kg，静脉注射，连用5 天为1 疗程。主要副作用是轻度溶血性输血反应和Coombs试验阳性。

6．脾切除 脾切除有效率约70％，适用于病程超过1 年，血小板持续<50×10^9/L（尤其是<20×10^9/L），有较严重的出血症状，内科治疗效果不好者，手术宜在6 岁以后进行。10 岁以内发病的患者，其5 年自然缓解机会较大，尽可能不作脾切除。术前必须作骨髓检查，巨核细胞数减少者不宜作脾切除。术前PAIgG极度增高者，脾切除的疗效亦较差。

7．部分性脾栓塞术 选择性插导管至脾门部脾动脉，经导管向脾动脉内注入直径300～550t μm的聚乙烯微粒，阻断脾脏外周皮质的供血动脉，保留脾脏中心部的髓质供血动脉，使脾脏皮质缺血、坏死、液化并逐渐吸收，达到部分切除脾脏之目的。部分性脾栓塞术后2 小时，血小板即可明显升高。由于保留了脾脏的髓质即保留了脾脏的免疫功能，部分性脾栓塞术尤适应于儿童期糖皮质激素治疗无效的ITP。

8．免疫抑制剂 目前主要用于治疗慢性型ITP。环孢素A 3～5 mg/（kg·d），分2～3次口服，开始治疗剂量可稍大，应根据血药浓度调整剂量，疗程3～4 个月，主要副作用是肝肾功能损害。其他如长春新碱0.75～1 mg/（m^2·次），加0.9%氯化钠溶液静脉注射，每周1 次，可连续用4～6 次；环磷酰胺300～400 mg/（m^2·次），加5%葡萄糖溶液静滴，每1～2 周1 次，可连续用3～4 次。亦可用硫唑嘌呤1.5～2.5 mg/（kg·d），口服8～12 周，观察疗效。对儿童慢性ITP应用细胞毒药物治疗一定要慎重，对其利弊要做综合评价。

9．其他 达那唑（danazol）是一种合成的雄性激素，对部分病例有效，剂量为每日10～15 mg/kg，分次口服，连用2～4 个月。干扰素-α2b对部分顽固病例有效，剂量为每次5 万～10 万单位/kg，皮下或肌肉注射，每周3 次，连用3 个月。

二、血友病

血友病（hemophilia）是一组遗传性凝血功能障碍的出血性疾病，包括：①血友病甲，即因子Ⅷ（又称抗血友病球蛋白，AHG）缺乏症；②血友病乙，即因子Ⅸ（又称血浆凝血活酶成分，PTC）缺乏症；③血友病丙，即因子Ⅺ（又称血浆凝血活酶前质，PTA）缺乏症。其发病率为5～10/10万，以血友病甲较为常见（占80%～85%），血友病乙次之，血友病丙罕见。其共同特点为终生在轻微损伤后发生长时间出血。

【病因和发病机制】

血友病甲和乙为X-连锁隐性遗传，由女性传递、男性发病。血友病丙为常染色体不完全性隐性遗传，男女均可发病或传递疾病。

因子Ⅷ、Ⅸ、Ⅺ缺乏均可使凝血过程第一阶段中的凝血活酶生成减少，引起血液凝固障碍，导致出血倾向。因子Ⅷ是血浆中的一种球蛋白（其抗原为Ⅷ：Ag，功能部分称为Ⅷ：C），它与von Willebrand Factor（vWF）以非共价形式结合成复合物存在于血浆中。因子Ⅷ和vWF是由不同基因编码、性质和功能完全不同的二种蛋白质。Ⅷ：C 仅占复合物的1％，水溶性，80％由肝脏合成，余20％由脾、肾和单核-巨噬细胞等合成，其活性易被破坏，在37℃储存24 小时后可丧失50％。vWF由血管内皮细胞合成，其功能主要有：①作为因子Ⅷ的载体而对因子Ⅷ起稳定作用；②参与血小板黏附和聚集功能。vWF缺乏时，可引起出血和因子Ⅷ缺乏。

因子Ⅸ是一种由肝脏合成的糖蛋白，在其合成过程中需要维生素K的参与。因子Ⅺ也是在肝内合成，在体外储存时其活性稳定，故给本病患者输适量储存血浆即可补充因子Ⅺ。

【临床表现】

出血症状的轻重及发病的早晚与凝血因子活性水平相关。血友病甲和乙大多在2 岁时发病，亦可在新生儿期即发病。血友病丙的出血症状一般较轻。

1．皮肤、黏膜出血 由于皮下组织、口腔、齿龈黏膜易于受伤，为出血好发部位。幼儿亦常见于头部碰撞后出血和血肿。

2．关节积血 是血友病最常见的临床表现之一，多见于膝关节，其次为踝、髋、肘、肩关节等。关节出血可以分为3 期：①急性期：关节腔内及周围组织出血，引起局部红肿、热痛和功能障碍。由于肌肉痉挛，关节多处于屈曲位置。②关节炎期：因反复出血、血液不能完全被吸收，刺激关节组织，形成慢性炎症，滑膜增厚。③后期：关节纤维化、强硬、畸形、肌肉萎缩、骨质破坏，导致功能丧失。膝关节反复出血，常引起膝屈曲、外翻、腓骨半脱位，形成特征性的血友病步态。

3．肌肉出血和血肿 重型血友病甲常发生肌肉出血和血肿，多发生在创伤或活动过久后，多见于用力的肌群。深部肌肉出血时可形成血肿，导致局部肿痛和活动受限，可引起局部缺血性损伤和纤维变性。在前臂可引起手挛缩，小腿可引起跟腱缩短，腰肌痉挛可引起下腹部疼痛。

4．创伤或手术后出血 不同程度的创伤、小手术，如拔牙、扁桃体摘除、脓肿切开、肌肉注射或针灸等，均可以引起严重的出血。

5．其他部位的出血 如鼻出血、咯血、呕血、黑便、血便和血尿等；也可发生颅内出血，是最常见的致死原因之一。

血友病乙的出血症状与血友病甲相似，患者多为轻型，出血症状较轻。

血友病丙较为少见，杂合子患儿无出血症状，只有纯合子者才有出血倾向。出血多发生于外伤或手术后，自发性出血少见。本病患儿常合并Ⅴ、Ⅶ等其他因子缺乏。

【实验室检查】

1．血友病甲、乙和丙实验室检查的共同特点是：①凝血时间延长（轻型者正常）；②凝血酶原消耗不良；③活化部分凝血活酶时间延长；④凝血活酶生成试验异常。出血时间、凝血酶原时间和血小板正常。

2．当凝血酶原消耗试验和凝血活酶生成试验异常时，为了进一步鉴别三种血友病，可作纠正试验，其原理为：正常血浆经硫酸钡吸附后尚含有因子Ⅷ和Ⅺ，不含因子Ⅸ，正常血清含有因子Ⅸ和Ⅺ，不含因子Ⅷ。据此，如患者凝血酶原消耗时间和凝血活酶生成时间被硫酸钡吸附后的正常血浆所纠正，而不被正常血清纠正，则为血友病甲；如以上两试验被正常血清所纠正而不被经硫酸钡吸附的正常血浆纠正，则为血友病乙；若以上两试验可被正常血清和经硫酸钡吸附正常血浆所纠正，则为血友病丙（表15-3）。

表15-3 血友病甲、乙和丙凝血纠正试验

患者血浆加入	血友病甲	血友病乙	血友病丙
正常血浆	纠正	纠正	纠正
正常血清	不能纠正	纠正	纠正
经硫酸钡吸附的正常人血浆	纠正	不能纠正	纠正

测定因子Ⅷ：C、因子Ⅸ：C的活性，对血友病甲或血友病乙有确诊意义。正常新鲜血浆所含因子Ⅷ：C或因子Ⅸ：C平均活性均为1 u/ml（以100％表示），根据因子Ⅷ：C或因子Ⅸ：C活性水平的高低，将血友病甲或血友病乙分为重型（<1％）、中型（1％～5％）、轻型（>5％～25％）及亚临床型（>25％～45％）4 种临床类型。

3．基因诊断 利用分子生物学技术，发现血友病患者基因突变位点和形式，并可于产前进行胎儿基因诊断。

【诊断和鉴别诊断】

根据病史、出血症状和家族史，即可考虑为血友病，进一步确诊须作有关实验室检查。基因序列分析除可确诊本病外，尚可发现轻症患者和疾病携带者。血友病须与血管性血友病鉴别，后者出血时间延长、阿司匹林耐量试验阳性、血小板粘附率降低、血小板对瑞斯托霉素无凝集反应、血浆Ⅷ：C减少或正常、血浆vWF减少或缺乏。此外，血管性血友病为常染色体显性遗传，家族调查亦有助于鉴别。

【治疗】

1．预防出血 自幼养成安静生活习惯，以减少和避免外伤出血，尽可能避免肌肉注射，如因患外科疾病需作手术治疗，应注意在术前、术中和术后补充所缺乏的凝血因子。

2．局部止血 对表面创伤、鼻或口腔出血可局部压迫止血，或用纤维蛋白泡沫、明胶海绵沾组织凝血活酶或凝血酶敷于伤口处。早期关节出血者，宜卧床休息，并用夹板固定肢体，放于功能位置，亦可用局部冷敷，并用弹力绷带缠扎。关节出血停止、肿痛消失时，可作适当体疗，以防止关节畸形。严重关节畸形可用手术矫形治疗。

3．替代疗法 本疗法的目的是将患者所缺乏的因子提高到止血水平，以治疗或预防出血。早期给予适当的补充治疗是血友病患儿最好的保护措施，年龄较大的儿童及家人学会居家照顾，急性出血时最好在家里治疗。

（1）因子Ⅷ和因子Ⅸ制剂：一些用新技术提取并经灭毒处理的高纯度因子Ⅷ和因子Ⅸ目前已完全取代了传统的制剂，从而大大地增加了治疗的安全性。近年基因工程重组人因子Ⅷ和因子Ⅸ制剂已应用于临床。因子Ⅷ的半衰期为8～12 小时，需每12 小时输注1 次，每输入1 U/kg可提高血浆因子Ⅷ活性约2%；因子Ⅸ的半衰期为18～24 小时，常24 小时输注1 次，每输入1 U/kg可提高血浆因子Ⅸ活性约1%。各种出血情况时因子Ⅷ和因子Ⅸ用量参见表15-4。

表15-4 因子Ⅷ和因子Ⅸ的剂量和使用方法

出 血 程 度	因 子 Ⅷ	因 子 Ⅸ
早期轻度出血	10～15 U/kg，q12h，共1～3 次	15～30 U/kg，qd，共1～3 次
中度出血（明显关节出血、轻度创伤）	20 U/kg，q12h，连用2 日后可隔日应用，直至止血	30 U/kg，qd，直至止血
重度出血（颅内出血、严重出血、严重创伤、大手术等）	首日每次50 U/kg，q12h，然后维持因子Ⅷ活性>50% 5～7 日，必要时再维持因子Ⅷ活性>30% 5～7 日	首日80 U/kg，以后维持因子Ⅸ活性>40% 5～7 日，必要时再维持因子Ⅸ活性>30% 5～7 日

（2）冷沉淀物：冷沉淀制剂通常以200 ml血浆制成，每袋容量为20 ml，含因子Ⅷ和因子XIII各80～100 U、纤维蛋白原250 mg、一定量的vWF及其他沉淀物。用于血友病甲和血管性血友病（vWD）等的治疗，要求与受血者ABO血型相同或相容，剂量和方法参阅表15-4。

（3）凝血酶原复合物：含有因子Ⅱ、Ⅶ、Ⅸ、Ⅹ，可用于血友病乙的治疗。

（4）输血浆或新鲜全血：血友病甲患者需输给新鲜血浆或冰冻新鲜血浆，按1 ml血浆含因子Ⅷ 1 U计算；血友病乙患者可输储存5天以内血浆，一次输入量不宜过多，以每次10 ml/kg为宜。无条件时，可输给6 小时内采集的全血，每次10 ml/kg，可提高患者血中因子Ⅷ活性10%。输血的疗效只能维持2 天左右，仅适用于轻症患儿。

约15％血友病甲患者经反复因子Ⅷ替代治疗后，血浆中会出现抗因子Ⅷ抗体，如输注常规剂量因子Ⅷ后无效者，常提示有因子Ⅷ抗体存在。对这些患者治疗方法是：①增加因子Ⅷ剂量达原剂量一倍以上，其中部分用于中和抗体，余下部分发挥止血作用；②活化因子Ⅶ（Ⅶa）或活化凝血酶原复合物，因Ⅶa可直接与组织因子共同作用活化因子X（Xa），从而促使凝血活酶的形成；③大剂量丙种球蛋白静脉输注；④免疫抑制剂，如环磷酰胺；⑤用链球菌蛋白A吸附抗体。因子Ⅸ抗体发生率低，如发生时，可加大因子Ⅸ剂量，即达到止血目的。

4．药物治疗

（1）1-脱氧-8-精氨酸加压素（DDAVP）：有提高血浆内因子Ⅷ活性和抗利尿作用，常用于治疗轻型血友病甲患者，可减轻其出血症状，剂量为0.2～0.3 μg/kg，溶于20 ml生理盐水中缓慢静注，此药能激活纤溶系统，故需与6-氨基己酸或氨甲环酸联用。如用滴鼻剂（100 μg/ml），0.25 ml/次，作用相同。

（2）性激素：雄性化激素达那唑（danazol）和女性避孕药复方炔诺酮均有减少血友病甲患者出血的作用，但其疗效均逊于替代疗法。

5．基因治疗 正在进行动物实验和临床前期验证。随着研究的不断深入，基因治疗可能成为治愈血友病的有效手段。

【预防】

根据本组疾病的遗传方式，应对患者的家族成员进行筛查，以确定可能的其他患者和携带者，通过遗传咨询，使他们了解遗传规律。运用现代诊断技术对家族中的孕妇进行基因分析和产前诊断，如确定胎儿为血友病，可及时终止妊娠。在医生指导下，对血友病患儿进行有计划的家庭治疗非常重要，尤其适合我国国情。除病情不稳定和3 岁以下婴幼儿外，其他患者均可家庭治疗。患者及其家属应接受本病相关知识的培训，当发生出血时，应及时采取有效的治疗；对于重症患儿，亦可采取预防性治疗以预防血肿形成和关节畸形。

三、弥散性血管内凝血

弥散性血管内凝血（disseminated intravascular coagulation，DIC）是由多种病因所引起、发生于许多疾病过程中的一种获得性出血综合征。其主要特征是在某些致病因素作用下，血液凝固机制被激活，凝血功能亢进，在毛细血管和（或）小动脉、小静脉内有大量纤维蛋白沉积和血小板凝集，形成广泛的微血栓。由于凝血过程加速，消耗了大量的血浆凝血因子和血小板，同时激活了纤维蛋白溶解系统，引起继发性纤维蛋白溶解亢进，从而导致广泛性出血、循环障碍、栓塞和溶血等一系列临床表现。

【病因和发病机制】

1．病因 许多疾病或理化因素都可诱发DIC，主要有：①各种感染，包括细菌、病毒、疟原虫等；②组织损伤，如严重外伤或挤压伤、颅脑损伤、大面积烧伤、大手术和产科并发症等；③免疫性疾病，如溶血性输血反应、暴发型紫癜、狼疮肾炎等；④新生儿疾病，如新生儿硬肿症、窒息、呼吸窘迫综合征、新生儿溶血症等；⑤恶性肿瘤，如白血病、恶性淋巴瘤等；⑥巨大血管瘤、动脉瘤、急性出血性坏死性小肠炎等。

2．发病机制 目前认为血管内皮细胞（endothelium of blood vessels）损伤在内毒素致DIC的过程中发挥关键作用。血管内皮细胞可以合成和释放多种生物活性物质，在生理条件下，血管内皮细胞主要表现抗血栓形成特征。引起DIC的病因，如内毒素、严重感染、免疫复合物、酸中毒和游离脂肪酸等都可损伤血管内皮细胞，致使内皮下组织暴露，从而激活因子Ⅻ，继而

启动内源性凝血系统；同时损伤的血管内皮细胞可释放多种生物活性物质，激活外源性凝血系统，促进止血或血栓形成以及炎症过程的发展。DIC的病因复杂，但都与血管内皮损伤伴血浆凝血因子活化和凝血活酶类物质进入血液有关，可以概括地分为下述两个基本病理过程。

（1）凝血系统被激活：在上述致病因子作用下，机体产生白介素（IL）-6 和IL-1 、肿瘤坏死因子、血小板活化因子等多种前炎症因子，促使组织因子释放，导致血管内皮细胞损伤。内毒素可诱发单核细胞产生组织因子，组织损伤可直接释放组织因子，红细胞和血小板损伤可直接释放促凝物质。组织因子结合并活化因子Ⅶ，进而激活外源凝血系统，这是DIC发病的最重要机制。内皮细胞损伤后胶原组织暴露、活化因子Ⅶ，或直接活化因子XI，进而激活内源凝血系统。凝血系统激活后产生大量病理性凝血酶，使血液呈高凝状态，导致微循环内广泛血栓形成。

单核-巨噬细胞功能损伤不能及时清除血循环内的凝血酶等凝血物质；代谢性酸中毒可使血管内皮损伤并抑制肝素的抗凝作用；循环障碍时因血液淤滞和浓缩易使血小板破坏，这些因素均可诱发或加重DIC。

在凝血系统被激活的同时，体内生理抗凝血因子被消耗和功能受抑制，如抗凝血酶III水平下降、蛋白C和蛋白S水平下降、组织因子通路抑制物（TFPI）缺乏，进一步促进微血栓形成。

体内广泛性凝血过程，消耗了血小板和大量凝血因子，使血液由高凝状态转变为消耗性低凝状态而引起出血。

（2）纤维蛋白溶解亢进：其机制为：①凝血过程所形成的纤维蛋白沉积于微血管内和肝、脾等脏器，刺激血管内皮释放活化素，并使肝脾等脏器损伤后释出纤溶酶原激活物进入血流；②活化的因子Ⅹ、Ⅻ能使血浆活化素原转化为活化素，并能使血管舒缓素原转变为血管舒缓素，激活纤溶酶原转变为纤溶酶；③缺氧和各种引起DIC的病因通过交感神经-肾上腺作用，刺激血管内皮释放活化素；④病理性凝血酶能激活纤溶酶原转化为纤溶酶，大量纤溶酶导致纤维蛋白溶解亢进。纤维蛋白降解产物（FDP）可干扰纤维蛋白单体聚合，又可与血小板膜结合造成血小板功能缺陷，同时FDP还有抗凝血酶作用，从而进一步损害凝血功能；加之，缺氧、酸中毒、创伤等可致部分凝血因子失活，加重出血倾向。

以上两个基本病理过程虽为相继发生，但几乎同时并进，而两者的进展程度则随病程的早晚有所差异，早期以凝血过程为主，晚期则以纤溶亢进为主。

激活的因子Ⅻ可激活缓激肽原，使之转变成缓激肽，导致小血管扩张和通透性增加，加之小血管栓塞后微循环受阻，回心血量及心排出量减少而导致血压下降，进而发生休克。

由于血管内凝血所形成纤维蛋白条状物与网眼使红细胞通过时受到机械损伤；同时红细胞因缺血、缺氧、毒素以及表面有纤维蛋白附着而脆性增加，导致红细胞变形、破裂而出现溶血。

【临床表现】

由于基础疾病的不同和疾病的发展缓急不一，因而临床上将DIC分为3 型：①急性型：大多数DIC表现为本型，常见于严重感染或大手术后，起病急，病情凶险，出血严重，持续数小时至数天；②亚急性型：病程持续数天或数周，常见于急性白血病、恶性肿瘤转移等；③慢性型：起病慢、病情轻，出血不严重，病程可长达数月，见于慢性疾病如巨大血管瘤、系统性红斑狼疮等。DIC 的主要临床表现：

1．出血 最常见，常为首发症状。在病程的不同阶段，有不同的出血表现：在高凝状态时一般无出血；在消耗性低凝状态时，出血明显并逐渐加重；在发生继发性纤溶时，出血更加严重。出血轻者仅见皮肤出血点或大便隐血试验阳性，重者则为自发性多部位出血。皮肤出血表

现为出血点、瘀点或片状瘀斑，多见于躯干或四肢；鼻黏膜、牙龈、胃肠道出血亦较常见；穿刺部位或伤口渗血不止，且渗出血液往往不凝固；严重者泌尿道出血或颅内出血。出血量多者可至贫血或休克，甚至死亡。

2．休克 表现为一过性或持久性血压下降。幼婴常表现为面色青灰或苍白、黏膜青紫、肢端冰冷和发绀、精神萎靡和尿少等。休克使血流进一步缓慢，加重缺氧和酸中毒，从而加重DIC。故DIC与休克互为因果，呈恶性循环，甚至发生不可逆休克。

3．栓塞 组织和脏器的微血栓使血流阻滞，导致受累器官缺血、缺氧、代谢紊乱和功能障碍，甚至坏死。临床表现随受累器官及其受累程度的不同而异：肺受累时可出现呼吸困难、发绀、咯血、呼吸衰竭，也可因肺动脉高压而引起右心衰竭；肾脏受累时表现为尿少、血尿，甚至肾衰竭；胃肠道受累时出现恶心、呕吐、腹痛和胃肠道出血等；脑栓塞时可出现昏迷、惊厥等。其他如肝功能障碍，四肢末端坏死，皮肤坏疽等。

4．溶血 急性溶血表现为发热、黄疸、苍白、乏力、腰背酸痛、血红蛋白尿等。如溶血严重、超过骨髓代偿能力时即出现贫血，称为微血管病性溶血性贫血（microangiopathic hemolytic anemia）。

【实验室检查】

实验室检查为确诊DIC的依据。

1．反映消耗性凝血障碍的检查

（1）血小板计数减少：常降至100×10^9/L以下，如呈进行性下降则更有诊断意义。

（2）出血时间和凝血时间延长：但在高凝状态时，出血时间可缩短。

（3）凝血酶原时间（PT）延长：超过正常对照3 秒以上有意义（出生4 天内的新生儿超过20 秒才有意义）。

（4）纤维蛋白原减少 低于1.6 g/L有意义，个别高凝期病例反可升高超过4.0 g/L。

（5）活化部分凝血活酶时间（APTT）延长：年长儿正常值为42 秒，新生儿为44～73 秒，早产儿范围更宽。APTT比正常对照延长10 秒以上才有临床意义。高凝期APTT可缩短，低凝期及继发性纤溶期APTT延长。

（6）抗凝血酶III（AT-III）测定：AT-III是重要生理抗凝物质，它使凝血酶、激活的因子X失去活性而起抗凝作用，在此过程中AT-III被消耗，故DIC早期血浆中AT-III明显减少。正常值为80％～100％（活性）。

（7）因子Ⅷ测定：DIC时Ⅷ：C减少。

2．反映纤维蛋白形成和纤维蛋白溶解亢进的检查

（1）血浆鱼精蛋白副凝试验（plasma protamine paracoagulation，3P试验）：血管内凝血时，血中纤维蛋白单体与FDP结合形成一种可溶性复合物，鱼精蛋白能与FDP结合，使纤维蛋白单体从复合物中分离出来，被分离出来的纤维蛋白单体又聚合成纤维蛋白而形成絮状沉淀，即为3P试验阳性。此试验在DIC早期时多阳性，但晚期以纤溶亢进为主时，因纤维蛋白单体形成很少，所形成的可溶性复合物也少，故3P试验常为阴性。此外，约20％脐带血3P阳性，第2 天后转阴性，故新生儿3P试验应在出生2 天以后才有诊断价值。有些疾病如恶性肿瘤、肝、肾疾病及手术创伤后也可出现3P阳性。

（2）优球蛋白溶解时间：正常血浆的优球蛋白含有纤维蛋白原、血浆素原及其激活因子，而不含抗血浆素，优球蛋白溶解时间缩短反映血浆素原及激活因子的活性增强，表示纤溶亢进。正常值>120 分钟，DIC纤溶亢进时缩短，常<70 分钟。

（3）FDP含量测定 正常人血清FDP<10 mg/L；超过20 mg/L提示纤溶亢进，但不能作为诊

断DIC的指标。肺栓塞或动、静脉栓塞患者也可升高。

（4）凝血时间（TT）测定 是反映凝血第3 阶段的试验，正常值为20 秒±1.6 秒，比正常对照延长3 秒以上有诊断意义。

（5）D-二聚体（D-dimer）测定：D-二聚体是一个新的抗原，产生于纤维蛋白原转变成纤维蛋白时，纤维蛋白交联和交联纤维蛋白降解的过程中。DIC患者D-二聚体异常升高，此试验对DIC有特异性。

3．其他检查 除上述检验项目外，近年来还开展了一些对DIC有诊断价值的方法，简述于下：

（1）反映血管内皮细胞损伤的分子标志物：如组织因子（TF）和内皮素-1（ET-1）等。

（2）反映血小板激活的分子标志物：如血小板因子4（PF-4）、β-血栓球蛋白（β-TG）和α-颗粒膜糖蛋白（GMP-140）等。

（3）反映凝血和纤维蛋白溶解激活的分子标志物：如纤维蛋白肽A（FPA）和纤维蛋白B-β15-42 肽等。

此外，观察外周血涂片中红细胞及血小板形态亦有一定的诊断价值，如红细胞呈盔状、皱缩、三角形、新月形及碎片等有意义；涂片上有巨大血小板或有核细胞亦有一定意义。

【诊断】

必须依据临床表现和实验室检查结果进行综合性分析，才能明确诊断。①临床特点：患儿有诱发DIC的原发病存在，并在此基础上呈现出血倾向、微血管栓塞、休克和溶血等临床征象，或对抗凝治疗有效，即应高度警惕DIC的可能性；②实验室检查：是诊断的重要依据，应根据病情及实验室条件选择检查项目，对化验结果的分析应结合患儿年龄、原发病性质、DIC不同病程等特点作出判断，动态观察其结果变化对确立诊断的意义更大。

如在血小板计数减少、凝血酶原时间延长、纤维蛋白原含量降低、3P试验阳性这4 项中有3 项阳性，结合临床特点即可作出诊断；如仅有2 项阳性，则需加测血清FDP含量、优球蛋白溶解时间和凝血酶时间，如其中有1 项阳性，结合临床特点也可作出诊断。条件许可时，测定AT-Ⅲ、因子Ⅷ活性和D-二聚体等指标均较为可靠。

【治疗】

早期诊断、及时治疗是提高DIC治愈率的关键。

1．治疗原发病 积极治疗原发病、去除诱发因素是终止DIC病理过程的重要措施，如果原发病及诱因没有消除，凝血异常继续进行。

2．改善微循环 低分子右旋糖酐不但能扩充血容量、疏通微循环，还有降低血液黏稠度、减低血小板黏附和抑制红细胞凝集等作用，因而可以改善微循环，防止或减少血栓形成。首次剂量为10 ml/kg静滴，以后每次5 ml/kg，每6 小时1 次，全日量不超过30 ml/kg。

3．纠正酸中毒 DIC多伴有酸中毒，往往也是肝素治疗失败的原因之一。因此，应及时发现酸中毒并予纠正，常用5％碳酸氢钠。

4．应用血管活性药物 血管扩张剂可解除血管痉挛，改善微循环，常用654-2、异丙基肾上腺素和多巴胺等。

5．抗凝治疗 其目的在于阻断或减缓血管内凝血过程的发展。

（1）抗血小板凝集药物：此类药物能阻抑血小板黏附和凝集，减轻微血栓形成，从而抑制DIC的发展。临床上对轻型DIC、疑似DIC而未肯定诊断者、或高凝状态者，在控制原发病的基础上可单独应用此类药物治疗。常用药物有：①阿司匹林，剂量为每日10 mg/kg，分2～3 次口服，持续用至血小板数恢复正常后数日才停药；②双嘧达莫（潘生丁）：剂量为每日10 mg/kg，

分次口服。

（2）肝素的应用：肝素可与AT-Ⅲ结合成复合物而起抗凝作用，对凝血3 个阶段均有抑制作用，并可抑制血小板聚集、裂解和促使纤维蛋白溶解。通常在给药1～3 小时后约50%因灭活而失效，4～6 小时即经肾脏排完。

肝素多在DIC早期应用，凡有以下指征者即可使用：①处于高凝状态者；②有明显栓塞症状者；③消耗性凝血期表现为凝血因子、血小板、纤维蛋白原进行性下降，出血逐渐加重，血压下降或休克者；④准备补充凝血因子（如输血、血浆等）或应用纤溶抑制药物而未能确定促凝物质是否仍在发生作用时，可先应用肝素。

以下情况禁用或慎用肝素：①颅内或脊髓内出血、肺结核空洞出血、溃疡出血；②伴有血管损伤或新鲜创面的患儿；③DIC晚期以继发性纤溶为主者；④原有重度出血症如血友病等；⑤对并有严重肝脏病患者，尚有争议，较多作者认为弊多利少。

常用方法为：每次60～125 U/kg（1 mg=125 U）加入等渗氯化钠或10%葡萄糖液50～100 ml中静滴，约1 小时滴完，每4～6 小时1 次；或先以50～75 U/kg静滴，然后按每小时15～25 U/kg速度持续静滴；或每次50～100 U/kg皮下注射，每4～6 小时1 次。

在应用肝素期间必须密切观察病情并监测凝血功能，在每次用药前测凝血时间（试管法），用药4 小时后再测定1 次凝血时间，要求凝血时间控制在20～30 分钟内，如<20 分钟可加大肝素剂量，如>30 分钟且出血加重可能是用量过大，应停用，必要时静脉缓慢注射鱼精蛋白中和之，其用量与最后1 次肝素用量相等（1 mg鱼精蛋白可中和125 U肝素），若出血仍不减轻，15 分钟后可再注射1 次鱼精蛋白。

停药指征为：①诱发DIC的原发病已控制或缓解；②用药后病情好转，出血停止，血压稳定；③凝血酶原时间和纤维蛋白原恢复正常或接近正常（前者一般于24 小时内恢复，后者于1～3 天恢复）时，即可逐渐减量至停药。用药时间一般可持续3～7 天。血小板的回升缓慢（数天至数周），不宜作为停药的指征。

6．抗凝血因子的应用 已应用临床的有：①抗凝血酶Ⅲ（AT-Ⅲ）浓缩剂：用于DIC早期补充AT-Ⅲ并可提升肝素的疗效；②蛋白-C浓缩剂：主要用于革兰氏阴性杆菌感染合并DIC，同肝素联合应用取得了较好的效果。

7．补充疗法 目前认为在活动性DIC未控制之前，补充下列成分是安全的：经洗涤的浓缩红细胞、浓缩血小板和不含凝血因子的扩容剂（如血浆蛋白、白蛋白和羧基淀粉等）。如果DIC过程停止（指征是AT-Ⅲ测定值正常）或肝素化后仍持续出血，此时有必要补充凝血因子，可输注新鲜冰冻血浆、凝血酶原复合物等。

8．抗纤溶药物 此类药物的主要作用是阻碍纤溶酶原转变为纤溶酶、抑制纤维蛋白的分解，从而防止纤维蛋白溶解亢进性出血。DIC时继发性纤溶亢进是机体防止血管内凝血的一种生理性保护机能，有助于防止或消除血管内纤维蛋白栓塞，因此在DIC时，特别是在早期高凝状态，应禁用抗纤溶药物；若病情发展并出现以纤溶为主时，最好在肝素化的基础上慎用纤溶抑制剂，可能有助于DIC后期的治疗。一般可选用6-氨基己酸（EACA），每次剂量为0.08～0.12 g/kg，缓慢静注或稀释后静滴，亦可采用对羧基苄胺（PAMBA）或氨甲环酸。

9．糖皮质激素的应用 在DIC时是否应该使用糖皮质激素尚未取得一致意见。一般认为如果因治疗原发病需要时，可在肝素化的基础上慎用。

（盛光耀）

第六节 急性白血病

白血病（1eukemia）是造血组织中某一血细胞系统过度增生，浸润到各组织和器官，从而引起一系列临床表现的恶性血液病。是我国最常见的小儿恶性肿瘤。据调查，我国10 岁以下小儿白血病的发生率为3～4/10 万，男性发病率高于女性。急性白血病占90％～95%，慢性白血病仅占3％～5％。

【病因】

尚未完全明了，可能与下列因素有关。

1．病毒感染 多年研究已证明属于RNA病毒的逆转录病毒（retrovirus，又称人类T细胞白血病病毒，HTLV）可引起人类T淋巴细胞白血病。

2．物理和化学因素 电离辐射能引起白血病。小儿对电离辐射较为敏感，在曾经放射治疗胸腺肥大的小儿中，白血病发生率较正常小儿高10 倍；妊娠妇女照射腹部后，其新生儿白血病的发病率比未经照射者高17.4 倍。苯及其衍生物、氯霉素、保泰松、乙双吗啉和细胞毒药物等均可诱发急性白血病。

3．遗传素质 白血病不属遗传性疾病，但在家族中却可有多发性恶性肿瘤的情况。少数患儿可能患有其他遗传性疾病，如21-三体综合征、先天性睾丸发育不全症、先天性再生障碍性贫血伴有多发畸形（Fanconi贫血）、先天性远端毛细血管扩张性红斑症（Bloom综合征）以及严重联合免疫缺陷病等。这些疾病患儿的白血病发病率比一般小儿明显增高。此外，单卵孪生儿中一个患急性白血病，另一个患白血病的几率为20％，比双卵孪生儿的发病率高12 倍。以上现象均提示白血病的发生与遗传素质有关。

【发病机制】

尚未完全明了，下列机制可能在白血病的发病中起重要作用。

1．原癌基因的转化 人类和许多哺乳动物的染色体基因组中存在原癌基因（又称细胞癌基因），在正常情况时，其主要功能是参与调控细胞的增殖、分化和衰老死亡。当机体受到致癌因素的作用下，原癌基因可发生点突变、染色体重排或基因扩增，转化为肿瘤基因，从而导致白血病的发生。

2．抑癌基因畸变 近年研究发现正常人体存在着抑癌基因，如RB、P53、P16、WT1等，当这些抑癌基因发生突变、缺失等变异时，失去其抑癌活性，造成癌细胞异常增殖而发病。

3．细胞凋亡受抑 细胞凋亡是在基因调控下的一种细胞主动性自我消亡过程，是人体组织器官发育中细胞清除的正常途径。当细胞凋亡通路受到抑制或阻断时，细胞没有正常凋亡而继续增殖导致恶变。研究发现，急性白血病时抑制凋亡的基因（如Bcl-2 、Bcl-XL等）常高表达，而促进凋亡的基因（如P53、Fas、Bax等）表达降低或出现突变；此外，特异染色体易位产生的融合基因也可抑制细胞凋亡（如M3中的PML/RARα融合基因）。由此可见，细胞凋亡受抑在白血病发病中的起重要作用。

【分类和分型】

急性白血病的分类或分型对于诊断、治疗和提示预后都有一定意义。根据增生的白细胞种类的不同，可分为急性淋巴细胞白血病（急淋，ALL）和急性非淋巴细胞白血病（急非淋，ANLL）两大类，前者约占小儿白血病的70%～85%。目前，常采用形态学（M）、免疫学（I）和细胞遗传学（C），即MIC综合分型，以指导治疗和提示预后。

1．急性淋巴细胞白血病（ALL）

（1）形态学分型（FAB分型）：根据原淋巴细胞形态学的不同，分为3 种类型：①L1型：以小细胞为主，其平均直径为6.6 μm，核染色质均匀，核形规则；核仁很小，一个或无；细胞质少，细胞质空泡不明显。②L2型：以大细胞为主，大小不一，其平均直径为8.7 μm，核染色

质不均匀，核形不规则；核仁一个或多个，较大；细胞质量中等，细胞质空泡不定。③L3型：以大细胞为主，细胞大小一致，核染色质细点状，均匀，核形规则；核仁一个或多个；细胞质量中等，细胞质空泡明显。上述3型中以L1型多见，占80％以上；L3型最少，占4%以下。

（2）免疫学分型：应用单克隆抗体检测淋巴细胞表面抗原标记，一般可将急性淋巴细胞白血病分T、B二大系列。

1）T系急性淋巴细胞白血病（T-ALL）：约占小儿ALL的10%～15%。具有阳性的T淋巴细胞标志，如CD1、CD3、CD5、CD8和TdT（末端脱氧核糖核酸转换酶）阳性。

2）B系急性淋巴细胞白血病(B-ALL)：约占小儿ALL的80%～90%。此型又分为3种亚型：①早期前B细胞型(early Pre B-ALL)：HLA-DR、CD79a、CD19和（或）CyCD22（细胞质CD22）阳性；SmIg、CyIg阴性。②前B细胞型(Pre B-ALL)：CyIg阳性，SmIg阴性；其他B系标志及HLA-DR阳性。③成熟B细胞型(B-ALL)：SmIg阳性，CyIg阴性；其他B系标记及HLA-DR阳性。

3）伴有髓系标志的ALL（My^+-ALL）：本型具有淋巴系的形态学特征，以淋巴系特异抗原为主但伴有个别、次要的髓系特异抗原标志，如CD13、CD33、CD14等阳性。

（3）细胞遗传学改变：主要有：①染色体数目异常，如≤45条的低二倍体，或≥47条的高二倍体；②染色体核型异常：如12号和21号染色体易位，即t(12；21)/AMLITEL（ETV6-CBFA2）融合基因；9号和22号染色体易位，即t（9；22）/BCR-ABL融合基因；或t（4；11）/MLL-AF4融合基因等。

（4）临床危险度分型：中华医学会儿科学分会血液组（2006年5月修订的全国方案）根据国内外分型情况，已建议分为三型。

1）高危型急性淋巴细胞白血病（HR-ALL）：凡具备下述1 项或多项危险因素者。①年龄<12 个月的婴儿白血病；②诊断时外周血白细胞计数≥$100×10^9$/L；③t（9；22）/BCR-ABL融合基因； t（4；11）/MLL-AF4融合基因；④泼尼松试验不良效应者（泼尼松每日60 mg/m^2诱导7 天，第8 天外周血白血病细胞>$1×10^9$/L）；⑤初治诱导缓解治疗失败（标准化疗方案6 周未获完全缓解）。

2）中危型急性淋巴细胞白血病（MR-ALL）：具备以下任何1 项或多项危险因素者。①年龄≥10 岁；②诊断时外周血白细胞计数≥$50×10^9$/L；③诊断时已发生中枢神经系统白血病和（或）睾丸白血病；④免疫表型为T细胞白血病；⑤染色体数目为<45 的低二倍体，或t（12；21）、t（9；22）核型以外的其他异常染色体核型，或t（4；11）以外的其他MLL基因重排。

3）低危型急性淋巴细胞白血病（SR-ALL）：不具备上述任何一项危险因素。

2．急性非淋巴细胞白血病

（1）FAB分型

1）原粒细胞白血病未分化型（M_1）：骨髓中原粒细胞≥90％，早幼粒细胞很少，中幼粒以下各阶段细胞极少见，可见Auer小体。

2）原粒细胞白血病部分分化型（M_2）：骨髓中原粒和早幼粒细胞共占50％以上，可见多少不一的中幼粒、晚幼粒和成熟粒细胞，可见Auer小体；M_2b型即以往命名的亚急性粒细胞白血病，骨髓中有较多的核、浆发育不平衡的中幼粒细胞。

3）颗粒增多的早幼粒细胞白血病（M_3）：骨髓中颗粒增多的异常早幼粒细胞占30％以上，细胞质多少不一，细胞质中的颗粒形态分为粗大密集和细小密集两类，据此又可分为两型，即粗颗粒型（M_{3a}）和细颗粒型（M_{3b}）。

4）粒-单核细胞白血病（M_4）：骨髓中幼稚的粒细胞和单核细胞同时增生，原始及幼稚粒细胞>20％；原始、幼稚单核和单核细胞≥20％；或原始、幼稚和成熟单核细胞>30％，原粒和

早幼粒细胞>10％。除以上特点外，骨髓中异常嗜酸粒细胞增多。

5）单核细胞白血病（M_5）：骨髓中以原始、幼稚单核细胞为主。可分为两型：①未分化型，原始单核细胞为主，>80％；②部分分化型，骨髓中原始及幼稚单核细胞>30％，原始单核细胞<80％。

6）红白血病（M_6）：骨髓中有核红细胞>50％，以原始及早幼红细胞为主，且常有巨幼样变；原粒及早幼粒细胞>30％。外周血可见幼红及幼粒细胞；粒细胞中可见Auer小体。

7）急性巨核细胞白血病（M_7）：骨髓中原始巨核细胞>30％；外周血有原始巨核细胞。

（2）免疫学分型 急性非淋巴细胞M1～M_5型可有CD33、CD13、CD14、CD15、MPO（抗髓过氧化物酶）等髓系标志中的1 项或多项阳性，也可有CD34阳性。其中CD14多见于单核细胞系，M_6可见血型糖蛋白A阳性，M_7可见血小板膜抗原Ⅱb/Ⅲa（GPⅡb/Ⅲa）阳性、或CD41、CD68阳性。

（3）细胞遗传学改变：①染色体数目异常以亚二倍体为主，超二倍体较少；②常见的核型改变有t（9；11）/MLL-AF9融合基因（常见于M_5），t（11；19）/ENL-MLL融合基因，t（8；21）/AML-ETO融合基因(M_{2b}的特异标记)、t（15；17）/PML-RAR_8融合基因（M_3特异标记）、inv16（多见于M_4Eo）等。

（4）临床危险度分型：中华医学会儿科学分会血液组（2006年11月）建议分为3 型：

1）低危ANLL（LR-ANLL）：指M_3、M_{2b}、M_4Eo及其他伴inv16者。

2）高危ANLL（HR-ANLL）：包括以下因素：①诊断时年龄≤1岁；②诊断时WBC≥100×10^9/L；③染色体核型-7；④MDS-ANLL；⑤标准方案一个疗程不缓解。

3）中危ANLL（MR-ANLL）：非低危，又不具备高危因素者。

【临床表现】

各型急性白血病的临床表现基本相同，主要表现如下。

1．起病 大多较急，少数缓慢。早期症状有：面色苍白、精神不振、乏力、食欲低下，鼻出血或齿龈出血等；少数患儿以发热和类似风湿热的骨关节痛为首发症状。

2．发热 多数患儿起病时有发热，热型不定，可低热、不规则发热、持续高热或弛张热，一般不伴寒战。发热原因之一是白血病性发热，多为低热且抗生素治疗无效；另一原因是感染，多为高热。

3．贫血 出现较早，并随病情发展而加重，表现为苍白、虚弱无力、活动后气促等。贫血主要是由于骨髓造血干细胞受到抑制所致。

4．出血 以皮肤和黏膜出血多见，表现为紫癜、瘀斑、鼻出血、齿龈出血，消化道出血和血尿。偶有颅内出血，为引起死亡的重要原因之一。出血的主要原因是：①骨髓被白血病细胞浸润，巨核细胞受抑制使血小板的生成减少和功能不足；②白血病细胞浸润肝脏，使肝功能受损，纤维蛋白原、凝血酶原和第Ⅴ因子等生成不足；③感染和白血病细胞浸润使毛细血管受损，血管通透性增加；④并发弥散性血管内凝血。在各类型白血病中，以M_3型白血病的出血最为显著。

5．白血病细胞浸润引起的症状和体征

（1）肝、脾、淋巴结肿大：白血病细胞浸润而发生于肝、脾而造成其肿大大，这在急性淋巴细胞白血病尤其显著。肿大的肝、脾质软，表面光滑，可有压痛。全身浅表淋巴结轻度肿大，但多局限于颈部、颌下、腋下和腹股沟等处，其肿大程度以急性淋巴细胞白血病较为显著。有时因纵隔淋巴结肿大引起压迫症状而发生呛咳、呼吸困难和静脉回流受阻。

（2）骨和关节浸润：小儿骨髓多为红髓，易被白血病细胞侵犯，故患儿骨、关节疼痛较

为常见。约25％患儿以四肢长骨、肩、膝、腕、踝等关节疼痛为首发症状，其中部分患儿呈游走性关节痛，局部红肿现象多不明显，并常伴有胸骨压痛。骨和关节痛多见于急性淋巴细胞白血病。骨骼X线检查可见骨质疏松、溶解，骨骺端出现密度减低横带和骨膜下新骨形成等征象。

（3）中枢神经系统浸润：白血病细胞侵犯脑实质和（或）脑膜时即引起中枢神经系统白血病（central nervous system leukemia，CNSL）。由于近年联合化疗的进展，使患儿的寿命得以延长，但因多数化疗药物不能透过血-脑屏障，故中枢神经系统便成为白血病细胞的"庇护所"，造成CNSL的发生率增高，这在急性淋巴细胞白血病尤其多见。浸润可发生于病程中任何时候，但多见于化疗后缓解期。它是导致急性白血病复发的主要原因。

常见症状为：颅内压增高，出现头痛、呕吐、嗜睡、视乳头水肿等；浸润脑膜时，可出现脑膜刺激征；浸润脑神经核或神经根时，可引起脑神经麻痹；脊髓浸润可引起横贯性损害而致截瘫。此外，也可有惊厥，昏迷。检查脑脊液可以确诊：脑脊液色清或微浊，压力增高；细胞数$>10\times10^6$/L，蛋白>0.45 g/L；将脑脊液离心沉淀作涂片检查可发现白血病细胞。

（4）睾丸浸润：白血病细胞侵犯睾丸时即引起睾丸白血病（testic leukemia，TL），表现为局部肿大、触痛，阴囊皮肤可呈红黑色。由于化疗药物不易进入睾丸，在病情完全缓解时，该处白血病细胞仍存在，因而常成为导致白血病复发的另一重要原因。

（5）绿色瘤：是急性粒细胞白血病的一种特殊类型，白血病细胞浸润眶骨、颅骨、胸骨、肋骨或肝、肾、肌肉等，在局部呈块状隆起而形成绿色瘤。此瘤切面呈绿色，暴露于空气中绿色迅速消退，这种绿色素的性质尚未明确，可能是光紫质或胆绿蛋白的衍生物。绿色瘤偶由急性单核细胞白血病局部浸润形成。

（6）其他器官浸润：少数患儿有皮肤浸润，表现为丘疹、斑疹、结节或肿块；心脏浸润可引起心脏扩大、传导阻滞、心包积液和心力衰竭等；消化系统浸润可引起食欲不振、腹痛、腹泻、出血等；肾脏浸润可引起肾肿大、蛋白尿、血尿、管型尿等；齿龈和口腔黏膜浸润可引起局部肿胀和口腔溃疡，这在急性单核细胞白血病较为常见。

【实验室检查】

1．外周血象 红细胞及血红蛋白均减少，大多为正细胞正血色素性贫血。网织红细胞数大多较低，少数正常，偶在外周血中见到有核红细胞。白细胞数增高者约占50％以上，其余正常或减少，但在整个病程中白细胞数可有增、减变化。白细胞分类示原始细胞和幼稚细胞占多数。血小板减少。

2．骨髓象 骨髓检查是确立诊断和评定疗效的重要依据。典型的骨髓象为该类型白血病的原始及幼稚细胞极度增生；幼红细胞和巨核细胞减少。但有少数患儿的骨髓表现为增生低下，其预后和治疗均有特殊之处。

3．组织化学染色 常用以下组织化学染色以协助鉴别细胞类型。

（1）过氧化酶：在早幼阶段以后的粒细胞为阳性；幼稚及成熟单核细胞为弱阳性；淋巴细胞和浆细胞均为阴性。各类型分化较低的原始细胞均为阴性。

（2）酸性磷酸酶：原始粒细胞大多为阴性，早幼粒以后各阶段粒细胞为阳性；原始淋巴细胞弱阳性，T细胞强阳性，B细胞阴性；原始和幼稚单核细胞强阳性。

（3）碱性磷酸酶：成熟粒细胞中此酶的活性在急性粒细胞白血病时明显降低，积分极低或为0；在急性淋巴细胞白血病时积分增加，在急性单核细胞白血病时积分大多正常。

（4）苏丹黑：此染色结果与过氧化物酶染色的结果相似：原始及早幼粒细胞阳性；原淋巴细胞阴性；原单核细胞弱阳性。

（5）糖原：原始粒细胞为阴性，早幼粒细胞以后各阶段粒细胞为阳性；原始及幼稚淋巴

细胞约半数为强阳性，余为阳性；原始及幼稚单核细胞多为阳性。

（6）非特异性酯酶（萘酚酯NASDA）：这是单核细胞的标记酶，幼稚单核细胞强阳性，原始粒细胞和早幼粒细胞以下各阶段细胞为阳性或弱阳性；原始淋巴细胞阴性或弱阳性。

4．溶菌酶检查 血清中的溶菌酶主要来源于破碎的单核细胞和中性粒细胞，测定血清与尿液中溶菌酶的含量可以协助鉴别白血病细胞类型。正常人血清含量为4～20 mg/L；尿液中不含此酶。在急性单核细胞白血病时，其血清及尿液的溶菌酶浓度明显增高；急性粒细胞白血病时中度增高；急性淋巴细胞白血病时则减少或正常。

【诊断和鉴别诊断】

典型病例根据临床表现、血象和骨髓象的改变即可作出诊断。发病早期症状不典型，特别是白细胞数正常或减少者，其血涂片不易找到幼稚白细胞时，可使诊断发生困难。须与以下疾病鉴别。

1．再生障碍性贫血 本病血象呈全血细胞减少；肝、脾、淋巴结不肿大；骨髓有核细胞增生低下，无幼稚白细胞增生。

2．传染性单核细胞增多症 本病肝、脾、淋巴结常肿大；白细胞数增高并出现异型淋巴细胞，易与急性淋巴细胞白血病混淆。但本病病程经过一般良好，血象多于1 个月左右恢复正常；血清嗜异性凝集反应阳性；骨髓无白血病改变。

3．类白血病反应 为造血系统对感染、中毒和溶血等刺激因素的一种异常反应，以外周血出现幼稚白细胞或白细胞数增高为特征。当原发疾病被控制后，血象即恢复正常。此外，根据血小板数多正常；白细胞中有中毒性改变，如中毒颗粒和空泡形成；中性粒细胞碱性磷酸酶积分显著增高等，可与白血病区别。

4．风湿性关节炎 有发热、关节疼痛症状者易与风湿性关节炎混淆，须注意鉴别。

【治疗】

急性白血病的治疗主要是以化疗为主的综合疗法，其原则是：早期诊断、早期治疗；应严格区分白血病类型，按照类型选用不同的化疗方案和相应的药物剂量；采用早期连续适度化疗和分阶段长期规范治疗的方针。同时要早期防治中枢神经系统白血病和睾丸白血病，注意支持疗法。持续完全缓解2.5～3 年者方可停止治疗。

1．支持疗法

（1）防治感染：在化疗阶段，保护性环境隔离对降低院内交叉感染具有较好效果。并发细菌性感染时，应首选强力的抗生素以控制病情，并根据药敏试验结果调整抗生素；并发真菌感染者，可选用抗真菌药物如二性霉素B或氟康唑等治疗；并发病毒感染者可选用抗病毒药物如阿昔洛韦、更昔洛韦等治疗；怀疑并发卡氏囊虫肺炎者，应使用复方新诺明。

（2）成分输血：明显贫血者可输红细胞；因血小板减少而致出血者，可输浓缩血小板。有条件时可酌情静脉输注丙种球蛋白。

（3）集落刺激因子：化疗期间如骨髓抑制明显者，可予以G-CSF、GM-CSF等集落刺激因子。

（4）高尿酸血症的防治：在化疗早期，由于大量白血病细胞破坏分解而引起高尿酸血症，导致尿酸结石梗阻、少尿或急性肾功能衰竭，故应注意水份补充。为预防高尿酸血症，可口服别嘌呤醇（allopurinol）。

（5）其他：在治疗过程中，要增加营养。有发热、出血时应卧床休息。要注意口腔卫生，防止感染和黏膜糜烂。并发弥散性血管内凝血时，可用肝素治疗。

2．化学药物治疗 简称化疗，其目的是杀灭白血病细胞，解除白血病细胞浸润引起的症状，

使病情缓解、并巩固治疗效果减少耐药，以至治愈。

ALL的化疗：高危（HR），中危（MR）和低危（SR）的小儿ALL均需经历下列阶段的治疗：

（1）诱导治疗：诱导缓解治疗是患儿能否长期无病生存的关键，需联合数种化疗药物，最大程度地杀灭白血病细胞，从而尽快达到完全缓解。HR-ALL推荐使用长春新碱（VCR）1.5 mg/m^2，静脉注射，每周1 次，共4 次，于化疗的第8 天（d8 下同）、d15、d22、d29使用；柔红霉素（DNR）30mg/m^2，静脉滴注，于d8～d10或d8起每周1 次共3 次；门冬酰胺酶（L-ASP）6000～10000 u/m^2，静脉滴注或肌注，于d11起隔天或隔两天1 次，共10 次；泼尼松（Pred）d1～d28为60 mg/（m^2•d），分次口服，d29起减量，至d36停用。MR-ALL者参照HR-ALL方案，仅减少L-ASP2 次。SR-ALL者则DNR减少1 次，L-ASP减少4 次。

2．巩固治疗：小儿ALL达到完全缓解（CR）时，体内仍残存约达10^8～10^9个白血病细胞，这种状态称为微小残留病（minimal residual disease，MRD）。因此，有必要采用较强的巩固治疗。全国方案推荐环磷酰胺（CTX）1000 mg/m^2，快速静滴，d1；阿糖胞苷（Ara-C）1 g/m^2，每12 小时1 次，共6 次，d2～d4静滴；6-巯基嘌呤（6-MP）50 mg/（m^2•d），晚间1 次口服，d1～d7。

3．预防髓外白血病：由于大多数药物不能进入中枢神经系统、睾丸等部位，如果不积极预防髓外白血病，CNSL在3 年化疗期间的发生率可高达50％左右；TL的发生率在男孩中亦可有5％～30％。CNSL和TL均会导致骨髓复发、治疗失败，因此有效的髓外白血病的预防是白血病特别是急性淋巴细胞白血病患儿获得长期生存的关键之一。预防性治疗的常用方法：

1）三联鞘内注射法（IT）：常用MTX、Ara-c 、地塞米松（Dex）3 种药物联合鞘内注射，剂量见表15-5。

表15-5 不同年龄三联鞘注药物剂量（mg/次）

年龄（月）	MTX	Ara-c	Dex
<12	5	12	2
12～24	7.5	15	2
25～35	10	25	5
≥36	12.5	35	5

2）大剂量甲氨蝶呤-四氢叶酸钙（HDMTX-CF）疗法：每10 天为1 疗程。每疗程MTX剂量为2～5 g/m^2，共用3～4疗程。其中1/6量（<500 mg）作为突击量，在30 分钟内快速静脉滴入，余量于12～24 小时内匀速滴入；突击量MTX滴入后0.5～2 小时内行三联鞘内注射1 次；开始滴注MTX 36 小时后用四氢叶酸钙（CF）解救，剂量为每次15 mg/m^2，首剂静脉注射，以后每6 小时口服或肌内注射，共6～8 次。HDMTX治疗前、后3 天口服碳酸氢钠1.0 g，每日3 次，并在治疗当天给5%碳酸氢钠3～5 ml/kg静脉滴注，使尿pH>7.0；用HDMTX当天及后3 天需水化治疗，每日液体总量3000 ml/m^2。在用HDMXT同时，每天口服6-MP 50mg/m^2，共7 天。有条件者监测血浆MTX浓度，以调整CF用量和次数；无监测者MTX不宜>3 g/m^2，但HR型或MR的T细胞型者远期复发的可能性增加。

3）颅脑放射治疗：多用于>4 岁的HR-ALL患儿，诊断时白细胞数>100×10^9/L、T-ALL、或有CNSL、或因各种原因不宜HDMTX-CF治疗者，均应进行颅脑放射治疗。在CR后6 个月时进行，放射总剂量为12 Gy，分15 次于3 周内完成。同时每周鞘内注射1 次。放疗第3 周用VDex

方案：VCR 1.5 mg/m^{2}静注1 次；Dex每日8 mg/m^2，口服7 天。

（4）早期强化治疗或再诱导治疗：目的仍然是治疗MRD，常用的VDLDex方案：VDR、DNR均于d1 、d8 各1 次，剂量和用法同诱导治疗。L-ASP 6000～10000 u/m2，d1 起隔天或隔两天1 次，共6～8 次。Dex 6 mg/（m^2·d），d1 ～d14 。休息1～2 周按CAM（除Ara-c为75 mg/（m^2·d），d1 ～d4 ，d8 ～d11 外），其余用法见巩固治疗。

（5）维持治疗和加强治疗：为了巩固疗效、达到长期缓解或治愈的目的，必须在上述疗程后进行维持治疗和加强治疗：对ALL 一般主张用 6-巯基嘌呤（6-MP）或 6-硫鸟嘌呤（6-TG）+ MTX 维持治疗，维持期间必须定期用原诱导缓解方案或其他方案强化，总疗程 2.5 ～3 年。

（6）中枢神经系统白血病（CNSL）的治疗：初诊时已发生CNSL者，照常进行诱导治疗，同时给予三联鞘内注射（表15-5），第 1 周 3 次，第 2 和第 3 周各 2 次，第 4 周 1次，共 8 次。一般在鞘内注射化疗 2～3 次后 CSF 常转为阴性。在完成诱导缓解、巩固、髓外白血病防治和早期强化后，作颅脑放射治疗，剂量同上。颅脑放疗后不再用 HDMTX-CF 治疗，但三联鞘内注射必须每 8 周 1 次，直到治疗终止。完全缓解后在维持巩固期发生 CNSL 者，也可按上述方法进行，但在完成第 5 次三联鞘注后，必须作全身强化治疗以免骨髓复发，常用早期强化治疗的 VDLDex 和 VPl6+Ara-C 方案各 1 疗程。此后每 8 周三联鞘内注射 1 次，直到终止治疗。

（7）睾丸白血病（TL）治疗：初诊时已发生 TL 者，先诱导治疗到完全缓解，双侧 TL 者作双侧睾丸放射治疗，总剂量为 24～30 Gy，分 6～8 天完成；单侧者可行切除术，亦可作睾丸放射治疗；与此同时继续进行巩固、髓外白血病防治和早期强化治疗。在缓解维持治疗期发生 TL 者，按上法予以治疗，紧接着用 VDLDex 和 VPl6+Ara-C 方案各 1 疗程。

3．急性非淋巴细胞白血病的治疗

（1）诱导治疗：与ALL相比，ANLL的诱导化疗难度更大，并发症较多，每个患者都必须经过骨髓抑制期才有可能完全缓解。

1）除M_3外，各型ANLL的诱导治疗常用的基本方案如下：①DA方案：DNR每日30～40 mg/m^2，静脉滴注，每日1 次，d1～d3；Ara-C每日150～200 mg/m^{2}静脉滴注或肌肉注射，分2 次（q12h），d1～d7。②DEA方案：DNR和Ara-C同上；VPl6（或VM26）每日100～150 mg/m^2，静脉滴注，每日1 次，d5～d7。

2）M_3者：任选以下方案：①全反式维A酸（ATRA）25～30 mg/（m^2·d），d1～d60，口服；DNR 40 mg/（m^2·d），d8～d10，静滴30 分钟；Ara-c 100 mg/（m^2·d），d8～d14，分2 次，q12h皮下注射。②ATRA 25～30 mg/（m^2·d），d14～d30，口服；三氧化砷（As_2O_3）0.3～0.5 mg/（kg·d），d1～d20，静滴。

（2）缓解后治疗：

1）巩固治疗：采用原有效的诱导方案1～2 个疗程。

2）骨髓抑制性维持治疗：只限于不能进行根治性治疗者。常选用DA、DEA、COAP、CAM中 3 个有效方案作序贯治疗，第 1 年每月 1 疗程，第 2 年每 6～8 周 1 疗程，第 3 年每 8～12周 1 疗程，维持 3 年左右终止治疗。

3）根治性强化治疗，含中、大剂量 Ara-C 的化疗方案，或造血干细胞移植。

4）中枢白血病治疗：用三联鞘内注射，诱导期每周 2 次，完全缓解后每 3～6 个月 1 次。

4．造血干细胞移植（HSCT） 联合化疗是目前根治大多数ALL和部分ANLL的首选方法。鉴于HSCT是一种高风险（移植相关合并症及死亡）、高投入（经济承受力）的医疗手段，即使移植成功，仍存在着复发的可能性。因此，要严格掌握移植时机：①高危型（HR）ALL第1 次

完全缓解（CR_1），中危型（MR）ALL或标危型（SR）ALL化疗期间CR_2；②HR或MR-ALL CR_1，复发ANLL CR_2；③M_3治疗1 年后融合基因仍持续阳性者。

【预后】

近十年来由于化疗的不断改进，急性淋巴细胞白血病已不再被认为是致死性疾病，5 年无病生存率达70％～80％；急性非淋巴细胞白血病的初治完全缓解率亦已达80％，5 年无病生存率约40％～60％。

（方建培）

第七节 郎格汉斯细胞组织细胞增生症

郎格汉斯细胞组织细胞增生症（Langerhans cell histiocytosis，LCH）以前称组织细胞增生症X（histiocytosis X），是一组病因不明、临床表现多样、多发于小儿的疾病，男多于女。根据临床主要表现将本症分为三型：勒-雪病（Letterer-Siwe disease，LS）、韩-薛-柯病（Hand-Schuller-Christian disease，HSC）和骨嗜酸细胞肉芽肿（eosinophilic granuloma of bone，EGB），但各型之间临床表现又可相互重叠而出现中间型。其共同的组织学特点是郎格汉斯细胞增生、浸润，并伴有嗜酸细胞、单核-巨噬细胞和淋巴细胞等不同程度的增生。目前多认为它们是一组与免疫功能异常有关的反应性增殖性疾病。国际组织细胞协会协作组（WGHS）将郎格汉斯细胞组织细胞增生症归为组织细胞增生症 I 类，以便与非郎格汉斯细胞组织细胞增生症（II类，如嗜血细胞综合征）及恶性组织细胞病和急性单核细胞白血病（III类）相区别。

【病理】

病变可只限于单个器官或为孤立病灶，也可同时侵犯多个器官，其中以肺、肝、淋巴结、骨骼、皮肤、垂体等处病变最为显著。原有组织结构因出血、坏死而遭到破坏，同一病变器官同时出现增生、纤维化或坏死等不同阶段的病灶。尸检材料观察同一患者的不同器官，或同一器官的不同部位，其组织学改变不同。显微镜下除组织细胞外，还可见到嗜酸性粒细胞、巨噬细胞、淋巴细胞、多核巨细胞和充脂性组织细胞（即泡沫细胞）等，但不见分化极差的恶性组织细胞。病变久者可见大量充脂性组织细胞和嗜酸性粒细胞，形成肉芽肿。各种病理改变中，郎格汉斯细胞（LC）增生最具特征性。LC 表达CD1，直径约12 μm，胞核不规则，有核裂或分叶，核仁明显，细胞质不规则，电镜下细胞质内含分散的呈网球拍状或棒状的细胞器，称为Bribeck颗粒。

【临床表现】

由于受累器官、部位以及年龄不同而有较大差异。一般年龄愈小，病情愈重，随年龄增长而病变愈局限，症状也愈轻。传统上分型方法已不能满足临床需要，现根据有关资料分如下五型：

1．勒-雪病

（1）发病年龄：多在1 岁以内发病，起病急，病情重，病变广泛，可侵犯全身多个系统器官。

（2）发热：热型不规则，高热与中毒症状不一致。

（3）皮疹：出现较早，多分布于躯干、头皮发际部，四肢较少；为红色或棕黄色斑丘疹，继而呈出血性，亦可呈湿疹样、脂溢性皮疹，以后结痂，脱痂后留有白斑或色素沉着。各期皮疹可同时存在，常成批发生。

（4）肝脾和淋巴结肿大：肝、脾中、重度肿大，脾大较为明显，肝功能异常和黄疸，多有淋巴结肿大。

（5）呼吸道症状：常有咳嗽、气促、青紫，但肺部体征不明显。可合并肺大泡或白发性气胸等。可有喘憋症状，甚至导致呼吸衰竭而死亡。

（6）其他：有贫血、中耳炎、腹泻和营养不良等。

2．韩-薛-柯病

（1）发病年龄：多见于2～4岁，5岁后减少。起病缓慢，骨和软组织器官均可损害。

（2）骨质缺损：最早、最常见为颅骨缺损，病变开始为头皮组织表面隆起，硬而有轻压痛；病变蚀穿颅骨外板后肿物变软，触之有波动感，缺损边缘锐利、分界清楚；此后肿物渐被吸收，局部凹陷。除颅骨外，可见下颌骨破坏，牙齿松动、脱落，齿槽脓肿等；骨盆、脊椎、肋骨、肩胛骨和乳突等亦常受累。

（3）突眼：因眶骨破坏而表现为眼球凸出和眼睑下垂，多为单侧。

（4）尿崩：垂体和下丘脑组织受浸润所致，个别患儿可见蝶鞍破坏。

（5）其它：有孤立、稀疏的黄色丘疹，呈黄色瘤状；久病者可导致发育迟缓。

3．骨嗜酸细胞肉芽肿

（1）发病年龄：各年龄组都可发病，尤多于4～7 岁。

（2）骨骼破坏：本型的主要表现多为单发病灶，常无软组织和器官的损害。病变局部肿胀而微痛，无红热，有时可见病理性骨折。任何骨均可受累，但以扁平骨较多见，颅骨最常见，其他有下颌骨、四肢骨、骨盆骨和脊椎等。椎骨受累可出现脊髓压迫症状。

（3）其他：多发病灶者可伴有发热、厌食、体重减轻等；偶有肺嗜酸性粒细胞肉芽肿。

4．混合型 多于1～2 岁起病，其临床表现相当于勒-雪病和韩-薛-柯氏病联合表现，主要为发热、贫血、耳溢脓、肺部浸润、肝脾大等。重者除有LS的典型皮疹和肺部改变外，同时又具有HSC的尿崩、突眼和骨质缺损；轻者可无皮疹或皮疹不典型，但多有骨骼破坏。

5．单一器官损害型 各年龄组都有报道，可单独发生于肺、肝、脾、淋巴结、皮肤等器官，而不伴其他器官损害。临床表现取决于所受累及的器官和损害的程度。

【辅助检查】

1．血液学检查 LS患者常呈不同程度的贫血；白细胞数正常、减少或增多；血小板数正常或减少。HSC血象改变较LS少而轻。EGB多无血象变化。10％～15％患者骨髓可见组织细胞增多，偶见巨核细胞减少。

2．X线检查 对诊断很有帮助，不少病例系由X线检查最先发现。

（1）胸部：肺部是最易受累的器官之一。典型改变为肺野透亮度减低呈毛玻璃状，两肺弥散的网状或网点状阴影，或在网点状基础上有局限或弥散的颗粒阴影，须与粟粒性肺结核鉴别。严重者可见弥散性小囊肿、肺气肿、气胸、纵隔气肿或皮下气肿等。婴幼儿常见胸腺肿大。

（2）骨骼：病变部位呈虫蚀样改变至巨大缺损，为溶骨性凿穿样损害，形状不规则，呈圆或椭圆形。脊椎改变多表现为椎体破坏，偶见椎旁脓肿。下颌骨浸润时牙槽硬板及支持骨破坏，出现漂浮齿征象。

3．病理检查 皮疹压片和病灶活体组织检查发现LC是诊断的重要依据。皮疹压片法检查操作简便，患者痛苦小，阳性率高。可作皮疹、淋巴结、齿龈或肿物的活体组织检查或病灶局部穿刺物或刮出物的病理检查。有条件时应作电镜检查。病理切片发现CD31/S-100、CD1a、CD40/CD40L和趋化因子CCR6及其配体CCL20/MIP3α表达增加。

4．其他 α-D甘露糖酶试验阳性，花生凝集素结合试验阳性。

【诊断】

凡原因不明的发热、皮疹、贫血、耳溢脓、反复肺部感染，肝、脾、淋巴结肿大，眼球凸出、尿崩、颅骨缺损、头部肿物等均应疑及本病。诊断需临床、X线和病理三方面结合。病理检查是本病诊断最可靠的依据，尤其是电镜下找到Birbeck颗粒的LC，结合临床即可确诊。1987年国际组织细胞协会协作组订出了病理诊断标准如下：

1．初诊 压片、皮肤活体组织检查、淋巴结、肿物穿刺或手术标本发现组织细胞浸润。

2．诊断 初诊的基础上，且具下述4 项指标的2 项或2 项以上：①ATP酶阳性；②CD31/100阳性表达；③α-D甘露糖酶阳性；④花生凝集素结合试验阳性。

3．确诊 电镜在病变细胞内发现Birbeck颗粒和（或）CD1a抗原阳性。

1987年Lavin和Osband根据影响预后的三大因素，即发病年龄、受累器官数目及有无功能损害进行积分，将本病分为4 级（0分： Ⅰ级，1分： Ⅱ级，2分：Ⅲ级，3分：Ⅳ级），对指导治疗、判断预后有较大的意义（表15-6）。

表15-8 LCH的临床评分及分级

	年龄		受累器官		器官功能损害*	
评分条件	>2 岁	≤2 岁	<4 个	≥4 个	无	有
计　　分	0	1	0	1	0	1

*注：肝功能有下列1 项异常者：如①低蛋白血症，总蛋白<55 g/L或白蛋白<25 g/L；②胆红素>25.7 μmol/L（1.5mg/dl）；③水肿或腹水。呼吸功能在无感染的情况下，有下列1项损害者：如呼吸困难、紫绀、胸水或气胸等。造血功能损害，出现下列1 项异常者：如血红蛋白<l00 g/L（除外缺铁性贫血），白细胞<4×10^9/L 和血小板<100~10^9/L。

【治疗】

由于本病变化多样、轻重悬殊，治疗方案应根据临床分型和分级而定。

1．药物治疗 近年来由于化学药物等综合治疗措施进展，本病尤其是重症患者的预后大为改观。由于本病不是恶性细胞浸润，目前多不主张强化疗方案，以避免严重的毒副反应。

（1）化学治疗：常用的药物有泼尼松、长春新碱、足叶乙甙（VP-16）、环磷酰胺等。VP方案：泼尼松，每日40～60 mg/m^2，分次口服；长春新碱每次1.5～2 mg/m^2，每周静脉注射1 次；一般用8～10 周。VP方案可使多数Ⅰ级或Ⅱ级患者获得缓解。VCP方案为上述方案加环磷酰胺（CTX）：VP同上，CTX每次200 mg/m^2，静脉滴注，每周1 次，共6～8 周。此后可用6-MP和MTX维持，或定期用原方案。总疗程根据病情而定，轻者半年，重者可长达2 年。近年来主张采用足叶乙甙150 mg/m^2，静脉滴注，或300 mg/m^2，口服，连用3 天，每3～4 周为1 疗程，共用6 个月。该药对其他化疗药物耐药者效果明显。

（2）免疫治疗：病情严重的Ⅲ～Ⅳ级患儿，在化疗的同时，可加用胸腺肽1～2 mg/次，肌肉注射，隔日1 次。亦可试用α-干扰素和环孢菌素A，对于减少化疗的毒副反应，改善免疫功能有一定作用。

（3）其他：对于单纯骨损害者，可试用吲哚美辛（indomethacin），每日1～2.5 mg/kg，平均疗程6 周，有一定的疗效。尿崩症可用鞣酸加压素或去氨加压素（DDAVP）治疗。生长发育障碍者可试用生长激素。

2．放射治疗 小剂量（4～6 Gy）局部照射可控制局限性损害，也适于病变广泛或病变部位不能手术者。

3．手术治疗 局部EGB可手术刮除。<5 岁者可采用手术加化疗，或单用化疗。

4．其他 控制感染，加强支持治疗。

【预后】

本病预后与发病年龄、受累器官多少、器官功能损害及初期治疗反应有关。年龄愈小，受累器官愈多，预后愈差；年龄>5 岁，单纯骨损害者多可自愈；肺、肝、脾、骨髓等受侵犯且对初期治疗反应较差者预后差。痊愈患儿中少数可有尿崩、智力低下、发育迟缓、颌骨发育不良等后遗症。

（盛光耀）

第十六章 神经肌肉系统疾病

第一节 神经系统疾病检查方法

一、神经系统体格检查

小儿神经系统的检查，原则上与成人相同，但由于小儿神经系统发育尚未成熟，加之体格检查时常不合作，因而小儿神经系统检查也有其特殊性。有的表现如伸直性跖反射，在成人或年长儿属病理性，但在婴幼儿却是一种暂时的生理现象。临床各种辅助检查中，年龄越小，不同年龄间正常差异也越大。因此，对小儿神经系统的检查与评价，均不能脱离相应年龄期的正常生理学特征。

（一）一般检查

1．意识和精神行为状态 可根据小儿对各种刺激的反应来判断意识水平（即意识深、浅度）有无障碍，由轻而重分为嗜睡、昏睡、半昏迷和昏迷等。少数主要表现为谵妄、定向力丧失和精神行为异常等意识内容的减少或异常。

智力低下者常表现为交流困难、脱离周围环境的异常情绪与行为等。

2．头颅 头围可粗略反映颅内组织容量。头围过大时要注意脑积水、硬膜下血肿、巨脑症等。头围过小警惕脑发育停滞或脑萎缩，但大约2%～7%的小头围儿童，智力仍可能正常。

注意囟门和颅骨缝，过早闭合见于小头畸形。囟门增大伴膨隆、张力增高、以及颅缝开裂等均提示颅压增高，颅骨叩诊时尚可得“破壶音”。对疑有硬膜下积液、脑穿通畸形婴儿，可在暗室内用电筒紧贴颅骨做透照试验，前额部光圈>2 cm，枕部>l cm，或两侧不对称时对诊断有提示作用。

3．皮肤 某些神经疾病可伴有特征性皮肤损害，包括皮肤色素脱失斑、面部血管纤维瘤、皮肤牛奶咖啡斑或面部血管痣等。

（二）颅神经检查

1．嗅神经 反复观察对香水、薄荷或某些不适气味的反应。嗅神经损伤常见于先天性节细胞发育不良，或额叶、颅底病变者。

2．视神经 主要检查视力、视野和眼底。

（1）视力：未成熟儿已能对强光表现皱眉或不安。3 个月婴儿开始用双眼注视并跟随移动中的物体。视力表测试下，2 岁的视力约为6/12，3 岁前达20/20的成人水平。

（2）视野：年长儿可直接用视野计。对婴幼儿，检查者可站在婴儿背后，或与其面对面地将色彩鲜艳玩具（对婴儿）或白色视标，由侧面远端缓慢移入视野内，注意婴儿眼和头是否转向玩具或患儿见到视标的表情，并以检查者自己视野作比较，粗测有无视野异常。

（3）眼底：检查婴幼儿眼底较困难，必要时扩瞳后进行。正常新生儿因血管少视乳头颜色较白，不要误为视神经萎缩。慢性颅内高压时可见视乳头水肿和视网膜静脉淤血。

3．动眼、滑车和展神经 观察有无眼睑下垂、眼球震颤、斜视等。检查眼球向上、向下和向两侧的眼外肌运动。注意瞳孔大小、形状以及对光反射、会聚和调节反应等。

4．三叉神经 注意张口下颌有无偏斜，咀嚼时扪两侧咬肌及颞肌收缩力以判断其运动支功能。观察额、面部皮肤对痛刺激反应，并用棉花絮轻触角膜，检查角膜反射以了解感觉支功能。

5．面神经 观察随意运动或表情运动（如哭或笑）中双侧面部是否对称。周围性面神经麻痹时，患侧上、下面肌同时受累，表现为病变侧皱额不能、眼睑不能闭合、鼻唇沟变浅和口角向健侧歪斜。中枢性面瘫时，病变对侧鼻唇沟变浅，口角向病变侧歪斜，但无皱额和眼睑闭合功能的丧失。

6．听神经和前庭神经 观察小儿对突然声响或语声反应，以了解有无听力损害。突然响声可引发新生儿惊跳或哭叫。3 个月起婴儿头可转向声源方向。对可疑患者，应进行特殊听力测验。

可选用旋转或冷水试验测定前庭功能。旋转试验时，检查者面对面地将婴儿平举，并原地旋转4～5 圈，休息5～10 分钟后用相同方法向另一侧旋转。冷水试验时，检查者以冷水（2～4 ml）外耳道灌注。此法可测定单侧前庭功能，其结果较旋转试验准确。正常小儿在旋转中或冷水灌注后均出现眼球震颤，前庭神经病变时则不能引出眼球震颤。

7．舌咽和迷走神经 舌咽神经损害引起咽后壁感觉减退和咽反射消失。临床常合并迷走神经损害，共同表现为吞咽困难、声音嘶哑、呼吸困难及鼻音等。由于受双侧皮层支配，单侧核上性病变时可无明显症状。

8．副神经 检查胸锁乳突肌和斜方肌的肌力、肌容积。病变时患侧肩部变低，耸肩、向对侧转头力减弱。

9．舌下神经 其主要作用是将舌伸出。一侧中枢性舌下神经麻痹时，伸舌偏向对侧，即舌肌麻痹侧；而一侧周围性舌下神经瘫痪时，伸舌偏向麻痹侧，且伴舌肌萎缩与肌纤维颤动。

（三）运动功能检查

1．肌容积 有无肌肉萎缩或假性肥大。

2．肌张力 指安静情况下的肌肉紧张度。检查时触扪肌肉硬度并作被动运动，以体会肌紧张度与阻力。肌张力增高多见于上运动神经元性损害和锥体外系病变，但注意半岁内正常婴儿肌张力也可稍增高。下运动神经元或肌肉疾病时肌张力降低，肌肉松软，甚至关节可以过伸。

3．肌力 是指肌肉做主动收缩时的力量。观察小儿力所能及的粗大和精细运动，以判断各部位肌群的肌力。年长儿则可按指令完成各种对抗运动。令小儿完成登楼梯、从蹲位或仰卧位站起等动作，可重点测试髋带和下肢近端肌力。用足尖或足跟走路分别反映小腿后群或前群肌肉肌力。一般把肌力分为0～5 级，0 级：完全瘫痪，无任何肌收缩活动；1 级：可见轻微肌收缩但无肢体移动；2 级：肢体能在床上移动但不能抬起；3 级：肢体能抬离床面但不能对抗阻力；4 级：能做部分对抗阻力的运动；5 级：正常肌力。

4．共济运动 可观察婴儿手拿玩具的动作是否准确。年长儿则能和成人一样完成指鼻、闭目难立、跟膝胫和轮替运动等检查。然而，当患儿存在肌无力或不自主运动时，也会出现随意运动不协调，不要误认为共济失调。

5．姿势和步态 姿势和步态与肌力、肌张力、深感觉、小脑以及前庭功能都有密切关系。观察小儿各种运动中姿势有何异常。常见的异常步态包括：双下肢的剪刀式或偏瘫性痉挛性步态，足间距增宽的小脑共济失调步态，高举腿、落足重的感觉性共济失调步态，髋带肌无力的髋部左右摇摆“鸭步”等。

6．不自主运动 主要见于锥体外系疾病，常表现为舞蹈样运动、扭转痉挛、手足徐动症或一组肌群的抽动等。每遇情绪紧张或进行主动运动时加剧，入睡后消失。

（四）感觉功能检查

由于疾病特征，对小儿的感觉检查一般不如成人重要。而且，临床很难在学龄前儿童获得充分合作。既使在学龄儿童，也往往需要检查者更多耐心及反复检查。具体检查方法与成人基本相同：

1．浅感觉 包括痛觉、触觉和温度觉。痛觉正常者可免去温度觉测试。

2．深感觉 位置觉、音叉震动觉。

3．皮层感觉 闭目状态下测试两点鉴别觉，或闭目中用手辨别常用物体的大小、形态或轻重等。

（五）反射检查

小儿的反射检查可分为两大类，第一类为终身存在的反射，即浅反射及腱反射；第二类为暂时性反射，或称原始反射（primitive reflexes）。

1．浅反射和腱反射

（1）浅反射：腹壁反射要到1 岁后才比较容易引出，最初的反应呈弥散性。提睾反射要到出生4～6 个月后才明显。

（2）腱反射：新生儿期已可引出肱二头肌、膝和踝反射。腱反射减弱或消失提示神经、肌肉、神经肌肉接合处或小脑疾病。反射亢进和踝阵挛提示上运动神经元疾患。恒定的一侧性反射缺失或亢进有定位意义。

2．小儿时期暂时性反射 生后最初数月婴儿存在许多暂时性反射。随年龄增大，各自在一定的年龄期消失，见表16-1。当它们在应出现的时间内不出现，或该消失的时间不消失，或两侧持续地不对称都提示神经系统异常。

表16-1 正常小儿暂时性反射的出现和消失年龄

反 射	出现年龄	消失年龄
拥抱反射	初生	3～6 个月
吸吮反射和觅食反射	初生	4～7 个月
掌握持反射	初生	3～4 个月
颈肢反射	2 个月	6 个月
支撑反射	初生	2～3 个月
迈步反射	初生	2 个月
颈拨正反射	初生	6 个月

另外，正常小儿9～10 个月出现降落伞反射，此反射可持续终生。如不能按时出现，则提示有脑瘫或发育迟缓的可能。

（六）病理反射

包括巴彬斯基（Babinski）征、卡道克（Chaddock）征、戈登（Gordon）征和奥本海姆（Oppenheim）征等，检查和判断方法同成人。

然而，正常2 岁以下婴儿可呈现巴彬斯基征阳性，多表现为拇趾背伸但少有其他脚趾的扇形分开。检查者用拇指紧压婴儿足底也可引出同样阳性反应。若该反射恒定不对称或2 岁后继续阳性时，提示锥体束损害。

（七）脑膜刺激征

包括颈强直、屈髋伸膝试验（Kernig征）和抬颈试验（Brudzinski征）。检查方法同成人。

二、神经系统辅助检查

（一）脑脊液检查

腰椎穿刺取脑脊液（cerebral spinal fluid，CSF）检查，是诊断颅内感染和蛛网膜下腔出血的重要依据。脑脊液可被用于多种项目的检测，主要包括外观、压力、常规、生化和病原学检查等。然而，对严重颅内压增高的患儿，在未有效降低颅压之前，腰椎穿刺有诱发脑疝的危险，应特别谨慎。颅内几种常见感染疾病的CSF改变特征见表16-2。

表16-2 颅内常见感染性疾病的脑脊液改变特点

	压力(kPa)	常规分析			生化分析			其他
		外观	Pandy试验	白细胞($\times 10^6$/L)	蛋白(g/L)	糖(mmol/L)	氯化物(mmol/L)	
正常	0.69~1.96<br>新生儿：0.29~0.78	清亮透明	−	0~10<br>婴儿：0~20	0.2~0.4<br>新生儿：0.2~1.2	2.8~4.5<br>婴儿：3.9~5.0	117~127<br>婴儿：110~122	
化脓性脑膜炎	不同程度增高	米汤样混浊	+~+++	数百~数千，多核为主	增高或明显增高	明显降低	多数降低	涂片Gram染色和培养可发现致病菌
结核性脑膜炎	不同程度增高	微浑，毛玻璃样	+~+++	数十~数百，淋巴为主	增高或明显增高	明显降低	多数降低	薄膜涂片抗酸染色及培养可发现抗酸杆菌
病毒性脑膜炎	不同程度增高	清亮，个别微浑	−~+	正常~数百，淋巴为主	正常或轻度增高	正常	正常	特异性抗体阳性，病毒培养可能阳性
隐球菌性脑膜炎	高或很高	微浑，毛玻璃样	+~+++	数十~数百，淋巴为主增高或明显增高	增高或明显增高	明显降低	多数降低	涂片墨汁染色和培养可发现致病菌

（二）脑电图和主要神经电生理检查

1．脑电图（electroencephalography，EEG） 是对大脑皮层神经元电生理功能的检查。包括：

（1）常规EEG：借助电子和计算机技术从头皮记录皮层神经元的生物电活动。主要观察：①有无棘波、尖波、棘-慢复合波等癫痫样波，以及它们在不同脑区的分布，是正确诊断癫痫、分型与合理选药的主要实验室依据；②清醒和睡眠记录的背景脑电活动是否正常。全脑或局部的各种原因脑损伤，均可引起相应脑区的脑电活动频率慢化。不同年龄期的背景脑活动差异很大，若只用一个标准去判断不同年龄期EEG易导致结论的假阳性。记录时间不足20 分钟，未作睡眠中记录是导致结论假阴性的主要因素。

（2）动态EEG（ambulatory EEG，AEEG）：连续进行24 小时甚至数日的EEG记录。因增加描记时间而提高异常阳性率。若同时获得发作期EEG，更有助癫痫诊断和分型。

（3）录像EEG（video-EEG，VEEG）：不仅可长时程地记录EEG，更可实时录下患者发作中表现以及同步的发作期EEG，对癫痫的诊断、鉴别诊断和分型有更大帮助。

2．诱发电位 分别经听觉、视觉和躯体感觉通路，刺激中枢神经诱发相应传导通路的反应电位。包括：

（1）脑干听觉诱发电位（BAEP）：以耳机声刺激诱发。因不受镇静剂、睡眠和意识障碍等因素影响，可用于包括新生儿在内任何不合作儿童的听力筛测，以及昏迷患儿脑干功能评价。

（2）视觉诱发电位（VEP）：以图像视觉刺激（patterned stimuli）诱发称PVEP，可分别检出单眼视网膜、视神经、视交叉、视交叉后和枕叶视皮层间视通路各段的损害。婴幼儿不能专心注视图像，可改闪光刺激诱发，称FVEP，但特异性较差。

（3）体感诱发电位（SEP）：以脉冲电流刺激肢体混合神经，沿体表记录感觉传入通路反应电位。脊神经根、脊髓和脑内病变者可出现异常。

3．周围神经传导功能 习称神经传导速度（NCV）。帮助了解被测周围神经有无损害、损害性质（髓鞘或轴索损害）和严重程度。据认为，当病变神经中有10％以上原纤维保持正常时，测试结果可能正常。

4．肌电图（EMG） 帮助了解被测肌肉有无损害和损害性质（神经源性或肌源性）。

（三）神经影像学检查

1．电子计算机断层扫描（computed tomography，CT） 可显示不同层面脑组织、脑室系统、脑池和颅骨等结构形态。必要时注入造影剂以增强扫描分辨率。CT能较好显示病变中较明显的钙化影和出血灶，但对脑组织分辨率不如MRI高，且对后颅窝、脊髓病变因受骨影干扰难以清楚辨认。

2．磁共振成像（magnetic resonance imaging，MRl） 无放射线。对脑组织和脑室系统分辨率较CT高，能清楚显示灰、白质和基底节等脑实质结构。由于不受骨影干扰，能很好地发现后颅窝和脊髓病灶。同样可作增强扫描进一步提高分辨率。主要缺点是费用较CT高，成像速度较慢，对不合作者需用镇静剂睡眠中检查，对钙化影的显示较CT差。

3．其他 如磁共振血管显影（MRA）、数字减影血管显影（DSA）用于脑血管疾病诊断。单光子发射断层扫描（SPECT）和正电子发射断层扫描（PET）均属于功能影像学，是根据放射性示踪剂在大脑组织内的分布或代谢状况，显示不同脑区的血流量或代谢率，对癫痫放电源的确认有重要帮助。

第二节 癫 痫

癫痫（epilepsy）是脑部的一种慢性疾患，其特点是大脑神经元反复发作性异常放电引起相应的突发性和一过性脑功能障碍。癫痫发作（epileptic seizures或seizures）大多短暂并有自限性，由于异常放电所累及的脑功能区不同，临床可有多种发作表现，包括局灶性或全身性的运动、感觉异常，或是行为认知、自主神经功能障碍。全身性发作和涉及一些较大范围皮质功能障碍的局灶性发作，往往伴有程度不同的意识障碍。

癫痫发作和癫痫是两个不同的概念，前者是指发作性皮质功能异常所引起的一组临床症状，而后者是指临床呈长期反复痫性发作的疾病过程。儿科临床常用惊厥（convulsion）这一概念，一般来说是指伴有骨骼肌强烈收缩的痫性发作。一些痫性发作如典型失神、感觉性发作等，于发作过程中并不伴有骨骼肌动作，因而属于非惊厥性的痫性发作。因此，无论是惊厥性的痫性发作还是非惊厥性的痫性发作，都是指一组临床症状。它们虽是癫痫患者的基本临床表现，但类似的临床发作也可出现在许多非癫痫性疾病过程中，如热性惊厥、颅内感染、颅脑损伤、代谢异常或中毒等，在这种情况下，它们仅是急性疾病的临床症状，随急性病的好转而消失，由于不具备癫痫患者长期慢性和反复发作的基本特征，因而不能诊断为癫痫。

据国内多次大样本调查，我国癫痫的年发病率约为35/10 万人口，累计患病率约3.5‰～

4.8‰。然而，其中60%的患者起源于小儿时期。长期、频繁或严重的痫性发作会导致进一步脑损伤，甚至出现持久性神经精神障碍。但是，由于医学的发展和进步，只要做到早期诊断与合理治疗，已能使80%以上的癫痫患儿发作得到满意控制。因此，做好小儿时期的癫痫防治工作具有十分重要的意义。

【病因】

根据病因，可粗略地将癫痫分为三大类，包括：①特发性（idiopathic）癫痫：又称原发性癫痫，是指由遗传因素决定的长期反复癫痫发作，不存在症状性癫痫可能性者；②症状性（symptomatic）癫痫：又称继发性癫痫，痫性发作与脑内器质性病变密切关联；③隐原性（cryptogenic）癫痫：虽未能证实有肯定的脑内病变，但很可能为症状性者。

随着脑影像学和功能影像学技术的发展，近年来对癫痫的病因有了重新认识。与遗传因素相关者约占癫痫总病例数的20%～30%，故多数患儿为症状性或隐原性癫痫，其癫痫发作与脑内存在的或可能存在的结构异常有关。

1. 脑内结构异常 先天或后天性脑损伤可产生异常放电的致痫灶，或降低了痫性发作阈值，如各种脑发育畸形、染色体病和先天性代谢病引起的脑发育障碍、脑变性和脱髓鞘性疾病、宫内感染、肿瘤以及颅内感染、中毒、产伤或脑外伤后遗症等。

2. 遗传因素 包括单基因遗传、多基因遗传、染色体异常伴癫痫发作、线粒体脑病等。过去主要依赖连锁分析和家族史来认定其遗传学病因。近年来依靠分子生物学技术，至少有10 余种特发性癫痫或癫痫综合征的致病基因得到了克隆确定，其中大多数为单基因遗传，系病理基因致神经细胞膜的离子通道功能异常，降低了病性发作阈值而患病。

3. 诱发因素 许多体内、外因素可促发癫痫的临床发作，如遗传性癫痫常好发于某一特定年龄阶段，有的癫痫则主要发生在睡眠或初醒时，女性患儿青春期来临时易有癫痫发作的加重等。此外，饥饿、疲劳、睡眠不足、过度换气、预防接种等均可能成为某些癫痫的诱发因素。

【癫痫发作的分类及其临床表现】

（一）痫性发作分类

对痫性发作进行正确分类有十分重要的临床意义。因为针对不同的发作类型，通常应选用不同的抗癫痫药物；而且对分析病因、估计患儿病情与预后，均有重要价值。结合发作中的临床表现和相伴随的脑电图特征，国际抗癫痫联盟（ILAE）于1981 年提出对发作类型的国际分类，迄今仍是临床工作的重要指南。1983 年我国小儿神经学术会议将其简化，见表16-3。2001年ILAE又提出了新的更详细的分类方案，但因太复杂，这里不作介绍，请参考有关专业书籍。

表16-3 痫性发作的国际分类

Ⅰ. 局灶性发作	Ⅱ. 全部性发作	Ⅲ. 不能分类的发作
单纯局灶性（不伴意识障碍）	强直-阵挛发作	
运动性发作	强直性发作	
感觉性发作	阵挛性发作	
植物神经性发作	失神发作	
精神症状发作	典型失神	
复杂局灶性（伴有意识障碍）	不典型失神	
单纯局灶性发作继发意识障碍	肌阵挛发作	
发作起始即有意识障碍的局灶性发作	失张力发作	
局灶性发作继发全身性发作	婴儿痉挛	

（二）癫痫发作的临床表现

1．局灶性（部分性、局限性）发作 发作期脑电图(EEG)可见某一脑区的局灶性痫性放电。

（1）单纯局灶性发作：发作中无意识丧失，也无发作后不适现象。持续时间平均10～20秒。其中以局灶性运动性发作最常见，表现为面、颈或四肢某部分的强直或阵挛性抽动，特别易见头、眼持续性同向偏斜的旋转性发作（adversive seizure）。年长儿可能会诉说发作初期有头痛、胸部不适等先兆。有的患儿于局限性运动发作后出现抽搐后肢体短暂麻痹，持续数分钟至数小时后消失，称为Todd麻痹。

局灶性感觉发作（躯体或特殊感觉异常）、自主神经性发作和局灶性精神症状发作在小儿时期少见，部分与其年幼无法表达有关。

（2）复杂局灶性发作：见于颞叶和部分额叶癫痫发作。可从单纯局灶性发作发展而来，或一开始即有意识部分丧失伴精神行为异常。50%～75%的儿科病例表现为意识混浊情况下的自动症（automatism），如吞咽、咀嚼、解衣扣、摸索行为或自言自语等。少数患者表现为发作性视物过大或过小、听觉异常、冲动行为等。

（3）局灶性发作演变为全面性发作：由单纯局灶性或复杂局灶性发作扩展为全面性发作。

2．全身性发作 指发作中两侧半球同步放电，均伴有程度不等的意识丧失。

（1）强直-阵挛发作：又称大发作（grand mal）。是临床最常见的发作类型之一，包括原发性以及从局灶性扩展而来的继发性全面性强直-阵挛发作。发作主要分为两期：一开始为全身骨骼肌伸肌或屈肌强直性收缩伴意识丧失、呼吸暂停与发绀，即强直期；紧接着全身反复、短促的猛烈屈曲性抽动，即阵挛期。常有头痛、嗜睡、疲乏等发作后现象。发作中EEG呈全脑棘波或棘-慢复合波发放，继发性者从局灶放电扩散到全脑。部分年长儿能回忆发作前先有眼前闪光、胸中一股气向上冲等先兆，直接提示继发性癫痫的可能性。

（2）失神发作：发作时突然停止正在进行的活动，意识丧失但不摔倒，手中物品不落地，两眼凝视前方，持续数秒钟后意识恢复，对刚才的发作不能回忆，过度换气往往可以诱发其发作。EEG有典型的全脑同步3 Hz棘-慢复合波。

（3）非典型失神发作：与典型失神发作表现类似，但开始及恢复速度均较典型失神发作慢，EEG为1.5～2.5 Hz的全脑慢-棘慢复合波。多见于伴有广泛性脑损害的患儿。

（4）肌阵挛发作：为突发的全身或部分骨骼肌触电样短暂收缩（<0.35 秒），常表现为突然点头、前倾或后仰，而两臂快速抬起。重者致跌倒，轻者感到患儿“抖”了一下。发作中通常伴有全脑棘-慢或多棘慢波爆发。大多见于有广泛性脑损伤的患儿。

（5）阵挛性发作：仅有肢体、躯干或面部肌肉节律性抽动而无强直发作成分。

（6）强直性发作：突发的全身肌肉强直收缩伴意识丧失，使患儿固定于某种姿势，但持续时间较肌阵挛长，约5～60 秒。常见到角弓反张、伸颈、头仰起、头躯体旋转或强制性张嘴、睁眼等姿势，通常有跌倒和发作后症状。发作间期EEG背景活动异常，伴多灶性棘-慢或多棘慢波爆发。

（7）失张力发作：全身或躯体某部分的肌肉张力突然短暂性丧失伴意识障碍。前者致患儿突然跌倒、头着地甚至头部碰伤。部分性失张力发作者表现为点头样或肢体突然下垂动作。EEG见节律性或不规则、多灶性棘-慢复合波。

（8）痉挛：这种发作最常见于婴儿痉挛，表现为同时出现点头、伸臂（或屈肘）、弯腰、踢腿（或屈腿）或过伸样等动作，其肌肉收缩的整个过程大约1～3秒，肌收缩速度比肌阵挛发作慢，持续时间较长，但比强直性发作短。

【小儿时期常见的几种癫痫和癫痫综合征】

某些癫痫患者无论其病因是否相同，因具有一组相同的发作症状和体征，在临床上称为特殊癫痫综合征，在治疗和预后的估计上有其特殊性。为此，国际抗癫痫联盟于1989 年提出了癫痫和癫痫综合征的分类，2001 年又有许多新的补充，具体内容可参阅神经科专业书籍。以下介绍儿科常见的几种癫痫综合征。

1．伴中央颞区棘波的儿童良性癫痫（benign childhood epilepsy with centrotemporal spikes）是儿童最常见的一种癫痫综合征，占小儿时期癫痫的15%～20%。约30%的患者有类似家族史。多数认为属常染色体显性遗传，但外显率低且有年龄依赖性。通常2～14 岁间发病，5～10 岁多见，8～9 岁为高峰，男略多于女。3/4的发作在入睡后不久及睡醒前。发作大多起始于口面部，呈局灶性发作，如唾液增多、喉头发声、不能主动发声或言语以及面部抽搐等，部分患儿很快继发全身性强直-阵挛发作而意识丧失，常因此时被家人发现而被描述为全身性抽搐。

体格检查无异常。发作间期EEG背景正常（图16-1），在中央区和颞中区可见棘、尖波或棘-慢复合波，一侧、两侧或交替出现，30%的患儿仅在睡眠记录中出现异常。本病预后良好，药物易于控制，生长发育不受影响，大多在12～16岁前停止发作，但不足2%的病例可能继续有癫痫发作。

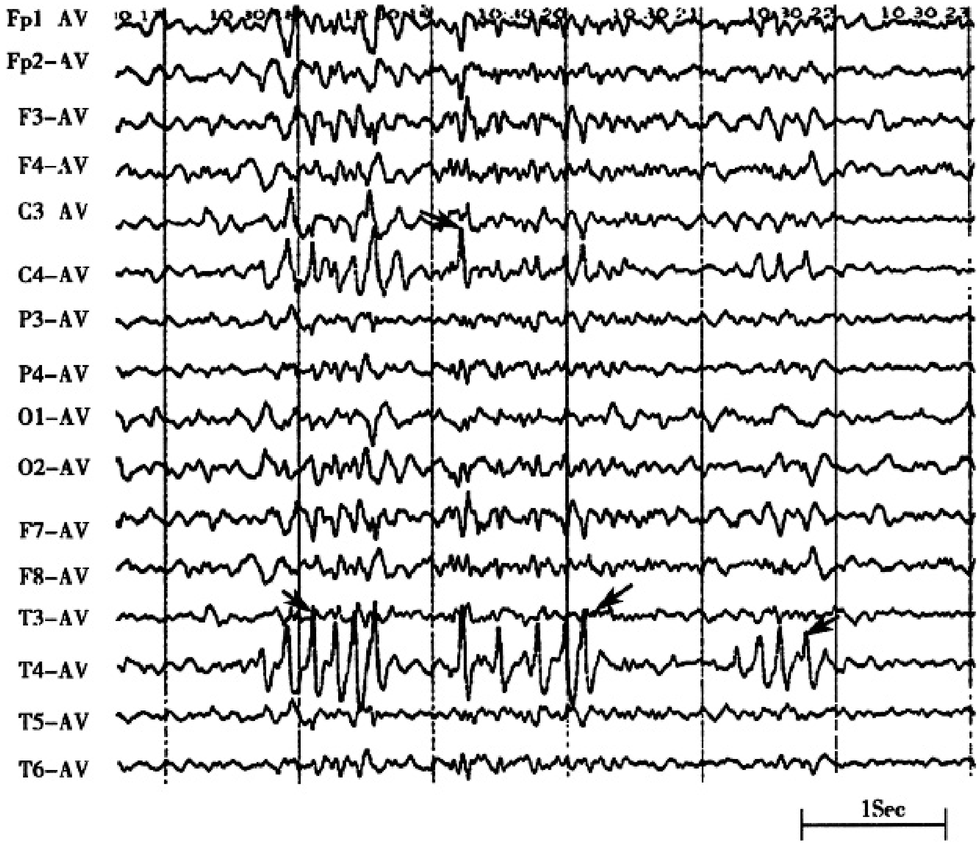

图16-1　伴中央颞区棘波的儿童良性癫痫患儿发作间期EEG

11岁半男孩浅睡中记录。患儿近1个月来多次入睡后不久突发全身强直-阵挛发作。

无论清醒或浅睡记录中，右中央和右颞中区均可见100~280 μV棘、尖波爆发

（如箭头所示），睡眠中更频繁，清醒EEG背景活动正常

2．儿童失神癫痫（childhood absence epilepsy） 大多于3～13 岁间发病，6～7 岁为高峰，近2/3为女孩，有明显遗传倾向。表现为频繁的失神发作，每日数次甚至上百次。每次发作数秒钟，不超过30 秒，因而不跌倒，也无明显体位改变。患儿不能回忆发作中的情况，无头痛、嗜睡等发作后症状，体格检查无异常。EEG为特征性全部性3 Hz棘-慢复合波爆发（图16-2），

过度换气常可诱发特征EEG爆发图形和临床发作。药物易于控制，预后大多良好。

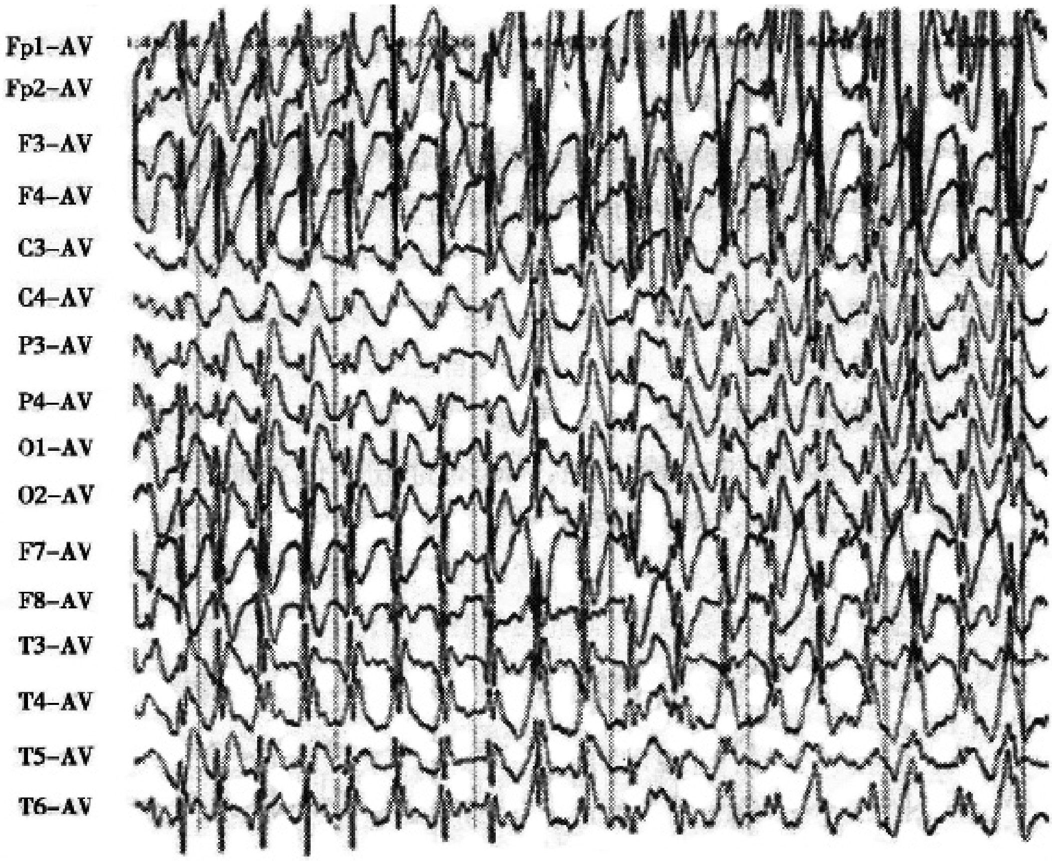

图16-2 儿童失神癫痫全部性3 Hz棘-慢复合波爆发

8 岁男性，近3 个月来经常短暂双目凝视失神，每日10 余次，但从无跌倒。

过度换气EEG中爆发全部性3 Hz棘-慢复合波，额、颞前区波幅最高

3．婴儿痉挛（infantile spasm） 又称West综合征。本病以1 岁前婴儿期起病（生后4～8 月为高峰）、频繁的痉挛发作、特异性高幅失律EEG图形以及病后精神运动发育倒退为其基本临床特征。痉挛发作主要表现为屈曲性、伸展性和混合性三种形式，但以混合性和屈曲性居多。典型屈曲性痉挛发作时，婴儿呈点头哈腰屈（或伸）腿状，伸展性发作时婴儿呈角弓反张样。痉挛多成串地发作，每串连续数次或数十次，动作急速，可伴有婴儿哭叫。常于思睡和甦醒期加重。高幅失律EEG图形对本病诊断有价值（图16-3）。在不同步、不对称并有爆发抑制交替倾向的高波幅慢波背景活动中，混有不规则的多灶性棘、尖与多棘慢波爆发。睡眠记录更易获得典型高幅失律图形。

其病因复杂，大致可分为隐原性和症状性两大类。后者是指发病前已有宫内、围生期或生后脑损伤证据，如精神运动发育迟缓、异常神经系统体征或头颅影像学改变等，治疗效果差，80%以上存在遗留智力低下的危险。约20%的婴儿痉挛病例属隐原性，病前无脑损伤证据可寻。若早期治疗，40%的患儿可望获得基本正常的智力和运动发育。

4．Lennox-Gastaut综合征（简称LGS） 本综合征以儿童期（1～8 岁）起病、频繁而多样的发作形式、慢-棘慢（<3Hz）复合波EEG以及智力、运动发育倒退为基本特征。25%以上有婴儿痉挛病史。患儿每天同时有多种形式发作，其中以强直性最多见，其次为不典型失神或失张力发作，还可有强直-阵挛、肌阵挛等。非快速眼动（NREM）睡眠期较清醒时发作更频繁。多数患儿的智力和运动发育倒退。EEG显示在异常慢波背景活动上重叠1.5～2.5 Hz慢-棘慢复合波（图16-4）。治疗困难，1/3以上患儿对多种抗癫痫药物无效，是儿童期最常见的一种难治性癫痫综合征。

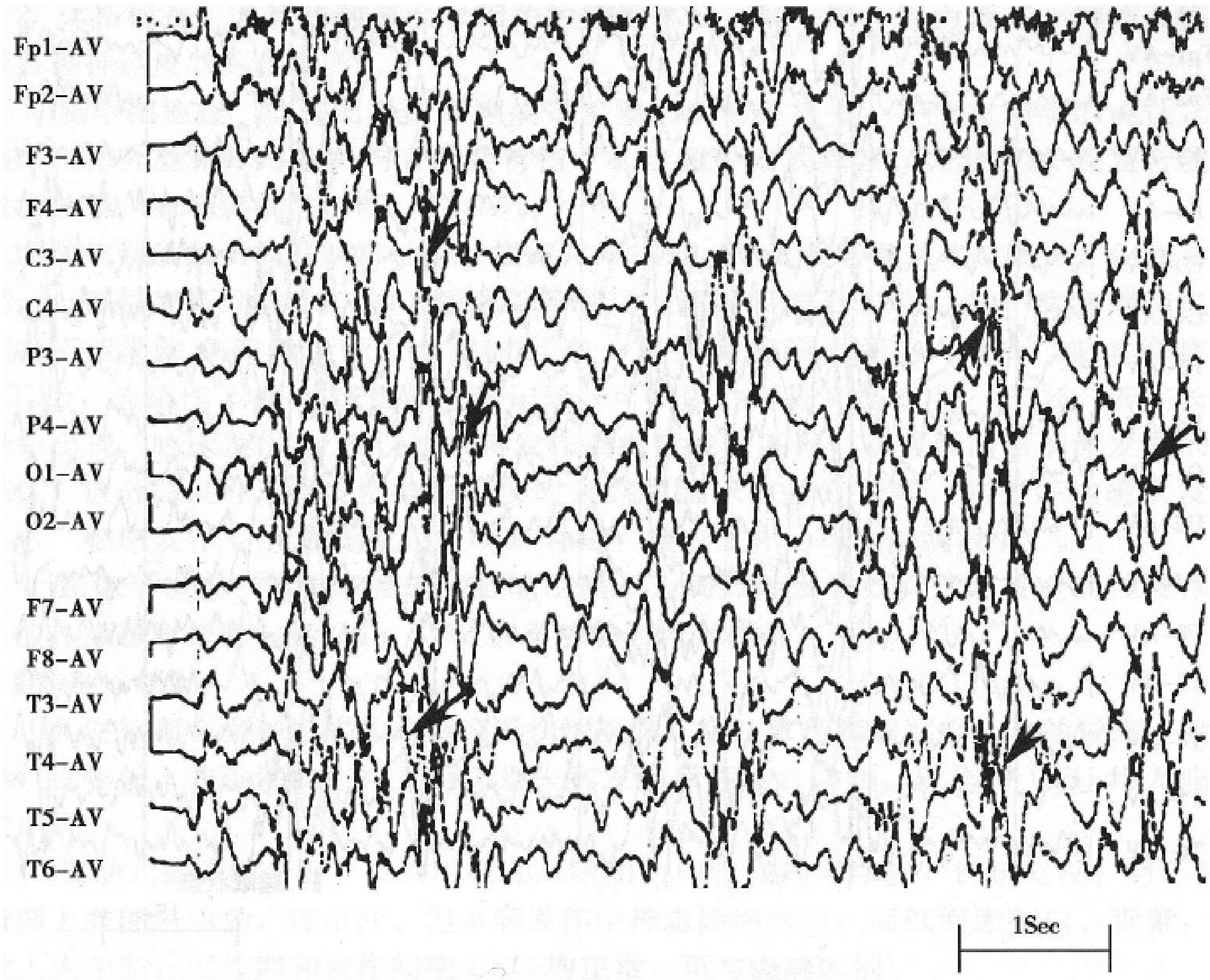

图16-3 高幅失律EEG

5 个月女婴，患婴儿痉挛。在不同步、不对称并有爆发抑制交替倾向的高波幅慢波背景中，

混有不规则、多灶性棘、尖与多棘慢波爆发（如箭头所示）

5．全面性癫痫伴热性惊厥附加症（generalized epilepsies with febrile seizures plus，GEFS）近年来，国际多数学者建议不再把热性惊厥（febrile seizure，FS）诊断为癫痫，但认定存在一种早期与一般热性惊厥有类似临床表现的儿童期常见癫痫综合征——GEFS+。然而，与一般热性惊厥不同，GEFS+患儿于6 岁后继续有频繁的、伴发热或无热的痫性发作，总发作次数超过一般热性惊厥，甚至可达数十次（2～100多次）。

GEFS+常有癫痫或热性惊厥家族史，一个家族中可有多种发作形式，多数仅表现为一般热性惊厥，但部分于6 岁后继续频繁的热性惊厥（强直-阵挛性发作）发作，称为热性惊厥附加症（FS+）。较少见的发作类型包括FS+伴失神发作、FS+伴肌阵挛发作和FS+伴失张力发作等。最近有报告，FS+伴肌阵挛站立不能性癫痫（MAE）和FS+伴婴儿严重肌阵挛癫痫（SMEI）者。除后两者外，GEFS+一般呈良性经过，智能运动发育正常，大多在25 岁前或儿童后期停止发作。

GEFS+的发生受遗传因素影响，一些人根据家系分析认为属常染色体显性遗传，故有人建议应称为常染色体显性遗传热性惊厥附加症（ADFS+）。由于不完全外显率导致了临床各种表型。但有学者主张为复杂性多基因遗传，以此解释GEFS+的表型异质性。近年来初步锁定本病的两个基因座分别在19q和2q上，预测近期还会有其他新的基因位点被发现。

【癫痫持续状态】

凡一次癫痫发作持续30分钟以上，或反复发作而间歇期意识不能恢复超过30分钟者，均称

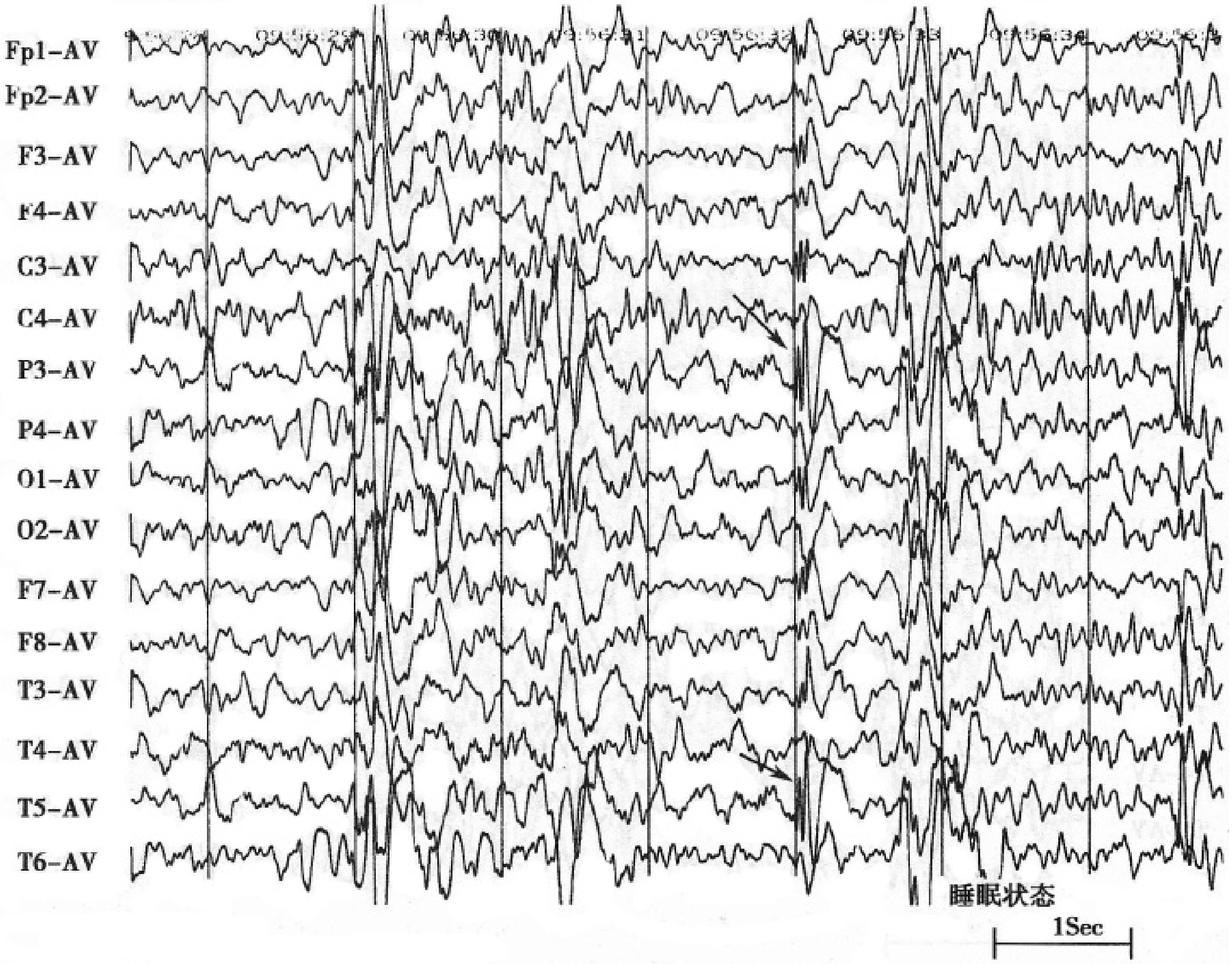

图16-4　Lennox-Gastaut综合征患儿EEG

2岁半女婴，半年来频繁多种形式癫痫混合发作，每日达十余次。EEG示

清醒异常慢波背景活动，左半球为主，频繁爆发2.5 Hz多棘一慢复合波

（如箭头所示）。小于3 Hz的慢棘-慢复合波为本病的EEG特征

者，均称为癫痫持续状态（status epilepicus，SE）。各种癫痫发作均可发生持续状态，但临床以强直-阵挛持续状态最常见。全身性发作的SE常伴有不同程度的意识、运动功能障碍，严重者还有脑水肿和颅内压增高的表现。即使积极抢救，病死率仍达3.6%。同时，智力低下、瘫痪和更严重癫痫发作等神经后遗症发生率高达9%～20%。

突然停药、药物中毒或高热等是癫痫持续状态的常见诱因。在非癫病患儿各种因素引起的脑部病变及热性惊厥有时也可发生持续状态。

【诊断】

确立癫痫诊断，应力求弄清以下三个问题：①其发作究竟是痫性发作，还是非癫痫性发作；②若系痫性发作，进一步弄清是什么发作类型，抑或属于某一特殊的癫痫综合征；③尽可能明确或推测癫痫发作的病因。一般按以下步骤搜集诊断依据：

1．相关病史

（1）发作史：癫痫患儿可无明显异常体征，详细而准确的发作史对诊断特别重要。癫痫发作应具有发作性和重复性这一基本特征。问清从先兆、发作起始到发作全过程，有无意识障碍，是局限性还是全面性发作，发作次数及持续时间，有无任何诱因以及与睡眠的关系等。

（2）提示与脑损伤相关的个人与过去史：如围生期异常、运动及智力发育落后、颅脑疾病与外伤史等。

（3）癫痫、精神病及遗传代谢病家族史。

2．体格检查 尤其与脑部疾患相关的阳性体征，如头围、智力低下、瘫痪、锥体束征或各种神经皮肤综合征等。

3．脑电图检查 脑电图是诊断癫痫最重要的实验室检查，不仅对癫痫的确认，而且对临床发作分型和转归分析均有重要价值。脑电图中出现棘波、尖波、棘-慢复合波等痫样发放波者，有利癫痫的诊断。

多数痫性波的发放是间歇性的，描记时间越长，异常图形发现率越高。若仅作常规清醒描记，阳性率不到40%，加上睡眠等各种诱发试验可增至70%，故一次常规脑电图报告正常不能排除癫痫的诊断。必要时可进一步作动态脑电图（AEEG）或录像脑电图（VEEG），连续作24 小时或更长时程记录，可使阳性率提高至80%～85%。若在长时程记录中出现“临床发作”，不仅能获得发作期痫性发放图形，还可弄清癫痫波发放的皮质起源区，区分原发性与继发性癫痫。实时观察“临床发作”录像，能更好地确认发作类型。若“临床发作”中无癫痫发作EEG伴随，癫痫发作的可能性就很小了。

4．影像学检查 当临床表现或脑电图提示为局灶性发作或局灶继发全面性发作的患儿，应作颅脑影像学包括CT，MRI甚至功能影像学检查。

【鉴别诊断】

小儿时期存在多种形式的非癫痫发作性疾病，应注意与癫痫鉴别。总的说来，除晕厥和屏气发作外，非痫性发作均无意识丧失和发作后症状，同时，发作中EEG均无痫性发作波出现。

1．婴幼儿擦腿综合征 发作时婴儿双腿用力内收或相互摩擦，神情专注，目不转睛，有时两上肢同时用劲，伴出汗。但本病发作中神志始终清楚，面红而无苍白、青紫，可随时被人为中断，发作期和发作间期EEG均正常，可与癫痫区别。

2．婴幼儿屏气 发作多发生于6～18 个月的婴儿。典型表现是当任何不愉快引起啼哭时，立即出现呼吸停止、青紫和全身肌张力低下，可有短暂意识障碍，一般不超过1 分钟，再现自主呼吸后随即恢复正常。与癫痫的区别在于本病明显以啼哭为诱因，意识丧失前先有呼吸暂停及青紫，EEG无异常，随年龄增大发作逐渐减少，5 岁后不再发作。

3．睡眠障碍 儿童期常见的睡眠障碍如夜惊、梦魇和梦游等。

夜惊常见于4～7 岁的儿童，属NREM期睡眠障碍。深睡中患儿突然坐起哭叫，表情惊恐，伴有瞳孔散大、出汗、呼吸急促等交感神经兴奋的表现，不易唤醒。数分钟后即再度安静入睡。次日对发作无记忆。根据其发作的自限性，EEG正常，可与癫痫区别。

梦魇以学龄前或学龄期儿童居多。常发生在后半夜和眼动（REM）睡眠期，患儿因噩梦引起惊恐状发作。与夜惊不同，梦魇中患儿易被唤醒，醒后对刚才梦境能清楚回忆，并因此心情惶恐无法立即再睡。根据其EEG正常和对发作中梦境的清楚回忆，可与癫痫鉴别。

梦游症也是NREM深睡期障碍。患儿从睡中突然起身，从事一些无目的的活动，如穿衣、搜寻、进食甚至开门窗等。发作中表情呆滞，自言自语地说一些听不懂的言词。醒后对发作无记忆。与精神运动性癫痫发作的区别在于，各次发作中梦游症的异常行为缺少一致性，发作中EEG正常，患儿很易被劝导回床上，也无发作后意识恍惚或乏力等表现。

4．偏头痛 本病是小儿时期反复头痛发作的主要病因。典型偏头痛主要表现为视觉先兆、偏侧性头痛、呕吐、腹痛和嗜睡等。儿童却以普通型偏头痛多见，无先兆，头痛部位也不固定。患儿常有偏头痛家族史，易伴恶心、呕吐等胃肠症状。实际上临床极少有单纯的头痛性或腹痛性癫痫患者，偏头痛绝不会合并惊厥性发作或自动症，EEG中也不会有局灶性痫性波发放。

5．抽动性疾患 抽动（Tics）是指突发性不规则肌群重复而间断的异常收缩（即所谓运动性抽动）或发声（即声音性抽动）。大多原因不明，精神因素可致发作加剧。主要有以下三种

形式：

（1）简单性抽动：仅涉及一组肌肉的短暂抽动，如眨眼、头部抽动或耸肩等，或突然爆发出含糊不清的单音，如吸气、清喉、吸吮、吹气甚至尖叫声。

（2）复杂性抽动：多组肌群的协同动作，如触摸、撞击、踢腿、跳跃等，缺乏目的性，成为不适时机的异常突发动作或模仿性姿势。语声性抽动表现为秽亵性语言、自身或模仿他人用词的重复性语言。

（3）Tourette综合征：是指多种运动性和语声性抽动症状持续一年以上的21 岁以下儿童及青少年患者，可能与遗传因素有关。发作程度时轻时重，形式常有变化。5～10 岁之间发病，男孩更多见。初期可能仅为简单性抽动，以后发展为复杂性抽动，病情波动，并反复迁延不愈甚至持续到成年。

抽动症需与癫痫肌阵挛发作鉴别。抽动症常为单侧肌群抽动，动作幅度较小，并可能伴发声性抽动。患者能有意识地暂时控制其发作，睡眠中消失，情绪紧张又导致发作加重。同时，EEG不会有癫痫样放电，也不会出现全部性慢波背景异常。

6．晕厥 是暂时性脑血流灌注不足引起的一过性意识障碍。年长儿多见，尤其是青春期。常发生在患儿持久站立，或从蹲位骤然起立，以及剧痛、劳累、阵发性心律不齐、家族性QT间期延长等情况。晕厥到来前，患儿常先有眼前发黑、头晕、苍白、出汗、无力等，继而出现短暂意识丧失，偶有肢体强直或抽动，清醒后对意识障碍不能回忆，并有疲乏感。与癫痫不同，晕厥患者意识丧失和倒地均逐渐发生，发作中少有躯体损伤，EEG正常，头竖直-平卧倾斜试验呈阳性反应。

7．癔症性发作 可与多种癫痫发作类型混淆。但癔症发作并无真正意识丧失，发作中慢慢倒下，不会有躯体受伤，无大小便失禁或舌咬伤。抽搐动作杂乱无规律，瞳孔无散大，深、浅反射存在，发作中面色正常，无神经系统阳性体征，无发作后嗜睡，常有夸张色彩。发作期与发作间期EEG正常，暗示治疗有效，与癫痫鉴别不难。

【治疗】

早期合理的治疗，能使90%以上患儿的癫痫发作得到完全或大部控制。多数患儿可望癫痫不再复发。家长、学校及社会应树立信心，批驳“癫痫是不治之症”这一错误观念。在帮助患儿接受正规治疗的同时，为其安排规律的生活、学习、作息，并注意其安全。

1．药物治疗 合理使用抗癫痫药物是当前治疗癫痫的主要手段。

（1）抗癫病药物使用原则：遵从以下原则是实现合理用药的基础：

1）早期治疗：反复的癫痫发作将导致新的脑损伤，早期规则治疗者成功率高。但对首次发作轻微，且无其他脑损伤等发作易感因素者，也可待第二次发作后再用药。

2）根据发作类型选药：见表16-4、表16-5。常用药物中，丙戊酸（VPA）与氯硝西泮（CZP）

表16-4 不同癫痫发作类型的药物选择*

发作类型	抗癫痫药物*	
	常用抗癫痫药物	抗癫痫新药
强直-阵挛性发作	VPA、CBZ、PB、PHT、CZP	TPM、LTG
肌阵挛、失张力、强直性或不典型失神发作	VPA、CZP、NZP	TPM、LTG
失神发作	ESM、VPA、CZP	LTG
局灶性发作、继发性强直-阵挛发作	CBZ、VPA、PHT、PB、CZP	TPM、OCBZ
婴儿痉挛	ACTH、NZP、CZP、VPA	VGB、TPM、LTG

*表中各种抗癫痫药物的英文缩写参见表16-5

是对大多数发作类型均有效的广谱抗癫痫药，在抗癫痫新药中，妥泰（托吡酯，TPM）、拉莫三嗪（LTG）等均有较广抗癫痫谱。

3）单药或联合用药的选择：近3/4的病例仅用一种抗癫痫药物即能控制其发作。但经2～3种单药合理治疗无效，尤其多种发作类型的患儿，应考虑2～3 种作用机制互补的药物联合治疗。

4）用药剂量个体化：从小剂量开始，依据疗效、患者依从性和血药浓度逐渐增加并调整剂量，达最大疗效或最大血药浓度时为止。一般经5 个半衰期的服药时间可达该药的稳态血浓度。

5）长期规则服药以保证稳定血药浓度：一般应在服药后完全不发作2～4 年，又经3～6 月逐渐减量过程才能停药。不同发作类型的疗程也不同，失神发作在停止发作2 年，复杂性局灶性发作、LGS等则要停止发作后4 年才考虑停药。婴幼儿期发病、不规则服药、EEG持续异常以及同时合并大脑功能障碍者，停药后复发率高。青春期来临易致癫痫复发或加重，故要避免在这个年龄期减量与停药。

6）定期复查：密切观察疗效与药物不良反应。除争取持续无临床发作外，至少每年应复查1 次常规EEG。针对所用药物主要副作用，定期监测血常规、血小板计数或肝、肾功能。在用药初期、联合用药、病情反复或更换新药时，均应监测血药浓度。

（2）传统抗癫痫药物与抗癫痫新药 见表16-5。

表15-5 传统抗癫痫药物与抗癫痫新药

	药物	剂量（mg/kg/d）	有效血浓度（μg/ml）	消除半衰期	主要不良反应
传统抗癫痫药物	丙戊酸（VPA）	15～40	50～110	6～16 小时	食欲和体重增加、肝功损害等
	卡马西平（CBZ）	15～30	4～12	8～20 小时	头晕、皮疹、白细胞减少、肝功能损害等
	苯妥英钠（PHT）	3～8	10～20	22 小时	齿龈增生、共济失调、皮疹、白细胞减少
	苯巴比妥（PB）	3～5	20～40	4 天	多动、注意力不集中、皮疹
	乙琥胺（ESX）	20～40	40～120	55 小时	胃肠道反应、头痛、白细胞减少
	氯硝基安定（CZP）	0.01～0.2	20～80	20～30 小时	嗜睡、共济失调、流涎、全身松软
	硝基安定（N2P）	0.2～1	-	8～36 小时	同 CZP
	促肾上腺皮质（ACTH）	25～40 单位（4～6 周）	-	-	肾上腺皮质功能亢进
抗癫痫新药	妥泰（托吡酯）（TPM）	2～5	-	15 小时	嗜睡、思维慢、食欲减退、体重减低、少汗
	拉莫三嗪（LTG）	5～15（与 VPA 合用时为 1 mg/kg•d～5 mg/kg•d）	1.5～3.0	20～30 小时	皮疹、嗜睡、头痛、共济失调、胃肠道反应
	氨乙烯酸（VGB）	40～80	-	5～6 小时	嗜睡、精神压抑、视野缺失
	奥卡西平（OCBZ）	10～40	-	8～6 小时	嗜睡、头痛、皮疹、共济失调

2．手术治疗 约有20%～25%的患儿对各种抗癫痫药物治疗无效而被称为难治性癫痫，对其中有明确局灶性癫痫发作起源的难治性癫痫，可考虑手术治疗。近年来对儿童难治性癫痫的手术治疗有增多趋势，其中2/3因颞叶病灶致癫痫难治而行病灶切除，术后约67.9%发作完全停止，24%有不同程度改善。其他手术方式包括非颞叶皮质区病灶切除术、病变半球切除术，以及不切除癫痫灶的替代手术（如胼胝体切断术、软脑膜下皮层横切术）。

做好术前评估是决定术后疗效的关键，术前评估的主要目的在于：

（1）确认手术中要切除的癫痫放电灶。主要借助EEG、AEEG、VEEG、颅内电极脑电图、影像学和功能影像学（PET, SPECT等）等检查技术。

（2）确认即将进行的手术能够回避对皮质重要功能区的损伤，以保证术后语言、肢体运动等重要功能的完好。

手术禁忌证包括：伴有进行性大脑疾病、严重精神智能障碍（IQ<70，或活动性精神病），或术后会导致更严重脑功能障碍的难治性癫痫患者。

3．癫痫持续状态的急救处理

（1）尽快控制SE发作：立即静脉注射有效而足量的抗癫痫药物，通常首选地西泮（安定）。大多在1～2 分钟内止惊。每次剂量0.3～0.5 mg/kg，一次总量不超过l0 mg。原液可不稀释直接静脉推注，速度不超过1～2 mg/min(新生儿0. 2 mg/min)。必要时1/2～1 小时后可重复1 次，24小时内可用2～4 次。静脉注射困难时用同样剂量经直肠注入比肌注见效快，5～10 分钟可望止惊。静脉推注中要密切观察有无呼吸抑制。

与地西泮同类的有效药物还有劳拉西泮、氯硝西泮、咪哒唑仑等。此外，苯妥英钠、苯巴比妥都属于抢救SE的第一线药物，其作用各有特色，单独或联合应用。

（2）支持治疗：主要包括：①生命体征监测，重点注意呼吸循环衰竭或脑疝体征；②保持呼吸道通畅，吸氧，必要时人工机械通气；③监测与矫治血气、血糖、血渗透压及血电解质异常；④防治颅内压增高。

第三节 惊　厥

惊厥（convulsion）是痫性发作的常见形式，以强直或阵挛等骨骼肌运动性发作为主要表现，常伴意识障碍。惊厥及其他形式的痫性发作也可在小儿许多急性疾病过程中出现，它们因急性原发病而出现，又随原发病结束而消失，因而此类惊厥不能诊断为癫痫。只有慢性的反复痫性发作才能诊断为癫痫。

小儿时期急性疾病中惊厥发作有以下特征：

1．惊厥是儿科临床常见急症。儿童期发生率约为4%～6%，较成人高10～15 倍。年龄愈小发生率愈高。

2．易有频繁或严重发作，甚至惊厥持续状态。

3．新生儿及婴儿常有不典型惊厥发作，如表现为面部、肢体局灶或多灶性抽动、局部或全身性肌阵挛，或表现为突发瞪眼、咀嚼、流涎、呼吸暂停、青紫等不显性发作（subtle seizures）。

4．引起惊厥的病因众多复杂。

【病因分类与特点】

1．感染性病因

（1）颅内感染：如由细菌、病毒、寄生虫、真菌引起的脑膜炎或脑炎。常表现为反复而严重的惊厥发作，大多出现在疾病初期或极期。伴有不同程度的意识障碍和颅内压增高表现。脑脊液检查对诊断和鉴别诊断有较大帮助。

（2）颅外感染：非颅内感染性疾病引起的惊厥发作。

1）热性惊厥：是儿科最常见的急性惊厥，见本节后文专述。

2）感染中毒性脑病：大多并发于败血症、重症肺炎、细菌性痢疾、百日咳等严重细菌性感染疾病，与感染和细菌毒素导致急性脑水肿有关。通常于原发病极期出现反复惊厥、意识障碍与颅内压增高症状。检查脑脊液除发现压力增高外，常规、生化均正常。

2．非感染性病因

（1）颅内疾病：

1）颅脑损伤与出血：如产伤、颅脑外伤和脑血管畸形等各种原因引起的颅内出血。伤后立即起病，反复惊厥伴意识障碍和颅内压增高，颅脑CT对诊断有重要价值。

2）先天发育畸形：如颅脑发育异常、脑积水、神经皮肤综合征等。大多表现为反复发作，常伴有智力和运动发育落后。

3）颅内占位性病变：如幕上、大脑半球的肿瘤、囊肿或血肿等。除反复惊厥发作外，伴颅内压增高和定位体征，病情进行性加重，头颅影像学检查对诊断起决定作用。

（2）颅外（全身性）疾病：

1）缺氧缺血性脑病：如分娩或生后窒息、溺水、心肺严重疾病等。窒息后立即起病，反复惊厥伴意识障碍和颅内压增高，头颅影像学检查对诊断起重要作用。

2）代谢性疾病：包括：①水、电解质紊乱：重度脱水、水中毒、低血钙、低血镁、低血钠、高血钠和低血糖症均可引起惊厥。患儿均有相应临床表现及其基础病因。血渗透压、电解质和血糖测定有助诊断，病因治疗能迅速控制惊厥发作。②肝、肾衰竭和Reye综合征：顽固惊厥伴严重肝、肾功能异常及电解质紊乱。③遗传代谢性疾病：常见如苯丙酮尿症、半乳糖血症等，表现为进行性加重的惊厥或癫痫发作，有异常代谢相关的特异体征，血、尿中代谢不全产物含量增高。④中毒：如杀鼠药、农药和中枢神经兴奋药中毒。大多有顽固惊厥发作伴意识障碍及肝、肾功能损伤。

【热性惊厥】

与前面介绍的GEFS+不同，热性惊厥的发作均与发热性疾病中体温骤然升高有关。由于有明显的诱发原因，国际抗癫痫联盟新近不主张把热性惊厥诊断为癫痫。

热性惊厥是小儿时期最常见的惊厥性疾病，儿童期患病率3%～4%，首次发作年龄多于生后6 个月至3 岁间，平均18～22 个月。男孩稍多于女孩。绝大多数5 岁后不再发作。患儿常有热性惊厥家族史，对若干大的家系连锁分析提示常染色体显性遗传伴不同外显率的可能性，基因位点在19p和8q13-21。

1．临床表现 热性惊厥发生在热性疾病初期体温骤然升高（大多39℃）时，70%以上与上呼吸道感染有关，其他伴发于出疹性疾病、中耳炎、下呼吸道感染等疾病，但绝不包括颅内感染和各种颅脑病变引起的急性惊厥。

单纯性热性惊厥（又称典型热性惊厥） 多数呈全身性强直-阵挛性发作，少数也可有其他发作形式，如肌阵挛、失神等。持续数秒至10 分钟，可伴有发作后短暂嗜睡。发作后患儿除原发疾病表现外，一切恢复如常，不留任何神经系统体征。在一次发热疾病过程中，大多只有一次，个别有两次发作。约50%的患儿会在今后发热时再次或多次热性惊厥发作，大多数（3/4）的再次发作发生在首次发作后1年内。

少数热性惊厥呈不典型经过，称复杂性热性惊厥。其主要特征包括：①一次惊厥发作持续15 分钟以上；②24 小时内反复发作≥2 次；③局灶性发作；④反复频繁的发作，累计发作总数5 次以上。单纯性热性惊厥与复杂性热性惊厥的主要区别见表16-6。

若干因素使热性惊厥患儿发生癫痫的危险性增加，称为癫痫危险因素，主要包括：①复杂性热性惊厥；②直系亲属中癫痫病史；③首次热性惊厥前已有神经系统发育延迟或异常体征。具有其中2～3 个危险因素者，7 岁时癫痫发生率平均达9%以上，而无危险因素的热性惊厥不到10%。EEG在癫痫危险性的预测上价值尚无定论，故对单纯性热性惊厥，一般无需作EEG检查。但对复杂性热性惊厥患儿，若EEG中新出现痫性波发放，则可能提示癫痫发生的危险性。

表16-6 单纯性与复杂性热性惊厥的鉴别要点

	单纯性热性惊厥	复杂性热性惊厥
发病率	在热性惊厥中约占 80%	在热性惊厥中约占 20%
惊厥发作形式	全身性发作	局限性或不对称
惊厥持续时间	短暂发作，大多数在 5～10 分钟内	长时间发作，≥15 分钟
惊厥发作次数	一次热程中仅有 1～2 次发作	24 小时内反复多次发作
热性惊厥复发总次数	≤4 次	≥5 次

2．热性惊厥的防治 对单纯性热性惊厥，仅针对原发病处理，包括用退热药物和其他物理降温措施即可。但对有复发倾向者，可于发热病开始即使用地西泮（安定）1 mg/(kg • d)，分3次口服，连服2～3 天，或直到本次原发病体温回复正常为止。对复杂性热性惊厥或总发作次数已达5 次以上者，若以地西泮临时口服未能阻止新的发作，可长期口服丙戊酸或苯巴比妥，剂量见表16-5，疗程1～2 年，个别需适当延长。其他传统抗癫痫药对热性惊厥发作的预防作用较差。

（孙若鹏）

第四节 化脓性脑膜炎

化脓性脑膜炎（purulent meningitis， 以下简称化脑）是小儿、尤其婴幼儿时期常见的中枢神经系统感染性疾病。临床以急性发热、惊厥、意识障碍、颅内压增高和脑膜刺激征、以及脑脊液脓性改变为特征。随着脑膜炎球菌及流感嗜血杆菌疫苗的接种和诊断、治疗水平不断发展，本病发病率和病死率明显下降。约1/3幸存者遗留各种神经系统后遗症，6 个月以下幼婴患本病预后更为严重。

【致病菌和入侵途径】

许多化脓菌都能引起本病。但2/3以上患儿是由脑膜炎球菌、肺炎链球菌和流感嗜血杆菌三种细菌引起。2 个月以下幼婴和新生儿以及原发或继发性免疫缺陷病者，易发生肠道革兰阴性杆菌和金黄色葡萄球菌脑膜炎，前者以大肠杆菌最多见，其次如变形杆菌、绿脓杆菌或产气杆菌等。然而与国外不同，我国很少发生B组β溶血性链球菌颅内感染。由脑膜炎球菌引起的脑膜炎呈流行性。

致病菌可通过多种途径侵入脑膜：

1．最常见的途径是通过血流，即菌血症抵达脑膜微血管。当小儿免疫防御功能降低时，细菌通过血脑屏障到达脑膜。致病菌大多由上呼吸道入侵血流，新生儿的皮肤、胃肠道黏膜或脐部也常是感染的侵入门户。

2．邻近组织器官感染，如中耳炎、乳突炎等扩散波及脑膜。

3．与颅腔存在直接通道，如颅骨骨折、皮肤窦道或脑脊髓膜膨出，细菌可因此直接进入蛛网膜下腔。

【病理】

在细菌毒素和多种炎症相关细胞因子作用下，形成以软脑膜、蛛网膜和表层脑组织为主的炎症反应，表现为广泛性血管充血、大量中性粒细胞浸润和纤维蛋白渗出，伴有弥漫性血管源性和细胞毒性脑水肿。在早期或轻型病例，炎性渗出物主要在大脑顶部表面，逐渐蔓延至大脑基底部和脊髓表面。严重者可有血管壁坏死和灶性出血，或发生闭塞性小血管炎而致灶性脑梗

死。

【临床表现】

90％的化脑为5 岁以下儿童，1 岁以下是患病高峰年龄，流感嗜血杆菌引起的化脑多集中在3 个月～3 岁儿童。一年四季均有化脑发生，但肺炎链球菌冬、春季多见，而脑膜炎球菌和流感嗜血杆菌引起的化脑分别以春、秋季发病多。大多急性起病。部分患儿病前有数日上呼吸道或胃肠道感染病史。

典型临床表现可简单概括为三个方面：

1．感染中毒及急性脑功能障碍症状 包括发热、烦躁不安和进行性加重的意识障碍。随病情加重，患儿逐渐从精神萎靡、嗜睡、昏睡、昏迷到深度昏迷。30％以上患儿有反复的全身或局限性惊厥发作。脑膜炎双球菌感染易有瘀点、瘀瘢和休克。

2．颅内压增高表现 包括头痛、呕吐，婴儿则有前囟饱满与张力增高、头围增大等。合并脑疝时，则有呼吸不规则、突然意识障碍加重及瞳孔不等大等体征。

3．脑膜刺激征 以颈项强直最常见，其他如Kernig征和Brudzinski征阳性。

年龄小于3 个月的幼婴和新生儿化脑表现多不典型，主要差异在：①体温可高可低或不发热，甚至体温不升；②颅内压增高表现可不明显。幼婴不会诉头痛，可能仅有吐奶、尖叫或颅缝开裂；③惊厥可不典型，如仅见面部、肢体局灶或多灶性抽动、局部或全身性肌阵挛、或呈眨眼、呼吸不规则、屏气等各种不显性发作；④脑膜刺激征不明显，与婴儿肌肉不发达，肌力弱和反应低下有关。

【实验室检查】

1．脑脊液检查 脑脊液检查是确诊本病的重要依据，参见表16-2。典型病例表现为压力增高，外观混浊似米汤样。白细胞总数显著增多，≥1000×10^6/L，但有20％的病例可能在250×10^6/L以下，分类中性粒细胞为主。糖含量常有明显降低，蛋白显著增高。

确认致病菌对明确诊断和指导治疗均有重要意义，涂片革兰染色检查致病菌简便易行，检出阳性率甚至较细菌培养高。细菌培养阳性者应送药物敏感试验。以乳胶颗粒凝集法为基础的多种免疫学方法可检测出脑脊液中致病菌的特异性抗原，对涂片和培养未能检测到致病菌的患者诊断有参考价值。

2．其他

（1）血培养：对所有疑似化脑的病例均应做血培养，以帮助寻找致病菌。

（2）皮肤瘀点、瘀斑找菌：是发现脑膜炎双球菌重要而简便的方法。

（3）外周血象：白细胞总数大多明显增高，中性粒细胞为主。但在感染严重或不规则治疗者，又可能出现白细胞总数的减少。

【并发症和后遗症】

1．硬脑膜下积液 约30％～60％的化脑并发硬脑膜下积液，若加上无症状者，其发生率可高达80％。本症主要发生在1 岁以下婴儿。凡经化脑有效治疗48～72 小时后脑脊液有好转，但体温不退或体温下降后再升高；或一般症状好转后又出现意识障碍、惊厥、前囟隆起或颅压增高等症状，首先应怀疑本病的可能性。头颅透光检查和CT扫描可协助诊断，但最后确诊仍有赖硬膜下穿刺放出积液，同时也达到治疗目的。积液应送常规和细菌学检查。正常婴儿硬脑膜下积液量不超过2 m1，蛋白定量小于0.4 g/L。

发生硬脑膜下积液的机制尚不完全明确，推测原因：①脑膜炎症时，血管通透性增加，血浆成分渗出，进入硬脑膜下腔；②脑膜及脑的表层小静脉，尤其穿过硬膜下腔的桥静脉发生炎性栓塞，导致渗出和出血，局部渗透压增高，水分进入硬膜下腔形成硬膜下积液。

2．脑室管膜炎 主要发生在治疗被延误的婴儿。患儿在有效抗生素治疗下发热不退，惊厥，意识障碍不改善，进行性加重的颈项强直甚至角弓反张，脑脊液始终无法正常化，以及CT见脑室扩大时，需考虑本症，确诊依赖侧脑室穿刺，取脑室内脑脊液显示异常。治疗大多困难，病死率和致残率高。

3．抗利尿激素异常分泌综合征 炎症刺激神经垂体致抗利尿激素过量分泌，引起低钠血症和血浆低渗透压，可能加剧脑水肿，致惊厥和意识障碍加重，或直接因低钠血症引起惊厥发作。

4．脑积水 炎症渗出物粘连堵塞脑室内脑脊液流出通道，如导水管、第Ⅳ脑室侧孔或正中孔等狭窄处，引起非交通性脑积水；也可因炎症破坏蛛网膜颗粒，或颅内静脉窦栓塞致脑脊液重吸收障碍，造成交通性脑积水。发生脑积水后，患儿出现烦躁不安、嗜睡、呕吐、惊厥发作，头颅进行性增大，骨缝分离，前囟扩大饱满、头颅破壶音和头皮静脉扩张。至疾病晚期，持续的颅内高压使大脑皮层退性萎缩，患儿出现进行性智力减退和其他神经功能倒退。

5．各种神经功能障碍 由于炎症波及耳蜗迷路，10％～30％的患儿并发神经性耳聋。其他如智力低下、癫痫、视力障碍和行为异常等。

【诊断】

早期诊断是保证患儿获得早期治疗的前提。凡急性发热起病，并伴有反复惊厥、意识障碍或颅内压增高表现的婴幼儿，均应注意本病可能性，应进一步依靠脑脊液检测确立诊断。然而，对有明显颅压增高者，应先适当降低颅内压后再行腰椎穿刺，以防腰椎穿刺后发生脑疝。

婴幼儿和不规则治疗者临床表现常不典型，后者的脑脊液改变也可不明显，病原学检查往往阴性，诊断时应仔细询问病史和详细进行体格检查，结合脑脊液中病原的特异性免疫学检查及治疗后病情转变，综合分析后确立诊断。

【鉴别诊断】

除化脓菌外，结核杆菌、病毒、真菌等皆可引起脑膜炎，并出现与化脑相似的临床表现而需注意鉴别。脑脊液检查，尤其病原学检查是鉴别诊断的关键，参见表16-2。

1．结核性脑膜炎 需与不规则治疗的化脑鉴别。结核性脑膜炎呈亚急性起病，不规则发热1～2 周才出现脑膜刺激征、惊厥或意识障碍等表现，或于昏迷前先有脑神经或肢体麻痹。具有结核接触史、PPD阳转或肺部等其他部位结核病灶者支持结核诊断。脑脊液外观呈毛玻璃样，白细胞数多$<500\times10^6$/L，分类以单核细胞为主，薄膜涂片抗酸染色和结核菌培养可帮助诊断确立。

2．病毒性脑膜炎 临床表现与化脑相似，感染中毒及神经系统症状均比化脑轻，病程自限，大多不超过2 周。脑脊液清亮，白细胞数0 至数百$\times10^6$/L，分类以淋巴细胞为主，糖含量正常。脑脊液中特异性抗体和病毒分离有助诊断。

3．隐球菌性脑膜炎 临床和脑脊液改变与结核性脑膜炎相似，但病情进展可能更缓慢，头痛等颅压增高表现更持续和严重。诊断有赖脑脊液涂片墨汁染色和培养找到致病真菌。

【治疗】

1．抗生素治疗

（1）用药原则：化脑预后严重，应力求用药24 小时内杀灭脑脊液中致病菌，故应选择对病原菌敏感，且能较高浓度透过血脑屏障的药物。急性期要静脉用药，做到用药早、剂量足和疗程够。

（2）病原菌明确前的抗生素选择：包括诊断初步确立但致病菌尚未明确，或院外不规则治疗者。应选用对肺炎链球菌、脑膜炎球菌和流感嗜血杆菌三种常见致病菌皆有效的抗生素。目前主要选择能快速在患者脑脊液中达到有效灭菌浓度的第三代头孢菌素，包括头孢噻肟（ce-

fotaxime）200 mg/（kg・d），或头孢曲松（ceftriaxone）l00 mg/（kg・d），疗效不理想时可联合使用万古霉素（vancomycin）40 mg/（kg・d）。对β内酰胺类药物过敏的患儿，可改用氯霉素l00 mg/（kg・d）。

（3）病原菌明确后的抗生素选择：

1）肺炎链球菌：由于当前半数以上的肺炎球菌对青霉素耐药，故应继续按上述病原菌未明确方案选药。仅当药敏试验提示致病菌对青霉素敏感，可改用青霉素20 万～40 万 U/（kg・d）。

2）脑膜炎球菌：与肺炎链球菌不同，目前该菌大多数对青霉素依然敏感，故首先选用，剂量同前。少数耐青霉素者需选用上述第三代头孢菌素。

3）流感嗜血杆菌：对敏感菌株可换用氨苄青霉素（ampicillin）200 mg/（kg・d）。耐药者使用上述第三代头孢菌素或氯霉素。

4）其他：致病菌为金黄色葡萄球菌者应参照药敏试验选用乙氧奈青霉素（nefcillin）、万古霉素或利福平等。革兰阴性杆菌者除考虑上述第三代头孢菌素外，可加用氨苄西林或氯霉素。

（4）抗生素疗程：对肺炎链球菌和流感嗜血杆菌脑膜炎，其抗生素疗程应是静脉滴注有效抗生素10～14 天，脑膜炎球菌者7 天，金黄色葡萄球菌和革兰阴性杆菌脑膜炎应21 天以上。若有并发症，还应适当延长。

2．肾上腺皮质激素的应用 细菌释放大量内毒素，可能促进细胞因子介导的炎症反应，加重脑水肿和中性粒细胞浸润，使病情加重。抗生素迅速杀死致病菌后，内毒素释放尤为严重，此时使用肾上腺皮质激素不仅可抑制多种炎症因子的产生，还可降低血管通透性，减轻脑水肿和颅内高压。常用地塞米松0.6 mg/（kg・d），分4 次静脉注射。一般连续用2～3 天，过长使用并无益处。

3．并发症的治疗

（1）硬膜下积液：少量积液无需处理。如积液量较大引起颅压增高症状时，应作硬膜下穿刺放出积液，放液量每次、每侧不超过15 ml。有的患儿需反复多次穿刺，大多数患儿积液逐渐减少而治愈。个别迁延不愈者，需外科手术引流。

（2）脑室管膜炎：进行侧脑室穿刺引流，以缓解症状。同时，针对病原菌并结合用药安全性，选择适宜抗生素脑室内注入。

（3）脑积水：主要依赖手术治疗，包括正中孔粘连松解、导水管扩张和脑脊液分流术。

4．对症和支持治疗

（1）急性期严密监测生命体征，定期观察患儿意识、瞳孔和呼吸节律改变，并及时处理颅内高压，预防脑疝发生。参见“病毒性脑炎”节。

（2）及时控制惊厥发作，并防止再发。参见“癫痫与惊厥”一节。

（3）监测并维持体内水、电解质、血浆渗透压和酸碱平衡。对有抗利尿激素异常分泌综合征表现者，积极控制脑膜炎同时，适当限制液体入量，对低钠症状严重者酌情补充钠盐。

第五节 病毒性脑炎

病毒性脑炎（viral encephalitis）是指多种病毒引起的颅内急性炎症。由于病原体致病性能和宿主反应过程的差异，形成不同类型疾病。若病变主要累及脑膜，临床表现为病毒性脑膜炎；

若病变主要影响大脑实质，则以病毒性脑炎为临床特征。由于解剖上两者相邻近，若脑膜和脑实质同时受累，此时称为病毒性脑膜脑炎。大多数患者病程呈自限性。

【病因】

临床工作中，目前仅能在1/3～1/4 的中枢神经病毒感染病例中确定其致病病毒，其中，80％为肠道病毒，其次为虫媒病毒、腺病毒、单纯疱疹病毒、腮腺炎病毒和其他病毒等。虽然目前在多数患者尚难确定其病原体，但从其临床和实验室资料，均能支持急性颅内病毒感染的可诊断。

【病理】

脑膜和（或）脑实质广泛性充血、水肿，伴淋巴细胞和浆细胞浸润。可见炎症细胞在小血管周围呈袖套样分布，血管周围组织神经细胞变性、坏死和髓鞘崩解。病理改变大多弥漫分布，但也可在某些脑叶突出，呈相对局限倾向。单纯疱疹病毒常引起颞叶为主的脑部病变。

有的脑炎患者，见到明显脱髓鞘病理表现，但相关神经元和轴突却相对完好。此种病理特征，代表病毒感染激发的机体免疫应答，提示“感染后”或“过敏性”脑炎的病理学特点。

【发病机理】

病毒经肠道（如肠道病毒）或呼吸道（如腺病毒和出疹性疾病）进入淋巴系统繁殖，然后经血流（虫媒病毒直接进入血流）感染颅外某些脏器，此时患者可有发热等全身症状。若病毒在定居脏器内进一步繁殖，即可能入侵脑或脑膜组织，出现中枢神经症状。因此，颅内急性病毒感染的病理改变主要是大量病毒对脑组织的直接入侵和破坏，然而，若宿主对病毒抗原发生强烈免疫反应，将进一步导致脱髓鞘、血管与血管周围脑组织损害。

【临床表现】

病情轻重差异很大，取决于脑膜或脑实质受累的相对程度。一般说来，病毒性脑炎的临床经过较脑膜炎严重，重症脑炎更易发生急性期死亡或后遗症。

1．病毒性脑膜炎 急性起病，或先有上呼吸道感染或前驱传染性疾病。主要表现为发热、恶心、呕吐、软弱、嗜睡。年长儿会诉头痛，婴儿则烦躁不安，易激惹。一般很少有严重意识障碍和惊厥。可有颈项强直等脑膜刺激征。但无局限性神经系统体征。病程大多在1～2 周内。

2．病毒性脑炎 起病急，但其临床表现因脑实质部位的病理改变、范围和严重程度而有不同。

（1）大多数患儿因弥漫性大脑病变而主要表现为发热、反复惊厥发作、不同程度意识障碍和颅压增高症状。惊厥大多呈全身性，但也可有局灶性发作，严重者呈惊厥持续状态。患儿可有嗜睡、昏睡、昏迷、深度昏迷，甚至去皮质状态等不同程度的意识改变。若出现呼吸节律不规则或瞳孔不等大，要考虑颅内高压并发脑疝可能性。部分患儿尚伴偏瘫或肢体瘫痪表现。

（2）有的患儿病变主要累及额叶皮层运动区，临床则以反复惊厥发作为主要表现，伴或不伴发热。多数为全部性或局灶性强直-阵挛或阵挛性发作，少数表现为肌阵挛或强直性发作。皆可出现癫痫持续状态。

（3）若脑部病变主要累及额叶底部、颞叶边缘系统，患者则主要表现为精神情绪异常，如躁狂、幻觉、失语、以及定向力、计算力与记忆力障碍等。伴发热或无热。多种病毒可引起此类表现，但由单纯疱疹病毒引起者最严重，该病毒脑炎的神经细胞内易见含病毒抗原颗粒的包涵体，有时被称为急性包涵体脑炎，常合并惊厥与昏迷，病死率高。

其他还有以偏瘫、单瘫、四肢瘫或各种不自主运动为主要表现者。不少患者可能同时兼有上述多种类型表现。当病变累及锥体束时出现阳性病理征。

本病病程大多2～3 周。多数完全恢复，但少数遗留癫痫、肢体瘫痪、智力倒退等后遗症。

【辅助检查】

1．脑电图 以弥漫性或局限性异常慢波背景活动为特征，少数伴有棘波、棘-慢综合波。慢波背景活动只能提示异常脑功能，不能证实病毒感染性质。某些患者脑电图也可正常。

2．脑脊液检查 外观清亮，压力正常或增加。白细胞数正常或轻度增多，分类计数以淋巴细胞为主，蛋白质大多正常或轻度增高，糖含量正常。涂片和培养无细菌发现。

3．病毒学检查 部分患儿脑脊液病毒培养及特异性抗体测试阳性。恢复期血清特异性抗体滴度高于急性期4 倍以上有诊断价值。

【诊断和鉴别诊断】

大多数病毒性脑炎的诊断有赖于排除颅内其他非病毒性感染、Reye综合征等急性脑部疾病后确立。少数患者若明确地并发于某种病毒性传染病，或脑脊液检查证实特异性病毒抗体阳性者，可支持颅内病毒性感染的诊断。

1．颅内其他病原感染 主要根据脑脊液外观、常规、生化和病原学检查，与化脓性、结核性、隐球菌脑膜炎鉴别。此外，合并硬膜下积液者支持婴儿化脓性脑膜炎。发现颅外结核病灶和皮肤PPD阳性有助于结核性脑膜炎诊断。

2．Reye综合征 因急性脑病表现和脑脊液无明显异常使两病易相混淆，但依据Reye综合征无黄疸而肝功能明显异常、起病后3～5 天病情不再进展、有的患者血糖降低等特点，可与病毒性脑炎鉴别。

【治疗】

本病缺乏特异性治疗。但由于病程自限性，急性期正确的支持与对症治疗，是保证病情顺利恢复、降低病死率和致残率的关键。主要治疗原则包括：

1．维持水、电解质平衡与合理营养供给 对营养状况不良者给予静脉营养剂或白蛋白。

2．控制脑水肿和颅内高压 可酌情采用以下方法：①严格限制液体入量；②过度通气，将$PaCO_2$控制于20～25 kPa；③静脉注射脱水剂，如甘露醇等。

3．控制惊厥发作 可给予止惊剂如地西泮、苯妥因钠等。如止惊无效，可在控制性机械通气下给予肌肉松弛剂。

4．抗病毒药物 无环鸟苷（aciclovir)，每次5～10 mg/kg，每8 小时1 次；或其衍生物丙氧鸟苷（ganciclovir)，每次5 mg/kg，每12 小时1 次。两种药物均需连用10～14 天，静脉滴注给药。抗病毒药物对单纯疱疹病毒作用最强，对水痘-带状疱疹病毒、巨细胞病毒，EB病毒也有抑制作用。

【附】Reye综合征

1963 年由Reye等首先报告而命名为Reye 综合征（Reye syndrome）。因出现急性弥漫性脑水肿和肝脏为主的内脏脂肪变性病理特征，曾被称为脑病合并内脏脂肪变性。

本病基本病理生理特点为广泛急性线粒体功能障碍。引起此种障碍的原因尚不完全清楚，90%与上呼吸道病毒感染有关。国外报道B型流感和水痘流行期间可见本病发病增多，并有作者强调，在流感和水痘患儿使用水杨酸药物有诱发本病的高度危险性。

多数患儿年龄在4～12 岁间，平素健康，大多有病毒性上呼吸道感染等前驱疾病。往往在前驱疾病恢复过程中突然出现频繁呕吐，其后病情迅速加重，出现反复惊厥和进行性意识障碍，

常在数小时内进入昏睡、昏迷至深度昏迷，严重者呈去大脑强直。患者多有颅内压增高，若出现呼吸节律不规则或两侧瞳孔不等大，要分别考虑并发枕骨大孔疝或天幕裂孔疝，若抢救不及时，很快死亡。一般无神经系统定位体征，肝脏可有轻、中度肿大，但也可不大，虽然肝功显著异常但临床无明显黄疸表现。

肝功异常包括转氨酶增高、高氨血症、高游离脂酸血症及凝血功能障碍。婴幼儿易有低血糖。脑脊液检查除压力增高外无其他异常。周围血白细胞反应性增高，分类计数以中性粒细胞占优势。

病程有自限性，大多在起病后3～5面天不再进展，并在1 周内恢复。重症患儿易在病初1～2 天内死亡。幸存者可能遗留各种神经后遗症，长时间持续昏迷者后遗症发生率高。

本病因有急性脑病的各种临床表现，需与化脓性、结核性或病毒性脑炎区别。又根据本病肝功虽异常，需与重症肝炎、肝性脑病相鉴别。

治疗包括：积极降低颅内压；纠正代谢紊乱；控制惊厥发作；抢救中应避免使用水杨酸或酚噻嗪类药物。

第六节 脑性瘫痪

脑性瘫痪（cerebral palsy，简称脑瘫）是指出生前到生后1 个月内由各种原因所致的非进行性脑损伤，临床主要表现为中枢性运动障碍和姿势异常。本病并不少见，在发达国家患病率为1‰～4‰，我国为2‰左右。

【病因】 ，

多年来，许多围生期危险因素被认为与脑瘫的发生有关，主要包括：早产与低出生体重、脑缺氧缺血性脑病、产伤、先天性脑发育异常、核黄疸和先天性感染等；然而，对很多患儿却无法明确其具体原因。人们还发现，虽然近20 年来产科和新生儿医疗保健有了极大发展，但脑瘫的发病率却未见下降。为此，近年对脑瘫的病因作了更深入的探讨，目前认为胚胎早期阶段的发育异常，很可能就是导致婴儿早产、低出生体重和易有围生期缺氧缺血等事件的重要原因。胚胎早期的这种发育异常主要来自受孕前后孕妇体内、外环境影响、遗传因素以及孕期疾病引起妊娠早期胎盘羊膜炎症等。

【临床表现】

1．基本表现 脑瘫以出生后非进行性运动发育异常为特征，一般都有以下4 种表现：

（1）运动发育落后和瘫痪肢体主动运动减少：患儿不能完成相同年龄正常小儿应有的运动发育进程，包括抬头、坐、站立、独走等大运动以及手指的精细动作。

（2）肌张力异常：因不同临床类型而异，痉挛型表现为肌张力增高；肌张力低下型则表现为瘫痪肢体松软，但仍可引出腱反射；而手足徐动型表现为变异性肌张力不全。

（3）姿势异常：受异常肌张力和原始反射延迟消失不同情况影响，患儿可出现多种肢体异常姿势，并因此影响其正常运动功能的发挥。体格检查中将患儿分别置于俯卧位、仰卧位、直立位、以及由仰卧牵拉成坐位时，即可发现瘫痪肢体的异常姿势和非正常体位。

（4）反射异常：多种原始反射消失延迟。痉挛型脑瘫患儿腱反射活跃，可引出踝阵挛和阳性Babinski征。

2．临床类型

（1）运动障碍性质分类：

1）痉挛型：最常见，约占全部病例的50％～60％。主要因锥体系受累，表现为上肢肘、腕关节屈曲，拇指内收，手紧握拳状。下肢内收交叉呈剪刀腿和尖足。

2）手足徐动型：除手足徐动外，也可表现扭转痉挛或其他锥体外系受累症状。

3）肌张力低下型：可能因锥体系和锥体外系同时受累，导致瘫痪肢体松软，但腱反射存在。本型常为脑瘫的暂时阶段，以后大多转为痉挛型或手足徐动型。

4）强直型：全身肌张力显著增高、僵硬，锥体外系受损症状。

5）共济失调型：小脑性共济失调。

6）震颤型：多为锥体外系相关的静止性震颤。

7）混合型：以上某几种类型同时存在。

（2）按瘫痪累及部位分类：可分为四肢瘫（四肢和躯干均受累）、双瘫（也是四肢瘫，但双下肢相对较重）、截瘫（双下肢受累，上肢及躯干正常）、偏瘫、三肢瘫和单瘫等。

3．伴随症状和疾病 作为脑损伤引起的共同表现，一半以上脑瘫患儿可能合并智力低下、听力和语言发育障碍，其他如视力障碍、过度激惹、小头畸形、癫痫等。有的伴随症状如流涎、关节脱位则与脑瘫自身的运动功能障碍相关。

【诊断】

脑瘫有多种类型，使其临床表现复杂，容易与婴幼儿时期其他神经及肌肉疾病引起的肌无力相混淆。然而，只要认真询问取病史和体格检查，遵循脑瘫的定义，建立正确诊断并不困难。

1/2～2/3 的患儿可有头颅CT、MRI异常，但正常者不能否定本病的诊断。脑电图可能正常，也可表现异常背景活动，伴有痫性放电波者应注意合并癫痫的可能性。

诊断脑瘫同时，需对患儿同时存在的伴随症状和疾病如智力低下、癫痫、语言听力障碍、关节脱位等做出判断，为本病的综合治疗创造条件。

【治疗】

1．治疗原则

（1）早期发现和早期治疗：婴儿运动系统正处发育阶段，早期治疗容易取得较好疗效。

（2）促进正常运动发育，抑制异常运动和姿势。

（3）采取综合治疗手段：除针对运动障碍外，应同时控制其癫痫发作，以阻止脑损伤的加重。对同时存在的语言障碍、关节脱位、听力障碍等也需同时治疗。

（4）医师指导和家庭训练相结合，以保证患儿得到持之以恒的正确治疗。

2．主要治疗措施

（1）功能训练：

1）体能运动训练（physical therapy）：针对各种运动障碍和异常姿势进行物理学手段治疗，目前常用Vojta和Bobath方法，国内还采用上田法。

2）技能训练（occupational therapy）：重点训练上肢和手的精细运动，提高患儿独立生活技能。

3）语言训练：包括听力、发音、语言和咀嚼吞咽功能的协同矫正。

（2）矫形器的应用：功能训练中，配合使用一些支具或辅助器械，有帮助矫正异常姿势，抑制异常反射的功效。

（3）手术治疗：主要用于痉挛型，目的是矫正畸形，恢复或改善肌力与肌张力的平衡。

（4）其他：如高压氧、水疗、电疗等，对功能训练起辅助作用。

第七节 吉兰-巴雷综合征

吉兰-巴雷综合征（Guillain-Barré Syndrome，GBS）又称急性炎症性脱髓鞘性多神经根病，是目前我国和多数国家小儿最常见的急性周围神经病。该病以肢体对称性弛缓性瘫痪为主要临床特征。病程自限，大多在数周内完全恢复，但严重者急性期可死于呼吸肌麻痹。

【病因及发病机制】

吉兰-巴雷综合征的病因虽不完全明了，但近年的相关研究取得了很大进展，多数学者强调本病是一种急性免疫性周围神经病，多种因素均能诱发本病，但以空肠弯曲菌等前驱感染为主要诱因。

1．感染因素 约2/3的吉兰-巴雷综合征患者在病前6 周内有明确前驱感染史。病原体主要包括：

（1）空肠弯曲菌：是吉兰-巴雷综合征最主要前驱感染病原体，在我国和日本，42％～76％的吉兰-巴雷综合征患者血清中有该菌特异性抗体滴度增高或有病前该菌腹泻史。其中以Penner血清型0：19 和0：4 与本病发病关系最密切。已证实它们的菌体脂多糖涎酸等终端结构与周围神经表位的多种神经节苷脂如GM_1、GD_{1a}等存在类似分子结构，从而发生交叉免疫反应。感染该菌后，血清中同时被激发抗GM_1和抗GD_{1a}等抗神经节苷脂自身抗体，导致周围神经免疫性损伤。

（2）巨细胞病毒：占前驱感染第二位病原体，欧洲和北美地区多见，患者抗该病毒特异性抗体和抗周围神经GM_2抗体同时增高，致病机理也认为与两者的某些抗原结构相互模拟有关。

（3）其他病原体：主要包括EB病毒、带状疱疹病毒、AIDS和其他病毒以及肺炎支原体感染等，致病机理与巨细胞病毒相似。

2.疫苗接种 仅少数吉兰-巴雷综合征的发病与某种疫苗注射有关，主要是狂犬病毒疫苗（发生率1/1000），其他可能有麻疹疫苗、破伤风类毒素和脊髓灰质炎口服疫苗（发生率百万分之一）。

3.免疫遗传因素 人群中虽经历相同病原体前驱感染，但仅有少数人发生吉兰-巴雷综合征，从而推测存在遗传背景的易感个体，如特异的HLA表型携带者，受到外来刺激（如感染）后引起的异常免疫反应，破坏神经原纤维，导致本病的发生。

【病理分类和特征】

周围神经束通常由数十或数百根神经原纤维组成，其中大多数为有髓鞘原纤维（见图16-5）。原纤维中心是脊髓前角细胞运动神经元伸向远端的轴突，轴突外周紧裹由Schwann细胞胞膜同

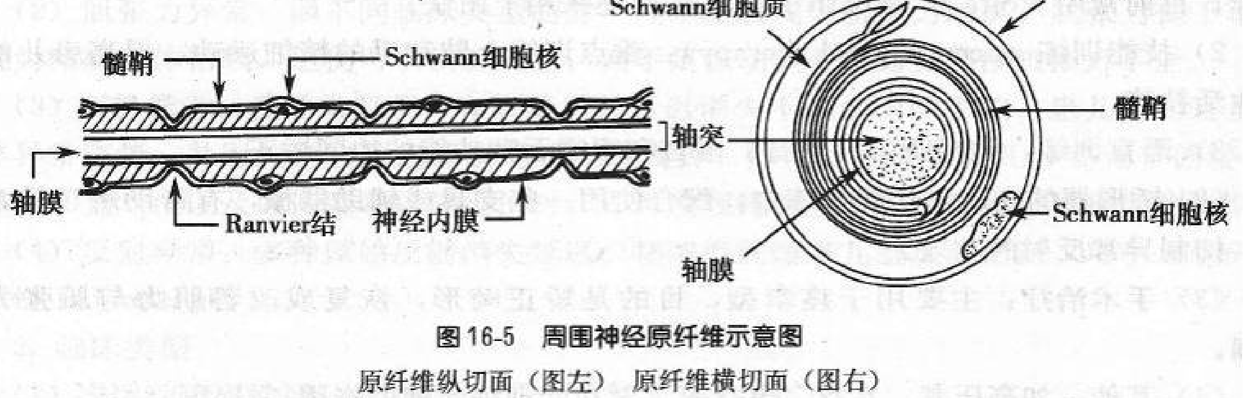

图16-5 周围神经原纤维示意图

原纤维纵切面（图左） 原纤维横切面（图右）

心圆似地围绕轴突旋转而形成的髓鞘。沿原纤维长轴，髓鞘被许多Ranvier结分割成长短相同的节段。相邻两个Ranvier结间的原纤维称结间段，每一结间段实际由一个Schwann细胞的胞膜紧裹。

由于前驱感染病原体种类的差异和宿主免疫遗传因素影响，吉兰-巴雷综合征患者周围神经可主要表现为髓鞘脱失或轴索变性，或两者皆有。主要损及周围神经的运动纤维、或同时损伤运动和感觉纤维，从而形成不同特征的临床和病理类型。当前主要分为以下四种类型：

1．急性炎症性脱髓鞘性多神经病（AIDP） 在T细胞、补体和抗髓鞘抗体作用下，周围神经运动和感觉原纤维同时受累，呈现多灶节段性髓鞘脱失，伴显著巨噬细胞和淋巴细胞浸润，轴索相对完整。

2．急性运动轴索性神经病（AMAN） 结合免疫复合物（补体和特异性抗体）的巨噬细胞经Ranvier结侵入运动神经原纤维的髓鞘和轴突间隙，共同对轴膜免疫性攻击，引起运动神经轴突瓦勒（Wallerian）样变性。病程初期髓鞘相对完整无损。

3．急性运动感觉轴索性神经病（AMSAN） 也是轴突Wallerian样变性为主，但同时波及运动和感觉神经原纤维，病情大多严重，恢复缓慢。

4．Miller-Fisher综合征（MFS） 为吉兰-巴雷综合征特殊亚型，目前尚缺少足够尸解病理资料。临床主要表现为眼部肌肉麻痹和共济失调，无肢体瘫痪。患者血清抗GQ_{1b}抗体增高，而支配眼肌的运动神经末梢、本体感觉通路和小脑神经元均富含此种神经节苷脂。

【临床表现】

任何年龄均可患病，但以学龄前和学龄期儿童居多。我国患儿常以空肠弯曲菌为前驱感染，故农村较城市多见，且夏、秋季发病增多。病前可有腹泻或呼吸道感染史。

1．运动障碍 是本病的主要临床表现。呈急性或亚急性起病，四肢尤其下肢弛缓性瘫痪是本病的基本特征。两侧基本对称，以肢体近段或远段为主，或近、远段同时受累。瘫痪可能在数天或数周内从下肢向上发展，但绝大多数的进行性加重不超过3～4 周。迅速者也可在起病24 小时或稍长时间内出现严重肢体瘫痪或（和）呼吸肌麻痹，后者引起呼吸急促，声音低微和紫绀。

部分患者伴有对称或不对称颅神经麻痹，以核下性面瘫最常见，其次为展神经。当波及两侧Ⅸ、Ⅹ、Ⅻ脑神经时，患者呛咳、声音低哑、吞咽困难，口腔唾液积聚，很易引起吸入性肺炎并加重呼吸困难，危及生命。个别病例出现从上向下发展的瘫痪。

2．感觉障碍 感觉障碍症状相对轻微，很少有感觉缺失者，主要表现为神经根痛和皮肤感觉过敏。由于惧怕牵拉神经根加重疼痛，可有颈项强直，Kernig征阳性。神经根痛和感觉过敏大多在数日内消失。

3．自主神经功能障碍 症状也较轻微，主要表现为多汗、便秘、不超过12～24 小时的一过性尿潴留、血压轻度增高或心律失常等。

本病病程自限。肌肉瘫痪停止进展后数周内，大多数患儿肌力逐渐复原，3～6 个月内完全恢复。但有10％～15％患儿遗留不同程度的肌无力，1.7％～5％死于急性期呼吸肌麻痹。

【实验室检查】

1．脑脊液检查 80％～90％的吉兰-巴雷综合征患者脑脊液中蛋白增高但白细胞计数和其它均正常，乃本病特征。然而，这种蛋白-细胞分离现象一般要到起病后第2 周才出现。

2．神经传导功能测试 以髓鞘脱失为病理改变者，如AIDP 患者，主要呈现运动和感觉神

经传导速度、远端潜伏期延长和反应电位时程增宽，波幅减低不明显。

以轴索变性为主要病变者，如AMAN患者，主要呈现运动神经反应电位波幅显著减低，而AMSAN则同时有运动和感觉神经电位波幅减低，传导速度基本正常。

【诊断】

凡具有急性或亚急性起病的肢体弛缓性瘫痪，两侧基本对称，瘫痪进展不超过4 周，起病时无发热，无传导束型感觉缺失和持续性尿潴留者，均应想到本病可能性。若证实脑脊液蛋白-细胞分离和（或）神经传导功能异常，即可确立本病诊断。

【鉴别诊断】

要注意和其他急性弛缓性瘫痪疾病鉴别，主要是：

1．肠道病毒引起的急性弛缓性麻痹 我国已基本消灭野生型病毒性脊髓灰质炎的发生，但仍有柯萨奇、埃可等其他肠道病毒引起的急性弛缓性瘫痪。根据其肢体瘫痪不对称，脑脊液中可有白细胞增多，周围神经传导功能正常，以及急性期粪便病毒分离阳性，容易与吉兰-巴雷综合征鉴别。

2．急性横贯性脊髓炎 在锥体束休克期表现四肢软瘫需与吉兰-巴雷综合征鉴别，但急性横贯性脊髓炎有尿潴留等持续括约肌功能障碍和感觉障碍平面，而且，急性期周围神经传导功能正常。

【治疗】

1．护理 本病虽缺少特效治疗，但病程自限，大多可望完全恢复，积极的支持治疗和护理措施是顺利康复的关键。对瘫痪正在继续进展的患儿，原则上都应住院观察。①保持呼吸道通畅，勤翻身，防止坠积性肺炎或褥疮；②吞咽困难者要鼻饲，以防吸入性肺炎；③保证足量的水分、热量和电解质供应；④尽早对瘫痪肌群康复训练，防止肌肉萎缩，促进恢复。

2．呼吸肌麻痹的抢救 呼吸肌麻痹是本病死亡的主要原因。对出现呼吸衰竭，或因咳嗽无力及Ⅸ、Ⅹ、Ⅻ脑神经麻痹致咽喉分泌物积聚者，应及时作气管切开或插管，必要时使用机械呼吸以保证有效通气和换气。

3．药物治疗 对病情进行性加重，尤其有呼吸肌或Ⅸ、Ⅹ、Ⅻ脑神经麻痹者，可试用静脉注射大剂量免疫球蛋白400 mg/（kg・d），连用5 天。也有按2 g/kg一次负荷剂量静脉滴注者。有效者24～48 小时内可见病情不再进展，但也有无效者。其总疗效与血浆置换相当。

目前多数专家认为肾上腺皮质激素对本病治疗无效。

第八节 重症肌无力

重症肌无力（myasthenia gravis，MG）是免疫介导的神经肌肉接头处传递障碍的慢性疾病。临床以骨骼肌运动中极易疲劳并导致肌无力，休息或用胆碱酯酶抑制剂后症状减轻为特征。

【病因和发病机理】

正常神经肌接头由突触前膜（即运动神经末梢突入肌纤维的部分）、突触间隙和突触后膜（即肌肉终板膜的接头皱褶）三部分组成。神经冲动电位促使突触前膜向突触间隙释放含有化学递质乙酰胆碱（Ach）的囊泡，在间隙中囊泡释出大量Ach，与近十万个突触后膜上的乙酰胆碱受体（Ach-R）结合，引起终板膜上Na^+通道开放，大量Na^+进入细胞内和K^+排出细胞外，而使突触后膜除极，产生肌肉终板动作电位，在数毫秒内完成神经肌接头处冲动由神经电位-化学递质-肌肉电位的复杂转递过程，引起肌肉收缩。

重症肌无力患者体液中存在抗Ach-R抗体，与Ach共同争夺Ach-R结合部位。同时，又在C3和细胞因子参与下，直接破坏Ach-R和突触后膜，使Ach-R数目减少，突触间隙增宽。虽然突触前膜释放Ach囊泡和Ach的量依然正常，但因受Ach-R抗体与受体结合的竞争，以及后膜上受体数目的减少，致Ach在重复冲动中与受体结合的机率越来越少，很快被突触间隙和终板膜上胆碱酯酶水解成乙酰与胆碱而灭活，或在增宽的间隙中弥散性流失，临床出现肌肉病态性易疲劳现象。抗胆碱脂酶可抑制Ach的降解，增加其与受体结合的机会从而增强终板电位，使肌力改善。

【临床表现】

1．儿童期重症肌无力 大多在婴幼儿期发病，最年幼者6个月，2～3 岁是发病高峰，女孩多见。临床主要表现三种类型：

（1）眼肌型：最多见。单纯眼外肌受累，多数见一侧或双侧眼睑下垂，早晨轻、起床后逐渐加重。反复用力作睁闭眼动作也使症状更明显。部分患儿同时有其他眼外肌如眼球外展、内收或上、下运动障碍，引起复视或斜视等。瞳孔光反射正常。

（2）脑干型：主要表现为第Ⅸ、Ⅹ、Ⅻ脑神经所支配的咽喉肌群受累。突出症状是吞咽或构音困难，声音嘶哑等。

（3）全身型：主要表现运动后四肢肌肉疲劳无力，严重者卧床难起，呼吸肌无力时危及生命。

少数患儿兼有上述2～3 种类型，或由1 种类型逐渐发展为混合型。病程经过缓慢，其间可交替地完全缓解或复发，呼吸道感染常使病情加重。但与成人不同，小儿重症肌无力很少与胸腺瘤并存，但偶可继发于桥本氏甲状腺炎等引起的甲状腺功能低下。约2％的患儿有家族史，提示这些患儿的发病与遗传因素有关。

2．新生儿期重症肌无力 病因特殊，包括两种类型：

（1）新生儿暂时性重症肌无力：重症肌无力女性患者妊娠后娩出的新生儿中，约1/7 的婴儿因体内遗留母亲抗Ach-R抗体，可能出现全身肌肉无力，严重者需要机械呼吸或鼻饲。因很少表现眼肌症状而易被误诊。待数天或数周后，婴儿体内的抗Ach-R抗体消失，肌力即可恢复正常，以后并不存在发生重症肌无力的特别危险性。

（2）先天性重症肌无力：因遗传性Ach-R离子通道异常而患病，与母亲是否重症肌无力无关，患儿出生后全身肌无力和眼外肌受累，症状持续、不会自然缓解，胆碱酯酶抑制剂和血浆交换治疗均无效果。

【诊断】

1．药物诊断性试验 当临床表现支持本病时，腾喜龙（tensilon，依酚氯铵）或新斯的明（neostigmine）药物试验有助诊断确立。前者是胆碱酯酶的短效抑制剂，由于顾忌心律失常副作用一般不用于婴儿。儿童每次0.2 mg/Kg（最大不超过10 mg），静脉注射或肌注，用药后1 分钟即可见肌力明显改善，2～5 分钟后作用消失。

新斯的明则很少有心律失常不良反应，剂量每次0.04 mg/kg，皮下或肌肉注射，最大不超过1 mg，最大作用在用药后15～40 分钟。婴儿反应阴性者4 小时后可加量为0.08 mg/kg。为避免新斯的明引起的面色苍白、腹痛、腹泻、心率减慢、气管分泌物增多等毒蕈碱样不良反应，注射该药前可先肌注阿托品0.01 mg/kg。

2．肌电图检查 对能充分合作完成肌电图检查的儿童，可作神经重复刺激检查，表现为重复电刺激中反应电位波幅的快速降低，对本病诊断较有特异性。本病周围神经传导速度多正常。

3．血清抗Ach-R抗体检查 阳性有诊断价值，但阳性率因检测方法不同而差异。婴幼儿阳

性率低，以后随年龄增加而增高。眼肌型（约40%）又较全身型（70%）低。

【治疗】

重症肌无力为慢性疾病过程，其间可有症状的缓解和复发。眼肌型起病两年后仍无其他肌群受累者，日后将很少发展为其他型。多数患儿经数月或数年可望自然缓解，但有的持续到成年，因此，对有症状者应长期服药治疗，以免肌肉失用性萎缩和肌无力症状进一步加重。

1．胆碱酯酶抑制剂 是多数患者的主要治疗药物。首选药物为溴吡斯的明，口服量新生儿每次新生儿5 mg，婴幼儿10～15 mg，年长儿20～30 mg，最大量每次不超过60 mg，每日3～4 次。根据症状控制的需求和是否有腹痛、黏膜分泌物增多、瞳孔缩小等毒蕈碱样不良反应发生，可适当增减每次剂量与间隔时间。

2．糖皮质激素 基于自身免疫发病机制，各种类型重症肌无力均可使用糖皮质激素。长期规则应用可明显降低复发率。首选药物泼尼松，1～2 mg/（kg・d），症状完全缓解后再维持4～8 周，然后逐渐减量达到能够控制症状的最小剂量，每日或隔日清晨顿服，总疗程2 年。要注意部分患者在糖皮质激素治疗最初1～2 周可能有一过性肌无力加重，故最初使用时最好能短期住院观察，同时要注意皮质激素长期使用的副作用。

3．胸腺切除术 对于药物难控制的病例可考虑胸腺切除术。血清抗Ach-R抗体滴度增高和病程不足两年者常有更好疗效。

4．大剂量静脉注射丙种球蛋白（IVIG）和血浆交换疗法 部分患者有效，但两者价格均昂贵，且一次治疗维持时间短暂，需重复用药以巩固疗效，故主要试用于难治性重症肌无力或重症肌无力危象的抢救。IVIG剂量按400 mg/（kg・d），连用5 天。循环中抗Ach-R抗体滴度增高者可能有更佳疗效。

5．肌无力危象的识别与抢救 治疗过程中患儿可发生两种肌无力危象：

（1）肌无力危象：因治疗延误或措施不当使重症肌无力本身病情加重，可因呼吸肌无力而呼吸衰竭。注射新斯的明能使症状迅速改善。

（2）胆碱能危象：因胆碱酯酶抑制剂过量引起，除明显肌无力外，尚有面色苍白、腹泻、呕吐、高血压、心动过缓、瞳孔缩小及黏膜分泌物增多等严重毒蕈碱样症状。采用腾喜龙l mg肌注，胆碱能危象者出现症状短暂加重，重症肌无力危象者会因用药而减轻。

6．禁用药物 氨基醣甙类抗生素、普鲁卡因胺、普萘洛尔、奎宁等药物有加重患儿神经肌接头传递障碍的作用，甚至呼吸肌严重麻痹，应禁用。

第九节 进行性肌营养不良

进行性肌营养不良（progressive muscular dystrophy）是一组原发于肌肉的遗传性变性疾病。临床特点为进行性加重的对称性肌无力、肌萎缩。根据发病年龄、肌无力分布、病程及预后可分为：假肥大型肌营养不良、Emery-Dreifuss肌营养不良、面肩肱型肌营养不良、肢带型肌营养不良、眼咽型肌营养不良、远端型肌营养不良、强直型肌营养不良及先天性肌营养不良。

假肥大型肌营养不良（pseudohypertrophic muscular dystrophy）是进行性肌营养不良中最常见，也是小儿时期最常见、最严重的一型，无种族或地域差异。本节主要介绍假肥大型肌营养不良。Duchenne和Becker肌营养不良（Duchenne/Becker muscular dystrophy，DMD/BMD）代表假肥大型肌营养不良的两种不同类型，主要发生在学龄前和学龄期，其临床表现相似。DMD发病率为1/3600 活产男婴，BMD仅为其1/l0。

【病因和发病机理】

假肥大型肌营养不良是由于染色体Xp21 上编码抗肌萎缩蛋白（dystrophin）的基因突变所

致，属X-连锁隐性遗传病，一般是男性患病，女性携带突变基因。然而，实际上仅2/3患者的病变基因来自母亲，另1/3患者是自身抗肌萎缩蛋白基因的突变，此类患儿的母亲不携带该突变基因，与患儿的发病无关。

抗肌萎缩蛋白位于肌细胞膜脂质层中，对稳定细胞膜，防止细胞坏死、自溶起重要作用。定量分析表明，DMD患者肌细胞内抗肌萎缩蛋白几乎完全缺失，故临床症状严重；而抗肌萎缩蛋白数量减少则导致BMD，后者预后相对良好，病程进展相对缓慢。由于该蛋白也部分地存在于心肌、脑细胞和周围神经结构中，故部分患者可合并心肌病变、智力低下或周围神经传导功能障碍。

【病理】

显微镜下见肌纤维轻重不等的广泛变性坏死，间有深染肌纤维。束内纤维组织增生或脂肪充填，并见针对坏死肌纤维的反应性灶性单核细胞浸润。

【临床表现】

男孩患病，但个别女孩除携带突变基因外，由于另一X染色体功能失活也可发病。本病主要表现包括：

1．进行性肌无力和运动功能倒退 患儿出生时或婴儿早期运动发育基本正常，少数有轻度运动发育延迟，或独立行走后步态不稳，易跌倒。一般5 岁后症状开始明显，骨盆带肌无力日益严重，行走摇摆如鸭步态，跌倒更频繁，不能上楼和跳跃。肩带和全身肌力随之进行性减退，大多数10 岁后丧失独立行走能力，20 岁前大多出现咽喉肌肉和呼吸肌无力，声音低微，吞咽和呼吸困难，很易发生吸入性肺炎等继发感染死亡。BMD症状较轻，可能存活至40岁后。

2．Gower 征 由于骨盆带肌早期无力，一般在3 岁后患儿即不能从仰卧位直接站起，必须先翻身成俯卧位，然后两脚分开，双手先支撑于地面，继而一只手支撑到同侧小腿，并与另一手交替移位支撑于膝部和大腿上，使躯干从深鞠躬位逐渐竖直，最后成腰部前凸的站立姿势。

3．假性肌肥大和广泛肌萎缩 早期即有骨盆带和大腿部肌肉进行性萎缩，但腓肠肌因脂肪和胶原组织增生而假性肥大，与其他部位肌萎缩对比鲜明。当肩带肌肉萎缩后，举臂时肩胛骨内侧远离胸壁，形成“翼状肩胛”，自腋下抬举患儿躯体时，病儿两臂向上，有从检查者手中滑脱之势。脊柱肌肉萎缩可导致脊柱弯曲畸形。疾病后期发生肌肉挛缩，引起膝、腕关节或上臂屈曲畸形。

4．其他 多数患儿有心肌病，甚至发生心力衰竭，但其严重度与骨骼肌无力并不一致。几乎所有患儿均有不同程度智力损害，与肌无力严重度也不平行，其中20％～30％较明显，IQ<70。

【实验室检查】

1．血清磷酸肌酸激酶（CK）显著增高 可高出正常数十甚至数百倍，这在其他肌病均很少见。其增高在症状出现以前就已存在。当疾病晚期，几乎所有肌纤维已经变性时，血清CK含量反可下降。

2．肌电图 呈典型肌病表现，周围神经传导速度正常。

3．肌肉活体组织检查 见病理描述。

4．遗传学诊断 对活体肌肉组织进行抗肌萎缩蛋白的细胞免疫化学诊断，或采血DNA序列分析可证实抗肌萎缩蛋白基因突变或缺失。

【诊断与鉴别诊断】

1．诊断 血清CK显著增高是诊断本病重要依据，再结合男性患病、腓肠肌假性肥大等典型临床表现，诊断大多不难。个别诊断仍困难者、可考虑肌电图、神经传导速度或肌肉活体组织

检查协助诊断。

2．鉴别诊断

（1）与其他神经疾病鉴别：①脊髓性肌萎缩：本病是由于5q11-13 位点上运动神经元存活基因缺失而引起脊髓前角细胞变性。临床表现为进行性骨骼肌萎缩和肌无力。婴儿型生后即发病，不存在鉴别诊断问题。但少年型脊髓性肌萎缩常在2～7 岁间发病，最初仅表现下肢近端肌无力，进展缓慢，需与本病鉴别。根据脊髓性肌萎缩患者血清CK不增高，肌电图有大量失神经电位，使两者鉴别并不困难。②肌张力低下型脑性瘫痪：根据婴儿期即有肌无力症状，血清CK不增高，无假性肌肥大，可与进行性肌营养不良区别。

（2）与其他肌营养不良肌病的鉴别：其他类型肌营养不良也具有进行性肌萎缩和肌力减退这一基本临床特征，需注意与本病鉴别：①Emery-Dreifuss肌营养不良：X-连锁隐性遗传，病变基因位于Xq28，可在儿童期发病。但该病罕见，进展缓慢，肩胛肌和心肌受累明显但面肌运动正常，智能正常，无假性肥大，血清CK仅轻度增加。②面肩肱型肌营养不良：常染色体显性遗传，故男、女均受累。起病较晚，多在青少年期。面部肌肉最先受累，呈特征性肌病面容，以后逐渐波及肩胛带。由于DMD、BMD几乎都从下肢起病，并有假性肥大，因而容易区别。③肢带型肌营养不良：常染色体隐性或显性遗传。主要影响骨盆带和肩带肌群，也可有远端肌萎缩和假性肥大。但起病晚，多在青少年或成年期起病，男女均受累，很少有心肌、面部肌肉和智力受损者。

【治疗】

迄今尚无特效治疗，但积极的对症和支持治疗措施有助于提高患儿生活质量与延长生命，包括鼓励并坚持主动和被动运动，以延缓肌肉挛缩。对逐渐丧失站立或行走能力者，使用支具以帮助运动和锻炼，并防止脊柱弯曲和肌肉挛缩。保证钙和蛋白质等营养摄入，积极防治致命性呼吸道感染。

曾试用多种药物治疗，皆无肯定效果。泼尼松似有改善肌力，延缓病情发展的功效，开始剂量1 mg/（kg・d），一般用药10 天后见肌力进步。有效者维持剂量平均0.75 mg/（kg・d），连续用药可维持缓解2 年以上。要注意长期使用肾上腺皮质激素的副反应。

针对抗肌萎缩蛋白的基因工程治疗正在研究中。

做好遗传咨询，通过家系调查、CK测定、DNA分析以及对已怀孕的基因携带者进行胎儿产前诊断，以正确开展生育指导。

（王治平）

第十七章 内分泌疾病

第一节 概　　述

激素（hormone）是内分泌系统的最基本物质，源于希腊文hormoa，意思是“激活”。最初将激素定义为由内分泌器官产生、经血循环运输到靶器官或组织发挥效应的微量化学物质。随着现代医学的飞速发展，内分泌学的相关概念发生了很大的改变，激素的范围也显著扩大。细胞因子、生长因子、神经递质、神经肽等都是重要的化学信使，这些化学信使与经典激素虽有一些不同，但都有共同的特征：①作为细胞-细胞间通讯的化学信使；②调节机体的代谢，协调机体各器官、系统的活动以维持内环境稳定，并参与细胞生长、发育和死亡的调控；③具有相同的作用模式，即与靶细胞特定的受体结合后方可发挥作用，且可共用相同的信号传导途径；④在生物学效应上相互交叉。基于这些共性，细胞因子、生长因子、神经递质、神经肽都可纳入激素的范畴。因此，激素在广义上相当于化学信使的总称，是一种参与细胞内外联系的内源性信息分子和调控分子。按其化学本质可将激素分为两类：蛋白质（肽）类与非蛋白质类。蛋白质类包括了蛋白、肽和多肽类激素，如胰岛素、促胃液素、甲状旁腺素和降钙素等；而非蛋白质类则包括类固醇激素（如孕酮、雌二醇、皮质类固醇、维生素D等）、氨基酸衍生物（如色氨酸衍生物包括5-羟色胺、褪黑素等，酪氨酸衍生物包括多巴胺、肾上腺素、甲状腺素等）和脂肪酸衍生物（如前列腺素、血栓素等）。各类激素传递信息的方式不尽相同，按其作用的受体又可分为膜受体激素和核受体激素。前者是亲水性的，又称亲水性激素；后者为脂溶性的，又称脂溶性激素。膜受体激素不能自由透过脂性细胞膜，需要和细胞膜上特异性受体结合，形成配体-受体复合物得以使信息传递至细胞内，进而激活细胞内的第二信使系统。这类激素包括肽类激素、神经递质、生长因子、前列腺素等。非蛋白质类激素大多为作用于核受体的激素，其受体位于细胞内，它可以自由穿透细胞膜及核膜，并识别和结合细胞核或细胞浆内相应受体上的专一DNA序列，诱导靶基因转录活性，完成配体-受体复合物的二聚化、磷酸化等，以此调节靶基因的表达与转录，从而改变细胞功能。

具有内分泌功能的细胞种类众多。经典的内分泌腺体是由多数内分泌细胞聚集形成，如垂体、甲状腺、甲状旁腺、胰岛、肾上腺和性腺等，共同组成传统的内分泌系统。而非经典内分泌器官（如心血管、肝、胃肠道、皮肤、免疫等组织器官）亦具有内分泌功能。如产生促胸腺生成素、促胃液素、促胰液素、促红细胞生成素、肾素-血管紧张素等激素的分泌细胞分散于相应的器官；分泌前列腺素以及胰岛素样生长因子、表皮生长因子、神经生长因子、血小板源性生长因子等各种生长因子的细胞则广泛分布于全身组织中；还有一些具有内分泌功能的神经细胞集中于下丘脑的视上核、室旁核、腹正中核及附近区域，其分泌的肽类激素亦称神经激素，可直接作用于相应的靶器官或靶细胞，也可通过垂体分泌间接调控机体的生理代谢过程。

在经典内分泌学概念中，内分泌细胞及所分泌的激素是特异性的，即一种内分泌细胞只产生一种激素，一种激素也只由一种内分泌细胞产生。新的研究结果则表明一种内分泌细胞可产生几种激素，而同一种激素也可由不同部位的内分泌细胞产生。如同一种垂体细胞可产生黄体生成素（LH）和卵泡刺激素（FSH ）；而生长抑素既可由下丘脑神经元产生，也可由甲状腺C细胞、胰岛D细胞及中枢和外周神经的许多神经元产生。既往认为一个基因只对应于一种肽类

激素的概念也已改变，某些肽类激素的基因由于不同启动子的作用，其转录本的大小不一，使最后的蛋白质产物不同。有些肽类激素在不同的组织中存在的主要形式不同，可出现同一基因产生不同形式激素的现象。

在激素概念演变的同时，对其分泌方式的认识也不断更新。经典的内分泌（endocrine）概念是相对于外分泌（exocrine）而言的，指激素释放入血循环，并转运至相应的靶细胞发挥其生物学效应。广义的概念则认为激素不仅能通过传统的内分泌方式起作用，还可通过邻（旁）分泌（paracrine）、自分泌（autocrine）、并列分泌（juxtacrine）、腔分泌（solinocrine）、胞内分泌（intracrine）、神经分泌（neurocrine）和神经内分泌（neuroendocrine）等方式发挥作用。而且一种激素还可以几种不同的方式起作用。

人们对内分泌系统与神经系统、免疫系统之间内在联系的认识亦日益加深。神经、内分泌、免疫系统构成的网络体系调控着生物的整体功能，三者之间存在着广泛的信息交流，可对感受的信息进行加工、处理、存贮及整合。神经系统通过广泛的外周神经突触及神经细胞分泌的神经递质、内分泌激素、细胞因子等共同调控免疫系统的功能；免疫系统通过免疫细胞产生的多种细胞因子和激素样物质反馈作用于神经内分泌系统，这种双向的复杂作用使两个系统内或系统之间得以相互作用、相互调节。如免疫细胞产生的多种细胞因子，诸如多种白介素、干扰素、肿瘤坏死因子等，可以不同方式参与自身免疫性内分泌疾病的发病，损伤内分泌细胞（如特发性垂体功能减退和尿崩症等）或促进HLA- Ⅱ类抗原异常表达（如甲状腺功能亢进时甲状腺细胞上畸变的HLA-DR抗原的表达）。而细胞因子形成的细胞因子网络对激素的作用与调节也有重大意义，如白介素（IL-1、IL-2、IL-3、IL-6）、肿瘤坏死因子（TNF）等，可刺激下丘脑分泌促肾上腺皮质激素释放激素（CRH），从而使ACTH和皮质醇分泌增加；IL-6还能刺激GH、PRL、LH以及FSH的分泌。胰岛素样生长因子（IGF-1、IGF-2 ）、表皮生长因子（EGF）、碱性成纤维细胞生长因子（bFGF）及转移生长因子-α。（TGF-α）等对激素的分泌和旁分泌的调节都有一定的作用。因此，神经-内分泌-免疫网络的联系对各系统的生理功能和机体的整体功能是必不可少的，其中任何环节的紊乱均不可避免地会影响其他系统的功能。

从胚胎形成直至青春发育期，整个机体处于不断生长、发育和成熟的阶段，内分泌系统本身也在不断的发育和成熟中，而内分泌系统的功能与胎儿器官的形成、分化与成熟以及青少年的生长发育、生理功能、免疫机制等密切相关。在此过程中，激素的产生、分泌、结构和功能异常均可造成内分泌疾病。如下丘脑-垂体是机体最重要的内分泌器官，是内分泌系统的中枢，可以分泌多种激素，控制甲状腺、肾上腺、性腺等内分泌器官的活动。若先天性下丘脑-垂体发育不良，则会造成甲状腺素、促肾上腺皮质激素、促性腺激素的分泌失常，引起相应的症状。在青春发育期开始前，性腺的生长发育过程缓慢，下丘脑-垂体-性腺轴功能处于较低水平，而当青春发育启动后，促性腺激素释放激素的脉冲分泌频率和峰值逐渐增加，LH和FSH的脉冲分泌峰也随之增高，因而出现性征和性器官发育。下丘脑-垂体-性腺轴功能异常的儿童就会出现性发育异常（性发育迟缓或性早熟）。甲状腺素不仅影响胎儿神经系统的成熟，还促进儿童的生长发育和调节新陈代谢，若先天性甲状腺激素分泌不足，则可引起智能落后、身材矮小等症状。生长激素是影响儿童身体增长的重要激素，若垂体生长激素缺乏即导致生长激素缺乏症，引起儿童身材矮小。

儿童内分泌疾病的种类与成人不同，部分内分泌疾病的临床特征、发病机制、治疗手段也与成人有较大区别，而且儿童内分泌疾病在不同的年龄阶段各有特点。儿童常见的内分泌疾病主要有生长迟缓、性分化异常、性早熟、甲状腺疾病、糖尿病、肾上腺疾病、尿崩症等。若患儿在出生后即存在生化代谢紊乱和激素功能障碍，则会严重影响其智能和体格发育，若未能早

期诊治，易造成残疾甚至夭折。如先天性甲状腺功能减低症、先天性肾上腺皮质增生症（失盐型）等。许多环境因素也可引起内分泌疾病，如生态环境中碘缺乏导致地方性甲状腺肿及甲状腺功能减低症，经济发达地区高热量饮食导致肥胖症等。此外还有一些是遗传因素和环境因素共同作用下引起的内分泌疾病，如糖尿病等。由环境因素所致的内分泌疾病也常有遗传学背景，但非单基因缺陷，而是多基因（包括多态性）异常所致。

儿童内分泌疾病一旦确诊，多数需要终生替代治疗，治疗剂量需个体化，并根据病情以及生长发育情况及时调整。在治疗的过程中需要密切随访，以保证患儿有正常的生长发育。自1922年始，先后分离、提纯了胰岛素等为数众多的多肽激素、类固醇激素，并陆续应用于临床，取得了较好的疗效。随着生物技术的不断改进，现已生产出多种高纯度激素、细胞因子、生长因子等制剂，如吸收特别迅速的赖脯胰岛素（lispro）和吸收特别缓慢的甘精胰岛素（glargine），以及重组人生长激素（rhGH ）、促性腺激素释放激素类似物的缓释剂（GnRHa）、生长激素抑制激素（SS）等，并已广泛应用于临床。

近年来，激素测定技术快速发展，放射免疫分析法（RIA）、放射受体分析法（RRA）、酶联免疫吸附法（ELISA）、荧光免疫法（FIA）和免疫化学发光法（ICL）等各种精确测定方法的广泛应用，以及一系列具有临床诊断价值的动态试验（兴奋或抑制）方法的建立和完善，极大地提高了内分泌疾病的诊断水平。内分泌腺的影像学检查，如B超、CT、SPECT、PET和MRI等大大提高了内分泌疾病定位诊断的水平。分子生物学技术在临床研究中的应用，促进了新的疾病的发现。通过基因克隆和测序的手段来诊断单基因遗传病已不困难。随着更多、更新的细胞分子生物学技术的深入发展和临床应用，儿科内分泌学的理论概念也会不断更新和发展。

第二节 生长激素缺乏症

生长激素缺乏症（growth hormone deficiency，GHD）是由于腺垂体合成和分泌生长激素（growth hormone，GH）部分或完全缺乏，或由于GH分子结构异常、受体缺陷等所致的生长发育障碍性疾病。患者身高处于同年龄、同性别正常健康儿童生长曲线第3 百分位数以下或低于平均数减两个标准差，符合矮身材（short stature）标准。发生率约为20/10 万～25/10 万。

【生长激素的合成、分泌和功能】

人生长激素（GH）是由腺垂体细胞合成和分泌，由191 个氨基酸组成的单链多肽，分子量为22 KD。人生长激素基因簇是由编码基因GH_1（GH-N）和$CSHP_1$、CSH_1、GH_2、CSH_2等基因组成的长约55 Kbp的DNA链。人GH编码基因GH_1位于17q22-q24。在血循环中，大约50%的GH与生长激素结合蛋白（GHBP）结合，以GH-GHBP复合物的形式存在。生长激素的释放受下丘脑分泌的两种神经激素，即促生长激素释放激素（GHRH）和生长激素释放抑制激素（somatostatin， SRIH或GHIH）的调节。GHRH是含有44 个氨基酸残基的多肽，促进垂体GH分泌细胞合成、分泌GH；SRIH是环状结构的14 肽，对GH的合成和分泌有抑制作用。垂体在这两种多肽的相互作用下以脉冲方式释放GH，而中枢神经系统则通过多巴胺、5-经色胺和去甲肾上腺素等神经递质调控下丘脑GHRH和SRIH的分泌。

GH的自然分泌呈脉冲式，约每2～3 小时出现一个峰值，夜间入睡后分泌量增高，且与睡眠深度有关，在Ⅲ或Ⅳ期睡眠相时达高峰；白天空腹时和运动后偶见高峰。初生婴儿血清GH水平较高，分泌节律尚未成熟，因此睡-醒周期中GH水平少有波动。生后2～3 周血清GH浓度开始下降，分泌节律在生后2 个月开始出现。儿童期每日GH分泌量超过成人，在青春发育期

更明显。

GH可以直接作用于细胞发挥生物效应，但其大部分功能必须通过胰岛素样生长因子（insulin-like growth factor，IGF）介导。IGF是一组具有促进生长作用的多肽，人体内有两种IGF，即IGF-1 和IGF-2 。IGF-1 是分子量为7.5 KD的单链多肽，其编码基因位于12q22-q24.1，长约85 kb，有6 个外显子和5 个内含子，分泌细胞广泛存在于肝、肾、肺、心、脑和肠等组织中，其合成主要受GH的调节，亦与年龄、营养和性激素水平等因素有关。各组织合成的IGF-1 大都以自分泌或邻分泌方式发挥其促生长作用。IGF-2 的作用尚未完全阐明。循环中的IGF主要由肝脏分泌。血循环中90%的IGF-1 与IGFBP结合，仅1%左右是游离的。GH是调节血IGF-1和IGFBP-3 浓度的最主要因素，IGF-1 和IGFBP-3 水平随GH分泌状态而改变，但其改变速度较慢。因此，血中 IGF-1 和IGFBP-3 水平相对稳定，而且无明显脉冲式分泌和昼夜节律变化，能较好地反映内源性生长激素分泌状态。血循环中的GH及IGF-1 可反馈调节垂体GH的分泌，或间接作用于下丘脑抑制GHRH的分泌，并可刺激SRIH分泌。

GH的基本功能是促进生长，同时也是体内多种物质代谢的重要调节因子。其主要生物效应为：①促生长效应：促进人体各种组织细胞增大和增殖，使骨骼、肌肉和各系统器官生长发育，骨骼的增长即导致身体长高。②促代谢效应：GH促生长作用的基础是促进合成代谢，可促进蛋白质的合成和氨基酸的转运和摄取；促进肝糖原分解，减少对葡萄糖的利用，降低细胞对胰岛素的敏感性，使血糖升高；促进脂肪组织分解和游离脂肪酸的氧化生酮过程；促进骨骺软骨细胞增殖并合成含有胶原和硫酸粘多糖的基质。

【病因】

下丘脑-垂体功能障碍或靶细胞对GH无应答反应等均会造成生长落后，根据病因可分为以下几类：

1．原发性

（1）下丘脑-垂体功能障碍垂体的发育异常，如不发育、发育不良或空蝶鞍均可引起生长激素合成和分泌障碍，其中有些伴有视中隔发育不全（septo-optic dysplasia)，唇裂、腭裂等畸形。由于下丘脑功能缺陷所造成的生长激素缺乏症远较垂体功能不足导致者为多。其中因神经递质-神经激素功能途径的缺陷，导致GHRH分泌不足引起的身材矮小者称为生长激素神经分泌功能障碍（GHND），这类患儿的GH分泌功能在药物刺激试验中可能表现正常。

（2）遗传性生长激素缺乏（HGHD） GH_1基因缺陷引起单纯性生长激素缺乏症（IGHD)，而垂体Pit-1转录因子缺陷导致多种垂体激素缺乏症（MPHD)，临床上表现为多种垂体激素缺乏。IGHD按遗传方式分为Ⅰ（AR)、Ⅱ（AD)、Ⅲ（X连锁）3 型。此外，还有少数矮身材儿童是由于GH分子结构异常、GH受体缺陷（Laron综合征）或IGF受体缺陷（非洲Pygmy人）所致，临床症状与生长激素缺乏症相似，但呈现GH抵抗或IGF-1抵抗，血清GH水平不降低或反而增高，是较罕见的遗传性疾病。

2．继发性 多为器质性，常继发于下丘脑、垂体或其他颅内肿瘤、感染、细胞浸润、放射性损伤和头颅创伤等，其中产伤是国内生长激素缺乏症最主要的病因。

3．暂时性 体质性青春期生长延迟、社会心理性生长抑制、原发性甲状腺功能减低等均可造成暂时性GH分泌功能低下，在外界不良因素消除或原发疾病治疗后即可恢复正常。

【临床表现】

特发性生长激素缺乏症多见于男孩，男：女为3：1。患儿出生时身长和体重均正常，1 岁以后出现生长速度减慢，身高落后比体重低下更为显著，身高低于同年龄、同性别正常健康儿童生长曲线第3 百分位数以下（或低于平均数减两个标准差），身高年增长速率<5 cm，智能发

育正常。患儿头颅呈圆形，面容幼稚，脸圆胖，皮肤细腻，头发纤细，下颌和颏部发育不良，牙齿萌出延迟且排列不整齐。患儿虽生长落后，但身体各部比例匀称，与其实际年龄相符。骨骼发育落后，骨龄落后于实际年龄2 岁以上，但与其身高年龄相仿，骨骺融合较晚。多数青春期发育延迟。

一部分生长激素缺乏患儿同时伴有一种或多种其他垂体激素缺乏，这类患儿除生长迟缓外，尚有其他伴随症状：伴有促肾上腺皮质激素（ACTH）缺乏者容易发生低血糖；伴促甲状腺激素（TSH）缺乏者可有食欲不振、活动较少等轻度甲状腺功能不足的症状；伴有促性腺激素缺乏者性腺发育不全，出现小阴茎，至青春期仍无性器官和第二性征发育等。

器质性生长激素缺乏症可发生于任何年龄，其中由围生期异常情况导致者，常伴有尿崩症。颅内肿瘤则多有头痛、呕吐、视野缺损等颅内压增高以及视神经受压迫的症状和体征。

【实验室检查】

1．生长激素刺激试验 生长激素缺乏症的诊断依靠GH水平的测定。生理状态下，GH呈脉冲式分泌，这种分泌与垂体、下丘脑、神经递质以及大脑结构和功能的完整性有关，有明显个体差异，并受睡眠、运动、摄食和应激的影响，故单次测定血GH水平不能真正地反映机体的GH分泌情况。因此，对疑诊患儿必须进行GH刺激试验，以判断其垂体分泌GH的功能。常用测定GH分泌功能试验见表17-1 。

表17-1 生长激素分泌功能试验

试　　验	方　　法	采血时间
生理性		
1．运动	禁食4～8 小时后，剧烈活动15～20 分钟	开始活动后20～40 分钟
2．睡眠	晚间入睡后用脑电图监护	Ⅲ～Ⅳ期睡眠时
药物刺激		
1．胰岛素	0.075 U/kg，静注	0，15，30，60，90分钟测血糖、GH
2．精氨酸	0.5/kg，用注射用水配成5%～10%溶液，30 分钟静滴完	0，30，60，90，120分钟测GH
3．可乐定	0.004 mg/kg，1 次口服	同上
4．左旋多巴	10 mg/kg，1 次口服	同上

经典的GH刺激试验包括生理性刺激试验（睡眠、运动）和药物刺激试验。生理性刺激试验要求一定的条件和设备：深睡眠试验必须在脑电图的监测下，于睡眠的第三期或第四期采血测GH才能得到正确的结果；运动试验则必须达到规定的强度，才能产生促进GH分泌的作用。因此，生理性试验在儿童中难以获得可靠的资料。药物刺激试验是借助于胰岛素、精氨酸、可乐定、高血糖素、左旋多巴等药物促进GH分泌而进行的，作用机制随药物而不同，GH分泌峰值的大小和呈现的时间也不同。为排除外源因素的影响，刺激试验前应禁食、卧床休息，于试验前30 分钟放好留置针头，在上午8～10 时进行试验。

一般认为GH的峰值在试验过程中<10 μg/L即为分泌功能不正常。GH峰值<5 μg/L，为GH完全缺乏；GH峰值5～10 μg/L，为GH部分缺乏。由于各种GH刺激试验均存在一定局限性，必须两种以上药物刺激试验结果都不正常时，.才可确诊为生长激素缺乏症。一般多选择胰岛素加可乐定或左旋多巴试验。对于年龄较小的儿童，尤空腹时有低血糖症状者应用胰岛素时应注意

监护，因其可能引起低血糖惊厥等严重反应。

此外，若需区别病变部位是在下丘脑还是在垂体，须进行GHRH刺激试验。

2．血GH的24 小时分泌谱测定 正常人生长激素峰值与基值差别很大，24 小时的GH分泌量可以比较准确地反映体内GH分泌情况。尤其是对GHND患儿，其GH分泌功能在药物刺激试验可为正常，但其24 小时分泌量则不足，夜晚睡眠时的GH峰值亦低。但该方法繁琐，抽血次数多，不易为患儿接受。

3．胰岛素样生长因子（IGF-1）和IGFBP-3的测定 IGF-1主要以蛋白结合的形式（IGFBPs）存在于血循环中，其中以IGFBP-3为主（95%以上）。IGFBP-3有运送和调节IGF-1的功能，其合成也受GH-IGF轴的调控，因此IGF-1和IGFBP-3都是检测GH-IGF轴功能的指标。两者分泌模式与GH不同，呈非脉冲式分泌，较少日夜波动，血循环中的水平比较稳定。血清IGF-1出生时的水平非常低，随后在儿童期缓慢升高，在青春发育期升高显著，以后随着年龄的增长而有所减少。青春期女孩出现高峰的时间约早于男孩2 年。IGFBP-3 的水平变动与其相似，但变化较小。目前IGF-1、IGFBP-3 一般可作为5 岁到青春发育期前儿童生长激素缺乏症筛查检测，但该指标有一定的局限性。正常人IGF-1 和IGFBP-3 水平受各种各样的因素影响，如性别、年龄、营养状态、性发育程度和甲状腺功能状况等，故必须建立不同性别和年龄组儿童的正常参考值范围。

另外，IGF-1 测定还可监测GH治疗后的反应，并具有一定的鉴别诊断意义。如矮小儿童GH激发试验中GH峰值正常，而IGF-1 低下，但在注射外源性GH后，IGF-1 升高，生长速率加快，表明该儿童的生长激素分子有变异；如IGF-1 不升高，生长不加速，则表明生长激素分子无变异，可能系生长激素受体缺陷。

4．其他辅助检查

（1）X线检查：常用左手腕、掌、指骨正位片评定骨龄。生长激素缺乏症患儿骨龄落后于实际年龄2 岁或2 岁以上。

（2） CT或MRI检查：已确诊为生长激素缺乏症的患儿，根据需要选择头颅CT或MRI检查，以了解下丘脑-垂体有无器质性病变，尤其对检测肿瘤有重要意义。

5．其他内分泌检查 生长激素缺乏症诊断一旦确立，应检查下丘脑-垂体轴的其他功能。根据临床表现可选择测定TSH、T_4或促甲状腺素释放激素（TRH）刺激试验和促性腺激素释放激素（GnRH）刺激试验以判断下丘脑-垂体-甲状腺轴和性腺轴的功能。

6．染色体检查 对矮身材患儿具有体态发育异常者应进行核型分析，尤其是女性矮小伴青春期发育延迟者，应常规行染色体分析，排除常见的染色体疾病如Turner综合征等。

【诊断和鉴别诊断】

1．诊断 依据：①匀称性身材矮小，身高落后于同年龄、同性别正常儿童生长曲线第3 百分位数以下者（或低于平均数减两个标准差）；②生长缓慢，生长速率<5 cm/年；③骨龄落后于实际年龄2 年以上；④两种药物激发试验结果均示GH峰值低下；⑤智能正常，与年龄相称；⑥排除其他影响生长的疾病。

2．鉴别诊断 引起生长落后的原因很多，需与生长激素缺乏症鉴别的主要有：

（1）家族性矮身材：父母身高均矮，小儿身高常在第3 百分位数左右，但其年生长速率>5 cm/年，骨龄和年龄相称，智能和性发育正常。

（2）体质性青春期延迟：多见于男孩。青春期开始发育的时间比正常儿童迟3～5 年，青春期前生长缓慢，骨龄也相应落后，但身高与骨龄一致，青春期发育后其最终身高正常。父母一方往往有青春期发育延迟病史。

（3）特发性矮身材（idiopathic short stature，ISS）：病因不明，出生时身长和体重正常；生长速率稍慢或正常，一般每年生长速率<5 cm；两项GH激发试验的GH峰值≥10 μg/L， IGF-1的浓度正常；骨龄正常或延迟。无明显的慢性器质性疾病（肝、肾、心、肺、内分泌代谢病和骨骼发育障碍），无心理和严重的情感障碍。

（4）先天性卵巢发育不全综合征（Turner综合征）：女孩身材矮小时应考虑此病。本病的临床特点为：身材矮小；第二性征不发育；具有特殊的躯体特征，如颈短、颈蹼、肘外翻、后发际低、乳距宽、色素痣多等。典型的Turner综合征与生长激素缺乏症不难区别，但嵌合型或等臂染色体所致者因症状不典型，应进行染色体核型分析以鉴别。

（5）先天性甲状腺功能减低症：该症除有生长发育落后、骨龄明显落后外，还有基础代谢率低、智能低下，故不难与生长激素缺乏症区别。但有些晚发性病例症状不明显，需借助血T_4降低、TSH升高等指标鉴别。

（6）骨骼发育障碍：各种骨、软骨发育不全等，均有特殊的面容和体态，可选择进行骨骼X线片检查以鉴别。

（7）其他内分泌代谢病引起的生长落后：先天性肾上腺皮质增生症、性早熟、皮质醇增多症、黏多糖病、糖原累积病等各有其特殊的临床表现，易于鉴别。

【治疗】

1. 生长激素 基因重组人生长激素（rhGH）替代治疗已被广泛应用，目前大都采用0. 1 U/kg，每晚临睡前皮下注射一次，每周6～7 次的方案。治疗应持续至骨髓闭合为止。治疗时年龄越小，效果越好，以第1 年效果最好，身高增长可达到10～12 cm/年以上，以后生长速度逐渐下降。在用rhGH治疗过程中可能出现甲状腺素缺乏，故须监测甲状腺功能，若有缺乏，应适当加用甲状腺素同时治疗。血清IGF-1和IGFBP-3水平检测可作为rhGH疗效和安全性评估的指标。

应用rhGH治疗的副作用较少，主要有：①注射局部红肿，与rhGH制剂纯度不够以及个体反应有关，停药后可消失；②少数患者注射后数月会产生抗体，但对促生长疗效无显著影响；③较少见的副作用有暂时性视乳头水肿、颅内高压等；④此外研究发现有增加股骨头骺部滑出和坏死的发生率，但危险性相当低。目前临床资料未显示rhGH治疗可增加肿瘤发生或复发的危险性，但对恶性肿瘤及严重糖尿病患者建议不用rhGH，

2．同时伴有性腺轴功能障碍的生长激素缺乏症患儿骨龄达12 岁时可开始用性激素治疗。男性可注射长效庚酸睾酮25 mg，每月1 次，每3 个月增加25 mg，直至每月100 mg；女性可用炔雌醇1～2 μg/日，或妊马雌酮（premarin）自每日0.3 mg起酌情逐渐增加，同时需监测骨龄。

第三节 中枢性尿崩症

尿崩症（diabetes insipidus，DI）是由于患儿完全或部分丧失尿液浓缩功能，以多饮、多尿、尿比重低为特点的临床综合征。造成尿崩症的原因很多，其中较多见的是由于抗利尿激素（anti-diuretic hormone，ADH，又名精氨酸加压素，arginine vasopressin，AVP）分泌或释放不足引起，称中枢性尿崩症。

【病因】

AVP是由下丘脑视上核和室旁核神经细胞合成的一种9 肽，其编码基因位于20p13。AVP的分泌受很多因素的影响，其中最重要的是细胞外液的渗透压和血容量。位于下丘脑视上核和渴觉中枢附近的渗透压感受器同时控制着AVP的分泌和饮水行为，正常人血浆渗透压为280～290 mmol/L，波动范围为士1.8%。AVP基因结构异常、下丘脑及神经垂体发育缺陷，或下丘脑-神经束-神经垂体区域受到炎症、肿瘤、外伤、手术、自身免疫损伤等均能产生中枢性尿崩症。

可分为三类：

1．特发性因下丘脑视上核或室旁核神经元发育不全或退行性病变所致。多数为散发，部分患儿与自身免疫反应有关。

2．器质性（继发性）任何侵犯下丘脑、垂体柄或神经垂体的病变都可发生尿崩症。

（1）肿瘤：约1/3以上患儿由颅内肿瘤所致，常见有颅咽管瘤、视神经胶质瘤、松果体瘤等。

（2）损伤：如颅脑外伤（特别是颅底骨折）、手术损伤（尤其下丘脑或垂体部位手术）、产伤等。

（3）感染：少数患儿是由于颅内感染、弓形虫病和放线菌病等所致。

（4）其他：如Langerhans细胞组织细胞增生症或白血病细胞浸润等。

3．家族性（遗传性） 极少数是由于编码AVP的基因或编码运载蛋白Ⅱ的基因突变所造成，为常染色体显性或隐性遗传。如同时伴有糖尿病、视神经萎缩和耳聋者，即为DIDMOD综合征，是由于4p16的*wfs*1 基因多个核苷酸变异所致，又称Wolfram综合征。

【临床表现】

本病可发生于任何年龄，以烦渴、多饮、多尿为主要症状。饮水多（可> 3000 ml / m^2），尿量可达 4～10 L，甚至更多，尿比重低且固定。夜尿增多，可出现遗尿。婴幼儿烦渴时哭闹不安，不肯吃奶，饮水后安静。由于喂水不足可发生便秘、低热、脱水甚至休克，严重脱水可致脑损伤及智能缺陷。儿童由于烦渴、多饮、多尿可影响学习和睡眠，出现少汗、皮肤干燥苍白、精神不振、食欲低下、体重不增、生长缓慢等症状。如充分饮水，一般情况正常，无明显体征。

【实验室检查】

1．尿液检查 每日尿量可达4～10 L，色淡，尿比重小于1.005，尿渗透压可<200 mmol/L，尿蛋白、尿糖及有形成分均为阴性。

2．血生化检查 血钠、钾、氯、钙、镁、磷等一般正常，肌酐、尿素氮正常，血渗透压正常或偏高。无条件查血浆渗透压者可用公式推算：渗透压=2×（血钠+血钾）+血糖+血尿素氮，计算单位均用 mmol/ L。

3．禁水试验 旨在观察患儿在细胞外液渗透压增高时的浓缩尿液的能力。患儿自试验前一天晚上 7～8 时开始禁食，直至试验结束。试验当日晨8 时开始禁饮，先排空膀胱，测定体重、采血测血钠及渗透压；然后每小时排尿一次，测尿量、尿渗透压（或尿比重）和体重，直至相邻两次尿渗透压之差连续两次< 30 mmol/L，或体重下降达 5%，或尿渗透压≥ 800 mmol/L，即再次采血测渗透压、血钠。结果：正常儿童禁饮后不出现脱水症状，每小时尿量逐渐减少，尿比重逐渐上升，尿渗透压可> 800 mmo1/L，而血钠、血渗透压均正常。尿崩症患者持续排出低渗尿，血清钠和血渗透压分别上升超过 145 mmol/L和 295 mmol/L，体重下降 3%～5% 。试验过程中必须严密观察，如患儿烦渴加重并出现严重脱水症状需终止试验并给予饮水。

4．加压素试验 禁水试验结束后，皮下注射垂体后叶素 5 U（或精氨酸加压素 0.1 U/kg），然后两小时内多次留尿，测定渗透压。如尿渗透压上升峰值超过给药前的50%，则为完全性中枢性尿崩症；在9%～50%者为部分性尿崩症；肾性尿崩症小于9%。

5．血浆 AVP 测定 血浆 AVP 水平对于中枢性尿崩症的诊断意义不大，但血浆 AVP 结合禁水试验有助于部分性中枢性尿崩症和肾性尿崩症的鉴别诊断。中枢性尿崩症血浆 AVP 浓度低于正常；肾性尿崩症血浆 AVP 基础状态可测出，禁饮后明显升高而尿液不能浓缩。精神性多饮 AVP 分泌能力正常，但病程久、病情严重者，由于长期低渗状态，AVP 的分泌可受到

抑制。

6．影像学检查 选择性进行头颅X线平片、CT 或 MRI 检查，以排除颅内肿瘤，明确病因，指导治疗。

【诊断和鉴别诊断】

中枢性尿崩症需与其他原因引起的多饮、多尿相鉴别：

1．高渗性利尿 如糖尿病、肾小管酸中毒等，根据血糖、尿比重、尿渗透压及其他临床表现即可鉴别。

2．高钙血症 见于维生素D中毒、甲状旁腺功能亢进症等。

3．低钾血症 见于原发性醛固酮增多症、慢性腹泻、Bartter综合征等。

4．继发性肾性多尿 慢性肾炎、慢性肾盂肾炎等导致慢性肾功能减退时。

5．原发性肾性尿崩症 为X连锁或常染色体显性遗传疾病，是由于肾小管上皮细胞对AVP无反应所致。发病年龄和症状轻重差异较大，重者生后不久即出现症状，可有多尿、脱水、体重不增、生长障碍、发热、末梢循环衰竭甚至中枢神经系统症状。轻者发病较晚，当患儿禁饮时，可出现高热、末梢循环衰竭、体重迅速下降等症状。禁水、加压素试验均不能提高尿渗透压。

6．精神性多饮 又称精神性烦渴。常有精神因素存在，由于某些原因引起多饮后导致多尿，多为渐进性起病，多饮多尿症状逐渐加重，但夜间饮水较少，且有时症状出现缓解。患儿血钠、血渗透压均处于正常低限。由于患儿分泌 AVP 能力正常，故禁水试验较加压素试验更能使其尿渗透压增高。

【治疗】

1．病因治疗 对有原发病灶的患儿必须针对病因治疗。肿瘤可手术切除。特发性中枢性尿崩症，应检查有无垂体及其他激素缺乏情况。渴感正常的患儿应充分饮水，但若有脱水、高钠血症时应缓慢给水，以免造成脑水肿。

2．药物治疗

（1）鞣酸加压素：即长效尿崩停，为混悬液，用前需稍加温并摇匀，再进行深部肌内注射，开始注射剂量为0.1～0.2 ml，作用可维持3～7 天，须待多饮多尿症状出现时再给用药，并根据疗效调整剂量。用药期间应注意控制患儿的饮水量，以免发生水中毒。

（2）1-脱氨-8-D-精氨酸加压素（DDAVP）：为合成的 AVP 类似物。喷鼻剂：含量100 μg/ml，用量0.05～0.15 ml/d，每日1～2次鼻腔滴入，用前需清洁鼻腔，症状复现时再给下次用药。口服片剂：醋酸去氨加压素（弥凝，minirin），50～100 μg/次，每日1～2次。DDAVIP 的副作用很小，偶有引起头痛或腹部不适者。

（3）其他药物：①噻嗪类利尿剂：一般用氢氯噻嗪（双氢克尿噻），每日3～4 mg/kg，分 3 次服用；②氯磺丙脲：增强肾脏髓质腺苷环化酶对 AVP 的反应，每日150 mg/m^2，一次口服；③氯贝丁酯：增加 AVP 的分泌或加强 AVP 的作用。每日15～25 mg/kg，分次口服。副作用为胃肠道反应、肝功能损害等；④卡马西平：具有使 AVP 释放的作用，每日10～15 mg/kg。上述药物临床已较少应用。

第四节 性 早 熟

性早熟（sexual precocity，或称 precocious puberty）是指女孩在 8 岁、男孩在 9 岁以前呈现第二性征。近年研究显示儿童青春发育时间有提前趋势，但我国民前仍沿用以往的标准。

【下丘脑-垂体-性腺轴功能】

人体生殖系统的发育和功能维持受下丘脑-垂体-性腺轴（HPGA）的控制。下丘脑以脉冲形式分泌促性腺激素释放激素（gonadotropin releasing hormone， GnRH）刺激腺垂体分泌促性腺激素（gonadotropin hormone，Gn），即黄体生成素（luteinizing hormone，LH）和卵泡刺激素（follicle stimulating hormone，FSH），促进卵巢和睾丸发育，并分泌雌二醇和睾酮。下丘脑的这种GnRH脉冲分泌在新生儿期开始即已存在，但由于受到中枢神经系统的控制以及对性激素的负反馈甚为敏感，GnRH的分泌量甚少。在整个儿童期，血清LH及FSH均较低下，FSH的水平稍高于LH，女孩尤为明显。待至10 岁左右进入青春期后，下丘脑对性激素负反馈作用的敏感度下降，GnRH的分泌脉冲数和分泌峰值在睡眠时逐渐增加，LH和 FSH的分泌脉冲峰也随之在晚间增高，特别是LH分泌量的上升高于FSH，这种现象逐渐扩展为全日持续性，使性腺和性器官得以进一步发育，青春期于是开始。

【正常青春发育】

青春期是指从第二性征开始发育到完全成熟这一时段。青春期开始的年龄取决于下丘脑-垂体-性腺轴的功能启动的迟早，通常女孩在10～12 岁时开始，男孩则在12～14 岁时开始，较女孩迟2 年。青春期性发育遵循一定的规律，女孩青春期发育顺序为：乳房发育，阴毛、外生殖器的改变，月经来潮，腋毛。整个过程约需1.5～6 年，平均4 年。在乳房开始发育一年后，身高会急骤增长。男孩性发育则首先表现为睾丸容积增大（睾丸容积超过3 ml时即标志着青春期开始，达到6 ml以上时即可有遗精现象），继之阴茎增长增粗，出现阴毛、腋毛生长及声音低沉、胡须等成年男性体态特征，整个过程需5 年以上。在第二性征出现时，小儿身高和体重增长加速。性发育过程的分期见表17-2。

表7-2 性发育过程的分期（Tanner）

分期	乳 房（B）	睾丸、阴茎（G）	阴 毛（P）	其 他
1	幼儿型	幼儿型，睾丸直径<2.5 cm（1～3 ml）*	无	
2	出现硬结，乳头及乳晕稍增大	双睾和阴囊增大；睾丸直径>2.5 cm（4～8ml）；阴囊皮肤变红、薄、起皱纹；阴茎稍增大	少许稀疏直毛，色浅；女孩限阴唇处；男孩限阴茎根部	生长增速
3	乳房和乳晕更增大，侧面呈半圆状	阴囊、双睾增大，睾丸长径约3.5cm（10～15ml）；阴茎开始增长	毛色变深、变粗，见于耻骨联合上	生长速率渐达高峰；女孩出现腋毛；男孩渐见胡须、痤疮、声音变调
4	乳晕、乳头增大，侧面观突起于乳房半圆上	阴囊皮肤色泽变深；阴茎增长、增粗，龟头发育；睾丸长径约 4 cm（15～20 ml）	如同成人，但分布面积较小	生长速率开始下降；女孩见初潮
5	成人型	成人型，睾丸长径>4 cm（>20 ml）	成人型	

*括号内数字系用Prader睾丸计测定的睾丸容积

【病因和分类】

性早熟按下丘脑-垂体-性腺轴（HPGA）功能是否提前发动分为中枢性（central precocious puberty，CPP或GnRH依赖性、真性、完全性）和外周性（peripheral precocious puberty, PPP或非GnRH依赖性、假性）两类。

不完全性性早熟（或部分性、变异型青春发育）为中枢性性早熟的变异，包括单纯性乳房早发育（premature thelarche）、单纯性阴毛早现（premature pubarche）和单纯性早初潮（premature menarche）等。

1．中枢性性早熟(centralprecociouspuberty，CPP) 亦称真性性早熟，由于下丘脑-垂体-性腺轴功能过早启动，GnRH脉冲分泌，患儿除有第二性征的发育外，还有卵巢或睾丸的发育。性发育的过程和正常青春期发育的顺序一致，只是年龄提前。

（1）特发性性早熟(idiopathic precocious puberty) ：又称体质性性早熟，是由于下丘脑对性激素的负反馈的敏感性下降，使促性腺素释放激素过早分泌所致。女性多见，约占女孩CPP的80％以上。

（2）继发性性早熟：多见于中枢神经系统异常，包括：①肿瘤或占位性病变：下丘脑错构瘤、囊肿、肉芽肿；②中枢神经系统感染；③获得性损伤：外伤、术后、放疗或化疗；④先天发育异常：脑积水，视中隔发育不全等。

（3）其他疾病：少数未经治疗的原发性甲状腺功能减低症患者可伴发中枢性性早熟。

2．外周性性早熟(peripheral precocious puberty) 亦称假性性早熟。是非受控于下丘脑-垂体-性腺功能所引起的性早熟，有第二性征发育和性激素水平升高，但下丘脑-垂体-性腺轴不成熟，无性腺的发育。

（1）性腺肿瘤：卵巢颗粒-泡膜细胞瘤、黄体瘤、睾丸间质细胞瘤、畸胎瘤等。

（2）肾上腺疾病：肾上腺肿瘤、先天性肾上腺皮质增生等。

（3）外源性：如含雌激素的药物、食物、化妆品等。

（4）其他疾病：如McCune-Albright 综合征。

3．部分性性早熟 单纯性乳房早发育、单纯性阴毛早发育、单纯性早初潮。

【临床表现】

性早熟以女孩多见，女孩发生特发性性早熟约为男孩的9 倍；而男孩性早熟以中枢神经系统异常（如肿瘤）的发生率较高。

中枢性性早熟的临床特征是提前出现的性征发育与正常青春期发育程序相似，但临床表现差异较大。在青春期前的各个年龄组都可以发病，症状发展快慢不一，有些可在性发育一定程度后停顿一段时期再发育，亦有的症状消退后再发育。在性发育的过程中，男孩和女孩皆有身高和体重过快的增长和骨骼成熟加速。早期患儿身高较同龄儿童高，但由于骨骼的过快增长可使骨骺融合较早，成年后的身材反而较矮小。在青春期成熟后，患儿除身高矮于一般群体外，其余均正常。

外周性性早熟的性发育过程与上述规律迥异。男孩性早熟应注意睾丸的大小。睾丸容积增大提示中枢性性早熟；如果睾丸未见增大，但男性化进行性发展，则提示外周性性早熟，其雄性激素可能来自肾上腺。

颅内肿瘤所致的性早熟患儿在病程早期常仅有性早熟表现，后期始见颅压增高、视野缺损等定位征象，需加以警惕。

【实验室检查】

1．GnRH刺激试验 特发性性早熟患儿血浆FSH、LH基础值可能正常，需借助于GnRH刺激试验，亦称黄体生成素释放激素（LHRH）刺激试验。一般采用静脉注射GnRH，按2.5 μg/kg（最大剂量=100 μg），于注射前（基础值）和注射后30、60、90 及120 分钟分别采血测定血清LH和FSH。当LH峰值>12 U/L（女），或>25 U/L（男）；或LH/FSH峰值>0.6～1.0，可以认为其性腺轴功能已经启动。

2．骨龄测定 根据手和腕部X线片评定骨龄，判断骨骼发育是否超前。性早熟患儿一般骨龄超过实际年龄。

3．B超检查 选择盆腔B超检查女孩卵巢、子宫的发育情况；男孩注意睾丸、肾上腺皮质等部位。若盆腔B超显示卵巢内可见4个以上直径≥4 mm的卵泡，则为性早熟；若发现单个直径>9 mm的卵泡，则多为囊肿；若卵巢不大而子宫长度>3.5 cm并见内膜增厚则多为外源性雌激素作用。

4．CT或MRI检查 对怀疑颅内肿瘤或肾上腺疾病所致者，应进行头颅或腹部CT或MRI检查。

5．其他检查 根据患儿的临床表现可进一步选择其他检查，如怀疑甲状腺功能低下可测定T_3、T_4、TSH；性腺肿瘤睾酮和雌二醇浓度增高；先天性肾上腺皮质增生症患儿的血17-羟孕酮（17-OHP）、ACTH和脱氢异雄酮（DHEA）明显增高。

【诊断和鉴别诊断】

性早熟的诊断包括3 个步骤，首先要确定是否为性早熟；其次是判断性早熟属于中枢性或外周性；第三是寻找病因。特发性性早熟的诊断过程主要是排除其他原因所致的性早熟，特别是与中枢神经系统、肾上腺、性腺、肝脏的肿瘤鉴别。女孩特发性性早熟，要注意与以下疾病鉴别：

1．单纯乳房早发育 是女孩不完全性性早熟的表现。起病年龄小，常<2 岁，乳腺仅轻度发育，且常呈现周期性变化。这类患儿不伴有生长加速和骨骼发育提前，不伴有阴道流血。血清雌二醇和FSH基础值常轻度增高，GnRH刺激试验中FSH峰值明显增高。由于部分患者可逐步演变为真性性早熟，故此类患儿应注意追踪检查。

2．外周性性早熟 多见于误服含雌激素的药物、食物或接触含雌激素的化妆品，女孩常有不规则阴道出血，且与乳房发育不相称，乳头、乳晕着色加深。女孩单纯出现阴道出血时，应注意排除阴道感染、异物或肿瘤等。对男孩出现性发育征象而睾丸容积仍与其年龄相称者，应考虑先天性肾上腺皮质增生症、肾上腺肿瘤。单侧睾丸增大者需除外性腺肿瘤。

3．McCune-Albright 综合征 多为女性，是由于Gs基因缺陷所致。患儿除性早熟征象外，尚伴有皮肤咖啡色素斑和骨纤维发育不良，偶见卵巢囊肿。少数患儿可能伴有甲状腺功能亢进或Cushing综合征。其性发育过程与特发性性早熟不同，常先有阴道流血，而后方有乳房发育等其他性征出现。

4．原发性甲状腺功能减低伴性早熟 仅见于少数未经治疗的原发性甲状腺功能减低。多见于女孩，其发病机制可能和下丘脑-垂体-性腺轴调节紊乱有关。甲低时，下丘脑分泌TRH增加，由于分泌TSH的细胞与分泌催乳素（PRL）、LH、FSH的细胞具有同源性，TRH不仅促进垂体分泌TSH增多，同时也促进PRL和LH、FSH分泌。临床除甲低症状外，可同时出现性早熟的表现，如女孩出现乳房增大、泌乳和阴道流血等，由于TRH不影响肾上腺皮质功能，故患儿不出现或极少出现阴毛或腋毛发育。给予甲状腺素替代治疗使甲低症状缓解或控制后，性早熟症状也随即消失。

【治疗】

本病治疗依病因而定。中枢性性早熟的治疗目的：①抑制或减慢性发育，特别是阻止女孩月经来潮；②抑制骨骼成熟，改善成人期最终身高；③预防与性发育有关的精神社会问题。

1．病因治疗 肿瘤引起者应手术摘除或进行化疗、放疗；甲状腺功能低下所致者予甲状腺制剂纠正甲状腺功能；先天性肾上腺皮质增生症患者可采用肾上腺皮质激素治疗。

2．药物治疗 促性腺激素释放激素类似物（GnRHa） 天然的GnRH为10 肽，目前常用的几种GnRHa都是将分子中第 6 个氨基酸，即甘氨酸换成 D-色氨酸、D-丝氨酸、D-组氨酸或 D-

亮氨酸而成的长效合成激素。其作用是通过下降调节，抑制垂体-性腺轴，使LH、FSH和性腺激素分泌减少，从而控制性发育，延迟骨骼成熟，最终改善成人期身高。

目前应用的缓释剂主要有曲普瑞林（triptorelin）和亮丙瑞林（leuprorelin），前者为天然GnRH 10 肽的第6 位氨基酸 L-甘氨酸被 D-色氨酸替代，后者则被 D-亮氨酸替代。

国内推荐剂量：每次 80～100 μg/kg，或通常应用每次 3.75 mg，每4 周肌内注射1 次。目前建议GnRHa应用至患者骨龄达11～12 岁。近年对开始 GnRHa 治疗较晚或其预测成年期身高显著低于其遗传靶身高者，或在应用 GnRHa 后生长速率明显减慢者，可同时应用重组人生长激素以改善终身高。

GnRHa 治疗特发性性早熟常见的副作用主要为注射部位局部反应如红斑、硬化、水疱、无菌性水肿以及首次应用可能出现阴道分泌物增多或阴道出血等。

第五节 先天性甲状腺功能减低症

甲状腺功能减低症（hypothyroidism）简称甲低，是由于各种不同的疾病累及下丘脑-垂体-甲状腺轴功能，以致甲状腺素缺乏；或是由于甲状腺素受体缺陷所造成的临床综合征。按病变涉及的位置可分为：①原发性甲低，是由于甲状腺本身疾病所致；②继发性甲低，其病变位于垂体或下丘脑，又称为中枢性甲低，多数与其他下丘脑-垂体轴功能缺陷同时存在。

儿科患者绝大多数为原发性甲低，根据其发病机制的不同和起病年龄又可分为先天性和获得性两类，获得性甲低在儿科主要由慢性淋巴细胞性甲状腺炎，即桥本甲状腺炎（Hashimoto thyroiditis）所引起。本节主要介绍先天性甲低。

先天性甲状腺功能减低症（congenital hypothyroidism），是由于甲状腺激素合成不足所造成的一种疾病。根据病因的不同可分为两类：①散发性：系先天性甲状腺发育不良、异位或甲状腺激素合成途径中酶缺陷所造成，发生率约为1/7000；②地方性：多见于甲状腺肿流行的山区，是由于该地区水、土和食物中碘缺乏所致，随着我国碘化食盐的广泛应用，其发病率明显下降。

【甲状腺激素生理和病理生理】

1．甲状腺激素的合成 甲状腺的主要功能是合成甲状腺素（thyroxine，T_4）和三碘甲腺原氨酸（triiodothyronine，T_3）。血循环中的无机碘被摄取到甲状腺滤泡上皮细胞内，经过甲状腺过氧化物酶的作用氧化为活性碘，再与酪氨酸结合成单碘酪氨酸（MIT）和双碘酪氨酸（DIT），两者再分别偶联缩和成T_3和T_4。这些合成步骤均在甲状腺滤泡上皮细胞合成的甲状腺球蛋白（TG）分子上进行。

2．甲状腺素的释放 甲状腺滤泡上皮细胞通过摄粒作用将TG形成的胶质小滴摄入胞内，由溶酶体吞噬后将TG水解，释放出T_3和T_4。

3．甲状腺素合成和释放的调节 甲状腺素的合成和释放受下丘脑分泌的促甲状腺激素释放激素（TRH）和垂体分泌的促甲状腺激素（TSH）的控制，下丘脑产生TRH，刺激腺垂体，产生TSH，TSH再刺激甲状腺分泌T_3、T_4。而血清T_4则可通过负反馈作用降低垂体对TRH的反应性、减少TSH的分泌。T_3、T_4释放入血循环后，约70％与甲状腺素结合蛋白（TBG）相结合，少量与前白蛋白和白蛋白结合，仅0.03％的T_4和0.3％的T_3为游离状态。正常情况下，T_4的分泌率较T_3高8～10 倍；T_3的代谢活性为T_4的3～4倍；机体所需的T_3约80％在周围组织由T_4转化而成，TSH亦促进这一过程。

4．甲状腺素的主要作用

（1）产热：甲状腺素能加速体内细胞氧化反应的速度，从而释放热量。

（2）促进生长发育及组织分化：甲状腺素促进细胞组织的生长发育和成熟；促进钙磷在

骨质中的合成代谢和骨、软骨的生长。

（3）对代谢的影响：促进蛋白质合成，增加酶的活力；促进糖的吸收、糖原分解和组织对糖的利用；促进脂肪分解和利用。

（4）对中枢神经系统影响：甲状腺素对神经系统的发育及功能调节十分重要。特别在胎儿期和婴儿期，甲状腺素不足会严重影响脑的发育、分化和成熟，且不可逆转。

（5）对维生素代谢的作用：甲状腺素参与各种代谢，使维生素B_1、B_2、B_3、C的需要量增加。同时，促进胡萝卜素转变成维生素A及维生素A 生成视黄醇。

（6）对消化系统影响：甲状腺素分泌过多时，食欲亢进，肠蠕动增加，大便次数多，但性质正常。分泌不足时，常有食欲不振，腹胀、便秘等。

（7）对肌肉的影响：甲状腺素过多时，常可出现肌肉神经应激性增高，出现震颤。

（8）对血液循环系统影响：甲状腺素能增强β-肾上腺素能受体对儿茶酚胺的敏感性，故甲亢患者出现心跳加速、心排出量增加等。

【病因】

1．散发性先天性甲低（sporadic congenital hypothyroidism）

（1）甲状腺不发育、发育不全或异位：是造成先天性甲低最主要的原因，约占90％。多见于女孩，女：男为2：1。其中1/3 病例为甲状腺完全缺如，其余为发育不全或在下移过程中停留在异常部位形成异位甲状腺，部分或完全丧失其功能。造成甲状腺发育异常的原因尚未阐明，可能与遗传素质与免疫介导机制有关。

（2）甲状腺激素（thyroid hormone）合成障碍：是导致先天性甲状腺功能低下的第2 位常见原因。多见于甲状腺激素合成和分泌过程中酶（过氧化物酶、耦联酶、脱碘酶及甲状腺球蛋白合成酶等）的缺陷，造成甲状腺素不足。多为常染色体隐性遗传病。

（3）TSH、TRH缺乏：亦称下丘脑-垂体性甲低或中枢性甲低。是因垂体分泌TSH障碍而引起的，常见于特发性垂体功能低下或下丘脑、垂体发育缺陷，其中因下丘脑TRH不足所致者较多见。TSH单一缺乏者甚为少见，常与GH、催乳素（PRL）、黄体生成素（LH）等其他垂体激素缺乏并存，是由于位于3p11 的Pit-1 基因突变所引起，临床上称为多垂体激素缺乏综合征（MPHD）。

（4）甲状腺或靶器官反应低下：前者是由于甲状腺细胞质膜上的 GSα 蛋白缺陷，使cAMP生成障碍，而对TSH无反应；后者是末梢组织 β-甲状腺受体缺陷，从而对T_3、T_4不反应。均为罕见病。

（5）母亲因素：母亲服用抗甲状腺药物或母亲患自身免疫性疾病，存在抗TSH受体抗体，均可通过胎盘而影响胎儿，造成甲低，亦称暂时性甲低，通常在3 个月后好转。

2．地方性先天性甲低（endemic congenital hypothyroidism） 多因孕妇饮食缺碘，致使胎儿在胚胎期即因碘缺乏而导致甲状腺功能低下。

【临床表现】

甲状腺功能减低症的症状出现的早晚及轻重程度与残留甲状腺组织的多少及甲状腺功能低下的程度有关。先天性无甲状腺或酶缺陷患儿在婴儿早期即可出现症状，甲状腺发育不良者常在生后3～6 个月时出现症状，亦偶有数年之后才出现症状。患儿的主要临床特征包括智能落后、生长发育迟缓和生理功能低下。

1．新生儿期 患儿常为过期产，出生体重常大于第90 百分位，身长和头围可正常，前、后囟大；胎便排出延迟，生后常有腹胀，便秘，脐疝，易被误诊为先天性巨结肠；生理性黄疸期延长；患儿常处于睡眠状态，对外界反应低下，肌张力低，吮奶差，呼吸慢，哭声低且少，

体温低，（常<35℃），四肢冷，末梢循环差，皮肤出现斑纹或有硬肿现象等。以上症状和体征均无特异性，极易误诊为其他疾病。

2．典型症状 多数先天性甲状腺功能减低症患儿常在出生半年后出现典型症状：

（1）特殊面容和体态：头大，颈短，皮肤粗糙、面色苍黄，毛发稀疏、无光泽，面部黏液水肿，眼睑浮肿，眼距宽，鼻梁低平，唇厚，舌大而宽厚、常伸出口外。患儿身材矮小，躯干长而四肢短小，上部量/下部量>1.5，腹部膨隆，常有脐疝。

（2）神经系统症状：智能发育低下，表情呆板、淡漠，神经反射迟钝；运动发育障碍，如翻身、坐、立、走的时间都延迟。

（3）生理功能低下：精神差，安静少动，对周围事物反应少，嗜睡，食欲不振，声音低哑，体温低而怕冷，脉搏、呼吸缓慢，心音低钝，肌张力低，肠蠕动慢，腹胀，便秘。可伴心包积液，心电图呈低电压、P-R间期延长、T波平坦等改变。

3．地方性甲状腺功能减低症 因在胎儿期碘缺乏而不能合成足量甲状腺激素，影响中枢神经系统发育。临床表现为两种不同的类型，但可相互交叉重叠：

（1）“神经性”综合征：主要表现为：共济失调、痉挛性瘫痪、聋哑、智能低下，但身材正常，甲状腺功能正常或轻度减低。

（2）“黏液水肿性”综合征：临床上有显著的生长发育和性发育落后、智力低下、黏液性水肿等。血清T_4降低、TSH增高。约25％患儿有甲状腺肿大。

4．TSH和TRH分泌不足 患儿常保留部分甲状腺激素分泌功能，因此临床症状较轻，但常有其他垂体激素缺乏的症状如低血糖（ACTH缺乏）、小阴茎（Gn缺乏）、尿崩症（AVP缺乏）等。

【实验室检查】

由于先天性甲低发病率高，在生命早期对神经系统功能损害严重，且其治疗容易、疗效佳，因此早期诊断、早期治疗至为重要。

1．新生儿筛查 我国1995 年6 月颁布的“母婴保健法”已将本病列入筛查的疾病之一。目前多采用出生后2～3 天的新生儿干血滴纸片检测TSH浓度作为初筛，结果大于15～20 mU/L（须根据所筛查实验室阳性切割值决定）时，再检测血清T_4、TSH以确诊。该法采集标本简便，假阳性和假阴性率较低，故为患儿早期确诊、避免神经精神发育严重缺陷、减轻家庭和社会负担的重要防治措施。

2．血清T_4、T_3、TSH 测定 任何新生儿筛查结果可疑或临床可疑的小儿都应检测血清T_4、TSH 浓度，如T_4降低、TSH明显升高即可确诊。血清T_3浓度可降低或正常。

3．TRH 刺激试验 若血清T_4、TSH均低，则疑TRH、TSH分泌不足，应进一步做TRH刺激试验：静注TRH 7 μg/kg，正常者在注射20～30 分钟内出现TSH峰值，90 分钟后回至基础值。若未出现高峰，应考虑垂体病变；若TSH峰值甚高或出现时间延长，则提示下丘脑病变。随着超敏感的第三代增强化学发光法TSH检测技术的应用，一般不需再进行TRH刺激试验。

4．X线检查患儿骨龄常明显落后于实际年龄。

5．核素检查 采用静脉注射^{99m}Tc后以单光子发射计算机体层摄影术（SPECT）检测患儿甲状腺发育情况及甲状腺的大小、形状和位置。

【诊断和鉴别诊断】

根据典型的临床症状和甲状腺功能测定，诊断不甚困难。但在新生儿期不易确诊，应对新生儿进行群体筛查。年长儿应与下列疾病鉴别：

1．先天性巨结肠 患儿出生后即开始便秘、腹胀，并常有脐疝，但其面容、精神反应及哭

声等均正常，钡灌肠可见结肠痉挛段与扩张段。

2．21-三体综合征 患儿智能及动作发育落后，但有特殊面容：眼距宽、外眼眦上斜、鼻梁低、舌伸出口外，皮肤及毛发正常，无黏液性水肿，常伴有其他先天畸形。染色体核型分析可鉴别。

3．佝偻病 患儿有动作发育迟缓、生长落后等表现。但智能正常，皮肤正常，有佝偻病的体征，血生化和X线片可鉴别。

4．骨骼发育障碍的疾病 如骨软骨发育不良、黏多糖病等都有生长迟缓症状，骨骼X线片和尿中代谢物检查可资鉴别。

【治疗】

本病应早期确诊，尽早治疗，以减小对脑发育的损害。一旦诊断确立，应终身服用甲状腺制剂，不能中断，否则前功尽弃。饮食中应富含蛋白质、维生素及矿物质。

常用甲状腺制剂有两种，①L-甲状腺素钠：100μg/片-或50 μg/片，含T_4，半衰期为1 周，因T_4浓度每日仅有小量变动，血清浓度较稳定，故每日服一次即可。一般起始剂量为每日8～14 μg/ kg，大剂量为每日10～15 μg/kg。替代治疗参考剂量见表17-3；②干甲状腺片：40 mg/片，是从动物甲状腺组织中提取，含T_3、T_4，若长期服用，可使T_3升高该制剂临床上已基本不用。

表17-3 甲状腺片治疗甲低的参考剂量

年　龄	μg/d	μg/（kg•d）
0～6 个月	25～50	8～10
6～12 个月	50～100	5～8
1～5 岁	75～100	5～6
6～12 岁	100～150	4～5
12 岁到成人	100～200	2～3

用药量可根据甲状腺功能及临床表现进行适当调整，应使①TSH浓度正常，血T_4正常或偏高值，以备部分T_4 转变成T_3。新生儿甲低应在开始治疗2～4 周内使血清T_4水平上升至正常高限，6～9 周内使血清TSH水平降至正常范围。②临床表现：大便次数及性状正常，食欲好转，腹胀消失，心率维持在正常范围，智能及体格发育改善。药物过量可出现烦躁、多汗、消瘦、腹痛、腹泻、发热等。因此，在治疗过程中应注意随访，治疗开始时每2 周随访1 次；血清TSH和T_4正常后，每3 个月1 次；服药1～2 年后，每6 个月1 次。在随访过程中根据血清T_4、TSH水平，及时调整剂量，并注意监测智能和体格发育情况。

【预后】

新生儿筛查阳性者确诊后应即开始正规治疗，预后良好。如果出生后3个月内开始治疗，预后尚可，智能绝大多数可达到正常；如果未能及早诊断而在6 个月后才开始治疗，虽然给予甲状腺素可以改善生长状况，但是智能仍会受到严重损害。

第六节 先天性肾上腺皮质增生症

先天性肾上腺皮质增生症（congenital adrenal hyperplasia，CAH）是一组由于肾上腺皮质激素合成过程中酶的缺陷所引起的疾病，属常染色体隐性遗传病。新生儿中的发病率为1/16 000～1/20 000。

【病因和病理生理】

肾上腺皮质由球状带、束状带、网状带组成。球状带位于最外层，约占皮质的5%～10%，

是盐皮质激素——醛固酮的唯一来源；束状带位于中间层，是最大的皮质带，约占75%， 是皮质醇和少量盐皮质激素（脱氧皮质酮、脱氧皮质醇、皮质酮）的合成场所；网状带位于最内层，主要合成肾上腺雄激素和少量雌激素。正常肾上腺以胆固醇为原料合成糖皮质激素、盐皮质激素、性激素（雄、雌激素和孕激素）3 类主要激素，其过程极为复杂，图17-1 为简化的合成途径，每一步骤都需经特殊的酶催化，有些酶是合成这3 类激素或其中两类激素的过程中所共同需要的。类固醇生成急性调节蛋白（steroidogenic acute regulatory protein，StAR）是类固醇激素合成过程中的重要调节因子，具有高度的组织特异性，其作用是将胆固醇从线粒体外膜转运到内膜，此过程是类固醇激素合成的限速步骤，StAR基因突变导致先天性肾上腺皮质脂质增生。表17-4概括了类固醇激素合成所需的酶，其中除3β-羟类固醇脱氢酶（3β-HSD）外，均为细胞色素P450（cytochrome P450）蛋白超家族成员。肾上腺合成皮质醇受垂体分泌的ACTH调控。先天性肾上腺皮质增生症时，由于上述激素合成过程中有不同部位的酶缺陷致使糖皮质激素、盐皮质激素合成不足，而在缺陷部位以前的各种中间产物在体内堆积。由于血皮质醇水平降低，其负反馈作用消除，致使腺垂体ACTH分泌增多，刺激肾上腺皮质增生，并使雄激素和一些中间代谢产物增多，由于醛固酮合成和分泌在常见类型的CAH中亦大多同时受到影响，故常导致血浆肾素（PRA）活性增高，从而产生各种临床症状。主要的酶缺陷有：21-羟化酶（CYP21）、11β-羟化酶（CYP11B1）、17-羟化酶（CYP17）、3β-羟类固醇脱氢酶（3β-HSD）和18-羟化酶（CYP11B2），其中以21-羟化酶缺乏最常见。

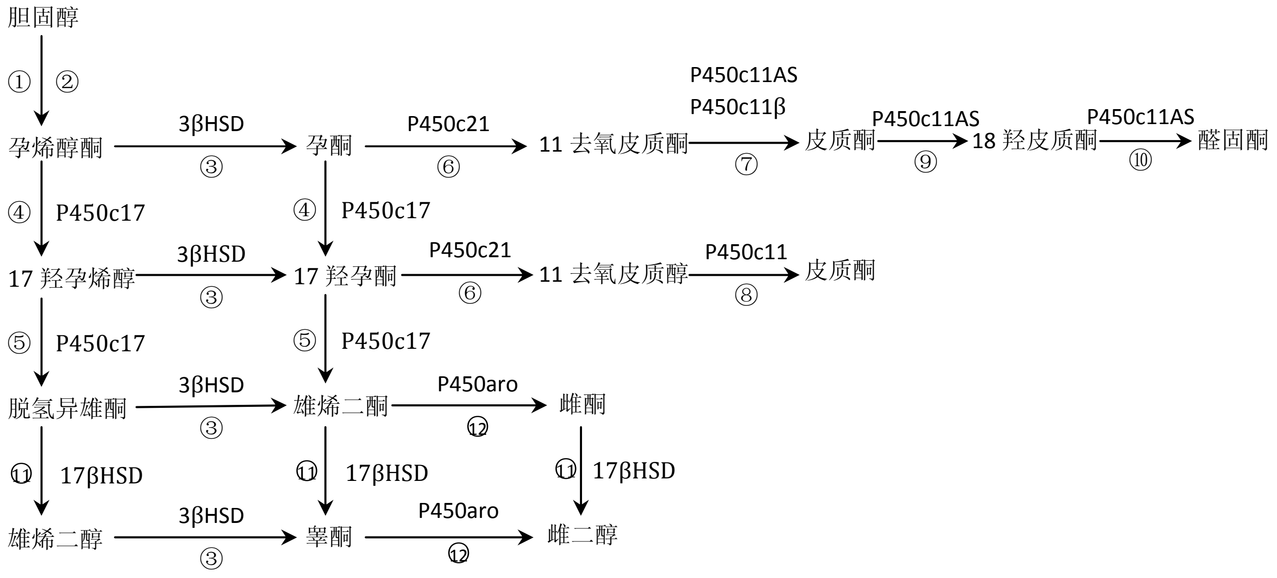

图17-1 类固醇激素生物合成途径

注：①类固醇生成急性调节蛋白（StAR) ②P450scc：胆固醇侧链裂解酶（CYP11A）

③3β-羟类固醇脱氢酶（3β-HSD） ④17-a 羟化酶（CYP17）

⑤17，20-碳裂解酶（CYP17） ⑥21-羟化酶（CYP21）

⑦11β-羟化酶（CYP11B2） ⑧11β-羟化酶（CYP11B1）

⑨18-羟化酶（CYP11B2） ⑨18-氧化酶（CYP11B2）

⑪17β-羟类固醇脱氢酶（17β-HSD） ⑫P450 芳香化酶

【临床表现】

本症以女孩多见，男女之比约为1：2。本病的临床表现取决于酶缺陷的部位及缺陷的严重程度。常见的有以下几种类型（表17-5)。

1．21-羟化酶缺乏症（21-hydroxylase deficiency，21-OHD） 是先天性肾上腺皮质增生症

中最常见的一种，占典型病例的90％～95％，21-羟化酶基因定位于第6 号染色体短臂（6p21.3），与HLA基因族紧密连锁，由A基因（CYP 21A）和B基因（CYP 21B）两个基因座构成。CYP 21B又称CYP 21，是21-羟化酶的编码基因；CYP 21A 又称CYP21p，是无功能的假基因。CYP21 基因突变，包括点突变、缺失和基因转换等，致使21-羟化酶部分或完全缺乏。由于皮质醇合成分泌不足，雄激素合成过多，致使临床出现轻重不等的症状。可表现为单纯男性化型、失盐型、非典型型3 种类型。

表17-4 参与肾上腺类固醇激素合成的酶

基 因	定 位	酶/蛋白	作 用
StAR	8p11.2	类固醇生成急性调节蛋白	将胆固醇从线粒体外膜转运至内膜
CYP11A	15q23-q24	P450scc	20α-羟化
			22α-羟化
			20-22裂解
HSD3B2	1p13.1	3β-HSD	3β-羟类固醇脱氢
			⊿5-⊿4类固醇异构
CYP 17	10q24.3	P450c17	17α-羟化
			17-20裂解
CYP 21	6p21.3	P450c21	21α-羟化
CYP 11B1	8q21	P450c11β	11β-羟化
CYP 11B2	8q21	P450c11AS	11β-羟化
			18-羟化
			18-氧化
HSD17B1	17q12-q21	17β-HSD	17β-羟类固醇脱氢
			17-酮类固醇还原
CYP 19	15q21.1	P-450arom	类固醇A环芳香化

表17-5 各种类型CAH临床特征

酶 缺 陷		盐 代 谢	临 床 类 型
21-羟化酶	失盐型	失盐	男性假性性早熟，女性假两性畸形
	单纯男性化型	正常	同上
11β-羟化酶		高血压	同上
17-羟化酶		高血压	男性假两性畸形，女性性幼稚
3β-羟类固醇脱氢酶		失盐	男性、女性假两性畸形
类脂性肾上腺皮质增生		失盐	男性假两性畸形，女性性幼稚
18-羟化酶		失盐	男、女性发育正常

（1）单纯男性化型（simple virilizing，SV）：系21-羟化酶不完全缺乏所致，酶缺乏呈中等程度，11-脱氧皮质醇和皮质醇、11-去氧皮质酮等不能正常合成，其前体物质17-羟孕酮、孕酮、脱氢异雄酮增多，但由于患儿仍有残存的21-羟化酶活力，可合成少量皮质醇和醛固酮，故临床无失盐症状，主要表现为雄激素增高的症状和体征。

女孩表现为假两性畸形。由于类固醇激素合成缺陷在胎儿期即存在，故女孩在出生时即呈现程度不同的男性化体征，如阴蒂肥大，类似男性的尿道下裂；大阴唇似男孩的阴囊，但无睾丸；或有不同程度的阴唇融合。虽然外生殖器有两性畸形，但内生殖器仍为女性型，有卵巢、输卵管、子宫。患儿在2～3 岁后可出现阴毛、腋毛。于青春期，女性性征缺乏，无乳房发育和月经来潮。

男孩表现为假性性早熟。出生时可无症状，生后6 个月以后出现性早熟征象，一般1～2 岁后外生殖器明显增大，阴囊增大，但睾丸大小与年龄相称。可早期出现阴毛、腋毛、胡须、痤疮、喉结，声音低沉和肌肉发达。无论男孩还是女孩均出现体格发育过快，骨龄超出年龄，因骨骼融合过早，其最终身材矮小。由于ACTH增高，可有皮肤黏膜色素沉着。一般缺陷愈严重，色素增加愈明显，以皮肤皱褶处为明显，如腹股沟、乳晕周围、腋窝、手指关节伸面等，新生儿多表现在乳晕和外生殖器。

（2）失盐型（salt wasting，SW）：是21-羟化酶完全缺乏所致。皮质醇的前体物质如孕酮、17-羟孕酮等分泌增多，而皮质醇、醛固酮合成减少，使远端肾小管排钠过多，排钾过少。因此，患儿除具有上述男性化表现外，生后不久即可有拒食、呕吐、腹泻、体重不增或下降、脱水、低血钠、高血钾、代谢性酸中毒等。若治疗不及时，可因循环衰竭而死亡。女性患儿出生时已有两性畸形，易于诊断，男性患儿诊断较为困难，常误诊为幽门狭窄而手术，或误诊为婴儿腹泻而耽误治疗。

（3）非典型型（nonclassic，NC）：亦称迟发型、隐匿型或轻型，是由于21-羟化酶轻微缺乏所致。本症的临床表现各异，发病年龄不一。在儿童期或青春期才出现男性化表现。男孩为阴毛早现、性早熟、生长加速、骨龄提前；女性患儿可出现初潮延迟、原发性闭经、多毛症及不育症等。

2．11β-羟化酶缺陷症（11β-hydroxylase deficiency，11β-OHD） 约占本病的5%～8%，此酶缺乏时，雄激素和11-脱氧皮质酮均增多。临床表现出与21-羟化酶缺乏相似的男性化症状，但程度较轻；可有高血压和钠潴留。多数患儿血压中等程度增高，其特点是给予糖皮质激素后血压可下降，而停药后血压又回升。

3．3β-羟类固醇脱氢酶缺乏症（3β-hydroxysteroid dehydrogenase deficiency，3β-HSD）本型较罕见，是由于3β-HSDⅡ基因突变所致。该酶缺乏时，醛固酮、皮质醇、睾丸酮的合成均受阻，男孩出现假两性畸形，如阴茎发育差、尿道下裂。女孩出生时出现阴蒂肥大、轻度男性化现象。由于醛固酮分泌低下，在新生儿期即发生失盐、脱水症状，病情较重。

4．17α-羟化酶缺乏症（17α-hydroxylase deficiency，17-OHD） 本型亦罕见，由于皮质醇和性激素合成受阻，而11-去氧皮质酮和皮质酮分泌增加，临床出现低钾性碱中毒和高血压，由于缺乏性激素，女孩可有幼稚型性征、原发性闭经等；男孩则表现为男性假两性畸形，外生殖器女性化，有乳房发育，但患儿有睾丸。

【实验室检查】

1．生化检测（见表17-6）

表17-6 各种类型CAH实验检查

酶缺陷	血液								尿液		
	Na	K	PRA	Aldo	17-OHP	DHEA	DOC	T	17-OHCS	17-KS	孕三醇
21-羟化酶 失盐型	↓	↑	↑↑	↓↓	↑↑	N↑	N↓	↑↑	↓	↑↑	↑↑
单纯男性化型	N	N	↑	N↓	↑↑	N↑	N↓	↑↑	↓	↑↑	↑↑
11β羟化酶	↑	↓	↓	↓	↑	N↑	↑↑	↑	↑	↑↑	↑
17-羟化酶	↑	↓	↓	N↓	↓	↓↓	↑↑	↓	↓	↓	↓
3β-羟类固醇脱氢酶	↓	↑	↑	↓	N↑	↑	N↓	↓	↓	↑	N↑
类脂性肾上腺皮质增生	↓	↑	↑	↓	↓	↓	↓	↓	↓	↑	↓
18-羟化酶	↓	↑	↑	↓	N	N	N	N	N	N	N

（1）尿液17-羟类固醇（17-OHCS）、17-酮类固醇（17-KS）和孕三醇测定。其中17-KS是反映肾上腺皮质分泌雄激素的重要指标，对本病的诊断价值优于17-OHCS。肾上腺皮质增生症患者17-KS明显升高。

（2）血液17-羟孕酮（17-OHP）、肾素血管紧张素原（PRA）、醛固酮（Aldo）、脱氢异雄酮（DHEA）、去氧皮质酮（DOC）及睾酮（T）等的测定。血17-OHP、孕酮、DHEA及T均可增高，其中17-OHP增高可为正常的几十倍至几百倍，是21-OHD较可靠的诊断依据。

（3）血电解质测定：失盐型可有低钠、高钾血症。

（4）血皮质醇、ACTH测定：典型失盐型CAH患者的皮质醇水平低于正常，单纯男性化型可在正常范围或稍低于正常。血ACTH不同程度升高，部分患儿尤其是非典型者可正常。

2．其他检查

（1）染色体检查：外生殖器严重畸形时，可进行染色体分析，以鉴定性别。

（2）X线检查：拍摄左手腕掌指骨正位片，判断骨龄。患者骨龄常超过年龄。

（3）CT或MRI检查：可发现双侧肾上腺增大。

（4）基因诊断：采用直接聚合酶链反应、寡核苷酸杂交、限制性内切酶片段长度多态性和基因序列分析可发现相关基因突变或缺失。

【诊断和鉴别诊断】

典型单纯男性化型患者无失盐及明显的糖皮质激素缺乏的症状，仅可见雄激素增高的症状，如多毛、阴毛早现、声音变粗、男孩阴茎粗大和女孩外生殖器男性化等；典型失盐型患儿在新生儿期即出现呕吐、腹泻、脱水和难以纠正的低血钠、高血钾和代谢性酸中毒，严重者出现循环衰竭等危象；无论男女均有生长加速，骨龄超前。非典型者在儿童早期无明显临床症状，以后往往因多毛、痤疮、月经过少、闭经和生育能力障碍等而就诊。

本病如能早期诊断、早期治疗，可维持患儿的正常发育和生活，因此早期确诊极为重要，并需与其他相关疾病鉴别：

1．失盐型易误诊为先天性肥厚性幽门狭窄或肠炎，故如遇新生儿反复呕吐、腹泻，应注意家族史、生殖器外形等，必要时进行有关生化检查。先天性肥厚性幽门狭窄症表现为特征性的喷射性呕吐，钡剂造影可发现狭窄的幽门，无皮质色素沉着，外生殖器正常。

2．单纯男性化型应与真性性早熟、男性化肾上腺肿瘤相鉴别，单纯男性化型睾丸大小与实际年龄相称，17-酮明显升高；而真性性早熟睾丸明显增大，17-酮增高，但不超过成人期水平。男性化肾上腺肿瘤和单纯男性化型均有男性化表现，尿17-酮均升高，需进行地塞米松抑制试验，男性化肾上腺肿瘤不被抑制，而单纯男性化型对较小剂量地塞米松即可显著抑制。

【治疗】

治疗本病的目的：①替代肾上腺分泌类固醇的不足，补充生理需要的糖、盐皮质激素，维持机体正常的生理代谢；②抑制ACTH的分泌，从而减少肾上腺雄激素的过度分泌，抑制男性化，阻止骨骺成熟加速，促进正常的生长发育。

1．对失盐型患儿应及时纠正水、电解质紊乱，静脉补液可用生理盐水，有代谢性酸中毒时则用0.45%氯化钠和碳酸氢钠溶液。忌用含钾溶液。重症失盐型需静脉滴注氢化可的松25～100 mg；若低钠和脱水不易纠正，可口服氟氢可的松(9α-fludrocortisone acetate)　0.05～0.1 mg/d。脱水纠正后，糖皮质激素改为口服；并长期维持，同时口服氯化钠2～4 g/d。其量可根据病情适当调整。

2．长期治疗

（1）糖皮质激素：糖皮质激素治疗一方面可补偿肾上腺分泌皮质醇的不足，一方面可抑

制过多的ACTH 释放，从而减少雄激素的过度产生，故可改善男性化、性早熟等症状，保证患儿正常的生长发育过程。诊断确立后应尽早给予治疗，一般氢化可的松口服量为每日10～20 mg/m^2，分2～3 次口服。

治疗过程中应根据血压、身高增长速率、雄烯二酮、DHEA、DHEAS、睾酮以及骨成熟度、尿17-酮类固醇等指标综合分析调整糖皮质激素的剂量。如应用糖皮质激素的剂量过大，则影响生长；如剂量不足，则不能抑制肾上腺雄激素继续过量产生，雄激素会促使骨骺过早成熟和关闭，同样对患儿生长造成影响，并产生其他一些雄激素过多的表现。一般不用17-OHP 作为治疗监测的指标，因为其每日变化较大，且易受应激影响。

（2）盐皮质激素：盐皮质激素可协同糖皮质激素的作用，使ACTH 的分泌进一步减少。可口服氟氢可的松0.05～0.1 mg/d，症状改善后，逐渐减量、停药。因长期应用可引起高血压。0.1 mg 氟氢可的松相当于1.5 mg 氢化可的松，应将其量计算于皮质醇的用量中，以免皮质醇过量。

在皮质激素治疗的过程中，对失盐型患儿还应监测血钾、钠、氯等，调节激素用量。患儿在应激情况下（如感染、过度劳累、手术等）或青春期，糖皮质激素的剂量应比平时增加1.5～2 倍。

3．手术治疗 男性患儿勿需手术治疗。女性假两性畸形患儿宜在6 个月～1 岁行阴蒂部分切除术或矫形术。

【预防】

1．新生儿筛查 应用干血滴纸片法，对生后2～5 天的婴儿采集足跟血样检测17-OHP 浓度可进行早期诊断。正常婴儿刚出生时血17-OHP 水平较高，12～24 小时后降至正常。低体重儿和患某些心肺疾病时17-OHP 也会上升，需注意鉴别。

2．产前诊断

（1）2l-OHD：在孕9～11 周取绒毛膜活检进行胎儿细胞DNA分析；孕16～20 周取羊水检测孕三醇、17-OHP 等。因大部分非典型21-OHD 患儿生后17-OHP 水平无明显升高，因此基因检测是此型患儿唯一早期诊断手段。

（2）11β-OHD：可检测羊水DOC 或取绒毛膜作相关基因分析进行诊断。

第七节 儿童糖尿病

糖尿病（diabetes mellitus，DM）是由于胰岛素缺乏所造成的糖、脂肪、蛋白质代谢紊乱症，分为原发性和继发性两类。原发性糖尿病又可分为：① 1 型糖尿病：以胰岛β细胞破坏，胰岛素分泌绝对不足所造成，必须使用胰岛素治疗，故又称胰岛素依赖性糖尿病（insulin dependent diabetes mellitus，IDDM）；② 2 型糖尿病：由于胰岛β细胞分泌胰岛素不足或靶细胞对胰岛素不敏感（胰岛素抵抗）所致，亦称非胰岛素依赖性糖尿病（noninsulin-dependent diabetes mellitus，NIDDM）；③ 青年成熟期发病型（maturity-onset diabetes of youth，MODY）：是一种罕见的遗传性β细胞功能缺陷症，属常染色体显性遗传。继发性糖尿病大多由一些遗传综合征（如21-三体、Turner 综合征和 Klinefelter 综合征等）和内分泌疾病（如 Cushing 综合征、甲状腺功能亢进症等）所引起。98％的儿童糖尿病为 1 型糖尿病，2 型糖尿病甚少，但随儿童肥胖症的增多而有增加趋势。

儿童 1 型糖尿病的发病率在各国之间差异较大，即使同一国家，不同民族之间也不相同。芬兰（发病率 36/10万）、意大利的撒丁岛（发病率 36.4/10 万）是儿童（<0～14 岁）1 型糖

尿病发病率最高者；其次为加拿大、瑞典、丹麦、美国和英国；韩国、日本及中国属低发病区，我国年发病率为 1.04/10 万。近年的流行病学研究表明，发病率逐年增高是世界的总趋势。4～6 岁和 10～14 岁为 1 型糖尿病的高发年龄，1 岁以下小儿发病较少见。本节主要叙述 1 型糖尿病。

【病因和发病机制】

1 型糖尿病确切病因机制尚未完全阐明。目前认为是在遗传易感性基因的基础上由外界环境因素的作用下引起自身免疫反应导致了胰岛β细胞的损伤和破坏，当胰岛素分泌减少至正常的10％时即出现临床症状。

1．遗传易感性 根据同卵双胎的研究， 1 型糖尿病的患病一致性为50％，说明本病因除遗传因素外还有环境因素作用，属多基因遗传病。通过对人类白细胞抗原（HLA）的研究发现，HLA的D区Ⅱ类抗原基因（位于6p21.3）与本病的发生有关，已证明与HLA-DR3和DR4的关联性特别显著。还有研究认为HLA-DQβ链上第 57 位非门冬氨酸及HLA-DQα链上第 52 位的精氨酸的存在决定 1 型糖尿病的易感性；反之HLA-DQβ57 位门冬氨酸和HLA-DQα52 位非精氨酸决定了 1 型糖尿病的保护性。但遗传易感基因在不同种族间有一定的差别，提示与遗传多态性有关。

2．环境因素 1 型糖尿病的发病与病毒感染（如风疹病毒、腮腺炎病毒、柯萨奇病毒等）、化学毒物（如链尿菌素、四氧嘧啶等）、食物中的某些成分（如牛乳蛋白中的α、β-酪蛋白、乳球蛋白等）有关，以上因素可能会激发易感性基因者体内免疫功能的变化，产生β细胞毒性作用，最后导致发生 1 型糖尿病。

3．自身免疫因素 约90％的 1 型糖尿病患者在初次诊断时血中出现胰岛细胞自身抗体（ICA）、胰岛β细胞膜抗体（ICSA）、胰岛素自身抗体（IAA）以及谷氨酸脱羧酶（GAD）自身抗体、胰岛素受体自身抗体（IRA）等多种抗体，并已证实这些抗体在补体和T淋巴细胞的协同作用下具有对胰岛细胞的毒性作用。新近证实细胞免疫异常对 1 型糖尿病的发病起作重要作用，树突状细胞源性细胞因子白细胞介素-12会促进初始型$CD4^+$ T 细胞（TH_0）向Ⅰ型辅助性 T（TH_1）细胞转化，使其过度活化而产生 TH_1 细胞类细胞因子，引起大量炎症介质的释放，进而损伤胰岛β细胞。

【病理生理】

胰岛β细胞大都被破坏，分泌胰岛素明显减少而分泌胰高糖素的细胞和其他细胞则相对增生即引起代谢紊乱。人体有 6 种涉及能量代谢的激素：胰岛素、胰高糖素、肾上腺素、去甲肾上腺素、皮质醇和生长激素。其中唯有胰岛素是促进能量储存的激素，其余 5 种激素在饥饿状态下均可促进能量释放，称为反调节激素。正常情况下，胰岛素可促进细胞内葡萄糖的转运，促进糖的利用和蛋白质的合成，促进脂肪合成，抑制肝糖原和脂肪的分解。糖尿病患儿的胰岛素分泌不足或缺如，使葡萄糖的利用减少，而反调节激素如胰高糖素、生长激素、皮质醇等增高，且又促进肝糖原分解和葡萄糖异生作用，使脂肪和蛋白质分解加速，造成血糖和细胞外液渗透压增高，细胞内液向细胞外转移。当血糖浓度超过肾阈值（10 mmol/L或180 mg/dl）时即产生糖尿。自尿中排出的葡萄糖可达到200～300 g/d，导致渗透性利尿，临床出现多尿症状，每日约丢失水分3～5 L，钠和钾200～400 mmol，因而造成严重的电解质失衡和慢性脱水。由于机体的代偿，患儿呈现渴感增强、饮水增多；因为组织不能利用葡萄糖，能量不足而产生饥饿感，引起多食。胰岛素不足和反调节激素增高促进了脂肪分解，使血中脂肪酸增高，肌肉和胰岛素依赖性组织即利用这类游离脂肪酸供能以弥补细胞内葡萄糖不足，而过多的游离脂肪酸进入肝脏后，则在胰高糖素等生酮激素的作用下加速氧化，导致乙酰辅酶A增加，超过了三

羧酸循环的氧化代谢能力，致使乙酰乙酸、β-羟丁酸和丙酮酸等酮体长期在体液中累积，形成酮症酸中毒。

酮症酸中毒时氧利用减低，大脑功能受损。酸中毒时CO_2严重潴留，为了排除较多的CO_2，呼吸中枢兴奋而出现不规则的呼吸深快，呼气中的丙酮产生特异的气味（腐烂水果味）。

【临床表现】

1 型糖尿病患者起病较急骤，多有感染或饮食不当等诱因。其典型症状为多饮、多尿、多食和体重下降（即“三多一少”）。但婴儿多饮多尿不易被发觉，很快即可发生脱水和酮症酸中毒。儿童因为夜尿增多可发生遗尿。年长儿还可出现消瘦、精神不振、倦怠乏力等体质显著下降症状。约40％糖尿病患儿在就诊时即处于酮症酸中毒状态，这类患儿常因急性感染、过食、诊断延误、突然中断胰岛素治疗等因素诱发，多表现为起病急，进食减少，恶心，呕吐，腹痛，关节或肌肉疼痛，皮肤黏膜干燥，呼吸深长，呼气中带有酮味，脉搏细速，血压下降，体温不升，甚至嗜睡，淡漠，昏迷。常被误诊为肺炎、败血症、急腹症或脑膜炎等。少数患儿起病缓慢，以精神呆滞、软弱、体重下降等为主。

体格检查时除见体重减轻、消瘦外，一般无阳性体征。酮症酸中毒时可出现呼吸深长，带有酮味，有脱水征和神志的改变。病程较久，对糖尿病控制不良时可发生生长落后、智能发育迟缓、肝大，称为Mauriac综合征。晚期可出现蛋白尿、高血压等糖尿病肾病表现，最后致肾衰竭，还可出现白内障、视力障碍、视网膜病变，甚至双目失明。

儿童糖尿病有特殊的自然病程：

1．急性代谢紊乱期 从出现症状到临床确诊，时间多在 1 个月以内。约20％患儿表现为糖尿病酮症酸中毒；20％～40％为糖尿病酮症，无酸中毒；其余仅为高血糖、糖尿和酮尿。

2．暂时缓解期 约75％的患儿经胰岛素治疗后，临床症状消失、血糖下降、尿糖减少或转阴，即进入缓解期。此时胰岛β细胞恢复分泌少量胰岛素，对外源性胰岛素需要量减至0.5 U/（kg·d）以下，少数患儿甚至可以完全不用胰岛素。这种暂时缓解期一般持续数周，最长可达半年以上。此期应定期监测血糖、尿糖水平。

3．强化期 经过缓解期后，患儿出现血糖增高和尿糖不易控制的现象，胰岛素用量逐渐或突然增多，称为强化期。在青春发育期，由于性激素增多等变化，增强了对胰岛素的拮抗，因此该期病情不甚稳定，胰岛素用量较大。

4．永久糖尿病期 青春期后，病情逐渐稳定，胰岛素用量比较恒定，称为永久糖尿病

【实验室检查】

1．尿液检查

（1）尿糖：尿糖定性一般阳性。在用胰岛素治疗过程中，应监测尿糖变化，以判断饮食及胰岛素用量是否恰当。一般在治疗开始时分段收集晨 8 时至午餐前；午餐后至晚餐前；晚餐后至次晨 8 时的尿液，以了解 24 小时尿糖的变动情况。餐前 30 分钟排空膀胱，再留尿检查尿糖，所得结果可粗略估计当时的血糖水平，更利于胰岛素剂量的调整。

（2）尿酮体：糖尿病伴有酮症酸中毒时呈阳性。

（3）尿蛋白：监测尿微量白蛋白，可及时了解肾脏的病变情况。

2．血液检查

（1）血糖：美国糖尿病学会2005 年公布糖尿病诊断的新标准，符合下列任一标准即可诊断为糖尿病：

1）在典型糖尿病症状并且餐后任意时刻血糖水平≥11.1mmol/L。

2）空腹全血（IFG）≥7.0 mmol/L。

3） 2 小时口服葡萄糖耐量试验（OGTT）血糖水平≥11.1mmol/L。

空腹血糖受损（IFG）：FPG为 5.6～6.9 mmol/L。糖耐量受损（IGT）：口服 75 g葡萄糖后2小时血糖在7.8～11.0 mmol/L。IFG和FPG被称为“糖尿病前期”。

（2）血脂：血清胆固醇、三酸甘油酯和游离脂肪酸明显增加，适当的治疗可使之降低，故定期检测血脂水平，有助于判断病情控制情况。

（3）血气分析：酮症酸中毒在 1 型糖尿病患儿中发生率极高，当血气分析显示患儿血pH<7.30，HCO_3^-<15 mmol/L时，即有代谢性酸中毒存在。

（4）糖化血红蛋白：血红蛋白在红细胞内与血中葡萄糖或磷酸化葡萄糖呈非酶化结合，形成糖化血红蛋白（HbA_1c），其量与血糖浓度呈正相关。正常人HbA_1c<7%，治疗良好的糖尿病患儿应<9%，如>12%时则表示血糖控制不理想。因此，HbA_1c可作为患儿在以往2～3 个月期间血糖是否得到满意控制的指标。

3．葡萄糖耐量试验 本试验用于空腹血糖正常或正常高限，餐后血糖高于正常而尿糖偶尔阳性的患儿。试验方法：试验当日自 0 时起禁食；清晨口服葡萄糖（1.75 g/kg），最大量不超过75 g，每克加水2.5 ml，于3～5 分钟内服完；口服前（0 分钟）及口服后60分钟，120分钟和180 分钟，分别测血糖。结果：正常人 0 分钟血糖<6.7 mmol/L，口服葡萄糖后60 分钟和120 分钟后血糖分别低于10.0 和7.8 mmol/L；糖尿病患儿 120 分钟血糖值>11 mmol/L。试验前应避免剧烈运动、精神紧张，停服双氢克尿噻、水杨酸等影响糖代谢的药物。

【诊断和鉴别诊断】

典型的病例诊断并不困难。对有口渴、消瘦、遗尿症状的患儿；或有糖尿病家族史者；或有不明原因的脱水、酸中毒的患儿都应考虑本病的可能性，避免误诊。本病应与下列情况相鉴别：

1．其它还原糖尿症 尿液中果糖和戊糖等其它还原糖均可使班氏试液呈色，用葡萄糖氧化酶法检测尿液可以鉴别。

2．非糖尿病性葡萄糖尿 有些先天性代谢病如Fanconi综合征、肾小管酸中毒、胱氨酸尿症或重金属中毒等患儿都可发生糖尿，主要依靠空腹血糖或葡萄糖耐量试验鉴别。

3．婴儿暂时性糖尿 病因不明，可能与患儿胰岛β细胞功能发育不够成熟有关。多在出生后6 周内发病，表现为发热、呕吐、体重不增、脱水等症状。血糖增高，尿糖及酮体阳性，经补液等一般处理或给予小量胰岛素（1 U/kg）即可恢复。对这类患儿应进行葡萄糖耐量试验和长期随访，以与 1 型糖尿病鉴别。

4．其他发生酸中毒、昏迷的疾病 如尿毒症、感染中毒性休克、低血糖症、急腹症、颅内感染、重症肺炎等。

【治疗】

糖尿病是终身的内分泌代谢性疾病。其治疗是综合性的，包括胰岛素治疗、饮食管理、运动及精神心理治疗。治疗目的是：消除高血糖引起的临床症状；积极预防并及时纠正酮症酸中毒；纠正代谢紊乱，力求病情稳定；使患儿获得正常生长发育，保证其正常的生活活动；预防并早期诊断并发症。

1．糖尿病酮症酸中毒的治疗 酮症酸中毒迄今仍然是儿童糖尿病急症死亡的主要原因。对糖尿病酮症酸中毒必须针对高血糖、脱水、酸中毒、电解质紊乱和可能并存的感染等情况制定综合治疗方案。密切观察病情变化、血气分析和血、尿液中糖和酮体的变化，随时采取相应措施，避免医源性损害。

（1）液体治疗：液体治疗主要针对脱水、酸中毒和电解质紊乱。酮症酸中毒时脱水量约

为100 ml/kg，一般均属等渗性脱水。因此，应遵循下列原则输液。

输液开始的第 1 小时，按20 ml/kg（最大量1000 ml）快速静滴0.85％氯化钠溶液，以纠正血容量、改善血循环和肾功能。第2～3小时，按10 ml/kg静滴0.45％氯化钠溶液。当血糖<17 mmol/L（300 mg/dl）后，改用含有0.2％氯化钠的5％葡萄糖液静滴。要求在开始的12 小时内至少补足累积损失量的一半，在此后的24 小时内，可视情况按60～80 ml/kg静滴同样溶液，以供给生理需要量和补充继续损失量。

患儿在输液开始前由于酸中毒、分解代谢和脱水的共同作用血清钾较高，但总的体钾储备可能被耗竭。随着液体的输入，特别是应用胰岛素后，血钾迅速降低。因此，在患儿开始排尿后应立即在输入液体中加入氯化钾溶液，一般按每日2～3 mmol/kg（150～225 mg/kg）补给，输入浓度不得>40 mmol/L（0.3 g/dl），并应监测心电图或血钾浓度。

酮症酸中毒时的酸中毒主要是由于酮体和乳酸的堆积，补充水分和胰岛素可以矫正酸中毒。为了避免发生脑细胞酸中毒和高钠血症，对酮症酸中毒不宜常规使用碳酸氢钠溶液，仅在pH<7.1，HCO_3^-<12 mmol/L时，可按2 mmol/kg给予1.4％碳酸氢钠溶液静滴，先用半量，当血pH≥7.2 时即停用，避免酸中毒纠正过快加重脑水肿。

需补充的Na HCO_3（mmol/L）＝（12－所测 HCO_3^- mmol/L）×0.6×kg。

在治疗过程中，应仔细监测生命体征、电解质、血糖和酸碱平衡状态，以避免酮症酸中毒治疗过程中发生合并症，如脑水肿等。其表现为：头痛、意识不清、嗜睡、痉挛、视神经乳头水肿或脑疝等。

（2）胰岛素治疗：糖尿病酮症酸中毒时多采用小剂量胰岛素静脉滴注治疗。首先静推正规胰岛素0.1 U/kg，然后将正规胰岛素25 U加入等渗盐水250 ml中，按每小时0.1 U/kg，自另一静脉通道缓慢匀速输入。输入1～2 小时后，复查血糖以调整输入量。当血糖<17 mmol/L时，应将输入液体换成含0.2％氯化钠的5％葡萄糖液，并停止静滴胰岛素，改为胰岛素皮下注射，每次0.25～0.5 U/kg，每4～6 小时1 次，直至患儿开始进食、血糖稳定为止。

（3）控制感染 酮症酸中毒常并发感染，须在急救同时采用有效抗生素治疗。

酮症酸中毒在处理不当时，可引起脑水肿、低血糖、低血钾、碱中毒、心功能或肾功能衰竭等情况，因此，在整个治疗过程中必须严密观察，随时调整治疗计划，避免因处理不妥而加重病情。

2．长期治疗措施

（1）饮食管理：糖尿病的饮食管理是进行计划饮食而不是限制饮食，其目的是维持正常血糖和保持理想体重。

1）每日总热卡需要量：食物的热量要适合患儿的年龄、生长发育和日常活动的需要，每日所需热量（卡）为1000+（年龄×80～100），对年幼儿宜稍偏高，此外，还要考虑体重、食欲及运动量。全日热卡分配为早餐1/5，中餐和晚餐分别为2/5，每餐中留出少量（5％）做餐间点心。

2）食物的成分和比例：饮食中能源的分配为：蛋白质15％～20％，碳水化合物50％～55％，脂肪30％。蛋白质成分在3 岁以下儿童应稍多，其中一半以上应为动物蛋白，因其含有必需的氨基酸。禽、鱼类、各种瘦肉类为较理想的动物蛋白质来源。糖类则以含纤维素高的，如糙米或玉米等粗粮为主，因为它们造成的血糖波动远较精制的白米、面粉或土豆等制品为小，蔗糖等精制糖应该避免。脂肪应以含多价不饱和脂肪酸的植物油为主。蔬菜选用含糖较少者。每日进食应定时，饮食量在一段时间内应固定不变。

（2）胰岛素治疗：胰岛素是治疗能否成功的关键。胰岛素的种类、剂量、注射方法都与

疗效有关。

1）胰岛素制剂（表17-7）：目前胰岛素的制剂有正规胰岛素（R1）、中效珠蛋白胰岛素（NPH）、长效的鱼精蛋白锌胰岛素（PZI）以及长效胰岛素类似物甘精胰岛素（glargine）和地特胰岛素（detemir）。

甘精胰岛素是在人胰岛素A链21 位以甘氨酸替代天门冬氨酸，B链的羧基端加上两个精氨酸。地特胰岛素是去掉B30 位的氨基酸，在B29 位点连接上含有14-C的脂肪酸链。其结构的改变使得该胰岛素稳定性增强，在酸性环境中呈溶解状态，即清澈溶液，注射前无需预先混匀，可直接皮下注射。一般1～2 小时起效，作用时间维持24 小时，每日只需注射1 次。

表17-7 胰岛素的种类和作用时间

胰岛素种类	开始作用时间（h）	作用最强时间（h）	作用最长时间（h）
短效 RI	0.5	3～4	6～8
中效 NPH	1.5～2	4～12	18～24
长效 PZI	3～4	14～20	24～36

2）胰岛素治疗方案：胰岛素需要量婴儿偏小，年长儿偏大。新诊断的患儿，轻症者胰岛素一般用量为每日0.5～1.0 U/kg，出现明显临床症状以及酮症酸中毒恢复期开始治疗时胰岛素需要量往往大于1 U/kg。NPH和RI按2：1 或3：1 混合，RI与PZI则按3：1 或4：1 混合使用。每日皮下注射两次：早餐前30 分钟，2/3 总量；晚餐前30 分钟，1/3 总量。混合胰岛素时应先抽取RI、后抽取NPH或PZI，每次尽量采用同一型号的注射器。皮下注射部位应选择大腿、上臂和腹壁等处，按顺序轮番注射，1 月内不要在同一部位注射2 次，两针间距2.0 cm左右，以防日久局部皮肤组织萎缩，影响疗效。

3）胰岛素剂量的调整：早餐前注射的胰岛素提供早餐和午餐后的胰岛素，晚餐前注射的胰岛素提供晚餐后及次日晨的胰岛素。应根据用药日血糖或尿糖结果，调整次日的胰岛素用量，每2～3 天调整剂量一次，直至尿糖不超过++；血、尿糖稳定后，在相当时期中可不用再调整。

4）胰岛素注射笔：胰岛素注射笔是普通注射器的改良，用喷嘴压力和极细针头推进胰岛素注入皮下，可减少皮肤损伤和注射精神压力。所用制剂为正规胰岛素和长效胰岛素或中效胰岛素，其成分和比例随笔芯的不同而不同。以普通注射器改用胰岛素注射笔时，应减少胰岛素用量的15%～20%，并仔细监测血糖和尿糖，适时进行调整。

5）胰岛素泵：胰岛素泵（CSII）应用于1 型糖尿病的治疗开始于1970 年代后期，迄今已有20 余年的历史。胰岛素泵不仅可以作为儿童青少年1 型糖尿病的强化治疗手段，还可应用于糖尿病酮症、酮症酸中毒和糖尿病代谢紊乱期的治疗。

胰岛素泵一般使用短效胰岛素，胰岛素用量为0.5～1.0 U/(kg·d)，将全日的总量分为基础量和餐前追加量两部分，两者的用量按1：1 的比例分配。将24 小时划分为日间（07：00～21：00）和夜间（21：00～次日07：00）两个阶段，日夜间基础量之比为2：1。餐前追加量按3 餐平均分配，于每次餐前输注。一般以空腹血糖来调整基础量，2～3 天调整一次剂量，每次不超过1～2 U。

用胰岛素泵治疗糖尿病酮症酸中毒，剂量为0.05～0. 1 U/（kg·h），当患儿的酸中毒被纠正，尿酮体转为阴性时，将胰岛素用量改为0. 5～1. 0 U/（kg·d）继续治疗。治疗过程中注意监测血糖、尿糖和尿酮体。

长期佩戴胰岛素泵的患儿，应注意注射局部的消毒和保持清洁，并定期更换部位，以防感染。

6）胰岛素长期治疗过程中的注意事项：①胰岛素过量：胰岛素过量可致Somogyi现象。由于胰岛素过量，在午夜至凌晨时发生低血糖，在反调节激素作用下使血糖升高，清晨出现高血

糖，即出现低血糖-高血糖反应。如未及时诊断，因日间血糖增高而盲目增加胰岛素用量，可造成恶性循环。故对于尿量增加，同时有低血糖出现或一日内血糖波动较大，胰岛素用量大于每日1.5 U/kg者，应怀疑Somogyi现象，可测午夜后1～3 时血糖，以及时诊断。②胰岛素不足：胰岛素不足可致清晨现象（dawn phenomenon）。因晚间胰岛素不足，在清晨5～9 时呈现血糖和尿糖增高，可加大晚间注射剂量或将NPH注射时间稍往后移即可。持久的胰岛素用量不足可使患儿长期处于高血糖状态，症状不能完全消除，导致生长停滞、肝脾肿大、高血糖、高血脂，并容易发生酮症酸中毒。③胰岛素耐药：患儿在无酮症酸中毒情况下，每日胰岛素用量>2 U/kg仍不能使高血糖得到控制时，在排除Somegyi现象后称为胰岛素耐药。可换用更纯的基因重组胰岛素。

（3）运动治疗：运动时肌肉对胰岛素的敏感性增高，从而增强葡萄糖的利用，有利于血糖的控制。运动的种类和剧烈程度应根据年龄和运动能力进行安排，有人主张1 型糖尿病的学龄儿童每天都应参加1 小时以上的适当运动。运动时必须做好胰岛素用量和饮食调节，运动前减少胰岛素用量或加餐，固定每天的运动时间，避免发生运动后低血糖。

（4）宣教和管理：由于小儿糖尿病的病情不稳定，易于波动，且本病需要终生饮食控制和注射胰岛素，给患儿及其家庭带来种种精神烦恼，因此，医生、家长和患儿应密切配合。医务人员必须向患儿及家长详细介绍有关知识，帮助患儿树立信心，使其能坚持有规律的生活和治疗，同时加强管理制度，定期随访复查。出院后家长和患儿应遵守医生的安排，接受治疗，同时在家做好家庭记录，包括饮食、胰岛素注射次数和剂量、尿糖情况等。

（5）预防并发症：积极预防微血管继发损害所造成的肾功能不全、视网膜和心肌等病变。

（罗小平）

第十八章 儿童急救

第一节 儿童心肺复苏

心跳呼吸骤停是指患儿突然呼吸及循环功能停止。心肺复苏（cardiopulmonary resuscitation，CPR）是包括采用一组简单的技术，使生命得以维持的方法。

【心肺复苏技术】

心肺复苏技术包括三个方面：

1．基本生命支持（basic life support） 儿童基本生命支持包括一系列支持或恢复呼吸或心跳呼吸停止儿童的有效通气或循环功能的技能。任何一个受过训练的医务人员或非医务人员都可以进行基本生命支持，它对伤病儿童的最终恢复是非常重要的。当心跳呼吸停止或怀疑停止时，同样需要迅速将患儿送到能给以进一步生命支持的医疗机构。

2．高级生命支持（advanced life support） 为心肺复苏的第二阶段，有经验的医护人员参与此时的抢救工作，并且常有明确的分工，协调处理呼吸、胸外心脏按压、辅助药物应用、输液、监护及必要的记录。小儿心跳呼吸骤停后对人工通气或用氧有反应、或需要高级生命支持的时间<5 分钟，在复苏成功后神经系统正常的可能性较大。

3．稳定及复苏后的监护 指为使复苏后的患者稳定而进行的进一步处理及监护。

【小儿心跳呼吸骤停病因】

引起小儿心跳呼吸骤停的原因甚多，如新生儿窒息、婴儿猝死综合征、喉痉挛、喉梗阻、气管异物、胃食管反流、严重肺炎及呼吸衰竭、药物、严重心律失常、中毒、代谢性疾病、心肌炎、心肌病、心力衰竭、心血管介入治疗操作过程、各种意外损伤等。心肺复苏的措施一旦启动，就应该开始考虑心肺骤停的原因。心肺骤停难以预料，但触发的高危因素应引起足够的重视，以便在心肺骤停发生前进行必要的干预以避免其发生。高危因素包括：

1．心血管系统的状态不稳定 如大量失血、难治性心衰、低血压和反复发作的心律失常。

2．急速进展的肺部疾病 如严重的哮喘、喉炎、重症肺炎、肺透明膜病等。

3．外科手术后的早期 如应用全身麻醉及大量镇静剂足以使患儿对各种刺激的反射能力改变。

4．安有人工气道的患儿气管插管发生堵塞或脱开。

5．神经系统疾病有急剧恶化时，如昏迷患者常无足够的呼吸驱动以保证正常的通气。

另外，临床的一些操作对于有高危因素的患儿能加重或触发心跳呼吸骤停，包括：① 气道的吸引：能引起低氧、肺泡萎陷及反射性心动过缓；② 不适当的胸部物理治疗（如拍背、翻身、吸痰等）：可使更多的分泌物溢出，阻塞气道，也可使患儿产生疲劳；③ 任何形式的呼吸支持（如人工呼吸机的应用）的撤离：使病人必须从以前的人工呼吸转变为自主呼吸做功，如降低吸入氧浓度、撤离 CPAP 或机械通气、拔除气管插管等；④ 镇静剂的应用：如麻醉剂、镇静药和止咳药的应用所致的呼吸抑制；⑤ 各种操作：如腰穿时使呼吸屏住，可出现心跳骤停；⑥ 迷走神经的兴奋性增加：一些临床操作可引起迷走神经的兴奋性增加，如鼻胃管的放置、气管插管操作等。

此外，高危婴儿喂养时由于吞咽-呼吸的不协调也可引起心跳呼吸骤停。应特别注意循环的

失代偿表现，包括外周循环不良、心动过缓、呼吸形式的改变或呼吸暂停、发绀、对刺激的反应性下降等。有上述表现时应尽可能停止相关的操作，并给以生命支持。

【诊断】

临床表现为突然昏迷，部分有一过性抽搐，呼吸停止，面色灰暗或紫绀，瞳孔散大和对光反射消失。大动脉（颈、股动脉）搏动消失，听诊心音消失。如做心电图检查可见等电位线、电机械分离或心室颤动等。

心跳呼吸骤停的诊断并不困难。一般在患儿突然昏迷及大血管搏动消失即可诊断，而不必反复触摸脉搏或听心音，以免延误抢救时机。

【治疗】

对于心跳呼吸骤停，现场抢救（first aid ）十分必要，应争分夺秒地进行，以保持呼吸道通畅、建立呼吸及建立人工循环的顺序进行，以保证心、脑等重要脏器的血液灌流及氧供应。心肺复苏的程序常推荐用A- B-C-D-E 方法，即：气道（Airway，A），呼吸（Breathing，B），循环（Circulation，C），药物（Drugs，D），电击除颤复律（Electricity，E）。

1．保持呼吸道通畅（Airway，A） 小儿低氧血症和呼吸停止可能引起或造成急剧恶化和心跳呼吸停止。因此建立和维持气道的开放和保持足够的通气是基本生命支持最重要的内容。首先应去除气道内的分泌物、异物或呕吐物，有条件时予以口、鼻等上气道吸引。异物吸入是儿童常见的气道阻塞原因，复苏时应予以考虑，尽可能去除气道异物。将患儿头向后仰，抬高下颌，一只手置于患儿的前额，将头向背部倾斜处于正中位，颈部稍微伸展，即嗅气位（sniffing position）。用另一只手的几个手指放在下颌骨的颏下，提起下颌骨向外上方，注意不要让嘴闭上或推颌下的软组织，以免阻塞气道。当颈椎损伤完全不能运动时，通过提下颌来开通气道（图18-1）。也可放置口咽导管，使口咽部处于开放状态。

2．建立呼吸（Breathing，B） 气道通畅后，患儿可能出现自主呼吸。如仍无自主呼吸时应采用人工辅助通气，维持气体交换。常对于新生儿，如无自主呼吸或为无效喘息、有自主呼吸但心率<100 次/分、在80%浓度的氧吸入后仍有中心性发绀时即可进行正压通气复苏。常用的方法有：

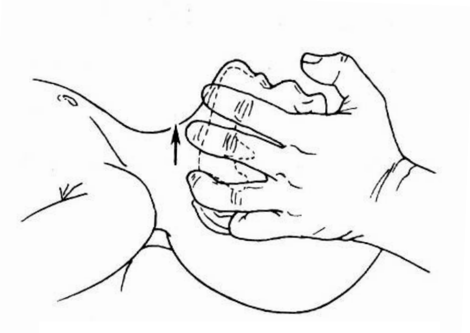

图 18-1 通过推下颌来开通气道

（1）口对口人工呼吸：此法适合于现场急救。操作者先深吸一口气，如患者是1 岁以下婴儿，将嘴覆盖婴儿的鼻和嘴；如果是较大的婴儿或儿童，用口对口封住，拇指和食指紧捏住患儿的鼻子，保持其头后倾；将气吹入，同时可见患儿的胸廓抬起。停止吹气后，放开鼻孔，使患儿自然呼气，排出肺内气体。重复上述操作，儿童18～20 次/分，婴儿可稍加快。口对口呼吸即使操作正确，吸入氧浓度也较低（<18％），操作时间过长，术者极易疲劳，也有感染疾病的潜在可能，故应尽快获取其他辅助呼吸的方法替代。

（2）复苏囊的应用：在多数儿科急诊中，婴幼儿可用气囊面罩进行有效的通气。常用的气囊通气装置为自膨胀气囊，递送的氧浓度为30％～40％。气囊尾部可配贮氧装置，保证输送高浓度的氧气。带有贮氧装置的气囊可以提供60％～95％浓度氧气。气囊常配有压力限制活瓣装置，压力水平在35～40 cmH_2O。将连接于复苏皮囊的面罩覆盖于患儿的口鼻。正确的面罩大小应该能保证将空气密闭在面部，从鼻梁到下颏间隙盖住口鼻，但露出眼睛。用一只手将面罩固定在脸上并将头或下颌向上翘起。对婴幼儿，术者4、5 指钩住下颌角向上抬，第3 指根部抵住下颌，保证面罩与面部紧密接触。在面罩吸氧时，一定程度的头部伸展能保证气道通畅。

婴儿和幼儿要最好保持在中间的吸气位置，而不要过度伸展头部，以免产生气道压迫梗阻。展头部，以免产生气道压迫梗阻。在上述操作时应观察患儿的胸廓起伏以了解辅助通气的效果；如无有效通气（表现为胸廓抬动不明显）应考虑是否仍存在气道梗阻，如气管异物仍未排出等。

对于新生儿复苏的用氧问题：采用空气（21%氧浓度）复苏可能与100%氧同样有效，甚至更为有利。可在开始用空气复苏，如在生后90 秒后无改善，则改为100%氧复苏。

（3）气管内插管人工呼吸法 当需要持久通气时，或面罩吸氧不能提供足够通气时，就需要用气管内插管代替面罩吸氧。小于8 岁的患儿用不带囊气管内插管，大于8 岁的患儿用带囊插管。插管内径的大小可用公式进行估算：内径（mm）=（16+患儿年龄）/4。插管后可继续进行皮囊加压通气，或连接人工呼吸机进行机械通气。

3．循环支持（Circulation，C） 当气道通畅和建立了有效通气后应检查脉搏，如无脉搏，应给以胸外心脏按压。胸外心脏按压的指征是：新生儿心率<60 次/分；婴儿或儿童心率<60 次/分伴有灌注不良的体征。

胸外心脏按压方法：对新生儿或小婴儿按压时可用一手托住患儿背部，将另一手两手指置于乳头线下一指处进行按压（图18-2），或两手掌及四手指托住两侧背部，双手大拇指按压（图18-3）。对于1～8 岁的儿童，可用一只手固定患儿头部，以便通气；另一手的手掌根部置于胸骨下半段（避开剑突），手掌根的长轴与胸骨的长轴一致（图18-4）。对于年长儿（>8 岁），胸部按压方法与成人相同，应将患儿置于硬板上，将一手掌根部交叉放在另一手背上，垂直按压胸骨下半部。每次按压与放松比例为1：1，按压深度为胸部厚度的1/3～1/2，频率在新生儿、婴儿和儿童为100 次。胸外心脏按压与呼吸的配合在新生儿为3：1，<8 岁为5：1；>8 岁为按15：2。按压后1 分钟判断有无改善，观察颈动脉（对于1～8 岁儿童）、股动脉搏动，瞳孔大小及皮肤颜色等。在临床上当触及大动脉搏动提示按压有效；如有经皮血氧饱和度监测，其值上升也提示有效。

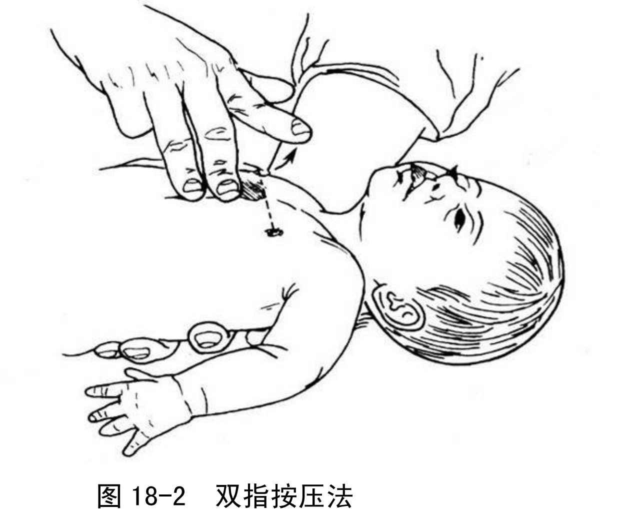

图 18-2 双指按压法

（用于新生儿和小婴儿）

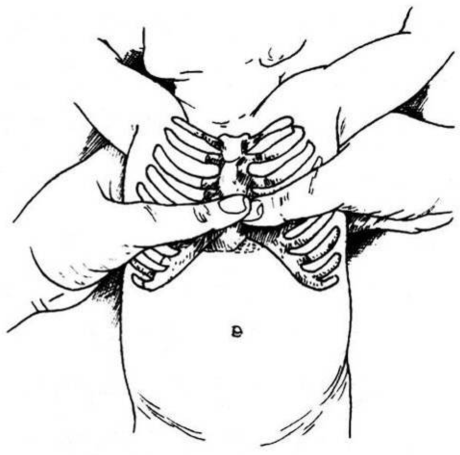

图 18-3 双手拇指按压法

（用于新生儿和小婴儿）

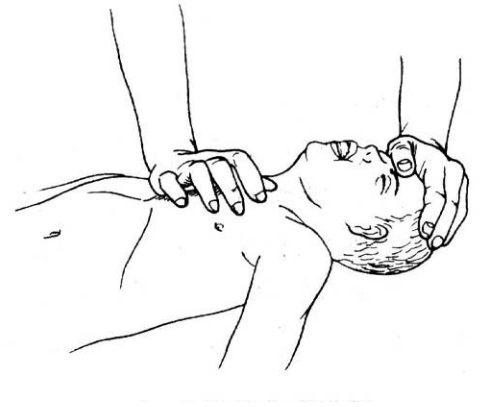

图 18-4 对于 1～8 岁的儿童进行心脏按压

4．药物治疗（Drugs，D） 大多数患儿，尤其是新生儿在呼吸道通畅，呼吸建立后心跳可恢复。如胸外心脏按压仍无效，可试用药物。在心跳骤停时，最好静脉内给药，但由于很难建立静脉通路，有些药物可在气管内给入，如阿托品、肾

上腺素、利多卡因等，其中肾上腺素是最常用的药物。儿童气管内用药最佳剂量尚不肯定，气管内用药剂量应比静脉内用量大，才能达到同样的疗效。药物从骨髓腔注入能很好地被吸收，骨髓腔内注射与静脉内注射效果相同。常用药物有：

（1）肾上腺素：儿科患者最常见的心律失常是心脏停搏和心动过缓，肾上腺素有正性肌力和正性频率作用。剂量： 0.01 mg/kg，（1：10000 溶液0.1 ml/kg），静脉或骨髓腔内给以；第二剂和以后的剂量可与首剂相同，也可用1：1000 溶液、剂量为0.1～0.2 mg/kg；气管内给药0.1 mg/kg。上述给药可间隔3～5 分钟重复1 次。

（2）碳酸氢钠：儿科患者中心脏骤停的主要病因是呼吸衰竭，快速有效的通气对于控制心跳呼吸骤停引起的酸中毒和低氧血症很必要。在心脏骤停常规应用碳酸氢钠并不一定能改善预后。碳酸氢钠应用可促进CO_2生成，而CO_2比HCO_3^-更易通过细胞膜，可以引起短暂的细胞内酸中毒，从而导致心肌功能不全。鉴于这些潜在毒性，轻、中度酸中毒、特别是有通气不足存在时，不宜使用碳酸氢钠。改善通气和扩容一般可以解决酸中毒。碳酸氢钠在较长时间的心脏骤停患儿可考虑使用，其剂量为1 mEq/kg，可经静脉或骨髓腔给予。当自主循环建立及抗休克液体输入后，碳酸氢钠的用量可依血气分析的结果而定。

（3）阿托品：应用指征：为低灌注和低血压性心动过缓、预防气管插管引起的迷走神经性心动过缓、房室传导阻滞所引起的少见的症状性心动过缓以及抗胆碱酯酶类药中毒等。剂量：0.01～0.02 mg/kg，静脉、气管内或骨髓腔给药，间隔5 分钟可重复使用。最大剂量儿童不能超过1 mg，青少年不超过2 mg。

（4）葡萄糖：在婴幼儿心脏复苏时，应快速进行床边的血糖检测，有低血糖时应立即给葡萄糖。当无血糖监测条件而患儿有低血糖症状或临床怀疑有低血糖时，也可给以葡萄糖。剂量：0.5～1.0 g/kg，以25％葡萄糖液静脉注射。对于新生儿，可用10%葡萄糖液1 ml/kg静脉注射。

（5）钙剂：仅在疑有低钙血症时才可给钙剂，在治疗高钾血症、高镁血症、钙通道阻滞剂过量时，也可考虑使用。对心跳已停搏者不适用。剂量：葡萄糖酸钙100～200 mg/kg（10％葡萄糖酸钙1～2 ml/kg）或氯化钙10～30 mg/kg（10％氯化钙0.1～0.3 ml/kg）。

（6）利多卡因：当存在室颤时可用利多卡因。剂量：负荷量为1 mg/kg，负荷量给以后即给静脉维持，剂量为20～50 μg/（kg・min）。

（7）纳络酮：用于阿片类药物过量。在新生儿，纳络酮仅用于在正压通气后心率和皮肤颜色正常而患儿仍有呼吸抑制，同时患儿母亲在分娩前4 小时内有使用过阿片类药物者。常用剂量为0.1 mg/kg，静脉或气管内应用，必要时可重复给药，最大剂量为2 mg。

5．电击除颤复律（Electricity，E）尽管患儿可能无基础心脏疾病，在复苏过程中可出现心律失常。当出现心室颤动、室性心动过速和室上性心动过速时，可用电击除颤复律。

6．其他治疗 对复苏后患儿出现的低血压、心律紊乱、颅内高压等应分别给以预防及处理。

第二节 急性呼吸衰竭

呼吸衰竭（respiratory failure）指由各种原因导致的中枢或（和）外周性的呼吸生理功能障碍，使动脉血氧分压降低，和（或）二氧化碳分压增加，患儿有呼吸困难（窘迫）的表现，如呼吸音降低或消失、严重的三凹征或吸气时有辅助呼吸肌参与及意识状态的改变。儿童呼吸衰竭多为急性呼吸衰竭，是儿科重要的危重病，具有较高的死亡率。低氧性呼吸衰竭（hypoxemic respiratory failure）可定义为：在排除发绀性心脏病的前提下，患儿在吸入氧浓度（FiO2）> 60%时，动脉氧分压<60 mmHg；高碳酸血症性呼吸衰竭（hypercapnia respiratory failure）定义为：

急性期$PaCO_2 > 50$ mmHg。但这些传统的单纯将血气分析指标作为呼吸衰竭的诊断存在一定的局限性，随着呼吸急救技术的进步，尤其是机械通气技术的普遍应用，对呼吸衰竭的定义和理解有了新的认识和发展。

【病因与病理生理】

呼吸衰竭主要病理生理是呼吸系统不能有效地在空气-血液间进行氧和二氧化碳的气体交换，导致机体氧的供应和二氧化碳的排出不能满足代谢的需求。

儿童呼吸衰竭的病因在不同年龄有较大的差异。根据年龄，常见的引起呼吸障碍的原发疾病有：

1．新生儿

（1）早产儿由于肺表面活性物质缺乏而导致的呼吸窘迫综合征（respiratory distress syndrome，RDS）。

（2）新生儿窒息。

（3）吸入性肺炎。

2．小于2 岁儿童

（1）支气管肺炎。

（2）哮喘持续状态。

（3）喉炎。

（4）先天性心脏病。

（5）气道异物吸入。

（6）先天性气道畸形（气管蹼、囊肿、大叶肺气肿等）。

（7）较大腺样体或扁桃体所致的鼻咽梗阻。

3．2 岁以上儿童

（1）哮喘持续状态。

（2）多发性神经根炎。

（3）中毒。

（4）溺水。

（5）脑炎。

（6）损伤。

也可根据引起呼吸衰竭的原发病因分为：①外周性呼吸衰竭：即由呼吸器官本身疾病引起，包括原发于气道、肺、胸廓、肺循环等病变，如重症支气管肺炎、哮喘持续状态、气胸等所致的呼吸衰竭。临床上以低氧血症为主，患儿常有呼吸困难、呼吸做功增加。②中枢性呼吸衰竭：即由呼吸的驱动障碍所致的呼吸衰竭，而呼吸器官本身可正常。中枢呼吸活动和外周呼吸肌的协调类似呼吸的泵驱动，这种泵驱动障碍导致肺泡通气不足，血二氧化碳分压增高；如脑炎、窒息、中毒等所致的呼吸衰竭。血氧分压在呼吸泵衰竭时也可降低，但一般给氧后较易纠正。

【临床表现】

1．原发疾病的临床表现 如肺炎、脑炎等症状和体征。

2．呼吸衰竭的早期表现 在严重肺部疾病使呼吸衰竭将要发生前，患儿常有明显的呼吸窘迫表现，如呼吸频率增加、过度使用辅助呼吸肌参与呼吸、鼻翼扇动等；由于儿童的胸廓顺应性好，三凹征出现特别明显。在新生儿及较小的婴儿，由于存在呼气时将会厌关闭以增加呼气末正压的保护机制，可在呼气时出现呻吟。

由于呼吸驱动（泵衰竭）所致的呼吸衰竭在早期无明显的呼吸窘迫表现，在临床上相对不

易发现。例如，患儿有神经肌肉性疾病可引起肺泡通气不足，而此时的三凹征并不出现，只有从呼吸浅表或呼吸率异常减慢等线索中发现。

3．重要脏器的功能异常 小儿呼吸衰竭除原发疾病的临床表现如肺炎、脑炎等症状和体征外，低氧、高碳酸血症、酸中毒等足以导致重要脏器的功能异常，包括：

（1）心血管系统：中等程度的低氧和高碳酸血症可引起心率和心排出量的增加，而严重低氧血症可致心排出量降低。中等程度的低氧血症可使心律失常的机会增加。低氧和高碳酸血症可引起肺血管阻力增加。

（2）呼吸系统：在外周和中枢化学感受器正常状态下，呼吸衰竭时患儿的每分通气量增加；随气道阻塞程度的加重，辅助呼吸肌常参与呼吸运动。急性呼吸窘迫综合征（acute respiratory distress syndrome，ARDS）是急性呼吸衰竭中较为严重的典型病症。由于严重的肺损伤而影响肺的气体交换、肺顺应性降低、胸部X线片显示肺弥漫性浸润。儿童ARDS的常见触发因素有：严重的窒息、休克、脓毒症、心脏外科手术后并发症、肺的化学损伤、血液系统恶性肿瘤、重症肺炎，尤其是重症病毒性肺炎如流感、副流感、禽流感等。

（3）中枢神经系统：因低氧和高碳酸血症，可出现头疼、神志模糊、嗜睡、激惹和焦虑等。

（4）肾脏：呼吸衰竭可导致钠、水排出减少。

（5）血液系统：慢性的呼吸衰竭可引起红细胞增多，由于血二氧化碳分压增加，氧离曲线右移，使红细胞携带的氧在外周更易释放。

（6）代谢：由于无氧代谢，乳酸产生增加，使血pH值明显降低。

【急性呼吸衰竭的诊断和评估】

1．根据呼吸衰竭的临床表现进行诊断和评估 尽管对小儿急性呼吸衰竭常用血液气体分析指标作为诊断和评估的方法，根据临床症状和体征作出诊断和病情判断十分重要。儿童的呼吸系统代偿能力有限，故早期认识呼吸衰竭很重要；应尽可能预测呼吸衰竭的发生，避免气体交换障碍的发生。当怀疑有呼吸衰竭时，应快速评估患儿的通气状态，包括呼吸运动是否存在及强弱程度、呼吸频率、呼吸运动幅度、是否存在发绀及上呼吸道梗阻。此外，在低氧及高碳酸血症时，患儿常有意识状态的改变，如少哭、少动、意识模糊与激惹交替等。

当患儿出现明显的呼吸困难且影响到重要脏器的功能，尤其是出现呼吸暂停时，往往提示为严重的呼吸衰竭。在处理已出现的呼吸衰竭伴低氧时，不必等待患儿只吸空气（21%氧）状态下的血气分析值，应立即纠正低氧血症，再针对引起呼吸衰竭的原发病进行诊断和治疗。

2．对肺气体交换障碍程度的评估 血液气体分析在呼吸衰竭的评估中有重要地位。将吸入氧浓度（FiO_2）> 60%时，动脉氧分压<60 mmHg、急性期$PaCO_2$>50 mmHg作为呼吸衰竭的诊断标准，是较客观可操作的指标，可反映通气和氧合状态。但PaO_2也受心脏右向左分流的影响，$PaCO_2$在慢性碱中毒时可代偿性增加，而这些情况本身并非呼吸系统问题，在这些情况下，单凭血气分析指标不能诊断为呼吸衰竭。对于呼吸衰竭患儿在用氧情况下，单凭动脉血氧分压（PaO_2）不能反映低氧程度和肺部病变的进展或好转，此时应采用包涵吸入氧浓度因素的评估指标，如肺泡-动脉氧分压差（$A\text{-}aDO_2$）。当评估氧合状态时应同时考虑血氧分压与给氧的浓度，此时采用$A\text{-}aD\,O_2$能对呼吸衰竭的严重程度及变化作定量的判断。$A\text{-}aDO_2$ = （713 mmHg ×FiO_2）－[（$PaCO_2$/0. 8 ）+ PaO2]，该指标的基本原理是：肺弥散功能正常时肺泡氧分压（通过肺泡气体方程式计算：P_AO_2 = 713 mmHg × FiO_2－$PaCO_2$/0. 8）与动脉血氧分压（PaO_2）的差值很小（<10 mmHg），当肺部疾病严重而影响气体弥散或存在肺内或肺外（心脏水平）分流时，肺泡氧分压与动脉血氧分压差值增大，差值越大疾病程度越重。该指标可作为动态评估

用。在临床上也常用PaO_2/FiO_2作为呼吸衰竭严重程度的评估指标，其意义与（$A\text{-}aD\ O_2$）类似，且不需要计算$P_A\ O_2$，便于应用。该比值越小，肺部疾病越重。临床上将$Pa\ O_2/Fi\ O_2<300$ 诊断为急性肺损伤、$Pa\ O_2/Fi\ O_2<200$ 诊断为急性呼吸窘迫综合征（ARDS）。

动脉血$PaCO_2$水平直接反映了肺泡通气量的变化，它一般不受吸入氧浓度的影响，$PaC\ O_2$的显著增高往往是需要机械辅助通气的指证。血pH值往往结合$PaC\ O_2$水平分析，判断是代谢性还是呼吸性酸碱平衡紊乱，这在呼吸衰竭的临床评估中也十分重要。

【治疗】

呼吸衰竭治疗目标是恢复正常的气体交换，同时使并发症减低到最小程度。

1．一般治疗 对于小儿急性呼吸衰竭，一般治疗包括应将患儿置于舒适的体位，对于重症呼吸衰竭需呼吸支持者，采用俯卧位可能对通气及患者预后更为有利。胸部物理治疗，如给以翻身、拍背、吸痰等，使气道保持通畅，减少呼吸道阻力和呼吸做功，是呼吸衰竭治疗的辅助措施。对重症呼吸衰竭的营养支持、合理液体平衡对原发病恢复、气道分泌物排出和保证呼吸肌的正常做功有重要意义。

2．原发疾病的治疗 针对原发疾病的治疗，如先天性心脏病心力衰竭肺水肿所致呼吸功能不全应采用强心药和利尿剂；对于哮喘持续状态，应用抗炎、解除气道痉挛等措施；对与肺部感染选用合理的抗感染治疗等。

3．氧疗与呼吸支持

（1）吸氧：低氧血症较高碳酸血症的危害更大，而用氧相对比较安全，故在呼吸衰竭早期应给以吸氧。常用鼻导管或面罩；对于新生儿和小婴儿，头罩吸氧能获得较高浓度和较均匀的氧吸入，同时也便于精确估计吸入氧浓度。应注意吸入氧的加温和湿化，以利呼吸道分泌物的稀释和排出。

（2）辅助机械通气：尽管吸氧可能纠正低氧，严重的呼吸衰竭常常需要机械通气给以支持。目前，机械通气已成为呼吸衰竭治疗的主要手段。机械通气应用的适应证常根据患儿有持续或进行性的气体交换障碍、出现呼吸暂停、及呼吸衰竭严重影响其他脏器功能等考虑。机械通气患儿常需进行气管插管。

4．特殊的呼吸支持 对重症呼吸衰竭在常规呼吸支持无效的情况下，可给以较特殊的呼吸或生命体征支持，包括：

（1）体外膜氧合（ECMO）：作为体外生命支持手段能降低其死亡率，其适应证之一必须是肺原发疾病是可逆性的。ECMO原理为将非氧合血引出体外，通过膜氧合器进行氧合，再进入患者循环，起到人工肺的作用。该治疗所需复杂设备、投入大量人力及费用。

（2）液体通气：全氟化碳液体由于其理化特性与众不同，对氧和二氧化碳高度溶解，对气流的阻力很低，能显著降低表面张力。以全氟化碳液体进行气体交换或部分液体通气（全氟化碳液体仅补充功能残气量，潮气量以常规呼吸机提供）能增加肺顺应性、改善氧合、降低二氧化碳分压及增加pH值。

（3）高频通气：高频通气越来越多被用于急性呼吸衰竭。通常在ARDS应用高频通气时将平均气道压较常频呼吸机提高，这种使用方法可提高氧合，同时，心排出量并未受到影响，气漏的发生率也未增加。在某些情况下，如支气管胸膜漏，高频通气明显优于常规呼吸机。高频通气也可与其他治疗方法，如NO吸入等联合应用，增加其疗效。

（4）NO吸入治疗：呼吸衰竭的病理生理机制包括肺血管收缩，导致通气/血流比值（V/Q）失调和低氧。通过吸入NO的方法可选择性扩张肺血管，当有通气的肺泡所支配的血管舒张时，氧合改善。

第二节 小儿急性中毒

某些物质接触人体或进入体内后，与体液和组织相互作用，破坏机体正常的生理功能，引起暂时或永久性的病理状态或死亡，这一过程称为中毒。小儿急性中毒（acute poisoning）多发生在婴幼儿至学龄前期，是儿科急诊的常见疾病之一。婴幼儿时期常为误服药物中毒，而学龄前期主要为有毒物质中毒。小儿的中毒与周围环境密切相关，常为急性中毒。小儿接触的各个方面，如食物、环境中的有毒动、植物，工、农业的化学药品，医疗药物，生活中使用的消毒防腐剂、杀虫剂和去污剂等，都可能发生中毒或意外事故。造成小儿中毒的原因主要是由于年幼无知，缺乏生活经验，不能辨别有毒或无毒。婴儿时期往往拿到东西就放入口中，使接触毒物的机会增多。因此小儿中毒的诊断和急救工作显得十分重要。

【中毒的途径】

1．经消化道吸收中毒 为最常见的中毒形式，可高达90％以上。毒物进入消化道后可经口腔黏膜、胃、小肠、结肠和直肠吸收，但小肠是主要吸收部位。常见的原因有食物中毒、药物误服、灭鼠或杀虫剂中毒、有毒动、植物中毒、灌肠时药物剂量过量等。

2．皮肤接触中毒 小儿皮肤较薄，脂溶性毒物易于吸收；毒物也可经毛孔到达毛囊，通过皮脂腺、汗腺吸收。常见有穿着有农药污染的衣服、蜂刺、虫咬、动物咬伤等。

3．呼吸道吸入中毒 多见于气态或挥发性毒物的吸入。由于肺泡表面积大，毛细血管丰富，进入的毒物易迅速吸收，这是气体中毒的特点。常见有一氧化碳中毒、有机磷吸入中毒等。

4．注入吸收中毒 多为误注药物。如毒物或过量药物直接注入静脉，则被机体吸收的速度最快。

5．经创伤口、面吸收 如大面积创伤而用药不当，可经创面或创口吸收中毒。

【中毒机制】

因毒物种类难以统计，很难了解所有毒物的中毒机理，常见的中毒机制包括：

1．干扰酶系统 毒物通过抑制酶系统，通过竞争性抑制、与辅酶或辅基反应或相竞争，夺取酶功能所必需的金属激活剂等。

2．抑制血红蛋白的携氧功能 如一氧化氮中毒使氧合血红蛋白形成碳氧血红蛋白、亚硝酸盐中毒形成高铁血红蛋白，使携氧功能丧失。

3．直接化学性损伤。

4．作用于核酸 如烷化剂氮芥和环磷酰胺，使DNA烷化，形成交叉联结，影响其功能。

5．变态反应 由抗原抗体作用在体内激发各种异常的免疫反应。

6．麻醉作用。

7．干扰细胞膜或细胞器的生理功能。

8．其他。

【毒物在人体内的分布与排泄】

1．毒物的分布 主要在体液和组织中，影响分布的因素有毒物与血浆蛋白的结合力、毒物与组织的亲和力等。

2．毒物的排泄 可经肾、胆道或肠道排泄；部分毒物在肠内可被再吸收形成肠肝循环，导致从体内延缓排泄。其他排泄途径有经汗腺、唾液腺、乳汁排至体外；有害气体则经肺排出。

【中毒的诊断】

1．病史 由于小儿，尤其是婴幼儿的特点，家属陈述病史非常重要。在急性中毒的诊断中，家长如能告知中毒经过，则诊断极易。否则，由于中毒种类极多，加上小儿不会陈述病情，诊

断有时极为困难。

应详细询问：发病经过，病前饮食内容，生活情况，活动范围，家长职业，环境中有无有毒物品，特别是杀虫、毒鼠药，家中有无常备药物，经常接触哪些人，同伴小儿是否同时患病等。

临床症状与体征常无特异性，小儿急性中毒首发症状多为腹痛、腹泻、呕吐、惊厥或昏迷，严重者可出现多脏器功能衰竭。

2．体格检查 要注意有重要诊断意义的中毒特征，如呼气、呕吐物与某种物质相关的特殊气味；口唇甲床是否发绀或樱红；出汗情况；皮肤色泽；呼吸状态、瞳孔、心律失常等。同时还需检查衣服、皮肤及口袋中是否留有毒物，以提供诊断线索。

3．毒源调查及检查 现场检查需注意病儿周围是否留有剩余毒物，如有否敞开的药瓶或散落的药片、可疑的食物等，尽可能保留患者饮食、用具，以备鉴定。仔细查找吐出物、胃液或粪便中有无毒物残渣；若症状符合某种中毒，而问不出中毒史时，可试用该种中毒的特效解毒药作为诊断性治疗。有条件时应采集患者呕吐物、血、尿、便或可疑的含毒物品进行毒物鉴定，这是诊断中毒的最可靠方法。

【中毒的处理】

处理原则为发生急性中毒时，应立即治疗，否则会失去抢救机会。在毒物性质未明时，按一般的中毒治疗原则抢救患儿。在一般情况下，以排除毒物为首要措施，尽快减少毒物对机体的损害；维持呼吸、循环等生命器官的功能；采取各种措施减少毒物的吸收，促进毒物的排泄。

1．现场急救使患儿稳定 使患儿呼吸道保持通畅，呼吸有效及循环良好是非常重要的。急救的方式与其他危重儿相似。应监测患儿的血氧饱和度、心率和心电图；建立静脉输液通路；对呼吸抑制或气道阻塞患儿应给予气管插管人工呼吸机应用；如明确是阿片类药物中毒所致的呼吸抑制，则可先用阿片类受体拮抗剂治疗，使呼吸恢复。

2．毒物的清除 根据中毒的途径、毒物种类及中毒时间采取相应的排毒方式。

（1）排除尚未吸收的毒物：大多数毒物经消化道或呼吸道很快被吸收，许多毒物可经皮肤吸收。一般来说，液体性药（毒）物在误服后30 分钟内被基本吸收，而固体药（毒）物在误服后1～2 小时内被基本吸收，故迅速采取措施减少毒物吸收可使中毒程度显著减轻。

1）催吐：适用于年龄较大、神志清醒和合作的患儿。对口服中毒的患儿，当神志清醒，无催吐禁忌证时，均可进行催吐。可用手指、筷子、压舌板刺激咽部引起反射性呕吐。一般在中毒后4～6 小时内进行，催吐越早效果越好。有严重心脏病、食管静脉曲张、溃疡病、昏迷或惊厥病人、强酸或强碱中毒、汽油、煤油等中毒及6 个月以下婴儿不能采用催吐。

2）洗胃：常在催吐方法不成功或病人有惊厥、昏迷而去除胃内容确有必要时进行。洗胃方法是经鼻或经口插入胃管后，用50 ml注射器抽吸，直至洗出液清澈为止，首次抽出物送毒物鉴定。常用的洗胃液有：温水、鞣酸、高锰酸钾（1：10000）、碳酸氢钠（2%～5%）、生理盐水或0.45%氯化钠溶液；洗胃禁忌的腐蚀性毒物中毒可用中和法，牛奶亦可起中和作用，同时可在胃内形成保护膜，减少刺激。可将活性炭加水，在洗胃后灌入或吞服，以迅速吸附毒物。

3）导泻：可在活性炭应用后进行，使活性炭-毒物复合物排出速度加快。常用的泻药有硫酸镁，每次0.25g/kg，配成25%的溶液，可口服或由胃管灌入。在较小的儿童，应注意脱水和电解质紊乱。

4）全肠灌洗（whole bowel irrigation）：中毒时间稍久，毒物主要存留在小肠或大肠，而又需尽快清除时，需作洗肠；对于一些缓慢吸收的毒物如铁中毒等较为有效。常用大量液体作高位连续灌洗（小儿约用1500～3000 ml），直至洗出液变清为止。洗肠液常用1%温盐水或清水，

也可加入活性炭，应注意水、电解质平衡。

5）皮肤、黏膜的毒物清除：接触中毒时应脱去衣服，用大量清水冲洗毒物接触部位，或用中和法即用弱酸，弱碱中和强碱、强酸；如用清水冲洗酸、碱等毒物应至少10 分钟以上。

6）对于吸入中毒，应将患儿移离现场，放置在通风良好、空气新鲜的环境，清理呼吸道分泌物，给氧气吸入。

7）止血带应用：注射或有毒动物咬伤所致的中毒，在肢体近心端加止血带，阻止毒物经静脉或淋巴管弥散，止血带应每10～30 分钟放松1 次。

（2）促进已吸收毒物的排除

1）利尿：大多数毒物进入机体后经由肾脏排泄，因此加强利尿是加速毒物排出的重要措施。静脉输注5％～10％葡萄糖溶液可以冲淡体内毒物浓度，增加尿量，促使排泄。患者较轻或没有静脉点滴条件时，可让其大量饮水。但如病人有脱水，应先纠正脱水。可应用利尿药，常用速尿1~2 mg/kg 静脉注射；20％甘露醇0.5～1 g/kg，或25％山梨醇1～2 g/kg静滴。大量利尿时应注意适当补充钾盐。保证尿量每小时在6～9 ml//kg。在利尿期间应监测尿排出量、液体入量、血清血电解质等。当病儿苏醒、严重中毒症状减轻或药物浓度低于中毒水平时，则可停止利尿。

2）碱化或酸化尿液：毒物肾脏的清除率与尿量并不成比例，单独利尿并不意味排泄增加。碱化尿液后可使弱酸如水杨酸和苯巴比妥清除率增加；降低尿pH值使弱碱类排出增加的方法在临床上较少应用。常采用碳酸氢钠溶液l～2 mmol/kg（1～2 mEq/kg）静脉滴注1～2 小时，在此期间检查尿pH，滴注速度以维持尿pH 7.5～8为标准。乙酰唑胺同时有利尿和使尿碱化作用。维生素C 1～2 g加于500 ml溶液中静脉滴入亦可获得酸性尿。

3）血液净化方法：①透析疗法：很多种危重的急性中毒患者，可采用透析疗法增加毒物排出。透析疗法有多种，常用腹膜透析和血液透析。腹膜透析较简便易行；血液透析（人工肾）是很好的透析方法，能代替部分肾脏功能，将血液中的有毒物质和身体的代谢废物排除。 ②血液灌流法（hemoperfusion）：此法是将患儿血液经过体外循环，用吸附剂吸收毒物后再输回体内，应用指征与血液透析相同。有的毒物血液透析不能析出，用血液灌流则有效。③换血疗法：当中毒不久，血液中毒物浓度极高时，可用换血疗法，但此法需血量极多，临床较少采用。④血浆置换：能清除患者血浆蛋白结合的毒物。

4）高压氧的应用：在高压氧情况下，血中氧溶解度增高，氧分压增高，促使氧更易于进入组织细胞中，从而纠正组织缺氧。可用于一氧化碳、硫化氢、氰化物、氨气等中毒。在一氧化碳中毒时，应用高压氧治疗，可以促使一氧化碳与血红蛋白分离。

3．特异性解毒剂的应用（详见表18-1）

4．其他对症治疗 及时处理各种中毒所致的严重症状，如惊厥、呼吸困难、循环衰竭等，若不及时治疗，随时可危及生命。在中毒原因不明或无特效治疗时，对症治疗尤为重要，以便支持患儿渡过危险期。

【中毒的预防】

为了防止小儿中毒的发生，要做好如下几项工作：

1．管好药品 药品用量、用法或存放不当是造成药物中毒的主要原因。家长切勿擅自给小儿用药，更不可把成人药随便给小儿吃。不要将外用药物装入内服药瓶中。儿科医务人员开处方时，应认真计算不同年龄小儿用药量，切勿过量；药剂人员应细心核对药量和剂型，耐心向家长说明服用方法。家庭中一切药品皆应妥善存放，不让小儿随便取到。

2．农村或家庭日常用的灭虫、灭蚊、灭鼠剧毒药品，更要妥善处理，避免小儿接触，各

表18-1　常见毒物的解毒剂、剂量及用法

中毒种类	有效解毒剂	剂量、用法及注意点
砷、汞、金、锑、铋、铜、铬、镍、钨、锌	二巯基丙醇（BAL）	每次3～5 mg/kg，深部肌注，每4 小时1 次，常用5～10 日为一疗程
	二巯基丙磺酸钠	每次5%溶液0.1 ml/kg，皮下或肌注，第1 日3～4 次，第2 日2～3 次，第3 日以后每日1～2 次，共用3～7 日，总剂量30～50 ml
	二巯基丁酸（DMSA）	10 mg/kg，口服，每8 小时1 次，共5 天，再以每12 小时1 次，共14 天
	硫代硫酸钠	每次10～20 mg/kg，配成5%～10%溶液，静脉注射或肌注，每日1次，3～5日。或l0～20 ml 口服，每日2 次（口服只能作用于胃肠道内未被吸收的毒物）
铅、锰、铀、镭、钒、钴、铁、硒、镉、铜、铬、汞	依地酸二钠钙（Ca-Na$_2$-EDTA）	1～1.5 g/（m^2•24h），分为每12 小时一次，肌注，共5 天。
	促排灵（diethylenetriamine pantaacetic acid，DTPA）	每次15～30 mg/kg，配成10%～25%溶液肌注，或以生理盐水稀释成0.2%～0.5%溶液静脉点滴，每日2 次，3 日为一疗程，间隔3 日再用第二疗程。
	去铁敏	15 mg/（kg•h），每天总量不超过6 g。
	青霉胺	治疗慢性铅、汞中毒l00 mg/（kg•d），分4 次口服，5～7 天为一疗程
高铁血红蛋白血症（亚硝酸盐、苯胺、非那西丁、硝基苯、安替比林、氯酸盐类、磺胺类等）	亚甲蓝	每次1～2 mg / kg，配成1%溶液，静脉注射，或每次2～3 mg / kg，口服，若症状不消失或重现，0.5～1 小时后可再重复。
	维生素C	每日500～1000 mg加在5%～10%葡萄糖溶液内静脉点滴，或每日口服1～2 g（作用比亚甲蓝慢）
氢氰酸及氰酸化合物（桃仁，杏仁、李仁、樱桃仁、枇杷仁，亚麻仁、木薯）	亚硝酸异戊酯	吸入剂用时压碎，每1～2 分钟吸入15～30 秒，反复吸入至硝酸钠注射为止
	亚硝酸钠	6～l0 mg / kg，配成1%溶液静脉注射，3～5 分钟注入，每次注射前要准备好肾上腺素，当血压急剧下降时应给注射肾上腺素
	硫代硫酸钠	25%溶液每次0.25～0.5 g/kg，静脉缓慢注射（约10～15 分钟内注完）
	亚甲蓝（美蓝）	1%溶液每次l0 mg/kg，静脉缓慢注射，注射时观察口唇，至口唇变暗紫色即停止注射
	以上三种药物，最好先注射亚硝酸钠，继之注射硫代硫酸钠，或先注射亚甲蓝，继之注射硫代硫酸钠，重复时剂量减半，注意血压下降时应给注射肾上腺素	
有机磷化合物类（1605、1059、3911、敌百虫、敌敌畏、乐果、其他有机磷农药）	碘解磷定<br>氯磷定	每次15～30mg/kg（成人0.5～1g/kg），配成2.5%溶液静脉缓慢注射或静点，严重患儿2 小时后可重复注射，并与阿托品同时应用，至肌肉颤动停止意识恢复。氯磷定可作肌肉注射
	双复磷	成人0.25～0.75 g/次，皮下、肌内或静脉注射均可。小儿酌减
	阿托品	严重中毒：首次剂量0.05～0.1 mg/kg，静脉注射，以后每次0.05 mg/kg，5～10 分钟1 次，至瞳孔开始散大，肺水肿消退，改为每次0.02～0.03 mg/kg，皮下注射，15～30 分钟1 次，至意识恢复改为每次0.01～0.02 mg/kg，30～60 分钟1 次。中度中毒：每次0.03～0.05 mg/kg，15～30 分钟1次<br>皮下注射，减量指征同上。轻度中毒每次0.02～0.03 mg/kg，口服或皮下注射，必要时重复。以上治疗均为瞳孔散后停药，严密观察24～48 小时，必要时应再给药。同时合并应用碘解磷定比单用阿托品效果好，阿托品的剂量也可以减小。

中毒种类	有效解毒剂	剂量、用法及注意点
烟碱、毛果芸香碱、新斯的明、毒扁豆碱、槟榔碱、毒蕈氟乙酰胺	碘解磷定，氯解磷定或双复磷	对烟碱、新斯的明、毒扁豆碱中毒有效，剂量同上
	阿托品	每次0.03～0.05 mg/kg皮下注射，必要时15～30 分钟1 次
	解氟灵	0.1～0.3 g/（kg·d），分2～4 次肌注，可连续注射5～7 日，危重病例第1 次可注射0.2 g/kg，与解痉药和半胱氨酸合用，效果更好
阿托品	毛果芸香碱	每次0.1 mg/kg，皮下或肌注，15 分钟1 次
莨菪碱类		本药只能对抗阿托品类引起副交感神经作用，对中枢神经中毒症状无效，故应加用短作用的巴比妥类药物，如戊巴比妥钠或异戊巴比妥等
蔓陀罗颠茄		
	水杨酸毒扁豆碱	重症患儿用0.5～2 mg缓慢静脉注射，至少2～3分钟；如不见效，2～5 分钟后再重复一次，一旦见效则停药。复发者缓慢减至最小用量，每30～60 分钟一次。能逆转阿托品类中毒引起的中枢神经系统及周围神经系统症状
四氯化碳	葡萄糖酸钙	10%溶液10～20 ml加等量的5%～25%葡萄糖溶液静脉缓慢注射
草酸盐	氯化钙	3%溶液10～20 ml加等量的5%～25%葡萄糖溶液静脉缓慢注射
氟化物		
麻醉剂和镇静剂（阿片、吗啡、可待因、海洛因、哌替啶、美沙酮、水合氯醛、苯巴比妥（鲁米那）、巴比妥、巴比妥钠、异戊巴比妥、司可巴比妥、硫喷妥钠）	纳络酮	每次0.01 mg/kg，静脉注射，如无效增加至0.1 mg/kg，可重复应用。可静滴维持。
	丙烯吗啡	每次0.1 mg/kg，静脉、皮下或肌内注射，需要时隔10～15 分钟再注于1 次
氯丙嗪	苯海拉明	每次1～2 mg/kg，口服或肌内注射，只对抗肌肉震颤
奋乃静		
苯丙胺（安非他明）	氯丙嗪	每次0.5 ～1 mg/kg，6 小时1 次，若已用巴比妥类，剂量应减少
异烟肼中毒	维生素B_6	剂量等于异烟肼用量
鼠药（敌鼠）	维生素K_1	l0 mg/kg肌注，每天2～3 次
β-阻滞剂或钙通道阻滞剂中毒	高血糖素	首剂0.15 mg/kg静脉应用，以0.05～0.1 mg/（kg·h）静滴维持
阿司匹林	已酰唑胺	每次5 mg/kg，口服或肌注，必要时24 小时内可重复2～3 次
	碳酸氢钠	纠正脱水后若仍有严重酸中毒，可用5%碳酸氢钠溶液每次6 ml/kg，静脉滴入，以后必要时可重复1 次，治疗开始后每半小时查尿一次，使尿保持为碱性，若变为酸性时，应静脉滴入1.4%碳酸氢钠溶液l0 ml/kg 乳酸钠 用1/6 mol浓度的乳酸钠溶液代替上述1.4%碳酸氢钠溶液亦可，但效果不如碳酸氢钠
	维生素K_1	20～50 mg肌内注射，预防出血
一氧化碳（煤气）	氧气	100%氧气吸入，高压氧舱
肉毒中毒	多价抗肉毒血清	1 万～5 万单位肌注
河豚中毒	半胱氨酸	成人剂量为0.1～0.2 g肌注，每天2 次，儿童酌情减量

种农药务必按照规定办法使用。

3．做好识别有毒植物的宣传工作，教育小儿不要随便采食野生植物。

4．禁止小儿玩耍带毒性物质的用具（如装敌敌畏的小瓶等）。

5．普及相关预防中毒的健康知识教育。

（杜立中）

全国高等学校教材

供基础、临床、预防、口腔医学类专业用

1. 医用高等数学／第5版
2. 医学物理学／第7版
3. 基础化学／第7版
4. 有机化学／第7版
5. 医学生物学／第7版
6. 系统解剖学／第7版
7. 局部解剖学／第7版
8. 组织学与胚胎学／第7版
9. 生物化学／第7版
10. 生理学／第7版
11. 医学微生物学／第7版
12. 人体寄生虫学／第7版
13. 医学免疫学／第5版
14. 病理学／第7版
15. 病理生理学／第7版
16. 药理学／第7版
17. 医学心理学／第5版
18. 法医学／第5版
19. 诊断学／第7版
20. 医学影像学／第6版
21. 内科学／第7版
22. 外科学／第7版
23. 妇产科学／第7版
24. 儿科学／第7版
25. 神经病学／第6版
26. 精神病学／第6版
27. 传染病学／第7版
28. 眼科学／第7版
29. 耳鼻咽喉-头颈外科学／第7版
30. 口腔科学／第7版
31. 皮肤性病学／第7版
32. 核医学／第7版
33. 流行病学／第7版
34. 卫生学／第7版
35. 预防医学／第5版
36. 中医学／第7版
37. 计算机应用基础／第4版
38. 体育／第4版
39. 医学细胞生物学／第4版
40. 医学分子生物学／第3版
41. 医学遗传学／第5版
42. 临床药理学／第4版
43. 医学统计学／第5版
44. 医学伦理学／第3版
45. 临床流行病学／第3版
46. 康复医学／第4版
47. 医学文献检索／第3版
48. 卫生法／第3版
49. 医学导论／第3版
50. 全科医学概论／第3版
51. 麻醉学／第2版
52. 急诊医学

策划编辑　祁　军　赵永昌
责任编辑　刘兴攀
封面设计　郭　森
版式设计　郭　森　李秋斋　何美玲

ISBN 978-7-117-09520-4
9 787117 095204 >
定价(含光盘)：46.00 元
